# Single-cell sequencing systematically analyzed the mechanism of Emdogain on the restoration of delayed replantation periodontal membrane

**DOI:** 10.1038/s41368-024-00345-5

**Published:** 2025-04-17

**Authors:** Yanyi Liu, Yuhao Peng, Lanhui Chen, Yangfan Xiang, Ximu Zhang, Jinlin Song

**Affiliations:** 1 The Affiliated Stomatological Hospital of Chongqing Medical University, Chongqing, China; 2 Chongqing Key Laboratory of Oral Diseases, Chongqing, China; 3 Chongqing Municipal Key Laboratory of Oral Biomedical Engineering of Higher Education, Chongqing, China; 4 Chongqing Municipal Health Commission Key Laboratory of Oral Biomedical Engineering, Chongqing, China

**Keywords:** Oral diseases, Computational biology and bioinformatics

## Abstract

The repair of the periodontal membrane is essential for the successful management of periodontal disease and dental trauma. Emdogain^®^ (EMD) is widely used in periodontal therapy due to its ability to promote repair. Despite substantial research, the cellular and molecular mechanisms underlying EMD’s effects, particularly at the single-cell resolution, remain incompletely understood. This study established a delayed tooth replantation model in rats to investigate these aspects. Tooth loss rate and degree of loosening were evaluated at 4 and 8 weeks. Micro-CT, HE staining, TRAP staining, and immunofluorescence staining were evaluated to assess EMD’s efficacy. Single-cell sequencing analyses generated single-cell maps that explored enrichment pathways, cell communication, and potential repair mechanisms. Findings indicated that EMD could reduce the rate of tooth loss, promote periodontal membrane repair, and reduce root and bone resorption. Single-cell analysis revealed that EMD promotes the importance of *Vtn+* fibroblasts, enhancing matrix and tissue regeneration functions. Additionally, EMD stimulated osteogenic pathways, reduced osteoclastic activity, and promoted angiogenesis-related pathways, particularly bone-related H-type vessel expression in endothelial cells. Gene modules associated with angiogenesis, osteogenesis, and odontoblast differentiation were identified, suggesting EMD might facilitate osteogenesis and odontoblast differentiation by upregulating endothelium-related genes. Immune cell analysis indicated that EMD did not elicit a significant immune response. Cell communication analysis suggested that EMD fostered pro-regenerative networks driven by interactions between mesenchymal stem cells, fibroblasts, and endothelial cells. In conclusion, EMD proves to be an effective root surface therapy agent that supports the restoration of delayed replantation teeth.

## Introduction

Periodontal regeneration is a complex and highly coordinated biological process involving the interaction of multiple cell types, extracellular matrix components, and signaling molecules. The periodontal ligament (PDL) plays a crucial role in maintaining tooth stability and function, making its regeneration essential for the successful treatment of periodontal disease and dental trauma^1–3^. Dental diseases and trauma often lead to periodontal destruction and tooth loss, necessitating the restoration of tooth function and aesthetics through the replantation of natural teeth^4^. However, natural tooth replantation often involves delayed treatment, which poses significant challenges such as periodontal ligament damage and bone resorption, leading to suboptimal treatment outcomes. Root surface treatment can facilitate the restoration of replanted teeth^5^, and EMD, as a root surface treatment agent, is a promising clinical material.

EMD is a protein complex extracted from developing pig enamel. Its main components include enamel matrix protein and amelogenin protein^6^, both of which are essential in tooth development and periodontal regeneration. Previous studies have demonstrated that EMD plays a crucial role in promoting periodontal tissue regeneration by regulating cytokines and signaling pathways, stimulating the proliferation and differentiation of osteoblasts and periodontal ligament cells, and promoting the formation of new bone, cementum, and periodontal ligament fibers. Additionally, it aids in the reattachment of teeth to alveolar cavities^7–9^. Clinically, EMD has been applied in guided tissue regeneration (GTR) and other procedures, with numerous studies reporting promising clinical outcomes^10–12^. However, the precise cellular and molecular mechanisms underlying EMD in periodontal ligament repair have yet to be thoroughly elucidated.

Single-cell RNA sequencing (scRNA-seq) has become a potent method for exploring intricate biological phenomena^13,14^. This technique allows for a comprehensive characterization of cell heterogeneity, identification of distinct cell populations, and elucidation of cell-specific gene expression profiles and signaling pathways. However, in certain cases, such as studies involving sensitive cells or tissues with limited availability (e.g., hippocampus and nerves), the number of sample cells may be insufficient to meet the requirements for scRNA-seq. In such instances, it is necessary to use mixed samples for sequencing, which not only meets the sample requirements but also introduces biological replication to mitigate differences between individuals as well as exclude outlier samples^15–17^. Souporcell is a clustering method designed for mixed-genotype scRNA-seq experiments. It can cluster cells by individual, identify doublet barcodes, and infer the amount of ambient RNA in the experiment^18^. This genotype-free demultiplexing software does not require the capture of donor SNP genotypes in multiple captures. Clustering cells based on genetic variants detected in scRNA-seq reads demonstrates high accuracy. By pooling periodontal tissue from different rats into a single group for scRNA-seq and employing Souporcell technology, we obtained unprecedented insights into the cellular composition and molecular landscape of periodontal repair tissue facilitated by EMD.

In this study, the effect of EMD on periodontal membrane regeneration was observed using delayed tooth replantation model in rats. At the same time, periodontal tissues were extracted, and scRNA-seq and analysis were performed. Cell experiments, animal experiments, and transcriptome analysis from a self-measured mRNA-seq dataset were used to validate the scRNA-seq results. This research offers fresh insights into the cellular and molecular mechanisms of periodontal membrane repair in delayed tooth replantation facilitated by EMD, deepens the understanding of EMD’s restorative role, and holds significant implications for developing more effective periodontal regeneration and treatment strategies for dental trauma.

## Results

### EMD promotes the restoration of periodontal membrane of delayed molar replantation in rats

**Fig. 1:**
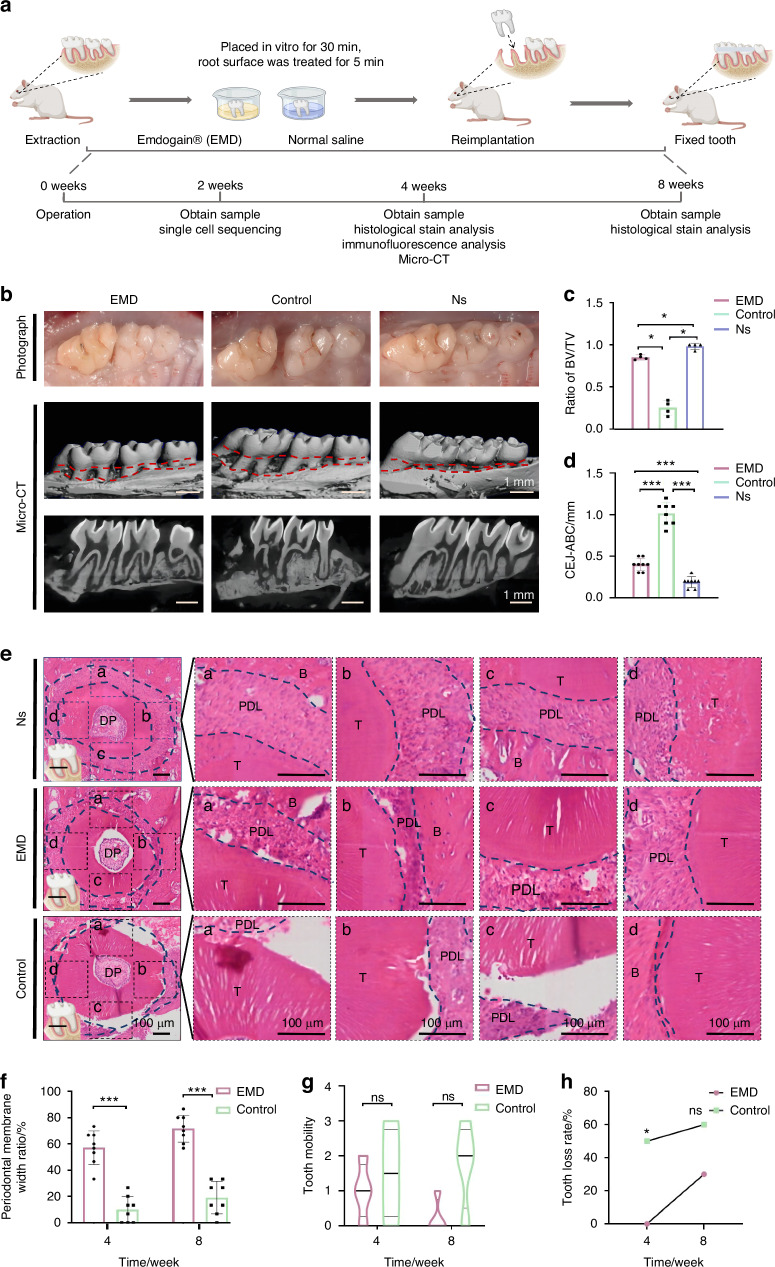
Results of delayed periodontal repair induced by EMD in SD rats. **a** Experimental diagram of delayed tooth replantation model in rats. The diagram generated in part with BioRender.com. **b** General view and micro-CT of maxillary first molar replantation after tooth extraction. **c** Statistical results of bone volume fraction. **d** Statistical results of alveolar resorption. CEJ the cementum junction, ABC alveolar bone crest. **e** HE staining results of 8 weeks. T means tooth, B means bone, and P means periodontium, DP means dental pulp. **f** Statistical results of periodontal membrane repair width ratio. **g** Statistical results of Looseness s. **h** Statistical chart of tooth loss rate

Animal experiments were carried out following the established protocol (Fig. 1a). The micro-CT results (Fig. 1b) after 4 weeks showed that the root and the alveolar bone around the root of the control group had extensive resorption. In contrast, the root of the EMD group was relatively complete with a small part resorption of the alveolar bone around the root. Bone volume fraction statistics showed that a small part of bone volume decreased in EMD (0.85 ± 0.03) compared with normal tissues. In contrast, the bone volume of control group (0.25 ± 0.09) decreased by more than 70%, and the differences were statistically significant (Fig. 1c). Additionally, the measurement from the alveolar bone crest to the cementum junction (Fig. 1d) indicated that the bone resorption in the EMD group (0.40 mm ± 0.08 mm) was significantly lower than in the control group (1.01 mm ± 0.14 mm). HE staining showed that the periodontal membrane repair effect in EMD group was better than that in control group (Figs. 1e, 8 weeks; Figs. S1a, 4 weeks). The results of the periodontal membrane width ratio indicated that the periodontal membrane repair width was 57.09% ± 0.13% in the EMD group and only 10% ± 0.1% in the control group at 4w. At 8w, the repair width of periodontal membrane was 71.68% ± 0.1% in EMD group and 19.16% ± 0.13% in control group (Fig. 1f). The results indicated that periodontal membrane repair was better in EMD. Next, the periodontal membrane around the single root of the HE section was divided into 12 equal fractions, which was used as the statistical point to calculate the root absorption rate. The results showed that the surface absorption after EMD treatment (0.08 ± 0) was significantly lower than that of the control group (0.79 ± 0.11) at 4 weeks, but there was no statistical difference between them in inflammatory absorption and substitute absorption (Fig. S1b, Table S1). The statistical results after 8 weeks indicated a lower root resorption rate for the EMD group, although the difference between the EMD and control groups was not statistically significant (Fig. S1c, Table S1). The results of the loosening test revealed that, although there was no statistical difference, the overall trend of EMD loosening was lower than that of the control group (Fig. 1g). At the same time, the tooth loss rate of the EMD group (0%, 4 weeks; 30%, 8 weeks) collected was significantly lower than that of the control group (50%, 4 weeks; 60%, 8 weeks) (Fig. 1h).

### ScRNA-seq of periodontal membrane tissue treated with EMD

**Fig. 2:**
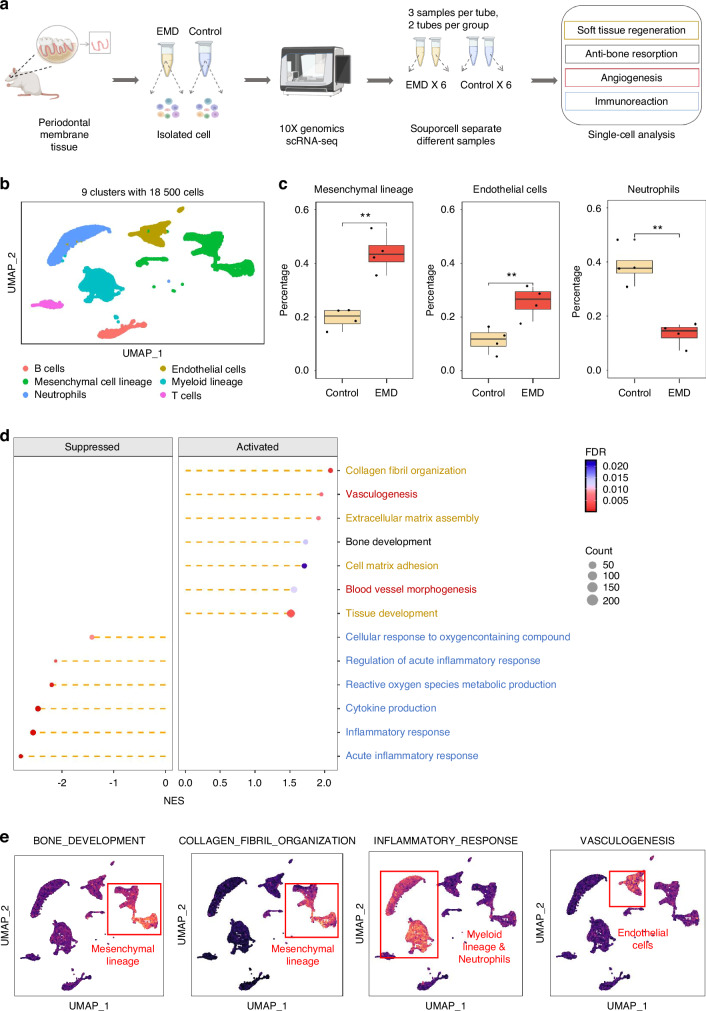
Single-cell RNA sequencing analysis of periodontal membrane tissue treated with EMD. **a** Schematic diagram of scRNA-seq. The diagram generated in part with BioRender.com. **b** t-SNE plot showing six distinct cell types identified from 18 500 cells. **c** Box plots comparing the percentages of mesenchymal cell lineage, endothelial cells, neutrophils, and myeloid lineage between control and EMD-treated samples. **d** Dot plot illustrates the enrichment of biological processes with significant activation and suppression in the EMD treated samples, with a focus on processes like soft tissue regeneration (yellow), anti-bone resorption (black), angiogenesis (red) and inflammation-related pathways (blue). The processes are color-coded by type, and the normalized enrichment score (NES) indicates the degree of activation (right) or suppression (left), with corresponding false discovery rate (FDR) values and gene set counts. **e** UMAP plots highlighting the enrichment of specific biological processes in distinct cell types

ScRNA-seq of periodontal membrane tissue from the EMD and control groups revealed significant insights into the cellular and molecular dynamics of periodontal regeneration (Fig. 2a). Using Souporcell technology, the identified gene variants were clustered into distinct cells to facilitate the separation of mixed samples. Subsequently, a statistical analysis was conducted to assess the cell count for each sample. The resulting *P*-value of 0.4433 indicated that the differences in cell counts were not statistically significant and the samples are suitable for further comparative analysis (Fig. S2b). After quality control and filtering, unsupervised clustering using the Seurat package identified ten distinct cellular populations. Based on the expression of known marker genes (Fig. S2a), these clusters were annotated as seven distinct cell types: B cells, endothelial cells, mesenchymal cell lineage, myeloid lineage, neutrophils, T cells, and epithelial cells. However, epithelial cells were excluded from further analysis due to their limited relevance to periodontal membrane repair. Uniform Manifold Approximation and projection (UMAP) visualization illustrated the distribution of these cell types across different samples (Fig. 2b). Dot plots showcased the expression of marker genes corresponding to different cell types (Fig. S3a, Table S4). The bar plots shows the distribution of cell types in each group (Fig. S2c). Next, four outliers were excluded (Control2, Control6, EMD5, and EMD3) and reordered them for subsequent analysis (Table S3, Fig. S3b). Comparative analysis between EMD-treated and control samples revealed significant changes in the cellular composition of the periodontal ligament. Notably, the proportion of mesenchymal cell lineage cells and endothelial cells was significantly increased in the EMD group (*P* < 0.05), while the proportion of neutrophils was reduced (Fig. 2c). GSEA analysis revealed significant differences in biological processes (BP) between the two groups (Table S5). Regeneration-related pathways, including bone development, collagen fibril organization, and vasculogenesis were predominantly upregulated (activated) in the EMD group. In contrast, inflammation-related pathways, such as cytokine production and inflammatory response were downregulated (suppressed) (Fig. 2d). Functional enrichment UMAP visualizations contextualized these findings, highlighting the distinct functional characteristics among cell types (Fig. 2e). Together with UMAP clustering (Fig. 2b), this analysis underscores the cellular heterogeneity and identifies key pathways influenced by EMD in periodontal ligament regeneration and osteogenesis.

### Identification and characterization of mesenchymal cell lineage subpopulations

**Fig. 3:**
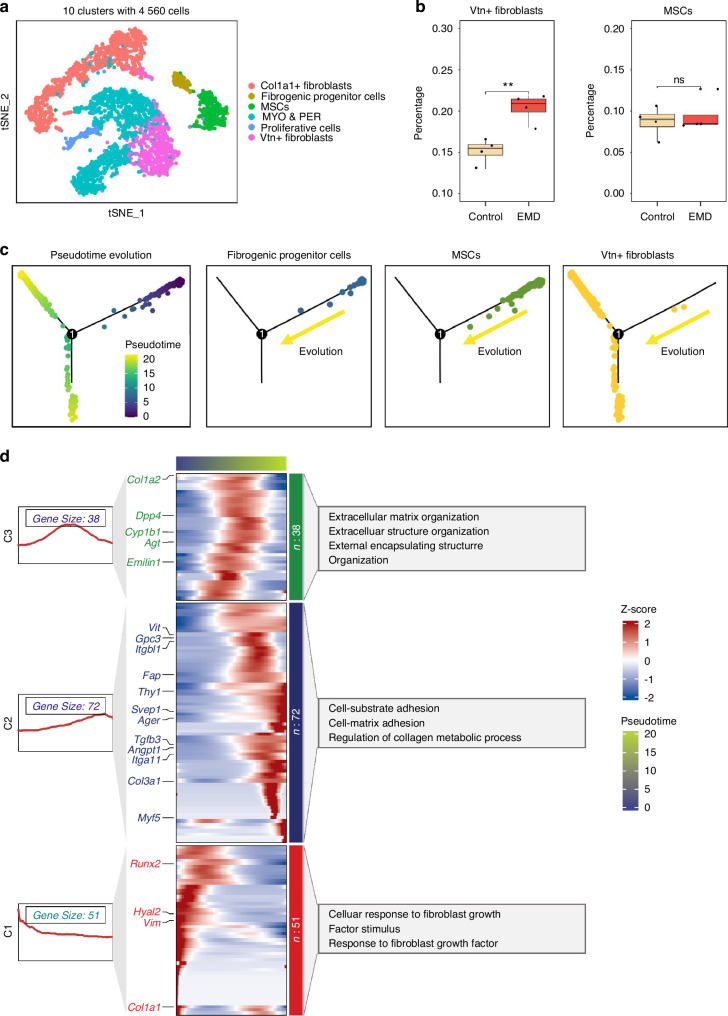
Identification and characterization of mesenchymal cell lineage subpopulations. **a** t-SNE plots showing the clustering of 4 560 cells into 10 distinct clusters. **b** Box plots comparing the proportion of specific fibroblast subtypes (*Vtn+* fibroblasts and MSCs) between control and EMD-treated samples. **c** Pseudotime analysis of mesenchymal cell lineage. From left to right, these plots show the overall pseudotime trajectory, the trajectories of specific subpopulations: fibrogenic progenitor cells, MSCs, and *Vtn*+ fibroblasts. **d** Heatmap of gene expression along the pseudotime trajectory, divided into three clusters (C1, C2, C3). The right side of the heatmap annotates the major biological processes associated with each gene cluster. The gene score plots on the left illustrate the progression of gene expression scores over pseudotime

UMAP visualization was performed to identify distinct subpopulations within the mesenchymal cell lineage, revealing ten clusters comprising 4 560 cells (Fig. 3a, Fig. S4a). Based on marker gene expression, these clusters were annotated as *Col1a1*+ fibroblasts, fibrogenic progenitor cells, mesenchymal stem cells (MSCs), Myofibroblasts and Pericytes (MYO & PER), proliferative cells, and *Vtn*+ fibroblasts. The expression of specific marker genes within these clusters was visualized using UMAP plots (Fig. S4c). Essential marker genes such as *Vtn*, *Col1a1*, *Mki67*, *Sox2*, and *Alcam* were distinctly expressed in different subpopulations, confirming the identities of these clusters. The proportional distribution of mesenchymal subpopulations between control and EMD-treated samples was analyzed (Fig. S4b). The proportion of *Vtn+* fibroblasts was significantly higher in the EMD-treated group compared to the control group, suggesting an increase in its importance. Although the absolute values (101.50 ± 67.89 in the control group and 102.25 ± 18.82 in the EMD group, with a *P*-value of 0.98) did not demonstrate a statistically significant difference at this time point (Fig. 3b). Besides, EMD-treated samples showed a significant higher expression of regeneration-associated genes according to the BP of GO enrichment analysis (GOBP) compared to the control group (Fig. S4d). Pseudotime trajectory analysis revealed the differentiation pathways of the mesenchymal subpopulations. The trajectory analysis showed the dynamic progression of fibrogenic progenitor cells and MSCs differentiating into *Vtn*^+^ fibroblasts (Fig. 3c). The heatmap of gene expression dynamics along the pseudotime trajectory highlighted the transition states of mesenchymal cells within the differentiation pathway. Cluster-specific gene sets (C1, C2, C3) reveal biorelated processes enriched during differentiation. As the differentiation time progressed, the gene set exhibited a gradual activation from C1 to C2 and C3. The C1 gene set was primarily enriched in pathways associated with cellular response to fibroblast growth, factor stimulus and so on. C2 gene set was primarily enriched in pathways associated with cell-substrate adhesion, cell-matrix adhesion and so on, and C3 gene set was mainly enriched in pathways associated with extracellular matrix organization, extracellular structure organization and so on (Fig. 3d). This indicates that, compared to the control group, EMD treatment may enhanced these repair-related functions during the differentiation of mesenchymal cells over time.

### Transcriptomic data from bone marrow-derived mesenchymal stem cells (BMSCs) were utilized to validate the role of *Vtn*+ fibroblasts in soft tissue regeneration, as evidenced by the scRNA-seq analysis results

**Fig. 4:**
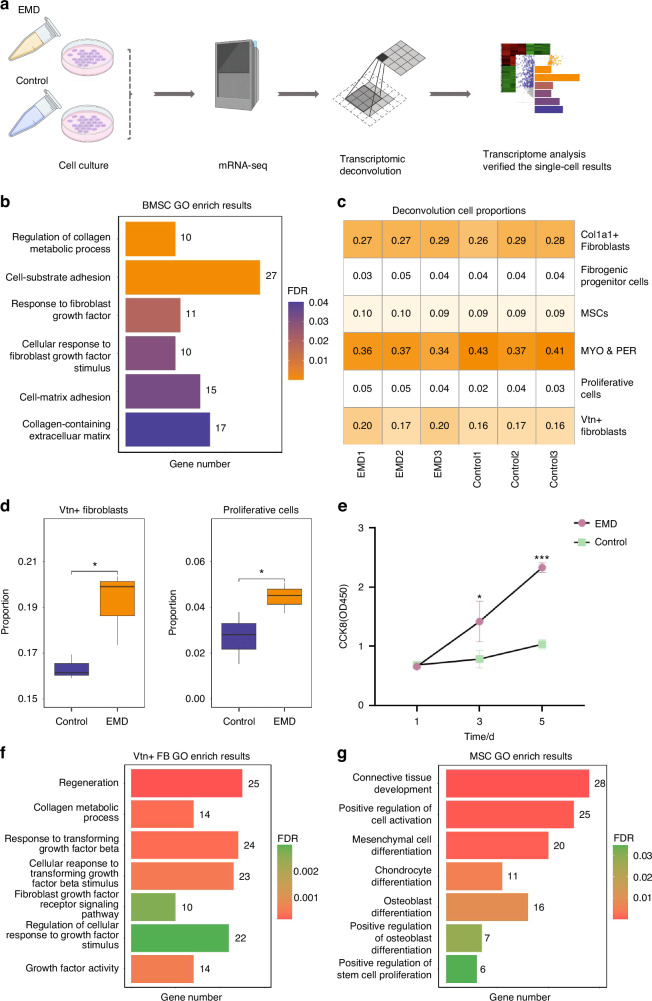
Transcriptomic data from BMSCs were utilized to validate the role of *Vtn+* fibroblasts in soft tissue regeneration, as evidenced by the scRNA-seq analysis results. **a** Schematic representation of the transcriptome experimental design. The diagram generated in part with BioRender.com. **b** The GO enrichment analysis of upregulated genes between the EMD-treated and control groups. **c** Heatmap of cell type proportions estimated from mRNA-seq data after deconvolution analysis. **d** Box plots illustrated the proportions of *Vtn*+ fibroblasts and proliferative cells in EMD-treated and control groups. **e** CCK8 results of EMD treated cells. **f**, **g** GO enrichment analysis for *Vtn*+ fibroblasts and MSCs based on scRNA-seq data.The bar plot displays enriched GO terms

Next, an in-house mRNA-seq dataset was used to verify the results of the previous scRNA-seq analysis (Fig. 4a). EMD and saline treated BMSCs were used for mRNA-seq. The BMSCs transcriptome data revealed enriched pathways such as collagen metabolism regulation, cell-substrate adhesion, response to fibroblasts growth factor, cellular response to fibroblasts growth factor stimulus, cell-matrix adhesion, and the presence of collagen-containing extracellular matrix (Fig. 4b). These enriched pathways were also identified in the single-cell pseudotime analysis and EMD treatment. The heatmap of cell type proportions in BMSCs transcriptome data after deconvolution presents the proportions of various cell types, including *Col1a1*+ fibroblasts, fibrogenic progenitor cells, MSCs, MYO & PER, proliferative cells, and *Vtn*+ fibroblasts (Fig. 4c). The boxplots revealed that the proportion of *Vtn+* fibroblasts and proliferation cells in EMD treated group was significantly higher than that in control group (Fig. 4d). The increase proportion of *Vtn+* fibroblasts was consistent with the results of scRNA-seq. The results of CCK8 cell proliferation experiment indicated that cell proliferation over time was significantly higher in the EMD treatment group than in the control group, especially on day 5, and confirmed the sequencing results (Fig. 4e). GO pathway enrichment analyses for *Vtn*+ fibroblasts displayed enriched pathways, including regeneration, collagen metabolic process, response to transforming growth factor beta (TGF-β), cellular response to transforming growth factor beta (TGF-β) stimulus, fibroblasts growth factor receptor signaling pathway, regulation of cellular response to growth factor stimulus, and growth factor activity (Fig. 4f, Table S6). These enriched pathways suggest that the functionality of *Vtn*+ fibroblasts treated with EMD differs from that of the control. Specifically, EMD treatment enhances the regenerative processes and the response to growth factor in *Vtn+* fibroblasts, highlighting the functional differences induced by EMD treatment. For MSCs, the enriched pathways encompass connective tissue development, positive regulation of cell activation, mesenchymal cell differentiation, chondrocyte differentiation, osteoblast differentiation, positive regulation of osteoblast differentiation, and positive regulation of stem cell proliferation (Fig. 4g, Table S6). This result emphasizes the functional differences of stem cells demonstrated by EMD treatment.

### Effects of EMD on osteogenesis and osteoclast processes

**Fig. 5:**
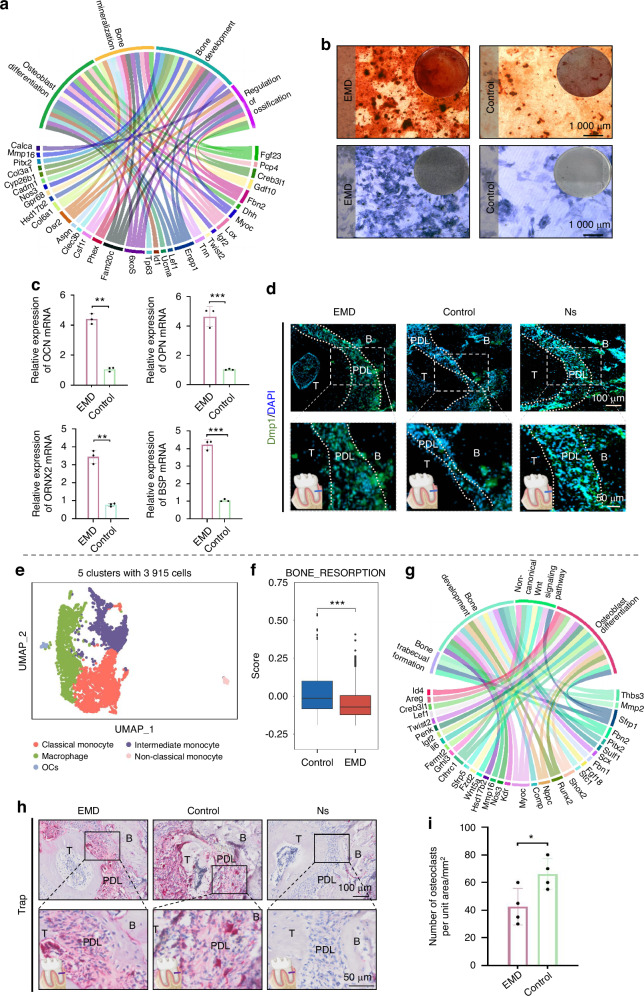
Effects of EMD on osteogenesis and osteoclast processes. **a** GO enrichment analysis chord diagram of *Vtn*+ fibroblasts, illustrating the relationship between GO terms and corresponding differentially expressed genes involved in various BP such as osteoblast differentiation, bone resorption, and cell adhesion. **b** ALP and ARS staining results of BMSCs. **c** Statistical map of expression of osteogenic genes by q-PCR. **d** The result of DMP1 immunofluorescence staining in rat sections. **e** UMAP plot showing the clustering of 3 915 cells into 5 distinct clusters. **f** Box plot comparing bone resorption activity between control and EMD-treated groups. **g** GO enrichment analysis chord diagram of osteoclasts, illustrating the relationship between gene ontology terms and corresponding differentially expressed genes involved in BP such as bone resorption, bone trabecula formation, and osteoblast differentiation. **h** Results of TRAP staining of rat sections. **i** Statistics of TRAP staining results

In the analysis of *Vtn*+ fibroblasts, pathways significantly enriched in osteogenesis-related processes were identified (Fig. 5a). This enrichment suggest a strong inclination towards bone formation mechanisms within the EMD-treated group. In order to further illustrate the osteogenic effect of EMD, BMSCs were extracted from 2-week-old SD rats and bone induction was performed. Alizarin red staining (ARS) with calcium ions to form an orange-red or red precipitate, thus visualizing the deposition of calcium salts. Alkaline phosphatase staining (ALP) results show purple or blue precipitate, which is used to display alkaline phosphatase activity in tissues. The ALP and ARS results (Fig. 5b) showed that EMD significantly promoted bone formation. The semi-quantitative statistical results showed statistical differences (Fig. S5a and b). After 7 days of osteogenic differentiation induced by EMD, the sample was detected by quantitative polymerase chain reaction (q-PCR). The results indicated that the expression of osteogenic genes increased significantly in the EMD treated group (Fig. 5c, Table S2). To further confirm the osteogenic effect of EMD through histological samples, immunofluorescence staining of DMP1 was performed on histological sections of rats (Fig. 5d), which showed that EMD-treated rats had more DMP1 cells in their periodontal region (Fig. S5c).

Further subpopulation annotation of the myeloid lineage was used to detect the expression of osteoclasts. The annotation of myeloid lineage reveals five distinct clusters (Fig. 5e), with specific marker genes detailed in Fig. S5d. The results of the GOPB pathway scoring analysis revealed that the control group demonstrated a statistically significant increase in the bone resorption pathway compared to the EMD group (Fig. 5f). These findings highlight a differential regulatory effect of EMD treatment, where bone resorption pathways are less pronounced, while osteogenesis pathways are more prominently enriched (Fig. 5g). The TRAP staining results of periodontal sections of rats showed that the number of osteoclasts in the EMD group was lower than that in the control group, and the difference was statistically significant, which confirmed our results (Fig. 5h, i).

### Identification and characterization of endothelial subpopulations

**Fig. 6:**
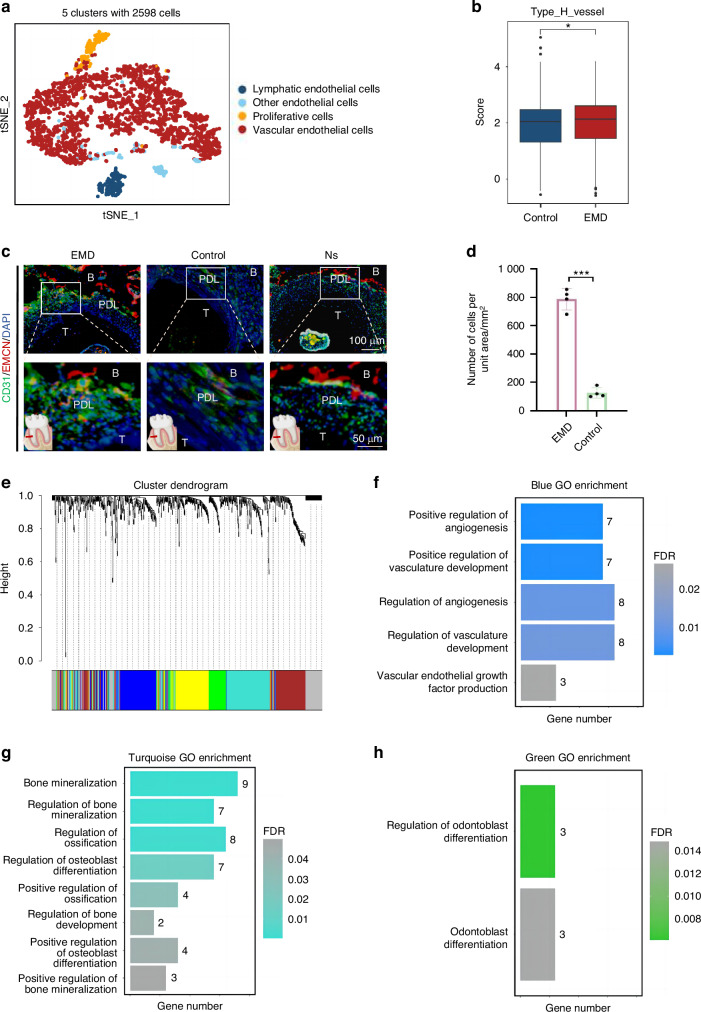
Identification and characterization of endothelial subpopulations. **a** UMAP plot showing the clustering of 2 598 endothelial cells into 5 distinct clusters. **b** Box plot comparing the expression of *Emcn* between the control and EMD treatment groups, indicating a significant increase in the EMD group. **c** The result of H-type vascular immunostaining in rat sections. **d** Statistical diagram of H-type vascular immunostaining results in rat sections. **e** Dendrogram and corresponding heatmap from hierarchical clustering analysis of gene expression, showing distinct clusters identified by dynamic tree cutting. **f**–**h** Bar plots showing the enriched GO terms related to angiogenesis, bone development and odontoblast differentiation, with numbers representing the gene count and color indicating the adjust *P*-value

Five distinct clusters were identified and annotated in the clustering analysis of endothelial cells, highlighting the diversity within the endothelial cell population (Fig. 6a). The proportional distribution of different cell types is illustrated in the bar plot, indicating variations across different samples (Fig. S6b). The UMAP plots for marker genes specific to endothelial cells, such as *Emcn*, *Mki67*, *Prox1*, and *Vwf*, further validate the identified clusters (Fig. S6a). The comparison of endothelial cell subtypes between EMD and control groups showed no significant differences in the proportions of vascular endothelial cells (Fig. S6d). Notably, the *Emcn* gene expression was significantly higher in the EMD group than the control, with statistical significance, suggesting a potential role in the observed phenotypic changes (Fig. 6b). This phenomenon was confirmed by immunofluorescence staining of *Emcn*-expressed endothelial cells on periodontal sections of rats (Fig. 6c, d). Pathway enrichment analysis for endothelial cells in the EMD group revealed significant enrichment in pathways related to angiogenesis and vascular regeneration (Fig. S6e). Differential gene expression analysis between EMD and control groups identified upregulated genes in the EMD group, which were then subjected to Weighted Gene Co-expression Network Analysis (WGCNA) (Fig. 6e, Fig. S6c, Table S7). This analysis identified distinct gene co-expression modules, with the blue module significantly associated with vascular regeneration pathways (Fig. 6f). Additionally, the turquoise module was found to be related to osteogenesis, and the green module was found to be related to odontoblast differentiation, indicating that EMD therapy may promote angiogenesis, osteogenesis and dentin regeneration to some extent by up-regulating the expression of endothelium-related genes(Fig. 6g–h, Table S8). These findings suggested that EMD was multifaceted in enhancing endothelial cell function, contributing to vascular, bone and dentin regeneration processes.

### Identification and characterization of immune subpopulations

**Fig. 7:**
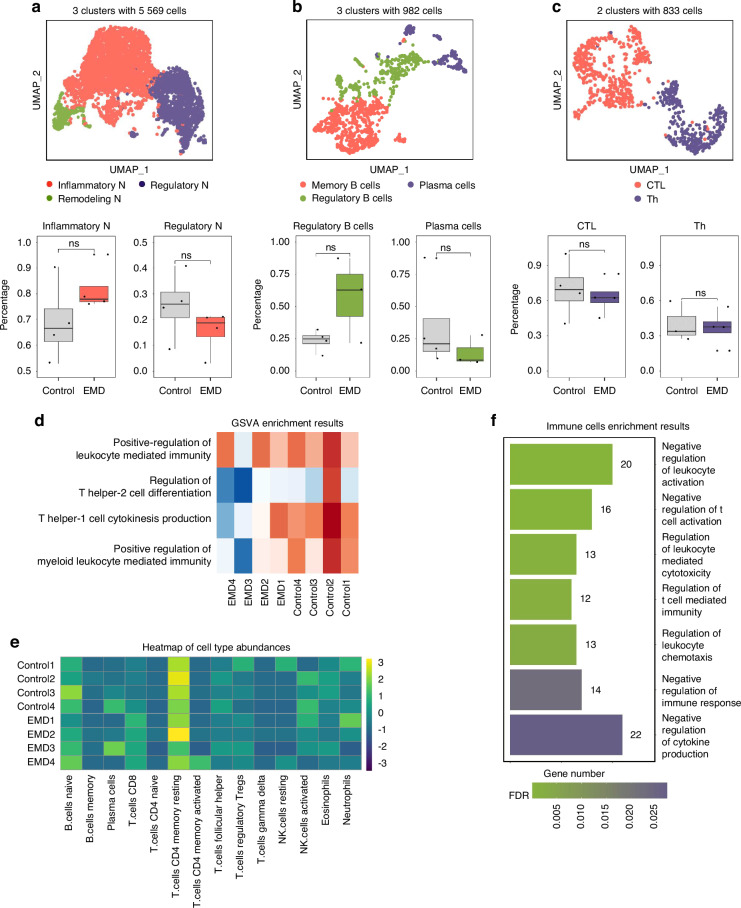
Identification and characterization of immune subpopulations. **a** UMAP plots of 5569 neutrophils classified into three clusters. Boxplots below show the proportions of inflammatory neutrophils and remodeling neutrophils in the control and EMD groups. **b** UMAP plots of 982 B cells classified into three clusters. Boxplots below show the proportions of plasma cells and memory B cells in the control and EMD groups. **c** UMAP plots of 833 T cells classified into two clusters. Boxplots below show the proportions of CTL and Th cells in the control and EMD groups. **d** The GSVA analysis heatmap illustrates the variations in inflammation-related pathways among different samples. **e** The heatmap, derived from LM22 deconvolution analysis, displays the abundances of various immune cell types across different samples, including the control and EMD groups. **f** The bar plot from GO enrichment analysis highlights significantly enriched pathways across all immune cells. Pathways are color-coded according to adjusted *P*-values

In analyzing different immune cell subpopulations, the clustering results revealed distinct subgroups for neutrophils, B cells, and T cells. Specifically, three cell types of neutrophils were identified: inflammatory neutrophils, regulatory neutrophils, and remodeling neutrophils (Fig. 7a). Marker gene expression for these subclusters, such as *Mmp8*, *Mmp9*, *S100a8*, and *Tnf*, was visualized, and no significant differences were found in the proportions of these subclusters between the EMD and control groups (Fig. S7a). Similarly, B cells were divided into memory B cells, plasma cells, and regulatory B cells, with marker genes like *Jchain*, *Il10*, and *Cd74*, again showing no significant differences in cell proportions between the EMD and control groups (Fig. 7b, Fig. S7b). T cells were categorized into cytotoxic T lymphocytes (CTLs) and helper T cells (Th), with markers such as *Il17a*, *Gzmb*, *Il12b*, and *Il19*, also showing no significant differences in proportions (Fig. 7c, Fig. S7c). Gene set variation analysis (GSVA) indicated higher expression of inflammation-related pathways in the control group, suggesting a more inflammatory immune profile without EMD treatment (Fig. 7d, Table S9). Analysis of immune cell infiltration further corroborated these findings, showing no significant differences in immune cell proportions between the EMD and control groups (Fig. 7e). GO enrichment analysis of all immune cells in the EMD group revealed significant enrichment in pathways related to the negative regulation of leukocyte activation and cytokine production, indicating a shift towards anti-inflammatory responses with EMD treatment (Fig. 7f). These results collectively suggested that while the overall proportions of immune cell subpopulations remain similar between EMD and control groups, EMD treatment might modulate the inflammatory state through promoting anti-inflammatory pathways.

### Comparative analysis of cellular interactions and signaling patterns between EMD and control conditions

**Fig. 8:**
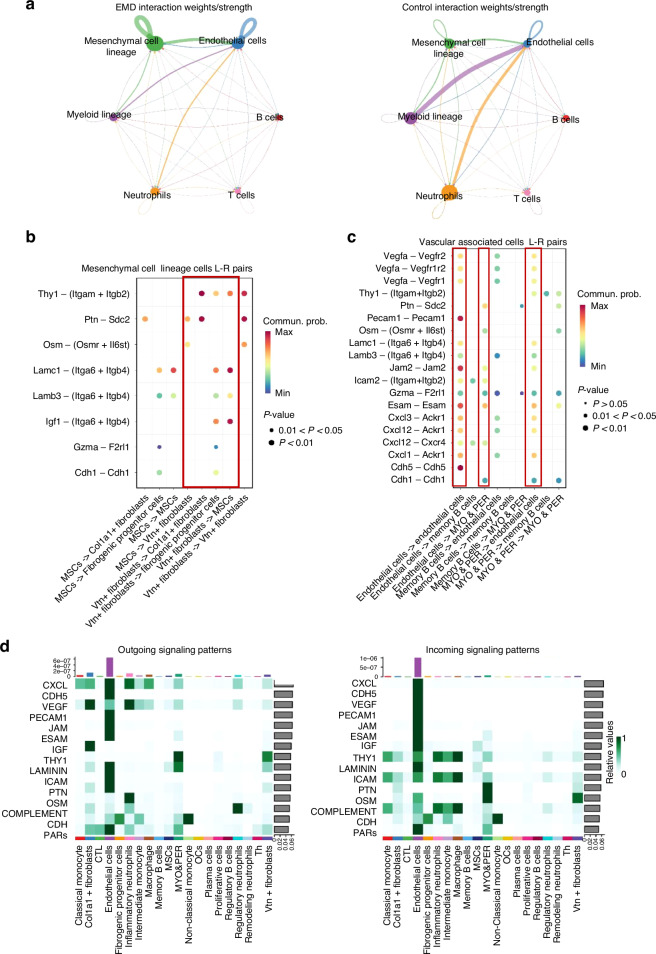
Comparative analysis of cellular interactions and signaling patterns between EMD and control conditions. **a** Circular network plots depicting the interaction weights/strengths among various cell types under EMD and control conditions. Each node represents a cell type, with the size of the node corresponding to the number of interactions. The thickness and color of the edges indicate the strength and nature of the interactions, respectively. The plot on the left illustrates interactions under EMD conditions, while the plot on the right shows interactions under control conditions. **b**, **c** Dot plot visualizing the communication probabilities of selected signaling pathways under EMD conditions. The x-axis lists the interacting cell types, while the y-axis lists the specific signaling pathways. The size of the dots represents the *P*-value significance of the interaction, and the color intensity indicates the communication probability. **d** Heatmaps depicting the outgoing (left) and incoming (right) signaling patterns of different cell types. The x-axis lists the source cell types, while the y-axis lists the target cell types. The color intensity reflects the relative strength of signaling from source to target cells. Outgoing signaling patterns show how different cell types under EMD and control conditions send signals, while incoming patterns illustrate how they receive signals

The cell communication analysis revealed distinct interaction patterns between the EMD and control groups. The EMD group showed a notable increase in communication strength, primarily between mesenchymal cell lineage and endothelial cells (Fig. 8a, left). In contrast, the control group exhibited more robust communication among endothelial cells and inflammation-related cells (Fig. 8a, right). Detailed analysis of specific cell subgroups further supported this observation, showing increased communication involving *Vtn*+ fibroblasts in the EMD group. Notably, *Vtn+* fibroblasts exhibited enhanced interactions with several cell types, including endothelial cells, fibrogenic progenitor cells, mesenchymal cells and *col1a1+* fibroblasts (Fig. S8a). Significant ligand-receptor (L-R) pairs, such as Thy1-(ltgam+ltgb2), Ptn-sdc2 between *Vtn+* fibroblasts and *Col1a1+* fibroblasts, Lamc1-(Itga6+Itgb4), Igf1-(Itga6+Itgb4) between *Vtn+* fibroblasts and fibrogenic progenitor cells, *Vtn+* fibroblasts and MSCs (Fig. 8b). In the results of the co-receptor interactions related to angiogenesis, in addition to the enhanced co-receptor interactions among endothelial cells, the co-receptor pair Esam-Esam, Cxcl1-Ackr1 between MYO&PER and endothelial cells also deserves our attention. This suggests that the interaction between pericytes and endothelial cells may play a significant role in EMD-mediated vascular remodeling (Fig. 8c). The relative information flow analysis shows the EMD group significantly increases the signaling through several key molecules, including LAMININ, PTN, and ESAM, among others (Fig. S8b). Analysis of signal patterns revealed that endothelial cells exhibited the most powerful output and input signals across multiple pathways, with signals showing enhanced LAMININ, PTN, and ESAM and so on. Similarly, *Vtn*+ fibroblasts are actively involved in signal transduction, with the output and input pathways showing enhanced THY1 and OSM signaling, respectively (Fig. 8d).

## Discussion

**Fig. 9:**
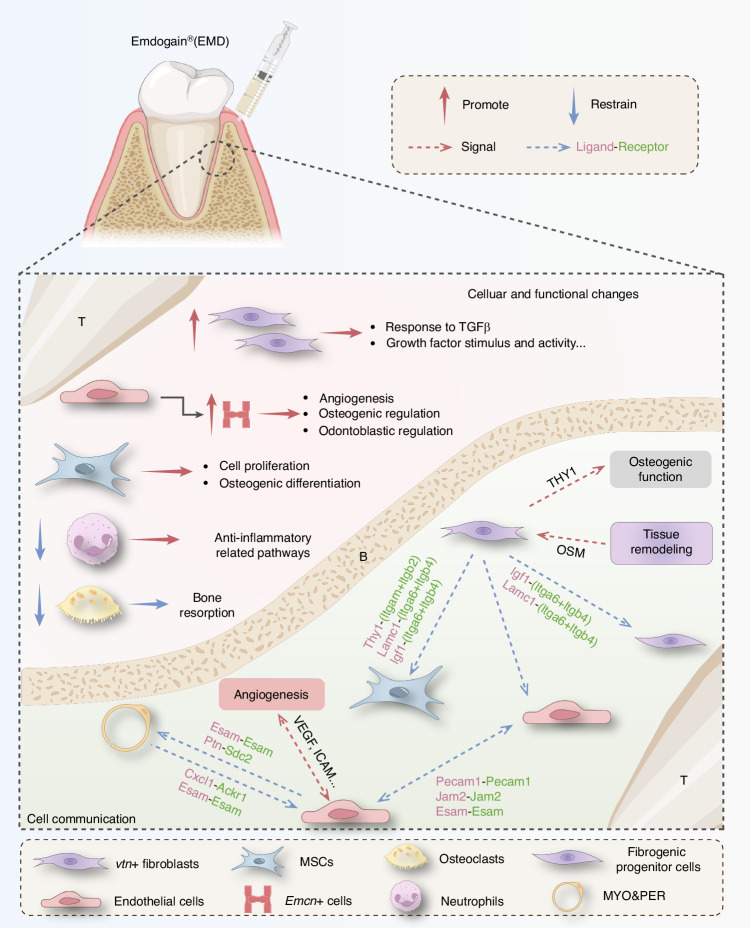
Potential cellular and mechanistic changes of EMD in promoting periodontal ligament repair. The diagram generated in part with BioRender.com. T means tooth, B means bone

This study aimed to comprehensively describe phenotypic, the cellular and molecular characteristics of EMD related to promoting periodontal repair in delayed replantation rats through experiments and scRNA-seq analysis, meanwhile, exploring its potential mechanisms. Animal experiment results demonstrated that EMD could reduce bone resorption and root resorption, decrease the rate of tooth loss, improve the stability of reimplanted teeth, and effectively promote periodontal membrane repair. To further investigate the cellular mechanisms of EMD treatment on reimplanted teeth, The rat periodontal tissue obtained 2w after orthotopic reimplantation was used for scRNA-seq. Due to the limited number of periodontal membrane samples, meeting the requirements for sc-RNA-seq loading was challenging. Using mixed test samples was more economical and straightforward, increasing biological replication and eliminating outliers. Souporcell utilized genetic variants detected in scRNA-seq reads to cluster cells, thereby more accurately illuminating changes in cell composition and gene expression profiles in periodontal tissue after EMD treatment. As the overall cell count analysis, the proportion of mesenchymal cell lines and endothelial cells increased significantly in the EMD-treated samples, suggesting that EMD promoted the proliferation or recruitment of these cell types. This observation is consistent with previous studies highlighting the role of MSCs and endothelial cells in tissue repair and angiogenesis^19–21^. Functional enrichment analysis revealed activation of pathways related to skeletal system development, collagen fiber organization, angiogenesis, and suppression of inflammatory pathways. UMAP^22^ showed AUCell scores for pathways associated with bone development, angiogenesis, collagen fiber tissue, and inflammatory response, providing additional validation for observed cellular and molecular changes, supporting the idea that EMD promoted periodontal regeneration and improved structural repair. Based on the sequencing results, this study comprehensively analyzed the cellular and molecular mechanisms. And these findings highlight the potential of EMD to promote periodontal tissue regeneration by regulating specific cell populations and activating key regenerative pathways (Fig. 9).

The analysis was conducted on four aspects: soft tissue regeneration, anti-bone resorption, blood vessels, and inflammatory reaction. We found that EMD significantly enhances the regenerative potential of mesenchymal cell lineage, particularly in promoting soft tissue regeneration. Using UMAP visualization, distinct subpopulations within the mesenchymal cell lineage were identified, highlighting the heterogeneity of these cells and their unique role in tissue repair. An increase proportion in the *Vtn+* fibroblasts subpopulation was identified in the EMD group. The *Vtn* gene, also knwn as the vitronectin gene, encodes a protein called vitronectin, a glycoprotein present in plasma and the extracellular matrix. Vitronectin plays a crucial role in various biological processes, including cell adhesion, migration, and proliferation, as well as in blood coagulation and immune responses^23^. Moreover, studies have shown that VTN protein can bind to OPG protein to regulate bone formation in mesenchymal stem cells^24^. In soft tissue regeneration, *Vtn* helps repair damaged tissues by enhancing cell-matrix adhesion, promoting extracellular matrix remodeling, cell migration, and supporting cell survival^25^. It also regulates cell response to growth factors, promoting cell proliferation and tissue repair^25–27^. Notably, the proportion of *Vtn*+ fibroblasts increased significantly in the EMD-treated group, suggesting that these cells play a crucial role in EMD-mediated regeneration. This observation was further supported by pseudo-time locus analysis, which revealed the dynamic differentiation pathway of MSCs into *Vtn*+ fibroblasts, highlighting the key GO enrichment pathways involved in this transition. Differentially expressed genes identified at various hypothetical time points are enriched in distinct pathways, reflecting the evolutionary trajectory of pathway enrichment throughout the differentiation process. Some of these pathways are associated with the extracellular matrix and possibly osteogenesis, while others are related to adhesion and collagen fiber metabolic processes involved in soft tissue regeneration. Similar results were observed in transcriptomic analyses of BMSCs, where these pathways were also enriched, suggesting that EMD might promotes the differentiation of BMSCs into two functionally distinct *Vtn*+ fibroblasts subtypes. Further transcriptome deconvolution results also showed an increase proportion in *Vtn*+ fibroblasts, reinforcing the idea that EMD promotes the expansion of this specific cell type. The increased number of proliferating cells and experimental validation further support the notion that EMD promotes tissue regeneration. When *Vtn*+ fibroblasts and MSCs were analyzed specifically, the results revealed that both cell types are rich in function associated with soft tissue regeneration. It is noteworthy that the analysis revealed EMD promotes the activation of some cytokines, especially promotes the activation of TGF-β in *Vtn+* fibroblasts, which is consistent with previous studies suggesting that TGF-β may play a unique role in EMD-mediated fibroblasts in promoting repair^28^. This phenomenon may also apply to *Vtn+* fibroblasts, or it could be even more pronounced. TGF-β regulates mesenchymal cell differentiation and extracellular matrix production, while also stimulating fibroblast proliferation and activation, all of which are crucial for tissue repair^29,30^. Furthermore, research has shown that TGF-β plays an important role in regulating tissue inflammatory responses^31^ and may also be involved in neovascularization, thereby supporting the repair process^32^. Additionally, there are reports indicating that TGF-β can influence the differentiation of stem cells^33^. These results support the idea that EMD may promote soft tissue repair by enhance the distribution and functional properties of mesenchymal cell lineages, particularly the ratio and functions of *Vtn+* fibroblasts.

Interestingly, mensenchymal cell lineage were also enriched in osteogenic pathways, suggesting that EMD has osteogenic potential. This finding deserves to be studied further to explore the dual regenerative capacity of EMD in both soft and hard tissue environments. Subsequent studies revealed the critical role of *Vtn*+ fibroblasts in the osteogenesis process and further examined the regulatory effects of EMD therapy on bone formation and resorption. Our analysis of *Vtn*+ fibroblasts demonstrated significant enrichment of pathways associated with bone formation, indicating a strong preference for osteogenesis in the EMD treatment group. This enrichment may be related to EMD’s ability to regulate the function of *Vtn*+ fibroblasts, thereby promoting bone tissue regeneration and repair. To further analyze the osteogenic potential of EMD, we utilized ALP and ARS staining, as well as q-PCR experiment of bone marrow mesenchymal stem cell osteogenic differentiation, to support this view. In vivo, DMP1 immunofluorescence staining also confirmed that EMD promotes osteogenesis^34^. To explore the regulatory role of EMD on bone resorption, we annotated the relevant subpopulations of myeloid lineage, including classical monocytes, intermediate monocytes, macrophages, non-classical monocytes, and osteoclasts. Further analysis revealed that pathways associated with bone resorption were significantly increased in the control group, consistent with enhanced osteoclast activity. In contrast, the bone resorption pathway was relatively inactive in the EMD treatment group, indicating a clearer promotion of bone formation pathways. These results suggested that EMD might exert a dual regulatory effect on bone metabolism by inhibiting bone resorption and promoting bone formation.

The analysis of endothelial cell subsets was used to reveal the effects of EMD treatment on endothelial cell subsets. It was observed that the expression of the *Emcn* gene was significantly higher in the EMD treatment group than in the control group, which was confirmed by H-type vascular immunostaining. Studies have shown that *Emcn* is closely related to the formation of H-type blood vessels^35^, which plays a crucial role in osteogenesis^36^. Differential gene expression analysis and WGCNA revealed a high concentration of blue modules significantly associated with angiogenesis pathways in the EMD treatment group. Additionally, turquoise modules were associated with osteogenesis, and green modules were associated with odontoblast differentiation, suggesting that EMD can also promote osteogenesis and dentin regeneration by upregulating the expression of endothelial-related genes.

Cluster analysis of immune cell subsets was performed to study differences in immune response after EMD treatment and revealed different subtypes of neutrophils, B cells, and T cells. However, the proportions of these subtypes did not differ significantly between the EMD treatment and control groups. Further analysis of immune cell infiltration supported this observation. These results suggest that EMD treatment did not significantly alter the overall proportions of immune cell subsets, potentially due to inflammatory infiltration caused by tooth extraction and repair^37^. However, this treatment may affect the functional state of immune cells differently. Therefore, a further GSVA analysis was performed^38^. The results indicated higher expression of inflammation-related pathways in the control group, suggesting a more robust inflammatory response without EMD treatment. GO enrichment analysis of all immune cells in the EMD-treated group showed significant enrichment of pathways associated with the negative regulation of leukocyte activation and cytokine production, suggesting that EMD treatment may promote anti-inflammatory responses. Although EMD treatment did not significantly change the overall proportions of immune cell subsets, it may regulate inflammatory states by activating anti-inflammatory pathways^39^. These findings suggested that EMD might create a more favorable environment for tissue repair during periodontal regeneration by reducing the inflammatory response.

Finally, cell communication analysis revealed significant differences between the EMD-treated and control groups. In the EMD group, the intensity of communication between mesenchymal cell lineage and endothelial cells was significantly enhanced. Conversely, in the control group, communication between immune-related cells was more pronounced. Detailed analysis of specific cell subpopulations supported this observation. The bubble plot highlights the interactions of ligands and receptors associated with *Vtn+* fibroblasts, particularly the Lamc1-(Itga6+Itgb4), Igf1-(Itga6+Itgb4) interactions between mesenchymal stem cells and Vtn+ fibroblasts, which are more pronounced in the EMD group. Among the angiogenesis-related signals, in addition to the enhanced interactions between endothelial cells, it is noteworthy that the interactions between MYO&PER and endothelial cells are also strengthened, with prominent ligand-receptor pairs including Ptn-Sdc2, Esam-Esam. and Cxcl1-Ackr1. Research has shown that the interactions between pericytes and endothelial cells are crucial in regenerative environments, and there is a significant correlation between the function of pericytes and angiogenesis^40^. Pericytes provide stability to blood vessels and regulate endothelial cell proliferation^41^. Our analysis suggests that EMD may have a promoting effect in this regard. The increased information flow of LAMININ^42^, PTN^43^, IGF^44^ and other pathways promoting cell adhesion, regeneration and migration suggest that EMD group may promote tissue regeneration and repair by activating these functional processes. Signal pattern analysis revealed the activation of VEGF^45^, ICAM^46^ and other signaling pathways in endothelial cells, suggesting that EMD plays a significant role in promoting and regulating angiogenesis. Similarly, the active involvement of *Vtn+* fibroblasts in enhanced THY1^47^ signaling was primarily linked to the osteogenic potential of stem cells, while OSM^48^ signaling was predominantly associated with tissue remodeling and regeneration. This shift in signal dynamics further supports the notion that EMD treatment promotes pro-regenerative communication networks primarily driven by interactions between mesenchymal cell lineage and endothelial cells.

To sum up, this study combines advanced scRNA-seq technology. Using the delayed tooth replantation model, the cellular mechanism and communication mode of EMD in promoting periodontal membrane repair, reducing root resorption, promoting angiogenesis and regulating inflammatory response were comprehensively analyzed. This study builds upon previous research and demonstrates specific progress in understanding EMD’s effects. However, there are limitations, such as a limited sample size and the absence of long-term follow-up studies. Future research should involve larger sample sizes and extended follow-up periods to verify these findings. Additionally, we utilized single-cell sequencing to explore potential mechanisms of action and conducted cytology preliminary validations on its effects and key cellular changes. However, this study only provides insights into the complex mechanisms involving related molecular pathways and cellular interactions. Future research will require further experimental validation to investigate its specific role in periodontal regeneration to provide a theoretical foundation for future treatment strategies.

## Materials and methods

### Animal experiment

#### The delayed replantation model of Sprague-Dawley (SD) rats was established

Six-week-old SD rats were obtained from the Laboratory Animal Center of Chongqing Medical University. The rats were housed in a specific pathogen-free environment with a 12 h light/dark cycle and had ad libitum access to food and water. All animal experiments in this study were approved by the Research Ethics Committee of the Stomatological Hospital of Chongqing Medical University (CQHS-REC-2023110).

The SD rats were anesthetized using 1% pentobarbital sodium at a dosage of 30 mg/kg. The experiment was conducted with two groups: the tooth extraction and replantation with EMD treatment group and the tooth extraction and replantation without treatment group, each comprising 10 rats. Tooth extraction surgery was performed on the left side of each rat, while the right side served as a normal control.

The maxillary first molar was extracted using the tip of a periodontal probe, and the extracted molar was stored in normal saline for 25 min. The molar was then treated with Emdogain^®^ (EMD, Straumann^®^, Switzerland) and normal saline for 5 min before replantation. After gently rinsing the alveolar socket with normal saline, the treated molar was replanted in situ. The buccal sides of the first and second molars were fixed with resin. Samples were collected at 4 weeks and 8 weeks, respectively, and the rates of tooth loosening and loss were assessed during sampling.

#### Micro-CT analysis

Following euthanasia, the maxilla with teeth was extracted and fixed overnight in 4% paraformaldehyde (Sigma-Aldrich, USA). The samples were then scanned using a micro-CT (Scano Medical, Switzerland) at 50 kV and 80 μA. The resulting micro-CT data were reconstructed and analyzed. The region of interest was defined as the alveolar bone surrounding the root bifurcation of the maxillary first molar, and the bone volume fraction (BV/TV) in this area was quantified. Additionally, the height from the cementoenamel junction (CEJ) to the alveolar bone crest (ABC) was measured to evaluate the extent of alveolar bone loss^32^.

#### Histological stain analysis

The maxilla containing teeth was dissected after the rats were euthanized. It was then fixed in 4% paraformaldehyde overnight, rinsed with distilled water after fixing, decalcified in 10% EDTA for two months, and then thoroughly rinsed with tap water. The well-decalcified tissues were dehydrated with graded ethanol, embedded in paraffin wax, and sliced into 6 micron slices for H&E staining (Mengbio, China). The image was obtained by the VS200 full-mount scanning system (Olympus VS200, Japan), and the root was divided into 12 equal parts as observation points to calculate the root absorptivities. The ratio of periodontal membrane width between the experimental group and the normal group was used to evaluate the periodontal membrane repair.

#### Immunofluorescence staining

After fixed, decalcified and rinsed with running water using the same method, 6 µm slices were prepared by dewatering with graded ethanol, embedding paraffin, and then treated with 0.3% Triton X-100 (Sigma, USA) for 15 min. Then goat serum was closed at 37 °C for 30 min and exposed to primary antibody at 4 °C overnight. After washing with PBS for three times, coated with secondary antibody and incubated at 37 °C for 1 h. After washing with phosphate-buffered saline (PBS) for three times, the sections were sealed with an anti-quenching agent containing DAPI. Images were captured using a fluorescence microscope (Zeiss, Germany) and analyzed using the Image software. The antibodies used were anti-DMP1 (Abcam, USA), anti-CD31 (Abcam, USA) and anti-EMCN (Protein Tech, USA).

#### TRAP staining

Tissue acquisition, fixation, decalcification, embedding, and sectioning. Stain the tissue using the TRAP staining kit (Beyotime, China). Count cells using ImageJ analysis.

### Cell culture

#### Extraction and culture of BMSCs

Bone marrow was extracted from the femur and tibia of 2-week-old SD rats under aseptic conditions. The marrow cavity was rinsed with PBS to obtain the bone marrow cell solution. Mononuclear cells were subsequently isolated from this solution using a density gradient centrifugation technique. The purified cells were then cultured in α-MEM supplemented with 10% fetal bovine serum (FBS) and 1% penicillin-streptomycin (P/S) in an incubator maintained at 37 °C with a 5% CO_2_ atmosphere. After 24 h, non-adherent cells were eliminated, the culture medium was replenished, and the medium was thereafter replaced every three days^49^.

#### CCK8 test

The cells were treated when the cell confluence reached 70%–80%, and the tests were carried out on the 1st, 3rd and 5th day after treatment. At each point in time, add 10 microliters of CCK8 solution to each well and stir gently. The cells were then incubated for two to four hours. The optical density (OD) of each hole at 450 nm wavelength was measured by microplate spectrophotometer, and the data were analyzed statistically.

#### Osteogenic induction

BMSCs were derived from 4-week-old SD rats. These cells were maintained in a temperature-controlled incubator set at 37 °C with a 5% CO_2_ environment, utilizing a growth medium that comprised 10% FBS and 1% P/S. The culture medium was replenished every three days, and the cells were passaged using 0.25% Trypsin-EDTA when they achieved 80%–90% confluence. Third-passage BMSCs were plated in a 6-well plate at a concentration of 5 000 cells per square centimeter. After 24 h, the culture medium was substituted with an osteogenic differentiation medium, which consisted of α-MEM, 10% FBS, 10 mmol/L β-Glycerophosphate, 50 μg/mL Ascorbic Acid, and 10 nmol/L Dexamethasone. In the experimental cohort, EMD was incorporated into the osteogenic differentiation medium, and this medium was replaced every three days over a period of 7 to 21 days.

#### ALP staining

On the 7th day of osteogenic induction, cells were removed and washed 3 times with PBS. Cells were fixed with 4% paraformaldehyde for 15 min. An alkaline phosphatase detection kit (Beyotime, China) was used to stain according to the instructions, and the ALP activity was observed.

#### Alizarin red staining

On the 21st day of osteogenic induction, cells were removed and washed 3 times with PBS. Cells were fixed with 4% paraformaldehyde for 15 min. Dye with 2% alizarin red solution (pH 4.2) for 20 min, remove the unbound dye and wash with PBS 3 times. The formation of mineralized nodules was observed under a microscope-Alizarin red after dissolution and staining with cetylpyridine chloride (Maclean, China). The absorbance of the solution at OD405 nm was measured by spectrophotometer, and the mineralized water level was quantitatively analyzed.

#### q-PCR detected the expression of osteogenic factors

On the seventh day of osteogenic induction, cells were harvested. RNA was extracted using a RNA extraction kit (Beyotime, China) and subsequently reverse-transcribed into cDNA with a reverse transcription kit (Takara, Japan). q-PCR was conducted with the SYBR Green Q-pcr kit (Takara, Japan). Specific primers for the target genes and the internal reference gene (*GAPDH*) were designed and synthesized. The reaction mix consisted of 10 μL SYBR Green Mix, 1 μL primer, 1 μL cDNA template, and 8 μL nuclease-free water, totaling 20 μL. The thermal cycling conditions were: 95 °C for 3 min for pre-denaturation, followed by 40 cycles of 95 °C for 10 s for denaturation, and 60 °C for 30 s for annealing. The 2^(-ΔΔCt) method was employed to analyze relative gene expression, and the expression levels of osteogenic genes (*Runx2*, *OPN*, *OCN*, *BSP*) were statistically evaluated^50^.

### Sequencing analysis

#### scRNA-seq using the 10X Genomics platform

The periodontal tissue of rats was obtained and treated with enzymes, isolated single cells, and the cell number and activity were detected to ensure a more than 80% cell survival rate. Using a microfluidic chip from 10X Genomics, individual cells were captured into tiny reaction chambers. A cell suspension was mixed with oil to form an oil-in-water droplet. Each drop contained a single cell and a small piece of barcoded reverse transcription reagent. Reverse transcription was performed in the droplet to convert mRNA into cDNA. Each cell’s cDNA was labeled with a unique barcode for subsequent analysis. The synthesized cDNA was amplified by PCR to obtain sufficient quantities for sequencing. High-throughput sequencing was performed using the Illumina sequencing platform to obtain scRNA-seq data. Sequencing data typically contained gene expression information for each bar code.

#### ScRNA-seq raw data processing

Raw sequencing data obtained from the 10X Genomics platform were processed with CellRanger (version 6.0.2) to produce gene expression matrices. CellRanger handled the demultiplexing process^51^, barcode processing, and alignment to the reference genome on a Linux platform.

#### ScRNA-seq sample demultiplexing

Mixed samples were demultiplexed using the souporcell algorithm (https://github.com/wheaton5/souporcell), executed within a Singularity container (version 4.1.3) on the Linux platform^17^. Souporcell separated different sample origins within the mixed scRNA-seq data. Subsequently, filtering was applied to include only single droplets and cells with unambiguous assignment, ensuring accurate attribution of cells to their respective samples.

#### Data processing and quality control

ScRNA-seq data were analyzed using Seurat (version 5.1.0) in R (version 4.4.1)^52^. Quality control involved filtering out cells with low RNA counts, elevated mitochondrial gene expression, and other outliers. Cells with fewer than 200 genes or those expressed in fewer than three cells were excluded. Additionally, cells exceeding 5% mitochondrial content were removed.

#### Normalization and scaling

The Seurat NormalizeData function was used to normalize the data, followed by scaling with ScaleData. Dimensionality reduction was achieved through Principal Component Analysis (PCA), with the top 25 components selected for further clustering and visualization.

#### Clustering and visualization

Clustering was performed using the Louvain algorithm via Seurat’s FindClusters function, and visualization was done with UMAP (RunUMAP). For specific subpopulations, t-SNE was also employed to explore additional data structure insights.

#### Gene set variation analysis (GSVA)

GSVA was performed using the GSVA package (version 1.52.3) with gene sets sourced from the Molecular Signatures Database (MsigDB). This analysis computed GSVA scores to evaluate pathway activity across different cell populations.

#### Scoring and visualization

The computed GSVA scores were further refined using the AUCell package (version 1.26.0) to assign activity levels to individual cells. UMAP was subsequently utilized for visualization.

#### Pseudotime analysis

Pseudotime trajectory analysis was performed with the monocle package (version 2.32.0). Cell differentiation paths were mapped, and the progression of individual cells along these paths was visualized using the ClusterGVis package (version 0.1.1).

#### Enrichment analysis

Functional enrichment analysis utilized the clusterProfiler package (version 4.12.0). GOBP pathway scores were computed with Seurat’s AddModuleScore function, followed by two-sided statistical tests. The enrichment outcomes were visualized through chord diagrams generated by the circlize package (version 0.4.16).

#### Weighted gene Co-expression network analysis (WGCNA)^53^

Co-expression networks were built using the WGCNA package (version 1.72-5) with a soft threshold power of 3. Modules were identified and their functional enrichment evaluated using the clusterProfiler package (version 4.12.0).

#### Immune infiltration analysis

Immune Profiling: Immune cell infiltration was analyzed using the CIBERSORTx tool (https://cibersortx.stanford.edu).^54^ with the LM22 immune dataset to estimate the relative abundance of immune cell types from scRNA-seq data.

#### Cell communication analysis

Cell-cell communication networks were inferred using the CellChat package, identifying significant ligand-receptor interactions between different cell populations.

#### Transcriptome sequencing of BMSCs

After 5 days of culture with EMD and blank control medium, BMSCs reached the logarithmic growth stage. The cells were cleaned with PBS, and total RNA was extracted using an RNA extraction kit (Trizol) to ensure the integrity and purity of RNA. The constructed libraries were sequenced using a high-throughput sequencing platform such as Illumina NovaSeq.

#### Bulk RNA sequencing raw data processing

Raw transcriptomic data were processed on a Linux platform. Quality control involved using trim galore (version 0.6.7) to remove adapters and low-quality bases. The reads were aligned to the reference genome with STAR (version 2.7.10a). Gene expression levels were quantified by featureCounts (version 2.0.1), producing the expression matrix.

#### Transcriptomic deconvolution

Transcriptomic deconvolution was performed using the BisqueRNA package (version 1.0.5) to estimate cell type proportions in mRNA-seq data^55^. After deconvolution, the statistical significance of cell proportion differences was assessed using one-sided statistical tests.

### Statistical analysis

Two-sided statistical tests were used to evaluate the significance of most comparisons, including cell quantity comparisons and GOBP pathway scores. One-sided statistical tests were employed to analyze cell proportion differences after transcriptomic deconvolution. The specific statistical tests and criteria used for pathway enrichment, cell communication, pseudotime analysis, and other algorithms follow the standards and descriptions provided by each respective package. Detailed descriptions of the statistical methods and parameters used can be found in the documentation provided by these packages.

The results of cell and animal experiments were statistically analyzed in the software GraphPad Prism (version 9.4.1), each experiment was repeated at least 3 times, and all data were displayed as mean ± SD unless otherwise noted. Variance (ANOVA) analysis or two-tailed Student’s *t* test were used for data analysis, and Wilcoxon signed rank test was used for rank data. When comparing two sample’s proportions, Fisher’s Exact Test was employed. (*P < 0.05; **P < 0.01; ***P < 0.001, as shown in the full text).

## Supplementary information

Supplementary 8 figures and 9 tables

## Data Availability

The data that support the findings of this study are included in the manuscript and the supplementary information.
