# Supplementary material for: Single-cell sequencing systematically analyzed the mechanism of Emdogain on the restoration of delayed replantation periodontal membrane
Source: Int J Oral Sci. 2025 Apr 17;17:33. doi: 10.1038/s41368-024-00345-5 (PMC12006293; doi:10.1038/s41368-024-00345-5)
Supplement: Supplementary file 1 — Supplementary 8 figures and 9 tables [file 41368_2024_345_MOESM1_ESM.pdf]

Supplementary 8 figures and 9 tables:

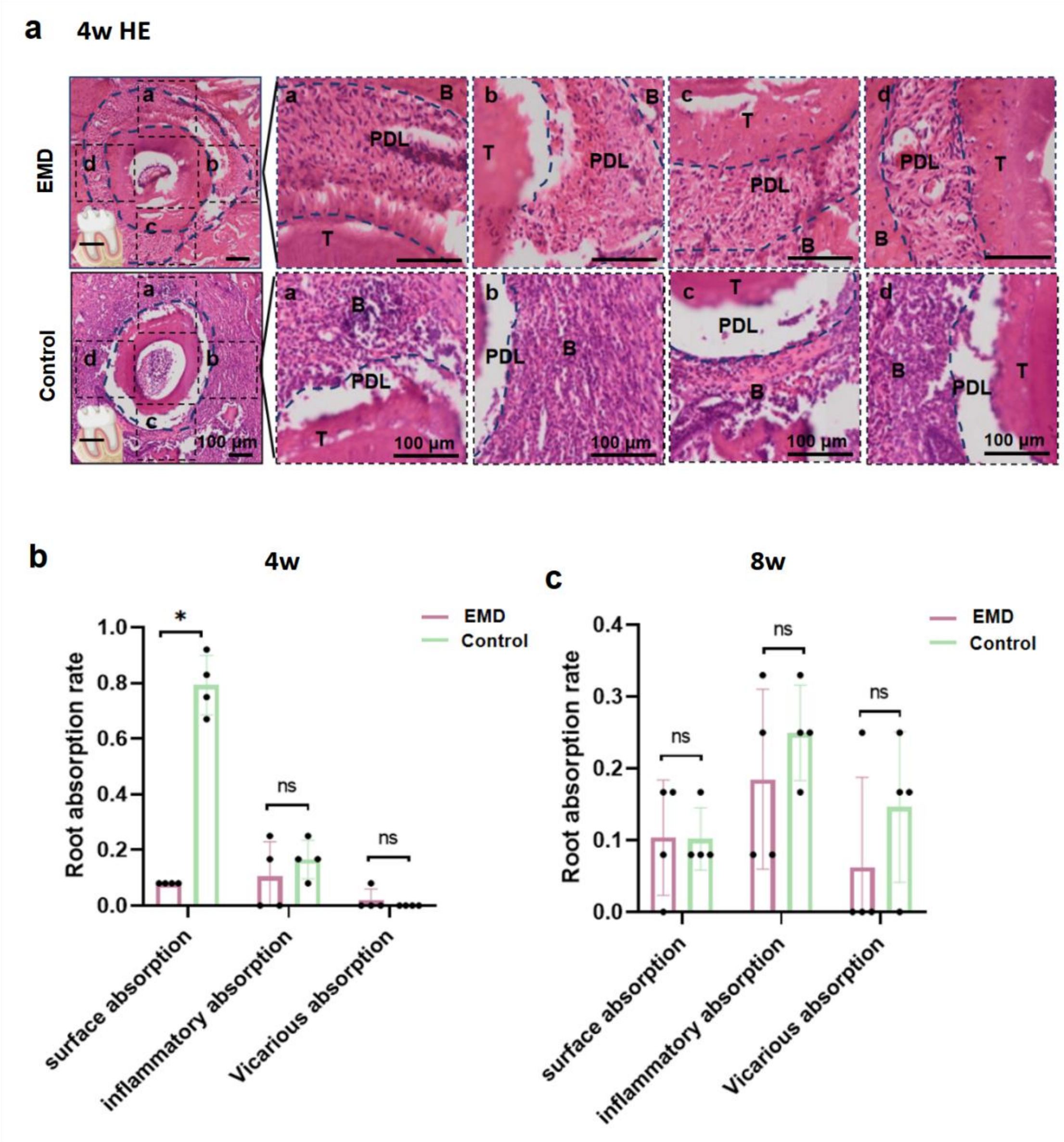

Fig. S1

a. HE staining results of 4w. T means tooth, B means bone, P means parodontium.

b. The root absorption rate of 4w after replantation was measured.

c. The root absorption rate of 8w after replantation was measured.

Statistical significance is indicated by *$P < 0.05$, **$P < 0.01$, ***$P < 0.001$, and ns (not significant)

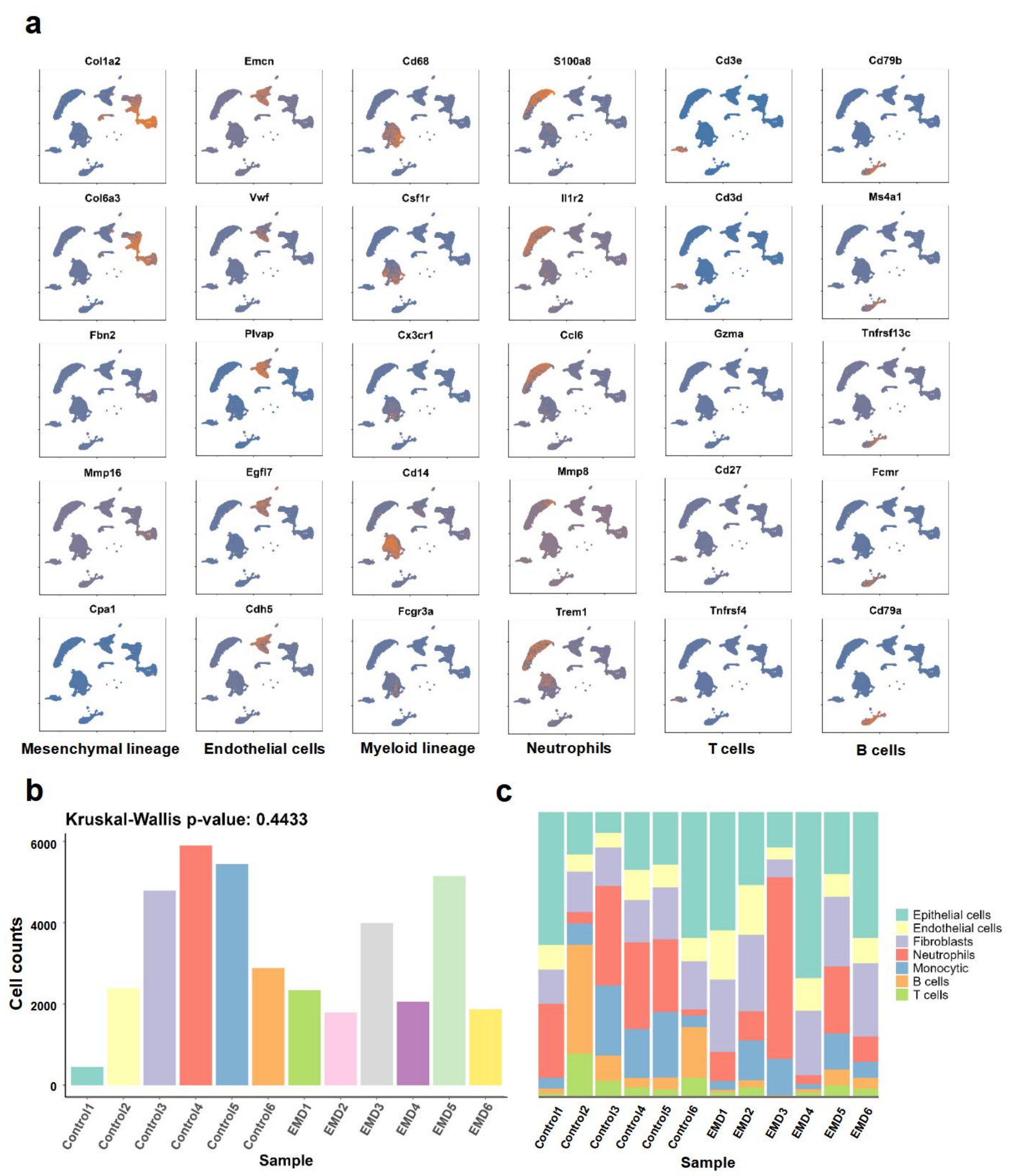

Fig. S2

a. UMAP plots showing the expression of marker genes for different cell types.

b. Bar plot displaying the cell counts for each sample after deconvolution of mixed samples. The Kruskal-Wallis test *P*-value (0.4433) indicates that the differences in cell counts among the samples are not statistically significant.

c. Stacked bar chart illustrating the proportions of various cell types in each sample.

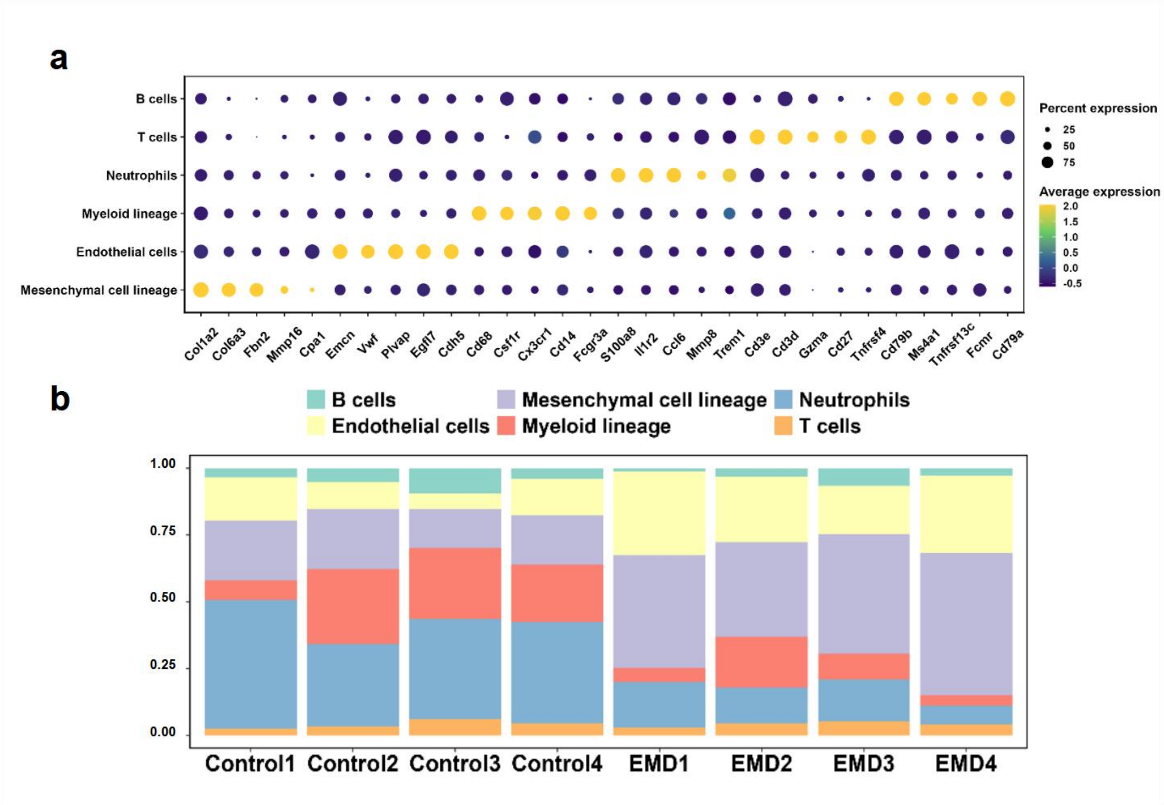

Fig. S3

a. Dot plot representing the expression levels and percentages of marker genes across different cell types in control and EMD-treated samples. The color intensity represents average expression levels, and the size of the dots indicates the percentage of cells expressing each marker gene.

b. Stacked bar plot showing the distribution of different cell types across control and EMD-treated samples. Each bar represents a different sample, and colors correspond to different cell types.

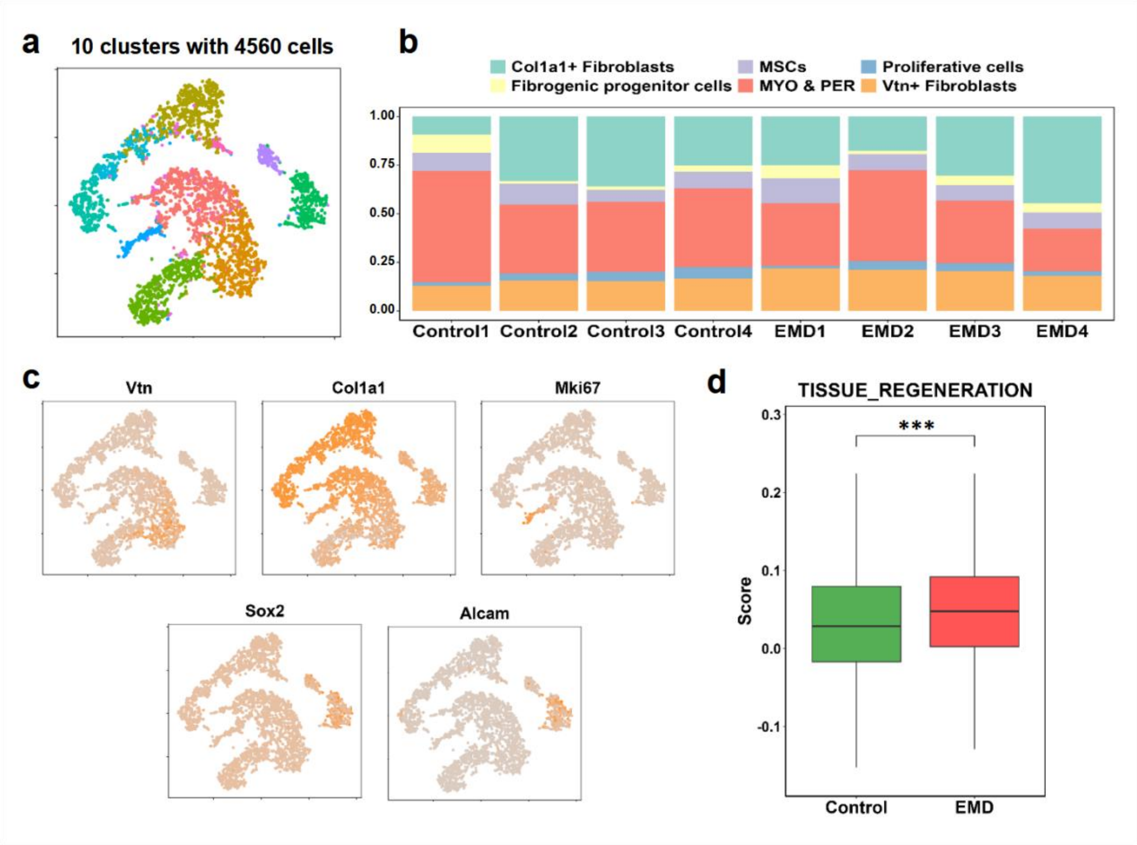

Fig. S4

a. t-SNE plots showing the clustering of 4560 cells into 10 distinct subpopulations.

b. Stacked bar plots illustrating the proportion of different fibroblast subtypes under control and EMD conditions across four different controls and EMD samples. Each color represents a distinct fibroblast subtype.

c. Feature plots demonstrating the expression of key marker genes (*Vtn*, *Col1a1*, *Mki67*, *Sox2*, *Alcam*) across the fibroblast subpopulations.

d. Box plot of GOBP pathway scores related to tissue regeneration for EMD-treated and control groups. The box plot indicates a significant increase in tissue regeneration pathways in EMD-treated cells compared to controls.

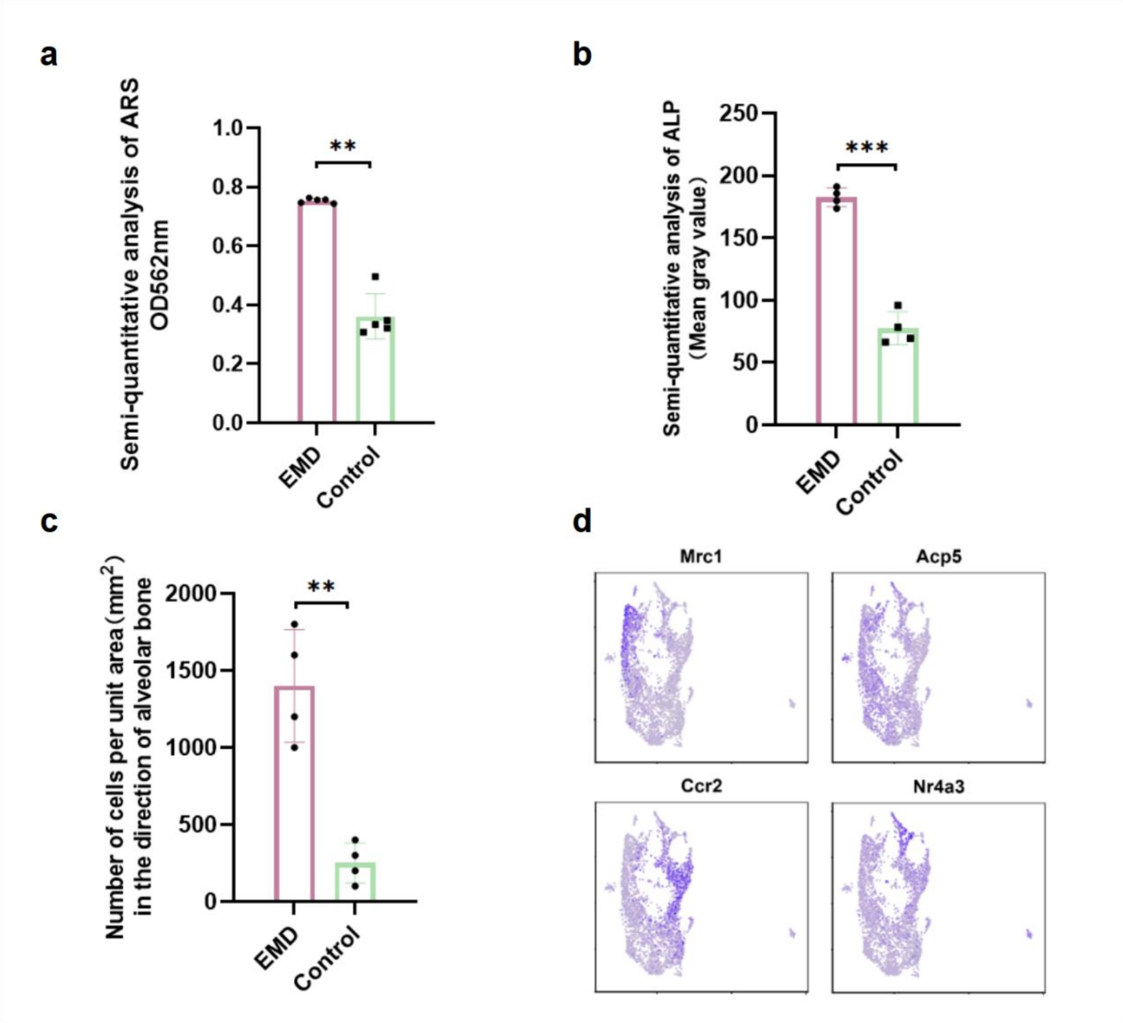

Fig. S5

a. Semi-quantitative results of alizarin red staining.

b. Semi-quantitative results of ALP staining.

c. Statistical results of immunofluorescence staining of cells expressing DMP1.

d. UMAP plots showing the expression of marker genes for myeloid lineage.

Statistical significance is indicated by **P* < 0.05, ***P* < 0.01, ****P* < 0.001, and ns (not significant).

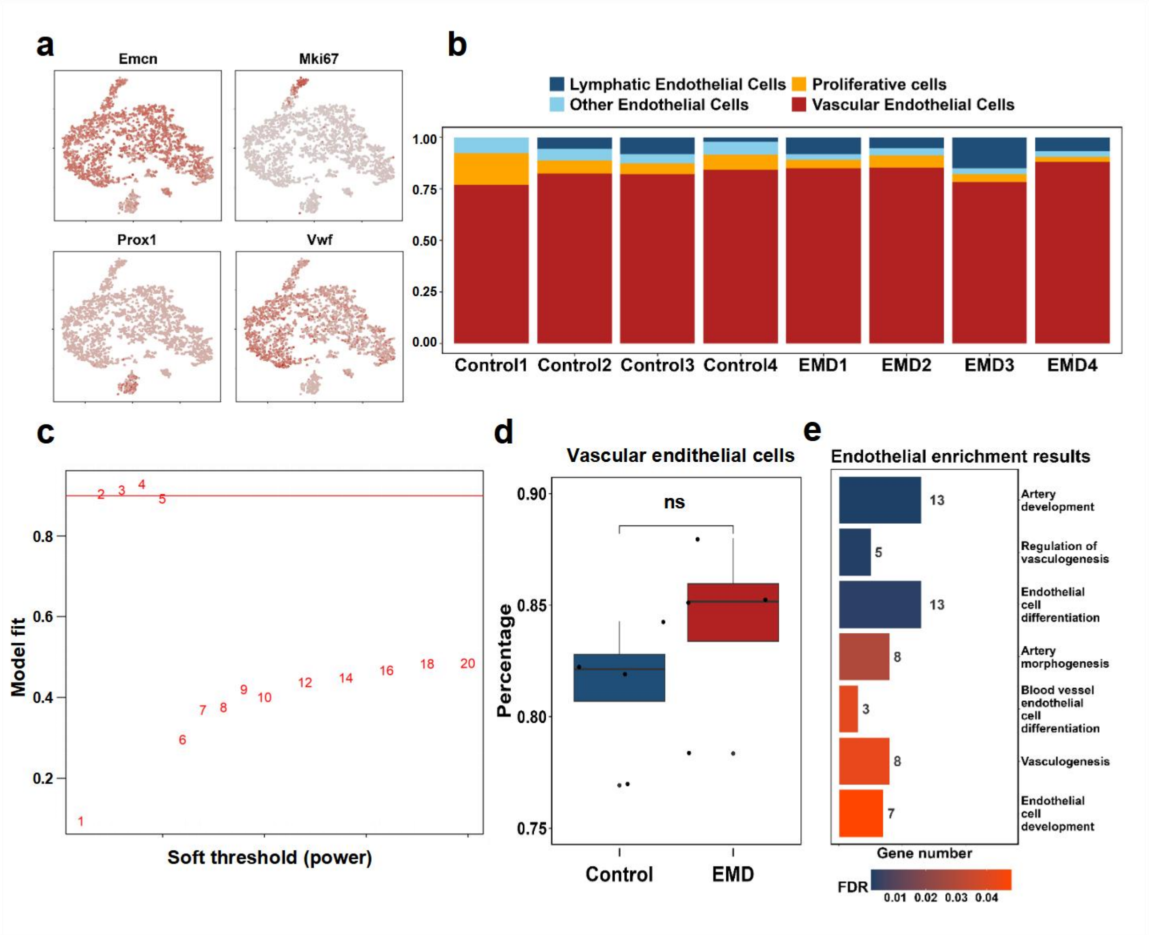

Fig. S6

a. Feature plots showing the expression of marker genes (*Emcn*, *Mki67*, *Prox1*, *Vwf*) across the endothelial cell clusters.

b. Bar plot showing the distribution of cell types across different control and EMD treatment groups.

c. Plot from WGCNA analysis showing the basis for selecting the soft threshold power. The plot indicates the scale-free topology model fit as a function of the soft threshold power.

d. Box plots comparing the proportions of vascular endothelial cells between EMD and control groups.

e. Bar plot displaying the enriched GO terms related to angiogenesis and endothelial cell differentiation processes, with color indicating the adjusted *P*-value.

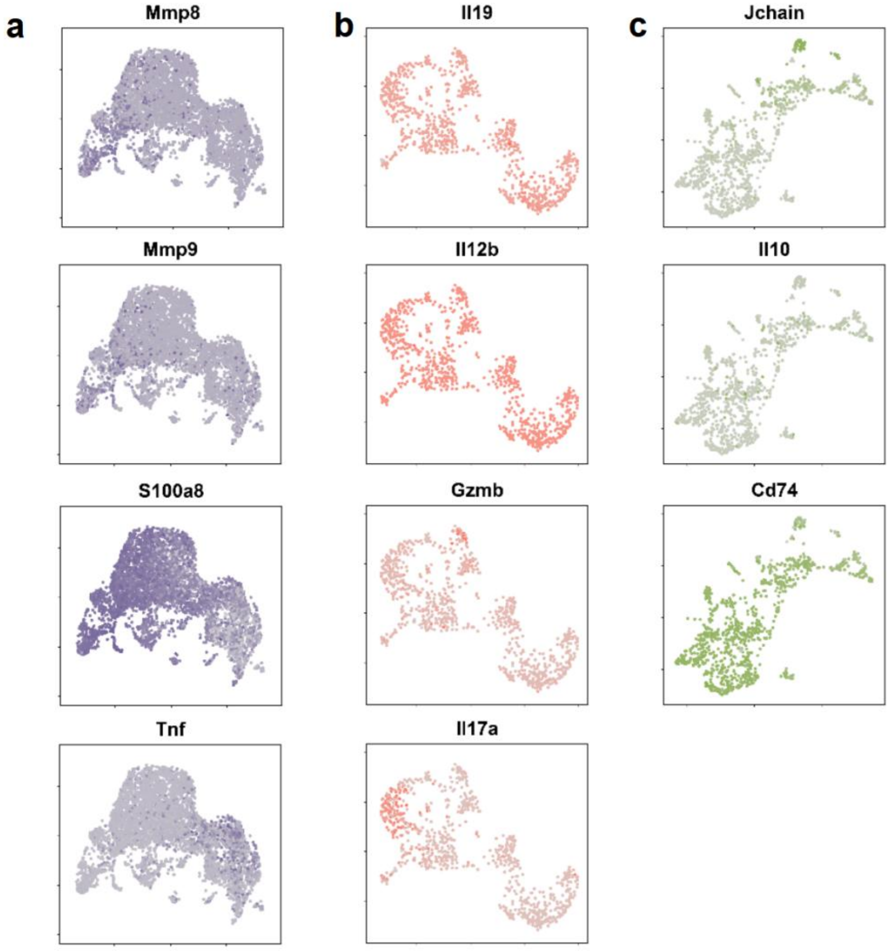

Fig. S7

a. UMAP plots showing the expression of marker genes for neutrophils.

b. UMAP plots showing the expression of marker genes for T cells.

c. UMAP plots showing the expression of marker genes for B cells.

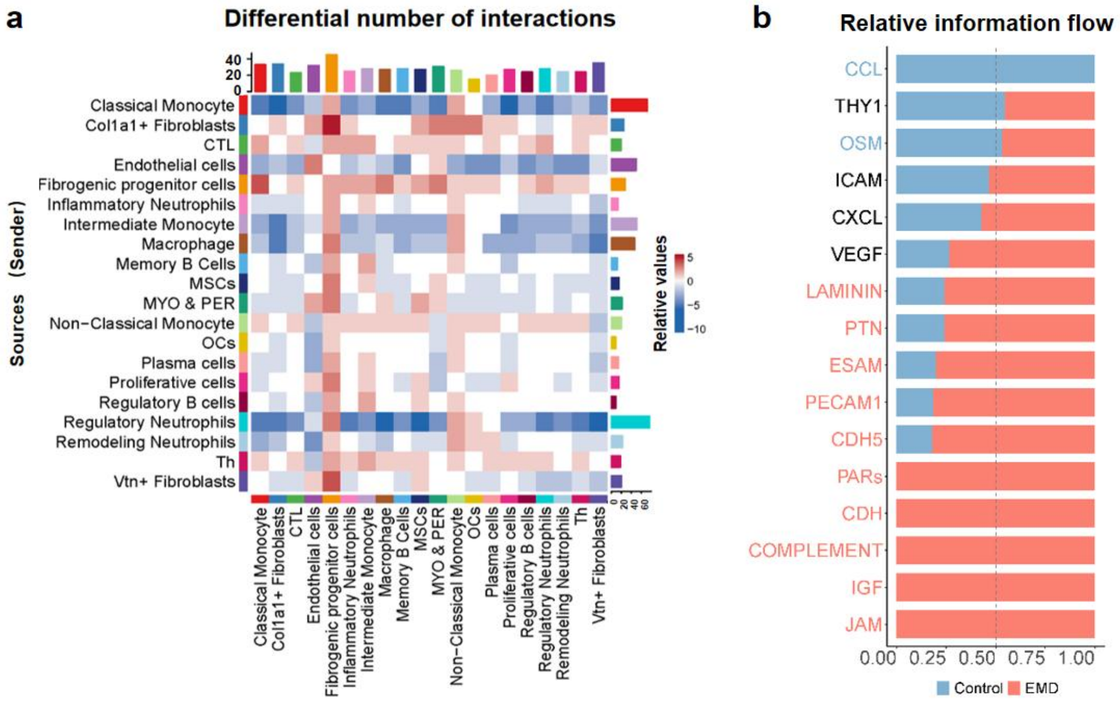

Fig. S8

a Heatmap showing the differential number of interactions between EMD and control conditions. The x-axis and y-axis represent the target and source cell types, respectively. Red and blue colors indicate an increase and decrease in interactions, respectively, in EMD compared to control. The color intensity reflects the magnitude of the difference.

b Bar plot showing the relative information flow of various signaling pathways under both EMD and control conditions. Each bar represents a signaling pathway, with the length of the bar corresponding to the relative information flow. Blue and red colors denote control and EMD conditions, respectively.

Table. S1 Statistical results of root resorption

Time	Group	surface absorption	inflammatory absorption	Vicarious absorption
4w	EMD	0.08 ± 0*	0.10 ± 0.13	0.02 ± 0.04
4w	Control	0.79 ± 0.11	0.17 ± 0.07	0 ± 0
8w	EMD	0.10 ± 0.08	0.19 ± 0.13	0.06 ± 0.13
8W	Control	0.10 ± 0.04	0.25 ± 0.07	0.146 ± 0.10

Result showed as mean ± standard devision

Table. S2 Q-pcr primer sequence

Target cDNA	Primer sequences (5’-3’)
GAPDH	TATGACTCTACCCACGGCAAG<br>TACTCAGCACCAGCATCACC
BSP	ATCCGTGCCACTCACTCACTTG<br>AGTAGCGTGGCCGGTACTTAAAGA
OCN	ACAAGTCCCACACAGCAACTC<br>GCCAGGTCAGAGAGGCAGAA
Runx2	CATGGCCGGGAATGATGAG<br>TGTGAAGACCGTTATGGTCAAAGTG
OPN	CCAGCCAAGGACCAACTACA<br>CTGCCAAACTCAGCCACTTTC

Table. S3 Results of the Sourporcell

barcode	status	assignment	cluster0	cluster1	cluster2	Samples
AAACCCAAGATTAGTG-1	singlet	2	-559.0660199	-445.5693642	-212.8649062	EMD1
AAACCCAAGTGCTCGC-1	singlet	2	-2925.038064	-2309.952784	-997.380121	EMD1
AAACCCACAATTTCGG-1	singlet	0	-256.2428459	-699.9579125	-708.2455686	EMD1
AAACCCACAGCCATTA-1	singlet	2	-367.260557	-356.6755901	-151.5007272	EMD1
AAACCCACAGGACTTT-1	singlet	2	-5721.320768	-4719.424698	-1735.43964	EMD1
AAACCCACATCGGCCA-1	singlet	1	-816.4217355	-292.740112	-645.2513363	EMD1
AAACCCAGTAAGGCCA-1	singlet	2	-3998.714515	-3292.360379	-1351.31209	EMD1
AAACCCAGTGAGATCG-1	singlet	2	-2793.435342	-2249.288213	-932.5710044	EMD1
AAACCCAGTGCATTTG-1	singlet	2	-1831.004751	-1438.902432	-594.954826	EMD1
AAACCCAGTTGGAGGT-1	singlet	2	-554.0807971	-495.3215836	-217.4670686	EMD1
AAACGAAAGCCTCGTG-1	singlet	2	-173.1508013	-200.3787831	-73.94289903	EMD1
AAACGAAAGTAACCTC-1	singlet	2	-405.5875366	-396.3402748	-174.583426	EMD1
AAACGAACAGACCCGT-1	singlet	0	-1015.196949	-2857.839987	-2825.681119	EMD1
AAACGAACATGCAGCC-1	singlet	0	-343.9956542	-1366.918029	-1284.433295	EMD1
AAACGAACATGTAACC-1	singlet	1	-4996.33785	-1591.432167	-3889.804858	EMD1
AAACGAAGTCGCTGCA-1	singlet	2	-262.0969233	-208.6277376	-109.7232983	EMD1
AAACGAAGTGTGACCC-1	singlet	2	-388.8953517	-347.1963378	-189.8763049	EMD1
AAACGAATCATTGTTC-1	singlet	2	-510.872723	-509.8622517	-249.4775159	EMD1
AAACGAATCGACGACC-1	singlet	1	-438.0519371	-184.7516128	-453.9811808	EMD1
AAACGAATCGGAGATG-1	singlet	1	-4162.867324	-1228.364998	-3091.557948	EMD1
AAACGAATCTATTGTC-1	singlet	0	-310.583348	-859.3629395	-844.4067238	EMD1
AAACGCTAGGGCTGAT-1	singlet	2	-301.5195271	-293.042321	-153.2724768	EMD1
AAACGCTCAGCAGTTT-1	singlet	2	-571.2003464	-479.1380783	-251.6388723	EMD1
AAACGCTGTGATAGTA-1	singlet	2	-1285.131712	-1251.77746	-480.9452931	EMD1
AAACGCTGTGCGCTCA-1	singlet	1	-631.4543086	-241.2018339	-572.6964738	EMD1
AAACGCTTCAGCAGAG-1	singlet	2	-860.6872681	-703.4970309	-304.2708735	EMD1
AAACGCTTCCTATGGA-1	singlet	0	-607.2347354	-1935.293381	-1694.430854	EMD1
AAACGCTTCGCATAGT-1	singlet	1	-3301.556459	-1054.469894	-2444.822186	EMD1
AAACGCTTCTATCCAT-1	singlet	2	-817.271073	-703.3175584	-315.4759691	EMD1
AAAGAACAGATACAGT-1	singlet	2	-552.790709	-500.7901015	-227.9783802	EMD1
AAAGAACAGCTATCCA-1	singlet	2	-557.8134714	-518.0077134	-240.5018576	EMD1
AAAGAACCAGTGTGCC-1	singlet	1	-118.0322889	-34.27758575	-76.70598023	EMD1
AAAGAACCATCATCTT-1	singlet	2	-2350.123834	-1955.404216	-755.0464706	EMD1
AAAGAACGTCAAACGG-1	singlet	0	-1883.148427	-7900.660926	-7127.878421	EMD1
AAAGAACTCGAGTTGT-1	singlet	0	-240.1741622	-725.8090977	-708.7805611	EMD1
AAAGGATAGCGTCAAG-1	singlet	1	-875.9388661	-278.2303447	-679.362796	EMD1
AAAGGATAGCTGCGAA-1	singlet	2	-296.4641966	-279.8083936	-125.4567548	EMD1
AAAGGATCAAGAGGCT-1	singlet	0	-528.0643869	-1496.801241	-1450.613404	EMD1
AAAGGATCAAGTACCT-1	singlet	0	-1369.187993	-4053.758498	-3963.119935	EMD1
AAAGGATGTATTTCGG-1	singlet	2	-551.1791233	-457.8171681	-219.9765623	EMD1
AAAGGATGTCCATCTC-1	singlet	2	-2137.762974	-1550.333317	-640.9026206	EMD1
AAAGGATGTCGGAAAC-1	singlet	1	-5750.312048	-1481.8135	-4379.657951	EMD1
AAAGGATGTTCCGTTC-1	singlet	2	-5645.061119	-4783.95711	-1663.912995	EMD1
AAAGGGCAGCCTATTG-1	singlet	2	-326.2306372	-252.1756591	-122.7947625	EMD1
AAAGGGCAGTAACCGG-1	singlet	0	-55.97634464	-199.0247638	-211.6544382	EMD1
AAAGGGCAGTTCATGC-1	singlet	1	-3191.057627	-705.4273398	-2650.050423	EMD1
AAAGGGCCACAGCGCT-1	singlet	2	-519.9304022	-423.9265978	-171.5185265	EMD1
AAAGGGCGTAGTCTTG-1	singlet	2	-329.1295844	-256.0450787	-129.3851556	EMD1
AAAGGGCGTATACCCA-1	singlet	1	-692.0462005	-246.8850494	-551.6531729	EMD1
AAAGGGCGTATCGTTG-1	singlet	0	-561.210061	-1765.284872	-1719.477791	EMD1
AAAGGGCGTCCATCTC-1	singlet	0	-120.0555483	-329.676259	-392.6241153	EMD1
AAAGGGCGTTGTTGTG-1	singlet	1	-2296.877618	-653.1115191	-1745.470614	EMD1
AAAGGGCTCCGATGCG-1	singlet	1	-5939.402223	-1520.570628	-4559.26259	EMD1
AAAGGTAAGATCGCTT-1	singlet	2	-400.7246225	-348.2520001	-169.3769574	EMD1
AAAGGTAAGCACCAGA-1	singlet	0	-1228.680384	-3534.070508	-3306.339177	EMD1
AAAGGTAAGTAACGTA-1	singlet	0	-192.0492571	-637.0857594	-640.6772496	EMD1
AAAGGTAAGTGCACCC-1	singlet	0	-181.2332047	-485.814336	-575.2909616	EMD1
AAAGGTACAGATACCT-1	singlet	2	-5282.07095	-4668.595584	-1920.88437	EMD1
AAAGGTACAGCGACCT-1	singlet	0	-389.1182743	-854.2572484	-832.8677285	EMD1
AAAGGTACATGTTTGG-1	singlet	0	-267.0409735	-856.0411643	-802.7333299	EMD1
AAAGGTAGTAAGCTCT-1	singlet	1	-1844.645656	-762.62513	-1576.151624	EMD1
AAAGGTAGTCACTCGG-1	singlet	0	-170.616426	-520.1365855	-495.7582326	EMD1
AAAGGTAGTCGATGCC-1	singlet	0	-121.550659	-299.5519172	-292.4237561	EMD1
AAAGGTATCAAATAGG-1	singlet	0	-248.0843337	-653.5594337	-665.8092978	EMD1
AAAGGTATCAGGTGTT-1	singlet	1	-2741.469612	-1075.608279	-1854.927293	EMD1
AAAGGTATCATACAGC-1	singlet	0	-1489.520742	-4936.541007	-5104.110194	EMD1
AAAGGTATCCCTCTCC-1	doublet	1/0	-56.26364664	-54.25108919	-432.6462951	EMD1
AAAGGTATCTTCCTAA-1	singlet	2	-457.2801241	-388.1389707	-179.7124278	EMD1
AAAGTCCAGCCATCCG-1	singlet	2	-398.4331896	-426.8517633	-219.2860881	EMD1
AAAGTCCAGTTGGAGC-1	singlet	0	-108.7945715	-329.7431514	-282.6637968	EMD1
AAAGTCCCAGGCCTGT-1	singlet	1	-1823.607952	-497.5678061	-1431.368256	EMD1
AAAGTCCGTACGAGCA-1	singlet	1	-4633.392551	-1363.037101	-3874.989342	EMD1
AAAGTCCGTATACCCA-1	singlet	2	-287.9223301	-201.6505537	-119.7200336	EMD1
AAAGTCCGTCGCAGTC-1	singlet	2	-480.6166943	-492.3082691	-197.6047623	EMD1
AAAGTCCTCCATCGTC-1	singlet	0	-74.34257429	-190.0652454	-199.0871299	EMD1
AAAGTCCTCGTCCTCA-1	singlet	0	-1947.958395	-6430.854095	-6350.654744	EMD1
AAAGTGAAGGAACGCT-1	singlet	2	-176.240473	-128.3267882	-56.5896638	EMD1
AAAGTGAAGTTACTCG-1	singlet	1	-3015.513816	-839.4972928	-2179.338843	EMD1
AAAGTGAAGTTTGGCT-1	singlet	2	-561.7529638	-518.5765485	-262.9930305	EMD1
AAAGTGACAGACTCTA-1	singlet	2	-670.7198321	-555.3535978	-230.4055458	EMD1
AAAGTGACATAACCCA-1	singlet	1	-1016.818124	-381.1573357	-775.3344719	EMD1
AAAGTGACATCTCGTC-1	singlet	0	-314.4869954	-1037.579304	-959.8124252	EMD1
AAAGTGACATTAGGAA-1	singlet	2	-484.6556396	-394.147019	-208.9738626	EMD1
AAAGTGAGTACCGTGC-1	singlet	2	-1310.820958	-1090.052114	-466.84435	EMD1
AAAGTGAGTCCACTTC-1	singlet	1	-1339.297559	-541.7280218	-1079.341922	EMD1
AAAGTGAGTGAGCCAA-1	singlet	2	-429.9566663	-346.4875812	-189.188738	EMD1
AAAGTGAGTGTTAAAG-1	singlet	0	-148.599132	-625.8672985	-582.0640777	EMD1
AAAGTGATCTGGGAGA-1	singlet	0	-41.68075766	-75.12535075	-467.309924	EMD1
AAAGTGATCTGGTGCG-1	singlet	0	-645.9764193	-1835.617882	-1968.019746	EMD1
AAATGGAAGATTAGAC-1	singlet	1	-3425.459051	-937.2655458	-2602.640022	EMD1
AAATGGAAGTGAGGCT-1	singlet	0	-271.6060901	-674.6521873	-650.7164707	EMD1
AAATGGACACATATGC-1	singlet	1	-8689.652993	-2272.842999	-6435.641764	EMD1
AAATGGACAGCACCCA-1	singlet	2	-576.9832456	-517.8748625	-270.8252766	EMD1
AAATGGACATCCAATG-1	singlet	1	-3267.015172	-983.3769619	-2948.385076	EMD1
AAATGGAGTAGGCAGT-1	singlet	2	-801.7665392	-610.9640504	-265.5860497	EMD1
AAATGGAGTCATCCGG-1	singlet	0	-244.8860512	-803.8847709	-780.0419798	EMD1
AAATGGAGTTCAAAGA-1	singlet	0	-1239.462023	-3578.02927	-3369.158803	EMD1
AAATGGATCAAGTGGG-1	singlet	0	-508.6500435	-1482.831181	-1665.717919	EMD1
AAATGGATCTACAGGT-1	singlet	2	-2941.465371	-2276.640279	-977.9650894	EMD1
AACAAAGAGCGTACAG-1	singlet	0	-227.3825671	-625.0838811	-574.418066	EMD1
AACAAAGAGTTCATCG-1	singlet	2	-448.7460886	-435.832061	-185.6603202	EMD1
AACAAAGCAGAGGAAA-1	singlet	2	-466.6103314	-360.680469	-177.7527987	EMD1
AACAAAGCATCATTTC-1	singlet	2	-349.9285233	-423.2558041	-174.3930399	EMD1
AACAAAGCATTGCTTT-1	singlet	0	-580.8350092	-2005.645642	-1798.115577	EMD1
AACAAAGCATTGGATC-1	singlet	1	-340.1342924	-163.0421103	-328.5240982	EMD1
AACAAAGGTAGTTAGA-1	singlet	2	-908.7851581	-745.8421088	-332.919544	EMD1
AACAAAGTCAAGAGTA-1	singlet	0	-175.8848245	-539.5662292	-550.8621421	EMD1

AACAAAGTCGCTCTCA-1	singlet	2	-209.9819557	-202.9755542	-109.3716282	EMD1
AACAAAGTCTCACTCG-1	singlet	2	-624.0558421	-445.5817071	-219.2567764	EMD1
AACAACCAGAGAGAAC-1	singlet	0	-71.47687617	-206.4099728	-181.0062563	EMD1
AACAACCAGCAGGTCA-1	singlet	0	-518.2916365	-1267.290122	-1195.2321	EMD1
AACAACCAGGATTTAG-1	singlet	2	-692.1249996	-566.3560741	-280.8188545	EMD1
AACAACCAGGGAGGAC-1	singlet	1	-1949.68199	-688.5483316	-1564.947871	EMD1
AACAACCCAGATCACT-1	singlet	2	-673.178728	-542.3490159	-244.9139158	EMD1
AACAACCCAGCGTATT-1	unassigned	0	-1.039772678	-1.704944545	-1.576209252	EMD1
AACAACCCATTACTCT-1	singlet	2	-294.2568056	-238.2861403	-151.4412889	EMD1
AACAACCTCCCAGGCA-1	singlet	2	-378.5845432	-294.7535415	-125.6636827	EMD1
AACAACCTCCGGTAAT-1	singlet	2	-2213.034714	-2346.709314	-834.9036572	EMD1
AACAACCTCGCAAGAG-1	singlet	2	-719.4732059	-610.2929749	-320.027139	EMD1
AACAAGACACCCTTGT-1	singlet	1	-4316.76465	-1394.185752	-3231.111959	EMD1
AACAAGACACGGTGCT-1	singlet	2	-398.3938667	-358.763606	-181.3746252	EMD1
AACAAGACAGGAGACT-1	singlet	0	-206.4988257	-487.5236278	-570.4176797	EMD1
AACAAGACATGGGCAA-1	singlet	1	-3463.539927	-997.3825777	-2546.257187	EMD1
AACAAGAGTAGCTGAG-1	singlet	1	-2741.302371	-935.0141021	-2186.237327	EMD1
AACAAGAGTGCAAGAC-1	singlet	0	-280.5223311	-878.2791425	-798.1701267	EMD1
AACAAGAGTGGTCAAG-1	singlet	1	-11766.40473	-2434.561752	-8238.312192	EMD1
AACAAGAGTTCTCGTC-1	singlet	2	-290.6526494	-265.7268521	-141.5353888	EMD1
AACAAGAGTTGCCGAC-1	singlet	0	-622.0900698	-1692.368215	-1555.824141	EMD1
AACAAGATCGGAGCAA-1	singlet	2	-324.7455581	-244.6469844	-112.9903256	EMD1
AACAAGATCTGTACAG-1	singlet	1	-1752.788121	-602.7349831	-1415.954246	EMD1
AACACACAGATCCTAC-1	singlet	0	-293.0804285	-919.4758049	-797.7193003	EMD1
AACACACAGATGTTAG-1	singlet	2	-423.1524927	-359.6841313	-157.5188771	EMD1
AACACACCAAGGATGC-1	singlet	2	-291.1389417	-214.4250113	-130.7265913	EMD1
AACACACCAATGGGTG-1	singlet	1	-2386.642527	-958.0997623	-1640.499599	EMD1
AACACACCACGACGTC-1	singlet	2	-424.3098077	-361.080936	-204.2741853	EMD1
AACACACCACTTCAGA-1	unassigned	45323	-53.95927212	-51.48157538	-26.48433576	EMD1
AACACACCAGCTCGGT-1	singlet	2	-1024.97241	-935.8912698	-459.6055964	EMD1
AACACACGTCAGGCAA-1	singlet	1	-1654.287529	-556.7587798	-1317.526189	EMD1
AACACACGTGTTATCG-1	singlet	2	-915.5607934	-689.9878865	-325.0988127	EMD1
AACACACGTTGCATAC-1	singlet	2	-392.9149608	-283.6796915	-153.2198716	EMD1
AACACACTCAAATAGG-1	singlet	1	-1994.331228	-782.5149974	-1679.397056	EMD1
AACACACTCAGAGCGA-1	singlet	0	-238.9039628	-683.3707323	-742.1277273	EMD1
AACACACTCCCAGTGG-1	singlet	0	-717.6498532	-2058.839114	-2043.506104	EMD1
AACAGGGAGCGTATGG-1	singlet	0	-440.6533023	-1273.21719	-1404.558521	EMD1
AACAGGGAGCTCCGAC-1	doublet	2/0	-693.4607292	-673.4741894	-403.5522841	EMD1
AACAGGGAGGATCATA-1	singlet	2	-1320.282273	-1001.471442	-455.9346123	EMD1
AACAGGGAGTAACCGG-1	singlet	2	-405.893316	-422.4049706	-191.4665933	EMD1
AACAGGGAGTGCAGCA-1	singlet	1	-145.1538507	-56.55635725	-107.0795492	EMD1
AACAGGGAGTGGACGT-1	singlet	1	-821.7405345	-311.3883759	-669.5133557	EMD1
AACAGGGCAATAAGGT-1	singlet	0	-942.4683433	-2628.784662	-2697.637344	EMD1
AACAGGGGTGAATGAT-1	singlet	0	-944.4532743	-2871.140395	-2800.61236	EMD1
AACAGGGTCTCCGTGT-1	singlet	0	-964.8647214	-2917.008572	-3014.85347	EMD1
AACCAACAGAAACTCA-1	singlet	1	-3998.762866	-1403.506014	-3347.334624	EMD1
AACCAACAGACGGTCA-1	singlet	1	-394.7694794	-135.4263526	-329.7296044	EMD1
AACCAACAGATAACAC-1	singlet	0	-734.6257847	-2417.816552	-2361.426855	EMD1
AACCAACCAGGCGATA-1	singlet	1	-1308.552412	-469.7654947	-1245.577955	EMD1
AACCAACGTACGCGTC-1	singlet	2	-1423.695986	-1204.45579	-484.4974931	EMD1
AACCAACGTCGCATGC-1	singlet	2	-519.3218348	-495.347506	-238.7954517	EMD1
AACCAACGTTAGAGAT-1	singlet	2	-405.4276608	-338.5831178	-155.4251118	EMD1
AACCAACGTTATGGTC-1	singlet	1	-2901.366054	-883.6891601	-2084.354236	EMD1
AACCAACGTTCCGGTG-1	singlet	0	-843.8727447	-2417.972763	-2497.708692	EMD1
AACCAACGTTGTTGTG-1	singlet	2	-1950.950568	-1683.941467	-697.096512	EMD1
AACCAACTCCTTTAGT-1	singlet	0	-379.1424154	-1135.330705	-1138.460852	EMD1
AACCAACTCTGTCAGA-1	singlet	0	-280.8883358	-1332.28605	-1108.613637	EMD1
AACCACAAGATGCTGG-1	singlet	0	-511.713239	-1444.98338	-1388.188031	EMD1
AACCACAAGCACTTTG-1	singlet	0	-133.8392342	-349.5242554	-314.9334143	EMD1
AACCACAAGCTGAAGC-1	singlet	0	-18.09720771	-38.78607654	-211.3681881	EMD1
AACCACACAACTAGAA-1	singlet	2	-9218.606703	-7233.030314	-3213.795835	EMD1
AACCACACAAGAAACT-1	singlet	2	-354.1289353	-281.9208205	-102.7616208	EMD1
AACCACACACACCTTC-1	singlet	2	-1183.150363	-1168.237225	-399.4773404	EMD1
AACCACACACGCTGTG-1	singlet	2	-581.6671421	-431.404669	-207.8962975	EMD1
AACCACACAGTCGTTA-1	singlet	0	-779.4780356	-2544.79941	-2334.466332	EMD1
AACCACAGTACCTTCC-1	singlet	1	-1512.909961	-639.1665895	-1450.976722	EMD1
AACCACAGTACGACTT-1	singlet	2	-1054.294751	-871.5941831	-370.2712015	EMD1
AACCACATCAGTCCGG-1	singlet	2	-688.7353638	-564.6970141	-234.1666813	EMD1
AACCATGCAGGTGAGT-1	doublet	45323	-1598.127006	-1066.753092	-814.1295561	EMD1
AACCATGCATGAAAGT-1	singlet	0	-346.2403753	-967.5058796	-882.8619339	EMD1
AACCATGGTTAGCGGA-1	singlet	0	-387.6186057	-1122.837323	-1272.197025	EMD1
AACCATGTCAGTCCGG-1	singlet	2	-481.6603956	-464.2000703	-187.3793793	EMD1
AACCATGTCAGTGATC-1	singlet	0	-591.3802868	-1862.414619	-1735.105554	EMD1
AACCATGTCGTGGTAT-1	singlet	1	-2286.904657	-832.8029444	-1996.254687	EMD1
AACCATGTCTAGACAC-1	singlet	2	-323.2288596	-270.4913629	-115.2821784	EMD1
AACCCAAAGACGCATG-1	singlet	2	-2014.434886	-1732.765366	-786.4291651	EMD1
AACCCAACAAACGTGG-1	singlet	2	-2202.782466	-1779.227436	-718.5579823	EMD1
AACCCAACAGGTTCAT-1	singlet	2	-1705.833835	-1470.503185	-758.9899475	EMD1
AACCCAACAGTCGCTG-1	singlet	1	-177.9355302	-72.11034163	-135.3963945	EMD1
AACCCAAGTACTGAGG-1	singlet	0	-1141.284695	-4979.26462	-4533.038722	EMD1
AACCCAAGTCTACAAC-1	singlet	0	-268.6509784	-707.0731116	-788.3724012	EMD1
AACCCAATCACTGTTT-1	singlet	0	-195.5322497	-529.4267732	-438.4213966	EMD1
AACCCAATCCCGTGAG-1	singlet	1	-3676.82297	-967.0941698	-2926.197539	EMD1
AACCTGAAGAGGGCGA-1	singlet	1	-3013.549279	-1153.730503	-2493.599022	EMD1
AACCTGAAGCAGGTCA-1	singlet	2	-599.6843762	-495.4628883	-267.3634694	EMD1
AACCTGAAGGCATTTC-1	singlet	0	-735.2284489	-2191.125922	-2298.350239	EMD1
AACCTGACAGACCATT-1	singlet	2	-544.4909729	-498.1597677	-255.7374079	EMD1
AACCTGACAGCATTGT-1	singlet	2	-421.2895466	-343.7554628	-172.2754479	EMD1
AACCTGACATCAACCA-1	singlet	2	-723.7352731	-596.2547822	-332.7251338	EMD1
AACCTGAGTACCAGAG-1	singlet	1	-3820.151595	-1170.758058	-2797.835032	EMD1
AACCTGATCTCATAGG-1	singlet	1	-1389.299616	-386.1243295	-1039.473303	EMD1
AACCTTTAGGCGTCCT-1	doublet	0/2	-709.5624601	-1002.215453	-883.5023329	EMD1
AACCTTTCACGGTCTG-1	singlet	0	-828.0290718	-2318.657205	-2320.352641	EMD1
AACCTTTCAGCGTATT-1	singlet	1	-696.2683483	-175.65602	-498.7665934	EMD1
AACCTTTGTAGCTAAA-1	doublet	1/0	-14.77334721	-12.52914644	-26.23285172	EMD1
AACCTTTTCACACCGG-1	singlet	2	-638.5233935	-527.9224071	-254.5548659	EMD1
AACCTTTTCTCTATGT-1	singlet	2	-533.0558546	-457.9578114	-197.7891327	EMD1
AACGAAAAGGAATCGC-1	singlet	2	-456.1040534	-329.4537951	-164.2596021	EMD1
AACGAAAAGTTTCGGT-1	singlet	0	-843.9778681	-2169.842627	-2289.315276	EMD1
AACGAAACATCCTATT-1	singlet	2	-576.4998352	-491.8459174	-252.298391	EMD1
AACGAAACATGCCGAC-1	singlet	2	-891.8250062	-745.5933749	-338.0541864	EMD1
AACGAAAGTATTCCTT-1	singlet	2	-424.9636992	-324.0583289	-140.4786236	EMD1
AACGAAAGTTCTTCAT-1	singlet	2	-214.030014	-164.0455823	-75.67810039	EMD1
AACGAAAGTTTATGCG-1	singlet	2	-6558.368503	-5437.037477	-2188.465098	EMD1
AACGAAATCAAGCGTT-1	singlet	0	-753.7922133	-2546.944354	-2380.573787	EMD1
AACGAAATCGAGAAGC-1	singlet	2	-627.9512447	-574.2765838	-279.0871708	EMD1
AACGAAATCGTGTCAA-1	singlet	2	-61.06841976	-64.03691435	-18.61705057	EMD1
AACGAAATCTCGCAGG-1	singlet	1	-1008.756704	-323.177548	-833.5464305	EMD1
AACGAAATCTGAACGT-1	singlet	0	-316.6505396	-1100.942014	-1022.937122	EMD1
AACGAAATCTGGACTA-1	singlet	2	-240.3409697	-208.8381015	-79.96416124	EMD1

AACGGGAAGACCAACG-1	singlet	0	-259.1688952	-636.1484564	-626.4912395	EMD1
AACGGGAAGATGACCG-1	singlet	1	-1391.01998	-530.3891422	-1105.883377	EMD1
AACGGGAAGCGTTAGG-1	singlet	1	-93.96876816	-26.78003942	-80.0054207	EMD1
AACGGGACAATAGTAG-1	singlet	2	-362.4779952	-267.0817777	-133.1524686	EMD1
AACGGGAGTAACCCTA-1	singlet	0	-204.6800358	-479.1595611	-473.441514	EMD1
AACGGGAGTCGTACAT-1	singlet	2	-512.2564565	-452.8856274	-229.0754984	EMD1
AACGGGAGTCTGCAAT-1	singlet	0	-1309.198006	-5685.137348	-5141.479562	EMD1
AACGGGAGTTTCAGAC-1	singlet	1	-3114.091434	-761.9637768	-2636.460404	EMD1
AACGGGATCTCAGAAC-1	singlet	2	-1765.250973	-1863.450001	-779.3841801	EMD1
AACGTCAAGAAATTGC-1	singlet	2	-58.96821437	-53.15830606	-20.89201553	EMD1
AACGTCAAGAGCCTGA-1	singlet	2	-916.5669538	-823.8912977	-388.2088091	EMD1
AACGTCAAGGATTCCT-1	singlet	2	-823.4890912	-749.3550488	-313.2497293	EMD1
AACGTCACACGGTGTC-1	singlet	2	-815.9831971	-807.6654433	-338.5858118	EMD1
AACGTCACATAACGGG-1	singlet	2	-577.9148488	-511.9832878	-220.944318	EMD1
AACGTCACATCGCCTT-1	singlet	1	-3756.767882	-898.6795223	-2757.502635	EMD1
AACGTCACATGACTCA-1	singlet	0	-16.41347729	-42.7358885	-48.05065109	EMD1
AACGTCAGTAACGGTG-1	singlet	0	-743.2626173	-2254.969793	-2154.151748	EMD1
AACGTCAGTGGAAGTC-1	singlet	2	-432.4497858	-314.439053	-160.306502	EMD1
AACGTCATCATTACTC-1	singlet	1	-3382.484465	-1196.44249	-2938.543086	EMD1
AACGTCATCCTAGCCT-1	singlet	2	-774.1221718	-641.1456335	-295.7573428	EMD1
AACGTCATCGCTTTAT-1	singlet	2	-342.2362472	-281.8071369	-134.9720967	EMD1
AACTTCTAGCCTTTCC-1	singlet	0	-574.2609073	-3686.944932	-2989.746676	EMD1
AACTTCTCATGTGGCC-1	singlet	1	-3349.854015	-1149.636967	-2771.25286	EMD1
AACTTCTGTAAGATAC-1	singlet	2	-562.1985583	-473.3494883	-231.6794221	EMD1
AACTTCTGTACAAGTA-1	singlet	2	-357.5243005	-294.9267775	-142.7464658	EMD1
AACTTCTGTCGTATGT-1	singlet	0	-726.3831493	-2122.142917	-2111.898513	EMD1
AACTTCTTCTTGCAGA-1	singlet	2	-641.5982692	-542.6514714	-238.4991869	EMD1
AACTTCTTCTTTGCGC-1	singlet	2	-330.3788425	-330.0663823	-180.9549319	EMD1
AAGAACAAGAATTGCA-1	singlet	1	-2684.5611	-872.1733161	-2001.43014	EMD1
AAGAACAAGTCAGGGT-1	singlet	2	-2758.359644	-2395.055158	-1078.155986	EMD1
AAGAACACAAGCGAGT-1	singlet	1	-6356.33408	-2001.373654	-5019.242475	EMD1
AAGAACACAATCTCGA-1	singlet	0	-203.1435896	-478.2580861	-465.2053532	EMD1
AAGAACACATGATGCT-1	singlet	1	-4301.048576	-1240.250751	-3275.859258	EMD1
AAGAACAGTCCCGTGA-1	singlet	2	-444.0069891	-384.2780729	-187.758125	EMD1
AAGAACAGTCGACTGC-1	singlet	2	-636.9819401	-538.5419087	-237.7422796	EMD1
AAGAACAGTCGGAAAC-1	singlet	0	-404.929251	-994.8651188	-1053.387703	EMD1
AAGAACAGTCTGTAGT-1	singlet	0	-1797.582343	-7988.741332	-6795.915129	EMD1
AAGAACAGTGCCTATA-1	unassigned	45323	-25.46421218	-16.70115834	-14.5318209	EMD1
AAGACAAAGCAAATGT-1	singlet	0	-310.9448938	-964.4832232	-864.9351832	EMD1
AAGACAAAGGATACGC-1	singlet	1	-155.5559574	-43.23836863	-110.2394879	EMD1
AAGACAAAGTCTGCAT-1	singlet	2	-87.04788356	-104.4973918	-18.09603364	EMD1
AAGACAAAGTTTCTTC-1	singlet	1	-1442.117755	-509.1759556	-1246.510531	EMD1
AAGACAACAGATCCTA-1	singlet	2	-549.7861051	-503.3242872	-201.0883826	EMD1
AAGACAACAGCCCACA-1	singlet	0	-2142.637678	-7464.237605	-7240.110014	EMD1
AAGACAAGTCCGAAAG-1	singlet	0	-221.8087461	-599.1285296	-660.079253	EMD1
AAGACAAGTCGTCTCT-1	singlet	1	-3412.818071	-1178.32058	-2691.915768	EMD1
AAGACAAGTCTAGGTT-1	singlet	2	-59.52589569	-55.47291446	-18.79078963	EMD1
AAGACAAGTTAGAAGT-1	singlet	2	-647.0701812	-594.0913523	-327.8494313	EMD1
AAGACAATCCTACAAG-1	singlet	2	-791.3518492	-713.6506165	-274.2278591	EMD1
AAGACAATCTTACGTT-1	singlet	2	-446.4159199	-353.9143086	-173.1638732	EMD1
AAGACTCAGATCGCTT-1	singlet	1	-848.3134296	-321.4631719	-673.1008084	EMD1
AAGACTCAGCGGGTAT-1	doublet	2/0	-3322.468627	-3908.435795	-2837.330334	EMD1
AAGACTCAGTTCTCTT-1	singlet	0	-321.4611628	-910.5977611	-851.2162483	EMD1
AAGACTCGTACGTTCA-1	singlet	0	-54.72928164	-179.3311152	-153.9208325	EMD1
AAGACTCTCGGAATTC-1	singlet	0	-150.1898646	-410.6320199	-419.2427335	EMD1
AAGATAGAGCTAGATA-1	singlet	0	-1314.046896	-4758.835922	-4546.192416	EMD1
AAGATAGCACGGGTAA-1	singlet	2	-600.4006729	-519.4983445	-228.2163144	EMD1
AAGATAGGTAACTAAG-1	singlet	0	-622.0516228	-2173.48345	-1964.887545	EMD1
AAGATAGGTGGTCCGT-1	singlet	2	-602.7808109	-470.3003957	-214.8622593	EMD1
AAGATAGGTTAACCTG-1	singlet	2	-526.487934	-447.6408924	-233.7069399	EMD1
AAGATAGTCCCATTCG-1	singlet	0	-431.6180705	-1372.711523	-1348.753603	EMD1
AAGCATCAGACTCAAA-1	singlet	1	-610.5523616	-186.0444207	-451.6092499	EMD1
AAGCATCAGTCAAGCG-1	singlet	1	-3853.163139	-1160.301248	-2870.863889	EMD1
AAGCATCCACTAGGCC-1	singlet	0	-1179.562261	-3617.36089	-3576.343747	EMD1
AAGCCATCAGGAGACT-1	singlet	2	-264.4107378	-191.3706569	-115.4028353	EMD1
AAGCCATCAGGCAATG-1	singlet	1	-4197.383326	-1263.799193	-3393.606351	EMD1
AAGCCATCATTGTCGA-1	singlet	0	-915.6895328	-1981.318527	-1783.347227	EMD1
AAGCCATGTTCTTGTT-1	singlet	2	-689.4432596	-580.6991147	-276.6539013	EMD1
AAGCCATTCGTTCCCA-1	singlet	0	-570.6531366	-2077.142338	-1857.753391	EMD1
AAGCGAGAGAATACAC-1	singlet	1	-1456.939338	-406.9436872	-1253.191545	EMD1
AAGCGAGAGGAACGTC-1	singlet	0	-1325.674579	-3687.148902	-3429.681327	EMD1
AAGCGAGCAACCGGAA-1	singlet	0	-286.5334796	-1110.202054	-965.0688667	EMD1
AAGCGAGCACGTAGAG-1	singlet	2	-325.0566867	-249.6052026	-137.919575	EMD1
AAGCGAGCAGCACCCA-1	singlet	1	-1219.076115	-518.7222309	-1085.580293	EMD1
AAGCGAGCAGCAGGAT-1	singlet	2	-336.27164	-280.830187	-134.5948076	EMD1
AAGCGAGCATAAGATG-1	singlet	0	-339.6927734	-996.0611828	-794.5739025	EMD1
AAGCGAGGTCGGCACT-1	singlet	0	-42.0953486	-185.4781279	-190.779836	EMD1
AAGCGAGTCGTTTACT-1	singlet	0	-1049.899959	-3224.630086	-3225.750789	EMD1
AAGCGTTAGTCCTACA-1	singlet	1	-2192.804867	-645.6845261	-1902.743452	EMD1
AAGCGTTCAGGCGATA-1	singlet	0	-289.3350683	-853.270268	-881.1160027	EMD1
AAGCGTTCATGGCCAC-1	singlet	0	-251.7198781	-621.5194454	-766.0963581	EMD1
AAGCGTTCATTCCTAT-1	singlet	0	-482.1216892	-1397.573596	-1292.415836	EMD1
AAGCGTTGTACTCCCT-1	singlet	2	-589.5220592	-413.8486182	-173.8560749	EMD1
AAGCGTTGTGGACAGT-1	singlet	2	-1951.95714	-2441.373276	-586.682735	EMD1
AAGCGTTGTGTGTGGA-1	singlet	0	-319.3247739	-843.7390256	-887.7127522	EMD1
AAGGAATAGCCTAGGA-1	singlet	0	-183.0168804	-528.1447901	-589.8398417	EMD1
AAGGAATAGGTCGTAG-1	singlet	2	-843.5670076	-792.2594704	-260.9869803	EMD1
AAGGAATCAAAGACGC-1	singlet	0	-450.8310533	-1984.526906	-1615.008807	EMD1
AAGGAATCAATTGCGT-1	doublet	0/1	-587.6768932	-714.1032807	-1044.110303	EMD1
AAGGAATCACAAAGTA-1	singlet	2	-4133.511706	-3533.820934	-1274.549709	EMD1
AAGGAATCACCATATG-1	singlet	2	-551.9086543	-462.8318358	-231.4035187	EMD1
AAGGAATTCACAACCA-1	singlet	2	-445.0288963	-422.1511726	-194.3683334	EMD1
AAGGAATTCATGCATG-1	singlet	2	-1250.766796	-1117.972586	-498.9710931	EMD1
AAGGAATTCCATTGTT-1	singlet	0	-664.4474013	-1952.715913	-2000.44339	EMD1
AAGGAATTCGAGTCCG-1	singlet	2	-2599.904174	-2185.330465	-780.5707576	EMD1
AAGGAATTCTAACACG-1	singlet	0	-468.9869503	-1185.514522	-789.8469868	EMD1
AAGGTAAAGCTTTCTT-1	singlet	1	-5824.161441	-1952.689251	-4720.250549	EMD1
AAGGTAAAGTGCAAAT-1	singlet	1	-576.8102769	-214.499872	-524.0040845	EMD1
AAGGTAACAAGGGCAT-1	singlet	2	-210.0941216	-177.7836231	-70.99336541	EMD1
AAGGTAAGTACCGTGC-1	singlet	0	-75.13712294	-258.1997304	-246.2133706	EMD1
AAGGTAAGTACGTGAG-1	unassigned	2/0	-5.74317047	-10.34690352	-4.67285748	EMD1
AAGGTAAGTATGGTTC-1	unassigned	0/2	-5.048313262	-7.861602386	-9.334664793	EMD1
AAGGTAAGTGTTAGCT-1	singlet	2	-1749.204074	-1639.174475	-667.7996464	EMD1
AAGGTAATCACTTGGA-1	singlet	1	-1005.518864	-405.1610453	-762.0158231	EMD1
AAGGTAATCCACCCTA-1	singlet	0	-130.5396742	-340.332801	-312.0036248	EMD1
AAGGTAATCGCGAAGA-1	singlet	0	-1221.993916	-3271.714811	-3289.916808	EMD1
AAGGTAATCTTTGGAG-1	singlet	1	-616.1623217	-229.2329138	-443.5372512	EMD1
AAGTACCAGAGATCGC-1	singlet	0	-735.1486322	-2212.022866	-2088.477284	EMD1
AAGTACCAGGACAAGA-1	singlet	0	-2.668141009	-7.075170585	-5.894655196	EMD1
AAGTACCAGGTCGTCC-1	singlet	0	-1084.308828	-3214.306619	-3240.596312	EMD1

AAGTACCAGTCAAGCG-1	doublet	45293	-4182.035276	-1649.275439	-3110.609851	EMD1
AAGTACCCAGCTCCTT-1	singlet	2	-323.675729	-309.2244768	-128.7721204	EMD1
AAGTACCCAGTATTCG-1	doublet	0/2	-185.9301073	-260.2597617	-195.8605685	EMD1
AAGTACCGTCCGTACG-1	singlet	0	-192.4268972	-807.9358021	-892.427239	EMD1
AAGTACCGTCTGTGGC-1	singlet	2	-336.2742193	-263.6986956	-131.9135823	EMD1
AAGTACCGTGCGTGCT-1	singlet	1	-1444.144628	-572.8559207	-1052.428102	EMD1
AAGTACCTCTATACTC-1	singlet	1	-1081.441542	-424.686099	-860.4729352	EMD1
AAGTCGTAGCATAGGC-1	singlet	2	-628.2922543	-581.6404597	-231.2386877	EMD1
AAGTCGTAGCGGTAAC-1	singlet	2	-91.06240925	-85.51359883	-31.90979151	EMD1
AAGTCGTAGCTTTGTG-1	singlet	2	-449.4856351	-367.7767198	-199.6810132	EMD1
AAGTCGTAGTAGACAT-1	singlet	1	-1753.007188	-628.953375	-1358.671407	EMD1
AAGTCGTCACAATCTG-1	singlet	1	-3355.04353	-990.2936716	-2780.081762	EMD1
AAGTCGTTCAGTCTTT-1	singlet	2	-589.3378873	-442.0396625	-217.2485289	EMD1
AAGTCGTTCATGGGAG-1	singlet	2	-497.663907	-411.9528912	-216.8209949	EMD1
AAGTCGTTCGTCGACG-1	unassigned	2	-5.777592545	-2.214154235	-1.913371048	EMD1
AAGTGAAAGCTCGCAC-1	singlet	0	-2256.228919	-13843.046	-11744.97889	EMD1
AAGTGAAAGCTGCCAC-1	singlet	2	-851.7720755	-704.4956732	-316.0393229	EMD1
AAGTGAAAGTACAGCG-1	doublet	45293	-1219.138373	-609.9069155	-914.0946076	EMD1
AAGTGAAAGTGCTACT-1	singlet	0	-344.9548611	-1079.86727	-943.7936736	EMD1
AAGTGAACACTCAAGT-1	singlet	2	-515.9888309	-439.605844	-206.4169137	EMD1
AAGTGAACAGCTGTCG-1	singlet	2	-680.8468892	-646.3792576	-304.6686057	EMD1
AAGTGAACAGGTGGAT-1	singlet	2	-312.1385213	-256.1689047	-130.0482792	EMD1
AAGTGAACATAGTCAC-1	singlet	0	-785.2937349	-2409.990823	-2195.393772	EMD1
AAGTGAAGTCAAGGCA-1	singlet	0	-68.72455876	-154.098115	-136.004309	EMD1
AAGTGAAGTCTCAGAT-1	singlet	0	-714.6802581	-1834.917646	-1876.016523	EMD1
AAGTGAAGTGCTGTCG-1	singlet	0	-124.8400946	-361.678331	-464.7101202	EMD1
AAGTGAATCAGGAAGC-1	singlet	1	-870.782119	-315.4736847	-728.6118916	EMD1
AAGTTCGAGACCAAGC-1	singlet	2	-3774.289372	-3107.216903	-1283.085354	EMD1
AAGTTCGAGCCGTTGC-1	singlet	2	-500.0754304	-420.0214515	-166.8883768	EMD1
AAGTTCGAGGTGCGAT-1	singlet	2	-91.1051378	-102.6924046	-36.68693686	EMD1
AAGTTCGAGTGTTGAA-1	singlet	2	-683.9407868	-536.7634714	-236.7105326	EMD1
AAGTTCGCACGCGTCA-1	singlet	0	-333.9103716	-909.842637	-813.4514516	EMD1
AAGTTCGTCCGCAACG-1	singlet	1	-459.9892622	-152.862671	-392.9959913	EMD1
AATAGAGAGAACGCGT-1	singlet	1	-5016.723094	-1399.789653	-4040.682509	EMD1
AATAGAGAGTATTAGG-1	singlet	2	-582.5776463	-490.7597241	-254.1588826	EMD1
AATAGAGCACACCAGC-1	singlet	2	-1693.626262	-1390.285136	-536.9143132	EMD1
AATAGAGCAGCTGGTC-1	singlet	0	-257.7821649	-949.8189862	-787.9850491	EMD1
AATAGAGCAGGTATGG-1	singlet	2	-680.2202051	-574.115502	-220.2551183	EMD1
AATAGAGCATGGCGCT-1	singlet	0	-1719.13801	-5008.130118	-4730.094112	EMD1
AATAGAGGTACAGTCT-1	singlet	2	-1851.603087	-1372.274352	-522.540226	EMD1
AATAGAGGTATCGTTG-1	singlet	2	-678.0100414	-671.0663362	-244.8696194	EMD1
AATAGAGGTGTGTTTG-1	singlet	0	-872.5880101	-3229.072068	-2916.88321	EMD1
AATAGAGTCCTGGTCT-1	singlet	2	-922.5720306	-727.3345221	-348.0863703	EMD1
AATAGAGTCGGCAGTC-1	singlet	1	-88.58274916	-29.18508469	-54.02720634	EMD1
AATAGAGTCGTGTGAT-1	singlet	0	-701.655472	-2043.132175	-2087.975029	EMD1
AATCACGAGAGAGCCT-1	singlet	1	-1120.958038	-367.7925668	-1029.036884	EMD1
AATCACGCAGCGTGCT-1	singlet	2	-63.51713772	-65.79686471	-25.38893422	EMD1
AATCACGCAGGGACTA-1	singlet	2	-691.1472695	-669.1757127	-290.3708104	EMD1
AATCACGGTGCCTGCA-1	singlet	2	-55.81391177	-66.3599941	-21.35355481	EMD1
AATCACGGTTCTCCTG-1	singlet	0	-314.426321	-1080.745321	-978.886846	EMD1
AATCACGTCCGAGGCT-1	singlet	1	-292.8069423	-126.7490242	-257.38455	EMD1
AATCACGTCCTTCGAC-1	singlet	2	-17044.21702	-13369.46544	-5614.760087	EMD1
AATCGACAGGTTGGAC-1	singlet	1	-3327.825714	-1072.558627	-2587.396956	EMD1
AATCGACCAAATGCGG-1	singlet	2	-319.8919277	-252.9783178	-131.2279337	EMD1
AATCGACCAGCGTGAA-1	singlet	2	-482.0833521	-376.7994786	-211.7431453	EMD1
AATCGACGTCACATTG-1	singlet	2	-487.5055141	-429.2502808	-156.6612013	EMD1
AATCGACTCTCCCATG-1	singlet	2	-571.0969663	-458.9588242	-198.7635134	EMD1
AATCGACTCTCCTGAC-1	singlet	2	-422.5186256	-334.2439669	-148.5672956	EMD1
AATCGTGAGGGTAGCT-1	singlet	2	-634.1566406	-404.693017	-226.7157763	EMD1
AATCGTGAGTCGGCCT-1	singlet	2	-546.8688152	-498.1412374	-247.3455246	EMD1
AATCGTGCAACACAGG-1	singlet	0	-637.3063122	-1924.620649	-1996.297206	EMD1
AATCGTGCACTACTTT-1	singlet	2	-127.9250825	-109.2000258	-54.31782712	EMD1
AATCGTGCAGAACGCA-1	singlet	1	-89.13282919	-36.80056443	-58.18634321	EMD1
AATCGTGGTACCGTCG-1	singlet	0	-221.600158	-870.8751404	-811.0422708	EMD1
AATCGTGGTCTTTATC-1	singlet	2	-2427.178433	-2139.915131	-901.836335	EMD1
AATCGTGGTGTCGCTG-1	singlet	2	-690.8931701	-565.7379652	-238.7210716	EMD1
AATCGTGGTTACGGAG-1	singlet	0	-214.0061079	-688.1805189	-628.0045926	EMD1
AATCGTGTCATCTACT-1	singlet	1	-2203.805718	-676.0032591	-1906.502498	EMD1
AATCGTGTCGGAAACG-1	singlet	1	-1475.537499	-491.3991984	-1107.411903	EMD1
AATCGTGTCTGTAAGC-1	singlet	0	-1772.204146	-6147.487624	-6095.706997	EMD1
AATCGTGTCTTACACT-1	singlet	1	-5692.850866	-1556.531889	-4173.648685	EMD1
AATGAAGAGAAGCGAA-1	singlet	2	-481.3930007	-417.9565938	-208.0998506	EMD1
AATGAAGAGGTAGACC-1	singlet	0	-246.9023539	-638.5118267	-605.3312748	EMD1
AATGAAGCAGGTGAGT-1	singlet	1	-640.0585873	-225.5203397	-552.8184413	EMD1
AATGAAGGTACTGTTG-1	singlet	1	-3826.156478	-1229.152408	-3015.746286	EMD1
AATGAAGGTATGACAA-1	singlet	0	-342.3483892	-930.0429458	-911.7591405	EMD1
AATGAAGTCCAAGCCG-1	singlet	0	-46.98453661	-102.899219	-134.2420118	EMD1
AATGAAGTCCGACATA-1	singlet	2	-247.9385257	-196.3758297	-101.5398468	EMD1
AATGACCAGCTAAACA-1	singlet	2	-478.9917564	-414.4432634	-192.8750715	EMD1
AATGACCAGTATAGAC-1	singlet	1	-1180.861255	-452.479763	-988.7893311	EMD1
AATGACCCAAAGGGTC-1	singlet	1	-477.6374041	-185.4347853	-534.7150527	EMD1
AATGACCCAGAAATTG-1	singlet	2	-1035.814514	-870.7395149	-458.1053837	EMD1
AATGACCGTTCCGCGA-1	singlet	2	-497.8479343	-458.0817482	-193.9557088	EMD1
AATGACCGTTGCTCGG-1	singlet	0	-711.7958964	-2132.034619	-2097.627235	EMD1
AATGACCTCCTTTAGT-1	singlet	0	-1074.806685	-3575.296488	-3319.509126	EMD1
AATGACCTCTCACGAA-1	singlet	2	-367.5316844	-349.514864	-157.7986409	EMD1
AATGACCTCTTCCCGA-1	singlet	2	-4945.054156	-4190.777906	-1820.784552	EMD1
AATGCCACACTCTAGA-1	singlet	2	-441.2822208	-414.1994237	-202.0348699	EMD1
AATGCCACAGGTTCAT-1	singlet	0	-895.384844	-2922.721216	-2937.241821	EMD1
AATGCCAGTACACGTT-1	singlet	2	-10804.27896	-8147.980575	-3375.538643	EMD1
AATGCCAGTGAAAGTT-1	singlet	0	-724.5312114	-2780.930422	-2538.295098	EMD1
AATGCCATCAAACGTC-1	singlet	0	-175.5621533	-539.7759391	-449.90928	EMD1
AATGCCATCCCTCTCC-1	singlet	1	-3216.688014	-1217.066857	-2629.066392	EMD1
AATGCCATCGGTTAGT-1	singlet	2	-3841.021348	-3157.687868	-1330.916755	EMD1
AATGGAAAGACCATGG-1	singlet	2	-430.7889336	-365.2772936	-175.1539878	EMD1
AATGGAAAGGGTGGGA-1	singlet	1	-2493.036036	-725.4304189	-1881.563827	EMD1
AATGGAACAAATCAAG-1	unassigned	0/1	-4.825544003	-6.828688516	-7.35576132	EMD1
AATGGAAGTATGATCC-1	singlet	0	-1505.988441	-4676.925944	-4653.696497	EMD1
AATGGAAGTCTCCCTA-1	singlet	2	-2301.525688	-1966.115914	-754.0555442	EMD1
AATGGAAGTCTTGGTA-1	singlet	1	-11778.23374	-1954.191482	-7041.672145	EMD1
AATGGAATCCAGTGCG-1	singlet	0	-26.78931528	-130.375846	-118.2047337	EMD1
AATGGAATCCGCAAAT-1	singlet	2	-809.9354651	-604.3049724	-277.4859782	EMD1
AATGGAATCGCGTTTC-1	singlet	1	-1436.644998	-433.3964721	-1123.176594	EMD1
AATGGCTAGAATTCAG-1	singlet	2	-491.5843617	-451.1986346	-211.2031804	EMD1
AATGGCTAGAGGGTGG-1	singlet	0	-218.0423418	-601.9984932	-608.5433299	EMD1
AATGGCTAGGCATCAG-1	singlet	2	-702.7224053	-539.4958306	-253.2704687	EMD1
AATGGCTCACCAGTTA-1	singlet	2	-614.6957878	-444.2522197	-243.5656911	EMD1
AATGGCTCACCCAACG-1	singlet	2	-877.1332238	-825.2864344	-397.5574214	EMD1
AATGGCTCAGCTACTA-1	singlet	2	-1368.197951	-1141.457861	-570.3603588	EMD1
AATGGCTCAGGTTCCG-1	singlet	2	-369.8435868	-379.3856122	-167.8958843	EMD1

AATGGCTTCGGCACTG-1	singlet	1	-649.7290442	-241.556585	-521.8803655	EMD1
AATGGCTTCTCCTGTG-1	singlet	2	-530.513163	-469.5286621	-252.4319086	EMD1
AATTCCTAGCAGTACG-1	singlet	0	-1480.328062	-3273.279893	-2853.236782	EMD1
AATTCCTAGCGATCGA-1	singlet	0	-1196.505912	-3415.110868	-3513.497339	EMD1
AATTCCTAGGTTAGTA-1	singlet	2	-3794.307292	-3114.532184	-1394.491038	EMD1
AATTCCTAGTGATCGG-1	singlet	1	-4670.229648	-1349.752365	-3726.706872	EMD1
AATTCCTAGTGTGTTC-1	singlet	2	-1488.10802	-1282.998657	-497.5982859	EMD1
AATTCCTCAACAAAGT-1	singlet	2	-790.1911757	-695.91793	-324.468069	EMD1
AATTCCTCACAATGTC-1	singlet	0	-69.75275199	-220.5461922	-224.0874098	EMD1
AATTCCTGTTGCCTAA-1	singlet	1	-663.3968943	-210.624986	-522.7640201	EMD1
AATTCCTTCATTCACT-1	singlet	1	-4713.384087	-1427.242885	-3756.674111	EMD1
AATTCCTTCTAGTGAC-1	singlet	1	-1903.589868	-682.0214378	-1642.42809	EMD1
AATTTCCAGAGCGACT-1	singlet	2	-469.7530299	-292.6142857	-57.76539693	EMD1
AATTTCCCACGCTGTG-1	singlet	2	-595.8725016	-443.6610961	-225.1769027	EMD1
AATTTCCCATCAACCA-1	singlet	2	-753.8868292	-671.7201868	-266.3176172	EMD1
AATTTCCGTCTGTCAA-1	singlet	2	-756.1666665	-615.8656752	-281.4116636	EMD1
AATTTCCGTGCCTTTC-1	singlet	2	-2913.524317	-2633.077124	-702.4623135	EMD1
AATTTCCTCCTCTGCA-1	singlet	2	-3628.215527	-3032.448097	-1359.684771	EMD1
AATTTCCTCGTTTACT-1	singlet	0	-1603.029674	-4339.503002	-4443.092397	EMD1
AATTTCCTCTCCCATG-1	singlet	1	-2892.916175	-1032.496543	-2357.740998	EMD1
ACAAAGAAGATACTGA-1	singlet	1	-2099.404266	-744.2016311	-1554.590056	EMD1
ACAAAGACACCAGCCA-1	singlet	1	-2069.019296	-694.0700703	-1722.052499	EMD1
ACAAAGACATATCTGG-1	singlet	2	-472.8923994	-415.1319706	-135.0985679	EMD1
ACAAAGACATCCTGTC-1	singlet	2	-557.5606974	-465.1280962	-234.3889661	EMD1
ACAAAGATCCATTGGA-1	singlet	0	-804.6199972	-3085.087219	-2809.030953	EMD1
ACAAAGATCTGGCCTT-1	singlet	0	-902.2236955	-3374.093907	-2829.268082	EMD1
ACAACCAAGCAAATCA-1	singlet	2	-361.2491147	-328.4145797	-157.4152416	EMD1
ACAACCAAGCTAATGA-1	singlet	0	-1494.817376	-5079.751219	-5589.063663	EMD1
ACAACCACATCTCAAG-1	singlet	2	-262.6486482	-217.2252115	-109.4015524	EMD1
ACAACCAGTCCTACGG-1	singlet	2	-646.4085626	-531.2461979	-258.2318192	EMD1
ACAACCAGTTCTATCT-1	singlet	0	-1160.877919	-2705.803941	-2439.46703	EMD1
ACAACCATCTATGCCC-1	singlet	2	-1144.561237	-995.5880712	-466.3898013	EMD1
ACAAGCTAGGGTAGCT-1	singlet	0	-29.56311551	-110.325323	-91.7321407	EMD1
ACAAGCTAGTCTAACC-1	singlet	2	-302.768602	-252.1847234	-125.6459775	EMD1
ACAAGCTCAAGAAATC-1	singlet	0	-729.06522	-2490.620308	-2319.823746	EMD1
ACAAGCTCAGAAACCG-1	singlet	2	-635.8952756	-521.9108461	-227.0135982	EMD1
ACAAGCTGTATGAAGT-1	singlet	1	-1776.289971	-640.295013	-1477.29617	EMD1
ACAAGCTGTGATATAG-1	singlet	0	-41.20854355	-136.6613542	-130.8027809	EMD1
ACAAGCTGTGCGGTAA-1	singlet	1	-224.6567925	-72.53529551	-182.1864038	EMD1
ACAAGCTTCGGTGAAG-1	singlet	2	-311.7961772	-251.7088871	-122.670214	EMD1
ACACAGTCAATAGGAT-1	singlet	2	-895.2873954	-764.9679664	-327.4663497	EMD1
ACACAGTCAGGAGGAG-1	singlet	0	-124.508736	-339.7646871	-356.8128342	EMD1
ACACAGTCATGGCGCT-1	singlet	0	-433.8583472	-1381.701911	-1281.232322	EMD1
ACACAGTGTAGTCACT-1	singlet	2	-342.6605198	-317.4664017	-161.9408382	EMD1
ACACAGTGTCACTTAG-1	singlet	2	-441.4590715	-370.8463881	-197.0257073	EMD1
ACACAGTGTTAGAAGT-1	singlet	2	-393.7022697	-347.3499886	-157.069423	EMD1
ACACAGTGTTCTTGCC-1	singlet	2	-481.4132655	-370.3253117	-207.8123123	EMD1
ACACAGTGTTGGGTAG-1	singlet	2	-376.9464198	-318.7253066	-148.5786149	EMD1
ACACAGTGTTGGGTTT-1	singlet	2	-246.1669988	-241.9678468	-122.2915348	EMD1
ACACAGTTCGAACGCC-1	singlet	2	-1365.498513	-1145.318295	-451.3535266	EMD1
ACACCAAAGAATCCCT-1	singlet	2	-461.8434542	-438.9712581	-211.9205031	EMD1
ACACCAAAGGTATAGT-1	singlet	2	-178.7330051	-106.6464193	-49.92903222	EMD1
ACACCAACACGACGCT-1	singlet	2	-602.0137617	-517.2904894	-235.8302502	EMD1
ACACCAACACGATAGG-1	singlet	0	-236.5856525	-698.7045244	-673.0367883	EMD1
ACACCAAGTGGCATCC-1	singlet	1	-1451.54603	-299.4726439	-1057.26313	EMD1
ACACCAAGTGTGCTTA-1	singlet	1	-1459.330483	-416.3920949	-1063.120438	EMD1
ACACCAAGTTTACGAC-1	singlet	0	-343.7858298	-1028.729228	-1143.238166	EMD1
ACACCAATCTTTCCAA-1	singlet	1	-6942.608495	-2372.183156	-5566.277629	EMD1
ACACGCGAGCCTGAGA-1	singlet	2	-138.9902702	-82.53577006	-41.41951055	EMD1
ACACGCGAGCGAACTG-1	singlet	2	-490.3330196	-499.4512821	-196.6893244	EMD1
ACACGCGCACATGTTG-1	singlet	0	-163.2952209	-417.4832077	-410.368705	EMD1
ACACGCGGTCTGATCA-1	singlet	2	-2356.678333	-2054.906775	-681.3049487	EMD1
ACACGCGGTTGCACGC-1	singlet	2	-345.6448799	-287.7030441	-107.7393104	EMD1
ACACGCGTCACAGTGT-1	singlet	0	-977.3862524	-2703.372072	-2889.977718	EMD1
ACACGCGTCAGAGCGA-1	doublet	2/0	-361.0483889	-432.8231549	-282.5883954	EMD1
ACACTGAAGCTTCTAG-1	singlet	0	-1426.991052	-3973.624679	-4240.255071	EMD1
ACACTGAAGGACAGCT-1	singlet	2	-4295.488944	-3465.324468	-1334.002101	EMD1
ACACTGAAGGGCGAGA-1	singlet	2	-348.369169	-337.1021883	-156.5875545	EMD1
ACACTGACAGGTTCGC-1	singlet	2	-468.5262515	-360.8824836	-163.2560212	EMD1
ACACTGACATATTCGG-1	singlet	0	-918.373365	-2814.489586	-2933.947626	EMD1
ACACTGAGTCCACTTC-1	singlet	1	-1855.221605	-638.1901663	-1618.727725	EMD1
ACACTGAGTGCATACT-1	singlet	2	-1226.831901	-992.5427559	-149.1604785	EMD1
ACACTGAGTGGATCAG-1	singlet	0	-447.9558713	-1386.659209	-1427.553897	EMD1
ACACTGAGTTTGGCTA-1	singlet	0	-139.7453969	-402.3472588	-345.0376552	EMD1
ACACTGATCCCAAGCG-1	singlet	2	-605.7429969	-506.9160676	-247.7041439	EMD1
ACACTGATCGCATTGA-1	singlet	1	-2959.419671	-772.0136349	-2279.41794	EMD1
ACACTGATCGCCAGTG-1	singlet	2	-581.3394508	-465.3220874	-199.1777684	EMD1
ACACTGATCGTCGGGT-1	singlet	2	-219.1328097	-208.2939094	-99.17315302	EMD1
ACACTGATCTTGCGCT-1	singlet	0	-1964.171244	-6317.868847	-5968.199358	EMD1
ACAGAAAAGCAGGTCA-1	singlet	0	-230.7058545	-819.6750706	-868.6281037	EMD1
ACAGAAAAGTCATACC-1	singlet	1	-2228.520618	-686.7592429	-1910.714916	EMD1
ACAGAAAAGTGCCAGA-1	singlet	2	-519.1259749	-419.7930167	-176.7951309	EMD1
ACAGAAACACCCAATA-1	singlet	1	-2505.611666	-740.3044092	-1933.919065	EMD1
ACAGAAACACTGTTCC-1	singlet	2	-3466.842105	-3016.276157	-1396.649375	EMD1
ACAGAAACATACCATG-1	singlet	2	-1453.620095	-1197.894069	-522.5516987	EMD1
ACAGAAATCCCGAAAT-1	singlet	2	-1520.379055	-1216.695977	-513.3020025	EMD1
ACAGCCGAGATGAATC-1	singlet	2	-368.9594764	-263.6891677	-133.7322768	EMD1
ACAGCCGAGCATGCGA-1	singlet	1	-819.7710722	-291.3785493	-605.6226125	EMD1
ACAGCCGCAATCTCGA-1	singlet	0	-234.795583	-655.7265031	-701.7891689	EMD1
ACAGCCGCATGGCCCA-1	singlet	1	-1917.813929	-641.9882566	-1529.416405	EMD1
ACAGCCGGTAGTAAGT-1	singlet	2	-54.86055856	-43.81211854	-24.38047622	EMD1
ACAGCCGGTCACCCTT-1	singlet	1	-1805.907847	-457.9780518	-1289.037573	EMD1
ACAGCCGGTCGCGTTG-1	singlet	0	-532.4893045	-1774.942082	-1624.613868	EMD1
ACAGCCGGTCGCTTGG-1	singlet	2	-384.5135371	-317.1591568	-151.8711128	EMD1
ACAGCCGGTTCCTAGA-1	singlet	1	-890.6019782	-288.8227985	-681.2476278	EMD1
ACAGCCGTCAGAGCGA-1	singlet	2	-737.4843887	-598.5120701	-270.6725584	EMD1
ACAGCCGTCGTTGTGA-1	singlet	2	-404.8153922	-347.9002016	-153.0061864	EMD1
ACAGGGAAGTCGTTAC-1	singlet	2	-1201.107095	-990.2399751	-387.322897	EMD1
ACAGGGACAACAGCCC-1	singlet	2	-62.88991615	-74.21915584	-23.16285162	EMD1
ACAGGGACACATTCGA-1	singlet	2	-638.247319	-552.2831665	-248.4243167	EMD1
ACAGGGACACCTGATA-1	singlet	0	-1018.713167	-2995.309907	-3074.193074	EMD1
ACAGGGACAGTCGAGA-1	singlet	2	-437.3490897	-430.3609248	-187.6954453	EMD1
ACAGGGAGTCCATCTC-1	singlet	1	-5960.420663	-1922.215504	-4760.875871	EMD1
ACAGGGAGTCTGTCAA-1	singlet	2	-285.5539049	-199.1166609	-121.1346111	EMD1
ACAGGGATCACCTTGC-1	singlet	2	-2322.905167	-2429.901296	-844.1254684	EMD1
ACAGGGATCTACCTTA-1	singlet	2	-507.9767238	-409.3622436	-218.5185761	EMD1
ACATCCCAGCTGAAAT-1	singlet	2	-494.527878	-480.969345	-265.9602448	EMD1
ACATCCCCAAACGAGC-1	singlet	2	-755.2780103	-658.078567	-305.9596525	EMD1
ACATCCCCACTAGTAC-1	singlet	0	-577.340615	-1722.813845	-1593.132389	EMD1
ACATCCCGTCCAAGAG-1	singlet	2	-297.6606717	-246.9326666	-113.3724894	EMD1

ACATCCCGTGAGGCAT-1	singlet	0	-1149.743446	-3389.265752	-3417.285608	EMD1
ACATCCCGTTCGAAGG-1	singlet	2	-1236.032395	-1077.320802	-404.2394334	EMD1
ACATCCCGTTGCCTAA-1	singlet	2	-692.9157715	-626.087043	-296.8906984	EMD1
ACATCCCGTTGGCCTG-1	singlet	2	-606.4935436	-553.0159472	-238.2340876	EMD1
ACATCCCTCACCGACG-1	singlet	2	-1516.454404	-1371.720783	-640.9979839	EMD1
ACATCCCTCATAGAGA-1	singlet	1	-426.0461836	-160.0338381	-421.2683162	EMD1
ACATCCCTCGGTGTTA-1	singlet	2	-3148.857162	-2329.254264	-1044.112795	EMD1
ACATCCCTCTAGCAAC-1	singlet	2	-156.9467235	-166.4042418	-50.36535827	EMD1
ACATCCCTCTTGGATG-1	singlet	2	-1767.985003	-1557.069308	-687.5034957	EMD1
ACATCGAAGAGTGACC-1	singlet	1	-1960.961706	-497.5222328	-1347.962953	EMD1
ACATCGAAGGTTCCGC-1	singlet	1	-1643.444602	-579.2608615	-1327.161132	EMD1
ACATCGACATCAGCAT-1	singlet	2	-427.0948494	-341.4837881	-188.5271008	EMD1
ACATCGAGTGCCTACG-1	singlet	2	-316.4074842	-273.3372272	-125.2712992	EMD1
ACATCGAGTTCCACGG-1	singlet	1	-2562.952879	-776.0611742	-1991.529156	EMD1
ACATCGATCTCTCTAA-1	singlet	1	-575.7316873	-262.0750226	-589.1508413	EMD1
ACATGCACACAGTGAG-1	singlet	0	-602.0060671	-1895.171845	-1905.757655	EMD1
ACATGCACAGTCTCTC-1	singlet	0	-927.6045305	-2431.114188	-2451.18456	EMD1
ACATGCAGTCACTACA-1	singlet	0	-624.0375786	-1589.048057	-1591.554595	EMD1
ACATGCAGTTTCCCAC-1	singlet	0	-67.00472124	-189.3633386	-170.969823	EMD1
ACATGCAGTTTGTTCT-1	singlet	0	-798.8178151	-2164.486615	-2070.470698	EMD1
ACATGCATCGTTCTAT-1	singlet	0	-767.6953792	-2432.512755	-2421.672121	EMD1
ACATTTCTCAATCAGC-1	singlet	1	-9944.463339	-3618.474496	-8319.170864	EMD1
ACATTTCTCCAAGCAT-1	singlet	2	-411.682203	-372.7157316	-169.1598894	EMD1
ACATTTCTCCGCAACG-1	singlet	2	-533.548054	-466.9367257	-233.719992	EMD1
ACATTTCTCTGGGAGA-1	singlet	2	-199.0878108	-164.6938722	-92.59772898	EMD1
ACCAAACAGAAACTGT-1	singlet	1	-531.4422163	-204.9273449	-497.9179391	EMD1
ACCAAACCAGAGCGTA-1	singlet	2	-530.9193309	-446.5196948	-202.3337575	EMD1
ACCAAACGTAGGTGCA-1	singlet	1	-1087.903	-400.9042341	-855.9489219	EMD1
ACCAAACGTTCTTAGG-1	singlet	2	-627.060728	-560.1619273	-272.0448456	EMD1
ACCAAACTCGTACACA-1	singlet	0	-373.5728147	-1039.777064	-971.4821201	EMD1
ACCAAACTCTCTTCAA-1	singlet	0	-372.8680909	-962.7612598	-985.2351177	EMD1
ACCAACAAGAATTGCA-1	singlet	1	-2540.672455	-813.7793427	-2242.80028	EMD1
ACCAACAAGATGAACT-1	singlet	0	-279.2170648	-923.1641602	-838.2406711	EMD1
ACCAACAAGATGAAGG-1	singlet	2	-739.0264482	-548.4411255	-230.7029104	EMD1
ACCAACAAGCATGAAT-1	singlet	0	-1978.893871	-6432.150877	-6172.663451	EMD1
ACCAACAAGCCAAGCA-1	unassigned	2/0	-4.665677797	-4.631032714	-4.023686434	EMD1
ACCAACAAGTGGTGAC-1	singlet	2	-364.3923836	-292.7224363	-162.3459359	EMD1
ACCAACAGTAATGATG-1	singlet	2	-387.372159	-308.9230475	-138.9865902	EMD1
ACCAACAGTTTCGGCG-1	singlet	0	-152.2587745	-369.1347583	-452.0722439	EMD1
ACCAACATCATGAAAG-1	singlet	2	-1181.689636	-1095.199387	-407.1288947	EMD1
ACCAACATCGGCTGTG-1	singlet	2	-3342.133105	-2863.10516	-1124.434444	EMD1
ACCAACATCGTTGTAG-1	doublet	2/0	-843.5978867	-1192.484226	-794.8288846	EMD1
ACCACAAAGATGGCGT-1	singlet	0	-1410.044073	-4997.369016	-4781.228914	EMD1
ACCACAAAGGGTCTTT-1	singlet	1	-6092.446934	-1869.826691	-4692.561473	EMD1
ACCACAAAGTGCTACT-1	singlet	2	-208.8935596	-174.4704383	-81.38846616	EMD1
ACCACAACAAGGTCGA-1	singlet	2	-1646.365398	-1435.635498	-566.7820001	EMD1
ACCACAACACCCTAGG-1	singlet	0	-962.874784	-2708.415245	-2578.528673	EMD1
ACCACAACAGCCATTA-1	singlet	2	-134.5055859	-118.9138216	-34.15294405	EMD1
ACCACAACAGCGGTTC-1	singlet	0	-848.9848894	-2416.898392	-2352.331778	EMD1
ACCACAACATACCATG-1	singlet	0	-495.9568415	-1407.372973	-1444.890492	EMD1
ACCACAATCACGATCA-1	singlet	2	-3840.537699	-3067.477932	-1441.182353	EMD1
ACCACAATCAGCCTTC-1	singlet	2	-474.376097	-480.6447655	-205.0171071	EMD1
ACCACAATCTAACACG-1	singlet	2	-607.4450386	-469.6058735	-274.0905181	EMD1
ACCACAATCTGCTTTA-1	singlet	0	-2349.041575	-7255.394609	-7664.526066	EMD1
ACCATTTAGACCTCCG-1	singlet	2	-780.1226555	-666.2143023	-322.5866462	EMD1
ACCATTTAGCGTTGTT-1	singlet	2	-615.6705299	-555.5147143	-233.9821459	EMD1
ACCATTTAGCTCGTGC-1	singlet	0	-929.1561849	-2526.671988	-2602.637197	EMD1
ACCATTTAGTCGAAGC-1	singlet	2	-215.12769	-140.0751994	-83.43552203	EMD1
ACCATTTCAGGTTCGC-1	singlet	2	-542.8060154	-476.2608994	-196.8659145	EMD1
ACCATTTCATGGGATG-1	singlet	0	-1405.729833	-4105.961124	-4193.323186	EMD1
ACCATTTGTCCTTTGC-1	singlet	2	-349.6186687	-299.0127663	-160.4493385	EMD1
ACCATTTGTGAACTAA-1	singlet	0	-682.0740693	-2184.3677	-2149.816909	EMD1
ACCATTTGTGATTAGA-1	singlet	2	-559.3516934	-461.0953962	-220.1491837	EMD1
ACCATTTGTGGCCCAT-1	singlet	2	-619.9216066	-545.7913638	-248.0766772	EMD1
ACCATTTTCGCAATTG-1	singlet	2	-542.2688113	-478.1772183	-211.3337213	EMD1
ACCATTTTCGCCTTGT-1	singlet	2	-1758.369501	-1530.377382	-681.0701369	EMD1
ACCATTTTCGCTGATA-1	unassigned	0	-6.950763796	-7.401248485	-82.83987465	EMD1
ACCATTTTCTCCTACG-1	singlet	1	-597.8671998	-249.6713126	-571.7887088	EMD1
ACCATTTTCTTTGGAG-1	singlet	0	-113.7339978	-388.8905157	-352.9238725	EMD1
ACCCAAAAGCTGCGAA-1	singlet	1	-885.9658783	-270.7336406	-792.0148393	EMD1
ACCCAAAAGTCAGCGA-1	singlet	1	-651.8882641	-258.1220563	-553.6526781	EMD1
ACCCAAAAGTCCCGGT-1	singlet	2	-636.3569574	-561.0023393	-279.7217722	EMD1
ACCCAAACAACCACAT-1	singlet	1	-672.1617407	-248.039155	-657.9071754	EMD1
ACCCAAACACAGTCGC-1	singlet	0	-620.4570688	-1673.453291	-1623.501328	EMD1
ACCCAAACACGTCTCT-1	singlet	2	-1063.850428	-954.0416899	-431.0242668	EMD1
ACCCAAAGTACTGACT-1	singlet	2	-10084.56506	-9263.209356	-2945.930768	EMD1
ACCCAAAGTCTGTGGC-1	singlet	2	-3721.872887	-3096.489477	-1090.800695	EMD1
ACCCAAAGTCTGTTAG-1	singlet	2	-353.9509083	-276.7126432	-135.0744867	EMD1
ACCCAAAGTTGTATGC-1	singlet	2	-304.7504786	-244.5839203	-126.805422	EMD1
ACCCTCAAGCAAGTCG-1	singlet	2	-337.0411894	-292.2405152	-133.9706124	EMD1
ACCCTCAAGCCTATTG-1	singlet	0	-521.0718671	-1328.6166	-1306.959684	EMD1
ACCCTCAAGTCCGCGT-1	singlet	0	-892.6315689	-3250.270189	-3207.222773	EMD1
ACCCTCACAAATAGCA-1	singlet	2	-558.3114136	-557.1816381	-235.9991874	EMD1
ACCCTCACAATTAGGA-1	singlet	1	-3448.760476	-1154.987867	-3111.574595	EMD1
ACCCTCACACCCAAGC-1	singlet	0	-330.5881092	-881.4616168	-1022.870063	EMD1
ACCCTCAGTACCGTGC-1	singlet	0	-17.49328682	-52.82761439	-63.06026431	EMD1
ACCCTCAGTATGGGAC-1	singlet	1	-128.8369696	-37.75020923	-114.3437102	EMD1
ACCCTCAGTCCGGCAT-1	singlet	2	-549.0343184	-470.0936778	-219.0395018	EMD1
ACCCTCAGTGGCGCTT-1	singlet	2	-3980.892201	-3384.443566	-1097.19033	EMD1
ACCCTCATCCATTGTT-1	singlet	2	-275.2877282	-218.4231506	-101.7463746	EMD1
ACCCTCATCGCATAGT-1	singlet	2	-755.1442328	-570.1253601	-253.1264519	EMD1
ACCCTCATCTAACGGT-1	doublet	2/0	-1318.183764	-1562.963114	-1225.402813	EMD1
ACCCTCATCTTCACAT-1	singlet	0	-1202.059948	-3894.729955	-3806.357376	EMD1
ACCCTTGAGAGGGTCT-1	singlet	1	-90.8445291	-22.26618933	-58.84667999	EMD1
ACCCTTGAGTCTTCCC-1	singlet	2	-2425.751581	-2530.611507	-882.2662387	EMD1
ACCCTTGCAACGTAAA-1	singlet	0	-305.8447058	-1023.865171	-992.0527974	EMD1
ACCCTTGCAGCATGCC-1	singlet	0	-316.5653457	-871.6250469	-868.9986407	EMD1
ACCCTTGCAGCCGTCA-1	singlet	2	-131.8233966	-178.5978595	-57.29713197	EMD1
ACCCTTGCAGTTGTTG-1	singlet	2	-1804.924661	-1399.379363	-712.3832978	EMD1
ACCCTTGGTGGACTAG-1	singlet	1	-484.2398575	-186.2477096	-502.6725253	EMD1
ACCCTTGGTTGGAGGT-1	singlet	2	-815.2329635	-674.6339793	-279.3672025	EMD1
ACCCTTGTCATGACAC-1	singlet	2	-723.1763149	-563.5107624	-268.5057018	EMD1
ACCCTTGTCCTATTGT-1	singlet	2	-882.2309881	-709.4373614	-294.5546347	EMD1
ACCCTTGTCCTGTTAT-1	singlet	0	-566.732411	-1762.946421	-1772.021793	EMD1
ACCGTTCAGTAGTGCG-1	singlet	0	-593.8345901	-1609.34195	-1583.064859	EMD1
ACCGTTCCAAGCGAGT-1	singlet	1	-457.903004	-178.9875375	-513.0508586	EMD1
ACCGTTCCACAGTGTT-1	singlet	2	-269.1212273	-249.9300409	-125.0954024	EMD1
ACCGTTCGTAATACCC-1	singlet	1	-1080.855271	-306.5200818	-718.33246	EMD1
ACCGTTCGTGATTCAC-1	singlet	2	-242.328856	-211.0042367	-85.85223769	EMD1
ACCGTTCTCCCTTGGT-1	singlet	1	-1007.826325	-315.468723	-702.8148923	EMD1

ACCGTTCTCTACGGTA-1	singlet	2	-700.394686	-610.0079814	-287.2764488	EMD1
ACCGTTCTCTGCAGCG-1	singlet	1	-4155.178453	-1476.719898	-3364.425297	EMD1
ACCTACCAGAAACTAC-1	singlet	1	-252.6104891	-72.20070056	-183.7294655	EMD1
ACCTACCAGATTACCC-1	singlet	1	-2129.152224	-737.8738301	-1731.914289	EMD1
ACCTACCAGCACTCGC-1	singlet	2	-645.0383188	-578.6090959	-293.2537875	EMD1
ACCTACCAGGAGCAAA-1	singlet	2	-506.3218186	-481.409141	-210.703088	EMD1
ACCTACCAGGGTGGGA-1	singlet	0	-678.0911089	-2010.912019	-1869.057492	EMD1
ACCTACCAGTATGACA-1	singlet	0	-361.6524338	-1167.951228	-1091.613929	EMD1
ACCTACCCATCTATCT-1	singlet	0	-733.5236311	-2532.614904	-2447.140538	EMD1
ACCTACCGTATGGAAT-1	singlet	2	-257.6905673	-246.3204934	-117.2081926	EMD1
ACCTACCGTGAACTAA-1	singlet	1	-1508.107695	-520.4530021	-1154.817971	EMD1
ACCTACCTCAGCATTG-1	singlet	0	-740.464051	-2436.40081	-2027.068766	EMD1
ACCTACCTCGTCGGGT-1	singlet	2	-857.454908	-591.1585177	-220.0518404	EMD1
ACCTGAACACATCCCT-1	singlet	2	-2035.169164	-1856.252909	-821.3688461	EMD1
ACCTGAAGTCGCACGT-1	singlet	2	-541.6309563	-445.2314256	-200.7420384	EMD1
ACCTGAATCGCCAGTG-1	singlet	0	-1310.518063	-3865.168919	-3696.264825	EMD1
ACCTGAATCGGCACTG-1	singlet	2	-1276.042034	-1136.451047	-469.8393656	EMD1
ACCTGTCAGACTGAGC-1	doublet	45323	-3096.7454	-1959.358594	-1761.202592	EMD1
ACCTGTCAGTACAGCG-1	singlet	2	-503.4688977	-445.585283	-199.4649864	EMD1
ACCTGTCCAAAGGGCT-1	singlet	2	-643.466745	-538.7033727	-215.7995747	EMD1
ACCTGTCGTACTGCGC-1	singlet	2	-637.8828994	-439.6819629	-243.0571589	EMD1
ACCTGTCGTCATCCCT-1	singlet	1	-136.0296699	-24.82361304	-70.0019037	EMD1
ACCTGTCGTCGCGGTT-1	singlet	0	-996.3991269	-3313.371607	-2945.730744	EMD1
ACCTGTCGTCTTTATC-1	singlet	2	-769.6633335	-611.9787629	-260.7940919	EMD1
ACCTGTCGTGCGACAA-1	doublet	45293	-780.0002654	-505.6641942	-518.0533722	EMD1
ACCTGTCGTTGTTTGG-1	singlet	2	-366.9406184	-357.5203897	-152.2983395	EMD1
ACGATCAAGGAACTAT-1	singlet	1	-721.5695402	-367.227265	-639.6202666	EMD1
ACGATCACAAATTAGG-1	singlet	0	-171.4382733	-472.4682658	-450.5728059	EMD1
ACGATCACAATCAGCT-1	singlet	2	-389.1687654	-378.8447288	-155.0430158	EMD1
ACGATCACAGACATCT-1	singlet	0	-657.6051885	-1717.437108	-1569.714302	EMD1
ACGATCACATTGCTGA-1	singlet	0	-519.146414	-1459.576767	-1570.119402	EMD1
ACGATCAGTGGATGAC-1	singlet	1	-616.3034431	-283.8651628	-618.9072106	EMD1
ACGATCATCACCTTAT-1	singlet	1	-697.2955118	-260.1621122	-649.5954849	EMD1
ACGATCATCCGTGACG-1	singlet	1	-767.2867942	-261.865486	-597.9135615	EMD1
ACGATCATCGAGAACG-1	unassigned	45323	-83.31603712	-69.22507929	-35.47860732	EMD1
ACGATGTAGAATACAC-1	singlet	1	-10379.50324	-3234.896015	-8139.37216	EMD1
ACGATGTAGAGTTGTA-1	singlet	0	-662.127305	-1828.746736	-1717.067518	EMD1
ACGATGTAGCACTCAT-1	singlet	2	-219.0670555	-212.2843865	-112.6907145	EMD1
ACGATGTAGCTGAGCA-1	singlet	2	-568.7365464	-433.5041577	-231.3245139	EMD1
ACGATGTAGGTGGCTA-1	singlet	2	-215.9477949	-197.0639725	-112.6020977	EMD1
ACGATGTCAACAAGTA-1	singlet	2	-219.2113131	-207.1235066	-90.8286138	EMD1
ACGATGTCAATCTCGA-1	singlet	2	-936.4268075	-896.5813678	-313.0650553	EMD1
ACGATGTCATGGTGGA-1	singlet	1	-972.278067	-249.8397332	-760.7889226	EMD1
ACGATGTCATGTGACT-1	singlet	0	-414.7158472	-1151.106784	-1056.388548	EMD1
ACGATGTGTAGCCAGA-1	singlet	2	-237.5057856	-167.9811481	-89.62376728	EMD1
ACGATGTGTGTTCCTC-1	singlet	0	-282.1399427	-858.5483057	-820.8037836	EMD1
ACGATGTTCACCATAG-1	singlet	0	-204.5437229	-669.2898675	-571.9569651	EMD1
ACGATGTTCCGTGGGT-1	singlet	2	-638.7574213	-606.063149	-282.5960445	EMD1
ACGATGTTCTTCGTAT-1	singlet	2	-568.6301406	-484.2600174	-234.3173097	EMD1
ACGCACGAGTACCCTA-1	singlet	0	-343.0998767	-1384.846608	-1446.274344	EMD1
ACGCACGCAGAGGTTG-1	singlet	1	-2472.912376	-744.3547611	-2045.622519	EMD1
ACGCACGCATCTCCCA-1	singlet	2	-475.098699	-371.7125658	-172.0969515	EMD1
ACGCACGGTAGCTTGT-1	singlet	0	-114.2948987	-396.0017112	-381.669615	EMD1
ACGCACGGTGCTCCGA-1	singlet	1	-2288.906879	-665.0923777	-1772.820837	EMD1
ACGCACGGTGTAGCAG-1	singlet	0	-29.54613614	-89.63814979	-69.19450753	EMD1
ACGCACGTCCTCGCAT-1	singlet	0	-1551.088612	-4350.538773	-4106.914789	EMD1
ACGCACGTCCTGTTGC-1	singlet	0	-433.3279691	-1011.828233	-1050.833473	EMD1
ACGCACGTCGCACGGT-1	singlet	2	-4990.285771	-3787.575835	-1439.811359	EMD1
ACGGAAGAGAAAGCGA-1	singlet	1	-840.3889995	-259.3397391	-591.0057007	EMD1
ACGGAAGCAAGTGATA-1	singlet	2	-320.142237	-336.4104556	-154.0576426	EMD1
ACGGAAGCATCTTAGG-1	singlet	2	-570.0256281	-404.1725355	-213.9952514	EMD1
ACGGAAGTCGGAAACG-1	singlet	1	-315.8199112	-120.3143772	-296.9214389	EMD1
ACGGGTCAGCAAGCCA-1	singlet	2	-1108.812034	-891.7133915	-534.2369323	EMD1
ACGGGTCAGTATGGAT-1	unassigned	0	-2.525904979	-4.886293375	-5.376055646	EMD1
ACGGGTCAGTGCTACT-1	singlet	0	-1278.975027	-5646.467473	-4850.134017	EMD1
ACGGGTCCAGTAGTTC-1	singlet	0	-206.7511746	-603.8777208	-635.9364749	EMD1
ACGGGTCGTGACTCTA-1	singlet	2	-384.0692408	-341.5921758	-123.2120109	EMD1
ACGGGTCGTGTCGCTG-1	singlet	0	-668.034962	-1853.459485	-1915.454518	EMD1
ACGGGTCTCACATACG-1	doublet	45293	-3972.185863	-1983.68352	-2733.137302	EMD1
ACGGGTCTCCGACATA-1	singlet	2	-3965.649377	-3188.973161	-1267.263168	EMD1
ACGGGTCTCCTGTTGC-1	singlet	2	-338.0275832	-318.6306015	-163.3455371	EMD1
ACGGGTCTCGCTTACC-1	singlet	2	-1832.574703	-1539.633823	-724.5181356	EMD1
ACGGTCGAGTCAAGCG-1	singlet	0	-316.5293611	-1077.76502	-979.5491609	EMD1
ACGGTCGCAGAACTTC-1	singlet	0	-31.11852336	-126.6712683	-111.8296721	EMD1
ACGGTCGCAGTGCGCT-1	singlet	2	-3548.402531	-2714.824059	-1197.973048	EMD1
ACGGTCGCATAATCCG-1	singlet	2	-199.0940104	-169.5267517	-71.52112719	EMD1
ACGGTCGGTATGGTTC-1	singlet	0	-242.1941489	-958.0078623	-974.3248406	EMD1
ACGGTCGGTGGGACAT-1	singlet	0	-316.2948211	-944.8031958	-911.791874	EMD1
ACGGTCGGTGTTACAC-1	singlet	1	-5482.153996	-2013.502353	-3995.167228	EMD1
ACGGTCGTCGCAAGAG-1	singlet	2	-1083.715819	-821.5835422	-364.3554259	EMD1
ACGGTCGTCTCGGCTT-1	singlet	1	-2381.15131	-878.8201267	-2082.240044	EMD1
ACGGTTAGTAGCGTTT-1	singlet	0	-513.9417166	-1973.909916	-1705.276987	EMD1
ACGGTTATCGCTTGCT-1	singlet	2	-333.5591303	-267.8785026	-127.4816588	EMD1
ACGTAACAGATGCAGC-1	singlet	2	-464.7741502	-391.8115758	-222.7741927	EMD1
ACGTAACAGCAATTAG-1	singlet	2	-95.06894521	-94.00971476	-39.76658508	EMD1
ACGTAACCAAATACAG-1	singlet	2	-278.9999957	-228.6278033	-108.3961917	EMD1
ACGTAACCAATGGCCC-1	singlet	2	-1162.030527	-1070.339608	-454.5773164	EMD1
ACGTAACGTATACAGA-1	singlet	2	-3121.709196	-2304.328042	-1112.126782	EMD1
ACGTAACGTGTTGACT-1	doublet	0/2	-758.6901312	-1368.071795	-1207.336067	EMD1
ACGTAACGTTCTCACC-1	doublet	0/2	-658.5718265	-962.7694747	-746.4375381	EMD1
ACGTACAAGATGCTTC-1	singlet	1	-1400.01585	-354.7728749	-1213.809411	EMD1
ACGTACAAGGAACGCT-1	singlet	2	-3850.36462	-3410.177454	-983.1453149	EMD1
ACGTACACAATGCAAA-1	singlet	2	-662.3993781	-560.91302	-259.7052709	EMD1
ACGTACAGTGGATCAG-1	singlet	0	-2837.483548	-11135.81452	-10117.62983	EMD1
ACGTACAGTTGGCCGT-1	singlet	2	-634.2107212	-578.599834	-256.2612975	EMD1
ACGTACAGTTGGCCTG-1	singlet	2	-723.5204035	-659.7003636	-272.2378089	EMD1
ACGTACATCTTGCAAG-1	singlet	0	-877.2080614	-2832.194003	-2410.387754	EMD1
ACGTAGTAGAGAGGGC-1	singlet	0	-381.7093783	-1088.506713	-1166.792793	EMD1
ACGTAGTCACAAGGTG-1	unassigned	0/1	-37.39899977	-74.50429522	-75.57653847	EMD1
ACGTAGTCACTGCGAC-1	singlet	2	-7572.421117	-5861.886765	-2388.136903	EMD1
ACGTAGTCATGGACAG-1	singlet	2	-152.66481	-142.6802534	-67.15736184	EMD1
ACGTAGTGTAGACAGC-1	singlet	0	-66.52568839	-182.0688254	-184.1154732	EMD1
ACGTAGTGTATTGGCT-1	singlet	1	-1000.143441	-382.1038702	-838.7492652	EMD1
ACGTAGTGTGCAATGG-1	singlet	2	-2259.477392	-1783.045887	-640.5234911	EMD1
ACGTAGTGTTTCGTAG-1	unassigned	0	-6.396823831	-6.834854901	-54.22492185	EMD1
ACGTAGTTCAAGAGTA-1	singlet	2	-2816.616733	-2056.629287	-828.5994283	EMD1
ACGTAGTTCAAGTGTC-1	singlet	2	-404.302322	-399.3192674	-161.2601993	EMD1
ACGTAGTTCGCATGAT-1	singlet	2	-392.7494711	-346.7608398	-123.4920663	EMD1
ACGTCCTAGAACGCGT-1	singlet	0	-53.41770031	-179.1126964	-154.2209783	EMD1
ACGTCCTAGAAGAGCA-1	singlet	2	-417.4962579	-372.4232927	-149.4965466	EMD1

ACGTCCTAGTGCAGCA-1	singlet	1	-2568.971863	-572.1315741	-2001.389493	EMD1
ACGTCCTCAAGTGGAC-1	singlet	2	-486.2713924	-448.931811	-217.2922196	EMD1
ACGTCCTCAGGTTACT-1	singlet	1	-333.7112703	-157.0557886	-279.0356379	EMD1
ACGTCCTCATTCACAG-1	singlet	1	-744.6645745	-273.9734738	-615.163946	EMD1
ACGTCCTTCACCTGTC-1	singlet	0	-306.8965978	-924.2808426	-967.3804892	EMD1
ACGTCCTTCCCATAGA-1	singlet	0	-22.35985421	-99.94902348	-105.7818547	EMD1
ACGTCCTTCTAAGAAG-1	singlet	1	-6432.060533	-1434.701485	-5632.353395	EMD1
ACGTCCTTCTGAGAGG-1	singlet	1	-3349.501557	-904.6679293	-2748.900693	EMD1
ACGTTCCAGAAACTCA-1	singlet	0	-895.4366495	-2491.800706	-2540.195407	EMD1
ACGTTCCAGGACACTG-1	singlet	0	-28.70458402	-110.8576085	-86.69355068	EMD1
ACGTTCCAGGCTTAGG-1	singlet	0	-478.3910607	-1190.284378	-1201.781881	EMD1
ACGTTCCAGTCTAGAA-1	singlet	2	-744.5134019	-704.3145287	-298.8150799	EMD1
ACGTTCCCACAGAGAC-1	singlet	2	-915.0564169	-769.8958242	-369.8990532	EMD1
ACGTTCCCAGAACCGA-1	singlet	1	-903.4271436	-318.6998155	-906.7031059	EMD1
ACGTTCCCATCCCGTT-1	singlet	0	-327.7638903	-958.4058612	-936.1940168	EMD1
ACGTTCCGTCGCAGTC-1	singlet	0	-1443.14002	-6716.231795	-5914.346827	EMD1
ACGTTCCGTCTGTGGC-1	singlet	2	-421.4726953	-420.2948224	-207.278025	EMD1
ACGTTCCGTCTTTCTA-1	singlet	0	-1025.830094	-2729.325011	-2865.070252	EMD1
ACGTTCCGTGATTCTG-1	singlet	2	-230.6606534	-223.0817127	-90.46697448	EMD1
ACGTTCCTCTATCGCC-1	singlet	1	-104.3532873	-18.62107937	-62.55070824	EMD1
ACGTTCCTCTTCGGAA-1	singlet	0	-1610.401364	-4543.023471	-4462.87375	EMD1
ACTACGAAGAGTTGTA-1	singlet	1	-2011.557644	-620.5245158	-1587.406326	EMD1
ACTACGAAGGAATTAC-1	singlet	0	-232.1792903	-881.6855055	-843.0577006	EMD1
ACTACGAAGTTTGGCT-1	singlet	0	-1507.049031	-5282.899346	-4676.599744	EMD1
ACTACGACAACGCATT-1	singlet	2	-583.4576168	-472.7737892	-228.5941676	EMD1
ACTACGACAATGCAAA-1	singlet	2	-2314.61219	-2008.035093	-924.7605023	EMD1
ACTACGAGTAGCGCTC-1	unassigned	1	-3.629068069	-2.961037731	-4.321296251	EMD1
ACTACGAGTGCCTGCA-1	singlet	1	-1167.906567	-299.8316393	-719.7966691	EMD1
ACTATCTAGATCCGAG-1	singlet	0	-791.8530101	-2052.856874	-1932.361235	EMD1
ACTATCTAGGAATGTT-1	singlet	2	-445.4854885	-297.8915125	-148.6569173	EMD1
ACTATCTAGTGCCCGT-1	singlet	2	-2841.639126	-2180.704901	-916.5095936	EMD1
ACTATCTCAGCCATTA-1	singlet	0	-46.69984638	-156.2792521	-165.8266149	EMD1
ACTATCTCATGATAGA-1	singlet	1	-1511.425499	-507.0732601	-1259.139717	EMD1
ACTATCTGTGATTCTG-1	singlet	2	-471.3475404	-390.4354998	-196.1937718	EMD1
ACTATCTGTGGGCTTC-1	singlet	2	-1260.002561	-1169.737252	-478.4448696	EMD1
ACTATCTGTGTATCCA-1	singlet	2	-711.8146464	-623.8374098	-304.9912214	EMD1
ACTATCTTCACCTACC-1	singlet	2	-4482.690837	-3678.709183	-1450.42349	EMD1
ACTATCTTCAGCGCAC-1	singlet	2	-295.7167684	-301.0541071	-150.5878431	EMD1
ACTATCTTCATTTACC-1	singlet	1	-177.9518767	-70.00737589	-155.2482196	EMD1
ACTATGGAGAGTCGAC-1	singlet	2	-227.0671618	-152.7276923	-74.40803196	EMD1
ACTATGGAGAGTTGAT-1	singlet	2	-735.0701131	-623.5280149	-265.116319	EMD1
ACTATGGAGATTTGCC-1	doublet	2/0	-634.0147957	-657.5268764	-378.5642567	EMD1
ACTATGGAGCGACCCT-1	singlet	1	-1647.287876	-448.8932523	-1486.228944	EMD1
ACTATGGCAGCGTTTA-1	singlet	1	-1652.027457	-519.8216365	-1293.277993	EMD1
ACTATGGGTTACGGAG-1	singlet	0	-188.1462338	-441.2915899	-437.3441153	EMD1
ACTATGGGTTCTGAGT-1	singlet	0	-90.56948928	-220.2021123	-221.2716437	EMD1
ACTATGGTCCGTACGG-1	singlet	0	-565.4302103	-1933.71155	-1906.109436	EMD1
ACTATGGTCGGTAACT-1	singlet	0	-325.4145012	-961.7060238	-820.934009	EMD1
ACTATTCAGCAAGTCG-1	singlet	2	-439.9168986	-369.1234385	-173.3588901	EMD1
ACTATTCAGGATTTAG-1	singlet	1	-424.0009899	-119.1417942	-287.8492314	EMD1
ACTATTCCAATCTGCA-1	singlet	2	-154.6719499	-104.9337432	-44.05085846	EMD1
ACTATTCCAATTGCAC-1	singlet	1	-3896.782102	-1306.754105	-3109.380381	EMD1
ACTATTCCAGTCTGGC-1	singlet	2	-1223.90684	-1019.532328	-469.361152	EMD1
ACTATTCGTCTTTCTA-1	singlet	2	-1020.494119	-1034.008647	-351.8177396	EMD1
ACTATTCGTGGAATGC-1	singlet	2	-1264.638344	-1066.715919	-502.2781182	EMD1
ACTATTCGTTCGAAGG-1	singlet	2	-377.2060624	-342.7387161	-186.9911567	EMD1
ACTATTCTCATTCGGA-1	singlet	2	-251.7062916	-240.3289012	-125.1432463	EMD1
ACTCCCAAGACCATAA-1	singlet	1	-2680.016616	-920.843205	-2116.306461	EMD1
ACTCCCAAGGCTTAAA-1	singlet	2	-700.7828816	-608.7464804	-253.0539923	EMD1
ACTCCCAAGTCAAGCG-1	singlet	2	-350.5124054	-283.8344202	-125.1730331	EMD1
ACTCCCACAACTTGGT-1	singlet	1	-2896.610329	-1071.650836	-2297.713863	EMD1
ACTCCCACATTGACCA-1	singlet	2	-457.8836121	-427.1250878	-182.3053425	EMD1
ACTCCCAGTAACCAGG-1	singlet	0	-672.423561	-2272.960085	-2025.097671	EMD1
ACTCCCAGTACTCGCG-1	singlet	0	-294.4405871	-1310.976333	-1049.886712	EMD1
ACTCCCATCGACCTAA-1	singlet	2	-470.2513146	-346.1779498	-136.7565865	EMD1
ACTCCCATCTAGCCAA-1	singlet	1	-3661.437647	-1069.971501	-2703.259446	EMD1
ACTCTCGTCCACGTAA-1	singlet	2	-90.78067419	-84.12161166	-28.41173043	EMD1
ACTCTCGTCCTTTGAT-1	singlet	0	-297.541749	-904.1098844	-823.7358457	EMD1
ACTCTCGTCGTGCTCT-1	doublet	0/2	-871.9794146	-1472.136646	-1221.604503	EMD1
ACTGATGAGACGCATG-1	singlet	1	-2733.165589	-863.1623182	-2133.837159	EMD1
ACTGATGAGAGGTCGT-1	singlet	0	-244.9278112	-886.395992	-843.1003403	EMD1
ACTGATGCACCAGCTG-1	singlet	2	-382.2443696	-330.3366882	-153.3402484	EMD1
ACTGATGCACGTGTGC-1	singlet	0	-843.4952117	-2617.715418	-2385.332322	EMD1
ACTGATGCAGAGATGC-1	singlet	2	-342.0662916	-258.2683019	-118.4542905	EMD1
ACTGATGTCAGAGTGG-1	singlet	2	-503.7506113	-465.5817792	-237.7196055	EMD1
ACTGATGTCATACGAC-1	singlet	2	-1423.578509	-1218.425016	-553.7728642	EMD1
ACTGATGTCCGTGGTG-1	singlet	2	-306.0637913	-258.5648715	-138.2846027	EMD1
ACTGATGTCCTAGCGG-1	singlet	0	-639.4604813	-2140.966349	-2165.913315	EMD1
ACTGATGTCTGTGTGA-1	singlet	2	-387.8615847	-337.9630208	-163.7443165	EMD1
ACTGCAAAGATGCTGG-1	singlet	2	-1510.272877	-1339.004276	-530.6547344	EMD1
ACTGCAAAGCGACAGT-1	singlet	0	-164.8184459	-455.6511508	-497.6813678	EMD1
ACTGCAAAGTCTGGTT-1	singlet	1	-4212.265762	-1315.693131	-3432.740371	EMD1
ACTGCAAAGTGCGCTC-1	singlet	2	-400.5030185	-337.7821803	-149.3345047	EMD1
ACTGCAAAGTTCATCG-1	singlet	2	-511.7594993	-535.0854368	-245.5739635	EMD1
ACTGCAACAGCAGAAC-1	singlet	2	-1470.975392	-1186.389925	-569.8992101	EMD1
ACTGCAAGTCAACCTA-1	singlet	2	-383.7856216	-360.0899858	-165.9420073	EMD1
ACTGTCCAGCCGTCGT-1	singlet	0	-426.528471	-1260.779154	-1155.904316	EMD1
ACTGTCCAGCTAAGTA-1	singlet	1	-941.6310851	-360.235802	-785.2907239	EMD1
ACTGTCCAGGTGGCTA-1	singlet	2	-4864.992831	-4809.464245	-1555.396673	EMD1
ACTGTCCAGTAGGCCA-1	singlet	2	-335.5220682	-256.5579538	-134.1694839	EMD1
ACTGTCCAGTTCATCG-1	singlet	1	-1714.32289	-644.5271226	-1421.407802	EMD1
ACTGTCCGTAGGTAGC-1	singlet	0	-1140.150559	-3687.967415	-3861.328258	EMD1
ACTGTCCGTCCATCTC-1	singlet	0	-904.5454	-2929.97055	-2929.32602	EMD1
ACTGTCCGTTCCGCAG-1	singlet	2	-391.5482114	-356.0631431	-165.2879472	EMD1
ACTGTCCTCAGACAAA-1	singlet	0	-109.1801836	-382.5710325	-400.5355121	EMD1
ACTGTCCTCCCGAAAT-1	singlet	2	-43.45759958	-46.00489231	-15.99484044	EMD1
ACTGTGAAGCACTCTA-1	singlet	0	-777.6964703	-2568.794889	-2519.909743	EMD1
ACTGTGAAGGTTTGAA-1	singlet	2	-374.4178426	-297.8853781	-151.5997586	EMD1
ACTGTGACACCAACAT-1	singlet	1	-7569.818594	-1493.366497	-5625.583309	EMD1
ACTGTGACACTTGACA-1	singlet	1	-381.2992704	-110.0826796	-246.6177664	EMD1
ACTGTGAGTCCGCAGT-1	singlet	2	-479.1828133	-451.6181367	-197.5031447	EMD1
ACTGTGAGTGAAGCGT-1	singlet	1	-1307.81327	-347.3067377	-952.521269	EMD1
ACTGTGAGTGTGTTTG-1	singlet	2	-465.9627333	-373.4653264	-172.1832686	EMD1
ACTGTGATCATATGGC-1	singlet	0	-724.1495728	-2281.724704	-2299.211245	EMD1
ACTGTGATCGGCTTCT-1	singlet	0	-236.9382624	-705.4993935	-831.9930021	EMD1
ACTGTGATCGTTCTAT-1	singlet	2	-519.4883334	-433.209285	-187.6593505	EMD1
ACTGTGATCTTGTGCC-1	singlet	1	-406.314879	-113.5236319	-298.7048214	EMD1
ACTGTGATCTTTGATC-1	singlet	0	-90.19448846	-252.3820992	-251.9273282	EMD1
ACTTAGGCAAATGGAT-1	singlet	2	-587.0663137	-480.9568392	-236.0548019	EMD1
ACTTAGGCAAGACGGT-1	singlet	0	-124.0693187	-495.4577246	-501.6470129	EMD1

ACTTAGGCATGGTGGA-1	singlet	0	-862.8312137	-2493.253964	-2443.874814	EMD1
ACTTAGGGTCTAGATC-1	singlet	0	-220.9221377	-631.9542614	-658.8545405	EMD1
ACTTAGGGTGTTACAC-1	singlet	1	-611.2054594	-247.0124801	-748.0766865	EMD1
ACTTAGGGTGTTGAGG-1	singlet	2	-312.1442626	-287.3316968	-127.7274929	EMD1
ACTTAGGGTTGTGGCC-1	singlet	0	-133.5858197	-718.0928576	-583.215391	EMD1
ACTTAGGTCGAGAGCA-1	singlet	2	-598.4084655	-515.108218	-243.8757321	EMD1
ACTTAGGTCTTACACT-1	singlet	2	-980.6613217	-876.0181386	-311.4478595	EMD1
ACTTATCAGACGCTCC-1	singlet	0	-577.6837618	-1559.278086	-1402.862665	EMD1
ACTTATCAGAGGCGGA-1	singlet	0	-463.2075091	-1365.913776	-1205.482609	EMD1
ACTTATCAGAGGGCGA-1	singlet	2	-345.0390058	-365.0180586	-145.0625896	EMD1
ACTTATCAGTACCCTA-1	singlet	0	-320.0230792	-1249.538082	-1028.442143	EMD1
ACTTATCCACAAAGTA-1	singlet	1	-600.1818958	-255.4204288	-634.9597787	EMD1
ACTTATCGTGATTAGA-1	singlet	1	-13339.95574	-4507.725499	-11104.39474	EMD1
ACTTATCGTTGCACGC-1	singlet	1	-302.5110408	-126.0029719	-254.9636587	EMD1
ACTTATCTCAAGGAGC-1	singlet	2	-739.2198089	-685.6817309	-345.0894235	EMD1
ACTTATCTCACTTCTA-1	singlet	2	-3726.346155	-3356.632928	-1213.845529	EMD1
ACTTATCTCGACCCAG-1	singlet	2	-717.1676456	-638.7122496	-315.3748841	EMD1
ACTTATCTCTAAGCCA-1	singlet	2	-985.7099877	-857.3258469	-400.7518689	EMD1
ACTTATCTCTCTCTAA-1	singlet	2	-441.7237297	-357.9066078	-169.3263953	EMD1
ACTTCCGAGCTACTAC-1	singlet	2	-1070.346247	-891.1989559	-357.7741569	EMD1
ACTTCCGCACAAGCAG-1	singlet	0	-49.36998078	-144.4156013	-175.3751714	EMD1
ACTTCCGCACCAGGTC-1	singlet	0	-796.5645623	-2170.755863	-2289.892207	EMD1
ACTTCGCAGCACCCAC-1	singlet	2	-498.7959477	-357.9899155	-206.4131756	EMD1
ACTTCGCAGTCTGCAT-1	singlet	0	-790.9359391	-2256.412627	-2088.153259	EMD1
ACTTCGCAGTTCCGTA-1	singlet	2	-1522.075286	-1185.577708	-522.1003093	EMD1
ACTTCGCCAAGTAGTA-1	singlet	2	-749.7698589	-654.8449262	-317.2038024	EMD1
ACTTCGCCACCGTCGA-1	singlet	1	-15.78970575	-3.488516002	-7.603378545	EMD1
ACTTCGCCATGACGAG-1	singlet	2	-718.3135322	-641.4312621	-283.1719402	EMD1
ACTTCGCCATTCACAG-1	singlet	1	-3838.695461	-1161.03964	-2976.465413	EMD1
ACTTCGCGTATTAAGG-1	singlet	1	-1142.790999	-295.0617997	-807.0285406	EMD1
ACTTCGCGTCCCGCAA-1	singlet	0	-133.2405138	-426.5641829	-359.7704668	EMD1
ACTTCGCGTCTAGATC-1	singlet	0	-230.1496821	-662.184687	-600.6751458	EMD1
ACTTCGCGTGTTTGCA-1	singlet	2	-284.0994239	-189.7489532	-95.19201115	EMD1
ACTTCGCGTTAAGGGC-1	singlet	2	-1750.189023	-1384.32412	-680.1519563	EMD1
ACTTCGCTCGGCTTCT-1	singlet	1	-1572.40727	-599.8060188	-1294.614356	EMD1
ACTTCGCTCGTGCAGC-1	singlet	1	-580.9766336	-177.2824307	-441.0324808	EMD1
ACTTCGCTCTCCTGCA-1	singlet	2	-3371.590548	-2637.686306	-1019.439986	EMD1
ACTTTCAAGCTACAAA-1	singlet	2	-435.328332	-329.8710654	-197.021008	EMD1
ACTTTCACAAGCTGCC-1	singlet	2	-521.7490628	-463.4637771	-195.0502811	EMD1
ACTTTCACAAGCTGTT-1	singlet	1	-213.5864905	-69.24637242	-149.4518245	EMD1
ACTTTCACAATAACGA-1	singlet	0	-281.7615326	-929.9449015	-917.491761	EMD1
ACTTTCACAATGAGCG-1	singlet	1	-1184.765469	-398.5852128	-893.0216534	EMD1
ACTTTCACAATTCGTG-1	singlet	2	-2501.196939	-2015.64266	-875.9441146	EMD1
ACTTTCACAGCGGTCT-1	singlet	0	-428.2694646	-1549.669076	-1386.6661	EMD1
ACTTTCAGTAAGGAGA-1	singlet	0	-290.7038892	-929.4029227	-1086.619233	EMD1
ACTTTCAGTATGGAAT-1	singlet	2	-612.0946943	-564.9553079	-252.3710079	EMD1
ACTTTCAGTGGAAGTC-1	singlet	0	-398.0537025	-1024.947311	-935.9542096	EMD1
ACTTTCAGTTACCGTA-1	singlet	1	-1976.49787	-518.2405875	-1498.701891	EMD1
ACTTTCAGTTCCGTTC-1	singlet	2	-1307.351777	-1108.940725	-442.2699649	EMD1
ACTTTCATCCTCTAAT-1	singlet	1	-711.3944366	-283.3621033	-675.2487691	EMD1
ACTTTCATCTGACAGT-1	unassigned	2/0	-20.35680142	-23.15390809	-15.64075199	EMD1
ACTTTCATCTGCCTGT-1	singlet	2	-1939.208974	-1773.382582	-663.5051962	EMD1
ACTTTGTCAATTGTGC-1	singlet	2	-82.07320139	-60.57165236	-29.56764313	EMD1
ACTTTGTGTAGCGTTT-1	singlet	2	-254.4158909	-209.3002362	-108.7342506	EMD1
ACTTTGTGTTATAGCC-1	singlet	1	-3529.733601	-1034.601584	-2583.76056	EMD1
ACTTTGTTCGGTATGT-1	singlet	2	-2552.281512	-2050.324209	-865.7635725	EMD1
ACTTTGTTCTCGGTAA-1	singlet	2	-572.6075955	-501.3042245	-243.1038506	EMD1
AGAAATGAGATGACAT-1	singlet	2	-773.4132953	-649.8843692	-285.5241491	EMD1
AGAAATGAGATTAGCA-1	singlet	0	-810.4365577	-2543.600589	-2773.990795	EMD1
AGAAATGAGTTCATGC-1	singlet	1	-3134.974032	-768.6831386	-2319.746535	EMD1
AGAAATGCACGCGTCA-1	singlet	0	-1336.239344	-4390.052022	-4615.312925	EMD1
AGAAATGCATCAGTCA-1	singlet	1	-5766.056529	-1299.642889	-4540.205417	EMD1
AGAAATGGTCAACGCC-1	singlet	0	-33.3513332	-155.5299141	-168.024361	EMD1
AGAAATGGTGTATCCA-1	singlet	2	-215.8843689	-179.0691615	-62.85713336	EMD1
AGAAATGGTGTGCCTG-1	singlet	2	-477.096839	-474.9109813	-203.8877526	EMD1
AGAAATGTCACTCCGT-1	singlet	2	-734.2865235	-619.5065678	-269.9065292	EMD1
AGAAATGTCGGTCTGG-1	singlet	2	-637.6442031	-545.8114823	-244.2827657	EMD1
AGAAATGTCTAGACAC-1	singlet	2	-945.1077639	-736.2288702	-384.5746448	EMD1
AGAACAAAGGCAGCTA-1	singlet	2	-279.50239	-223.6170759	-105.3457269	EMD1
AGAACAAGTGCAATAA-1	singlet	0	-8.617506969	-26.88078946	-24.48604728	EMD1
AGAACAAGTGGATTTC-1	singlet	0	-706.8559671	-2020.67102	-2049.008342	EMD1
AGAACCTAGGCCGCTT-1	singlet	1	-4150.046905	-1258.660091	-3312.894	EMD1
AGAACCTAGGTATTGA-1	singlet	1	-2188.053948	-681.6795847	-1738.912136	EMD1
AGAACCTCAATCAAGA-1	singlet	0	-320.7381766	-1046.130903	-1076.841913	EMD1
AGAACCTCAGACACAG-1	singlet	0	-703.6326317	-2555.1844	-2156.969237	EMD1
AGAACCTCAGCAGATG-1	singlet	1	-1662.594429	-512.1618373	-1313.722561	EMD1
AGAACCTCATTGTGCA-1	singlet	0	-266.2552415	-875.0526117	-735.5048961	EMD1
AGAACCTGTACTTGTG-1	unassigned	2/0	-8.796996847	-13.17245745	-7.085626793	EMD1
AGAACCTGTCAGTTTG-1	singlet	2	-4986.560181	-4767.209134	-2489.328053	EMD1
AGAACCTTCAATCTTC-1	singlet	2	-5834.726733	-4913.153471	-1546.435367	EMD1
AGAACCTTCCGTGTAA-1	singlet	1	-3412.831739	-1211.241797	-2735.332498	EMD1
AGAAGCGAGCTGCCTG-1	singlet	1	-287.284681	-101.7018737	-226.4680775	EMD1
AGAAGCGAGTCGCTAT-1	singlet	2	-1292.407256	-1241.557334	-500.0665108	EMD1
AGAAGCGCAAGCAGGT-1	singlet	0	-92.78558128	-241.6153897	-199.3402047	EMD1
AGAAGCGCACCCAATA-1	singlet	0	-24.88411231	-63.25234683	-86.44549937	EMD1
AGAAGCGCAGCCCAGT-1	singlet	0	-2166.95877	-7566.904766	-7491.352059	EMD1
AGAAGCGGTACCGTCG-1	singlet	2	-4500.601784	-3631.247586	-1455.250321	EMD1
AGAAGCGGTACGCTAT-1	singlet	1	-2251.742424	-583.9817742	-1705.728565	EMD1
AGAAGCGGTCACTGAT-1	singlet	0	-10.88977357	-50.25597781	-47.56705313	EMD1
AGAAGCGTCAAAGACA-1	singlet	1	-2341.867412	-751.4483458	-2083.763456	EMD1
AGAAGTAAGACTCCGC-1	singlet	1	-85.52436386	-41.51871298	-77.98807581	EMD1
AGAAGTAAGCGCATCC-1	singlet	0	-950.1204823	-3220.290513	-2871.482713	EMD1
AGAAGTAAGTTGCTCA-1	singlet	2	-613.3655778	-564.6892277	-234.6260281	EMD1
AGAAGTACACCGGAAA-1	singlet	2	-398.2839687	-319.0809743	-169.25633	EMD1
AGAAGTACATAGTCGT-1	singlet	2	-681.3600883	-639.194089	-291.5840252	EMD1
AGAAGTAGTCCAACGC-1	singlet	0	-192.6307649	-711.2368997	-662.3399734	EMD1
AGAAGTAGTCGCTCGA-1	singlet	2	-678.7119417	-554.6978476	-292.4582079	EMD1
AGAAGTAGTCTCGGAC-1	singlet	1	-666.1092643	-204.0336201	-537.6435986	EMD1
AGAAGTAGTGGACCTC-1	singlet	2	-546.7358003	-482.593795	-188.1204841	EMD1
AGAAGTAGTTAAACAG-1	singlet	2	-527.3344547	-437.4207941	-223.3744789	EMD1
AGAAGTAGTTAAGACA-1	singlet	2	-6590.691171	-5141.14071	-2173.077381	EMD1
AGAAGTAGTTCCTTGC-1	singlet	0	-2967.588128	-8081.234209	-8236.163075	EMD1
AGAAGTATCACTACTT-1	singlet	2	-1544.868519	-1256.474436	-574.6181439	EMD1
AGAAGTATCGGTCTAA-1	singlet	1	-3073.886928	-890.2735664	-2604.696348	EMD1
AGAAGTATCTCACCCA-1	singlet	1	-5449.257196	-1303.526495	-4602.742397	EMD1
AGAAGTATCTCCTGTG-1	singlet	2	-257.5717834	-218.8064616	-106.3134617	EMD1
AGAAGTATCTCGCGTT-1	singlet	1	-4783.929332	-1211.533165	-4056.782584	EMD1
AGACAAACACTTGAAC-1	singlet	0	-1125.204691	-2674.592578	-2650.904447	EMD1
AGACAAAGTTCGCGTG-1	singlet	2	-890.0419276	-664.9290413	-303.4254243	EMD1
AGACAAATCCTTTGAT-1	singlet	2	-3389.750744	-2732.520271	-1138.93451	EMD1

AGACAAATCGGTAGGA-1	singlet	2	-449.3235613	-410.1978555	-196.7132533	EMD1
AGACAAATCTCCAAGA-1	singlet	1	-1213.817363	-447.244489	-922.2271974	EMD1
AGACACTCACATGAAA-1	singlet	2	-3500.28208	-2868.627469	-1271.012153	EMD1
AGACACTCATTCTCTA-1	singlet	2	-716.3690457	-669.2952867	-262.7595757	EMD1
AGACACTGTAACAGTA-1	singlet	2	-2066.736179	-1877.07694	-824.3870634	EMD1
AGACACTGTCCACATA-1	singlet	1	-1641.743112	-535.3485448	-1364.283247	EMD1
AGACACTGTTTCGTAG-1	doublet	0/2	-1097.999068	-1915.241122	-1630.928024	EMD1
AGACACTTCCTACAAG-1	singlet	2	-828.3983067	-804.4987564	-322.2196432	EMD1
AGACAGGAGACGACTG-1	singlet	1	-2142.374535	-732.7937999	-1638.947242	EMD1
AGACAGGAGACGCCAA-1	singlet	1	-1909.639986	-598.8831525	-1436.30001	EMD1
AGACAGGAGGGAGGCA-1	singlet	2	-418.4328101	-309.5475083	-173.8481769	EMD1
AGACAGGCACGACGCT-1	unassigned	45323	-364.7440506	-289.2634119	-167.6220877	EMD1
AGACAGGCACTGAATC-1	singlet	0	-187.6176778	-848.4388788	-808.3016153	EMD1
AGACAGGCAGAGCTAG-1	singlet	2	-548.4508511	-473.8032748	-233.1832451	EMD1
AGACAGGCAGTCGCAC-1	singlet	2	-1292.648051	-1045.03989	-442.0893387	EMD1
AGACAGGGTCGCTCGA-1	singlet	2	-194.9529702	-159.2956075	-62.55466796	EMD1
AGACAGGGTGGACCTC-1	singlet	2	-4107.506767	-3504.817069	-1464.500802	EMD1
AGACAGGGTTATGTCG-1	singlet	2	-976.0431164	-749.9616089	-370.1822874	EMD1
AGACAGGTCCACACCT-1	singlet	2	-4737.089615	-3828.155079	-1578.10539	EMD1
AGACAGGTCGGCTTCT-1	singlet	2	-590.2153579	-518.1505715	-241.6189726	EMD1
AGACAGGTCTCGTGGG-1	singlet	0	-142.2328514	-425.8603127	-438.6749284	EMD1
AGACCATAGACTCCGC-1	singlet	2	-13.23984986	-12.69115002	-7.929700857	EMD1
AGACCATAGGATTTAG-1	singlet	2	-564.778252	-528.9786683	-220.6817691	EMD1
AGACCATAGGCTCAAG-1	singlet	0	-228.4901772	-712.2289529	-716.4611204	EMD1
AGACCATAGTCGAAAT-1	singlet	2	-688.823622	-600.8724137	-259.2304762	EMD1
AGACCATCACAGCATT-1	singlet	0	-227.6355458	-685.5542162	-832.6460116	EMD1
AGACCATCATCCTAAG-1	singlet	1	-2289.581815	-784.6222514	-1702.850832	EMD1
AGACCATGTAATGTGA-1	singlet	1	-902.8273385	-262.3735105	-811.8728266	EMD1
AGACCATGTCATCCGG-1	singlet	0	-696.1547741	-2168.279114	-2058.757629	EMD1
AGACCATTCTAATTCC-1	singlet	1	-1454.512099	-519.750584	-1210.625433	EMD1
AGACCATTCTGACGCG-1	singlet	0	-653.2051546	-2071.691822	-1975.602334	EMD1
AGACCCGCACAAGCTT-1	singlet	0	-1626.848021	-4885.880392	-4771.979978	EMD1
AGACCCGCACGGTCTG-1	doublet	0/2	-1186.731821	-1921.26488	-1702.758286	EMD1
AGACCCGTCTGGGAGA-1	doublet	0/1	-824.6765502	-932.6891524	-1180.011163	EMD1
AGACTCAAGACTTCAC-1	singlet	2	-693.3700448	-632.2905248	-301.3384164	EMD1
AGACTCACACTCTGCT-1	singlet	0	-442.0951688	-1507.583341	-1610.490307	EMD1
AGACTCAGTACCTAAC-1	singlet	2	-374.1308913	-366.4013329	-157.5861714	EMD1
AGACTCAGTACGATTC-1	singlet	2	-1836.981736	-1742.334376	-538.9076679	EMD1
AGACTCAGTAGCTGAG-1	singlet	2	-182.378378	-176.8793937	-78.54054043	EMD1
AGACTCAGTCCACATA-1	singlet	2	-1789.486983	-1625.210788	-638.1739266	EMD1
AGACTCAGTTTCGTGA-1	singlet	2	-379.684767	-346.4808922	-180.4427659	EMD1
AGACTCATCCCAGTGG-1	singlet	1	-3188.875355	-970.868173	-2401.368609	EMD1
AGACTCATCGATCCAA-1	singlet	2	-1003.37319	-942.6135106	-323.9377187	EMD1
AGACTCATCGGAACTT-1	singlet	2	-1553.397313	-1283.939245	-562.5925619	EMD1
AGACTCATCTATCCAT-1	singlet	2	-645.3105861	-557.4070751	-279.9587383	EMD1
AGAGAATAGAATCGTA-1	singlet	1	-3466.472164	-885.7120882	-2675.182572	EMD1
AGAGAATAGGTACATA-1	singlet	2	-489.7990794	-474.506845	-212.8730509	EMD1
AGAGAATGTAACAGGC-1	singlet	2	-675.5900167	-564.1329777	-290.8655226	EMD1
AGAGAATGTAATGATG-1	singlet	1	-662.5833524	-297.8097951	-608.6898693	EMD1
AGAGAATGTTTGAAAG-1	singlet	2	-10471.10881	-8229.239385	-3366.524902	EMD1
AGAGAATTCCAACTAG-1	singlet	0	-392.2707324	-1107.294569	-1108.25711	EMD1
AGAGAATTCGGCAGTC-1	singlet	0	-175.2266828	-362.8717843	-367.7021188	EMD1
AGAGAATTCTGCGGGT-1	singlet	1	-1910.918937	-632.868672	-1521.710293	EMD1
AGAGAGCAGAAACTCA-1	singlet	2	-12606.47797	-10050.28037	-4087.448371	EMD1
AGAGAGCAGGACGCTA-1	singlet	1	-3559.737063	-889.4293167	-2935.894864	EMD1
AGAGAGCAGTCCCGGT-1	singlet	1	-3450.552296	-1008.529513	-2582.649941	EMD1
AGAGAGCCAATGTCTG-1	singlet	0	-332.1473428	-873.4100855	-865.689307	EMD1
AGAGAGCCACCCTATC-1	singlet	1	-1326.796789	-485.3727463	-964.4866235	EMD1
AGAGAGCCACGCTATA-1	singlet	0	-168.2137576	-669.4298961	-512.5226514	EMD1
AGAGAGCCACGGCCAT-1	singlet	2	-1471.406365	-1440.145922	-587.0871009	EMD1
AGAGAGCCACTTTAGG-1	singlet	2	-349.8968532	-388.4477601	-162.5393063	EMD1
AGAGAGCGTAAGGCCA-1	singlet	2	-1788.960595	-1544.475225	-654.8053626	EMD1
AGAGAGCTCATTACTC-1	singlet	1	-2064.91728	-533.2549142	-1782.745127	EMD1
AGAGAGCTCGCTTACC-1	singlet	2	-374.4115809	-328.7041179	-158.5243749	EMD1
AGAGAGCTCTCGAGTA-1	singlet	2	-235.7205129	-216.9279173	-86.42995511	EMD1
AGAGAGCTCTGTGTGA-1	singlet	2	-1774.822217	-1648.581569	-626.8623848	EMD1
AGAGCAGAGATGCGAC-1	singlet	0	-477.3507251	-1836.568565	-1644.110196	EMD1
AGAGCAGAGGAATCGC-1	singlet	0	-351.1836665	-1061.629479	-1041.913877	EMD1
AGAGCAGCATCGGAGA-1	unassigned	0/1	-29.11461464	-38.24620858	-265.477175	EMD1
AGAGCAGGTTCTCCCA-1	singlet	1	-3994.511894	-921.7060827	-3215.151137	EMD1
AGAGCAGGTTTACTTC-1	singlet	1	-400.4761908	-154.2522003	-275.4669849	EMD1
AGAGCAGTCAACTGAC-1	singlet	1	-3765.80227	-1047.04554	-2851.2036	EMD1
AGAGCAGTCCTCTTTC-1	singlet	2	-3168.731527	-2503.373493	-1083.500695	EMD1
AGAGCAGTCGACCCAG-1	singlet	2	-434.470399	-353.9211346	-164.2626934	EMD1
AGAGCAGTCGGTGTAT-1	singlet	2	-284.6130608	-255.9643312	-120.7961095	EMD1
AGAGCCCAGCACCCAC-1	singlet	2	-415.9079839	-361.0275347	-200.3895408	EMD1
AGAGCCCAGTGGCGAT-1	singlet	0	-336.800347	-1044.068993	-927.1493409	EMD1
AGAGCCCAGTTTGAGA-1	singlet	2	-586.5626813	-434.5182856	-223.4500634	EMD1
AGAGCCCCACTGAATC-1	singlet	2	-309.7017487	-280.0561628	-143.2972016	EMD1
AGAGCCCCATCGATCA-1	singlet	0	-782.6890466	-2089.591374	-2160.563362	EMD1
AGAGCCCGTATCGTTG-1	singlet	0	-202.3829217	-558.2895866	-555.9267904	EMD1
AGAGCCCGTCTCGGGT-1	singlet	0	-207.809574	-666.2896102	-650.3502865	EMD1
AGAGCCCTCAAGCCTA-1	singlet	0	-140.4091086	-412.4114493	-395.8181591	EMD1
AGAGCCCTCGCTTACC-1	singlet	0	-606.8445435	-1843.084702	-1843.585499	EMD1
AGAGCCCTCTATACTC-1	singlet	2	-1229.968411	-1159.833707	-505.8377503	EMD1
AGAGCCCTCTCCGTGT-1	singlet	2	-371.4181281	-286.1881413	-147.6580182	EMD1
AGATAGAAGGTTAAAC-1	singlet	2	-508.750376	-383.3712079	-169.3632595	EMD1
AGATAGACAATCGAAA-1	singlet	2	-693.9262639	-573.6825575	-275.7148424	EMD1
AGATAGAGTGCGAACA-1	singlet	0	-220.8327374	-766.5029851	-731.0216574	EMD1
AGATAGAGTGCGTTTA-1	singlet	1	-745.8327503	-233.8622141	-566.713628	EMD1
AGATAGATCACAGTGT-1	singlet	2	-310.0819177	-261.8122083	-110.9189868	EMD1
AGATAGATCGCTACGG-1	singlet	2	-1799.772715	-1482.55405	-678.7828355	EMD1
AGATAGATCTCAACGA-1	singlet	0	-3947.871584	-15037.0849	-15251.28846	EMD1
AGATAGATCTGCGGAC-1	singlet	2	-391.6134744	-347.8743379	-132.6030901	EMD1
AGATCCACAATTCGTG-1	singlet	2	-695.3599265	-602.4680537	-250.8470383	EMD1
AGATCCACACCGGTCA-1	singlet	0	-602.3693185	-2461.929983	-2121.807226	EMD1
AGATCCAGTGCAACGA-1	singlet	2	-2358.80834	-2109.306199	-705.7572185	EMD1
AGATCCATCATGTCTT-1	singlet	0	-1205.64867	-3475.242771	-3500.989991	EMD1
AGATCCATCCAAACCA-1	singlet	0	-1308.508445	-4763.477256	-4535.838539	EMD1
AGATCCATCTACGCAA-1	singlet	0	-240.5241705	-692.0354281	-679.7250456	EMD1
AGATCGTAGTGCGTCC-1	singlet	0	-573.4507178	-1341.19856	-1266.739513	EMD1
AGATCGTCAATTTCTC-1	singlet	2	-379.2091181	-360.5184115	-158.6367727	EMD1
AGATCGTCAGGGAATC-1	singlet	2	-397.0905475	-344.233642	-181.9939684	EMD1
AGATCGTGTAACGATA-1	singlet	1	-1751.491758	-444.7081178	-1268.546786	EMD1
AGATCGTGTGTACAGG-1	singlet	2	-130.6295033	-90.94517591	-46.39824641	EMD1
AGATCGTTCATTGCCC-1	singlet	2	-756.5810483	-812.0449899	-262.9063757	EMD1
AGATGAAAGAGGGTGG-1	doublet	0/1	-2398.674065	-3373.653839	-4184.916578	EMD1
AGATGAACACAATCTG-1	singlet	0	-985.076954	-3778.87107	-3203.513006	EMD1
AGATGAACACCGGCTA-1	doublet	2/0	-1611.307119	-2250.045336	-1391.349632	EMD1
AGATGAACATCCTAAG-1	singlet	0	-677.6090114	-2204.754006	-2199.159084	EMD1

AGATGAAGTCATAACC-1	singlet	2	-461.0135103	-401.9175593	-170.4168844	EMD1
AGATGAAGTCCCTCAT-1	singlet	0	-25.19690112	-96.43964966	-97.85092287	EMD1
AGATGAATCATTCGTT-1	singlet	0	-647.2094758	-1805.957536	-1667.334638	EMD1
AGATGAATCGAGAAAT-1	singlet	2	-513.4260729	-420.1765427	-192.2008342	EMD1
AGATGAATCTTGGATG-1	singlet	2	-183.6110685	-143.4828187	-57.89948909	EMD1
AGATGCTAGAAGGTAG-1	singlet	2	-814.2145003	-674.287604	-356.8687065	EMD1
AGATGCTAGTACCGGA-1	singlet	2	-1292.809096	-1139.05692	-463.5314913	EMD1
AGATGCTAGTCACGAG-1	singlet	1	-413.6254795	-172.3286009	-434.0423725	EMD1
AGATGCTCAACTAGAA-1	singlet	1	-588.2727083	-241.8663349	-643.609702	EMD1
AGATGCTCAACTCCAA-1	singlet	1	-168.3430689	-55.25256321	-133.4778517	EMD1
AGATGCTCACTTCCTG-1	singlet	1	-1424.467082	-549.5406408	-1076.021334	EMD1
AGATGCTCAGGGTTGA-1	singlet	0	-93.53370197	-241.6530696	-246.3686268	EMD1
AGATGCTCATTGACCA-1	singlet	2	-902.7113634	-848.492035	-387.2740453	EMD1
AGATGCTGTAACCCGC-1	singlet	0	-65.97386709	-261.4373477	-274.3278279	EMD1
AGATGCTGTCAACCTA-1	singlet	2	-391.9791104	-333.370848	-129.1574973	EMD1
AGATGCTGTGATTCAC-1	singlet	2	-607.7581419	-604.1080344	-252.9174297	EMD1
AGATGCTTCAAGTGTC-1	singlet	0	-235.8675028	-766.822875	-779.1403972	EMD1
AGATGCTTCCATAGAC-1	singlet	0	-276.2276785	-814.9861974	-859.0838931	EMD1
AGATGCTTCCGTTTCG-1	singlet	2	-410.8229102	-367.7752398	-201.9269025	EMD1
AGATGCTTCCTTATAC-1	singlet	0	-262.3290652	-769.2718597	-757.065191	EMD1
AGATGCTTCGACGCTG-1	singlet	0	-94.57438916	-208.666211	-217.4893213	EMD1
AGATGCTTCTGCCCTA-1	singlet	2	-655.3556007	-570.3447088	-298.2963928	EMD1
AGCATCAAGCTAATGA-1	singlet	0	-238.2595953	-819.6709884	-678.2761262	EMD1
AGCATCAAGGACGCTA-1	singlet	2	-511.1622386	-410.3601019	-211.9206981	EMD1
AGCATCAAGTGTACCT-1	singlet	0	-1896.654588	-5854.408994	-5733.311909	EMD1
AGCATCACAGATACTC-1	singlet	1	-3018.534648	-947.5436091	-2312.855674	EMD1
AGCATCAGTCAAAGTA-1	singlet	0	-679.3144255	-2046.364775	-2068.072091	EMD1
AGCATCAGTCATAGTC-1	singlet	2	-698.5608339	-613.7040584	-329.5525015	EMD1
AGCATCAGTCCCGCAA-1	singlet	1	-273.4416325	-97.10993726	-226.9426417	EMD1
AGCATCAGTCTACAAC-1	doublet	1/0	-2014.813608	-1170.202865	-2073.797114	EMD1
AGCATCATCCATGAGT-1	singlet	2	-373.7444353	-381.2764256	-175.5891377	EMD1
AGCCAATAGACCTCCG-1	singlet	2	-2179.079712	-1962.759601	-807.5935335	EMD1
AGCCAATAGACTGTTC-1	singlet	2	-374.3444201	-369.5718911	-147.5026641	EMD1
AGCCAATAGAGCACTG-1	singlet	2	-184.5907791	-148.1100809	-54.04677756	EMD1
AGCCAATAGGAGTACC-1	singlet	1	-584.596651	-191.3622398	-435.5552988	EMD1
AGCCAATCAAAGGCGT-1	singlet	1	-539.4278522	-182.2017917	-384.9144961	EMD1
AGCCAATCAATGTTGC-1	singlet	0	-1418.73532	-4227.596756	-3920.032656	EMD1
AGCCAATCATCCGGCA-1	singlet	0	-256.9707003	-702.7067733	-745.453121	EMD1
AGCCAATGTATGGTTC-1	doublet	45293	-3365.003932	-1434.17889	-2433.597449	EMD1
AGCCAATGTATGTGTC-1	singlet	1	-2253.742416	-1139.027573	-1938.492142	EMD1
AGCCAATGTTCGAAGG-1	singlet	2	-364.863865	-345.3497693	-148.2982873	EMD1
AGCCAATGTTGTAGCT-1	singlet	0	-287.3668463	-955.7559767	-855.1613238	EMD1
AGCCAATTCAAAGAAC-1	singlet	2	-698.3301302	-553.5664818	-291.6228957	EMD1
AGCCAATTCGCATAGT-1	singlet	1	-6481.063011	-1979.060539	-4841.090296	EMD1
AGCCAATTCGGATTAC-1	singlet	1	-189.7717923	-45.43021612	-137.5882395	EMD1
AGCCACGAGACGAGCT-1	singlet	0	-124.0489496	-337.9212705	-451.6784836	EMD1
AGCCACGAGGCTCACC-1	singlet	2	-416.1776979	-486.084248	-150.9339183	EMD1
AGCCACGCACATCCCT-1	singlet	1	-1828.156933	-619.1596348	-1437.00777	EMD1
AGCCACGCACGAAGAC-1	singlet	2	-907.3320287	-810.7668213	-382.1880358	EMD1
AGCCACGCAGCTTCGG-1	singlet	0	-26.09484431	-78.62715239	-92.03136398	EMD1
AGCCACGGTCGCATCG-1	singlet	2	-750.6750576	-557.7049551	-242.5226035	EMD1
AGCCACGGTGGCAACA-1	singlet	2	-447.6583732	-360.5188267	-163.9293069	EMD1
AGCCACGGTTAATGAG-1	singlet	0	-1003.790265	-3542.492788	-3309.635075	EMD1
AGCCACGTCAACCTTT-1	singlet	2	-155.8780009	-144.6687002	-59.34835882	EMD1
AGCCACGTCGCCAGTG-1	singlet	2	-624.16062	-460.9974382	-196.9777344	EMD1
AGCCAGCAGACTACGG-1	singlet	2	-211.4879919	-200.1999987	-101.4945012	EMD1
AGCCAGCAGAGCAGTC-1	singlet	2	-490.0722861	-404.6299581	-191.5825981	EMD1
AGCCAGCAGGTGGTTG-1	singlet	2	-266.8869299	-201.7862928	-106.5218745	EMD1
AGCCAGCAGTATTCCG-1	singlet	0	-293.7951879	-832.842018	-907.9127569	EMD1
AGCCAGCAGTCCCTAA-1	singlet	0	-1423.969542	-6437.533666	-5491.622166	EMD1
AGCCAGCCAGCGACAA-1	singlet	0	-56.30867266	-170.7536639	-171.554389	EMD1
AGCCAGCGTCCACTCT-1	singlet	2	-4293.66594	-3306.358662	-1274.593787	EMD1
AGCCAGCGTGTTATCG-1	singlet	0	-1156.404328	-4615.100621	-4270.484881	EMD1
AGCGATTAGGACTGGT-1	singlet	2	-292.1661447	-248.2429863	-123.5978798	EMD1
AGCGATTCAGAGTGAC-1	singlet	2	-562.5092975	-511.1135159	-233.2153309	EMD1
AGCGATTCAGCGCGTT-1	singlet	2	-677.3510596	-634.1920124	-270.560591	EMD1
AGCGATTGTACGCGTC-1	singlet	2	-1161.861197	-1042.41854	-420.235648	EMD1
AGCGATTGTGTCTCCT-1	singlet	0	-273.3997024	-918.7750674	-1067.721183	EMD1
AGCGATTGTTATGGTC-1	singlet	2	-709.399544	-569.4223242	-251.469294	EMD1
AGCGATTTCCGTGTAA-1	singlet	0	-341.9443868	-879.6443296	-978.9304958	EMD1
AGCGCCAAGCGTTCAT-1	singlet	0	-94.26208141	-340.4808011	-315.3175445	EMD1
AGCGCCAAGGCTTCCG-1	singlet	2	-904.519869	-772.6187984	-363.0694308	EMD1
AGCGCCAAGGGTTAAT-1	singlet	1	-2280.623893	-702.5630264	-1790.151523	EMD1
AGCGCCACAAGCCATT-1	singlet	0	-13.46104905	-45.6734593	-55.66026756	EMD1
AGCGCCACAATGGCCC-1	singlet	2	-1861.475493	-1473.109534	-623.320937	EMD1
AGCGCCACACCTCAGG-1	singlet	0	-890.2858264	-2469.694418	-2494.843108	EMD1
AGCGCCACAGAGGCTA-1	singlet	1	-646.7873375	-229.4394225	-671.4313041	EMD1
AGCGCCACAGCATCTA-1	singlet	0	-201.7250839	-562.711681	-582.7707127	EMD1
AGCGCCAGTCGTTGCG-1	singlet	0	-151.346553	-367.322122	-339.8510447	EMD1
AGCGCCAGTCTGCAAT-1	singlet	2	-199.9053877	-183.1012867	-84.27191795	EMD1
AGCGCCAGTTTACACG-1	singlet	2	-444.5310936	-364.2843844	-178.2200179	EMD1
AGCGCCATCAGGGATG-1	singlet	0	-235.6960488	-615.1714952	-755.773906	EMD1
AGCGCCATCCCAGTGG-1	singlet	2	-2551.493065	-2186.980981	-725.3290566	EMD1
AGCGCTGAGTCTCCTC-1	singlet	2	-318.3774053	-292.9896098	-106.0004007	EMD1
AGCGCTGAGTTAGTGA-1	singlet	2	-370.8809694	-318.3396097	-158.1582927	EMD1
AGCGCTGCACCAGCTG-1	singlet	2	-407.0578576	-322.7621862	-164.780373	EMD1
AGCGCTGGTCGAGCAA-1	singlet	0	-883.1198953	-2624.483015	-2573.758907	EMD1
AGCGCTGTCAGAGTGG-1	singlet	1	-2646.515977	-699.392671	-1680.803341	EMD1
AGCGCTGTCGTAATGC-1	doublet	0/1	-15.13439678	-18.50528635	-211.4212693	EMD1
AGCGTATAGGTGAGCT-1	singlet	0	-1073.969826	-3510.756103	-3429.710868	EMD1
AGCGTATAGGTTACAA-1	singlet	2	-2022.991546	-2009.891909	-726.5179227	EMD1
AGCGTATGTCGCATGC-1	singlet	0	-371.4936303	-2240.278151	-1821.976013	EMD1
AGCGTATGTTAGGCTT-1	singlet	1	-75.33020581	-25.6251594	-46.11552846	EMD1
AGCGTATGTTCCTAGA-1	singlet	0	-305.407254	-842.8965851	-927.9183186	EMD1
AGCGTATTCAGACCCG-1	singlet	2	-2001.054719	-1716.521095	-778.9162954	EMD1
AGCGTATTCCAACTGA-1	singlet	0	-531.229538	-2508.817472	-2278.18139	EMD1
AGCGTATTCCATTTAC-1	singlet	0	-402.9447688	-1370.294209	-1266.674515	EMD1
AGCGTATTCGGAATTC-1	singlet	0	-662.4874915	-2048.355888	-2003.350561	EMD1
AGCGTATTCTATACGG-1	singlet	2	-582.850563	-430.7181429	-216.0787985	EMD1
AGCGTCGAGGATTACA-1	singlet	1	-993.3263788	-259.1682194	-783.8579283	EMD1
AGCGTCGGTAACTTCG-1	singlet	2	-391.4160829	-299.6960853	-116.3137612	EMD1
AGCGTCGGTATTTCCT-1	singlet	0	-168.0310712	-671.7095265	-565.8731605	EMD1
AGCGTCGGTGCCCGTA-1	singlet	0	-34.75045611	-134.2125194	-141.0178127	EMD1
AGCTACAAGACCTGGA-1	singlet	2	-1992.971113	-1583.246683	-688.5273999	EMD1
AGCTACAAGCTCGTGC-1	singlet	0	-412.087292	-1516.440367	-1541.783612	EMD1
AGCTACACAAACAGGC-1	singlet	2	-1452.738253	-1256.708234	-514.0638678	EMD1
AGCTACACATGCTGCG-1	singlet	0	-461.3751073	-1682.09951	-1648.263127	EMD1
AGCTACAGTAGCACAG-1	singlet	2	-3721.280211	-3030.495972	-1376.371858	EMD1
AGCTACAGTCAGACTT-1	singlet	2	-7081.64047	-5364.425491	-2279.365278	EMD1
AGCTACAGTGAGATAT-1	singlet	1	-2023.778349	-598.6369444	-1560.640213	EMD1

AGCTACAGTGCAGTGA-1	singlet	2	-528.3941258	-444.5761033	-241.6322499	EMD1
AGCTACAGTGCTAGCC-1	singlet	0	-1679.950561	-3278.59275	-3613.537033	EMD1
AGCTCAAAGGCGCTCT-1	singlet	0	-171.7411172	-463.0867476	-406.9244658	EMD1
AGCTCAACAATACAGA-1	singlet	0	-2827.145127	-8074.984156	-7982.237286	EMD1
AGCTCAACACCAGCTG-1	singlet	2	-1117.869465	-962.7737414	-386.6020585	EMD1
AGCTCAACAGCGGATA-1	singlet	2	-511.192013	-381.0322251	-169.9885709	EMD1
AGCTCAACATCCGGCA-1	singlet	0	-780.9588015	-2541.006978	-2427.155228	EMD1
AGCTCAACATGACAAA-1	singlet	0	-223.170574	-633.2947973	-624.8279196	EMD1
AGCTCAAGTAGTACGG-1	singlet	0	-1968.620157	-7929.281085	-7103.146591	EMD1
AGCTCAAGTCGCTTAA-1	singlet	2	-168.2300753	-177.0502302	-86.8138219	EMD1
AGCTCAAGTTCCCAAA-1	singlet	0	-148.7693494	-470.1300172	-651.2242958	EMD1
AGCTCAATCCGGTAGC-1	singlet	1	-2211.388163	-793.454886	-1726.008061	EMD1
AGCTCAATCCGTAATG-1	singlet	2	-204.3396032	-173.0854309	-117.8797495	EMD1
AGCTCAATCTGCTGAA-1	singlet	2	-461.5024082	-425.7133017	-186.9666508	EMD1
AGCTTCCAGACTGTTC-1	singlet	2	-517.5015284	-487.8543841	-220.7573288	EMD1
AGCTTCCCACACCGCA-1	singlet	0	-1433.485276	-4164.12082	-4157.772726	EMD1
AGCTTCCCACACCTTC-1	singlet	2	-1606.222573	-1332.105354	-475.3742128	EMD1
AGCTTCCGTACGACTT-1	singlet	1	-504.9368347	-210.2023956	-403.1019665	EMD1
AGCTTCCGTACTCGAT-1	singlet	0	-206.6108065	-733.4286065	-726.9294328	EMD1
AGCTTCCGTTGCCAAT-1	singlet	0	-449.3839683	-1106.008695	-1053.774358	EMD1
AGCTTCCGTTTGTTGG-1	singlet	1	-653.9685382	-205.3611913	-457.5612581	EMD1
AGCTTCCTCCAAACCA-1	singlet	0	-1452.476104	-3778.261412	-3686.085496	EMD1
AGCTTCCTCCCGAGTG-1	singlet	0	-2008.160353	-7808.548278	-7213.909911	EMD1
AGGAAATCAGGTCCCA-1	singlet	2	-378.282522	-354.7110415	-163.9769756	EMD1
AGGAAATCAGGTGACA-1	singlet	0	-530.3357283	-1562.953429	-1462.138941	EMD1
AGGAAATTCAGAATAG-1	singlet	2	-637.4669891	-495.0575561	-225.8007556	EMD1
AGGAAATTCCAGGACC-1	singlet	2	-460.3513824	-371.7218673	-176.0850479	EMD1
AGGAAATTCCGGCTTT-1	singlet	1	-703.6452861	-287.3803303	-631.5547389	EMD1
AGGAAATTCGATACTG-1	singlet	2	-599.4680171	-599.9944073	-248.2308823	EMD1
AGGAAATTCGGATACT-1	singlet	2	-95.17608166	-60.81626847	-25.0354836	EMD1
AGGAATAAGAGCATAT-1	singlet	0	-71.38534937	-263.6889856	-247.8621045	EMD1
AGGAATAAGTAGTCCT-1	singlet	1	-218.3267999	-102.5780981	-173.1210137	EMD1
AGGAATACACGCTGCA-1	singlet	0	-180.8545954	-553.2940164	-517.7040483	EMD1
AGGAATACACTACTTT-1	singlet	2	-2795.504542	-2643.393625	-960.0326059	EMD1
AGGAATACACTCCGAG-1	singlet	2	-271.0433835	-224.1227212	-103.0339167	EMD1
AGGAATACATCTATCT-1	singlet	0	-234.913614	-845.1925852	-902.8759637	EMD1
AGGAATATCCTCCACA-1	singlet	1	-1206.98678	-497.1732786	-961.0388893	EMD1
AGGACGAAGCATGCAG-1	singlet	0	-62.74332308	-204.3918103	-140.5530356	EMD1
AGGACGAAGCGACATG-1	singlet	2	-248.1374714	-225.827114	-75.03743345	EMD1
AGGACGAGTGTGTACT-1	singlet	2	-610.5765933	-522.2142905	-248.2088926	EMD1
AGGACGAGTGTTATCG-1	singlet	2	-4449.720535	-3414.903453	-1269.264325	EMD1
AGGACGATCGACGCTG-1	singlet	1	-85.2652404	-21.26654133	-54.63321946	EMD1
AGGACGATCGCTTGAA-1	singlet	2	-474.2543289	-399.2614923	-185.0622386	EMD1
AGGACTTAGCCTTTCC-1	unassigned	0/1	-4.707698544	-5.791297511	-3.500756189	EMD1
AGGACTTAGGCACTAG-1	singlet	0	-14.71972899	-27.03738766	-175.8803147	EMD1
AGGACTTCACCCATAA-1	singlet	2	-216.5892808	-172.6326793	-82.18623161	EMD1
AGGACTTCACTCAGAT-1	singlet	2	-404.478768	-291.5575551	-143.7850205	EMD1
AGGACTTCATTAGGAA-1	singlet	2	-352.4189911	-281.5252684	-148.8170691	EMD1
AGGACTTGTACACTCA-1	singlet	2	-383.1710194	-314.5265886	-154.6025687	EMD1
AGGACTTGTTATCTGG-1	singlet	2	-400.7754875	-365.5998074	-128.880816	EMD1
AGGACTTTCAATCTCT-1	singlet	0	-473.0766418	-1580.882971	-1735.205783	EMD1
AGGACTTTCAATCTTC-1	singlet	2	-8430.803968	-7852.015864	-2265.651057	EMD1
AGGACTTTCAGCGCAC-1	singlet	2	-321.5990636	-277.4355464	-137.4097954	EMD1
AGGAGGTAGTCCCGAC-1	singlet	2	-288.9280947	-249.9135924	-108.4663318	EMD1
AGGAGGTGTAACGTTC-1	singlet	0	-13.31874974	-16.74107987	-203.4128806	EMD1
AGGAGGTGTATGGTTC-1	singlet	1	-1552.91365	-514.1390798	-1231.543246	EMD1
AGGAGGTGTTCTAAGC-1	singlet	0	-215.0832523	-513.0971739	-577.7389664	EMD1
AGGAGGTTCCGCTTAC-1	singlet	2	-655.7252262	-527.07113	-211.9527352	EMD1
AGGAGGTTCGGCATCG-1	singlet	2	-401.4279219	-329.7300366	-157.9152583	EMD1
AGGATAAAGCGTCAGA-1	singlet	2	-226.8708825	-219.4215419	-126.8896833	EMD1
AGGATAAAGGTTCTTG-1	singlet	2	-539.3866593	-492.2661642	-225.4406853	EMD1
AGGATAAAGTACAACA-1	singlet	1	-339.2982443	-63.22566401	-224.4525616	EMD1
AGGATAACACCCAATA-1	singlet	0	-69.28217463	-232.3300945	-223.9723426	EMD1
AGGATAACATCAGCGC-1	singlet	0	-284.7701316	-967.0836281	-914.1054244	EMD1
AGGATAAGTGGTACAG-1	singlet	1	-384.3438521	-100.1722938	-263.1488222	EMD1
AGGATAAGTTGCGTAT-1	singlet	2	-513.5391711	-460.2476959	-232.177703	EMD1
AGGATAATCGTCTCAC-1	singlet	1	-11179.68237	-1922.719628	-7249.732394	EMD1
AGGATCTAGCTGGTGA-1	singlet	2	-3768.336842	-3024.64764	-1294.609388	EMD1
AGGATCTAGTAGGTTA-1	singlet	2	-2671.305331	-2240.739907	-894.5287099	EMD1
AGGATCTAGTGATCGG-1	singlet	1	-2240.703667	-582.5993666	-1882.863309	EMD1
AGGATCTCAGCACCCA-1	singlet	0	-513.4546989	-1366.771487	-1275.072062	EMD1
AGGATCTGTGCGCTCA-1	singlet	0	-569.4125254	-1701.14614	-1673.229172	EMD1
AGGATCTGTTGTCCCT-1	singlet	1	-482.8226006	-192.2140593	-438.7459878	EMD1
AGGATCTTCCCATGGG-1	singlet	1	-2612.880181	-793.576425	-1966.784495	EMD1
AGGATCTTCGACGCTG-1	singlet	2	-176.4813473	-134.5325492	-51.05335991	EMD1
AGGCATTAGCCGCACT-1	singlet	1	-1374.091441	-388.9772536	-1161.961626	EMD1
AGGCATTAGCGAAACC-1	singlet	0	-313.2253813	-896.8012171	-839.561964	EMD1
AGGCATTAGCTAGAAT-1	singlet	1	-716.1655042	-254.9123122	-582.9466971	EMD1
AGGCATTAGTCTAGAA-1	singlet	0	-212.3864473	-643.7653759	-775.7337133	EMD1
AGGCATTGTACCGGAA-1	singlet	1	-1096.833784	-361.6915375	-882.7182528	EMD1
AGGCCACAGAAGAGCA-1	singlet	1	-6870.929016	-1653.913664	-5531.4777	EMD1
AGGCCACAGAAGGCTC-1	singlet	2	-2448.548408	-1945.016832	-912.2670899	EMD1
AGGCCACAGGAGTCTG-1	singlet	2	-450.3646663	-394.930281	-192.6793436	EMD1
AGGCCACCAAGCTCTA-1	singlet	2	-1960.296846	-1488.965694	-598.3460698	EMD1
AGGCCACCAAGTATAG-1	singlet	2	-484.7453515	-403.7937536	-213.0117627	EMD1
AGGCCACCATAACGGG-1	singlet	2	-198.794468	-148.2374202	-74.58769257	EMD1
AGGCCACGTACGTGAG-1	singlet	2	-1115.735497	-1006.12353	-460.3684484	EMD1
AGGCCACGTGATGAAT-1	singlet	0	-148.5078104	-480.6122628	-448.3438793	EMD1
AGGCCACGTGCCGGTT-1	singlet	1	-2339.440239	-642.0618461	-1727.422772	EMD1
AGGCCACTCTCGTTTA-1	singlet	2	-1526.644933	-1436.490483	-630.7976944	EMD1
AGGCCACTCTTCGACC-1	singlet	2	-12549.31718	-10255.24449	-3705.088885	EMD1
AGGCTGCAGCATGCGA-1	singlet	1	-979.2305949	-197.8654943	-723.0672707	EMD1
AGGCTGCCATCGGTTA-1	singlet	0	-264.3435642	-791.6255039	-730.2307208	EMD1
AGGCTGCGTAACCCGC-1	singlet	2	-1055.400785	-951.6505347	-377.837143	EMD1
AGGCTGCGTGCACGCT-1	singlet	0	-219.8097425	-622.7920651	-641.1640055	EMD1
AGGCTGCGTGCCGTAC-1	singlet	2	-187.1340207	-188.2089852	-91.0925114	EMD1
AGGCTGCGTTAGCTAC-1	singlet	1	-344.121269	-148.704947	-352.8750014	EMD1
AGGCTGCGTTTCGACA-1	singlet	0	-200.0644705	-553.7598388	-707.9585136	EMD1
AGGCTGCTCAACTGGT-1	singlet	1	-1507.524437	-586.1719763	-1271.87069	EMD1
AGGCTGCTCACTCCGT-1	singlet	2	-738.7819193	-655.0678714	-272.7460272	EMD1
AGGCTGCTCATTGCGA-1	singlet	2	-553.4456649	-430.0974749	-209.6332232	EMD1
AGGGAGTAGACTCGAG-1	singlet	2	-326.718161	-269.8125029	-121.2869843	EMD1
AGGGAGTAGGTAATCA-1	singlet	2	-1763.16763	-1601.48552	-618.3031047	EMD1
AGGGAGTAGTGACACG-1	singlet	2	-615.3510748	-490.1255673	-241.7779206	EMD1
AGGGAGTCAGTAGAAT-1	singlet	2	-232.4673877	-209.2436902	-90.12552391	EMD1
AGGGAGTCATAGAATG-1	singlet	2	-415.3809646	-338.006866	-168.3470627	EMD1
AGGGAGTCATATGGCT-1	singlet	1	-3005.076601	-869.6851348	-2043.361307	EMD1
AGGGAGTTCGCAGATT-1	singlet	2	-1909.280839	-1829.611545	-621.5936134	EMD1
AGGGAGTTCGTCGATA-1	singlet	2	-275.8718878	-240.3342316	-104.2994621	EMD1
AGGGCCTAGCTTAGTC-1	singlet	2	-554.7538757	-476.3880662	-219.7057473	EMD1

AGGGCCTAGGGTCACA-1	singlet	1	-5491.442474	-1133.043393	-4219.979574	EMD1
AGGGCCTAGTAGTGCG-1	singlet	1	-2480.088937	-736.5786889	-2112.383894	EMD1
AGGGCCTCATTACGGT-1	singlet	0	-912.0943611	-3104.206578	-2558.273838	EMD1
AGGGCCTGTAGTGGCA-1	singlet	2	-329.2426936	-251.4409464	-124.2538119	EMD1
AGGGCCTTCTCCGAAA-1	singlet	2	-3633.864807	-2905.415677	-1114.83785	EMD1
AGGGCTCAGCCGCTTG-1	singlet	1	-1651.70807	-544.1780441	-1364.205051	EMD1
AGGGCTCCACGGTGCT-1	singlet	2	-526.7751949	-470.8560495	-254.1320601	EMD1
AGGGCTCCAGAGTGAC-1	singlet	1	-3901.467269	-956.5880184	-2876.943049	EMD1
AGGGCTCCAGGTTCGC-1	singlet	2	-548.0531851	-447.2708389	-193.8496027	EMD1
AGGGCTCCATCCGCGA-1	singlet	0	-109.0758254	-510.6608618	-429.7966993	EMD1
AGGGCTCCATGTTCAG-1	singlet	2	-602.6298973	-590.6929845	-281.5136769	EMD1
AGGGCTCGTGACCGAA-1	singlet	2	-923.3446848	-969.4742786	-287.3320835	EMD1
AGGGCTCTCGGAACTT-1	singlet	1	-7559.110505	-1852.700724	-6218.565667	EMD1
AGGGCTCTCGTGCGAC-1	singlet	2	-1291.514254	-1219.228908	-512.8493053	EMD1
AGGGCTCTCTCCAAGA-1	singlet	2	-122.1450188	-86.80858577	-46.57872709	EMD1
AGGGTCCAGGTCCTGC-1	singlet	1	-958.5938637	-336.1444553	-791.0601363	EMD1
AGGGTCCAGTAAGACT-1	singlet	0	-1229.789621	-6377.111454	-5388.368591	EMD1
AGGGTCCAGTTCCTGA-1	singlet	0	-125.5689683	-294.1002995	-312.1120742	EMD1
AGGGTCCCAACCGCCA-1	singlet	2	-837.3134366	-776.3150809	-337.6793011	EMD1
AGGGTCCCACGACTAT-1	singlet	2	-520.5172125	-469.3947108	-236.2992467	EMD1
AGGGTCCCACGTGTGC-1	singlet	2	-1595.992656	-1340.451057	-576.8842937	EMD1
AGGGTCCGTAGACGGT-1	singlet	0	-723.9794349	-2056.300082	-2051.438995	EMD1
AGGGTCCGTAGTCTGT-1	singlet	2	-462.7192405	-366.9217079	-177.1261378	EMD1
AGGGTCCGTCAACCAT-1	singlet	0	-724.8569985	-2213.612942	-2240.935099	EMD1
AGGGTCCGTTGCGAAG-1	singlet	2	-469.6664165	-369.9610988	-221.5283552	EMD1
AGGGTCCTCCAGGACC-1	singlet	0	-1035.724103	-3102.853367	-2984.912788	EMD1
AGGGTCCTCCTAGCGG-1	singlet	0	-69.89582726	-207.9572249	-209.5596845	EMD1
AGGGTCCTCGTTATCT-1	singlet	2	-768.6282923	-661.383472	-284.1999432	EMD1
AGGGTGAAGCCTCACG-1	singlet	2	-1064.549407	-1013.980938	-392.7529164	EMD1
AGGGTGAAGGTGCATG-1	singlet	0	-269.4760108	-805.2611374	-792.427608	EMD1
AGGGTGAAGTGGATTA-1	singlet	2	-314.2911453	-250.0583218	-85.92706822	EMD1
AGGGTGACAAGACCGA-1	singlet	2	-3921.226652	-2940.476464	-1196.391225	EMD1
AGGGTGACAAGTACCT-1	singlet	2	-1929.183712	-1822.737027	-1025.759104	EMD1
AGGGTGACACCGCTGA-1	singlet	2	-325.1423938	-259.1430353	-131.1054777	EMD1
AGGGTGACAGGGTCTC-1	singlet	0	-80.41318119	-244.829096	-223.5134186	EMD1
AGGGTGAGTTTCAGAC-1	singlet	2	-367.8054736	-413.8262199	-187.1637078	EMD1
AGGGTGAGTTTCCAAG-1	singlet	2	-390.2000976	-334.6731187	-152.3001312	EMD1
AGGGTGATCCTATGGA-1	singlet	2	-380.4907291	-272.7291297	-122.8277302	EMD1
AGGGTGATCGCCTTGT-1	singlet	2	-357.1598113	-347.6665052	-172.0405864	EMD1
AGGGTGATCTAGGAAA-1	singlet	0	-24.68627008	-143.6591703	-120.669892	EMD1
AGGGTGATCTTGGATG-1	singlet	1	-4465.861872	-1291.056829	-3577.309251	EMD1
AGGGTTTCACCTCTGT-1	singlet	1	-18.71194489	-7.09474424	-16.72086928	EMD1
AGGGTTTCAGGCCTGT-1	singlet	0	-1117.414352	-2732.351768	-2951.565287	EMD1
AGGGTTTGTCTACACA-1	singlet	2	-353.7316051	-321.8230859	-151.4769532	EMD1
AGGGTTTGTGGTCAAG-1	singlet	2	-723.0873178	-669.2778336	-254.2999125	EMD1
AGGGTTTGTTATCTGG-1	singlet	2	-1609.789085	-1652.383341	-609.426752	EMD1
AGGGTTTTCTCTATGT-1	singlet	2	-555.939531	-501.8211114	-231.965278	EMD1
AGGGTTTTCTTCGTGC-1	singlet	0	-11.11518773	-66.23285729	-49.40754466	EMD1
AGGTAGGAGTTCACTG-1	singlet	0	-647.5023401	-1793.092737	-1673.272882	EMD1
AGGTAGGCAAGTGGGT-1	singlet	2	-323.2679443	-326.9135444	-149.8062681	EMD1
AGGTAGGCAATTCACG-1	singlet	2	-513.0930594	-414.3346257	-239.5678863	EMD1
AGGTAGGCACAACGTT-1	singlet	0	-38.81242382	-125.7721621	-116.18118	EMD1
AGGTAGGCAGAGGCAT-1	singlet	2	-295.7555765	-300.8071927	-160.6525375	EMD1
AGGTAGGCATGTGCTA-1	singlet	0	-781.5911621	-2748.841947	-2554.845116	EMD1
AGGTAGGTCCGATCGG-1	doublet	0/1	-557.0247519	-726.3990511	-684.3331754	EMD1
AGGTCATAGAGAGGTA-1	singlet	1	-2445.605922	-846.7633321	-1737.812049	EMD1
AGGTCATCACCTGATA-1	singlet	2	-1163.38	-989.2300797	-417.3912941	EMD1
AGGTCATCATAGATGA-1	singlet	2	-498.6177964	-495.2386902	-227.2025702	EMD1
AGGTCATCATCTAACG-1	singlet	2	-613.2268075	-478.8531109	-225.4768443	EMD1
AGGTCATGTTCCGCAG-1	singlet	0	-515.5263543	-1757.208663	-1665.651899	EMD1
AGGTCATGTTGCGAAG-1	singlet	1	-5591.425766	-1236.254893	-3881.380404	EMD1
AGGTCATTCAACCTCC-1	singlet	0	-662.3097556	-2052.310084	-2075.449945	EMD1
AGGTCTAAGTTAACGA-1	singlet	2	-777.3271622	-580.325366	-303.6990613	EMD1
AGGTCTAGTCCAAGAG-1	singlet	0	-15.21421204	-64.189792	-49.52223203	EMD1
AGGTCTAGTCGCTCGA-1	singlet	2	-859.7472192	-741.7013623	-322.0072025	EMD1
AGGTCTAGTGAGCCAA-1	singlet	1	-994.8429241	-306.7525605	-838.2666987	EMD1
AGGTCTAGTTTACTTC-1	singlet	1	-1702.465404	-525.9459897	-1366.001972	EMD1
AGGTCTATCATTGCCC-1	singlet	0	-128.7972786	-335.5603062	-361.6575529	EMD1
AGGTCTATCCTTCTGG-1	singlet	1	-2914.470823	-917.3191725	-2269.667521	EMD1
AGGTCTATCGCGAAGA-1	singlet	0	-142.2397378	-403.43323	-414.2210256	EMD1
AGGTGTTCACGATTCA-1	singlet	1	-285.678748	-143.8362782	-229.2017884	EMD1
AGGTGTTCATGTCTAG-1	singlet	0	-602.055989	-2417.827983	-2214.093982	EMD1
AGGTGTTGTAGACTGG-1	singlet	0	-393.2346896	-1133.874396	-1089.545377	EMD1
AGGTGTTGTGGTCTTA-1	singlet	2	-514.8995278	-456.5963017	-222.8792579	EMD1
AGGTGTTTCGTTCAGA-1	doublet	45293	-7200.814061	-3608.284187	-4559.372304	EMD1
AGGTGTTTCTCGTGGG-1	singlet	2	-1042.334676	-924.6891361	-387.8421992	EMD1
AGGTTACAGCGCTTCG-1	singlet	1	-534.2496006	-223.8055415	-581.975374	EMD1
AGGTTACAGGCGAACT-1	singlet	2	-466.1742853	-362.1941336	-172.3364363	EMD1
AGGTTACAGTTACGTC-1	singlet	0	-34.56126203	-110.4902421	-76.79280674	EMD1
AGGTTACCACCATATG-1	singlet	0	-552.0210649	-1834.869839	-1653.065334	EMD1
AGGTTACCATGCGGTC-1	singlet	1	-3742.809808	-1202.91618	-3229.061551	EMD1
AGGTTACGTAGAGGAA-1	singlet	0	-357.3899898	-988.0940792	-880.387145	EMD1
AGGTTACGTCGAACAG-1	singlet	2	-2256.730757	-1846.828225	-794.0795079	EMD1
AGGTTACGTTCGGTTA-1	singlet	1	-2302.955304	-569.3612932	-1855.055337	EMD1
AGGTTACTCCAATCTT-1	singlet	2	-651.4866948	-491.4958091	-237.0718415	EMD1
AGGTTACTCCGGACTG-1	singlet	0	-1401.662232	-4016.309773	-3712.389271	EMD1
AGGTTACTCGGTCTGG-1	singlet	2	-70.16246596	-68.28263816	-29.49144437	EMD1
AGGTTACTCGGTGAAG-1	singlet	1	-2899.672453	-901.1968874	-2140.967168	EMD1
AGGTTACTCGTCGATA-1	singlet	2	-586.4349715	-479.615546	-258.872574	EMD1
AGGTTACTCTCGTGGG-1	singlet	2	-550.8685005	-499.6293064	-200.8830058	EMD1
AGGTTGTAGAGTTCGG-1	singlet	2	-614.7453702	-472.3359906	-274.3359668	EMD1
AGGTTGTAGCTGTACT-1	unassigned	0	-8.282251626	-11.64537072	-59.84239515	EMD1
AGGTTGTAGTATTGCC-1	singlet	1	-2848.889137	-991.0712145	-2411.43504	EMD1
AGGTTGTCACCTGTCT-1	singlet	2	-306.9823912	-257.5302827	-96.94286617	EMD1
AGGTTGTCACTTGGGC-1	singlet	0	-1038.324327	-3261.367657	-3290.060198	EMD1
AGGTTGTGTCATCACA-1	singlet	0	-123.7536451	-377.0659887	-398.3133989	EMD1
AGGTTGTTCCTTCGAC-1	singlet	0	-823.7306415	-2166.275518	-2222.22891	EMD1
AGGTTGTTCTTCACAT-1	doublet	0/2	-1123.796293	-1911.959178	-1670.919417	EMD1
AGTAACCAGCTAGAGC-1	singlet	0	-239.0170281	-907.8525373	-877.1502655	EMD1
AGTAACCAGTCAATCC-1	singlet	0	-41.10806832	-99.97134105	-97.28174327	EMD1
AGTAACCCACCTTCGT-1	singlet	0	-690.2999951	-1972.573197	-1866.927845	EMD1
AGTAACCGTGGGATTG-1	singlet	2	-441.8548757	-483.3545044	-221.9426883	EMD1
AGTAACCTCACTAGCA-1	singlet	1	-1418.65374	-530.2937002	-1150.082752	EMD1
AGTAACCTCCGTGACG-1	singlet	2	-574.1324508	-498.6499072	-217.8996355	EMD1
AGTAACCTCTGAATGC-1	singlet	0	-24.22389708	-52.3680483	-62.19090441	EMD1
AGTACCAAGCTGCGAA-1	singlet	1	-3939.934568	-1051.716105	-3217.349588	EMD1
AGTACCAAGGGCTGAT-1	singlet	1	-519.3254481	-187.8862904	-569.4938827	EMD1
AGTACCACACACACGC-1	singlet	2	-3784.728454	-2902.26991	-1211.791234	EMD1
AGTACCACACAGGATG-1	singlet	2	-1014.542673	-818.0445593	-358.748138	EMD1
AGTACCAGTACGTGAG-1	singlet	2	-1641.4194	-1583.628524	-587.1067446	EMD1

AGTACCAGTCCACATA-1	singlet	0	-37.16488619	-86.02794726	-118.662614	EMD1
AGTACCAGTCCCTAAA-1	singlet	2	-9437.235223	-7881.520889	-3014.691416	EMD1
AGTACCAGTGAATGTA-1	singlet	2	-305.6458585	-239.631712	-117.6243619	EMD1
AGTACCATCATGCTAG-1	singlet	2	-532.6313929	-472.9437683	-230.5469415	EMD1
AGTACCATCTCTTCAA-1	singlet	1	-2647.743694	-890.4625452	-2015.130456	EMD1
AGTACTGAGAGTAACT-1	doublet	1/0	-2586.210925	-2374.861164	-2952.66695	EMD1
AGTACTGAGCCTATTG-1	singlet	2	-461.8067723	-372.4469701	-204.4446487	EMD1
AGTACTGAGTCCGTCG-1	singlet	1	-2605.34412	-561.3356263	-2072.381949	EMD1
AGTACTGAGTTACGAA-1	singlet	0	-259.1215925	-769.7142974	-812.7408481	EMD1
AGTACTGCACACCGAC-1	singlet	1	-537.7082867	-233.1393967	-541.9683638	EMD1
AGTACTGCAGGGAGAG-1	singlet	1	-6970.442978	-1605.685685	-4752.083999	EMD1
AGTACTGCATCTTAGG-1	singlet	1	-905.5416813	-449.1888006	-768.4086834	EMD1
AGTACTGGTAGATTGA-1	singlet	2	-281.1833296	-259.7417239	-114.8179781	EMD1
AGTACTGGTATTTCGG-1	singlet	1	-1637.085793	-654.2872622	-1368.423502	EMD1
AGTACTGGTTAAGGGC-1	singlet	1	-2611.502048	-836.475735	-2216.59179	EMD1
AGTACTGTCCTAGCTC-1	singlet	2	-4045.825995	-3923.889555	-1170.231434	EMD1
AGTAGCTAGGTGGCTA-1	singlet	0	-346.9428492	-1123.420714	-1097.587194	EMD1
AGTAGCTCACCGGCTA-1	singlet	1	-1239.591601	-384.3692021	-842.298495	EMD1
AGTAGCTCACGACTAT-1	singlet	1	-487.2817124	-203.3009892	-488.3653215	EMD1
AGTAGCTCACTCTCGT-1	singlet	2	-1998.805135	-1797.840313	-668.2325253	EMD1
AGTAGCTGTATGCGGA-1	singlet	2	-385.281059	-296.4954538	-138.3441848	EMD1
AGTAGCTGTCTCCCTA-1	singlet	2	-463.668947	-401.2834812	-212.9186535	EMD1
AGTAGCTTCGAAACAA-1	singlet	1	-404.4442889	-168.0219131	-318.1307613	EMD1
AGTAGCTTCGCTACGG-1	singlet	0	-384.7948926	-1157.191149	-1179.60535	EMD1
AGTAGCTTCTGCGGGT-1	singlet	2	-473.2606857	-364.8848212	-193.7081802	EMD1
AGTAGTCAGATTGAGT-1	singlet	2	-818.2474661	-634.3485346	-301.6479453	EMD1
AGTAGTCAGCGTCTCG-1	singlet	2	-239.5721217	-222.3275793	-109.1658582	EMD1
AGTAGTCAGTAATACG-1	singlet	2	-228.2117963	-209.4517715	-108.7313548	EMD1
AGTAGTCCAACGGCCT-1	singlet	2	-543.3862945	-473.2935989	-211.2967122	EMD1
AGTAGTCCACTGAGTT-1	singlet	2	-3948.99998	-3355.735609	-1260.587056	EMD1
AGTAGTCCACTTTAGG-1	singlet	1	-3159.946975	-865.9100245	-2586.375519	EMD1
AGTAGTCTCAATCCAG-1	singlet	0	-373.1518426	-1236.13893	-1211.574074	EMD1
AGTAGTCTCTCCGTGT-1	singlet	2	-416.11122	-332.2311537	-142.1625971	EMD1
AGTCAACAGCCAGTAG-1	unassigned	1	-0.939830357	-0.935759779	-1.594400348	EMD1
AGTCAACAGCGCTGAA-1	singlet	1	-199.7771785	-51.76571168	-126.7349928	EMD1
AGTCAACAGGAGAATG-1	singlet	0	-383.0514855	-1566.524289	-1275.774183	EMD1
AGTCAACAGTTAGAAC-1	singlet	1	-1550.028602	-595.767905	-1239.494784	EMD1
AGTCAACCAAAGGATT-1	doublet	2/0	-1693.82628	-2385.845371	-1604.872031	EMD1
AGTCAACCAAATCGGG-1	singlet	2	-379.8824611	-335.8359629	-141.366592	EMD1
AGTCAACCACGACAAG-1	singlet	0	-645.3771216	-1665.53676	-1438.111079	EMD1
AGTCAACCACTTGAGT-1	singlet	2	-611.1926501	-557.8791651	-207.0229276	EMD1
AGTCAACGTCCGACGT-1	singlet	2	-1503.350979	-1281.518455	-583.0490703	EMD1
AGTCAACGTCGACGCT-1	singlet	2	-7159.801826	-5754.522752	-1760.671565	EMD1
AGTCAACGTCTTACAG-1	singlet	2	-53.25788878	-42.20009309	-17.71932509	EMD1
AGTCAACGTGTGGACA-1	singlet	2	-1245.809254	-1040.637314	-434.4711658	EMD1
AGTCAACGTTACCCTC-1	singlet	2	-595.3487738	-607.9326869	-251.0519402	EMD1
AGTCAACTCCGCATAA-1	singlet	1	-1882.027479	-583.51569	-1430.862888	EMD1
AGTCACAAGATCACTC-1	singlet	0	-158.1803873	-488.6887962	-495.6081892	EMD1
AGTCACAAGTTGCATC-1	singlet	0	-922.1964799	-2629.42563	-2445.257329	EMD1
AGTCACACAGCGCGTT-1	singlet	1	-462.5230625	-167.0201286	-374.4292477	EMD1
AGTCACACAGCTGCCA-1	singlet	0	-103.7625682	-377.6012137	-249.4113644	EMD1
AGTCACACATACTGAC-1	singlet	0	-1177.932018	-3746.174788	-3628.182679	EMD1
AGTCACACATGGAAGC-1	singlet	0	-983.4903124	-2777.957703	-2701.633213	EMD1
AGTCACATCTAGAACC-1	singlet	1	-896.7680867	-359.0320835	-765.3229127	EMD1
AGTCATGAGAACGTGC-1	singlet	2	-1111.116967	-1000.974847	-496.0730725	EMD1
AGTCATGAGACCGTTT-1	singlet	2	-608.6598419	-601.5493975	-224.9339118	EMD1
AGTCATGAGCTGGCTC-1	singlet	0	-220.055677	-987.7685175	-912.6407172	EMD1
AGTCATGAGTCAGGGT-1	singlet	1	-2890.223998	-768.8240665	-1896.208407	EMD1
AGTCATGCACGCGCAT-1	singlet	1	-430.056046	-160.9527861	-331.0982062	EMD1
AGTCATGCACTCATAG-1	singlet	0	-1433.527258	-6275.268034	-5634.42452	EMD1
AGTCATGCAGGATGAC-1	singlet	1	-382.8947499	-153.3172223	-422.1855477	EMD1
AGTCATGCATCCGAGC-1	singlet	1	-2549.279649	-507.0810317	-2076.510958	EMD1
AGTCATGGTGAATTGA-1	singlet	2	-398.6350581	-301.7081567	-138.3666313	EMD1
AGTCATGGTTCGGCCA-1	singlet	2	-294.8413497	-270.7181855	-157.4314718	EMD1
AGTCATGTCCATGCAA-1	singlet	2	-703.7919246	-541.0791154	-253.7763924	EMD1
AGTCATGTCGTCCTTG-1	singlet	2	-1057.709828	-997.8390463	-329.7125187	EMD1
AGTCTCCAGCTAAGTA-1	singlet	2	-4745.405513	-3895.388508	-1641.319433	EMD1
AGTCTCCAGCTAATCC-1	singlet	0	-316.7945976	-1186.14846	-1119.087818	EMD1
AGTCTCCAGCTGAAGC-1	singlet	2	-2938.284783	-2341.532723	-1085.327683	EMD1
AGTCTCCAGGAGAGTA-1	singlet	2	-291.4267166	-246.73033	-145.812905	EMD1
AGTCTCCGTACCGTCG-1	singlet	2	-606.760201	-486.0785756	-252.9202113	EMD1
AGTCTCCTCGAGATGG-1	singlet	1	-3948.70927	-1230.669297	-3419.045079	EMD1
AGTGACTAGCTCGGCT-1	singlet	0	-264.4247942	-937.3410533	-925.5673758	EMD1
AGTGACTAGGGACAGG-1	singlet	2	-2093.338617	-1963.987289	-794.222091	EMD1
AGTGACTCAAGCTCTA-1	singlet	2	-2622.873903	-2215.70489	-949.0105744	EMD1
AGTGACTCAAGGAGTC-1	singlet	2	-1284.200584	-995.3437602	-471.9392658	EMD1
AGTGACTTCCGAACGC-1	singlet	1	-3243.274467	-1041.276646	-2683.768933	EMD1
AGTGACTTCTATGTGG-1	singlet	0	-960.9611836	-2744.831218	-2399.475854	EMD1
AGTGACTTCTGCTCTG-1	singlet	2	-484.0830855	-441.6444862	-217.4020639	EMD1
AGTGATCAGCTGAAGC-1	singlet	2	-539.0822944	-486.726638	-197.3168295	EMD1
AGTGATCAGTATGAAC-1	singlet	1	-365.1113985	-154.5130118	-325.2914066	EMD1
AGTGATCCAAGGCTTT-1	singlet	0	-1424.996819	-4545.376188	-4849.025321	EMD1
AGTGATCCACACGCCA-1	singlet	0	-486.8066764	-1777.59602	-1485.443195	EMD1
AGTGATCCACCAGACC-1	singlet	0	-605.4636387	-1938.771666	-1814.285604	EMD1
AGTGATCCACCCTTGT-1	singlet	2	-339.2079253	-268.8338736	-148.0537433	EMD1
AGTGATCCACGAGGTA-1	doublet	2/0	-289.7352041	-363.3989753	-231.3539753	EMD1
AGTGATCGTCCGGACT-1	singlet	0	-189.4830737	-514.2315605	-574.4916303	EMD1
AGTGATCGTGCCAAGA-1	singlet	1	-154.369364	-43.07199125	-94.23720342	EMD1
AGTGATCGTTTCACAG-1	singlet	1	-6691.163282	-2037.316241	-5274.775756	EMD1
AGTGATCTCCGCTAGG-1	singlet	2	-267.1797415	-254.5308082	-121.1403472	EMD1
AGTGATCTCCGGCAAC-1	singlet	0	-80.59204009	-213.8761245	-195.1973293	EMD1
AGTGATCTCGCAATTG-1	singlet	1	-528.8315359	-232.0048964	-592.5472726	EMD1
AGTGATCTCGTTCCCA-1	singlet	2	-268.5720493	-149.6397226	-82.31011715	EMD1
AGTGATCTCTCTGCTG-1	singlet	2	-2589.68472	-2004.793829	-786.0710422	EMD1
AGTGCCGAGACAACAT-1	singlet	2	-6405.011572	-5191.604	-2051.584097	EMD1
AGTGCCGAGTTGTACC-1	singlet	1	-3741.858703	-842.8861032	-3246.59845	EMD1
AGTGCCGCATATGCGT-1	singlet	0	-1438.626367	-4370.688998	-4407.192284	EMD1
AGTGCCGGTACAGGTG-1	singlet	1	-710.2604618	-286.7515256	-518.5889716	EMD1
AGTGCCGGTACCTAAC-1	singlet	2	-466.033831	-379.9397781	-191.0070476	EMD1
AGTGCCGGTACTCGTA-1	singlet	2	-553.4382414	-476.3242536	-239.289145	EMD1
AGTGCCGGTCACTGAT-1	singlet	1	-246.6699711	-77.72273081	-161.7998362	EMD1
AGTGCCGGTGGATACG-1	singlet	2	-374.6397017	-327.9550591	-135.571059	EMD1
AGTGCCGTCCCATTTA-1	singlet	2	-1108.98824	-940.8717758	-409.9672657	EMD1
AGTGTTGAGAGAACCC-1	singlet	1	-87.48716587	-33.32589093	-55.20775879	EMD1
AGTGTTGAGCGACTGA-1	singlet	2	-539.1513673	-447.2916133	-225.1383378	EMD1
AGTGTTGAGGAGAGTA-1	singlet	1	-4190.941529	-1302.849537	-3524.249623	EMD1
AGTGTTGAGGCAATGC-1	singlet	2	-634.5907949	-472.2124209	-250.3047358	EMD1
AGTGTTGAGGCCATAG-1	singlet	0	-311.1010021	-968.2949392	-979.2572038	EMD1
AGTGTTGAGTACTGGG-1	singlet	1	-908.0985998	-354.3651334	-724.556623	EMD1
AGTGTTGCAATACAGA-1	singlet	0	-936.0057447	-3621.909702	-3568.601849	EMD1

AGTGTTGCACATACTG-1	singlet	2	-390.849276	-283.5030248	-143.3491484	EMD1
AGTGTTGCATAATCCG-1	singlet	1	-830.7637772	-301.4655434	-704.9261099	EMD1
AGTGTTGTCCACGTGG-1	singlet	1	-2975.782176	-1091.32438	-2490.430091	EMD1
AGTGTTGTCGACGACC-1	singlet	2	-333.0985163	-288.8336991	-133.1767884	EMD1
AGTGTTGTCGTGCAGC-1	singlet	2	-553.9279021	-475.4052911	-211.2162838	EMD1
AGTTAGCAGCTGAGTG-1	singlet	2	-214.1852046	-190.4909306	-95.9491248	EMD1
AGTTAGCAGGGAGTGG-1	singlet	2	-150.952817	-128.4141566	-41.46809994	EMD1
AGTTAGCAGGGTTAAT-1	singlet	2	-307.5127718	-248.6261536	-126.6400058	EMD1
AGTTAGCCATCTATCT-1	singlet	0	-1603.033807	-4662.101726	-4595.677738	EMD1
AGTTAGCCATTCTTCA-1	singlet	2	-624.2915174	-548.5161228	-239.1608989	EMD1
AGTTAGCGTACAAAGT-1	singlet	0	-1348.656646	-3946.142036	-3973.828923	EMD1
AGTTAGCGTATGGGAC-1	singlet	2	-360.3035208	-351.2752764	-148.3617425	EMD1
AGTTAGCTCAGATGCT-1	singlet	0	-1509.295525	-5692.330868	-5081.851056	EMD1
AGTTAGCTCATATGGC-1	singlet	2	-249.3862759	-209.275379	-99.19739514	EMD1
AGTTAGCTCGACCAAT-1	doublet	45323	-634.351129	-513.7195276	-324.873796	EMD1
AGTTCCCAGCCTCTTC-1	singlet	0	-659.9420128	-1834.068872	-1760.912681	EMD1
AGTTCCCCAACGACAG-1	singlet	2	-339.4034807	-303.5964958	-154.4072546	EMD1
AGTTCCCCATTAAGCC-1	singlet	0	-1019.125671	-3833.33745	-3543.809256	EMD1
AGTTCCCGTTTGGGTT-1	singlet	2	-332.8054396	-295.5141212	-141.250269	EMD1
AGTTCCCTCACCTCTG-1	singlet	2	-2713.876568	-2235.358553	-909.7109855	EMD1
AGTTCCCTCGACGACC-1	singlet	0	-462.622724	-1380.698132	-1391.025985	EMD1
AGTTCGAAGATAGGGA-1	singlet	2	-678.5127796	-551.9058517	-253.7608147	EMD1
AGTTCGAAGCCTAACT-1	singlet	0	-1063.843119	-3678.663792	-3629.299141	EMD1
AGTTCGAAGGGTGAAA-1	singlet	2	-436.9258615	-333.4820243	-164.8352224	EMD1
AGTTCGAAGTGCACTT-1	singlet	2	-352.4504951	-319.4981554	-167.9093601	EMD1
AGTTCGACAGCTTCCT-1	singlet	0	-94.5588757	-245.4558313	-231.0629389	EMD1
AGTTCGAGTAACGCGA-1	singlet	2	-490.2747761	-390.2153098	-206.3721908	EMD1
AGTTCGAGTGGGATTG-1	singlet	0	-428.9903007	-1219.566013	-1128.615229	EMD1
AGTTCGAGTTACGTAC-1	singlet	0	-682.8560721	-2597.104418	-2616.952087	EMD1
AGTTCGATCAAGTGTC-1	singlet	2	-1461.380865	-1348.892909	-517.9681879	EMD1
AGTTCGATCCGAGAAG-1	singlet	0	-576.7689934	-1492.504014	-1559.085397	EMD1
ATACCGAAGAAATTGC-1	singlet	1	-2943.215734	-1075.703538	-2498.603409	EMD1
ATACCGAAGCAGCCTC-1	singlet	2	-1021.882489	-855.7117277	-348.7740202	EMD1
ATACCGAAGGTCATAA-1	singlet	2	-550.9848598	-540.0422104	-221.2145644	EMD1
ATACCGAGTTCAAGGG-1	singlet	2	-384.4076267	-356.6913913	-160.5863179	EMD1
ATACCGAGTTTACCAG-1	singlet	0	-261.5752261	-604.2262327	-776.878483	EMD1
ATACCGATCCACACCT-1	singlet	2	-741.7777999	-617.6936661	-292.309402	EMD1
ATACCGATCCTCGCAT-1	singlet	1	-357.5959774	-90.59015083	-225.5944271	EMD1
ATACCGATCGCGGTAC-1	doublet	45293	-1995.585073	-963.350148	-1313.584081	EMD1
ATACCGATCTAGCAAC-1	singlet	0	-521.764208	-1596.150424	-1454.257605	EMD1
ATACCTTAGACTCTAC-1	singlet	0	-23.66742467	-76.82833126	-91.426237	EMD1
ATACCTTAGAGCCGTA-1	singlet	0	-59.40270285	-180.1103519	-163.7211385	EMD1
ATACCTTAGAGTCCGA-1	singlet	0	-608.6391331	-1671.004791	-1505.13865	EMD1
ATACCTTAGTCACAGG-1	singlet	2	-482.4202932	-411.7357397	-189.8170675	EMD1
ATACCTTAGTTGCCTA-1	singlet	2	-1152.114813	-1032.340359	-410.2979404	EMD1
ATACCTTCAAGTATAG-1	singlet	2	-502.0921699	-372.6582388	-172.7810059	EMD1
ATACCTTCATAGGTAA-1	singlet	2	-443.9423614	-373.8175782	-181.8120836	EMD1
ATACCTTGTAGTATAG-1	singlet	2	-1287.821867	-982.6553316	-418.346529	EMD1
ATACCTTGTCAGATTC-1	singlet	0	-1276.9505	-4224.835888	-3953.521113	EMD1
ATACCTTGTCCATAGT-1	singlet	2	-485.0693655	-398.0726941	-169.9042932	EMD1
ATACCTTGTGTCATTG-1	singlet	0	-160.981422	-469.4113705	-574.8018329	EMD1
ATACCTTGTTCGAGCC-1	singlet	1	-3005.616076	-1064.988597	-2307.649605	EMD1
ATACTTCAGATACTGA-1	singlet	1	-196.101801	-83.28680197	-162.4928204	EMD1
ATACTTCCAAGCGCAA-1	singlet	0	-301.3284134	-948.4628144	-919.417907	EMD1
ATACTTCGTCAAGCCC-1	singlet	0	-1968.774397	-6295.710654	-6317.608088	EMD1
ATACTTCGTGAACGGT-1	singlet	1	-509.1255138	-215.1246211	-578.6674795	EMD1
ATACTTCTCCAAGCAT-1	singlet	1	-2965.434643	-978.3729372	-2224.113202	EMD1
ATACTTCTCGAAACAA-1	singlet	2	-483.2309251	-438.5286423	-246.407472	EMD1
ATACTTCTCGATCCCT-1	singlet	1	-438.3141637	-155.4170389	-458.3526728	EMD1
ATAGACCAGAAGATCT-1	singlet	2	-717.9366437	-592.9091702	-263.8040979	EMD1
ATAGACCAGACGAGCT-1	singlet	0	-315.8608927	-1108.771454	-953.0765783	EMD1
ATAGACCAGGCGATAC-1	singlet	2	-530.1074188	-440.4907966	-205.2665552	EMD1
ATAGACCCACATAACC-1	singlet	2	-546.6539069	-524.9066797	-237.1964305	EMD1
ATAGACCCAGACATCT-1	singlet	0	-668.9702285	-2714.751185	-2296.456158	EMD1
ATAGACCCAGGAATCG-1	singlet	1	-2477.701356	-864.6055362	-1946.832585	EMD1
ATAGACCTCAACGAGG-1	singlet	1	-521.9123933	-252.8883697	-580.9095069	EMD1
ATAGACCTCAGTCACA-1	singlet	2	-163.6813297	-170.4185541	-48.81290167	EMD1
ATAGACCTCTACGCAA-1	singlet	0	-354.6238066	-997.1969942	-961.9831797	EMD1
ATAGACCTCTAGCCTC-1	singlet	1	-2539.796823	-807.3763709	-1898.567203	EMD1
ATAGACCTCTCGTGAA-1	singlet	2	-289.0137146	-231.4842565	-112.375566	EMD1
ATAGAGAAGGTAATCA-1	singlet	0	-355.9020134	-982.4830228	-828.6299393	EMD1
ATAGAGAAGGTTGGTG-1	singlet	1	-88.5337636	-42.61956654	-102.2824663	EMD1
ATAGAGAGTACTGTTG-1	singlet	2	-657.4954288	-508.5415308	-229.080673	EMD1
ATAGAGAGTAGGTACG-1	singlet	0	-484.6377588	-1216.683247	-1162.088672	EMD1
ATAGAGATCATCACTT-1	singlet	1	-5887.395769	-1301.436956	-4024.215415	EMD1
ATAGAGATCTATACGG-1	doublet	0/1	-1376.57112	-1397.471762	-1705.889347	EMD1
ATAGAGATCTGCCTCA-1	singlet	1	-3828.415667	-1181.100656	-2918.725829	EMD1
ATAGGCTCAAATCAAG-1	singlet	0	-556.9942928	-1407.739879	-1360.03751	EMD1
ATAGGCTCAGCAGTTT-1	singlet	1	-4401.138303	-1179.221962	-3477.406887	EMD1
ATAGGCTTCGAACACT-1	singlet	2	-248.3464864	-157.621012	-67.45155667	EMD1
ATAGGCTTCTGGCCTT-1	singlet	1	-4726.694088	-1071.029572	-4158.022194	EMD1
ATAGGCTTCTTTGGAG-1	singlet	0	-196.824202	-542.6587403	-488.9109297	EMD1
ATATCCTAGACATATG-1	singlet	2	-170.2633135	-160.6104747	-81.82777944	EMD1
ATATCCTAGGCATGCA-1	singlet	0	-377.589181	-1417.973899	-1199.017928	EMD1
ATATCCTAGGTCGCCT-1	singlet	1	-670.3094609	-256.8669398	-558.7458527	EMD1
ATATCCTCAATCTAGC-1	singlet	0	-1072.528012	-3603.545164	-3342.788732	EMD1
ATATCCTCAGCGTGCT-1	singlet	2	-1218.609052	-1095.040946	-414.3787243	EMD1
ATATCCTCAGTCCCGA-1	singlet	2	-5560.032803	-4359.767143	-1623.964673	EMD1
ATATCCTGTAAGACCG-1	doublet	2/0	-624.0654077	-835.2899654	-592.081979	EMD1
ATATCCTGTAGGCTCC-1	doublet	2/0	-394.031539	-384.5799202	-250.381785	EMD1
ATATCCTGTCATGCAT-1	singlet	2	-526.6761889	-419.0639349	-217.5310379	EMD1
ATATCCTGTCCAACGC-1	singlet	0	-383.8364475	-1450.715559	-1208.024222	EMD1
ATATCCTTCCAACCAA-1	singlet	1	-181.3333664	-73.34802769	-148.215758	EMD1
ATATCCTTCTCCCAAC-1	singlet	2	-773.3528128	-656.5855627	-285.599567	EMD1
ATCACAGAGAAAGCGA-1	singlet	2	-577.4493308	-486.0098874	-198.9822427	EMD1
ATCACAGAGCGTTACT-1	singlet	1	-1174.422469	-433.8894013	-942.8147395	EMD1
ATCACAGCATGACTAC-1	singlet	0	-2846.301327	-10093.65228	-9511.367358	EMD1
ATCACAGGTCTTGCGG-1	singlet	0	-188.9259822	-582.7824011	-511.3050576	EMD1
ATCACAGGTGAACTAA-1	singlet	2	-2983.914613	-2706.216105	-1216.234517	EMD1
ATCACAGGTGTTACTG-1	doublet	1/0	-15.15653542	-13.49748219	-13.92421022	EMD1
ATCACAGGTTGAATCC-1	singlet	2	-478.6158117	-376.2202276	-195.2901543	EMD1
ATCACAGTCACTGGTA-1	singlet	1	-3130.721837	-1018.041181	-2348.351631	EMD1
ATCACAGTCACTTGGA-1	singlet	2	-455.3827601	-360.1283912	-193.3119262	EMD1
ATCACAGTCCGGCAAC-1	singlet	0	-1955.409371	-5802.365921	-5893.960812	EMD1
ATCACAGTCTTGGGCG-1	singlet	2	-360.5843314	-349.1178891	-137.1531652	EMD1
ATCACGAAGGATATAC-1	singlet	0	-720.2434501	-2119.424003	-2016.318161	EMD1
ATCACGAAGTCGGGAT-1	singlet	2	-3066.923416	-2431.502322	-1031.344833	EMD1
ATCACGACACAATCTG-1	singlet	0	-50.84109766	-229.0307445	-199.4798012	EMD1
ATCACGAGTGAGATTA-1	singlet	0	-1625.134474	-4930.170832	-4536.26244	EMD1
ATCACGATCAGTGTCA-1	singlet	0	-558.5217638	-2132.613701	-1815.478867	EMD1

ATCACTTAGAATTCAG-1	singlet	2	-118.1669442	-79.22594947	-27.72743272	EMD1
ATCACTTCAACTCATG-1	singlet	1	-3792.900075	-1312.809963	-3077.739205	EMD1
ATCACTTCAACTGAAA-1	singlet	1	-4946.184394	-1683.259952	-3951.059631	EMD1
ATCACTTCACCCTGTT-1	singlet	1	-4130.821964	-1256.908905	-3183.717686	EMD1
ATCACTTCACTTTATC-1	singlet	1	-3140.482268	-1113.923571	-2523.111754	EMD1
ATCACTTGTCAGTTTG-1	unassigned	2	-5.189807938	-1.304542899	-0.904771975	EMD1
ATCACTTGTCGTCAGC-1	singlet	2	-490.0962875	-457.2893811	-193.4009058	EMD1
ATCACTTGTTCGTTCC-1	singlet	2	-416.7325057	-371.9740621	-147.4103124	EMD1
ATCACTTGTTGCTGAT-1	singlet	2	-742.4629067	-633.0009011	-257.6837264	EMD1
ATCACTTTCAGGGTAG-1	singlet	2	-491.6364246	-416.3924247	-230.4734228	EMD1
ATCACTTTCGGCACTG-1	singlet	2	-691.997901	-571.7625961	-271.6034985	EMD1
ATCAGGTAGATTAGTG-1	singlet	2	-551.3367057	-526.9805866	-253.0731769	EMD1
ATCAGGTAGCCTCCAG-1	singlet	2	-443.495336	-381.6834256	-178.3089774	EMD1
ATCAGGTAGCGGATCA-1	singlet	1	-1096.972889	-374.4156381	-877.3153981	EMD1
ATCAGGTAGTATTAGG-1	singlet	0	-724.408873	-2331.23825	-2240.510625	EMD1
ATCAGGTCAACAAGTA-1	singlet	0	-1075.441126	-3488.041129	-3480.044551	EMD1
ATCAGGTCACTTGGCG-1	singlet	2	-2121.365013	-1826.660377	-766.0433083	EMD1
ATCAGGTCATCTCAAG-1	singlet	0	-292.4316378	-886.5281273	-855.3843949	EMD1
ATCAGGTTCAGAGTGG-1	singlet	0	-392.7348075	-1331.189913	-1258.469937	EMD1
ATCAGGTTCTCCTGCA-1	singlet	0	-1203.49886	-4276.862218	-3882.876375	EMD1
ATCATTCAGACGCAGT-1	singlet	1	-1654.092714	-601.0263883	-1369.611396	EMD1
ATCATTCCAACACACT-1	singlet	0	-356.6581587	-1091.977897	-1037.843092	EMD1
ATCATTCCACTGAGGA-1	singlet	1	-294.3362693	-82.75490788	-238.4064297	EMD1
ATCATTCCAGGTGGAT-1	singlet	2	-72.56241744	-59.12038784	-18.08346656	EMD1
ATCATTCGTCTGTCCT-1	singlet	2	-407.5803643	-321.3159828	-185.5587739	EMD1
ATCATTCGTGCAACAG-1	singlet	2	-333.8620972	-317.6694755	-148.7928883	EMD1
ATCATTCTCAATGTCG-1	singlet	2	-709.2637098	-561.0907762	-264.786462	EMD1
ATCATTCTCATGGCCG-1	singlet	0	-482.8611008	-1617.047684	-1612.076568	EMD1
ATCATTCTCCGTTGGG-1	singlet	0	-795.4552465	-2189.155855	-2180.183312	EMD1
ATCATTCTCCTGCTAC-1	singlet	0	-1368.555574	-3936.488665	-4000.402977	EMD1
ATCATTCTCGCCTATC-1	singlet	2	-1403.666478	-1292.954051	-563.11221	EMD1
ATCATTCTCGGCGATC-1	singlet	2	-443.0802583	-389.0659567	-202.002455	EMD1
ATCATTCTCGGTCGAC-1	singlet	1	-2833.078936	-1009.001958	-2483.708421	EMD1
ATCATTCTCTACTGCC-1	singlet	2	-4513.522161	-3566.742606	-1367.159879	EMD1
ATCATTCTCTCGCGTT-1	singlet	2	-590.5153543	-533.4039224	-237.3701538	EMD1
ATCATTCTCTTAAGGC-1	singlet	1	-621.908667	-201.8770881	-593.38173	EMD1
ATCCACCAGATAGTGT-1	singlet	0	-223.7928858	-580.4170027	-639.4169592	EMD1
ATCCACCAGGCACTCC-1	singlet	1	-1247.635723	-512.3722886	-1052.412702	EMD1
ATCCACCAGTCGTTAC-1	singlet	1	-60.51710729	-23.61378377	-34.69534427	EMD1
ATCCACCAGTGTACCT-1	singlet	2	-378.2337521	-350.4647159	-151.3582849	EMD1
ATCCACCCACATCATG-1	singlet	2	-555.1540472	-481.389596	-213.5894253	EMD1
ATCCACCCACGACGTC-1	singlet	2	-661.5830405	-568.664508	-268.2866654	EMD1
ATCCACCCAGCCCAGT-1	singlet	0	-612.4190678	-1904.588051	-1754.241314	EMD1
ATCCACCCAGGTGTTT-1	singlet	0	-620.3899773	-1785.476063	-1642.529765	EMD1
ATCCACCGTTCTCTAT-1	singlet	2	-481.3513187	-425.2290059	-187.7542686	EMD1
ATCCACCTCACAGAGG-1	singlet	1	-704.9054625	-243.4941189	-592.9293901	EMD1
ATCCATTAGTAAAGCT-1	singlet	2	-697.8000073	-663.5367319	-279.6509239	EMD1
ATCCATTCACCTCGTT-1	singlet	2	-440.3817959	-401.424337	-183.7620108	EMD1
ATCCATTCAGAGGACT-1	singlet	2	-942.7138895	-940.5358603	-352.0901795	EMD1
ATCCATTCAGCAGACA-1	singlet	2	-428.0572685	-415.9745782	-193.8405219	EMD1
ATCCATTCAGCGGTTC-1	singlet	2	-511.5169106	-406.1196221	-181.2949646	EMD1
ATCCATTGTCTAACTG-1	singlet	2	-353.4039438	-362.7806391	-161.4534567	EMD1
ATCCATTGTTCGGCTG-1	singlet	0	-399.7435999	-1220.532517	-1173.017384	EMD1
ATCCATTGTTCTATCT-1	singlet	1	-1284.328976	-480.2797292	-1079.211493	EMD1
ATCCCTGAGAGTAACT-1	singlet	1	-273.2718451	-126.7912836	-242.1243628	EMD1
ATCCCTGAGATGTTGA-1	singlet	0	-295.7133815	-960.6351036	-807.2675037	EMD1
ATCCCTGAGGTCGCCT-1	singlet	2	-751.0135755	-685.3614587	-278.0802791	EMD1
ATCCCTGAGGTGCCAA-1	singlet	1	-3191.77013	-973.3801257	-2531.411631	EMD1
ATCCCTGCAGAACGCA-1	singlet	2	-1758.701629	-1431.699906	-643.2648593	EMD1
ATCCCTGCAGACTCTA-1	singlet	2	-865.9351671	-816.9228596	-314.4077923	EMD1
ATCCCTGCAGTATTCG-1	singlet	0	-344.4166987	-830.4084597	-942.82032	EMD1
ATCCCTGGTTAACCTG-1	singlet	2	-1544.283145	-1494.154597	-621.2487535	EMD1
ATCCCTGGTTGGAGAC-1	singlet	2	-589.0324001	-564.5680146	-241.3087726	EMD1
ATCCCTGTCGCTTACC-1	singlet	0	-916.6491002	-2646.153709	-2603.942094	EMD1
ATCCCTGTCTGACAGT-1	singlet	0	-1079.341825	-3826.911325	-3312.286963	EMD1
ATCCGTCAGGAGAGTA-1	singlet	0	-416.4511852	-1392.829231	-1291.342998	EMD1
ATCCGTCAGGCTCACC-1	singlet	2	-528.0666433	-433.1360276	-185.5629546	EMD1
ATCCGTCCAAGGACAC-1	singlet	0	-721.4313996	-2100.899677	-2150.197121	EMD1
ATCCGTCCACATGACT-1	singlet	2	-368.7300847	-236.9128832	-139.9383557	EMD1
ATCCGTCCACCAACAT-1	singlet	0	-879.7758303	-2576.16237	-2399.857061	EMD1
ATCCGTCCATGATCTG-1	singlet	2	-790.7113782	-626.8534631	-313.0470529	EMD1
ATCCGTCCATTATGCG-1	singlet	0	-610.3888882	-2507.690865	-2305.297448	EMD1
ATCCGTCTCATACGAC-1	singlet	2	-556.1931239	-420.1030365	-218.6800703	EMD1
ATCCGTCTCCGTGGTG-1	singlet	2	-259.5673896	-247.9148935	-138.6577351	EMD1
ATCCGTCTCGCTGTTC-1	singlet	2	-741.02528	-595.6444979	-263.1448533	EMD1
ATCCTATAGTGGTGGT-1	singlet	2	-889.7581139	-807.9316752	-352.4798823	EMD1
ATCCTATCACGACGTC-1	singlet	2	-395.6451527	-415.3464737	-205.6222182	EMD1
ATCCTATCACTAGTAC-1	singlet	2	-944.529467	-835.2711182	-391.6171116	EMD1
ATCCTATCAGAAGTTA-1	singlet	2	-459.5454439	-347.9683215	-179.5124231	EMD1
ATCCTATTCTCCTGAC-1	singlet	2	-699.6945402	-560.506422	-258.2134432	EMD1
ATCGATGAGACCAGAC-1	doublet	45293	-876.62657	-551.4672418	-566.6117671	EMD1
ATCGATGAGACTGTTC-1	singlet	2	-1224.862186	-968.3709005	-473.1664477	EMD1
ATCGATGAGCCTAACT-1	singlet	2	-1692.56619	-1432.991634	-605.5539564	EMD1
ATCGATGGTACTAAGA-1	singlet	1	-11.4098879	-5.00748282	-15.61537325	EMD1
ATCGATGGTAGTTACC-1	singlet	2	-1361.974541	-1101.839777	-488.7571705	EMD1
ATCGATGGTATGGAGC-1	singlet	2	-543.1122777	-455.2941966	-215.0197662	EMD1
ATCGATGGTGGTCTCG-1	singlet	2	-289.5966653	-216.1845533	-44.15377319	EMD1
ATCGCCTAGCCTCTCT-1	singlet	0	-252.0275472	-727.7986876	-709.0763898	EMD1
ATCGCCTAGTACAACA-1	singlet	2	-662.0596016	-620.6345016	-284.8816479	EMD1
ATCGCCTGTAACTTCG-1	singlet	0	-210.5334468	-582.4651273	-599.4707144	EMD1
ATCGCCTGTAGCACAG-1	singlet	2	-537.4638384	-545.3910906	-227.7974688	EMD1
ATCGCCTGTAGCTTAC-1	singlet	1	-5510.979706	-1784.717031	-4245.512495	EMD1
ATCGCCTGTTCGTAAC-1	singlet	0	-696.93364	-2132.221357	-2134.544956	EMD1
ATCGCCTGTTCTTGCC-1	singlet	0	-333.4633059	-880.5778043	-861.7852437	EMD1
ATCGCCTGTTGGTAGG-1	singlet	0	-730.7130669	-2289.208964	-2322.124989	EMD1
ATCGCCTTCCGTGGTG-1	singlet	1	-1442.801018	-576.6259685	-1476.791177	EMD1
ATCGGATAGCATCTTG-1	singlet	0	-452.654101	-1328.855157	-1301.7773	EMD1
ATCGGATCACTGGATT-1	singlet	2	-3601.913691	-3323.830949	-1201.825955	EMD1
ATCGGATGTCCCACGA-1	singlet	2	-1695.645517	-1368.923179	-615.9949109	EMD1
ATCGGATGTCCGAAGA-1	singlet	1	-2114.18625	-696.7013869	-1773.042208	EMD1
ATCGGATGTCCGGCAT-1	singlet	0	-654.3699709	-2304.147619	-2277.433818	EMD1
ATCGGATTCTGAGAAA-1	singlet	1	-4058.388002	-911.6156246	-3225.457176	EMD1
ATCGGCGAGGACTTCT-1	singlet	2	-488.5615227	-353.4172154	-181.6463172	EMD1
ATCGGCGAGTCGCGAA-1	singlet	0	-663.0686772	-2231.72584	-2158.587519	EMD1
ATCGGCGCAAGAGTTA-1	singlet	2	-72.56004858	-46.36623025	-25.82055302	EMD1
ATCGGCGCAAGCTACT-1	singlet	2	-305.4561178	-306.6264008	-160.1523916	EMD1
ATCGGCGCACTACGGC-1	singlet	1	-1977.201155	-426.3561308	-1635.079319	EMD1
ATCGGCGCAGCGTATT-1	singlet	2	-1570.116946	-1151.611046	-520.4870576	EMD1
ATCGGCGCAGCTTCCT-1	singlet	2	-4208.172016	-3996.775407	-1285.567398	EMD1
ATCGGCGCATGACAGG-1	singlet	2	-1624.88541	-1301.105743	-591.266235	EMD1

ATCGGCGGTGTAGGAC-1	singlet	0	-168.7768804	-538.3267583	-487.271321	EMD1
ATCGGCGGTGTCGATT-1	singlet	2	-1125.497959	-881.2490215	-349.010151	EMD1
ATCGGCGTCGAACTCA-1	singlet	2	-1139.778835	-585.1148916	-247.920687	EMD1
ATCGGCGTCGGAGCAA-1	doublet	0/1	-2269.123347	-2697.790587	-3218.438876	EMD1
ATCGGCGTCTGGTCAA-1	singlet	2	-1018.988326	-932.8819333	-408.4399782	EMD1
ATCGTAGAGAGCTGCA-1	singlet	2	-1272.358227	-1057.62512	-426.0024676	EMD1
ATCGTAGAGGTATTGA-1	singlet	2	-2105.771986	-1616.965021	-836.3947336	EMD1
ATCGTAGCAGACTCTA-1	singlet	1	-441.1419122	-201.0263698	-531.0721247	EMD1
ATCGTAGCAGCGACCT-1	singlet	2	-2021.600635	-1713.757801	-799.0090046	EMD1
ATCGTAGCAGCTCATA-1	singlet	1	-2760.486024	-981.8088911	-2209.151143	EMD1
ATCGTAGGTTACCGTA-1	singlet	2	-1404.197789	-1145.193349	-509.5931699	EMD1
ATCGTAGTCAAGTCTG-1	singlet	2	-614.1136176	-465.2253769	-230.8839562	EMD1
ATCGTAGTCCACAGGC-1	singlet	2	-1039.003211	-939.4218894	-411.0384849	EMD1
ATCGTCCAGCAACTTC-1	singlet	0	-691.506627	-1941.300122	-1874.365333	EMD1
ATCGTCCAGCCAAGTG-1	singlet	1	-1212.299984	-390.8480368	-931.2208607	EMD1
ATCGTCCAGGTTGTTC-1	singlet	0	-121.7912581	-255.1064746	-281.6797178	EMD1
ATCGTCCCAGAACCGA-1	singlet	2	-648.8816808	-551.8845567	-225.8657643	EMD1
ATCGTCCGTATCTCTT-1	singlet	2	-1405.70427	-1223.513818	-535.9976765	EMD1
ATCGTCCGTCATATGC-1	singlet	0	-1625.741574	-4577.39841	-4512.34074	EMD1
ATCGTCCGTCCTGAAT-1	singlet	0	-2064.393044	-6336.947703	-6556.301218	EMD1
ATCGTCCGTGACAACG-1	singlet	0	-760.2187985	-2341.322482	-2232.365746	EMD1
ATCGTCCTCAAGAAAC-1	singlet	2	-460.9755925	-477.9320126	-193.298938	EMD1
ATCGTCCTCGACGCTG-1	singlet	0	-314.8713993	-1091.513687	-1254.109225	EMD1
ATCGTCCTCGTTCTGC-1	singlet	1	-2835.177093	-615.6307178	-2198.880536	EMD1
ATCGTCCTCTGGAGAG-1	singlet	1	-4444.443011	-1206.181441	-3145.844496	EMD1
ATCGTCCTCTTTGCGC-1	singlet	1	-308.5840148	-104.4875374	-268.8405873	EMD1
ATCGTGAAGGGTATAT-1	singlet	0	-247.3046174	-667.2723698	-621.8899678	EMD1
ATCGTGACAAGGGCAT-1	singlet	1	-1894.971414	-471.3192825	-1738.665952	EMD1
ATCGTGACAGCCCACA-1	singlet	2	-1602.828596	-1606.672041	-485.7955904	EMD1
ATCGTGACAGCGTGCT-1	singlet	1	-82.85564795	-28.70227892	-52.32392372	EMD1
ATCGTGACATGACGGA-1	singlet	2	-1571.273445	-1363.702073	-580.2793681	EMD1
ATCGTGAGTAGCTGTT-1	singlet	2	-469.3479532	-431.849334	-175.9470194	EMD1
ATCGTGAGTCACGACC-1	singlet	0	-512.6726775	-1472.454294	-1581.84048	EMD1
ATCGTGAGTTCAGTAC-1	singlet	2	-406.1510344	-330.6728507	-169.2370489	EMD1
ATCGTGATCAGACCTA-1	singlet	2	-1212.139552	-1041.358248	-403.2925916	EMD1
ATCGTGATCGATACTG-1	singlet	2	-630.1262641	-511.7246629	-244.9519683	EMD1
ATCGTGATCGCATTAG-1	singlet	0	-364.3519975	-1032.259221	-1064.252347	EMD1
ATCTCTAAGAGTCTTC-1	singlet	2	-2429.976492	-2059.687631	-925.8417957	EMD1
ATCTCTAAGCTGTTCA-1	singlet	2	-640.2819537	-659.2015445	-280.5505509	EMD1
ATCTCTACACCGTACG-1	singlet	2	-663.7535205	-530.1098285	-238.8629665	EMD1
ATCTCTACAGGTCCGT-1	singlet	1	-612.0873427	-209.1440573	-616.3008777	EMD1
ATCTCTAGTACAGCGA-1	singlet	2	-398.0290256	-266.601878	-140.6927718	EMD1
ATCTCTAGTATCAAGA-1	singlet	2	-324.6960748	-272.6465862	-132.3668403	EMD1
ATCTCTAGTTATCCAG-1	singlet	1	-1126.768076	-433.2236645	-917.2588906	EMD1
ATCTCTAGTTGGTACT-1	singlet	0	-37.41087329	-116.3437805	-123.55545	EMD1
ATCTCTATCCAACTGA-1	singlet	1	-821.9761562	-292.4583527	-587.8747456	EMD1
ATCTCTATCTGCGAGC-1	singlet	2	-671.4896831	-567.21134	-270.3579206	EMD1
ATCTTCAAGACTAAGT-1	singlet	1	-2345.920241	-787.4920975	-1921.314266	EMD1
ATCTTCAAGCATCAGG-1	singlet	0	-246.4783849	-789.0721729	-656.6939322	EMD1
ATCTTCAAGCATCCTA-1	singlet	2	-809.9426358	-699.0321581	-316.1078878	EMD1
ATCTTCAAGTGCTAGG-1	singlet	2	-311.5776468	-293.4794145	-163.3324776	EMD1
ATCTTCACAGAGCTAG-1	singlet	2	-605.7073854	-553.8548532	-233.7908352	EMD1
ATCTTCACATGCCGAC-1	singlet	0	-369.2176648	-974.6220343	-924.2243754	EMD1
ATCTTCAGTCTGTCCT-1	singlet	1	-3003.656734	-797.3391189	-2191.663219	EMD1
ATCTTCAGTCTGTGCG-1	singlet	2	-383.4694981	-236.4089464	-131.508454	EMD1
ATCTTCAGTGAATTGA-1	singlet	2	-1824.309334	-1536.076929	-613.59734	EMD1
ATCTTCAGTGCGAACA-1	singlet	2	-75.86447824	-64.3191159	-28.5333222	EMD1
ATCTTCATCAGCTTCC-1	singlet	2	-3216.708331	-2627.852136	-1106.625569	EMD1
ATGAAAGAGATGTAGT-1	singlet	1	-3808.753937	-1250.513367	-3033.178934	EMD1
ATGAAAGAGTAACCTC-1	singlet	0	-439.1402952	-987.7720316	-988.8374861	EMD1
ATGAAAGCACCAGCCA-1	singlet	0	-130.849802	-356.5773753	-348.6186503	EMD1
ATGAAAGCACCTTCGT-1	singlet	0	-414.6125918	-1107.467871	-1105.942304	EMD1
ATGAAAGCAGCATCTA-1	singlet	2	-298.4761251	-245.2683638	-131.5532621	EMD1
ATGAAAGCAGGGACTA-1	singlet	1	-3358.028336	-1251.472366	-2799.188311	EMD1
ATGAAAGGTCTGGTTA-1	singlet	2	-934.5760399	-797.6065199	-377.3950356	EMD1
ATGAAAGTCGCAACAT-1	singlet	1	-508.5143656	-217.1582904	-536.692506	EMD1
ATGACCAAGAATCTAG-1	singlet	0	-449.7720795	-1448.567074	-1242.473824	EMD1
ATGACCAAGAGGCCAT-1	singlet	1	-3441.646338	-1129.802195	-2941.397342	EMD1
ATGACCAAGAGGTTAT-1	singlet	0	-221.0565058	-576.0538416	-588.2767459	EMD1
ATGACCAAGCTAATGA-1	singlet	0	-1262.859129	-3506.872778	-3528.202884	EMD1
ATGACCACATCGTTCC-1	singlet	2	-521.3967935	-422.1832954	-205.723203	EMD1
ATGACCACATGGGTCC-1	singlet	2	-543.1578663	-468.2806374	-248.3047445	EMD1
ATGACCAGTAGCGAGT-1	singlet	1	-1841.52713	-534.0672755	-1247.009517	EMD1
ATGACCAGTAGCGTTT-1	unassigned	0	-4.446371352	-5.208213579	-4.859007346	EMD1
ATGACCAGTCATTCCC-1	singlet	2	-677.2928385	-557.711688	-216.2952167	EMD1
ATGACCAGTCCAAGAG-1	singlet	0	-666.5695542	-2464.464209	-2130.873595	EMD1
ATGAGGGCAATCAAGA-1	singlet	1	-389.1727377	-148.7965533	-280.8068859	EMD1
ATGAGGGCACAAATGA-1	singlet	0	-38.93326042	-150.6512325	-142.497952	EMD1
ATGAGGGCACCGAATT-1	singlet	1	-3945.57545	-1062.516029	-3265.592853	EMD1
ATGAGGGTCATGTCAG-1	singlet	2	-3278.519015	-2994.348339	-1115.881036	EMD1
ATGAGGGTCCTCACCA-1	singlet	2	-661.4355263	-342.2892046	-204.746706	EMD1
ATGAGGGTCGGATTAC-1	doublet	45293	-1309.710251	-738.5574525	-810.3439544	EMD1
ATGAGTCAGCATCCCG-1	singlet	2	-577.9692146	-498.9410301	-249.2716597	EMD1
ATGAGTCAGTAACGAT-1	singlet	0	-2699.715035	-9285.941726	-8764.130139	EMD1
ATGAGTCAGTCAGAGC-1	singlet	2	-385.2813849	-323.4122389	-136.0853158	EMD1
ATGAGTCAGTCGGCAA-1	singlet	2	-260.6834856	-193.4462643	-69.82428841	EMD1
ATGAGTCCACGGGTAA-1	singlet	2	-3385.744984	-2832.685332	-1282.660966	EMD1
ATGAGTCCATAATGAG-1	singlet	1	-162.4333813	-48.0240153	-135.6705028	EMD1
ATGAGTCCATACAGCT-1	singlet	2	-1336.58499	-1268.230494	-491.1895876	EMD1
ATGAGTCCATGACACT-1	singlet	1	-3930.646757	-1287.547091	-3119.464723	EMD1
ATGAGTCGTCTACAGT-1	singlet	2	-580.6554757	-470.6901464	-187.380842	EMD1
ATGAGTCGTGATAGTA-1	singlet	2	-61.7279118	-48.85472431	-33.17395979	EMD1
ATGAGTCGTTTCCATT-1	singlet	0	-210.6596409	-712.7553785	-685.8987763	EMD1
ATGAGTCTCAGGAAAT-1	singlet	2	-436.9242723	-426.5242581	-177.5353277	EMD1
ATGAGTCTCCATTCAT-1	singlet	2	-4378.770641	-3316.944698	-1421.054813	EMD1
ATGAGTCTCTCTGCCA-1	singlet	2	-1269.000374	-1320.423502	-501.1383298	EMD1
ATGATCGAGAAAGCGA-1	singlet	2	-203.8557427	-198.8815262	-100.961665	EMD1
ATGATCGAGCATGAAT-1	singlet	1	-2236.258557	-674.3996964	-1673.033088	EMD1
ATGATCGGTACACTCA-1	singlet	2	-277.8664869	-207.7151522	-97.49162265	EMD1
ATGATCGGTATCGTTG-1	singlet	2	-431.6524546	-355.1867806	-189.9101211	EMD1
ATGATCGGTGAATTGA-1	singlet	2	-2091.703044	-1684.364809	-762.4275058	EMD1
ATGATCGGTTGTACGT-1	singlet	0	-212.3222495	-736.394216	-570.8614682	EMD1
ATGATCGTCAAACCTG-1	singlet	2	-699.3095885	-517.0738415	-231.3537085	EMD1
ATGATCGTCATCGGGC-1	singlet	2	-233.332476	-248.7357993	-94.05550454	EMD1
ATGATCGTCGACATTG-1	singlet	2	-1416.278878	-1144.459591	-492.8256511	EMD1
ATGATCGTCGGCTGAC-1	singlet	2	-1319.606161	-1171.268634	-452.0521253	EMD1
ATGATCGTCGGTAACT-1	singlet	1	-798.7808259	-276.8737856	-634.3385217	EMD1
ATGATCGTCTGGAAGG-1	singlet	0	-285.7285719	-868.5860756	-797.1457644	EMD1
ATGATCGTCTGGTGGC-1	singlet	0	-1398.122784	-5607.741528	-5248.322816	EMD1
ATGCATGAGGTCTGGA-1	singlet	0	-234.6060467	-715.4191146	-719.831658	EMD1

ATGCATGCAGAGATTA-1	singlet	2	-959.7275975	-869.6780939	-440.2484543	EMD1
ATGCATGCAGCCCACA-1	singlet	2	-140.8172937	-112.7192024	-45.59991851	EMD1
ATGCATGCAGCTGTTA-1	singlet	1	-5145.727023	-1978.045541	-4479.516603	EMD1
ATGCATGGTAGCCCTG-1	singlet	0	-793.2594754	-2294.249444	-2358.698469	EMD1
ATGCATGTCAACGAGG-1	singlet	0	-388.612233	-1448.492961	-1200.323571	EMD1
ATGCATGTCTAGACCA-1	singlet	0	-164.4151561	-469.413197	-536.8762358	EMD1
ATGCATGTCTTCTGGC-1	singlet	1	-5073.728153	-1137.817047	-4386.380623	EMD1
ATGCCTCAGACTCGAG-1	doublet	45293	-3715.033828	-2105.281039	-2138.052069	EMD1
ATGCCTCAGAGCTGAC-1	singlet	2	-306.1559671	-285.5026705	-130.8936268	EMD1
ATGCCTCAGCTGTGCC-1	singlet	0	-246.8854253	-601.2468168	-688.8584219	EMD1
ATGCCTCCACGTTCGG-1	singlet	1	-1721.936373	-599.1436606	-1454.285475	EMD1
ATGCCTCCAGTTGAAA-1	singlet	2	-455.7751225	-416.803313	-220.0753446	EMD1
ATGCCTCGTCACTCTC-1	singlet	2	-398.2231344	-311.9207422	-148.2700622	EMD1
ATGCCTCGTCCAAGAG-1	singlet	0	-868.442902	-3373.457196	-3138.17578	EMD1
ATGCCTCGTTCCGCAG-1	singlet	2	-539.9751864	-467.0530871	-225.804594	EMD1
ATGCCTCGTTTACACG-1	singlet	2	-1623.894439	-1391.784826	-590.3371083	EMD1
ATGCCTCTCCATCACC-1	singlet	2	-456.4912465	-342.4892215	-174.6481022	EMD1
ATGCCTCTCCGTTTCG-1	singlet	0	-1593.076195	-6821.209868	-6393.784851	EMD1
ATGCGATAGGCAATGC-1	singlet	0	-320.4209494	-970.4722887	-994.9873927	EMD1
ATGCGATAGGTCCTGC-1	singlet	0	-152.0432166	-457.8026841	-429.365258	EMD1
ATGCGATAGGTTAGTA-1	singlet	2	-499.7573179	-427.7520082	-186.7506339	EMD1
ATGCGATAGTCTGGAG-1	singlet	2	-396.2390256	-256.3110544	-130.4699791	EMD1
ATGCGATCAAGCTCTA-1	singlet	1	-2330.192154	-745.9734139	-1722.614884	EMD1
ATGCGATCACGACAAG-1	singlet	2	-1365.218406	-1825.2589	-291.1860153	EMD1
ATGCGATCATCCGAGC-1	singlet	2	-430.5469044	-326.3389418	-163.1860631	EMD1
ATGCGATCATCCTTGC-1	singlet	0	-45.84252794	-423.9523627	-291.9222372	EMD1
ATGCGATGTACGCGTC-1	singlet	1	-1435.062371	-518.6684634	-1116.269858	EMD1
ATGCGATGTCTGTGCG-1	singlet	2	-1127.650959	-940.8039672	-387.6335246	EMD1
ATGCGATTCAACACGT-1	unassigned	0/2	-21.72578253	-46.9310299	-35.60726422	EMD1
ATGCGATTCAATGCAC-1	singlet	2	-623.9920882	-584.3683562	-254.7946461	EMD1
ATGCGATTCCTCTTTC-1	singlet	0	-117.0347865	-326.3999399	-309.2381032	EMD1
ATGCGATTCGAATGCT-1	singlet	2	-470.8730833	-340.1324842	-167.4915741	EMD1
ATGCGATTCGGTCATA-1	singlet	0	-28.65509815	-105.1870519	-87.64786734	EMD1
ATGGAGGAGGACGGAG-1	singlet	2	-525.5829651	-424.1704829	-196.5274753	EMD1
ATGGAGGAGGCTAACG-1	singlet	1	-3597.360405	-1172.072613	-2923.003588	EMD1
ATGGAGGAGGGACAGG-1	singlet	2	-355.5768688	-311.9702122	-124.4753434	EMD1
ATGGAGGAGTCGCCAC-1	singlet	2	-381.6025953	-302.8718184	-142.8502169	EMD1
ATGGAGGCATGAAAGT-1	singlet	0	-740.7038264	-2174.257723	-2128.227442	EMD1
ATGGAGGGTACATACC-1	singlet	2	-2349.88158	-2390.647628	-627.4439058	EMD1
ATGGAGGTCACGATCA-1	singlet	2	-522.6583365	-488.6765648	-215.2326067	EMD1
ATGGAGGTCATACGGT-1	singlet	1	-10858.20693	-2332.150237	-8026.676035	EMD1
ATGGAGGTCCTCACTG-1	singlet	1	-982.7102291	-356.2795393	-757.2281086	EMD1
ATGGATCAGTCAATCC-1	singlet	0	-557.0855432	-1993.794988	-2055.473077	EMD1
ATGGATCCAACAAGTA-1	singlet	2	-330.0290601	-272.3961467	-121.7542436	EMD1
ATGGATCCACACTTAG-1	singlet	2	-694.8895554	-559.1460869	-270.8330067	EMD1
ATGGATCCACGCTATA-1	doublet	0/2	-1157.654864	-1841.82871	-1377.080592	EMD1
ATGGATCTCAAGCGTT-1	singlet	0	-1211.462772	-3744.809662	-3793.38317	EMD1
ATGGGAGCACCTCTGT-1	singlet	1	-2299.957585	-740.0221088	-1840.584315	EMD1
ATGGGAGCAGCCTATA-1	singlet	1	-3641.967262	-1140.338201	-3061.055673	EMD1
ATGGGAGCAGTAGGAC-1	singlet	2	-442.9885445	-348.4064134	-175.2085892	EMD1
ATGGGAGCATAATGAG-1	singlet	1	-943.0408316	-305.3307206	-712.3808484	EMD1
ATGGGAGCATGATCTG-1	singlet	2	-3204.125195	-2913.130513	-1009.262645	EMD1
ATGGGAGCATTACGGT-1	singlet	2	-507.5387184	-422.1149613	-236.5657712	EMD1
ATGGGAGGTGGTAACG-1	singlet	2	-766.0220711	-663.0981846	-295.7162386	EMD1
ATGGGAGGTTCATCGA-1	singlet	2	-290.7993088	-300.9206152	-121.3499767	EMD1
ATGGGAGTCAAATGCC-1	singlet	2	-672.714474	-593.431891	-251.733479	EMD1
ATGGGTTAGCACCGAA-1	singlet	2	-1374.117527	-1171.225573	-582.8622149	EMD1
ATGGGTTAGGGCCCTT-1	singlet	2	-1473.774717	-1399.762718	-476.6646324	EMD1
ATGGGTTAGTGCAAAT-1	singlet	0	-460.9753197	-1402.422988	-1446.190183	EMD1
ATGGGTTCAAGCACAG-1	singlet	1	-2223.609608	-865.7013614	-1924.237263	EMD1
ATGGGTTCACTCCTGT-1	singlet	0	-251.4903135	-804.0123482	-813.2527904	EMD1
ATGGGTTCATAAGCGG-1	singlet	0	-137.4175371	-394.9010076	-336.7823082	EMD1
ATGGGTTCATAATGAG-1	singlet	0	-227.0889914	-619.7672338	-573.9738592	EMD1
ATGGGTTCATCCCGTT-1	singlet	2	-1982.988608	-1634.314229	-633.5750255	EMD1
ATGGGTTGTAAGTTAG-1	singlet	2	-526.3732629	-381.4321199	-50.94946699	EMD1
ATGGGTTTCCTTCGAC-1	singlet	1	-1350.409573	-510.419491	-1078.361236	EMD1
ATGGGTTTCGACCCAG-1	singlet	2	-321.1954877	-300.4495404	-118.215981	EMD1
ATGGGTTTCGCAAGAG-1	singlet	2	-682.3413891	-626.4849288	-232.7564403	EMD1
ATGGTTGAGCACACAG-1	singlet	0	-844.5650761	-2074.420293	-2095.069047	EMD1
ATGGTTGAGTCAGAGC-1	singlet	2	-671.8882187	-545.1715754	-295.6460199	EMD1
ATGGTTGCAACGCATT-1	singlet	2	-302.1622769	-218.4984652	-100.6113216	EMD1
ATGGTTGCACGTGAGA-1	singlet	2	-441.5198089	-366.2753746	-182.3945336	EMD1
ATGGTTGCATAGCACT-1	singlet	2	-1197.300122	-839.7755506	-390.6792107	EMD1
ATGGTTGCATGACTCA-1	singlet	0	-152.1245727	-396.0723997	-385.0578468	EMD1
ATGGTTGTCACCGCTT-1	singlet	0	-678.8583013	-2117.98308	-2034.385861	EMD1
ATGGTTGTCGCCAACG-1	singlet	1	-664.5666595	-259.3933345	-607.3025167	EMD1
ATGTCCCAGATACAGT-1	singlet	0	-39.39756548	-189.3577586	-154.081954	EMD1
ATGTCCCAGATAGCTA-1	singlet	0	-215.6100018	-642.2755515	-648.5662571	EMD1
ATGTCCCAGTAAGCAT-1	singlet	1	-1326.181079	-457.240839	-937.4472007	EMD1
ATGTCCCCACAATGTC-1	singlet	2	-694.8984203	-600.8119011	-302.3391151	EMD1
ATGTCCCCAGACTGCC-1	singlet	0	-290.2273861	-785.7513191	-840.2696335	EMD1
ATGTCCCGTATACCTG-1	singlet	1	-107.8694369	-34.16812564	-87.72478749	EMD1
ATGTCCCGTCATGACT-1	singlet	2	-368.5048556	-300.7296785	-146.2185536	EMD1
ATGTCCCGTCTCTCCA-1	singlet	1	-7909.418644	-2224.191462	-6080.325145	EMD1
ATGTCCCGTGTGACCC-1	singlet	2	-1250.277733	-1156.17868	-445.1980206	EMD1
ATGTCCCTCATCCTAT-1	singlet	2	-823.3666093	-710.3676714	-334.4015715	EMD1
ATGTCCCTCCACCTCA-1	singlet	2	-435.9560603	-357.0815066	-170.5671567	EMD1
ATGTCCCTCCCTTGTG-1	singlet	2	-766.7396422	-593.5778944	-288.7947692	EMD1
ATGTCCCTCCGTCCTA-1	singlet	0	-634.2078055	-1921.918238	-1824.095013	EMD1
ATGTCTTAGCCGATCC-1	singlet	2	-585.8957446	-486.9289927	-241.4869538	EMD1
ATGTCTTAGTAACGTA-1	singlet	2	-510.7283405	-433.4686641	-184.9755093	EMD1
ATGTCTTAGTGGCCTC-1	singlet	2	-1393.822915	-1330.862701	-468.2886069	EMD1
ATGTCTTCAAATTAGG-1	singlet	0	-1055.204458	-3478.727363	-3588.061462	EMD1
ATGTCTTCACACCTTC-1	singlet	2	-453.3095165	-367.1585005	-221.2000853	EMD1
ATGTCTTCACTCACTC-1	singlet	0	-108.4572132	-322.7553069	-270.3854622	EMD1
ATGTCTTGTATGGAAT-1	singlet	1	-2193.985214	-479.1602383	-1722.242488	EMD1
ATGTCTTGTCTTTCAT-1	singlet	0	-653.7156301	-1775.81329	-1824.374221	EMD1
ATGTCTTTCCAGCTCT-1	singlet	2	-547.2416578	-428.7931355	-219.758089	EMD1
ATGTCTTTCTGGAGAG-1	singlet	1	-2886.622542	-1055.061474	-2312.392971	EMD1
ATTACCTAGCGGATCA-1	doublet	45293	-162.5490997	-62.56864111	-102.1160034	EMD1
ATTACCTAGGATACAT-1	singlet	2	-629.551775	-525.2788212	-227.8956933	EMD1
ATTACCTAGTATAACG-1	singlet	2	-611.761889	-489.2227421	-241.451747	EMD1
ATTACCTCACACCTGG-1	singlet	1	-1739.877011	-543.8151847	-1432.076236	EMD1
ATTACCTCACCGTACG-1	singlet	1	-1558.888889	-613.8977127	-1315.635663	EMD1
ATTACCTCAGTGAGCA-1	singlet	1	-1137.848608	-390.0061543	-871.3722959	EMD1
ATTACCTCATAGGTAA-1	singlet	2	-2109.910032	-2061.722684	-731.5508522	EMD1
ATTACCTCATTAGGAA-1	singlet	2	-302.8304992	-238.3507326	-117.8783329	EMD1
ATTACCTGTATTAAGG-1	singlet	2	-1966.95096	-1811.054152	-747.5336447	EMD1
ATTACCTGTTAGTTCG-1	singlet	0	-271.3749818	-738.7261047	-968.0120949	EMD1
ATTACCTTCAACCCGG-1	singlet	0	-509.7154382	-1450.837093	-1587.843266	EMD1

ATTACCTTCAGTGATC-1	singlet	2	-1544.019756	-1533.721933	-526.6305807	EMD1
ATTACCTTCGAAGTGG-1	singlet	2	-513.3609321	-408.6865743	-162.9040777	EMD1
ATTACCTTCTGTGCTC-1	singlet	0	-659.5654255	-2492.121049	-2340.685293	EMD1
ATTACTCAGAGGCCAT-1	singlet	2	-1177.781589	-1040.077045	-543.2667372	EMD1
ATTACTCCACCAGTTA-1	singlet	2	-1027.890783	-1084.622611	-404.8352382	EMD1
ATTACTCGTAAGACCG-1	singlet	0	-256.3769634	-814.8263183	-754.7258192	EMD1
ATTACTCGTACGTTCA-1	singlet	0	-363.3569584	-1219.497466	-1222.982573	EMD1
ATTACTCGTTCATCGA-1	singlet	2	-1815.316647	-1451.25837	-666.228948	EMD1
ATTATCCAGCCGGAAT-1	singlet	2	-850.8313359	-719.7395266	-370.5985925	EMD1
ATTATCCAGCTGTCCG-1	singlet	1	-1340.184923	-459.2857462	-1028.726918	EMD1
ATTATCCCAAGTTCGT-1	singlet	1	-1422.813255	-478.1113841	-1064.571184	EMD1
ATTATCCCAGACCTAT-1	singlet	0	-292.64465	-829.6373025	-777.4436192	EMD1
ATTATCCCAGTCAGTT-1	singlet	1	-78.70889076	-25.49494119	-51.77698711	EMD1
ATTATCCCATATCGGT-1	singlet	1	-1041.930357	-397.7354114	-866.135213	EMD1
ATTATCCCATTGCCGG-1	singlet	1	-2588.136178	-859.6928463	-2133.47607	EMD1
ATTATCCGTATCTTCT-1	singlet	2	-714.5355216	-615.7246586	-309.7812172	EMD1
ATTATCCGTGACCGTC-1	singlet	2	-1322.879731	-1042.841613	-492.4120675	EMD1
ATTATCCTCCGTAGTA-1	singlet	2	-402.9517768	-357.4284493	-162.1550634	EMD1
ATTATCCTCCGTCCTA-1	singlet	1	-453.4134251	-199.7070787	-354.8036122	EMD1
ATTATCCTCTTTACAC-1	singlet	0	-303.2778959	-1037.068162	-1068.430468	EMD1
ATTCACTAGTTGAAGT-1	singlet	2	-2954.24863	-2918.232935	-1036.499058	EMD1
ATTCACTCACTGCGTG-1	singlet	2	-154.2041108	-148.7377317	-50.23546002	EMD1
ATTCACTCAGACCGCT-1	singlet	2	-1803.071474	-1723.543114	-624.3370887	EMD1
ATTCACTCATTGCCGG-1	singlet	1	-5893.402436	-1985.169892	-4810.77531	EMD1
ATTCACTGTCCAAAGG-1	singlet	1	-1721.477461	-698.4698001	-1420.846299	EMD1
ATTCACTGTGGCCTCA-1	singlet	1	-1195.38569	-370.318564	-1022.864268	EMD1
ATTCACTGTGGCGTAA-1	singlet	2	-2260.866906	-2044.333775	-779.1302298	EMD1
ATTCACTGTGTGTGGA-1	singlet	0	-2003.092015	-6766.734184	-6467.31971	EMD1
ATTCACTGTGTTCGAT-1	singlet	0	-2240.240548	-7624.497567	-7498.994926	EMD1
ATTCACTTCGGTCATA-1	singlet	2	-535.9126635	-461.2522314	-253.0374059	EMD1
ATTCAGGCACTGTGAT-1	singlet	2	-585.6368292	-491.6781885	-244.0695761	EMD1
ATTCAGGCAGCACAAG-1	singlet	0	-153.5559533	-443.2768487	-503.7222037	EMD1
ATTCAGGCATGACAGG-1	singlet	0	-1560.462002	-4645.494937	-4528.897425	EMD1
ATTCAGGGTACCGTCG-1	singlet	2	-3346.051592	-2828.494983	-1109.754399	EMD1
ATTCAGGGTACGACTT-1	singlet	1	-6789.203089	-1069.033269	-5352.799658	EMD1
ATTCAGGGTACTGGGA-1	singlet	0	-238.0045214	-565.8035012	-633.0573598	EMD1
ATTCAGGTCTAGACCA-1	singlet	2	-507.9773207	-441.3312491	-234.697259	EMD1
ATTCATCAGATGCAGC-1	singlet	1	-2703.157278	-816.5685796	-1923.303853	EMD1
ATTCATCAGGGTCACA-1	singlet	1	-1977.237611	-685.20509	-1613.010702	EMD1
ATTCATCAGTAAATGC-1	singlet	0	-902.4451709	-3166.328147	-2923.243306	EMD1
ATTCATCCAACAGCTT-1	singlet	2	-1403.003971	-1291.881857	-441.3887405	EMD1
ATTCATCCACGAGAAC-1	singlet	2	-630.9152223	-635.2080313	-283.0895855	EMD1
ATTCATCCACTTGAGT-1	singlet	1	-537.8833619	-214.4053946	-466.0094834	EMD1
ATTCATCTCACTTGTT-1	singlet	0	-1067.433238	-4051.292302	-4360.641448	EMD1
ATTCATCTCATCGCCT-1	singlet	0	-163.1504934	-601.8214406	-541.3772961	EMD1
ATTCATCTCGCCGATG-1	doublet	0/1	-576.4407463	-652.6436926	-869.2748261	EMD1
ATTCATCTCGTGGCTG-1	singlet	2	-493.9698716	-482.9331898	-229.3014546	EMD1
ATTCCATCAACGGCCT-1	singlet	2	-873.5193225	-871.5871522	-374.0436842	EMD1
ATTCCATCAAGAAATC-1	singlet	2	-436.9853824	-390.983204	-179.3438364	EMD1
ATTCCATCAAGATGGC-1	doublet	45323	-10095.99055	-6090.667366	-5644.883228	EMD1
ATTCCATCACCATTCC-1	singlet	2	-496.9103098	-423.6406807	-204.7947167	EMD1
ATTCCATGTAGCACGA-1	singlet	2	-507.0997073	-480.5811319	-224.1633786	EMD1
ATTCCATTCACTACGA-1	singlet	1	-1146.949766	-400.1922091	-819.3429276	EMD1
ATTCCATTCCTTCACG-1	singlet	0	-31.53604421	-67.67596304	-89.87996887	EMD1
ATTCCATTCTACGCAA-1	singlet	1	-821.165555	-262.515066	-770.9135041	EMD1
ATTCCATTCTTCCAGC-1	singlet	2	-3567.371246	-2670.393176	-1030.110798	EMD1
ATTCCCGCACTAACCA-1	doublet	0/2	-1432.534523	-2245.661867	-1901.106696	EMD1
ATTCCCGGTCAGGCAA-1	singlet	1	-3948.785923	-1357.651829	-3073.012066	EMD1
ATTCCCGGTGACTCTA-1	singlet	1	-1282.779455	-505.5810661	-1107.52859	EMD1
ATTCCCGGTGCCTTCT-1	singlet	0	-332.7900052	-982.8404749	-896.8115789	EMD1
ATTCCCGTCCCGAAAT-1	singlet	1	-1158.636729	-474.8402649	-880.5812114	EMD1
ATTCCCGTCCCTGGTT-1	singlet	2	-852.4254219	-683.683189	-337.2990851	EMD1
ATTCCCGTCCTGCTAC-1	singlet	1	-1659.570505	-620.1037741	-1250.163222	EMD1
ATTCCCGTCTTAGCTT-1	doublet	0/2	-826.1483009	-1486.470789	-1237.248133	EMD1
ATTCCTAAGGGCAGGA-1	singlet	0	-1499.933359	-4507.972611	-4301.30894	EMD1
ATTCCTACACCCTCTA-1	singlet	2	-753.5518812	-679.4609467	-317.1149316	EMD1
ATTCCTACATGACTGT-1	singlet	2	-593.1512463	-520.8429167	-265.3283491	EMD1
ATTCCTAGTGCAATGG-1	singlet	0	-998.3243677	-4417.496322	-3811.490378	EMD1
ATTCCTAGTGTACAGG-1	singlet	2	-422.7449299	-373.4918345	-205.6477475	EMD1
ATTCCTATCATTACCT-1	singlet	2	-671.9216289	-525.5251889	-245.9900902	EMD1
ATTCCTATCATTTGTC-1	singlet	2	-565.5091175	-399.8146745	-212.106299	EMD1
ATTCCTATCGGTTCAA-1	singlet	2	-1610.513498	-1267.277535	-560.0100051	EMD1
ATTCGTTAGCTCCGAC-1	singlet	2	-275.9946152	-257.9784928	-122.4713765	EMD1
ATTCGTTCAAAGCTCT-1	singlet	2	-1827.196421	-1757.005388	-731.69642	EMD1
ATTCGTTGTCAGGAGT-1	singlet	2	-4518.533459	-3707.215279	-1622.376544	EMD1
ATTCGTTGTCTAGATC-1	singlet	0	-2045.276107	-6511.102291	-6444.20486	EMD1
ATTCGTTGTGACTCGC-1	singlet	0	-186.246338	-442.6963009	-466.269051	EMD1
ATTCGTTGTGCTCGTG-1	singlet	1	-1522.515253	-364.9628209	-1165.097531	EMD1
ATTCGTTGTTGGGCCT-1	singlet	2	-432.3508912	-368.7505115	-207.8035573	EMD1
ATTCGTTTCACAACCA-1	singlet	2	-2568.554119	-2002.062063	-801.4050708	EMD1
ATTCTACAGAATCCCT-1	singlet	0	-350.9286304	-1158.819908	-1068.356878	EMD1
ATTCTACAGGCCTAAG-1	singlet	1	-6212.574517	-1823.540829	-5045.093902	EMD1
ATTCTACAGGCTAACG-1	singlet	2	-504.6013764	-435.8978534	-194.6574009	EMD1
ATTCTACAGGTGCGAT-1	doublet	45293	-3357.324456	-1373.458412	-2398.857916	EMD1
ATTCTACCAAACCGGA-1	singlet	0	-598.6350347	-1851.637351	-1848.887323	EMD1
ATTCTACGTAGTAAGT-1	singlet	1	-2287.469934	-588.5763872	-2082.908375	EMD1
ATTCTACGTATGCAAA-1	singlet	1	-557.7338846	-219.4188319	-561.4131213	EMD1
ATTCTACGTGCTTATG-1	singlet	1	-3144.929922	-991.5241611	-2426.099836	EMD1
ATTCTTGAGAAGCCTG-1	singlet	1	-250.5920583	-108.5897219	-188.4847395	EMD1
ATTCTTGAGATAACAC-1	singlet	2	-770.5501871	-662.9067528	-227.1711275	EMD1
ATTCTTGAGATGTTCC-1	singlet	1	-1829.018757	-633.5027743	-1579.338486	EMD1
ATTCTTGAGGTTGGAC-1	doublet	45293	-2617.338471	-1300.378037	-1704.682724	EMD1
ATTCTTGAGTATGATG-1	singlet	1	-1623.717842	-606.9462545	-1347.376777	EMD1
ATTCTTGCAAGGTTGG-1	singlet	2	-360.4238518	-243.2435744	-127.5116777	EMD1
ATTCTTGCATCAGTGT-1	singlet	2	-670.7744639	-516.0671348	-221.850852	EMD1
ATTCTTGCATGGAACG-1	singlet	2	-488.1828034	-408.3245155	-189.6369625	EMD1
ATTCTTGGTAAGAACT-1	singlet	2	-357.7546015	-338.5604917	-134.9252892	EMD1
ATTCTTGGTCCGGACT-1	doublet	0/2	-474.1746149	-673.7018823	-527.0588263	EMD1
ATTCTTGGTGTGCCTG-1	singlet	2	-858.3290798	-851.9789662	-378.4160524	EMD1
ATTGGGTAGACTTCGT-1	singlet	0	-48.99292609	-178.323432	-252.0065638	EMD1
ATTGGGTCACATATCG-1	singlet	2	-585.8095013	-531.6477511	-274.3831136	EMD1
ATTGGGTCACGAGAAC-1	singlet	2	-2061.843579	-1816.85412	-664.3536822	EMD1
ATTGGGTCAGGCGAAT-1	singlet	1	-3811.689297	-1305.776068	-2989.274053	EMD1
ATTGGGTGTATCGCAT-1	singlet	0	-85.98866524	-307.9008372	-267.5758546	EMD1
ATTGGGTGTGGTGATG-1	singlet	0	-1012.322045	-2162.159752	-2363.941155	EMD1
ATTGGGTGTTCGTTCC-1	singlet	1	-125.343075	-30.23193336	-88.12693491	EMD1
ATTGGGTGTTGTTGAC-1	singlet	0	-398.6272928	-1260.176161	-1362.3684	EMD1
ATTGGGTTCAAGCCTA-1	singlet	1	-5539.258579	-1352.165137	-4353.813153	EMD1
ATTGGGTTCAGCTTGA-1	singlet	1	-1648.183593	-512.3888932	-1319.267406	EMD1
ATTGGGTTCATAGGCT-1	singlet	2	-1552.999189	-1242.600749	-560.9138337	EMD1

ATTGTTCAGCCTGAGA-1	singlet	1	-3637.692473	-1734.648938	-3417.108547	EMD1
ATTGTTCAGCTAATGA-1	singlet	0	-306.6235945	-878.0893539	-960.5609492	EMD1
ATTGTTCCACTCCGAG-1	singlet	0	-1034.770652	-2665.926281	-2743.486785	EMD1
ATTGTTCCATTCACCC-1	singlet	0	-138.7891133	-445.0927174	-393.558597	EMD1
ATTGTTCGTACGTACT-1	doublet	0/1	-2678.191609	-3995.473796	-4538.428822	EMD1
ATTGTTCGTATGGAGC-1	singlet	2	-184.5247302	-139.1077713	-57.51056416	EMD1
ATTGTTCTCAGTGATC-1	singlet	0	-904.9269122	-2608.956327	-2558.630426	EMD1
ATTGTTCTCTGCGATA-1	singlet	0	-197.563801	-550.0078831	-657.8797514	EMD1
ATTTACCAGCTACAAA-1	singlet	0	-193.7161779	-681.988204	-873.381324	EMD1
ATTTACCAGCTGAGTG-1	singlet	0	-380.120121	-1310.535379	-1595.6406	EMD1
ATTTACCAGGCTCTCG-1	singlet	0	-2015.031382	-6567.516328	-6623.775291	EMD1
ATTTACCAGTCGCGAA-1	singlet	2	-426.5803939	-399.7572823	-171.8514104	EMD1
ATTTACCAGTGCTCGC-1	singlet	2	-2275.996	-1786.753872	-793.2177201	EMD1
ATTTACCCAATTGTGC-1	singlet	2	-2095.399102	-1772.183626	-803.8514412	EMD1
ATTTACCCAGATCACT-1	singlet	2	-3428.886148	-2365.282233	-1023.629355	EMD1
ATTTACCCATCGGCCA-1	singlet	0	-340.3571032	-826.4612865	-842.9136476	EMD1
ATTTACCGTAAGGAGA-1	singlet	0	-448.8448129	-2167.956889	-1928.775492	EMD1
ATTTACCGTTGTCTAG-1	singlet	2	-585.8583703	-486.8589876	-243.5882713	EMD1
ATTTACCTCACCTACC-1	singlet	2	-2561.62011	-1974.908673	-900.9592981	EMD1
ATTTACCTCATGAAAG-1	doublet	1/0	-4104.709941	-2592.620182	-4371.139696	EMD1
ATTTCACAGCAGATAT-1	singlet	2	-728.9533084	-573.7683263	-305.9025088	EMD1
ATTTCACAGCCGTTAT-1	singlet	0	-273.7915433	-743.9702816	-670.1639367	EMD1
ATTTCACAGCTACTAC-1	singlet	0	-610.6506264	-1783.758787	-1722.501566	EMD1
ATTTCACCAAGTCGTT-1	singlet	0	-1113.366683	-2461.289202	-2313.470225	EMD1
ATTTCACCAGGTCAGA-1	singlet	1	-4435.26179	-1516.736175	-3476.60472	EMD1
ATTTCACCATGTGCTA-1	singlet	0	-103.1004303	-330.1928319	-334.9698739	EMD1
ATTTCACCATTCACCC-1	singlet	1	-3460.957376	-1197.656901	-3087.626747	EMD1
ATTTCACCATTGCCTC-1	singlet	1	-8082.36574	-2607.380964	-6787.085139	EMD1
ATTTCACGTGGGCTCT-1	singlet	2	-1494.29139	-1382.823151	-561.0222573	EMD1
ATTTCACTCGTAACAC-1	singlet	2	-113.5538827	-107.7397047	-43.68075384	EMD1
ATTTCACTCGTAGCCG-1	singlet	0	-1129.32218	-2963.644367	-2934.604087	EMD1
ATTTCTGAGATAGCAT-1	singlet	2	-854.2025544	-676.7075618	-299.9434954	EMD1
ATTTCTGAGGACGCAT-1	singlet	1	-96.98607929	-30.92860326	-60.36027197	EMD1
ATTTCTGAGGTCCTGC-1	doublet	0/2	-636.0610142	-857.7680863	-762.6568604	EMD1
ATTTCTGCAAGAAATC-1	singlet	1	-2823.65684	-1020.8219	-2191.38398	EMD1
ATTTCTGCAATAGTAG-1	singlet	2	-688.4052892	-551.7390166	-218.2848705	EMD1
ATTTCTGCAGATCCAT-1	singlet	1	-2559.983114	-612.726004	-2051.680295	EMD1
ATTTCTGCAGATTCGT-1	singlet	1	-6174.384389	-2689.910703	-4458.456869	EMD1
ATTTCTGCAGGAACCA-1	singlet	1	-4551.762769	-1484.054258	-3614.033306	EMD1
ATTTCTGCATCATTGG-1	singlet	0	-532.3282209	-1573.976139	-1510.416759	EMD1
ATTTCTGCATCGGATT-1	singlet	0	-276.0408077	-816.7249836	-736.4724025	EMD1
ATTTCTGGTAACGATA-1	singlet	0	-2303.972897	-10694.37157	-9552.118944	EMD1
ATTTCTGGTTTGGAGG-1	singlet	2	-465.844827	-301.3365998	-179.2729398	EMD1
ATTTCTGTCATTATCC-1	singlet	0	-832.4052669	-2879.543665	-2580.233674	EMD1
ATTTCTGTCGCATAGT-1	singlet	2	-394.7315666	-360.6001357	-190.2290209	EMD1
CAAAGAACAGCTATAC-1	singlet	0	-476.2949415	-1391.821134	-1418.669799	EMD1
CAAAGAAGTTGTTTGG-1	singlet	2	-81.84330548	-121.7461326	-32.07843321	EMD1
CAAAGAATCCGTGGTG-1	singlet	2	-501.4303947	-417.0500205	-192.276671	EMD1
CAAAGAATCGACATCA-1	singlet	2	-941.7614044	-883.797012	-365.7901628	EMD1
CAAAGAATCGCAAGAG-1	singlet	2	-330.4853086	-337.0432629	-139.04922	EMD1
CAAAGAATCTACAGGT-1	singlet	0	-318.7909713	-924.5628009	-895.5531877	EMD1
CAAAGAATCTACTTCA-1	doublet	2/0	-864.4093415	-771.7784275	-436.4254813	EMD1
CAACAACAGAAGCGCT-1	singlet	2	-243.5162997	-215.2697037	-106.5221089	EMD1
CAACAACAGCATCAAA-1	singlet	2	-550.5362118	-442.59226	-213.7481942	EMD1
CAACAACAGCCTCGTG-1	singlet	0	-898.1316913	-2797.829304	-2610.470067	EMD1
CAACAACAGCTGACAG-1	singlet	2	-2508.061128	-2122.477454	-838.9581592	EMD1
CAACAACAGTATCTGC-1	singlet	2	-616.0138935	-553.2725314	-294.3437894	EMD1
CAACAACAGTCCGCGT-1	singlet	2	-1220.784242	-965.9632442	-502.9102507	EMD1
CAACAACCAGCGGATA-1	singlet	0	-200.224788	-492.9553524	-526.0345088	EMD1
CAACAACGTACCTAGT-1	singlet	1	-605.9925687	-218.5680991	-443.6714126	EMD1
CAACAACTCATAAGGA-1	singlet	0	-196.4655929	-416.4868677	-623.9631232	EMD1
CAACAACTCCCGTGTT-1	singlet	1	-3115.302644	-1047.003466	-2624.618854	EMD1
CAACAACTCCTGCTAC-1	singlet	1	-37.29068574	-12.67081721	-20.34219486	EMD1
CAACAACTCGAACACT-1	singlet	1	-1103.617746	-426.5976921	-904.1195661	EMD1
CAACAGTCACCAGTAT-1	singlet	0	-360.5379665	-1040.297185	-1061.57042	EMD1
CAACAGTGTAAGATCA-1	singlet	2	-468.8621274	-388.9619273	-118.9951479	EMD1
CAACAGTGTATATGGA-1	singlet	1	-517.0140331	-192.7748132	-537.1080494	EMD1
CAACAGTTCAAACGTC-1	singlet	2	-584.4619157	-500.3021264	-213.5641649	EMD1
CAACAGTTCAGACTGT-1	singlet	1	-1666.481521	-571.9213959	-1465.19803	EMD1
CAACAGTTCCACGAAT-1	singlet	1	-659.3790935	-271.9997626	-699.4557182	EMD1
CAACAGTTCCTCTTTC-1	singlet	0	-280.4194965	-919.5126204	-813.9028441	EMD1
CAACAGTTCCTGGCTT-1	singlet	2	-545.8865494	-442.9815159	-208.8651233	EMD1
CAACAGTTCTACCACC-1	singlet	0	-144.7537421	-454.9354967	-424.5544919	EMD1
CAACAGTTCTACGCGG-1	singlet	2	-365.1075756	-304.9460383	-159.4977583	EMD1
CAACCAAAGCTGCCTG-1	doublet	0/1	-1081.951528	-1208.005465	-1547.554611	EMD1
CAACCAAAGTAAACAC-1	singlet	1	-4991.272642	-1410.389303	-3976.689788	EMD1
CAACCAATCCGACATA-1	singlet	2	-1512.427663	-1373.973769	-570.0460664	EMD1
CAACCAATCGCTTTAT-1	singlet	1	-97.88588848	-33.88462345	-71.02681787	EMD1
CAACCTCAGCAAATGT-1	singlet	1	-1606.44088	-573.478828	-1287.369499	EMD1
CAACCTCAGCTGTTCA-1	singlet	1	-9665.124472	-3067.475405	-7904.160376	EMD1
CAACCTCAGTGAGCCA-1	singlet	0	-112.6863954	-339.0964283	-313.6448527	EMD1
CAACCTCAGTGATAAC-1	singlet	1	-3118.967368	-1143.552833	-2791.170765	EMD1
CAACCTCCACGGGCTT-1	singlet	0	-966.8859755	-2734.733673	-2630.495204	EMD1
CAACCTCCAGAGGACT-1	singlet	1	-5447.592657	-1892.52976	-4305.109804	EMD1
CAACCTCCATGTGGTT-1	singlet	2	-1100.126486	-893.5511989	-418.6354001	EMD1
CAACCTCCATTGAAAG-1	singlet	2	-506.2429217	-424.5877011	-171.1777416	EMD1
CAACCTCGTACGAAAT-1	singlet	2	-671.6294664	-546.488712	-213.8328461	EMD1
CAACCTCGTCGCAACC-1	singlet	2	-825.9051736	-655.9336559	-303.5644168	EMD1
CAACCTCGTGCTCCGA-1	singlet	1	-1729.722563	-544.9083121	-1510.20476	EMD1
CAACCTCTCCGAGTGC-1	singlet	0	-457.2394899	-1195.850602	-1265.330845	EMD1
CAACCTCTCCTGTAGA-1	doublet	45323	-4633.845673	-3209.595292	-2317.320895	EMD1
CAACCTCTCTACTCAT-1	singlet	2	-2355.724146	-1911.779874	-854.4330326	EMD1
CAACGATAGTGACCTT-1	singlet	2	-931.6057792	-681.5983813	-298.559756	EMD1
CAACGATGTACCTAAC-1	singlet	0	-529.2268645	-1554.41826	-1588.010003	EMD1
CAACGATGTCGCGGTT-1	singlet	2	-335.054995	-272.7373788	-133.9026265	EMD1
CAACGATGTGTACGCC-1	singlet	0	-462.7292715	-1227.987018	-1104.936053	EMD1
CAACGATGTGTCATTG-1	singlet	1	-1023.188994	-375.296733	-806.3017257	EMD1
CAACGATGTTAGCGGA-1	singlet	2	-335.2817538	-284.8321741	-130.6225178	EMD1
CAACGATTCATTGTTC-1	singlet	2	-321.2677299	-307.2436837	-123.1651522	EMD1
CAACGGCAGCAACCAG-1	singlet	0	-19.53510611	-79.0302743	-85.13666321	EMD1
CAACGGCAGTTGTCGT-1	doublet	0/2	-251.7049986	-375.902186	-378.6329875	EMD1
CAACGGCCAAATCAGA-1	singlet	2	-230.8961324	-288.3380815	-117.6570636	EMD1
CAACGGCCACGAAAGC-1	singlet	0	-195.6052183	-558.8791037	-555.4253717	EMD1
CAACGGCCAGCTTTGA-1	singlet	2	-1171.302601	-870.2574556	-394.0586924	EMD1
CAACGGCGTATCGTAC-1	singlet	2	-2222.563321	-2033.19452	-644.4295042	EMD1
CAACGGCGTTCGGACC-1	singlet	2	-1744.015329	-1453.365489	-426.3869369	EMD1
CAACGGCTCACAATGC-1	singlet	1	-3890.082459	-1288.561674	-3282.792177	EMD1
CAACGGCTCAGTAGGG-1	singlet	0	-770.7282554	-2397.920077	-2241.256801	EMD1
CAACGGCTCTCGCCTA-1	singlet	0	-1101.968426	-2662.484464	-3004.404905	EMD1
CAACGGCTCTTCCGTG-1	singlet	0	-147.2458345	-570.4190037	-411.8691163	EMD1

CAAGACTAGAACCCGA-1	singlet	2	-424.0130329	-297.871319	-164.3047479	EMD1
CAAGACTAGACATAGT-1	singlet	2	-1650.294854	-1576.402037	-596.7030901	EMD1
CAAGACTAGAGTGACC-1	singlet	1	-494.1624763	-193.618885	-448.0644483	EMD1
CAAGACTAGTTCTACG-1	singlet	2	-4151.27286	-3387.298808	-1248.386932	EMD1
CAAGACTCAGGAGACT-1	singlet	1	-755.8105351	-312.1169066	-731.5533336	EMD1
CAAGACTGTCTACAAC-1	singlet	0	-557.3061089	-1778.201821	-1665.770534	EMD1
CAAGACTGTTGCAACT-1	singlet	0	-1221.843748	-3579.485066	-3439.672433	EMD1
CAAGAGGCAATACGCT-1	singlet	2	-1401.882518	-1129.088779	-527.836837	EMD1
CAAGAGGCACGCAGTC-1	singlet	2	-511.1610611	-505.4581011	-222.8771216	EMD1
CAAGAGGCACTATCGA-1	singlet	2	-656.4907567	-582.9887722	-242.5531734	EMD1
CAAGAGGCATACACCA-1	singlet	2	-289.0245925	-248.5187906	-111.72518	EMD1
CAAGAGGCATCTAACG-1	singlet	0	-683.410125	-2405.094651	-2182.837926	EMD1
CAAGAGGGTACGGATG-1	singlet	2	-744.1497095	-508.0061969	-259.7791043	EMD1
CAAGAGGGTAGTTAGA-1	singlet	2	-1123.93137	-918.4523719	-416.8242737	EMD1
CAAGAGGGTATCATGC-1	singlet	0	-335.3950423	-1250.277534	-1064.940523	EMD1
CAAGAGGGTCCTACGG-1	singlet	2	-221.9077417	-179.900002	-86.67721544	EMD1
CAAGAGGTCTTGAACG-1	singlet	2	-273.8923224	-215.7247468	-109.5130421	EMD1
CAAGCTAAGGTAAACT-1	singlet	2	-477.4534401	-385.3902111	-195.6913897	EMD1
CAAGCTAGTTATCCAG-1	singlet	2	-469.0898846	-395.9085459	-191.0376063	EMD1
CAAGCTATCAAATGAG-1	singlet	1	-5333.879019	-1705.197386	-3905.290395	EMD1
CAAGCTATCCGATAGT-1	singlet	2	-289.4109977	-290.5587417	-121.5869352	EMD1
CAAGCTATCCTATTTG-1	singlet	2	-553.4603618	-522.6390667	-255.6772166	EMD1
CAAGCTATCGACCAAT-1	singlet	2	-192.7930707	-202.3003755	-107.3163511	EMD1
CAAGCTATCGAGTTGT-1	singlet	2	-563.7286221	-506.3014605	-219.515034	EMD1
CAAGGGAAGAGCAGAA-1	singlet	0	-291.1771844	-916.6249603	-944.721691	EMD1
CAAGGGAAGCGCCTCA-1	singlet	2	-412.8582549	-344.5942615	-150.6343123	EMD1
CAAGGGACAACTGAAA-1	singlet	1	-4755.845457	-1606.964187	-3699.058687	EMD1
CAAGGGACATCCTTCG-1	singlet	2	-478.1816502	-376.3298014	-182.1829514	EMD1
CAAGGGAGTAGATCGG-1	singlet	2	-639.1694241	-619.7820765	-263.1125662	EMD1
CAAGGGAGTGCCTGAC-1	singlet	2	-1061.09801	-965.5216404	-346.8829838	EMD1
CAAGGGAGTGCTAGCC-1	singlet	0	-655.3695889	-1958.245756	-1885.926891	EMD1
CAAGGGAGTTAGTCGT-1	singlet	2	-157.4136791	-139.5947382	-47.76231049	EMD1
CAATACGAGGGTGAGG-1	singlet	2	-587.8324964	-655.0284753	-113.4853989	EMD1
CAATACGAGTAGAGTT-1	singlet	1	-4231.770271	-905.2084847	-3300.118405	EMD1
CAATACGAGTCGCCAC-1	singlet	0	-176.3269238	-458.8901263	-519.7134943	EMD1
CAATACGCAAGCCTGC-1	singlet	0	-36.34908839	-94.25927944	-85.62929767	EMD1
CAATACGCACTGCGAC-1	singlet	1	-895.147145	-329.490081	-658.03385	EMD1
CAATACGCAGACTCTA-1	singlet	2	-462.2925298	-371.6402319	-185.7862273	EMD1
CAATACGCAGAGATGC-1	singlet	2	-616.200921	-534.1704129	-220.7899924	EMD1
CAATACGCATCGTCCT-1	singlet	0	-226.697868	-607.422726	-586.8951784	EMD1
CAATACGGTCCAGCGT-1	singlet	2	-1578.429932	-1622.37845	-588.4739183	EMD1
CAATACGGTTATCTTC-1	singlet	2	-389.4666551	-325.1203223	-177.8134092	EMD1
CAATACGTCATACGAC-1	singlet	2	-1899.788635	-1611.668426	-705.2896541	EMD1
CAATACGTCCTCTAAT-1	singlet	2	-803.7483609	-668.4368952	-328.0037894	EMD1
CAATACGTCTCATTTG-1	singlet	2	-3248.036027	-2554.86412	-1066.347844	EMD1
CAATACGTCTCCATAT-1	singlet	2	-1731.670829	-1603.507028	-717.9933516	EMD1
CAATACGTCTTCACAT-1	singlet	0	-1510.757073	-4328.566212	-4429.700235	EMD1
CAATACGTCTTCTGGC-1	singlet	2	-334.1856702	-255.0297855	-131.5618187	EMD1
CAATCGAAGAGCAGAA-1	singlet	0	-372.3763648	-1304.945096	-1275.243535	EMD1
CAATCGAAGGACGGAG-1	singlet	1	-8531.187315	-2232.513964	-5994.334937	EMD1
CAATCGAAGTCGGCCT-1	singlet	2	-613.4322063	-482.0023809	-235.7331172	EMD1
CAATCGAAGTTGTAAG-1	singlet	2	-396.4090189	-295.8354545	-148.2572994	EMD1
CAATCGACAATCCTTT-1	singlet	2	-742.4681206	-582.1722863	-253.6624076	EMD1
CAATCGACAGCTTCCT-1	singlet	2	-444.8682585	-407.2206486	-168.6136739	EMD1
CAATCGAGTAAGACCG-1	singlet	2	-425.9596596	-362.0466565	-158.1469797	EMD1
CAATCGAGTACCTATG-1	singlet	2	-393.449664	-283.8473152	-188.587195	EMD1
CAATCGAGTGTTATCG-1	singlet	2	-247.5380345	-198.1677591	-87.4024694	EMD1
CAATCGATCACCTGTC-1	doublet	1/0	-1867.552448	-1147.30093	-1826.516624	EMD1
CAATCGATCTCTATGT-1	singlet	2	-866.3443995	-874.169663	-383.7548556	EMD1
CAATCGATCTGTGCTC-1	singlet	2	-437.473511	-391.9611582	-187.957814	EMD1
CAATGACAGGGAGGAC-1	singlet	2	-552.4208736	-542.0987919	-235.8100219	EMD1
CAATGACCAACAGCTT-1	singlet	0	-282.1939197	-790.1893083	-784.9312387	EMD1
CAATGACTCAATGTCG-1	singlet	1	-1450.942266	-505.4002127	-1185.998229	EMD1
CAATTTCAGCATTTCG-1	singlet	2	-1673.188333	-1455.600889	-694.1120081	EMD1
CAATTTCAGGTTAAAC-1	singlet	1	-1488.779648	-502.6998522	-1207.07032	EMD1
CAATTTCAGTACAGCG-1	singlet	2	-595.5431194	-568.1315844	-241.4760313	EMD1
CAATTTCAGTACTCGT-1	singlet	2	-336.9425516	-301.5416163	-153.9313576	EMD1
CAATTTCAGTGGTCAG-1	singlet	1	-508.2801355	-178.8884302	-454.1806724	EMD1
CAATTTCCAAGCGAGT-1	singlet	2	-355.8670186	-379.8655962	-127.7781947	EMD1
CAATTTCCACCAGGTC-1	singlet	0	-429.7989435	-1292.753032	-1285.19351	EMD1
CAATTTCCACGAAGAC-1	singlet	1	-2656.645975	-621.2272636	-2021.928305	EMD1
CAATTTCCATCACAGT-1	singlet	2	-976.1932834	-881.9833874	-395.3342811	EMD1
CAATTTCTCAGGAAAT-1	singlet	0	-531.3609017	-1838.081113	-1646.770801	EMD1
CAATTTCTCAGTGTGT-1	singlet	0	-107.557751	-298.6044273	-244.5100464	EMD1
CAATTTCTCATTTCGT-1	singlet	2	-545.7422164	-464.6439869	-216.4151661	EMD1
CAATTTCTCGCCTCTA-1	singlet	2	-1503.761081	-1409.014123	-687.8634938	EMD1
CAATTTCTCTACTATC-1	singlet	1	-2134.16194	-825.8236573	-1648.440625	EMD1
CAATTTCTCTGCATAG-1	singlet	2	-448.162238	-402.0396289	-217.8901322	EMD1
CACAACAAGGAAAGGT-1	singlet	0	-700.7126258	-2350.365624	-2135.200332	EMD1
CACAACAAGGTAGCCA-1	singlet	1	-365.6376726	-87.34012641	-277.2797452	EMD1
CACAACAAGGTGAGAA-1	singlet	2	-408.750047	-271.0653087	-129.6504176	EMD1
CACAACAAGTGCACTT-1	singlet	2	-3040.765205	-3043.974683	-839.0168927	EMD1
CACAACACAAGAGCTG-1	singlet	0	-473.5750674	-1238.613996	-1189.334168	EMD1
CACAACAGTCTCCCTA-1	singlet	2	-588.2661609	-548.0887181	-246.259371	EMD1
CACACAAAGAGCAGCT-1	singlet	1	-1603.843242	-525.7728272	-1409.531547	EMD1
CACACAACAACGCCCA-1	singlet	2	-358.4867052	-270.1168912	-138.3098433	EMD1
CACACAACAGCCCAGT-1	singlet	2	-804.579712	-708.4386781	-335.2058468	EMD1
CACACAAGTCCATAGT-1	singlet	0	-265.9638122	-755.5030454	-758.8275283	EMD1
CACACAAGTGGAAATT-1	singlet	1	-2692.560308	-817.8360946	-2170.173614	EMD1
CACACAAGTGTGGTCC-1	singlet	2	-466.3049498	-390.4369933	-186.7709735	EMD1
CACACAAGTTAACCTG-1	singlet	2	-1958.738685	-1728.786564	-770.0304507	EMD1
CACACAAGTTGCCAAT-1	singlet	0	-34.85939397	-60.12280474	-398.3516008	EMD1
CACACAATCAGAGCAG-1	singlet	0	-4.816510026	-11.41212999	-67.5316513	EMD1
CACAGATAGAACTTCC-1	singlet	1	-9183.680252	-3115.554349	-7808.070459	EMD1
CACAGATAGAAGTCTA-1	singlet	2	-1656.017273	-1204.017399	-572.0346856	EMD1
CACAGATAGGACAGTC-1	singlet	1	-4351.928906	-1126.106979	-3292.115659	EMD1
CACAGATAGGCCCACT-1	singlet	1	-705.8011873	-249.6795769	-506.4465195	EMD1
CACAGATCAACATCGT-1	singlet	0	-481.1643741	-1217.603406	-1226.894434	EMD1
CACAGATCACTCAGAT-1	singlet	2	-4369.646037	-3378.689304	-1373.098039	EMD1
CACAGATGTGCGACAA-1	singlet	2	-303.7918033	-326.0257317	-164.3955115	EMD1
CACAGATGTTCTTGTT-1	singlet	0	-179.7785218	-481.636695	-429.069099	EMD1
CACAGATTCAGCTGAT-1	singlet	2	-453.6928287	-393.6934783	-171.10128	EMD1
CACAGATTCTACTTCA-1	singlet	1	-3031.263514	-1035.719755	-2533.114933	EMD1
CACAGGCAGCAATAGT-1	singlet	2	-1696.874369	-1528.282867	-712.9990783	EMD1
CACAGGCAGGCAATGC-1	singlet	2	-4618.71055	-3839.916752	-1339.737452	EMD1
CACAGGCCACGAGAAC-1	singlet	2	-627.8594982	-562.0959367	-261.1654112	EMD1
CACAGGCCAGAGTCAG-1	singlet	2	-379.8089599	-280.9200191	-134.1409618	EMD1
CACAGGCCATTGTGCA-1	singlet	0	-1053.847887	-2926.751941	-2963.416238	EMD1
CACAGGCGTATGGTTC-1	singlet	0	-218.2315145	-558.3599817	-549.3260501	EMD1
CACAGGCTCCATCTGC-1	singlet	2	-343.6381577	-246.4410212	-145.9198123	EMD1

CACATGAAGAGTTCGG-1	singlet	2	-912.2797743	-669.9151319	-272.1845784	EMD1
CACATGAAGCCTCTCT-1	singlet	2	-1024.43137	-809.6198091	-384.0735597	EMD1
CACATGAAGGGAGGCA-1	singlet	0	-515.2742722	-1407.092937	-1420.369911	EMD1
CACATGAAGTACAGAT-1	singlet	0	-318.7049417	-900.1978509	-875.1061439	EMD1
CACATGAAGTGCTCGC-1	singlet	2	-169.484277	-223.2936713	-73.37927821	EMD1
CACATGACACATGTTG-1	singlet	1	-1819.577228	-486.0688396	-1433.593441	EMD1
CACATGACAGCGTGCT-1	singlet	1	-1258.051059	-386.0026628	-1026.429707	EMD1
CACATGACAGTGACCC-1	singlet	2	-2892.831592	-2264.665845	-1099.541179	EMD1
CACATGACATAACCCA-1	singlet	1	-5370.707464	-1879.289779	-4388.248732	EMD1
CACATGAGTGATTCTG-1	singlet	2	-1140.592883	-1060.469264	-353.3871162	EMD1
CACATGAGTTTCGGCG-1	singlet	2	-1786.273048	-1646.750348	-714.156466	EMD1
CACATGATCAGGAACG-1	singlet	2	-159.4533657	-168.7531762	-90.39685612	EMD1
CACCAAAAGACAACTA-1	singlet	2	-271.1798805	-273.7112302	-129.5161265	EMD1
CACCAAAAGCTACGTT-1	singlet	1	-448.2537629	-176.0904641	-517.5219166	EMD1
CACCAAAAGTACTGTC-1	singlet	2	-837.9264241	-750.7099891	-355.6777258	EMD1
CACCAAACAATTCTCT-1	singlet	1	-1684.387853	-510.7468108	-1336.772904	EMD1
CACCAAACACATCCCT-1	singlet	2	-736.65987	-708.6870341	-328.0054081	EMD1
CACCAAACACCGAATT-1	singlet	2	-332.403455	-330.0604675	-171.793014	EMD1
CACCAAACAGATACTC-1	singlet	2	-798.9839659	-704.3939055	-358.2542068	EMD1
CACCAAACATGAATAG-1	singlet	2	-1176.141188	-1026.633582	-437.1146181	EMD1
CACCAAAGTAGAGTTA-1	singlet	2	-5029.026945	-3911.007112	-1686.925161	EMD1
CACCAAATCACCTACC-1	singlet	2	-1160.096006	-1004.905489	-460.3958621	EMD1
CACCAAATCAGTGATC-1	singlet	0	-285.0254865	-970.8060134	-1204.339966	EMD1
CACCAAATCCCAGCGA-1	singlet	2	-1126.56572	-910.8159037	-358.7283315	EMD1
CACCAAATCCCTCAAC-1	singlet	2	-1778.207934	-1579.894785	-647.8164473	EMD1
CACCAAATCGCCGATG-1	singlet	0	-166.7019386	-454.4623919	-574.9399296	EMD1
CACCGTTAGGTAACTA-1	singlet	1	-6766.658608	-1904.041193	-4826.474101	EMD1
CACCGTTGTATCGATC-1	singlet	2	-460.7585809	-371.7196386	-203.6012941	EMD1
CACCGTTTCACACCCT-1	singlet	1	-8627.000076	-2563.832619	-6854.957121	EMD1
CACGAATCATGTTCAG-1	doublet	0/2	-835.7340052	-1216.473951	-880.5814584	EMD1
CACGAATGTCGTTATG-1	singlet	2	-11622.1776	-9040.294525	-3400.90956	EMD1
CACGAATGTTCCAGGC-1	singlet	1	-2663.353658	-1226.888385	-2112.817655	EMD1
CACGAATTCGATGCTA-1	singlet	2	-664.3689908	-584.4344774	-251.6983568	EMD1
CACGAATTCGCACGGT-1	singlet	0	-589.315296	-1012.333487	-935.2856041	EMD1
CACGAATTCGCCTAGG-1	singlet	2	-444.5778061	-388.1069531	-187.452062	EMD1
CACGAATTCTACTATC-1	singlet	0	-3242.795631	-10841.83416	-10411.69901	EMD1
CACGAATTCTATCGCC-1	unassigned	0/2	-14.18039313	-22.03526929	-23.62247736	EMD1
CACGAATTCTCGCTTG-1	singlet	1	-1121.583212	-385.2334511	-835.2045776	EMD1
CACGGGTAGCGATGAC-1	singlet	0	-241.605803	-945.185036	-1051.243647	EMD1
CACGGGTAGTCAGAGC-1	doublet	0/2	-42.95581877	-96.31716195	-58.24046112	EMD1
CACGGGTAGTTTAGGA-1	singlet	1	-235.0765491	-89.94683948	-200.5886551	EMD1
CACGGGTCAACGGGTA-1	singlet	1	-1654.498862	-495.4372671	-1372.919644	EMD1
CACGGGTCACGAGGTA-1	singlet	1	-1645.024723	-589.8689851	-1338.333485	EMD1
CACGGGTCACTATCGA-1	singlet	1	-1980.06047	-631.252181	-1699.159233	EMD1
CACGGGTCATGACCCG-1	singlet	2	-171.8702678	-154.3670393	-52.90686606	EMD1
CACGGGTTCGAGTCCG-1	singlet	2	-2001.030958	-1685.534837	-716.3879023	EMD1
CACGGGTTCGCTAATG-1	singlet	1	-155.3312654	-54.06343354	-119.0082611	EMD1
CACGGGTTCGTAGTCA-1	singlet	0	-366.5228101	-1012.932012	-1042.89653	EMD1
CACGGGTTCTCCAAGA-1	singlet	0	-210.5365477	-620.430614	-612.8435491	EMD1
CACGTGGCAACCGCTG-1	singlet	0	-1040.534829	-3714.960322	-3469.151806	EMD1
CACGTGGGTATGCTTG-1	singlet	2	-3090.839039	-2543.519852	-933.8295258	EMD1
CACGTGGGTATTGCCA-1	singlet	1	-515.9998228	-173.8247239	-427.1296252	EMD1
CACGTGGGTTCGATTG-1	singlet	1	-1972.675924	-693.880083	-1668.987662	EMD1
CACGTGGTCACACCGG-1	singlet	2	-1921.72163	-1474.358189	-634.8824603	EMD1
CACGTTCAGACTTAAG-1	singlet	2	-3694.74201	-2918.155683	-951.3465605	EMD1
CACGTTCAGGAGAGGC-1	singlet	2	-477.5744619	-416.9347101	-205.3771054	EMD1
CACGTTCCAGAAGTGC-1	singlet	2	-269.7600546	-238.8236209	-116.9003783	EMD1
CACGTTCCATGTGCTA-1	singlet	2	-4260.731524	-3339.268984	-1361.752144	EMD1
CACGTTCGTTCAAGTC-1	singlet	2	-1037.171844	-946.2819003	-408.5697571	EMD1
CACGTTCGTTCTCGCT-1	singlet	1	-3196.349479	-789.9975665	-2161.447597	EMD1
CACGTTCGTTGCTGAT-1	singlet	0	-128.394243	-325.1360955	-362.6040341	EMD1
CACTAAGAGAAATTCG-1	singlet	2	-661.8214177	-552.6942193	-273.3116435	EMD1
CACTAAGAGGAAGAAC-1	singlet	0	-293.8370052	-910.3489209	-778.6782378	EMD1
CACTAAGAGGTGCAGT-1	singlet	0	-906.2756103	-2967.288532	-2877.038098	EMD1
CACTAAGCACCTGTCT-1	singlet	2	-649.2260554	-556.734838	-275.9570896	EMD1
CACTAAGCACTGATTG-1	singlet	1	-4174.869689	-748.6124007	-3416.196294	EMD1
CACTAAGGTATTAAGG-1	singlet	0	-152.6946476	-443.633739	-437.2778277	EMD1
CACTAAGGTTCGCGTG-1	singlet	0	-165.3555655	-667.6193678	-665.056605	EMD1
CACTAAGTCTGCGATA-1	singlet	2	-489.7215335	-437.4725038	-226.850613	EMD1
CACTGAAAGACTACGG-1	singlet	2	-579.9586095	-414.36846	-213.7863663	EMD1
CACTGAAAGAGGTTTA-1	singlet	1	-4117.462369	-1206.857995	-3482.660403	EMD1
CACTGAAAGAGTTGTA-1	singlet	2	-701.17626	-528.0604243	-286.4993274	EMD1
CACTGAAAGGTAAGTT-1	singlet	0	-871.5103809	-2339.571695	-2373.104828	EMD1
CACTGAACAATCAGCT-1	singlet	2	-1859.849606	-1544.916828	-609.8076594	EMD1
CACTGAACAATCCAGT-1	singlet	1	-608.2897364	-186.9505659	-522.1107513	EMD1
CACTGAACATGCGGTC-1	singlet	1	-983.4015681	-352.2578174	-829.3078799	EMD1
CACTGAAGTGCCGTTG-1	singlet	2	-562.4381784	-415.6271588	-229.6909503	EMD1
CACTGAAGTGTTACAC-1	singlet	2	-1023.600363	-968.4071412	-478.3185651	EMD1
CACTGAATCAAGTGGG-1	singlet	0	-424.4126565	-1275.890406	-1218.258791	EMD1
CACTGAATCCAATCTT-1	singlet	0	-647.897515	-1884.367672	-1916.515337	EMD1
CACTGAATCGACCAAT-1	singlet	0	-951.6020169	-2079.789078	-1913.997902	EMD1
CACTGAATCGCTTAAG-1	singlet	2	-495.3865897	-421.4332077	-234.5715249	EMD1
CACTGGGAGACGGTTG-1	singlet	0	-228.2138235	-795.5418669	-878.1800386	EMD1
CACTGGGCAATTCTTC-1	singlet	2	-689.4955626	-602.7852343	-293.9483626	EMD1
CACTGGGCACTTGGGC-1	singlet	2	-1055.581096	-936.009343	-439.5679007	EMD1
CACTGGGGTAGGTACG-1	singlet	1	-452.487513	-177.8258897	-505.9296399	EMD1
CACTGGGTCAACTCTT-1	singlet	0	-423.597286	-1420.905746	-1421.395777	EMD1
CACTGGGTCATTCGTT-1	singlet	2	-211.0961686	-131.5563687	-77.38716089	EMD1
CACTGGGTCTGTGTGA-1	singlet	0	-340.5450463	-762.3741346	-765.1285602	EMD1
CACTGTCAGGAACTCG-1	singlet	0	-414.156368	-1205.879485	-1115.376656	EMD1
CACTGTCAGTTCCATG-1	singlet	0	-164.013681	-463.0028762	-427.0247492	EMD1
CACTGTCCAATCGCAT-1	singlet	2	-576.5380126	-554.7593703	-213.4336814	EMD1
CACTGTCCACCTGCTT-1	singlet	1	-179.4623612	-67.72125556	-161.195349	EMD1
CACTGTCCAGAGTGAC-1	singlet	2	-1274.503943	-1010.147006	-424.7711142	EMD1
CACTGTCGTCACTTCC-1	singlet	1	-592.817748	-265.7503851	-586.5862625	EMD1
CACTGTCTCACTGTCC-1	singlet	2	-2431.371711	-1948.392296	-867.7797147	EMD1
CACTGTCTCCGTATAG-1	singlet	0	-242.2344034	-818.7252686	-856.5532976	EMD1
CACTGTCTCGCTCTAC-1	singlet	2	-201.9768565	-156.3715676	-92.99517126	EMD1
CACTTCGAGCATTGAA-1	singlet	2	-277.7131469	-224.1665315	-114.750216	EMD1
CACTTCGAGGGCAATC-1	singlet	2	-1306.882359	-1128.545861	-532.4037202	EMD1
CACTTCGAGGTCGCCT-1	singlet	2	-614.431471	-501.6092747	-226.4234002	EMD1
CACTTCGAGTAGTCCT-1	singlet	2	-123.6677234	-141.2063981	-57.53125992	EMD1
CACTTCGCAAATGATG-1	singlet	1	-4522.169944	-1531.023877	-4004.944056	EMD1
CACTTCGCACGACAAG-1	singlet	0	-261.7521081	-681.2186945	-739.0805231	EMD1
CACTTCGCAGGCTTGC-1	singlet	1	-1311.686888	-525.8184446	-1208.334146	EMD1
CACTTCGCATACTGTG-1	singlet	1	-1117.312502	-422.4641333	-952.1777882	EMD1
CACTTCGGTACGATTC-1	singlet	0	-45.30285208	-160.3041711	-115.9781365	EMD1
CACTTCGGTCTCCCTA-1	doublet	2/0	-885.9073898	-1053.490494	-777.1599599	EMD1
CACTTCGGTGAGATAT-1	singlet	2	-594.7166581	-520.969233	-233.291939	EMD1
CACTTCGTCTAGGCCG-1	singlet	1	-1519.372454	-428.6066162	-1160.535571	EMD1

CACTTCGTCTGGACCG-1	singlet	0	-42.31052102	-136.3900062	-109.3975065	EMD1
CAGAGCCAGTCACTCA-1	singlet	2	-264.5972724	-256.157468	-114.7746999	EMD1
CAGAGCCCAACCAATC-1	singlet	2	-3031.071031	-2158.990668	-847.9340415	EMD1
CAGAGCCGTATCATGC-1	singlet	2	-888.4153902	-709.6101717	-264.9422309	EMD1
CAGAGCCGTATCTTCT-1	singlet	0	-300.7608371	-1092.828915	-1048.28886	EMD1
CAGAGCCGTTGACTGT-1	singlet	0	-156.8265381	-540.5819213	-519.1843837	EMD1
CAGAGCCTCAAAGGTA-1	singlet	2	-349.6003776	-282.382045	-136.4265196	EMD1
CAGATACAGGCTAAAT-1	singlet	0	-1704.038175	-5306.319925	-5282.277484	EMD1
CAGATACAGTCATGCT-1	singlet	0	-41.50148006	-153.5344375	-155.7613936	EMD1
CAGATACCAGGCACAA-1	singlet	1	-1422.560632	-532.6219697	-1537.487049	EMD1
CAGATACGTCAACGCC-1	singlet	2	-268.5803263	-236.7989331	-120.0433276	EMD1
CAGATACTCCGTGGGT-1	singlet	0	-337.6372877	-944.9537857	-863.3993999	EMD1
CAGATACTCGCTGCGA-1	singlet	1	-346.9425667	-115.2072835	-284.0881627	EMD1
CAGATACTCGGCCAAC-1	singlet	0	-764.1346911	-2136.87838	-1962.437058	EMD1
CAGATACTCTACACAG-1	singlet	0	-1091.192271	-2927.140574	-3065.827826	EMD1
CAGATACTCTACTGAG-1	singlet	2	-588.3691435	-481.1536241	-189.2566718	EMD1
CAGATCAAGATAGTCA-1	singlet	1	-4775.648963	-1243.095624	-4324.612759	EMD1
CAGATCAAGGCTATCT-1	singlet	2	-3437.379844	-3024.035135	-1075.26745	EMD1
CAGATCACACATACTG-1	singlet	2	-4893.101344	-3995.43039	-1664.047115	EMD1
CAGATCACAGACCGCT-1	singlet	0	-24.0708384	-147.8061619	-126.2666543	EMD1
CAGATCACAGAGATGC-1	singlet	2	-2720.520321	-2139.38267	-980.3575145	EMD1
CAGATCACATGGAATA-1	singlet	2	-457.6593011	-398.7208983	-177.597872	EMD1
CAGATCAGTTTATGCG-1	singlet	2	-2183.485245	-1700.133621	-800.5886396	EMD1
CAGATCATCGGTCGGT-1	singlet	2	-246.5240258	-178.6230564	-82.40686242	EMD1
CAGATCATCGTGGGAA-1	singlet	2	-576.9669396	-524.2498269	-221.8017341	EMD1
CAGATCATCTCAACGA-1	singlet	2	-399.0944316	-331.692434	-163.9542593	EMD1
CAGATTGAGAAACCAT-1	singlet	2	-870.9835763	-737.5028634	-333.2335859	EMD1
CAGATTGAGACTGTTC-1	singlet	2	-1802.948155	-1522.641398	-685.0526948	EMD1
CAGATTGAGGTCCTGC-1	singlet	0	-326.2330322	-1251.258825	-1056.231199	EMD1
CAGATTGAGTAGCTCT-1	singlet	2	-977.8208444	-825.2327825	-355.1523881	EMD1
CAGATTGGTAGAAACT-1	singlet	0	-864.5094247	-3995.846611	-3423.799569	EMD1
CAGATTGGTGAGATAT-1	singlet	1	-164.8238009	-55.72566099	-149.7893244	EMD1
CAGATTGGTGCCTATA-1	singlet	2	-414.1148494	-303.9884809	-155.7850413	EMD1
CAGATTGGTGTTCGTA-1	singlet	0	-323.6369285	-856.7408633	-874.8909674	EMD1
CAGATTGTCTCGGTAA-1	singlet	2	-469.9622197	-369.8713905	-193.7093399	EMD1
CAGATTGTCTTCCACG-1	singlet	2	-218.8896194	-213.2409645	-113.9260768	EMD1
CAGCAATAGACCTCAT-1	singlet	2	-677.5845828	-521.3025911	-284.2519689	EMD1
CAGCAATAGGGAGGTG-1	singlet	2	-464.1017776	-420.2812905	-208.1051837	EMD1
CAGCAATAGGTAGTAT-1	singlet	0	-1193.521015	-3108.719543	-3266.111559	EMD1
CAGCAATAGTAGCTCT-1	singlet	0	-293.4614608	-833.6339555	-880.8565037	EMD1
CAGCAATCAAACTCGT-1	singlet	0	-268.8501949	-770.0840388	-695.4556187	EMD1
CAGCAATGTAGTGTGG-1	singlet	0	-2.563136009	-8.594234077	-9.175983579	EMD1
CAGCAATTCAACGAGG-1	singlet	2	-217.6726248	-215.2903514	-107.8036081	EMD1
CAGCAATTCAGCTCTC-1	singlet	0	-639.3880543	-2361.194806	-2262.363752	EMD1
CAGCAATTCCTCACTG-1	singlet	2	-901.4090489	-575.0224002	-262.7182432	EMD1
CAGCAATTCTTCGTAT-1	singlet	2	-266.0746127	-203.3215575	-89.07804339	EMD1
CAGCACGAGTGATCGG-1	singlet	0	-42.33229828	-232.5727579	-172.0302474	EMD1
CAGCACGCACGCGCAT-1	singlet	2	-600.4670576	-481.4064012	-236.5415168	EMD1
CAGCACGCAGATACCT-1	singlet	1	-171.0775737	-48.62793914	-104.7958603	EMD1
CAGCACGGTCACTCTC-1	singlet	2	-657.3530584	-583.174044	-270.1901124	EMD1
CAGCACGGTCCAAATC-1	singlet	2	-910.7156785	-680.1626633	-277.1360779	EMD1
CAGCACGTCGCCGAGT-1	singlet	2	-396.6771545	-426.9636911	-168.0626279	EMD1
CAGCACGTCTGAGGTT-1	singlet	0	-1381.744406	-4702.199042	-4430.523715	EMD1
CAGCACGTCTTTCCAA-1	singlet	2	-2184.369407	-2167.775934	-851.2528668	EMD1
CAGCAGCAGCGTCTCG-1	singlet	0	-155.0812166	-585.0802145	-602.2677641	EMD1
CAGCAGCAGTCTCGTA-1	singlet	2	-2995.296468	-2821.724727	-908.1514683	EMD1
CAGCAGCCAGAGTTCT-1	singlet	1	-925.0268645	-267.1796895	-693.1173922	EMD1
CAGCAGCCAGTAGGAC-1	singlet	0	-1050.346672	-3195.516008	-3100.753072	EMD1
CAGCAGCTCCATGATG-1	singlet	1	-3182.819344	-1118.957114	-2679.022859	EMD1
CAGCAGCTCGCACGGT-1	singlet	2	-1070.194973	-911.2803258	-414.8589026	EMD1
CAGCAGCTCTGTCAGA-1	singlet	0	-636.4200746	-2212.70603	-1903.758737	EMD1
CAGCCAGAGCGCTTCG-1	singlet	2	-844.9274085	-664.325141	-320.3699587	EMD1
CAGCCAGAGTAGGAAG-1	singlet	0	-229.2800651	-639.9516837	-608.6337499	EMD1
CAGCCAGAGTGATTCC-1	unassigned	2	-2.794150239	-18.16850154	-2.135713335	EMD1
CAGCCAGTCCGTGGGT-1	singlet	1	-368.4537596	-137.0456078	-331.1932111	EMD1
CAGCCAGTCCTAGAGT-1	singlet	2	-660.1169042	-566.0731356	-209.6557617	EMD1
CAGCGTGAGAATTGCA-1	singlet	2	-441.0639779	-384.4931468	-199.921836	EMD1
CAGCGTGAGGTAGCAC-1	singlet	2	-308.0588087	-256.0954528	-130.1356525	EMD1
CAGCGTGAGTCGTCTA-1	singlet	0	-187.8799509	-487.4019659	-504.7450251	EMD1
CAGCGTGCATCCGGCA-1	singlet	2	-1324.397265	-1142.788393	-519.4319324	EMD1
CAGCGTGGTACACGTT-1	singlet	1	-9359.104116	-2020.927147	-7205.8141	EMD1
CAGCGTGGTGCGGCTT-1	singlet	1	-4921.657708	-1259.056657	-4017.437263	EMD1
CAGCGTGTCACCTTAT-1	singlet	2	-320.4258471	-324.578738	-112.7407959	EMD1
CAGCGTGTCACTGTCC-1	singlet	1	-122.4909939	-42.45688778	-87.65793362	EMD1
CAGCGTGTCTACTGAG-1	singlet	1	-2062.359325	-582.8663205	-1534.310383	EMD1
CAGCGTGTCTATTCGT-1	singlet	1	-181.5608057	-38.10646751	-92.338037	EMD1
CAGGCCAAGATGCTGG-1	singlet	1	-2178.686598	-704.0725784	-1743.704001	EMD1
CAGGCCAAGCCTGCCA-1	singlet	2	-307.4031473	-272.0351789	-130.9577921	EMD1
CAGGCCAAGGCTCTCG-1	singlet	1	-3804.047371	-771.6162081	-3124.214838	EMD1
CAGGCCAAGGTAACTA-1	singlet	1	-65.50023259	-30.49667587	-74.56498928	EMD1
CAGGCCAAGGTCTGGA-1	singlet	2	-929.0862748	-777.9427686	-358.6013754	EMD1
CAGGCCAAGTAAGCAT-1	singlet	2	-365.7988003	-318.2555207	-136.7027207	EMD1
CAGGCCAAGTATAGGT-1	singlet	0	-276.1024835	-794.4997661	-835.9901157	EMD1
CAGGCCACAAGAATGT-1	singlet	2	-431.8097782	-313.6726851	-144.1453454	EMD1
CAGGCCACAATAGGGC-1	singlet	0	-2026.092318	-7139.082657	-6887.789468	EMD1
CAGGCCAGTCCCTCAT-1	singlet	0	-213.3195032	-600.2876876	-486.0734588	EMD1
CAGGCCATCATTTGCT-1	singlet	0	-1580.759864	-5147.179321	-4254.5059	EMD1
CAGGCCATCCCATAAG-1	singlet	0	-185.3570033	-488.1792031	-538.0984668	EMD1
CAGGGCTAGCCTCTTC-1	singlet	2	-630.376926	-594.1730975	-242.845092	EMD1
CAGGGCTAGTGGCCTC-1	singlet	0	-324.0643459	-1035.767716	-1017.602842	EMD1
CAGGGCTCATCATTGG-1	singlet	0	-610.9748052	-1927.986397	-1972.282993	EMD1
CAGGGCTGTAACACCT-1	singlet	2	-1488.493581	-1135.09862	-513.3880471	EMD1
CAGGGCTGTGACCGAA-1	singlet	2	-258.0330729	-211.477591	-121.8333599	EMD1
CAGGGCTGTTATCTGG-1	singlet	1	-3763.397752	-1143.065905	-2957.01286	EMD1
CAGGGCTTCAGAGTTC-1	singlet	1	-530.075338	-207.7344414	-492.8644265	EMD1
CAGGGCTTCATTATCC-1	singlet	1	-7613.735466	-1906.700597	-5379.4121	EMD1
CAGGGCTTCCACTTTA-1	singlet	2	-402.5742819	-334.0710817	-154.2571601	EMD1
CAGGGCTTCGGTAACT-1	singlet	0	-270.9022172	-1151.169141	-1055.47949	EMD1
CAGGTATAGCTAGATA-1	singlet	1	-1498.691884	-596.0636284	-1230.800973	EMD1
CAGGTATAGGGCTTCC-1	doublet	1/0	-3371.145169	-1568.72649	-3234.119928	EMD1
CAGGTATAGTTCATGC-1	singlet	0	-983.8970205	-2808.916653	-2730.254143	EMD1
CAGGTATCAACACACT-1	singlet	2	-601.0823805	-442.2924976	-234.344852	EMD1
CAGGTATGTCAAGTTC-1	doublet	1/0	-3587.425083	-3567.469134	-5361.424796	EMD1
CAGGTATGTTCTATCT-1	singlet	2	-489.2696369	-396.3901649	-207.3100774	EMD1
CAGGTATGTTGGGAAC-1	singlet	2	-423.207605	-322.4504133	-171.1507696	EMD1
CAGGTATTCGGATAAA-1	singlet	1	-363.2570571	-149.743748	-287.0262624	EMD1
CAGGTATTCGTGACTA-1	singlet	1	-2267.834819	-651.3911119	-1732.847558	EMD1
CAGGTATTCTCTGGTC-1	singlet	0	-1568.350008	-4371.377741	-4732.12401	EMD1
CAGTGCGAGCACCCAC-1	doublet	0/2	-1493.137429	-3094.657443	-2199.186813	EMD1
CAGTGCGAGGATATGT-1	singlet	2	-293.666898	-256.4571945	-129.2429268	EMD1

CAGTGCGAGTCGGCAA-1	singlet	2	-147.5278247	-103.1865718	-44.32975379	EMD1
CAGTGCGCAAAGGCGT-1	singlet	1	-5695.477657	-1838.992634	-4671.955285	EMD1
CAGTGCGCACCCTCTA-1	singlet	2	-512.1567416	-468.2728679	-236.0774808	EMD1
CAGTGCGCACGACGCT-1	doublet	0/2	-1809.749027	-2785.395945	-2373.474281	EMD1
CAGTGCGCAGCTTTCC-1	singlet	2	-684.3629478	-588.6620182	-240.3156875	EMD1
CAGTGCGGTACAGTTC-1	singlet	0	-169.5725769	-399.7339217	-488.6448952	EMD1
CAGTGCGGTTTCGATG-1	singlet	0	-434.310393	-1549.384679	-1253.762006	EMD1
CAGTGCGTCCGCGAGT-1	singlet	1	-334.9013173	-116.8646677	-240.3242986	EMD1
CAGTTAGAGGAGAATG-1	singlet	2	-252.4460926	-206.4502801	-102.5364046	EMD1
CAGTTAGAGTCTCCTC-1	singlet	1	-2869.740351	-831.0971804	-2453.774825	EMD1
CAGTTAGCAAGCACCC-1	singlet	0	-143.4258449	-353.451699	-351.7764976	EMD1
CAGTTAGCAGTGCCTG-1	singlet	2	-498.5761049	-506.1958214	-216.4340865	EMD1
CAGTTAGGTTCTTGCC-1	singlet	1	-5377.516658	-1873.627979	-4494.697648	EMD1
CAGTTAGGTTGTATGC-1	singlet	0	-554.0810966	-2076.007183	-1833.273804	EMD1
CAGTTAGGTTTAAGGA-1	singlet	2	-737.3347033	-640.7779787	-272.6648554	EMD1
CAGTTAGTCCATCAGA-1	singlet	2	-683.8881841	-498.742637	-239.4126625	EMD1
CAGTTCCAGACATCCT-1	singlet	0	-570.8295963	-1742.726207	-1684.393562	EMD1
CAGTTCCAGCTGCCAC-1	singlet	2	-700.9045987	-564.5781896	-309.9230583	EMD1
CAGTTCCAGTCGGCCT-1	singlet	2	-717.7624004	-593.3932535	-258.6172971	EMD1
CAGTTCCCACAGCGCT-1	singlet	0	-453.4641395	-1672.326842	-1383.850917	EMD1
CAGTTCCCAGCAAGAC-1	singlet	2	-4553.323584	-3993.506868	-966.938919	EMD1
CAGTTCCCAGGTTCAT-1	singlet	2	-393.2516946	-320.0376462	-151.203029	EMD1
CAGTTCCGTAGAGCTG-1	singlet	2	-1782.471508	-1763.657638	-681.157678	EMD1
CAGTTCCGTGGACCAA-1	singlet	0	-931.8956631	-2664.070966	-2599.027152	EMD1
CAGTTCCTCGACCTAA-1	singlet	0	-286.5240537	-715.0583401	-856.9886697	EMD1
CATAAGCCACACAGAG-1	singlet	1	-3491.978591	-1286.06286	-2766.513036	EMD1
CATAAGCCAGCCGGTT-1	singlet	1	-1522.881121	-548.6419731	-1160.670194	EMD1
CATAAGCGTATCGCGC-1	doublet	45323	-29.3990558	-25.07184405	-23.21988775	EMD1
CATAAGCGTGCAACAG-1	singlet	0	-1213.430514	-3911.660904	-4006.318269	EMD1
CATAAGCTCACTGCTC-1	singlet	0	-484.7022179	-1461.170151	-1365.308967	EMD1
CATAAGCTCCGATAGT-1	singlet	0	-387.9350529	-979.0236511	-964.2550478	EMD1
CATAAGCTCCGGCAAC-1	singlet	0	-637.7811314	-1623.038369	-1534.402185	EMD1
CATACAGAGGCCATAG-1	singlet	1	-3532.399804	-1148.130476	-2890.148489	EMD1
CATACAGAGGCGTCCT-1	singlet	0	-1162.801191	-3932.601267	-4151.437007	EMD1
CATACAGAGGTGATCG-1	singlet	0	-287.4739714	-799.6912191	-723.2864991	EMD1
CATACAGCAGCGTACC-1	singlet	2	-593.3502976	-449.1623077	-247.2352678	EMD1
CATACAGCATATCTCT-1	singlet	0	-302.0352593	-757.3718278	-969.8607049	EMD1
CATACAGCATCCAACA-1	singlet	1	-2697.366633	-904.396722	-2244.998431	EMD1
CATACAGGTATCTCGA-1	singlet	0	-285.8319429	-881.8009605	-759.1562846	EMD1
CATACAGGTTCCTAAG-1	singlet	0	-3.704303031	-8.453344793	-9.211560061	EMD1
CATACAGTCCCGATCT-1	singlet	0	-987.1744953	-2776.356726	-2729.398128	EMD1
CATACCCAGACCCGT-1	singlet	0	-1372.031197	-3983.970303	-3670.563389	EMD1
CATACCCCAGGATCTT-1	singlet	0	-591.6573104	-3719.912568	-3039.179356	EMD1
CATACCCGTCGACTTA-1	singlet	2	-659.2584635	-493.5728668	-238.7119323	EMD1
CATACCCGTTACACTG-1	singlet	1	-2114.563994	-644.084566	-1910.325819	EMD1
CATACCCGTTGATGTC-1	singlet	2	-533.0447653	-417.4093388	-229.9547298	EMD1
CATACCCTCACTCACC-1	singlet	0	-290.7715131	-895.2174444	-844.8403425	EMD1
CATACCCTCATCGGGC-1	singlet	1	-1640.636354	-647.9138328	-1358.076844	EMD1
CATACCCTCATTACCT-1	singlet	2	-432.1166405	-332.1881456	-168.1373061	EMD1
CATACCCTCCTTATCA-1	singlet	0	-1489.645713	-4684.416445	-4573.728145	EMD1
CATACCCTCGAGAAGC-1	singlet	2	-397.1705684	-349.6013505	-159.8522702	EMD1
CATACCCTCGCACGGT-1	doublet	45293	-3189.206318	-1422.734203	-2294.90701	EMD1
CATACCCTCGTCAAAC-1	singlet	0	-606.9801267	-1618.061435	-1481.693184	EMD1
CATACTTAGAGAGGTA-1	singlet	2	-3636.9942	-3375.819167	-974.3423399	EMD1
CATACTTAGTCTAGCT-1	singlet	1	-1471.095785	-542.7786296	-1175.144996	EMD1
CATACTTAGTGAGTTA-1	singlet	0	-643.3381074	-2308.492232	-2432.420562	EMD1
CATACTTCACTAAACC-1	singlet	0	-3169.96715	-9349.243724	-9255.164762	EMD1
CATACTTTCCTCAGAA-1	singlet	0	-1004.524707	-3012.065912	-2946.250113	EMD1
CATACTTTCTACTGAG-1	singlet	0	-319.5366875	-1147.725665	-1139.336499	EMD1
CATAGACAGCACTCAT-1	singlet	2	-671.5964821	-568.8771947	-277.1384963	EMD1
CATAGACCAACTTCTT-1	singlet	2	-987.5696183	-782.4290724	-342.7116672	EMD1
CATAGACCAATAAGGT-1	singlet	1	-724.3735088	-240.2708248	-528.1343669	EMD1
CATAGACGTGTCCACG-1	singlet	1	-1911.234377	-538.3939395	-1583.583545	EMD1
CATAGACTCCGTTTCG-1	singlet	1	-1135.232762	-396.9209132	-876.4768989	EMD1
CATAGACTCTGAGGTT-1	singlet	2	-311.5060176	-283.8267508	-112.5724446	EMD1
CATCAAGAGTGTTCCA-1	singlet	1	-214.528393	-93.60973092	-164.9246736	EMD1
CATCAAGCAATTAGGA-1	singlet	2	-702.1945586	-587.6857517	-225.2978419	EMD1
CATCAAGCATACTGAC-1	singlet	2	-494.0085886	-447.146401	-220.9807093	EMD1
CATCAAGCATCAGCTA-1	singlet	2	-1194.247634	-866.399259	-383.2278982	EMD1
CATCAAGGTCACTAGT-1	singlet	2	-359.5378151	-273.1352109	-144.2663861	EMD1
CATCAAGGTCACTCTC-1	singlet	0	-739.6765048	-1903.809182	-1979.851248	EMD1
CATCAAGGTTTATGCG-1	singlet	2	-777.7993005	-708.8703534	-337.3925406	EMD1
CATCAAGTCATTGAGC-1	singlet	1	-2317.058418	-738.0423898	-1689.473275	EMD1
CATCAAGTCGCTGATA-1	singlet	2	-1666.712795	-1288.416526	-644.7390487	EMD1
CATCCACAGACCATTC-1	singlet	2	-540.4521065	-483.5044988	-208.7770534	EMD1
CATCCACAGACGCAGT-1	singlet	2	-202.0498668	-170.814079	-55.23105539	EMD1
CATCCACAGTCAACAA-1	singlet	2	-4510.461039	-3507.513987	-1657.378736	EMD1
CATCCACAGTGGTTAA-1	singlet	2	-5657.051967	-4407.36637	-1921.399482	EMD1
CATCCACCAAAGGCAC-1	singlet	0	-499.2719451	-1848.263143	-1459.565386	EMD1
CATCCACCAAATTAGG-1	singlet	1	-6823.982221	-2471.018435	-5319.544059	EMD1
CATCCACCAGTCCCGA-1	singlet	2	-458.542206	-344.0194043	-181.7837717	EMD1
CATCCACCATGCACTA-1	singlet	0	-1517.854549	-5763.818933	-4759.28461	EMD1
CATCCACCATGTTCGA-1	singlet	2	-3249.810878	-2716.071918	-990.7563527	EMD1
CATCCACGTCACAGTT-1	singlet	2	-440.6983844	-431.5914687	-191.5456955	EMD1
CATCCACGTCGAGATG-1	singlet	2	-471.3938631	-405.1000072	-188.5038549	EMD1
CATCCACGTCTGTCCT-1	singlet	1	-3469.054131	-1045.768077	-2622.602715	EMD1
CATCCACGTCTTCTAT-1	singlet	1	-3327.176453	-1621.692027	-2503.938882	EMD1
CATCCACTCAAGAGGC-1	singlet	1	-604.5989454	-266.5994307	-635.3875725	EMD1
CATCCACTCAGACAAA-1	singlet	1	-20.56275223	-6.949974998	-10.79665141	EMD1
CATCCACTCCGGACGT-1	singlet	2	-1865.785612	-1363.829189	-687.161229	EMD1
CATCCACTCGCCAGAC-1	singlet	0	-435.6520096	-1285.289828	-1176.580993	EMD1
CATCCACTCGCGTGAC-1	singlet	2	-43.21385876	-46.26321885	-23.47323689	EMD1
CATCCCAAGCGTGTTT-1	singlet	2	-328.2067852	-262.7134587	-154.2614945	EMD1
CATCCCAAGCTATCCA-1	singlet	0	-487.2135059	-1353.724181	-1302.135378	EMD1
CATCCCAAGTGTAGAT-1	singlet	0	-315.9616556	-1022.867115	-901.2379033	EMD1
CATCCCAAGTGTGTTC-1	singlet	2	-605.4405899	-570.4487359	-229.2008313	EMD1
CATCCCACACTGCATA-1	singlet	1	-2446.071464	-721.8375704	-1743.682053	EMD1
CATCCCACAGATACTC-1	singlet	0	-781.8472037	-2284.043059	-1992.122388	EMD1
CATCCCACATCGTTCC-1	singlet	1	-6091.816835	-1931.186301	-4673.286169	EMD1
CATCCCAGTCTGCGCA-1	singlet	2	-524.5635247	-429.6030977	-210.9703484	EMD1
CATCCCAGTGTTATCG-1	singlet	0	-183.1238223	-569.0846034	-578.820465	EMD1
CATCCCAGTTTGATCG-1	singlet	2	-736.7853436	-581.4953311	-255.7868492	EMD1
CATCCCATCGCGCCAA-1	singlet	0	-527.8911298	-1721.199885	-1561.710103	EMD1
CATCCCATCGGATTAC-1	singlet	2	-518.1680968	-448.6389145	-203.6262911	EMD1
CATCCGTAGCTAATGA-1	singlet	0	-244.4284752	-577.3295209	-543.5110331	EMD1
CATCCGTCAACCGTGC-1	singlet	2	-1437.423077	-1242.45599	-618.1208542	EMD1
CATCCGTCACTCCACT-1	singlet	2	-728.7093018	-580.3167203	-291.8347376	EMD1
CATCCGTCATTGGATC-1	singlet	0	-240.4872649	-657.5988201	-588.1337399	EMD1
CATCCGTGTCAGTCTA-1	singlet	1	-890.0236085	-294.7562966	-654.9524267	EMD1
CATCCGTGTGCTCTCT-1	singlet	2	-637.2726747	-540.1402814	-230.0789862	EMD1

CATCCGTTCACGAGGA-1	singlet	1	-2866.991482	-988.0769534	-2318.081767	EMD1
CATCGCTAGTGTTGAA-1	singlet	2	-3086.315364	-2364.329176	-882.0168899	EMD1
CATCGCTCAATTTCGG-1	singlet	2	-162.104825	-95.14166425	-36.01053426	EMD1
CATCGCTCATGACTTG-1	singlet	0	-821.112376	-2099.120111	-1982.427434	EMD1
CATCGCTGTATCAGCT-1	singlet	0	-455.0872482	-1221.37365	-1161.921779	EMD1
CATCGCTGTCTAACTG-1	singlet	0	-1396.461588	-4114.948435	-4073.822656	EMD1
CATCGCTGTTTCGTAG-1	singlet	2	-1174.540267	-1010.155925	-473.8696933	EMD1
CATCGCTTCGTACACA-1	singlet	2	-1818.566334	-1800.647998	-621.9329803	EMD1
CATCGCTTCTCCTGCA-1	singlet	2	-1441.963854	-1353.898379	-486.4319929	EMD1
CATCGGGAGCGGTAGT-1	singlet	1	-269.3043392	-114.0459572	-251.0761564	EMD1
CATCGGGAGTGAGTGC-1	singlet	0	-357.7684727	-1121.028375	-1064.894329	EMD1
CATCGGGCAGAACTTC-1	singlet	1	-349.7445062	-169.2439273	-310.1540929	EMD1
CATCGGGCAGGCGAAT-1	singlet	2	-514.1609638	-413.849795	-191.3682526	EMD1
CATCGGGGTCAAACGG-1	singlet	0	-358.5240303	-1101.15951	-937.3276137	EMD1
CATCGGGGTTATCCAG-1	singlet	2	-2262.980492	-1757.852553	-829.8893394	EMD1
CATCGGGGTTCTCACC-1	singlet	2	-1240.760579	-875.9718712	-434.2902573	EMD1
CATCGGGGTTGTGCAT-1	singlet	1	-3613.581578	-991.4694578	-2830.18722	EMD1
CATCGGGTCATTCCTA-1	singlet	2	-527.4270034	-448.1814417	-200.9505087	EMD1
CATCGGGTCGGAAGGT-1	singlet	0	-293.8682136	-950.5551024	-963.8039158	EMD1
CATCGTCAGCTCTATG-1	singlet	0	-273.3685172	-664.6352061	-780.0728363	EMD1
CATCGTCCAGATGCGA-1	singlet	2	-365.175688	-293.6039112	-137.9920671	EMD1
CATCGTCCAGCTAACT-1	singlet	1	-414.6689236	-167.4345638	-373.77579	EMD1
CATCGTCGTATCAGCT-1	singlet	2	-2628.778857	-2067.606786	-886.7193318	EMD1
CATCGTCGTCCCTCAT-1	singlet	0	-490.5821556	-1706.886865	-1515.259085	EMD1
CATCGTCTCCTGGGAC-1	singlet	1	-3383.484887	-1229.87413	-2682.368737	EMD1
CATCGTCTCTAGTGTG-1	singlet	0	-1749.643967	-6588.695214	-6060.438679	EMD1
CATCGTCTCTGAGATC-1	singlet	2	-572.8133431	-520.5630245	-215.2957442	EMD1
CATGAGTAGATTCGAA-1	singlet	1	-5567.535745	-1843.406888	-4476.074679	EMD1
CATGAGTAGGAACTCG-1	singlet	1	-1583.771162	-457.8064247	-1144.566064	EMD1
CATGAGTAGGGACAGG-1	singlet	0	-266.4920269	-754.595806	-1054.665622	EMD1
CATGAGTCAGTGGGTA-1	singlet	2	-476.6613695	-400.1406067	-203.9235241	EMD1
CATGAGTGTATCATGC-1	singlet	2	-3281.229544	-2538.177629	-1045.676347	EMD1
CATGAGTGTCAAAGCG-1	singlet	2	-155.5751309	-144.2676996	-72.04054966	EMD1
CATGAGTGTGAGGCAT-1	singlet	2	-285.9324722	-233.1188278	-132.8435944	EMD1
CATGAGTGTGGAGGTT-1	singlet	2	-1222.308574	-995.4048144	-448.4353604	EMD1
CATGAGTTCAGCGCGT-1	singlet	2	-262.0883963	-211.8975392	-103.8853505	EMD1
CATGAGTTCGACGACC-1	singlet	1	-3498.092852	-1304.851406	-2834.620326	EMD1
CATGCAAAGAGTTGCG-1	singlet	0	-193.1212692	-702.1416083	-611.4181779	EMD1
CATGCAAAGCATCTTG-1	singlet	0	-1289.924644	-3710.528927	-3626.609695	EMD1
CATGCAACAATGGCCC-1	singlet	0	-347.629782	-1090.24935	-950.0878199	EMD1
CATGCAAGTACGTGAG-1	singlet	2	-588.7813045	-501.409843	-219.6676104	EMD1
CATGCAAGTCAACCAT-1	singlet	1	-405.5430871	-198.5582192	-376.7028412	EMD1
CATGCAAGTCCCTAAA-1	singlet	2	-1492.308892	-1474.325032	-639.8472174	EMD1
CATGCCTAGCCTAACT-1	singlet	1	-2371.010568	-643.867817	-1804.445942	EMD1
CATGCCTAGGTCCGAA-1	singlet	1	-2293.252573	-839.945815	-1865.321682	EMD1
CATGCCTCAACTACGT-1	singlet	0	-282.1305605	-857.5246311	-804.2124793	EMD1
CATGCCTCACAGTCAT-1	singlet	1	-4421.078143	-1400.543668	-3328.567147	EMD1
CATGCCTCACATCATG-1	singlet	0	-2081.512196	-7062.590583	-7221.210274	EMD1
CATGCCTCAGCGTGCT-1	singlet	2	-544.4860246	-473.9490246	-226.4356478	EMD1
CATGCCTCAGGCTATT-1	singlet	0	-1279.039374	-3851.026638	-3838.366934	EMD1
CATGCCTCAGGGTCTC-1	singlet	1	-10299.53882	-2403.020549	-7287.739873	EMD1
CATGCCTGTATTGACC-1	singlet	1	-4062.321903	-1219.18498	-3178.790117	EMD1
CATGCCTGTCCCTGTT-1	singlet	2	-724.3330443	-560.8471555	-283.5607778	EMD1
CATGCCTGTTTGGGTT-1	singlet	2	-723.6269117	-572.2600698	-257.5346251	EMD1
CATGCCTTCCCTCATG-1	singlet	2	-1408.512682	-1095.826902	-510.7499789	EMD1
CATGCCTTCCGATCGG-1	singlet	2	-2638.569495	-2186.000988	-856.0258661	EMD1
CATGCCTTCCTTATGT-1	singlet	1	-3478.959896	-711.2202624	-2842.876294	EMD1
CATGCGGAGACGGTCA-1	singlet	1	-2592.340182	-522.5520524	-2154.841538	EMD1
CATGCGGAGGAACGAA-1	singlet	2	-1225.76931	-688.2430411	-323.8904202	EMD1
CATGCGGCAAGGTACG-1	singlet	2	-254.2088702	-238.7738628	-121.4011997	EMD1
CATGCGGCACCTTCGT-1	singlet	2	-1311.36755	-1292.604754	-387.2503866	EMD1
CATGCGGGTACGATGG-1	singlet	1	-1368.362954	-473.79096	-1097.957777	EMD1
CATGCTCAGGAACTCG-1	singlet	2	-4283.682022	-3331.79741	-1228.410541	EMD1
CATGCTCAGTATTCCG-1	singlet	2	-551.6523568	-473.1874611	-218.1793956	EMD1
CATGCTCAGTCTAACC-1	singlet	2	-3836.686198	-2935.858196	-1323.353864	EMD1
CATGCTCCAACTCATG-1	doublet	45293	-3947.619379	-2091.667013	-2627.858541	EMD1
CATGCTCCATACTGTG-1	singlet	1	-2483.275972	-850.9104397	-2016.760374	EMD1
CATGCTCCATGGAATA-1	singlet	0	-1411.793493	-5009.297348	-4638.961615	EMD1
CATGCTCGTAACATAG-1	singlet	1	-2077.903147	-536.4363206	-1782.43627	EMD1
CATGCTCGTAATCAGA-1	singlet	1	-9331.049147	-2280.744156	-8150.247198	EMD1
CATGCTCGTGATTAGA-1	singlet	2	-1517.381899	-1507.769312	-522.0358535	EMD1
CATGCTCGTTAGAGTA-1	singlet	2	-748.1907146	-605.8875271	-291.8101246	EMD1
CATGCTCGTTCAGCTA-1	singlet	2	-1594.437199	-1598.82293	-489.0640372	EMD1
CATGCTCTCCGGCTTT-1	singlet	2	-305.3972419	-258.8765373	-130.327286	EMD1
CATGGATAGAGGGTCT-1	doublet	0/1	-24.77540156	-29.41071849	-201.7989087	EMD1
CATGGATAGTACAGCG-1	singlet	0	-906.7872823	-2138.857181	-2110.782494	EMD1
CATGGATAGTGACACG-1	singlet	2	-673.3831711	-551.9249764	-227.7348608	EMD1
CATGGATCAACGGCTC-1	unassigned	1/0	-16.49592282	-14.78863584	-19.32234949	EMD1
CATGGATCACAATGCT-1	singlet	1	-4999.546837	-1752.688969	-3942.790609	EMD1
CATGGATCATCTATCT-1	singlet	0	-651.7378738	-2169.070775	-1944.913173	EMD1
CATGGTAAGAAACTGT-1	singlet	1	-317.9510464	-140.3070828	-252.2151881	EMD1
CATGGTAAGCCATCCG-1	singlet	1	-1338.644823	-403.2531722	-911.9588208	EMD1
CATGGTAAGGGATGTC-1	singlet	0	-1304.591784	-4498.370454	-4864.41692	EMD1
CATGGTAAGGTCACTT-1	singlet	0	-858.1433043	-2821.684007	-2350.72725	EMD1
CATGGTAAGTCCGCGT-1	singlet	1	-3328.057302	-1076.795216	-2751.39063	EMD1
CATGGTACACGGCTAC-1	singlet	0	-798.9497676	-2323.817893	-2403.374075	EMD1
CATGGTACATCATTGG-1	singlet	0	-208.5139865	-492.9120493	-601.5331825	EMD1
CATGGTAGTGGTACAG-1	singlet	2	-421.2001493	-344.5718159	-164.4845305	EMD1
CATGGTATCCCTTGTG-1	singlet	1	-784.0998683	-302.4130149	-576.9544833	EMD1
CATGGTATCGCTTGCT-1	singlet	2	-516.9770506	-446.5403093	-189.093106	EMD1
CATGGTATCGTAACAC-1	singlet	2	-449.8918071	-359.8628314	-184.3431387	EMD1
CATTCATAGCGTCTGC-1	singlet	1	-1130.605915	-322.8987324	-1051.566712	EMD1
CATTCATCACACAGCC-1	singlet	2	-1350.615083	-1198.641987	-543.7949047	EMD1
CATTCATCAGCTTCGG-1	singlet	0	-168.8525312	-487.3340051	-485.7300762	EMD1
CATTCATCATAATCGC-1	singlet	0	-2863.195599	-11630.71788	-9855.31979	EMD1
CATTCATGTATATGGA-1	singlet	2	-516.2575632	-439.6205519	-207.0257963	EMD1
CATTCATGTCCGGTGT-1	singlet	0	-246.9477297	-676.7880512	-674.1589427	EMD1
CATTCATGTGACTGAG-1	singlet	2	-1257.587368	-1148.118412	-371.714542	EMD1
CATTCATGTTGCCATA-1	singlet	0	-651.4101765	-1977.180062	-1938.195048	EMD1
CATTCATTCACTTGTT-1	singlet	2	-899.3377744	-709.1554893	-310.2729278	EMD1
CATTCCGAGAGATTCA-1	singlet	0	-152.2428881	-427.6641686	-519.7787966	EMD1
CATTCCGAGTGAACAT-1	singlet	2	-485.20067	-358.7582863	-204.1333692	EMD1
CATTCCGGTTGTAAAG-1	singlet	0	-158.0097509	-431.6773	-411.7805113	EMD1
CATTCCGTCAAATAGG-1	singlet	2	-11159.26905	-8382.728343	-2658.409957	EMD1
CATTCCGTCATTTACC-1	singlet	2	-1471.813801	-1089.642902	-550.0086497	EMD1
CATTCTAAGAGGTTAT-1	singlet	2	-4292.191804	-3176.324713	-1527.488014	EMD1
CATTCTAAGCTGTACT-1	singlet	2	-751.4584695	-645.4956911	-290.6471072	EMD1
CATTCTAAGTATAACG-1	singlet	2	-476.8705352	-388.8530039	-209.7530995	EMD1
CATTCTACAACGTTAC-1	singlet	2	-166.1656825	-145.9997752	-51.6652579	EMD1
CATTCTACATCGATGT-1	singlet	2	-115.4651248	-126.5960128	-36.55834478	EMD1

CATTCTAGTAAGATTG-1	singlet	2	-1466.1186	-1660.001067	-462.8183974	EMD1
CATTCTAGTCGATTAC-1	singlet	1	-1901.959091	-757.3543222	-1689.128997	EMD1
CATTCTAGTTCCATTT-1	singlet	2	-1080.716885	-966.7104516	-434.5841008	EMD1
CATTCTATCTCGCGTT-1	singlet	2	-1246.36893	-1155.043992	-423.5624127	EMD1
CATTGAGAGAAGGGAT-1	singlet	0	-28.15895816	-102.4029152	-87.64272528	EMD1
CATTGAGAGATACATG-1	singlet	0	-1349.70396	-3722.919225	-3774.36681	EMD1
CATTGAGAGTCATTGC-1	singlet	2	-459.3253204	-380.9123086	-170.9917293	EMD1
CATTGAGCAAAGGTTA-1	doublet	1/0	-2512.138411	-1426.693715	-2417.723755	EMD1
CATTGAGCACAACATC-1	singlet	2	-376.2644918	-336.5482484	-141.3998565	EMD1
CATTGAGCACAGCCAC-1	singlet	1	-5090.809201	-1567.020999	-3884.154073	EMD1
CATTGAGCACCCTTAC-1	singlet	1	-7304.365518	-2301.710661	-5951.252761	EMD1
CATTGAGCACCGGTCA-1	singlet	0	-443.5459884	-1354.776896	-1233.874242	EMD1
CATTGAGCATTAGGCT-1	singlet	0	-201.5374891	-677.6066444	-598.1454801	EMD1
CATTGAGCATTCTTCA-1	singlet	2	-435.1229292	-372.9017812	-180.1495557	EMD1
CATTGAGGTAGGCTGA-1	singlet	2	-1807.444731	-1481.475561	-662.4211731	EMD1
CATTGAGGTGGATCGA-1	doublet	45323	-2896.996839	-1718.591925	-1712.537364	EMD1
CATTGAGTCAGTGCGC-1	singlet	0	-409.5878646	-1000.788626	-928.7575096	EMD1
CATTGAGTCTAGCCAA-1	singlet	0	-258.9857519	-733.8919029	-759.4731742	EMD1
CATTGCCCACAGCGCT-1	singlet	0	-785.4005508	-3109.748929	-2539.013161	EMD1
CATTGCCCACTATGTG-1	singlet	2	-318.7671728	-286.7715916	-138.9766034	EMD1
CATTGCCGTAACTGCT-1	singlet	0	-195.1266558	-595.6673827	-544.1873073	EMD1
CATTGCCGTAGCGCTC-1	singlet	1	-1813.227311	-552.6769381	-1370.418975	EMD1
CATTGCCTCGAGTCCG-1	singlet	0	-551.7448105	-1898.97993	-1739.963693	EMD1
CATTGCCTCGCTCATC-1	singlet	1	-3908.171859	-1417.978797	-3118.11705	EMD1
CATTGTTAGCTGAGTG-1	singlet	0	-209.3701599	-756.6168174	-703.9017433	EMD1
CATTGTTAGGTTAAAC-1	singlet	1	-1832.858329	-701.2622569	-1478.770762	EMD1
CATTGTTCAACGCATT-1	singlet	2	-657.9477383	-541.3163254	-235.4846738	EMD1
CATTGTTCAACTCGTA-1	singlet	0	-514.1201495	-1404.448198	-1282.304185	EMD1
CATTGTTCAAGATGTA-1	singlet	1	-192.7121918	-63.33818352	-137.0000411	EMD1
CATTGTTCAGTTTCAG-1	singlet	2	-498.4996563	-395.8989887	-170.4301394	EMD1
CATTGTTCATTACGGT-1	singlet	2	-4804.8991	-3759.163068	-1743.346083	EMD1
CATTGTTGTGTCTTGA-1	singlet	1	-1318.081606	-458.8987935	-874.0315283	EMD1
CATTGTTGTTGTGGCC-1	singlet	0	-419.9161465	-1155.354405	-1152.851872	EMD1
CATTGTTTCCCGTTGT-1	singlet	0	-287.9210824	-869.0443298	-781.5496906	EMD1
CATTGTTTCTATCGGA-1	singlet	2	-6403.300145	-4901.70306	-2120.865441	EMD1
CATTGTTTCTGCACCT-1	singlet	1	-368.5169537	-140.6137217	-293.6092905	EMD1
CATTTCACAATCCTAG-1	singlet	0	-323.4071478	-758.0228639	-909.7317464	EMD1
CATTTCACAGCCCAGT-1	singlet	2	-408.4476728	-406.3450019	-179.284988	EMD1
CATTTCACATGTTACG-1	singlet	2	-2183.333006	-2186.835684	-779.9204219	EMD1
CATTTCAGTCAGACGA-1	singlet	2	-3391.053969	-2595.510863	-1138.968316	EMD1
CATTTCAGTCCGGCAT-1	singlet	2	-336.9188527	-323.827867	-142.3565646	EMD1
CATTTCAGTGTTACAC-1	singlet	2	-865.7156251	-786.1624421	-371.6108716	EMD1
CATTTCATCAGGAAAT-1	singlet	2	-824.9173474	-683.0192973	-346.8623339	EMD1
CATTTCATCCACACCT-1	singlet	1	-85.63307171	-39.48084884	-91.07361345	EMD1
CCAAGCGCAAGGTCGA-1	singlet	2	-480.980287	-404.8281542	-181.6467025	EMD1
CCAAGCGGTCAGCTTA-1	singlet	2	-506.3809216	-541.2841416	-228.5580616	EMD1
CCAAGCGGTCGTGGTC-1	singlet	1	-1535.72565	-457.6332348	-1231.760758	EMD1
CCAAGCGGTCTCAAGT-1	singlet	2	-475.7989716	-425.6872649	-174.5358263	EMD1
CCAAGCGGTGTGCCTG-1	singlet	2	-236.3493741	-181.3736432	-90.47007283	EMD1
CCAAGCGGTTAGGGAC-1	singlet	2	-482.9504786	-405.7772858	-170.7078218	EMD1
CCAAGCGGTTCAAGTC-1	singlet	2	-1848.857047	-1487.610437	-612.1510638	EMD1
CCAAGCGGTTCGGCGT-1	singlet	2	-523.9256391	-435.6859334	-198.2983614	EMD1
CCAAGCGTCATCTATC-1	singlet	2	-278.9793789	-216.0192919	-108.8766118	EMD1
CCAAGCGTCATGCCAA-1	singlet	1	-455.4170697	-200.5018603	-443.5529526	EMD1
CCAAGCGTCATGTCAG-1	singlet	2	-450.8641744	-395.4865826	-164.9246634	EMD1
CCAAGCGTCCAGTGCG-1	singlet	2	-250.8812107	-199.7489887	-97.43325958	EMD1
CCAATGACAGAGAAAG-1	singlet	2	-473.9831501	-395.5341443	-213.6676803	EMD1
CCAATGAGTGCATCTA-1	singlet	2	-434.4555887	-354.5595682	-200.4819124	EMD1
CCAATGATCCCTGGTT-1	singlet	1	-2013.075959	-590.15128	-1615.580328	EMD1
CCAATGATCTTCGTGC-1	singlet	2	-1080.545066	-980.9715112	-426.1785253	EMD1
CCAATTTAGACCAACG-1	singlet	2	-1041.416852	-1000.966851	-439.4902774	EMD1
CCAATTTAGTAGAATC-1	singlet	2	-426.7565122	-432.9488779	-174.3992478	EMD1
CCAATTTCAAGCTGCC-1	singlet	2	-2196.354936	-2023.636773	-866.5821821	EMD1
CCAATTTCAGCAGACA-1	singlet	1	-694.7378953	-290.0730078	-785.2732175	EMD1
CCAATTTGTAACTAAG-1	singlet	0	-29.25816972	-66.84765953	-64.69933933	EMD1
CCAATTTGTACAAAGT-1	singlet	2	-1181.238569	-1086.44335	-428.569548	EMD1
CCAATTTTCACCGGGT-1	doublet	0/2	-349.663562	-646.5599145	-460.7792965	EMD1
CCAATTTTCTTAAGGC-1	singlet	2	-454.1726584	-366.2213345	-188.5998307	EMD1
CCACAAAAGCTAATCC-1	singlet	1	-3576.686435	-1035.27991	-2816.875829	EMD1
CCACAAAGTCTAACTG-1	singlet	1	-636.0208829	-287.1076399	-686.1436561	EMD1
CCACAAATCACACCCT-1	singlet	2	-628.6744392	-559.3094401	-259.8281717	EMD1
CCACAAATCACTGTCC-1	singlet	2	-701.0153173	-579.9012978	-271.7137412	EMD1
CCACAAATCCACGTCT-1	singlet	0	-274.0050295	-623.74269	-735.1174953	EMD1
CCACAAATCCGAACGC-1	singlet	2	-395.1394542	-334.6927624	-194.9066281	EMD1
CCACAAATCCTGTTGC-1	doublet	1/0	-4456.749898	-3036.769416	-5100.789307	EMD1
CCACAAATCTCAATCT-1	unassigned	0	-10.47567763	-12.70254072	-94.78248468	EMD1
CCACACTAGATTGCGG-1	singlet	1	-8055.642327	-2292.805324	-6566.65185	EMD1
CCACACTAGCCATTTG-1	singlet	1	-502.2669884	-172.0572691	-343.7908564	EMD1
CCACACTAGTGGCGAT-1	singlet	1	-11474.92191	-2866.725784	-8123.268319	EMD1
CCACACTCAAGATCCT-1	singlet	0	-931.7734712	-3432.678374	-3192.895112	EMD1
CCACACTCAAGCTACT-1	singlet	0	-432.976232	-1198.998923	-1098.525424	EMD1
CCACACTCAATCTCTT-1	singlet	1	-4793.122734	-1580.980237	-3544.89025	EMD1
CCACACTCACAGAAGC-1	singlet	1	-4037.144763	-1136.268505	-3608.838588	EMD1
CCACACTCACCTGCTT-1	singlet	2	-461.3306536	-378.5143411	-191.4134316	EMD1
CCACACTCAGACCAAG-1	singlet	2	-178.7409601	-122.6417455	-55.05317312	EMD1
CCACACTGTAGACAGC-1	singlet	0	-223.3370617	-889.2257561	-793.2198844	EMD1
CCACACTGTAGGGTAC-1	singlet	2	-1885.638149	-1708.916574	-624.0483106	EMD1
CCACACTGTAGTGTGG-1	singlet	2	-364.3702844	-292.6968738	-153.0423706	EMD1
CCACACTGTCACCCTT-1	singlet	0	-479.2364988	-1228.584641	-1178.903206	EMD1
CCACACTGTGAGTTTC-1	singlet	1	-5584.908503	-1934.553563	-4524.730741	EMD1
CCACACTGTTTACGTG-1	singlet	1	-109.5442827	-28.66338367	-68.94528796	EMD1
CCACACTTCACGAGGA-1	singlet	0	-194.5553263	-498.7872641	-638.4664329	EMD1
CCACACTTCCAATCCC-1	singlet	2	-1675.402055	-1298.908355	-617.1311134	EMD1
CCACACTTCCATACAG-1	singlet	2	-653.6371944	-504.1450006	-235.7538811	EMD1
CCACACTTCGAAGCAG-1	singlet	2	-588.2291558	-499.2947762	-212.0437971	EMD1
CCACCATAGATGTAGT-1	singlet	2	-479.6437115	-408.4138632	-181.9651008	EMD1
CCACCATAGCATCGAG-1	singlet	2	-3745.591463	-3044.339095	-1168.165099	EMD1
CCACCATAGCCTAGGA-1	singlet	2	-828.2567398	-610.4393487	-303.1771658	EMD1
CCACCATAGCGAGAAA-1	singlet	2	-499.2636346	-394.3019525	-187.9016918	EMD1
CCACCATAGGATTCCT-1	singlet	2	-4242.459872	-3309.455445	-1490.049505	EMD1
CCACCATAGTCATCCA-1	singlet	0	-410.3353311	-1187.287845	-1066.498156	EMD1
CCACCATCAAGTGCAG-1	singlet	0	-915.3152959	-2670.520454	-2763.359962	EMD1
CCACCATCACCCAAGC-1	singlet	1	-1905.736877	-590.6737855	-1453.811477	EMD1
CCACCATCAGCGGTCT-1	singlet	2	-956.4369269	-785.9130546	-349.7869536	EMD1
CCACCATCATTGAAAG-1	singlet	1	-1014.55555	-311.3987544	-821.4794726	EMD1
CCACCATGTCGCCTAG-1	singlet	2	-321.7546651	-221.0404395	-111.3536538	EMD1
CCACCATGTCGTACAT-1	singlet	0	-533.8881477	-2253.839293	-2172.760644	EMD1
CCACCATGTTCAGTAC-1	singlet	1	-2112.959554	-832.091773	-1883.457051	EMD1
CCACCATTCCTGGCTT-1	singlet	2	-813.9004791	-668.9586151	-287.4166564	EMD1
CCACGAGAGCTGTTCA-1	singlet	0	-67.4714953	-212.4193478	-216.6480342	EMD1

CCACGAGAGGTGCATG-1	singlet	2	-612.5871361	-525.399391	-207.6164375	EMD1
CCACGAGCAATGACCT-1	singlet	2	-915.0039236	-776.3794178	-363.1307706	EMD1
CCACGAGGTGATAGTA-1	singlet	2	-2055.217595	-1924.025051	-662.1608666	EMD1
CCACGAGGTGGGTATG-1	singlet	0	-298.8201183	-1006.634	-888.4797726	EMD1
CCACGAGGTTGGGTAG-1	singlet	2	-462.9954612	-324.5027342	-168.2214774	EMD1
CCACGAGTCAAGGTGG-1	singlet	0	-1009.430123	-3398.837243	-3134.147134	EMD1
CCACGAGTCACCCTCA-1	singlet	2	-4052.50334	-3115.82242	-1409.453916	EMD1
CCACGAGTCGCATTAG-1	singlet	2	-419.5286622	-352.2551212	-171.3426595	EMD1
CCACGTTAGATTGAGT-1	singlet	2	-462.4884247	-418.150725	-197.9452423	EMD1
CCACGTTAGGCGTCCT-1	singlet	2	-202.4161147	-168.0478257	-70.42357242	EMD1
CCACGTTAGGCTTCCG-1	singlet	0	-59.70498639	-243.8952933	-248.1699276	EMD1
CCACGTTAGTTGGGAC-1	singlet	0	-297.6903409	-779.9096024	-764.1611409	EMD1
CCACGTTCAAGACAAT-1	singlet	1	-244.5348342	-60.58928586	-166.1042353	EMD1
CCACGTTGTTGTTGCA-1	singlet	2	-437.2826022	-347.1568628	-176.7416199	EMD1
CCACGTTTCAACCCGG-1	singlet	1	-504.6041475	-149.957262	-401.8252124	EMD1
CCACGTTTCCCAGGAC-1	singlet	2	-1410.163606	-1694.016932	-421.0341125	EMD1
CCACGTTTCGTGTTCC-1	singlet	0	-112.3367384	-271.459235	-286.2227815	EMD1
CCACGTTTCTCGACGG-1	singlet	0	-239.3757584	-544.9826659	-563.3018914	EMD1
CCACTTGAGAGAGAAC-1	singlet	0	-905.90756	-2468.785218	-2478.714548	EMD1
CCACTTGAGCCTGACC-1	singlet	1	-2573.646478	-789.7779202	-1892.250533	EMD1
CCACTTGAGGCCTGCT-1	singlet	1	-2202.836196	-739.3838646	-1679.234593	EMD1
CCACTTGAGGGTACAC-1	singlet	2	-1774.820191	-1686.182301	-757.3536724	EMD1
CCACTTGCACGGGCTT-1	singlet	0	-426.7268853	-1432.297575	-1356.780699	EMD1
CCACTTGCATCCTGTC-1	singlet	0	-326.5488235	-1171.404956	-1069.210658	EMD1
CCACTTGCATGACTTG-1	singlet	1	-264.4317361	-94.83223808	-200.328738	EMD1
CCACTTGGTCGCATTA-1	singlet	1	-262.0717018	-128.477423	-245.7820304	EMD1
CCACTTGTCACGGGCT-1	singlet	2	-1092.923183	-917.2321429	-406.6279275	EMD1
CCATAAGAGAAGGATG-1	singlet	0	-1689.781183	-4080.629339	-3592.248248	EMD1
CCATAAGAGACGGTTG-1	singlet	1	-895.6278487	-332.1546638	-619.0399724	EMD1
CCATAAGAGCAGCGAT-1	singlet	0	-289.39196	-724.6390329	-695.0347384	EMD1
CCATAAGAGGACTGGT-1	singlet	0	-29.37051377	-104.3341952	-84.25406444	EMD1
CCATAAGCATCCTATT-1	singlet	0	-1575.271461	-10318.06465	-8013.746533	EMD1
CCATAAGCATCCTTCG-1	singlet	1	-2050.842308	-700.1688278	-1596.041833	EMD1
CCATAAGGTCAAGGCA-1	singlet	2	-631.5641555	-561.5345345	-267.2083668	EMD1
CCATAAGGTGCCGTAC-1	singlet	0	-234.9207842	-619.2079433	-721.2667838	EMD1
CCATAAGGTGTCATCA-1	singlet	0	-662.8461263	-2028.181525	-1859.963595	EMD1
CCATAAGTCCGTCAAA-1	singlet	1	-1699.95224	-455.8730417	-1510.852772	EMD1
CCATAAGTCGCGGTAC-1	singlet	2	-3820.144288	-2947.830808	-1106.64925	EMD1
CCATCACAGGTCGTGA-1	doublet	2/0	-3024.446076	-3637.313107	-2490.100014	EMD1
CCATCACAGTTCATGC-1	singlet	0	-203.80034	-605.324688	-536.8017583	EMD1
CCATCACCAGCAAGAC-1	singlet	0	-585.5032676	-1895.230785	-1929.438782	EMD1
CCATCACGTCTCCTGT-1	singlet	2	-645.8367447	-630.8197056	-296.2370794	EMD1
CCATCACTCACACGAT-1	singlet	0	-624.2233452	-2000.641504	-1754.13719	EMD1
CCATCACTCAGAATAG-1	singlet	1	-1769.700714	-575.3910714	-1350.700952	EMD1
CCATCACTCGCTCTCA-1	singlet	2	-1581.893294	-1499.235282	-465.1245155	EMD1
CCATCACTCGTGTGAT-1	singlet	2	-453.9056924	-396.176011	-200.5024919	EMD1
CCCAACTAGATTGATG-1	singlet	0	-221.5199938	-559.0132462	-617.9655815	EMD1
CCCAACTAGGCCTAAG-1	singlet	1	-4088.689643	-1302.009596	-3218.309936	EMD1
CCCAACTAGTGGAATT-1	singlet	2	-380.5481129	-335.4896128	-163.9754337	EMD1
CCCAACTCACGGTGTC-1	singlet	1	-1788.576179	-574.6045473	-1173.824763	EMD1
CCCAACTCATGAGTAA-1	singlet	0	-1032.393869	-3143.762373	-2663.543638	EMD1
CCCAACTGTAGACGTG-1	singlet	2	-156.8285613	-109.2774183	-45.81425644	EMD1
CCCAACTGTCAACATC-1	singlet	1	-1868.84525	-743.5780905	-1575.300344	EMD1
CCCAACTTCACTTCTA-1	singlet	1	-4228.92307	-1253.805779	-3332.74257	EMD1
CCCATTGAGAACCGCA-1	doublet	2/0	-2108.990018	-2655.091563	-1897.647224	EMD1
CCCATTGAGAATCGCG-1	singlet	2	-648.2210596	-537.0469896	-269.5753978	EMD1
CCCATTGAGATACAGT-1	singlet	2	-458.1520154	-393.5191719	-207.7797323	EMD1
CCCATTGAGATAGTGT-1	singlet	2	-2241.743599	-1649.607001	-662.9975847	EMD1
CCCATTGAGTCTAACC-1	singlet	1	-4275.183542	-1442.121309	-3254.086131	EMD1
CCCATTGAGTTAGTAG-1	singlet	2	-2087.441512	-1892.244392	-779.3966128	EMD1
CCCATTGCAGGTCAGA-1	singlet	2	-524.9946684	-468.1773842	-202.8035828	EMD1
CCCATTGCATATCGGT-1	singlet	1	-3117.657038	-926.6061184	-2513.077724	EMD1
CCCATTGCATGGTACT-1	singlet	0	-494.6095508	-1238.438655	-1226.263907	EMD1
CCCATTGGTAACGCGA-1	doublet	45323	-1160.921335	-793.1364533	-675.6343822	EMD1
CCCATTGGTCGCAACC-1	singlet	2	-376.5866112	-358.234715	-160.871682	EMD1
CCCATTGGTGAGAGGG-1	singlet	2	-472.5135707	-376.327769	-218.3840603	EMD1
CCCATTGGTTGTCAGT-1	singlet	1	-2242.286518	-568.4425002	-1832.166441	EMD1
CCCATTGTCAATCGGT-1	singlet	2	-583.732379	-447.1521078	-211.1276888	EMD1
CCCATTGTCGTCCTCA-1	singlet	2	-325.7993627	-228.3243142	-85.0140739	EMD1
CCCATTGTCTTCCGTG-1	singlet	0	-454.6535947	-1326.632564	-1401.622619	EMD1
CCCGAAGAGCTCGACC-1	singlet	2	-263.7661701	-220.6554425	-116.4459654	EMD1
CCCGAAGCAAAGCTCT-1	singlet	2	-9837.981347	-7718.941576	-2603.221006	EMD1
CCCGAAGCAGTGGCTC-1	singlet	0	-599.7910868	-1826.003301	-1558.675215	EMD1
CCCGAAGCATACCACA-1	singlet	0	-344.6249504	-950.7822588	-1021.278346	EMD1
CCCGAAGGTGTCGATT-1	singlet	2	-354.0044761	-342.8242773	-139.8573044	EMD1
CCCGAAGGTTCTCAGA-1	singlet	2	-864.8237912	-764.0047984	-344.5941966	EMD1
CCCGAAGTCAAGTAAG-1	singlet	0	-82.78636491	-183.0526388	-170.6066851	EMD1
CCCGAAGTCCGCTAGG-1	singlet	0	-628.6747148	-1986.140513	-2034.302668	EMD1
CCCGAAGTCGGCTTGG-1	singlet	1	-1041.949617	-344.032327	-897.1827557	EMD1
CCCGGAAAGTATGAAC-1	singlet	0	-232.7087962	-675.3899631	-647.0041365	EMD1
CCCGGAACAATGACCT-1	singlet	2	-654.6611694	-439.0104188	-227.8536341	EMD1
CCCGGAACACAACATC-1	singlet	0	-126.8211372	-453.4115926	-424.5348828	EMD1
CCCGGAACACTGAATC-1	singlet	0	-420.6410988	-1147.173643	-1128.796762	EMD1
CCCGGAACACTTTAGG-1	doublet	45323	-393.6613536	-401.9058258	-250.4572842	EMD1
CCCGGAACATAGGAGC-1	doublet	2/0	-1551.830402	-1867.61817	-1325.214493	EMD1
CCCGGAAGTGCTTCAA-1	singlet	2	-252.9358816	-202.8503696	-104.9168833	EMD1
CCCGGAAGTGTATCCA-1	singlet	2	-1908.530831	-1454.629182	-621.8118481	EMD1
CCCGGAATCCACTGAA-1	singlet	2	-762.2926191	-661.5028329	-270.2829273	EMD1
CCCGGAATCCTACTGC-1	singlet	0	-286.5676786	-1285.359729	-1145.281316	EMD1
CCCTAACAGAACAAGG-1	singlet	1	-2815.25094	-873.4375981	-2011.188179	EMD1
CCCTAACAGGGTTAGC-1	singlet	2	-708.1834904	-509.6003239	-299.4593438	EMD1
CCCTAACCAACCAGAG-1	singlet	1	-5431.169251	-1814.336394	-4281.335705	EMD1
CCCTAACCAAGCTGCC-1	singlet	0	-1003.561193	-2548.161812	-2541.994672	EMD1
CCCTAACCACCGCTGA-1	singlet	1	-1952.37565	-595.6575402	-1714.628044	EMD1
CCCTAACCATCCGCGA-1	singlet	0	-350.2271989	-1275.818731	-1292.758646	EMD1
CCCTAACCATCCTTCG-1	singlet	0	-344.89219	-883.0703463	-840.2251974	EMD1
CCCTAACGTGTAGCAG-1	singlet	2	-219.3573946	-172.2496721	-103.2428339	EMD1
CCCTAACGTGTCCAAT-1	singlet	0	-366.1470658	-1132.128398	-1014.452597	EMD1
CCCTAACGTTGCGGCT-1	singlet	2	-539.941407	-393.6056644	-182.9033606	EMD1
CCCTAACTCGTAACAC-1	singlet	2	-649.3614232	-487.3184009	-243.4539582	EMD1
CCCTAACTCTAGATCG-1	singlet	2	-225.2556895	-165.2006248	-85.69285898	EMD1
CCCTCAAAGCCGATAG-1	singlet	1	-2340.334701	-778.6666212	-1838.626269	EMD1
CCCTCAACACGCTTAA-1	singlet	1	-807.7542416	-349.2100922	-820.8535061	EMD1
CCCTCAACACTCCGAG-1	singlet	0	-3691.799519	-10561.05358	-10636.15493	EMD1
CCCTCAAGTCTGTCCT-1	singlet	0	-88.45186849	-297.401309	-251.9684259	EMD1
CCCTCAAGTGAGCAGT-1	singlet	2	-561.8209342	-426.4961043	-227.8443385	EMD1
CCCTCAAGTTACACTG-1	singlet	2	-452.6389744	-387.3723754	-158.8049705	EMD1
CCCTCAATCACTACTT-1	singlet	2	-495.5407508	-431.6562245	-204.3880126	EMD1
CCCTCAATCCCGATCT-1	singlet	2	-719.9525464	-732.9713548	-245.1541443	EMD1
CCCTCTCAGACGATAT-1	singlet	0	-44.15030742	-121.0759426	-100.7934847	EMD1

CCCTCTCAGCATTTCG-1	singlet	1	-2004.316109	-827.364101	-1865.916874	EMD1
CCCTCTCCAGTAACGG-1	singlet	2	-277.923407	-246.2252122	-114.5615768	EMD1
CCCTCTCCATAATGAG-1	singlet	0	-66.80919974	-212.7558483	-233.0856615	EMD1
CCCTCTCCATGCCGGT-1	singlet	1	-2408.046713	-757.7638474	-2518.137241	EMD1
CCCTCTCGTATTTCGG-1	singlet	0	-213.8263108	-679.4930121	-669.6716396	EMD1
CCCTCTCGTTGTTTGG-1	singlet	1	-2935.100034	-1062.635018	-2349.210072	EMD1
CCCTCTCTCACCTCAC-1	singlet	0	-271.8293935	-685.0123028	-755.3892442	EMD1
CCCTCTCTCATTCGGA-1	singlet	2	-399.48921	-377.0955052	-159.5288503	EMD1
CCCTCTCTCCATCTCG-1	singlet	0	-564.2866108	-2080.83166	-2399.641548	EMD1
CCCTCTCTCGCATTAG-1	singlet	2	-351.5884407	-391.4056503	-164.4377152	EMD1
CCCTCTCTCGGTGAAG-1	singlet	2	-669.4320917	-451.3899952	-241.7502316	EMD1
CCCTGATAGCAGCCTC-1	singlet	1	-362.6575061	-134.1832428	-294.8287615	EMD1
CCCTGATAGCGCCGTT-1	singlet	2	-468.9705315	-427.5413047	-207.7318956	EMD1
CCCTGATAGCTGTACT-1	singlet	2	-652.2807722	-472.856322	-237.8150308	EMD1
CCCTGATAGGACTAAT-1	singlet	1	-563.8075883	-232.2065482	-513.4845544	EMD1
CCCTGATAGTCGAAAT-1	singlet	0	-103.8720935	-340.4550056	-285.1153525	EMD1
CCCTGATCAAACAGGC-1	doublet	1/0	-322.1157515	-221.8113608	-306.9598275	EMD1
CCCTGATCAGCTATAC-1	singlet	2	-529.9632998	-419.8189508	-198.3399195	EMD1
CCCTGATCATGACGAG-1	singlet	2	-392.037991	-347.5846876	-154.422651	EMD1
CCCTGATCATTGGCAT-1	singlet	1	-1451.630061	-489.0217674	-1090.521648	EMD1
CCCTGATGTATGTCAC-1	singlet	1	-2799.710393	-1030.870627	-2450.222239	EMD1
CCCTGATGTCAGGTGA-1	singlet	1	-2092.011651	-619.8678022	-1600.798267	EMD1
CCCTGATGTCCACACG-1	singlet	1	-2748.313102	-636.9326114	-1849.136156	EMD1
CCCTGATGTCGTGTTA-1	singlet	0	-287.713705	-881.9866456	-790.6039021	EMD1
CCCTGATTCATGCCCT-1	singlet	0	-276.0162018	-811.606369	-778.9161746	EMD1
CCCTGATTCATTTACC-1	singlet	1	-2332.100127	-915.772337	-1982.456055	EMD1
CCCTGATTCGATCCAA-1	singlet	0	-865.6721895	-2547.223594	-2732.95539	EMD1
CCCTGATTCTCTCCGA-1	singlet	0	-1263.239395	-3491.415991	-3261.121369	EMD1
CCCTTAGAGCACTGGA-1	singlet	1	-1772.372107	-617.6675153	-1465.849391	EMD1
CCCTTAGAGGACGCAT-1	singlet	2	-576.9039733	-528.9213448	-229.4816006	EMD1
CCCTTAGAGTACAGAT-1	singlet	0	-15.01095925	-74.52603446	-61.58079723	EMD1
CCCTTAGAGTAGCCAG-1	singlet	2	-376.2906177	-331.0891037	-150.8341704	EMD1
CCCTTAGCACCATATG-1	singlet	1	-2239.77843	-768.4962505	-1772.913682	EMD1
CCCTTAGCACCCTTAC-1	singlet	0	-2958.552187	-6506.664578	-6898.247346	EMD1
CCCTTAGGTGGCTGCT-1	doublet	0/1	-25.10624797	-26.4183091	-299.5534159	EMD1
CCCTTAGTCAAATGAG-1	singlet	0	-1765.266098	-6596.403745	-6735.360585	EMD1
CCCTTAGTCGATGCAT-1	singlet	2	-1840.624063	-1438.702393	-613.6315084	EMD1
CCGAACGAGAAGCTCG-1	singlet	2	-243.0861717	-230.1558935	-114.59902	EMD1
CCGAACGAGAGTGTTA-1	singlet	2	-988.3361328	-818.516562	-333.6171751	EMD1
CCGAACGGTCAGGTGA-1	singlet	2	-2396.059651	-1928.884931	-840.0409573	EMD1
CCGAACGGTGTGTACT-1	singlet	1	-2212.790215	-844.3142439	-1770.710602	EMD1
CCGAACGGTTGAAGTA-1	singlet	0	-192.6353841	-587.5829756	-550.5812213	EMD1
CCGAACGTCCCTCTAG-1	singlet	2	-477.0926306	-374.7099534	-177.2634335	EMD1
CCGAACGTCCTGTAAG-1	singlet	0	-297.394303	-1136.021756	-918.6010806	EMD1
CCGATCTCAAGGTCAG-1	unassigned	0/2	-8.358789267	-21.90767143	-10.24201878	EMD1
CCGATCTCACTCAAGT-1	singlet	1	-2742.293267	-865.7588324	-2154.78691	EMD1
CCGATCTGTAAGGAGA-1	singlet	2	-486.3768678	-366.8353452	-207.5099441	EMD1
CCGATCTGTATTTCGG-1	singlet	2	-477.8039133	-394.650071	-207.6050521	EMD1
CCGATCTTCACCCTGT-1	singlet	2	-665.4932308	-524.3760641	-215.4682862	EMD1
CCGATCTTCACGGAGA-1	singlet	2	-416.3730311	-337.7420097	-141.9202427	EMD1
CCGATCTTCGCACTCT-1	singlet	2	-622.2681882	-548.4769353	-210.9664953	EMD1
CCGATCTTCTCCGATC-1	singlet	0	-79.54584389	-194.9329198	-174.3419113	EMD1
CCGATGGAGACAGCGT-1	singlet	1	-271.4118486	-96.44450511	-204.6163541	EMD1
CCGATGGAGTTAACGA-1	singlet	1	-70.3181171	-32.26417167	-73.03096582	EMD1
CCGATGGCAAGCACCC-1	singlet	2	-792.038972	-667.6078389	-303.20879	EMD1
CCGATGGCAGGCGATA-1	singlet	2	-287.0238525	-241.9730628	-105.3083507	EMD1
CCGATGGCAGGGTCTC-1	singlet	0	-109.9440334	-430.115654	-368.7162225	EMD1
CCGATGGCAGTCGGAA-1	singlet	2	-286.1234905	-243.0727507	-117.9599905	EMD1
CCGATGGGTCACAGAG-1	singlet	2	-1435.688604	-1177.400595	-477.2516603	EMD1
CCGATGGGTGAGTGAC-1	singlet	0	-213.2933661	-560.1376343	-596.4178421	EMD1
CCGATGGGTGCTCTCT-1	singlet	2	-488.0985869	-520.4177825	-245.0635284	EMD1
CCGATGGGTGCTGATT-1	singlet	1	-2584.858077	-971.4203234	-1991.237516	EMD1
CCGATGGTCGAAGTGG-1	singlet	2	-3041.941654	-1885.488687	-781.2403341	EMD1
CCGCAAGCACGTATAC-1	singlet	0	-448.8929065	-1825.123141	-1619.954003	EMD1
CCGCAAGCACGTCGTG-1	singlet	0	-107.3398914	-386.0613847	-357.9120017	EMD1
CCGCAAGGTACGTTCA-1	singlet	1	-1291.430773	-521.5633355	-1133.421452	EMD1
CCGCAAGTCCGCAAAT-1	singlet	2	-209.1794539	-232.2383083	-118.5642762	EMD1
CCGGACAAGACGTCGA-1	singlet	0	-572.9408169	-1756.828002	-1420.673333	EMD1
CCGGACAAGATGACCG-1	singlet	2	-406.1780812	-314.7208734	-155.9768784	EMD1
CCGGACAAGCCGCTTG-1	singlet	2	-468.1952684	-382.4073453	-187.9486878	EMD1
CCGGACAAGCTGTGCC-1	doublet	45293	-3098.702645	-1200.778269	-1826.910993	EMD1
CCGGACAAGTCGGGAT-1	singlet	1	-4719.145343	-1599.217311	-3753.983072	EMD1
CCGGACAAGTGATAGT-1	singlet	2	-1293.28053	-1056.767909	-516.842749	EMD1
CCGGACAAGTGTAGTA-1	singlet	0	-32.4262596	-81.02898318	-74.68805864	EMD1
CCGGACACATTGTAGC-1	doublet	45293	-2180.70233	-1195.813881	-1444.641166	EMD1
CCGGACAGTAGCACAG-1	doublet	2/0	-1020.317789	-1247.873671	-846.5864098	EMD1
CCGGACAGTGCAATGG-1	singlet	1	-1417.693929	-400.1730712	-1019.167419	EMD1
CCGGACATCCTACGGG-1	singlet	2	-7755.135048	-6188.18624	-2370.819245	EMD1
CCGGACATCTCCGAAA-1	singlet	0	-169.6012459	-470.9763568	-577.260745	EMD1
CCGGACATCTTGGTGA-1	singlet	1	-703.7368528	-239.2865579	-505.15631	EMD1
CCGGGTAAGAAGCTCG-1	singlet	0	-257.9826667	-782.9447939	-743.2718139	EMD1
CCGGGTAAGCACCTGC-1	singlet	0	-122.9989686	-349.9319568	-325.7738488	EMD1
CCGGGTAAGCCTCCAG-1	singlet	2	-322.0687138	-269.8554155	-129.881336	EMD1
CCGGGTAAGGAACGCT-1	singlet	0	-1344.624497	-4829.097653	-4563.522339	EMD1
CCGGGTAAGGGTCTTT-1	singlet	1	-1707.893237	-410.4208196	-1162.32363	EMD1
CCGGGTAAGTCGCTAT-1	singlet	2	-467.0750804	-354.9922324	-190.5139826	EMD1
CCGGGTAAGTTCAACC-1	singlet	2	-577.5514093	-463.5210964	-214.8275012	EMD1
CCGGGTACATCGGAAG-1	singlet	2	-356.1112744	-323.8387273	-143.171568	EMD1
CCGGGTAGTCCCACGA-1	singlet	2	-1073.066928	-915.9402932	-394.818916	EMD1
CCGGGTATCAAACGAA-1	singlet	2	-426.6784922	-342.409008	-163.0910112	EMD1
CCGGGTATCTGTCCGT-1	singlet	2	-1406.176366	-1374.897651	-496.2377737	EMD1
CCGGTAGAGACATACA-1	singlet	0	-302.6419894	-940.1350031	-891.3672256	EMD1
CCGGTAGAGATCGCCC-1	singlet	0	-557.2566346	-1483.242431	-1508.356443	EMD1
CCGGTAGAGGCACGAT-1	singlet	2	-488.0511524	-388.4683163	-192.8350897	EMD1
CCGGTAGAGGTTCATC-1	singlet	0	-124.1182098	-333.1233891	-395.367715	EMD1
CCGGTAGAGTTGTAGA-1	singlet	1	-814.1550037	-271.6562775	-719.1113819	EMD1
CCGGTAGCAAGACGAC-1	singlet	2	-403.1519531	-334.4234274	-157.2119736	EMD1
CCGGTAGGTTGATGTC-1	singlet	2	-536.143793	-389.1706578	-190.6972576	EMD1
CCGGTAGGTTGTGGCC-1	singlet	1	-1343.784049	-477.3461201	-1046.006935	EMD1
CCGGTAGTCAGGAAAT-1	singlet	0	-562.5166661	-2212.09159	-1906.451514	EMD1
CCGGTAGTCGCATTAG-1	singlet	2	-613.8984899	-534.0602422	-233.7961069	EMD1
CCGGTAGTCTAGGCCG-1	singlet	0	-443.9411251	-1320.844042	-1324.788685	EMD1
CCGGTAGTCTCGGCTT-1	singlet	2	-629.7358633	-555.9607614	-260.9527147	EMD1
CCGGTAGTCTGAGTCA-1	singlet	2	-2256.275773	-1788.432748	-625.3246002	EMD1
CCGGTGAAGTCGGCCT-1	singlet	1	-1887.436794	-830.1455622	-1616.666621	EMD1
CCGGTGACAGGGCTTC-1	singlet	0	-1024.126018	-2817.653977	-2939.428868	EMD1
CCGGTGACATGCAGCC-1	singlet	1	-5008.97958	-1658.841117	-3864.061525	EMD1
CCGGTGACATGTAACC-1	singlet	1	-4072.383868	-1393.034913	-3298.838209	EMD1
CCGGTGAGTAGTGCGA-1	singlet	2	-507.0146111	-419.5692977	-197.4127953	EMD1
CCGGTGATCTGGCCTT-1	singlet	2	-672.9688166	-569.8990416	-233.2632237	EMD1

CCGTAGGAGACATGCG-1	singlet	0	-301.7486868	-836.2005296	-886.7136832	EMD1
CCGTAGGAGGATTCAA-1	singlet	2	-677.3702204	-546.1682842	-274.3665224	EMD1
CCGTAGGAGGTAGCAC-1	singlet	2	-1817.321838	-1676.084666	-592.2365206	EMD1
CCGTAGGCAGCTTCGG-1	singlet	1	-187.4390888	-85.39290417	-250.2441356	EMD1
CCGTAGGGTTCTCGTC-1	singlet	2	-44.68633145	-33.72445199	-18.17733772	EMD1
CCGTAGGTCAGCATTG-1	singlet	1	-519.2818178	-129.6681785	-396.768031	EMD1
CCGTAGGTCATCCTAT-1	singlet	0	-494.6560886	-1316.125988	-1290.112924	EMD1
CCGTAGGTCGCCGAGT-1	singlet	0	-478.0376271	-1536.871545	-1497.829703	EMD1
CCGTAGGTCTCCGAAA-1	singlet	2	-624.6492917	-573.5304424	-229.9796792	EMD1
CCGTAGGTCTGGACTA-1	singlet	1	-675.8547365	-259.7481109	-459.2129865	EMD1
CCGTAGGTCTTCTGGC-1	singlet	2	-456.3394549	-354.8759306	-168.2698305	EMD1
CCGTGAGAGTACAGAT-1	singlet	1	-2987.583412	-1036.54166	-2378.178826	EMD1
CCGTGAGGTATCGATC-1	singlet	1	-260.8294277	-117.7216768	-220.0202719	EMD1
CCGTGAGGTCTTGGTA-1	singlet	0	-330.5057989	-929.2625521	-823.4131446	EMD1
CCGTGAGGTGTCATGT-1	singlet	1	-55.20570855	-27.16223935	-74.29588903	EMD1
CCGTGAGGTTTGGGTT-1	singlet	0	-171.3418191	-550.5292863	-534.4354126	EMD1
CCGTTCAAGAAACACT-1	singlet	2	-1487.976826	-1178.380226	-532.2124604	EMD1
CCGTTCAAGCGTGAAC-1	singlet	0	-1298.166403	-4101.945061	-4432.587117	EMD1
CCGTTCAAGTCTCGTA-1	singlet	2	-376.6945418	-376.2845306	-180.212166	EMD1
CCGTTCACAGAACTTC-1	singlet	2	-621.6950363	-570.4260711	-280.6307662	EMD1
CCGTTCAGTGTCCGTG-1	singlet	1	-4024.061485	-872.4086888	-2818.51535	EMD1
CCGTTCAGTTTCTATC-1	singlet	0	-347.7346278	-967.1790086	-1019.905438	EMD1
CCGTTCATCGGTGTAT-1	singlet	2	-1248.751982	-1090.47461	-454.7297513	EMD1
CCGTTCATCTGGGTCG-1	singlet	0	-412.1843892	-1043.256957	-1285.059909	EMD1
CCTAACCAGTGTAGTA-1	singlet	1	-346.476262	-89.77898647	-275.2424876	EMD1
CCTAACCCAGCAGATG-1	singlet	0	-673.1299792	-1920.900118	-1944.750164	EMD1
CCTAACCCAGCCGGTT-1	singlet	2	-503.4010086	-479.7048549	-238.0130921	EMD1
CCTAACCGTAATGCGG-1	singlet	2	-613.2359159	-500.9700541	-229.8437606	EMD1
CCTAACCTCTCACCCA-1	singlet	2	-377.0982782	-293.0940685	-150.3010489	EMD1
CCTAAGAAGACTGAGC-1	singlet	1	-1051.96187	-342.7277139	-874.5423975	EMD1
CCTAAGAAGCAAGTGC-1	singlet	2	-636.595531	-510.1721682	-237.7585499	EMD1
CCTAAGAAGCCGATTT-1	singlet	2	-2175.586546	-1763.588234	-712.4078575	EMD1
CCTAAGACAGGATTCT-1	singlet	2	-708.1257359	-645.0143699	-340.7600477	EMD1
CCTAAGACATTCTTCA-1	singlet	2	-2693.639526	-2163.616708	-994.6836034	EMD1
CCTAAGAGTAAGTTAG-1	singlet	2	-2095.719161	-1605.321184	-636.6363403	EMD1
CCTAAGAGTCCGGTCA-1	singlet	2	-586.768964	-437.3976928	-251.8405008	EMD1
CCTAAGATCCAGCACG-1	singlet	2	-173.3443862	-151.8095156	-86.72853649	EMD1
CCTAAGATCCATTGGA-1	singlet	2	-377.7456684	-345.9807951	-142.6727154	EMD1
CCTACGTAGAACGCGT-1	singlet	2	-3467.632118	-2954.393925	-1020.439669	EMD1
CCTACGTAGGACGGAG-1	singlet	2	-6207.492286	-5185.696497	-2233.914254	EMD1
CCTACGTAGTCTGCAT-1	singlet	0	-288.6492237	-888.7728288	-773.3690569	EMD1
CCTACGTCACCGTACG-1	singlet	2	-366.9171075	-283.7838897	-149.3801268	EMD1
CCTACGTCAGACACCC-1	singlet	0	-976.408552	-2972.737591	-2986.014475	EMD1
CCTACGTCATTATGCG-1	singlet	2	-532.3968608	-437.7316861	-206.0104852	EMD1
CCTACGTGTACTGCGC-1	singlet	2	-466.1953495	-441.5077067	-179.9619172	EMD1
CCTACGTGTCGTTTCC-1	singlet	1	-430.8890574	-145.9022965	-455.6770059	EMD1
CCTACGTGTGTCCGGT-1	doublet	0/2	-1887.903546	-3033.804837	-2403.522036	EMD1
CCTACGTTCCATACTT-1	singlet	2	-552.5633599	-462.4974092	-219.8770311	EMD1
CCTACGTTCCATTTCA-1	singlet	2	-634.7456603	-557.2334715	-287.7219477	EMD1
CCTATCGAGTATGATG-1	singlet	0	-54.1704775	-152.0069301	-157.991291	EMD1
CCTATCGCAACCTAAC-1	singlet	2	-1688.283592	-1351.165508	-549.5151368	EMD1
CCTATCGCAGAAGCGT-1	singlet	0	-477.0524197	-1664.160422	-1453.58366	EMD1
CCTATCGCAGGTCCCA-1	singlet	0	-285.8162696	-727.6927369	-725.786782	EMD1
CCTATCGCATCCCACT-1	singlet	2	-845.0499526	-700.7547041	-294.216482	EMD1
CCTATCGGTCTGTAGT-1	singlet	0	-175.9558469	-484.731144	-499.7751548	EMD1
CCTATCGTCGTAGGAG-1	singlet	2	-286.4640778	-277.4416182	-123.2459169	EMD1
CCTATCGTCTAGCCTC-1	singlet	2	-305.5824603	-296.1048863	-119.7152555	EMD1
CCTCAACAGACCAGAC-1	singlet	2	-710.0901279	-609.4211291	-279.845723	EMD1
CCTCAACAGGAATGTT-1	singlet	1	-53.32495206	-22.10395523	-44.37891026	EMD1
CCTCAACAGGGATCGT-1	singlet	0	-581.0095992	-2031.477352	-1685.898536	EMD1
CCTCAACAGTCTGCGC-1	singlet	1	-2434.711233	-686.1482798	-1624.29932	EMD1
CCTCAACCAGCAGACA-1	singlet	0	-531.2129741	-1781.698361	-1671.076796	EMD1
CCTCAACCAGGCGAAT-1	singlet	2	-338.3222997	-320.8284795	-153.9599578	EMD1
CCTCAACCAGTTACCA-1	singlet	0	-1048.791521	-3065.58924	-2960.598356	EMD1
CCTCAACGTACGTGTT-1	singlet	1	-502.4342185	-194.812534	-446.5226381	EMD1
CCTCAACGTCCCGCAA-1	singlet	2	-568.841525	-517.3730602	-263.8688664	EMD1
CCTCAACGTTAGGAGC-1	singlet	2	-406.8355688	-377.1611793	-186.6643681	EMD1
CCTCAACTCAAACCCA-1	singlet	0	-725.4360044	-2199.500739	-2103.13842	EMD1
CCTCAACTCACGATCA-1	singlet	0	-27.64665343	-58.06424014	-288.4757195	EMD1
CCTCAACTCCTCTAAT-1	singlet	2	-107.6937715	-105.1502447	-44.11625515	EMD1
CCTCAACTCTAGTACG-1	singlet	2	-291.2570895	-249.2952543	-126.7420041	EMD1
CCTCACAAGCCTCTGG-1	singlet	1	-1777.50919	-551.4186504	-1375.06209	EMD1
CCTCACAAGGAGCAAA-1	singlet	0	-1142.139734	-2472.947711	-2278.375214	EMD1
CCTCACACAACATACC-1	singlet	2	-804.5933121	-685.5835105	-268.1750528	EMD1
CCTCACACAGTTGCGC-1	singlet	1	-1282.061723	-493.9592272	-1141.389814	EMD1
CCTCACACATAGGTAA-1	singlet	0	-672.890827	-2214.968325	-2151.643461	EMD1
CCTCACAGTGGGTCAA-1	singlet	1	-1606.928416	-591.0708827	-1316.081082	EMD1
CCTCACAGTTAGCTAC-1	singlet	0	-352.0060271	-1006.93553	-1194.568531	EMD1
CCTCACAGTTGTGCCG-1	singlet	0	-309.2791665	-787.7165489	-804.1904589	EMD1
CCTCACAGTTTCGTAG-1	singlet	2	-610.4835617	-533.6107838	-234.9587493	EMD1
CCTCAGTAGAGAGTTT-1	singlet	2	-656.0122013	-543.3050699	-204.2072896	EMD1
CCTCAGTAGCTCTTCC-1	doublet	0/1	-2366.644661	-3913.285594	-4456.383979	EMD1
CCTCAGTCAAGTCATC-1	singlet	2	-61.09371161	-62.52800251	-26.6905537	EMD1
CCTCAGTCATAACAGA-1	singlet	0	-45.34426461	-158.5119732	-122.686639	EMD1
CCTCAGTGTGACTCGC-1	singlet	2	-96.41185096	-97.73087098	-48.54987768	EMD1
CCTCAGTGTTTCGGCG-1	singlet	0	-357.6957475	-1281.722339	-1316.070077	EMD1
CCTCAGTTCAAGTGTC-1	singlet	2	-696.5174419	-567.9840371	-245.4714778	EMD1
CCTCAGTTCCATCGTC-1	singlet	0	-267.3031387	-712.8052399	-793.1945837	EMD1
CCTCAGTTCGGTGAAG-1	singlet	0	-24.31309753	-87.4848406	-76.59217017	EMD1
CCTCAGTTCGTAGCCG-1	singlet	1	-3915.001857	-1280.676235	-2691.900285	EMD1
CCTCAGTTCTGTGCAA-1	singlet	1	-187.1537056	-100.1783245	-171.8338083	EMD1
CCTCATGCAAGTATCC-1	singlet	0	-293.001039	-1038.291693	-992.3904721	EMD1
CCTCATGGTAGTGATA-1	singlet	2	-2985.41709	-2403.926884	-994.2006161	EMD1
CCTCATGTCAGCGCAC-1	singlet	2	-446.3048185	-415.3861765	-209.7341014	EMD1
CCTCATGTCATGAGAA-1	singlet	1	-2711.378074	-814.1567275	-2301.003969	EMD1
CCTCATGTCGAAGCCC-1	singlet	0	-357.982852	-1062.058898	-951.7566699	EMD1
CCTCATGTCGTTGTGA-1	singlet	2	-301.319093	-250.8927931	-145.36488	EMD1
CCTCATGTCTATCGGA-1	singlet	2	-264.9340422	-219.0537983	-94.01292331	EMD1
CCTCCAAAGCGTGTCC-1	singlet	1	-2277.886266	-783.1868929	-1957.600995	EMD1
CCTCCAACAGCAGACA-1	singlet	0	-1168.008319	-3364.277174	-3321.344628	EMD1
CCTCCAACATACAGAA-1	singlet	0	-312.9305432	-862.0257461	-805.5298523	EMD1
CCTCCAACATGTGTCA-1	singlet	0	-213.9392528	-621.1555435	-554.8331944	EMD1
CCTCCAAGTAGGAAAG-1	singlet	2	-456.8852011	-390.193448	-181.3877168	EMD1
CCTCCAAGTATTAAGG-1	singlet	2	-545.3440368	-490.4094226	-282.6555575	EMD1
CCTCCAATCAGAGTTC-1	singlet	0	-716.5081535	-4116.309207	-3578.373638	EMD1
CCTCCTCAGCCTTTGA-1	singlet	2	-140.8003918	-116.1392083	-47.14024951	EMD1
CCTCCTCAGCTATCCA-1	singlet	1	-1826.105268	-824.7817314	-1389.639701	EMD1
CCTCCTCAGGGCCTCT-1	singlet	1	-1988.950297	-684.9230023	-1669.305813	EMD1
CCTCCTCAGTCACTAC-1	singlet	2	-757.9888757	-634.0077027	-334.914382	EMD1
CCTCCTCCACTACTTT-1	singlet	2	-423.9966405	-420.2325459	-195.4287992	EMD1

CCTCCTCGTCAGCTTA-1	singlet	2	-638.8691268	-566.2691921	-274.6643578	EMD1
CCTCCTCGTCGTTGGC-1	singlet	2	-764.102527	-598.286698	-281.8011851	EMD1
CCTCCTCGTGGAGGTT-1	singlet	2	-2808.159888	-2118.568384	-899.6146171	EMD1
CCTCCTCGTGGCTCTG-1	singlet	1	-1137.391832	-374.9455151	-826.1158696	EMD1
CCTCCTCGTTTGCCGG-1	singlet	2	-774.1661371	-548.6147458	-278.6758902	EMD1
CCTCCTCTCAAAGCCT-1	singlet	1	-2676.997802	-895.2983246	-2055.310115	EMD1
CCTCCTCTCAAGAGTA-1	singlet	2	-2558.577722	-2076.889673	-861.6235599	EMD1
CCTCCTCTCCGATAAC-1	singlet	0	-598.3641847	-1840.244045	-1679.230968	EMD1
CCTCCTCTCGGCATAT-1	singlet	2	-2221.696095	-1972.440915	-836.8567613	EMD1
CCTCCTCTCTTAGTTC-1	singlet	0	-605.4359446	-1804.72409	-1831.596403	EMD1
CCTCTAGAGACTTAAG-1	singlet	2	-4486.328964	-3513.861025	-1542.789568	EMD1
CCTCTAGAGCTGACAG-1	singlet	2	-558.9206527	-444.9218832	-189.8068528	EMD1
CCTCTAGAGTGAATAC-1	singlet	2	-3919.94748	-2995.052761	-1350.334179	EMD1
CCTCTAGCAGGACAGT-1	singlet	1	-758.2177121	-232.6314495	-579.0041464	EMD1
CCTCTAGCATACCAGT-1	singlet	0	-675.8763649	-2032.513223	-1791.057447	EMD1
CCTCTAGCATCGTGGC-1	singlet	2	-480.9117003	-466.0752147	-166.7042292	EMD1
CCTCTAGGTACCTAAC-1	singlet	0	-1170.080723	-2868.364784	-2767.27968	EMD1
CCTCTAGGTGCAATGG-1	singlet	1	-207.3125629	-93.15129617	-164.473002	EMD1
CCTCTAGGTGGTATGG-1	singlet	2	-385.1987808	-473.5142879	-188.1155234	EMD1
CCTCTAGTCAAGCGTT-1	singlet	2	-434.1921303	-383.4073011	-154.3512686	EMD1
CCTCTAGTCATTACGG-1	singlet	2	-425.5736311	-447.2295021	-217.1704582	EMD1
CCTCTAGTCCCATTCG-1	singlet	2	-444.1729557	-344.0337568	-189.0619198	EMD1
CCTCTAGTCCGTACGG-1	unassigned	2	-6.293057126	-11.00491079	-4.599377077	EMD1
CCTCTAGTCGACACCG-1	singlet	1	-4541.303233	-1454.945028	-3743.039644	EMD1
CCTCTAGTCGCCACTT-1	singlet	1	-1882.900158	-575.4763247	-1358.761978	EMD1
CCTCTAGTCTCGGTCT-1	singlet	2	-549.3815111	-486.0117685	-215.4227943	EMD1
CCTCTCCAGACTCTAC-1	singlet	1	-3440.354966	-1142.721716	-2332.625203	EMD1
CCTCTCCCAACTACGT-1	singlet	2	-749.2680338	-599.2622658	-268.6886898	EMD1
CCTCTCCCACTGCGTG-1	singlet	1	-4674.224868	-1285.57836	-3750.085083	EMD1
CCTCTCCCATCTTAGG-1	singlet	2	-740.1352062	-624.3115442	-308.8838766	EMD1
CCTCTCCGTAGCTTAC-1	singlet	0	-9.997947975	-58.2032951	-59.24948648	EMD1
CCTCTCCGTGTCATCA-1	singlet	2	-509.4217858	-413.1630443	-227.4144533	EMD1
CCTCTCCTCCACTAGA-1	singlet	0	-1007.502098	-2869.909045	-2710.472007	EMD1
CCTCTCCTCTGCCCTA-1	singlet	0	-1341.768203	-3802.513854	-3824.158205	EMD1
CCTGCATAGAATACAC-1	singlet	2	-1478.038072	-1255.693108	-567.4973029	EMD1
CCTGCATAGGAGTACC-1	singlet	2	-471.3946091	-368.2765456	-187.0635302	EMD1
CCTGCATAGGTTCCAT-1	singlet	2	-2000.278293	-1694.84015	-878.8116069	EMD1
CCTGCATAGTGGTCAG-1	singlet	1	-441.2874445	-120.4489096	-412.5432537	EMD1
CCTGCATCAAGATCCT-1	doublet	0/2	-38.40917353	-57.16458198	-40.88802995	EMD1
CCTGCATGTCCAACGC-1	singlet	2	-945.7968362	-644.3614278	-354.1614956	EMD1
CCTGCATGTGCCTATA-1	singlet	2	-175.4551905	-161.9251302	-69.44538128	EMD1
CCTGCATGTGGTATGG-1	singlet	2	-1565.978944	-1433.290904	-488.7827526	EMD1
CCTGCATTCAGGACGA-1	singlet	0	-38.00215176	-148.6828773	-160.9284156	EMD1
CCTGCATTCGCCTAGG-1	singlet	0	-303.8301998	-875.5454117	-803.1053552	EMD1
CCTGCATTCTATGTGG-1	singlet	2	-615.9638938	-475.3949145	-250.5403528	EMD1
CCTGTTGAGAGGTTTA-1	singlet	2	-1101.700297	-874.6995473	-376.1061	EMD1
CCTGTTGCAATGTGGG-1	singlet	0	-401.9327359	-1002.946849	-991.9475243	EMD1
CCTGTTGGTCGGATTT-1	singlet	2	-100.0171224	-82.9824544	-32.99988937	EMD1
CCTGTTGGTCTTGAGT-1	singlet	0	-1673.257924	-4146.262517	-4016.945017	EMD1
CCTGTTGGTTTACACG-1	singlet	2	-1705.92433	-1502.339617	-533.932027	EMD1
CCTGTTGTCGGAGTGA-1	singlet	2	-393.7118567	-295.9428759	-134.8178421	EMD1
CCTGTTGTCGTCGCTT-1	singlet	0	-845.4644435	-2499.010286	-2468.630597	EMD1
CCTTCAGAGCACTCTA-1	doublet	1/0	-320.6175394	-257.2083613	-330.373547	EMD1
CCTTCAGAGCCATGCC-1	singlet	2	-205.1768536	-154.237274	-94.80005297	EMD1
CCTTCAGAGGTCGAGT-1	singlet	0	-785.5072533	-2641.797441	-2310.157582	EMD1
CCTTCAGAGGTGATCG-1	singlet	1	-1106.260785	-375.338078	-865.3523139	EMD1
CCTTCAGAGTGCCCGT-1	singlet	0	-77.39471939	-342.2202038	-320.2329057	EMD1
CCTTCAGCAAAGCTCT-1	singlet	0	-1427.442719	-3991.262069	-3766.309547	EMD1
CCTTCAGCAAATCGTC-1	singlet	2	-968.5174701	-886.2110324	-392.1145298	EMD1
CCTTCAGCAACACAGG-1	singlet	2	-143.7340929	-121.1553347	-67.76184224	EMD1
CCTTCAGCACCGTACG-1	singlet	0	-495.708095	-1496.163185	-1409.058274	EMD1
CCTTCAGCACGAGGAT-1	singlet	1	-1136.872908	-394.7052376	-958.2233131	EMD1
CCTTCAGCACGCAGTC-1	singlet	1	-1897.3442	-616.6337099	-1608.603262	EMD1
CCTTCAGCATAATCGC-1	singlet	2	-292.5870871	-260.2399278	-143.8612903	EMD1
CCTTCAGGTCACTCGG-1	singlet	2	-280.3684433	-211.9455866	-100.0262822	EMD1
CCTTCAGGTCAGGCAA-1	singlet	1	-5432.574606	-1907.262403	-4359.768109	EMD1
CCTTCAGTCGATCCAA-1	singlet	0	-16.25795531	-38.22120931	-54.53923089	EMD1
CCTTCAGTCTGCTCTG-1	singlet	2	-647.0175393	-638.1220572	-256.7962262	EMD1
CCTTGTGAGAGCCATG-1	singlet	0	-317.7258732	-1093.091629	-955.1438476	EMD1
CCTTGTGAGGGTACAC-1	singlet	2	-377.6867255	-326.9753253	-170.3350021	EMD1
CCTTGTGAGTCTGGAG-1	singlet	2	-315.2502687	-287.4465569	-139.072095	EMD1
CCTTGTGAGTCTGTAC-1	singlet	2	-497.1121802	-475.6925558	-241.6912518	EMD1
CCTTGTGCAATGAGCG-1	singlet	0	-1342.123378	-4177.759963	-4061.091875	EMD1
CCTTGTGCAGAGGAAA-1	singlet	0	-554.3838526	-1658.051927	-1578.31118	EMD1
CCTTGTGGTACCATAC-1	singlet	2	-69.84414871	-64.69194528	-22.6689937	EMD1
CCTTGTGTCACCGGGT-1	singlet	2	-2881.385375	-2173.838363	-801.8397967	EMD1
CCTTGTGTCGAACCAT-1	singlet	0	-358.6339564	-1419.563359	-1231.201993	EMD1
CCTTGTGTCGAGTACT-1	singlet	0	-121.0870141	-373.0102745	-386.3385515	EMD1
CCTTGTGTCTCCCATG-1	singlet	1	-1915.945463	-923.2305635	-1623.298645	EMD1
CCTTGTGTCTTCGCTG-1	singlet	2	-613.141948	-496.8049605	-187.6613153	EMD1
CCTTTGGAGAAACTAC-1	singlet	2	-349.2471715	-269.2917439	-147.4744252	EMD1
CCTTTGGAGTAATCCC-1	singlet	2	-971.115433	-792.3757422	-356.1820935	EMD1
CCTTTGGAGTCAGCGA-1	singlet	2	-524.948095	-408.2369593	-186.5201227	EMD1
CCTTTGGCAATCGCGC-1	singlet	0	-509.5774868	-1643.527345	-1525.645279	EMD1
CCTTTGGCAATGCTCA-1	singlet	0	-281.3141413	-767.3090727	-740.4511119	EMD1
CCTTTGGGTGCGTTTA-1	singlet	1	-1455.546396	-384.5700422	-1106.836241	EMD1
CCTTTGGGTGGATCGA-1	singlet	2	-291.7080063	-301.0099614	-146.223246	EMD1
CCTTTGGGTGGGTCAA-1	singlet	2	-960.8661841	-864.1753279	-401.0064086	EMD1
CCTTTGGGTGTTGAGG-1	singlet	2	-2123.925243	-1666.018545	-732.04123	EMD1
CGAAGGAGTTAATGAG-1	singlet	2	-506.7321652	-398.5737755	-186.4587645	EMD1
CGAAGGAGTTCCAGGC-1	singlet	1	-3178.949196	-1349.059384	-2264.122876	EMD1
CGAAGGATCATGTCTT-1	singlet	1	-3161.919173	-964.4945486	-2352.965202	EMD1
CGAAGGATCTTCTAAC-1	singlet	0	-1408.891894	-4403.808044	-4404.860543	EMD1
CGAAGTTAGCCGCTTG-1	singlet	2	-2334.078185	-1830.596297	-879.1453986	EMD1
CGAAGTTCAGACACCC-1	singlet	0	-147.5523848	-555.8592527	-494.2782829	EMD1
CGAAGTTCATCAACCA-1	singlet	0	-165.0321227	-401.1642817	-361.3130953	EMD1
CGAAGTTCATGGCTGC-1	singlet	1	-262.5714864	-73.10167955	-197.7147803	EMD1
CGAAGTTGTGCCGTAC-1	singlet	2	-523.10277	-505.6598374	-252.4617192	EMD1
CGAAGTTGTGGATTTC-1	singlet	1	-504.5690972	-151.0823317	-399.5461944	EMD1
CGAATTGAGAGGTTTA-1	singlet	0	-558.4323065	-1757.051639	-1661.168869	EMD1
CGAATTGAGATAACGT-1	singlet	2	-650.5338286	-503.8846176	-249.0312656	EMD1
CGAATTGCATGATAGA-1	singlet	0	-620.1850404	-1690.794538	-1746.785676	EMD1
CGAATTGGTATCAGGG-1	singlet	2	-641.2901353	-498.0762827	-210.9783765	EMD1
CGAATTGTCTATCGCC-1	unassigned	2/0	-6.292476031	-3.784651497	-4.756681651	EMD1
CGACAGCAGCCAGACA-1	singlet	0	-567.1989966	-1809.802377	-1585.746962	EMD1
CGACAGCAGCGAAACC-1	singlet	0	-243.7470587	-664.5719953	-643.199367	EMD1
CGACAGCAGCGTCAAG-1	singlet	2	-1160.831447	-857.9824206	-394.888116	EMD1
CGACAGCAGGCTCTCG-1	singlet	0	-157.8716196	-386.4692904	-416.0214424	EMD1
CGACAGCAGTCTAACC-1	singlet	0	-888.2151737	-2876.073838	-2995.748821	EMD1
CGACAGCCAATTGCAC-1	singlet	1	-361.5481958	-89.11570463	-276.1326725	EMD1

CGACAGCCACAACGAG-1	singlet	2	-412.5428504	-343.1824829	-188.008322	EMD1
CGACAGCCACCCTGAG-1	singlet	1	-114.9073687	-35.63949437	-84.29139825	EMD1
CGACAGCCAGCAGATG-1	singlet	2	-287.193777	-284.3767686	-134.2765967	EMD1
CGACAGCGTACGCTTA-1	singlet	2	-4141.835099	-3292.610846	-1244.318511	EMD1
CGACAGCGTATCGCTA-1	singlet	1	-4522.090745	-1366.85145	-3466.03896	EMD1
CGACAGCGTTCGTTCC-1	singlet	0	-187.1707197	-815.6052126	-734.290773	EMD1
CGACAGCGTTGGGTTT-1	singlet	2	-411.8369332	-342.4620965	-158.9615713	EMD1
CGACAGCTCGAGAGAC-1	singlet	1	-1316.036388	-359.6773099	-1213.425194	EMD1
CGAGAAGAGTGCAGCA-1	singlet	2	-509.8014558	-369.8623708	-165.5539318	EMD1
CGAGAAGCACAAATGA-1	singlet	1	-534.7501952	-211.9388114	-624.1023325	EMD1
CGAGAAGCATAATCCG-1	singlet	0	-11.83606759	-54.0880695	-57.86703708	EMD1
CGAGAAGCATCTGTTT-1	singlet	1	-2144.073659	-613.3680837	-1729.987819	EMD1
CGAGAAGGTAGCTTTG-1	singlet	0	-234.0061293	-709.0896042	-615.1466151	EMD1
CGAGAAGTCAAGTCGT-1	singlet	0	-307.4085457	-1100.658792	-1130.86607	EMD1
CGAGAAGTCCCTCAAC-1	singlet	2	-647.2821996	-523.3104518	-248.2811171	EMD1
CGAGAAGTCCTTCGAC-1	singlet	1	-5243.720331	-1842.674091	-4212.716848	EMD1
CGAGGAAAGCCGATCC-1	singlet	2	-259.0195077	-231.271267	-113.6864235	EMD1
CGAGGAACACATGTTG-1	singlet	2	-422.0891382	-344.8966831	-180.7708768	EMD1
CGAGGAAGTGACAGGT-1	singlet	0	-218.3249232	-684.8147824	-654.4330128	EMD1
CGAGGAAGTGTAGCAG-1	doublet	45293	-2197.101779	-1183.04297	-1526.793272	EMD1
CGAGGAATCGACACCG-1	singlet	0	-427.83523	-1543.703333	-1456.514618	EMD1
CGAGGAATCGATTCCC-1	singlet	0	-626.5178022	-1790.658726	-1763.964624	EMD1
CGAGGAATCGGTCGGT-1	singlet	0	-644.273864	-2365.822536	-2389.532029	EMD1
CGAGGAATCGGTCTAA-1	doublet	2/0	-1819.505582	-2381.033588	-1764.996958	EMD1
CGAGGCTAGAAGTCCG-1	singlet	1	-54.06632525	-30.48429261	-52.99684804	EMD1
CGAGGCTAGCAGTAAT-1	singlet	0	-249.9490949	-622.9685605	-614.455926	EMD1
CGAGGCTAGGAGAGGC-1	singlet	1	-1617.027637	-348.5804714	-1354.460539	EMD1
CGAGGCTAGGCACTCC-1	singlet	1	-1721.267936	-684.4560302	-1432.169761	EMD1
CGAGGCTAGTTTAGGA-1	singlet	2	-639.7552186	-533.8498609	-265.0930445	EMD1
CGAGGCTCAAAGCTAA-1	singlet	2	-432.3354395	-364.5662336	-166.7971436	EMD1
CGAGGCTCAATCGCAT-1	singlet	2	-1685.981215	-1706.895454	-666.4537326	EMD1
CGAGGCTCACTGCTTC-1	singlet	2	-615.2204159	-413.3276654	-207.0993364	EMD1
CGAGGCTCACTTGACA-1	singlet	2	-1303.20733	-1205.06827	-564.7396688	EMD1
CGAGGCTCATGTCTAG-1	singlet	0	-283.438293	-805.7513116	-947.4969474	EMD1
CGAGGCTGTTCTTGCC-1	singlet	1	-2477.279966	-782.9497955	-1801.073866	EMD1
CGAGGCTTCCATGATG-1	singlet	1	-11293.04044	-2857.78729	-8782.455898	EMD1
CGAGGCTTCCGCACGA-1	singlet	1	-790.6329367	-302.9826832	-682.0219222	EMD1
CGAGGCTTCGTGGACC-1	singlet	0	-290.7998058	-867.6821864	-765.0875098	EMD1
CGAGTGCAGACAACTA-1	singlet	0	-812.6169909	-2712.703892	-2568.583583	EMD1
CGAGTGCAGCACCTGC-1	singlet	0	-220.1050316	-699.3081928	-838.2393458	EMD1
CGAGTGCAGTGAGGTC-1	singlet	0	-712.0481392	-2157.055191	-2163.879258	EMD1
CGAGTGCCACCTGATA-1	singlet	2	-242.3270414	-221.2355612	-97.95743815	EMD1
CGAGTGCCAGAGATGC-1	singlet	2	-1094.98297	-880.2791501	-390.2422269	EMD1
CGAGTGCCAGGAAGTC-1	singlet	1	-1496.621031	-473.1863583	-1154.492506	EMD1
CGAGTGCGTATCGAAA-1	singlet	2	-211.2724289	-183.2295367	-103.8952033	EMD1
CGAGTGCGTTAGGCTT-1	singlet	0	-1863.734631	-6460.998438	-6462.051337	EMD1
CGAGTTAAGCGGTAAC-1	singlet	0	-251.2447433	-643.360862	-713.8646275	EMD1
CGAGTTAAGGGTATAT-1	singlet	2	-1602.098015	-1299.873286	-568.3989989	EMD1
CGAGTTAAGTTGAAGT-1	singlet	1	-613.0881148	-202.13089	-472.6014208	EMD1
CGAGTTACAAAGCACG-1	singlet	2	-610.7908687	-555.0584671	-248.6531935	EMD1
CGAGTTACAACCGTGC-1	singlet	0	-251.8784365	-873.0963467	-952.076339	EMD1
CGAGTTACAACCTAAC-1	singlet	1	-3280.810572	-1026.065415	-2422.738578	EMD1
CGAGTTACACTAACCA-1	singlet	2	-817.137202	-667.6272085	-300.7584577	EMD1
CGAGTTACAGTTGTTG-1	singlet	0	-446.2489383	-1258.1116	-1198.786004	EMD1
CGAGTTAGTATAGGAT-1	singlet	2	-4048.431363	-3691.7582	-1183.18811	EMD1
CGAGTTAGTTGTTGCA-1	singlet	2	-479.5579833	-427.7500259	-187.4894752	EMD1
CGAGTTATCATAGCAC-1	singlet	2	-669.7465388	-516.1794049	-236.8958322	EMD1
CGAGTTATCCGTGGGT-1	singlet	1	-580.2182278	-216.8754171	-469.0547247	EMD1
CGAGTTATCTACTGCC-1	singlet	0	-1462.083385	-4492.300048	-4555.540086	EMD1
CGAGTTATCTGCGATA-1	singlet	0	-282.142891	-760.1076732	-704.1555841	EMD1
CGATCGGAGAAGTATC-1	singlet	2	-442.363233	-369.8431691	-164.2597299	EMD1
CGATCGGAGCGTTACT-1	singlet	2	-317.2555629	-263.9809267	-129.6829123	EMD1
CGATCGGAGGTAAGAG-1	singlet	2	-499.723084	-446.3870345	-217.7705407	EMD1
CGATCGGCAAGGCAAC-1	singlet	1	-641.8930913	-235.7042116	-579.262214	EMD1
CGATCGGCACCCTAGG-1	singlet	1	-264.1190098	-86.18858569	-198.7916919	EMD1
CGATCGGGTAGCTTGT-1	singlet	1	-3037.493686	-916.1698882	-2398.505295	EMD1
CGATCGGGTTAGTTCG-1	singlet	0	-414.604121	-1268.260331	-1283.247055	EMD1
CGATCGGTCACCTGGG-1	singlet	2	-526.7044878	-364.1875712	-210.5174563	EMD1
CGATCGGTCCATCGTC-1	singlet	2	-156.8263936	-127.5673503	-63.19521973	EMD1
CGATCGGTCGATTTCT-1	singlet	0	-73.0619506	-252.7133604	-193.6567587	EMD1
CGATGCGAGTTAACGA-1	singlet	0	-943.4761962	-2794.522529	-2912.205636	EMD1
CGATGCGCAAGAAATC-1	doublet	45323	-589.7226639	-438.4684367	-296.3192741	EMD1
CGATGCGCACTCCGGA-1	singlet	2	-1108.625699	-975.408556	-359.9036913	EMD1
CGATGCGCAGCGTTTA-1	singlet	2	-378.9027261	-358.4588457	-192.3687515	EMD1
CGATGCGGTAGCACGA-1	singlet	1	-1076.747103	-326.8692764	-966.0629925	EMD1
CGATGCGGTCGCCACA-1	singlet	2	-380.2156891	-298.483642	-125.9799179	EMD1
CGATGCGGTCGTTGCG-1	singlet	2	-283.9697777	-254.9396266	-94.3654914	EMD1
CGATGCGGTGTCCGGT-1	singlet	1	-2803.706602	-1002.018816	-2245.180216	EMD1
CGATGCGGTTGGTAGG-1	singlet	1	-2151.875396	-631.2311607	-1697.242704	EMD1
CGATGGCAGACAACTA-1	singlet	0	-138.5881298	-337.3941279	-333.4902114	EMD1
CGATGGCAGCGAAACC-1	singlet	1	-2620.92308	-909.2361614	-2051.543524	EMD1
CGATGGCAGGAGTACC-1	singlet	0	-518.2491621	-1892.116321	-1832.911477	EMD1
CGATGGCCACAATTCG-1	singlet	2	-1482.648301	-1353.564123	-566.7154092	EMD1
CGATGGCCACGCAAAG-1	singlet	1	-1475.629678	-476.1287076	-1105.325771	EMD1
CGATGGCGTACAATAG-1	singlet	0	-698.2482508	-2363.236136	-3014.23724	EMD1
CGATGGCGTAGGGTAC-1	singlet	0	-1008.270983	-3092.793123	-3122.199098	EMD1
CGATGGCGTTGCCGAC-1	singlet	2	-3402.094627	-2550.233967	-1003.519795	EMD1
CGATGGCTCGGCATAT-1	singlet	1	-205.2598963	-72.9637627	-169.1708308	EMD1
CGATGGCTCTGTGTGA-1	singlet	2	-602.7666156	-535.7132651	-249.5832557	EMD1
CGATGGCTCTTGGTGA-1	singlet	1	-2652.283154	-837.4306904	-2069.420074	EMD1
CGCAGGTAGACGCCAA-1	singlet	0	-150.1684433	-377.5108101	-385.2665271	EMD1
CGCAGGTCAATCCAGT-1	singlet	2	-466.8998224	-471.0468926	-198.1082791	EMD1
CGCAGGTCACTTCTCG-1	singlet	2	-1778.150159	-1602.994225	-736.4670138	EMD1
CGCAGGTGTACTCCGG-1	singlet	2	-477.1059647	-425.3570816	-215.4792571	EMD1
CGCAGGTGTGAATATG-1	singlet	1	-539.8087801	-214.7375256	-579.1035471	EMD1
CGCAGGTGTTGAGTCT-1	singlet	2	-451.5371714	-375.9398053	-180.3890389	EMD1
CGCAGGTTCAGTGTCA-1	singlet	0	-265.7148582	-895.3546204	-826.384588	EMD1
CGCATAAAGATTCGCT-1	singlet	0	-879.647687	-2731.776307	-2830.692881	EMD1
CGCATAAAGTCCCTAA-1	singlet	2	-671.4326042	-529.7598649	-239.0825905	EMD1
CGCATAACAAGCAGGT-1	singlet	1	-2030.26715	-579.4498885	-1552.588167	EMD1
CGCATAAGTAGGACTG-1	singlet	2	-308.8530201	-278.2577133	-155.3800535	EMD1
CGCATAAGTAGGCAGT-1	singlet	0	-188.0139264	-562.9207507	-577.6679821	EMD1
CGCATAAGTCTGCATA-1	singlet	1	-423.5137676	-178.4456886	-428.0192975	EMD1
CGCATAATCCACGGGT-1	singlet	1	-2785.010601	-1066.574726	-2353.788094	EMD1
CGCATAATCCTGTAGA-1	singlet	2	-1267.800927	-1068.743416	-377.933215	EMD1
CGCATAATCGGTCTAA-1	singlet	2	-386.9586563	-335.1611492	-170.0693493	EMD1
CGCATGGAGGCTAGCA-1	singlet	2	-809.0996924	-689.2388678	-333.5846794	EMD1
CGCATGGCAACAAGTA-1	singlet	0	-146.7715761	-480.7810848	-440.2616309	EMD1
CGCATGGCAATAGAGT-1	singlet	2	-7497.816414	-7127.379359	-1809.57079	EMD1
CGCATGGCAGTGGGTA-1	singlet	0	-233.8191268	-782.9213877	-820.6530347	EMD1

CGCATGGGTACGAGCA-1	singlet	0	-265.3581623	-917.6336596	-877.0852117	EMD1
CGCCAGAAGAGAGCAA-1	singlet	2	-737.8595042	-705.5493207	-314.033365	EMD1
CGCCAGAAGATGAAGG-1	singlet	0	-788.3465295	-2526.906097	-2305.2898	EMD1
CGCCAGAAGCGTATAA-1	singlet	2	-404.0347337	-307.7876857	-150.7511228	EMD1
CGCCAGAAGCTGCCAC-1	singlet	2	-292.5050977	-355.316522	-156.3100836	EMD1
CGCCAGAAGGTAGCAC-1	singlet	1	-374.1216277	-123.9309154	-335.446394	EMD1
CGCCAGACAAAGCTAA-1	singlet	2	-432.642757	-360.9951284	-187.3271882	EMD1
CGCCAGACAACAGAGC-1	singlet	2	-341.7618305	-269.1796136	-133.4981145	EMD1
CGCCAGACACTAACCA-1	singlet	2	-2341.464171	-1879.884581	-724.9373788	EMD1
CGCCAGATCACACCCT-1	singlet	2	-514.5233548	-381.6585662	-225.6507796	EMD1
CGCCATTAGAAATGGG-1	singlet	1	-1308.661517	-406.406033	-1027.271953	EMD1
CGCCATTAGCTGTTCA-1	singlet	2	-469.5428225	-439.5441869	-190.0916709	EMD1
CGCCATTAGGCACCAA-1	singlet	1	-1960.770947	-695.9191346	-1580.975247	EMD1
CGCCATTAGTGCCCGT-1	singlet	2	-2020.68322	-1685.907258	-676.1329181	EMD1
CGCCATTCACCGTCGA-1	singlet	1	-2148.832026	-543.351048	-1654.373838	EMD1
CGCCATTGTACCGTGC-1	singlet	2	-442.4495255	-389.8177646	-147.0157026	EMD1
CGCCATTGTATCGTGT-1	singlet	2	-392.2967916	-304.5889859	-131.6945179	EMD1
CGCCATTGTCCGGACT-1	singlet	2	-448.593517	-418.3615946	-194.1755898	EMD1
CGCCATTGTTACCTTT-1	singlet	0	-1021.006359	-3058.328764	-3059.165164	EMD1
CGCCATTGTTATGACC-1	singlet	2	-2090.585274	-1875.644315	-794.2130017	EMD1
CGCCATTTCGACATTG-1	singlet	2	-502.2108883	-420.0324678	-190.8419463	EMD1
CGCCATTTCGGCATCG-1	singlet	0	-106.8999044	-551.3504863	-412.2425636	EMD1
CGCCATTTCGGCGATC-1	singlet	2	-482.1836749	-345.96246	-190.831932	EMD1
CGCCATTTCTACTATC-1	singlet	0	-349.6931882	-1513.202769	-1304.937862	EMD1
CGCGTGAAGCACGTCC-1	singlet	2	-736.5418762	-605.5570115	-272.8695128	EMD1
CGCGTGAAGCACTCCG-1	singlet	0	-263.8498887	-868.0905224	-829.377887	EMD1
CGCGTGAAGGGAGTGG-1	singlet	1	-344.6106748	-114.6076706	-356.0137505	EMD1
CGCGTGACAAACTAGA-1	singlet	0	-6.486529871	-9.815043272	-107.4942164	EMD1
CGCGTGACAAGTCGTT-1	singlet	0	-890.2514469	-2872.51841	-2829.911336	EMD1
CGCGTGACACAAGTGG-1	singlet	2	-280.2288176	-238.3402483	-109.5028716	EMD1
CGCGTGAGTAAGTTAG-1	singlet	0	-585.0520657	-1520.975712	-1615.742232	EMD1
CGCGTGAGTACTAACC-1	singlet	2	-376.2716716	-292.1044102	-156.0123203	EMD1
CGCGTGAGTACTAAGA-1	singlet	2	-817.4217029	-649.2455161	-313.9167647	EMD1
CGCGTGAGTATCCTCC-1	singlet	0	-633.1308161	-1958.891518	-1802.709763	EMD1
CGCGTGAGTGTCTAAC-1	doublet	0/1	-603.9558748	-888.7729933	-804.2422812	EMD1
CGCGTGAGTTGTCAGT-1	singlet	0	-132.7195374	-408.471502	-440.0509254	EMD1
CGCGTGATCCGACAGC-1	singlet	0	-694.2835873	-2429.799796	-2156.573442	EMD1
CGCGTGATCGTGAGAG-1	singlet	1	-1200.181197	-425.4502519	-1030.593632	EMD1
CGCGTGATCTAAACGC-1	singlet	2	-418.9447652	-372.8169217	-156.1425155	EMD1
CGGAACCAGCGTCTCG-1	singlet	0	-27.8063967	-138.2014631	-101.9436267	EMD1
CGGAACCAGCTGCGAA-1	doublet	0/2	-921.7755805	-1554.927993	-1194.088231	EMD1
CGGAACCAGGGAGGTG-1	singlet	1	-904.6809995	-260.1125886	-684.9583051	EMD1
CGGAACCAGGTCTACT-1	singlet	0	-406.817228	-1161.273726	-1123.280185	EMD1
CGGAACCAGGTGCAGT-1	singlet	1	-1425.440651	-466.1940065	-1110.690503	EMD1
CGGAACCAGTCCTGCG-1	singlet	0	-539.7078051	-1695.80743	-1625.928788	EMD1
CGGAACCAGTTCGCAT-1	singlet	2	-401.2039427	-309.2211863	-144.7244958	EMD1
CGGAACCCACTCCCTA-1	singlet	1	-3856.948108	-1021.020823	-3305.402658	EMD1
CGGAACCCATAATCGC-1	singlet	1	-2705.583175	-964.4155828	-2374.921692	EMD1
CGGAACCGTCAACATC-1	singlet	2	-249.8173147	-218.677107	-124.2820737	EMD1
CGGAACCGTCGGTGTC-1	singlet	0	-463.9689968	-1467.713516	-1483.421808	EMD1
CGGAACCGTGACTGTT-1	singlet	2	-682.0569286	-441.4678665	-233.2898187	EMD1
CGGAACCGTGCCGTAC-1	singlet	2	-2161.845785	-2089.558138	-707.2236628	EMD1
CGGAACCGTGTCGATT-1	singlet	1	-1552.788362	-437.3840146	-1051.589822	EMD1
CGGAACCTCGCTACGG-1	singlet	0	-373.3402331	-1178.499165	-1140.874333	EMD1
CGGAACCTCGTCTCAC-1	doublet	2/0	-1328.840971	-1405.262503	-878.5749758	EMD1
CGGAACCTCTATCGTT-1	singlet	2	-1174.795427	-972.9028483	-396.924337	EMD1
CGGAATTAGTATGACA-1	singlet	2	-499.6770391	-524.4819416	-226.7183832	EMD1
CGGAATTAGTGGATTA-1	singlet	1	-1263.548457	-516.2510614	-1152.861911	EMD1
CGGAATTAGTTGTCAC-1	singlet	0	-382.2019342	-1074.271507	-969.9877775	EMD1
CGGAATTCACATTCGA-1	singlet	2	-400.5150211	-308.3440307	-143.1424281	EMD1
CGGAATTCACGGTGCT-1	singlet	2	-300.8355777	-217.4655009	-92.04950826	EMD1
CGGAATTCATAGGAGC-1	singlet	0	-1105.25906	-3703.864947	-3215.063571	EMD1
CGGAATTGTCGCACGT-1	singlet	2	-2192.046071	-1915.185007	-605.9146143	EMD1
CGGAATTGTCTGTGGC-1	singlet	2	-2901.912015	-2423.905341	-1027.53811	EMD1
CGGAATTGTGGGCTCT-1	singlet	2	-1096.978186	-944.9027912	-422.3563096	EMD1
CGGAATTGTGTTAACC-1	singlet	0	-356.0888389	-898.3933023	-1128.358897	EMD1
CGGAATTTCAAGCCCG-1	singlet	2	-1231.674895	-1097.97569	-383.8100556	EMD1
CGGAATTTCACCTTGC-1	singlet	2	-630.4573556	-546.1560147	-258.4746459	EMD1
CGGAATTTCGCATTAG-1	singlet	1	-4046.793914	-635.5823075	-3502.997713	EMD1
CGGAATTTCTCACCCA-1	singlet	0	-635.251519	-2612.657056	-2134.000041	EMD1
CGGACACAGCGAGTCA-1	singlet	2	-1507.220705	-1279.118144	-491.9236138	EMD1
CGGACACAGTACCGGA-1	singlet	1	-1814.875365	-589.5636828	-1421.435748	EMD1
CGGACACAGTTCCTGA-1	singlet	0	-733.9859182	-2420.546639	-2323.607557	EMD1
CGGACACGTACTCGCG-1	singlet	2	-66.36311711	-60.78486503	-21.49565636	EMD1
CGGACACGTATCATGC-1	singlet	1	-3497.746763	-972.5651816	-2919.280416	EMD1
CGGACACGTGAATGAT-1	singlet	2	-3471.385471	-2812.011389	-1076.920985	EMD1
CGGACACGTGGTATGG-1	singlet	1	-984.5614609	-405.9598977	-861.0835011	EMD1
CGGACACTCAAGTTGC-1	singlet	2	-307.4588055	-187.5069051	-85.30089362	EMD1
CGGACACTCCTCTAGC-1	singlet	2	-736.695587	-645.7713672	-262.2447862	EMD1
CGGAGAAAGATCCTAC-1	singlet	2	-624.8130323	-528.2354099	-242.9123283	EMD1
CGGAGAAAGCCTCCAG-1	singlet	2	-2818.66319	-2254.187622	-978.0073711	EMD1
CGGAGAACAGCTCATA-1	singlet	2	-3447.946893	-2705.887131	-1178.859609	EMD1
CGGAGAACAGCTGAAG-1	singlet	1	-1051.293911	-338.4843128	-747.9078166	EMD1
CGGAGAACATACTGTG-1	singlet	2	-14136.30239	-11095.53718	-4438.224455	EMD1
CGGAGAAGTCTTACAG-1	singlet	0	-184.3771925	-521.1617318	-523.5835334	EMD1
CGGAGAATCCATCGTC-1	singlet	0	-352.6865681	-916.834094	-913.4262917	EMD1
CGGAGAATCCGCACTT-1	singlet	1	-3712.231154	-822.4361477	-2705.210358	EMD1
CGGAGAATCTCTATGT-1	singlet	0	-877.5014936	-3213.361343	-3048.41486	EMD1
CGGCAGTAGCCAAGGT-1	singlet	2	-480.2482389	-509.3719205	-222.2231206	EMD1
CGGCAGTAGTCTCGTA-1	singlet	2	-464.5342833	-389.221196	-193.6415368	EMD1
CGGCAGTCACTTCAGA-1	singlet	0	-395.5733581	-1353.558198	-1272.552715	EMD1
CGGCAGTGTACCTAGT-1	singlet	1	-2549.770723	-788.7360195	-2062.611287	EMD1
CGGCAGTTCCAAACCA-1	singlet	0	-997.8285877	-2797.543125	-2898.497713	EMD1
CGGGACTAGATCACCT-1	singlet	0	-577.9093748	-1676.642217	-1522.383125	EMD1
CGGGACTAGCCAACCC-1	singlet	0	-371.1642583	-1270.299907	-1420.789952	EMD1
CGGGACTAGGGCCAAT-1	singlet	2	-1057.367489	-1063.114897	-417.9990412	EMD1
CGGGACTAGTGAACAT-1	singlet	2	-1557.469514	-1467.375943	-562.1067057	EMD1
CGGGACTCATAACGGG-1	singlet	0	-289.3697141	-755.9752408	-869.2596874	EMD1
CGGGACTCATAAGATG-1	singlet	0	-700.3429321	-1939.878705	-1930.016439	EMD1
CGGGACTCATGACTAC-1	singlet	0	-87.03826715	-345.007556	-413.2123394	EMD1
CGGGACTGTACGGTTT-1	singlet	1	-4439.773825	-966.1833216	-3092.747356	EMD1
CGGGACTGTGGAAATT-1	singlet	0	-875.0872747	-2441.85946	-2517.690212	EMD1
CGGGACTGTTGAAGTA-1	singlet	2	-911.0843218	-709.2738731	-296.0466558	EMD1
CGGGACTTCAACCTTT-1	singlet	2	-361.8652369	-298.4897981	-122.8845889	EMD1
CGGGACTTCACTAGCA-1	singlet	0	-520.9636667	-1418.628688	-1526.721386	EMD1
CGGGACTTCATACGAC-1	singlet	1	-2454.710081	-697.2855626	-2098.654411	EMD1
CGGGACTTCATTCACT-1	singlet	0	-351.782151	-1127.230989	-1033.708921	EMD1
CGGGACTTCGTCTAAG-1	singlet	2	-444.6320206	-433.94726	-165.6653478	EMD1
CGGGACTTCTCCAAGA-1	singlet	2	-7176.545341	-5627.456509	-2582.572315	EMD1
CGGGACTTCTTGAACG-1	singlet	2	-753.6256388	-582.2054806	-217.8409106	EMD1

CGGGCATAGGCCTGCT-1	singlet	0	-8.504848607	-17.73464739	-12.22434401	EMD1
CGGGCATCAATCTAGC-1	singlet	2	-698.9837958	-624.347209	-238.7832408	EMD1
CGGGCATCAATTCACG-1	singlet	0	-188.7861688	-610.6102443	-630.1695175	EMD1
CGGGCATCACATTACG-1	singlet	2	-373.7581404	-324.5232637	-164.333544	EMD1
CGGGCATCAGAGCCCT-1	singlet	2	-564.6151188	-386.9463634	-213.6370366	EMD1
CGGGCATCAGCTGTTA-1	singlet	1	-2874.481961	-674.9582784	-1925.790054	EMD1
CGGGCATTCATGGGAG-1	singlet	1	-551.5759202	-106.1102751	-429.9744702	EMD1
CGGGCATTCTCAAAGC-1	singlet	0	-760.0246535	-2235.170894	-2226.202476	EMD1
CGGGTCAAGAGTTGCG-1	singlet	2	-304.719204	-284.9732942	-119.430903	EMD1
CGGGTCAAGATGCGAC-1	singlet	1	-454.4000897	-156.2595916	-369.0040624	EMD1
CGGGTCAAGGGCGAGA-1	singlet	2	-4638.194502	-4945.490646	-1284.503803	EMD1
CGGGTCAAGGTGGGTT-1	singlet	2	-4134.97692	-3694.035825	-1180.389042	EMD1
CGGGTCAAGTTGGCGA-1	singlet	2	-1349.759473	-1136.769875	-505.6921432	EMD1
CGGGTCACAACAGATA-1	singlet	0	-176.7048196	-535.2023797	-682.6512733	EMD1
CGGGTCACAAGGAGTC-1	singlet	0	-1128.550702	-3119.70669	-3221.431456	EMD1
CGGGTCACACAGAAGC-1	doublet	0/2	-587.444737	-803.9518309	-742.9505023	EMD1
CGGGTCAGTAAGACCG-1	singlet	2	-1340.203424	-1073.541327	-515.0144614	EMD1
CGGGTCAGTATACCTG-1	singlet	0	-890.1882545	-2782.687898	-2829.492874	EMD1
CGGGTCAGTGAATTGA-1	singlet	1	-4352.380211	-1312.123717	-3552.816	EMD1
CGGGTCAGTGTTCGAT-1	singlet	1	-608.1675213	-270.2661307	-442.8182062	EMD1
CGGGTCAGTTCAACGT-1	singlet	2	-558.686553	-357.5879679	-205.0487776	EMD1
CGGGTCATCAGGAGAC-1	singlet	1	-2029.070112	-699.4510143	-1705.359664	EMD1
CGGGTCATCCGTCAAA-1	singlet	2	-148.6142677	-124.5833018	-53.75665286	EMD1
CGGGTCATCGAGTACT-1	singlet	2	-529.8581743	-436.6931256	-221.5466811	EMD1
CGGGTCATCGGTCACG-1	doublet	0/2	-1535.89326	-2236.711736	-1568.873867	EMD1
CGGGTCATCTTCGTAT-1	singlet	1	-2384.874656	-805.9140578	-1920.334163	EMD1
CGGGTGTAGCTCCATA-1	singlet	2	-84.90867523	-66.36243134	-26.30507557	EMD1
CGGGTGTAGGTCACAG-1	singlet	2	-441.1961784	-415.0135083	-182.0234777	EMD1
CGGGTGTAGTTGGCGA-1	singlet	1	-280.6618061	-87.67902146	-203.542484	EMD1
CGGGTGTCACGGTCTG-1	singlet	0	-2240.735118	-10237.52399	-8851.753657	EMD1
CGGGTGTCATCACGGC-1	singlet	2	-337.6163311	-279.9042331	-144.6038251	EMD1
CGGGTGTGTCGGTAAG-1	singlet	0	-536.2789092	-1751.898431	-1949.524163	EMD1
CGGGTGTGTTCGCGTG-1	singlet	1	-1475.229114	-492.0417059	-1336.157714	EMD1
CGGGTGTTCGCTGTTC-1	singlet	2	-2060.437609	-1752.276412	-712.9519969	EMD1
CGGGTGTTCGCTTTAT-1	singlet	2	-672.2290115	-560.8872028	-262.3505135	EMD1
CGGGTGTTCTAGACCA-1	singlet	2	-283.0385076	-250.0240302	-131.2019606	EMD1
CGGGTGTTCTTACCGC-1	singlet	2	-4716.729878	-3434.642	-1567.427354	EMD1
CGGTCAGAGACCGTTT-1	singlet	2	-284.0958811	-253.3425517	-114.9629989	EMD1
CGGTCAGAGCGAATGC-1	doublet	45323	-2162.830178	-1622.989324	-1042.250654	EMD1
CGGTCAGAGCTTCGTA-1	singlet	1	-317.9832089	-102.7145083	-214.9431771	EMD1
CGGTCAGAGTTCATGC-1	singlet	1	-1855.071499	-630.888169	-1394.544587	EMD1
CGGTCAGGTCTACTGA-1	singlet	1	-1563.577874	-558.2781595	-1261.556483	EMD1
CGGTCAGGTGGAGAAA-1	singlet	2	-846.9903598	-596.5987204	-290.4865561	EMD1
CGGTCAGGTGGTCTTA-1	singlet	1	-1040.849579	-412.0016195	-920.5049067	EMD1
CGGTCAGTCGACGCTG-1	singlet	2	-393.6394919	-405.6291116	-164.1657123	EMD1
CGGTCAGTCGTGGACC-1	singlet	2	-631.2166791	-495.7641309	-219.1902609	EMD1
CGTAAGTAGAATTGTG-1	singlet	1	-2268.0274	-697.7730961	-1594.439101	EMD1
CGTAAGTCACATTCTT-1	singlet	0	-319.4598017	-919.3883398	-943.8637041	EMD1
CGTAAGTGTTGGGCCT-1	singlet	0	-356.3614038	-973.5456571	-997.0007679	EMD1
CGTAAGTTCCCAACTC-1	singlet	2	-1403.636407	-1396.150698	-531.6429981	EMD1
CGTAAGTTCCCGAGAC-1	singlet	2	-345.6300575	-305.9922523	-131.3797936	EMD1
CGTAATGAGAGCATAT-1	singlet	1	-324.1854848	-116.5672741	-301.3855206	EMD1
CGTAATGAGCGATCGA-1	singlet	2	-2099.651908	-1578.325858	-636.5557671	EMD1
CGTAATGAGCGGGTTA-1	singlet	2	-547.69824	-445.5009583	-217.9277631	EMD1
CGTAATGAGTACAACA-1	singlet	0	-203.9316657	-548.1623617	-655.1318441	EMD1
CGTAATGAGTTACGAA-1	singlet	2	-391.5720963	-379.9450324	-168.8533275	EMD1
CGTAATGCAGAGGGTT-1	singlet	0	-169.6666982	-463.3133468	-539.9154576	EMD1
CGTAATGCAGCTAACT-1	singlet	0	-54.72897598	-205.9152642	-212.752655	EMD1
CGTAATGCATACCGTA-1	singlet	1	-334.1087289	-136.5262817	-283.5101771	EMD1
CGTAATGGTTCAATCG-1	singlet	2	-466.0784455	-421.5950987	-198.5566242	EMD1
CGTAATGTCGGTAGGA-1	singlet	1	-138.7712531	-29.73053425	-128.7951174	EMD1
CGTAGTAAGGACATCG-1	singlet	2	-1487.263297	-1440.80619	-477.9876967	EMD1
CGTAGTACATAACGGG-1	singlet	0	-255.0917961	-705.9051124	-686.0636667	EMD1
CGTAGTACATTCAGCA-1	singlet	2	-775.2102535	-629.8677583	-322.7311065	EMD1
CGTAGTAGTACAAGCG-1	singlet	0	-161.4896545	-454.4498065	-411.6705967	EMD1
CGTAGTAGTCGTCATA-1	singlet	1	-1870.923079	-669.3244772	-1486.719875	EMD1
CGTAGTATCACGATAC-1	singlet	2	-6334.317289	-4929.677659	-1844.506139	EMD1
CGTAGTATCCAGCCTT-1	singlet	2	-970.0863043	-908.0320275	-339.2456838	EMD1
CGTAGTATCGTGTCAA-1	singlet	2	-1336.363961	-1193.653755	-568.623936	EMD1
CGTAGTATCTTCGTGC-1	singlet	2	-1932.093889	-1753.56139	-611.0453738	EMD1
CGTCAAAAGAAGCCTG-1	singlet	2	-2310.425849	-1642.460097	-799.1042611	EMD1
CGTCAAAAGCCAAGTG-1	singlet	2	-368.451222	-337.4362782	-152.0214534	EMD1
CGTCAAAAGCGACCCT-1	singlet	0	-291.3722142	-1024.376825	-959.0236996	EMD1
CGTCAAAAGCGCCATC-1	singlet	0	-702.6439732	-2164.709336	-2088.358903	EMD1
CGTCAAAAGCGCTGCT-1	singlet	0	-287.1917789	-684.0663595	-742.9739797	EMD1
CGTCAAACAACGATTC-1	singlet	1	-123.0039184	-38.30687064	-83.03316839	EMD1
CGTCAAACAGACATCT-1	singlet	1	-4207.893074	-1371.428087	-3265.253097	EMD1
CGTCAAATCAGGCGAA-1	singlet	2	-615.6799699	-467.6838456	-203.2978449	EMD1
CGTCAAATCAGTGCGC-1	singlet	2	-417.9667288	-313.3927415	-176.9053988	EMD1
CGTCCATCAGAGCGTA-1	singlet	0	-315.7143356	-911.0972596	-851.6876833	EMD1
CGTCCATCATCTGCGG-1	singlet	0	-46.24944428	-134.1633826	-158.4670309	EMD1
CGTCCATGTAAGGTCG-1	singlet	1	-1841.370357	-647.3603475	-1512.513668	EMD1
CGTCCATGTTCGATTG-1	singlet	2	-500.4304545	-399.357709	-203.9497484	EMD1
CGTCCATGTTCTAACG-1	singlet	0	-51.8685111	-148.5538909	-146.714695	EMD1
CGTCCATGTTTCCAAG-1	singlet	2	-511.9215069	-545.5204671	-235.4890609	EMD1
CGTCCATTCGGAATGG-1	singlet	0	-209.5330394	-859.7049489	-765.8097067	EMD1
CGTCCATTCTTTGATC-1	singlet	0	-144.2172803	-381.4207267	-443.941548	EMD1
CGTGAATAGACTGAGC-1	singlet	0	-112.242162	-342.3452101	-351.6667698	EMD1
CGTGAATAGATAGTCA-1	doublet	0/2	-627.8680127	-1267.551386	-960.3069506	EMD1
CGTGAATAGCACTCCG-1	singlet	2	-1137.439547	-983.7420005	-384.12266	EMD1
CGTGAATAGTGCCGAA-1	singlet	2	-301.0590945	-291.7432873	-148.4762171	EMD1
CGTGAATGTACACGTT-1	singlet	1	-2124.453518	-635.0448425	-1665.189824	EMD1
CGTGAATGTCGTTATG-1	singlet	2	-2202.81121	-1762.882603	-710.1598807	EMD1
CGTGAATGTGATCGTT-1	singlet	2	-1423.815178	-1267.076032	-553.2154532	EMD1
CGTGAATGTTTCGCTC-1	singlet	2	-374.4492902	-325.1875866	-164.8472285	EMD1
CGTGAATTCACCGGTG-1	singlet	2	-6699.069129	-5249.559082	-1994.246012	EMD1
CGTGAATTCATGCATG-1	singlet	0	-314.0806607	-844.6105902	-848.9069892	EMD1
CGTGAATTCCGAGAAG-1	singlet	1	-968.6362312	-357.7169908	-727.3363224	EMD1
CGTGAATTCCGCGGAT-1	singlet	2	-303.4044228	-264.066324	-106.7954025	EMD1
CGTGAATTCTGCTCTG-1	singlet	1	-453.081026	-164.3737513	-394.9946936	EMD1
CGTGATAAGCGCCTTG-1	singlet	2	-310.6899501	-257.8329529	-106.8278077	EMD1
CGTGATAAGGTAAGGA-1	singlet	0	-404.4744695	-1190.30868	-1050.560201	EMD1
CGTGATACAACATACC-1	singlet	2	-887.699782	-695.6768024	-299.2501979	EMD1
CGTGATACACCAGTAT-1	singlet	2	-294.0500536	-288.1078068	-124.4162524	EMD1
CGTGATACATCTAGAC-1	singlet	0	-335.9310069	-915.3754785	-936.7833392	EMD1
CGTGATAGTAGTCCTA-1	singlet	1	-1840.23566	-553.9256897	-1598.447621	EMD1
CGTGATAGTGATTGGG-1	singlet	2	-351.8173338	-274.9256968	-128.5533644	EMD1
CGTGCTTAGAACAAGG-1	unassigned	1/0	-9.312490116	-9.224430739	-153.1945877	EMD1
CGTGCTTAGCTGGCTC-1	singlet	0	-316.0781709	-880.2189993	-866.1302991	EMD1
CGTGCTTAGGGACCAT-1	singlet	0	-237.4264862	-938.5604392	-780.3382944	EMD1

CGTGCTTCAAACAGGC-1	singlet	0	-1081.994778	-3642.220801	-3116.511703	EMD1
CGTGCTTCAAATGCGG-1	singlet	2	-493.2012857	-466.7412407	-182.2539076	EMD1
CGTGCTTCAACACTAC-1	singlet	2	-306.3109559	-308.0770655	-131.8296728	EMD1
CGTGCTTCAAGACCGA-1	singlet	1	-2942.183598	-808.6602913	-2270.202088	EMD1
CGTGCTTCATGTGTCA-1	singlet	2	-1915.71569	-1494.43499	-602.5827092	EMD1
CGTGCTTCATTGTGCA-1	singlet	2	-342.3385115	-297.0714304	-127.9405731	EMD1
CGTGCTTGTCGCAACC-1	singlet	0	-517.2641475	-1691.716929	-1645.134788	EMD1
CGTGCTTGTTCTGAGT-1	singlet	0	-392.1421126	-970.8706565	-960.2867555	EMD1
CGTGCTTTCGCAGTGC-1	singlet	2	-420.792939	-295.7899647	-144.9223065	EMD1
CGTGTCTAGAGGACTC-1	singlet	2	-295.1302221	-196.5871259	-83.07098493	EMD1
CGTGTCTAGGACTTCT-1	singlet	2	-464.4643988	-376.263068	-166.8923659	EMD1
CGTGTCTCACACAGCC-1	singlet	2	-504.0138764	-351.9293649	-139.9021234	EMD1
CGTGTCTCATTCTGTT-1	singlet	2	-434.0121252	-347.2880961	-165.4316427	EMD1
CGTGTCTGTGGTTTAC-1	doublet	45293	-2800.03093	-1363.749314	-1916.466479	EMD1
CGTGTCTTCACCTCTG-1	singlet	2	-1072.776449	-1118.241254	-356.5903834	EMD1
CGTGTCTTCTATTGTC-1	singlet	1	-2104.909951	-667.6893204	-1671.619569	EMD1
CGTTAGACAAGCCATT-1	singlet	0	-28.31792788	-94.79961644	-72.83022243	EMD1
CGTTAGACACGGTAGA-1	singlet	2	-2847.453378	-3027.178996	-750.7632803	EMD1
CGTTAGACAGGCGTTC-1	singlet	2	-3968.34381	-3427.365725	-1117.282371	EMD1
CGTTAGAGTGATAGAT-1	singlet	1	-1730.749866	-612.8541175	-1440.949832	EMD1
CGTTAGAGTGTTTCTT-1	singlet	0	-337.713411	-1208.910323	-1088.333337	EMD1
CGTTAGATCGTTGCCT-1	singlet	2	-295.5393041	-286.6622576	-120.3393628	EMD1
CGTTAGATCTAAGCCA-1	singlet	0	-441.8831145	-1267.646341	-1200.801602	EMD1
CGTTAGATCTTTCGAT-1	singlet	2	-3657.395382	-2946.074001	-1375.816863	EMD1
CGTTCTGAGCGGATCA-1	singlet	1	-1581.101536	-506.5315005	-1308.198248	EMD1
CGTTCTGAGGTAGATT-1	singlet	2	-638.8754076	-607.4799405	-187.674945	EMD1
CGTTCTGCAACGTTAC-1	singlet	2	-764.6694043	-644.4524178	-298.2311644	EMD1
CGTTCTGCAATACCCA-1	singlet	2	-8134.480388	-6866.705471	-2212.257159	EMD1
CGTTCTGGTTGTATGC-1	singlet	1	-4592.686129	-1047.501704	-3826.851425	EMD1
CGTTGGGAGGTGGCTA-1	singlet	0	-39.16369853	-104.1510109	-116.0765641	EMD1
CGTTGGGCAATGTCTG-1	singlet	2	-4710.228733	-3705.65551	-1421.048618	EMD1
CGTTGGGGTACTAAGA-1	singlet	2	-435.7189431	-462.2828556	-166.7533203	EMD1
CGTTGGGTCAGACATC-1	singlet	1	-304.4827439	-127.112908	-276.1342333	EMD1
CTAACCCAGACTACGG-1	singlet	2	-1726.040988	-1316.440834	-623.0419515	EMD1
CTAACCCGTAATTGGA-1	singlet	2	-3338.428719	-2824.13923	-1143.024333	EMD1
CTAACCCGTATCACGT-1	doublet	2/0	-1910.467198	-2783.218593	-1661.375961	EMD1
CTAACCCGTCGAGTTT-1	singlet	2	-11370.75112	-8712.781226	-2948.616904	EMD1
CTAACCCGTCGCTTGG-1	singlet	2	-403.1548356	-357.7470744	-174.9328348	EMD1
CTAACCCGTCGGTGAA-1	singlet	0	-205.4157042	-439.656406	-485.2696477	EMD1
CTAACCCGTCTCGCGA-1	singlet	2	-1695.147342	-1495.281457	-652.2897579	EMD1
CTAACCCGTGTCACAT-1	singlet	2	-436.0623802	-391.4291738	-193.0491495	EMD1
CTAACCCTCACTGTCC-1	singlet	2	-702.8815868	-519.8658193	-243.1081084	EMD1
CTAACCCTCAGGAGAC-1	singlet	2	-555.4345026	-456.5797502	-209.9901527	EMD1
CTAACTTAGCGTGCCT-1	singlet	0	-394.1639158	-1217.708913	-1152.729557	EMD1
CTAACTTAGCTGTTAC-1	doublet	0/2	-235.8072922	-397.5316199	-382.2568367	EMD1
CTAACTTCACTGCATA-1	singlet	2	-2464.324864	-2054.887052	-830.2367252	EMD1
CTAACTTGTAGCTTAC-1	singlet	1	-2535.186509	-1434.434081	-2613.278238	EMD1
CTAACTTGTATCGTTG-1	singlet	0	-4.091892673	-13.7048679	-44.04134409	EMD1
CTAACTTGTGTCTAAC-1	singlet	0	-140.1889902	-304.9011814	-321.5571583	EMD1
CTAACTTGTTCGTACA-1	singlet	1	-2154.770977	-788.6306836	-1966.583644	EMD1
CTAACTTTCATTACGG-1	singlet	1	-190.0451739	-60.26072222	-145.0119548	EMD1
CTAACTTTCTACGCGG-1	singlet	2	-601.5018043	-456.7772174	-237.5869609	EMD1
CTAAGTGAGACGTCCC-1	singlet	0	-980.1936268	-2840.429917	-2832.880034	EMD1
CTAAGTGAGATGCCGA-1	singlet	0	-1230.187098	-3458.198771	-3460.74908	EMD1
CTAAGTGAGTGTACAA-1	singlet	2	-492.0165577	-415.6332256	-201.3460692	EMD1
CTAAGTGAGTGTTCAC-1	singlet	0	-1057.746636	-3808.183891	-3869.778107	EMD1
CTAAGTGCAACGATCT-1	singlet	2	-1018.023544	-847.1300273	-404.7810128	EMD1
CTAAGTGCAGGAATAT-1	singlet	2	-418.4599828	-336.6297796	-153.7030578	EMD1
CTAAGTGGTAACGATA-1	singlet	2	-411.8043338	-405.0584777	-205.5662719	EMD1
CTAAGTGGTCCGTACG-1	singlet	0	-997.8604337	-3292.228582	-3280.28174	EMD1
CTAAGTGGTCGGCTAC-1	singlet	2	-346.7250454	-290.6542568	-154.8091094	EMD1
CTAAGTGGTGACGTCC-1	singlet	2	-264.0974422	-210.0900778	-86.01499972	EMD1
CTAAGTGGTTACCGTA-1	singlet	1	-541.0027478	-164.1435481	-453.2346565	EMD1
CTAAGTGGTTATTCTC-1	singlet	2	-407.4199653	-368.95144	-191.0486404	EMD1
CTAAGTGTCTTTGCAT-1	singlet	0	-924.044463	-2549.449753	-2602.788549	EMD1
CTACAGACACGTATAC-1	singlet	0	-69.67025223	-144.8958339	-141.4214133	EMD1
CTACAGACAGAGGTTG-1	singlet	2	-4382.372826	-3546.625467	-1517.423101	EMD1
CTACAGACAGCGACCT-1	singlet	0	-1841.543842	-6151.066028	-6191.85477	EMD1
CTACAGACAGGACATG-1	singlet	2	-599.0601165	-460.4051321	-216.6915908	EMD1
CTACAGACATTCCTAT-1	singlet	2	-1737.683399	-1455.331241	-672.2761791	EMD1
CTACAGACATTGTAGC-1	singlet	0	-456.5796263	-1304.420722	-1292.481396	EMD1
CTACAGAGTGACCGTC-1	singlet	2	-2337.868667	-1885.228392	-694.7931513	EMD1
CTACAGAGTGTGGACA-1	singlet	2	-515.1314514	-423.8591896	-174.5125866	EMD1
CTACAGATCCGATGTA-1	singlet	1	-6022.431847	-2117.103864	-5203.251835	EMD1
CTACAGATCGAGCACC-1	singlet	0	-206.7201605	-638.1255727	-687.3701421	EMD1
CTACAGATCGGTAACT-1	singlet	2	-392.7045513	-315.7649128	-152.3441829	EMD1
CTACATTAGGAGAATG-1	singlet	0	-422.5498771	-1101.516305	-1006.001879	EMD1
CTACATTAGTACCATC-1	singlet	0	-69.11664746	-156.8356617	-163.7376844	EMD1
CTACATTCACCCAAGC-1	singlet	2	-494.5308398	-372.9501945	-182.1821803	EMD1
CTACATTCACCCTTAC-1	singlet	2	-674.0663807	-597.5352828	-245.0732121	EMD1
CTACATTCATTGCCGG-1	singlet	2	-493.2215886	-410.3612383	-211.5899251	EMD1
CTACATTGTGGAACAC-1	singlet	0	-1262.639528	-3776.283801	-3790.512034	EMD1
CTACATTGTGGTCCGT-1	singlet	2	-8399.982648	-6510.035976	-2500.859924	EMD1
CTACATTGTTTCGATG-1	singlet	2	-427.1747019	-377.0247172	-172.1864488	EMD1
CTACATTTCACTCCGT-1	singlet	2	-531.4806318	-425.1568482	-181.5980813	EMD1
CTACATTTCCACCCTA-1	singlet	1	-771.1133769	-347.4804633	-891.1737881	EMD1
CTACCCAAGTGAATAC-1	singlet	0	-214.0458633	-702.2889512	-624.5836162	EMD1
CTACCCACAGCTAACT-1	singlet	0	-1616.977495	-4748.73629	-4879.442527	EMD1
CTACCCAGTAAGCGGT-1	singlet	2	-649.9723262	-544.0205104	-258.1693965	EMD1
CTACCCAGTTGCATGT-1	singlet	0	-219.5704032	-512.8401865	-475.6719567	EMD1
CTACCCATCACCCTTG-1	singlet	2	-1854.182363	-1685.553348	-681.9527772	EMD1
CTACCCATCGGCATTA-1	singlet	0	-695.2630186	-2293.990167	-2267.823027	EMD1
CTACCCATCGGTAACT-1	singlet	2	-638.0247964	-566.958043	-226.2441733	EMD1
CTACCCATCTGGTTGA-1	singlet	0	-1205.668034	-3527.590257	-3650.590244	EMD1
CTACCTGAGCACGTCC-1	singlet	1	-1746.422355	-473.2274442	-1289.884071	EMD1
CTACCTGAGCGCCTCA-1	singlet	1	-2383.043768	-693.8004931	-1890.233743	EMD1
CTACCTGAGGATTTCC-1	singlet	2	-2727.024195	-2199.243225	-972.9952905	EMD1
CTACCTGAGGCGTCCT-1	singlet	2	-1028.827803	-892.689106	-400.3893051	EMD1
CTACCTGAGTTGAATG-1	singlet	0	-677.2188983	-1927.03939	-2010.528222	EMD1
CTACCTGCAGTATGAA-1	singlet	1	-701.3835376	-267.5524308	-648.0673149	EMD1
CTACCTGCATCGATAC-1	singlet	1	-3307.713976	-1208.292455	-2608.22383	EMD1
CTACCTGGTGAGACCA-1	singlet	2	-641.7633076	-605.3235493	-257.389213	EMD1
CTACCTGGTTTCGTAG-1	singlet	1	-5107.922509	-1626.565254	-3992.710127	EMD1
CTACCTGTCAACCCGG-1	singlet	0	-482.9012119	-1624.009221	-1527.803738	EMD1
CTACGGGAGTTACGGG-1	singlet	0	-729.0816999	-2280.91006	-2292.823205	EMD1
CTACGGGCACCTGTCT-1	singlet	2	-5793.217309	-4943.575387	-1928.279606	EMD1
CTACGGGCATCGAAGG-1	singlet	0	-264.8718489	-767.5866905	-682.7323537	EMD1
CTACGGGGTACGGGAT-1	singlet	2	-4698.514312	-3601.071569	-1569.357439	EMD1
CTACGGGGTATCGCAT-1	singlet	0	-211.1445545	-673.2449152	-680.5867041	EMD1
CTACGGGGTCTTTCTA-1	singlet	0	-727.4533957	-2279.351318	-2267.640805	EMD1

CTACGGGTCAGGGATG-1	singlet	2	-442.6520977	-410.8433004	-171.3305186	EMD1
CTACGGGTCCGTACGG-1	singlet	2	-502.7270787	-437.1130564	-194.7846932	EMD1
CTACTATAGACTAAGT-1	singlet	1	-102.7689512	-53.85118885	-92.68128971	EMD1
CTACTATAGCAACAGC-1	singlet	2	-2554.352698	-2237.900641	-950.5942103	EMD1
CTACTATAGGCTAGCA-1	singlet	2	-308.4819754	-273.4237569	-141.9865875	EMD1
CTACTATAGTAATCCC-1	singlet	2	-487.1085574	-485.5683269	-229.3938988	EMD1
CTACTATAGTCAGCCC-1	singlet	0	-892.9801847	-3335.587614	-2731.349917	EMD1
CTACTATCAAAGCTCT-1	singlet	2	-1399.974796	-1090.710726	-499.1359611	EMD1
CTACTATCATCTTAGG-1	singlet	2	-707.0897937	-565.2258787	-259.3582493	EMD1
CTACTATCATGCCATA-1	singlet	2	-415.0651294	-369.7943008	-188.4717726	EMD1
CTACTATGTATTGGCT-1	singlet	1	-2935.210536	-1245.443074	-2163.697032	EMD1
CTACTATGTCTGCAAT-1	singlet	2	-257.6451644	-265.8694985	-126.1453477	EMD1
CTACTATGTTCCCACT-1	singlet	2	-663.7723548	-537.1891445	-278.2203784	EMD1
CTACTATTCACTACTT-1	singlet	0	-429.2642994	-1864.679563	-1878.179055	EMD1
CTACTATTCGTGGGAA-1	doublet	45293	-688.7828311	-304.0922218	-436.5380456	EMD1
CTAGACAAGATACCAA-1	singlet	1	-1351.25672	-572.4778655	-1087.619958	EMD1
CTAGACAAGTCGCGAA-1	singlet	2	-269.9563501	-236.742707	-123.0713559	EMD1
CTAGACACACACACGC-1	singlet	0	-2465.892054	-8880.24438	-7471.694231	EMD1
CTAGACACAGTGACCC-1	singlet	0	-762.6186613	-2017.321287	-1865.403767	EMD1
CTAGACAGTAACAGGC-1	singlet	2	-440.5022251	-352.6539586	-194.3652397	EMD1
CTAGACATCGCAGAGA-1	singlet	0	-149.245477	-504.8922704	-519.0922646	EMD1
CTAGACATCGTAGTGT-1	singlet	2	-1389.02438	-1249.238457	-503.1491787	EMD1
CTAGACATCTCCAATT-1	singlet	2	-404.710801	-349.1647457	-180.9571653	EMD1
CTAGGTAAGACCTTTG-1	singlet	2	-394.8746745	-379.7883944	-185.070358	EMD1
CTAGGTAAGCGTGAGT-1	singlet	1	-1650.648814	-467.9279104	-1324.021234	EMD1
CTAGGTACAAAGGCAC-1	singlet	2	-2147.817495	-1672.523456	-717.3701835	EMD1
CTAGGTACAAGAGTTA-1	singlet	0	-948.1451229	-2669.441976	-2519.677627	EMD1
CTAGGTAGTATGTCTG-1	singlet	2	-755.284556	-583.2893481	-260.4246703	EMD1
CTAGGTATCAAGCCAT-1	singlet	0	-1906.22199	-5480.832686	-5387.364848	EMD1
CTAGGTATCAGCTGTA-1	singlet	1	-1906.814141	-715.224244	-1759.354848	EMD1
CTAGGTATCATTTCGT-1	singlet	2	-265.638535	-199.7034622	-86.57233124	EMD1
CTAGGTATCCAACCGG-1	singlet	2	-449.2476412	-380.7720908	-180.8536124	EMD1
CTAGGTATCCATATGG-1	singlet	1	-1264.901293	-496.3670019	-1028.1166	EMD1
CTATAGGAGAAGTCTA-1	singlet	2	-343.5938594	-254.9711514	-128.9569276	EMD1
CTATAGGAGAGAGTGA-1	singlet	0	-1296.699388	-3558.951368	-3554.229926	EMD1
CTATAGGAGCCATTGT-1	singlet	2	-345.6050761	-354.5591653	-179.1181281	EMD1
CTATAGGCAAGACCTT-1	singlet	2	-244.0640402	-279.8237326	-120.1458009	EMD1
CTATAGGCAGACTGCC-1	singlet	0	-307.0997924	-1002.709202	-1021.79449	EMD1
CTATAGGCATAGATCC-1	singlet	1	-1251.824242	-404.3518287	-1068.33123	EMD1
CTATAGGTCACCCTCA-1	singlet	2	-1743.930539	-1486.418761	-575.2591277	EMD1
CTATAGGTCCAACCAA-1	singlet	1	-3553.769288	-1349.409966	-2691.781781	EMD1
CTATAGGTCCGTGACG-1	singlet	2	-488.6387241	-426.9042478	-185.2954046	EMD1
CTATAGGTCGCTCTAC-1	singlet	2	-518.9203915	-443.0920612	-233.0810372	EMD1
CTATCCGAGTTTAGGA-1	singlet	1	-3517.063739	-1019.766557	-2405.911776	EMD1
CTATCCGCAAAGGCAC-1	singlet	2	-1113.168852	-888.8898595	-446.1033698	EMD1
CTATCCGCAACGATTC-1	singlet	0	-549.3852173	-1360.798079	-1255.598161	EMD1
CTATCCGCAATTGAAG-1	doublet	45323	-82.09361347	-68.00122283	-42.97523233	EMD1
CTATCCGCACCGTGCA-1	singlet	0	-624.6813562	-1908.806545	-1905.716838	EMD1
CTATCCGCACGGGTAA-1	singlet	2	-690.160987	-549.0042882	-253.6837105	EMD1
CTATCCGCAGATCCAT-1	singlet	1	-5436.223035	-1583.954986	-4354.409156	EMD1
CTATCCGCATTGACAC-1	singlet	2	-357.6379665	-301.6627194	-144.1598303	EMD1
CTATCCGGTCCGACGT-1	singlet	2	-731.5923931	-618.1162658	-308.7901731	EMD1
CTATCCGGTGTCACAT-1	singlet	2	-295.1656464	-272.0459403	-119.655956	EMD1
CTATCCGGTGTCATTG-1	singlet	2	-417.6718022	-359.6844832	-177.67365	EMD1
CTATCCGGTTTGGCTA-1	singlet	2	-485.0883556	-434.2278799	-222.7076844	EMD1
CTATCCGTCATTCATC-1	singlet	0	-2131.500863	-5649.454546	-5726.266448	EMD1
CTATCCGTCATTGTGG-1	singlet	1	-2132.860258	-394.8177468	-1407.280225	EMD1
CTATCCGTCTGGGATT-1	singlet	2	-287.4700441	-218.8235174	-101.1048966	EMD1
CTATCTAAGCCTTTGA-1	singlet	0	-363.5272547	-1285.582781	-1154.013247	EMD1
CTATCTACACACCTTC-1	singlet	0	-379.656397	-1631.901337	-1517.052877	EMD1
CTATCTAGTTCGTAAC-1	singlet	1	-1258.681839	-629.8114086	-1174.723842	EMD1
CTATCTATCGTTCGCT-1	singlet	2	-398.2557	-341.7349005	-145.303182	EMD1
CTCAACCAGTCACTGT-1	singlet	0	-678.8270432	-2329.336539	-2154.170124	EMD1
CTCAACCAGTGCACTT-1	singlet	1	-3057.681153	-1235.443505	-2720.460735	EMD1
CTCAACCCAGAACCGA-1	singlet	2	-334.9546812	-262.6983456	-146.231266	EMD1
CTCAACCCAGCCCACA-1	singlet	2	-1078.838487	-823.7006875	-357.9253035	EMD1
CTCAACCGTACCATAC-1	singlet	1	-82.223858	-39.30388115	-73.38587968	EMD1
CTCAACCTCATCCCGT-1	singlet	2	-704.4291753	-653.1915725	-266.993857	EMD1
CTCAACCTCATCGACA-1	singlet	2	-271.0301674	-182.5062324	-97.81910774	EMD1
CTCAACCTCGCAGTCG-1	singlet	2	-717.1526275	-655.9130474	-294.6380916	EMD1
CTCAACCTCGCCGAGT-1	singlet	1	-1571.944464	-483.3062702	-1247.976787	EMD1
CTCAACCTCTACTGCC-1	singlet	2	-1821.921216	-1574.14954	-663.6229345	EMD1
CTCAAGAAGAAATGGG-1	singlet	2	-492.2392979	-426.760792	-217.9519168	EMD1
CTCAAGAAGTGTGTTC-1	singlet	1	-2384.409979	-805.5007149	-1859.373911	EMD1
CTCAAGACAGGTTACT-1	singlet	1	-1616.968531	-600.4536419	-1266.700207	EMD1
CTCAAGAGTCCAAGAG-1	singlet	0	-557.767083	-3295.844671	-2791.053685	EMD1
CTCAAGAGTCCTCCTA-1	singlet	1	-1575.808822	-365.6311459	-1127.398629	EMD1
CTCAAGAGTCTCCTGT-1	singlet	2	-499.4947676	-398.688861	-196.0601429	EMD1
CTCAAGAGTGCGTCGT-1	singlet	2	-308.2836703	-244.4788188	-142.4547248	EMD1
CTCAAGAGTTTCTTAC-1	singlet	1	-947.4378592	-391.0816036	-635.8016165	EMD1
CTCAAGATCTGCTAGA-1	singlet	2	-377.9809205	-241.5396675	-97.40887139	EMD1
CTCAATTAGATACATG-1	doublet	1/0	-4150.489786	-3729.01073	-4902.953707	EMD1
CTCAATTCAAAGGCGT-1	singlet	2	-237.2994877	-193.3383909	-83.69397764	EMD1
CTCAATTCAAGAGGTC-1	singlet	2	-414.2482526	-381.6104243	-183.7709191	EMD1
CTCAATTCACAGTGTT-1	singlet	0	-996.5673645	-3013.151055	-2915.540352	EMD1
CTCAATTCACCGTCTT-1	singlet	0	-872.1614635	-2441.401203	-2247.074922	EMD1
CTCAATTCACTACCGG-1	singlet	2	-1633.379925	-1386.782519	-582.4914155	EMD1
CTCAATTCATAACAGA-1	singlet	1	-1290.461826	-508.231585	-979.5822222	EMD1
CTCAATTCATGACTTG-1	singlet	2	-2131.778813	-1903.099494	-760.6462982	EMD1
CTCAATTGTGGCAACA-1	singlet	1	-86.65472244	-30.65960291	-67.74622259	EMD1
CTCAATTGTGTTGATC-1	singlet	0	-165.0666238	-750.5579971	-724.6331476	EMD1
CTCACTGAGACGGAAA-1	singlet	2	-524.781536	-407.5984835	-192.7399917	EMD1
CTCACTGAGCTGCCAC-1	singlet	2	-666.5884325	-549.2451419	-210.1481912	EMD1
CTCACTGAGCTTCGTA-1	singlet	1	-218.113361	-101.7271928	-230.3070268	EMD1
CTCACTGAGCTTGTGT-1	singlet	0	-247.6725557	-883.4615393	-745.0988948	EMD1
CTCACTGAGGATCACG-1	unassigned	1	-2.491825922	-1.714967942	-4.912694831	EMD1
CTCACTGAGTGCGTCC-1	singlet	2	-295.1267674	-236.1941931	-133.5499864	EMD1
CTCACTGCATCATCTT-1	singlet	0	-873.513306	-2735.059026	-2764.634967	EMD1
CTCACTGGTAATGCGG-1	doublet	1/0	-2429.404229	-1911.487256	-2803.415941	EMD1
CTCACTGGTGGCAGAT-1	singlet	1	-2280.202791	-731.9101655	-1846.382515	EMD1
CTCACTGGTTTGAAAG-1	singlet	1	-106.7032977	-41.3090567	-89.75162642	EMD1
CTCACTGGTTTGCAGT-1	singlet	2	-370.8592584	-291.5497901	-144.8765902	EMD1
CTCACTGTCACTTGGA-1	singlet	2	-544.6081546	-480.0253778	-216.7353107	EMD1
CTCACTGTCGATGCTA-1	singlet	2	-1084.518228	-988.9016348	-415.0655828	EMD1
CTCACTGTCGGCACTG-1	singlet	1	-525.0427796	-206.5650222	-432.2912117	EMD1
CTCACTGTCTAGCCTC-1	unassigned	0/1	-12.39834652	-17.57010012	-121.0283929	EMD1
CTCAGAAAGTGGCCTC-1	singlet	1	-1847.028167	-688.7405962	-1509.994557	EMD1
CTCAGAACACACACGC-1	singlet	0	-213.9193567	-523.6811485	-526.0366834	EMD1
CTCAGAAGTCTCAGAT-1	singlet	1	-321.7547418	-80.93405185	-204.2694015	EMD1
CTCAGAAGTGAGGATC-1	singlet	1	-511.7490075	-207.6290793	-475.2362612	EMD1

CTCAGAAGTTTGAAAG-1	singlet	1	-3512.386606	-1082.173619	-2732.244345	EMD1
CTCAGAATCACAAGAA-1	singlet	1	-5377.88371	-1580.026916	-4019.834593	EMD1
CTCAGAATCACTGAAC-1	singlet	1	-394.9580051	-167.2154596	-369.4793395	EMD1
CTCAGAATCCCAGCGA-1	singlet	1	-4851.702234	-1461.140385	-3881.676753	EMD1
CTCAGAATCTACCAGA-1	singlet	2	-4735.856102	-5152.972673	-2352.082588	EMD1
CTCAGAATCTAGCATG-1	singlet	2	-2805.231064	-2179.75322	-1001.817027	EMD1
CTCAGAATCTCAGAAC-1	singlet	1	-78.53173046	-33.20916264	-52.03085498	EMD1
CTCAGGGAGCGTATGG-1	singlet	2	-513.2073926	-399.392297	-206.90869	EMD1
CTCAGGGAGGCTGTAG-1	unassigned	1	-13.52260924	-4.13657088	-6.415289752	EMD1
CTCAGGGCAGCGTACC-1	singlet	1	-1414.010086	-509.6747635	-1248.853125	EMD1
CTCAGGGGTCGAATGG-1	singlet	0	-46.93699193	-111.4397696	-132.4202737	EMD1
CTCAGGGTCGCGATCG-1	singlet	0	-299.4031094	-855.0682291	-889.337087	EMD1
CTCAGGGTCTCTAAGG-1	singlet	0	-156.9194629	-475.0376271	-432.677737	EMD1
CTCAGTCAGATACAGT-1	singlet	0	-68.45925005	-133.7746238	-170.9998336	EMD1
CTCAGTCAGGCATTTC-1	singlet	1	-1147.34053	-355.6285233	-954.2069394	EMD1
CTCAGTCCAATAACGA-1	singlet	2	-590.839827	-511.7281782	-237.7155497	EMD1
CTCAGTCCACGACCTG-1	singlet	2	-614.8803486	-530.8738954	-243.6269638	EMD1
CTCAGTCCATTGCAAC-1	singlet	0	-329.2092484	-1021.219864	-1108.555916	EMD1
CTCAGTCGTCTCGGAC-1	singlet	2	-673.9157219	-601.2506264	-257.6534385	EMD1
CTCATCGAGCCTATTG-1	singlet	2	-140.1845761	-105.1744845	-39.21094833	EMD1
CTCATCGCAGGCTTGC-1	singlet	1	-2188.002098	-797.2212109	-1746.477606	EMD1
CTCATCGTCGAAGCAG-1	unassigned	45323	-4.721268555	-3.980368481	-3.692358192	EMD1
CTCATCGTCGAGCACC-1	singlet	2	-1727.136647	-1372.970904	-543.7351877	EMD1
CTCATGCCACGTAACT-1	singlet	0	-786.3837729	-2742.599249	-2807.056338	EMD1
CTCATGCCATCTGCGG-1	singlet	2	-542.6936401	-514.1609965	-222.6780506	EMD1
CTCATGCGTCACCGAC-1	singlet	0	-346.4778366	-896.0839757	-918.2850675	EMD1
CTCATGCGTGACAGGT-1	singlet	1	-1512.28058	-563.6266228	-1192.574729	EMD1
CTCATGCGTGGGAGAG-1	singlet	2	-1447.964694	-1268.753528	-523.451666	EMD1
CTCATGCGTTTGGGAG-1	singlet	2	-244.5040186	-251.1313008	-124.9418991	EMD1
CTCATGCTCTACGGTA-1	singlet	0	-492.0534899	-1541.895053	-1584.600339	EMD1
CTCATTAAGCGCCGTT-1	singlet	2	-294.5342962	-266.8789661	-121.9292318	EMD1
CTCATTACACTCAAGT-1	singlet	0	-58.33765341	-188.9411666	-189.1203275	EMD1
CTCATTACATAGATGA-1	singlet	0	-732.5022649	-1896.59742	-2045.425709	EMD1
CTCATTAGTTGTAAAG-1	singlet	0	-602.764554	-1642.814648	-1565.811709	EMD1
CTCATTATCCTCTAGC-1	singlet	0	-557.9876238	-1764.259365	-1720.16565	EMD1
CTCATTATCGAGAATA-1	singlet	1	-1623.810821	-481.4259917	-1189.099419	EMD1
CTCATTATCTTACACT-1	singlet	1	-995.0557505	-411.0672206	-941.8756946	EMD1
CTCCAACAGGATTACA-1	singlet	2	-350.9419232	-339.6024713	-177.2421295	EMD1
CTCCAACAGTTGTAGA-1	singlet	0	-304.9690487	-807.6933539	-894.7987653	EMD1
CTCCAACGTAACATAG-1	singlet	2	-3386.309237	-2840.009396	-1133.174564	EMD1
CTCCAACGTTCTCGTC-1	singlet	2	-747.2380725	-634.0305541	-297.4425887	EMD1
CTCCAACTCAGCGGAA-1	singlet	0	-245.9747849	-633.2072389	-594.5642952	EMD1
CTCCAACTCTGCCTCA-1	singlet	1	-450.4537876	-156.7931864	-420.9580276	EMD1
CTCCACAAGACTAAGT-1	singlet	1	-3703.758455	-1347.454671	-2950.516356	EMD1
CTCCACAAGCGTGTCC-1	singlet	2	-2994.813364	-2226.234486	-943.7538339	EMD1
CTCCACAAGGCGCTTC-1	singlet	1	-1443.50466	-418.5880189	-1404.574569	EMD1
CTCCACAAGGTGCGAT-1	singlet	2	-1457.245669	-1385.589398	-535.7946595	EMD1
CTCCACAAGTGGAAAG-1	singlet	1	-1296.475694	-482.4681595	-1023.731285	EMD1
CTCCACACAGAGGCTA-1	singlet	0	-573.8197304	-1444.597577	-1425.176378	EMD1
CTCCACAGTAAGAACT-1	singlet	0	-280.6999447	-861.241177	-833.5459043	EMD1
CTCCACAGTACTAAGA-1	singlet	1	-1008.0642	-334.4305254	-778.1634263	EMD1
CTCCACAGTCGGCACT-1	singlet	2	-3287.622515	-2566.344568	-1127.909674	EMD1
CTCCACAGTCTCTCCA-1	singlet	1	-571.9997989	-206.5002175	-531.3105022	EMD1
CTCCACAGTGAGGATC-1	singlet	2	-604.250799	-572.5935303	-239.7839951	EMD1
CTCCACAGTTTGATCG-1	singlet	0	-110.4058368	-276.0480388	-264.0975474	EMD1
CTCCACATCACAATGC-1	singlet	2	-2273.558169	-1690.314819	-683.7061534	EMD1
CTCCATGAGCCGTTGC-1	singlet	2	-462.5244994	-412.2292433	-197.7643264	EMD1
CTCCATGAGGCTAGCA-1	singlet	1	-2472.028609	-820.9275675	-2025.879457	EMD1
CTCCATGAGGTGAGAA-1	singlet	2	-446.608758	-388.6217085	-190.9262319	EMD1
CTCCATGAGTCTAGCT-1	singlet	0	-201.3718478	-607.648578	-660.3172554	EMD1
CTCCATGCAAGCCCAC-1	singlet	0	-222.8723847	-616.1349099	-591.2209297	EMD1
CTCCATGCAGCGAACA-1	singlet	2	-3941.957965	-3407.803748	-1257.236731	EMD1
CTCCATGGTCTCCCTA-1	singlet	2	-1171.672302	-1071.573853	-461.2494535	EMD1
CTCCATGGTGCAATGG-1	singlet	2	-586.0862001	-544.3748204	-230.4464617	EMD1
CTCCATGGTGCCCACA-1	singlet	2	-396.497824	-394.2593383	-129.6984317	EMD1
CTCCATGGTTATGGTC-1	singlet	0	-196.5701428	-510.8719344	-657.0143357	EMD1
CTCCATGGTTCAAGTC-1	unassigned	45293	-11.92544582	-4.616592492	-4.749584296	EMD1
CTCCATGTCGCCGATG-1	singlet	2	-823.6796243	-600.6787361	-275.769551	EMD1
CTCCCAAAGACGAGCT-1	singlet	1	-4484.729182	-1528.765607	-3908.370049	EMD1
CTCCCAAAGCATAGGC-1	singlet	2	-2064.467947	-1740.609501	-794.2622275	EMD1
CTCCCAACAACCGATT-1	singlet	1	-191.8498149	-75.81946482	-141.6988671	EMD1
CTCCCAACACCAGTTA-1	singlet	2	-408.3515952	-396.3733832	-232.1346551	EMD1
CTCCCAACAGAATGTA-1	singlet	2	-3895.89522	-3522.838449	-1187.013505	EMD1
CTCCCAAGTAATACCC-1	singlet	0	-172.6684673	-473.2372349	-427.3033248	EMD1
CTCCCAAGTCGCAACC-1	singlet	1	-2084.46525	-753.4600025	-1694.81211	EMD1
CTCCCAAGTCTTCAAG-1	singlet	2	-262.1874027	-244.0747146	-122.5923708	EMD1
CTCCCTCAGACCTCAT-1	singlet	2	-284.2280698	-224.0125709	-98.77016543	EMD1
CTCCCTCAGTCCTACA-1	singlet	2	-485.0400865	-429.2492093	-203.4802985	EMD1
CTCCCTCAGTTCAACC-1	singlet	2	-374.3988709	-312.1436162	-138.5525106	EMD1
CTCCCTCCATTCTCTA-1	singlet	2	-1484.626059	-1189.679313	-569.0813222	EMD1
CTCCCTCGTCACTTAG-1	singlet	0	-462.2877708	-1325.343992	-1137.594509	EMD1
CTCCCTCGTCCTACAA-1	singlet	0	-147.4791738	-461.9745428	-442.544098	EMD1
CTCCCTCTCAAGAGGC-1	singlet	2	-766.2167737	-595.9960827	-278.0473998	EMD1
CTCCCTCTCCGCTTAC-1	singlet	2	-398.6275931	-341.3477866	-157.2826485	EMD1
CTCCCTCTCTATTGTC-1	singlet	2	-1390.877844	-1176.991736	-589.2561382	EMD1
CTCCGATCAATCCTAG-1	singlet	2	-1288.329715	-1089.412996	-458.8827216	EMD1
CTCCGATCACAATGAA-1	doublet	0/2	-454.3173963	-617.0942753	-482.0148475	EMD1
CTCCGATCAGACGATG-1	singlet	2	-370.5373734	-311.9286199	-154.7065251	EMD1
CTCCGATGTACGCGTC-1	singlet	1	-142.3492313	-41.2962552	-154.2781448	EMD1
CTCCGATGTAGGAAAG-1	singlet	2	-1391.951193	-1128.322393	-581.5124221	EMD1
CTCCGATGTATCACCA-1	singlet	0	-1161.375342	-3556.107708	-3491.969863	EMD1
CTCCGATGTATGGGAC-1	singlet	2	-459.7094221	-395.9662811	-160.4167988	EMD1
CTCCGATGTCCAGCCA-1	singlet	2	-528.5965992	-439.3738891	-222.5926987	EMD1
CTCCGATGTCCAGGTC-1	singlet	2	-511.4555771	-379.4082992	-234.1838044	EMD1
CTCCGATGTGAATTAG-1	singlet	0	-692.6432384	-2111.420953	-2211.013628	EMD1
CTCCGATGTTAGGCCC-1	singlet	2	-785.1705179	-657.4414669	-326.4481207	EMD1
CTCCGATTCGGTGTTA-1	singlet	1	-98.1199331	-30.89361672	-107.3125952	EMD1
CTCCTCCAGACCTCCG-1	singlet	0	-658.565914	-1792.982081	-1723.252332	EMD1
CTCCTCCAGCCATATC-1	singlet	2	-559.8903202	-536.6299564	-249.345504	EMD1
CTCCTCCCACATCCCT-1	singlet	2	-829.6772499	-777.1817711	-393.3101036	EMD1
CTCCTCCCACCTAAAC-1	singlet	2	-1162.953978	-910.9675149	-476.2961865	EMD1
CTCCTCCCAGTATGAA-1	singlet	0	-267.7071056	-743.850739	-754.7159393	EMD1
CTCCTCCCATGGCCAC-1	singlet	2	-663.8557479	-591.7459923	-260.5438629	EMD1
CTCCTCCGTCTAGGCC-1	singlet	1	-7433.364521	-2059.381711	-5712.292202	EMD1
CTCCTCCGTTCGATTG-1	singlet	1	-784.4330426	-294.2045831	-926.941799	EMD1
CTCCTCCGTTTCGATG-1	singlet	2	-604.971815	-451.6086602	-244.2176615	EMD1
CTCCTCCTCAACTACG-1	singlet	1	-581.5115873	-233.2994268	-460.9994756	EMD1
CTCCTCCTCCTGTAGA-1	singlet	2	-761.7558118	-585.0537735	-265.0285523	EMD1
CTCCTTTAGATTTGCC-1	singlet	1	-7757.233583	-2385.353785	-6287.877805	EMD1
CTCCTTTAGTGATTCC-1	singlet	2	-3970.967972	-3604.862372	-1334.53105	EMD1

CTCCTTTGTGTCCCTT-1	doublet	45293	-6668.764332	-3733.536982	-3970.702183	EMD1
CTCCTTTTCAATCCGA-1	singlet	2	-604.0214765	-499.8617123	-250.112166	EMD1
CTCCTTTTCATTTGTC-1	singlet	2	-653.6725502	-580.3476202	-269.6351661	EMD1
CTCGAGGAGCACCCAC-1	singlet	2	-303.4770584	-267.2804415	-113.8263228	EMD1
CTCGAGGAGCAGCCTC-1	singlet	2	-189.6583298	-141.2185292	-96.91265284	EMD1
CTCGAGGAGCTTCATG-1	singlet	1	-862.9521674	-303.7597643	-581.2126029	EMD1
CTCGAGGAGGCGACAT-1	singlet	0	-245.8913088	-816.4138013	-796.1143336	EMD1
CTCGAGGCAATTTCGG-1	singlet	0	-41.67638964	-125.0828913	-129.9147531	EMD1
CTCGAGGCACTAGTAC-1	singlet	0	-1152.092476	-4630.119098	-4437.811802	EMD1
CTCGAGGGTAGGAGGG-1	singlet	2	-1086.237535	-850.3425123	-343.9344725	EMD1
CTCGAGGGTCACAATC-1	singlet	1	-5212.570533	-1331.728374	-4270.468097	EMD1
CTCGAGGTCAATCTTC-1	singlet	0	-1005.255194	-4076.890101	-3445.971275	EMD1
CTCTCAGAGGCTCCCA-1	singlet	0	-362.170714	-1326.168029	-1108.528055	EMD1
CTCTCAGCACAGCTGC-1	singlet	0	-461.0464124	-1332.498852	-1272.792034	EMD1
CTCTCAGGTATTAAGG-1	singlet	1	-702.1145152	-231.718816	-771.6449267	EMD1
CTCTCAGGTTCTATCT-1	singlet	1	-7286.18373	-2296.871696	-5482.682315	EMD1
CTCTCAGTCAAAGGTA-1	doublet	0/1	-336.6532553	-478.0475075	-904.3243747	EMD1
CTCTCAGTCACGTCCT-1	singlet	1	-1120.098843	-315.6494853	-869.9590923	EMD1
CTCTCGACAACGCATT-1	singlet	0	-349.6388787	-825.4078379	-1018.586285	EMD1
CTCTCGACACGTAGAG-1	singlet	1	-2320.301608	-950.8229591	-2032.956691	EMD1
CTCTCGAGTACCCAGC-1	singlet	0	-657.1038317	-2077.453473	-1955.285902	EMD1
CTCTCGAGTCTGTAAC-1	singlet	2	-564.8987182	-473.8713416	-202.966866	EMD1
CTCTCGATCCAACTGA-1	singlet	1	-4676.277903	-1761.558955	-3733.194737	EMD1
CTCTCGATCCCATTTA-1	singlet	1	-390.9750391	-164.9286811	-385.6285415	EMD1
CTCTCGATCTGGGTCG-1	singlet	0	-1934.996324	-5871.398013	-5766.413138	EMD1
CTCTGGTAGATCACTC-1	unassigned	1/0	-37.78621438	-18.65006764	-31.63414086	EMD1
CTCTGGTAGATGGTAT-1	singlet	2	-1958.648071	-1872.295646	-709.0824196	EMD1
CTCTGGTAGCGTCAAG-1	singlet	0	-420.0286184	-1229.531698	-1013.6932	EMD1
CTCTGGTAGTAGGCCA-1	singlet	2	-236.5768151	-217.0923335	-119.6267511	EMD1
CTCTGGTCATCGAAGG-1	singlet	2	-615.2246965	-492.9443542	-231.3276582	EMD1
CTCTGGTGTACTCCGG-1	singlet	1	-3774.662904	-1200.861206	-3065.459902	EMD1
CTCTGGTGTGGCGTAA-1	singlet	2	-633.7255248	-577.6040484	-248.1214984	EMD1
CTCTGGTTCAGCGCGT-1	singlet	2	-325.47303	-258.8755342	-161.7547439	EMD1
CTCTGGTTCTTAGCAG-1	singlet	0	-168.9950417	-318.1870765	-359.4130177	EMD1
CTCTGGTTCTTCGGTC-1	singlet	2	-557.8646676	-476.4898274	-210.7290299	EMD1
CTGAATGAGATGAATC-1	doublet	45293	-783.7343367	-557.9430115	-569.8641842	EMD1
CTGAATGAGCACACCC-1	singlet	2	-1299.730398	-1085.027718	-465.474532	EMD1
CTGAATGAGCGTTCCG-1	singlet	2	-258.8696389	-238.4537692	-123.0873766	EMD1
CTGAATGCAAAGGGTC-1	singlet	2	-471.6509946	-345.0607032	-176.8945035	EMD1
CTGAATGCAACTCCCT-1	singlet	0	-489.2269343	-1409.38841	-1497.806814	EMD1
CTGAATGCACATAGCT-1	singlet	0	-264.4695964	-670.4325262	-675.7295945	EMD1
CTGAATGGTACTGCCG-1	singlet	2	-4880.494873	-4514.82474	-1509.639206	EMD1
CTGAATGGTATACCCA-1	singlet	1	-491.503979	-198.7735183	-506.1015452	EMD1
CTGAATGTCTCCCATG-1	singlet	2	-725.1637707	-640.6854678	-255.5857708	EMD1
CTGAGCGAGGCTGAAC-1	singlet	2	-415.2709078	-344.8342628	-178.5924983	EMD1
CTGAGCGCAAGTCCAT-1	singlet	0	-70.95527456	-256.0306814	-242.6645247	EMD1
CTGAGCGCACCACATA-1	singlet	0	-364.638883	-1030.699525	-1039.876997	EMD1
CTGAGCGCAGGTGTTT-1	singlet	2	-383.776958	-333.1859458	-171.5566583	EMD1
CTGAGCGGTCTCTCCA-1	singlet	1	-793.9324094	-270.4574377	-662.8022222	EMD1
CTGAGCGGTGATTGGG-1	singlet	0	-171.1881049	-419.9536967	-447.5389158	EMD1
CTGAGCGGTGGGATTG-1	singlet	2	-557.8406129	-469.9042632	-225.0492152	EMD1
CTGAGCGGTGGTATGG-1	singlet	2	-2820.798604	-2292.427456	-795.4243642	EMD1
CTGAGCGGTTACCGTA-1	singlet	1	-19.24983117	-4.149848596	-16.45972598	EMD1
CTGAGCGGTTAGAAAC-1	singlet	2	-248.789948	-292.2733901	-107.4783564	EMD1
CTGAGCGTCACGATCA-1	singlet	1	-1581.699446	-385.8577753	-1277.519733	EMD1
CTGAGCGTCCATACTT-1	singlet	0	-12.35249401	-49.96130874	-50.06267207	EMD1
CTGAGCGTCCGCGATG-1	singlet	2	-486.2008758	-468.199824	-160.0437088	EMD1
CTGAGCGTCTGGACCG-1	singlet	1	-4574.207523	-1315.448661	-3622.510229	EMD1
CTGAGGCAGACTCATC-1	singlet	2	-1013.830892	-819.0638075	-368.0967565	EMD1
CTGAGGCAGCAAATGT-1	singlet	2	-514.4780843	-460.4230457	-196.1208616	EMD1
CTGAGGCAGGCAGCTA-1	singlet	0	-1003.033739	-3036.573404	-2839.822175	EMD1
CTGAGGCGTAATACCC-1	singlet	1	-2065.64096	-759.6216816	-1618.12538	EMD1
CTGAGGCTCTCCAAGA-1	singlet	2	-568.9559765	-549.1479763	-236.8131131	EMD1
CTGAGGCTCTGGACTA-1	singlet	0	-384.7562766	-1133.119845	-1040.962071	EMD1
CTGATCCAGAGGCGGA-1	singlet	0	-474.9988468	-1508.041595	-1519.013505	EMD1
CTGATCCAGATCGACG-1	singlet	2	-719.63019	-532.836682	-208.7534276	EMD1
CTGATCCAGCGCCGTT-1	singlet	0	-107.7675504	-490.3482526	-471.163569	EMD1
CTGATCCAGGTGCTAG-1	singlet	2	-541.6352156	-451.8071827	-224.3997167	EMD1
CTGATCCCAGTAGATA-1	singlet	0	-125.7428541	-369.7247366	-396.5021566	EMD1
CTGATCCTCCGCAACG-1	singlet	1	-1173.496479	-447.3028616	-1049.076972	EMD1
CTGATCCTCTGTGCTC-1	singlet	0	-298.8105198	-993.8762136	-790.2222563	EMD1
CTGCAGGAGCTCATAC-1	singlet	1	-7679.119058	-2475.322221	-5857.480305	EMD1
CTGCAGGAGGTTGGAC-1	singlet	2	-764.4173781	-550.0712254	-268.6432006	EMD1
CTGCAGGAGTCGCCCA-1	singlet	2	-371.2560194	-301.3131355	-146.5775255	EMD1
CTGCAGGGTTCCAAAC-1	singlet	2	-419.7890534	-346.1291943	-158.2084992	EMD1
CTGCAGGTCCCTCAAC-1	doublet	45293	-3225.491722	-1478.891776	-2065.87225	EMD1
CTGCAGGTCGGATTAC-1	singlet	0	-741.3089581	-2981.303478	-2664.746569	EMD1
CTGCAGGTCTCAACCC-1	singlet	2	-3807.808623	-3054.430113	-1174.634837	EMD1
CTGCATCCACTTCTCG-1	singlet	2	-237.9918923	-187.7823882	-80.99904211	EMD1
CTGCATCGTGTCGCTG-1	singlet	0	-221.7306975	-675.6700665	-714.4623537	EMD1
CTGCATCTCCATTCAT-1	singlet	1	-1658.696542	-428.9725933	-1457.372279	EMD1
CTGCCATAGCTACTAC-1	singlet	2	-566.1430086	-574.7555937	-255.97956	EMD1
CTGCCATAGGCTCAAG-1	singlet	2	-312.2905527	-287.480402	-139.6704322	EMD1
CTGCCATAGTTCACTG-1	singlet	0	-746.768681	-2527.505221	-2193.470042	EMD1
CTGCCATAGTTCCTGA-1	singlet	2	-793.1107481	-724.2895409	-264.6906399	EMD1
CTGCCATCATCTAACG-1	singlet	2	-863.562794	-738.3017782	-368.2653152	EMD1
CTGCCATGTCTCCTGT-1	singlet	1	-560.4562483	-236.5299279	-467.8808256	EMD1
CTGCCATTCAATCTTC-1	singlet	0	-583.2820764	-2229.262411	-2286.427143	EMD1
CTGCCATTCGTAGGGA-1	singlet	1	-2612.931666	-777.3373651	-1921.599779	EMD1
CTGCCATTCTCCCATG-1	singlet	1	-2462.032653	-779.6527782	-1945.234687	EMD1
CTGCCTAAGGGAGGGT-1	singlet	2	-3550.413983	-3269.617046	-1486.539482	EMD1
CTGCCTAAGTGATAAC-1	doublet	2/0	-4758.237843	-5878.273969	-3797.637199	EMD1
CTGCCTAAGTGGAATT-1	singlet	0	-244.6770503	-1128.486244	-865.4495163	EMD1
CTGCCTAGTGTGTGTT-1	singlet	1	-5339.004567	-1431.189489	-4075.1134	EMD1
CTGCCTATCAAACCCA-1	singlet	1	-12245.94355	-3264.450599	-9138.976955	EMD1
CTGCCTATCAGCGGAA-1	singlet	1	-418.2300791	-135.6517451	-391.5154895	EMD1
CTGCCTATCCTGTAAG-1	singlet	0	-185.9713354	-518.3759491	-482.167538	EMD1
CTGCGAGAGACCAACG-1	singlet	0	-451.2616701	-1239.851044	-1323.902946	EMD1
CTGCGAGAGGCGCTTC-1	singlet	1	-831.7514241	-309.9601666	-595.306277	EMD1
CTGCGAGAGGTAGACC-1	singlet	1	-471.6666046	-147.1145048	-325.6453284	EMD1
CTGCGAGCACGGTGCT-1	singlet	1	-571.7575703	-206.0372955	-499.2857925	EMD1
CTGCGAGCATCGATAC-1	singlet	0	-753.8932274	-2599.402462	-2500.202621	EMD1
CTGCGAGCATCTTCGC-1	singlet	0	-35.21045281	-145.6816176	-126.7926726	EMD1
CTGCGAGGTCAGTCCG-1	singlet	1	-1670.485018	-601.3507961	-1350.877445	EMD1
CTGCGAGGTGCAATGG-1	singlet	2	-2468.611508	-2100.504243	-945.3246142	EMD1
CTGCGAGGTGTAAATG-1	singlet	2	-624.7093547	-509.1710259	-212.5548363	EMD1
CTGCGAGGTTTCGTAG-1	singlet	1	-3529.161274	-1038.850285	-2544.610621	EMD1
CTGCGAGTCGACACCG-1	singlet	2	-615.1943232	-540.7087031	-262.4771667	EMD1
CTGCGAGTCTAGAGCT-1	singlet	2	-397.1508435	-343.53899	-146.909272	EMD1
CTGCTCAAGGACAGCT-1	singlet	0	-52.84482071	-75.38564492	-493.5426781	EMD1

CTGCTCAAGTAACGAT-1	singlet	1	-615.894832	-267.6655516	-593.8885909	EMD1
CTGCTCAAGTCCCAGC-1	singlet	0	-62.10127559	-224.7716707	-227.2992049	EMD1
CTGCTCACAACAGAGC-1	singlet	2	-1368.016479	-1139.931087	-518.6646022	EMD1
CTGCTCACAACCTATG-1	singlet	2	-1224.528243	-1060.239305	-468.6789236	EMD1
CTGCTCACAATTCGTG-1	singlet	1	-5874.70188	-1417.727712	-4459.773227	EMD1
CTGCTCACACTTACAG-1	singlet	0	-115.0863514	-313.7945925	-345.8671117	EMD1
CTGCTCACAGTCTACA-1	singlet	0	-259.2617886	-637.4220704	-844.3461969	EMD1
CTGCTCATCACTGGTA-1	singlet	2	-234.4425534	-204.8508605	-91.31486435	EMD1
CTGCTCATCCGCTTAC-1	singlet	2	-293.4342019	-270.760461	-127.6943924	EMD1
CTGCTCATCGAGAACG-1	singlet	2	-508.3616461	-457.4252214	-177.256673	EMD1
CTGCTCATCTAAGAAG-1	singlet	2	-3187.050624	-3119.650982	-920.7796898	EMD1
CTGCTCATCTGCATGA-1	singlet	1	-419.9982934	-207.3469096	-484.0524864	EMD1
CTGGACGAGCAACTCT-1	singlet	2	-274.0023133	-236.211172	-120.7190758	EMD1
CTGGACGAGCCATTGT-1	singlet	2	-583.9133499	-512.4649159	-210.7743393	EMD1
CTGGACGAGCTTAGTC-1	singlet	2	-439.7390858	-360.3609403	-106.0137098	EMD1
CTGGACGAGGATCATA-1	unassigned	1/0	-15.71991993	-14.90250183	-238.5244637	EMD1
CTGGACGAGGGTCACA-1	singlet	2	-148.8248848	-123.4119907	-53.69776761	EMD1
CTGGACGAGTCTCGTA-1	singlet	0	-159.8658478	-474.0551918	-444.4202629	EMD1
CTGGACGAGTGATAGT-1	singlet	0	-442.4085734	-1337.511719	-1302.952671	EMD1
CTGGACGAGTTAACGA-1	singlet	2	-806.0305977	-697.4222553	-312.6963944	EMD1
CTGGACGGTACTCAAC-1	singlet	2	-1532.631328	-1214.701832	-537.7145419	EMD1
CTGGACGGTATCACCA-1	singlet	1	-4581.745128	-1796.437207	-3395.916642	EMD1
CTGGACGGTGATGAAT-1	singlet	0	-1040.162181	-2905.425859	-2764.920875	EMD1
CTGGACGGTGGTCTAT-1	singlet	1	-864.4115066	-276.4310255	-678.3118077	EMD1
CTGGACGTCTGTCGTC-1	singlet	2	-361.1069242	-278.4278286	-108.2245223	EMD1
CTGGCAGAGCAGGTCA-1	singlet	0	-783.32454	-2688.197644	-2711.748989	EMD1
CTGGCAGAGCTGCGAA-1	singlet	1	-2186.937968	-620.152588	-1460.846367	EMD1
CTGGCAGAGTCATGAA-1	singlet	2	-673.4388824	-583.4898459	-234.7503623	EMD1
CTGGCAGCAATCTCTT-1	singlet	1	-4803.840461	-1719.819528	-3912.782526	EMD1
CTGGCAGCACGATTCA-1	singlet	2	-474.6361806	-460.9502325	-166.8021923	EMD1
CTGGCAGCATGGCACC-1	singlet	0	-575.8114268	-1601.499019	-1639.462265	EMD1
CTGGCAGGTTTGGGTT-1	singlet	2	-375.4926324	-312.2540987	-136.3904602	EMD1
CTGGCAGTCCTCATAT-1	singlet	1	-1144.835514	-419.385454	-877.2672683	EMD1
CTGGCAGTCGGCAGTC-1	singlet	2	-501.3416684	-402.7483211	-196.0961246	EMD1
CTGGCAGTCGTGCTCT-1	singlet	1	-1899.616328	-626.3569119	-1437.618402	EMD1
CTGGTCTCAATCAGCT-1	singlet	0	-373.4822816	-976.7182846	-956.7216556	EMD1
CTGGTCTCACGGATCC-1	singlet	2	-98.9305415	-65.74692582	-19.32321746	EMD1
CTGGTCTCATTCGGGC-1	singlet	2	-1286.148281	-1088.365822	-465.9347677	EMD1
CTGGTCTTCAGAGCGA-1	singlet	1	-103.8421325	-30.00697397	-69.11417305	EMD1
CTGGTCTTCATGAGTC-1	singlet	1	-724.7109638	-269.2097628	-583.6434167	EMD1
CTGGTCTTCCAACCGG-1	singlet	1	-1404.561035	-423.0644155	-1122.762627	EMD1
CTGGTCTTCGTTCTGC-1	singlet	2	-196.091704	-159.834253	-52.77453989	EMD1
CTGTACCAGGTAAAGG-1	singlet	2	-2810.526765	-2293.061783	-1027.03037	EMD1
CTGTACCAGTCCTGCG-1	singlet	0	-422.667252	-1150.746626	-1219.315548	EMD1
CTGTACCAGTCTCTGA-1	singlet	1	-123.3357628	-32.63278742	-101.1793142	EMD1
CTGTACCCATCGTGCG-1	singlet	0	-1088.807369	-3413.97902	-2914.267968	EMD1
CTGTACCGTAGCCAGA-1	doublet	0/2	-1275.52167	-2504.821423	-2186.340886	EMD1
CTGTACCGTGACTCTA-1	singlet	2	-624.8845963	-551.6238041	-248.9575198	EMD1
CTGTAGAAGCAGGCAT-1	singlet	2	-2613.993591	-2477.948554	-785.0318438	EMD1
CTGTAGAAGCGAAACC-1	singlet	1	-1655.576173	-626.5489341	-1332.990633	EMD1
CTGTAGAAGTGGTGAC-1	singlet	0	-2716.758111	-11917.89118	-11519.27863	EMD1
CTGTAGACAGCTGTAT-1	singlet	2	-361.7879154	-336.6897418	-124.0789285	EMD1
CTGTAGAGTGAGTAGC-1	singlet	2	-1124.108948	-886.9248078	-357.2779481	EMD1
CTGTAGAGTGTCTTGA-1	singlet	0	-19.23196905	-44.71695704	-42.79641124	EMD1
CTGTAGATCAACCTTT-1	singlet	2	-765.1498438	-565.6788545	-250.6915637	EMD1
CTGTAGATCATAGGCT-1	singlet	0	-97.12360615	-280.3049093	-273.3583474	EMD1
CTGTAGATCCCAGCGA-1	singlet	1	-4718.084451	-1296.417077	-3679.797256	EMD1
CTGTAGATCTTCCAGC-1	singlet	0	-138.8773055	-396.5474739	-410.9386766	EMD1
CTGTATTAGCCTGTGC-1	singlet	1	-4019.511635	-1409.509986	-3199.652734	EMD1
CTGTATTAGGTTACCT-1	singlet	2	-431.0473631	-408.4124727	-209.3859178	EMD1
CTGTATTAGTAGTGCG-1	singlet	1	-2219.853024	-644.5608222	-1792.622512	EMD1
CTGTATTCAACAACAA-1	singlet	0	-82.83022688	-180.5984402	-172.3703575	EMD1
CTGTATTCACTGAGGA-1	singlet	1	-3128.740672	-1194.516512	-2767.88437	EMD1
CTGTATTCAGTCCCGA-1	singlet	0	-330.7779547	-930.6839561	-846.8031035	EMD1
CTGTATTCATGAAGCG-1	singlet	1	-1898.189275	-574.394481	-1565.429213	EMD1
CTGTATTGTATTAAGG-1	singlet	2	-1201.592812	-1093.370475	-445.3089339	EMD1
CTGTATTGTCTAACTG-1	singlet	1	-2072.099063	-668.0010572	-1583.605894	EMD1
CTGTATTGTCTGTGAT-1	singlet	2	-941.0445139	-745.9751243	-396.5109654	EMD1
CTGTATTTCAGCGTCG-1	singlet	2	-331.3409513	-308.9928443	-147.9369017	EMD1
CTGTATTTCCCAGGAC-1	singlet	1	-548.8847913	-220.3022196	-562.9759081	EMD1
CTGTATTTCTAGGCAT-1	singlet	1	-333.6918237	-119.2151865	-215.0590548	EMD1
CTGTATTTCTATGCCC-1	singlet	0	-596.9600325	-1606.136167	-1548.328976	EMD1
CTGTCGTAGGAAAGAC-1	singlet	2	-6344.277994	-5719.504093	-1626.801372	EMD1
CTGTCGTAGTTTGCTG-1	singlet	1	-7780.618118	-2185.023832	-6025.776522	EMD1
CTGTCGTCAATCTGCA-1	singlet	1	-2634.243921	-623.2219602	-2283.858879	EMD1
CTGTCGTCACAAACGG-1	singlet	1	-702.5653923	-289.5189561	-722.3263288	EMD1
CTGTCGTCACTACAGT-1	singlet	2	-2230.728949	-1752.130589	-770.5414908	EMD1
CTGTCGTGTCACAATC-1	singlet	2	-241.913618	-205.9721457	-80.95865335	EMD1
CTGTCGTGTCGAAGCA-1	singlet	1	-424.0039342	-128.0956144	-342.3793986	EMD1
CTGTCGTGTGATACCT-1	singlet	2	-239.749445	-205.7155884	-113.0388576	EMD1
CTGTCGTTCACCTACC-1	doublet	0/2	-1171.37678	-2604.368962	-2238.548311	EMD1
CTGTCGTTCCAACCAA-1	singlet	0	-238.875901	-888.7190008	-765.1566087	EMD1
CTGTCGTTCGTCGATA-1	singlet	2	-233.9118699	-159.260648	-73.49426055	EMD1
CTGTGAACAAGAATAC-1	singlet	0	-503.1817005	-1508.875849	-1351.435928	EMD1
CTGTGAACACGCACCA-1	singlet	2	-1870.646233	-2031.216195	-535.3517432	EMD1
CTGTGAAGTACTGCGC-1	singlet	0	-1364.482638	-5495.456463	-4945.271955	EMD1
CTGTGAAGTCCGAAGA-1	singlet	0	-575.6817942	-1887.793859	-2126.30038	EMD1
CTGTGAAGTCGCTTGG-1	singlet	1	-432.8286667	-154.429451	-385.4528423	EMD1
CTGTGAAGTGCACATT-1	singlet	0	-335.8483573	-840.0265228	-845.8614497	EMD1
CTGTGAATCAGGGTAG-1	singlet	2	-641.7132048	-567.4562789	-271.5742745	EMD1
CTGTGAATCTGTAAGC-1	singlet	1	-6571.748139	-1754.716684	-5431.03835	EMD1
CTGTGGGAGAATAGTC-1	singlet	2	-334.9372239	-268.2021723	-131.1672427	EMD1
CTGTGGGCACATATCG-1	singlet	1	-1024.716724	-435.316446	-931.8739773	EMD1
CTGTGGGCACTCCCTA-1	singlet	1	-3155.721032	-571.483149	-2791.494546	EMD1
CTGTGGGCAGTTAGAA-1	singlet	2	-618.574119	-517.0172671	-268.8884628	EMD1
CTGTGGGCATTCAGCA-1	singlet	2	-278.8505778	-268.6671109	-122.4845022	EMD1
CTGTGGGGTCAAGCCC-1	singlet	2	-374.2031575	-363.9308431	-145.8643554	EMD1
CTGTGGGGTCACTCAA-1	singlet	2	-470.6885885	-431.5791582	-222.9253871	EMD1
CTGTGGGGTGTAGTGG-1	singlet	0	-71.84343431	-330.9923443	-284.851308	EMD1
CTGTGGGGTTGAGGAC-1	singlet	2	-391.2665573	-342.380292	-134.2207722	EMD1
CTGTGGGGTTGCGGAA-1	singlet	0	-1264.92825	-6971.734611	-6134.357709	EMD1
CTGTGGGTCACGAACT-1	singlet	0	-746.5586304	-2169.551538	-2120.407775	EMD1
CTGTGGGTCTCACTCG-1	singlet	2	-528.9934054	-400.9797557	-193.1925113	EMD1
CTTACCGCAAGGCTTT-1	singlet	0	-60.04458616	-152.6544351	-178.2203342	EMD1
CTTACCGCACTGTTCC-1	singlet	1	-8315.88345	-2460.688705	-6174.696438	EMD1
CTTACCGCACTTGTCC-1	singlet	2	-557.9414486	-414.1781944	-178.930802	EMD1
CTTACCGGTAAGAACT-1	singlet	2	-1217.821189	-1185.736563	-510.5743745	EMD1
CTTACCGGTTGAGAGC-1	singlet	0	-252.1100086	-810.5513262	-798.1721916	EMD1
CTTACCGGTTTACGTG-1	singlet	1	-2022.034119	-840.9642318	-1618.583944	EMD1
CTTACCGTCCATGCAA-1	singlet	0	-1155.494988	-3468.447756	-3171.735417	EMD1

CTTACCGTCTCGCGTT-1	singlet	2	-1971.455721	-1905.423407	-625.2923725	EMD1
CTTACCGTCTCTCAAT-1	singlet	1	-1527.51109	-536.4374109	-1097.209491	EMD1
CTTAGGAAGATTAGAC-1	singlet	1	-3875.334921	-940.4473467	-3369.746606	EMD1
CTTAGGACACCCAAGC-1	singlet	1	-1099.402084	-410.6498255	-873.5515862	EMD1
CTTAGGAGTAGGAGTC-1	singlet	0	-212.4864122	-674.5751679	-744.4929585	EMD1
CTTAGGATCAGTGTCA-1	singlet	2	-418.9081773	-358.5758813	-198.6133044	EMD1
CTTAGGATCCGTGCGA-1	singlet	2	-542.1778729	-533.6523937	-216.2833683	EMD1
CTTAGGATCTTCGGAA-1	singlet	2	-481.0456673	-372.8661103	-185.4271688	EMD1
CTTCAATAGGTGCGAT-1	singlet	1	-465.0311834	-140.7202879	-356.1910018	EMD1
CTTCAATAGTAATTGG-1	singlet	0	-333.6468759	-929.6989145	-1169.934893	EMD1
CTTCAATCAGATTTCG-1	singlet	0	-254.7119662	-752.2786781	-913.7836719	EMD1
CTTCAATCAGGTGAGT-1	singlet	0	-43.06083362	-157.0032274	-142.5878039	EMD1
CTTCAATCATGCACTA-1	singlet	2	-831.9948878	-735.2768713	-313.7971729	EMD1
CTTCAATCATGGAGAC-1	singlet	1	-1924.13336	-486.9613887	-1535.476279	EMD1
CTTCAATGTAAGACCG-1	singlet	2	-5801.781384	-4714.94676	-2093.770414	EMD1
CTTCAATGTGTTGACT-1	singlet	2	-1172.218845	-943.2130335	-475.2765568	EMD1
CTTCAATTCCACCCTA-1	singlet	1	-4572.278862	-1163.941151	-3779.851219	EMD1
CTTCAATTCGCAGTTA-1	singlet	2	-4330.291117	-3148.494533	-1078.209075	EMD1
CTTCCGAAGCACGGAT-1	singlet	2	-680.6843278	-581.6376946	-258.8885042	EMD1
CTTCCGACATACAGCT-1	singlet	2	-427.4411734	-358.2944418	-155.0346234	EMD1
CTTCCGAGTGAGATAT-1	singlet	1	-1693.025171	-681.0123856	-1442.600003	EMD1
CTTCCGAGTTGGCTAT-1	singlet	2	-573.4145555	-480.7778295	-239.2774934	EMD1
CTTCCGAGTTTGAAAG-1	singlet	2	-449.1845949	-368.9090857	-170.4024823	EMD1
CTTCCGATCAACCTTT-1	singlet	0	-37.62643446	-139.7842027	-110.9946849	EMD1
CTTCCGATCAAGAAAC-1	singlet	2	-731.5054655	-628.9539556	-276.4110955	EMD1
CTTCCTTAGCAAGTCG-1	singlet	2	-877.3003101	-753.8476522	-333.2282242	EMD1
CTTCCTTAGCTATCTG-1	singlet	2	-1055.237062	-886.4709665	-418.4820182	EMD1
CTTCCTTAGGACTATA-1	singlet	0	-1787.076381	-6208.514958	-5984.020933	EMD1
CTTCCTTAGTCGTCTA-1	singlet	1	-7995.876223	-2498.713839	-6384.656938	EMD1
CTTCCTTCAGACAATA-1	singlet	2	-1269.030787	-1081.414441	-494.575106	EMD1
CTTCCTTCAGAGGACT-1	singlet	2	-4852.156817	-4037.833271	-1691.315233	EMD1
CTTCCTTCAGTCGCAC-1	singlet	1	-2222.594814	-661.4741073	-1687.639527	EMD1
CTTCCTTCATAGCTGT-1	singlet	2	-6056.630114	-4932.35297	-2261.901044	EMD1
CTTCCTTCATGACGAG-1	singlet	1	-2511.781038	-835.96347	-2024.668448	EMD1
CTTCCTTCATGGAGAC-1	singlet	2	-1922.134675	-1808.510935	-610.2481979	EMD1
CTTCCTTGTCGTTATG-1	singlet	0	-738.4251526	-2256.376933	-2440.999555	EMD1
CTTCCTTGTGTTGACT-1	singlet	1	-9677.983937	-3250.813655	-7537.94866	EMD1
CTTCCTTGTTGTTGAC-1	singlet	1	-5775.937789	-2881.587568	-5518.33682	EMD1
CTTCCTTTCCGTTTCG-1	doublet	0/1	-3132.633315	-4562.036011	-5711.483832	EMD1
CTTCCTTTCGCTCTAC-1	singlet	1	-2452.030723	-1059.151661	-2130.511932	EMD1
CTTCCTTTCGGACTGC-1	singlet	2	-419.3714413	-341.496597	-194.9520158	EMD1
CTTCCTTTCGTAATGC-1	singlet	2	-4066.257815	-2888.643081	-1056.290606	EMD1
CTTCGGTAGAATTCAG-1	singlet	2	-1656.831437	-1569.570277	-635.523568	EMD1
CTTCGGTAGATAGCAT-1	singlet	2	-606.2594825	-521.1834589	-210.5433363	EMD1
CTTCGGTAGGCTTTCA-1	singlet	1	-2289.518463	-462.4595526	-1955.852235	EMD1
CTTCGGTAGTATAGGT-1	singlet	1	-1329.626522	-515.049138	-1152.681777	EMD1
CTTCGGTCATTAGGAA-1	singlet	2	-235.598646	-208.7504946	-104.6312963	EMD1
CTTCGGTGTAAGGAGA-1	singlet	2	-1629.525611	-1544.375173	-639.2001326	EMD1
CTTCGGTGTATGCGTT-1	singlet	2	-661.2485766	-554.3449285	-278.6210458	EMD1
CTTCGGTTCCCTTCCC-1	singlet	0	-594.3828515	-2392.298564	-2127.26469	EMD1
CTTCTAAAGTCACGCC-1	singlet	1	-2032.738438	-501.3064008	-1489.005732	EMD1
CTTCTAACAACATCGT-1	singlet	2	-646.6455532	-626.8654942	-234.8810154	EMD1
CTTCTAACAACTTGGT-1	singlet	0	-308.2898961	-995.7622624	-979.8224806	EMD1
CTTCTAACACCAGCTG-1	singlet	2	-2879.969246	-2549.472922	-1001.68717	EMD1
CTTCTAACAGGGAGAG-1	doublet	2/0	-6046.565974	-6581.616779	-4353.350752	EMD1
CTTCTAACATTCATCT-1	singlet	0	-570.8150166	-1866.34516	-1757.940387	EMD1
CTTCTAAGTAGCGTCC-1	singlet	2	-521.4853614	-374.14615	-199.524335	EMD1
CTTCTAAGTCCATCTC-1	singlet	2	-482.0580768	-589.8252641	-233.9508136	EMD1
CTTCTAAGTTACGATC-1	singlet	0	-656.1588938	-1784.833635	-1694.703601	EMD1
CTTCTAATCCAACCAA-1	singlet	0	-40.07891098	-105.9278094	-134.5948468	EMD1
CTTCTAATCCGGTTCT-1	singlet	0	-206.3011207	-641.8483031	-548.3614935	EMD1
CTTCTAATCGCTCTCA-1	singlet	2	-981.7170292	-761.4942568	-364.0043885	EMD1
CTTCTAATCGTACACA-1	singlet	0	-269.3439155	-791.8951879	-693.3745838	EMD1
CTTCTAATCTCCCAAC-1	singlet	2	-2496.314298	-2301.862083	-971.7726733	EMD1
CTTCTCTAGCACTTTG-1	singlet	2	-420.3316317	-372.8122709	-168.1646604	EMD1
CTTCTCTAGGATCATA-1	singlet	0	-91.59324588	-235.8080662	-249.7395709	EMD1
CTTCTCTCATCATTTC-1	singlet	0	-502.5444729	-1565.486513	-1496.451963	EMD1
CTTCTCTGTAGTGGCA-1	singlet	0	-268.639301	-918.6409234	-1086.405702	EMD1
CTTCTCTTCACGATCA-1	singlet	2	-654.7731823	-556.5953196	-283.2119763	EMD1
CTTCTCTTCCCTTGTG-1	singlet	1	-1059.440113	-415.1233226	-890.1699604	EMD1
CTTCTCTTCGTCTCAC-1	singlet	0	-247.2906913	-631.6718376	-651.4641232	EMD1
CTTCTCTTCTGCGAGC-1	singlet	2	-471.1656041	-405.8018864	-182.8743126	EMD1
CTTGAGAAGACATAGT-1	singlet	2	-564.7417928	-476.4466156	-219.7198739	EMD1
CTTGAGAAGAGTTGCG-1	singlet	2	-458.5451395	-391.1669629	-197.3818269	EMD1
CTTGAGAAGTTAACGA-1	singlet	2	-948.9108472	-920.5789057	-428.0075241	EMD1
CTTGAGACAAGGCGTA-1	singlet	2	-295.8469418	-255.586907	-111.7357498	EMD1
CTTGAGACAATCCAGT-1	singlet	0	-166.8586681	-369.7885808	-442.3769853	EMD1
CTTGAGACAGGGAGAG-1	singlet	2	-573.314971	-487.3916892	-212.1954394	EMD1
CTTGAGACATGATGCT-1	singlet	1	-1788.430546	-589.8942589	-1454.309395	EMD1
CTTGAGAGTTAGTCGT-1	singlet	2	-1846.596217	-1426.441763	-617.5461738	EMD1
CTTGAGATCGGTAACT-1	singlet	1	-1033.797928	-304.184042	-838.617326	EMD1
CTTGATTCATTGTGCA-1	singlet	1	-1109.414783	-436.1345547	-891.2025804	EMD1
CTTGATTGTGGCATCC-1	singlet	0	-498.4550505	-1342.737285	-1379.531035	EMD1
CTTGATTTCAATGCAC-1	singlet	2	-159.5360106	-105.3273879	-55.55943124	EMD1
CTTGATTTCATCACTT-1	singlet	2	-750.8783135	-682.3660349	-289.2430149	EMD1
CTTGATTTCCTACCAC-1	singlet	2	-3977.668946	-3894.821876	-1218.715323	EMD1
CTTGATTTCGCCAGTG-1	singlet	2	-1076.618877	-1023.421649	-331.0484743	EMD1
CTTGATTTCTCAGTCC-1	singlet	1	-2331.033124	-685.3477586	-1708.735498	EMD1
CTTTCAAAGACTTCAC-1	singlet	1	-9182.060359	-2775.751983	-7142.73266	EMD1
CTTTCAAAGAGGTCGT-1	singlet	0	-673.5777415	-2310.581876	-2410.460452	EMD1
CTTTCAAAGCCAGACA-1	singlet	2	-385.1292161	-415.5875171	-190.0530631	EMD1
CTTTCAAAGGCAGTCA-1	singlet	0	-18.96859187	-66.52557878	-44.48529732	EMD1
CTTTCAACAACCCTAA-1	singlet	1	-608.8327133	-223.2849706	-600.3912118	EMD1
CTTTCAACAACTGCTA-1	singlet	0	-527.5073307	-1731.256253	-1691.368422	EMD1
CTTTCAACAGACCTAT-1	singlet	2	-479.7194379	-391.9199668	-164.5413463	EMD1
CTTTCAACAGCTAACT-1	singlet	0	-298.9241567	-825.9164102	-795.29462	EMD1
CTTTCAAGTCCCTCAT-1	singlet	2	-4098.619267	-3234.91098	-1188.225396	EMD1
CTTTCAAGTGGTCAAG-1	singlet	2	-463.6249103	-368.0185581	-167.5165776	EMD1
CTTTCGGAGCTTTCTT-1	singlet	2	-641.7058633	-501.9532977	-251.9444731	EMD1
CTTTCGGAGTCCCAAT-1	singlet	0	-428.7848164	-1228.269148	-1104.791412	EMD1
CTTTCGGAGTGCCGAA-1	singlet	0	-168.1564348	-606.1288301	-596.9735751	EMD1
CTTTCGGCATCCTTGC-1	singlet	1	-2439.160766	-893.0660507	-2004.655756	EMD1
CTTTCGGCATTATGCG-1	singlet	2	-3700.558075	-2880.862652	-1060.951792	EMD1
CTTTCGGGTAACACCT-1	singlet	2	-1630.469351	-1962.503741	-419.6086326	EMD1
CTTTCGGGTATCGGTT-1	singlet	0	-1655.38285	-5299.502925	-4958.554857	EMD1
CTTTCGGGTGTGAATA-1	singlet	2	-356.5657803	-348.295157	-155.5795524	EMD1
CTTTCGGGTTCCGCGA-1	singlet	2	-500.219563	-429.554835	-200.207847	EMD1
CTTTCGGGTTCTGACA-1	doublet	0/2	-377.4883053	-921.075518	-638.9865909	EMD1
CTTTCGGTCATCCTAT-1	singlet	1	-64.29186821	-18.19401859	-42.38545284	EMD1
CTTTCGGTCTCTTGCG-1	singlet	2	-423.4107136	-352.368652	-162.6029999	EMD1

GAAACCTAGGTGCCAA-1	singlet	0	-596.1918648	-2106.149593	-2324.096164	EMD1
GAAACCTGTAACTTCG-1	singlet	2	-1001.244238	-780.5966974	-333.3850359	EMD1
GAAACCTGTGTAGGAC-1	singlet	2	-4520.141488	-3490.368259	-1387.553866	EMD1
GAAACCTTCACGATCA-1	singlet	2	-1076.0964	-811.9601276	-253.0223021	EMD1
GAAACCTTCGGCTTCT-1	singlet	1	-1172.998306	-320.0751424	-847.7615842	EMD1
GAAATGAAGTAGCTCT-1	singlet	2	-588.190162	-492.4648222	-208.8221019	EMD1
GAAATGACACCACATA-1	singlet	2	-163.0475264	-135.0377324	-85.54944516	EMD1
GAAATGACACTACCCT-1	singlet	2	-63.14499672	-53.55288589	-22.06989588	EMD1
GAAATGACAGTCCGTG-1	doublet	0/2	-583.9711738	-809.9601769	-888.8656081	EMD1
GAAATGACATTATGCG-1	singlet	2	-111.5517176	-88.16674205	-35.29120407	EMD1
GAAATGAGTGGCCTCA-1	singlet	2	-3030.048222	-2555.66105	-937.3383252	EMD1
GAAATGATCAGGTAAA-1	singlet	0	-374.8310429	-1228.364972	-1090.756577	EMD1
GAAATGATCCGTTGGG-1	singlet	1	-688.1557724	-262.8573217	-668.5735611	EMD1
GAAATGATCGGTCGGT-1	singlet	0	-441.3138051	-1394.853075	-1296.544887	EMD1
GAACACTAGAGCTGCA-1	singlet	2	-2914.651286	-2183.334608	-801.8170094	EMD1
GAACACTCAAATAAGC-1	singlet	0	-2564.788651	-9419.765077	-7757.203663	EMD1
GAACACTGTTCAGCTA-1	singlet	2	-524.9297282	-374.0666355	-207.5616085	EMD1
GAACACTTCGCTCTAC-1	singlet	0	-520.4239882	-1456.715011	-1396.730994	EMD1
GAACGTTAGACACACG-1	singlet	1	-4087.091831	-1148.083401	-3616.511564	EMD1
GAACGTTAGACTTGTC-1	singlet	0	-884.4447828	-2858.37312	-2758.525582	EMD1
GAACGTTAGCGTTCAT-1	singlet	0	-164.4146587	-444.0889046	-431.8234934	EMD1
GAACGTTAGGGATCTG-1	singlet	1	-5336.893663	-1606.177004	-4063.866853	EMD1
GAACGTTCAATTTCTC-1	singlet	2	-751.1962153	-629.5798351	-297.8672067	EMD1
GAACGTTCATCTCCCA-1	singlet	2	-2132.273467	-1672.841569	-775.5277734	EMD1
GAACGTTTCAAGCGTT-1	singlet	2	-285.3224367	-234.2426201	-124.4183911	EMD1
GAACGTTTCCTACACC-1	unassigned	2	-6.131347759	-3.018663589	-2.085791221	EMD1
GAACGTTTCCTACGAA-1	singlet	0	-162.1381403	-519.4153874	-594.551168	EMD1
GAACGTTTCCTCAGGG-1	singlet	1	-3331.985048	-956.1179869	-2521.744462	EMD1
GAACTGTCAATCAGCT-1	singlet	0	-28.43322889	-72.7629016	-80.37849763	EMD1
GAACTGTCATAATCCG-1	singlet	2	-763.3146895	-652.0707168	-323.3809678	EMD1
GAACTGTTCACGTCCT-1	singlet	2	-1926.323974	-1897.813071	-700.4369245	EMD1
GAACTGTTCTCTGCCA-1	singlet	1	-3364.885172	-1076.643789	-2385.871209	EMD1
GAACTGTTCTTCGATT-1	singlet	2	-502.5047174	-514.9954563	-219.5673906	EMD1
GAAGAATAGAACGCGT-1	singlet	1	-625.816357	-252.6808572	-493.0526038	EMD1
GAAGAATAGAGAGAAC-1	singlet	2	-1244.617583	-1283.303287	-408.1405987	EMD1
GAAGAATAGCTACAAA-1	singlet	2	-1656.391996	-1551.601648	-648.5275019	EMD1
GAAGAATAGCTCGACC-1	singlet	1	-2678.271023	-799.2165457	-2014.175546	EMD1
GAAGAATCACCGGAAA-1	doublet	0/2	-750.0956493	-1132.912927	-947.3698418	EMD1
GAAGAATCACGCCAGT-1	singlet	0	-550.9678224	-1357.930015	-1285.489015	EMD1
GAAGAATCATTGACTG-1	singlet	0	-142.029011	-467.4328726	-444.3933855	EMD1
GAAGAATGTCTTGCTC-1	singlet	1	-2129.783791	-774.4717848	-1761.733984	EMD1
GAAGAATGTGTTCAGT-1	singlet	2	-2307.381363	-2251.978874	-853.2168376	EMD1
GAAGAATTCCAAGGGA-1	singlet	2	-1001.449631	-868.4221643	-383.5172128	EMD1
GAAGCCCCAGGGAGAG-1	singlet	1	-291.1238188	-99.03157564	-236.1476973	EMD1
GAAGCCCCATCTCATT-1	singlet	2	-677.4945728	-554.1084543	-258.8731872	EMD1
GAAGCCCGTAACTTCG-1	singlet	0	-269.156176	-620.3910124	-654.7686583	EMD1
GAAGCCCGTAGCGCCT-1	singlet	0	-341.0973606	-993.3742689	-951.0824262	EMD1
GAAGCCCGTCTCGACG-1	singlet	2	-400.1526497	-340.5541939	-150.1305948	EMD1
GAAGCCCGTCTGTGGC-1	singlet	0	-114.5454418	-367.5704222	-322.4004883	EMD1
GAAGCCCTCCATTTCA-1	singlet	2	-827.5279916	-661.1269472	-343.7143051	EMD1
GAAGCCCTCCGAAATC-1	singlet	2	-162.0774456	-134.7708228	-66.35806368	EMD1
GAAGCCCTCGTGCACG-1	singlet	0	-166.3755191	-637.0468617	-619.2878373	EMD1
GAAGCGAAGCCTCGTG-1	singlet	2	-5206.332422	-4468.08124	-1384.272927	EMD1
GAAGCGAAGCGATGGT-1	singlet	1	-471.289364	-152.6265474	-314.8079867	EMD1
GAAGCGACAAGCTGCC-1	singlet	0	-771.2808874	-2759.077215	-2899.955128	EMD1
GAAGCGACAGGCACTC-1	singlet	2	-2915.577755	-2057.232748	-919.0597542	EMD1
GAAGCGATCAACGTGT-1	singlet	1	-1370.749129	-463.4806169	-1035.558701	EMD1
GAAGCGATCACTGCTC-1	singlet	0	-440.9468802	-1252.946774	-1327.318609	EMD1
GAAGGACAGACAACAT-1	singlet	0	-214.2276856	-608.0905575	-656.767892	EMD1
GAAGGACAGGCTCCCA-1	singlet	2	-582.1555983	-506.1348097	-245.0933651	EMD1
GAAGGACAGTCTTCCC-1	singlet	2	-395.113033	-337.7618167	-150.4928271	EMD1
GAAGGACCAAGGCTTT-1	singlet	2	-383.3486436	-254.8449585	-162.6202248	EMD1
GAAGGACCAGCAATTC-1	singlet	2	-534.2617908	-486.380412	-209.2587922	EMD1
GAAGGACGTGTATTGC-1	singlet	2	-3204.634955	-2714.819674	-960.842297	EMD1
GAAGGACTCGACGCTG-1	singlet	0	-845.451106	-2517.993472	-2447.497886	EMD1
GAAGGGTAGCCGTTAT-1	singlet	2	-381.417488	-257.75015	-144.1049139	EMD1
GAAGGGTCACGCAGTC-1	singlet	2	-301.9026417	-273.5287394	-156.64211	EMD1
GAAGGGTCAGCATCTA-1	singlet	2	-1116.508446	-1186.252465	-392.9488111	EMD1
GAAGGGTGTCGTATGT-1	singlet	2	-751.2716038	-603.9082365	-291.3277086	EMD1
GAAGGGTGTGGTCTTA-1	singlet	2	-439.9549367	-390.9347338	-195.3957722	EMD1
GAAGTAAAGAATTTGG-1	singlet	2	-2068.146385	-1942.369355	-706.0336441	EMD1
GAAGTAAAGAGCATCG-1	singlet	2	-364.533235	-277.1681884	-136.3482761	EMD1
GAAGTAAAGCGTCAAG-1	singlet	2	-371.5051908	-307.3570389	-151.4984212	EMD1
GAAGTAAAGGTCACTT-1	singlet	2	-4072.416356	-3273.695525	-1387.713835	EMD1
GAAGTAAAGTAGCATA-1	singlet	2	-989.1558749	-887.4759218	-441.8577513	EMD1
GAAGTAACATCTAACG-1	singlet	1	-1550.170225	-523.9768401	-1348.104267	EMD1
GAAGTAACATGTGGCC-1	singlet	2	-129.475439	-94.17301868	-39.41647846	EMD1
GAAGTAAGTCGTTGCG-1	singlet	2	-421.0896756	-343.0258001	-165.1544377	EMD1
GAAGTAAGTCTACGAT-1	singlet	2	-5995.209985	-4939.160937	-2166.457944	EMD1
GAAGTAATCACATTGG-1	singlet	2	-790.6486284	-687.9370158	-307.3355392	EMD1
GAATAGAAGGCGATAC-1	singlet	2	-289.2251901	-309.4721329	-133.3509479	EMD1
GAATAGAAGGGAGGTG-1	singlet	1	-3086.082657	-783.3463727	-2382.80601	EMD1
GAATAGACACCGCTAG-1	singlet	1	-430.4601998	-173.9455099	-414.7589495	EMD1
GAATAGACACGACCTG-1	singlet	2	-446.0408836	-332.65226	-173.1790027	EMD1
GAATAGACATCAGCAT-1	singlet	2	-346.2039409	-274.2091761	-151.3512815	EMD1
GAATAGACATGTGGCC-1	singlet	2	-132.8720391	-111.5362897	-44.36261297	EMD1
GAATAGAGTCGTTGGC-1	doublet	45293	-151.4114064	-47.82173827	-83.49388936	EMD1
GAATAGATCCGATCGG-1	singlet	1	-228.3840666	-93.21102237	-184.7089182	EMD1
GAATAGATCGCCACTT-1	singlet	2	-620.7461039	-518.1224824	-230.3835189	EMD1
GAATCACAGCACTCAT-1	singlet	2	-396.4963666	-338.2758625	-172.9509007	EMD1
GAATCACAGGCACTCC-1	singlet	2	-651.2819632	-587.9641777	-255.1635828	EMD1
GAATCACCACACAGAG-1	singlet	0	-326.4457809	-1071.931521	-1026.35663	EMD1
GAATCACCAGACTGCC-1	singlet	0	-315.469356	-962.7443546	-930.1642978	EMD1
GAATCACCATCGTCCT-1	singlet	2	-370.6310611	-402.1276318	-170.5244882	EMD1
GAATCACGTAGTCGGA-1	singlet	0	-233.1491392	-614.3939479	-566.1408829	EMD1
GAATCACGTGCGCTCA-1	singlet	1	-1872.83585	-648.0193446	-1423.898712	EMD1
GAATCACGTGGGCTTC-1	singlet	0	-256.3844828	-738.9914358	-767.5704933	EMD1
GAATCACGTTGTACGT-1	singlet	2	-2076.373803	-2292.353157	-732.0638673	EMD1
GAATCACTCCACTGAA-1	doublet	45293	-1122.251389	-761.5108765	-848.7651609	EMD1
GAATCACTCTCATTTG-1	singlet	2	-578.8948691	-469.6503767	-208.8732077	EMD1
GAATCGTAGACATACA-1	singlet	0	-18.17006742	-43.41304406	-77.45241585	EMD1
GAATCGTAGGGACTGT-1	singlet	0	-425.7940887	-1412.140594	-1307.341207	EMD1
GAATCGTCAAGCAATA-1	doublet	2/0	-1517.742045	-1496.346497	-1134.26847	EMD1
GAATCGTCAATTGAGA-1	singlet	0	-202.7239783	-460.2473829	-632.5058176	EMD1
GAATCGTCAGCTGAGA-1	singlet	1	-1817.63404	-597.5874126	-1448.394505	EMD1
GAATCGTCAGGTCAAG-1	singlet	2	-1101.959715	-1010.874816	-423.9443986	EMD1
GAATCGTCAGTTTCGA-1	singlet	2	-258.4323214	-210.283672	-121.7229886	EMD1
GAATCGTGTTGCTAGT-1	singlet	2	-352.5953211	-344.5072538	-115.9009918	EMD1
GAATCGTTCATCGTAG-1	singlet	2	-5359.874379	-4533.624126	-1690.62164	EMD1
GAATCGTTCGTCGCTT-1	singlet	2	-2922.950952	-2226.131804	-1026.946682	EMD1

GACACGCAGTGTTCCA-1	singlet	2	-561.3694436	-567.1199693	-261.1666804	EMD1
GACACGCCACAAGCCC-1	singlet	2	-711.0285678	-753.9417454	-353.1526527	EMD1
GACACGCCATGAGTAA-1	singlet	1	-170.2932209	-56.70261863	-128.0713298	EMD1
GACACGCGTACGGTTT-1	singlet	1	-2017.071653	-552.1144163	-1368.338194	EMD1
GACACGCGTAGGTAGC-1	singlet	2	-363.4057116	-297.2837277	-150.2031026	EMD1
GACACGCGTCTACATG-1	singlet	2	-417.5423593	-427.6302156	-184.0532992	EMD1
GACACGCTCAAGATAG-1	singlet	0	-198.353065	-471.7108699	-504.9636259	EMD1
GACACGCTCTGTCAGA-1	singlet	2	-740.9839616	-631.3955548	-272.3387444	EMD1
GACAGCCAGCGACATG-1	singlet	2	-4598.68851	-3625.60077	-1489.028888	EMD1
GACAGCCCAAATAGCA-1	singlet	2	-637.3922644	-485.3085348	-244.371491	EMD1
GACAGCCCAAGATTGA-1	doublet	0/1	-648.0689026	-1124.451929	-1358.31975	EMD1
GACAGCCCAATAGTAG-1	singlet	1	-2244.062752	-814.3291322	-1766.706069	EMD1
GACAGCCCAATGACCT-1	singlet	2	-553.4044505	-472.981627	-196.5816401	EMD1
GACAGCCCACATAGCT-1	singlet	2	-330.74377	-239.3693145	-132.5635188	EMD1
GACAGCCCAGCTAACT-1	singlet	2	-698.1659064	-543.4719161	-244.2368215	EMD1
GACAGCCGTTCAGCGC-1	singlet	2	-530.8561128	-479.1299739	-222.9523612	EMD1
GACAGCCTCTGCAGCG-1	singlet	2	-273.6326659	-203.9285244	-123.0054961	EMD1
GACAGCCTCTGTCAGA-1	singlet	2	-201.6249117	-153.5205783	-63.88761293	EMD1
GACATCAAGCCTATCA-1	singlet	2	-392.5784011	-373.8700826	-172.8399287	EMD1
GACATCACACGCACCA-1	singlet	1	-1386.210466	-495.5410273	-1169.731821	EMD1
GACATCACACTACCGG-1	singlet	0	-53.46170031	-121.8310099	-136.7941958	EMD1
GACATCACACTCAGAT-1	singlet	2	-253.4898678	-273.2334207	-126.0348119	EMD1
GACATCAGTATACGGG-1	singlet	1	-2178.684504	-729.9313391	-1830.214894	EMD1
GACATCAGTTCCCAAA-1	singlet	2	-737.7952624	-578.4247145	-255.6682586	EMD1
GACATCAGTTTCGGCG-1	singlet	2	-370.1748714	-351.9138578	-165.8775458	EMD1
GACCAATAGATGAACT-1	singlet	2	-3060.814879	-2438.079263	-1151.091276	EMD1
GACCAATAGGATTCAA-1	singlet	0	-233.9677122	-652.1858854	-622.8811773	EMD1
GACCAATAGGCCTAAG-1	singlet	2	-711.6388434	-558.3482644	-236.7163197	EMD1
GACCAATGTTTGGAGG-1	unassigned	0	-4.096521915	-6.93536398	-7.487681164	EMD1
GACCAATTCAGGTAAA-1	singlet	0	-351.0361608	-1230.571227	-1236.838949	EMD1
GACCAATTCTAGAGCT-1	singlet	2	-452.5499068	-474.1329364	-232.1765742	EMD1
GACCCAGCAGGTCCCA-1	singlet	1	-1623.29739	-608.7585403	-1429.221506	EMD1
GACCCAGCAGTTGCGC-1	singlet	1	-2965.923955	-1050.098322	-2388.958103	EMD1
GACCCAGGTTAGGAGC-1	singlet	1	-707.729726	-245.1865201	-516.2682646	EMD1
GACCCAGTCACCCATC-1	singlet	0	-187.9528649	-488.1566569	-530.3122794	EMD1
GACCCAGTCCCTCTAG-1	singlet	2	-809.0575068	-610.4560858	-309.3106422	EMD1
GACCCAGTCGAATCCA-1	singlet	2	-1678.807167	-1421.128598	-671.3261469	EMD1
GACCCTTAGCACTGGA-1	singlet	1	-1447.456792	-529.4020803	-1265.611604	EMD1
GACCCTTAGTATGAGT-1	singlet	2	-180.1284668	-135.1182701	-65.57490258	EMD1
GACCCTTAGTCTCGTA-1	singlet	2	-366.9173819	-349.6281986	-158.5504364	EMD1
GACCCTTAGTTCCGGC-1	singlet	0	-387.8313	-1298.09557	-1110.238555	EMD1
GACCCTTCACAGTGAG-1	singlet	2	-496.1725096	-405.9539049	-198.5546583	EMD1
GACCCTTCACCGGCTA-1	singlet	2	-361.3247845	-356.2914181	-167.6274302	EMD1
GACCCTTCACTACAGT-1	singlet	2	-1500.204767	-1285.689183	-477.8154011	EMD1
GACCCTTCAGAGGACT-1	singlet	2	-1858.025897	-1623.300771	-634.7748527	EMD1
GACCCTTGTGCTCCGA-1	singlet	1	-2218.496718	-806.8883777	-1771.16566	EMD1
GACCCTTGTGTCGCTG-1	singlet	1	-8901.564545	-2074.335632	-7132.515613	EMD1
GACCCTTGTTCGGCTG-1	singlet	2	-1382.668889	-1133.963905	-500.5246251	EMD1
GACCCTTGTTGATCGT-1	singlet	1	-1054.64292	-399.5896616	-799.8734627	EMD1
GACCCTTTCGGAATTC-1	singlet	0	-257.4882484	-818.2381925	-712.794956	EMD1
GACCCTTTCTTTACAC-1	singlet	0	-765.7378906	-2182.371642	-2234.127331	EMD1
GACCGTGAGACACACG-1	singlet	2	-3558.69375	-2833.59969	-1347.970063	EMD1
GACCGTGAGGTCTACT-1	singlet	2	-4643.472826	-3889.629618	-1842.704099	EMD1
GACCGTGGTCACCACG-1	singlet	2	-6400.090006	-6548.761844	-1840.644006	EMD1
GACCGTGGTCTGCGCA-1	singlet	1	-375.8893875	-135.3332944	-383.8636827	EMD1
GACCGTGTCACGTAGT-1	singlet	2	-1127.685765	-898.426067	-427.4929263	EMD1
GACCGTGTCCAAGCCG-1	singlet	2	-532.3393611	-498.8898079	-239.3010925	EMD1
GACCGTGTCCAGCACG-1	singlet	0	-274.7712055	-956.2297202	-1252.134271	EMD1
GACCGTGTCGTCAACA-1	singlet	1	-1825.700338	-613.2630485	-1436.443651	EMD1
GACCTTCAGACAACTA-1	singlet	1	-14561.78359	-3041.94118	-10719.14584	EMD1
GACCTTCAGTAGCAAT-1	singlet	0	-173.222769	-440.7937658	-532.6418938	EMD1
GACCTTCCAAGACCTT-1	singlet	1	-712.9536526	-294.005909	-663.3913598	EMD1
GACCTTCGTAGTTCCA-1	singlet	0	-79.81776176	-325.2661869	-378.9908319	EMD1
GACCTTCGTATAGCTC-1	singlet	0	-2060.442275	-10401.20933	-9007.93286	EMD1
GACCTTCGTCCGGCAT-1	singlet	2	-567.2606755	-475.0633819	-218.3476077	EMD1
GACGCTGAGAATTCAG-1	singlet	1	-16784.51123	-5410.926052	-13263.96526	EMD1
GACGCTGAGACATATG-1	singlet	0	-145.5235897	-539.7198426	-470.8887476	EMD1
GACGCTGAGATAGCTA-1	singlet	1	-4910.18321	-1342.181789	-4148.391895	EMD1
GACGCTGAGGCCTTCG-1	singlet	0	-1640.539829	-4725.613975	-4803.311626	EMD1
GACGCTGCAAGGTACG-1	singlet	0	-29.66781138	-116.3716629	-94.15783938	EMD1
GACGCTGCATAATGCC-1	singlet	2	-700.8091403	-653.1675886	-309.0612656	EMD1
GACGCTGGTAGGAGTC-1	singlet	1	-2437.158695	-751.8662131	-1862.715759	EMD1
GACGCTGGTATCATGC-1	singlet	2	-531.5509352	-450.4244332	-203.9881322	EMD1
GACGCTGGTGAGGCAT-1	singlet	2	-521.8705883	-424.4676272	-231.6166265	EMD1
GACGCTGGTGTCATGT-1	singlet	2	-1074.795556	-1069.580357	-454.3308315	EMD1
GACGCTGGTTACTCAG-1	singlet	1	-1487.188817	-480.4510748	-1161.544688	EMD1
GACGCTGTCAAGCTGT-1	singlet	2	-293.8732062	-325.8693476	-139.5197132	EMD1
GACGCTGTCAATGTCG-1	singlet	0	-611.3805092	-1988.966575	-2003.090357	EMD1
GACGTTAAGAAGGATG-1	singlet	2	-318.6866782	-240.5915181	-116.130657	EMD1
GACGTTAAGCCTCGTG-1	singlet	0	-337.8181143	-1006.712122	-1078.714153	EMD1
GACGTTAAGGAGGCAG-1	singlet	1	-2709.050674	-1022.417937	-2256.471766	EMD1
GACGTTACACTTACAG-1	singlet	1	-538.1560487	-250.1845972	-463.1605637	EMD1
GACGTTACATGACTTG-1	singlet	2	-421.6437873	-449.8338099	-191.1838227	EMD1
GACGTTAGTTATAGCC-1	singlet	0	-1702.404364	-4687.99489	-4278.524225	EMD1
GACGTTATCATTCATC-1	singlet	1	-2857.267369	-870.8779743	-2234.224601	EMD1
GACGTTATCTGTCGTC-1	singlet	2	-280.6254072	-240.8843372	-104.0224607	EMD1
GACTATGAGAAACCAT-1	singlet	0	-163.5255818	-528.2961318	-531.3961287	EMD1
GACTATGCAATGGCCC-1	singlet	2	-814.719171	-680.2939824	-319.4324199	EMD1
GACTATGCAGCATACT-1	singlet	1	-2881.787882	-854.4924365	-2299.763884	EMD1
GACTATGCAGGATTCT-1	singlet	2	-333.3960781	-285.433802	-126.5175499	EMD1
GACTATGCATATTCGG-1	singlet	1	-2238.414826	-850.6160895	-1837.446811	EMD1
GACTATGGTACCTGTA-1	singlet	2	-6241.882983	-5000.718362	-2160.605416	EMD1
GACTATGTCACCTACC-1	doublet	45323	-943.5111286	-660.3844694	-537.3516679	EMD1
GACTATGTCACGAACT-1	singlet	2	-734.7487975	-551.5524612	-236.8643251	EMD1
GACTATGTCATTTGCT-1	singlet	1	-3729.102271	-939.000131	-2531.213101	EMD1
GACTCAAAGAGTTGAT-1	singlet	2	-1612.653261	-1846.525807	-488.4305611	EMD1
GACTCAAAGTCAGCGA-1	singlet	0	-98.12349573	-332.1672848	-353.5098606	EMD1
GACTCAACAGAATCGG-1	singlet	1	-376.3838498	-154.3355859	-329.7244707	EMD1
GACTCAACATTGACTG-1	singlet	2	-64.51100489	-64.67672057	-27.82862531	EMD1
GACTCAAGTAAGTCAA-1	singlet	2	-1515.500341	-1299.479185	-665.5027184	EMD1
GACTCAAGTACCGGAA-1	singlet	2	-515.6465235	-426.1418283	-183.2239857	EMD1
GACTCAAGTAGTTCCA-1	singlet	2	-1366.462605	-1094.903059	-429.6648437	EMD1
GACTCAAGTCGATTTG-1	singlet	2	-226.0524393	-219.1517185	-92.94434034	EMD1
GACTCAAGTTGTTGAC-1	singlet	1	-1266.880823	-413.1531231	-1052.852374	EMD1
GACTCAATCATCCTAT-1	singlet	2	-585.1594161	-574.1882545	-253.6402583	EMD1
GACTCAATCCACTTCG-1	singlet	0	-185.4458693	-468.780087	-451.8870332	EMD1
GACTCAATCCGTAGTA-1	singlet	2	-1363.89571	-1424.385542	-404.2102801	EMD1
GACTCAATCTGCGGGT-1	singlet	0	-117.7470035	-348.153447	-349.4891173	EMD1
GACTCTCCAAAGGGTC-1	singlet	0	-599.9845361	-1623.630382	-1503.41203	EMD1
GACTCTCGTGCCTGAC-1	singlet	2	-4164.044333	-3093.301337	-1205.721243	EMD1

GACTCTCGTGCCTTTC-1	singlet	2	-307.8584851	-266.4910864	-130.5749367	EMD1
GACTCTCGTGTCACAT-1	singlet	2	-501.9774331	-468.7189858	-227.430243	EMD1
GACTCTCGTTCGATTG-1	singlet	1	-1649.155073	-556.5044856	-1237.567275	EMD1
GACTCTCTCAGTCCGG-1	singlet	0	-903.4772622	-2491.987047	-2474.524199	EMD1
GACTCTCTCGACCAAT-1	singlet	2	-564.8179503	-480.43239	-256.2670507	EMD1
GACTCTCTCGATAACC-1	singlet	0	-23.74916648	-86.22079114	-51.16852198	EMD1
GACTCTCTCTTTACAC-1	singlet	0	-474.2670216	-1695.531142	-1568.656359	EMD1
GACTGATAGGCATCGA-1	singlet	1	-1878.604102	-377.8038816	-1680.221907	EMD1
GACTGATAGGCCTTGC-1	singlet	2	-1441.502179	-1117.637903	-420.7572169	EMD1
GACTGATAGTGCAGCA-1	singlet	0	-434.0928023	-1455.764497	-1627.184555	EMD1
GACTGATCAACGACTT-1	singlet	2	-733.8178978	-661.4408774	-309.3820581	EMD1
GACTGATCAACGTAAA-1	singlet	2	-1200.078793	-1266.308493	-671.2079482	EMD1
GACTGATCAATTCACG-1	singlet	1	-42.67301319	-21.65311705	-40.30025256	EMD1
GACTGATCACGTAGTT-1	singlet	2	-592.8777948	-494.4616718	-264.1212677	EMD1
GACTGATGTCCTTTGC-1	singlet	0	-339.240171	-767.8044665	-843.0820895	EMD1
GACTGATTCAATCGGT-1	singlet	1	-4796.07717	-1325.168051	-3624.145728	EMD1
GACTGATTCGCTTGAA-1	singlet	2	-353.0132455	-304.7757689	-141.411654	EMD1
GACTGATTCGGATTAC-1	singlet	1	-2796.762244	-834.4516344	-2254.517168	EMD1
GACTGATTCTTCGGAA-1	singlet	2	-444.4297563	-303.1057446	-132.7375117	EMD1
GACTTCCAGAGTCAAT-1	singlet	2	-632.7940701	-552.7459657	-242.2252815	EMD1
GACTTCCAGGTCGTAG-1	singlet	2	-2934.753247	-2879.637424	-892.966283	EMD1
GACTTCCCAAGACCTT-1	singlet	2	-4183.103904	-4503.990804	-1521.468183	EMD1
GACTTCCGTCTACGAT-1	singlet	2	-448.6194158	-339.9487678	-190.2993341	EMD1
GACTTCCGTGCGAGTA-1	singlet	2	-413.6640409	-371.7216063	-196.204831	EMD1
GACTTCCGTTACACTG-1	singlet	2	-1747.78118	-1336.553509	-557.2966121	EMD1
GACTTCCTCGCTAGCG-1	singlet	0	-842.0712659	-2520.534975	-2577.910225	EMD1
GACTTCCTCTTCCCGA-1	singlet	2	-604.6216832	-527.0849404	-193.9937991	EMD1
GAGAAATAGATGGTCG-1	singlet	0	-28.98648866	-56.57682705	-218.2421292	EMD1
GAGAAATAGGCAGGGA-1	singlet	1	-1819.975962	-632.5704974	-1444.348458	EMD1
GAGAAATAGGCTCTAT-1	singlet	0	-112.973847	-291.1436531	-287.5506248	EMD1
GAGAAATAGGGTGAGG-1	singlet	1	-4120.188979	-1247.547791	-3214.776457	EMD1
GAGAAATAGTATCCTG-1	singlet	1	-5129.782593	-1321.575367	-3654.597767	EMD1
GAGAAATAGTTCCGTA-1	singlet	1	-387.1009483	-88.80698153	-273.9979725	EMD1
GAGAAATGTTCTCTCG-1	singlet	2	-582.929353	-536.1323717	-239.7181186	EMD1
GAGAAATTCTCGAGTA-1	singlet	1	-559.7862634	-255.9360431	-464.8531787	EMD1
GAGAAATTCTGGACCG-1	singlet	1	-2835.150489	-837.1026935	-2041.902887	EMD1
GAGACCCAGATACGAT-1	singlet	0	-431.588913	-1172.413384	-1155.15442	EMD1
GAGACCCAGCATGCGA-1	singlet	2	-383.2492939	-280.1566901	-140.7515094	EMD1
GAGACCCCAAGCCTGC-1	singlet	2	-372.0576509	-307.9404817	-185.7560374	EMD1
GAGACCCCAATACCTG-1	doublet	1/0	-2679.586748	-1387.512357	-2688.922613	EMD1
GAGACCCCAGGGTTGA-1	singlet	1	-584.0991714	-236.6066442	-555.4276374	EMD1
GAGACCCGTACCAATC-1	singlet	1	-4615.962543	-1157.58781	-3278.419645	EMD1
GAGACCCGTACGCGTC-1	singlet	0	-311.6158909	-1019.905252	-918.8673872	EMD1
GAGACCCGTAGGGTAC-1	singlet	2	-1080.310086	-820.5705813	-384.4243229	EMD1
GAGACCCGTAGGTCAG-1	singlet	0	-44.00052575	-129.7706769	-134.0447863	EMD1
GAGACCCGTGATGTAA-1	singlet	0	-19.79498652	-85.04316512	-61.37335419	EMD1
GAGACCCTCAGCTAGT-1	singlet	0	-243.3490923	-882.0730227	-801.0966729	EMD1
GAGACCCTCAGGAAGC-1	singlet	2	-731.0479421	-611.3336238	-280.1001729	EMD1
GAGACCCTCCGAACGC-1	singlet	2	-290.2994404	-227.0826746	-114.2025567	EMD1
GAGACCCTCGCTGCGA-1	singlet	2	-584.9712752	-562.6815695	-250.7424243	EMD1
GAGACCCTCGGACCAC-1	singlet	2	-372.702572	-326.8566573	-143.8502727	EMD1
GAGACCCTCGTCCATC-1	singlet	0	-639.4498137	-2069.630358	-1910.718833	EMD1
GAGACCCTCTACCCAC-1	singlet	2	-359.7840377	-308.2197072	-171.0162112	EMD1
GAGACTTAGAGCAAGA-1	singlet	0	-1235.718954	-3361.631062	-3487.450428	EMD1
GAGACTTAGGATATGT-1	singlet	2	-617.0514203	-489.0609327	-196.0375495	EMD1
GAGACTTAGGCATCTT-1	singlet	0	-430.8993547	-1197.34115	-1106.355203	EMD1
GAGACTTCACGTAGTT-1	singlet	0	-390.2541438	-1032.555655	-1207.823339	EMD1
GAGACTTTCACTGGTA-1	singlet	0	-175.4688885	-496.9034783	-503.2162492	EMD1
GAGACTTTCCGTCACT-1	singlet	2	-497.7255378	-420.7958911	-213.5698425	EMD1
GAGAGGTAGCTAAACA-1	singlet	2	-858.788283	-665.0000481	-324.7059626	EMD1
GAGAGGTAGTCACTGT-1	singlet	0	-170.4129064	-792.6524811	-610.0416539	EMD1
GAGAGGTAGTTACGGG-1	singlet	0	-560.2846468	-1676.837302	-1554.645592	EMD1
GAGAGGTCAAAGGCTG-1	singlet	2	-279.3460475	-236.2945885	-132.3973113	EMD1
GAGAGGTCAACCTATG-1	singlet	2	-326.3034734	-312.0739275	-145.8327928	EMD1
GAGAGGTCACTTGAAC-1	singlet	0	-706.8276822	-2306.834986	-2078.344569	EMD1
GAGAGGTCAGTGACCC-1	singlet	2	-619.7446321	-465.6018761	-202.886874	EMD1
GAGAGGTGTCATCGCG-1	singlet	0	-655.699008	-2000.350322	-1980.192143	EMD1
GAGAGGTGTCCGACGT-1	singlet	1	-415.0597969	-157.3741371	-348.4279591	EMD1
GAGAGGTTCTATTGTC-1	singlet	0	-458.4093027	-1224.691333	-1330.296378	EMD1
GAGATGGAGAGAGAAC-1	singlet	0	-113.2463696	-287.6769036	-330.9486306	EMD1
GAGATGGAGGGATCTG-1	singlet	2	-643.5952164	-589.838574	-253.7821189	EMD1
GAGATGGGTGTTTCTT-1	doublet	0/2	-628.7994722	-1219.215647	-924.7100537	EMD1
GAGATGGTCCTAGCCT-1	singlet	0	-253.5689508	-639.7217453	-620.7831044	EMD1
GAGCCTGAGACTTCAC-1	singlet	0	-227.8872882	-540.0737365	-553.7510358	EMD1
GAGCCTGAGAGAGTTT-1	singlet	2	-500.7222618	-468.615141	-249.6525036	EMD1
GAGCCTGAGGTAAAGG-1	singlet	2	-1553.21142	-1454.806591	-509.4565515	EMD1
GAGCCTGCAACGCATT-1	unassigned	0/2	-8.709731218	-19.32730984	-10.82017685	EMD1
GAGCCTGCAGAATTCC-1	singlet	1	-299.4800501	-120.1434499	-263.9549746	EMD1
GAGCCTGGTATGAGAT-1	singlet	2	-1245.426094	-1122.756542	-400.7298665	EMD1
GAGCCTGGTATGCTTG-1	singlet	0	-229.32618	-904.2833489	-744.3760765	EMD1
GAGCCTGGTCGCGTTG-1	singlet	2	-451.7356775	-390.065065	-164.4219439	EMD1
GAGCCTGGTCGGTGAA-1	singlet	2	-3897.076899	-3044.649136	-1209.753178	EMD1
GAGCCTGGTGAGTCAG-1	singlet	2	-421.5551962	-350.4061914	-138.972721	EMD1
GAGCCTGTCCCGAGAC-1	singlet	2	-422.0407344	-351.2259616	-161.9166218	EMD1
GAGCCTGTCTCATTGT-1	singlet	1	-1777.780569	-562.1920433	-1406.270182	EMD1
GAGCTGCCACAAACGG-1	singlet	0	-216.6069599	-1465.178431	-1163.892615	EMD1
GAGCTGCCAGAGCCCT-1	singlet	2	-359.8702683	-335.5284572	-153.0111272	EMD1
GAGCTGCGTGGGTTGA-1	singlet	1	-1885.784257	-564.0019194	-1447.370629	EMD1
GAGCTGCGTTATCTGG-1	singlet	2	-3735.833793	-2637.15274	-1149.056583	EMD1
GAGCTGCGTTCAGTAC-1	singlet	0	-194.7023544	-582.0304157	-503.2202825	EMD1
GAGCTGCTCGGTGAAG-1	singlet	2	-323.9769917	-246.5641613	-118.5948819	EMD1
GAGGCAAAGACGGTCA-1	singlet	2	-330.2224796	-273.4205391	-119.5848929	EMD1
GAGGCAAAGTGCACCC-1	singlet	0	-1312.54967	-4299.632955	-4462.647564	EMD1
GAGGCAACACCTCGTT-1	singlet	1	-2717.309636	-609.2776605	-2148.986458	EMD1
GAGGCAACACGACGTC-1	singlet	0	-201.7109164	-510.7135971	-504.1185906	EMD1
GAGGCAAGTGTACGCC-1	singlet	0	-322.3948998	-1049.27078	-948.9561288	EMD1
GAGGCAATCACGTAGT-1	singlet	1	-1340.221606	-438.1177738	-969.61552	EMD1
GAGGCCTAGACATCCT-1	singlet	2	-629.1366273	-508.7786181	-226.6557401	EMD1
GAGGCCTAGCCACTCG-1	singlet	2	-2374.287823	-2092.564137	-710.3754319	EMD1
GAGGCCTCAGGTAGTG-1	singlet	1	-175.020368	-74.45617273	-132.7182689	EMD1
GAGGCCTGTTGAGAGC-1	singlet	1	-256.481647	-95.88182556	-173.2944001	EMD1
GAGGCCTTCACGGTCG-1	singlet	0	-200.4123299	-583.3943172	-486.2263094	EMD1
GAGGCCTTCACGTCCT-1	singlet	1	-3939.994946	-1204.123709	-2823.53156	EMD1
GAGGCCTTCGAGTACT-1	singlet	1	-1785.580103	-653.2887563	-1471.31472	EMD1
GAGGCCTTCGTGGCTG-1	singlet	2	-577.2662645	-567.9169841	-209.6999486	EMD1
GAGGGATAGACTTAAG-1	singlet	2	-105.0590204	-90.75013378	-35.95868169	EMD1
GAGGGATAGCGTATGG-1	singlet	1	-711.1304892	-242.9750536	-741.1875298	EMD1
GAGGGATAGGTACATA-1	singlet	2	-611.2649882	-480.6445595	-205.6810081	EMD1
GAGGGATAGTACAGAT-1	singlet	0	-680.6852857	-1722.444	-1719.582672	EMD1
GAGGGATCAATTGGTC-1	singlet	2	-467.2388863	-405.7383066	-238.5097338	EMD1

GAGGGATCACTTCAGA-1	singlet	2	-1568.582854	-1313.129068	-600.5222833	EMD1
GAGGGATCAGTCGCAC-1	singlet	1	-572.9774181	-205.6434448	-544.282864	EMD1
GAGGGATGTTATAGAG-1	singlet	1	-3570.008761	-1134.752269	-2747.239063	EMD1
GAGGGATTCCGACAGC-1	singlet	2	-255.0760353	-203.8031886	-101.5030976	EMD1
GAGGGATTCCGTATGA-1	singlet	2	-5325.79576	-4121.823079	-1539.403289	EMD1
GAGGGATTCTATTCGT-1	singlet	2	-646.1274495	-511.9472262	-190.3053703	EMD1
GAGGGATTCTCCAATT-1	singlet	2	-643.3922045	-549.1031272	-261.739592	EMD1
GAGGGTAAGAATAGTC-1	singlet	2	-171.1739986	-129.8809089	-58.94980037	EMD1
GAGGGTAAGCATGTTC-1	singlet	1	-1438.21175	-371.245231	-1138.981344	EMD1
GAGGGTAAGGTGCTAG-1	singlet	2	-386.4732402	-348.7184628	-146.6894711	EMD1
GAGGGTAAGTCTGTAC-1	singlet	0	-158.1864102	-396.1409678	-439.5550405	EMD1
GAGGGTACAGAGTTCT-1	singlet	1	-3123.666538	-941.1255512	-2467.631755	EMD1
GAGGGTACAGCAGGAT-1	singlet	2	-460.732977	-399.6255333	-154.3620368	EMD1
GAGGGTAGTCTGTAGT-1	singlet	2	-300.8406435	-294.3690135	-132.1376686	EMD1
GAGGGTATCACTCGAA-1	singlet	2	-387.6015292	-316.6662524	-170.7360134	EMD1
GAGGGTATCCCAACTC-1	singlet	0	-1124.520795	-4196.469167	-3961.59908	EMD1
GAGGGTATCTAGACCA-1	singlet	0	-487.9515964	-1422.068293	-1356.318125	EMD1
GAGTCATAGCATCAAA-1	singlet	0	-1035.632734	-3300.541105	-3380.754827	EMD1
GAGTCATCAGCCTACG-1	singlet	2	-729.6581707	-610.8508833	-298.7298293	EMD1
GAGTCATCATCTTAGG-1	singlet	0	-479.4868239	-1447.159685	-1333.068171	EMD1
GAGTCATCATGTCAGT-1	singlet	2	-2823.846662	-2378.637446	-946.8887289	EMD1
GAGTCATGTATCGTTG-1	singlet	0	-43.56751504	-123.7739271	-132.7866106	EMD1
GAGTCATGTCAAAGTA-1	singlet	0	-2197.017463	-9670.144119	-8074.504968	EMD1
GAGTCATGTCCCTGAG-1	singlet	0	-414.8148605	-1262.417143	-1182.927185	EMD1
GAGTCATGTCTGCAAT-1	singlet	1	-1960.296321	-696.9729857	-1592.223125	EMD1
GAGTCATTCAGTCTTT-1	singlet	2	-521.47622	-381.4791769	-233.7832495	EMD1
GAGTCATTCCATGCAA-1	singlet	2	-355.9853298	-284.5921683	-128.0936784	EMD1
GAGTCTAAGCGCACAA-1	singlet	1	-5588.548043	-1969.955127	-4446.873872	EMD1
GAGTCTAAGGCCTTGC-1	singlet	2	-8578.150802	-6653.219378	-2581.916469	EMD1
GAGTCTACAACCCTAA-1	singlet	2	-782.0757103	-832.0000421	-336.5970404	EMD1
GAGTCTACATCAGCTA-1	singlet	2	-2362.006314	-2214.015768	-928.3517345	EMD1
GAGTCTAGTGGAGAAA-1	singlet	1	-3277.132124	-1206.70185	-2497.796496	EMD1
GAGTCTATCAGAGTTC-1	singlet	2	-471.1012503	-376.2868433	-211.2133657	EMD1
GAGTCTATCCGCATAA-1	singlet	1	-3672.256598	-1187.289319	-2911.654566	EMD1
GAGTCTATCGGCAGTC-1	singlet	2	-211.0002195	-202.7437345	-72.14556833	EMD1
GAGTGAGAGCTTTCTT-1	singlet	2	-658.0376069	-550.9720676	-281.8928553	EMD1
GAGTGAGCAAGGCGTA-1	singlet	2	-4132.147888	-3321.283124	-1274.318322	EMD1
GAGTGAGCACCCTATC-1	singlet	1	-2229.288448	-663.008283	-1791.236178	EMD1
GAGTGAGCACGCGTGT-1	singlet	2	-338.387462	-265.7787629	-148.380122	EMD1
GAGTGAGCAGTCAGTT-1	singlet	0	-553.454305	-1658.70913	-1611.672565	EMD1
GAGTGAGTCAAGTTGC-1	singlet	1	-2012.914145	-695.2387021	-1567.12318	EMD1
GAGTGAGTCTTCGTGC-1	singlet	2	-1270.622017	-1021.188413	-476.7035963	EMD1
GAGTGTTAGACTACCT-1	singlet	2	-594.5273978	-460.1645253	-221.8232598	EMD1
GAGTGTTAGAGGCGTT-1	singlet	2	-6817.07932	-6098.022997	-1589.071741	EMD1
GAGTGTTAGCGGGTAT-1	singlet	1	-529.5385634	-234.8847488	-414.3643802	EMD1
GAGTGTTAGTAAGACT-1	singlet	2	-2700.398862	-2368.725522	-826.0003892	EMD1
GAGTGTTCAAGGGCAT-1	singlet	2	-1630.825022	-1282.141234	-583.3362701	EMD1
GAGTGTTCAGAGCGTA-1	singlet	1	-1490.653863	-468.8737828	-1136.958151	EMD1
GAGTGTTCAGCGGTCT-1	singlet	0	-1958.966025	-8699.365703	-7042.186419	EMD1
GAGTGTTGTCAAACGG-1	singlet	0	-108.063118	-358.2005553	-370.1980932	EMD1
GAGTGTTGTTCCGCGA-1	singlet	2	-314.8330318	-310.4757652	-131.1133676	EMD1
GAGTGTTGTTCGGCGT-1	doublet	0/1	-749.191339	-1168.832527	-1240.897726	EMD1
GAGTGTTGTTTACCAG-1	singlet	2	-506.2087356	-412.2836276	-182.7385908	EMD1
GAGTGTTTCACACCCT-1	singlet	0	-1676.493603	-5279.831598	-4909.207852	EMD1
GAGTGTTTCCTGATAG-1	singlet	2	-294.2884513	-246.4298149	-105.8494633	EMD1
GAGTTACAGCAGATAT-1	singlet	0	-343.3253385	-898.0917609	-996.2294598	EMD1
GAGTTACAGGAGCTGT-1	singlet	2	-709.056287	-533.9976908	-256.5670325	EMD1
GAGTTACAGTTTCGGT-1	singlet	1	-593.893832	-201.3977689	-587.6166131	EMD1
GAGTTACCAGAAATCA-1	singlet	0	-1374.089311	-3960.425985	-4018.237803	EMD1
GAGTTACGTTCTCTCG-1	doublet	45293	-1279.609476	-670.6797114	-950.1939185	EMD1
GAGTTACTCCATCACC-1	singlet	2	-490.2072702	-413.6057516	-224.823852	EMD1
GAGTTACTCCCGAGGT-1	singlet	2	-576.7321943	-498.8222887	-225.4166922	EMD1
GAGTTACTCGTAGGGA-1	singlet	0	-69.39934996	-159.7575837	-162.1378553	EMD1
GAGTTACTCTGATGGT-1	singlet	1	-1387.975991	-445.7069968	-1097.233572	EMD1
GAGTTGTAGCAGCACA-1	singlet	1	-1270.713204	-376.8923275	-885.4327587	EMD1
GAGTTGTAGCTCCCTT-1	singlet	2	-746.7638557	-700.7422256	-355.9524948	EMD1
GAGTTGTAGGAGTCTG-1	singlet	1	-1391.177874	-395.7677274	-1230.254595	EMD1
GAGTTGTAGGATTCCT-1	singlet	2	-1410.743891	-1131.261346	-518.8604648	EMD1
GAGTTGTAGGCAGCTA-1	singlet	2	-403.9887778	-334.3805254	-163.5601137	EMD1
GAGTTGTCACGCAAAG-1	singlet	0	-236.5219413	-580.0966552	-702.5918465	EMD1
GAGTTGTCATAGAATG-1	singlet	2	-267.1218525	-259.4594988	-122.9681156	EMD1
GAGTTGTGTCAAAGAT-1	singlet	0	-99.95894091	-246.605877	-242.5850148	EMD1
GAGTTGTTCCGCGAGT-1	singlet	0	-219.3133864	-570.3119356	-611.4121291	EMD1
GAGTTGTTCCGGTAAT-1	singlet	2	-2937.829954	-2332.218582	-1028.292991	EMD1
GAGTTGTTCTCGCTCA-1	singlet	2	-3755.405579	-3099.026117	-1361.770792	EMD1
GAGTTTGAGAAGCGGG-1	singlet	1	-2316.954642	-791.4807537	-1637.103377	EMD1
GAGTTTGAGCATGCAG-1	singlet	2	-402.8485991	-416.6276323	-185.7901569	EMD1
GAGTTTGAGGTCTACT-1	singlet	0	-165.1091063	-583.2854795	-541.9589482	EMD1
GAGTTTGAGTTCCGTA-1	singlet	2	-1289.258131	-1142.208736	-410.9481596	EMD1
GAGTTTGCACAGTACT-1	singlet	2	-111.139142	-81.38846902	-35.12335804	EMD1
GAGTTTGCACATAACC-1	singlet	2	-670.7928311	-583.5619612	-269.6475791	EMD1
GAGTTTGCAGCAGACA-1	singlet	1	-1453.595375	-605.3665759	-1174.829918	EMD1
GAGTTTGTCGGAGTGA-1	singlet	0	-147.0166965	-600.6943794	-486.1973107	EMD1
GATAGAAAGATACGAT-1	singlet	1	-2070.727795	-691.4415069	-1628.310862	EMD1
GATAGAAAGTACTGGG-1	singlet	0	-35.67498119	-111.1468847	-135.4546434	EMD1
GATAGAACACAACGAG-1	singlet	2	-757.4176914	-592.23198	-299.0461848	EMD1
GATAGAAGTCCAGCGT-1	singlet	2	-291.838303	-215.3848415	-116.9500335	EMD1
GATAGAAGTGCACATT-1	singlet	2	-372.8601089	-310.6940096	-117.6765898	EMD1
GATAGAAGTGTGTGGA-1	singlet	2	-493.7268715	-398.0971105	-226.8324703	EMD1
GATAGAATCGCAGAGA-1	singlet	1	-2506.264734	-761.2780699	-2019.431845	EMD1
GATAGAATCTGGCTGG-1	singlet	1	-12437.5418	-2300.156053	-8615.192747	EMD1
GATAGCTAGAAACCCG-1	singlet	2	-287.1208737	-290.7765787	-146.8102041	EMD1
GATAGCTAGAGCAGAA-1	singlet	2	-1510.979494	-1148.475382	-525.4845447	EMD1
GATAGCTAGCAGCGAT-1	singlet	1	-448.1757062	-175.9718528	-414.6917821	EMD1
GATAGCTAGCGTGCTC-1	singlet	2	-540.730387	-390.4872148	-198.1171553	EMD1
GATAGCTCATGAGAAT-1	singlet	2	-368.5909686	-306.3470451	-197.1459956	EMD1
GATAGCTGTAACAGTA-1	singlet	2	-710.2687416	-605.8579331	-250.9996624	EMD1
GATAGCTGTGCTGTCG-1	singlet	1	-497.4690201	-196.4779672	-598.7670605	EMD1
GATAGCTGTGGTGATG-1	singlet	2	-3491.280136	-2523.854068	-1118.057845	EMD1
GATAGCTGTTTATGCG-1	singlet	2	-1019.182423	-951.6393444	-426.6998081	EMD1
GATAGCTTCAAAGCCT-1	singlet	0	-199.5118757	-540.3054918	-632.2204511	EMD1
GATCACAAGACACACG-1	singlet	2	-1621.148875	-1365.485619	-662.9879156	EMD1
GATCACAAGGTGCTTT-1	singlet	2	-727.3652835	-664.0379935	-305.8120151	EMD1
GATCACACAACCCTCT-1	singlet	1	-406.2729471	-186.825576	-337.3803336	EMD1
GATCACAGTAAGGTCG-1	singlet	0	-240.1930653	-822.5262399	-788.7341077	EMD1
GATCACAGTAATGCTC-1	singlet	1	-1559.384858	-598.4575689	-1343.598022	EMD1
GATCACAGTGCCTTTC-1	singlet	2	-596.7610606	-561.9048951	-255.628953	EMD1
GATCACATCCGGTTCT-1	singlet	2	-239.3818548	-233.0199407	-112.9007074	EMD1
GATCACATCGACCATA-1	singlet	2	-71.16373133	-50.63041966	-26.35668856	EMD1
GATCAGTAGCCGTTGC-1	singlet	2	-497.827308	-495.6695682	-254.28603	EMD1

GATCAGTAGCCTCCAG-1	singlet	0	-116.7521834	-353.7533068	-370.6158225	EMD1
GATCAGTAGCGCATCC-1	singlet	2	-554.2068376	-421.7710749	-187.1030587	EMD1
GATCAGTAGGTCTTTG-1	singlet	0	-37.51628464	-119.8290592	-99.99679433	EMD1
GATCAGTCACGGGCTT-1	singlet	2	-877.5518538	-644.9196284	-306.6238875	EMD1
GATCAGTGTAACAGTA-1	singlet	0	-262.6798024	-937.8513974	-785.9624501	EMD1
GATCAGTGTACCTAGT-1	singlet	2	-405.2245634	-314.5973346	-164.6829439	EMD1
GATCAGTGTATCACGT-1	doublet	45293	-5088.296342	-2538.550637	-3410.44506	EMD1
GATCAGTTCGGAATTC-1	singlet	1	-4514.495818	-1342.298635	-3540.744025	EMD1
GATCATGAGCGAGGAG-1	singlet	2	-465.6796359	-408.8070216	-193.9800798	EMD1
GATCATGAGCGTTGTT-1	singlet	2	-271.2696934	-292.5377748	-137.1010434	EMD1
GATCATGAGCTAGAAT-1	singlet	0	-666.7417966	-1895.071238	-1895.128431	EMD1
GATCATGAGGACGGAG-1	singlet	2	-450.6613263	-448.5623567	-199.0154394	EMD1
GATCATGAGGGCAACT-1	singlet	1	-1035.313724	-412.1185947	-868.7952505	EMD1
GATCATGCACGTCTCT-1	singlet	1	-362.6332895	-145.2026538	-288.3422073	EMD1
GATCATGGTCGACTGC-1	singlet	1	-5752.465203	-1774.336374	-4420.641081	EMD1
GATCATGTCTCTCGCA-1	singlet	2	-1100.037084	-1053.10205	-325.2025189	EMD1
GATCCCTAGAGTGTGC-1	unassigned	1/0	-25.52870472	-21.50777256	-249.9507055	EMD1
GATCCCTCAACACGAG-1	singlet	1	-4567.700299	-1526.386442	-3216.663249	EMD1
GATCCCTCAGCTGAAG-1	singlet	0	-33.19416154	-117.9239296	-125.6368065	EMD1
GATCCCTGTATGAAAC-1	singlet	1	-3413.751106	-1073.722529	-2725.065125	EMD1
GATCCCTGTGGAACCA-1	singlet	2	-393.884527	-268.4753499	-147.1215268	EMD1
GATCCCTTCCCAGCGA-1	singlet	2	-1073.794675	-971.4739055	-445.7861221	EMD1
GATCCCTTCCCAGTGG-1	singlet	2	-260.6965732	-221.9590089	-115.9318038	EMD1
GATCCCTTCCTGCTAC-1	singlet	1	-786.7037334	-276.9681779	-660.6927614	EMD1
GATCCCTTCGCAGTGC-1	singlet	2	-664.1561828	-610.007876	-282.8751585	EMD1
GATCGTAAGGTCGAGT-1	singlet	2	-435.71651	-382.366583	-152.2968916	EMD1
GATCGTAAGTACTCGT-1	singlet	2	-364.3561881	-325.4833488	-120.9000958	EMD1
GATCGTACACGGTGAA-1	singlet	0	-1015.773488	-6218.574717	-5032.424267	EMD1
GATCGTACAGAACTCT-1	singlet	0	-728.9425312	-2149.505071	-2267.362491	EMD1
GATCGTACAGATACTC-1	singlet	1	-3868.828739	-1511.782342	-3070.409053	EMD1
GATCGTACAGTGGGTA-1	singlet	1	-6975.086838	-2354.936098	-5547.35192	EMD1
GATCGTAGTGAGTTGG-1	singlet	2	-600.8028301	-484.5415699	-224.9394847	EMD1
GATCGTAGTGGCAGAT-1	singlet	2	-442.2367337	-394.4181619	-198.3586222	EMD1
GATCGTAGTGTGTGGA-1	singlet	1	-770.8446786	-279.3484192	-711.5392892	EMD1
GATCGTAGTGTTGCCG-1	singlet	1	-906.7261141	-335.103605	-738.1941962	EMD1
GATCGTAGTTCCTTGC-1	singlet	1	-633.3598357	-187.4718199	-601.2278182	EMD1
GATCGTATCAGACTGT-1	singlet	1	-1445.651909	-451.3669086	-1118.653126	EMD1
GATCGTATCCGGTAAT-1	singlet	0	-485.3257486	-1435.503744	-1374.62931	EMD1
GATCGTATCGGACCAC-1	singlet	1	-1328.188792	-384.162004	-912.2081022	EMD1
GATCGTATCTATCGCC-1	singlet	1	-74.05127324	-19.75473491	-44.57020153	EMD1
GATGACTAGAGCCGTA-1	singlet	0	-871.9106234	-2585.584707	-2346.613739	EMD1
GATGACTAGAGGATGA-1	singlet	0	-21.61868615	-56.78341338	-264.2876904	EMD1
GATGACTAGGCATCAG-1	singlet	0	-225.5055284	-741.0940142	-649.7648779	EMD1
GATGACTCAGCTCATA-1	singlet	0	-1393.211016	-3973.5208	-4047.301539	EMD1
GATGACTCAGGTTCGC-1	singlet	1	-1306.48666	-449.6712004	-1061.001126	EMD1
GATGACTGTTATGACC-1	singlet	0	-602.6265508	-1711.663443	-1677.105752	EMD1
GATGACTTCCACTGGG-1	singlet	2	-763.7468614	-663.620511	-252.9057068	EMD1
GATGAGGCAAGATGTA-1	singlet	2	-484.263928	-446.4219531	-195.322617	EMD1
GATGAGGCAAGTGTCT-1	singlet	0	-415.0638857	-1253.864685	-1131.807507	EMD1
GATGAGGCAGACCAAG-1	singlet	0	-381.8494037	-1303.72363	-1272.783606	EMD1
GATGAGGCATACAGAA-1	singlet	2	-321.7036359	-270.7317026	-131.9552746	EMD1
GATGAGGGTTGTCAGT-1	singlet	2	-477.8478992	-409.3848625	-203.8805457	EMD1
GATGAGGTCAAATGCC-1	singlet	2	-160.2843364	-152.2654755	-64.43185985	EMD1
GATGAGGTCCATCTCG-1	singlet	1	-285.9286755	-70.60362353	-215.0460982	EMD1
GATGATCAGAGGACTC-1	singlet	2	-627.1448932	-552.2677716	-241.0978464	EMD1
GATGATCAGCTCCGAC-1	singlet	0	-47.35224163	-191.0522751	-176.8226631	EMD1
GATGATCAGTAAATGC-1	singlet	0	-202.314519	-536.0120279	-646.1877161	EMD1
GATGATCAGTCGCCAC-1	singlet	2	-525.183886	-432.054091	-188.687082	EMD1
GATGATCAGTCTGCAT-1	singlet	2	-3914.680971	-2892.624017	-1259.191073	EMD1
GATGATCCAGAGTGAC-1	singlet	2	-491.3980078	-405.7561383	-185.8890052	EMD1
GATGATCCAGCGTATT-1	singlet	2	-478.8447373	-440.2161553	-196.1765052	EMD1
GATGATCGTACAATAG-1	singlet	2	-3070.969638	-2401.809037	-1006.630358	EMD1
GATGATCGTCTCTCCA-1	singlet	1	-1324.017775	-430.8580009	-1092.88598	EMD1
GATGATCGTTAACCTG-1	singlet	2	-608.5059733	-560.061635	-234.8914758	EMD1
GATGATCGTTGCTGAT-1	singlet	1	-1910.764984	-645.0415231	-1472.28157	EMD1
GATGATCTCATCACAG-1	singlet	0	-1284.743515	-4689.542835	-4361.400473	EMD1
GATGATCTCATCGCAA-1	singlet	0	-206.5498608	-602.6728653	-544.3238574	EMD1
GATGATCTCATTGCCC-1	singlet	2	-2079.008595	-1875.521208	-790.7049792	EMD1
GATGATCTCCGAGATT-1	singlet	2	-352.1407248	-324.4039246	-147.0870873	EMD1
GATGCTAAGATCCAAA-1	singlet	0	-28.15525041	-58.49869186	-54.73302178	EMD1
GATGCTAAGCGCTTCG-1	singlet	0	-29.91467251	-158.3475506	-106.9296924	EMD1
GATGCTAAGGTGATAT-1	singlet	2	-649.7979932	-459.2025046	-222.3141172	EMD1
GATGCTAAGTTGGGAC-1	singlet	0	-210.7266902	-593.80582	-499.2074197	EMD1
GATGCTACAAGCTACT-1	singlet	1	-540.0146719	-197.4347577	-433.4195174	EMD1
GATGCTACAGACCCGT-1	singlet	0	-192.4040479	-607.4667923	-630.3222151	EMD1
GATGCTACATAATGAG-1	singlet	0	-223.9993991	-1072.584491	-868.2001523	EMD1
GATGCTAGTCATCCGG-1	singlet	2	-577.9366507	-476.1452312	-215.9855166	EMD1
GATGCTAGTCTTGAAC-1	singlet	1	-825.52983	-257.4280693	-675.49079	EMD1
GATGCTAGTGGTACAG-1	singlet	1	-2273.144856	-774.60515	-1640.959223	EMD1
GATGCTAGTTGCCAAT-1	singlet	0	-91.91665672	-263.5264535	-268.8925219	EMD1
GATGCTATCACTTATC-1	singlet	0	-33.91800275	-116.4685845	-117.8320465	EMD1
GATGCTATCTAGTTCT-1	singlet	1	-3480.951372	-1152.03353	-2641.538283	EMD1
GATGCTATCTTGCGCT-1	singlet	2	-608.6559442	-460.928896	-222.9678966	EMD1
GATGGAGAGCTGTTAC-1	singlet	2	-562.7049064	-384.3788532	-174.4279809	EMD1
GATGGAGCAACGCCCA-1	singlet	0	-370.9956131	-1320.788504	-1286.770224	EMD1
GATGGAGTCAGTGCGC-1	singlet	0	-168.7559682	-518.9049189	-392.2123104	EMD1
GATGTTGAGTCCCGGT-1	singlet	0	-49.03471961	-153.0352629	-153.3893243	EMD1
GATGTTGCAAGCCCAC-1	singlet	2	-931.110038	-853.3806898	-381.3618165	EMD1
GATGTTGCAAGGACAC-1	singlet	2	-407.0078814	-324.9895032	-141.2224889	EMD1
GATGTTGCATCGCTCT-1	singlet	0	-343.5028399	-1559.459481	-1157.003246	EMD1
GATGTTGCATGATAGA-1	singlet	0	-1316.414703	-4745.456732	-4348.370966	EMD1
GATGTTGTCCAACTAG-1	singlet	2	-356.2580919	-265.6424011	-141.7089163	EMD1
GATGTTGTCTAGCCAA-1	singlet	2	-696.6005769	-593.2629575	-256.1595232	EMD1
GATGTTGTCTCACTCG-1	singlet	2	-300.9235626	-262.1754795	-137.2220741	EMD1
GATGTTGTCTGGGCAC-1	singlet	0	-930.8437755	-2993.986808	-2957.922714	EMD1
GATTCGAAGCGTCTGC-1	singlet	0	-156.5759804	-404.1543883	-496.7513699	EMD1
GATTCGAAGGAAGTAG-1	singlet	0	-32.21908446	-113.6071918	-105.4679321	EMD1
GATTCGAAGGCACTAG-1	singlet	0	-388.838137	-1210.915452	-1102.010634	EMD1
GATTCGACACCATAAC-1	singlet	1	-3158.677518	-801.2626992	-2587.125093	EMD1
GATTCGAGTGCCCTTT-1	singlet	2	-734.6005254	-771.893634	-307.1954779	EMD1
GATTCGATCACTCTTA-1	singlet	2	-433.7620594	-271.65508	-153.4325833	EMD1
GATTCGATCGTAGTCA-1	singlet	0	-111.0182895	-334.9869809	-376.0275401	EMD1
GATTCTTAGATAACGT-1	singlet	2	-1671.578153	-1573.575232	-634.9229824	EMD1
GATTCTTAGCAGGCTA-1	singlet	2	-410.8314066	-340.2168252	-150.6461672	EMD1
GATTCTTAGGTCGCCT-1	singlet	0	-37.89879308	-129.3466385	-100.6887303	EMD1
GATTCTTCAGCTATAC-1	singlet	2	-451.8717056	-432.1282118	-220.4035421	EMD1
GATTCTTCAGCTCTGG-1	singlet	0	-684.8431927	-2335.197137	-2271.14809	EMD1
GATTCTTGTACAGGTG-1	singlet	2	-457.4252679	-347.9099101	-199.8343399	EMD1
GATTGGTAGACGGTCA-1	singlet	0	-610.0783791	-1843.111103	-1965.204749	EMD1
GATTGGTAGCACCGTC-1	singlet	1	-3327.652426	-1291.125702	-2998.527796	EMD1

GATTGGTAGGAAAGAC-1	singlet	2	-806.5698665	-726.3086197	-282.5557937	EMD1
GATTGGTAGTCCGCGT-1	singlet	2	-406.7610042	-294.248378	-159.8202068	EMD1
GATTGGTCACACGGTC-1	singlet	0	-139.606522	-374.4978391	-426.6316769	EMD1
GATTGGTCAGCTCATA-1	singlet	2	-294.9704289	-303.9298594	-135.6092547	EMD1
GATTGGTGTCGCTTGG-1	singlet	0	-832.6779436	-2242.09074	-2135.191618	EMD1
GATTGGTTCGAGTGGA-1	singlet	0	-719.1289044	-2086.045504	-2029.948245	EMD1
GATTTCTAGTGATTCC-1	singlet	1	-1785.772966	-515.4449374	-1431.354332	EMD1
GATTTCTCAATTCGTG-1	singlet	2	-813.5969269	-662.2514632	-312.6749274	EMD1
GATTTCTCACATAACC-1	singlet	0	-240.9068683	-709.8004051	-842.1822001	EMD1
GATTTCTCACGTGTGC-1	singlet	2	-525.5123997	-407.5441936	-230.8471581	EMD1
GATTTCTTCCATCACC-1	singlet	2	-681.8605222	-627.4837278	-295.8818237	EMD1
GATTTCTTCCGCTGTT-1	singlet	0	-926.0396369	-1789.647827	-1529.876844	EMD1
GATTTCTTCTGGGATT-1	singlet	2	-268.197291	-241.8145212	-110.0895866	EMD1
GCAACATAGAGGCGGA-1	singlet	2	-2264.617328	-2127.702166	-826.7359793	EMD1
GCAACATAGCGTTCCG-1	singlet	2	-407.7142273	-410.3584244	-171.0583897	EMD1
GCAACATAGTAACGAT-1	singlet	2	-1334.254634	-1038.510481	-465.1200797	EMD1
GCAACATCAAGTGGGT-1	singlet	2	-1475.991636	-1440.692268	-565.6046839	EMD1
GCAACATCACGGCACT-1	singlet	1	-3419.956663	-1214.985336	-2665.351398	EMD1
GCAACATCAGAGTTCT-1	singlet	1	-2442.949867	-858.4175755	-2055.22681	EMD1
GCAACATCAGGCGATA-1	singlet	0	-423.437325	-1492.395299	-1412.199024	EMD1
GCAACATCAGGTACGA-1	singlet	2	-2162.124247	-1856.985112	-801.6677755	EMD1
GCAACATTCAAATAGG-1	singlet	1	-1633.737868	-603.9084482	-1271.998679	EMD1
GCAACCGAGAAGTGTT-1	singlet	1	-3276.158194	-984.7788599	-2681.595196	EMD1
GCAACCGAGCTCTATG-1	singlet	2	-628.030073	-506.9254651	-244.3503558	EMD1
GCAACCGCAAGAGATT-1	singlet	0	-327.2676893	-998.1300818	-991.5556234	EMD1
GCAACCGCACCTCTAC-1	singlet	1	-2230.385332	-730.0170085	-1957.681418	EMD1
GCAACCGGTGAGTAGC-1	singlet	0	-554.9588407	-1810.416509	-1895.383214	EMD1
GCAACCGGTTGGTGTT-1	singlet	0	-158.2650964	-402.5030635	-413.8348203	EMD1
GCAACCGTCCCGTGAG-1	singlet	0	-233.8343134	-629.053576	-667.6289995	EMD1
GCACATAAGAGAGTTT-1	singlet	2	-653.5030094	-550.6392975	-271.9408476	EMD1
GCACATAAGATGGCAC-1	singlet	2	-678.6875879	-549.2374579	-225.128647	EMD1
GCACATAAGGCACGAT-1	singlet	2	-3342.259037	-2802.491002	-1293.900087	EMD1
GCACATAAGGTTTGAA-1	singlet	2	-3322.620208	-2663.169653	-1048.43854	EMD1
GCACATACAGAAGTGC-1	singlet	2	-228.8806911	-189.2945009	-93.67159864	EMD1
GCACATACATCACCAA-1	singlet	2	-523.8432796	-481.3447412	-203.7432948	EMD1
GCACATACATTACTCT-1	doublet	0/2	-551.7611788	-771.6190507	-637.903718	EMD1
GCACATAGTACAAGTA-1	singlet	1	-2888.358801	-1154.252133	-2087.972319	EMD1
GCACATAGTGGGACAT-1	singlet	0	-580.7616391	-1658.343885	-1475.881109	EMD1
GCACATATCAGCGCGT-1	singlet	1	-6558.948825	-1755.170472	-4832.212654	EMD1
GCACATATCGAAATCC-1	singlet	2	-405.4127846	-330.0030191	-143.1897335	EMD1
GCACATATCTTCTTCC-1	singlet	2	-340.186641	-353.4363616	-163.5613028	EMD1
GCACATATCTTTGATC-1	singlet	1	-1378.686085	-493.3779454	-1151.497023	EMD1
GCACGGTAGCGGACAT-1	singlet	2	-629.9938487	-540.4324166	-205.1656217	EMD1
GCACGGTAGCTGAGTG-1	singlet	1	-184.7424679	-66.10675531	-152.6395315	EMD1
GCACGGTAGTAGGAAG-1	singlet	2	-863.210541	-724.5890952	-318.783245	EMD1
GCACGGTAGTAGGCCA-1	singlet	0	-47.78216008	-149.3673607	-120.2895812	EMD1
GCACGGTCAAATAGCA-1	singlet	0	-456.4869802	-1901.648863	-1712.43405	EMD1
GCACGGTCATGACGAG-1	singlet	0	-143.4784638	-422.9456385	-445.9931062	EMD1
GCACGGTGTATGAAGT-1	singlet	0	-79.43710764	-204.6120441	-392.2058625	EMD1
GCACGGTGTCTTGAAC-1	singlet	1	-123.4265634	-25.20614539	-80.78000925	EMD1
GCACGGTGTGGTCTGC-1	singlet	2	-361.9449284	-257.5628821	-116.5393984	EMD1
GCACGGTGTTACACAC-1	singlet	0	-132.8735382	-351.8003636	-288.0842531	EMD1
GCACGGTTCAGTCATG-1	singlet	1	-84.47940943	-24.82657592	-58.40552666	EMD1
GCACGGTTCAGTCTTT-1	singlet	2	-4257.282953	-3386.447197	-1590.354262	EMD1
GCACGGTTCTGATGGT-1	singlet	2	-1120.279416	-965.597272	-443.4030189	EMD1
GCACGGTTCTTCCACG-1	singlet	0	-45.17666641	-182.4749737	-164.3782614	EMD1
GCACGGTTCTTGCAGA-1	singlet	0	-548.0963171	-1707.971458	-1785.836957	EMD1
GCACGTGGTGGCTTAT-1	singlet	0	-603.8716235	-2003.432619	-1803.930165	EMD1
GCACTAAAGATTGCGG-1	singlet	2	-3556.331445	-2745.996398	-1353.618688	EMD1
GCACTAAAGGTTCTAC-1	singlet	2	-684.2175549	-808.1800889	-283.3697474	EMD1
GCACTAAAGTACCGGA-1	singlet	0	-225.8083775	-572.9445881	-775.2590551	EMD1
GCACTAATCGAAGCAG-1	singlet	2	-538.9160241	-448.7963789	-205.7531689	EMD1
GCACTAATCTTTACAC-1	singlet	1	-535.0925959	-232.1518237	-566.1717506	EMD1
GCAGCCAAGACCCGCT-1	singlet	2	-462.994279	-439.4361514	-202.8542242	EMD1
GCAGCCAAGAGGCGTT-1	singlet	2	-585.0090766	-469.4002551	-198.0320035	EMD1
GCAGCCAAGTAACAGT-1	singlet	0	-270.4521689	-716.4764983	-652.1432226	EMD1
GCAGCTGAGCTAATGA-1	singlet	2	-553.700539	-510.7001651	-244.2519728	EMD1
GCAGCTGAGGGCAATC-1	singlet	2	-517.994438	-404.2326342	-180.0074715	EMD1
GCAGCTGAGTGCTCGC-1	singlet	0	-66.85124446	-180.9485178	-137.6724933	EMD1
GCAGCTGCAACACACT-1	singlet	2	-10266.08998	-9100.520616	-3099.581253	EMD1
GCAGCTGGTGTTCCAA-1	singlet	0	-357.7908106	-1417.117388	-1371.768481	EMD1
GCAGCTGGTTACCTGA-1	singlet	0	-704.3464602	-1974.86758	-1837.738288	EMD1
GCAGGCTCAAATCGTC-1	singlet	0	-1084.530657	-4572.882744	-4034.508482	EMD1
GCAGGCTCAATCCAGT-1	singlet	1	-2790.256575	-895.5797627	-2292.760863	EMD1
GCAGGCTCAGCGACAA-1	singlet	1	-7187.842964	-1884.27955	-5652.219468	EMD1
GCAGGCTGTACGGTTT-1	singlet	2	-1492.049975	-1265.105368	-568.9722445	EMD1
GCAGGCTGTATGGTAA-1	doublet	45323	-871.1272366	-502.3265285	-484.6534554	EMD1
GCAGGCTGTGCATGAG-1	singlet	2	-472.9972769	-472.8331818	-220.1305803	EMD1
GCAGGCTGTGTACGCC-1	singlet	0	-203.577721	-544.0464333	-621.4339001	EMD1
GCAGGCTTCACGGGAA-1	singlet	2	-1160.867856	-1034.984033	-459.7059121	EMD1
GCAGGCTTCCCTCGTA-1	singlet	0	-479.4957627	-1474.870408	-1440.604173	EMD1
GCAGGCTTCCTCATAT-1	singlet	0	-1760.553031	-5504.242241	-5945.373697	EMD1
GCAGGCTTCGCTGATA-1	singlet	0	-145.3073605	-446.75173	-450.3836863	EMD1
GCAGTTACAAGTCATC-1	singlet	2	-3120.988783	-2396.675788	-1051.392944	EMD1
GCAGTTACAGACCATT-1	singlet	2	-2578.465653	-1913.618399	-766.5465957	EMD1
GCAGTTACAGATTTCG-1	singlet	0	-720.2164871	-2084.281666	-2187.152824	EMD1
GCAGTTACATGCCATA-1	singlet	2	-381.7099379	-333.0503975	-139.6463995	EMD1
GCAGTTAGTCACAGTT-1	singlet	0	-23.28534129	-76.80161667	-75.09492521	EMD1
GCAGTTAGTTGCTCCT-1	singlet	0	-249.2749832	-868.610332	-795.02315	EMD1
GCAGTTATCAGCGCGT-1	singlet	2	-509.9794902	-447.2086617	-184.7917927	EMD1
GCAGTTATCTCTCTAA-1	singlet	2	-1051.509041	-879.8440373	-377.1105075	EMD1
GCATCGGAGAGGATGA-1	singlet	0	-148.0812863	-420.4414319	-431.6514776	EMD1
GCATCGGAGGACTATA-1	singlet	2	-331.3165613	-278.9105284	-139.5198423	EMD1
GCATCGGAGTCTTCCC-1	singlet	0	-76.23400383	-240.3015022	-236.5080223	EMD1
GCATCGGAGTGTGTTC-1	singlet	2	-308.4630698	-231.4746706	-97.5610035	EMD1
GCATCGGCAACCCTCT-1	singlet	2	-245.5329346	-175.8843974	-75.52789896	EMD1
GCATCGGCAATTAGGA-1	singlet	1	-411.100565	-179.9347256	-427.9812073	EMD1
GCATCGGGTAACGGTG-1	singlet	2	-301.0601304	-221.8613414	-112.6806013	EMD1
GCATCGGGTCTAGGTT-1	singlet	0	-677.9959081	-2058.968316	-1884.980864	EMD1
GCATCGGGTTCAAACC-1	doublet	0/1	-174.030132	-235.8763368	-274.375469	EMD1
GCATCGGTCACTGCTC-1	singlet	2	-493.2905537	-398.2905299	-207.3481641	EMD1
GCATCGGTCCGTCAAA-1	singlet	2	-396.5622807	-315.4792173	-166.6229414	EMD1
GCATCGGTCGCGATCG-1	singlet	1	-178.1705407	-53.43203066	-127.1182054	EMD1
GCATCTCAGAAGGCTC-1	singlet	0	-440.3247575	-1614.884721	-1683.802613	EMD1
GCATCTCAGGGATCAC-1	singlet	0	-979.0562135	-2943.352445	-2901.692274	EMD1
GCATCTCCACACTTAG-1	singlet	0	-778.5260136	-2481.492302	-2259.379082	EMD1
GCATCTCCATGGGTCC-1	unassigned	45323	-66.69956437	-45.87499346	-31.76257424	EMD1
GCATCTCCATGTAACC-1	singlet	1	-652.6812815	-273.8972312	-460.9357595	EMD1
GCATCTCGTAGGCTGA-1	singlet	0	-33.87688829	-153.562523	-147.2010647	EMD1
GCATCTCGTGTGTGTT-1	singlet	0	-631.3524256	-1686.431732	-1660.56365	EMD1

GCATCTCTCAACGAGG-1	singlet	1	-5220.461352	-1699.098516	-4140.249572	EMD1
GCATCTCTCGCACGGT-1	singlet	2	-547.0356881	-410.0463505	-232.1705744	EMD1
GCATCTCTCTACTCAT-1	singlet	2	-567.7472241	-526.0834838	-229.3650645	EMD1
GCATGATCAAATACGA-1	singlet	2	-1370.50836	-1138.994339	-505.9291368	EMD1
GCATGATCAGCGTTTA-1	singlet	0	-1103.673932	-3383.988071	-3310.285651	EMD1
GCATGATCATAGATGA-1	singlet	1	-3041.411007	-1027.719059	-2358.617122	EMD1
GCATGATCATCCTATT-1	singlet	0	-1345.875714	-4371.177909	-4388.27775	EMD1
GCATGATGTTACGGAG-1	singlet	2	-366.9059475	-307.5732383	-123.7844605	EMD1
GCATGATTCATGGATC-1	singlet	2	-604.0689956	-491.4524244	-220.188273	EMD1
GCATGATTCCAGCTCT-1	singlet	2	-1168.686398	-1093.900613	-351.8211797	EMD1
GCATTAGAGGACAGTC-1	singlet	2	-510.2124113	-409.4951552	-152.2867557	EMD1
GCATTAGAGTCGAAAT-1	singlet	2	-112.890665	-120.1615748	-50.41683841	EMD1
GCATTAGCAATTTCTC-1	singlet	2	-484.4788241	-382.3630813	-207.1333841	EMD1
GCATTAGCACGGGCTT-1	singlet	2	-349.418189	-301.230953	-158.2774193	EMD1
GCATTAGCAGGTTACT-1	singlet	2	-352.4569369	-279.7681743	-138.6171614	EMD1
GCATTAGGTACCACGC-1	singlet	2	-433.1403495	-367.719154	-172.8983411	EMD1
GCATTAGTCTCCAAGA-1	singlet	0	-44.94793792	-91.32251367	-104.6378581	EMD1
GCCAACGAGGAAAGTG-1	singlet	2	-179.8806582	-155.4038542	-67.67454934	EMD1
GCCAACGAGTTGTCAC-1	singlet	2	-1128.464179	-1060.752646	-426.4000521	EMD1
GCCAACGCACCCGTAG-1	singlet	2	-495.3222539	-433.2754226	-198.8320572	EMD1
GCCAACGCAGGAGACT-1	singlet	2	-568.571919	-471.0967656	-201.3688288	EMD1
GCCAACGCATCCCACT-1	singlet	1	-4418.899607	-955.082276	-3558.440296	EMD1
GCCAACGTCCACGTGG-1	singlet	0	-32.14650936	-129.4721064	-284.226288	EMD1
GCCAACGTCCCAACTC-1	singlet	2	-574.1136118	-508.9018869	-258.9935923	EMD1
GCCAACGTCCGATAGT-1	singlet	1	-2549.832452	-1027.739991	-2042.402134	EMD1
GCCAACGTCGGTAGAG-1	singlet	0	-649.4655323	-1907.937757	-1848.738762	EMD1
GCCAGCAAGGACATCG-1	singlet	0	-102.8662805	-344.3552101	-281.5190369	EMD1
GCCAGCACACAGTATC-1	singlet	0	-941.1894189	-2738.047911	-2893.407307	EMD1
GCCAGCACACGACAGA-1	singlet	2	-200.8249753	-164.0721278	-97.33357825	EMD1
GCCAGCATCCTAGAGT-1	singlet	0	-38.61225181	-163.4485947	-123.6401761	EMD1
GCCAGCATCCTGATAG-1	singlet	2	-263.8508166	-237.3284525	-106.0217931	EMD1
GCCAGGTAGAACCCGA-1	singlet	0	-37.80215653	-126.4036351	-98.80136389	EMD1
GCCAGGTAGCAGCACA-1	singlet	2	-414.6403505	-375.5416111	-161.9622008	EMD1
GCCAGGTAGGTTTGAA-1	singlet	2	-1880.6284	-1685.406782	-607.0963823	EMD1
GCCAGGTAGTCAGCGA-1	singlet	0	-312.81868	-1541.417744	-1256.18681	EMD1
GCCAGGTAGTCCGCCA-1	singlet	0	-541.3836398	-1597.969528	-1549.404684	EMD1
GCCAGGTAGTTCGCAT-1	singlet	2	-299.8894349	-261.5076303	-133.7937248	EMD1
GCCAGGTGTCAAATCC-1	singlet	1	-3032.412619	-1090.198925	-2406.352444	EMD1
GCCAGGTGTGGACAGT-1	singlet	1	-1753.830351	-533.0770056	-1405.982727	EMD1
GCCAGGTGTTGTCTAG-1	singlet	0	-280.5006447	-857.8792997	-767.7335628	EMD1
GCCAGGTGTTTCTTAC-1	singlet	2	-2428.783046	-1926.286842	-847.2615448	EMD1
GCCAGGTTCGCAACAT-1	singlet	2	-570.6895276	-481.4099233	-232.741764	EMD1
GCCAGTGAGTTGGAAT-1	singlet	2	-423.732713	-401.5779426	-190.1443092	EMD1
GCCAGTGGTAGTCGTT-1	singlet	2	-533.5885991	-411.9038113	-194.1279148	EMD1
GCCAGTGTCTCACGAA-1	singlet	0	-32.22427101	-83.51784915	-93.59333263	EMD1
GCCAGTGTCTGGAGAG-1	singlet	1	-1490.705311	-535.2096744	-1154.93495	EMD1
GCCAGTGTCTTCGACC-1	singlet	1	-71.18082526	-38.54424913	-85.34112106	EMD1
GCCATGGAGCCTCTCT-1	singlet	2	-1752.410119	-1652.743331	-567.9089978	EMD1
GCCATGGAGTATGGCG-1	singlet	2	-54.0882396	-46.00931469	-17.34096704	EMD1
GCCATGGCATCGTTCC-1	singlet	2	-383.0901234	-358.4469726	-196.804604	EMD1
GCCATGGGTCGCACGT-1	doublet	2/0	-1985.439264	-2336.256078	-1490.951685	EMD1
GCCATGGTCACGATAC-1	singlet	0	-450.7126499	-1448.804879	-1360.872782	EMD1
GCCATGGTCATAGAGA-1	singlet	0	-176.8916208	-430.0403882	-441.6461092	EMD1
GCCATGGTCGATTCCC-1	singlet	0	-707.0810291	-1857.791479	-1974.823137	EMD1
GCCATTCAGCAGGCAT-1	singlet	2	-781.6488734	-713.4657975	-329.6752832	EMD1
GCCATTCAGCCAGTAG-1	singlet	2	-646.4973314	-689.6182548	-362.8888169	EMD1
GCCATTCAGCCTATTG-1	singlet	0	-170.266853	-458.9914403	-425.7779164	EMD1
GCCATTCAGCGGTAAC-1	singlet	1	-2910.29171	-1230.025591	-2100.975505	EMD1
GCCATTCAGTTAGAAC-1	singlet	1	-11577.75603	-3007.028128	-8601.339794	EMD1
GCCATTCCAACTCATG-1	singlet	1	-2190.721808	-642.5222379	-1722.017339	EMD1
GCCATTCGTCATTCCC-1	singlet	2	-597.891551	-585.720249	-236.1073204	EMD1
GCCATTCGTTTGTGGT-1	singlet	2	-446.5743341	-344.3486979	-196.8434185	EMD1
GCCATTCTCACTTCTA-1	singlet	2	-1851.554818	-1438.341702	-707.6618669	EMD1
GCCATTCTCATTGGTG-1	singlet	1	-1008.064334	-335.1361142	-818.4731651	EMD1
GCCATTCTCCATCACC-1	singlet	2	-9667.526136	-8332.201078	-3201.948987	EMD1
GCCATTCTCGCTGACG-1	singlet	1	-4843.096899	-1331.881982	-3478.122805	EMD1
GCCATTCTCTGCCTGT-1	singlet	0	-130.5893327	-291.3693517	-353.7531113	EMD1
GCCCAGAAGACGCCCT-1	singlet	0	-236.5527265	-707.4329441	-669.8653033	EMD1
GCCCAGAAGCGCCTTG-1	singlet	2	-1126.797519	-938.4856156	-334.2351587	EMD1
GCCCAGAAGCTGGCCT-1	singlet	1	-3646.020895	-715.7354007	-3013.669186	EMD1
GCCCGAAAGACAAGCC-1	singlet	2	-140.8187301	-126.8344724	-70.00733833	EMD1
GCCCGAAAGAGGTCAC-1	singlet	2	-259.0826961	-252.3487268	-128.666989	EMD1
GCCCGAAAGCCATCCG-1	singlet	0	-1065.595077	-3604.323972	-3457.16506	EMD1
GCCCGAAAGGAACGAA-1	singlet	1	-659.2043345	-279.1387611	-648.0346228	EMD1
GCCCGAACAACTCCCT-1	singlet	2	-3444.063715	-2927.399509	-1290.472372	EMD1
GCCCGAAGTACCACGC-1	doublet	0/2	-1160.824507	-1891.503864	-1517.200922	EMD1
GCCCGAAGTCACTCGG-1	singlet	2	-369.917773	-355.6465917	-166.0807337	EMD1
GCCCGAAGTGACACGA-1	singlet	2	-641.3672143	-528.3691109	-279.8910577	EMD1
GCCCGAAGTTCAATCG-1	singlet	2	-1663.144252	-1458.483941	-594.479196	EMD1
GCCCGAATCAATGTCG-1	singlet	0	-28.09587113	-48.3674412	-285.6090272	EMD1
GCCCGAATCATTTGTC-1	singlet	2	-955.6759065	-746.334675	-291.3827362	EMD1
GCCCGAATCCAGTGTA-1	singlet	0	-220.9576288	-526.1741865	-558.8694814	EMD1
GCCCGAATCCATCCGT-1	singlet	1	-440.5541155	-177.0341433	-514.4802933	EMD1
GCCCGAATCCGTAATG-1	singlet	1	-1058.801376	-309.9853079	-837.5579925	EMD1
GCCCGAATCGTAGCCG-1	singlet	2	-818.6858626	-650.5947013	-273.6111596	EMD1
GCCGATGAGCATCGAG-1	doublet	45293	-1341.151635	-758.5369638	-963.4579652	EMD1
GCCGATGAGCTTAAGA-1	singlet	2	-623.1831879	-517.9538813	-203.1538748	EMD1
GCCGATGCAACACGTT-1	singlet	1	-4788.441881	-1367.474947	-3849.341299	EMD1
GCCGATGCACCCTGAG-1	singlet	2	-620.4375159	-496.1844684	-216.5216793	EMD1
GCCGATGCAGACAAGC-1	singlet	0	-27.44686881	-54.93517114	-276.5052984	EMD1
GCCGATGCATGGATCT-1	singlet	0	-563.1995621	-1865.225975	-1530.314623	EMD1
GCCGATGGTATTTCCT-1	doublet	45293	-1175.749486	-778.1912741	-890.4803097	EMD1
GCCGATGGTCCGGTCA-1	singlet	0	-121.5105398	-367.4017726	-389.4528931	EMD1
GCCGATGTCCGTAATG-1	singlet	2	-382.4717181	-345.9279717	-172.0033596	EMD1
GCCGATGTCGCCTAGG-1	singlet	1	-750.0099758	-302.9221906	-774.4161675	EMD1
GCCGATGTCTAGCCTC-1	singlet	2	-1967.903495	-2002.721696	-833.6088001	EMD1
GCCGATGTCTCATGGA-1	singlet	2	-620.2874878	-549.5843497	-263.8169478	EMD1
GCCGTGAAGAACAAGG-1	singlet	1	-1419.617682	-439.6976213	-1157.067891	EMD1
GCCGTGAAGATTTGCC-1	singlet	2	-452.8455508	-422.3431228	-130.6707305	EMD1
GCCGTGACATCCGAGC-1	singlet	0	-62.27980792	-217.16711	-183.0549967	EMD1
GCCGTGAGTCATGGCC-1	singlet	2	-1973.135179	-1659.721796	-684.4479719	EMD1
GCCGTGAGTCGTTGCG-1	singlet	1	-2443.372947	-761.1235267	-1902.127772	EMD1
GCCGTGAGTGTTGACT-1	singlet	2	-435.6941597	-358.5929529	-118.9940858	EMD1
GCCGTGAGTTAAGGGC-1	singlet	2	-355.1469196	-267.9323765	-141.1531415	EMD1
GCCGTGATCAGGAACG-1	singlet	2	-293.8422622	-209.0346319	-95.4912716	EMD1
GCCGTGATCCCGAAAT-1	singlet	0	-597.2204158	-1825.338348	-1819.631516	EMD1
GCCGTGATCCGCAACG-1	singlet	2	-356.5880884	-286.920218	-112.6654518	EMD1
GCCTGTTAGCTGCCTG-1	singlet	0	-71.4198652	-306.2949884	-251.2490099	EMD1
GCCTGTTAGTTGGCGA-1	singlet	1	-2237.322869	-601.6594195	-1656.789269	EMD1
GCCTGTTCAGACACAG-1	singlet	2	-292.8689615	-270.8386233	-128.008767	EMD1

GCCTGTTCAGCCGTCA-1	singlet	1	-11887.94624	-3931.567961	-10245.8537	EMD1
GCCTGTTGTGGCAGAT-1	singlet	1	-2884.64523	-991.1676232	-2172.9563	EMD1
GCCTGTTGTTTACCAG-1	unassigned	1	-3.164981749	-1.028148908	-1.85224686	EMD1
GCCTGTTTCACACCCT-1	singlet	2	-1809.534022	-1444.434427	-670.190956	EMD1
GCCTGTTTCACCGACG-1	singlet	0	-624.9341161	-1738.336857	-1614.397972	EMD1
GCCTGTTTCACGATAC-1	singlet	1	-2314.361288	-496.7798121	-1915.316524	EMD1
GCCTGTTTCAGCAGAG-1	singlet	2	-250.1954517	-220.244601	-107.5505484	EMD1
GCCTGTTTCCAGCCTT-1	singlet	2	-543.0729619	-499.7272496	-204.039507	EMD1
GCCTGTTTCCATTCGC-1	singlet	1	-2530.527699	-891.0633761	-2029.218797	EMD1
GCCTGTTTCCTTATCA-1	singlet	2	-217.7331828	-189.7366315	-70.11417073	EMD1
GCCTGTTTCGCTATTT-1	singlet	2	-450.955943	-314.6028248	-163.0379976	EMD1
GCCTGTTTCTCAAAGC-1	singlet	0	-122.9464414	-323.1786283	-336.0232716	EMD1
GCGAGAAAGAGCAGTC-1	singlet	1	-963.544427	-319.461836	-800.196351	EMD1
GCGAGAAAGAGGGTCT-1	singlet	1	-3782.245895	-1012.875663	-3000.718641	EMD1
GCGAGAAAGCCTCTGG-1	singlet	1	-428.7400562	-150.3923643	-313.9785613	EMD1
GCGAGAAAGGAACGCT-1	singlet	0	-542.7646572	-1710.403933	-1704.607534	EMD1
GCGAGAACAAGAGAGA-1	singlet	0	-34.78829371	-120.9131551	-102.5979613	EMD1
GCGAGAACACGGGCTT-1	singlet	0	-404.7319147	-1006.31293	-948.1799239	EMD1
GCGAGAACAGACTCTA-1	singlet	0	-781.5194454	-2153.413314	-2206.270081	EMD1
GCGAGAAGTAGAATAC-1	singlet	0	-553.2725443	-1676.205166	-1432.305979	EMD1
GCGAGAAGTATTGACC-1	singlet	0	-1059.348228	-2853.501482	-2763.443759	EMD1
GCGAGAATCCGGTAGC-1	singlet	2	-10158.59835	-8237.812786	-3461.078845	EMD1
GCGATCGAGGAACTCG-1	singlet	2	-478.4129303	-350.4602559	-151.3830842	EMD1
GCGATCGAGTGTTGTC-1	singlet	0	-107.4051216	-368.1258928	-369.8213509	EMD1
GCGATCGAGTTGCTGT-1	singlet	0	-18.22058071	-34.41766438	-258.141137	EMD1
GCGATCGCACAGAAGC-1	singlet	2	-409.6978128	-335.7204056	-162.3007038	EMD1
GCGATCGCAGTCGGTC-1	singlet	0	-72.68245517	-198.1200451	-191.9891415	EMD1
GCGATCGCATAGGTAA-1	singlet	0	-616.2427954	-1920.231411	-1881.825479	EMD1
GCGATCGGTATTCCTT-1	singlet	1	-4801.75325	-1688.205324	-4041.882734	EMD1
GCGATCGGTTCAAGTC-1	singlet	2	-336.5062727	-307.3634452	-127.2092305	EMD1
GCGATCGGTTCGCGTG-1	singlet	1	-238.5560926	-80.09436266	-233.854653	EMD1
GCGATCGTCTGGCCGA-1	singlet	0	-212.4074655	-544.0385428	-545.2783495	EMD1
GCGGAAAAGAATAGTC-1	singlet	2	-444.5775133	-448.2568162	-205.9230589	EMD1
GCGGAAAAGAGTCTTC-1	singlet	0	-159.7707366	-569.5688123	-685.2641514	EMD1
GCGGAAAAGCACGGAT-1	doublet	1/0	-3054.071519	-2646.947987	-4610.777447	EMD1
GCGGAAAAGCTAATGA-1	singlet	2	-299.4096757	-233.6490726	-136.2099798	EMD1
GCGGAAAAGGTTCTTG-1	singlet	1	-452.2261875	-176.0828373	-454.2644026	EMD1
GCGGAAAAGTTGTAGA-1	singlet	0	-1655.422952	-4971.455739	-4724.391828	EMD1
GCGGAAAAGTTTGAGA-1	singlet	1	-2934.904324	-820.3218455	-2430.557754	EMD1
GCGGAAACAGAGCGTA-1	singlet	2	-334.0457108	-233.2214731	-89.7649882	EMD1
GCGGAAAGTCCCTCAT-1	singlet	2	-287.5649297	-274.6126606	-116.6450052	EMD1
GCGGAAAGTTGCTCAA-1	singlet	2	-296.9611038	-240.0116427	-122.0759154	EMD1
GCGGAAATCACTTGGA-1	singlet	0	-1622.75563	-5357.127655	-5136.60502	EMD1
GCGGAAATCAGGTGTT-1	singlet	2	-479.5545965	-353.4641419	-197.5581454	EMD1
GCGGAAATCGACGCGT-1	singlet	1	-1017.814856	-349.126766	-805.3804291	EMD1
GCGGAAATCTGTAACG-1	singlet	1	-1750.490749	-607.0742416	-1331.939596	EMD1
GCGGATCAGAATTGTG-1	singlet	1	-3782.048495	-1233.510016	-3122.497384	EMD1
GCGGATCAGCCTGAGA-1	singlet	2	-1347.202743	-1024.875286	-435.088329	EMD1
GCGGATCAGGAGCAAA-1	singlet	2	-316.2423113	-285.669955	-140.237139	EMD1
GCGGATCAGGATACGC-1	singlet	1	-3740.927378	-948.6985523	-2922.93266	EMD1
GCGGATCAGGTCATAA-1	singlet	0	-410.4864316	-1277.180921	-1089.986781	EMD1
GCGGATCAGTCTGCAT-1	singlet	0	-99.69504403	-300.4698702	-302.4910306	EMD1
GCGGATCCACATCATG-1	singlet	0	-2356.178047	-7669.917582	-7328.617824	EMD1
GCGGATCCATCCGATA-1	singlet	2	-824.2093471	-665.4270841	-351.1018745	EMD1
GCGGATCGTCGCAACC-1	singlet	1	-860.5477219	-270.5441178	-748.2878795	EMD1
GCGGATCTCGACATTG-1	singlet	1	-1885.104393	-531.4611149	-1556.72433	EMD1
GCGGATCTCTAATTCC-1	singlet	0	-352.1882907	-894.7746249	-776.3068863	EMD1
GCGTGCAAGATACAGT-1	singlet	1	-1443.561904	-503.1638578	-1226.32833	EMD1
GCGTGCAAGATACTGA-1	singlet	0	-1724.098705	-5138.14934	-5148.543623	EMD1
GCGTGCAAGGTTTGAA-1	singlet	2	-227.0735598	-214.8333315	-90.03285408	EMD1
GCGTGCAAGTGATGGC-1	singlet	2	-2026.748891	-1625.811997	-613.3651997	EMD1
GCGTGCACAATAGTAG-1	singlet	2	-268.6320618	-182.3093319	-93.6374067	EMD1
GCGTGCACACGGCACT-1	singlet	1	-3275.694104	-1083.60967	-2542.147367	EMD1
GCGTGCACACGTACTA-1	unassigned	1/0	-5.466686923	-1.893520305	-2.39702567	EMD1
GCGTGCACATGAAGGC-1	singlet	0	-264.9963063	-714.8366304	-750.8087942	EMD1
GCGTGCAGTCGGTGTC-1	singlet	0	-18.74715758	-106.2662624	-100.9041367	EMD1
GCGTTTCAGCTGGAGT-1	unassigned	2	-22.21386052	-6.750691729	-5.202998108	EMD1
GCGTTTCCAAGAGGTC-1	singlet	2	-2070.238172	-1534.410661	-691.1710882	EMD1
GCGTTTCCAGCATTGT-1	singlet	2	-2578.882907	-2202.613075	-808.7528032	EMD1
GCGTTTCTCACGGAGA-1	singlet	2	-425.6601829	-337.5881266	-152.1035768	EMD1
GCGTTTCTCGCCGAAC-1	singlet	0	-190.3241691	-525.402678	-621.3199417	EMD1
GCGTTTCTCTACAGGT-1	singlet	2	-1488.031448	-1213.173316	-481.7427877	EMD1
GCTACAAAGCTGAAGC-1	singlet	1	-2422.116504	-784.3608223	-1950.590228	EMD1
GCTACAAAGGGCAGGA-1	singlet	0	-772.5109419	-2441.131008	-2359.178728	EMD1
GCTACAAAGTGATAGT-1	singlet	0	-1586.959204	-4821.71001	-4933.806739	EMD1
GCTACAACACGGGTAA-1	singlet	0	-347.4068193	-1624.741457	-1509.34432	EMD1
GCTACAAGTACAGTCT-1	unassigned	2	-4.299128276	-6.888397506	-3.236555853	EMD1
GCTACAAGTGTAGTGG-1	singlet	0	-237.8744145	-932.2149055	-814.8951879	EMD1
GCTACAATCCTAAACG-1	singlet	0	-323.9072513	-1010.663959	-1018.362077	EMD1
GCTACAATCGGTATGT-1	singlet	2	-360.0312537	-308.423239	-146.3768372	EMD1
GCTACCTAGACCAGAC-1	singlet	0	-1764.855547	-5620.426584	-5294.163808	EMD1
GCTACCTAGTGTAGAT-1	singlet	2	-346.9314652	-356.3231712	-167.2303091	EMD1
GCTACCTCAAGCTGTT-1	singlet	2	-580.9074931	-480.160909	-222.7333494	EMD1
GCTACCTCAGACATCT-1	singlet	0	-910.4471421	-2566.815086	-2418.563199	EMD1
GCTACCTCATAATGAG-1	singlet	2	-964.1003122	-754.6207075	-374.0558401	EMD1
GCTACCTGTGGCTGAA-1	singlet	0	-636.0250408	-1955.888697	-1809.157285	EMD1
GCTACCTGTTTGGGTT-1	singlet	2	-542.730725	-495.4546653	-211.158255	EMD1
GCTACCTTCACACGAT-1	singlet	2	-536.3514519	-445.2240753	-195.9835327	EMD1
GCTACCTTCCAACTGA-1	singlet	2	-1707.029199	-1503.905445	-573.3596592	EMD1
GCTACCTTCGCCTCTA-1	singlet	2	-1029.090167	-953.7442631	-386.7812019	EMD1
GCTACCTTCTAACACG-1	singlet	2	-421.4912527	-363.5523442	-140.0529863	EMD1
GCTACCTTCTGCATGA-1	singlet	1	-6224.519876	-1984.03476	-4898.565975	EMD1
GCTCAAAAGCCGATCC-1	singlet	2	-809.8937289	-730.319553	-388.2333464	EMD1
GCTCAAAAGGCACTCC-1	singlet	2	-338.651237	-234.2633142	-141.409669	EMD1
GCTCAAACAATCTCGA-1	singlet	2	-805.2442649	-712.3735818	-285.2216327	EMD1
GCTCAAACAGCCGTTG-1	singlet	0	-1074.568259	-5230.288717	-4525.354034	EMD1
GCTCAAATCGCATGAT-1	singlet	0	-392.6852546	-770.2968098	-798.5322748	EMD1
GCTGAATAGACCAAAT-1	singlet	2	-298.1685715	-255.0133976	-120.6628672	EMD1
GCTGAATAGCGTTCAT-1	singlet	2	-3565.862075	-2891.930256	-1137.579912	EMD1
GCTGAATAGCTTTGTG-1	singlet	2	-436.5764461	-291.7856806	-159.5780604	EMD1
GCTGAATCAGCGTACC-1	singlet	1	-2105.055257	-728.0034846	-1693.008828	EMD1
GCTGAATCAGGATTCT-1	singlet	2	-887.6706121	-853.0480255	-378.1159704	EMD1
GCTGAATCATCACAGT-1	singlet	2	-332.0074569	-263.0151397	-134.0278648	EMD1
GCTGAATCATTCTGTT-1	singlet	2	-507.1723671	-426.8564954	-210.6623147	EMD1
GCTGAATGTACTCCCT-1	unassigned	0	-3.422959552	-4.414371679	-5.117750697	EMD1
GCTGAATGTGTGGACA-1	singlet	2	-546.6281718	-411.9612933	-195.5253044	EMD1
GCTGCAGAGAGCTGAC-1	singlet	2	-1928.46694	-1608.368073	-710.0221174	EMD1
GCTGCAGAGGATGTTA-1	singlet	2	-1001.832793	-750.8668544	-350.0407121	EMD1
GCTGCAGAGGGATCAC-1	singlet	1	-458.0791843	-162.4845877	-386.4833632	EMD1
GCTGCAGAGGTCTACT-1	singlet	2	-1296.993921	-1151.405296	-503.5323732	EMD1

GCTGCAGAGTGCGTCC-1	singlet	2	-1798.20254	-1432.856338	-561.5388491	EMD1
GCTGCAGCACCGCTGA-1	singlet	2	-458.7967047	-400.3291081	-174.3748198	EMD1
GCTGCAGCAGAACTAA-1	singlet	0	-599.4332452	-1624.028066	-1659.451486	EMD1
GCTGCAGTCATGAGTC-1	singlet	0	-508.2340269	-1486.002918	-1365.784908	EMD1
GCTGCAGTCCCAGCGA-1	singlet	0	-395.0425031	-1534.739922	-1273.001677	EMD1
GCTGCAGTCTTGGAAC-1	singlet	0	-2910.138336	-9803.599696	-9596.333836	EMD1
GCTGGGTAGCTAAATG-1	singlet	0	-99.25324448	-246.0223441	-263.0951946	EMD1
GCTGGGTAGGCTAAAT-1	singlet	2	-1243.725724	-967.7163173	-468.1529458	EMD1
GCTGGGTAGGCTCTAT-1	singlet	2	-597.8751161	-517.1634255	-234.6242492	EMD1
GCTGGGTAGGTAGCAC-1	singlet	0	-59.24202593	-210.285459	-189.6732141	EMD1
GCTGGGTCACAGCATT-1	singlet	2	-587.5417571	-489.9021979	-203.9492231	EMD1
GCTGGGTGTCTCCCTA-1	singlet	0	-736.061259	-2078.980848	-2005.912103	EMD1
GCTGGGTGTTCTTGTT-1	singlet	2	-590.9367641	-497.5213714	-256.8365999	EMD1
GCTGGGTTCAACCTTT-1	singlet	2	-1760.017423	-1514.235749	-597.5813113	EMD1
GCTGGGTTCCTGCCAT-1	singlet	1	-5545.454925	-1802.093183	-4452.044962	EMD1
GCTGGGTTCTGGCTGG-1	singlet	2	-2663.148567	-2368.962645	-877.8400422	EMD1
GCTTCACAGAGGTTAT-1	doublet	0/2	-853.414958	-1181.965867	-997.0498084	EMD1
GCTTCACCACACCTGG-1	unassigned	1	-4.941243214	-4.503527294	-5.299331729	EMD1
GCTTCACCATCCGTGG-1	singlet	0	-240.6796587	-804.7776719	-735.6688424	EMD1
GCTTCACCATGACGTT-1	singlet	2	-360.4957355	-384.4678241	-172.9425297	EMD1
GCTTCACGTAGTGCGA-1	singlet	0	-414.2317574	-1139.934824	-1139.907401	EMD1
GCTTCACTCCAGCAAT-1	singlet	0	-303.6955469	-983.5522894	-1013.822463	EMD1
GCTTGGGAGCATCTTG-1	singlet	2	-380.4990899	-303.9292298	-133.6924134	EMD1
GCTTGGGCACCTGCGA-1	singlet	0	-1714.146812	-6047.870746	-5803.846927	EMD1
GCTTGGGCATACCACA-1	singlet	2	-2036.862565	-1511.799907	-590.9971829	EMD1
GCTTGGGCATGGAAGC-1	singlet	2	-485.3567281	-396.3810494	-184.0835837	EMD1
GCTTGGGCATGTGACT-1	singlet	2	-4446.216366	-3954.891891	-1200.131957	EMD1
GCTTGGGGTAGCTGCC-1	singlet	1	-3931.09353	-1151.006704	-2912.767088	EMD1
GCTTGGGGTTGTGCAT-1	singlet	2	-1023.780683	-958.7880473	-422.5157873	EMD1
GCTTGGGTCGAGGCAA-1	singlet	1	-3480.746691	-641.0238121	-3007.905628	EMD1
GCTTGGGTCTCACGAA-1	singlet	2	-536.4938409	-375.8150349	-165.9621157	EMD1
GCTTTCGCAACTCCCT-1	singlet	1	-389.0844251	-159.7395779	-344.776201	EMD1
GCTTTCGCACAAGTGG-1	singlet	2	-2078.136517	-1621.474947	-704.7900638	EMD1
GCTTTCGCACACTTAG-1	singlet	0	-746.1033351	-2379.877411	-2300.369299	EMD1
GCTTTCGGTCCTCCAT-1	singlet	1	-377.7831569	-103.1944134	-254.1315278	EMD1
GCTTTCGGTCTGCCTT-1	singlet	1	-4534.334404	-1440.752387	-3428.618638	EMD1
GCTTTCGGTGATACTC-1	singlet	2	-4547.49964	-4036.59316	-1200.630164	EMD1
GCTTTCGGTGGTCTGC-1	singlet	2	-459.2478769	-411.5238175	-189.149597	EMD1
GCTTTCGTCCGTGTCT-1	singlet	0	-358.3828532	-814.9158129	-827.0172871	EMD1
GCTTTCGTCTCGTGAA-1	singlet	2	-996.4354846	-1027.880998	-366.8932279	EMD1
GGAACCCAGGCGAACT-1	singlet	2	-1368.728159	-1197.491544	-479.7872791	EMD1
GGAACCCAGTAGAATC-1	singlet	0	-579.7570341	-2261.314372	-1765.86568	EMD1
GGAACCCAGTTGTCAC-1	singlet	2	-563.4153638	-503.7900026	-243.3305345	EMD1
GGAACCCCACTGCACG-1	singlet	1	-784.0819358	-359.2781267	-652.9094168	EMD1
GGAACCCGTAACAGTA-1	singlet	0	-596.2583979	-3644.437812	-3014.897634	EMD1
GGAACCCGTAAGACCG-1	singlet	0	-568.2737521	-1615.078399	-1510.956717	EMD1
GGAACCCGTGCTCGTG-1	singlet	2	-378.1801529	-359.9751314	-143.0760358	EMD1
GGAACCCGTGCTTATG-1	singlet	2	-662.7248764	-518.4094374	-220.3786156	EMD1
GGAACCCTCGGAACTT-1	singlet	1	-1193.376364	-408.7177772	-894.3942399	EMD1
GGAACCCTCTACGGGC-1	singlet	2	-477.4507982	-399.87316	-204.6217327	EMD1
GGAAGTGAGGGCTAAC-1	singlet	2	-1368.242091	-1425.096494	-410.6744136	EMD1
GGAAGTGCACGGTAGA-1	singlet	2	-449.4119065	-412.0164283	-180.8381212	EMD1
GGAAGTGGTAGAATGT-1	singlet	2	-695.4644231	-667.7961386	-241.2752924	EMD1
GGAAGTGGTGTTACTG-1	doublet	0/2	-1813.944683	-2758.958316	-2223.879728	EMD1
GGAAGTGTCAGGAGAC-1	singlet	2	-1119.619415	-1086.692919	-386.286339	EMD1
GGAAGTGTCCCTCAAC-1	singlet	2	-602.8105804	-482.5964507	-205.1994093	EMD1
GGAAGTGTCCGATGCG-1	singlet	2	-1519.989079	-1320.988279	-540.8391669	EMD1
GGAAGTGTCCGTCCTA-1	singlet	0	-361.0402562	-1161.60119	-1042.063076	EMD1
GGAATCTAGATCGCCC-1	singlet	2	-1785.934693	-1541.801505	-580.5111783	EMD1
GGAATCTAGCTACTGT-1	singlet	1	-3289.360467	-1176.769553	-2631.030892	EMD1
GGAATCTAGTAGGAAG-1	singlet	2	-500.2068157	-434.7372428	-196.0201446	EMD1
GGAATCTAGTGATAGT-1	singlet	0	-1958.817946	-7000.44403	-7089.539743	EMD1
GGAATCTTCGCCACTT-1	singlet	0	-57.81344236	-130.9764761	-133.4097561	EMD1
GGAATCTTCTACGCAA-1	singlet	2	-491.3708328	-415.2884184	-202.2536705	EMD1
GGAATGGAGACGATAT-1	singlet	2	-475.5196537	-435.8575236	-192.2586286	EMD1
GGAATGGCAACACACT-1	singlet	2	-528.9135402	-484.6678871	-231.3354245	EMD1
GGAATGGCAATCGAAA-1	singlet	2	-507.7305909	-393.4469072	-215.0519175	EMD1
GGAATGGCAATTCACG-1	singlet	2	-1801.719731	-1560.26124	-682.9562807	EMD1
GGAATGGCACTAGTAC-1	singlet	2	-750.3185779	-663.5900974	-303.917604	EMD1
GGAATGGCATAATGCC-1	singlet	2	-1248.498276	-996.2522399	-449.8320994	EMD1
GGAATGGGTAACGGTG-1	singlet	2	-740.2569509	-569.6040007	-298.3106568	EMD1
GGAATGGTCGCCTCTA-1	singlet	0	-31.29406895	-86.0445653	-110.4298461	EMD1
GGACGTCAGACCCTTA-1	singlet	2	-1777.19055	-1378.495829	-510.4808931	EMD1
GGACGTCGTTCTGACA-1	singlet	2	-415.8031006	-348.3732274	-125.8523375	EMD1
GGACGTCGTTTATGCG-1	singlet	2	-367.8826961	-367.6153677	-179.4977751	EMD1
GGACGTCGTTTCCAAG-1	singlet	1	-1264.041396	-440.1782522	-1069.124699	EMD1
GGACGTCTCAAACGAA-1	singlet	1	-128.69697	-58.32125204	-133.3737497	EMD1
GGACGTCTCAGGTGTT-1	singlet	2	-3363.749502	-2921.566755	-1102.864106	EMD1
GGACGTCTCCTTCTTC-1	singlet	0	-616.3621312	-2049.061626	-1899.959967	EMD1
GGACGTCTCTCGCTCA-1	singlet	1	-6.484587722	-2.925920842	-10.20313848	EMD1
GGAGAACAGATCCAAA-1	singlet	2	-2458.945397	-1930.185379	-890.3656226	EMD1
GGAGAACAGCTTACGT-1	singlet	1	-4663.206971	-1030.330293	-3813.251106	EMD1
GGAGAACCAAAGTATG-1	singlet	2	-5765.496295	-4488.878662	-2090.808435	EMD1
GGAGAACCAACCTAAC-1	singlet	1	-1287.38569	-396.5265663	-1120.895024	EMD1
GGAGAACCACAGACGA-1	singlet	0	-422.7931051	-1381.051138	-1168.441041	EMD1
GGAGAACCACTAGTAC-1	singlet	2	-1119.822305	-1089.546405	-343.0757544	EMD1
GGAGAACGTGCTGCAC-1	singlet	2	-1319.385648	-1142.713133	-501.6481009	EMD1
GGAGAACGTGTTCGTA-1	singlet	2	-1048.230436	-870.1184402	-410.0814279	EMD1
GGAGAACGTTAGCTAC-1	singlet	2	-472.8389599	-369.973635	-165.0997576	EMD1
GGAGATGAGGAACGCT-1	singlet	0	-287.1692977	-878.5242839	-1143.147582	EMD1
GGAGATGAGTCTGTAC-1	singlet	2	-461.4910687	-462.0794484	-167.1877528	EMD1
GGAGATGCAACAGCTT-1	singlet	2	-4774.572897	-3906.104523	-1576.729882	EMD1
GGAGATGCAATCCTTT-1	singlet	0	-310.9131954	-1117.425724	-1022.848721	EMD1
GGAGATGGTACCTGTA-1	singlet	1	-526.6604613	-203.0417007	-564.3659061	EMD1
GGAGATGGTAGACGTG-1	singlet	2	-251.1284416	-211.4479561	-87.19347855	EMD1
GGAGATGGTCCGGACT-1	singlet	1	-5352.523991	-1859.879679	-4389.740698	EMD1
GGAGATGGTCGTATTG-1	singlet	1	-1477.365354	-424.426317	-1063.669587	EMD1
GGAGATGGTGGTAATA-1	singlet	2	-386.6920568	-328.47929	-145.0049905	EMD1
GGAGATGTCACAGTGT-1	singlet	1	-1767.370678	-608.4706839	-1417.79241	EMD1
GGAGATGTCAGATGCT-1	singlet	0	-228.9963492	-667.91744	-747.1312008	EMD1
GGAGATGTCATTCGTT-1	singlet	2	-3877.269687	-2962.681621	-1223.77519	EMD1
GGAGATGTCCCTCTTT-1	singlet	0	-153.3264131	-360.8447324	-386.3453417	EMD1
GGAGATGTCTTAGCTT-1	doublet	0/2	-1044.683757	-1882.680201	-1630.780589	EMD1
GGAGCAAAGCCGATTT-1	singlet	0	-250.4496797	-699.8010746	-653.1968139	EMD1
GGAGCAAAGCTACAAA-1	singlet	2	-433.8827516	-375.3337687	-170.44825	EMD1
GGAGCAAAGGGCAACT-1	singlet	1	-2205.621615	-778.27946	-1772.955575	EMD1
GGAGCAAAGTGGTTGG-1	unassigned	1	-3.985394512	-1.9329566	-5.457995763	EMD1
GGAGCAACAAACTAGA-1	singlet	0	-543.1732875	-1940.583823	-1733.794467	EMD1
GGAGCAACAATAAGGT-1	singlet	0	-18.13805668	-36.94497559	-239.8679693	EMD1
GGAGCAACACAGTCGC-1	singlet	2	-6239.861573	-4870.780171	-1956.624117	EMD1

GGAGCAACAGCACAGA-1	singlet	2	-1583.286151	-1177.88271	-536.6742679	EMD1
GGAGCAAGTAGTCGTT-1	singlet	0	-404.4764493	-1176.092271	-1075.215422	EMD1
GGAGCAAGTCCAGCCA-1	singlet	2	-1920.123737	-1597.320983	-576.8572331	EMD1
GGAGCAAGTGGAACCA-1	singlet	0	-662.1016778	-2251.201925	-2255.062193	EMD1
GGAGCAAGTGGCTGAA-1	singlet	1	-4740.306678	-1590.140461	-3626.220949	EMD1
GGAGCAATCCGATAGT-1	singlet	0	-550.4618488	-1566.046202	-1521.780574	EMD1
GGAGCAATCCTCACTG-1	singlet	0	-898.306841	-2527.731796	-2610.863209	EMD1
GGAGCAATCGCTACAA-1	singlet	0	-594.9351959	-1574.42876	-1634.600601	EMD1
GGAGCAATCGCTAGCG-1	singlet	0	-534.8535556	-1965.583313	-1879.336214	EMD1
GGAGGATAGGGTTAAT-1	singlet	2	-373.0029278	-304.3263439	-159.6815455	EMD1
GGAGGATAGTCAGCCC-1	singlet	0	-942.3251564	-3065.837079	-3297.971409	EMD1
GGAGGATAGTGGCGAT-1	singlet	2	-921.58938	-717.0256963	-347.1309883	EMD1
GGAGGATCAAAGCAAT-1	singlet	1	-2238.854248	-724.1285484	-1897.670399	EMD1
GGAGGATCATACTGTG-1	singlet	2	-502.2537788	-456.6484063	-210.1799503	EMD1
GGAGGATCATGATGCT-1	singlet	1	-124.7529434	-37.82044686	-71.94735519	EMD1
GGAGGATGTACCTTCC-1	singlet	2	-574.8830663	-475.0929969	-204.5528929	EMD1
GGAGGATGTAGGACCA-1	singlet	1	-1219.635746	-476.7979084	-1046.19321	EMD1
GGAGGATGTTGGTGTT-1	singlet	2	-428.2201276	-388.5790161	-198.28402	EMD1
GGAGGTAAGGTTACAA-1	singlet	2	-438.9306364	-349.6512809	-192.8715138	EMD1
GGAGGTAAGTAGGCCA-1	singlet	2	-655.8218517	-570.7303592	-255.8881913	EMD1
GGAGGTAGTCATAGTC-1	singlet	0	-629.6232504	-1686.016917	-1761.152186	EMD1
GGAGGTAGTCCATACA-1	singlet	1	-1865.316255	-641.9058599	-1420.892485	EMD1
GGAGGTAGTTAAACAG-1	singlet	0	-1246.596406	-4594.53936	-4138.806993	EMD1
GGAGGTAGTTCAAACC-1	singlet	2	-299.8009773	-294.3271767	-125.9422554	EMD1
GGAGGTATCATCGCCT-1	singlet	1	-1202.807568	-424.0121232	-1043.998702	EMD1
GGAGGTATCGTCAAAC-1	singlet	1	-224.8993634	-98.30461098	-207.1134613	EMD1
GGAGGTATCGTTGCCT-1	singlet	0	-627.8047719	-2052.610351	-2157.064764	EMD1
GGAGGTATCTACCCAC-1	singlet	0	-875.1259982	-2566.43907	-2376.037847	EMD1
GGATCTAAGACATCAA-1	singlet	2	-481.8230369	-417.3400114	-178.4180414	EMD1
GGATCTAAGAGTGAAG-1	singlet	2	-539.5947899	-427.8881299	-185.0745399	EMD1
GGATCTAAGCCTCAGC-1	singlet	0	-828.4000112	-2165.901759	-2166.997179	EMD1
GGATCTACATCCTGTC-1	singlet	2	-420.9681738	-398.5835609	-197.3669047	EMD1
GGATCTAGTATGGGAC-1	singlet	1	-3193.013198	-961.9483174	-2649.707174	EMD1
GGATCTAGTGAGGCAT-1	singlet	2	-283.9426062	-331.6710424	-160.720391	EMD1
GGATCTAGTGTATTGC-1	singlet	2	-334.4048398	-268.8093171	-119.3396851	EMD1
GGATCTATCGTGGAAG-1	singlet	2	-317.6956751	-297.592094	-140.5281114	EMD1
GGATGTTAGAAGGCTC-1	singlet	1	-2142.412853	-778.9281075	-1686.560861	EMD1
GGATGTTAGGTCCCTG-1	singlet	2	-256.3689797	-223.3130255	-94.62572184	EMD1
GGATGTTCAATCTCGA-1	singlet	2	-602.9205875	-513.6337745	-253.5907818	EMD1
GGATGTTGTAACCAGG-1	singlet	1	-403.8274278	-120.9562407	-285.8229252	EMD1
GGATGTTGTGCAATAA-1	singlet	0	-425.3089528	-1393.215798	-1298.215691	EMD1
GGATGTTGTGTTAACC-1	singlet	2	-408.8065009	-325.5258321	-137.4648963	EMD1
GGATGTTGTTCTTCAT-1	singlet	0	-511.1865172	-1271.668554	-1319.759471	EMD1
GGATGTTTCAAGCTTG-1	singlet	2	-1512.999031	-1314.983859	-502.8589289	EMD1
GGATGTTTCAGCGCAC-1	singlet	0	-973.2821703	-3513.284925	-2937.284277	EMD1
GGATGTTTCCTCTAGC-1	singlet	2	-492.018192	-426.8680461	-216.9379705	EMD1
GGCACGTAGGCAATGC-1	singlet	2	-718.7088428	-592.6378522	-304.5934111	EMD1
GGCACGTAGTGATCGG-1	singlet	2	-388.3265853	-338.6628669	-149.6363015	EMD1
GGCACGTAGTGTACAA-1	singlet	0	-30.74267407	-75.75423497	-58.90019795	EMD1
GGCACGTAGTTTCAGC-1	singlet	2	-1946.263217	-1609.129515	-619.9054093	EMD1
GGCACGTCACTGCGTG-1	singlet	2	-308.7719078	-315.5755447	-149.9590866	EMD1
GGCACGTCAGCTTCGG-1	singlet	1	-88.06290441	-27.14403007	-65.0727115	EMD1
GGCACGTCATACCATG-1	singlet	1	-2017.433932	-504.1761678	-1647.5784	EMD1
GGCACGTCATGATGCT-1	singlet	2	-437.054895	-373.4370283	-195.2160256	EMD1
GGCACGTGTCAAGGCA-1	singlet	2	-2475.729795	-2014.471052	-903.887001	EMD1
GGCACGTGTGGACTGA-1	singlet	0	-687.4268812	-2178.994527	-2222.504098	EMD1
GGCACGTGTTTGCCGG-1	singlet	1	-1567.611128	-538.6103185	-1269.021242	EMD1
GGCAGTCAGACTGGGT-1	singlet	1	-3131.428576	-1006.032357	-2470.152765	EMD1
GGCAGTCAGAGCGACT-1	singlet	2	-219.3121622	-201.2168445	-81.03162041	EMD1
GGCAGTCAGCGCAATG-1	singlet	0	-186.9706227	-468.1696485	-557.5269713	EMD1
GGCAGTCCACAACGTT-1	singlet	1	-3698.173359	-1143.185475	-2749.035003	EMD1
GGCAGTCCACCACTGG-1	singlet	0	-606.1142966	-1854.640387	-1817.298243	EMD1
GGCAGTCCATCTAGAC-1	singlet	0	-1265.134335	-3876.344833	-3555.892178	EMD1
GGCAGTCGTACGAGTG-1	singlet	0	-206.3234027	-689.3516114	-656.262273	EMD1
GGCAGTCGTAGTTAGA-1	singlet	0	-439.2130989	-1051.56241	-1003.327746	EMD1
GGCAGTCGTTCAATCG-1	singlet	2	-462.8839945	-331.2099451	-170.4735437	EMD1
GGCAGTCTCGACACTA-1	singlet	1	-713.0935788	-290.7833892	-570.516037	EMD1
GGCAGTCTCGAGTCCG-1	doublet	45293	-920.2148721	-491.3014224	-545.5612914	EMD1
GGCAGTCTCTTCCGTG-1	singlet	0	-361.6004268	-968.8300771	-812.5117541	EMD1
GGCGTCAAGCTAAGTA-1	singlet	2	-2202.479747	-1744.284591	-772.8803882	EMD1
GGCGTCACAGCGAACA-1	singlet	2	-2708.898109	-2078.444871	-1001.208602	EMD1
GGCGTCACAGGAAGTC-1	singlet	2	-377.226247	-287.3733019	-147.8967261	EMD1
GGCGTCATCACCGACG-1	singlet	1	-671.0079995	-312.4088131	-584.8089069	EMD1
GGCGTCATCGTAGAGG-1	singlet	2	-482.1410632	-417.790161	-208.6029736	EMD1
GGCGTCATCTACCACC-1	singlet	2	-69.37410188	-77.78637106	-17.85424542	EMD1
GGCGTCATCTCATTGT-1	singlet	2	-296.2882208	-276.1344517	-136.2143192	EMD1
GGCGTCATCTTGAGCA-1	singlet	2	-1590.963015	-1362.916433	-584.872853	EMD1
GGCTGTGAGAGCAAGA-1	singlet	1	-92.90498988	-47.55576947	-93.67647791	EMD1
GGCTGTGAGTCACGAG-1	singlet	1	-1704.980963	-533.1349929	-1240.548733	EMD1
GGCTGTGCACTTGAGT-1	singlet	0	-268.9866429	-888.7237764	-795.5494442	EMD1
GGCTGTGCAGAACTAA-1	singlet	0	-175.7296447	-426.7173208	-471.0539634	EMD1
GGCTGTGCATCTCCCA-1	singlet	1	-2750.452861	-721.4743519	-2191.362838	EMD1
GGCTGTGGTAGTTCCA-1	singlet	2	-450.7953102	-422.0729953	-168.5516368	EMD1
GGCTGTGGTCCACAGC-1	singlet	1	-1673.464157	-483.5286195	-1377.818367	EMD1
GGCTGTGGTTAGAAAC-1	singlet	1	-2375.216937	-775.2531423	-1892.937119	EMD1
GGCTGTGTCAACCTTT-1	singlet	2	-633.3395488	-486.8312031	-217.362264	EMD1
GGCTGTGTCATTGGTG-1	singlet	1	-2529.696569	-908.9355023	-1991.288085	EMD1
GGCTGTGTCGTCAGAT-1	singlet	0	-650.5274137	-1919.580447	-1684.16062	EMD1
GGCTGTGTCTGCGATA-1	singlet	1	-426.3913578	-185.1625079	-440.7410316	EMD1
GGCTTGGAGACTCTTG-1	singlet	2	-275.9875466	-294.6972035	-125.4821095	EMD1
GGCTTGGAGCCGTAAG-1	singlet	0	-69.70158632	-252.3335675	-254.3265175	EMD1
GGCTTGGAGGGCAAGG-1	singlet	0	-573.7605328	-1724.869283	-1795.567023	EMD1
GGCTTGGCATCCGAGC-1	singlet	2	-553.7938097	-464.2500719	-214.3613787	EMD1
GGCTTGGCATGATAGA-1	singlet	1	-1412.888095	-476.4406032	-1008.823114	EMD1
GGCTTGGCATTGACCA-1	singlet	2	-347.9587675	-265.0179328	-115.2740018	EMD1
GGCTTGGGTATCATGC-1	singlet	2	-471.5883272	-393.0238933	-186.1668831	EMD1
GGCTTGGGTCTGCGCA-1	singlet	0	-101.3057571	-341.9932922	-375.176	EMD1
GGCTTGGGTTCGGTAT-1	singlet	2	-578.8185912	-466.4066973	-238.2666108	EMD1
GGCTTGGTCTGAGAGG-1	singlet	2	-457.1530905	-344.3698961	-151.2864529	EMD1
GGCTTTCAGAAACTAC-1	singlet	2	-219.0390839	-162.5247026	-53.26360563	EMD1
GGCTTTCAGACCATGG-1	singlet	2	-471.5569924	-370.5262201	-171.8245716	EMD1
GGCTTTCCAGAGCGTA-1	singlet	2	-3638.302968	-3152.929882	-1198.08746	EMD1
GGCTTTCGTACAAAGT-1	singlet	0	-840.9082001	-2987.767073	-3018.772061	EMD1
GGCTTTCGTAGACACG-1	singlet	2	-376.594939	-311.554079	-137.450028	EMD1
GGCTTTCGTATAATGG-1	singlet	1	-1002.914539	-398.2068975	-746.0578868	EMD1
GGCTTTCGTCGCGTCA-1	singlet	2	-503.9592447	-375.1636676	-186.3119086	EMD1
GGCTTTCGTCGGCTAC-1	singlet	2	-368.0318201	-296.7447129	-149.5061313	EMD1
GGCTTTCGTGGTTTAC-1	singlet	1	-234.1071094	-97.82580051	-184.4573445	EMD1
GGCTTTCTCAGAGCGA-1	singlet	0	-1134.653864	-3636.696074	-3753.839291	EMD1
GGCTTTCTCCATATGG-1	singlet	1	-52.64597567	-15.94376792	-42.86106231	EMD1

GGGAAGTAGTGTGTTC-1	singlet	2	-486.2199382	-468.1003424	-185.7980053	EMD1
GGGAAGTAGTTTAGGA-1	singlet	2	-1852.611884	-1387.952878	-600.8664374	EMD1
GGGAAGTGTGGGAGAG-1	singlet	0	-649.1375713	-2020.842542	-1971.805929	EMD1
GGGAAGTGTTCGGTAT-1	singlet	2	-482.4246134	-421.356299	-206.9781314	EMD1
GGGAAGTTCACACCGG-1	singlet	0	-18.13882736	-80.05842231	-47.78162403	EMD1
GGGACAAAGAGAAGGT-1	singlet	2	-205.5980847	-204.8588142	-82.10614365	EMD1
GGGACAAAGATGTAGT-1	singlet	0	-1224.803336	-5131.012285	-4567.285279	EMD1
GGGACAACACCTAAAC-1	singlet	1	-3372.35013	-961.3253134	-2636.50828	EMD1
GGGACAACACGACGTC-1	singlet	2	-391.996254	-354.6898356	-150.3831965	EMD1
GGGACAAGTAGAGCTG-1	singlet	2	-408.6686801	-360.1626622	-179.5240747	EMD1
GGGACAAGTTGCATAC-1	singlet	2	-966.7081802	-896.6371126	-385.6206411	EMD1
GGGACAATCATGCATG-1	singlet	2	-945.0708662	-855.166586	-344.5692723	EMD1
GGGACAATCTGCGTCT-1	singlet	2	-1637.06863	-1297.515501	-561.9272541	EMD1
GGGACAATCTTGCAGA-1	singlet	0	-194.5503869	-626.8123086	-672.8297492	EMD1
GGGACCTAGCATCAAA-1	singlet	0	-217.6874953	-586.1699721	-596.0231376	EMD1
GGGACCTCAGAGGACT-1	singlet	0	-258.7982458	-781.0475737	-745.6281218	EMD1
GGGACCTGTAGCTGCC-1	singlet	2	-884.6836158	-739.6095627	-311.0547409	EMD1
GGGACCTGTCTCCCTA-1	singlet	0	-251.8409681	-630.1567392	-688.4983538	EMD1
GGGACCTTCCTGTACC-1	singlet	1	-628.2952117	-347.8959838	-593.4236222	EMD1
GGGACCTTCGAATGCT-1	singlet	2	-437.9644717	-312.6172545	-169.0159821	EMD1
GGGACCTTCTGGTTGA-1	singlet	1	-2802.906857	-849.4436231	-2104.632965	EMD1
GGGACTCAGACGACTG-1	singlet	2	-296.6323824	-322.3815101	-116.2926261	EMD1
GGGACTCAGCTGCCTG-1	singlet	2	-453.8541558	-427.768684	-173.9355147	EMD1
GGGACTCAGGCCTAGA-1	singlet	1	-240.0974956	-94.94153896	-184.2297344	EMD1
GGGACTCCAACAACAA-1	singlet	0	-168.7693545	-467.8611087	-421.4846966	EMD1
GGGACTCCAAGTCATC-1	singlet	2	-513.0513081	-460.5537018	-248.705338	EMD1
GGGACTCCACGACTAT-1	singlet	0	-345.7149942	-941.7914767	-1064.372622	EMD1
GGGACTCCATGGGTCC-1	singlet	2	-653.0244522	-595.0223763	-305.8377625	EMD1
GGGACTCGTACTGTTG-1	singlet	0	-469.0965035	-1588.896105	-1440.570689	EMD1
GGGACTCGTTGGGCCT-1	singlet	2	-430.4160243	-317.5996956	-185.0422609	EMD1
GGGACTCTCAACTCTT-1	singlet	2	-732.8964409	-693.1085152	-261.4568437	EMD1
GGGACTCTCATTACGG-1	singlet	2	-300.1533828	-281.9528211	-136.7662871	EMD1
GGGACTCTCCGGCAGT-1	singlet	0	-336.8190693	-983.1953951	-898.6212487	EMD1
GGGAGATAGACTACCT-1	singlet	2	-555.9843662	-436.4966484	-177.5326439	EMD1
GGGAGATAGCGTCTGC-1	singlet	0	-137.6433488	-363.5847527	-419.3788412	EMD1
GGGAGATAGCTGTCCG-1	singlet	0	-3.245467642	-8.304831742	-15.25719386	EMD1
GGGAGATAGTAGCTCT-1	singlet	2	-833.2442403	-617.7776837	-280.9729542	EMD1
GGGAGATCAACTGCTA-1	singlet	2	-491.6922836	-408.3454682	-184.2339483	EMD1
GGGAGATCACTCTCGT-1	singlet	0	-392.7036127	-1765.296902	-1516.797267	EMD1
GGGAGATCATATCTGG-1	singlet	1	-1839.598586	-647.5619921	-1520.10468	EMD1
GGGAGATCATATGCGT-1	singlet	0	-37.20843837	-122.210212	-127.5142286	EMD1
GGGAGATGTACAACGG-1	singlet	2	-415.7209649	-378.1223678	-150.9188437	EMD1
GGGAGATTCACGAGGA-1	singlet	2	-608.7932402	-473.7802522	-238.6902954	EMD1
GGGAGATTCAGCAATC-1	singlet	0	-77.52221549	-269.8179599	-257.9647169	EMD1
GGGAGATTCCATCAGA-1	singlet	0	-182.8476253	-436.2125587	-431.415548	EMD1
GGGAGATTCGAGTGGA-1	singlet	2	-409.7907352	-384.7292122	-114.3191223	EMD1
GGGAGTAAGTTCATCG-1	singlet	2	-417.2326256	-339.9451475	-156.8232365	EMD1
GGGAGTACAAACGTGG-1	singlet	2	-736.8637694	-604.6080344	-269.1778665	EMD1
GGGAGTACAATTCACG-1	singlet	1	-412.056904	-185.2701956	-349.2748707	EMD1
GGGAGTACATGGTACT-1	singlet	2	-1011.635287	-884.9425179	-383.5197427	EMD1
GGGAGTAGTCTCAGAT-1	singlet	0	-153.6004871	-507.2886269	-531.9045909	EMD1
GGGAGTAGTTATTCCT-1	singlet	0	-687.4493334	-2111.295868	-2030.333377	EMD1
GGGAGTAGTTTCTATC-1	singlet	2	-771.4997643	-663.2498098	-326.77051	EMD1
GGGAGTATCATGAAAG-1	singlet	0	-409.2153257	-912.0606442	-857.354552	EMD1
GGGATCCAGACCATGG-1	singlet	2	-817.0216412	-645.4787314	-276.16246	EMD1
GGGATCCAGAGCGACT-1	singlet	1	-2758.751464	-992.0891363	-1997.216208	EMD1
GGGATCCAGATTACCC-1	singlet	1	-151.5671727	-68.8264563	-123.6006189	EMD1
GGGATCCCAAAGCTCT-1	singlet	0	-97.89676494	-178.88473	-188.2014322	EMD1
GGGATCCGTTAAGGGC-1	singlet	2	-231.7755578	-192.6624936	-100.0311728	EMD1
GGGATCCTCAGGCGAA-1	singlet	2	-670.9614781	-502.6456045	-236.9383688	EMD1
GGGATCCTCCACCTGT-1	singlet	2	-2072.111541	-1724.167039	-729.5719369	EMD1
GGGATGACAAGACGAC-1	singlet	0	-84.57559547	-277.998013	-273.9666115	EMD1
GGGATGACACCGGTCA-1	singlet	1	-3650.652415	-1254.210159	-2985.517699	EMD1
GGGATGACAGACACAG-1	singlet	2	-611.1416542	-574.8664159	-279.4929583	EMD1
GGGATGATCATGTCTT-1	singlet	2	-294.9700069	-345.5235449	-157.2371602	EMD1
GGGATGATCGGACTTA-1	singlet	0	-10.83460429	-17.27282672	-187.0714213	EMD1
GGGATGATCGGCAGTC-1	singlet	0	-18.04221928	-41.88817599	-304.4825419	EMD1
GGGATGATCGGTCGAC-1	singlet	0	-1655.009851	-6292.227291	-6306.616945	EMD1
GGGATGATCTCCTACG-1	unassigned	0	-2.365802916	-3.575323305	-6.570940068	EMD1
GGGCCATAGATCCTAC-1	singlet	2	-504.3700085	-380.3815281	-221.3009467	EMD1
GGGCCATAGATGGTCG-1	singlet	1	-2226.652399	-649.8745673	-1984.059642	EMD1
GGGCCATAGTCCGCCA-1	singlet	2	-587.5252387	-544.4685273	-259.4125764	EMD1
GGGCCATCAAACCGGA-1	singlet	1	-2366.125449	-850.7348619	-1890.822368	EMD1
GGGCCATCACAGTCCG-1	singlet	2	-1753.276423	-1959.71975	-507.3200072	EMD1
GGGCCATCAGTGTGCC-1	singlet	2	-903.4971441	-846.3050196	-372.1059251	EMD1
GGGCCATGTACCCGAC-1	singlet	0	-32.48463438	-90.22285107	-105.2791074	EMD1
GGGCCATGTAGCTGAG-1	singlet	1	-1725.830303	-441.3740229	-1229.11332	EMD1
GGGCCATGTCATGACT-1	singlet	2	-492.68572	-405.981407	-208.7527514	EMD1
GGGCCATGTTGCTCGG-1	singlet	2	-260.9278959	-234.0502854	-100.8790333	EMD1
GGGCCATTCGAAATCC-1	singlet	2	-992.0681139	-806.0410098	-400.0462837	EMD1
GGGCCATTCGCGTGCA-1	singlet	0	-546.8981638	-1787.490574	-1773.588674	EMD1
GGGCCATTCGTCTACC-1	singlet	2	-1555.826487	-1316.260175	-537.5643914	EMD1
GGGCCATTCTGACAGT-1	singlet	2	-498.0225999	-448.722792	-179.2003042	EMD1
GGGCGTTAGGAATGTT-1	singlet	2	-126.4782246	-99.07992654	-45.95764292	EMD1
GGGCGTTAGGGACACT-1	singlet	0	-456.240249	-1864.449076	-1709.391537	EMD1
GGGCGTTAGGTTCTAC-1	singlet	0	-700.1776369	-2101.81447	-2077.605302	EMD1
GGGCGTTCAACCACGC-1	singlet	1	-453.4834619	-164.7434757	-376.7095831	EMD1
GGGCGTTCAATCAAGA-1	singlet	0	-1365.814949	-6246.591573	-5295.157877	EMD1
GGGCGTTCACGACGCT-1	doublet	1/0	-2341.142975	-1259.209653	-2339.323834	EMD1
GGGCGTTCACGGTAGA-1	singlet	2	-1712.562329	-1311.857799	-516.6467133	EMD1
GGGCGTTCAGGCCCTA-1	singlet	2	-2052.728625	-1708.621182	-751.0365363	EMD1
GGGCGTTGTACCATAC-1	singlet	0	-765.9000984	-2695.173251	-2407.017373	EMD1
GGGCGTTGTCCGAAAG-1	singlet	2	-291.9874937	-255.7050274	-102.2429828	EMD1
GGGCGTTGTTAGAGAT-1	singlet	1	-2033.191399	-563.9711164	-1499.259664	EMD1
GGGCGTTGTTGGACCC-1	singlet	2	-443.3791679	-371.5357902	-180.778164	EMD1
GGGCGTTGTTTACCAG-1	singlet	1	-3858.722559	-1317.347217	-3194.680807	EMD1
GGGCGTTTCCAGCCTT-1	singlet	0	-1239.873725	-4635.667799	-4532.278702	EMD1
GGGCGTTTCCGTTTCG-1	singlet	1	-928.5101385	-360.0343985	-699.2621636	EMD1
GGGCGTTTCGGACCAC-1	singlet	2	-1683.875016	-1322.164801	-533.859006	EMD1
GGGCTACAGATGCAGC-1	singlet	0	-174.6485776	-454.7378115	-413.6014966	EMD1
GGGCTACAGTGCAGCA-1	singlet	1	-2009.92996	-700.7940046	-1679.133344	EMD1
GGGCTACAGTGGTTAA-1	singlet	2	-342.0680622	-247.746363	-144.7547818	EMD1
GGGCTACAGTTAACAG-1	singlet	2	-542.0000404	-506.7649818	-218.9401228	EMD1
GGGCTACCAAGCTGTT-1	singlet	2	-1458.38077	-1021.490575	-473.5648552	EMD1
GGGCTACCACGTGAGA-1	singlet	0	-2194.589511	-10280.83592	-8788.03803	EMD1
GGGCTACGTTAGAGTA-1	singlet	2	-357.7306727	-289.2110927	-127.2959417	EMD1
GGGCTACTCAGACTGT-1	singlet	2	-1106.882078	-940.3359621	-421.7435086	EMD1
GGGCTACTCCTGCTAC-1	singlet	2	-1362.265442	-1071.660021	-461.1684725	EMD1
GGGCTACTCCTGGGAC-1	singlet	2	-509.9182962	-478.6482779	-228.67257	EMD1
GGGCTACTCTGCAGCG-1	singlet	2	-129.1810874	-137.3671532	-57.28959082	EMD1

GGGCTCAAGAGTTCGG-1	singlet	1	-663.8152763	-218.4986859	-500.5631768	EMD1
GGGCTCAAGCGAACTG-1	singlet	2	-307.6320859	-272.2970693	-127.7923447	EMD1
GGGCTCAAGCGACATG-1	singlet	2	-590.5973287	-504.4876711	-212.5903443	EMD1
GGGCTCAAGGCTGAAC-1	singlet	0	-547.1735931	-2038.544698	-1901.059126	EMD1
GGGCTCACACAGAGCA-1	singlet	2	-196.2716325	-145.724953	-60.34766241	EMD1
GGGCTCACATCTAGAC-1	singlet	1	-296.4123136	-111.8814195	-214.918256	EMD1
GGGCTCAGTAGAAACT-1	singlet	0	-171.3711028	-443.0712529	-536.0927963	EMD1
GGGCTCATCTGCGATA-1	singlet	0	-316.3190816	-884.6851874	-880.3732829	EMD1
GGGTAGAAGACCCTTA-1	singlet	0	-362.2014354	-904.3762372	-912.3753366	EMD1
GGGTAGAAGACTAAGT-1	doublet	45293	-292.7575086	-120.5066507	-179.6957081	EMD1
GGGTAGAAGAGTCAGC-1	singlet	2	-260.0193762	-252.1324825	-102.0126701	EMD1
GGGTAGAAGCTCAGAG-1	singlet	2	-576.3384159	-522.3236938	-216.5519884	EMD1
GGGTAGAAGGAGAGTA-1	singlet	1	-764.4068466	-270.9936989	-568.2968309	EMD1
GGGTAGAAGGCATCTT-1	singlet	2	-301.2801793	-278.6325779	-133.9647248	EMD1
GGGTAGAAGTCACAGG-1	singlet	2	-301.1177894	-307.6347775	-139.3623494	EMD1
GGGTAGACAAATGGTA-1	singlet	2	-1527.391441	-1170.440791	-546.6909557	EMD1
GGGTAGAGTACGATCT-1	singlet	2	-455.0124492	-366.7856733	-152.9073938	EMD1
GGGTAGAGTGTCCATA-1	singlet	1	-4799.273293	-1110.597169	-3900.779781	EMD1
GGGTAGAGTTTGGCTA-1	singlet	0	-904.7618254	-3715.086422	-3517.56383	EMD1
GGGTAGATCCGAAGGA-1	singlet	2	-333.0502464	-285.0925972	-131.6483289	EMD1
GGGTATTAGGATACCG-1	singlet	0	-314.5433463	-1237.385288	-1044.236885	EMD1
GGGTATTAGGATCACG-1	singlet	2	-470.9532276	-427.7804324	-209.1744953	EMD1
GGGTATTCAACACAAA-1	singlet	1	-1713.153472	-575.5734544	-1340.207689	EMD1
GGGTATTCAATGAAAC-1	singlet	1	-258.3118713	-71.85737387	-193.0524794	EMD1
GGGTATTCACGACGTC-1	singlet	2	-357.4349385	-331.3234308	-167.6096139	EMD1
GGGTATTCATCGGAAG-1	singlet	1	-2042.958768	-627.9071552	-1647.97757	EMD1
GGGTATTGTAGCGCTC-1	singlet	0	-70.29140024	-220.4456206	-169.3186819	EMD1
GGGTATTGTTCGATTG-1	singlet	0	-148.1991661	-520.238557	-527.9008567	EMD1
GGGTATTTCATGAGTC-1	singlet	2	-646.7506504	-607.7944829	-285.6809778	EMD1
GGGTATTTCGAGTCTA-1	singlet	2	-374.6058809	-298.253116	-126.7827592	EMD1
GGGTATTTCGCCGAGT-1	singlet	2	-428.6424361	-282.6284402	-159.3607871	EMD1
GGGTATTTCGGATTAC-1	singlet	2	-391.0176119	-298.8790657	-163.1969109	EMD1
GGGTATTTCTATCACT-1	singlet	1	-689.8810021	-289.5703637	-679.6786267	EMD1
GGGTATTTCTCCGCAT-1	singlet	2	-522.2651153	-383.7167587	-165.4319785	EMD1
GGGTATTTCTCTAAGG-1	singlet	1	-979.1109708	-364.2758332	-876.957496	EMD1
GGGTCACAGACAGTCG-1	singlet	2	-1161.058996	-1050.118683	-407.599558	EMD1
GGGTCACAGACGAGCT-1	singlet	0	-592.2250903	-1817.526438	-1619.437236	EMD1
GGGTCACAGCAACTTC-1	singlet	2	-472.8024838	-504.7925667	-194.5299673	EMD1
GGGTCACAGGTGCATG-1	singlet	2	-397.8884718	-346.1188682	-171.7458066	EMD1
GGGTCACAGTTTCGGT-1	singlet	2	-336.7129387	-300.8336472	-159.532556	EMD1
GGGTCACCAACGTATC-1	singlet	2	-295.4300666	-292.4745816	-143.6100653	EMD1
GGGTCACCAGTGGTGA-1	singlet	2	-241.9971443	-192.5243483	-99.97923914	EMD1
GGGTCACGTTGCTCCT-1	singlet	2	-2015.29229	-1816.296018	-784.840697	EMD1
GGGTCTGAGAATAACC-1	singlet	0	-350.432624	-991.4874239	-881.317095	EMD1
GGGTCTGAGCCTGTGC-1	singlet	2	-398.6785727	-380.7659701	-178.6135142	EMD1
GGGTCTGAGGAGCAAA-1	singlet	0	-490.0097601	-2237.731813	-1756.943796	EMD1
GGGTCTGAGGCCTTGC-1	singlet	1	-6750.407989	-2011.181085	-5869.811629	EMD1
GGGTCTGAGTGGATAT-1	singlet	1	-395.0786858	-131.6321619	-373.6752399	EMD1
GGGTCTGCACCGTCGA-1	singlet	2	-514.2921571	-449.9389851	-211.9699598	EMD1
GGGTCTGCACTGGACC-1	singlet	2	-281.8235414	-251.7821636	-113.1220987	EMD1
GGGTCTGCAGATTCGT-1	singlet	2	-453.422554	-370.3349003	-168.9985778	EMD1
GGGTCTGCAGGCTATT-1	singlet	0	-349.9022662	-968.0049592	-878.0983537	EMD1
GGGTCTGGTACATACC-1	singlet	2	-460.177713	-416.2766984	-171.0427508	EMD1
GGGTCTGGTTGTACGT-1	singlet	2	-325.5597958	-289.2168817	-149.6636981	EMD1
GGGTCTGTCAAGCGTT-1	singlet	2	-540.4147336	-410.3966527	-203.0421263	EMD1
GGGTCTGTCTAGGCAT-1	singlet	1	-1938.265399	-702.0930749	-1473.133516	EMD1
GGGTGAAAGAAACACT-1	singlet	0	-208.5645698	-492.328741	-548.0073234	EMD1
GGGTGAACAATGAAAC-1	singlet	2	-692.6571543	-591.7526813	-279.7622893	EMD1
GGGTGAACACCGTGAC-1	singlet	2	-655.1205591	-536.1748204	-226.1117045	EMD1
GGGTGAACAGATCCAT-1	singlet	1	-4195.387891	-1415.192412	-3108.189785	EMD1
GGGTGAAGTAACGGTG-1	singlet	2	-573.6834713	-462.6336286	-198.7667383	EMD1
GGGTGAAGTAGTCCTA-1	singlet	0	-226.6635816	-569.17343	-557.4676625	EMD1
GGGTGAATCGTCCTTG-1	singlet	2	-2331.149556	-1934.838165	-775.0268088	EMD1
GGGTGAATCTAAACGC-1	singlet	0	-442.8160971	-1350.365181	-1271.665122	EMD1
GGGTGAATCTAGTACG-1	singlet	2	-421.6748691	-278.243599	-147.2408989	EMD1
GGGTGTCCAAATGCTC-1	singlet	2	-1536.416184	-1193.65919	-573.0308666	EMD1
GGGTGTCCAAGTGGGT-1	singlet	1	-5871.60495	-1180.525648	-4924.70681	EMD1
GGGTGTCCAGTAGTTC-1	doublet	2/0	-243.3200063	-243.3758131	-130.9513524	EMD1
GGGTGTCCAGTTGGTT-1	singlet	2	-338.4737653	-313.9275168	-126.4945697	EMD1
GGGTGTCCATTGTACG-1	singlet	2	-279.0251777	-225.3455669	-115.6474702	EMD1
GGGTGTCGTCACCGCA-1	singlet	0	-70.5347012	-277.4818636	-228.262235	EMD1
GGGTGTCGTTACACTG-1	singlet	0	-75.52108058	-297.5037119	-437.6791223	EMD1
GGGTGTCGTTTGTTGG-1	singlet	2	-268.9649764	-232.3198121	-113.8877301	EMD1
GGGTGTCTCACCTCGT-1	singlet	0	-498.6884085	-2503.886776	-2260.213872	EMD1
GGGTGTCTCCTTACCG-1	singlet	2	-1537.798429	-1324.394602	-647.7553239	EMD1
GGGTGTCTCGCCAGAC-1	singlet	0	-935.6393214	-2516.230111	-2550.894894	EMD1
GGGTGTCTCGCTGACG-1	singlet	2	-1533.389347	-1295.031269	-551.0476876	EMD1
GGGTTATAGACAGTCG-1	singlet	2	-327.0692704	-269.0693279	-140.4343253	EMD1
GGGTTATAGATCACCT-1	singlet	0	-1669.672807	-4908.840053	-4811.16249	EMD1
GGGTTATAGCGGACAT-1	singlet	2	-456.4489456	-350.7939095	-190.9176699	EMD1
GGGTTATAGGTCACCT-1	singlet	0	-60.95496301	-167.6491771	-181.842683	EMD1
GGGTTATCACATTGTG-1	singlet	2	-481.4685723	-447.4728678	-217.3326622	EMD1
GGGTTATCAGCTGTCG-1	singlet	1	-846.6465104	-211.6820861	-702.3746089	EMD1
GGGTTATCAGGGATAC-1	singlet	0	-21.99063526	-67.06295938	-62.03517668	EMD1
GGGTTATGTATACCCA-1	singlet	2	-1785.966085	-1334.133955	-604.1195921	EMD1
GGGTTATGTCCAGCGT-1	singlet	1	-1441.574265	-422.0089414	-1177.795043	EMD1
GGGTTATGTCCGTTTC-1	singlet	2	-363.1405577	-259.3787057	-110.3969755	EMD1
GGGTTATGTCTCGCGA-1	singlet	2	-920.2455305	-732.3333284	-335.6097175	EMD1
GGGTTATGTTAATCGC-1	singlet	2	-1102.54537	-468.6821003	-189.5600113	EMD1
GGGTTATGTTCTCTAT-1	singlet	1	-451.4877833	-152.901227	-361.6688415	EMD1
GGGTTATTCACTCACC-1	singlet	1	-3224.732196	-814.2139125	-2528.563269	EMD1
GGGTTATTCATGGTAC-1	singlet	2	-548.9457386	-453.8609653	-181.5986448	EMD1
GGGTTATTCGTTCCCA-1	singlet	0	-1227.938191	-3633.688128	-3906.941109	EMD1
GGGTTTAAGATTAGCA-1	singlet	2	-295.6759402	-224.6036229	-102.9215295	EMD1
GGGTTTACAGTATGAA-1	singlet	2	-451.986382	-401.9376295	-190.8922055	EMD1
GGGTTTAGTAACATCC-1	singlet	1	-2292.227185	-901.496594	-1697.632609	EMD1
GGGTTTAGTAGCTGTT-1	singlet	0	-1768.270444	-4796.163724	-5087.591618	EMD1
GGGTTTAGTCGCCTAG-1	singlet	1	-1424.128177	-471.2165436	-1202.358863	EMD1
GGGTTTAGTGGCTTGC-1	singlet	1	-684.4866349	-276.2982753	-658.9199971	EMD1
GGGTTTATCATTTACC-1	singlet	2	-296.567045	-290.265727	-105.2803568	EMD1
GGGTTTATCTGGCCAG-1	singlet	0	-153.172697	-545.5498633	-523.1372235	EMD1
GGTAACTAGAGAGTGA-1	singlet	2	-942.1254636	-815.1772253	-374.9075537	EMD1
GGTAACTAGTCCCAGC-1	singlet	0	-405.1369801	-1114.389026	-1073.445853	EMD1
GGTAACTCAAGCGCTC-1	singlet	2	-315.6950921	-287.678025	-145.5107453	EMD1
GGTAACTCACCTCTGT-1	singlet	1	-2240.796549	-634.7893108	-1744.843239	EMD1
GGTAACTGTAATCAAG-1	singlet	0	-463.4081392	-2007.962029	-1950.409632	EMD1
GGTAACTGTCGCGTCA-1	singlet	2	-476.1471952	-414.5411057	-202.7016578	EMD1
GGTAACTGTGACACAG-1	singlet	2	-401.5645178	-415.3243301	-195.0590192	EMD1
GGTAACTTCCGTCAAA-1	singlet	1	-1365.354914	-443.8458172	-1095.102268	EMD1
GGTAATCAGCCGAATG-1	singlet	1	-2610.679313	-931.827467	-2076.043965	EMD1

GGTAATCAGCGCCTCA-1	singlet	2	-301.1076995	-256.0032103	-129.9070446	EMD1
GGTAATCAGTTCACTG-1	singlet	2	-595.9493934	-556.4073389	-245.4799844	EMD1
GGTAATCCAATCTAGC-1	singlet	1	-600.15882	-228.9749908	-669.2265292	EMD1
GGTAATCCACAGCGCT-1	singlet	0	-319.0390213	-984.7791173	-904.5931772	EMD1
GGTAATCGTGGTACAG-1	doublet	0/2	-1232.866883	-2110.166599	-1585.308148	EMD1
GGTAATCTCCGATCGG-1	singlet	1	-2208.501613	-666.4854265	-1764.284329	EMD1
GGTAATCTCGGAATGG-1	singlet	2	-942.8242955	-714.3139464	-339.0294118	EMD1
GGTAATCTCTGAGCAT-1	singlet	2	-601.8250746	-510.5970602	-179.5325883	EMD1
GGTAATCTCTGAGGCC-1	singlet	1	-1409.572419	-438.4276457	-1161.991709	EMD1
GGTAGAGAGACCGCCT-1	singlet	0	-803.6272482	-2485.431256	-2503.55852	EMD1
GGTAGAGAGACGGTCA-1	singlet	1	-483.2039408	-200.7109238	-511.4260115	EMD1
GGTAGAGAGATGCTTC-1	singlet	0	-323.3050001	-1066.46381	-990.5748563	EMD1
GGTAGAGAGTTCCAGT-1	singlet	0	-521.1504641	-1825.503683	-1740.716936	EMD1
GGTAGAGCAATTCACG-1	singlet	2	-494.7952281	-357.6108927	-186.9673004	EMD1
GGTAGAGGTAGACGGT-1	singlet	1	-1575.227079	-547.2864957	-1272.89217	EMD1
GGTAGAGGTATCACGT-1	singlet	1	-665.7386075	-252.1604639	-516.3218008	EMD1
GGTAGAGTCATTCCTA-1	singlet	2	-425.9651589	-332.078658	-152.0497483	EMD1
GGTAGAGTCCATCTGC-1	singlet	2	-488.472613	-504.4473805	-235.3771829	EMD1
GGTCACGAGGGATGTC-1	singlet	2	-362.0307376	-313.3709145	-131.2820018	EMD1
GGTCACGAGTGTAGTA-1	singlet	2	-554.8249355	-453.8876301	-214.565299	EMD1
GGTCACGGTCGAATTC-1	singlet	0	-701.5218531	-2038.102966	-2025.541787	EMD1
GGTCACGGTGAGTCAG-1	singlet	2	-1465.428546	-1237.987459	-565.3987109	EMD1
GGTCACGGTGTGTGGA-1	singlet	2	-634.7098228	-491.4328481	-219.1840211	EMD1
GGTCACGTCCAAGCTA-1	singlet	0	-1125.326094	-3230.552068	-3371.92105	EMD1
GGTCACGTCGTTCTCG-1	singlet	2	-456.0933709	-387.7374886	-185.4504079	EMD1
GGTCACGTCTTCACGC-1	singlet	1	-1276.099024	-500.1060601	-1015.368601	EMD1
GGTCTGGAGACCAACG-1	singlet	0	-37.93617999	-78.87167617	-74.27927942	EMD1
GGTCTGGAGCCGCTTG-1	singlet	2	-352.9636071	-367.4678434	-176.1401413	EMD1
GGTCTGGAGTCGCCCA-1	singlet	2	-157.1440689	-110.5620804	-61.38983775	EMD1
GGTCTGGCACAATCTG-1	singlet	2	-455.7443428	-353.2617237	-145.0151461	EMD1
GGTCTGGCACTTTAGG-1	singlet	2	-446.2555143	-371.038684	-174.3661339	EMD1
GGTCTGGGTCCCTGAG-1	singlet	2	-125.8979204	-95.94145166	-43.13236009	EMD1
GGTCTGGGTGGCTGAA-1	singlet	2	-442.3749496	-347.6373281	-162.326914	EMD1
GGTCTGGGTTACAGCT-1	singlet	2	-498.8963191	-383.3529933	-196.8752036	EMD1
GGTCTGGGTTCTATCT-1	singlet	0	-846.9553818	-2776.69109	-2472.959893	EMD1
GGTCTGGTCCATTTAC-1	singlet	2	-508.4902913	-437.6428487	-186.2163726	EMD1
GGTGAAGAGCGAGAAA-1	singlet	2	-152.6501982	-120.8092819	-56.20856702	EMD1
GGTGAAGCACTGATTG-1	singlet	2	-458.9064596	-386.2954986	-188.1238123	EMD1
GGTGAAGCAGCTTCCT-1	singlet	0	-334.6506856	-1247.353628	-1014.481384	EMD1
GGTGAAGGTCTTCCGT-1	singlet	2	-959.0195279	-555.7551451	-276.174447	EMD1
GGTGAAGTCATGCATG-1	singlet	2	-396.4930427	-344.2973436	-163.7969513	EMD1
GGTGATTAGAACCCGA-1	singlet	0	-539.9515038	-1382.707098	-1376.729757	EMD1
GGTGATTAGAGCATTA-1	singlet	1	-3198.891531	-933.9503008	-2495.233707	EMD1
GGTGATTAGATGATTG-1	singlet	0	-444.2899087	-1153.965189	-1097.213862	EMD1
GGTGATTAGGGACACT-1	singlet	1	-673.5014637	-285.1946032	-533.903519	EMD1
GGTGATTAGGGTTAGC-1	singlet	0	-2413.726973	-9007.545898	-8912.84279	EMD1
GGTGATTAGTATTCCG-1	singlet	0	-251.2850919	-666.6530439	-872.0621568	EMD1
GGTGATTAGTTTGGCT-1	singlet	0	-589.78704	-1534.406281	-1574.800696	EMD1
GGTGATTCAACCGTGC-1	singlet	2	-833.3446676	-640.4132249	-316.5415856	EMD1
GGTGATTCAACTTCTT-1	singlet	2	-2914.453675	-2386.698365	-830.2228025	EMD1
GGTGATTCAATAGTGA-1	singlet	2	-124.0502076	-86.85899872	-49.5933499	EMD1
GGTGATTCACAGAGCA-1	singlet	1	-5467.632176	-1628.11236	-4771.551962	EMD1
GGTGATTCAGTCAGCC-1	singlet	2	-354.8172232	-277.6347677	-114.6277811	EMD1
GGTGATTGTCCACAGC-1	singlet	1	-1013.738771	-420.7950779	-1009.118372	EMD1
GGTGATTTCATGGGAG-1	singlet	2	-970.4329749	-902.1036261	-354.0573714	EMD1
GGTGATTTCGGTAACT-1	singlet	0	-537.3614897	-1765.636083	-1622.549239	EMD1
GGTGATTTCTAACGGT-1	singlet	0	-270.8446864	-693.1391609	-804.5860027	EMD1
GGTGGCTAGAAGCTCG-1	singlet	0	-372.7769741	-1339.167566	-1293.295333	EMD1
GGTGGCTAGATTCGAA-1	singlet	2	-562.3805334	-489.9608195	-207.9822197	EMD1
GGTGGCTAGTGAACAT-1	singlet	2	-351.9804632	-315.9290104	-141.6040386	EMD1
GGTGGCTAGTGTTGAA-1	singlet	1	-987.0210552	-397.9014963	-798.5851857	EMD1
GGTGGCTCAGCACACC-1	singlet	2	-400.496143	-406.0393669	-153.7100694	EMD1
GGTGGCTGTACATACC-1	singlet	1	-1880.284512	-532.4312437	-1269.158636	EMD1
GGTGGCTGTCTCTCAC-1	singlet	2	-547.0616314	-496.8150829	-195.4540148	EMD1
GGTGGCTGTGTCTAAC-1	singlet	0	-798.7915428	-2263.872613	-2317.426315	EMD1
GGTGGCTGTTTGGCTA-1	singlet	0	-615.709148	-2369.66078	-1955.618232	EMD1
GGTGGCTTCACGGGCT-1	singlet	1	-808.5795194	-309.0156833	-631.8472522	EMD1
GGTGGCTTCAGGTAAA-1	singlet	2	-363.2867796	-306.2311814	-147.4589361	EMD1
GGTGGCTTCTGCTTAT-1	singlet	2	-369.8309665	-384.7384711	-171.1658514	EMD1
GGTGTCGAGCTAATCC-1	singlet	0	-472.8694234	-1461.91092	-1495.273882	EMD1
GGTGTCGCAATTTCCT-1	singlet	2	-1058.6622	-1016.208998	-404.2260426	EMD1
GGTGTCGCACAAGCAG-1	singlet	2	-353.6602049	-272.5405343	-122.8935565	EMD1
GGTGTCGGTAGTATAG-1	singlet	0	-153.4054927	-446.6674982	-395.2172472	EMD1
GGTGTCGGTATCTCTT-1	singlet	1	-2072.512714	-690.5103766	-1720.686697	EMD1
GGTGTCGGTGCTAGCC-1	singlet	2	-2837.095646	-2338.640584	-827.6304132	EMD1
GGTGTCGTCGTTATCT-1	singlet	0	-1.168216769	-5.787416193	-5.694239339	EMD1
GGTGTTAAGCCTCATA-1	singlet	2	-1305.323863	-1219.517979	-463.6324402	EMD1
GGTGTTAAGGAGATAG-1	singlet	2	-356.1705325	-278.6318823	-147.9157881	EMD1
GGTGTTAAGGCCATAG-1	singlet	2	-378.0896827	-331.0192802	-178.9356741	EMD1
GGTGTTAAGGCCGCTT-1	singlet	1	-1961.658849	-1064.99936	-1908.303268	EMD1
GGTGTTAAGGCGTTAG-1	singlet	0	-318.3113601	-1240.623054	-1115.159441	EMD1
GGTGTTAAGGGACACT-1	singlet	2	-384.2679724	-330.8367037	-180.1379678	EMD1
GGTGTTACAACTTCTT-1	singlet	2	-1905.879401	-1669.380058	-515.9737464	EMD1
GGTGTTACACCTTCGT-1	singlet	2	-2079.402171	-1843.725752	-708.5796215	EMD1
GGTGTTAGTAGCTGTT-1	singlet	1	-3948.402026	-1196.891065	-4353.670339	EMD1
GGTGTTAGTATGGAGC-1	singlet	2	-553.1718406	-457.7673221	-205.4588451	EMD1
GGTGTTAGTCAAATCC-1	singlet	2	-550.030899	-498.3983496	-218.0376179	EMD1
GGTGTTAGTCGCGGTT-1	singlet	2	-3043.974971	-2309.234168	-925.975318	EMD1
GGTGTTATCCAGTTCC-1	singlet	0	-203.8789467	-734.9128629	-745.9192382	EMD1
GGTTAACAGACGCATG-1	singlet	2	-597.0903225	-536.4682917	-274.9168463	EMD1
GGTTAACAGCCATTCA-1	singlet	0	-556.0673013	-1981.351585	-1787.555644	EMD1
GGTTAACAGTTGTAAG-1	singlet	2	-559.5618008	-403.8914804	-189.7566048	EMD1
GGTTAACCAAGGTCAG-1	singlet	2	-10074.31015	-8908.477693	-4051.500116	EMD1
GGTTAACCACGGTAGA-1	singlet	0	-918.4756868	-2450.42597	-2536.18183	EMD1
GGTTAACCACTCACTC-1	singlet	1	-126.3613239	-46.8106471	-103.9161253	EMD1
GGTTAACGTGAGTGAC-1	singlet	0	-16.42543428	-27.17377506	-231.8526726	EMD1
GGTTAACGTGCCCAGT-1	singlet	0	-1863.448254	-5562.177171	-5420.108276	EMD1
GGTTAACGTGCCGTAC-1	singlet	2	-233.3939948	-190.9814058	-71.1853914	EMD1
GGTTAACGTGTGTACT-1	singlet	1	-4215.898603	-1145.594964	-3265.9117	EMD1
GGTTAACGTTCTATCT-1	unassigned	0/1	-13.52144985	-19.77646122	-17.08282332	EMD1
GGTTAACTCCATTTCA-1	singlet	0	-1097.494635	-2880.593018	-2875.836698	EMD1
GGTTAACTCGGTAGAG-1	singlet	2	-341.7139277	-341.515057	-173.0362796	EMD1
GGTTAACTCTCCGAGG-1	singlet	1	-895.6167648	-281.4478191	-697.1937787	EMD1
GGTTCTCAGAATCTAG-1	singlet	2	-506.5070064	-369.5458305	-233.9519936	EMD1
GGTTCTCAGGCACAAC-1	doublet	0/2	-672.5630037	-1096.871985	-938.9202419	EMD1
GGTTCTCAGTAGCAAT-1	singlet	2	-162.0386386	-139.4763574	-59.85278659	EMD1
GGTTCTCCAATGGGTG-1	singlet	0	-857.794862	-2673.77887	-2695.137138	EMD1
GGTTCTCGTACAGAAT-1	singlet	2	-429.2065306	-372.8101264	-203.8656582	EMD1
GGTTCTCGTATGTCCA-1	singlet	2	-674.7466028	-469.736118	-234.9371906	EMD1
GGTTCTCGTCCCAAAT-1	singlet	0	-2194.360926	-6652.635166	-6811.10217	EMD1

GGTTCTCTCGTAGTGT-1	singlet	0	-2025.185026	-7337.846995	-7409.606227	EMD1
GGTTCTCTCTGCGGCA-1	singlet	0	-268.9961338	-767.9871232	-783.1640391	EMD1
GGTTGTAAGAACGTGC-1	singlet	0	-462.8856659	-1318.35861	-1227.609835	EMD1
GGTTGTAAGGAACGAA-1	singlet	2	-1663.806704	-1479.728804	-557.5384649	EMD1
GGTTGTACAACACGAG-1	singlet	0	-363.5265178	-1019.139431	-964.8624613	EMD1
GGTTGTACACGGTGCT-1	singlet	1	-6186.726014	-1676.802939	-4753.825868	EMD1
GGTTGTACACTGGAAG-1	singlet	1	-3203.885865	-735.8069794	-2655.365559	EMD1
GGTTGTAGTCAGTCGC-1	singlet	1	-232.9621586	-123.0146484	-226.133897	EMD1
GGTTGTATCTGAATGC-1	singlet	2	-2658.914523	-2168.907804	-924.1679379	EMD1
GGTTGTATCTTCGTGC-1	singlet	0	-10.97244889	-39.20812037	-22.6677146	EMD1
GTAACACCACGACGAA-1	singlet	0	-29.26586937	-86.87905976	-117.9232381	EMD1
GTAACACCACGTAGTT-1	singlet	2	-728.7138414	-579.4956081	-275.880108	EMD1
GTAACACCAGGCAATG-1	singlet	1	-2057.847066	-727.7212459	-1626.222942	EMD1
GTAACACCATCCGATA-1	singlet	1	-1407.875222	-484.7199346	-1179.781883	EMD1
GTAACACGTAGCTTGT-1	doublet	0/2	-120.7723576	-245.1997041	-176.3904051	EMD1
GTAACACGTATCGTGT-1	singlet	0	-989.5984596	-3042.204109	-2976.318995	EMD1
GTAACACGTGTTTCTT-1	singlet	0	-270.1088	-1140.209569	-883.3712817	EMD1
GTAACACTCCGTCCTA-1	singlet	1	-705.4312493	-266.9111288	-751.5890403	EMD1
GTAACCAAGCCGGAAT-1	singlet	0	-1143.398581	-4398.006521	-4129.083174	EMD1
GTAACCAAGGCCACCT-1	singlet	2	-606.1845748	-522.6763428	-261.5191032	EMD1
GTAACCACATGACGTT-1	singlet	2	-845.6332738	-664.7764797	-303.4850189	EMD1
GTAACCAGTCTTCATT-1	unassigned	45323	-6.678342288	-5.932606928	-3.855932047	EMD1
GTAACCAGTGGGTATG-1	singlet	1	-4205.048416	-1436.051008	-3355.434257	EMD1
GTAACCAGTTCTAAGC-1	singlet	0	-651.9063179	-1744.692802	-1988.257274	EMD1
GTAACCAGTTCTCGTC-1	singlet	2	-593.1902604	-461.7793662	-206.4459618	EMD1
GTAACCATCACGGGAA-1	singlet	0	-29.04653041	-76.00514102	-76.64155493	EMD1
GTAACCATCCCATAGA-1	singlet	1	-3737.14226	-988.3785484	-2539.442992	EMD1
GTAACCATCGTGCAGC-1	singlet	1	-1053.440543	-303.7591817	-835.9193659	EMD1
GTAACCATCTGGCCAG-1	singlet	2	-986.7883928	-933.5974817	-341.5000587	EMD1
GTAAGTCAGACGTCCC-1	singlet	2	-339.7017149	-289.4548309	-138.3879578	EMD1
GTAAGTCAGACTCGAG-1	doublet	2/0	-156.3368597	-208.5303564	-152.4848184	EMD1
GTAAGTCAGGTTTACC-1	singlet	2	-200.1211118	-184.2574126	-82.25989039	EMD1
GTAAGTCCAAACCGGA-1	singlet	2	-332.1190069	-293.9323191	-139.7802505	EMD1
GTAAGTCCAAAGCTCT-1	singlet	2	-581.4264745	-521.984529	-261.3233226	EMD1
GTAAGTCCAGCTTCGG-1	singlet	0	-704.9215728	-2289.181437	-2211.486023	EMD1
GTAAGTCCATCCTTGC-1	singlet	2	-627.7341769	-548.1501452	-279.9086269	EMD1
GTAAGTCCATGTTCGA-1	singlet	2	-344.0399853	-343.081071	-148.6457026	EMD1
GTAAGTCGTAGTTCCA-1	singlet	2	-398.0579511	-369.2612629	-173.198535	EMD1
GTAAGTCGTCTCCTGT-1	singlet	2	-246.1809715	-246.2944869	-130.6114676	EMD1
GTAAGTCTCAACACGT-1	singlet	1	-1714.412129	-461.3651955	-1281.93904	EMD1
GTAAGTCTCATTGCGA-1	singlet	2	-82.39964472	-88.05570702	-33.22212008	EMD1
GTAAGTCTCGCGTTTC-1	singlet	2	-589.761234	-488.632091	-231.5483704	EMD1
GTAAGTCTCTGCGGCA-1	singlet	1	-1486.100456	-360.4950964	-1192.234776	EMD1
GTAATCGAGGTAAGTT-1	singlet	2	-318.91644	-316.4400754	-135.0464375	EMD1
GTAATCGAGTCGCGAA-1	singlet	1	-1415.267605	-459.8422995	-1040.582518	EMD1
GTAATCGAGTGCACTT-1	singlet	2	-629.9081077	-511.5482699	-259.2812182	EMD1
GTAATCGGTCGATTCA-1	singlet	1	-3874.936511	-747.5675189	-3518.611061	EMD1
GTAATCGTCGAGTGGA-1	singlet	0	-311.702175	-988.6928163	-811.1230225	EMD1
GTAATCGTCGGTCATA-1	singlet	2	-85.07709262	-94.73478613	-16.57775802	EMD1
GTAATCGTCGGTCGAC-1	singlet	0	-997.5005245	-3004.048122	-3012.82217	EMD1
GTAATCGTCTTCCAGC-1	singlet	0	-454.1641683	-1517.204472	-1364.13568	EMD1
GTAATGCAGAATCCCT-1	singlet	1	-829.2089333	-310.383467	-642.5300298	EMD1
GTAATGCAGATGCGAC-1	singlet	1	-1031.009779	-361.2047306	-849.9053302	EMD1
GTAATGCCACTATCCC-1	singlet	2	-316.8229075	-249.7603047	-100.696982	EMD1
GTAATGCGTTTACGAC-1	singlet	2	-789.5949844	-706.4765489	-305.504237	EMD1
GTAATGCTCACGGGAA-1	singlet	2	-337.0454618	-285.4775295	-139.2049504	EMD1
GTAATGCTCGATAACC-1	singlet	0	-290.8249824	-1149.472792	-1032.12492	EMD1
GTACAACAGAGTAACT-1	singlet	1	-1162.852356	-347.2703747	-963.6071193	EMD1
GTACAACAGTACTGGG-1	singlet	2	-502.1176688	-464.0760713	-196.4783411	EMD1
GTACAACAGTCCCAGC-1	singlet	1	-639.04568	-235.9226416	-560.3414635	EMD1
GTACAACCATACCACA-1	doublet	0/1	-748.920752	-1104.455959	-1104.293769	EMD1
GTACAACGTACTCGAT-1	singlet	0	-645.6319633	-1821.128	-1669.243492	EMD1
GTACAACGTAGGTACG-1	singlet	2	-379.967486	-332.8824211	-159.652506	EMD1
GTACAACTCAAAGGAT-1	singlet	2	-757.2562976	-587.2442881	-316.6448321	EMD1
GTACAACTCCGAACGC-1	singlet	0	-21.66320676	-106.9595664	-100.2279435	EMD1
GTACAACTCGGTAGAG-1	singlet	1	-336.0816585	-108.2423704	-218.1965464	EMD1
GTACAGTAGACGTCGA-1	singlet	2	-564.2434057	-493.5030901	-237.4037767	EMD1
GTACAGTAGCACCTGC-1	singlet	2	-1168.86796	-997.6801205	-465.6187646	EMD1
GTACAGTAGGATGTTA-1	doublet	45323	-1373.525952	-1060.224777	-776.0418294	EMD1
GTACAGTAGGCACCAA-1	singlet	1	-968.7580472	-313.680241	-780.0257396	EMD1
GTACAGTAGGGCTGAT-1	singlet	2	-497.4216409	-477.3433005	-195.9898165	EMD1
GTACAGTAGGTCACAG-1	singlet	0	-99.45265242	-297.4093855	-308.7094592	EMD1
GTACAGTAGTAGAGTT-1	singlet	1	-1010.146692	-332.2455725	-791.1282992	EMD1
GTACAGTCAGACATCT-1	singlet	2	-1483.910126	-1108.256831	-463.0977504	EMD1
GTACAGTGTACAGAGC-1	singlet	0	-725.0391571	-2211.860499	-2204.018499	EMD1
GTACAGTTCATTGTGG-1	singlet	0	-212.1326611	-642.594788	-607.7897621	EMD1
GTACAGTTCGTGCGAC-1	singlet	1	-3070.946723	-1029.857872	-2442.27307	EMD1
GTAGAAAAGAGCATTA-1	singlet	2	-1518.297807	-1202.494155	-519.8843595	EMD1
GTAGAAAAGCTGCCAC-1	singlet	1	-3575.021172	-1394.43796	-2511.645856	EMD1
GTAGAAAAGTATCTGC-1	singlet	2	-309.2451831	-271.8419089	-139.9230821	EMD1
GTAGAAAAGTTCGCAT-1	singlet	0	-173.4271473	-564.4759508	-538.7484389	EMD1
GTAGAAAGTCGAGCAA-1	singlet	1	-3924.463706	-665.4467677	-3299.944218	EMD1
GTAGAAAGTCGTACTA-1	singlet	2	-2381.113228	-1621.808919	-552.2737367	EMD1
GTAGAAAGTTCTTCAT-1	singlet	2	-437.3228615	-470.7004996	-191.1539327	EMD1
GTAGAAATCCGATCGG-1	singlet	0	-99.86357722	-349.8282361	-313.5308648	EMD1
GTAGAGGAGAGATGCC-1	singlet	1	-5925.258013	-1378.205251	-5035.874191	EMD1
GTAGAGGCAAATTAGG-1	singlet	1	-3591.126641	-1211.229432	-2794.005756	EMD1
GTAGAGGCAAGTCATC-1	singlet	2	-1403.45301	-1217.228032	-549.6363725	EMD1
GTAGAGGCACTGTCCT-1	singlet	0	-480.0710851	-1361.110293	-1235.247673	EMD1
GTAGAGGCAGCTGAAG-1	singlet	2	-710.8672663	-593.0215595	-285.3299146	EMD1
GTAGAGGGTTGTCCCT-1	singlet	1	-1736.762221	-589.6674956	-1376.23952	EMD1
GTAGAGGGTTTCGTAG-1	singlet	0	-308.5903607	-1246.4453	-1145.126299	EMD1
GTAGAGGTCACAATGC-1	singlet	2	-1180.994882	-882.8810158	-385.7869264	EMD1
GTAGAGGTCATGGGAG-1	singlet	1	-4828.795531	-1749.035537	-4137.192416	EMD1
GTAGATCAGACTCGAG-1	singlet	1	-6107.521655	-1835.576261	-4710.664066	EMD1
GTAGATCAGCCTATCA-1	singlet	1	-2646.75389	-692.0403019	-1867.354714	EMD1
GTAGATCAGCTTCGTA-1	singlet	1	-630.9550553	-262.1450952	-521.3230587	EMD1
GTAGATCAGGACTGGT-1	singlet	1	-1375.272305	-466.0297709	-1022.483275	EMD1
GTAGATCAGGAGAGTA-1	singlet	2	-360.503526	-340.1624934	-142.6425806	EMD1
GTAGATCAGGCGAAGG-1	singlet	2	-2184.167837	-1774.517702	-793.9415315	EMD1
GTAGATCAGTATCCTG-1	singlet	0	-951.8682237	-2637.314014	-2466.197258	EMD1
GTAGATCCAAGGTCGA-1	singlet	0	-995.9998653	-3568.322667	-3443.233841	EMD1
GTAGATCCAGCCTATA-1	singlet	0	-548.6225838	-1407.084777	-1339.163544	EMD1
GTAGATCCAGGAGGAG-1	singlet	0	-82.2745575	-215.8066307	-238.0477262	EMD1
GTAGATCGTCTCGGGT-1	singlet	2	-1183.004303	-1523.254842	-316.1560151	EMD1
GTAGATCGTTCGGACC-1	doublet	2/0	-450.5623036	-464.7810172	-260.4411744	EMD1
GTAGATCTCGACATCA-1	singlet	2	-437.6339234	-363.8225298	-170.8757769	EMD1
GTAGATCTCTCACGAA-1	singlet	0	-161.3301997	-443.802625	-513.4068441	EMD1
GTAGATCTCTCTTGCG-1	singlet	2	-3988.972933	-3080.556558	-1274.517334	EMD1
GTAGATCTCTTGCAAG-1	singlet	2	-176.6462445	-193.8407298	-92.70164783	EMD1

GTAGCTAAGGTTAAAC-1	singlet	1	-3135.70779	-1211.591344	-2622.334599	EMD1
GTAGCTAAGGTTCACT-1	singlet	0	-411.8071877	-1284.722313	-1130.57067	EMD1
GTAGCTAAGTCACGCC-1	singlet	2	-479.1534972	-423.1361569	-186.8138234	EMD1
GTAGCTAAGTGAGGTC-1	singlet	2	-874.7464825	-899.4720702	-365.3990004	EMD1
GTAGCTACAACACTAC-1	singlet	2	-2595.694228	-1911.671071	-788.5964916	EMD1
GTAGCTACACGTTGGC-1	singlet	2	-456.5602439	-349.0149339	-174.9195178	EMD1
GTAGCTACATAGATCC-1	singlet	1	-609.64362	-190.0928281	-453.0709711	EMD1
GTAGCTACATATCTGG-1	singlet	0	-3294.86622	-8377.493819	-7011.60254	EMD1
GTAGCTAGTCTACGTA-1	singlet	2	-324.8958897	-261.3021367	-137.2773549	EMD1
GTAGCTAGTGCCAAGA-1	singlet	1	-2176.2565	-553.2913698	-1917.81362	EMD1
GTAGCTAGTGTGCCTG-1	singlet	1	-1811.143225	-602.1017635	-1352.032257	EMD1
GTAGCTAGTTCTCTAT-1	singlet	0	-424.641314	-1252.677688	-1141.632799	EMD1
GTAGCTATCAGGAAAT-1	singlet	0	-1385.401927	-4597.610729	-4762.645423	EMD1
GTAGCTATCATCACTT-1	singlet	1	-1102.601601	-377.2086421	-868.522968	EMD1
GTAGCTATCGAATCCA-1	singlet	2	-307.8611978	-276.0107309	-123.8390205	EMD1
GTAGCTATCTGGAAGG-1	singlet	2	-600.5575401	-537.8682614	-255.6843365	EMD1
GTAGGAGAGGTGCTTT-1	singlet	1	-1641.604754	-593.6849274	-1295.525675	EMD1
GTAGGAGAGTGAATAC-1	singlet	2	-327.8315794	-242.6487633	-90.59380997	EMD1
GTAGGAGCACATGACT-1	doublet	45293	-1562.53104	-984.2663192	-1084.056574	EMD1
GTAGGAGCAGAGAATT-1	singlet	2	-437.1025069	-389.3167411	-170.1130784	EMD1
GTAGGAGGTGCAATAA-1	singlet	2	-233.3690071	-260.4145584	-128.5507978	EMD1
GTAGGAGGTGTGTACT-1	singlet	0	-356.9450064	-1171.503937	-1083.921834	EMD1
GTAGGTTCACCAGCGT-1	singlet	2	-270.7138861	-190.49153	-107.775149	EMD1
GTAGGTTCAGACCCGT-1	singlet	0	-673.0134756	-2026.650398	-1961.563199	EMD1
GTAGGTTCAGGCATTT-1	singlet	2	-690.5272838	-571.3122726	-203.9065324	EMD1
GTAGGTTGTTGATGTC-1	singlet	1	-3650.120836	-1336.666906	-3206.193459	EMD1
GTAGGTTTCGTTACCC-1	singlet	1	-255.9538482	-67.66268366	-168.021164	EMD1
GTAGTACAGAAGTCAT-1	singlet	0	-1241.921348	-3220.974018	-3265.529153	EMD1
GTAGTACCAATATCCG-1	singlet	1	-299.39858	-103.618676	-205.9997333	EMD1
GTAGTACCACTGATTG-1	singlet	2	-1878.285611	-1632.202675	-773.4326914	EMD1
GTAGTACGTCTGTCCT-1	singlet	0	-743.9007217	-2505.474547	-2552.213279	EMD1
GTAGTACTCACTGCTC-1	singlet	2	-1891.38338	-1640.483102	-650.8054628	EMD1
GTAGTACTCAGACCTA-1	singlet	2	-2319.993082	-1954.996408	-961.6892079	EMD1
GTATTGGAGCAGGGAG-1	singlet	1	-319.1598242	-110.3840624	-290.3905509	EMD1
GTATTGGAGTTGAAAC-1	unassigned	0/2	-6.389807664	-4.585783441	-6.969019333	EMD1
GTATTGGCACAGACGA-1	singlet	2	-76.08552558	-60.57626249	-31.32830104	EMD1
GTATTGGCACATGTTG-1	singlet	2	-1143.865631	-1034.222449	-459.0576781	EMD1
GTATTGGGTCATAGTC-1	singlet	0	-209.7831894	-479.2420957	-489.609344	EMD1
GTATTGGTCTAACGCA-1	singlet	1	-592.3773506	-233.2086738	-437.5478495	EMD1
GTATTTCAGTGATTCC-1	singlet	2	-540.5647848	-462.6666705	-215.7491808	EMD1
GTATTTCCAACTCGTA-1	singlet	0	-640.1698079	-1993.315416	-1825.61989	EMD1
GTATTTCCAATCGCAT-1	singlet	0	-521.1784506	-1368.032062	-1499.208965	EMD1
GTATTTCCAATTGCTG-1	singlet	1	-308.9414663	-128.8937928	-250.9433164	EMD1
GTATTTCCACTCATAG-1	singlet	2	-1165.391521	-962.1095112	-474.7037029	EMD1
GTATTTCCATAGGTTC-1	singlet	0	-1037.531388	-5366.938582	-4585.731434	EMD1
GTATTTCCATGGAATA-1	singlet	1	-1911.110765	-648.4136091	-1649.759543	EMD1
GTATTTCCATTGACTG-1	singlet	2	-168.7502234	-165.4931068	-78.34649164	EMD1
GTATTTCGTACCCGCA-1	singlet	2	-4748.214172	-3733.660768	-1486.040721	EMD1
GTATTTCGTCTCACAA-1	singlet	2	-2708.306378	-2475.653876	-885.2026612	EMD1
GTATTTCTCGCTGATA-1	singlet	2	-352.800554	-287.9627115	-127.2550405	EMD1
GTATTTCTCTGCATGA-1	singlet	2	-755.2732175	-632.6600053	-257.446379	EMD1
GTCAAACAGGGCCCTT-1	singlet	0	-452.9730667	-1349.000601	-1291.946353	EMD1
GTCAAACCACGGTAGA-1	singlet	2	-397.8491722	-317.3259133	-167.3460955	EMD1
GTCAAACCAGAAATCA-1	singlet	0	-229.0566074	-633.7487193	-807.122751	EMD1
GTCAAACCATCTCATT-1	singlet	2	-3431.964035	-3350.969156	-1033.001715	EMD1
GTCAAACGTATACCCA-1	singlet	0	-180.9090559	-441.0761407	-564.3940896	EMD1
GTCAAACGTATCAGCT-1	singlet	2	-2038.401529	-1740.218929	-632.1481169	EMD1
GTCAAACGTCAACCAT-1	singlet	0	-1270.74857	-4043.183779	-3934.442087	EMD1
GTCAAACTCAAAGAAC-1	singlet	2	-485.2272331	-441.4719453	-201.353067	EMD1
GTCAAACTCGAAACAA-1	singlet	1	-381.7748226	-79.69951542	-250.4176858	EMD1
GTCAAGTAGCCTATCA-1	singlet	1	-3206.451895	-952.5079088	-2624.017011	EMD1
GTCAAGTAGGGCATGT-1	singlet	0	-135.798617	-423.6291162	-441.533104	EMD1
GTCAAGTCAGATTTCG-1	singlet	2	-150.0839527	-122.5763428	-72.06043476	EMD1
GTCAAGTCATGCCGAC-1	singlet	0	-416.562371	-921.180674	-784.680112	EMD1
GTCAAGTGTCATATGC-1	singlet	1	-1087.168982	-310.6078382	-843.1080335	EMD1
GTCAAGTGTCATCTAG-1	singlet	2	-1116.748814	-914.1142011	-484.4168785	EMD1
GTCAAGTGTCTCGCGA-1	singlet	1	-239.3021738	-79.70146421	-185.5641534	EMD1
GTCAAGTGTTCGTTCC-1	singlet	2	-180.4715435	-136.867617	-58.56875277	EMD1
GTCAAGTGTTGTCCCT-1	singlet	0	-166.5545362	-441.5499102	-486.5629108	EMD1
GTCAAGTTCCCAGCGA-1	singlet	0	-470.0953814	-1964.117197	-1736.790888	EMD1
GTCAAGTTCGTAACTG-1	singlet	1	-1517.94643	-474.1512842	-1330.212966	EMD1
GTCACGGAGACCGCCT-1	singlet	0	-134.6979168	-373.7689091	-421.0635211	EMD1
GTCACGGCAAGTCATC-1	singlet	0	-23.26801761	-75.86896269	-77.32440177	EMD1
GTCACGGCATCTCAAG-1	doublet	1/0	-2903.430014	-2280.507857	-3346.107199	EMD1
GTCACGGCATGGTGGA-1	singlet	0	-149.5189785	-369.6094893	-458.9100088	EMD1
GTCACGGGTGGCTGCT-1	singlet	2	-314.1724763	-296.051316	-147.0970017	EMD1
GTCACGGTCTTCACGC-1	singlet	2	-1309.331566	-1119.646398	-520.9366564	EMD1
GTCACTCAGAATCGCG-1	singlet	1	-2183.524151	-620.7859533	-1653.783842	EMD1
GTCACTCAGAGGTCAC-1	singlet	2	-1447.200207	-1189.335437	-566.8414203	EMD1
GTCACTCAGCCATTGT-1	singlet	0	-227.0348563	-837.532224	-787.8156595	EMD1
GTCACTCCAATACCTG-1	singlet	0	-375.1287712	-893.2945698	-888.9339912	EMD1
GTCACTCCACGGATCC-1	singlet	2	-1997.046552	-1474.007768	-725.979101	EMD1
GTCACTCCATGGGATG-1	singlet	1	-4518.252338	-1340.530548	-3585.855976	EMD1
GTCACTCGTAGCTTAC-1	singlet	0	-162.5517111	-416.6128664	-414.2507141	EMD1
GTCACTCTCTAACGGT-1	singlet	0	-550.4216062	-1382.083265	-1485.887493	EMD1
GTCAGCGAGCACTCCG-1	singlet	0	-1587.744846	-4912.470121	-4826.905954	EMD1
GTCAGCGCAAATAGCA-1	singlet	0	-233.0906236	-710.3073816	-705.07636	EMD1
GTCAGCGCACAGGATG-1	singlet	2	-322.418371	-275.843694	-149.7782619	EMD1
GTCAGCGCATAACCCA-1	singlet	0	-1228.506568	-3267.156751	-3453.392118	EMD1
GTCAGCGGTATCGTGT-1	singlet	2	-549.3073922	-533.2764304	-212.2408698	EMD1
GTCAGCGGTCTGTCAA-1	singlet	1	-869.9177963	-323.8671632	-701.8368841	EMD1
GTCAGCGGTGCACAAG-1	singlet	0	-168.9515962	-468.8870516	-538.3978694	EMD1
GTCAGCGGTTCCGCGA-1	singlet	1	-599.3567631	-182.2340319	-444.1103508	EMD1
GTCAGCGGTTCTCGTC-1	singlet	0	-49.63671964	-99.00233974	-88.53489275	EMD1
GTCAGCGGTTTACCTT-1	singlet	1	-1176.954914	-413.9164145	-1075.195338	EMD1
GTCAGCGTCACGATAC-1	singlet	0	-41.36574221	-185.0390272	-145.2325305	EMD1
GTCATCCAGTTGAAAC-1	singlet	1	-4028.292888	-1188.578635	-2934.332253	EMD1
GTCATCCCAAGCGAAC-1	singlet	1	-1274.955983	-443.5166995	-993.7920778	EMD1
GTCATCCCAATACCTG-1	singlet	0	-719.110102	-2591.38705	-2348.22955	EMD1
GTCATCCCACGCGCTA-1	singlet	0	-881.5639215	-2520.701558	-2497.643814	EMD1
GTCATCCGTATCCTCC-1	singlet	2	-1883.715364	-1597.770639	-646.3271456	EMD1
GTCATCCTCAGCGTCG-1	singlet	0	-69.68585199	-309.3507782	-273.8077306	EMD1
GTCATCCTCGACCTAA-1	singlet	0	-456.3376246	-1167.628277	-1212.57312	EMD1
GTCATGAAGATACGAT-1	singlet	2	-614.6278218	-455.650645	-236.8792313	EMD1
GTCATGAAGATAGCTA-1	singlet	2	-1880.160747	-1346.277446	-552.8729557	EMD1
GTCATGAAGTAGTCCT-1	singlet	2	-1378.141436	-1180.532901	-516.1594101	EMD1
GTCATGAAGTCATAGA-1	singlet	0	-167.3407935	-592.5981696	-463.5173194	EMD1
GTCATGACAGGTTCCG-1	doublet	45293	-362.1525191	-196.8396406	-255.8984268	EMD1
GTCATGAGTAGTCTTG-1	singlet	2	-410.7873866	-375.8475275	-177.1982727	EMD1
GTCATGAGTGTATCCA-1	singlet	2	-388.3616496	-321.9292554	-149.0534773	EMD1

GTCATGATCATGGATC-1	singlet	2	-581.3508665	-499.6102165	-220.7392591	EMD1
GTCATGATCCTCTTTC-1	singlet	1	-3362.372292	-1178.553194	-2738.005278	EMD1
GTCATGATCGATTGGT-1	singlet	2	-3194.50843	-2434.932578	-968.1044402	EMD1
GTCATGATCTTAGCTT-1	singlet	1	-4501.623712	-1341.905917	-3495.660815	EMD1
GTCATTTCATCCCACT-1	singlet	0	-2365.087794	-7997.48907	-7018.277399	EMD1
GTCATTTGTCAGGTAG-1	singlet	1	-217.4713992	-80.98820453	-179.6793328	EMD1
GTCATTTTCTATTCGT-1	singlet	0	-1055.329108	-3021.779522	-3092.137558	EMD1
GTCCACTAGACACACG-1	singlet	1	-2189.119476	-430.3205868	-1425.357211	EMD1
GTCCACTAGAGAGCCT-1	singlet	1	-3645.4733	-833.4000775	-2881.339539	EMD1
GTCCACTAGGCATCAG-1	singlet	2	-416.3344938	-464.5040049	-191.9351611	EMD1
GTCCACTAGGCCTTCG-1	singlet	2	-147.2118443	-86.87466785	-48.84496985	EMD1
GTCCACTAGTCCGCCA-1	singlet	2	-118.6588205	-103.841618	-38.68535059	EMD1
GTCCACTAGTCGAATA-1	singlet	0	-1288.32899	-3626.171968	-3951.583508	EMD1
GTCCACTCAAAGGGTC-1	singlet	1	-2447.00462	-879.9917034	-1839.081631	EMD1
GTCCACTCAGCTTTGA-1	singlet	0	-1289.87722	-6734.184888	-5485.456579	EMD1
GTCCACTGTACGCGTC-1	singlet	1	-613.8613683	-178.3426321	-448.0512964	EMD1
GTCCACTGTCCGGTCA-1	singlet	2	-992.4025589	-988.4629193	-317.075798	EMD1
GTCCACTGTTCAACGT-1	singlet	2	-327.3329948	-284.1084995	-141.7151334	EMD1
GTCCACTTCAGTCCGG-1	doublet	0/2	-941.1519795	-1802.182488	-1498.915937	EMD1
GTCCACTTCTACCAGA-1	singlet	2	-329.594452	-254.7602968	-150.032908	EMD1
GTCCCATAGCACCGAA-1	singlet	2	-1095.99106	-774.6309269	-386.6688027	EMD1
GTCCCATAGCATTGTC-1	singlet	2	-617.260392	-591.3511934	-252.0446462	EMD1
GTCCCATAGCCTTCTC-1	singlet	0	-23.16374793	-29.59938618	-249.1299373	EMD1
GTCCCATAGGGCAGTT-1	singlet	1	-4200.738473	-1429.033695	-3606.060288	EMD1
GTCCCATCACAGTGTT-1	singlet	2	-1610.801662	-1225.811623	-573.9170578	EMD1
GTCCCATGTAGGCTGA-1	singlet	0	-152.8474443	-501.4885918	-467.357156	EMD1
GTCCCATGTGGTAACG-1	singlet	0	-605.4144228	-2312.837618	-2339.638123	EMD1
GTCCCATGTTGCGAAG-1	singlet	2	-1984.144744	-1675.994057	-685.2913723	EMD1
GTCCCATTCCGCATAA-1	singlet	2	-228.6343023	-201.9645406	-102.5907009	EMD1
GTCCCATTCGGTAGAG-1	singlet	2	-342.53976	-328.4473653	-151.6390736	EMD1
GTCCCATTCTACGGGC-1	singlet	2	-776.9313066	-574.0390678	-282.6579875	EMD1
GTCCTCAAGCGTTAGG-1	singlet	0	-299.6674017	-1136.866331	-977.2115854	EMD1
GTCCTCAAGTAAATGC-1	singlet	1	-5388.930067	-1947.235225	-4501.217249	EMD1
GTCCTCAAGTAGTCAA-1	singlet	2	-1668.200785	-1961.513632	-459.3185475	EMD1
GTCCTCACAACAACAA-1	singlet	0	-1114.227403	-3192.15486	-3237.473437	EMD1
GTCCTCACACACGCCA-1	singlet	2	-287.5398616	-305.3812925	-123.2271488	EMD1
GTCCTCACACGGCCAT-1	singlet	2	-732.0308835	-666.0007136	-271.4406634	EMD1
GTCCTCACAGTAACGG-1	singlet	1	-3077.680356	-949.0842334	-2392.232351	EMD1
GTCCTCACATGGGTCC-1	singlet	2	-394.4343194	-292.2475586	-154.0458466	EMD1
GTCCTCAGTACGGGAT-1	singlet	2	-403.6828109	-343.0496629	-169.6668715	EMD1
GTCCTCAGTCCTGTTC-1	singlet	2	-853.83719	-734.3406462	-304.4023644	EMD1
GTCCTCAGTCTCTCCA-1	singlet	0	-371.6813953	-1177.979751	-1237.144507	EMD1
GTCCTCAGTGAATGAT-1	singlet	0	-190.0491444	-686.5016347	-617.4412768	EMD1
GTCCTCATCAGCGCGT-1	singlet	2	-380.5152935	-330.4995239	-161.9033801	EMD1
GTCCTCATCCCGTGTT-1	singlet	2	-1603.707789	-1555.671396	-601.7033865	EMD1
GTCCTCATCGATGCTA-1	singlet	2	-435.5680726	-402.5382737	-186.7906961	EMD1
GTCCTCATCTGCTGAA-1	singlet	0	-155.0982015	-747.2972793	-758.9773162	EMD1
GTCGAATAGCGTACAG-1	singlet	2	-224.7755428	-215.8045296	-101.594854	EMD1
GTCGAATAGCTATCTG-1	singlet	1	-644.3429208	-217.6440632	-530.324779	EMD1
GTCGAATAGTTGTACC-1	singlet	1	-1654.625979	-484.6415174	-1215.655835	EMD1
GTCGAATCACTTGAAC-1	singlet	1	-216.6039522	-60.09984277	-179.6011654	EMD1
GTCGAATCATGGAATA-1	singlet	2	-375.3324811	-319.6944888	-146.740243	EMD1
GTCGAATGTGGATCGA-1	singlet	0	-342.0610701	-942.9021058	-874.8002831	EMD1
GTCGAATGTGGCAGAT-1	singlet	0	-144.133559	-455.3307597	-593.0859965	EMD1
GTCGAATTCAGAATAG-1	singlet	0	-391.8603882	-1472.412789	-1405.2081	EMD1
GTCGCGAAGTTGGCGA-1	singlet	2	-193.0746185	-168.3254365	-77.0704249	EMD1
GTCGCGACACCGAATT-1	singlet	2	-217.547585	-152.5507094	-79.82246097	EMD1
GTCGCGACAGCGCGTT-1	singlet	2	-377.4014614	-260.1347932	-132.9853908	EMD1
GTCGCGACAGTGTGGA-1	singlet	0	-662.7130641	-2092.992694	-2015.979545	EMD1
GTCGCGAGTAAGCTCT-1	singlet	0	-436.6674144	-1195.159261	-1228.799359	EMD1
GTCGCGAGTTGCAAGG-1	singlet	1	-86.91842326	-38.39427159	-76.4592409	EMD1
GTCGCGAGTTGTTGAC-1	singlet	1	-3128.21106	-852.8783311	-2362.153924	EMD1
GTCGCGATCAAGCCCG-1	singlet	2	-415.7656976	-407.4752708	-172.3489235	EMD1
GTCGCGATCATACGGT-1	singlet	1	-603.0387684	-229.7793626	-410.5683255	EMD1
GTCGCGATCCGCAAAT-1	singlet	1	-4330.370257	-1198.864146	-3472.892384	EMD1
GTCGCGATCGAGTACT-1	singlet	1	-371.2879415	-88.1425193	-271.8429528	EMD1
GTCGCGATCGGTTAGT-1	singlet	2	-229.5689878	-189.1809389	-98.44288653	EMD1
GTCGTAAAGAGGGTAA-1	singlet	0	-54.17142298	-245.5342919	-238.1483896	EMD1
GTCGTAAAGGCCTGCT-1	singlet	2	-282.1611149	-258.0565268	-96.89620674	EMD1
GTCGTAAAGTAAGGGA-1	singlet	2	-287.2788181	-264.765723	-120.2526781	EMD1
GTCGTAAAGTCGCTAT-1	singlet	0	-806.3080915	-2372.624829	-2467.673093	EMD1
GTCGTAACAATAGGGC-1	singlet	2	-1103.004582	-872.3563621	-365.703866	EMD1
GTCGTAACACCTGCTT-1	singlet	1	-2395.799897	-686.9952257	-1930.623342	EMD1
GTCGTAACACCTGTCT-1	singlet	2	-222.9745726	-196.1936815	-98.12752176	EMD1
GTCGTAAGTAGGCTCC-1	singlet	0	-418.8605574	-1482.929325	-1355.611309	EMD1
GTCGTAAGTAGTCGGA-1	singlet	0	-216.4719236	-662.1622446	-654.7194987	EMD1
GTCGTAAGTGATGGCA-1	singlet	0	-65.58892951	-187.4962068	-173.1891954	EMD1
GTCGTAAGTTGGTGTT-1	singlet	2	-300.5659434	-212.8971122	-120.2538604	EMD1
GTCGTAATCAAGCCAT-1	singlet	0	-834.4203672	-1976.343676	-1818.213929	EMD1
GTCGTAATCCATTCAT-1	singlet	2	-281.3672431	-229.9549448	-122.8946576	EMD1
GTCGTTCAGCATGGGT-1	singlet	2	-165.0775613	-160.6636423	-66.86758608	EMD1
GTCGTTCAGCCTCATA-1	doublet	1/0	-2899.115497	-1670.316323	-2878.104807	EMD1
GTCGTTCAGGCTTAAA-1	singlet	0	-272.8435647	-892.308283	-891.9417022	EMD1
GTCGTTCCAATCCTAG-1	singlet	0	-506.0268505	-1420.153586	-1398.294637	EMD1
GTCGTTCCAGACCAAG-1	singlet	2	-2868.496185	-2257.122023	-843.380284	EMD1
GTCGTTCGTACACGTT-1	singlet	2	-591.0281734	-507.9113794	-235.8206074	EMD1
GTCGTTCGTCTAGTGT-1	singlet	2	-258.7165952	-202.690254	-78.82383532	EMD1
GTCTACCAGACTCAAA-1	singlet	1	-3266.517375	-981.1634157	-2668.021103	EMD1
GTCTACCAGCTCGAAG-1	singlet	0	-660.7489802	-2461.335323	-2268.85121	EMD1
GTCTACCAGTGCAGGT-1	singlet	1	-5232.450574	-1490.697501	-3970.305477	EMD1
GTCTACCCACCGGTCA-1	singlet	1	-6212.767338	-2064.226217	-5115.647267	EMD1
GTCTACCGTCCACTTC-1	singlet	2	-214.1684502	-167.6830059	-86.03938135	EMD1
GTCTACCTCAAAGGTA-1	singlet	1	-1543.858344	-595.4859215	-1207.423209	EMD1
GTCTACCTCAGACCCG-1	singlet	2	-2603.466408	-2179.834445	-960.6829711	EMD1
GTCTAGAAGAGCACTG-1	singlet	0	-283.1525067	-868.6954068	-867.2912358	EMD1
GTCTAGAAGGTGCTTT-1	singlet	1	-1097.861098	-338.7623732	-890.4323572	EMD1
GTCTAGACACGCGCTA-1	singlet	2	-605.880794	-576.2294214	-234.4125014	EMD1
GTCTAGACACTGAGGA-1	singlet	2	-379.6872	-317.4599522	-155.8008293	EMD1
GTCTAGACAGCAATTC-1	singlet	2	-2330.179129	-1834.769689	-778.863556	EMD1
GTCTAGAGTACCTAGT-1	singlet	0	-132.9432708	-568.7014936	-604.2634512	EMD1
GTCTAGATCAAGCCAT-1	singlet	2	-454.9656478	-458.2421886	-218.1067037	EMD1
GTCTAGATCAGGAAAT-1	singlet	2	-718.8690828	-599.6146735	-305.4996061	EMD1
GTCTAGATCAGGAACG-1	singlet	2	-358.1402285	-276.4087035	-146.6587908	EMD1
GTCTAGATCCCGAGAC-1	singlet	0	-517.0644979	-2868.149243	-2298.530693	EMD1
GTCTAGATCGTCAAAC-1	singlet	2	-1073.034675	-1093.466616	-462.9103172	EMD1
GTCTAGATCTGTCGTC-1	singlet	0	-1398.968733	-8534.269922	-7092.311582	EMD1
GTCTCACAGCGTTACT-1	singlet	2	-1561.086027	-1826.121499	-590.2371746	EMD1
GTCTCACAGCTAGAAT-1	singlet	1	-2755.944517	-904.2922121	-2026.563079	EMD1
GTCTCACAGGCCCACT-1	singlet	0	-7.499911723	-16.41533871	-9.869287049	EMD1
GTCTCACAGGCGAACT-1	singlet	0	-179.0137598	-465.9659101	-528.1714732	EMD1

GTCTCACCACCAGACC-1	singlet	0	-104.533248	-253.0776194	-258.7620038	EMD1
GTCTCACCACGCAAAG-1	singlet	0	-460.030135	-2251.476251	-2042.476662	EMD1
GTCTCACGTCAAGCCC-1	singlet	2	-3150.519987	-2361.712043	-1062.13437	EMD1
GTCTCACGTTCGTAAC-1	singlet	2	-1142.781619	-870.9621605	-394.1878256	EMD1
GTCTCACGTTGCTCCT-1	singlet	2	-3206.923786	-2657.088948	-1096.435015	EMD1
GTCTCACTCACTTGTT-1	singlet	2	-5543.440031	-4537.34676	-1828.88489	EMD1
GTCTCACTCCGTAGTA-1	singlet	0	-119.5364013	-321.5835213	-330.8040503	EMD1
GTCTCACTCCTAAACG-1	singlet	2	-99.01661352	-83.29266508	-27.77322189	EMD1
GTCTCACTCGTGCTCT-1	singlet	2	-530.3248524	-487.776705	-207.0428386	EMD1
GTCTCACTCTCGTTTA-1	singlet	1	-3000.955658	-929.1918339	-2331.126581	EMD1
GTCTCACTCTCTCGAC-1	singlet	2	-2260.293728	-2108.276728	-727.5037265	EMD1
GTCTGTCAGCCATGCC-1	unassigned	0	-5.543219121	-7.564306701	-91.58679047	EMD1
GTCTGTCAGCCTGCCA-1	singlet	2	-499.6566633	-404.1492202	-201.5266141	EMD1
GTCTGTCAGGAGGGTG-1	singlet	2	-271.4411866	-230.7217409	-99.30230771	EMD1
GTCTGTCCAACTGATC-1	singlet	2	-485.1570609	-382.9259683	-177.1683667	EMD1
GTCTGTCCAAGCGAGT-1	singlet	0	-694.109245	-2102.180933	-1847.011962	EMD1
GTCTGTCCATCTTTCA-1	singlet	0	-347.9913875	-944.8223519	-893.3383273	EMD1
GTCTGTCGTAGAGCTG-1	singlet	2	-3609.722647	-2725.408068	-1161.698934	EMD1
GTCTGTCGTTTGTTCT-1	singlet	2	-360.8656655	-333.4652486	-160.1291406	EMD1
GTCTGTCTCACCTGTC-1	singlet	2	-439.103009	-358.643079	-186.214097	EMD1
GTCTGTCTCGGTCATA-1	singlet	2	-2610.342303	-1890.824675	-654.3328341	EMD1
GTCTTTAAGCAGCCTC-1	singlet	2	-2577.857405	-2425.871093	-1071.931102	EMD1
GTCTTTAAGTCTGTAC-1	singlet	1	-1519.601161	-578.1150814	-1257.937792	EMD1
GTCTTTAAGTTACTCG-1	singlet	0	-39.64387892	-109.0629921	-114.3416516	EMD1
GTCTTTACACAAGCTT-1	singlet	0	-65.01995328	-136.986499	-142.6641816	EMD1
GTCTTTAGTCGACTTA-1	singlet	2	-457.0227301	-378.9837012	-177.1111984	EMD1
GTCTTTAGTGATTCAC-1	singlet	0	-177.9863066	-492.006839	-524.3392923	EMD1
GTCTTTAGTGGCGTAA-1	doublet	1/0	-1438.072681	-1055.342362	-1525.773463	EMD1
GTCTTTATCACCGGTG-1	singlet	2	-332.6033764	-264.2623177	-130.1696201	EMD1
GTCTTTATCCAGTGTA-1	singlet	2	-1313.806637	-1093.123226	-478.4831569	EMD1
GTCTTTATCCTAACAG-1	singlet	1	-2652.21547	-945.6444917	-2082.596328	EMD1
GTGACGCAGAGTTGAT-1	singlet	2	-2158.435842	-2266.598657	-638.252155	EMD1
GTGACGCAGCCTAGGA-1	singlet	2	-310.500806	-251.3356135	-113.5517685	EMD1
GTGACGCAGTAGAGTT-1	singlet	2	-418.3375451	-350.5155841	-184.6166132	EMD1
GTGACGCAGTTATGGA-1	singlet	0	-148.7763089	-421.5818704	-489.7743907	EMD1
GTGACGCGTCTCGGGT-1	singlet	1	-3254.299316	-888.1807273	-2664.043256	EMD1
GTGACGCTCACCATAG-1	singlet	2	-1966.493315	-1652.199767	-687.2792913	EMD1
GTGACGCTCCTTATAC-1	singlet	2	-443.8889389	-361.5679853	-171.3814586	EMD1
GTGACGCTCGGCACTG-1	singlet	1	-2996.987858	-841.618111	-2298.150383	EMD1
GTGACGCTCGTGTGGC-1	singlet	2	-1334.05914	-1255.317385	-498.2315803	EMD1
GTGAGCCAGATCCCGC-1	singlet	1	-2963.719346	-610.8061769	-2553.087708	EMD1
GTGAGCCAGGTTCTTG-1	singlet	1	-627.0341761	-256.6234644	-544.5841738	EMD1
GTGAGCCCACCTGCGA-1	singlet	2	-605.2490094	-479.2128539	-174.1605558	EMD1
GTGAGCCGTACCGTCG-1	singlet	2	-132.3307454	-113.4549818	-42.71483757	EMD1
GTGAGCCGTATAGGGC-1	singlet	1	-1639.996205	-408.7741569	-1379.615629	EMD1
GTGAGCCGTCAAGTTC-1	singlet	1	-273.1660397	-107.7043791	-264.8015615	EMD1
GTGAGCCGTGGGTTGA-1	singlet	0	-369.496273	-1237.667952	-1152.984016	EMD1
GTGAGCCGTGTGCTTA-1	singlet	0	-658.2247456	-2066.64082	-2120.68176	EMD1
GTGAGCCGTTTCCCAC-1	singlet	2	-297.9015623	-240.2806404	-112.8670409	EMD1
GTGAGCCTCAACACCA-1	singlet	2	-118.0955885	-113.2685826	-47.73220389	EMD1
GTGAGGAAGCTTTGTG-1	singlet	0	-614.3165384	-1778.812296	-1773.821189	EMD1
GTGAGGAGTGAGACGT-1	singlet	0	-821.2759489	-2172.661049	-2144.736681	EMD1
GTGAGGAGTGTAGGAC-1	singlet	1	-1353.080301	-519.072843	-1147.410743	EMD1
GTGAGGAGTTCGGACC-1	singlet	2	-267.2733922	-214.2286892	-111.5653372	EMD1
GTGAGTTAGGGTTGCA-1	singlet	2	-3833.885686	-3089.90739	-1315.6075	EMD1
GTGAGTTAGTGATGGC-1	singlet	2	-595.3371827	-550.1356704	-289.6692209	EMD1
GTGAGTTCAACCAACT-1	singlet	2	-1172.246704	-894.6243116	-429.5815532	EMD1
GTGAGTTCAAGCTCTA-1	singlet	0	-235.3236166	-562.5565932	-595.5212532	EMD1
GTGAGTTCACCTAAAC-1	singlet	0	-210.3479864	-562.0575207	-653.0768194	EMD1
GTGAGTTGTTCTCTCG-1	singlet	2	-257.7672429	-285.6476326	-113.9292465	EMD1
GTGATGTAGGAGCTGT-1	singlet	2	-441.1041933	-362.0753355	-175.7866964	EMD1
GTGATGTAGGGAGGGT-1	singlet	2	-505.0130997	-412.1510919	-199.8673455	EMD1
GTGATGTCAAGAGCTG-1	singlet	1	-870.7182644	-284.9197755	-750.2742799	EMD1
GTGATGTCAATGGGTG-1	singlet	2	-467.6376172	-405.3134218	-136.1530643	EMD1
GTGATGTCACTATCGA-1	singlet	2	-468.7384563	-351.5985276	-166.610016	EMD1
GTGATGTCAGCGGTCT-1	singlet	2	-1330.432391	-1015.884151	-472.7906373	EMD1
GTGATGTGTCGGTGAA-1	singlet	2	-1399.352075	-1254.067499	-481.7771608	EMD1
GTGATGTGTTACCCAA-1	singlet	2	-313.4402792	-286.3393715	-132.8525575	EMD1
GTGATGTGTTCAGCGC-1	singlet	0	-161.7663679	-515.8113436	-493.9819643	EMD1
GTGATGTTCAGCGGAA-1	singlet	0	-38.97647664	-126.7352356	-112.4649282	EMD1
GTGATGTTCATAGAGA-1	singlet	2	-346.0598755	-381.1742681	-155.855476	EMD1
GTGATGTTCATCGCAA-1	singlet	0	-349.5267745	-1109.205042	-947.8605013	EMD1
GTGATGTTCGCCAACG-1	singlet	1	-250.7958998	-76.09487716	-207.3500643	EMD1
GTGCACGAGTAGCTCT-1	singlet	2	-545.9507205	-402.9893274	-203.1850782	EMD1
GTGCACGCAACAAAGT-1	singlet	2	-401.8963922	-298.3951564	-155.8536063	EMD1
GTGCACGCAACTCATG-1	doublet	45293	-798.2262295	-459.6854343	-542.3351957	EMD1
GTGCACGGTTGGACTT-1	singlet	2	-460.2102661	-456.8338391	-228.4378706	EMD1
GTGCACGTCCGTCCTA-1	singlet	0	-886.7043194	-2580.861187	-2639.336776	EMD1
GTGCAGCAGCATTGTC-1	singlet	2	-688.0848629	-556.0718625	-284.2235971	EMD1
GTGCAGCAGCTAATGA-1	singlet	2	-640.3285868	-640.5743555	-278.5212895	EMD1
GTGCAGCGTATTTCCT-1	singlet	1	-429.6328051	-208.0458526	-455.0310504	EMD1
GTGCAGCGTCAACGCC-1	singlet	0	-358.2628333	-1501.904146	-1367.321365	EMD1
GTGCAGCGTGCTCTTC-1	singlet	0	-175.9744978	-453.9112537	-441.5929114	EMD1
GTGCAGCGTGTCCCTT-1	singlet	1	-3016.607575	-1057.599977	-2364.491512	EMD1
GTGCAGCTCATAGGCT-1	singlet	0	-304.5723595	-811.9662658	-759.3076399	EMD1
GTGCAGCTCCTTTAGT-1	singlet	2	-365.8211828	-354.990009	-162.7543817	EMD1
GTGCGTGAGATCACCT-1	singlet	2	-567.275812	-471.044129	-219.3222779	EMD1
GTGCGTGAGTTACTCG-1	singlet	2	-378.9329838	-375.9831807	-180.5617072	EMD1
GTGCGTGCACTTTAGG-1	singlet	0	-300.6460544	-1340.706083	-1167.795208	EMD1
GTGCGTGCATTGTGCA-1	singlet	1	-395.2949652	-132.5050615	-316.7491137	EMD1
GTGCGTGGTCAAGCGA-1	singlet	1	-101.5665693	-38.81023675	-61.51314948	EMD1
GTGCGTGGTTAACCTG-1	singlet	0	-370.4207422	-1655.97885	-1324.719778	EMD1
GTGCGTGTCGTAACCA-1	singlet	1	-1832.211404	-636.044386	-1487.835434	EMD1
GTGCGTGTCTGCATGA-1	singlet	1	-1795.768393	-524.5514512	-1374.791831	EMD1
GTGCTGGAGAGTCTTC-1	singlet	0	-560.132078	-1842.720046	-1757.177748	EMD1
GTGCTGGAGCCAGACA-1	singlet	2	-1169.519125	-913.5789895	-380.4132979	EMD1
GTGCTGGAGCCTCCAG-1	singlet	0	-45.0036111	-138.4339259	-133.5481088	EMD1
GTGCTGGCAATTCTCT-1	singlet	0	-1144.300427	-2444.52769	-2352.725016	EMD1
GTGCTGGCACTTTATC-1	singlet	0	-509.6559642	-1580.558048	-1536.957392	EMD1
GTGCTGGGTCATTCCC-1	singlet	0	-823.7323967	-2391.246387	-2463.843605	EMD1
GTGCTGGGTGGTTTGT-1	singlet	2	-469.7729579	-515.8136634	-231.596626	EMD1
GTGCTGGTCAACGTGT-1	singlet	1	-2871.67302	-843.7377039	-1999.475721	EMD1
GTGCTGGTCATTGCCC-1	singlet	2	-455.765094	-394.6628976	-186.9749079	EMD1
GTGCTGGTCGGACGTC-1	singlet	0	-1380.171453	-4052.050268	-3973.953115	EMD1
GTGCTTCAGGAGTACC-1	singlet	2	-17.89599296	-9.875121151	-5.299026517	EMD1
GTGCTTCCACCCTTAC-1	singlet	2	-450.4563297	-406.3407673	-174.6507333	EMD1
GTGCTTCCACGTACTA-1	singlet	0	-755.9348551	-2114.431104	-2139.487927	EMD1
GTGCTTCGTAAGTAGT-1	singlet	1	-2198.907751	-627.8776469	-1608.939484	EMD1
GTGCTTCGTACCGCGT-1	unassigned	2	-6.360941714	-5.497565435	-3.06196879	EMD1
GTGCTTCGTTACGTAC-1	singlet	1	-438.7366758	-161.1650586	-424.4918939	EMD1

GTGCTTCTCAAACCTG-1	singlet	1	-2232.915792	-599.4489875	-1815.788869	EMD1
GTGCTTCTCAGTGCGC-1	singlet	0	-146.8776858	-418.668308	-365.8086511	EMD1
GTGCTTCTCCAAGCAT-1	singlet	2	-295.6918286	-247.4835224	-115.5465392	EMD1
GTGCTTCTCCCTAGGG-1	singlet	1	-1629.49445	-484.1585853	-1266.494671	EMD1
GTGCTTCTCCGTGACG-1	singlet	2	-85.36664102	-66.52037105	-26.63113959	EMD1
GTGGAAGAGTGGAAAG-1	singlet	1	-2145.780143	-769.8056678	-1723.310949	EMD1
GTGGAAGCAAATACGA-1	singlet	1	-585.3273809	-208.7614688	-567.3435998	EMD1
GTGGAAGCAAGGGTCA-1	doublet	0/2	-1392.512308	-2196.931485	-1723.690226	EMD1
GTGGAAGCACCCTGTT-1	singlet	0	-579.473945	-1536.36375	-1570.257826	EMD1
GTGGAAGCATGTGGCC-1	singlet	0	-1415.819102	-5035.037053	-4660.816369	EMD1
GTGGAAGGTCACTCGG-1	singlet	2	-3035.007405	-2485.826361	-942.3545957	EMD1
GTGGAAGGTCATGCAT-1	singlet	0	-112.8702386	-397.2233256	-538.3726339	EMD1
GTGGAAGGTGAGGATC-1	singlet	2	-211.8247262	-147.3923088	-76.37282843	EMD1
GTGGAAGGTGTTAGCT-1	singlet	1	-2038.123852	-682.1123943	-1659.509734	EMD1
GTGGAAGTCCCTGTTG-1	singlet	2	-315.0281422	-247.9784631	-136.9260833	EMD1
GTGGAAGTCCTTCACG-1	singlet	2	-256.9849812	-224.4556088	-108.7435703	EMD1
GTGGAAGTCGGAGTGA-1	singlet	2	-416.0267663	-401.2834236	-154.6554467	EMD1
GTGGAAGTCTCGTGAA-1	singlet	1	-3549.460024	-925.1709822	-2662.760795	EMD1
GTGGAGAAGTCTAGCT-1	singlet	1	-5489.014241	-1751.29325	-4284.931614	EMD1
GTGGAGAAGTCTGTAC-1	singlet	0	-522.6554159	-1554.952546	-1712.338701	EMD1
GTGGAGAAGTTTAGGA-1	singlet	0	-461.3882602	-1141.772988	-1232.300061	EMD1
GTGGAGAGTACGGTTT-1	singlet	2	-1618.608719	-1365.39899	-519.4758954	EMD1
GTGGAGAGTACGTGTT-1	singlet	2	-638.6879641	-503.1039242	-245.2510939	EMD1
GTGGAGAGTCAACCTA-1	singlet	1	-333.6628136	-121.4229685	-318.5047432	EMD1
GTGGAGAGTCTACGAT-1	singlet	2	-283.8540496	-243.9618048	-127.6336165	EMD1
GTGGAGAGTGAGAGGG-1	singlet	0	-483.183988	-1703.819059	-1511.606545	EMD1
GTGGAGATCCAGCCTT-1	singlet	1	-700.5761846	-211.2727947	-567.6194495	EMD1
GTGGCGTCATTGTACG-1	singlet	0	-362.3903979	-1141.230382	-1194.117189	EMD1
GTGGCGTGTACCACGC-1	singlet	0	-130.9707103	-384.5992932	-526.3289952	EMD1
GTGGCGTGTGTCCGGT-1	singlet	0	-392.2482733	-1195.239352	-1094.214443	EMD1
GTGGCGTTCAGCTGTA-1	singlet	1	-261.9985405	-117.9655125	-275.13642	EMD1
GTGGCGTTCTGAGAGG-1	singlet	2	-950.9553761	-842.1723067	-391.4723784	EMD1
GTGGCGTTCTTCCCGA-1	singlet	1	-2847.182839	-809.2020317	-1903.123088	EMD1
GTGGGAAAGGCATGGT-1	singlet	0	-457.2604177	-1598.829457	-1347.936065	EMD1
GTGGGAACAATGTCTG-1	singlet	0	-2176.62385	-6778.321512	-5543.581404	EMD1
GTGGGAACACTTCTCG-1	singlet	0	-554.5155642	-1829.691778	-1783.981615	EMD1
GTGGGAAGTAATGCTC-1	singlet	0	-1039.896513	-2811.779262	-2957.871813	EMD1
GTGGGAAGTCAAAGAT-1	singlet	0	-381.005176	-1287.531379	-1154.656331	EMD1
GTGGGAAGTCGTAATC-1	singlet	0	-620.8181648	-1984.533589	-2071.505949	EMD1
GTGGGAAGTGAACGGT-1	singlet	2	-245.6960057	-218.1002683	-108.8738116	EMD1
GTGGGAAGTTATGACC-1	singlet	2	-2898.906964	-2553.046935	-975.9995489	EMD1
GTGGGAAGTTGCGGCT-1	singlet	2	-3740.954522	-2746.909877	-1116.889761	EMD1
GTGGGAATCTAACACG-1	singlet	0	-184.251635	-437.4184416	-416.5898126	EMD1
GTGGTTAAGTGCAGGT-1	singlet	0	-216.3028288	-595.5418686	-619.6180854	EMD1
GTGGTTAAGTTTCAGC-1	unassigned	0/1	-9.310863222	-12.91300955	-20.61577996	EMD1
GTGGTTAGTCAATCTG-1	singlet	2	-247.4188285	-177.4253985	-89.98409134	EMD1
GTGGTTATCGGAAACG-1	doublet	2/0	-3428.424192	-3706.912277	-2509.89655	EMD1
GTGTAACAGACCAACG-1	singlet	0	-3525.234864	-12864.84405	-13044.4662	EMD1
GTGTAACAGGGTTAGC-1	singlet	0	-537.5062725	-1858.695333	-1872.711879	EMD1
GTGTAACAGGTATTGA-1	singlet	0	-499.7493458	-1630.690369	-1478.01323	EMD1
GTGTAACAGTTCACTG-1	singlet	0	-319.6912799	-1153.65782	-999.6918001	EMD1
GTGTAACCACGCTGAC-1	singlet	0	-1191.774583	-3753.302445	-3583.003029	EMD1
GTGTAACGTAACGTTC-1	singlet	1	-1074.971164	-388.7396084	-828.7516396	EMD1
GTGTAACGTGAACGGT-1	singlet	1	-3008.904027	-645.9864842	-2554.609	EMD1
GTGTCCTAGATTGCGG-1	singlet	1	-1691.59933	-477.8179518	-1407.666157	EMD1
GTGTCCTAGCTAGAGC-1	singlet	0	-2576.016738	-8570.267703	-8803.430863	EMD1
GTGTCCTAGTGACACG-1	singlet	1	-3216.175528	-1168.229989	-2423.176104	EMD1
GTGTCCTCAATGGCAG-1	singlet	2	-738.8946358	-644.5274438	-253.7627288	EMD1
GTGTCCTCACATCATG-1	doublet	2/0	-646.6456702	-569.8510904	-343.3368313	EMD1
GTGTCCTCACGCTATA-1	singlet	2	-480.7042308	-373.6027363	-233.2429484	EMD1
GTGTCCTCACGCTGAC-1	singlet	2	-1200.833422	-1075.651768	-421.3902159	EMD1
GTGTCCTCACGCTGCA-1	singlet	2	-508.248279	-458.9676227	-207.1811512	EMD1
GTGTCCTTCTTGGTGA-1	singlet	1	-1544.992326	-493.5305691	-1335.577426	EMD1
GTGTGATAGCAGTACG-1	singlet	0	-426.1671127	-961.9329889	-803.6488939	EMD1
GTGTGATAGGCCTTCG-1	singlet	0	-237.8540063	-763.1624548	-752.3509359	EMD1
GTGTGATAGGTCCAGA-1	singlet	2	-547.8177886	-435.5726867	-187.00036	EMD1
GTGTGATCAATGGGTG-1	singlet	0	-426.9428973	-1517.496059	-1733.930967	EMD1
GTGTGATCATTGTCGA-1	singlet	1	-1728.959566	-701.350049	-1475.417291	EMD1
GTGTGATGTATCAGGG-1	singlet	1	-300.5040155	-127.5439541	-256.7589692	EMD1
GTGTGATGTGTTCGTA-1	singlet	1	-1815.232436	-561.4454165	-1419.117658	EMD1
GTGTGATTCCTGCCAT-1	singlet	2	-2604.58238	-2483.038919	-773.2932741	EMD1
GTGTGGCAGACCTCCG-1	singlet	2	-1272.773611	-1006.735603	-377.1681355	EMD1
GTGTGGCCACTAACGT-1	singlet	0	-922.8742448	-2905.15967	-2955.718169	EMD1
GTGTGGCCACTAGGCC-1	singlet	1	-591.3059729	-194.4911135	-419.2877823	EMD1
GTGTGGCCAGGTTCAT-1	singlet	0	-1044.606423	-3243.95031	-3461.467079	EMD1
GTGTGGCGTCCGACGT-1	singlet	1	-1877.050883	-907.8988778	-2016.00879	EMD1
GTGTGGCGTTGCACGC-1	singlet	1	-933.6415622	-349.5614557	-913.5368203	EMD1
GTGTTAGAGCTGACAG-1	singlet	1	-5413.635913	-1194.543051	-4196.083186	EMD1
GTGTTAGAGGGAGGGT-1	singlet	2	-521.4612175	-431.3569548	-199.7942164	EMD1
GTGTTAGCAATTGCGT-1	singlet	2	-2137.969608	-1685.907168	-569.9175645	EMD1
GTGTTAGCATTGACCA-1	singlet	2	-553.6187558	-449.8026727	-211.6234064	EMD1
GTGTTAGGTAATCAGA-1	singlet	2	-6459.730215	-5061.488063	-2310.741236	EMD1
GTGTTAGGTCTGCATA-1	singlet	0	-618.2717088	-1869.524118	-1940.516343	EMD1
GTGTTAGGTGACACAG-1	singlet	0	-179.3153274	-409.1522397	-517.0834247	EMD1
GTGTTAGTCCAAGAGG-1	singlet	0	-608.3632503	-2183.21557	-1857.934929	EMD1
GTGTTAGTCCCTCGTA-1	singlet	0	-375.0316255	-1332.890104	-1232.523812	EMD1
GTGTTAGTCGCTGTCT-1	doublet	1/0	-1144.021104	-1121.644861	-1442.969386	EMD1
GTGTTAGTCTCCATAT-1	singlet	2	-1741.170567	-1413.253369	-637.8738164	EMD1
GTGTTCCAGAAACTCA-1	singlet	0	-143.1376323	-369.4851278	-412.2750198	EMD1
GTGTTCCAGTAATACG-1	singlet	0	-34.40039458	-94.4784696	-100.8571963	EMD1
GTGTTCCCATCTCATT-1	singlet	1	-190.1456173	-51.19814131	-153.1254021	EMD1
GTGTTCCGTAGCTCGC-1	singlet	2	-328.2339945	-278.3843942	-125.015335	EMD1
GTGTTCCGTCGATTAC-1	singlet	1	-1386.48709	-494.2328264	-1191.977757	EMD1
GTGTTCCGTGCATTAC-1	singlet	2	-513.7026859	-386.6504476	-229.5957678	EMD1
GTGTTCCGTTCGGTCG-1	singlet	1	-3503.44419	-1172.45014	-2692.36851	EMD1
GTGTTCCGTTGGAGAC-1	singlet	0	-466.4395649	-1478.831407	-1484.990097	EMD1
GTGTTCCTCAGTCATG-1	singlet	2	-1612.603747	-1631.575655	-608.6864532	EMD1
GTTACAGAGAGAAGGT-1	singlet	0	-70.0692495	-245.3484106	-226.1048967	EMD1
GTTACAGTCCATCTAT-1	singlet	1	-2231.443641	-779.9883852	-1868.046873	EMD1
GTTACAGTCTGATTCT-1	singlet	2	-593.6496499	-495.7895656	-279.4302073	EMD1
GTTACCCCACTAAACC-1	singlet	2	-662.676771	-593.9346197	-252.2936711	EMD1
GTTACCCCAGAGATTA-1	singlet	0	-344.5525499	-1303.551677	-1158.722896	EMD1
GTTACCCCAGAGTCTT-1	singlet	2	-519.2494562	-494.6606353	-204.1041049	EMD1
GTTACCCCATACACCA-1	singlet	0	-454.6210748	-1583.66986	-1635.18383	EMD1
GTTACCCGTCTTTATC-1	singlet	2	-6819.929811	-5441.075708	-2209.790356	EMD1
GTTACCCGTGAGATCG-1	singlet	1	-138.2418787	-57.53656836	-139.0063144	EMD1
GTTACCCTCATTACCT-1	singlet	2	-786.7761758	-632.9520159	-308.1853924	EMD1
GTTACCCTCCACGGGT-1	singlet	1	-961.4119564	-566.8862461	-801.4508154	EMD1
GTTACCCTCTATCGTT-1	singlet	1	-171.7627546	-43.89338636	-98.46865736	EMD1
GTTACGAAGAGCCCAA-1	singlet	0	-604.1832225	-2205.260581	-2101.223531	EMD1

GTTACGAAGATCCTAC-1	singlet	1	-4546.183531	-1258.986156	-3389.705097	EMD1
GTTACGAGTATCGCTA-1	singlet	2	-1391.448985	-1103.209666	-507.7789659	EMD1
GTTACGAGTCATCCGG-1	singlet	0	-264.7469918	-713.7154041	-711.7497514	EMD1
GTTACGAGTGCGACAA-1	singlet	1	-1136.129181	-237.1342695	-987.7575513	EMD1
GTTACGAGTTAAGGGC-1	doublet	45323	-464.6051495	-350.9268641	-239.9788592	EMD1
GTTACGAGTTGGTAGG-1	singlet	2	-137.9405765	-84.85196186	-39.67983602	EMD1
GTTACGATCCGTGTCT-1	doublet	45323	-3234.693736	-2068.760789	-1747.628005	EMD1
GTTACGATCGTGCTCT-1	singlet	2	-458.8215262	-335.0440317	-169.1214478	EMD1
GTTACGATCGTGTGAT-1	singlet	0	-183.0219688	-564.1814672	-542.8592322	EMD1
GTTACGATCTAGTGTG-1	singlet	0	-165.8903745	-686.6026421	-532.9464364	EMD1
GTTACGATCTTTGCAT-1	singlet	0	-2010.657686	-8843.216505	-7237.977228	EMD1
GTTAGACAGCCATATC-1	singlet	2	-2417.581895	-2023.59391	-864.0693611	EMD1
GTTAGACGTACAGCGA-1	singlet	1	-1356.295276	-471.8367456	-1021.113067	EMD1
GTTAGACGTCCACACG-1	singlet	1	-174.4233452	-60.72215268	-148.4327581	EMD1
GTTAGACGTCCAGGTC-1	singlet	1	-527.4958321	-237.4289205	-484.3147587	EMD1
GTTAGACGTCTTGCGG-1	singlet	0	-26.6486257	-76.63315324	-81.77535398	EMD1
GTTAGACTCCAGGACC-1	singlet	2	-414.4747182	-385.7966192	-153.4420808	EMD1
GTTAGACTCTCGACCT-1	singlet	2	-646.1816876	-593.4753944	-287.1902087	EMD1
GTTAGTGAGGCAGGTT-1	singlet	2	-896.7028983	-863.5870729	-348.6541686	EMD1
GTTAGTGAGGGTACAC-1	singlet	1	-1039.908337	-353.9868543	-865.365724	EMD1
GTTAGTGAGGTAGCAC-1	singlet	2	-266.1042285	-246.2400342	-123.6896615	EMD1
GTTAGTGAGTGTTCCA-1	singlet	0	-68.80943615	-175.545675	-214.4834515	EMD1
GTTAGTGCATAATGAG-1	singlet	1	-4100.549419	-1003.883028	-3391.966446	EMD1
GTTAGTGGTATGAAAC-1	singlet	0	-417.9434153	-1167.012159	-1073.715492	EMD1
GTTAGTGGTCTATGAC-1	singlet	0	-192.9800271	-492.9637383	-527.8537129	EMD1
GTTAGTGGTGGAGAAA-1	singlet	2	-452.5144383	-367.8754915	-197.6145011	EMD1
GTTAGTGTCACCGACG-1	singlet	2	-6058.72292	-4714.889749	-1952.230525	EMD1
GTTAGTGTCATACGAC-1	singlet	1	-3630.342461	-1099.520254	-2880.033176	EMD1
GTTAGTGTCGCCGAAC-1	singlet	1	-4325.804872	-1110.48979	-3134.716181	EMD1
GTTATGGAGGCAGCTA-1	singlet	2	-487.2709808	-398.1470524	-176.8713033	EMD1
GTTATGGAGGTCCAGA-1	singlet	2	-1122.145095	-1027.354695	-440.4533844	EMD1
GTTATGGAGTACAACA-1	singlet	1	-3370.182232	-1059.476953	-2855.60843	EMD1
GTTATGGCACGGCTAC-1	singlet	0	-464.8282777	-1267.94703	-1144.750874	EMD1
GTTATGGGTGAGGCAT-1	singlet	1	-409.6419926	-141.1789595	-364.5307442	EMD1
GTTATGGGTGTGTACT-1	singlet	0	-443.3891983	-1397.338666	-1522.38588	EMD1
GTTATGGTCCCTCGAT-1	singlet	2	-664.2218557	-565.5867435	-270.4250297	EMD1
GTTCATTCAATATCCG-1	singlet	2	-2646.202594	-2460.803416	-1050.962258	EMD1
GTTCATTCACGACGTC-1	singlet	0	-568.6347042	-1857.683415	-1649.973487	EMD1
GTTCATTTCGTTACCC-1	singlet	2	-402.6845838	-323.8675057	-185.1921095	EMD1
GTTCATTTCGTTGTGA-1	singlet	0	-3547.933704	-12229.67557	-12338.15808	EMD1
GTTCATTTCTCTCGCA-1	singlet	0	-513.0436499	-1414.651835	-1433.104924	EMD1
GTTCATTTCTGAGAAA-1	singlet	0	-349.185753	-1051.613132	-1064.17328	EMD1
GTTCCGTAGATTGAGT-1	singlet	1	-567.9222549	-249.9360083	-591.1953995	EMD1
GTTCCGTAGTACCATC-1	singlet	0	-252.9657852	-858.5597044	-806.0401656	EMD1
GTTCCGTAGTGCCAGA-1	singlet	0	-185.4352581	-568.8126351	-515.933732	EMD1
GTTCCGTCAATACCTG-1	singlet	2	-4437.428695	-3727.227233	-2434.695151	EMD1
GTTCCGTCACTGGAAG-1	singlet	0	-38.56348917	-93.22691594	-83.17209026	EMD1
GTTCCGTGTAACTGCT-1	doublet	45293	-706.79347	-467.6893044	-478.5967579	EMD1
GTTCCGTGTAGCGTAG-1	singlet	1	-499.6770839	-144.1477457	-600.1851839	EMD1
GTTCCGTGTGTTTGCA-1	singlet	0	-1331.446885	-4124.009204	-3739.305836	EMD1
GTTCCGTTCGGCTATA-1	singlet	1	-1271.644052	-434.7787385	-1052.306327	EMD1
GTTCGCTAGACATCAA-1	singlet	1	-1829.308424	-610.3379462	-1531.198327	EMD1
GTTCGCTAGCAAATGT-1	singlet	1	-573.3812459	-226.3365438	-541.5959011	EMD1
GTTCGCTCAACCGATT-1	singlet	0	-64.142187	-180.8183416	-164.5701322	EMD1
GTTCGCTCACTTTATC-1	singlet	1	-871.005123	-384.601952	-745.9960797	EMD1
GTTCGCTCAGATTCGT-1	singlet	1	-2961.748062	-892.5897216	-1954.87664	EMD1
GTTCGCTCATGACTCA-1	singlet	2	-708.862996	-589.4285361	-258.7809773	EMD1
GTTCGCTGTATGCAAA-1	singlet	0	-230.7396099	-567.9120354	-538.9423533	EMD1
GTTCGCTGTCGTTATG-1	singlet	2	-299.7269835	-246.9583457	-126.8464081	EMD1
GTTCGCTGTGCTGCAC-1	singlet	2	-6788.488344	-5547.753476	-2143.748537	EMD1
GTTCGCTGTGTAGGAC-1	singlet	1	-1718.201485	-621.0005252	-1255.320009	EMD1
GTTCTATAGAGTTGTA-1	singlet	0	-1256.204714	-4283.232298	-4053.177641	EMD1
GTTCTATCAAGCGAGT-1	singlet	2	-1302.081008	-1107.822908	-504.8514449	EMD1
GTTCTATCACTCGATA-1	singlet	2	-525.8509067	-441.7529595	-210.2135104	EMD1
GTTCTATGTCCTCCAT-1	singlet	0	-519.4426215	-1759.558875	-1616.832744	EMD1
GTTCTATGTCCTCCTA-1	singlet	2	-509.1205959	-396.6037832	-201.2771696	EMD1
GTTCTATGTGGTTTAC-1	singlet	2	-5835.415708	-4281.090686	-1773.858818	EMD1
GTTCTATGTGTGATGG-1	singlet	1	-505.9465753	-221.3798541	-542.2115206	EMD1
GTTCTATTCTTCCACG-1	singlet	1	-4328.695078	-1410.044248	-3341.663104	EMD1
GTTGAACAGATACGAT-1	singlet	2	-636.4655971	-561.2639486	-234.7512211	EMD1
GTTGAACAGCAAGGAA-1	singlet	2	-592.4940601	-573.2244009	-246.509869	EMD1
GTTGAACCAAGCGAAC-1	singlet	2	-675.161537	-591.3004158	-269.5865197	EMD1
GTTGAACCACGTCGTG-1	singlet	1	-1332.19335	-428.4998019	-1081.857894	EMD1
GTTGAACCAGAGGACT-1	singlet	2	-2224.686316	-1901.863926	-730.2636048	EMD1
GTTGAACTCACGTAGT-1	singlet	2	-5313.478451	-4478.891546	-1798.425671	EMD1
GTTGAACTCATGACAC-1	singlet	2	-2732.058247	-2146.81385	-973.0486243	EMD1
GTTGAACTCCGGACGT-1	singlet	1	-1782.218399	-599.9456865	-1454.9848	EMD1
GTTGAACTCCTATTGT-1	singlet	2	-610.8625421	-492.6006476	-250.361396	EMD1
GTTGAACTCGGCATCG-1	singlet	1	-445.9033826	-156.866453	-440.9110434	EMD1
GTTGAACTCTACTTCA-1	singlet	1	-3387.477658	-1163.422147	-2656.073941	EMD1
GTTGAACTCTGCAGCG-1	singlet	1	-5960.362691	-1494.668695	-4896.922672	EMD1
GTTGCGGAGCATTGAA-1	singlet	2	-420.7968663	-403.829171	-173.0915427	EMD1
GTTGCGGAGCGAGTCA-1	singlet	2	-358.2311583	-309.3645234	-134.5574946	EMD1
GTTGCGGAGTAAGAGG-1	singlet	1	-717.0616187	-180.185077	-479.3073894	EMD1
GTTGCGGAGTTGGCTT-1	singlet	2	-3783.86696	-2983.659886	-1092.880359	EMD1
GTTGCGGCAATGTGGG-1	singlet	0	-132.2506392	-470.1195408	-504.3438242	EMD1
GTTGCGGCACCAATTG-1	singlet	2	-1712.505566	-1782.328422	-488.744801	EMD1
GTTGCGGGTATGTCCA-1	singlet	2	-383.5922796	-310.047816	-142.2044441	EMD1
GTTGCGGGTCGTTCAA-1	singlet	0	-271.884556	-860.5323363	-796.4135032	EMD1
GTTGCGGGTGAAAGTT-1	singlet	0	-206.902011	-860.5762002	-743.1466484	EMD1
GTTGCGGGTTTACACG-1	singlet	1	-4122.903	-809.8648497	-3429.511196	EMD1
GTTGCGGTCCAAGCAT-1	singlet	0	-360.4980958	-1357.255055	-1216.937586	EMD1
GTTGCGGTCTGAACGT-1	singlet	0	-394.3510279	-1655.057399	-1398.751407	EMD1
GTTGCTCAGTATGCAA-1	singlet	2	-264.815236	-189.5445702	-90.53006496	EMD1
GTTGCTCCAATGCTCA-1	singlet	0	-409.3389252	-1313.846159	-1217.600542	EMD1
GTTGCTCCACGGCACT-1	doublet	1/0	-3865.687801	-2772.598382	-4365.840143	EMD1
GTTGCTCCACTGCGTG-1	singlet	1	-2892.321391	-803.7747635	-2974.157818	EMD1
GTTGCTCCAGGTCAGA-1	singlet	2	-4712.248233	-3652.297655	-1157.539612	EMD1
GTTGCTCCAGTGCGCT-1	singlet	2	-290.0357267	-259.4859787	-144.0138079	EMD1
GTTGCTCGTATACGGG-1	singlet	0	-682.2392977	-1968.606075	-1796.587016	EMD1
GTTGCTCGTGAGAACC-1	singlet	2	-1902.369804	-1841.778781	-656.6997862	EMD1
GTTGCTCGTGTCCTAA-1	singlet	1	-2412.921547	-691.7662875	-1831.037811	EMD1
GTTGCTCGTTCTAAGC-1	singlet	0	-1002.223222	-3181.117639	-3172.068821	EMD1
GTTGCTCTCAAAGCCT-1	singlet	1	-1052.968504	-382.6906107	-908.1382053	EMD1
GTTGCTCTCCACGAAT-1	singlet	2	-324.0796744	-264.2057872	-148.4882646	EMD1
GTTGCTCTCGAGTGGA-1	singlet	0	-600.5335394	-2342.513311	-2498.072276	EMD1
GTTGCTCTCGGCATAT-1	singlet	0	-1036.733474	-2789.794803	-2681.767213	EMD1
GTTGCTCTCGTAATGC-1	singlet	1	-2456.003838	-877.9909999	-2061.559056	EMD1
GTTGCTCTCTCGCGTT-1	singlet	2	-2040.126108	-1849.602661	-700.3409065	EMD1
GTTGCTCTCTTTGATC-1	singlet	2	-62.40766922	-66.65640381	-28.96360009	EMD1

GTTGTAGAGACCATGG-1	singlet	0	-58.52300916	-235.4374528	-211.0096829	EMD1
GTTGTAGAGGAGCTGT-1	singlet	2	-557.3594751	-485.7133958	-233.0300804	EMD1
GTTGTAGCACAAATCC-1	singlet	1	-130.375201	-50.60160855	-86.20778732	EMD1
GTTGTAGCAGCTGAGA-1	singlet	2	-497.3134583	-486.2944408	-200.7768951	EMD1
GTTGTAGCAGGATTCT-1	singlet	2	-377.7826497	-297.1363804	-137.015964	EMD1
GTTGTAGCATCTGTTT-1	singlet	2	-2022.573331	-1842.671928	-690.3652292	EMD1
GTTGTAGGTATGACAA-1	singlet	2	-633.5345604	-551.4210448	-240.0120369	EMD1
GTTGTAGGTCGTTATG-1	doublet	2/0	-1495.316491	-1927.320164	-1136.055976	EMD1
GTTGTAGGTTAAGGGC-1	singlet	0	-150.9969159	-352.3696826	-346.7804621	EMD1
GTTGTAGTCGATTGAC-1	singlet	1	-690.1487217	-259.8590854	-682.3438547	EMD1
GTTGTCCAGAATCCCT-1	singlet	2	-641.6463727	-502.7765138	-249.403298	EMD1
GTTGTCCAGCTCGACC-1	singlet	2	-997.9185456	-989.6789175	-395.2137776	EMD1
GTTGTCCAGTGCCCGT-1	singlet	2	-486.582017	-421.3476006	-204.2178823	EMD1
GTTGTCCCAGCAGAAC-1	singlet	0	-165.1402121	-382.4707329	-482.5403617	EMD1
GTTGTCCCATCGGTTA-1	singlet	0	-920.5970406	-3107.47897	-3067.486562	EMD1
GTTGTCCCATGACTTG-1	singlet	2	-539.5913796	-468.697864	-238.3893037	EMD1
GTTGTCCGTCTATGAC-1	singlet	2	-2989.080986	-2525.679225	-882.749751	EMD1
GTTGTCCGTTAAACCC-1	singlet	0	-582.3946384	-1770.763532	-1734.868367	EMD1
GTTGTCCGTTATGTCG-1	singlet	2	-1876.1698	-2010.588355	-530.8317674	EMD1
GTTGTCCTCGGCCTTT-1	singlet	2	-712.8349593	-547.0950891	-242.8158527	EMD1
GTTGTGAAGACATAGT-1	singlet	1	-1124.09084	-467.7452544	-939.5534248	EMD1
GTTGTGAAGAGTGAAG-1	singlet	2	-516.3427884	-446.9288352	-185.883798	EMD1
GTTGTGACATCAACCA-1	singlet	2	-6046.127636	-4794.824109	-1813.701261	EMD1
GTTGTGAGTACCAATC-1	singlet	2	-2459.146772	-2046.29595	-621.3498787	EMD1
GTTGTGAGTATAATGG-1	singlet	2	-718.4218689	-573.3860169	-301.0367065	EMD1
GTTGTGAGTATCTCTT-1	singlet	2	-864.2758685	-712.6154301	-276.8837507	EMD1
GTTGTGAGTCGATTCA-1	singlet	1	-4978.274905	-1765.823609	-4262.136031	EMD1
GTTGTGAGTGTATTGC-1	singlet	0	-235.134099	-829.2909696	-791.5433443	EMD1
GTTTACTCATTGCCGG-1	singlet	0	-1013.290637	-3564.804372	-3104.758401	EMD1
GTTTACTTCATTTGCT-1	singlet	1	-2277.444623	-729.5826142	-1843.932747	EMD1
GTTTACTTCCCATGGG-1	singlet	2	-773.8280009	-611.7059981	-262.4122933	EMD1
GTTTACTTCGCTACAA-1	singlet	1	-1362.492126	-448.7119736	-997.1911786	EMD1
GTTTACTTCGGCTATA-1	singlet	1	-2011.257721	-682.7808249	-1534.23079	EMD1
GTTTACTTCGTAGGGA-1	singlet	0	-210.9006289	-800.8381139	-770.2113368	EMD1
GTTTGGAAGCAGCCTC-1	singlet	2	-1799.843122	-1504.412109	-626.497701	EMD1
GTTTGGAAGCGTTAGG-1	singlet	2	-2659.286941	-1939.718486	-853.4478555	EMD1
GTTTGGAAGCTTTCTT-1	singlet	0	-34.66138447	-125.1243526	-98.52959742	EMD1
GTTTGGAAGGCATGCA-1	singlet	1	-2491.343488	-1059.176683	-1819.622809	EMD1
GTTTGGAAGGCGATAC-1	singlet	2	-2625.120292	-2148.663818	-1168.433624	EMD1
GTTTGGAAGGTTGGAC-1	singlet	2	-576.5974723	-445.3872803	-198.2029554	EMD1
GTTTGGACAACGGTAG-1	singlet	1	-1416.233623	-294.8114837	-1145.885988	EMD1
GTTTGGACACCCTAGG-1	singlet	2	-329.6812655	-304.6088347	-157.9020666	EMD1
GTTTGGAGTCTAGTGT-1	singlet	2	-418.0646658	-283.2444165	-151.6061876	EMD1
GTTTGGAGTTCCGGTG-1	singlet	1	-1974.157889	-642.4962766	-1516.92929	EMD1
GTTTGGATCCCAGGCA-1	singlet	2	-1676.212571	-1272.472956	-559.16068	EMD1
GTTTGGATCGGAATTC-1	singlet	2	-269.6517797	-258.0807958	-111.8200992	EMD1
TAACACGCAATGAGCG-1	singlet	1	-6659.636947	-1600.636106	-5245.183559	EMD1
TAACACGCATCACCAA-1	singlet	1	-164.3019778	-56.60173004	-126.1611797	EMD1
TAACACGGTACGTGAG-1	singlet	1	-3500.024976	-1312.303452	-2493.619733	EMD1
TAACACGGTATTTCCT-1	singlet	2	-1237.232123	-1080.441537	-532.1082844	EMD1
TAACACGGTCAAAGAT-1	singlet	0	-148.9213834	-444.558055	-389.8390081	EMD1
TAACACGGTCTAACTG-1	singlet	1	-2049.36764	-615.4265422	-1923.269385	EMD1
TAACACGGTCTAATCG-1	singlet	2	-3159.781522	-2707.977868	-1100.684458	EMD1
TAACACGGTGTGAGCA-1	singlet	0	-40.65342981	-146.0130953	-108.993216	EMD1
TAACACGTCACGACTA-1	singlet	2	-339.6827033	-315.4077393	-90.60300995	EMD1
TAACACGTCAGTGTCA-1	singlet	2	-529.4433062	-429.9281678	-215.8429616	EMD1
TAACACGTCGATCCCT-1	singlet	2	-636.3469399	-496.252448	-260.9302035	EMD1
TAACCAGAGCATAGGC-1	singlet	0	-748.6160915	-2238.555162	-1897.710588	EMD1
TAACCAGAGGATGTTA-1	singlet	0	-902.9131304	-3008.085535	-2879.160847	EMD1
TAACCAGCAATAGGGC-1	singlet	1	-452.9996256	-179.3760004	-432.4970902	EMD1
TAACCAGCACCAGACC-1	singlet	0	-489.9121123	-1564.164343	-1463.033725	EMD1
TAACCAGCAGATGCGA-1	singlet	2	-1771.401067	-1672.919844	-606.4247254	EMD1
TAACCAGGTATACCTG-1	singlet	1	-6206.888729	-2137.611515	-4761.44407	EMD1
TAACCAGGTGCGTGCT-1	singlet	2	-432.427026	-355.5265665	-131.0693588	EMD1
TAACCAGGTGGATCGA-1	doublet	1/0	-1175.454468	-1053.049932	-1341.276871	EMD1
TAACCAGGTTGCATCA-1	singlet	2	-617.0736033	-539.3043854	-272.3783515	EMD1
TAACCAGTCAGTGGGA-1	singlet	2	-476.9749814	-384.6048564	-192.0263243	EMD1
TAACCAGTCATTTCCA-1	singlet	1	-5647.234021	-1939.419767	-4492.526057	EMD1
TAACCAGTCGAGCCAC-1	singlet	2	-345.9641583	-312.7034005	-159.8686588	EMD1
TAACGACAGCCTGAGA-1	singlet	2	-827.4581448	-752.2128555	-320.5966695	EMD1
TAACGACAGGTCATAA-1	singlet	1	-648.2999742	-246.1540306	-514.325413	EMD1
TAACGACAGTAAACAC-1	singlet	0	-277.952359	-793.3627505	-874.3919878	EMD1
TAACGACCACCGCTAG-1	singlet	2	-348.8316756	-257.6969161	-140.695042	EMD1
TAACGACGTCATGCAT-1	singlet	2	-718.7516818	-566.376094	-307.2548545	EMD1
TAACGACGTCGAACAG-1	singlet	2	-461.9563387	-418.4797969	-177.2342965	EMD1
TAACGACGTGGCTAGA-1	singlet	2	-421.9191535	-317.0603702	-123.0144853	EMD1
TAACGACGTTCAGCTA-1	unassigned	45323	-9.867777117	-2.927526539	-2.806821591	EMD1
TAACGACTCCATCGTC-1	singlet	1	-1265.368662	-328.1453338	-968.8434462	EMD1
TAACGACTCGACTCCT-1	singlet	0	-230.3094248	-734.250953	-625.9216021	EMD1
TAACTTCAGCACACAG-1	singlet	2	-572.7384354	-474.283419	-232.972587	EMD1
TAACTTCCACACAGCC-1	singlet	2	-1417.800206	-1335.81523	-500.0643539	EMD1
TAACTTCCATAAGATG-1	singlet	2	-352.7004272	-302.8772156	-150.4118614	EMD1
TAACTTCCATCCTAAG-1	singlet	2	-525.7237067	-425.8904563	-217.5306167	EMD1
TAACTTCGTATCGCAT-1	singlet	0	-947.7005248	-2830.340773	-2794.255523	EMD1
TAACTTCGTTGTACGT-1	singlet	1	-1192.68006	-253.6929472	-852.12747	EMD1
TAACTTCTCCCTTTGG-1	singlet	2	-1208.080582	-1069.587236	-478.7793022	EMD1
TAAGCACAGGCTCAAG-1	singlet	1	-228.4967125	-101.2578655	-215.5835679	EMD1
TAAGCACCAAATCAAG-1	singlet	2	-397.4097519	-318.519477	-185.4707455	EMD1
TAAGCACCACTGGCCA-1	singlet	2	-294.5184708	-262.4724536	-135.4813132	EMD1
TAAGCACCATGTGCTA-1	singlet	2	-563.8183473	-504.2588916	-218.8703187	EMD1
TAAGCACGTAGCGATG-1	singlet	1	-1031.819795	-398.1265781	-858.9962983	EMD1
TAAGCACGTCAACGCC-1	singlet	0	-118.1496284	-397.394046	-435.8886662	EMD1
TAAGCACGTCTACGTA-1	singlet	2	-4337.138105	-3434.208387	-1413.014797	EMD1
TAAGCACGTGGACTGA-1	singlet	0	-155.8546068	-493.7668708	-418.0377432	EMD1
TAAGCACTCAATCAGC-1	singlet	2	-3119.068076	-2932.652934	-1061.927769	EMD1
TAAGCACTCATAGACC-1	singlet	2	-717.0503669	-800.9077195	-298.7330802	EMD1
TAAGCACTCTACGGTA-1	singlet	1	-1826.469539	-693.7190691	-1510.080276	EMD1
TAAGCCAAGATGAACT-1	singlet	1	-443.8094487	-183.7128369	-403.2005154	EMD1
TAAGCCACAACGCCCA-1	singlet	2	-963.6940274	-765.9957413	-332.3838642	EMD1
TAAGCCACAAGATGTA-1	singlet	0	-1203.198276	-5657.433313	-4694.640893	EMD1
TAAGCCACACATCCCT-1	singlet	1	-676.1770386	-231.0964286	-523.3541094	EMD1
TAAGCCACACGTGTGC-1	singlet	0	-385.5864319	-918.7304917	-935.962607	EMD1
TAAGCCACATGGGTCC-1	singlet	2	-600.1526783	-512.6333666	-226.6503702	EMD1
TAAGCCAGTGTTGATC-1	singlet	1	-2367.287358	-840.1907162	-2040.977454	EMD1
TAAGCCAGTTCGAGCC-1	singlet	2	-696.6580923	-574.6418481	-272.8695218	EMD1
TAAGCCATCAAAGGAT-1	singlet	1	-3142.555928	-907.4556338	-2498.89114	EMD1
TAAGCCATCACTGAAC-1	singlet	2	-3081.661636	-2432.674596	-985.2417499	EMD1
TAAGCCATCGCCAACG-1	singlet	2	-502.6196456	-370.8911576	-192.8451104	EMD1
TAAGCCATCTGATTCT-1	singlet	1	-794.0465487	-326.0005317	-826.2557279	EMD1
TAAGCGTAGCGATGAC-1	doublet	0/1	-30.23971257	-50.9760534	-219.8158763	EMD1

TAAGCGTAGGGACAGG-1	singlet	2	-501.4579022	-390.5862937	-196.6843292	EMD1
TAAGCGTCAGACAAGC-1	singlet	0	-1011.66316	-2884.953788	-2858.945319	EMD1
TAAGCGTCAGCCGGTT-1	singlet	2	-293.1205939	-285.0042002	-102.0955845	EMD1
TAAGCGTCATCGGCCA-1	singlet	0	-127.2503205	-372.6817367	-368.3488997	EMD1
TAAGCGTGTCATATGC-1	singlet	1	-378.3877487	-137.8577718	-313.9219143	EMD1
TAAGCGTGTTGTATGC-1	singlet	2	-1731.234883	-1478.529484	-559.3415975	EMD1
TAAGCGTTCAGCAGAG-1	singlet	0	-176.4743761	-713.2149087	-660.7421448	EMD1
TAAGCGTTCTGTGCAA-1	singlet	2	-93.39640916	-65.13162744	-27.41838513	EMD1
TAAGTCGAGCCTTTCC-1	singlet	2	-644.1833389	-473.4457586	-210.6634873	EMD1
TAAGTCGAGCGGTAAC-1	singlet	0	-133.4358462	-415.8810801	-388.4583902	EMD1
TAAGTCGAGGTAAGAG-1	doublet	0/2	-813.90883	-1313.298366	-1112.057341	EMD1
TAAGTCGGTATCGTGT-1	singlet	2	-1394.681079	-1037.017585	-463.6610965	EMD1
TAAGTCGGTCGGCACT-1	singlet	0	-151.8470328	-435.5924804	-464.2179975	EMD1
TAAGTCGGTCTACGAT-1	singlet	1	-1311.516193	-498.3569087	-1033.912908	EMD1
TAAGTCGGTGGACAGT-1	singlet	0	-288.7668557	-872.6430723	-918.0814756	EMD1
TAATCTCAGTGGTGGT-1	singlet	0	-209.991698	-590.0716012	-671.6726587	EMD1
TAATCTCCACTAACGT-1	singlet	2	-4348.41165	-3875.058974	-1404.252527	EMD1
TAATCTCGTCACCCTT-1	singlet	2	-705.3939027	-601.8248384	-274.9348547	EMD1
TAATCTCGTCATCACA-1	singlet	1	-738.2289625	-279.716507	-629.3028496	EMD1
TAATCTCTCTCACTCG-1	singlet	1	-771.2544499	-272.6197111	-598.531407	EMD1
TAATTCCCAAGAGCTG-1	singlet	0	-742.5856869	-2419.571836	-2501.904208	EMD1
TAATTCCCAAGATCCT-1	singlet	2	-558.5209896	-469.0091429	-209.6934097	EMD1
TAATTCCCAGGAATCG-1	singlet	1	-2049.120108	-704.1790668	-1649.694468	EMD1
TAATTCCGTGGTGATG-1	singlet	0	-628.4097156	-2314.214012	-2124.7685	EMD1
TAATTCCTCGTTATCT-1	singlet	1	-5077.790209	-1553.034019	-3985.563746	EMD1
TACAACGAGATTGCGG-1	singlet	0	-58.97975262	-175.3683495	-198.5318246	EMD1
TACAACGAGCCGATAG-1	singlet	2	-296.8269083	-247.3927017	-121.1633258	EMD1
TACAACGAGCGAAACC-1	singlet	0	-708.2545913	-2345.951927	-2180.180726	EMD1
TACAACGAGTGGATAT-1	singlet	1	-930.009543	-271.0501389	-766.8104019	EMD1
TACAACGCAAGAGGTC-1	singlet	2	-253.3555112	-275.1776504	-123.1455133	EMD1
TACAACGCACCACATA-1	singlet	0	-463.7703269	-1221.10946	-1214.077203	EMD1
TACAACGGTAAGTTAG-1	doublet	0/2	-1014.822213	-1367.752614	-1066.140083	EMD1
TACAACGGTCCGGCAT-1	singlet	1	-1507.075363	-605.6899499	-1286.499639	EMD1
TACAACGGTCGATGCC-1	singlet	2	-347.359998	-388.9125052	-158.3487585	EMD1
TACAACGGTCGTTATG-1	singlet	0	-315.7639351	-932.1594062	-850.6383688	EMD1
TACAACGTCAGTAGGG-1	singlet	0	-244.3816128	-911.1322818	-830.5581168	EMD1
TACACCCAGACGTCCC-1	singlet	1	-550.0667852	-210.5973492	-451.7125646	EMD1
TACACCCAGCCAGTAG-1	singlet	2	-687.0550099	-577.8005303	-272.7326234	EMD1
TACACCCAGCGAGTCA-1	singlet	1	-2802.724585	-844.3893434	-2160.847448	EMD1
TACACCCAGTCCCTAA-1	singlet	0	-1445.926152	-4631.616205	-4561.210737	EMD1
TACACCCCATATACCG-1	singlet	2	-789.1714289	-675.8951465	-286.367929	EMD1
TACACCCCATCATTGG-1	singlet	2	-628.0957105	-521.6419815	-232.7468674	EMD1
TACACCCGTACCCAGC-1	singlet	2	-2265.238821	-1892.741976	-824.0305956	EMD1
TACACCCGTAGATTAG-1	singlet	1	-1501.836023	-655.0900895	-1216.905112	EMD1
TACACCCTCATTGCCC-1	singlet	1	-952.5954382	-311.2721978	-772.0969198	EMD1
TACACCCTCCTCACCA-1	singlet	0	-53.44683568	-204.4831631	-221.4122254	EMD1
TACACCCTCGCCAGAC-1	singlet	2	-1218.236477	-902.9391776	-386.1975473	EMD1
TACACCCTCGCCTCTA-1	singlet	0	-142.1280268	-392.5859214	-382.8584748	EMD1
TACAGGTAGACCTCCG-1	singlet	0	-1010.186627	-4406.007996	-4058.202968	EMD1
TACAGGTAGCACCCAC-1	singlet	1	-126.2903122	-64.57968368	-131.3501829	EMD1
TACAGGTAGGATGAGA-1	singlet	0	-648.5604289	-2175.536421	-2013.922165	EMD1
TACAGGTAGTAGATCA-1	singlet	2	-286.8522418	-211.1617929	-111.5257638	EMD1
TACAGGTCAACACAGG-1	singlet	0	-382.5201584	-1268.702838	-1101.479285	EMD1
TACAGGTCATCAGCGC-1	singlet	1	-2448.716236	-872.5850526	-1879.414501	EMD1
TACAGGTCATGTCAGT-1	singlet	0	-1979.627271	-6036.802724	-5803.955398	EMD1
TACAGGTGTGCGTTTA-1	singlet	1	-2285.152223	-832.8664257	-1907.262167	EMD1
TACAGGTTCGAGTTGT-1	singlet	2	-281.361393	-266.7355901	-117.4885855	EMD1
TACAGGTTCGGCCCAA-1	singlet	0	-65.98930323	-271.8562921	-239.1927368	EMD1
TACATTCAGCCAACCC-1	singlet	2	-335.9896234	-283.2965132	-153.3184717	EMD1
TACATTCAGGTCTTTG-1	singlet	1	-3258.704707	-917.1392222	-2630.851164	EMD1
TACATTCCAACTCGAT-1	singlet	1	-598.8055141	-234.4316419	-522.7857454	EMD1
TACATTCCAAGCACAG-1	singlet	0	-85.04769114	-382.6630779	-312.2399478	EMD1
TACATTCGTACACTCA-1	singlet	0	-132.4638169	-363.9801537	-398.8567775	EMD1
TACATTCGTCAAGTTC-1	singlet	2	-447.6272111	-329.4354538	-155.843707	EMD1
TACATTCGTGTCGATT-1	singlet	0	-437.16605	-1875.165751	-1893.320988	EMD1
TACATTCTCTGGACTA-1	singlet	0	-1413.398849	-3885.239338	-4059.306639	EMD1
TACCCACCAACTTGCA-1	singlet	0	-627.9185479	-1851.73394	-1915.122786	EMD1
TACCCACCAAGCCTGC-1	singlet	2	-1029.87614	-862.5715833	-387.7634065	EMD1
TACCCACCAAGGTACG-1	singlet	1	-819.3574346	-330.0075361	-659.4365414	EMD1
TACCCACGTCAAACGG-1	singlet	0	-255.4380586	-787.1995317	-970.6290397	EMD1
TACCCACTCCTGTAGA-1	singlet	2	-4818.389179	-3767.469209	-1524.796994	EMD1
TACCCACTCTTCCCAG-1	singlet	0	-1462.13402	-4162.346945	-4170.379473	EMD1
TACCCGTAGACGGTTG-1	singlet	0	-136.0044014	-452.445953	-425.3939388	EMD1
TACCCGTAGCATGGGT-1	singlet	2	-811.7202944	-675.3431781	-337.9204182	EMD1
TACCCGTAGGTGGGTT-1	singlet	1	-1858.12863	-699.6743	-1614.675163	EMD1
TACCCGTCACAAGCAG-1	singlet	1	-2431.027032	-825.4447119	-1938.660109	EMD1
TACCCGTCACCCGTAG-1	singlet	2	-397.2726021	-411.241745	-189.517595	EMD1
TACCCGTGTACGACTT-1	singlet	0	-449.3346354	-1195.241372	-1206.206475	EMD1
TACCCGTGTCCCGTGA-1	singlet	1	-1436.713032	-578.2229854	-1164.047731	EMD1
TACCCGTTCGTAACTG-1	singlet	0	-152.5405004	-439.9729386	-413.2486885	EMD1
TACCCGTTCTCAAAGC-1	singlet	2	-393.7442045	-330.0357751	-171.8523458	EMD1
TACCGAAAGACTTGTC-1	singlet	0	-30.36155542	-111.9537729	-86.61440867	EMD1
TACCGAAAGAGAACCC-1	singlet	2	-547.2758941	-494.5121546	-239.8939469	EMD1
TACCGAAAGAGCAGAA-1	singlet	1	-1505.35142	-535.3813267	-1300.399254	EMD1
TACCGAAAGATACTGA-1	singlet	1	-1097.644816	-389.4341362	-855.4760897	EMD1
TACCGAAAGCTGACTT-1	singlet	0	-185.3687784	-770.4816836	-829.273237	EMD1
TACCGAACAATTTCTC-1	singlet	0	-1674.003093	-5471.85643	-5688.233	EMD1
TACCGAAGTAGTCGGA-1	singlet	1	-331.8934253	-142.2872719	-322.053881	EMD1
TACCGAAGTCGCTCGA-1	singlet	1	-1307.205717	-411.6978127	-987.8547359	EMD1
TACCGAAGTGGTTTAC-1	singlet	0	-681.0757986	-2091.010351	-2254.53278	EMD1
TACCGAATCCACATAG-1	singlet	2	-805.6720185	-681.1994384	-301.3975232	EMD1
TACCGAATCCTCACGT-1	singlet	2	-255.5655799	-209.1516264	-111.5672757	EMD1
TACCGAATCGAGGCAA-1	singlet	0	-295.2417864	-705.159888	-706.1461086	EMD1
TACCGGGAGAAGTCCG-1	singlet	0	-241.4326209	-660.4166487	-603.8161967	EMD1
TACCGGGAGCCTATTG-1	singlet	0	-704.1590626	-3047.179959	-2920.454162	EMD1
TACCGGGAGTCGTTAC-1	singlet	1	-246.1030882	-131.2143973	-220.9392545	EMD1
TACCGGGCAACCGGAA-1	doublet	0/2	-1902.474012	-2628.983484	-2200.658522	EMD1
TACCGGGCATCGGTTA-1	singlet	2	-509.3966238	-441.1176811	-202.763263	EMD1
TACCGGGGTAACACGG-1	singlet	2	-368.0864068	-299.9247694	-128.4463451	EMD1
TACCGGGGTACGGCAA-1	singlet	0	-321.6202711	-1383.781316	-1073.39145	EMD1
TACCGGGGTAGCGTAG-1	singlet	2	-1506.252041	-1895.1277	-462.3802841	EMD1
TACCGGGGTTGGGACA-1	singlet	2	-469.1930263	-418.9844911	-208.3629616	EMD1
TACCGGGTCGAACTCA-1	singlet	2	-3417.500432	-3273.197328	-1037.790214	EMD1
TACCGGGTCGCCCAGA-1	singlet	2	-345.6444384	-367.5716709	-160.7806251	EMD1
TACCGGGTCTACGCGG-1	singlet	0	-510.72062	-1449.018628	-1225.164945	EMD1
TACCTCGAGGACAGTC-1	singlet	2	-833.4343515	-619.209829	-285.2226249	EMD1
TACCTCGCAACCACAT-1	doublet	45293	-1581.191036	-967.648716	-1188.807284	EMD1
TACCTCGCAAGAGTTA-1	singlet	1	-1685.324556	-574.17953	-1369.868884	EMD1
TACCTCGCACGCTGTG-1	singlet	2	-622.4833235	-525.0988498	-242.4192141	EMD1
TACCTCGCAGGGATAC-1	singlet	2	-648.4814933	-571.9550403	-308.130042	EMD1

TACCTCGGTAATCAGA-1	singlet	2	-123.9605394	-82.60483491	-36.07917788	EMD1
TACCTCGGTACAGGTG-1	singlet	2	-409.0328757	-374.6305659	-159.1335184	EMD1
TACCTCGGTGATTGGG-1	singlet	1	-124.2156735	-40.98001157	-94.88973292	EMD1
TACCTCGTCATATGGC-1	singlet	1	-523.7075513	-132.1660815	-353.9393388	EMD1
TACCTCGTCCTTCTGG-1	singlet	2	-868.3219803	-724.1094932	-358.5336106	EMD1
TACCTCGTCTTGCGCT-1	singlet	1	-64.60302442	-31.42401441	-50.50846172	EMD1
TACCTGCAGCATTGAA-1	singlet	2	-885.0119965	-692.2689172	-344.0248853	EMD1
TACCTGCAGGGAACAA-1	singlet	0	-27.35685527	-94.4675883	-96.17982472	EMD1
TACCTGCCAAGCTGCC-1	singlet	2	-566.4242176	-467.8323184	-196.6474145	EMD1
TACCTGCCACAAGCTT-1	doublet	45323	-10392.20464	-6782.341652	-5398.639839	EMD1
TACCTGCCAGTCGCAC-1	singlet	1	-14126.43078	-5742.898876	-9847.165265	EMD1
TACCTGCGTACGTGTT-1	singlet	2	-3399.719408	-2720.253003	-1035.775342	EMD1
TACCTGCGTATACCTG-1	doublet	45323	-134.3547089	-102.913756	-63.20238525	EMD1
TACCTGCTCGTTCTGC-1	singlet	2	-309.1326701	-295.5494679	-140.8318916	EMD1
TACGCTCAGGGTACGT-1	singlet	0	-120.5233592	-401.8034685	-360.9355485	EMD1
TACGCTCCACCTGTCT-1	singlet	2	-1514.508504	-1347.391242	-569.8546918	EMD1
TACGCTCCAGTATTCG-1	singlet	2	-196.0416345	-152.9739097	-62.88342469	EMD1
TACGCTCCATGAATAG-1	singlet	0	-1225.048701	-3060.164472	-3103.330744	EMD1
TACGCTCGTACAGCGA-1	singlet	0	-201.3502203	-529.6176558	-537.9155939	EMD1
TACGCTCGTATGAGAT-1	singlet	2	-5179.09899	-4243.671294	-1752.615876	EMD1
TACGCTCGTTCTAAGC-1	singlet	1	-2121.353363	-679.9038929	-1644.082895	EMD1
TACGCTCTCCAATCCC-1	singlet	0	-306.3758695	-1353.748988	-1181.348577	EMD1
TACGCTCTCCTTCAGC-1	singlet	1	-632.8425734	-220.4075727	-573.3982652	EMD1
TACGCTCTCGCACGGT-1	singlet	2	-5436.990583	-4276.838296	-1612.057051	EMD1
TACGCTCTCGCAGTGC-1	singlet	2	-624.9628044	-500.7490134	-256.1849147	EMD1
TACGCTCTCTGCTCTG-1	singlet	1	-1827.468753	-664.386632	-1495.298418	EMD1
TACGGGCCAACTTCTT-1	singlet	0	-358.0445243	-1081.74926	-915.5031524	EMD1
TACGGGCCACTGTCCT-1	singlet	0	-422.5264024	-1250.713652	-1111.218888	EMD1
TACGGGCCAGTGAGCA-1	singlet	1	-928.5568812	-519.8652423	-757.6942288	EMD1
TACGGGCTCAACCTCC-1	unassigned	45323	-10.90818795	-2.579438951	-2.57751044	EMD1
TACGGGCTCCTACAAG-1	singlet	2	-2165.048548	-1718.222765	-735.7883553	EMD1
TACGGGCTCCTTCTGG-1	singlet	1	-1815.470527	-503.3013882	-1566.283482	EMD1
TACGGGCTCGGTAACT-1	singlet	2	-492.3290255	-433.7969144	-184.7404861	EMD1
TACGGTAAGTCAGCCC-1	singlet	1	-297.0962979	-141.8779822	-277.3179913	EMD1
TACGGTACAGTAGATA-1	singlet	0	-574.4376604	-2253.191931	-2464.792651	EMD1
TACGGTACATGACGGA-1	singlet	0	-641.6471712	-1782.010095	-1790.953095	EMD1
TACGGTACATTAAAGG-1	singlet	0	-1912.709575	-6211.700512	-6166.188996	EMD1
TACGGTAGTTAACCTG-1	singlet	0	-122.718922	-490.652467	-419.3481326	EMD1
TACGGTAGTTAGGCCC-1	singlet	2	-577.0846037	-495.8627657	-186.5474081	EMD1
TACGGTAGTTATGTGC-1	singlet	1	-1314.465092	-482.730057	-1012.059176	EMD1
TACGGTATCACAAGAA-1	singlet	2	-2792.254044	-2456.818227	-924.2445307	EMD1
TACGGTATCGTCGCTT-1	singlet	2	-81.05614894	-63.91422268	-26.77880946	EMD1
TACGGTATCTACGGGC-1	singlet	2	-429.9387631	-352.6542101	-169.0416514	EMD1
TACGTCCCAGGTACGA-1	singlet	0	-1163.867341	-3551.256305	-3542.671477	EMD1
TACGTCCGTAAGTCAA-1	singlet	2	-630.944896	-568.3124574	-233.3244941	EMD1
TACGTCCGTGCACAAG-1	singlet	2	-720.6793805	-542.9714177	-225.8263712	EMD1
TACGTCCGTTAAGAAC-1	singlet	2	-610.8337465	-479.1607026	-225.4923971	EMD1
TACGTCCGTTCTTAGG-1	singlet	2	-452.4195433	-405.5209667	-171.2136883	EMD1
TACGTCCTCGTAATGC-1	singlet	1	-850.006799	-322.2356625	-680.7895793	EMD1
TACGTCCTCTACACAG-1	singlet	2	-381.9112021	-332.2608834	-154.4810745	EMD1
TACGTCCTCTGGGATT-1	singlet	0	-711.0824768	-2138.337027	-2027.762298	EMD1
TACTGCCAGGTGCTTT-1	singlet	0	-243.8351145	-672.4119952	-700.236709	EMD1
TACTGCCAGTCGCCCA-1	singlet	1	-6061.711757	-1429.46101	-4811.364855	EMD1
TACTGCCAGTGGAATT-1	singlet	2	-745.324524	-685.2116918	-255.1277722	EMD1
TACTGCCCAAATAGCA-1	singlet	0	-317.8683733	-988.768464	-1043.156372	EMD1
TACTGCCGTAATTGGA-1	singlet	2	-353.2792196	-259.0865474	-148.7337759	EMD1
TACTGCCGTACATACC-1	singlet	1	-6753.879301	-2568.037492	-4879.488007	EMD1
TACTGCCGTGATTCTG-1	singlet	2	-460.1129672	-404.7686574	-164.0292239	EMD1
TACTGCCTCGCTCTCA-1	singlet	2	-481.4328021	-432.1485327	-190.9827446	EMD1
TACTTACAGGGCAAGG-1	singlet	1	-414.9042541	-185.4672645	-410.5518531	EMD1
TACTTACCAATTGCTG-1	singlet	2	-911.0349788	-836.5081948	-350.2511073	EMD1
TACTTACCACGACCTG-1	singlet	1	-3569.27623	-1297.557652	-2891.476217	EMD1
TACTTACCAGAGGTAC-1	singlet	2	-446.7095627	-375.4005163	-172.6262026	EMD1
TACTTACCAGTGACCC-1	singlet	2	-289.9378671	-259.7570165	-109.3217991	EMD1
TACTTACCATCCCACT-1	singlet	1	-743.0850809	-332.2442392	-770.7135527	EMD1
TACTTACGTCGAAGCA-1	singlet	2	-404.5552526	-342.0473651	-141.3230391	EMD1
TACTTACTCAACTTTC-1	singlet	2	-578.5205877	-556.2829993	-265.0638895	EMD1
TACTTACTCGACATAC-1	singlet	2	-1453.0135	-1314.813638	-614.7900187	EMD1
TACTTCACACACCGAC-1	singlet	2	-600.2522153	-571.1388898	-257.6038281	EMD1
TACTTCACACAGACGA-1	doublet	0/2	-779.09242	-1569.648931	-1277.818439	EMD1
TACTTCACAGCAGATG-1	doublet	0/2	-1095.693149	-1606.088452	-1149.303723	EMD1
TACTTCAGTCGGTGTC-1	singlet	1	-3254.807093	-719.6034477	-2740.206019	EMD1
TACTTCAGTCTAGTGT-1	singlet	2	-322.4145785	-224.9942571	-119.4892966	EMD1
TACTTCAGTGCACGCT-1	singlet	0	-2271.384757	-7714.229716	-7322.755899	EMD1
TACTTCAGTGCGAACA-1	singlet	1	-286.3506684	-101.7842049	-189.8644247	EMD1
TACTTCAGTTGGGCCT-1	singlet	1	-3980.910903	-881.3694488	-3237.810124	EMD1
TACTTCATCACAATGC-1	singlet	2	-383.9095461	-323.1435659	-129.3292618	EMD1
TACTTCATCGGCCAAC-1	singlet	1	-951.14511	-233.346852	-763.6339964	EMD1
TACTTGTAGACGGTTG-1	singlet	2	-215.4621023	-166.9637304	-79.21600481	EMD1
TACTTGTAGACTAAGT-1	singlet	1	-1640.301826	-491.3864808	-1280.315025	EMD1
TACTTGTAGACTGTTC-1	singlet	0	-306.0080275	-993.2655424	-838.3163201	EMD1
TACTTGTAGAGGCGGA-1	singlet	0	-47.64289504	-157.1687281	-171.4477725	EMD1
TACTTGTAGGATCATA-1	singlet	1	-492.3235239	-171.781048	-401.1217012	EMD1
TACTTGTAGTAACGTA-1	singlet	2	-451.5403832	-376.3016858	-181.8318556	EMD1
TACTTGTAGTCAACAA-1	singlet	2	-374.5692143	-357.1656344	-163.6003499	EMD1
TACTTGTCAAATTGCC-1	singlet	1	-2951.281982	-1133.482933	-2524.635177	EMD1
TACTTGTCACAATGAA-1	singlet	2	-1553.503519	-1390.21076	-622.260411	EMD1
TACTTGTCACGCTTAA-1	singlet	2	-515.3863493	-497.3455938	-241.7039382	EMD1
TACTTGTCACTAAACC-1	singlet	1	-4930.023712	-1515.352517	-4191.97166	EMD1
TACTTGTCAGCTGAGA-1	singlet	0	-99.43994535	-227.5435716	-300.8737657	EMD1
TACTTGTCAGCTGTGC-1	singlet	2	-652.3223065	-516.1301781	-240.8397539	EMD1
TACTTGTCAGTCGCTG-1	singlet	0	-215.5948807	-566.9732113	-578.7425911	EMD1
TACTTGTGTCTTTCTA-1	singlet	0	-33.18230064	-97.09768223	-80.45958094	EMD1
TACTTGTGTTGACGGA-1	doublet	0/2	-598.984993	-884.1951423	-757.5533028	EMD1
TACTTGTTCCTCAGGG-1	singlet	2	-596.755791	-533.5612068	-267.3685112	EMD1
TACTTGTTCTTCTGTA-1	singlet	2	-959.7630115	-781.2678624	-388.0340631	EMD1
TAGACCACACGAAAGC-1	singlet	1	-4246.60626	-1387.093596	-3374.082779	EMD1
TAGACCAGTACGATCT-1	singlet	2	-595.6338898	-503.2313372	-240.0755248	EMD1
TAGACCAGTCCACGCA-1	singlet	0	-68.62334985	-337.2885497	-290.3052614	EMD1
TAGACTGAGAGGTGCT-1	singlet	2	-361.6723865	-343.5816894	-176.6809276	EMD1
TAGACTGAGCGATTCT-1	singlet	0	-419.4352895	-1157.16305	-1198.129579	EMD1
TAGACTGAGGCGAACT-1	singlet	0	-26.95036305	-81.94930834	-81.28446475	EMD1
TAGACTGCAACAAAGT-1	singlet	1	-2692.383871	-855.5138031	-2103.090261	EMD1
TAGACTGCACGTCGGT-1	singlet	1	-649.9892032	-213.9600623	-600.4058162	EMD1
TAGACTGCACTGGCCA-1	singlet	2	-376.1723437	-323.7659027	-176.5893169	EMD1
TAGACTGCAGCTGTAT-1	singlet	1	-468.1173672	-161.0067897	-471.9275685	EMD1
TAGACTGTCACGTAGT-1	singlet	2	-6473.405987	-4962.377087	-2105.641483	EMD1
TAGACTGTCAGAGTTC-1	singlet	0	-192.5850851	-443.4033598	-579.1028644	EMD1
TAGACTGTCCGGCTTT-1	singlet	2	-2203.239239	-1648.943832	-673.232396	EMD1
TAGAGTCAGACTAAGT-1	singlet	2	-476.6487744	-386.1031076	-196.1499994	EMD1

TAGAGTCCACGCGCAT-1	singlet	2	-251.1968459	-193.0020427	-112.536776	EMD1
TAGAGTCCAGAACGCA-1	singlet	1	-741.4446743	-312.9549601	-830.201123	EMD1
TAGAGTCCAGAACTCT-1	singlet	1	-3499.508327	-1213.400933	-2843.484514	EMD1
TAGAGTCGTTTGGGAG-1	singlet	2	-6067.519417	-4827.210688	-2054.368359	EMD1
TAGAGTCTCTATGTGG-1	singlet	2	-912.279197	-664.1621513	-322.7394501	EMD1
TAGATCGAGAAGTCTA-1	singlet	1	-3192.096412	-1011.711983	-2522.820095	EMD1
TAGATCGAGCGAGAAA-1	singlet	0	-271.1053251	-732.5488177	-902.0023779	EMD1
TAGATCGCAGTCCCGA-1	singlet	0	-334.9220468	-1055.462812	-1081.414055	EMD1
TAGATCGCAGTGTGGA-1	singlet	2	-1208.559443	-1277.184534	-365.7822005	EMD1
TAGATCGGTACTCCCT-1	singlet	0	-448.2919734	-1363.732017	-1233.584962	EMD1
TAGATCGGTTCAAGGG-1	doublet	0/2	-510.8504799	-1056.867362	-918.929508	EMD1
TAGATCGGTTGATGTC-1	singlet	0	-611.6187452	-1897.844803	-1968.926036	EMD1
TAGATCGTCACGGGAA-1	singlet	2	-2041.237486	-1801.774038	-679.7257429	EMD1
TAGATCGTCATTTGCT-1	singlet	2	-365.3370858	-310.4720087	-145.3255897	EMD1
TAGATCGTCCCGAGTG-1	singlet	0	-585.6733654	-1793.795201	-1878.877499	EMD1
TAGATCGTCTAGCATG-1	singlet	0	-920.6935922	-3053.64093	-2767.529661	EMD1
TAGCACAAGATTGATG-1	singlet	2	-1011.529484	-877.9773621	-364.9559483	EMD1
TAGCACAAGCCGCACT-1	singlet	2	-1044.520697	-782.0173149	-347.7059516	EMD1
TAGCACAAGTAAGCAT-1	singlet	0	-645.1275307	-3360.516596	-2778.826027	EMD1
TAGCACACACATATGC-1	singlet	2	-1695.159168	-1940.746985	-543.4321652	EMD1
TAGCACACACCAAATC-1	singlet	2	-1135.514853	-939.9998482	-437.5104119	EMD1
TAGCACACACCTGCGA-1	singlet	0	-246.8588832	-838.0089709	-765.2670575	EMD1
TAGCACACAGAGAATT-1	singlet	0	-523.4315054	-1497.744123	-1446.97199	EMD1
TAGCACATCGCTTACC-1	singlet	2	-497.852164	-393.8557452	-187.8381994	EMD1
TAGCACATCGTCCATC-1	singlet	0	-60.21377356	-175.8090717	-161.7251487	EMD1
TAGCACATCTCAAAGC-1	singlet	1	-1617.560674	-507.4540574	-1385.609668	EMD1
TAGGAGGAGAGAACCC-1	singlet	0	-271.0135303	-1146.184061	-932.6849553	EMD1
TAGGAGGAGGCTGGAT-1	singlet	1	-2812.241171	-637.7565605	-2396.067727	EMD1
TAGGAGGAGTGTGTTC-1	singlet	1	-2497.284853	-757.5645713	-2087.086804	EMD1
TAGGAGGCAACGAGGT-1	singlet	1	-653.294807	-232.367186	-682.0361066	EMD1
TAGGAGGCACCGTACG-1	singlet	0	-1258.154739	-3414.324736	-3268.413324	EMD1
TAGGAGGGTCTGGTTA-1	singlet	2	-283.3566954	-271.9675363	-127.6007597	EMD1
TAGGAGGTCCACAGCG-1	singlet	2	-402.4067846	-390.743973	-183.8556496	EMD1
TAGGAGGTCCCAACTC-1	singlet	2	-185.7450141	-102.76915	-50.9512535	EMD1
TAGGGTTAGTGCGCTC-1	singlet	1	-300.6017673	-110.8793487	-251.5146403	EMD1
TAGGGTTCACAAAGTA-1	singlet	1	-1005.227367	-450.565298	-868.2746275	EMD1
TAGGGTTCACAAGTGG-1	singlet	2	-310.2257538	-214.4582901	-127.3574171	EMD1
TAGGGTTGTTCAAGGG-1	singlet	2	-1446.716556	-1283.156629	-482.5369004	EMD1
TAGGGTTTCATTACGG-1	singlet	0	-19.53337027	-94.31280039	-64.27180324	EMD1
TAGGGTTTCCAGCCTT-1	singlet	0	-740.1277717	-2245.08712	-2288.889647	EMD1
TAGGGTTTCTTTGCTA-1	singlet	0	-395.0750519	-1270.04095	-1099.533704	EMD1
TAGGTACAGGATGGCT-1	singlet	2	-292.8942123	-229.0740484	-121.4776335	EMD1
TAGGTACAGGTAAGAG-1	singlet	0	-257.4803019	-760.5592117	-711.719753	EMD1
TAGGTACCACTAACGT-1	singlet	2	-604.324386	-416.811206	-187.3002576	EMD1
TAGGTACCAGCTGAAG-1	singlet	0	-178.0973478	-448.8756478	-607.3651871	EMD1
TAGGTACGTATTCTCT-1	singlet	0	-931.6773605	-2252.593875	-2324.400721	EMD1
TAGGTACGTATTGACC-1	singlet	0	-1029.582139	-2719.256924	-2329.241507	EMD1
TAGGTACGTGCCGTAC-1	singlet	2	-558.0120523	-446.4794509	-266.115407	EMD1
TAGGTACGTGTCTTAG-1	singlet	0	-207.3105072	-589.6234794	-581.1128625	EMD1
TAGGTACTCACTCCGT-1	singlet	1	-2901.422597	-910.52983	-2261.581161	EMD1
TAGGTACTCAGTCTTT-1	singlet	1	-2192.282897	-808.1392317	-1931.457263	EMD1
TAGGTTGAGAATAGTC-1	singlet	0	-215.9860825	-660.9079138	-567.6753633	EMD1
TAGGTTGAGGGTAATT-1	singlet	1	-2539.725243	-847.0311962	-2043.514429	EMD1
TAGGTTGCACTTGACA-1	singlet	1	-1392.104225	-333.359523	-1344.222331	EMD1
TAGGTTGCAGTCTTCC-1	singlet	0	-151.3272594	-342.4532036	-449.6026604	EMD1
TAGGTTGCATCCTAAG-1	singlet	2	-761.6378772	-684.8330794	-306.7873443	EMD1
TAGGTTGGTCTAACTG-1	singlet	2	-228.3202667	-183.3079513	-82.6789731	EMD1
TAGGTTGGTGATCATC-1	singlet	2	-2363.63388	-2085.292211	-962.0520678	EMD1
TAGGTTGGTGTCTTGA-1	singlet	2	-602.5995717	-495.2517756	-212.2403697	EMD1
TAGGTTGGTTTGTGGT-1	singlet	1	-2757.831893	-660.0553765	-2402.792536	EMD1
TAGGTTGTCACGTAGT-1	singlet	0	-198.1524078	-802.2685185	-993.9478074	EMD1
TAGGTTGTCACTTCTA-1	singlet	2	-388.7670972	-363.8022122	-172.4092724	EMD1
TAGGTTGTCAGAGTTC-1	singlet	2	-488.347067	-445.4092846	-184.5565446	EMD1
TAGGTTGTCAGCAATC-1	singlet	1	-993.0318488	-379.945572	-859.5308417	EMD1
TAGGTTGTCAGGAAAT-1	singlet	2	-446.0282519	-429.7158613	-204.9381946	EMD1
TAGGTTGTCATTATCC-1	singlet	0	-668.5928853	-2293.742895	-2034.241068	EMD1
TAGGTTGTCCTTATAC-1	singlet	0	-417.9919737	-1135.330013	-1063.651152	EMD1
TAGGTTGTCGACCACG-1	singlet	0	-157.4668368	-600.273089	-559.3624233	EMD1
TAGTGCAAGACCTCCG-1	singlet	0	-192.7455438	-661.8272375	-588.2306713	EMD1
TAGTGCAAGGCCACCT-1	singlet	2	-433.1846624	-411.616292	-204.1921146	EMD1
TAGTGCACAGCGTTGC-1	singlet	1	-6875.062166	-1266.058405	-4565.975245	EMD1
TAGTGCAGTGGGTCAA-1	singlet	0	-386.5377157	-1524.577625	-1128.296787	EMD1
TAGTGCAGTGTTGAGG-1	singlet	0	-2458.684176	-7534.217507	-7373.387128	EMD1
TAGTGCAGTTGTCCCT-1	singlet	0	-256.0087569	-622.9145015	-596.2827973	EMD1
TAGTGCATCCATACAG-1	doublet	2/0	-814.466806	-1050.579996	-795.4009654	EMD1
TATACCTAGACTCCGC-1	singlet	0	-1207.374419	-3566.420729	-3419.21683	EMD1
TATACCTAGCAATAAC-1	singlet	2	-598.6871345	-461.2599904	-215.4805602	EMD1
TATACCTCAATGGGTG-1	singlet	1	-869.6551333	-374.4819472	-723.0340633	EMD1
TATACCTCAATTGCGT-1	singlet	2	-690.0081129	-686.0033511	-305.5521588	EMD1
TATACCTCAGGTCCCA-1	singlet	0	-1114.75562	-2758.097605	-2862.452774	EMD1
TATACCTGTCAGTCCG-1	singlet	0	-644.7901969	-1899.349945	-1781.281034	EMD1
TATACCTGTTGCCGCA-1	singlet	0	-1047.254946	-4719.816061	-3853.457668	EMD1
TATACCTGTTTGTTGG-1	singlet	0	-598.6429293	-1802.267164	-1812.201855	EMD1
TATACCTTCATTATCC-1	singlet	2	-496.6321645	-464.8411153	-210.040559	EMD1
TATACCTTCTAAACGC-1	doublet	45293	-111.4401079	-41.45304301	-67.31268298	EMD1
TATACCTTCTCTCAAT-1	singlet	2	-489.203822	-387.1591172	-178.2952968	EMD1
TATATCCAGCAACTTC-1	singlet	1	-2925.254418	-955.4313945	-2427.109391	EMD1
TATATCCCACAAATGA-1	singlet	0	-508.3113395	-1397.666878	-1413.06662	EMD1
TATATCCCACCATATG-1	singlet	0	-102.2749159	-290.8072513	-263.4234901	EMD1
TATATCCGTCAGTCCG-1	singlet	2	-3335.293068	-2623.325831	-1055.130157	EMD1
TATATCCGTGAATGTA-1	singlet	2	-974.6984846	-764.9535412	-375.6206534	EMD1
TATATCCTCAAATGAG-1	singlet	2	-362.4182312	-332.0884332	-153.878668	EMD1
TATATCCTCCAGTACA-1	singlet	2	-367.195487	-321.6563463	-150.2537012	EMD1
TATATCCTCGACCACG-1	doublet	2/0	-556.0172061	-550.9332472	-314.8182935	EMD1
TATATCCTCGCATTGA-1	singlet	1	-3206.842054	-1072.045337	-2490.546835	EMD1
TATCAGGAGCGACTTT-1	singlet	2	-2127.167915	-1863.877057	-714.4854877	EMD1
TATCAGGAGGCCGCTT-1	singlet	0	-306.362253	-705.3977808	-710.0532781	EMD1
TATCAGGAGTATTCCG-1	singlet	0	-449.6195959	-1068.876878	-1086.40168	EMD1
TATCAGGAGTGAACAT-1	singlet	1	-5402.45172	-1355.469904	-4046.023102	EMD1
TATCAGGCAAACCGGA-1	singlet	2	-447.6924678	-340.6177746	-176.3064243	EMD1
TATCAGGGTAAGAACT-1	singlet	2	-2395.960764	-1850.273638	-825.9837719	EMD1
TATCAGGGTCGGTAAG-1	singlet	0	-247.9896724	-773.3637533	-648.7024287	EMD1
TATCAGGGTCTACGAT-1	singlet	2	-537.1033454	-461.0537823	-242.0481132	EMD1
TATCAGGGTTACCTGA-1	singlet	2	-428.3710354	-354.6391352	-156.036421	EMD1
TATCAGGGTTCTCGCT-1	singlet	1	-1994.261172	-828.9924553	-1614.736192	EMD1
TATCAGGTCATGGGAG-1	singlet	2	-602.1600712	-462.7829714	-216.9944483	EMD1
TATCAGGTCATTGTGG-1	singlet	1	-1962.710711	-520.1332186	-1442.100496	EMD1
TATCAGGTCCAGCACG-1	singlet	1	-2445.869262	-674.3366311	-1721.221226	EMD1
TATCAGGTCGGAATTC-1	singlet	2	-1704.437377	-1251.394536	-637.6122292	EMD1
TATCAGGTCTAAGCCA-1	singlet	1	-3792.757745	-1272.398525	-2951.685636	EMD1

TATCCTAAGCGTCAGA-1	doublet	45293	-2070.358911	-925.9212509	-1346.410628	EMD1
TATCCTACAACAGCCC-1	singlet	2	-966.206398	-814.8036808	-360.3416391	EMD1
TATCCTACACTTGTCC-1	singlet	2	-2132.388771	-1753.512922	-774.8419875	EMD1
TATCCTACATAACCCA-1	singlet	2	-708.6267156	-664.762417	-264.4550076	EMD1
TATCCTACATGACAGG-1	singlet	2	-414.9874684	-374.1850224	-200.2614458	EMD1
TATCCTAGTACGATCT-1	singlet	2	-189.6452838	-212.0758558	-98.04468322	EMD1
TATCCTAGTCACCACG-1	singlet	2	-13512.72601	-10634.83727	-4143.341955	EMD1
TATCCTAGTCGATTTG-1	singlet	2	-433.6298481	-347.0920571	-192.0728447	EMD1
TATCCTAGTCTGTTAG-1	singlet	1	-2503.00673	-797.3935052	-1916.677539	EMD1
TATCCTATCAAGATAG-1	singlet	0	-73.41534315	-262.9829313	-245.0267187	EMD1
TATCCTATCCAACTAG-1	singlet	2	-20.52330598	-13.9184938	-7.018891026	EMD1
TATCCTATCCATGATG-1	singlet	2	-443.6366524	-328.2831446	-173.5253027	EMD1
TATCGCCAGGAACGTC-1	singlet	2	-3052.658554	-2351.670375	-850.0208616	EMD1
TATCGCCAGGCCCAAA-1	singlet	2	-4493.978192	-3560.071373	-1602.48679	EMD1
TATCGCCCAACAAGTA-1	singlet	2	-567.1336733	-480.1894749	-234.8154295	EMD1
TATCGCCCATCTATCT-1	singlet	2	-347.5728282	-276.6377322	-108.2121926	EMD1
TATCGCCGTCATCGGC-1	singlet	2	-496.0797921	-479.3895387	-234.9295485	EMD1
TATCGCCGTTGGACCC-1	singlet	0	-300.4257277	-1278.42413	-1147.177761	EMD1
TATCGCCTCATCAGTG-1	singlet	2	-271.2651448	-219.9706958	-103.6838062	EMD1
TATCGCCTCCATTTCA-1	singlet	2	-2172.87902	-1741.239238	-786.7888959	EMD1
TATCGCCTCCCATGGG-1	singlet	2	-389.8044635	-331.6527695	-184.615106	EMD1
TATCGCCTCCGAGGCT-1	singlet	1	-5976.407416	-1712.083626	-5205.520941	EMD1
TATCTGTCAGAAATCA-1	singlet	0	-880.9103325	-2364.285479	-2484.402442	EMD1
TATCTGTCAGTTAGAA-1	singlet	2	-297.1096457	-256.9972225	-128.0565096	EMD1
TATCTGTGTCTGTCCT-1	singlet	1	-984.6763789	-270.7460652	-707.8933147	EMD1
TATCTGTGTGACTCTA-1	doublet	1/0	-10.44569239	-9.879974036	-17.73307131	EMD1
TATCTGTGTGAGATAT-1	singlet	2	-552.5392851	-499.6671451	-213.2406273	EMD1
TATCTGTTCCTGTTAT-1	singlet	0	-690.9212255	-1822.497645	-1970.486548	EMD1
TATCTGTTCTCAATCT-1	singlet	2	-618.3178553	-424.2153082	-227.1073206	EMD1
TATCTTGAGCTGGAGT-1	singlet	2	-2526.440734	-2159.144929	-794.3990782	EMD1
TATCTTGAGGTGGCTA-1	singlet	0	-2718.001914	-7633.666939	-8034.17536	EMD1
TATCTTGCAAGCGGAT-1	singlet	1	-1536.594184	-477.1753954	-1218.71402	EMD1
TATCTTGTCAATCTTC-1	singlet	1	-2335.190049	-833.9454801	-1849.341961	EMD1
TATGTTCAGCATTTCG-1	singlet	0	-205.5450814	-610.3526795	-599.9563463	EMD1
TATGTTCAGCGCTGAA-1	singlet	0	-183.0504183	-487.5456536	-497.0787266	EMD1
TATGTTCCAAGCTGCC-1	singlet	0	-706.3645358	-2121.472735	-2200.647894	EMD1
TATGTTCCACTTCTCG-1	singlet	2	-349.3631007	-300.5388731	-131.5536938	EMD1
TATGTTCGTATGTCCA-1	singlet	0	-248.1929992	-842.5908237	-927.0541108	EMD1
TATGTTCGTTATCTTC-1	singlet	0	-131.5689093	-370.554964	-361.8155333	EMD1
TATGTTCTCCGAAATC-1	singlet	1	-576.6451699	-236.1034541	-609.5153466	EMD1
TATGTTCTCTCTGAGA-1	singlet	1	-180.862117	-71.21796354	-155.6865069	EMD1
TATTCCAAGAGTGTGC-1	singlet	2	-359.6125687	-292.3586704	-141.5906183	EMD1
TATTCCAAGATAGCTA-1	singlet	2	-550.3954189	-413.5930812	-193.7655965	EMD1
TATTCCACACGACAAG-1	singlet	0	-449.4246664	-1360.306996	-1383.564033	EMD1
TATTCCACATGTTTGG-1	singlet	1	-2599.638425	-966.3124572	-2282.240598	EMD1
TATTCCACATTACGGT-1	singlet	2	-521.5348437	-445.8501483	-262.4649135	EMD1
TATTCCAGTCCACTCT-1	singlet	2	-358.7847249	-299.4418937	-146.6064712	EMD1
TATTCCAGTTAAACCC-1	singlet	2	-18030.46171	-14340.84261	-6073.591728	EMD1
TATTCCATCAAGCCGC-1	singlet	1	-2150.759738	-863.7517957	-1835.827553	EMD1
TATTCCATCATCCTGC-1	singlet	2	-576.107974	-479.2368818	-223.3591673	EMD1
TATTCCATCCATATGG-1	singlet	2	-550.2735461	-513.3371699	-226.2416029	EMD1
TATTCCATCCCGTTCA-1	doublet	1/0	-4413.771941	-2946.08659	-5804.240474	EMD1
TATTGCTAGCGACTAG-1	singlet	2	-1641.230259	-1370.81299	-648.9980992	EMD1
TATTGCTAGGTGAGAA-1	singlet	0	-169.5035261	-399.4800278	-442.8272602	EMD1
TATTGCTAGTGGAATT-1	singlet	0	-158.0661168	-503.6051863	-499.9261825	EMD1
TATTGCTCATACCAGT-1	singlet	0	-665.1281232	-1982.264764	-1804.28656	EMD1
TATTGCTCATTGTCGA-1	singlet	2	-2329.873999	-1764.445132	-693.1550468	EMD1
TATTGCTGTGATACCT-1	singlet	0	-1376.447814	-4217.166264	-4294.708643	EMD1
TATTGCTGTGGACTGA-1	singlet	2	-569.8295139	-493.4647135	-218.5897567	EMD1
TATTGCTTCAGGAGAC-1	singlet	2	-518.5635625	-489.250847	-208.540253	EMD1
TATTGCTTCATGAGAA-1	singlet	2	-416.1247372	-297.7927636	-134.7786807	EMD1
TATTGCTTCCATGCAA-1	singlet	2	-2383.898497	-1985.667378	-862.5455936	EMD1
TATTGCTTCTGCACCT-1	singlet	2	-1865.116858	-1567.410007	-656.6726947	EMD1
TATTGGGAGGAATGTT-1	singlet	2	-509.0699194	-474.1060112	-225.9840943	EMD1
TATTGGGCAACAGTGG-1	singlet	2	-485.8311954	-368.6623405	-171.8243064	EMD1
TATTGGGCAAGCGCAA-1	singlet	1	-1596.790659	-494.8129397	-1340.385712	EMD1
TATTGGGCAGAACTCT-1	singlet	0	-103.8206562	-280.6835991	-320.2115748	EMD1
TATTGGGGTCAGTCCG-1	singlet	2	-1639.720171	-1301.482087	-611.9508435	EMD1
TATTGGGTCAGCCCAG-1	singlet	2	-515.4358168	-460.3823266	-221.4587234	EMD1
TATTGGGTCTCAGAAC-1	singlet	1	-2802.448949	-1257.574516	-2075.51317	EMD1
TATTGGGTCTCTCAAT-1	singlet	1	-1605.750453	-511.8217689	-1349.334158	EMD1
TATTGGGTCTGTCTCG-1	singlet	0	-754.8839266	-2213.947803	-2101.202519	EMD1
TATTTCGAGAATAACC-1	singlet	2	-513.6732884	-427.7583941	-115.1790646	EMD1
TATTTCGAGTAGTCCT-1	singlet	0	-791.0806962	-2622.043027	-2610.059458	EMD1
TATTTCGCAGGAATCG-1	singlet	2	-312.9830547	-236.4649417	-115.0331424	EMD1
TATTTCGCATGGGCAA-1	singlet	2	-420.7589307	-338.7639898	-190.1173442	EMD1
TATTTCGGTAGTTACC-1	singlet	2	-382.1343178	-352.5058556	-149.3175658	EMD1
TATTTCGGTCACTCGG-1	singlet	2	-9098.149247	-7374.930508	-2977.317362	EMD1
TATTTCGGTGACTATC-1	singlet	0	-759.3903266	-2212.579442	-1995.110554	EMD1
TATTTCGGTGCACGCT-1	singlet	2	-2389.244654	-1888.532851	-816.4954335	EMD1
TATTTCGGTTGGTAGG-1	singlet	2	-465.4956862	-356.8769954	-177.1379979	EMD1
TATTTCGTCGAAGCCC-1	singlet	2	-510.0876395	-428.3669031	-221.0476972	EMD1
TATTTCGTCGGAAGGT-1	singlet	2	-312.7094415	-262.2988662	-111.5890297	EMD1
TCAAGACAGAATTGCA-1	singlet	1	-5638.991537	-1906.01447	-4464.110947	EMD1
TCAAGACAGGAAGTAG-1	singlet	0	-298.8100529	-1227.639953	-1072.924141	EMD1
TCAAGACAGTCGGGAT-1	singlet	0	-350.8548888	-1043.068405	-896.2381572	EMD1
TCAAGACCAATTTCCT-1	singlet	1	-1719.085659	-676.1370937	-1446.913543	EMD1
TCAAGACGTCACCTTC-1	singlet	1	-1563.741955	-487.1349812	-1158.460855	EMD1
TCAAGACGTGTCACAT-1	singlet	2	-362.5627973	-358.139451	-124.8251692	EMD1
TCAAGCAAGGAGCTGT-1	singlet	0	-275.0092384	-853.8436758	-790.7930565	EMD1
TCAAGCACAAATACGA-1	singlet	1	-248.5336955	-128.6269796	-268.804259	EMD1
TCAAGCACACCCTATC-1	singlet	2	-1354.764253	-1215.439682	-586.7257944	EMD1
TCAAGCACATCCTTGC-1	singlet	1	-3098.966924	-1005.315136	-2486.602928	EMD1
TCAAGCAGTACGGGAT-1	singlet	2	-443.0360009	-472.3822004	-200.0792351	EMD1
TCAAGCAGTCCAAAGG-1	singlet	2	-585.9821815	-501.3588989	-242.2429821	EMD1
TCAAGCAGTGTACAGG-1	singlet	2	-429.8312706	-326.9149854	-178.0865987	EMD1
TCAAGCAGTTCAAACC-1	singlet	1	-274.9463891	-88.57752448	-228.5663111	EMD1
TCAAGCAGTTCACCGG-1	singlet	2	-3882.055762	-3358.968537	-1446.529041	EMD1
TCAAGCATCATCCTAT-1	singlet	1	-4284.698937	-1049.042569	-3572.109339	EMD1
TCAAGCATCGATACTG-1	singlet	1	-1249.250597	-477.9451551	-957.6984427	EMD1
TCAAGTGAGAGCTGCA-1	singlet	1	-1339.165532	-503.5461971	-1036.022671	EMD1
TCAAGTGAGTAACAGT-1	singlet	0	-2910.339594	-10519.49736	-10364.27021	EMD1
TCAAGTGAGTATCTGC-1	singlet	0	-24.08276113	-86.13048648	-56.56678618	EMD1
TCAAGTGCAAGACCTT-1	singlet	1	-1030.907055	-347.5858736	-788.2458325	EMD1
TCAAGTGTCTGCTTAT-1	singlet	2	-613.3218334	-559.3751817	-254.6113087	EMD1
TCAAGTGTCTTACCGC-1	singlet	1	-690.7873576	-256.3033061	-503.5284203	EMD1
TCAATCTAGCTCGCAC-1	singlet	2	-1681.372763	-1524.752158	-634.0128217	EMD1
TCAATCTAGTCCTACA-1	singlet	0	-1756.840815	-6745.181128	-5822.373561	EMD1
TCAATCTGTTGGGACA-1	singlet	2	-5173.184478	-4224.660068	-1707.01329	EMD1
TCAATCTTCGATCCAA-1	singlet	2	-4581.560562	-3673.46123	-1637.997185	EMD1

TCAATCTTCGGTAGGA-1	singlet	0	-663.4059321	-2167.984024	-2144.525404	EMD1
TCAATCTTCTCCTGCA-1	singlet	2	-596.2964954	-529.1870908	-263.3132603	EMD1
TCAATTCAGATTGACA-1	singlet	2	-9599.530409	-7437.63566	-2830.885265	EMD1
TCAATTCAGCTTGTTG-1	singlet	0	-82.48457402	-216.223455	-214.7104339	EMD1
TCAATTCAGGCAGCTA-1	singlet	1	-1023.0689	-380.8699207	-791.5932401	EMD1
TCAATTCAGGTCATAA-1	singlet	2	-471.7502001	-452.8227001	-208.2362276	EMD1
TCAATTCGTAGTCGTT-1	singlet	2	-195.7592191	-239.4438097	-82.69651587	EMD1
TCAATTCGTCCATCTC-1	singlet	2	-813.4551687	-674.9136702	-326.5885082	EMD1
TCAATTCTCGACGACC-1	singlet	2	-345.2624214	-340.1476033	-155.1480223	EMD1
TCACAAGAGACAGCGT-1	singlet	0	-935.347569	-2902.768682	-3147.982189	EMD1
TCACAAGAGTGGTGAC-1	singlet	1	-5257.899844	-1527.871161	-4198.0504	EMD1
TCACAAGCACAAAGCG-1	singlet	0	-56.22047263	-165.7671015	-175.2391156	EMD1
TCACAAGCACCCATAA-1	singlet	2	-603.6204612	-544.5230955	-249.8608469	EMD1
TCACAAGCAGCCGTTG-1	singlet	2	-1378.213179	-1312.538288	-492.5207989	EMD1
TCACAAGCATCCCACT-1	singlet	2	-2630.150586	-2143.654991	-950.0702061	EMD1
TCACAAGGTTCACCGG-1	singlet	2	-3393.074042	-2708.869358	-1133.78515	EMD1
TCACAAGTCCCGATCT-1	singlet	0	-253.6898768	-756.7624584	-905.8800892	EMD1
TCACAAGTCGTCGGGT-1	singlet	0	-664.8392497	-2050.557568	-1942.470612	EMD1
TCACACCAGTATAGGT-1	singlet	1	-3296.902793	-1108.221986	-2622.039212	EMD1
TCACACCCAGCAGGAT-1	singlet	0	-1203.979922	-4782.009383	-4732.484345	EMD1
TCACACCCAGTCGGTC-1	singlet	2	-100.3990267	-72.17133497	-27.98637145	EMD1
TCACACCGTAGCGTCC-1	singlet	0	-380.5063084	-944.4969564	-861.5621062	EMD1
TCACATTAGACCTTTG-1	singlet	0	-505.3432977	-1611.984043	-1416.775766	EMD1
TCACATTAGCTGCCTG-1	singlet	2	-450.2586375	-328.4890111	-136.8637205	EMD1
TCACATTAGCTGTTCA-1	singlet	1	-1251.477662	-510.0751502	-1037.184034	EMD1
TCACATTGTACCTAAC-1	singlet	0	-174.5710387	-459.8959393	-549.8107811	EMD1
TCACATTGTTCAACGT-1	singlet	0	-3019.601138	-10611.18134	-10225.26057	EMD1
TCACATTGTTGAGTCT-1	singlet	2	-4161.474651	-3199.531659	-1383.633477	EMD1
TCACATTTCCTGGTCT-1	singlet	0	-49.56697836	-149.8757772	-109.4108522	EMD1
TCACGCTAGATCCAAA-1	singlet	1	-3122.836224	-944.452532	-2263.020158	EMD1
TCACGCTAGCCTCAGC-1	singlet	2	-976.4477184	-859.8175306	-420.5682569	EMD1
TCACGCTAGCCTCGTG-1	singlet	2	-338.5448779	-301.851158	-152.5207222	EMD1
TCACGCTAGTGCGACA-1	singlet	0	-211.1052011	-573.933427	-566.3294512	EMD1
TCACGCTCACAGGATG-1	singlet	0	-443.4901186	-1152.559386	-1145.264475	EMD1
TCACGCTGTAATGCTC-1	singlet	1	-11195.49958	-2733.643589	-8616.166427	EMD1
TCACGCTGTCTGCCTT-1	singlet	1	-2211.003079	-684.584199	-1719.433411	EMD1
TCACGCTTCACCGGTG-1	singlet	2	-788.8474492	-696.2839805	-304.3479719	EMD1
TCACGGGCACACGGTC-1	singlet	2	-696.0191351	-536.9600034	-295.3186473	EMD1
TCACGGGCACCAGCTG-1	singlet	0	-399.2320079	-1105.018243	-1224.983014	EMD1
TCACGGGCACCTAAAC-1	singlet	0	-644.1499178	-2599.187229	-2336.968777	EMD1
TCACGGGCATCCTATT-1	singlet	1	-21652.92352	-6705.379317	-17314.26235	EMD1
TCACGGGGTAACACGG-1	singlet	2	-741.460549	-537.9448536	-313.4054622	EMD1
TCACGGGGTGGTCTTA-1	singlet	2	-2653.181666	-2153.99249	-955.6065551	EMD1
TCACGGGGTGTGAATA-1	singlet	2	-387.1385027	-311.3695847	-122.4415578	EMD1
TCACGGGGTTTAGACC-1	singlet	2	-3804.839044	-3813.471163	-1078.176775	EMD1
TCACGGGTCATAGAGA-1	singlet	2	-556.9925046	-431.7742895	-171.0982627	EMD1
TCACGGGTCCTACGAA-1	singlet	1	-2308.13939	-754.7630745	-1704.033042	EMD1
TCACGGGTCTACCACC-1	singlet	2	-443.1126508	-402.2226626	-177.0292466	EMD1
TCACGGGTCTTTGGAG-1	singlet	2	-336.5648052	-360.1088172	-163.4013736	EMD1
TCACTATAGCTATCCA-1	singlet	1	-2722.756821	-921.0402364	-2217.736085	EMD1
TCACTATAGGGCTTCC-1	singlet	2	-724.2338872	-585.9499892	-268.129245	EMD1
TCACTATAGGTTACAA-1	singlet	0	-1333.465442	-3590.873583	-3547.076895	EMD1
TCACTATCAACAGCTT-1	singlet	2	-613.2226603	-566.2916553	-251.7720239	EMD1
TCACTATCACACTGGC-1	doublet	45323	-935.3529944	-644.3138131	-365.1271177	EMD1
TCACTATTCATACGGT-1	singlet	2	-569.182294	-454.705073	-237.2264154	EMD1
TCACTATTCGATTTCT-1	singlet	0	-406.6615986	-1162.396645	-1306.989781	EMD1
TCACTATTCGCCGAGT-1	singlet	2	-407.8894067	-355.1488602	-147.396481	EMD1
TCACTATTCGCTACGG-1	singlet	1	-6.939390488	-3.231340274	-6.219613305	EMD1
TCACTATTCTAGCCTC-1	singlet	2	-4639.215505	-3804.352561	-1619.169514	EMD1
TCACTATTCTATCCAT-1	singlet	2	-3684.380212	-2961.574049	-1276.424805	EMD1
TCACTATTCTTCCAGC-1	singlet	2	-2454.190661	-1860.90472	-775.1379898	EMD1
TCACTCGAGGAATCGC-1	singlet	2	-915.0338993	-813.5661781	-356.829161	EMD1
TCACTCGCACTGCATA-1	singlet	2	-774.2179285	-652.056086	-250.9270934	EMD1
TCACTCGGTGCATTTG-1	singlet	0	-193.0538594	-490.3218158	-615.7725402	EMD1
TCACTCGTCGACACTA-1	singlet	2	-76.96540571	-74.5991279	-20.75131447	EMD1
TCACTCGTCTTTCTAG-1	singlet	0	-1063.646168	-2937.286272	-2867.301921	EMD1
TCAGCAAAGAAGGATG-1	singlet	2	-5500.72967	-4379.077746	-1892.078231	EMD1
TCAGCAAAGACGTCCC-1	doublet	2/0	-360.9288924	-391.0687691	-249.8548111	EMD1
TCAGCAAAGTTCACTG-1	singlet	0	-2325.776686	-7271.063109	-7460.016079	EMD1
TCAGCAACAGACCCGT-1	singlet	0	-628.3900754	-1732.262331	-1693.760898	EMD1
TCAGCAACAGAGACTG-1	singlet	2	-5146.93477	-4092.909039	-1366.276864	EMD1
TCAGCAACAGCACGAA-1	singlet	2	-499.3450978	-414.5091951	-192.1868558	EMD1
TCAGCAACAGCATCTA-1	singlet	0	-237.0295539	-847.7735799	-758.6472373	EMD1
TCAGCAAGTCACAGTT-1	singlet	2	-422.1792314	-375.009703	-181.9010185	EMD1
TCAGCAAGTGATGTAA-1	singlet	1	-131.6964789	-65.95246627	-136.6494752	EMD1
TCAGCAAGTTCACGAT-1	singlet	2	-343.3386811	-334.7427295	-151.5619821	EMD1
TCAGCAATCTCATTAC-1	singlet	2	-624.8272479	-512.9411875	-244.1890436	EMD1
TCAGCCTAGATCACTC-1	singlet	1	-603.826148	-250.5040685	-609.2023015	EMD1
TCAGCCTAGATGGCGT-1	singlet	2	-365.3390819	-282.3549988	-125.8089611	EMD1
TCAGCCTAGATTGTGA-1	singlet	1	-3641.203681	-607.4262146	-3083.499175	EMD1
TCAGCCTAGTGATCGG-1	singlet	2	-515.8498385	-423.9587696	-202.866378	EMD1
TCAGCCTCATCTGTTT-1	singlet	0	-68.30818474	-186.201734	-169.4022281	EMD1
TCAGCCTGTAACGGTG-1	singlet	1	-2263.030034	-749.315908	-1542.250636	EMD1
TCAGCCTGTGCTAGCC-1	singlet	1	-728.9094123	-237.7343723	-586.1007537	EMD1
TCAGGGCAGGAGATAG-1	singlet	1	-814.4561947	-328.5167378	-714.6790283	EMD1
TCAGGGCAGTGCACCC-1	unassigned	2/0	-249.0612672	-202.7107871	-127.0529317	EMD1
TCAGGGCAGTTTGAGA-1	singlet	2	-575.596891	-481.8856297	-246.8101355	EMD1
TCAGGGCCACTTGAGT-1	singlet	2	-405.7876676	-340.5173529	-185.6112337	EMD1
TCAGGGCCAGTAGATA-1	singlet	2	-438.4474371	-349.3934563	-160.3798786	EMD1
TCAGGGCCAGTCAGCC-1	singlet	2	-344.9970337	-289.0562036	-113.3870586	EMD1
TCAGGGCCATCATCCC-1	singlet	2	-806.4182546	-643.2204605	-262.0731239	EMD1
TCAGGGCGTCGCAACC-1	singlet	2	-292.6006985	-288.8066285	-115.0226615	EMD1
TCAGGGCGTGGCAGAT-1	singlet	0	-777.9652462	-2090.896153	-1797.806465	EMD1
TCAGGGCGTTCATCGA-1	singlet	2	-1291.772376	-1362.485086	-395.9495202	EMD1
TCAGGGCTCTAGCCAA-1	singlet	0	-80.42061448	-243.1590398	-214.9045348	EMD1
TCAGGGCTCTGCAGCG-1	singlet	2	-858.4524415	-712.8902793	-308.8217308	EMD1
TCAGGTAAGACAGCGT-1	singlet	0	-101.2609105	-383.2752713	-319.4271429	EMD1
TCAGGTACAGCCCACA-1	singlet	1	-4737.06434	-1473.717313	-3653.74823	EMD1
TCAGGTACAGGCGAAT-1	singlet	0	-882.4377196	-2409.193625	-2291.686567	EMD1
TCAGGTACATCCGGCA-1	singlet	2	-520.5546316	-426.2003698	-219.1887416	EMD1
TCAGGTAGTAGAATAC-1	singlet	2	-473.1413006	-414.5552856	-190.8823415	EMD1
TCAGGTATCCAAGCTA-1	singlet	0	-644.0976286	-2006.913181	-2047.485249	EMD1
TCAGTCCAGAAGATCT-1	singlet	1	-1850.017872	-620.2404674	-1498.047747	EMD1
TCAGTCCAGCCATTGT-1	singlet	2	-897.4825739	-683.8954447	-317.369516	EMD1
TCAGTCCAGCGTTAGG-1	singlet	2	-628.7859767	-508.9626446	-230.34632	EMD1
TCAGTCCAGGCTCTAT-1	singlet	2	-434.1273393	-421.3923256	-160.5776834	EMD1
TCAGTCCGTGCTGATT-1	singlet	0	-214.9105877	-616.2315574	-598.2506626	EMD1
TCAGTCCGTGTCTAAC-1	singlet	0	-306.5484985	-1020.170115	-977.7806969	EMD1
TCAGTCCGTTATAGCC-1	singlet	0	-325.7161041	-984.388531	-910.4918391	EMD1
TCAGTCCGTTGGGTTT-1	singlet	2	-513.7337677	-427.1490411	-176.0663077	EMD1

TCAGTCCGTTGTCCCT-1	singlet	0	-92.01027188	-191.7709659	-170.560904	EMD1
TCAGTCCTCCACAGCG-1	singlet	2	-349.5534108	-292.3000658	-135.5170307	EMD1
TCAGTCCTCGGATTAC-1	singlet	2	-1251.611158	-1019.208255	-448.1090974	EMD1
TCAGTCCTCGTGCTCT-1	singlet	2	-507.3320252	-453.6947696	-212.955508	EMD1
TCAGTCCTCTGAGGTT-1	singlet	2	-112.1366804	-92.36170183	-34.991647	EMD1
TCAGTGAAGCGCACAA-1	singlet	1	-65.65731696	-12.53957445	-54.89321241	EMD1
TCAGTGACAGATAAAC-1	singlet	2	-264.4880358	-240.8532945	-102.6256915	EMD1
TCAGTGACAGCTTCGG-1	singlet	2	-414.9376765	-340.3011112	-177.3669396	EMD1
TCAGTGAGTAATCAAG-1	singlet	2	-2697.432811	-2303.638454	-992.3870482	EMD1
TCAGTGAGTGATACAA-1	singlet	2	-683.8927576	-592.1825721	-271.0264503	EMD1
TCAGTGAGTGTAAACA-1	singlet	2	-2175.535225	-1542.062683	-615.5684995	EMD1
TCAGTGAGTTGCTGAT-1	singlet	2	-442.9094954	-430.2156614	-199.6922734	EMD1
TCAGTGATCACCATGA-1	singlet	2	-277.0072811	-251.0701603	-91.98529571	EMD1
TCAGTGATCGGAGTAG-1	singlet	2	-394.6622828	-340.4256019	-161.3011864	EMD1
TCAGTTTAGCTTTCCC-1	singlet	2	-8869.702283	-7117.984597	-2827.774537	EMD1
TCAGTTTCAGACCTGC-1	singlet	2	-2846.252824	-2225.489463	-872.6665454	EMD1
TCAGTTTGTCTGCGCA-1	singlet	0	-549.7571148	-1896.978083	-1610.774297	EMD1
TCAGTTTGTTGCTTGA-1	singlet	0	-566.8876588	-1659.564869	-1515.652096	EMD1
TCAGTTTTCATGCATG-1	singlet	2	-602.3004026	-502.3970383	-227.2658902	EMD1
TCAGTTTTCCACAAGT-1	singlet	0	-298.3676118	-950.9344178	-829.0295827	EMD1
TCAGTTTTCTCGTGGG-1	singlet	2	-11783.66113	-9448.312448	-3577.630937	EMD1
TCATACTAGAGTCACG-1	singlet	1	-1891.34894	-591.5746901	-1485.505905	EMD1
TCATACTAGGTAAGAG-1	singlet	0	-274.457649	-912.8866501	-792.7174732	EMD1
TCATACTAGGTCGTCC-1	singlet	2	-581.7740488	-450.4451796	-222.5041197	EMD1
TCATACTCAACTGAAA-1	singlet	2	-546.8974262	-471.6676482	-213.7172664	EMD1
TCATACTCACATTCTT-1	singlet	2	-388.4822238	-344.7936588	-163.1592435	EMD1
TCATACTCAGGTACGA-1	singlet	2	-579.8562278	-456.4334439	-193.0148098	EMD1
TCATACTGTGACCGAA-1	singlet	0	-2156.031253	-4619.033352	-4196.198565	EMD1
TCATACTGTTCATCGA-1	singlet	0	-179.5906835	-571.0138654	-622.0282827	EMD1
TCATACTGTTGCATCA-1	singlet	2	-574.3213664	-476.1932166	-228.3926914	EMD1
TCATACTGTTTCTTAC-1	singlet	0	-557.2731229	-1977.844666	-1786.475571	EMD1
TCATACTTCATGAGGG-1	singlet	1	-1232.710324	-421.4678931	-847.2648123	EMD1
TCATACTTCATTTCGT-1	singlet	0	-292.9229615	-1215.459258	-1003.712291	EMD1
TCATACTTCCATACAG-1	singlet	2	-4507.559621	-3713.781677	-1709.487925	EMD1
TCATACTTCGCTTGAA-1	singlet	1	-249.0750023	-72.92975555	-145.081937	EMD1
TCATACTTCGGCCAAC-1	singlet	2	-1112.269489	-1029.426105	-329.2841027	EMD1
TCATATCAGATACGAT-1	singlet	0	-744.9881714	-2154.048657	-2207.278669	EMD1
TCATATCAGTATGACA-1	singlet	0	-283.9272424	-922.6499328	-1174.405564	EMD1
TCATATCAGTGGTTGG-1	singlet	0	-583.7396596	-1697.744755	-1587.164594	EMD1
TCATATCCAGGGTCTC-1	singlet	2	-543.3252291	-409.0072259	-215.5082251	EMD1
TCATATCGTCTAGGCC-1	singlet	1	-9192.513795	-3440.623922	-8175.750931	EMD1
TCATATCGTGTGAGCA-1	singlet	1	-93.3188893	-33.17313091	-79.18354713	EMD1
TCATATCGTTGCACGC-1	singlet	1	-1360.20085	-487.5745414	-978.0196257	EMD1
TCATATCGTTGTGTTG-1	singlet	2	-399.6983978	-359.2569545	-187.2890204	EMD1
TCATATCTCACTTGGA-1	singlet	2	-817.0871654	-657.3321897	-304.9222699	EMD1
TCATATCTCGAAGGAC-1	singlet	2	-3288.323311	-2730.070428	-1215.986913	EMD1
TCATATCTCGACCATA-1	singlet	0	-647.3684597	-2106.298048	-1904.675078	EMD1
TCATCATAGACGGAAA-1	singlet	2	-633.7546707	-523.328511	-251.0608755	EMD1
TCATCATAGGAACTAT-1	singlet	0	-956.1293794	-2837.208053	-2551.401153	EMD1
TCATCATAGGGTGGGA-1	singlet	2	-2302.961366	-2103.131008	-860.0824809	EMD1
TCATCATAGTCTTCGA-1	singlet	2	-590.2357765	-466.2411866	-213.1903693	EMD1
TCATCATAGTTCATCG-1	singlet	2	-2290.920013	-1872.788771	-687.3091446	EMD1
TCATCATCAGCGGATA-1	singlet	1	-3073.640675	-835.8294359	-2496.694977	EMD1
TCATCATGTGCTCTCT-1	singlet	2	-448.0024794	-349.9954556	-163.3364977	EMD1
TCATCATGTTTACTGG-1	singlet	0	-1339.67293	-3835.04091	-3859.573936	EMD1
TCATCCGCAACAGCTT-1	singlet	2	-8580.97189	-6785.44273	-2838.337944	EMD1
TCATCCGCAATCGTCA-1	singlet	2	-419.5135146	-382.56668	-170.111853	EMD1
TCATCCGCACCGAATT-1	singlet	2	-757.5217357	-699.4828454	-336.2997303	EMD1
TCATCCGGTCACTTAG-1	singlet	2	-2678.900551	-2576.252357	-899.9790421	EMD1
TCATCCGGTGAGCGAT-1	singlet	2	-28.31974981	-26.48797306	-5.384918019	EMD1
TCATCCGTCAATCTTC-1	singlet	2	-282.7118338	-220.4889525	-111.3114412	EMD1
TCATCCGTCGTTAGTG-1	singlet	2	-915.8672537	-892.3442306	-266.5493964	EMD1
TCATGAGAGAGCGACT-1	singlet	0	-583.0415106	-1713.261015	-1741.682588	EMD1
TCATGAGAGATGGCGT-1	singlet	0	-35.84842077	-121.9083475	-164.2567553	EMD1
TCATGAGAGCCATCCG-1	singlet	1	-63.17908478	-35.18516475	-53.46189388	EMD1
TCATGAGCACAATTCG-1	singlet	2	-2090.946412	-1761.289675	-764.9519714	EMD1
TCATGAGCATACCGTA-1	singlet	2	-434.4510242	-424.2850985	-158.8743852	EMD1
TCATGAGTCTAATTCC-1	singlet	2	-183.970158	-157.1704426	-57.1531727	EMD1
TCATGAGTCTTGGTCC-1	singlet	2	-400.5795454	-375.6148834	-152.6457353	EMD1
TCATGCCAGTGTTCCA-1	singlet	1	-455.5853585	-160.9337448	-477.0916203	EMD1
TCATGCCGTAGCCAGA-1	singlet	0	-23.64959122	-91.15818924	-77.34141855	EMD1
TCATGCCGTCACGCTG-1	doublet	45323	-3635.673873	-2190.158863	-2061.510776	EMD1
TCATGCCGTCTAACTG-1	singlet	0	-464.8011861	-1403.015417	-1192.421254	EMD1
TCATGCCTCGCCCAGA-1	singlet	0	-271.0746129	-771.6665399	-865.0258169	EMD1
TCATGCCTCGCTAAAC-1	singlet	2	-1566.75431	-1344.56203	-598.4533567	EMD1
TCATGCCTCGCTGCGA-1	singlet	1	-2639.796575	-662.3595231	-2047.350939	EMD1
TCATGGAAGACGCTCC-1	singlet	2	-296.6060042	-229.8415561	-99.88813379	EMD1
TCATGGAAGAGGTGCT-1	singlet	2	-352.1666376	-267.787946	-147.269334	EMD1
TCATGGAAGGCTAAAT-1	singlet	0	-418.5770097	-1605.720492	-1382.565704	EMD1
TCATGGAAGTAAACAC-1	singlet	0	-2626.057206	-9077.833567	-8357.866218	EMD1
TCATGGACAATTCTCT-1	singlet	1	-170.2016919	-57.17454539	-168.4350664	EMD1
TCATGGACACCCATAA-1	singlet	1	-3406.08431	-915.3876034	-2452.97857	EMD1
TCATGGAGTATCACGT-1	singlet	1	-4770.86438	-1255.053011	-3792.711973	EMD1
TCATGGAGTGCATTAC-1	singlet	0	-238.6848428	-654.4887954	-672.2791915	EMD1
TCATGGATCAGTGGGA-1	singlet	0	-615.3864441	-1287.660873	-1140.729459	EMD1
TCATGTTAGTGCGACA-1	singlet	2	-593.4371503	-558.1461537	-257.3012137	EMD1
TCATGTTCACAGAAGC-1	singlet	0	-219.7256008	-594.8145679	-588.3754666	EMD1
TCATGTTCACCCGTAG-1	singlet	2	-80.12804694	-65.19973398	-28.42788202	EMD1
TCATGTTGTCCAAAGG-1	singlet	1	-1891.098769	-615.4892168	-1567.027072	EMD1
TCATGTTGTCCTCCTA-1	singlet	0	-934.2694529	-2758.938641	-2713.246062	EMD1
TCATGTTGTGCGGATA-1	singlet	0	-339.0643318	-1117.851921	-1009.010448	EMD1
TCATGTTGTGTGTGTT-1	singlet	2	-742.0112895	-583.947918	-283.1828612	EMD1
TCATGTTGTGTTCGAT-1	singlet	1	-1714.374309	-426.854923	-1359.179173	EMD1
TCATGTTTCACTGTCC-1	singlet	1	-866.9174747	-349.1598087	-758.9997925	EMD1
TCATGTTTCGTTCCCA-1	singlet	2	-1839.134594	-1628.736377	-553.8561635	EMD1
TCATTACAGCTTAGTC-1	singlet	2	-538.9544083	-358.5696586	-183.7877487	EMD1
TCATTACAGTAGATCA-1	singlet	0	-203.0838395	-467.0853909	-593.1135406	EMD1
TCATTACCAGAGGTAC-1	singlet	2	-512.3560323	-425.8549454	-180.0714952	EMD1
TCATTACCATCCTCAC-1	singlet	0	-744.9002427	-1953.059558	-1867.376222	EMD1
TCATTACGTCACGCTG-1	singlet	2	-328.8491973	-268.0040001	-133.8988623	EMD1
TCATTACGTTGCTGAT-1	singlet	0	-438.7270184	-1031.614183	-1019.647264	EMD1
TCATTACGTTTCCATT-1	singlet	1	-658.0370226	-142.478532	-471.5311523	EMD1
TCATTACTCCGTTTCG-1	singlet	2	-6663.310255	-5156.107677	-2175.981669	EMD1
TCATTACTCTGATGGT-1	singlet	2	-740.263235	-607.6515165	-299.742353	EMD1
TCATTCAAGATCCGAG-1	singlet	2	-562.1514222	-527.4237436	-271.9982512	EMD1
TCATTCAAGGTCATAA-1	singlet	2	-535.8879747	-466.1261552	-229.3778369	EMD1
TCATTCAAGTGCGCTC-1	singlet	0	-343.834376	-1082.127966	-1074.529937	EMD1
TCATTCAAGTTTCTTC-1	singlet	2	-6084.515552	-5096.221184	-2042.734062	EMD1
TCATTCACACTTACAG-1	singlet	2	-441.5859313	-357.6399411	-193.2642999	EMD1
TCATTCACAGCACGAA-1	singlet	2	-346.6595815	-280.0742406	-142.5152513	EMD1

TCATTCACAGCTATTG-1	singlet	0	-280.0665922	-1193.581506	-1155.383922	EMD1
TCATTCAGTATATGGA-1	singlet	0	-181.6950307	-579.6431995	-588.5056268	EMD1
TCATTCAGTATCGTGT-1	singlet	2	-554.3407032	-454.9822741	-215.738296	EMD1
TCATTCAGTCTAATCG-1	singlet	1	-2136.916071	-736.0767375	-1712.996181	EMD1
TCATTCATCAGGCGAA-1	singlet	0	-60.78449447	-169.3274921	-207.104839	EMD1
TCATTCATCAGTGATC-1	singlet	0	-205.6441108	-512.2069417	-554.4721828	EMD1
TCATTCATCTCAAAGC-1	singlet	2	-962.7259945	-856.3513883	-315.3466465	EMD1
TCATTCATCTGTCAGA-1	singlet	0	-521.5092566	-1362.84731	-1222.30765	EMD1
TCATTGTAGCAAACAT-1	singlet	2	-678.1430296	-544.1464818	-251.9271618	EMD1
TCATTGTAGTGCGACA-1	singlet	0	-1566.77046	-4951.889624	-5197.257222	EMD1
TCATTGTAGTTTGTCG-1	singlet	0	-628.8262878	-1821.598293	-1584.712567	EMD1
TCATTGTCAATAGTAG-1	singlet	2	-598.3010747	-528.6188175	-214.649018	EMD1
TCATTGTCACACGTGC-1	singlet	2	-345.7992966	-264.3375367	-115.1682116	EMD1
TCATTGTGTAAGGCCA-1	singlet	0	-317.4370275	-1101.175602	-1074.810564	EMD1
TCATTGTGTACTCCCT-1	singlet	2	-3086.533242	-2497.449885	-778.055663	EMD1
TCATTGTGTCAGTTTG-1	singlet	1	-75.14580556	-32.64949067	-77.67727242	EMD1
TCATTGTGTCCTACAA-1	singlet	0	-373.4916128	-1241.91767	-1083.82651	EMD1
TCATTGTGTGCACAAG-1	singlet	2	-775.1014933	-652.9548999	-279.4703418	EMD1
TCATTGTGTGGATTTC-1	singlet	2	-2038.749613	-1527.318763	-714.70639	EMD1
TCATTGTGTGGCCCAT-1	singlet	2	-1450.145176	-1209.768341	-528.490195	EMD1
TCATTGTGTTCTGAGT-1	singlet	0	-742.6859371	-2744.320884	-2471.383292	EMD1
TCATTGTTCGATGGAG-1	singlet	1	-1325.375171	-373.9317764	-1100.504378	EMD1
TCATTGTTCGATTTCT-1	singlet	1	-3240.530226	-1152.067891	-2731.292531	EMD1
TCATTTGCATGAATAG-1	singlet	2	-532.9490483	-418.4816656	-232.6968004	EMD1
TCATTTGGTCAACATC-1	singlet	2	-6564.629746	-5769.243129	-2203.238216	EMD1
TCATTTGGTGTTGCCG-1	singlet	1	-4745.603605	-1596.040442	-4145.424827	EMD1
TCATTTGTCCGAGCTG-1	singlet	0	-1065.349806	-2960.337679	-3154.487804	EMD1
TCATTTGTCCTTATAC-1	singlet	2	-561.9605503	-453.8026729	-261.3074296	EMD1
TCCACCAAGCTAAGTA-1	singlet	1	-3515.41107	-934.1275314	-2527.792298	EMD1
TCCACCAAGGAAAGAC-1	singlet	2	-1908.520552	-1753.737584	-650.9936017	EMD1
TCCACCAAGGCCACCT-1	doublet	45323	-3691.940533	-2489.271724	-2201.643419	EMD1
TCCACCACAGCAGTCC-1	singlet	0	-1121.500945	-3379.857509	-3168.854199	EMD1
TCCACCACAGTGAGCA-1	singlet	2	-442.0999943	-310.8269951	-170.7747999	EMD1
TCCACCAGTATCAGGG-1	singlet	0	-1224.48995	-3780.877005	-3753.10002	EMD1
TCCACCATCATGTCAG-1	singlet	0	-216.6361252	-675.7838533	-682.0001735	EMD1
TCCACGTAGACCTCAT-1	doublet	0/2	-458.2492596	-737.4620835	-684.3198873	EMD1
TCCACGTAGACGCAGT-1	singlet	0	-686.3456707	-2687.948422	-2248.604929	EMD1
TCCACGTAGGTGGCTA-1	singlet	2	-345.2372022	-236.0030764	-113.1759154	EMD1
TCCACGTGTAGTTACC-1	singlet	2	-263.7977193	-290.5050266	-79.98872963	EMD1
TCCACGTGTGGGACAT-1	singlet	2	-436.6586881	-418.675306	-183.2533975	EMD1
TCCACGTTCATTTGGG-1	singlet	2	-129.9877445	-52.65150602	-31.89363377	EMD1
TCCACGTTCCATTGCC-1	singlet	2	-306.2377778	-259.6222174	-130.696565	EMD1
TCCACGTTCTCAGAAC-1	singlet	2	-673.3490362	-465.3713296	-228.5257359	EMD1
TCCAGAAAGCTCTATG-1	singlet	2	-746.7065171	-641.7771247	-288.3700861	EMD1
TCCAGAAAGTCGCCAC-1	singlet	2	-328.9230052	-307.5408727	-151.3224036	EMD1
TCCAGAACACCACATA-1	singlet	0	-457.158584	-1540.867259	-1387.306133	EMD1
TCCAGAACACCGGCTA-1	singlet	0	-233.7140737	-681.2817571	-728.887702	EMD1
TCCAGAACAGCGAGTA-1	singlet	2	-651.9061494	-503.0890049	-226.5046892	EMD1
TCCAGAAGTAGACGTG-1	singlet	0	-1098.283076	-3159.077822	-2929.536179	EMD1
TCCAGAAGTAGCGAGT-1	doublet	45293	-2281.851748	-1186.629584	-1610.949055	EMD1
TCCAGAAGTCTTCATT-1	singlet	0	-751.0164964	-1910.311981	-2044.115564	EMD1
TCCAGAATCATTGCCC-1	singlet	1	-143.3218698	-56.5239996	-152.7064617	EMD1
TCCAGAATCGAGCTGC-1	doublet	0/2	-1192.454459	-2412.484459	-2074.131613	EMD1
TCCAGAATCGCACGGT-1	singlet	2	-624.2104104	-455.7199355	-221.3900687	EMD1
TCCAGAATCTGCTAGA-1	singlet	2	-1540.811578	-1322.256361	-590.9653767	EMD1
TCCATCGCAACGTAAA-1	singlet	0	-326.1703533	-958.7541899	-971.2272963	EMD1
TCCATCGCAGATAAAC-1	singlet	1	-496.8029421	-193.1101825	-468.1642165	EMD1
TCCATCGGTGGTTTGT-1	doublet	0/1	-171.867514	-281.5356134	-273.5586859	EMD1
TCCATCGGTTCAACGT-1	singlet	1	-3613.168439	-1230.731021	-2988.617516	EMD1
TCCATCGTCCGTCAAA-1	singlet	0	-107.7530587	-336.4254504	-352.1983947	EMD1
TCCATCGTCGGACTGC-1	singlet	2	-85.21364956	-75.53333556	-19.68211487	EMD1
TCCATGCAGCCACTCG-1	singlet	1	-835.6364885	-404.4305568	-790.1648997	EMD1
TCCATGCCAGGCCTGT-1	singlet	1	-3412.538175	-997.0330528	-2502.244028	EMD1
TCCATGCCATCAGCAT-1	singlet	1	-89.55639433	-63.42689604	-79.15493538	EMD1
TCCATGCGTAGGACCA-1	singlet	1	-1661.828277	-525.7338512	-1259.594908	EMD1
TCCATGCGTCATATGC-1	singlet	2	-2531.640914	-2142.191501	-881.850951	EMD1
TCCATGCGTTCTCCTG-1	singlet	0	-178.9022453	-506.6759039	-514.1618804	EMD1
TCCATGCGTTGTCCCT-1	singlet	0	-1235.715938	-4050.373624	-3989.129624	EMD1
TCCATGCTCAATGCAC-1	singlet	1	-2847.239698	-888.2178321	-2274.800151	EMD1
TCCATGCTCACGATAC-1	doublet	45323	-1369.959687	-835.1706846	-827.9355351	EMD1
TCCATGCTCCTGTACC-1	singlet	0	-3172.32265	-12566.01451	-11201.91674	EMD1
TCCATGCTCGGCTTGG-1	singlet	1	-4928.803948	-1290.365023	-3739.071768	EMD1
TCCATGCTCTATCACT-1	singlet	2	-304.4186422	-264.9253111	-120.3837998	EMD1
TCCCACAAGAAGTCTA-1	singlet	2	-399.3645607	-362.9809403	-168.2187697	EMD1
TCCCACAAGAGTGGCT-1	singlet	0	-862.6185024	-2592.520198	-2582.492283	EMD1
TCCCACAAGATCGCTT-1	singlet	0	-678.3288671	-1992.989657	-2155.12985	EMD1
TCCCACAAGATTGATG-1	singlet	2	-329.0424813	-317.3252895	-124.4078124	EMD1
TCCCACAAGCGAGTAC-1	singlet	1	-1110.189104	-456.0447373	-1017.122409	EMD1
TCCCACAAGGAGATAG-1	singlet	2	-673.4875698	-492.1829867	-235.4948703	EMD1
TCCCACACAACTGCCG-1	unassigned	1/0	-6.429503455	-3.258096201	-3.311107266	EMD1
TCCCACACAGACCGCT-1	singlet	1	-43.63957803	-12.1833735	-40.47820137	EMD1
TCCCACAGTAGTGTGG-1	singlet	2	-2382.386547	-2195.380117	-731.0360184	EMD1
TCCCACAGTATAGCTC-1	singlet	2	-797.4806194	-665.916332	-308.8210227	EMD1
TCCCACAGTTCCGCAG-1	singlet	2	-653.6792671	-523.3314492	-268.1400453	EMD1
TCCCAGTAGGTGATAT-1	singlet	0	-593.7386181	-1758.022266	-1717.214485	EMD1
TCCCAGTAGTGTACCT-1	singlet	1	-3416.177378	-963.0440993	-2471.618402	EMD1
TCCCAGTCACACAGAG-1	singlet	1	-755.622124	-294.5974045	-766.8306466	EMD1
TCCCAGTCACTTGACA-1	singlet	2	-530.5125393	-412.4143809	-192.2386275	EMD1
TCCCAGTCACTTGAGT-1	singlet	1	-2185.899762	-611.3143878	-1737.400451	EMD1
TCCCAGTGTCTACAGT-1	singlet	2	-374.5953093	-297.8337349	-131.4877089	EMD1
TCCCAGTGTGTGAATA-1	singlet	2	-291.5265614	-216.9275587	-105.4588603	EMD1
TCCCAGTGTTAGGCTT-1	singlet	2	-553.3968682	-519.5738122	-252.1435794	EMD1
TCCCAGTGTTGGGATG-1	singlet	2	-770.9434933	-694.8503887	-251.1131792	EMD1
TCCCAGTTCGAGCCAC-1	singlet	1	-7618.616968	-1497.846407	-4626.587746	EMD1
TCCCAGTTCTGCGGCA-1	singlet	2	-379.5249851	-329.7737313	-158.2517785	EMD1
TCCCAGTTCTGGCTGG-1	singlet	2	-2630.524492	-2278.878786	-947.1671029	EMD1
TCCCATGAGCTGAGCA-1	singlet	2	-666.147771	-635.4712911	-300.9512309	EMD1
TCCCATGAGGACAACC-1	singlet	2	-2116.43913	-1724.978352	-804.7189114	EMD1
TCCCATGAGTCAAGCG-1	singlet	2	-367.8956137	-318.2915079	-132.1078187	EMD1
TCCCATGCACAAATGA-1	singlet	2	-456.3670411	-329.3074753	-151.1559313	EMD1
TCCCATGCATATGGCT-1	singlet	2	-1160.373593	-1119.22477	-458.2546493	EMD1
TCCCATGGTACATTGC-1	singlet	0	-719.7715238	-2237.572948	-2104.738156	EMD1
TCCCATGGTAGTCACT-1	singlet	2	-831.6017553	-636.4000085	-224.2196293	EMD1
TCCCATGGTATCGTTG-1	singlet	2	-327.2326574	-299.8774576	-119.6155099	EMD1
TCCCATGGTCTTCAAG-1	singlet	2	-1646.370409	-1439.583982	-584.2905542	EMD1
TCCCATGGTGTACATC-1	singlet	1	-228.5957582	-68.86905929	-223.3081183	EMD1
TCCCATGTCTAGTGTG-1	singlet	2	-279.7271961	-264.7141395	-117.988103	EMD1
TCCGAAAAGGACGGAG-1	singlet	1	-1825.094865	-555.9757228	-1587.666462	EMD1
TCCGAAACAACCGGAA-1	singlet	2	-232.0189193	-215.5181416	-87.33591929	EMD1
TCCGAAACAATTAGGA-1	singlet	2	-501.6710967	-441.1185421	-208.4876927	EMD1

TCCGAAACAGAGTTCT-1	singlet	0	-1121.356039	-2621.540188	-2971.248146	EMD1
TCCGAAAGTAACAGTA-1	singlet	2	-516.3051872	-425.4103646	-209.6302329	EMD1
TCCGAAAGTCCGACGT-1	singlet	2	-474.3798095	-371.7742969	-189.79637	EMD1
TCCGAAAGTTCTAAGC-1	singlet	0	-7.607307737	-11.22664834	-106.0454918	EMD1
TCCGAAAGTTGGGTTT-1	singlet	1	-2110.79439	-653.8565556	-1718.883612	EMD1
TCCGAAATCCATTGCC-1	singlet	0	-1369.521642	-3090.880313	-2871.567804	EMD1
TCCGATCAGAAGTCCG-1	singlet	2	-266.2958199	-221.4388708	-101.9959664	EMD1
TCCGATCCAAAGAACT-1	singlet	2	-654.8222857	-488.2770857	-245.3214425	EMD1
TCCGATCCAAGCTCTA-1	singlet	2	-789.4826256	-711.4889775	-309.8130212	EMD1
TCCGATCCAGAGGACT-1	singlet	0	-199.1450297	-534.3652374	-614.3954164	EMD1
TCCGATCGTAGTGATA-1	singlet	2	-601.3619392	-541.750447	-264.1383588	EMD1
TCCGATCGTATTGACC-1	singlet	1	-1021.403394	-370.3190141	-720.7559297	EMD1
TCCGATCGTCACTTCC-1	singlet	2	-592.664036	-447.9160756	-197.3722135	EMD1
TCCGATCTCTACTCAT-1	singlet	2	-504.6091472	-505.946058	-207.6173337	EMD1
TCCGATCTCTATTCGT-1	singlet	2	-2270.079748	-1732.26612	-690.7272044	EMD1
TCCGGGAAGGGATCTG-1	singlet	1	-2388.395229	-711.6892515	-1937.373904	EMD1
TCCGGGACAAACGTGG-1	singlet	2	-518.1995449	-406.8301942	-190.4020707	EMD1
TCCGGGACATGGGATG-1	singlet	1	-1346.13615	-404.3027145	-1040.817213	EMD1
TCCGGGAGTGGACAGT-1	singlet	2	-409.7852325	-277.7787279	-113.3874387	EMD1
TCCGGGAGTTGCAAGG-1	singlet	2	-462.6496871	-398.2891622	-206.8338576	EMD1
TCCGGGATCCGGACGT-1	singlet	2	-1558.309869	-1271.51447	-564.1956672	EMD1
TCCGGGATCTGGGCGT-1	singlet	0	-562.3568376	-1683.979757	-1699.569507	EMD1
TCCGGGATCTTCGTAT-1	singlet	2	-883.3418951	-804.4133504	-291.4112193	EMD1
TCCGTGTCAATCGAAA-1	singlet	2	-745.0807437	-579.2081208	-264.8530541	EMD1
TCCGTGTCACGTGAGA-1	singlet	1	-194.8924164	-66.28783723	-147.1848627	EMD1
TCCGTGTCAGTGTGGA-1	singlet	2	-4962.348918	-3921.482667	-1677.081959	EMD1
TCCGTGTCATCTCCCA-1	singlet	0	-456.4919733	-1402.701438	-1310.721976	EMD1
TCCGTGTCATGACGGA-1	singlet	2	-309.8286629	-219.7231167	-127.4091621	EMD1
TCCGTGTCATTGACTG-1	singlet	0	-447.5219901	-1175.114118	-1201.835529	EMD1
TCCGTGTGTCACAGAG-1	singlet	1	-1144.243996	-360.3802693	-823.744366	EMD1
TCCGTGTGTCGTTATG-1	singlet	1	-983.475064	-274.3980873	-865.2636828	EMD1
TCCGTGTGTGAGATAT-1	singlet	2	-1453.423989	-1217.470194	-510.4226142	EMD1
TCCGTGTTCCGTGACG-1	singlet	1	-5682.945641	-1412.54109	-5461.447035	EMD1
TCCGTGTTCGATACGT-1	singlet	1	-455.663544	-153.416237	-364.4888748	EMD1
TCCTAATAGATGGCAC-1	singlet	1	-1377.089467	-440.9385694	-1179.796601	EMD1
TCCTAATCAGTGTACT-1	singlet	0	-1058.838775	-3467.61393	-3480.319849	EMD1
TCCTAATGTCATAGTC-1	doublet	1/0	-1798.617539	-1417.518051	-2052.219567	EMD1
TCCTAATGTCGTCATA-1	singlet	2	-932.3568358	-765.7886206	-369.211568	EMD1
TCCTAATTCACGGTCG-1	singlet	0	-254.6112842	-663.8910047	-622.7763704	EMD1
TCCTAATTCGGCGATC-1	singlet	2	-1021.85615	-866.2808296	-416.2017216	EMD1
TCCTCCCAGGAACTAT-1	singlet	2	-459.2576615	-357.7459794	-160.1500019	EMD1
TCCTCCCAGGTTCATC-1	singlet	2	-369.4000362	-282.2722696	-179.7167907	EMD1
TCCTCCCAGTAGAATC-1	singlet	0	-29.36184514	-73.77642641	-56.39919322	EMD1
TCCTCCCAGTCACTCA-1	singlet	0	-296.0884847	-708.7356577	-768.7219024	EMD1
TCCTCCCCACGACTAT-1	singlet	2	-822.2524451	-703.0137759	-304.6857251	EMD1
TCCTCCCCATTATGCG-1	doublet	45293	-8448.759961	-3670.76312	-5213.492762	EMD1
TCCTCCCGTTGGCTAT-1	singlet	2	-499.8694993	-425.5432115	-208.2155174	EMD1
TCCTCCCTCATGCTAG-1	singlet	2	-786.8231057	-658.1786802	-316.3703108	EMD1
TCCTCCCTCCCAGTGG-1	singlet	1	-88.76639952	-25.47523784	-59.63152617	EMD1
TCCTCCCTCGAGTCCG-1	singlet	1	-6702.3089	-1507.564762	-5726.436849	EMD1
TCCTCGAAGAGAACCC-1	singlet	2	-288.2197678	-244.8292161	-122.5060098	EMD1
TCCTCGAAGCCATTGT-1	singlet	2	-1230.505563	-1126.462847	-520.6224868	EMD1
TCCTCGACAATGCAGG-1	singlet	0	-605.8277698	-1703.34129	-1696.124744	EMD1
TCCTCGACAGGAGACT-1	singlet	2	-304.5347554	-286.8935465	-122.0002459	EMD1
TCCTCGACATCGAGCC-1	singlet	1	-1699.198147	-587.8311415	-1431.872932	EMD1
TCCTCGAGTACTTCCC-1	singlet	2	-1658.672233	-1288.17169	-692.1100288	EMD1
TCCTCGAGTCTGATAC-1	singlet	1	-3476.228886	-1120.600645	-2937.414535	EMD1
TCCTCGAGTGAAAGTT-1	singlet	2	-1278.750458	-1131.286376	-443.1239876	EMD1
TCCTCTTCACCCTCTA-1	singlet	1	-2655.149752	-663.7180687	-2323.21001	EMD1
TCCTCTTCATGTGGCC-1	singlet	2	-602.6645174	-546.2870092	-235.9278997	EMD1
TCCTCTTGTCATTGCA-1	singlet	1	-2459.883443	-697.5863096	-1824.23291	EMD1
TCCTCTTTCAAACCCA-1	singlet	1	-1766.125621	-516.3724798	-1530.570523	EMD1
TCCTCTTTCAGGCGAA-1	singlet	2	-571.1275968	-475.2637908	-226.4283819	EMD1
TCCTCTTTCCGTTGGG-1	singlet	0	-42.32682781	-132.1417429	-116.3824049	EMD1
TCCTGCAAGACGATAT-1	singlet	2	-346.7659978	-307.529038	-136.5511739	EMD1
TCCTGCAAGCCGATTT-1	singlet	2	-575.0553846	-520.9102018	-255.1604755	EMD1
TCCTGCAAGCTCGGCT-1	singlet	2	-455.7352366	-354.8944111	-172.0051068	EMD1
TCCTGCACACCCAACG-1	singlet	0	-810.3098179	-2484.621273	-2624.762241	EMD1
TCCTGCACAGCGTATT-1	doublet	45323	-650.6205562	-407.4671431	-358.4551345	EMD1
TCCTGCACAGGATTCT-1	singlet	1	-654.5091205	-272.7099532	-525.6420264	EMD1
TCCTGCACATAGACTC-1	singlet	2	-697.1547655	-564.8377248	-247.0731022	EMD1
TCCTGCACATGACGTT-1	singlet	1	-2320.8465	-665.8577047	-1850.464804	EMD1
TCCTGCAGTCATACCA-1	singlet	2	-513.8978917	-554.306334	-243.1586384	EMD1
TCCTGCAGTGTTCGTA-1	singlet	1	-1639.532541	-529.8441774	-1340.089548	EMD1
TCCTGCAGTTATCTTC-1	singlet	0	-595.9591534	-1629.468239	-1640.855385	EMD1
TCCTGCATCCAAGCAT-1	singlet	2	-1566.79128	-1455.739658	-526.7067903	EMD1
TCCTGCATCCCGTGTT-1	singlet	2	-531.3417182	-418.6561944	-225.2778731	EMD1
TCCTTCTAGCATGATA-1	singlet	2	-103.8687425	-52.62491015	-33.98133166	EMD1
TCCTTCTAGTCGTTAC-1	singlet	2	-450.5776413	-357.706338	-166.6105138	EMD1
TCCTTCTCAACCGTAT-1	singlet	1	-92.6900217	-42.0099638	-82.05890095	EMD1
TCCTTCTCAAGAGGCT-1	singlet	2	-760.5750554	-644.9667641	-289.2711581	EMD1
TCCTTCTCAATTGCGT-1	singlet	0	-1175.394132	-4390.310439	-4310.880965	EMD1
TCCTTCTGTTCTCGTC-1	singlet	2	-986.4458632	-812.2128887	-428.0095846	EMD1
TCCTTCTTCATCGTAG-1	singlet	2	-2567.902612	-2454.196103	-938.5295755	EMD1
TCCTTCTTCCATTGTT-1	singlet	2	-571.8760547	-467.9537004	-246.1692104	EMD1
TCCTTCTTCTAGCATG-1	singlet	2	-1551.50181	-1313.861584	-550.4487894	EMD1
TCCTTTCAGACGCCCT-1	singlet	0	-424.9363846	-1391.78271	-1491.173907	EMD1
TCCTTTCAGAGGCTGT-1	singlet	0	-23.90047719	-40.33060089	-215.0997321	EMD1
TCCTTTCAGATTGAGT-1	singlet	2	-173.9221835	-154.3295391	-68.67097966	EMD1
TCCTTTCAGATTGTGA-1	singlet	0	-1100.015644	-2918.072344	-2848.780594	EMD1
TCCTTTCAGCAGCACA-1	singlet	2	-494.0024737	-543.6679357	-259.7079567	EMD1
TCCTTTCAGCATCCTA-1	singlet	2	-1776.777098	-1427.876886	-703.5204814	EMD1
TCCTTTCAGCGACAGT-1	singlet	2	-384.2280359	-345.130136	-142.0885359	EMD1
TCCTTTCAGCTCGGCT-1	singlet	2	-301.6589096	-251.0424539	-125.8782737	EMD1
TCCTTTCAGCTTTCCC-1	doublet	0/1	-5700.649765	-6061.550785	-7954.416363	EMD1
TCCTTTCAGGGAGTGG-1	singlet	1	-95.90222971	-41.8250984	-80.48715554	EMD1
TCCTTTCAGGTGAGCT-1	singlet	0	-1447.801133	-4084.606124	-3903.593764	EMD1
TCCTTTCAGGTTGGTG-1	singlet	2	-444.9441059	-374.6657609	-163.5117349	EMD1
TCCTTTCAGTTCAACC-1	singlet	0	-412.0447626	-1427.29394	-1271.823168	EMD1
TCCTTTCCAAAGTGTA-1	singlet	2	-115.6692028	-87.22289305	-43.03249127	EMD1
TCCTTTCCACTACAGT-1	singlet	0	-726.3097689	-2480.197968	-2233.520023	EMD1
TCCTTTCCAGTCTTCC-1	singlet	0	-1239.727544	-3517.126093	-3643.464203	EMD1
TCCTTTCGTACAAGCG-1	singlet	1	-2255.552141	-689.0752991	-1783.648335	EMD1
TCCTTTCGTCCATAGT-1	singlet	1	-3952.822695	-1240.836903	-2797.686317	EMD1
TCCTTTCGTGGTACAG-1	singlet	1	-1854.774229	-553.6976429	-1477.730365	EMD1
TCCTTTCTCACTGTCC-1	singlet	2	-639.8634553	-564.2791717	-237.5260289	EMD1
TCCTTTCTCCATTTCA-1	singlet	0	-1411.425192	-4155.282078	-3736.319726	EMD1
TCCTTTCTCCTGCTAC-1	singlet	1	-108.5473903	-26.80414201	-70.95529197	EMD1
TCGAACAAGCGACATG-1	singlet	2	-626.2034504	-506.360214	-263.6852704	EMD1
TCGAACACAAGACCTT-1	doublet	0/1	-872.8688283	-1029.110903	-1438.424459	EMD1

TCGAACAGTGGCACTC-1	singlet	2	-449.8787714	-354.7066052	-162.6308725	EMD1
TCGAACAGTGTCCTAA-1	singlet	2	-1764.428751	-1594.792192	-572.9180559	EMD1
TCGAACATCGCCAGTG-1	singlet	0	-291.7870205	-819.5206306	-720.6938884	EMD1
TCGAACATCTACACTT-1	singlet	0	-139.1567876	-326.8906394	-279.2063148	EMD1
TCGAAGTCACATGTTG-1	singlet	0	-597.6786798	-1795.95972	-1671.767711	EMD1
TCGAAGTCATAACTCG-1	singlet	2	-1752.398384	-2216.36156	-545.0961423	EMD1
TCGAAGTGTCTAGGCC-1	singlet	2	-306.2646463	-292.0479298	-150.0540025	EMD1
TCGAAGTTCGAACCTA-1	singlet	2	-2805.277832	-2245.561797	-800.1991225	EMD1
TCGAAGTTCGATCCAA-1	singlet	1	-2684.846586	-720.5128931	-2259.507746	EMD1
TCGAAGTTCTAGTACG-1	singlet	2	-313.7396563	-325.1460615	-157.9684788	EMD1
TCGACCTAGAGCTGCA-1	singlet	1	-4091.695798	-1150.460158	-3089.567021	EMD1
TCGACCTAGTATGAGT-1	singlet	2	-506.404324	-392.849762	-225.4653498	EMD1
TCGACCTCAATCCAGT-1	singlet	0	-526.9027308	-1645.398258	-1649.043772	EMD1
TCGACCTCACCTTCCA-1	singlet	1	-2403.204877	-780.8329065	-2510.716427	EMD1
TCGACCTGTGTCACAT-1	singlet	2	-284.1473307	-246.9010102	-117.7703041	EMD1
TCGACCTTCTACTTCA-1	singlet	0	-180.340796	-445.7442304	-539.9562385	EMD1
TCGACCTTCTTCACAT-1	singlet	2	-4180.666198	-3290.133112	-1520.210936	EMD1
TCGACGGCAGAGGCTA-1	singlet	1	-1301.16837	-388.3792125	-1018.167211	EMD1
TCGACGGGTCGCATGC-1	singlet	0	-181.3833712	-404.559974	-460.3352245	EMD1
TCGACGGGTGGCTCTG-1	singlet	2	-289.501547	-213.5245969	-88.95273915	EMD1
TCGACGGGTTTAGAGA-1	doublet	1/0	-2156.371712	-2048.022385	-2618.371239	EMD1
TCGACGGTCAGCGCAC-1	singlet	2	-308.1821295	-246.6441501	-127.0647272	EMD1
TCGATTTAGCACGTCC-1	singlet	0	-659.344668	-1698.069731	-1533.523114	EMD1
TCGATTTCAAAGTGTA-1	singlet	2	-505.9156059	-404.0473211	-165.0710274	EMD1
TCGATTTCAACCGATT-1	singlet	0	-344.4087309	-1205.235877	-1040.416202	EMD1
TCGATTTCATTGAGGG-1	singlet	0	-286.7400051	-968.0452389	-909.4081298	EMD1
TCGATTTGTGTTCGAT-1	singlet	1	-171.3082457	-65.9807327	-155.4678492	EMD1
TCGATTTGTTTCGTTT-1	singlet	2	-650.2550902	-547.851264	-213.8685566	EMD1
TCGATTTTCGCATGAT-1	singlet	0	-178.8662975	-594.4986701	-619.4576621	EMD1
TCGCACTAGATGACAT-1	singlet	2	-7966.707268	-5925.641867	-3339.582687	EMD1
TCGCACTAGGGAGATA-1	singlet	1	-3528.626649	-1118.173678	-3022.570729	EMD1
TCGCACTAGTTCGGTT-1	singlet	0	-25.75834514	-74.35796958	-69.15035236	EMD1
TCGCACTAGTTTCGAC-1	singlet	2	-354.2308062	-319.7654175	-157.3820431	EMD1
TCGCACTCACGCACCA-1	singlet	2	-1744.120761	-1532.187407	-610.4477253	EMD1
TCGCACTCAGTCAGTT-1	singlet	2	-596.6161739	-488.4539887	-232.0221352	EMD1
TCGCACTGTAGGATAT-1	singlet	1	-4520.972234	-1240.209897	-3408.260784	EMD1
TCGCACTGTCAAGCGA-1	singlet	1	-1440.556492	-584.1741915	-1225.786493	EMD1
TCGCACTGTGTCCATA-1	singlet	2	-604.2547961	-478.1967504	-221.2021911	EMD1
TCGCACTTCGACCCAG-1	singlet	0	-262.8878064	-665.4069097	-757.476036	EMD1
TCGCACTTCGTCAAAC-1	singlet	1	-2572.732942	-666.070421	-2065.179051	EMD1
TCGCAGGAGATGTTAG-1	singlet	2	-345.6574412	-350.7221508	-159.054762	EMD1
TCGCAGGAGCACGATG-1	singlet	2	-625.7230932	-529.3636985	-209.8027547	EMD1
TCGCAGGAGCGTTCAT-1	singlet	1	-1879.36164	-683.1877919	-1639.05437	EMD1
TCGCAGGAGTATTGCC-1	singlet	2	-490.9754524	-400.8937662	-160.2074017	EMD1
TCGCAGGAGTCACAGG-1	singlet	0	-260.4774427	-1116.651152	-1011.845048	EMD1
TCGCAGGCAAACCATC-1	singlet	0	-1241.426602	-3830.763715	-3894.297026	EMD1
TCGCAGGCAAAGCGTG-1	singlet	0	-198.5484784	-592.3646421	-567.485565	EMD1
TCGCAGGGTACTGAGG-1	singlet	1	-851.1573635	-323.7151284	-691.1232231	EMD1
TCGCAGGGTAGTCGTT-1	doublet	0/2	-432.6909582	-645.8808567	-557.0600915	EMD1
TCGCAGGGTCCGGTCA-1	singlet	2	-579.917301	-518.8095189	-213.1813516	EMD1
TCGCAGGGTCTAATCG-1	doublet	45323	-9615.755328	-5979.770563	-3952.899047	EMD1
TCGCAGGGTTATAGCC-1	singlet	1	-3467.934785	-683.7791364	-2905.815003	EMD1
TCGCAGGTCGCCGAGT-1	singlet	0	-80.8196709	-261.4796096	-225.8448915	EMD1
TCGCAGGTCGTCTCAC-1	doublet	2/0	-1941.76157	-2124.783369	-1422.834808	EMD1
TCGCAGGTCTAATTCC-1	singlet	2	-448.4137543	-454.6662945	-205.9154806	EMD1
TCGCAGGTCTTGTGCC-1	singlet	2	-2887.502851	-2260.232284	-954.6595747	EMD1
TCGCTCAAGCCTATCA-1	singlet	2	-441.9623933	-376.3222176	-176.9257253	EMD1
TCGCTCAAGGCAATGC-1	singlet	2	-717.9105502	-599.1870498	-308.8830234	EMD1
TCGCTCAAGTAAACGT-1	singlet	2	-513.2799496	-398.4382575	-215.8291649	EMD1
TCGCTCACAAGACCGA-1	singlet	2	-2690.004319	-2021.008204	-874.3375411	EMD1
TCGCTCACAATCCTTT-1	singlet	2	-2655.266806	-2111.887326	-923.8483522	EMD1
TCGCTCAGTAAGACCG-1	doublet	1/0	-690.1173118	-639.0294776	-782.3330208	EMD1
TCGCTCAGTAAGCAAT-1	singlet	1	-2392.136042	-935.6033827	-1959.910625	EMD1
TCGCTCAGTCAAACGG-1	singlet	2	-529.4421575	-444.1880024	-206.5159759	EMD1
TCGCTCATCACTCGAA-1	singlet	2	-753.3420285	-755.3542376	-312.8836039	EMD1
TCGCTCATCGTACACA-1	singlet	1	-2761.807498	-723.9463269	-2177.01775	EMD1
TCGCTTGAGGTAGTAT-1	singlet	2	-1282.029594	-1193.076631	-504.2777837	EMD1
TCGCTTGCAATTCACG-1	singlet	0	-49.72377166	-262.7240106	-180.9365405	EMD1
TCGCTTGCAGCTTTGA-1	singlet	1	-5396.578481	-1607.613027	-4154.568545	EMD1
TCGCTTGCATACAGCT-1	singlet	0	-152.4656293	-488.4663327	-374.8707137	EMD1
TCGCTTGTCCGTCACT-1	singlet	2	-310.9948108	-287.6312906	-140.5955134	EMD1
TCGCTTGTCGATGCAT-1	singlet	2	-776.6171311	-690.3357901	-299.7736799	EMD1
TCGCTTGTCGTAGGGA-1	singlet	2	-2085.818021	-1908.333692	-636.9904406	EMD1
TCGGATAAGATTCGAA-1	singlet	2	-280.3317643	-248.5771157	-101.6600101	EMD1
TCGGATAAGGCTGGAT-1	singlet	2	-555.3747565	-468.8160477	-193.9924425	EMD1
TCGGATAAGTAACCGG-1	singlet	2	-2372.599539	-1785.365593	-787.0075889	EMD1
TCGGATAAGTCTACCA-1	singlet	0	-825.9703677	-2538.944087	-2476.213989	EMD1
TCGGATAAGTCTAGCT-1	singlet	2	-483.3203973	-426.6160756	-193.5059389	EMD1
TCGGATAAGTGAACAT-1	singlet	2	-143.0306373	-111.3986832	-42.57463363	EMD1
TCGGATAAGTTTCGAC-1	singlet	0	-532.1595835	-1761.046513	-1624.982231	EMD1
TCGGATACAGGCACTC-1	singlet	0	-182.8935882	-554.1819664	-539.5199252	EMD1
TCGGATACATGGGTCC-1	singlet	1	-159.2055933	-59.50804018	-145.1387349	EMD1
TCGGATAGTAGCACAG-1	singlet	2	-435.2291122	-384.9600674	-191.0650108	EMD1
TCGGATAGTATGAAAC-1	singlet	0	-395.5526629	-1120.298739	-1095.574801	EMD1
TCGGATAGTTAAGAAC-1	singlet	2	-3752.245127	-3305.890064	-1435.38777	EMD1
TCGGATATCCAGTGTA-1	singlet	2	-5823.238314	-4534.99023	-1841.222194	EMD1
TCGGATATCCATCGTC-1	singlet	2	-393.9397375	-284.3114609	-163.1496959	EMD1
TCGGGACAGGAGTCTG-1	singlet	2	-291.4219607	-270.4246751	-120.969428	EMD1
TCGGGACCACTCCTTG-1	singlet	1	-276.295644	-109.1301885	-231.0249665	EMD1
TCGGGACGTACAGTTC-1	singlet	1	-141.2558512	-54.44417868	-152.7428439	EMD1
TCGGGACGTTATGTCG-1	singlet	1	-550.5295486	-149.3712874	-412.6340945	EMD1
TCGGGACTCCTGTTAT-1	singlet	2	-490.5192128	-413.7504389	-199.1681746	EMD1
TCGGGACTCTCCGCAT-1	singlet	2	-365.4708767	-275.3372516	-141.5751667	EMD1
TCGGGCAAGAGGGTCT-1	singlet	2	-435.278599	-333.8610773	-167.7287072	EMD1
TCGGGCAGTAAGATAC-1	singlet	1	-3454.162441	-1247.904013	-2635.473692	EMD1
TCGGGCAGTATGCGGA-1	singlet	1	-436.8191787	-154.45923	-416.66849	EMD1
TCGGGCAGTGATTGGG-1	singlet	1	-309.7341914	-66.79297955	-196.1783705	EMD1
TCGGGCAGTGGAGGTT-1	singlet	2	-273.9701595	-308.476057	-99.64014566	EMD1
TCGGGCAGTTACCCAA-1	singlet	0	-197.4725489	-553.5753921	-594.7756134	EMD1
TCGGGCAGTTCGGGTC-1	singlet	2	-238.5242474	-197.0847195	-111.3397684	EMD1
TCGGGTGAGACAGCGT-1	singlet	2	-196.2454701	-154.7451795	-58.03670766	EMD1
TCGGGTGAGATGAATC-1	singlet	2	-128.4318015	-152.5791009	-67.45568623	EMD1
TCGGGTGAGGACAACC-1	singlet	0	-945.0149739	-3044.687282	-3192.459046	EMD1
TCGGGTGAGTGCCTCG-1	singlet	1	-1026.468151	-351.4407584	-808.6664445	EMD1
TCGGGTGCAAAGAACT-1	singlet	2	-761.2878421	-603.5721756	-235.9033119	EMD1
TCGGGTGCAAGGTCTT-1	singlet	2	-3060.65684	-2554.714796	-1051.230948	EMD1
TCGGGTGGTCTGCCTT-1	singlet	1	-1262.501618	-536.4264444	-1085.055348	EMD1
TCGGGTGGTTCATCGA-1	singlet	2	-615.084851	-475.6181366	-189.1615396	EMD1
TCGGGTGTCGCGCCAA-1	singlet	2	-1174.42923	-1302.603348	-345.1531931	EMD1
TCGGTCTAGCCGGAAT-1	singlet	0	-872.4972297	-2915.806571	-2836.578021	EMD1

TCGGTCTAGGCTCTAT-1	singlet	0	-80.97932202	-243.6567417	-214.9113312	EMD1
TCGGTCTCAAGTCCAT-1	singlet	0	-154.6253547	-495.3268036	-414.0806346	EMD1
TCGGTCTCAGGAGGTT-1	singlet	2	-403.5993511	-376.7853498	-166.2845551	EMD1
TCGGTCTGTCAACACT-1	singlet	2	-4483.298151	-3520.370942	-1400.197577	EMD1
TCGGTCTGTGTGAATA-1	singlet	1	-4620.454027	-1689.508485	-3467.251111	EMD1
TCGGTCTTCAGGGATG-1	doublet	45293	-3067.609992	-1525.255574	-2011.789664	EMD1
TCGGTCTTCAGTCACA-1	singlet	2	-974.7160101	-841.7093552	-289.5341516	EMD1
TCGGTCTTCCCAGCGA-1	singlet	0	-27.40846754	-73.13293353	-67.74723837	EMD1
TCGGTCTTCGACACCG-1	singlet	2	-281.8378305	-226.5100196	-127.344618	EMD1
TCGTAGAAGCCATTCA-1	doublet	0/2	-1725.294998	-2421.054217	-1902.930513	EMD1
TCGTAGAAGCTCGCAC-1	singlet	1	-2033.985958	-525.3218813	-1541.607201	EMD1
TCGTAGAAGGTACATA-1	singlet	1	-436.5615068	-165.1344255	-346.2568701	EMD1
TCGTAGACAAACGTGG-1	singlet	1	-2900.776869	-702.5956392	-1877.599367	EMD1
TCGTAGAGTAGCTAAA-1	singlet	0	-830.7676757	-2598.089549	-2611.612088	EMD1
TCGTAGAGTCGAACAG-1	singlet	0	-729.6460458	-2150.721408	-2090.644919	EMD1
TCGTAGAGTGACCGAA-1	singlet	2	-246.3771079	-175.3206414	-95.56173785	EMD1
TCGTAGAGTGGAAATT-1	singlet	0	-623.0460341	-1850.263536	-1862.170759	EMD1
TCGTAGATCAGAACCT-1	singlet	1	-2652.461231	-697.0391369	-1792.295091	EMD1
TCGTAGATCTGAGTCA-1	singlet	2	-695.7395049	-588.8472194	-254.135777	EMD1
TCGTCCAAGATTGATG-1	singlet	1	-362.0954665	-155.0897199	-360.0324534	EMD1
TCGTCCAAGGACATCG-1	singlet	0	-375.7936549	-1162.104526	-1298.052318	EMD1
TCGTCCACAAAGAGTT-1	singlet	2	-3097.631172	-2294.224868	-1012.240466	EMD1
TCGTCCACAAAGGCTG-1	singlet	0	-56.9722662	-175.4112185	-166.0373785	EMD1
TCGTCCACAAGGACAC-1	singlet	0	-229.3067908	-509.0239661	-584.0711817	EMD1
TCGTCCACAGACAAAT-1	singlet	2	-272.6452676	-220.8861601	-101.3660957	EMD1
TCGTCCACAGGATTCT-1	singlet	2	-182.9365247	-150.8735803	-53.1518571	EMD1
TCGTCCACATGGATCT-1	singlet	2	-1372.674992	-1379.814621	-544.3619017	EMD1
TCGTCCAGTACAACGG-1	singlet	2	-2898.633076	-2717.283567	-1630.98884	EMD1
TCGTCCAGTCATCCCT-1	singlet	0	-133.6171377	-468.7049718	-433.8363634	EMD1
TCGTCCAGTTCCGGTG-1	singlet	1	-2653.738478	-661.0474884	-2239.435698	EMD1
TCGTCCATCGGCATAT-1	singlet	1	-930.4342055	-277.5156232	-710.8768367	EMD1
TCGTGCTCAATTGTGC-1	singlet	2	-1757.35482	-1193.905012	-490.1674964	EMD1
TCGTGCTCACCGCTGA-1	doublet	0/1	-436.9181995	-500.2348483	-808.710944	EMD1
TCGTGCTCAGAGGTAC-1	singlet	2	-178.2468322	-134.4691761	-70.67840006	EMD1
TCGTGCTCATGACACT-1	singlet	2	-405.142834	-358.9921083	-150.8062254	EMD1
TCGTGCTGTAACCAGG-1	singlet	0	-91.59838206	-268.8736284	-236.6132351	EMD1
TCGTGCTGTAACGCGA-1	singlet	2	-879.5595491	-812.9899373	-341.4223408	EMD1
TCGTGCTGTTCAGCTA-1	singlet	2	-164.4783332	-128.2108489	-70.08512004	EMD1
TCGTGCTTCCACTAGA-1	singlet	0	-175.3856764	-686.6210026	-855.5288039	EMD1
TCGTGCTTCGTAGTCA-1	singlet	0	-104.1912144	-426.7162479	-319.3733862	EMD1
TCGTGCTTCTCTGACC-1	singlet	2	-204.3531815	-175.8923669	-73.0918178	EMD1
TCGTGGGAGCGGATCA-1	singlet	2	-174.4382126	-155.3413511	-65.21055133	EMD1
TCGTGGGGTCAACACT-1	singlet	0	-45.34346933	-178.2662165	-144.7860407	EMD1
TCGTGGGGTGTCCTAA-1	singlet	1	-266.4649132	-92.24747741	-213.7320655	EMD1
TCGTGGGGTTGGGTTT-1	singlet	0	-207.6227492	-698.4189945	-630.659831	EMD1
TCGTGGGTCCTGCCAT-1	singlet	2	-210.8495331	-170.9707067	-83.35787716	EMD1
TCGTGGGTCGGTGTTA-1	singlet	0	-278.8717693	-855.6944676	-856.0207651	EMD1
TCTAACTAGCACCGAA-1	singlet	2	-841.1545532	-719.9311564	-328.5162065	EMD1
TCTAACTCAACGGCTC-1	singlet	0	-122.680431	-382.8894286	-338.1085302	EMD1
TCTAACTCAAGTGTCT-1	singlet	1	-215.9605409	-71.47533982	-160.4459969	EMD1
TCTAACTCAATAGTAG-1	singlet	1	-6994.845415	-2207.517133	-5122.123245	EMD1
TCTAACTCACAAGGTG-1	doublet	2/0	-379.5664755	-388.4179868	-222.4330469	EMD1
TCTAACTGTAGAGGAA-1	singlet	2	-778.9762458	-643.5569817	-302.4368958	EMD1
TCTAACTGTGTCGATT-1	singlet	2	-570.9361342	-648.3600956	-291.8197613	EMD1
TCTAACTTCTACGCAA-1	singlet	0	-24.02633692	-80.21815113	-86.01708605	EMD1
TCTACATAGGCCCACT-1	singlet	2	-734.9970613	-755.7492709	-302.0109946	EMD1
TCTACATGTCCGGTGT-1	singlet	0	-250.3757607	-770.1712305	-663.7898382	EMD1
TCTACATGTCCTCATC-1	singlet	1	-1727.023573	-637.3168638	-1459.735249	EMD1
TCTACATGTGTTGCCG-1	singlet	0	-70.64513454	-233.9545646	-283.9415627	EMD1
TCTACATGTTCGAAGG-1	singlet	2	-1429.233554	-1244.973644	-506.0673467	EMD1
TCTACATTCATTACGG-1	singlet	1	-7455.594574	-2511.966921	-6075.926786	EMD1
TCTACCGAGAGAGCCT-1	singlet	0	-533.7699663	-1500.216562	-1411.736699	EMD1
TCTACCGAGCAGTACG-1	singlet	2	-404.9858148	-342.9811373	-167.617153	EMD1
TCTACCGAGGTGCCAA-1	singlet	0	-24.06058216	-94.22768364	-77.95782425	EMD1
TCTACCGAGGTTGACG-1	singlet	0	-1146.180731	-3488.557652	-3476.149813	EMD1
TCTACCGCAATGGGTG-1	singlet	0	-1401.784282	-4345.790346	-4455.022086	EMD1
TCTACCGCACATGAAA-1	doublet	0/1	-1089.190192	-1700.976659	-1767.336582	EMD1
TCTACCGGTCCATACA-1	singlet	1	-2355.647715	-878.376915	-2071.264292	EMD1
TCTACCGGTGACAACG-1	singlet	0	-1077.453919	-3461.404331	-3331.290207	EMD1
TCTACCGGTTAGGCCC-1	singlet	2	-382.8106403	-325.8585265	-137.9814503	EMD1
TCTACCGTCCGATAAC-1	singlet	2	-1632.914311	-1245.346007	-573.5079834	EMD1
TCTATACAGAAGAACG-1	singlet	2	-664.6035078	-519.9351121	-214.3188427	EMD1
TCTATACAGAGTTCGG-1	singlet	2	-761.200751	-686.2623363	-320.9389885	EMD1
TCTATACAGCATCTTG-1	doublet	0/2	-773.6933444	-1467.92998	-1239.267141	EMD1
TCTATACAGTAAACTG-1	singlet	0	-234.5710236	-744.9077531	-646.5216588	EMD1
TCTATACAGTGGAATT-1	singlet	2	-246.9715109	-204.7011628	-137.0120045	EMD1
TCTATACCAACTGCTA-1	singlet	2	-649.2245375	-588.3698526	-264.5662991	EMD1
TCTATACCAAGGAGTC-1	singlet	0	-10.62413404	-53.72018409	-41.48487832	EMD1
TCTATACCAGGTTCCG-1	singlet	0	-208.0186346	-607.297817	-625.9540707	EMD1
TCTATACCATAGAGGC-1	singlet	1	-2443.85125	-677.1614465	-2031.423736	EMD1
TCTATACGTGATTCAC-1	singlet	2	-332.0182855	-284.0827303	-122.9144733	EMD1
TCTATACGTGGAATGC-1	singlet	2	-1270.231051	-1711.827926	-469.8223994	EMD1
TCTATACTCAAAGACA-1	singlet	1	-1494.724189	-610.0068771	-1148.138655	EMD1
TCTATACTCCTTGACC-1	singlet	2	-1183.941399	-946.5676301	-392.0402996	EMD1
TCTATCAAGGTCTGGA-1	singlet	0	-922.3013197	-2734.122185	-2674.196563	EMD1
TCTATCAAGTAACCGG-1	singlet	2	-192.7154673	-145.4036253	-71.65221385	EMD1
TCTATCAAGTTCGCAT-1	singlet	0	-21.33234022	-64.83460532	-53.8823651	EMD1
TCTATCACATGAGGGT-1	singlet	2	-3948.640519	-3009.283309	-1178.512499	EMD1
TCTATCAGTCAACATC-1	singlet	2	-608.2848754	-468.6187277	-205.6856769	EMD1
TCTATCAGTCCGGCAT-1	singlet	2	-1033.071862	-858.4294013	-420.2939552	EMD1
TCTATCAGTGAGCAGT-1	singlet	2	-279.359946	-220.0808676	-93.30711983	EMD1
TCTATCAGTTGGGTTT-1	singlet	0	-131.610539	-632.1275499	-496.1944942	EMD1
TCTATCAGTTTGGAAA-1	singlet	2	-372.5252157	-281.1543626	-133.0158924	EMD1
TCTATCATCACTCGAA-1	singlet	1	-4412.377846	-1301.07044	-3430.117469	EMD1
TCTATCATCCTACCAC-1	singlet	0	-518.2174995	-1592.947071	-1647.928739	EMD1
TCTCACGAGCGTCAGA-1	singlet	2	-456.9318722	-382.7857287	-140.3068917	EMD1
TCTCACGAGGACGCAT-1	singlet	0	-1597.261373	-5123.911248	-5166.175454	EMD1
TCTCACGAGTGCCTCG-1	singlet	2	-655.3621379	-529.3714208	-275.6073161	EMD1
TCTCACGAGTTAGTAG-1	singlet	0	-835.9884897	-2406.157689	-2340.720335	EMD1
TCTCACGCAAATCAGA-1	singlet	2	-550.6581893	-479.1146342	-223.2746493	EMD1
TCTCACGCAATCGTCA-1	singlet	0	-276.3749752	-679.2656233	-833.3184321	EMD1
TCTCACGCACATACGT-1	singlet	1	-3178.343978	-1011.012036	-2504.873755	EMD1
TCTCACGCACTATGTG-1	singlet	1	-2395.420693	-690.1741038	-1892.874832	EMD1
TCTCACGCAGACATCT-1	singlet	1	-3561.483326	-1335.53232	-2975.424307	EMD1
TCTCACGGTAAGGCCA-1	singlet	0	-961.541892	-2994.251882	-2693.261532	EMD1
TCTCACGGTCAACCAT-1	singlet	2	-1774.301507	-1330.129522	-501.64958	EMD1
TCTCACGGTGAGTTGG-1	singlet	2	-170.4279461	-135.7075367	-92.9676098	EMD1
TCTCACGGTTGCGAAG-1	singlet	2	-425.3399022	-357.7064098	-172.0668293	EMD1
TCTCACGTCAAGTCGT-1	singlet	1	-1017.293824	-436.2737253	-832.6466367	EMD1
TCTCACGTCCGAAATC-1	singlet	1	-1690.331903	-561.7035359	-1425.773148	EMD1

TCTCACGTCGACACTA-1	singlet	2	-2432.62766	-2207.480438	-618.0095011	EMD1
TCTCACGTCTATACGG-1	singlet	2	-490.3563788	-517.3680615	-240.0295879	EMD1
TCTCAGCAGGAGGTTC-1	singlet	1	-453.5691556	-169.2776308	-548.9234763	EMD1
TCTCAGCAGGTGGGTT-1	singlet	2	-740.5115326	-572.9211709	-249.8331081	EMD1
TCTCAGCAGTAGACAT-1	singlet	2	-566.9726472	-469.8328486	-226.8668774	EMD1
TCTCAGCCAACCGGAA-1	singlet	1	-3529.102456	-1121.758187	-2856.716982	EMD1
TCTCAGCGTCACGTGC-1	singlet	2	-332.0781047	-234.7757895	-136.181049	EMD1
TCTCAGCGTGTAAACA-1	singlet	0	-156.5733738	-356.9625449	-364.0995802	EMD1
TCTCAGCGTTTCGGCG-1	singlet	2	-3370.423376	-2562.683326	-987.5090701	EMD1
TCTCAGCTCCACCTCA-1	singlet	2	-5605.515639	-4451.82362	-2004.094249	EMD1
TCTCAGCTCCCTTGTG-1	singlet	1	-539.3106662	-256.9438578	-463.4122172	EMD1
TCTCCGAAGAGTCCGA-1	singlet	0	-307.5360051	-785.4647848	-870.4876411	EMD1
TCTCCGAAGCACGTCC-1	singlet	2	-100.1005094	-84.79164071	-31.91303638	EMD1
TCTCCGACAAATGGAT-1	singlet	1	-290.2040993	-89.35533433	-219.4385482	EMD1
TCTCCGACACGTACAT-1	singlet	1	-1159.79608	-402.4713825	-851.4714525	EMD1
TCTCCGACAGACAAAT-1	singlet	2	-726.8980996	-510.8360133	-244.8082085	EMD1
TCTCCGACATCTGGGC-1	singlet	1	-1278.292551	-445.4244074	-993.036602	EMD1
TCTCCGACATGAGTAA-1	singlet	1	-1354.83572	-478.2678018	-1129.746919	EMD1
TCTCCGAGTCTACTGA-1	singlet	0	-1796.328049	-5977.428451	-5475.863621	EMD1
TCTCCGAGTTATTCCT-1	singlet	1	-2445.183906	-852.3737961	-2083.199804	EMD1
TCTCCGATCCCGAAAT-1	singlet	2	-601.4602154	-447.1240315	-250.7540589	EMD1
TCTCCGATCCCTCTTT-1	singlet	2	-2115.084294	-2295.591559	-778.3970419	EMD1
TCTCTGGAGAAACTCA-1	singlet	0	-42.65159093	-96.80636872	-81.70402747	EMD1
TCTCTGGAGAAGCCTG-1	singlet	2	-525.724537	-419.0746077	-182.2007287	EMD1
TCTCTGGAGCACTTTG-1	singlet	2	-384.9515063	-382.6135391	-153.2754134	EMD1
TCTCTGGAGGATACAT-1	singlet	1	-5107.507809	-1253.201326	-4068.28983	EMD1
TCTCTGGAGGCATCTT-1	singlet	1	-1676.008566	-545.8855275	-1428.169292	EMD1
TCTCTGGAGGTTAGTA-1	singlet	2	-668.8242436	-517.6604765	-236.5229557	EMD1
TCTCTGGCACGGATCC-1	singlet	2	-326.1266987	-311.9305964	-175.9156637	EMD1
TCTCTGGGTCTAGTGT-1	singlet	2	-357.181228	-332.410636	-144.1931339	EMD1
TCTCTGGTCGTCTCAC-1	singlet	0	-150.2134586	-723.3054329	-577.6515456	EMD1
TCTGCCAAGCCGGATA-1	singlet	1	-509.7512655	-183.1704459	-468.4787651	EMD1
TCTGCCAAGTACAGCG-1	singlet	1	-170.3369058	-33.71696227	-90.96578973	EMD1
TCTGCCACACTTGTCC-1	singlet	2	-918.8475739	-822.7084719	-341.9150473	EMD1
TCTGCCACAGGTAGTG-1	singlet	0	-1441.107811	-5127.129259	-5069.683142	EMD1
TCTGCCACATGGTGGA-1	singlet	2	-447.6431995	-293.9328904	-137.2887074	EMD1
TCTGCCAGTTTACCTT-1	singlet	2	-571.881282	-514.8556656	-212.8704512	EMD1
TCTGCCATCCGGTAAT-1	doublet	0/1	-1991.380547	-3158.706592	-3435.958261	EMD1
TCTGCCATCTCTTGCG-1	singlet	1	-390.4744627	-144.2867066	-382.6930604	EMD1
TCTGCCATCTTCCACG-1	singlet	0	-147.125087	-485.4747968	-499.320675	EMD1
TCTGGCTCACAGCCAC-1	singlet	0	-395.1278602	-976.1833571	-903.2195952	EMD1
TCTGGCTGTACAGAAT-1	singlet	0	-203.9590132	-707.2573449	-770.2643074	EMD1
TCTGGCTGTAGCACAG-1	singlet	2	-432.1947818	-351.7954238	-153.3661404	EMD1
TCTGGCTGTCACCCTT-1	doublet	45323	-5067.501758	-2918.130851	-2903.159147	EMD1
TCTGGCTGTCATCCCT-1	singlet	2	-79.75023897	-78.14272574	-31.14404553	EMD1
TCTGGCTGTCCGACGT-1	singlet	1	-8993.484888	-2694.104593	-7224.030651	EMD1
TCTGGCTGTCGTTATG-1	singlet	2	-2946.282804	-2181.431847	-981.4418129	EMD1
TCTGGCTTCCCGTAAA-1	singlet	2	-711.8465014	-641.1183653	-276.7977426	EMD1
TCTGGCTTCTAGTACG-1	singlet	2	-226.1833305	-193.3200203	-69.84445066	EMD1
TCTGGCTTCTCACCCA-1	singlet	1	-706.5264725	-267.3260531	-647.9045276	EMD1
TCTGGCTTCTCCTGCA-1	singlet	2	-663.63722	-637.5295541	-241.8004974	EMD1
TCTGTCGAGCCATTTG-1	singlet	0	-211.4953851	-640.2697495	-578.5071043	EMD1
TCTGTCGAGCTCGGCT-1	singlet	1	-549.9717316	-161.1163574	-358.0907485	EMD1
TCTGTCGAGGTTGGTG-1	singlet	2	-3872.169241	-2955.598525	-1143.894077	EMD1
TCTGTCGAGTCTCTGA-1	singlet	2	-385.5891822	-337.8390821	-167.6031264	EMD1
TCTGTCGAGTCTGCGC-1	singlet	2	-235.4514467	-184.6119998	-88.16613048	EMD1
TCTGTCGAGTGGTTGG-1	singlet	0	-288.4568658	-956.4113264	-956.0142968	EMD1
TCTGTCGCAAGTGACG-1	singlet	0	-7.84368285	-14.35970875	-67.97104953	EMD1
TCTGTCGGTAGTCTTG-1	singlet	1	-2129.470128	-716.6586184	-1638.827264	EMD1
TCTGTCGGTATCTCTT-1	singlet	1	-5808.625604	-1647.731404	-4671.777155	EMD1
TCTGTCGGTCAAGGCA-1	singlet	1	-608.3493491	-225.9531308	-603.2989968	EMD1
TCTGTCGGTCAGGTAG-1	singlet	1	-3261.869623	-872.5639976	-2547.469907	EMD1
TCTGTCGGTCGCCACA-1	singlet	0	-213.648582	-920.8198203	-816.7293372	EMD1
TCTGTCGTCCCTTGTG-1	singlet	2	-1041.243247	-775.3995927	-317.9932225	EMD1
TCTTAGTAGCCATATC-1	singlet	1	-415.5051856	-119.1804785	-257.3997782	EMD1
TCTTAGTAGCTGCCTG-1	singlet	0	-199.8040166	-738.2049561	-710.0269523	EMD1
TCTTAGTCAAGACTGG-1	singlet	0	-541.5568741	-1500.777979	-1590.619255	EMD1
TCTTAGTCACACCGAC-1	singlet	2	-548.3689768	-391.8670242	-217.9320473	EMD1
TCTTAGTGTACTGCGC-1	singlet	2	-788.9278102	-604.3845931	-276.990731	EMD1
TCTTAGTGTTAGAAAC-1	singlet	2	-2208.944375	-1904.056716	-784.8149652	EMD1
TCTTAGTTCACTGGTA-1	singlet	2	-683.138018	-623.1586513	-285.4319278	EMD1
TCTTAGTTCTAGGCAT-1	singlet	2	-837.8464848	-685.5175722	-309.9542733	EMD1
TCTTCCTAGATGACAT-1	singlet	2	-420.2625614	-385.0763707	-156.7952465	EMD1
TCTTCCTCAACCAACT-1	singlet	1	-300.0445363	-107.3169401	-223.4834126	EMD1
TCTTCCTCAACCTAAC-1	unassigned	1	-1.890340548	-1.341690971	-1.687395909	EMD1
TCTTCCTCAAGCAATA-1	singlet	2	-527.3407188	-416.23326	-242.393638	EMD1
TCTTCCTCAGGGAATC-1	singlet	1	-185.4649222	-45.63342888	-128.7392338	EMD1
TCTTCCTCATATCGGT-1	singlet	1	-1767.850011	-511.1082497	-1376.289602	EMD1
TCTTCCTGTACAAACA-1	singlet	2	-452.7795138	-441.8560767	-194.3825759	EMD1
TCTTCCTGTGGATCAG-1	singlet	2	-563.3897054	-543.1342807	-284.2720472	EMD1
TCTTCCTGTTCTCCAC-1	singlet	0	-478.0213199	-1938.0229	-1723.316686	EMD1
TCTTCCTTCACCTCTG-1	singlet	2	-670.1574431	-590.0344021	-300.0681063	EMD1
TCTTCCTTCAGCTTCC-1	singlet	2	-384.2482628	-287.9478497	-135.5875016	EMD1
TCTTCCTTCATTTGGG-1	singlet	0	-335.1473428	-984.9527716	-915.6177893	EMD1
TCTTCCTTCGGTTCAA-1	singlet	0	-3105.101143	-8814.311404	-8886.986987	EMD1
TCTTCCTTCGTTCAGA-1	singlet	2	-393.7291353	-371.364991	-176.1169998	EMD1
TCTTCCTTCGTTCTGC-1	singlet	1	-94.5386081	-29.42619462	-63.53951881	EMD1
TCTTGCGAGCTTCTAG-1	singlet	2	-351.3431794	-332.3755803	-144.9333744	EMD1
TCTTGCGCAACTCCAA-1	singlet	2	-234.5109757	-251.9704883	-119.686778	EMD1
TCTTGCGCACTGGATT-1	singlet	2	-2623.72594	-2176.837116	-985.0377337	EMD1
TCTTGCGCAGCCGGTT-1	singlet	1	-3641.870562	-866.9360189	-3051.122178	EMD1
TCTTGCGGTCGAAACG-1	singlet	0	-572.171414	-1671.912838	-1751.85613	EMD1
TCTTGCGGTCTACAGT-1	singlet	0	-241.0699871	-1151.73663	-1134.18329	EMD1
TCTTTGAAGAGCAACC-1	singlet	0	-27.55129145	-71.78961249	-53.23464653	EMD1
TCTTTGAAGGTTCCAT-1	singlet	2	-1655.398063	-1394.739391	-579.050428	EMD1
TCTTTGACAAGTCGTT-1	singlet	1	-14154.80231	-4514.635575	-11248.55646	EMD1
TCTTTGACAATTGCAC-1	singlet	1	-1369.57656	-516.3861745	-1028.654295	EMD1
TCTTTGACACGCACCA-1	singlet	2	-570.358492	-420.9715013	-223.2904902	EMD1
TCTTTGACATCAGCTA-1	singlet	0	-245.5979579	-681.0336345	-790.6333027	EMD1
TCTTTGACATGCTGCG-1	singlet	1	-1548.086857	-485.0129131	-1257.181761	EMD1
TCTTTGAGTACAAACA-1	singlet	0	-354.9743847	-1053.587846	-919.2891079	EMD1
TCTTTGAGTTAGAAGT-1	singlet	2	-680.7616171	-582.3642163	-267.5980345	EMD1
TCTTTGATCCACTGGG-1	singlet	0	-172.6154137	-419.0562733	-362.7514753	EMD1
TCTTTGATCGCATTAG-1	singlet	0	-594.3314387	-2088.801078	-2876.473566	EMD1
TCTTTGATCGGCTGTG-1	singlet	2	-526.8535368	-443.4129517	-191.4129402	EMD1
TGAACGTAGGATATGT-1	singlet	0	-1281.230738	-4297.237459	-4224.472677	EMD1
TGAACGTCAGACGATG-1	singlet	2	-498.8343032	-436.0459526	-224.6554824	EMD1
TGAACGTCATGCGTGC-1	singlet	2	-882.5713938	-630.9471418	-309.3742453	EMD1
TGAACGTGTAACATCC-1	singlet	2	-605.1507887	-552.1612668	-229.9523256	EMD1
TGAACGTGTCAGGAGT-1	doublet	0/1	-40.81740024	-81.81760502	-80.0651733	EMD1

TGAACGTGTGGCCCAT-1	singlet	2	-527.2805025	-461.6359282	-197.5180285	EMD1
TGAACGTGTTCGGTCG-1	singlet	2	-207.6931389	-178.3146267	-85.74091909	EMD1
TGAACGTTCAACTGGT-1	singlet	2	-659.3084428	-548.0406657	-250.0138112	EMD1
TGAACGTTCTTACTGT-1	singlet	0	-1580.830466	-5187.706691	-4831.320518	EMD1
TGAACGTTCTTGCGCT-1	singlet	2	-484.5147427	-399.7065837	-199.7677471	EMD1
TGAATCGAGAACCGCA-1	singlet	2	-607.0449113	-579.4706961	-251.9466716	EMD1
TGAATCGAGAAGAACG-1	singlet	2	-1499.43211	-1288.970505	-498.2841995	EMD1
TGAATCGCAACTTGCA-1	singlet	2	-578.1209448	-413.5711672	-216.6148082	EMD1
TGAATCGCAGAATTCC-1	singlet	1	-1174.567205	-436.6702594	-820.5288986	EMD1
TGAATCGCAGCAATTC-1	singlet	2	-681.9528636	-415.9291419	-249.6709807	EMD1
TGAATCGGTTCCTACC-1	singlet	2	-889.9460078	-695.9914048	-332.9300239	EMD1
TGAATCGGTTGGTACT-1	singlet	0	-200.6320889	-583.2261549	-711.0554799	EMD1
TGAATCGTCTCCAATT-1	singlet	1	-4332.401194	-1471.011852	-3829.419432	EMD1
TGAATGCAGGTTGAGC-1	singlet	0	-309.1408839	-1004.910936	-949.5837693	EMD1
TGAATGCCAATTGAAG-1	singlet	2	-266.0554895	-267.1663289	-135.0623621	EMD1
TGAATGCCAGTGTATC-1	singlet	0	-190.2047382	-574.8819896	-453.6417899	EMD1
TGAATGCCATACCATG-1	singlet	2	-447.0352667	-340.5576897	-179.6293147	EMD1
TGAATGCGTGCAACGA-1	singlet	0	-67.0545364	-186.5351349	-162.9197874	EMD1
TGAATGCGTGTTCGTA-1	singlet	2	-1688.62495	-1451.129774	-682.6091379	EMD1
TGAATGCTCAATCAGC-1	singlet	0	-22.97712345	-28.45863206	-211.9489718	EMD1
TGAATGCTCCTAAACG-1	singlet	1	-495.8527672	-156.4350812	-408.663799	EMD1
TGACAGTAGTTACTCG-1	singlet	2	-1327.047175	-1220.015111	-656.051562	EMD1
TGACAGTAGTTGAAGT-1	singlet	2	-585.2702239	-530.9256663	-244.757001	EMD1
TGACAGTGTACAGTCT-1	singlet	2	-611.4049171	-491.9049125	-206.1070397	EMD1
TGACAGTGTCTCCTGT-1	singlet	1	-2217.18764	-811.9494461	-1670.304834	EMD1
TGACAGTGTGTCACAT-1	singlet	1	-2152.809204	-681.2915646	-1645.403961	EMD1
TGACAGTTCATCCTAT-1	singlet	0	-179.8435982	-565.4910225	-480.4266455	EMD1
TGACAGTTCCGAGCTG-1	singlet	1	-109.6891867	-44.93355596	-117.7102418	EMD1
TGACAGTTCGGAAACG-1	singlet	2	-1208.440173	-1061.762177	-416.0280196	EMD1
TGACCCTAGAAATTCG-1	singlet	1	-43.80534351	-13.44017414	-32.10110797	EMD1
TGACCCTAGGTAAGAG-1	singlet	0	-263.043746	-760.3997347	-715.002621	EMD1
TGACCCTCACGTACAT-1	singlet	1	-2425.46736	-703.7495328	-1710.569567	EMD1
TGACCCTCAGAGGCAT-1	singlet	2	-423.7407619	-308.8379696	-166.1282117	EMD1
TGACCCTCAGCGCTTG-1	singlet	1	-476.9334375	-194.6724654	-436.8602025	EMD1
TGACCCTCAGCTACCG-1	singlet	2	-1447.827071	-1165.603193	-538.3156972	EMD1
TGACCCTGTATGCTTG-1	singlet	2	-519.9459472	-405.8462698	-199.9632853	EMD1
TGACCCTGTGTTCCAA-1	singlet	2	-711.526939	-572.3600854	-270.4682847	EMD1
TGACCCTTCTCTAAGG-1	singlet	2	-510.4611787	-418.2190668	-192.6333672	EMD1
TGACCCTTCTGCTTTA-1	singlet	2	-880.316324	-777.9512927	-341.4163252	EMD1
TGACGCGAGGAATGTT-1	singlet	1	-934.7400464	-279.7658989	-721.4964373	EMD1
TGACGCGAGGCTTAGG-1	singlet	0	-625.252084	-1825.413894	-1861.112708	EMD1
TGACGCGAGTGCACCC-1	singlet	2	-573.6402731	-586.2152439	-256.2712569	EMD1
TGACGCGCAAACTAAG-1	singlet	1	-1704.795879	-610.3542105	-1339.69312	EMD1
TGACGCGCAACTGATC-1	doublet	2/0	-2531.259939	-3404.943295	-1903.826861	EMD1
TGACGCGCAAGACTGG-1	singlet	2	-6855.000684	-5714.668675	-2339.986132	EMD1
TGACGCGCATCGATAC-1	singlet	1	-165.8465756	-40.74970191	-117.6101725	EMD1
TGACGCGGTGGACAGT-1	singlet	2	-245.4716796	-206.7864916	-107.8859854	EMD1
TGACGCGGTGTATACC-1	singlet	1	-3435.620937	-956.9037958	-2718.71903	EMD1
TGACGCGGTTTGGAAA-1	singlet	2	-453.981765	-393.7107899	-167.7459978	EMD1
TGACGCGTCAGACATC-1	singlet	2	-507.0666312	-420.3839668	-217.0975967	EMD1
TGACGCGTCAGTCACA-1	singlet	2	-664.30477	-575.4153364	-208.3515067	EMD1
TGACGCGTCTCGCAGG-1	singlet	2	-209.17821	-182.4850981	-85.04579666	EMD1
TGACTCCAGATGCCGA-1	singlet	1	-602.4245252	-197.7634607	-524.8420744	EMD1
TGACTCCAGCGACTAG-1	singlet	2	-4355.467431	-3722.879841	-1232.783899	EMD1
TGACTCCAGGCACAAC-1	singlet	0	-534.6003193	-1753.404933	-1599.044014	EMD1
TGACTCCCAGTTACCA-1	singlet	2	-117.9193749	-99.82325497	-44.99446804	EMD1
TGACTCCGTAACATCC-1	singlet	2	-628.3157679	-560.8435573	-205.7403734	EMD1
TGACTCCGTACCTGTA-1	singlet	2	-786.5162146	-679.2117513	-287.0410148	EMD1
TGACTCCGTACTCGAT-1	singlet	2	-895.9812359	-848.2681141	-346.4566042	EMD1
TGACTCCGTGCCCACA-1	singlet	2	-4204.83242	-3295.589471	-1265.223371	EMD1
TGACTCCGTGTTTACG-1	singlet	0	-360.605057	-1132.310587	-1155.867774	EMD1
TGACTCCTCGTACACA-1	singlet	0	-1193.942559	-3481.123755	-3340.239126	EMD1
TGACTCCTCTAACGGT-1	singlet	2	-1289.092986	-1116.593871	-459.4704929	EMD1
TGACTCCTCTACAGGT-1	singlet	1	-1854.019827	-561.3087529	-1373.003498	EMD1
TGAGACTAGTCTAGAA-1	singlet	0	-587.5462191	-1677.236034	-1613.000948	EMD1
TGAGACTCAAAGGTTA-1	singlet	1	-543.8091688	-247.3388343	-433.6028222	EMD1
TGAGACTCAAGCGCAA-1	singlet	1	-258.298732	-103.2769052	-203.2290603	EMD1
TGAGACTCAAGTCGTT-1	singlet	2	-547.8794432	-427.3806241	-197.594186	EMD1
TGAGACTCACATCCCT-1	singlet	0	-1712.091993	-6120.751345	-5832.18182	EMD1
TGAGACTGTCGTCATA-1	singlet	0	-4.642154124	-7.630617404	-8.062489002	EMD1
TGAGACTTCCCACAGG-1	singlet	2	-1050.141901	-938.2432529	-379.5829523	EMD1
TGAGCATAGAAATTGC-1	singlet	1	-2603.314685	-841.0036832	-2114.749205	EMD1
TGAGCATAGGCACAAC-1	singlet	0	-365.4930184	-1577.141208	-1565.078274	EMD1
TGAGCATAGTACTCGT-1	singlet	1	-1325.961671	-442.1115499	-1073.285271	EMD1
TGAGCATAGTGAGTGC-1	singlet	0	-141.8878505	-329.2042954	-264.5620136	EMD1
TGAGCATGTAGTGTGG-1	singlet	0	-1219.776205	-3633.057743	-3584.175944	EMD1
TGAGCATGTCCAGCAC-1	singlet	0	-768.5068364	-2527.191173	-2544.811559	EMD1
TGAGCATGTGCTAGCC-1	doublet	2/0	-2943.089473	-3064.482626	-1778.523352	EMD1
TGAGCATGTGTTGATC-1	singlet	1	-3019.795572	-835.1625847	-2707.88038	EMD1
TGAGCATTCAGCTAGT-1	singlet	0	-309.7334271	-782.2937167	-774.198545	EMD1
TGAGCATTCTAAGGAA-1	singlet	0	-637.4664412	-1936.071693	-1950.028519	EMD1
TGAGCGCAGCCAACCC-1	singlet	1	-405.5511925	-142.3273002	-240.5455324	EMD1
TGAGCGCAGTCCCAGC-1	singlet	0	-785.5572171	-2380.406729	-2315.676046	EMD1
TGAGCGCAGTTTCGGT-1	singlet	2	-408.9347701	-323.8524675	-160.7544026	EMD1
TGAGCGCCAACATCGT-1	singlet	1	-7845.181678	-1809.967128	-5885.317634	EMD1
TGAGCGCCAGAGGCTA-1	singlet	2	-1605.328744	-1355.739863	-453.1899945	EMD1
TGAGCGCGTCGCTGCA-1	singlet	2	-180.2732762	-157.1827244	-77.31614536	EMD1
TGAGCGCGTGCAGTGA-1	singlet	2	-108.9190427	-64.22045011	-24.16296169	EMD1
TGAGCGCTCATGGCCG-1	singlet	2	-316.3092609	-304.5697705	-148.046998	EMD1
TGAGCGCTCGGCTGGT-1	singlet	2	-601.2979292	-526.3298315	-243.171538	EMD1
TGAGGAGAGATTTGCC-1	singlet	2	-1857.875345	-1443.342841	-649.913876	EMD1
TGAGGAGAGCATACTC-1	singlet	2	-288.2417016	-236.7809563	-96.92572202	EMD1
TGAGGAGAGCTGTACT-1	singlet	0	-110.4250548	-412.6016259	-446.5250225	EMD1
TGAGGAGGTCACATTG-1	singlet	2	-549.4821172	-494.6656371	-230.8084631	EMD1
TGAGGAGTCCATACTT-1	singlet	2	-4896.285772	-4268.938536	-1592.486919	EMD1
TGAGGGAAGATTGAGT-1	singlet	2	-435.760676	-346.5979743	-160.0283344	EMD1
TGAGGGAAGGATGTTA-1	singlet	2	-514.9703709	-485.5553238	-209.2208813	EMD1
TGAGGGACACGCGGTT-1	singlet	2	-384.7613064	-345.5672274	-139.851555	EMD1
TGAGGGAGTCGCAACC-1	singlet	2	-502.5753658	-364.5221316	-208.5169675	EMD1
TGAGGGAGTTGCTGAT-1	singlet	2	-345.875999	-297.6903233	-122.4380069	EMD1
TGAGGGATCAGAGTGG-1	singlet	0	-722.5609378	-2231.277639	-2117.053513	EMD1
TGAGGGATCGAAGAAT-1	singlet	2	-164.6249795	-131.3721436	-36.89217856	EMD1
TGAGGGATCTCCCTAG-1	singlet	2	-3017.025765	-2446.687044	-858.8932407	EMD1
TGAGGGATCTTACACT-1	singlet	2	-5276.053377	-4298.338334	-1782.58227	EMD1
TGAGGTTGTAACTGCT-1	singlet	0	-400.923061	-1241.191908	-1179.494711	EMD1
TGAGGTTGTACGGGAT-1	singlet	1	-463.1127357	-167.9882422	-432.3524515	EMD1
TGAGGTTGTGACACGA-1	singlet	2	-478.4681974	-354.1091521	-187.5196115	EMD1
TGAGTCAAGACTGAGC-1	singlet	2	-728.447403	-561.158562	-252.5625946	EMD1
TGAGTCAAGACTGTTC-1	singlet	2	-753.6627485	-654.5559647	-343.993957	EMD1
TGAGTCAAGCTACGTT-1	singlet	0	-847.4223534	-2899.754047	-2501.613641	EMD1

TGAGTCAAGGTGCAGT-1	singlet	2	-228.6494524	-213.8246977	-101.9809706	EMD1
TGAGTCACATAATGCC-1	singlet	2	-509.5355847	-400.4280337	-198.8666082	EMD1
TGAGTCACATCTCAAG-1	singlet	1	-619.0112927	-238.3312425	-590.7290023	EMD1
TGAGTCAGTGGACCTC-1	singlet	2	-386.9688804	-338.1538169	-158.1633376	EMD1
TGAGTCAGTGGGCTCT-1	singlet	0	-105.1468227	-322.8731473	-291.6176661	EMD1
TGAGTCAGTTGCCGAC-1	singlet	0	-222.0823649	-552.9244757	-479.3042251	EMD1
TGAGTCATCTAGGAAA-1	singlet	2	-383.1181932	-341.6187544	-155.8958897	EMD1
TGATCAGAGCCTCTGG-1	singlet	0	-119.4424077	-407.6508331	-398.7690448	EMD1
TGATCAGAGCCTTTGA-1	singlet	1	-3497.851983	-1065.525478	-2549.337881	EMD1
TGATCAGCACAGTGAG-1	singlet	2	-3277.369419	-2680.433437	-1084.274526	EMD1
TGATCAGCACTATCGA-1	doublet	1/0	-4232.765227	-3177.971061	-5227.600871	EMD1
TGATCAGGTATAGGAT-1	singlet	1	-603.1934821	-274.6792669	-768.9729965	EMD1
TGATCAGGTGTCCACG-1	singlet	0	-103.6147627	-303.4308537	-358.1477356	EMD1
TGATCAGTCACCATCC-1	singlet	1	-2473.726212	-714.6008728	-1969.674274	EMD1
TGATCAGTCGAGTCTA-1	singlet	2	-2020.609373	-1749.529518	-741.3417602	EMD1
TGATCAGTCTTGAGCA-1	doublet	45323	-442.5487467	-338.6587648	-235.71582	EMD1
TGATCTTAGAGAACCC-1	singlet	0	-322.2201877	-957.6939589	-896.8263552	EMD1
TGATCTTAGCTGACTT-1	singlet	1	-219.4729521	-73.48341409	-205.5057501	EMD1
TGATCTTAGGCCCACT-1	singlet	2	-534.9097523	-433.797898	-204.09863	EMD1
TGATCTTCAGTTGCGC-1	singlet	1	-795.7188215	-313.6009662	-614.509298	EMD1
TGATCTTCATAGCACT-1	singlet	1	-211.375835	-92.64121378	-196.847647	EMD1
TGATCTTCATCCAATG-1	singlet	2	-636.8037425	-513.3551058	-277.3963906	EMD1
TGATCTTCATCTCAAG-1	singlet	0	-288.9755275	-878.2715053	-850.3821161	EMD1
TGATCTTGTAGTTACC-1	singlet	2	-348.0793485	-294.7635297	-138.8232456	EMD1
TGATCTTGTGATAGTA-1	singlet	1	-154.5823968	-41.69686939	-132.8587419	EMD1
TGATCTTGTGATTCAC-1	singlet	0	-1114.224171	-3914.575583	-3511.725843	EMD1
TGATCTTTCCTATGGA-1	singlet	2	-645.7998483	-439.2561054	-230.480723	EMD1
TGATCTTTCTGTAAGC-1	singlet	2	-733.0901459	-624.6715231	-325.1211015	EMD1
TGATGCAAGGTAGACC-1	singlet	1	-1960.342828	-604.3299253	-1567.101831	EMD1
TGATGCAAGTGCAGCA-1	doublet	2/0	-1606.376088	-1741.02156	-1127.835056	EMD1
TGATGCAGTCCCGTGA-1	singlet	2	-418.5965379	-405.7573197	-176.7954312	EMD1
TGATGCAGTTCCACGG-1	singlet	2	-656.1171267	-457.633716	-216.6314772	EMD1
TGATGCATCAAGCGTT-1	singlet	0	-990.8500945	-3393.570878	-3326.410134	EMD1
TGATGCATCAATCTTC-1	singlet	0	-594.794671	-1941.591729	-2005.103232	EMD1
TGATGCATCATACGGT-1	singlet	1	-1416.596562	-517.1043071	-1114.232831	EMD1
TGATGCATCCGTGTAA-1	singlet	0	-491.6044447	-2255.426457	-1938.384411	EMD1
TGATGGTAGCGCCTAC-1	singlet	0	-272.1392352	-668.9341861	-789.3308504	EMD1
TGATGGTCAAACTAGA-1	singlet	2	-752.0470545	-585.2476356	-281.0995547	EMD1
TGATGGTCAAGAGAGA-1	singlet	2	-498.0315957	-423.0359179	-181.232561	EMD1
TGATGGTCAAGTCCCG-1	singlet	2	-938.7736041	-739.888187	-330.985499	EMD1
TGATGGTCAGCTCTGG-1	singlet	0	-234.7468312	-655.5089115	-638.9334681	EMD1
TGATGGTCAGTCGCTG-1	singlet	1	-6456.079456	-1626.199229	-5050.873852	EMD1
TGATGGTCATGGGTCC-1	singlet	0	-22.64433916	-106.4937595	-119.2900694	EMD1
TGATGGTGTTCTCGCT-1	singlet	2	-542.4492462	-433.3022174	-214.5073911	EMD1
TGATGGTGTTCTCTCG-1	singlet	1	-4333.728489	-1405.797492	-3348.81398	EMD1
TGATGGTTCAGATGCT-1	doublet	2/0	-699.7613505	-789.6988617	-430.7930546	EMD1
TGATGGTTCCAGTACA-1	singlet	2	-441.6713182	-408.1087742	-168.6096423	EMD1
TGATGGTTCGCATGAT-1	singlet	2	-621.9240421	-504.405931	-217.8780088	EMD1
TGATGGTTCGCCTAGG-1	singlet	1	-1110.92649	-348.6988121	-916.130976	EMD1
TGATTCTAGGCTAAAT-1	singlet	1	-887.3394144	-311.288106	-822.5036114	EMD1
TGATTCTCACACAGCC-1	singlet	0	-395.8906875	-1486.838125	-1467.706308	EMD1
TGATTCTCAGACCTAT-1	singlet	2	-750.8622662	-756.3293914	-310.9912584	EMD1
TGATTCTGTCGCACAC-1	singlet	2	-543.0162443	-479.6771045	-187.7212141	EMD1
TGATTCTGTGCTAGCC-1	singlet	2	-672.7379157	-555.2003314	-283.7538079	EMD1
TGATTCTGTGGCTGAA-1	singlet	1	-53.88564353	-18.61033057	-46.93850315	EMD1
TGATTCTGTTGACTAC-1	singlet	0	-41.92596836	-164.0288468	-121.647445	EMD1
TGATTCTTCTACGGGC-1	singlet	0	-487.8396293	-1556.681946	-1484.296128	EMD1
TGATTCTTCTGGAGAG-1	singlet	2	-1144.635503	-1201.795781	-435.3043898	EMD1
TGATTTCAGAGTGTTA-1	singlet	1	-69.14062743	-24.44408412	-54.1133298	EMD1
TGATTTCAGATTGCGG-1	singlet	2	-533.7377452	-435.4075398	-224.9318457	EMD1
TGATTTCAGGTAAGAG-1	singlet	0	-72.2053135	-198.8921978	-158.0215457	EMD1
TGATTTCCACAGTCGC-1	singlet	2	-487.5427847	-446.8073592	-183.8737475	EMD1
TGATTTCCAGCGAGTA-1	singlet	1	-2124.267961	-638.0877102	-1785.837506	EMD1
TGATTTCGTCAAAGTA-1	singlet	0	-88.51523438	-248.1768521	-250.5212408	EMD1
TGATTTCGTTGTGGAG-1	singlet	1	-132.7458716	-51.2229411	-81.61193679	EMD1
TGATTTCTCAAATAGG-1	singlet	0	-379.8320286	-1180.521007	-1043.379336	EMD1
TGATTTCTCATACAGC-1	singlet	2	-430.4049366	-359.6325869	-190.5620209	EMD1
TGATTTCTCCGTAGTA-1	singlet	1	-8032.828666	-2156.532074	-6415.29454	EMD1
TGCACGGCACATGACT-1	singlet	2	-1900.915062	-1531.533422	-727.1791518	EMD1
TGCACGGCAGCGACAA-1	singlet	1	-2001.610644	-806.8425906	-1794.993834	EMD1
TGCACGGGTTTAGAGA-1	singlet	0	-663.0536185	-1880.717335	-1728.663358	EMD1
TGCACGGTCACCACAA-1	singlet	0	-970.8887577	-2973.9384	-3000.870696	EMD1
TGCACGGTCCGCATAA-1	singlet	2	-1960.613027	-1615.881849	-598.106548	EMD1
TGCACGGTCGAACGGA-1	singlet	2	-438.7364727	-380.8842838	-179.2172764	EMD1
TGCACGGTCGCACTCT-1	singlet	2	-567.4031738	-489.525448	-216.1017748	EMD1
TGCACGGTCGTAACCA-1	doublet	45293	-5702.185208	-2909.524558	-3701.780933	EMD1
TGCAGATGTGGCTCTG-1	singlet	1	-1921.097084	-675.4763777	-1480.731764	EMD1
TGCAGATGTTCTGACA-1	singlet	2	-274.2915988	-223.2851368	-137.9128031	EMD1
TGCAGATTCACCGACG-1	singlet	0	-1439.209628	-4301.461831	-4197.364209	EMD1
TGCAGATTCGATCCAA-1	singlet	1	-1632.515367	-561.2460211	-1226.194453	EMD1
TGCAGATTCGTCGCTT-1	singlet	0	-653.1062647	-2084.627316	-1993.084544	EMD1
TGCAGGCAGGTGCTAG-1	singlet	2	-4503.797477	-3810.703408	-1147.383747	EMD1
TGCAGGCCAGGTGTGA-1	singlet	0	-793.6570961	-2226.258264	-2332.711364	EMD1
TGCAGGCCATGCAGCC-1	singlet	2	-444.8269825	-405.6789587	-191.233226	EMD1
TGCAGGCGTGTCTAAC-1	singlet	2	-1114.209506	-1050.981482	-391.4740432	EMD1
TGCAGGCTCATCCCGT-1	singlet	2	-377.8499537	-361.863218	-175.866903	EMD1
TGCAGGCTCGCTAAAC-1	singlet	2	-579.4521565	-499.1235347	-212.8047507	EMD1
TGCAGGCTCTCTAAGG-1	singlet	2	-723.0636158	-564.1091625	-266.9609284	EMD1
TGCAGTAAGAGCCGTA-1	singlet	2	-929.0782143	-900.1781093	-316.711837	EMD1
TGCAGTAAGTAATCCC-1	singlet	1	-414.0965047	-167.2650162	-329.1011638	EMD1
TGCAGTACACGGCACT-1	singlet	2	-2098.407592	-1655.583035	-690.1385198	EMD1
TGCAGTACAGCGGTTC-1	singlet	0	-2298.330408	-7518.251539	-8087.479906	EMD1
TGCAGTAGTGAAGCGT-1	singlet	0	-350.0690921	-1412.109516	-1148.343166	EMD1
TGCAGTAGTGAATGTA-1	singlet	0	-954.8431081	-3889.962825	-3676.313248	EMD1
TGCAGTATCCGGTTCT-1	singlet	2	-263.987167	-238.8768471	-115.2272689	EMD1
TGCAGTATCGGTTAGT-1	singlet	2	-494.5941133	-430.9684468	-214.7928168	EMD1
TGCAGTATCTATACTC-1	singlet	0	-396.7678808	-1058.411861	-1146.245502	EMD1
TGCAGTATCTGCCTGT-1	singlet	2	-1340.754534	-1149.202793	-489.2692721	EMD1
TGCATCCAGCGTCTGC-1	singlet	1	-748.8245002	-243.7715822	-672.7204193	EMD1
TGCATCCCAGCCTTCT-1	singlet	2	-1468.888226	-1664.072939	-481.5938001	EMD1
TGCATCCCAGCTCGGT-1	singlet	2	-1405.862354	-1185.325182	-597.0042204	EMD1
TGCATCCCATACAGGG-1	singlet	0	-675.7187457	-1989.05292	-1977.653704	EMD1
TGCATCCTCAAGCCTA-1	singlet	0	-1134.7428	-3894.804367	-3470.980452	EMD1
TGCATGAAGCTGTGCC-1	singlet	1	-98.77237439	-34.65263295	-63.56415927	EMD1
TGCATGAAGTTTCTTC-1	singlet	2	-82.24845867	-73.23807089	-36.31624336	EMD1
TGCATGACAAACTGCT-1	singlet	1	-1844.829444	-801.6457277	-1390.445613	EMD1
TGCATGAGTATCGATC-1	singlet	2	-577.0321922	-491.9599276	-204.8941307	EMD1
TGCATGAGTCAGTCCG-1	singlet	2	-760.9855313	-689.6862261	-276.6243179	EMD1
TGCATGATCACCGGTG-1	singlet	2	-401.5163378	-344.4495292	-164.3959959	EMD1
TGCATGATCCGATAAC-1	singlet	2	-785.0330899	-621.7308991	-278.4678505	EMD1

TGCCGAGAGGACTTCT-1	singlet	0	-692.1138386	-2021.865852	-1956.922182	EMD1
TGCCGAGAGGCTCTCG-1	singlet	2	-1360.853898	-1218.753378	-501.4833194	EMD1
TGCCGAGAGGTGCCTC-1	singlet	0	-424.5887567	-998.5586397	-1038.677613	EMD1
TGCCGAGCAGCTTCGG-1	singlet	0	-1491.044128	-4453.190087	-4532.950838	EMD1
TGCCGAGGTACGAAAT-1	singlet	0	-142.8523663	-399.4189959	-390.1795653	EMD1
TGCCGAGGTACTGCCG-1	singlet	2	-1141.922848	-923.2851489	-418.5480309	EMD1
TGCCGAGGTCAGACTT-1	singlet	1	-756.9568835	-241.6281167	-646.2473934	EMD1
TGCCGAGTCAACACGT-1	singlet	2	-1112.116077	-848.6358186	-424.8452843	EMD1
TGCCGAGTCACAGTGT-1	singlet	2	-605.9201187	-526.1474183	-202.5549758	EMD1
TGCCGAGTCGTTCAGA-1	singlet	2	-485.245978	-460.1415105	-221.6935738	EMD1
TGCGACGAGCTCCCTT-1	singlet	2	-404.1418758	-327.3895601	-172.2141558	EMD1
TGCGACGAGTCCCAGC-1	singlet	2	-594.6381867	-486.9978404	-187.0029576	EMD1
TGCGACGCAAAGCTCT-1	singlet	2	-363.019419	-301.1359034	-139.6593216	EMD1
TGCGACGCATCGTGCG-1	singlet	0	-352.8444632	-1020.747091	-1086.691628	EMD1
TGCGACGGTGGGATTG-1	singlet	2	-325.0391081	-351.3039779	-137.6407945	EMD1
TGCGACGTCATTTGCT-1	singlet	1	-792.7122979	-268.6650516	-565.5775949	EMD1
TGCGACGTCCCAACTC-1	singlet	0	-2558.392396	-6373.470066	-6586.187312	EMD1
TGCGACGTCCGCGGAT-1	singlet	0	-275.9529364	-446.1297852	-433.4018152	EMD1
TGCGATAAGGATACGC-1	singlet	2	-432.2382606	-376.0586523	-198.9077128	EMD1
TGCGATAAGTAACGAT-1	singlet	2	-5935.64755	-4605.406851	-2082.244371	EMD1
TGCGATAAGTCAATCC-1	singlet	1	-654.5100484	-239.3935186	-606.7491918	EMD1
TGCGATAAGTGACCTT-1	singlet	1	-5083.703091	-1421.521841	-4159.217324	EMD1
TGCGATACAAGAGAGA-1	singlet	2	-7487.083309	-6489.582662	-1848.40213	EMD1
TGCGATACAATGGCCC-1	singlet	0	-65.61183651	-173.0069634	-191.2186804	EMD1
TGCGATACACAGAGCA-1	singlet	2	-526.4315036	-406.8582919	-189.4810004	EMD1
TGCGATACACCCAAGC-1	singlet	1	-79.75529605	-30.79726367	-75.77918553	EMD1
TGCGATACATGACACT-1	singlet	0	-619.0792063	-1779.452709	-1755.914876	EMD1
TGCGATACATGACCCG-1	singlet	2	-502.0708458	-402.1895001	-169.1287513	EMD1
TGCGATAGTCCTTGTC-1	singlet	2	-972.5349393	-955.8545642	-351.3167719	EMD1
TGCGATATCACATCAG-1	singlet	0	-449.547019	-1202.942664	-1272.604874	EMD1
TGCGATATCGACATCA-1	singlet	1	-1063.460903	-389.0031247	-886.7693551	EMD1
TGCGATATCTTGGTGA-1	singlet	1	-1598.073316	-586.690649	-1307.625376	EMD1
TGCGGCAAGAGAGGGC-1	singlet	2	-427.1953988	-366.9918887	-169.7885349	EMD1
TGCGGCACAGACCTGC-1	singlet	0	-171.6913147	-418.9986814	-503.1532576	EMD1
TGCGGCAGTCCAGGTC-1	singlet	2	-605.0793287	-488.7166206	-243.9016106	EMD1
TGCGGCAGTTGTTGCA-1	singlet	2	-569.6535919	-479.8747205	-233.5740442	EMD1
TGCGGCATCCATCTCG-1	singlet	2	-663.0370713	-501.6485894	-227.7439493	EMD1
TGCGGGTAGATTTGCC-1	doublet	45323	-2905.123468	-1918.334092	-1605.165879	EMD1
TGCGGGTCAAGTGACG-1	singlet	2	-555.9531071	-429.7493045	-186.5230126	EMD1
TGCGGGTCACCAGTTA-1	singlet	1	-6436.55212	-1569.143818	-5382.165632	EMD1
TGCGGGTGTGTACATC-1	singlet	2	-516.3380463	-411.8592076	-209.2510394	EMD1
TGCTCCAAGACTCTAC-1	singlet	2	-647.3146085	-511.1890358	-267.3844068	EMD1
TGCTCCAAGAGAGAAC-1	singlet	1	-9.470712003	-4.295213207	-12.00451776	EMD1
TGCTCCAAGATCCCGC-1	singlet	0	-187.6476192	-482.5500444	-533.7039463	EMD1
TGCTCCAAGCAGCGAT-1	singlet	2	-1392.238565	-1275.755972	-442.1106974	EMD1
TGCTCCAAGCATGGGT-1	singlet	1	-3457.656325	-1288.973641	-2608.318387	EMD1
TGCTCCAAGCGGTATG-1	singlet	1	-3355.875362	-1147.96914	-2866.278899	EMD1
TGCTCCACAAACTAAG-1	singlet	0	-796.0119806	-2317.339368	-2352.649204	EMD1
TGCTCCAGTACCAGAG-1	singlet	0	-351.1488135	-1114.207254	-902.4175026	EMD1
TGCTCCATCCAACTGA-1	singlet	2	-643.1996002	-660.6378308	-305.6222272	EMD1
TGCTCCATCTACCCAC-1	singlet	1	-262.5065916	-98.00073772	-193.3640102	EMD1
TGCTCGTAGGCTCACC-1	singlet	2	-3128.831934	-2618.806369	-1169.635132	EMD1
TGCTCGTAGTATCCTG-1	singlet	1	-157.0264145	-58.44243005	-161.2777815	EMD1
TGCTCGTCACCAGACC-1	singlet	0	-272.9687737	-867.7643876	-754.1106609	EMD1
TGCTCGTCAGATTAAG-1	singlet	1	-1258.341328	-458.5526323	-1024.433018	EMD1
TGCTCGTCATCAGCTA-1	singlet	2	-499.2222347	-405.673265	-182.3717532	EMD1
TGCTCGTGTTAAGCAA-1	singlet	0	-20.02884747	-28.30019058	-189.2165566	EMD1
TGCTCGTTCCGTGGCA-1	singlet	2	-226.0104854	-199.7215323	-102.3803592	EMD1
TGCTGAACACAAAGTA-1	singlet	1	-5011.742777	-1506.470841	-3959.534628	EMD1
TGCTGAACACCTGCGA-1	singlet	0	-392.4056057	-1037.332265	-977.3296876	EMD1
TGCTGAAGTCTACAAC-1	singlet	2	-449.2609611	-304.9775022	-172.9106295	EMD1
TGCTGAAGTGCATACT-1	singlet	2	-200.8993597	-157.559102	-70.7978366	EMD1
TGCTGAAGTGCTCTCT-1	singlet	1	-1904.801969	-530.0432324	-1672.704781	EMD1
TGCTGAAGTGTTCGTA-1	singlet	2	-638.6231742	-529.6379574	-264.3944993	EMD1
TGCTGAATCAAGAAAC-1	singlet	2	-5643.724246	-4411.564361	-1654.961586	EMD1
TGCTGAATCCACAAGT-1	singlet	1	-3488.982416	-1305.974374	-3165.687065	EMD1
TGCTTCGAGTCTAGAA-1	singlet	2	-240.3397943	-267.8641797	-104.8246752	EMD1
TGCTTCGAGTGCACAG-1	singlet	2	-193.0025974	-184.5459053	-95.79850191	EMD1
TGCTTCGGTAACAGGC-1	singlet	0	-20.44348837	-73.08306697	-56.02149132	EMD1
TGCTTCGGTAGGACCA-1	singlet	0	-207.3055715	-476.2381978	-702.9320364	EMD1
TGCTTCGGTCGTAATC-1	singlet	2	-679.0809129	-503.8732756	-230.268229	EMD1
TGCTTCGGTCTCGGAC-1	singlet	0	-284.5372826	-614.9167521	-742.2702733	EMD1
TGCTTCGGTCTGTAGT-1	singlet	2	-1612.259669	-1326.69188	-619.6075711	EMD1
TGCTTCGGTTCGTTCC-1	singlet	2	-5704.973527	-4562.680929	-1929.075966	EMD1
TGCTTCGTCCCTCATG-1	singlet	1	-2006.190852	-707.1103912	-1571.064589	EMD1
TGCTTCGTCCTCTAGC-1	singlet	2	-389.213016	-256.0267731	-142.3709872	EMD1
TGCTTCGTCGCCTCTA-1	singlet	1	-7670.00321	-1892.221621	-5870.714102	EMD1
TGCTTCGTCTCTTGCG-1	singlet	0	-1131.160596	-4184.816561	-4285.660265	EMD1
TGCTTGCAGACCGTTT-1	singlet	0	-1194.723718	-3772.914154	-3778.236602	EMD1
TGCTTGCAGAGGTGCT-1	singlet	1	-8850.561306	-1852.409478	-6470.478035	EMD1
TGCTTGCAGCAACAAT-1	singlet	1	-5532.439419	-1058.728496	-4625.597609	EMD1
TGCTTGCCATGACGGA-1	singlet	1	-2629.409145	-1037.353553	-2039.376764	EMD1
TGCTTGCGTATGAGAT-1	singlet	1	-1891.824545	-565.5070174	-1377.097736	EMD1
TGCTTGCGTGTTTCTT-1	singlet	0	-827.5109253	-3001.189191	-2912.214992	EMD1
TGCTTGCGTTAACCTG-1	singlet	2	-1128.552812	-1232.827463	-445.1708594	EMD1
TGCTTGCTCCGGCAAC-1	singlet	1	-19.08246132	-8.732667934	-25.23047736	EMD1
TGGAACTCACAAGGTG-1	singlet	0	-1244.565781	-3552.399033	-3635.202338	EMD1
TGGAACTCAGCAGGAT-1	singlet	2	-205.0036432	-172.7988736	-81.20562327	EMD1
TGGAACTCAGTCTCTC-1	singlet	2	-861.0809354	-758.9356911	-314.7924248	EMD1
TGGAACTCAGTGCCTG-1	singlet	2	-373.7753024	-303.0987561	-141.9632863	EMD1
TGGAACTGTTTAGTCG-1	singlet	1	-1899.244929	-642.6687686	-1474.889692	EMD1
TGGAACTTCCATAGGT-1	singlet	2	-317.5781306	-288.1154872	-152.153172	EMD1
TGGAACTTCGCTTAAG-1	singlet	2	-636.8572474	-483.0279893	-229.5984057	EMD1
TGGAACTTCTATACTC-1	singlet	0	-295.1143713	-801.8499227	-972.8036862	EMD1
TGGAGAGAGCAGTAAT-1	singlet	0	-97.4362454	-232.1848993	-258.0576095	EMD1
TGGAGAGCAGCGTTGC-1	singlet	2	-1719.743473	-1402.887327	-515.5809665	EMD1
TGGAGAGCATCTGGGC-1	singlet	0	-194.4808137	-530.2943824	-457.3103589	EMD1
TGGAGAGGTGAGTGAC-1	singlet	2	-2016.16177	-1450.728156	-636.6540925	EMD1
TGGAGGAAGCAAGTGC-1	singlet	2	-347.9877443	-342.6432159	-137.6230602	EMD1
TGGAGGAGTGGTAACG-1	singlet	2	-750.2200028	-595.8870445	-298.2460888	EMD1
TGGAGGATCACCCTTG-1	singlet	2	-203.7481674	-135.2794108	-70.20917663	EMD1
TGGAGGATCTGGTGGC-1	singlet	0	-769.5525093	-2582.064543	-2361.633877	EMD1
TGGATCACAATGCTCA-1	singlet	0	-256.4553296	-854.4273914	-799.6811117	EMD1
TGGATCAGTGTTAAAG-1	singlet	0	-25.32272992	-63.81338689	-82.67891359	EMD1
TGGATCAGTTGCAACT-1	singlet	2	-3979.073784	-3860.313836	-2105.055547	EMD1
TGGATCAGTTTCCATT-1	singlet	2	-550.886006	-527.4446978	-250.1594628	EMD1
TGGATCATCGGAGCAA-1	singlet	2	-787.7921397	-594.0741679	-326.4198655	EMD1
TGGATCATCGGTGTAT-1	singlet	2	-399.3024593	-437.534534	-189.8012979	EMD1
TGGATGTAGTTGGCGA-1	singlet	2	-656.7088585	-600.7152858	-250.5528898	EMD1
TGGATGTCACAAATCC-1	singlet	2	-323.2007911	-286.3051185	-124.6649035	EMD1

TGGATGTCAGAGAGGG-1	singlet	1	-504.3355165	-204.1518824	-412.192634	EMD1
TGGATGTCAGATAAAC-1	singlet	2	-1517.649908	-1401.981265	-615.7619748	EMD1
TGGATGTCATACACCA-1	singlet	0	-384.1367002	-1230.817079	-1123.445618	EMD1
TGGATGTCATGGAAGC-1	singlet	1	-204.8298577	-59.54796246	-181.8291137	EMD1
TGGATGTGTGCCCTTT-1	singlet	0	-907.9538308	-2766.292886	-2678.52358	EMD1
TGGATGTTCCACTGGG-1	singlet	2	-217.3791961	-188.3899113	-97.55706448	EMD1
TGGCGTGAGACATATG-1	singlet	2	-444.5358559	-371.8073584	-178.4910787	EMD1
TGGCGTGAGACTCTTG-1	singlet	0	-425.3858569	-1345.237941	-1199.30439	EMD1
TGGCGTGAGCCGTTAT-1	singlet	2	-1865.683887	-1721.689805	-490.4062465	EMD1
TGGCGTGAGGCGCTCT-1	singlet	1	-11960.05366	-2405.60207	-8772.636531	EMD1
TGGCGTGAGTATGATG-1	singlet	1	-1325.449333	-436.6836127	-1043.280852	EMD1
TGGCGTGCATGCGTGC-1	singlet	1	-7151.694311	-2271.868628	-5784.116967	EMD1
TGGCGTGCATTCGGGC-1	singlet	0	-524.990935	-1391.099008	-1489.641395	EMD1
TGGCGTGTCCGTGGCA-1	singlet	1	-1299.935115	-416.7492592	-1031.759525	EMD1
TGGGAAGAGGCAGTCA-1	singlet	1	-3617.803471	-1215.193187	-2705.138716	EMD1
TGGGAAGAGGTCTGGA-1	singlet	1	-104.3553682	-33.26821684	-93.30732661	EMD1
TGGGAAGAGTAAGCAT-1	singlet	0	-533.9679715	-1511.486209	-1435.993195	EMD1
TGGGAAGCACAAATAG-1	singlet	2	-692.3530732	-653.700781	-246.8161387	EMD1
TGGGAAGCACGCGTGT-1	singlet	0	-446.2616573	-1372.699039	-1388.202944	EMD1
TGGGAAGCACTTGAGT-1	singlet	2	-405.4744418	-312.9614335	-142.8239551	EMD1
TGGGAAGGTGTGTTTG-1	singlet	0	-183.4420611	-727.2151046	-667.9534837	EMD1
TGGGAAGGTTTGTGGT-1	doublet	2/0	-417.088391	-338.0123764	-235.8769027	EMD1
TGGGAAGTCAGGGATG-1	singlet	2	-1537.685958	-1060.100818	-446.5669624	EMD1
TGGGAGAAGATGTTAG-1	singlet	0	-535.6430446	-1752.231673	-1673.406939	EMD1
TGGGAGAAGCTCCGAC-1	singlet	1	-6199.894438	-1910.553962	-4911.174972	EMD1
TGGGAGACAAGCACCC-1	singlet	2	-439.1872326	-382.8668038	-168.9513597	EMD1
TGGGAGACACATAGCT-1	singlet	0	-90.78835692	-331.4717558	-311.7338199	EMD1
TGGGAGACAGTATGAA-1	singlet	1	-3449.474037	-964.8527482	-2707.148242	EMD1
TGGGAGAGTCGTCAGC-1	singlet	0	-478.8958409	-1295.403184	-1311.066053	EMD1
TGGGAGATCTTGAGCA-1	singlet	2	-255.589054	-229.415401	-111.1233806	EMD1
TGGGATTAGCGTGTCC-1	singlet	0	-834.0869826	-2825.793237	-2499.420029	EMD1
TGGGATTCAGCTGTAT-1	doublet	1/0	-3764.047296	-2853.440941	-4273.259133	EMD1
TGGGATTGTGCCCGTA-1	singlet	2	-528.0750372	-403.2227826	-214.8540693	EMD1
TGGGATTTCATCACTT-1	singlet	2	-144.4262404	-146.7947331	-63.08384663	EMD1
TGGGATTTCCCTGGTT-1	singlet	0	-878.7648796	-2403.79253	-2418.778871	EMD1
TGGGCGTAGGCTGGAT-1	singlet	2	-420.5746074	-347.4230264	-171.0301075	EMD1
TGGGCGTAGTTTCTTC-1	singlet	2	-492.8958105	-410.8819781	-179.5253161	EMD1
TGGGCGTCAAGACCTT-1	singlet	2	-4269.898893	-3427.520807	-1353.55971	EMD1
TGGGCGTCACCATATG-1	singlet	0	-163.8262663	-534.7307859	-583.1715774	EMD1
TGGGCGTCATAATGCC-1	singlet	1	-142.6567094	-50.76883103	-80.47903562	EMD1
TGGGCGTGTCTTGAGT-1	singlet	1	-972.579726	-385.0203299	-752.2904971	EMD1
TGGGCGTTCAGGAGAC-1	singlet	1	-3850.53583	-796.9494206	-2951.311034	EMD1
TGGGCGTTCATAGAGA-1	singlet	2	-1251.719586	-1004.608429	-446.1718417	EMD1
TGGGCGTTCGCAGTGC-1	singlet	2	-531.1407727	-478.9682458	-212.2829152	EMD1
TGGGCGTTCGCCAGTG-1	singlet	0	-783.2136072	-2092.828845	-2230.789787	EMD1
TGGGCGTTCGCTCTAC-1	singlet	1	-1212.01907	-405.2525412	-875.4369473	EMD1
TGGGCTGCAACACGAG-1	singlet	0	-1633.195908	-3491.520262	-3462.27271	EMD1
TGGGCTGCAAGAGTAT-1	singlet	1	-1482.284774	-508.9444079	-1178.366737	EMD1
TGGGCTGCAAGTGCAG-1	singlet	2	-525.4767585	-439.4424753	-225.9630031	EMD1
TGGGCTGGTAGACAGC-1	singlet	0	-24.53492373	-109.5876477	-81.77591554	EMD1
TGGGCTGGTGTTAAAG-1	singlet	1	-1825.937413	-662.7796828	-1485.12865	EMD1
TGGGCTGGTTAAGGAT-1	singlet	2	-474.422858	-387.9615427	-184.0144499	EMD1
TGGGCTGGTTTGGGTT-1	singlet	2	-919.6611513	-776.6351948	-343.239166	EMD1
TGGGTTAAGTACCGGA-1	singlet	2	-1588.775989	-1264.656719	-592.6244953	EMD1
TGGGTTAAGTGCTAGG-1	singlet	1	-1606.019661	-516.2157291	-1344.33335	EMD1
TGGGTTAAGTGGACTG-1	singlet	2	-608.655795	-534.929459	-264.4653794	EMD1
TGGGTTAAGTTCGCAT-1	singlet	2	-106.8287899	-98.79625531	-41.82450751	EMD1
TGGGTTACAAGTACCT-1	singlet	0	-223.3572519	-778.600828	-936.6874867	EMD1
TGGGTTACAATCGAAA-1	singlet	2	-443.3563103	-444.6527198	-205.5354766	EMD1
TGGGTTACAATCTAGC-1	singlet	1	-4085.871689	-1012.655331	-3347.503439	EMD1
TGGGTTACAGTCACGC-1	singlet	2	-723.2342908	-629.8387704	-282.1636074	EMD1
TGGGTTAGTATCACCA-1	singlet	2	-619.3420911	-508.3473891	-201.0915774	EMD1
TGGGTTAGTCCTGGTG-1	singlet	2	-345.7172291	-274.3673651	-135.8226869	EMD1
TGGGTTAGTTGGTAGG-1	singlet	2	-552.2731489	-551.3606079	-222.6737594	EMD1
TGGGTTATCCTCATAT-1	singlet	2	-2764.912703	-2686.299461	-1019.179407	EMD1
TGGGTTATCTCCATAT-1	singlet	1	-2351.025	-808.93114	-1814.475096	EMD1
TGGGTTATCTTGTGCC-1	singlet	1	-3204.30279	-1063.725764	-2566.217847	EMD1
TGGTACAAGAAACACT-1	singlet	1	-3777.284082	-1356.153842	-3002.378816	EMD1
TGGTACAAGTCTAGAA-1	doublet	0/2	-10.64861686	-9.838490925	-11.21571804	EMD1
TGGTACACAAAGCACG-1	singlet	1	-2397.380519	-756.1733758	-1883.071001	EMD1
TGGTACACATTCCTAT-1	singlet	1	-2610.718009	-654.572215	-2319.456756	EMD1
TGGTACAGTCCGAAGA-1	singlet	1	-742.6223496	-267.3643375	-517.3397144	EMD1
TGGTACAGTTCTCGTC-1	singlet	2	-692.0591341	-602.826629	-286.7865347	EMD1
TGGTACATCAGGAGAC-1	unassigned	45293	-85.51118628	-25.62931246	-37.32019658	EMD1
TGGTACATCATCGGGC-1	singlet	2	-684.1391961	-572.6681526	-298.7913546	EMD1
TGGTACATCGCGCCAA-1	singlet	1	-348.1490547	-128.7437459	-338.3383227	EMD1
TGGTACATCTCATGGA-1	singlet	0	-1154.792748	-4276.511815	-3983.076088	EMD1
TGGTACATCTTAATCC-1	singlet	2	-595.368531	-451.5326061	-227.9753539	EMD1
TGGTACATCTTAGGAC-1	singlet	0	-37.87651612	-112.6462688	-97.76832022	EMD1
TGGTAGTCACCAATTG-1	singlet	0	-630.2751867	-2502.468479	-2156.213342	EMD1
TGGTAGTCACTACACA-1	singlet	0	-215.2536348	-507.4759598	-510.1204928	EMD1
TGGTAGTCACTCCTGT-1	singlet	1	-1520.727205	-483.4631167	-1218.45845	EMD1
TGGTAGTCATGACTAC-1	singlet	0	-162.7313517	-528.4734209	-566.5543978	EMD1
TGGTAGTGTTGTATGC-1	singlet	1	-2108.664039	-631.8890939	-1470.279474	EMD1
TGGTAGTGTTGTGTAC-1	singlet	0	-524.5068744	-1517.496773	-1584.11184	EMD1
TGGTAGTGTTGTTGAC-1	singlet	2	-567.4245614	-488.2414843	-253.8110949	EMD1
TGGTGATAGGTAAACT-1	singlet	2	-393.573001	-345.072597	-151.8741123	EMD1
TGGTGATAGTGTGTTC-1	singlet	2	-2108.030267	-1767.523746	-726.5044514	EMD1
TGGTGATAGTTTGTCG-1	singlet	1	-1986.194142	-694.6509742	-1570.12499	EMD1
TGGTGATCAAGCAGGT-1	singlet	2	-3202.229128	-2871.60464	-1034.737202	EMD1
TGGTGATCAGGGTTGA-1	singlet	2	-562.8907817	-453.0085295	-248.4453164	EMD1
TGGTGATCAGTCACGC-1	singlet	0	-127.7185406	-325.3116117	-297.4889361	EMD1
TGGTGATGTCCACTTC-1	singlet	2	-501.3493344	-412.5177086	-192.6476566	EMD1
TGGTGATGTTGGATCT-1	singlet	0	-151.4379231	-401.8427796	-368.7312481	EMD1
TGGTGATTCGCTGCGA-1	singlet	0	-996.4389794	-3725.833115	-3146.678871	EMD1
TGGTTAGAGAGTCAGC-1	singlet	2	-585.6548552	-414.1124505	-181.9271082	EMD1
TGGTTAGAGCCTGACC-1	singlet	0	-474.2601293	-1241.025371	-1306.710265	EMD1
TGGTTAGAGGCAATGC-1	singlet	1	-1453.075048	-456.9977458	-1180.485373	EMD1
TGGTTAGAGGTAACTA-1	singlet	0	-550.8488816	-1727.780375	-1602.143416	EMD1
TGGTTAGGTCAGATTC-1	singlet	1	-861.5871524	-301.4169697	-735.6942831	EMD1
TGGTTAGTCTGAGAGG-1	singlet	2	-2015.752373	-1966.724932	-609.7706527	EMD1
TGTAACGAGGATTTCC-1	singlet	2	-494.0060894	-398.6134538	-177.2353913	EMD1
TGTAACGAGTATGTAG-1	singlet	0	-549.3309998	-1949.613799	-1670.266338	EMD1
TGTAACGCAACAAGAT-1	singlet	2	-492.6154998	-381.385994	-185.5146483	EMD1
TGTAACGCAGCGTGCT-1	singlet	2	-374.9035587	-351.4229802	-149.2985856	EMD1
TGTAACGGTTCAGCGC-1	singlet	1	-5853.201316	-1426.529587	-4345.861318	EMD1
TGTAACGGTTGACTGT-1	singlet	2	-6398.433727	-5152.992938	-2280.837799	EMD1
TGTAACGGTTGCTTGA-1	singlet	0	-390.4762963	-1084.57465	-1164.209403	EMD1
TGTAAGCAGAGTCTGG-1	singlet	1	-1699.237415	-540.4368347	-1332.156132	EMD1
TGTAAGCAGATCCGAG-1	singlet	0	-14.03552125	-51.96794599	-52.11878551	EMD1

TGTAAGCAGCATGCAG-1	singlet	2	-678.4055575	-572.8567279	-256.5357726	EMD1
TGTAAGCAGCGAGGAG-1	singlet	0	-137.5272263	-440.3176903	-421.4076894	EMD1
TGTAAGCAGTGGATAT-1	singlet	0	-176.3286134	-447.910137	-502.6945114	EMD1
TGTAAGCCACCCAAGC-1	singlet	2	-2935.101769	-2302.139811	-1073.446071	EMD1
TGTAAGCGTCAAACGG-1	singlet	2	-1305.870847	-1013.712006	-444.7217989	EMD1
TGTAAGCGTGCCAAGA-1	singlet	0	-167.9978072	-429.2742061	-414.4806535	EMD1
TGTAAGCGTGTTCCTC-1	singlet	0	-35.64773016	-167.1629359	-109.4497241	EMD1
TGTAAGCTCCACAGGC-1	singlet	0	-167.1201083	-630.5789246	-525.4034521	EMD1
TGTAAGCTCCGCGGAT-1	singlet	2	-436.6529691	-411.8157695	-170.9216457	EMD1
TGTAAGCTCGCTGATA-1	singlet	2	-668.2875854	-464.6686423	-251.5496511	EMD1
TGTAAGCTCTCGCAGG-1	singlet	1	-158.3454967	-54.83836228	-124.1109407	EMD1
TGTAAGCTCTGTAACG-1	singlet	0	-55.12651816	-246.1800305	-294.7851291	EMD1
TGTACAGAGCAACTCT-1	singlet	1	-152.4819698	-52.72499408	-122.7720131	EMD1
TGTACAGAGCTGTGCC-1	singlet	2	-1126.728386	-935.990855	-411.5419504	EMD1
TGTACAGAGTTATGGA-1	singlet	1	-182.969901	-58.25421149	-135.4924212	EMD1
TGTACAGCATCACGGC-1	singlet	0	-36.66137596	-102.4085617	-114.3820986	EMD1
TGTACAGTCCATGCAA-1	singlet	1	-532.4687631	-196.5676236	-409.2394438	EMD1
TGTACAGTCGTTTACT-1	singlet	0	-1648.149748	-5027.076796	-5106.91193	EMD1
TGTAGACAGATACTGA-1	singlet	0	-1014.654353	-3629.829257	-3777.651716	EMD1
TGTAGACAGATGTAGT-1	singlet	2	-734.9521054	-515.1295599	-218.0037319	EMD1
TGTAGACAGCACACCC-1	singlet	2	-11.45568062	-13.31661073	-5.726633894	EMD1
TGTAGACAGGCCACTC-1	singlet	2	-434.4201564	-425.8696351	-220.3184918	EMD1
TGTAGACCAAGATGGC-1	singlet	2	-893.1487439	-669.5008364	-326.2993654	EMD1
TGTAGACCACACCAGC-1	singlet	0	-421.8141989	-1302.586597	-1224.857381	EMD1
TGTAGACCACGGTAGA-1	singlet	2	-423.3592742	-344.1262051	-161.492081	EMD1
TGTAGACCAGGCGATA-1	doublet	1/0	-427.7622731	-419.871046	-686.8372183	EMD1
TGTAGACGTCCTCCAT-1	singlet	2	-406.3221295	-338.9323966	-179.5637527	EMD1
TGTAGACGTCCTGGGT-1	singlet	2	-592.2710813	-502.8558916	-207.0593857	EMD1
TGTCAGAAGCATATGA-1	singlet	0	-316.3154307	-879.9567259	-953.6910572	EMD1
TGTCAGAAGCCTCAAT-1	singlet	2	-510.5879986	-375.9867895	-180.5565	EMD1
TGTCAGACAACTAGAA-1	singlet	1	-3772.549144	-1111.220405	-3050.691575	EMD1
TGTCAGACACTTGAAC-1	singlet	2	-11086.42497	-8564.439624	-3531.110596	EMD1
TGTCAGACAGTTAGGG-1	singlet	0	-122.0480481	-283.2400979	-274.3907973	EMD1
TGTCAGAGTCGGTGTC-1	singlet	0	-241.5763375	-659.5778993	-654.9057243	EMD1
TGTCAGAGTGGAAATT-1	singlet	2	-1954.827893	-1641.468535	-695.8620708	EMD1
TGTCAGAGTTTATGCG-1	singlet	2	-2393.949761	-2082.902459	-852.7228322	EMD1
TGTCAGATCCATTCAT-1	singlet	0	-617.0677751	-2370.750734	-2214.137348	EMD1
TGTCAGATCCTCATAT-1	singlet	2	-2302.651554	-1938.971199	-803.2639962	EMD1
TGTCAGATCCTCTCTT-1	singlet	1	-1368.81153	-454.263037	-1056.537104	EMD1
TGTCAGATCGTAGGGA-1	singlet	1	-1363.438468	-337.6833312	-1039.37167	EMD1
TGTCAGATCGTGCATA-1	singlet	0	-329.0949404	-952.899947	-885.1240616	EMD1
TGTCCACAGGGCCCTT-1	singlet	2	-605.269692	-442.2488769	-193.5100916	EMD1
TGTCCACAGTCGGCCT-1	singlet	2	-230.5147238	-188.3294104	-94.78619349	EMD1
TGTCCACCAATAACGA-1	singlet	0	-444.168283	-1158.188626	-1482.726613	EMD1
TGTCCACGTAAGGAGA-1	singlet	0	-276.0294777	-1015.882228	-1166.881487	EMD1
TGTCCACGTACGAAAT-1	singlet	2	-699.645347	-615.0302186	-290.7588044	EMD1
TGTCCACGTATACCCA-1	singlet	1	-441.5713738	-188.7097394	-324.0978581	EMD1
TGTCCACGTCCTACAA-1	singlet	1	-232.4289357	-73.21117052	-213.2979759	EMD1
TGTCCACGTTGTTGAC-1	singlet	2	-605.5408157	-576.8518439	-256.3593974	EMD1
TGTCCACTCACGGAGA-1	singlet	0	-59.19902544	-158.7331775	-144.0692984	EMD1
TGTCCACTCCAACCGG-1	singlet	1	-160.3971327	-74.17855873	-118.4901805	EMD1
TGTCCACTCTCTAAGG-1	doublet	45323	-853.6397593	-604.9232666	-529.4187469	EMD1
TGTCCCAAGACATATG-1	singlet	2	-570.4086077	-510.5169377	-274.1831257	EMD1
TGTCCCAAGATTCGAA-1	singlet	1	-5224.926729	-1424.786715	-4259.883382	EMD1
TGTCCCAAGCTTAGTC-1	singlet	1	-1971.092224	-675.2842044	-1468.114669	EMD1
TGTCCCAAGGCATGCA-1	singlet	2	-293.5851145	-210.8492822	-110.6225932	EMD1
TGTCCCACAATGGCCC-1	singlet	2	-552.3446751	-473.6007392	-233.3761958	EMD1
TGTCCCAGTAGCTTTG-1	singlet	2	-277.2566883	-213.9758304	-119.8802794	EMD1
TGTCCCAGTGGACAGT-1	singlet	2	-593.2297884	-508.8334905	-218.0120094	EMD1
TGTCCCAGTTATGACC-1	singlet	0	-567.5125574	-2779.01645	-2257.037197	EMD1
TGTCCCAGTTCCCAAA-1	singlet	2	-522.3181363	-446.5619374	-230.8014874	EMD1
TGTCCCAGTTGCTCCT-1	singlet	0	-386.1007231	-1269.730094	-1256.407962	EMD1
TGTCCCATCGCTTACC-1	singlet	1	-2492.812432	-687.8148735	-2064.397823	EMD1
TGTCCCATCGGTTGTA-1	singlet	2	-633.5041444	-561.9492536	-277.6896694	EMD1
TGTCCCATCGTTCATT-1	singlet	0	-242.0746704	-661.6890994	-648.8287038	EMD1
TGTCCTGAGCACCAGA-1	singlet	2	-1213.546046	-1190.71661	-478.8031015	EMD1
TGTCCTGAGCATGTTC-1	singlet	1	-1218.168463	-414.0983385	-922.0549642	EMD1
TGTCCTGCATCGAAGG-1	singlet	2	-3076.164175	-2320.868284	-1041.109205	EMD1
TGTCCTGGTAGCACAG-1	singlet	1	-455.3815289	-170.3615195	-446.8135723	EMD1
TGTCCTGGTTCGGCGT-1	singlet	1	-244.2971109	-96.29192781	-228.4890612	EMD1
TGTCCTGTCGCCTTGT-1	singlet	2	-322.6455759	-268.8515415	-123.4457764	EMD1
TGTGAGTAGTACAACA-1	singlet	2	-2063.834423	-1849.015886	-790.7009638	EMD1
TGTGAGTCAAAGGATT-1	singlet	2	-744.0482859	-623.7804152	-292.3876327	EMD1
TGTGAGTCACACGTGC-1	singlet	1	-3242.565781	-1143.828499	-2842.214908	EMD1
TGTGAGTCACAGCATT-1	singlet	2	-281.8353352	-245.5962141	-120.7756307	EMD1
TGTGAGTCACGGCTAC-1	singlet	2	-1443.83591	-1275.573159	-546.5844271	EMD1
TGTGAGTCACTGGCCA-1	singlet	0	-2072.094148	-6022.664233	-6248.432268	EMD1
TGTGAGTCATGTGTCA-1	singlet	2	-517.5916378	-454.4278894	-193.4924686	EMD1
TGTGAGTGTATGTCCA-1	singlet	2	-138.3751537	-149.9845812	-55.8595136	EMD1
TGTGAGTGTGTCCTAA-1	singlet	2	-698.5939452	-832.1805885	-183.8205777	EMD1
TGTGAGTGTTTACACG-1	singlet	1	-638.9439764	-204.3978665	-661.6833726	EMD1
TGTGATGAGGCATGGT-1	singlet	0	-967.8763586	-2496.235275	-2578.550054	EMD1
TGTGATGAGGTTGTTC-1	singlet	0	-752.0517538	-2144.673392	-2193.83631	EMD1
TGTGATGAGTAAAGCT-1	singlet	0	-367.7566499	-983.6172931	-1087.104736	EMD1
TGTGATGCAGGAGGTT-1	singlet	2	-387.6878666	-362.3500233	-188.1518999	EMD1
TGTGATGCAGGGTTGA-1	singlet	1	-2739.062231	-962.1259387	-2146.538581	EMD1
TGTGATGGTCCACTCT-1	singlet	2	-345.0318414	-313.8946989	-163.4902386	EMD1
TGTGATGGTCGCACAC-1	singlet	1	-3800.499573	-882.5221027	-2555.216283	EMD1
TGTGATGGTGTGACCC-1	singlet	2	-1873.544825	-1662.570991	-690.3862714	EMD1
TGTGATGGTTGGCCTG-1	singlet	0	-334.1535308	-1163.235761	-1003.719183	EMD1
TGTGCGGAGCTAAATG-1	singlet	2	-4324.693018	-3503.891215	-1049.259443	EMD1
TGTGCGGAGGGCATGT-1	singlet	0	-128.6634791	-420.6369959	-358.157764	EMD1
TGTGCGGCACGTCGGT-1	singlet	0	-726.2250447	-2815.46927	-2724.106438	EMD1
TGTGCGGTCACCTCAC-1	singlet	2	-4921.84041	-5423.766974	-1291.163318	EMD1
TGTGCGGTCCAAGGGA-1	singlet	2	-460.325265	-332.9805121	-152.4742281	EMD1
TGTGGCGCAATCTCTT-1	singlet	2	-478.6760826	-488.5218107	-223.4406423	EMD1
TGTGGCGCATGAGGGT-1	singlet	2	-2860.180541	-2237.808817	-977.5075908	EMD1
TGTGGCGGTACACTCA-1	singlet	2	-1681.706663	-1628.002523	-617.2063843	EMD1
TGTGGCGGTCGCGGTT-1	singlet	2	-1446.640601	-1163.748222	-525.9216316	EMD1
TGTGGCGGTCGCTGCA-1	singlet	2	-1506.636705	-1183.411771	-470.4675562	EMD1
TGTGGCGGTCTCTCTG-1	singlet	1	-828.3416832	-288.8147083	-780.3658926	EMD1
TGTGGCGGTTAGGACG-1	singlet	2	-223.2654898	-203.8857918	-107.8606509	EMD1
TGTGGCGGTTCTCCAC-1	singlet	2	-1118.515487	-866.5026653	-368.3510732	EMD1
TGTGGCGTCTAGAACC-1	singlet	2	-565.5336101	-540.6780042	-237.5972969	EMD1
TGTGGCGTCTAGAGCT-1	singlet	2	-650.9565259	-522.6047487	-238.0236606	EMD1
TGTGGCGTCTCTCTAA-1	singlet	2	-2610.42754	-2045.691422	-882.7342486	EMD1
TGTGTGAAGCCTGGAA-1	singlet	1	-388.6648762	-144.7265766	-316.9705833	EMD1
TGTGTGAAGGCTAAAT-1	singlet	0	-399.7661774	-1174.148934	-1016.431153	EMD1
TGTGTGACACGTGAGA-1	singlet	2	-1501.415997	-1371.620922	-543.8641911	EMD1
TGTGTGACATATAGCC-1	singlet	1	-1040.848067	-333.2882623	-876.8841066	EMD1

TGTGTGAGTGAATGTA-1	singlet	1	-1750.283084	-614.6713663	-1426.586433	EMD1
TGTGTGATCAACTGAC-1	singlet	1	-3632.093075	-834.1833859	-2926.22057	EMD1
TGTGTGATCCAAGCAT-1	singlet	0	-284.9416791	-859.2120225	-785.7230926	EMD1
TGTGTGATCGAACCAT-1	singlet	1	-3275.148784	-870.4550672	-2295.004279	EMD1
TGTGTGATCGGCTTCT-1	singlet	2	-67.85122486	-80.8381337	-32.89204586	EMD1
TGTGTGATCTCTCCGA-1	singlet	0	-124.8742823	-283.7972973	-413.1370533	EMD1
TGTTACTAGCTAGATA-1	singlet	1	-546.9152556	-225.2808154	-564.5729545	EMD1
TGTTACTAGGTGATAT-1	singlet	2	-2113.658159	-1641.357732	-699.3212505	EMD1
TGTTACTCAACAGTGG-1	singlet	2	-587.3878046	-423.8284085	-207.7275067	EMD1
TGTTACTCACAGTCAT-1	singlet	1	-6884.472933	-3255.591139	-6026.154212	EMD1
TGTTACTCACCGCTAG-1	singlet	0	-298.2258715	-1016.78671	-803.6785851	EMD1
TGTTACTGTCTAGTGT-1	singlet	1	-1069.472341	-394.9140412	-759.4731914	EMD1
TGTTACTGTGGCATCC-1	singlet	2	-93.74312381	-77.01167045	-39.27250929	EMD1
TGTTACTTCATTGCTT-1	singlet	2	-620.6629804	-436.9448304	-188.0690924	EMD1
TGTTACTTCCAGTGTA-1	singlet	1	-157.373931	-57.58007864	-96.46567491	EMD1
TGTTACTTCGCAATTG-1	singlet	2	-441.0379412	-390.6071014	-184.9937253	EMD1
TGTTCATAGACGCCCT-1	singlet	2	-6410.145583	-4909.298026	-2095.867055	EMD1
TGTTCATAGTCCGCCA-1	singlet	2	-445.1598886	-424.9365752	-166.3777965	EMD1
TGTTCATAGTGACACG-1	singlet	1	-3665.116927	-1106.801836	-2667.729511	EMD1
TGTTCATCAACAGAGC-1	singlet	1	-88.07384065	-25.66789185	-80.09232418	EMD1
TGTTCATCAGAGGTTG-1	singlet	0	-888.5890049	-2573.707247	-2444.559897	EMD1
TGTTCATCAGGTACGA-1	singlet	2	-865.4370956	-607.8424747	-294.374905	EMD1
TGTTCATGTATGGTAA-1	singlet	2	-311.8957054	-257.1525366	-116.8015666	EMD1
TGTTCCGAGAAACTCA-1	singlet	1	-199.628827	-68.07077074	-156.0807218	EMD1
TGTTCCGAGCAAACAT-1	singlet	2	-6860.735716	-4945.41505	-2639.219729	EMD1
TGTTCCGAGCATGTTC-1	singlet	1	-2593.598382	-821.2639908	-2037.787017	EMD1
TGTTCCGCAAGAGTGC-1	singlet	0	-470.0544211	-1613.653104	-2098.172312	EMD1
TGTTCCGCAGCAGTAG-1	singlet	1	-129.3053068	-32.41913323	-91.2660704	EMD1
TGTTCCGCATCGGCCA-1	singlet	1	-1987.547982	-665.5914708	-1577.249505	EMD1
TGTTCCGGTGTTGAGG-1	singlet	2	-2002.74303	-2081.264343	-702.9842162	EMD1
TGTTCCGGTTGCCGAC-1	singlet	1	-1315.078711	-371.4940416	-1115.463101	EMD1
TGTTCCGGTTTCGGCG-1	singlet	1	-3048.91498	-1085.463897	-2313.24614	EMD1
TGTTCCGTCAAGTTGC-1	singlet	1	-1424.807922	-468.4890993	-1174.100531	EMD1
TGTTCCGTCCAATCTT-1	singlet	0	-1368.729331	-4996.676395	-3966.073787	EMD1
TGTTCTACAAGTGGCA-1	singlet	1	-2606.057014	-773.8659454	-1970.248872	EMD1
TGTTCTACACGACAAG-1	singlet	1	-3672.031752	-917.0383443	-3140.662785	EMD1
TGTTCTACAGGCGAAT-1	singlet	0	-105.254915	-324.9584541	-333.0709381	EMD1
TGTTCTAGTACAAGTA-1	singlet	2	-1071.779354	-916.9036465	-348.4619124	EMD1
TGTTCTAGTCAAGTTC-1	singlet	1	-456.6698603	-212.4160176	-532.1574883	EMD1
TGTTCTAGTCGCTTGG-1	singlet	0	-172.7288119	-422.2004347	-461.2239186	EMD1
TGTTCTAGTCGTATTG-1	singlet	0	-1069.855274	-3056.52075	-2980.658478	EMD1
TGTTCTATCCGTAGTA-1	singlet	1	-582.4878338	-221.9630132	-593.2949311	EMD1
TGTTCTATCGCTCTAC-1	singlet	1	-2937.635196	-717.3455915	-2005.661386	EMD1
TGTTGAGAGTCATGCT-1	singlet	2	-2591.923027	-2105.081412	-816.4429614	EMD1
TGTTGAGAGTGCCGAA-1	singlet	2	-314.9061094	-262.518665	-114.1120551	EMD1
TGTTGAGCAATCTGCA-1	singlet	1	-1135.685302	-387.3254466	-997.8317831	EMD1
TGTTGAGCAGCTGAGA-1	singlet	0	-522.722722	-1387.071787	-1491.218398	EMD1
TGTTGAGCATTACGGT-1	singlet	0	-251.4924469	-852.5343849	-769.4936975	EMD1
TGTTGAGGTAGCACGA-1	singlet	2	-912.7259441	-829.3761	-345.2340382	EMD1
TGTTGAGTCAAAGAAC-1	singlet	1	-3716.435387	-1300.49515	-3024.164879	EMD1
TGTTGAGTCAGAGTGG-1	singlet	0	-655.812535	-1791.210291	-1760.638864	EMD1
TGTTGAGTCCGTGACG-1	singlet	0	-12.79268475	-100.3705151	-84.35661795	EMD1
TGTTGAGTCTCTCTAA-1	singlet	0	-451.6983931	-1227.702726	-1140.442345	EMD1
TGTTGGAAGAGGGCGA-1	singlet	2	-359.9990216	-290.1603258	-126.2005487	EMD1
TGTTGGAAGCGGTATG-1	singlet	2	-304.5583704	-275.614396	-144.834179	EMD1
TGTTGGAAGGATACAT-1	singlet	1	-1637.412754	-576.1829774	-1395.449	EMD1
TGTTGGACACGCGCAT-1	doublet	0/2	-601.2351227	-845.2063315	-734.445032	EMD1
TGTTGGACATATGGCT-1	singlet	1	-1221.247827	-403.8011429	-1008.252783	EMD1
TGTTGGAGTAAGATTG-1	singlet	0	-803.2232347	-2521.698127	-2680.219229	EMD1
TGTTGGAGTCTAGTGT-1	singlet	2	-311.6402378	-252.4068949	-113.904858	EMD1
TGTTGGAGTTTAGAGA-1	singlet	2	-455.5140276	-448.9073711	-208.1436207	EMD1
TGTTGGATCAACCCGG-1	singlet	1	-96.59860102	-24.68578277	-79.71978176	EMD1
TGTTGGATCATTGCTT-1	unassigned	45293	-131.6858553	-41.09665433	-53.57725347	EMD1
TGTTGGATCCGCGAGT-1	singlet	2	-31.25550384	-10.76036012	-7.203820692	EMD1
TGTTGGATCCTCACCA-1	singlet	2	-409.8660476	-389.3965877	-148.312073	EMD1
TGTTGGATCGACTCCT-1	singlet	0	-210.5498325	-471.8886178	-506.2700526	EMD1
TGTTGGATCGTCAACA-1	singlet	2	-412.3632744	-261.7696272	-165.8386847	EMD1
TGTTTGTAGCAGCACA-1	singlet	2	-4780.98016	-3851.631186	-1673.762153	EMD1
TGTTTGTAGCCATTGT-1	singlet	0	-180.410324	-688.2300113	-694.9310918	EMD1
TGTTTGTAGCTCCCTT-1	singlet	0	-1125.623299	-3242.843108	-3445.306356	EMD1
TGTTTGTAGGCGATAC-1	singlet	2	-105.2295581	-72.87867325	-34.0603724	EMD1
TGTTTGTAGGCTCTCG-1	singlet	1	-8574.619741	-1807.602069	-6206.406389	EMD1
TGTTTGTAGGTCATAA-1	singlet	1	-189.2213397	-70.37049725	-175.592642	EMD1
TGTTTGTAGTCTCGTA-1	singlet	2	-315.1383322	-281.9259578	-116.6394719	EMD1
TGTTTGTCAACGCCCA-1	singlet	2	-332.9141967	-287.429812	-147.6475453	EMD1
TGTTTGTCAATCGAAA-1	singlet	2	-444.2684139	-384.5691825	-200.8673462	EMD1
TGTTTGTTCGATTCCC-1	singlet	1	-3752.834011	-1029.726431	-2760.972667	EMD1
TGTTTGTTCTAAGCGT-1	singlet	0	-121.2369591	-385.3059232	-427.2915592	EMD1
TGTTTGTTCTCGACGG-1	singlet	2	-3528.478293	-3324.883217	-1074.205309	EMD1
TTAATCCAGACGGATC-1	singlet	2	-379.3340371	-319.8272387	-156.6172088	EMD1
TTAATCCAGAGGGTAA-1	singlet	0	-1265.410967	-3995.10567	-4177.480105	EMD1
TTAATCCAGCAGCACA-1	singlet	2	-1996.62457	-1681.57821	-776.9566629	EMD1
TTAATCCAGCTGACCC-1	singlet	0	-342.2288756	-968.2993494	-907.4186511	EMD1
TTAATCCCAGTCGCTG-1	singlet	0	-309.5413117	-952.9301349	-873.09651	EMD1
TTAATCCGTATCGTTG-1	singlet	1	-535.2778408	-231.0401735	-553.9651242	EMD1
TTAATCCGTGGAACAC-1	singlet	1	-1676.973751	-502.9254928	-1356.360707	EMD1
TTAATCCTCATCACAG-1	singlet	0	-682.2515239	-2085.148899	-1888.008838	EMD1
TTAATCCTCGCGGACT-1	singlet	2	-278.8565328	-293.5926955	-121.7449881	EMD1
TTAATCCTCGGAAACG-1	singlet	0	-432.7238052	-1521.506388	-1472.810961	EMD1
TTAATCCTCTTGTGCC-1	singlet	0	-531.6237802	-1742.91066	-1830.738519	EMD1
TTACAGGAGGATGTTA-1	singlet	2	-595.6631271	-505.5635427	-234.421214	EMD1
TTACAGGCAAGCTCTA-1	singlet	2	-30177.69717	-23568.8568	-8863.624024	EMD1
TTACAGGCATGCCGAC-1	singlet	2	-866.8281622	-715.673235	-293.9143655	EMD1
TTACAGGGTACAAACA-1	singlet	2	-1542.431587	-1262.470211	-640.5280594	EMD1
TTACAGGGTCCCACGA-1	singlet	0	-1172.074097	-3595.360249	-3643.503167	EMD1
TTACAGGGTGGGCTCT-1	singlet	0	-36.38882183	-108.2385218	-105.8450909	EMD1
TTACAGGTCAAATGAG-1	singlet	0	-1086.346019	-3425.517004	-3236.607218	EMD1
TTACAGGTCAATCTTC-1	doublet	2/0	-837.6748633	-1018.597117	-508.2052144	EMD1
TTACAGGTCTACCACC-1	singlet	1	-4631.814597	-1542.505623	-3287.218345	EMD1
TTACAGGTCTAGGAAA-1	singlet	2	-2605.064122	-2064.096858	-945.923891	EMD1
TTACAGGTCTGCACCT-1	singlet	2	-8422.270427	-6527.861359	-2731.554013	EMD1
TTACAGGTCTGGTTGA-1	singlet	2	-421.5089181	-313.1919235	-126.235421	EMD1
TTACCATAGCATTTCG-1	singlet	0	-528.5255347	-1551.954746	-1602.888559	EMD1
TTACCATAGTTAGTAG-1	singlet	2	-136.7143423	-83.7034151	-49.05638546	EMD1
TTACCATCAAAGGCTG-1	singlet	2	-3127.805099	-2242.895795	-771.1112166	EMD1
TTACCATCAATTGCTG-1	doublet	45323	-5680.837564	-3477.463679	-3258.205386	EMD1
TTACCATGTCAGTCGC-1	singlet	1	-1822.322752	-628.4660995	-1453.218431	EMD1
TTACCATGTCCTTAAG-1	singlet	0	-317.7755161	-904.9544958	-898.3741553	EMD1
TTACCATGTTTCGACA-1	singlet	0	-182.4606033	-492.4660766	-544.8942328	EMD1
TTACCATTCATTCCTA-1	doublet	45293	-2985.985871	-1572.017313	-2173.090774	EMD1

TTACCATTCCATGATG-1	singlet	2	-804.0547753	-544.2625081	-285.9417193	EMD1
TTACCATTCCCTATTA-1	singlet	2	-407.9439016	-286.8782583	-151.5257866	EMD1
TTACCATTCGCCGTGA-1	singlet	2	-1294.032096	-945.9892208	-407.00298	EMD1
TTACCATTCTACGCAA-1	singlet	2	-326.3575228	-297.4306331	-149.2500925	EMD1
TTACCATTCTGGGATT-1	singlet	1	-2709.125168	-844.0052201	-2112.92812	EMD1
TTACCGCAGAGGGTAA-1	singlet	2	-245.2491509	-200.2499697	-104.4746274	EMD1
TTACCGCAGCTCGGCT-1	singlet	2	-438.9129867	-455.516182	-192.1777998	EMD1
TTACCGCCAAACACCT-1	singlet	2	-697.61525	-595.8722608	-262.7365055	EMD1
TTACCGCCAGAGAATT-1	singlet	1	-3695.239782	-1258.12512	-3025.358486	EMD1
TTACCGCCATTCCTAT-1	singlet	2	-87.65914286	-81.92907701	-43.38646585	EMD1
TTACCGCGTTAAACCC-1	singlet	2	-465.0368676	-352.9631842	-195.6323766	EMD1
TTACCGCGTTATCTTC-1	singlet	2	-487.8310494	-392.9605806	-207.8152267	EMD1
TTACCGCGTTTCGTGA-1	singlet	1	-368.8128141	-121.1449397	-419.7248578	EMD1
TTACCGCTCACGAGGA-1	singlet	1	-2095.818499	-603.216586	-1636.296705	EMD1
TTACCGCTCACGGAGA-1	unassigned	45323	-13.94710208	-10.70212409	-4.582559813	EMD1
TTACCGCTCCTGGTCT-1	singlet	2	-511.8637924	-475.2098635	-203.6100839	EMD1
TTACCGCTCGTGCAGC-1	doublet	2/0	-1388.003218	-1615.192895	-925.1288093	EMD1
TTACCGCTCTGTCGCT-1	singlet	1	-116.3723292	-50.42298698	-134.4858721	EMD1
TTACCGCTCTTGCGCT-1	singlet	0	-121.7600356	-274.4287868	-261.6148292	EMD1
TTACGCCAGCCGTTAT-1	singlet	0	-77.82221874	-338.5824223	-262.1456377	EMD1
TTACGCCAGGAGCAAA-1	doublet	0/2	-1046.036516	-1370.208626	-1082.416681	EMD1
TTACGCCAGGCCTTGC-1	singlet	2	-1762.575921	-1263.609503	-561.172234	EMD1
TTACGCCCAAATTGGA-1	singlet	2	-1370.829271	-1338.077651	-532.4938502	EMD1
TTACGCCGTGTGGACA-1	singlet	0	-262.8572575	-768.1810689	-710.4924044	EMD1
TTACGCCGTGTTCCTC-1	singlet	1	-1008.171537	-304.3048142	-796.9497111	EMD1
TTACGCCGTTGTTGCA-1	singlet	1	-1077.251018	-431.3436908	-875.949552	EMD1
TTACGCCTCGGTAACT-1	singlet	1	-6589.674442	-1617.690163	-5147.824239	EMD1
TTACGTTAGAGCAGTC-1	singlet	1	-3678.312364	-840.7489765	-3147.646348	EMD1
TTACGTTAGTTGTACC-1	singlet	0	-213.7383483	-555.6500863	-596.3689015	EMD1
TTACGTTCAAGAGATT-1	singlet	0	-1609.891034	-4416.156679	-4350.564474	EMD1
TTACGTTCAGTAACCT-1	singlet	0	-515.6696361	-1438.248834	-1396.081516	EMD1
TTACGTTCATCAGTCA-1	singlet	2	-2923.605603	-2323.174677	-992.7553053	EMD1
TTACGTTGTCATTCCC-1	singlet	1	-1464.209257	-492.1725792	-1200.566742	EMD1
TTACGTTGTGAACCGA-1	singlet	2	-826.5195679	-664.0018795	-334.2588374	EMD1
TTACGTTGTTCTTGTT-1	singlet	2	-194.3046987	-140.7807905	-43.37126233	EMD1
TTACGTTTCCTACGAA-1	doublet	0/2	-1137.74405	-2114.66153	-1765.274957	EMD1
TTACGTTTCCTGGTCT-1	singlet	2	-2268.62124	-1874.495093	-785.5186639	EMD1
TTACTGTCACAGCTGC-1	singlet	2	-41.78149522	-23.50566554	-15.71884144	EMD1
TTACTGTCAGGTCAAG-1	singlet	2	-342.8660057	-315.9480086	-156.2609708	EMD1
TTACTGTCATGTGGCC-1	singlet	1	-117.0800033	-44.26377507	-87.76557306	EMD1
TTACTGTGTACCTAAC-1	singlet	0	-195.3516207	-589.6038554	-520.3770059	EMD1
TTACTGTGTATGCTTG-1	singlet	2	-290.5460564	-256.4599094	-118.9236998	EMD1
TTACTGTTCCAACTAG-1	singlet	0	-166.9014382	-407.1721426	-459.2188447	EMD1
TTACTGTTCCACTAGA-1	singlet	0	-2303.828161	-6721.456564	-6146.460001	EMD1
TTACTGTTCGCATTAG-1	singlet	1	-831.1252033	-307.5602618	-1016.457243	EMD1
TTACTGTTCGCTTAAG-1	singlet	1	-6878.347763	-2108.399193	-5356.514455	EMD1
TTACTGTTCTGTCCGT-1	singlet	2	-1314.638119	-1133.13925	-466.1110705	EMD1
TTAGGCAAGAATAGTC-1	singlet	0	-279.1686022	-944.4275234	-884.2537762	EMD1
TTAGGCAAGCTCCCTT-1	singlet	2	-1361.494013	-1235.96015	-484.3922263	EMD1
TTAGGCAAGTAACCGG-1	singlet	1	-1076.332632	-345.4856712	-915.6853591	EMD1
TTAGGCACAACCGGAA-1	singlet	2	-1424.549449	-1265.571525	-479.8683997	EMD1
TTAGGCACAGGTCAAG-1	singlet	1	-684.8416126	-288.1090273	-659.4903779	EMD1
TTAGGCACAGGTCAGA-1	singlet	2	-1143.452628	-1207.498139	-377.7595112	EMD1
TTAGGCACATGACAAA-1	singlet	2	-2388.427246	-2209.085555	-860.4880708	EMD1
TTAGGCAGTCAAGCCC-1	singlet	1	-2301.93289	-721.8931895	-1657.096065	EMD1
TTAGGCAGTGACCGAA-1	singlet	1	-468.7627536	-204.6805435	-457.0358822	EMD1
TTAGGCAGTGGTCCGT-1	singlet	2	-373.2012592	-288.4803756	-142.8444582	EMD1
TTAGGCAGTTAATGAG-1	singlet	2	-1632.526605	-1306.130744	-632.4312448	EMD1
TTAGGCATCTACTCAT-1	singlet	0	-636.6963703	-1819.589791	-1660.14398	EMD1
TTAGGGTAGATGGCGT-1	singlet	1	-11498.51191	-3499.468876	-9234.522399	EMD1
TTAGGGTAGCGTGTCC-1	singlet	1	-445.3485545	-145.4959556	-372.6034315	EMD1
TTAGGGTAGTAAAGCT-1	singlet	2	-673.9622568	-508.6414066	-236.7362638	EMD1
TTAGGGTAGTTGCGCC-1	singlet	2	-455.5800598	-344.5835774	-172.6465832	EMD1
TTAGGGTCAACGGTAG-1	singlet	1	-622.4816233	-241.5007321	-459.5338248	EMD1
TTAGGGTCAAGAAATC-1	singlet	2	-3141.975254	-2495.431054	-1003.771177	EMD1
TTAGGGTCACAACATC-1	singlet	1	-4186.204329	-1410.242992	-3282.007779	EMD1
TTAGGGTCACCGAATT-1	singlet	2	-652.3951052	-527.5543477	-230.0114015	EMD1
TTAGGGTCACTGTCGG-1	singlet	2	-447.1261675	-408.7325219	-206.1603696	EMD1
TTAGGGTCAGACATCT-1	singlet	2	-474.1374951	-400.1130319	-183.1604223	EMD1
TTAGGGTCAGTGTATC-1	singlet	0	-1142.358418	-3657.436721	-3610.321655	EMD1
TTAGGGTGTCACTTAG-1	singlet	0	-771.1627994	-2449.946785	-2327.822834	EMD1
TTAGGGTGTTGTGGCC-1	singlet	0	-1544.021478	-3436.839692	-3166.254301	EMD1
TTAGGGTTCAAGATAG-1	singlet	1	-2016.271422	-731.0960662	-1624.346746	EMD1
TTAGGGTTCCTGTACC-1	singlet	2	-3288.362392	-2598.475808	-1005.160791	EMD1
TTAGGGTTCCTTCTAA-1	singlet	1	-530.3276663	-176.573881	-552.6552762	EMD1
TTAGGGTTCGCTCCTA-1	singlet	2	-675.306649	-519.4967547	-221.3428091	EMD1
TTAGTCTAGCCACTCG-1	singlet	0	-441.6912049	-1481.35974	-1673.630145	EMD1
TTAGTCTAGGCACAAC-1	singlet	0	-760.0412017	-2357.382591	-2399.711672	EMD1
TTAGTCTAGGTAGTCG-1	singlet	1	-864.9542143	-260.9502906	-597.7266581	EMD1
TTAGTCTCACAAGCCC-1	singlet	2	-915.1469027	-792.6356322	-329.2726497	EMD1
TTAGTCTCAGGTAGTG-1	singlet	0	-176.5909292	-425.4413496	-517.456779	EMD1
TTAGTCTGTAATGCTC-1	singlet	2	-544.1662703	-463.6752134	-162.9411277	EMD1
TTAGTCTGTCCTATAG-1	singlet	2	-692.3146737	-667.1858169	-245.3487774	EMD1
TTAGTCTTCCAACTAG-1	singlet	0	-273.4308288	-1195.096564	-919.5042755	EMD1
TTATTGCAGAAGTCTA-1	singlet	2	-380.0928379	-302.3967804	-174.9852283	EMD1
TTATTGCAGAGCCGTA-1	singlet	0	-150.4170504	-367.9846569	-361.6949786	EMD1
TTATTGCAGAGTTCGG-1	singlet	0	-112.1499715	-393.9498635	-407.6536829	EMD1
TTATTGCAGCCTTGAT-1	singlet	2	-2002.469619	-1684.629588	-644.0297949	EMD1
TTATTGCAGGTTATAG-1	singlet	1	-948.8278636	-322.465033	-915.1257801	EMD1
TTATTGCTCACACCGG-1	singlet	1	-692.4860258	-175.000185	-566.3107198	EMD1
TTATTGCTCTCCCATG-1	singlet	0	-270.5582042	-694.3270516	-673.118272	EMD1
TTATTGCTCTCTGAGA-1	singlet	2	-488.1245843	-456.9119607	-196.6129665	EMD1
TTATTGCTCTTCGATT-1	singlet	2	-650.9338414	-540.1433185	-273.9862141	EMD1
TTCAATCAGAAGCCAC-1	singlet	2	-352.001551	-282.7174174	-129.0022137	EMD1
TTCAATCAGACATATG-1	singlet	2	-1490.755606	-1419.05567	-543.9133523	EMD1
TTCAATCAGATACCAA-1	singlet	1	-475.0845498	-203.3613674	-510.5334773	EMD1
TTCAATCAGATGCGAC-1	singlet	2	-770.5080837	-656.7608272	-280.4910571	EMD1
TTCAATCAGCATGAAT-1	singlet	0	-292.9533726	-671.6356909	-888.3984794	EMD1
TTCAATCAGCGTATAA-1	singlet	1	-1787.981846	-488.9702272	-1332.793423	EMD1
TTCAATCAGTGAACAT-1	doublet	2/0	-1993.136863	-2355.989485	-1329.563287	EMD1
TTCAATCCAATGTCAC-1	singlet	0	-814.5752546	-2607.322847	-2585.669894	EMD1
TTCAATCCAGCTTTCC-1	singlet	1	-3689.415315	-1578.795717	-2891.413262	EMD1
TTCAATCGTAAGCTCT-1	singlet	0	-1071.462517	-2879.874922	-3098.658742	EMD1
TTCAATCGTCCATAGT-1	singlet	0	-33.63351704	-125.0867734	-113.3684631	EMD1
TTCAATCGTCTTGAAC-1	singlet	1	-331.7261872	-105.7772967	-266.0505693	EMD1
TTCAATCGTTAGGGTG-1	singlet	1	-1887.224822	-695.9242631	-1599.940294	EMD1
TTCAATCTCGAAGAAT-1	singlet	1	-536.0289155	-199.711439	-480.2558963	EMD1
TTCAATCTCTGCTTAT-1	singlet	0	-354.2927286	-1021.410603	-935.1086845	EMD1
TTCAATCTCTGTCTCG-1	singlet	2	-625.8956843	-710.2021968	-285.8871328	EMD1
TTCACCGAGGGAGGGT-1	singlet	1	-4185.363368	-1741.992541	-3721.897053	EMD1

TTCACCGCACGCTGCA-1	singlet	0	-258.7675815	-633.1618045	-593.0653421	EMD1
TTCACCGGTGGTCTTA-1	singlet	2	-1263.702751	-1143.469749	-413.1865122	EMD1
TTCACCGGTGTGTGTT-1	singlet	2	-485.7423642	-461.9731571	-214.429342	EMD1
TTCACCGGTGTTAACC-1	singlet	1	-9804.258191	-2730.718717	-7325.140558	EMD1
TTCACCGTCTATCGCC-1	singlet	2	-4531.639326	-3689.819842	-1601.123746	EMD1
TTCACGCAGACCGCCT-1	singlet	0	-1064.690456	-3071.905708	-3143.106495	EMD1
TTCACGCAGATGCAGC-1	singlet	0	-383.7855042	-993.6823455	-916.1955379	EMD1
TTCACGCAGCGGATCA-1	doublet	2/0	-6579.772653	-7806.539053	-4989.368441	EMD1
TTCACGCAGGAGGGTG-1	singlet	2	-1939.271839	-1679.176653	-732.9601569	EMD1
TTCACGCAGTACGTCT-1	singlet	1	-830.7787203	-302.9923449	-645.9429325	EMD1
TTCACGCCACTCGATA-1	singlet	0	-357.0310969	-1098.431449	-1075.970544	EMD1
TTCACGCCAGCTTTCC-1	singlet	2	-232.8389667	-227.5222604	-97.6680432	EMD1
TTCACGCCAGGGACTA-1	singlet	0	-192.0713445	-520.4479921	-619.1893392	EMD1
TTCACGCGTGTTCATG-1	singlet	1	-4275.246666	-985.7677849	-3326.235226	EMD1
TTCACGCGTTCTTGCC-1	singlet	0	-951.870456	-2474.838156	-2567.174434	EMD1
TTCACGCTCCCTTGGT-1	singlet	0	-83.84264023	-235.4585377	-255.2574726	EMD1
TTCACGCTCCGTTGGG-1	singlet	1	-1012.754814	-372.8414641	-831.772157	EMD1
TTCACGCTCCTCTTTC-1	singlet	1	-61.77029854	-25.28092144	-50.26280792	EMD1
TTCACGCTCCTGGCTT-1	singlet	0	-506.2074223	-1537.801824	-1551.487851	EMD1
TTCACGCTCGGCACTG-1	singlet	2	-506.996205	-449.8538031	-214.9669779	EMD1
TTCAGGAAGATGTTAG-1	singlet	0	-383.6430279	-1004.056781	-968.985519	EMD1
TTCAGGAAGCTAGATA-1	singlet	0	-235.0639124	-606.976205	-566.110066	EMD1
TTCAGGACAAACCACT-1	singlet	0	-1324.033631	-3818.810798	-3673.493229	EMD1
TTCAGGACACCAATTG-1	singlet	2	-1694.711946	-1374.978285	-602.8443591	EMD1
TTCAGGACACGGAAGT-1	singlet	1	-1509.410564	-489.3142551	-1301.838717	EMD1
TTCAGGACACGTCGTG-1	singlet	0	-145.6790892	-462.8571794	-490.3766777	EMD1
TTCAGGACAGAAGCTG-1	singlet	2	-399.5593548	-339.994418	-135.4842952	EMD1
TTCAGGACATACTGTG-1	singlet	2	-619.5375014	-531.2319309	-252.1190741	EMD1
TTCAGGACATGCCGAC-1	singlet	0	-240.5272853	-625.0300942	-811.6792389	EMD1
TTCAGGAGTACTGCGC-1	singlet	0	-40.09446083	-55.0048818	-285.3483662	EMD1
TTCAGGAGTTGCATGT-1	singlet	0	-830.0031329	-1880.912539	-1770.488245	EMD1
TTCAGGATCCAACACA-1	singlet	2	-1710.100156	-1359.591438	-582.4675293	EMD1
TTCAGGATCCGTATAG-1	singlet	1	-450.8224727	-183.3934277	-357.7795889	EMD1
TTCAGGATCGACACCG-1	singlet	2	-567.0066269	-397.2098808	-190.8901978	EMD1
TTCAGGATCGTGCGAC-1	doublet	1/0	-2120.488295	-1594.719856	-2056.258082	EMD1
TTCATGTAGACCCTTA-1	singlet	2	-400.7141004	-393.801476	-132.5819415	EMD1
TTCATGTCATAAGCAA-1	singlet	0	-255.1733083	-984.2132713	-982.1366829	EMD1
TTCATGTGTCACTCTC-1	doublet	1/0	-992.0568418	-789.4098584	-1155.554382	EMD1
TTCATGTGTGACGCCT-1	singlet	0	-87.73527565	-294.4108374	-348.1586238	EMD1
TTCATGTGTGTGTACT-1	singlet	1	-1648.347894	-439.7456389	-1251.365921	EMD1
TTCATGTGTGTTAAAG-1	singlet	1	-269.4536811	-104.868373	-221.0147393	EMD1
TTCATGTGTTGTGGCC-1	singlet	2	-461.4055389	-366.8939087	-172.7818905	EMD1
TTCATGTTCGAGAAGC-1	singlet	1	-1618.476778	-677.8715328	-1459.896191	EMD1
TTCATGTTCTCTCCGA-1	singlet	1	-2830.833003	-982.5790231	-2210.597587	EMD1
TTCATTGAGGAACGAA-1	singlet	2	-1228.266884	-1079.634522	-394.0266606	EMD1
TTCATTGCAACACACT-1	singlet	1	-10420.69102	-2545.322094	-7349.451592	EMD1
TTCATTGCAGCCATTA-1	singlet	2	-499.3354964	-390.3805335	-204.2842144	EMD1
TTCATTGGTATCGAGG-1	singlet	2	-1065.179801	-867.4552618	-406.7049863	EMD1
TTCATTGTCTCGTCGT-1	unassigned	45293	-31.02506688	-13.84800551	-19.34556803	EMD1
TTCCAATAGTATGATG-1	singlet	2	-526.9953124	-468.3373573	-229.023645	EMD1
TTCCAATAGTGGAAGA-1	singlet	2	-1888.067349	-1497.575454	-604.7411383	EMD1
TTCCAATAGTTCCGGC-1	singlet	0	-1062.447451	-3433.309638	-3231.022363	EMD1
TTCCAATCAGATACTC-1	singlet	0	-400.7900516	-1278.399146	-1186.233156	EMD1
TTCCAATGTAACGATA-1	singlet	0	-1521.633095	-4263.341515	-4124.419916	EMD1
TTCCAATGTAGGTACG-1	singlet	2	-405.8540373	-360.1680615	-161.211793	EMD1
TTCCAATGTCGACTGC-1	singlet	1	-2987.768578	-989.7953964	-2134.629562	EMD1
TTCCAATGTTGAGAGC-1	singlet	2	-443.7346422	-345.9540092	-181.8389282	EMD1
TTCCAATTCATACGGT-1	singlet	2	-487.166792	-376.5666495	-201.7162044	EMD1
TTCCAATTCCAGTACA-1	singlet	0	-281.9076818	-733.2105549	-834.2822227	EMD1
TTCCAATTCCATACAG-1	singlet	1	-2059.707122	-725.6921373	-1607.733108	EMD1
TTCCAATTCTGAGGTT-1	singlet	0	-225.4105033	-877.0597021	-968.6970856	EMD1
TTCCACGCATTAGGCT-1	singlet	1	-1648.825448	-555.25734	-1394.513955	EMD1
TTCCACGGTAAGTAGT-1	singlet	0	-292.3871601	-1033.276831	-928.7006615	EMD1
TTCCACGGTCTCACAA-1	doublet	2/0	-815.7004131	-1199.764405	-779.4843318	EMD1
TTCCACGGTCTCAGGC-1	singlet	2	-719.5707058	-561.916323	-291.8750783	EMD1
TTCCACGGTTGGTAGG-1	singlet	1	-3117.564119	-589.3976064	-2509.799278	EMD1
TTCCACGGTTGTGGCC-1	singlet	2	-651.6692468	-533.6424865	-224.5777461	EMD1
TTCCACGTCAGTAGGG-1	singlet	0	-6.248265351	-9.965054886	-11.01965909	EMD1
TTCCGGTAGAATACAC-1	singlet	1	-2105.519348	-810.756279	-1640.458502	EMD1
TTCCGGTAGATTCGCT-1	singlet	0	-408.1599104	-874.1327814	-858.37223	EMD1
TTCCGGTAGGGCAGTT-1	singlet	1	-248.7085933	-117.1246279	-195.2131377	EMD1
TTCCGGTCAATCGTCA-1	singlet	0	-220.1497906	-599.8684751	-662.7890236	EMD1
TTCCGGTCAATCTAGC-1	singlet	2	-658.9348656	-608.155547	-273.6176364	EMD1
TTCCGGTCAGGAAGTC-1	singlet	2	-559.2145961	-450.3030281	-216.948003	EMD1
TTCCGGTCATACAGCT-1	singlet	0	-2820.358251	-9497.549641	-8785.532483	EMD1
TTCCGGTGTAGCTAAA-1	singlet	2	-250.0408848	-247.3295209	-120.0952267	EMD1
TTCCGGTGTTTCAGAC-1	singlet	2	-715.2879015	-544.0015486	-261.5431825	EMD1
TTCCGGTTCAGACCCG-1	singlet	2	-524.2614225	-437.8353176	-202.0901102	EMD1
TTCCGGTTCCACGAAT-1	singlet	2	-717.2661203	-502.4645164	-295.8443861	EMD1
TTCCGGTTCGAATGCT-1	singlet	1	-2767.023124	-939.0177209	-2148.729774	EMD1
TTCCGGTTCGCACGGT-1	singlet	2	-4124.145507	-3323.147444	-1212.582152	EMD1
TTCCGGTTCGGTCATA-1	singlet	0	-514.9644643	-2594.809115	-2222.929177	EMD1
TTCCGGTTCGGTTAGT-1	doublet	2/0	-392.5189692	-449.5090966	-303.5626913	EMD1
TTCCGGTTCTTCTAAC-1	singlet	2	-779.3362933	-613.3000843	-286.4871767	EMD1
TTCCGTGAGGTACCTT-1	singlet	2	-860.5316346	-667.7527957	-310.0492414	EMD1
TTCCGTGCACATGTTG-1	singlet	0	-1978.788475	-6669.119593	-7089.508393	EMD1
TTCCGTGCAGAGAATT-1	singlet	0	-298.1615984	-930.0252106	-887.127895	EMD1
TTCCGTGCATTCGGGC-1	singlet	1	-3157.970039	-911.7944948	-2172.568044	EMD1
TTCCGTGGTACGAAAT-1	singlet	1	-433.9356131	-201.667983	-374.8723369	EMD1
TTCCGTGGTGATGAAT-1	singlet	2	-2818.335948	-2934.0384	-892.1047845	EMD1
TTCCGTGTCGTTACCC-1	doublet	0/2	-95.60592171	-173.8920827	-194.4821462	EMD1
TTCCGTGTCTCACTCG-1	singlet	2	-635.942899	-550.5706571	-208.945487	EMD1
TTCCGTGTCTTCGCTG-1	singlet	1	-446.7315729	-177.0320339	-327.0656738	EMD1
TTCCTAAAGCCATTTG-1	singlet	0	-99.14993749	-436.6315626	-332.8937355	EMD1
TTCCTAAAGTGAACAT-1	singlet	2	-568.1960713	-497.9147433	-224.2901042	EMD1
TTCCTAAGTATTCCGA-1	singlet	2	-1081.970021	-907.2025198	-425.8234604	EMD1
TTCCTAAGTGAGTCAG-1	singlet	2	-838.929307	-665.9012191	-301.307503	EMD1
TTCCTAAGTGGGCTTC-1	singlet	0	-397.1992704	-1100.647331	-1116.283827	EMD1
TTCCTAAGTTCTCGCT-1	singlet	2	-1236.220127	-1131.037296	-429.9897776	EMD1
TTCCTAAGTTGAGGAC-1	singlet	1	-745.0822298	-257.6921161	-732.3817401	EMD1
TTCCTAATCAACCTCC-1	singlet	2	-732.0910563	-644.0520179	-281.0075698	EMD1
TTCCTAATCGTTCTCG-1	singlet	0	-130.612052	-269.7675963	-320.3698614	EMD1
TTCCTAATCTCGTCAC-1	singlet	0	-1199.77876	-3285.944132	-3029.88204	EMD1
TTCCTAATCTGACCCT-1	singlet	0	-891.3188739	-2643.933651	-2150.749923	EMD1
TTCCTCTAGATCCCGC-1	singlet	1	-1049.585929	-365.7023352	-940.4650823	EMD1
TTCCTCTAGCGGTATG-1	singlet	2	-424.6265511	-349.4685384	-178.1375024	EMD1
TTCCTCTAGGATGAGA-1	singlet	1	-4027.940231	-1528.911873	-3026.166935	EMD1
TTCCTCTAGGCAGGGA-1	singlet	2	-296.5839918	-243.3757899	-117.9236856	EMD1
TTCCTCTAGGCTCTCG-1	singlet	2	-578.9176941	-538.0680459	-260.2873851	EMD1
TTCCTCTAGTCAAGCG-1	singlet	2	-10029.78141	-8098.588258	-3449.383846	EMD1

TTCCTCTAGTGTTCAC-1	singlet	2	-414.953379	-312.1887407	-154.7174382	EMD1
TTCCTCTCACCAAATC-1	singlet	1	-479.8986347	-172.3457713	-401.3159441	EMD1
TTCCTCTCACTGGCGT-1	singlet	1	-1576.017445	-578.1978157	-1155.61761	EMD1
TTCCTCTCATAACCCA-1	singlet	0	-255.3336614	-701.3777901	-687.233757	EMD1
TTCCTCTGTATTGAGA-1	singlet	0	-474.8650074	-1579.684326	-1380.870271	EMD1
TTCCTCTGTGGCTTGC-1	singlet	1	-690.5898458	-262.993957	-498.0826245	EMD1
TTCCTCTGTGTAGGAC-1	singlet	1	-3987.410101	-954.9220432	-3295.403791	EMD1
TTCCTCTTCACAGTGT-1	unassigned	0	-1.497781432	-3.928911061	-4.133662923	EMD1
TTCCTCTTCGTGAGAG-1	singlet	0	-155.8094741	-485.7553097	-396.1283804	EMD1
TTCCTTCAGGTGATAT-1	singlet	2	-419.3255403	-376.6033069	-182.8874013	EMD1
TTCCTTCCAAGCAGGT-1	singlet	0	-918.6070808	-2496.415836	-2528.54162	EMD1
TTCCTTCCAATGGGTG-1	singlet	2	-770.5141726	-623.2157185	-245.987132	EMD1
TTCCTTCCAGACTCTA-1	singlet	1	-2137.786185	-530.8592292	-1760.204574	EMD1
TTCCTTCCAGCGAACA-1	singlet	2	-687.6245827	-596.416296	-312.3408354	EMD1
TTCCTTCCAGCTATAC-1	singlet	2	-1771.342248	-1454.704565	-648.2467198	EMD1
TTCCTTCGTACTTCCC-1	singlet	2	-1599.389328	-1493.373537	-605.1632001	EMD1
TTCCTTCGTAGAGTTA-1	singlet	0	-635.4512352	-2439.028136	-2176.634694	EMD1
TTCCTTCGTCTCACGG-1	singlet	2	-1582.697787	-1457.737895	-666.1809174	EMD1
TTCCTTCTCCAGCAAT-1	singlet	2	-355.8805825	-371.2523298	-152.0985019	EMD1
TTCCTTCTCCTAAACG-1	singlet	1	-1386.970146	-457.2186948	-1264.640764	EMD1
TTCGATTAGCAATAAC-1	singlet	2	-779.2848981	-704.5207313	-350.9227948	EMD1
TTCGATTAGGACATCG-1	singlet	2	-2312.671282	-1754.4776	-646.3777324	EMD1
TTCGATTCAAGATCCT-1	singlet	2	-1078.880664	-940.0855547	-377.015095	EMD1
TTCGATTCAGCGTGCT-1	singlet	0	-605.1874055	-1976.91812	-1924.450768	EMD1
TTCGATTCATAAGCAA-1	singlet	1	-4763.765606	-1634.711789	-4148.753248	EMD1
TTCGATTCATGGGAAC-1	singlet	0	-841.1554009	-2297.319199	-2425.816454	EMD1
TTCGATTGTAGTCACT-1	singlet	2	-812.718717	-724.338457	-328.8593095	EMD1
TTCGATTTCAAGTGTC-1	singlet	2	-3560.269075	-2652.532377	-940.209801	EMD1
TTCGATTTCAGTCCGG-1	singlet	1	-4649.733841	-1822.837126	-3387.948358	EMD1
TTCGCTGAGCTCCGAC-1	singlet	2	-2588.24964	-2316.775142	-901.8207166	EMD1
TTCGCTGCACCCTCTA-1	singlet	2	-321.6929418	-267.3415653	-123.5263751	EMD1
TTCGCTGCACTTGTGA-1	singlet	2	-437.2088499	-431.5631701	-172.4775993	EMD1
TTCGCTGTCGCTAATG-1	singlet	2	-2149.141629	-1780.92509	-796.9591713	EMD1
TTCGGTCAGGTGCGAT-1	singlet	2	-337.2130582	-261.0912199	-108.0175303	EMD1
TTCGGTCAGTTAGTAG-1	singlet	0	-172.1459177	-526.2613283	-586.7460275	EMD1
TTCGGTCCAGATTTCG-1	singlet	1	-3783.364215	-905.2887037	-3245.753953	EMD1
TTCGGTCTCAGCCTTC-1	singlet	2	-556.5290101	-391.9880311	-203.9915562	EMD1
TTCGGTCTCCAACTGA-1	singlet	1	-531.3922032	-210.189077	-536.0092645	EMD1
TTCGGTCTCCGAGGCT-1	singlet	2	-226.766903	-180.5872898	-88.22340944	EMD1
TTCTAACAGAATCGTA-1	singlet	1	-1195.515162	-393.17287	-918.6636015	EMD1
TTCTAACAGGTAGATT-1	singlet	0	-25.65977536	-81.99345894	-74.74144989	EMD1
TTCTAACAGTGGCCTC-1	singlet	0	-73.9594365	-200.1676545	-171.1991176	EMD1
TTCTAACAGTTAGAAC-1	doublet	45293	-1953.921034	-970.9696192	-1348.795702	EMD1
TTCTAACCAATTGGTC-1	singlet	0	-410.5328315	-1216.599523	-1081.744859	EMD1
TTCTAACCATGGATCT-1	singlet	1	-371.319896	-139.7836267	-252.6258064	EMD1
TTCTAACGTAGTACGG-1	singlet	0	-414.1891486	-1187.09298	-1161.596324	EMD1
TTCTAACGTCGACGCT-1	singlet	2	-173.3479338	-168.6648509	-66.03503046	EMD1
TTCTAACGTTAGTCGT-1	singlet	2	-1270.738543	-1199.490951	-441.6929746	EMD1
TTCTAACTCATCGACA-1	singlet	1	-1574.774791	-620.6292585	-1264.345916	EMD1
TTCTAACTCCATAGAC-1	singlet	2	-1648.589814	-1396.455412	-616.1274664	EMD1
TTCTAACTCTGCCTGT-1	singlet	1	-561.910643	-225.461759	-686.1196313	EMD1
TTCTAACTCTGGGATT-1	singlet	0	-291.5176912	-882.839277	-846.6170589	EMD1
TTCTAGTAGCGAGTCA-1	singlet	2	-531.1890726	-570.6797397	-256.4641989	EMD1
TTCTAGTAGGGTACGT-1	singlet	2	-1742.425414	-1448.822841	-602.3751268	EMD1
TTCTAGTAGTATTCCG-1	singlet	2	-706.1366787	-581.2602901	-279.2798476	EMD1
TTCTAGTAGTGCTCAT-1	singlet	2	-491.5074437	-371.4453027	-177.126545	EMD1
TTCTAGTCATAATCGC-1	singlet	2	-793.5143618	-748.8813341	-349.467944	EMD1
TTCTAGTCATGTTCAG-1	singlet	2	-761.2480992	-693.7293523	-287.2720921	EMD1
TTCTAGTGTGCAAGAC-1	singlet	0	-290.465955	-998.9718986	-972.8082806	EMD1
TTCTAGTGTGGATGAC-1	singlet	2	-330.7266939	-277.0719612	-127.0007155	EMD1
TTCTAGTGTTCGGCCA-1	singlet	2	-1353.261645	-1034.886313	-417.3590991	EMD1
TTCTAGTTCTACCTTA-1	singlet	0	-186.9257218	-424.7389958	-439.9810761	EMD1
TTCTAGTTCTGCGGCA-1	singlet	0	-919.1700041	-2618.163441	-2517.974366	EMD1
TTCTCTCAGCATCAGG-1	singlet	2	-398.0643398	-329.8693131	-187.2548955	EMD1
TTCTCTCAGGTTCAGG-1	singlet	0	-192.9701258	-651.7576043	-593.3919509	EMD1
TTCTCTCCAAATCCCA-1	singlet	2	-2082.931841	-1846.514447	-816.9853129	EMD1
TTCTCTCCAAGCGAGT-1	singlet	2	-384.042705	-302.9386059	-166.8845197	EMD1
TTCTCTCCATCGGATT-1	singlet	2	-438.5664842	-382.7456809	-182.077578	EMD1
TTCTCTCGTACTCGTA-1	singlet	2	-298.4635102	-289.6468242	-118.2197709	EMD1
TTCTCTCGTGGTCCCA-1	singlet	1	-1052.796334	-269.9162132	-768.2232656	EMD1
TTCTCTCGTTTACGTG-1	singlet	0	-569.6743651	-1493.376291	-1424.096047	EMD1
TTCTCTCTCCGTAGTA-1	singlet	2	-181.3432432	-133.884133	-60.4182333	EMD1
TTCTCTCTCGTTCTGC-1	singlet	2	-314.7374054	-244.3544455	-151.6934007	EMD1
TTCTGTAAGAACTCCT-1	singlet	2	-1575.329792	-1596.638139	-596.3215279	EMD1
TTCTGTAAGAAGCGGG-1	singlet	2	-409.1013575	-357.370002	-140.1371417	EMD1
TTCTGTAAGACCAGAC-1	unassigned	0/1	-23.29548028	-34.7047301	-262.212211	EMD1
TTCTGTAAGCATTGTC-1	singlet	0	-21.6734237	-77.40328584	-89.70138443	EMD1
TTCTGTAAGCTAAACA-1	singlet	2	-486.1111526	-448.1701548	-187.6548361	EMD1
TTCTGTACAAGATTGA-1	singlet	2	-343.9087895	-243.0144015	-154.3063119	EMD1
TTCTGTACACCTCTGT-1	singlet	1	-3381.269849	-1116.643554	-2647.487119	EMD1
TTCTGTACAGAGATGC-1	singlet	2	-724.3379621	-574.852072	-286.0369253	EMD1
TTCTGTACAGCATACT-1	singlet	2	-919.2072003	-822.5407966	-351.682761	EMD1
TTCTGTAGTTAAGCAA-1	singlet	2	-417.3580031	-328.4797204	-161.396259	EMD1
TTCTGTAGTTAGGACG-1	singlet	2	-793.0692774	-689.332467	-318.0794676	EMD1
TTCTGTATCAGAACCT-1	unassigned	0	-7.105566179	-7.558351038	-78.14291249	EMD1
TTCTTCCAGCTACTGT-1	singlet	2	-112.0904533	-122.2639208	-50.35430903	EMD1
TTCTTCCCACAGAGAC-1	singlet	2	-1179.315183	-889.7686982	-434.6234123	EMD1
TTCTTCCCATCTCAAG-1	doublet	45293	-2872.648012	-1764.875062	-1779.677108	EMD1
TTCTTCCGTATGCGGA-1	singlet	2	-495.1220109	-441.2105554	-218.5089139	EMD1
TTCTTCCGTCATATGC-1	singlet	2	-404.4950238	-386.3880701	-169.9351005	EMD1
TTCTTCCGTCTACGTA-1	singlet	1	-144.7315726	-39.21728729	-69.16783756	EMD1
TTCTTCCTCATGTCAG-1	singlet	0	-491.3380639	-1335.616748	-1305.17141	EMD1
TTCTTCCTCATTCGGA-1	singlet	2	-2956.504742	-2350.835249	-856.6358479	EMD1
TTCTTCCTCCAGGACC-1	singlet	1	-4104.066249	-1386.055955	-3212.071194	EMD1
TTCTTCCTCCATCACC-1	singlet	1	-1263.122848	-500.9479245	-996.3015274	EMD1
TTCTTCCTCCCAAGCG-1	doublet	0/2	-1045.887748	-1614.840869	-1287.40441	EMD1
TTCTTCCTCGCCTATC-1	doublet	1/0	-221.2584952	-160.1241821	-212.9551272	EMD1
TTCTTCCTCGTAGGGA-1	singlet	0	-793.5578701	-2895.568487	-2672.562291	EMD1
TTCTTGAAGGCTCTCG-1	singlet	0	-12.14785938	-73.61148033	-53.88234451	EMD1
TTCTTGAAGGTGCTTT-1	singlet	0	-375.0908393	-1135.818566	-996.9577194	EMD1
TTCTTGAAGTCTGCAT-1	singlet	2	-2168.950037	-1842.952887	-814.5969607	EMD1
TTCTTGACAATAGTGA-1	singlet	2	-564.6359566	-465.8334394	-243.373807	EMD1
TTCTTGACACCGTCTT-1	singlet	2	-1674.32206	-1253.357693	-552.9341052	EMD1
TTCTTGACACGACCTG-1	singlet	0	-400.3716058	-1232.200269	-1169.619342	EMD1
TTCTTGACATTCGGGC-1	singlet	2	-2125.369517	-1829.146553	-987.3234517	EMD1
TTCTTGACATTGGGAG-1	singlet	2	-464.7897828	-449.8471109	-204.7848754	EMD1
TTCTTGAGTATCAGGG-1	singlet	0	-478.6546537	-1534.317577	-1641.093265	EMD1
TTCTTGAGTCAAAGAT-1	singlet	2	-450.0043709	-382.2311764	-164.7745976	EMD1
TTCTTGAGTGGACTAG-1	singlet	1	-444.8246172	-185.7306038	-423.3535223	EMD1
TTCTTGATCACTGGTA-1	singlet	1	-1487.323804	-433.0802482	-1128.520318	EMD1

TTCTTGATCATGTCAG-1	doublet	45293	-4009.222047	-1888.197942	-2790.303018	EMD1
TTCTTGATCCTGTAAG-1	singlet	1	-3264.774251	-866.7030199	-2356.371541	EMD1
TTCTTGATCTGTCGTC-1	singlet	2	-229.8459227	-206.7866953	-104.6071136	EMD1
TTGAACGAGGCGCTCT-1	singlet	0	-306.3943657	-736.9993986	-758.4882812	EMD1
TTGAACGAGTGATGGC-1	singlet	2	-291.7356781	-226.5181085	-82.76287715	EMD1
TTGAACGGTACCAGAG-1	singlet	2	-862.6986483	-776.8704656	-390.1140403	EMD1
TTGAACGGTCAACATC-1	singlet	2	-1491.739783	-1387.054561	-491.2601044	EMD1
TTGAACGGTCGCATGC-1	singlet	0	-1458.916303	-4587.6893	-4232.624108	EMD1
TTGAACGGTGGCGCTT-1	singlet	1	-2067.634166	-666.5593994	-1661.569618	EMD1
TTGAACGTCAGATGCT-1	singlet	0	-507.4044962	-2024.68581	-1827.267616	EMD1
TTGAACGTCCATCTCG-1	singlet	0	-103.5074058	-309.0607561	-299.6312012	EMD1
TTGAACGTCGACCTAA-1	singlet	2	-280.264179	-260.9592184	-90.28640309	EMD1
TTGAACGTCGACGTCG-1	singlet	2	-298.3006865	-251.6466861	-86.71850263	EMD1
TTGACCCAGACATAAC-1	doublet	0/2	-3429.133245	-5375.78936	-3710.786408	EMD1
TTGACCCGTAAGATAC-1	singlet	0	-174.2276485	-470.4107222	-443.1756679	EMD1
TTGACCCGTGGCGCTT-1	singlet	0	-831.118394	-2328.752849	-2287.57201	EMD1
TTGACCCTCCACGAAT-1	singlet	2	-1054.813447	-954.1984921	-384.4827619	EMD1
TTGACCCTCCCTTGTG-1	singlet	0	-615.3266278	-2188.759606	-1969.632361	EMD1
TTGACCCTCGAGCACC-1	singlet	1	-99.90138987	-34.26514754	-87.19214444	EMD1
TTGACCCTCGATGGAG-1	singlet	0	-564.1274463	-1789.423465	-1592.794798	EMD1
TTGACCCTCGGTCAGC-1	singlet	1	-6164.263836	-1900.463248	-4169.212077	EMD1
TTGACCCTCGTCTCAC-1	singlet	2	-260.9595972	-191.6475255	-95.68213494	EMD1
TTGAGTGAGTAAGACT-1	singlet	1	-1497.011544	-546.0818475	-1130.491451	EMD1
TTGAGTGCAAACCATC-1	singlet	2	-6284.798796	-4915.87971	-2053.323825	EMD1
TTGAGTGCAACATACC-1	singlet	0	-286.8637872	-804.9014651	-961.2533232	EMD1
TTGAGTGCATCAGTCA-1	singlet	1	-599.5211282	-198.8309458	-406.2292093	EMD1
TTGAGTGCATCGGTTA-1	singlet	0	-43.87781081	-91.29378815	-94.83948714	EMD1
TTGAGTGCATTCCTCG-1	singlet	0	-356.2650023	-978.3688508	-958.6709181	EMD1
TTGAGTGGTACTGGGA-1	singlet	0	-329.4424126	-1113.345342	-982.1351438	EMD1
TTGAGTGGTGCAATAA-1	singlet	0	-148.2660298	-457.2368089	-435.8303904	EMD1
TTGAGTGGTTGCGGCT-1	singlet	1	-2310.825278	-757.8423798	-1851.033525	EMD1
TTGAGTGTCAAATAGG-1	singlet	0	-317.9553566	-808.0269431	-758.8821361	EMD1
TTGAGTGTCAAGCCCG-1	singlet	2	-362.8386688	-329.0541078	-185.9686336	EMD1
TTGAGTGTCATTACCT-1	singlet	2	-852.1172224	-826.564202	-378.6291174	EMD1
TTGAGTGTCCATAGAC-1	singlet	1	-1654.375136	-628.0238808	-1338.83075	EMD1
TTGAGTGTCGTAGAGG-1	doublet	45293	-6108.562442	-2713.843818	-3799.00055	EMD1
TTGATGGAGGGTTGCA-1	singlet	1	-2789.081021	-803.1588028	-2016.532528	EMD1
TTGATGGAGGTCCCTG-1	singlet	1	-256.0204061	-121.5317355	-234.3498796	EMD1
TTGATGGCAGGTTCAT-1	singlet	2	-158.9276312	-137.0814539	-68.21637719	EMD1
TTGATGGCATCGCCTT-1	singlet	0	-527.0172066	-1563.460696	-1646.465263	EMD1
TTGATGGGTTGTGTTG-1	singlet	1	-1583.453819	-371.999349	-1282.852302	EMD1
TTGATGGTCCTACCGT-1	singlet	0	-1282.876918	-3685.510611	-3608.077349	EMD1
TTGCATTAGACATGCG-1	singlet	2	-354.6793027	-297.7981426	-143.7425201	EMD1
TTGCATTAGACGGTCA-1	singlet	0	-264.505576	-725.5698338	-773.8030855	EMD1
TTGCATTAGGCATCAG-1	singlet	0	-240.6888578	-663.7510852	-675.4650544	EMD1
TTGCATTAGTTTCTTC-1	singlet	2	-395.6596695	-382.0061165	-155.2830859	EMD1
TTGCATTGTAAGGTCG-1	singlet	2	-5595.827259	-4482.771556	-1715.361548	EMD1
TTGCATTGTACACGCC-1	singlet	2	-525.27559	-442.4372581	-239.1073298	EMD1
TTGCATTGTATAATGG-1	singlet	2	-672.0214971	-513.1397317	-255.1337569	EMD1
TTGCATTGTCCTGGTG-1	singlet	2	-287.6855756	-264.1925718	-146.4170904	EMD1
TTGCATTGTCGGTGTC-1	singlet	2	-3710.646302	-3083.52001	-1211.428507	EMD1
TTGCATTGTGTCTCCT-1	singlet	0	-18.49712014	-34.56657985	-256.4849084	EMD1
TTGCATTTCAACTGAC-1	singlet	2	-732.2067492	-532.6709099	-237.2507266	EMD1
TTGCATTTCCTAGAGT-1	singlet	2	-531.302952	-441.0757655	-174.4992529	EMD1
TTGCATTTCTCCACTG-1	singlet	1	-2750.272472	-886.180502	-1967.585454	EMD1
TTGCCTGCAAGCGGAT-1	singlet	2	-500.799614	-459.3134842	-217.7898609	EMD1
TTGCCTGCAGGAAGTC-1	singlet	0	-235.9909308	-666.3567701	-774.1585557	EMD1
TTGCCTGCATGTGCTA-1	singlet	1	-2801.184647	-903.967985	-2126.038135	EMD1
TTGCCTGCATTACGGT-1	singlet	2	-1206.86455	-1095.764397	-493.8353548	EMD1
TTGCCTGGTTCGTGCG-1	singlet	0	-112.8337082	-356.4167224	-408.3926405	EMD1
TTGCGTCAGCCGTCGT-1	singlet	2	-385.3623404	-338.2023522	-166.0359053	EMD1
TTGCGTCAGCTGGCCT-1	singlet	0	-1263.543334	-3791.2407	-3727.357272	EMD1
TTGCGTCCACGTGTGC-1	singlet	2	-418.586289	-363.8527975	-176.3509852	EMD1
TTGCGTCGTATACAGA-1	singlet	1	-296.3410779	-89.93686912	-242.8483997	EMD1
TTGCGTCGTATCCCTC-1	unassigned	2/0	-126.3497911	-143.8705443	-64.71074698	EMD1
TTGCGTCTCATGCATG-1	singlet	1	-1732.359124	-510.1668093	-1398.718959	EMD1
TTGCGTCTCCTGGTCT-1	singlet	1	-1867.084689	-605.8355983	-1412.458924	EMD1
TTGCGTCTCGATTGAC-1	singlet	2	-342.2708661	-284.4049447	-132.6504379	EMD1
TTGCGTCTCGTTATCT-1	singlet	2	-844.6133396	-784.7857684	-287.6826484	EMD1
TTGCTGCAGAACGTGC-1	singlet	1	-4947.517521	-1349.04872	-3550.720856	EMD1
TTGCTGCAGGACACTG-1	singlet	0	-432.4724925	-1222.065462	-1195.380012	EMD1
TTGCTGCCACCTGTCT-1	singlet	2	-372.5150604	-302.1633179	-139.8067568	EMD1
TTGCTGCCAGTCGCTG-1	singlet	2	-1651.62234	-1438.837888	-584.6466511	EMD1
TTGCTGCGTCAGACTT-1	singlet	2	-1795.123113	-1491.566067	-646.9452347	EMD1
TTGCTGCGTGAATTAG-1	singlet	2	-1886.422525	-1470.958594	-643.4141519	EMD1
TTGCTGCGTGCGAGTA-1	singlet	2	-274.8337831	-240.5734125	-99.64140744	EMD1
TTGCTGCTCACCTGTC-1	singlet	0	-31.4962674	-130.2601641	-110.9995359	EMD1
TTGCTGCTCTCTTGCG-1	singlet	0	-3764.986847	-14484.07108	-12817.76914	EMD1
TTGGATGAGACTACCT-1	singlet	2	-665.137937	-592.0290601	-249.6312378	EMD1
TTGGATGAGCGATGAC-1	singlet	0	-796.4703084	-3531.526415	-2927.15635	EMD1
TTGGATGAGGCGAAGG-1	singlet	2	-547.7758874	-361.8017994	-184.6396289	EMD1
TTGGATGAGGTGATCG-1	singlet	2	-2743.962284	-2136.795045	-789.1915958	EMD1
TTGGATGAGTGGACGT-1	singlet	2	-367.7243205	-332.4440967	-177.4569615	EMD1
TTGGATGCAACACACT-1	singlet	0	-1166.769529	-3478.628019	-3492.363672	EMD1
TTGGATGCAAGCGAAC-1	singlet	2	-428.3721369	-308.709879	-143.2277554	EMD1
TTGGATGCAAGGTCTT-1	singlet	2	-422.7344892	-353.2403357	-177.1045349	EMD1
TTGGATGCATGCAGGA-1	singlet	1	-493.2437706	-205.4136665	-416.4279553	EMD1
TTGGATGGTCTGTGGC-1	singlet	1	-3103.912222	-1030.229297	-2334.829164	EMD1
TTGGATGGTTTGAACC-1	singlet	2	-399.8113814	-334.6858167	-172.3043996	EMD1
TTGGATGTCATGACAC-1	singlet	2	-4000.394283	-3206.003165	-1487.64532	EMD1
TTGGATGTCCACTTTA-1	singlet	2	-606.8591972	-553.9427744	-243.3254596	EMD1
TTGGATGTCGTAACTG-1	singlet	2	-644.2869943	-560.9981703	-265.4678356	EMD1
TTGGGATAGTAATACG-1	singlet	2	-315.6767875	-327.860053	-141.5296682	EMD1
TTGGGATCAGGTGTTT-1	singlet	2	-1460.33998	-1214.75347	-522.9531907	EMD1
TTGGGATCATCTTTCA-1	singlet	2	-347.2521787	-297.4354538	-132.6358361	EMD1
TTGGGATGTCTTCATT-1	singlet	2	-1493.479471	-1524.274585	-503.4853051	EMD1
TTGGGATGTGGATCAG-1	singlet	1	-1876.169224	-632.8258458	-1634.687265	EMD1
TTGGGATGTTAACAGA-1	singlet	2	-407.2261468	-393.0767231	-180.8817504	EMD1
TTGGGATTCCAAGAGG-1	singlet	1	-2041.537191	-732.4452344	-1631.422665	EMD1
TTGGGATTCCGTGTGG-1	singlet	1	-3828.378713	-1277.952202	-3114.471917	EMD1
TTGGGCGAGATCCTAC-1	singlet	2	-875.266661	-659.3373754	-262.0520824	EMD1
TTGGGCGAGATGCTAA-1	singlet	0	-833.6147649	-2437.422363	-2459.695893	EMD1
TTGGGCGAGGACTTCT-1	doublet	0/2	-809.5907436	-1289.494822	-1069.192598	EMD1
TTGGGCGCAAATCAAG-1	singlet	0	-567.5431311	-2025.845623	-1912.263392	EMD1
TTGGGCGCAATAGGGC-1	unassigned	0	-1.334791228	-1.405261155	-14.69734816	EMD1
TTGGGCGCAGGTGACA-1	singlet	2	-6139.795598	-5160.204798	-2011.039896	EMD1
TTGGGCGCATCACAGT-1	singlet	2	-1392.293871	-1303.463618	-576.802654	EMD1
TTGGGCGGTAACAGGC-1	singlet	1	-5316.275812	-1473.743016	-4127.506972	EMD1
TTGGGCGGTGTTAAAG-1	singlet	0	-838.387832	-2444.907892	-2453.957983	EMD1
TTGGGCGTCACCTGTC-1	singlet	1	-5841.526029	-1284.156048	-4568.949954	EMD1

TTGGGCGTCGCTAATG-1	singlet	0	-122.6318231	-362.5415465	-339.8173234	EMD1
TTGGGTAAGGTGCCTC-1	singlet	2	-1946.470736	-1719.580297	-718.1756788	EMD1
TTGGGTAAGTCGAAAT-1	singlet	1	-1788.765831	-653.6166787	-1516.765408	EMD1
TTGGGTAAGTTACGAA-1	singlet	2	-246.3022878	-213.309987	-92.83307424	EMD1
TTGGGTACAACGCATT-1	singlet	1	-1579.012006	-430.4505046	-1172.270533	EMD1
TTGGGTACAGACCTGC-1	singlet	2	-415.0769261	-349.5266892	-140.2972571	EMD1
TTGGGTAGTTAGGCCC-1	singlet	0	-126.9503468	-311.0675032	-267.0249925	EMD1
TTGGGTAGTTGCATTG-1	singlet	1	-6492.054679	-1531.656042	-4956.428118	EMD1
TTGGGTAGTTGCCGCA-1	singlet	2	-477.2152833	-444.6405519	-182.0595313	EMD1
TTGGGTATCAATCGGT-1	singlet	2	-624.5845188	-440.8877335	-187.1005338	EMD1
TTGGGTATCATTCTTG-1	singlet	2	-430.6539745	-364.6591858	-167.5420066	EMD1
TTGGGTATCCGGACGT-1	singlet	1	-2971.926466	-890.2616401	-2339.25009	EMD1
TTGGTTTAGCAGCAGT-1	singlet	0	-163.3972678	-456.9249673	-592.0016763	EMD1
TTGGTTTAGCGTTACT-1	singlet	2	-458.9576478	-329.7498599	-163.0608487	EMD1
TTGGTTTCACTGAGTT-1	singlet	2	-450.2929962	-480.1197469	-229.9810351	EMD1
TTGGTTTCATCGCCTT-1	singlet	2	-386.7388773	-277.3877708	-142.0335207	EMD1
TTGGTTTGTTCCATTT-1	singlet	0	-629.3187007	-1654.970813	-1872.828188	EMD1
TTGGTTTTCAGGAAGC-1	singlet	1	-4990.970422	-1662.124476	-3936.603026	EMD1
TTGGTTTTCCTTCTGG-1	singlet	0	-761.2688625	-2160.911646	-2074.29838	EMD1
TTGTGGAAGACAGTCG-1	unassigned	0	-1.272365682	-1.338323461	-5.865514467	EMD1
TTGTGGAAGAGTTGTA-1	singlet	2	-1268.880646	-958.1499098	-437.3992536	EMD1
TTGTGGAAGGCGAACT-1	singlet	1	-5727.300847	-1140.664616	-3872.230399	EMD1
TTGTGGACACTTCAAG-1	singlet	2	-105.779211	-63.10572135	-20.64548424	EMD1
TTGTGGAGTGCATGAG-1	singlet	0	-691.2821495	-1967.244853	-2062.958641	EMD1
TTGTGGAGTTCTCACC-1	singlet	1	-1021.491542	-263.4835668	-870.3498772	EMD1
TTGTGGATCAACACGT-1	singlet	2	-1974.678497	-1714.580158	-688.9076742	EMD1
TTGTGTTCATATCTCT-1	singlet	2	-606.5711376	-478.9263861	-219.2820427	EMD1
TTGTGTTGTCATAAAG-1	singlet	2	-1841.281467	-1484.013934	-640.0950523	EMD1
TTGTGTTGTCTTGTCC-1	singlet	0	-455.5754676	-1302.774177	-1258.207717	EMD1
TTGTGTTGTTTACCTT-1	singlet	0	-39.7156704	-144.4932736	-274.7947624	EMD1
TTGTGTTTCCCGGTAG-1	singlet	0	-543.4844001	-1617.606844	-1625.090017	EMD1
TTGTTCAAGAGTCTGG-1	singlet	2	-583.2413904	-530.966227	-247.8246939	EMD1
TTGTTCAAGGTACATA-1	singlet	0	-231.0232307	-560.3131109	-556.7710067	EMD1
TTGTTCAAGTTCCATG-1	singlet	1	-4715.849927	-1329.549112	-3691.829011	EMD1
TTGTTCACAGAGGTTG-1	singlet	0	-147.8602111	-533.6488893	-526.3988464	EMD1
TTGTTCACAGTCTTCC-1	singlet	0	-337.4085841	-884.4453654	-812.0543221	EMD1
TTGTTCATCCAAGGGA-1	singlet	1	-5308.076537	-1139.022339	-3736.53612	EMD1
TTGTTGTAGACCATTC-1	singlet	2	-479.4609977	-323.0877175	-165.0875789	EMD1
TTGTTGTAGAGAATCT-1	singlet	2	-3959.313801	-3137.606287	-1344.271781	EMD1
TTGTTGTAGATAGTCA-1	singlet	2	-466.9205416	-400.3004101	-176.7138999	EMD1
TTGTTGTAGATGGCAC-1	singlet	0	-159.5853234	-529.2413301	-525.1950471	EMD1
TTGTTGTAGCCATCCG-1	singlet	2	-1210.947847	-1116.619737	-299.7039408	EMD1
TTGTTGTAGCCTCATA-1	singlet	2	-702.0242886	-493.9819543	-211.0457295	EMD1
TTGTTGTAGTGGCGAT-1	singlet	2	-1117.714756	-923.3035003	-415.506021	EMD1
TTGTTGTAGTTACTCG-1	singlet	0	-1072.036585	-3424.048084	-3326.321379	EMD1
TTGTTGTCAACACAAA-1	singlet	1	-5070.080245	-1208.662174	-4245.936121	EMD1
TTGTTGTCAAGCGAGT-1	singlet	0	-498.3609138	-1452.867681	-1498.897192	EMD1
TTGTTGTCAAGCTCTA-1	singlet	0	-206.187035	-512.0463461	-685.9802679	EMD1
TTGTTGTCAAGTTTGC-1	singlet	2	-415.5933235	-398.4637045	-178.2749937	EMD1
TTGTTGTCACAACGTT-1	singlet	0	-665.0532379	-1948.084741	-2033.116359	EMD1
TTGTTGTCAGGCTATT-1	singlet	2	-758.9958921	-693.1863727	-327.8928968	EMD1
TTGTTGTCAGTCTACA-1	singlet	2	-1405.049963	-1307.941867	-562.693079	EMD1
TTGTTGTTCAAATGCC-1	singlet	2	-564.3586146	-427.2007708	-210.4061682	EMD1
TTGTTGTTCACATTGG-1	singlet	2	-278.2124561	-212.8421401	-89.37087816	EMD1
TTGTTGTTCAGGGTAG-1	singlet	2	-703.9643275	-405.8687088	-71.15970999	EMD1
TTGTTGTTCTGGAGAG-1	singlet	0	-89.61675194	-215.0980466	-240.0004739	EMD1
TTGTTTGAGACCTCAT-1	doublet	2/0	-790.9899237	-976.6080149	-702.0028086	EMD1
TTGTTTGAGACGCTCC-1	singlet	0	-297.6170161	-787.4421732	-974.094092	EMD1
TTGTTTGAGATACCAA-1	singlet	0	-38.18666154	-77.33854966	-56.82391539	EMD1
TTGTTTGAGCCGTAAG-1	singlet	0	-321.1145087	-986.1770663	-913.6365805	EMD1
TTGTTTGAGCTGGCTC-1	singlet	2	-285.5383753	-245.1450041	-119.9319306	EMD1
TTGTTTGAGGCGTTGA-1	singlet	0	-527.9223307	-1721.423432	-1640.694567	EMD1
TTGTTTGAGGGCTGAT-1	singlet	2	-375.7070705	-389.0020005	-150.9300652	EMD1
TTGTTTGCAAAGGTTA-1	singlet	2	-1196.663578	-1055.35717	-402.1468558	EMD1
TTGTTTGCAATGAACA-1	singlet	0	-104.3038349	-345.7550527	-314.4760495	EMD1
TTGTTTGCACAGTCAT-1	singlet	1	-3732.300156	-1296.014346	-2991.909172	EMD1
TTGTTTGCAGGAATAT-1	singlet	0	-565.5277761	-1548.294711	-1611.463082	EMD1
TTGTTTGCATCTATCT-1	singlet	2	-581.9403411	-533.1315682	-239.2092957	EMD1
TTGTTTGCATGTACGT-1	singlet	1	-3928.553765	-1185.175761	-3047.101526	EMD1
TTGTTTGCATTGCTTT-1	singlet	0	-143.9196304	-358.7071585	-344.2666048	EMD1
TTGTTTGGTTTGAACC-1	singlet	1	-3723.491537	-1238.102134	-2985.969697	EMD1
TTGTTTGTCAGACAAA-1	singlet	2	-669.6676	-500.0416832	-207.092123	EMD1
TTGTTTGTCCCGAGGT-1	singlet	2	-512.2007779	-420.7991405	-205.4271339	EMD1
TTGTTTGTCTCAACCC-1	singlet	2	-1042.619161	-842.8312067	-359.389651	EMD1
TTGTTTGTCTGCAGCG-1	singlet	1	-2166.785732	-460.2560861	-1470.776965	EMD1
TTTACCAAGACGCAGT-1	singlet	2	-319.4297135	-216.0659734	-118.1925469	EMD1
TTTACCACAATCCAGT-1	singlet	0	-484.7628521	-1328.08564	-1258.969772	EMD1
TTTACCACACTACCCT-1	singlet	2	-2131.42119	-1759.284341	-690.2275077	EMD1
TTTACCACAGTGTGCC-1	singlet	1	-70.52472201	-24.8154631	-54.42379919	EMD1
TTTACCAGTGGGTTGA-1	singlet	2	-198.2183526	-159.8994665	-105.2367606	EMD1
TTTACCATCATAGAGA-1	singlet	1	-884.9576288	-343.6988741	-850.9760274	EMD1
TTTACCATCTACGCAA-1	singlet	1	-430.92562	-185.3990706	-379.2330884	EMD1
TTTACGTAGAAACTGT-1	singlet	0	-1361.362221	-3694.658596	-3769.82298	EMD1
TTTACGTAGACTTCAC-1	singlet	0	-491.4374163	-1839.240304	-1572.867268	EMD1
TTTACGTAGGCTATCT-1	singlet	2	-356.4994304	-323.9830141	-148.7357789	EMD1
TTTACGTCAACACAAA-1	singlet	1	-1152.812491	-430.5341069	-898.2437715	EMD1
TTTACGTCACACACTA-1	singlet	2	-422.7408281	-332.4482156	-166.5350049	EMD1
TTTACGTGTATCATGC-1	doublet	45293	-3564.890377	-2027.755916	-2150.154447	EMD1
TTTACGTGTCAGACGA-1	singlet	2	-486.0740077	-434.2981993	-205.1294659	EMD1
TTTACGTGTTAAGGGC-1	singlet	2	-466.7480612	-460.8194569	-195.3606515	EMD1
TTTACGTTCAGAATAG-1	singlet	0	-271.5370448	-859.1728005	-845.8205972	EMD1
TTTACGTTCTGAGAAA-1	singlet	2	-480.8477415	-464.6076997	-222.2365493	EMD1
TTTACTGAGACAGCTG-1	singlet	1	-780.4232569	-297.8622257	-823.9144153	EMD1
TTTACTGAGACTCTTG-1	singlet	0	-1724.528368	-5751.046402	-5838.348623	EMD1
TTTACTGAGCGCCTTG-1	singlet	2	-1371.365413	-1270.745738	-502.0877852	EMD1
TTTACTGAGGTGCCTC-1	singlet	1	-216.5790211	-89.16008786	-206.9371518	EMD1
TTTACTGAGTACAGCG-1	singlet	1	-1836.480949	-603.8942423	-1469.661439	EMD1
TTTACTGAGTAGACAT-1	singlet	0	-762.3292367	-1818.888204	-1781.927973	EMD1
TTTACTGCAAGGTCAG-1	singlet	2	-452.7334907	-357.8359128	-174.9721722	EMD1
TTTACTGCACTTGAGT-1	singlet	1	-55.3317331	-19.09513851	-37.27641904	EMD1
TTTACTGCAGTAGGAC-1	singlet	0	-903.5043261	-3137.044971	-3194.977574	EMD1
TTTACTGCATATAGCC-1	singlet	0	-747.8485542	-2450.821999	-2205.779176	EMD1
TTTACTGCATCGATCA-1	singlet	2	-451.7492817	-393.3951692	-200.1489728	EMD1
TTTACTGGTCAGACGA-1	singlet	2	-634.2179514	-540.3181433	-243.4143803	EMD1
TTTACTGGTGGTCTAT-1	singlet	0	-14.60466335	-24.41420848	-216.4916665	EMD1
TTTACTGGTTCTCGCT-1	singlet	0	-411.7532214	-1329.857533	-1226.243141	EMD1
TTTACTGGTTTGAAAG-1	singlet	1	-776.4787674	-101.9543038	-700.3500094	EMD1
TTTACTGTCAATCCAG-1	singlet	1	-4364.619523	-1800.417714	-3054.512392	EMD1
TTTACTGTCACTGGGC-1	singlet	2	-649.6273161	-511.55802	-221.2925036	EMD1
TTTACTGTCCGTATAG-1	singlet	2	-293.7241893	-284.2297004	-122.4008576	EMD1

TTTACTGTCTCTATGT-1	singlet	2	-2910.515166	-2374.022112	-1057.735039	EMD1
TTTAGTCAGTTGAATG-1	singlet	2	-4879.056955	-3622.20514	-1651.762169	EMD1
TTTAGTCCAAAGCTAA-1	singlet	2	-8094.787143	-6591.460256	-2577.905018	EMD1
TTTAGTCCAGCACGAA-1	singlet	0	-216.5238117	-569.0076538	-581.7030208	EMD1
TTTAGTCCATTGCAAC-1	singlet	0	-385.0222681	-1056.011728	-974.6115225	EMD1
TTTAGTCGTGCGACAA-1	singlet	2	-246.1544522	-207.3213653	-107.616539	EMD1
TTTAGTCGTTGCTCCT-1	singlet	2	-357.7977125	-382.1073987	-175.3158221	EMD1
TTTATGCAGACGCCAA-1	singlet	1	-876.2216514	-360.4413004	-785.7224023	EMD1
TTTATGCAGTAACAGT-1	singlet	0	-365.6384185	-1169.442396	-1203.54791	EMD1
TTTATGCCAAATGCTC-1	singlet	2	-660.4678774	-659.4479802	-291.5462035	EMD1
TTTATGCCAACTGTGT-1	singlet	2	-567.3021811	-444.0198132	-202.3527314	EMD1
TTTATGCCAAGAAACT-1	singlet	1	-1434.976521	-454.7780537	-1190.710755	EMD1
TTTATGCCACAGCGCT-1	singlet	2	-305.2392962	-214.8849223	-92.55724347	EMD1
TTTATGCCACCCAAGC-1	singlet	2	-2090.42178	-1881.439793	-801.1620486	EMD1
TTTATGCCACTCATAG-1	singlet	2	-516.5762278	-422.8549981	-238.6205299	EMD1
TTTATGCCATGGCGCT-1	singlet	2	-1596.643544	-1465.294244	-621.3998939	EMD1
TTTATGCCATTGCAAC-1	singlet	2	-676.8049269	-473.3091772	-222.8014622	EMD1
TTTATGCGTATCCCTC-1	singlet	2	-1881.480024	-1537.376781	-765.7241085	EMD1
TTTATGCGTCATCCCT-1	singlet	0	-264.5526393	-740.5235684	-800.8747696	EMD1
TTTATGCTCTCGGGAC-1	singlet	1	-1935.447123	-550.489222	-1613.702065	EMD1
TTTATGCTCTGCTTTA-1	singlet	1	-2803.383311	-890.5538321	-1849.895443	EMD1
TTTATGCTCTTCCTAA-1	singlet	0	-1447.16698	-4414.408776	-4473.239249	EMD1
TTTCACAAGGTTTGAA-1	singlet	2	-694.7593607	-616.1958724	-239.0216371	EMD1
TTTCACACACCAAAGG-1	singlet	2	-4029.00988	-3265.358773	-1448.388623	EMD1
TTTCACACAGCAGTCC-1	singlet	0	-118.0626155	-471.578576	-413.7554611	EMD1
TTTCACACATGCCGGT-1	singlet	0	-21.757361	-72.85142133	-62.82669987	EMD1
TTTCACAGTAGTCGGA-1	singlet	2	-1319.857784	-1148.141375	-442.479974	EMD1
TTTCACAGTATCGTTG-1	singlet	2	-1795.96507	-1562.066062	-672.738274	EMD1
TTTCACAGTTACCCTC-1	singlet	1	-6453.295727	-1953.730371	-4922.935893	EMD1
TTTCACAGTTTGCCGG-1	singlet	0	-1979.108057	-5844.46652	-5896.482371	EMD1
TTTCACATCTGAGAGG-1	singlet	2	-397.015017	-321.5196676	-160.0208116	EMD1
TTTCACATCTGGTGCG-1	singlet	2	-495.6632693	-406.446925	-201.775071	EMD1
TTTCAGTAGGGTACAC-1	singlet	0	-171.8848717	-475.1592312	-447.86724	EMD1
TTTCAGTAGTGTTCCA-1	singlet	0	-406.4734036	-1254.987384	-1258.161871	EMD1
TTTCAGTCATCCGATA-1	singlet	0	-500.9165044	-1325.173323	-1438.713428	EMD1
TTTCAGTCATGACTGT-1	singlet	0	-364.2341286	-1064.180118	-1034.805491	EMD1
TTTCAGTGTACCATAC-1	singlet	2	-381.1459436	-343.3047365	-180.7662605	EMD1
TTTCAGTGTATAATGG-1	singlet	0	-126.6210844	-421.9087214	-386.5146153	EMD1
TTTCAGTGTGACTCTA-1	singlet	0	-492.9380507	-1267.946632	-1259.393347	EMD1
TTTCAGTTCGCTTTAT-1	singlet	0	-253.2038361	-762.9936688	-787.0769456	EMD1
TTTCAGTTCTTCGTGC-1	singlet	0	-438.1591722	-1118.225055	-1134.202539	EMD1
TTTCATGAGCGATGAC-1	singlet	1	-3097.354691	-762.0950548	-2136.468248	EMD1
TTTCATGAGGCGCTCT-1	singlet	1	-4961.055852	-1767.595758	-3884.730655	EMD1
TTTCATGCATGGCCAC-1	singlet	1	-677.0792095	-233.9951569	-533.6324791	EMD1
TTTCATGGTTCCACAA-1	singlet	0	-1444.822097	-4378.929642	-4152.628603	EMD1
TTTCATGTCAACTACG-1	doublet	45323	-4588.878436	-3303.511314	-2364.387327	EMD1
TTTCATGTCACGACTA-1	singlet	1	-3863.592769	-1131.286541	-3166.025461	EMD1
TTTCCTCCAATGTTGC-1	singlet	0	-469.5787826	-1349.262268	-1408.828362	EMD1
TTTCCTCCACTGCACG-1	singlet	2	-566.9077794	-574.4771129	-256.100277	EMD1
TTTCCTCGTGGAAATT-1	singlet	2	-1011.221444	-859.5626393	-343.6602482	EMD1
TTTCCTCGTGGTCAAG-1	singlet	2	-492.7422712	-418.4921805	-164.7592487	EMD1
TTTCCTCTCGGAATGG-1	singlet	2	-657.7617369	-545.0411307	-208.9556318	EMD1
TTTCCTCTCTCAGTCC-1	singlet	2	-6505.798825	-5216.076415	-2108.432543	EMD1
TTTCGATAGATCCTAC-1	singlet	2	-593.483212	-526.0130589	-289.9793546	EMD1
TTTCGATCACACGTGC-1	singlet	0	-49.94099952	-125.5398872	-127.911147	EMD1
TTTCGATCATCTGTTT-1	singlet	0	-1421.886021	-5015.105077	-4828.837712	EMD1
TTTCGATGTCACCACG-1	singlet	2	-646.6939773	-571.3321861	-237.8553208	EMD1
TTTCGATTCACGAGGA-1	singlet	0	-274.1476004	-661.1782961	-799.9086611	EMD1
TTTCGATTCAGTGTGT-1	singlet	1	-6947.862861	-2122.267143	-5651.803361	EMD1
TTTCGATTCATTACGG-1	singlet	2	-749.9258387	-570.9569402	-296.715347	EMD1
TTTCGATTCCAGCACG-1	singlet	1	-4047.933057	-1323.274742	-3324.627734	EMD1
TTTCGATTCCGTATGA-1	doublet	2/0	-3221.857165	-3007.970332	-2197.565314	EMD1
TTTCGATTCGCAATGT-1	singlet	2	-447.9953941	-389.2763054	-159.0053431	EMD1
TTTCGATTCTAGGAAA-1	singlet	2	-1764.20137	-1576.320779	-693.413738	EMD1
TTTCGATTCTCTAGGA-1	singlet	1	-2695.351937	-901.5480481	-2222.368047	EMD1
TTTGACTAGCGGTAAC-1	singlet	0	-970.4963616	-3448.7543	-3302.331301	EMD1
TTTGACTAGGGAGATA-1	singlet	2	-422.657838	-358.8859254	-170.0224418	EMD1
TTTGACTCAGAGAAAG-1	singlet	2	-3499.145746	-2774.886808	-1181.063824	EMD1
TTTGACTCATTGTAGC-1	singlet	0	-1365.789067	-3990.558203	-3744.350209	EMD1
TTTGACTGTCGGAAAC-1	singlet	1	-2301.153721	-746.9300402	-1885.094532	EMD1
TTTGACTGTGAATGTA-1	singlet	2	-3699.710204	-2973.597811	-1105.190238	EMD1
TTTGACTTCACAACCA-1	singlet	1	-724.361254	-288.276887	-640.5450294	EMD1
TTTGACTTCACCTTAT-1	singlet	2	-660.5128469	-536.5293347	-245.1209939	EMD1
TTTGACTTCCCTTCCC-1	doublet	2/0	-431.3181229	-516.1623163	-363.3728573	EMD1
TTTGACTTCTCGCTTG-1	singlet	1	-7554.726905	-2455.19733	-6120.359727	EMD1
TTTGATCAGATACGAT-1	singlet	2	-624.2815948	-474.5176929	-218.4870999	EMD1
TTTGATCGTCCTACGG-1	singlet	2	-278.7140078	-222.0647422	-95.98838099	EMD1
TTTGATCGTGAGTCAG-1	singlet	2	-354.5048855	-318.9947854	-151.9499156	EMD1
TTTGATCGTGGCTTGC-1	singlet	2	-114.6649978	-106.7295621	-56.43858981	EMD1
TTTGATCGTTGTAAAG-1	singlet	2	-415.307629	-355.5113906	-182.1871109	EMD1
TTTGATCTCGGTAGGA-1	singlet	2	-150.0129549	-140.044595	-62.72443113	EMD1
TTTGATCTCTGTTCAT-1	singlet	2	-6528.89865	-5263.474114	-1868.376377	EMD1
TTTGATCTCTTGGGCG-1	singlet	1	-122.0132441	-31.46913231	-81.94558984	EMD1
TTTGGAGAGAAGCGGG-1	singlet	1	-1589.931158	-662.6179012	-1357.173656	EMD1
TTTGGAGAGAAGTGTT-1	singlet	0	-136.1871217	-314.0314301	-333.5585002	EMD1
TTTGGAGAGTCATACC-1	singlet	2	-524.8801428	-387.8193366	-210.3860817	EMD1
TTTGGAGAGTCGAATA-1	singlet	0	-871.167365	-4392.427007	-3510.427644	EMD1
TTTGGAGCAGTCTTCC-1	singlet	2	-1458.549053	-1272.777283	-498.421729	EMD1
TTTGGAGGTCATCACA-1	singlet	2	-85.28887043	-62.35093393	-27.190439	EMD1
TTTGGAGGTCGCTTGG-1	singlet	0	-539.7375963	-1600.736517	-1553.754289	EMD1
TTTGGAGGTTGCTCCT-1	singlet	2	-614.16124	-504.202558	-229.4918536	EMD1
TTTGGAGTCAGCTAGT-1	singlet	0	-1547.543245	-3491.4348	-3567.881764	EMD1
TTTGGAGTCATTGTTC-1	singlet	2	-552.1041666	-479.0001594	-179.8307384	EMD1
TTTGGAGTCCTATTGT-1	singlet	2	-259.8671631	-259.4516176	-133.5889466	EMD1
TTTGGTTAGAGAGCCT-1	doublet	2/0	-469.3056649	-459.0290915	-308.2390085	EMD1
TTTGGTTAGGTGCATG-1	singlet	1	-3768.390782	-1071.336439	-2752.813227	EMD1
TTTGGTTAGTCCTACA-1	singlet	2	-527.2143008	-432.2300168	-194.8535046	EMD1
TTTGGTTCAGGACTAG-1	singlet	0	-329.5924898	-775.7313385	-799.4721655	EMD1
TTTGGTTGTACGAAAT-1	doublet	0/1	-1875.87406	-2018.456848	-2754.962567	EMD1
TTTGGTTGTCGCCACA-1	singlet	0	-136.6526045	-366.337802	-366.8647124	EMD1
TTTGGTTGTCGTGGTC-1	singlet	0	-209.7673342	-615.6872765	-629.3096905	EMD1
TTTGGTTGTTACGCCG-1	singlet	2	-483.7181524	-416.0950111	-212.1671151	EMD1
TTTGGTTTCTGAGTCA-1	singlet	1	-132.4083763	-39.69127484	-125.5103433	EMD1
TTTGTTGAGATGTAGT-1	singlet	2	-2229.275002	-1702.233175	-720.4419152	EMD1
TTTGTTGAGCACTCAT-1	singlet	2	-2180.558791	-1850.416972	-883.3276991	EMD1
TTTGTTGAGCGTACAG-1	singlet	2	-361.1622871	-289.8558724	-146.0606693	EMD1
TTTGTTGAGTCGCTAT-1	singlet	0	-133.1871476	-477.8499237	-508.9531915	EMD1
TTTGTTGCACCTGCAG-1	singlet	1	-1311.550621	-519.9925182	-1055.40119	EMD1
TTTGTTGCATGTGCCG-1	singlet	0	-294.1691161	-963.7032798	-849.6919357	EMD1
TTTGTTGGTACTGAGG-1	singlet	2	-1309.94145	-1460.603229	-430.1545015	EMD1

TTTGTTGGTCCGGTCA-1	singlet	2	-462.149205	-410.7237779	-165.7770816	EMD1
TTTGTTGTCGCGGACT-1	singlet	1	-135.5253429	-60.80743092	-92.59081835	EMD1
TTTGTTGTCGGTCAGC-1	singlet	2	-539.6059722	-489.355174	-218.4337008	EMD1
TTTGTTGTCTATCCAT-1	singlet	2	-1850.038558	-1559.850783	-655.1912426	EMD1
AAACCCAAGAAACCAT-1	singlet	1	-2259.443215	-698.2943592	-2009.092069	EMD2
AAACCCAAGCGCACAA-1	singlet	1	-4651.061052	-1039.700179	-3291.93491	EMD2
AAACCCAAGGCGTTGA-1	singlet	1	-1768.774204	-551.9327019	-1402.177611	EMD2
AAACCCAAGTTAACGA-1	singlet	1	-1256.353639	-347.4024508	-1092.305674	EMD2
AAACCCACAAGTACCT-1	singlet	1	-755.461089	-250.3172893	-538.3341637	EMD2
AAACCCACAATTGAAG-1	singlet	1	-1131.159264	-394.6919156	-973.1144364	EMD2
AAACCCACACTAACCA-1	unassigned	1/0	-12.73146726	-7.724337002	-18.96410258	EMD2
AAACCCACAGCTTCGG-1	singlet	1	-2376.882911	-753.4416012	-2096.393844	EMD2
AAACCCAGTTAGGGAC-1	singlet	1	-1720.888087	-546.2080757	-1303.653612	EMD2
AAACGAAAGAATAACC-1	unassigned	2/0	-10.39386045	-12.28135074	-6.880533618	EMD2
AAACGAAAGCATTGAA-1	singlet	1	-1763.270768	-546.0342928	-1471.754254	EMD2
AAACGAAAGTGCCGAA-1	singlet	1	-391.5895737	-174.8933022	-376.3605056	EMD2
AAACGAAAGTTGCTGT-1	doublet	0/2	-1471.781365	-2557.043025	-1774.915033	EMD2
AAACGAACACATTGTG-1	singlet	1	-1395.636451	-530.0507836	-1118.512203	EMD2
AAACGAACACGACGTC-1	singlet	1	-1138.888294	-477.393655	-1015.67134	EMD2
AAACGAAGTCTATGAC-1	singlet	2	-3258.858795	-2935.965626	-1132.448652	EMD2
AAACGAAGTTGCTAGT-1	singlet	0	-276.2410802	-882.092762	-701.9760437	EMD2
AAACGAATCGGTGAAG-1	singlet	2	-2111.091347	-1694.740906	-659.2285183	EMD2
AAACGAATCGTGGGTC-1	singlet	1	-221.4892568	-96.14455973	-196.8289732	EMD2
AAACGAATCGTGTGGC-1	singlet	0	-630.7219256	-2267.604011	-2100.85335	EMD2
AAACGAATCTCTTAAC-1	unassigned	1/0	-316.7808492	-121.4001652	-262.4766988	EMD2
AAACGCTAGTGACACG-1	singlet	1	-1676.15475	-503.8197352	-1311.860458	EMD2
AAACGCTAGTGGTTCT-1	singlet	0	-316.1062145	-953.2555574	-903.2878229	EMD2
AAACGCTCAAATGAAC-1	singlet	0	-434.5088572	-1383.28023	-1303.199551	EMD2
AAACGCTCACATACGT-1	singlet	2	-1942.46928	-1742.448123	-756.1492151	EMD2
AAACGCTGTAACCCGC-1	singlet	1	-1305.594851	-401.6392784	-1077.007925	EMD2
AAACGCTGTCGAATGG-1	singlet	1	-542.2607553	-193.2388333	-477.1862758	EMD2
AAACGCTGTTATCTGG-1	singlet	1	-1069.886139	-310.5920128	-722.0773536	EMD2
AAACGCTTCATGAGTC-1	singlet	1	-3490.044726	-1071.932705	-2531.500957	EMD2
AAACGCTTCCGCTGTT-1	singlet	1	-2187.89265	-713.9598142	-1920.737297	EMD2
AAACGCTTCCGTAATG-1	singlet	1	-15271.75	-3628.004384	-11770.35109	EMD2
AAACGCTTCCTCACGT-1	singlet	2	-463.4404048	-491.6193689	-179.1297664	EMD2
AAAGAACAGAAATCCA-1	unassigned	1/0	-6.191610668	-4.629059653	-13.4825909	EMD2
AAAGAACAGGTATTGA-1	singlet	0	-584.2076219	-1756.836845	-1734.660646	EMD2
AAAGAACAGTATGTAG-1	singlet	1	-962.2635936	-362.7650146	-765.5748287	EMD2
AAAGAACAGTTAGTGA-1	singlet	0	-963.5094463	-2713.590091	-2548.918079	EMD2
AAAGAACCAAGCAGGT-1	singlet	1	-2534.535627	-762.2359423	-1993.111008	EMD2
AAAGAACGTAACCCTA-1	singlet	2	-5992.353952	-4653.968003	-2100.550333	EMD2
AAAGAACGTCGTATTG-1	singlet	1	-1382.977692	-441.7592173	-1180.031615	EMD2
AAAGAACGTCTTTCTA-1	singlet	1	-817.6236137	-284.9311974	-617.1252548	EMD2
AAAGAACGTTTCACAG-1	singlet	0	-516.7308671	-1549.818383	-1318.033194	EMD2
AAAGAACTCCGCGATG-1	singlet	2	-489.027997	-454.5530893	-192.6574641	EMD2
AAAGAACTCGAAGAAT-1	singlet	1	-1435.96193	-504.2769134	-1261.525075	EMD2
AAAGAACTCGGACTTA-1	singlet	1	-1163.89114	-355.8459938	-880.4712966	EMD2
AAAGAACTCGGATAAA-1	singlet	2	-2512.572023	-2046.534301	-838.1497501	EMD2
AAAGAACTCTATGTGG-1	singlet	1	-965.6740106	-327.7303549	-811.9800758	EMD2
AAAGAACTCTCATTGT-1	singlet	1	-2594.999044	-853.7220832	-2174.788013	EMD2
AAAGAACTCTTAATCC-1	singlet	1	-402.6287119	-197.7431205	-393.7042089	EMD2
AAAGGATAGGATTTGA-1	singlet	1	-984.562998	-338.4444504	-781.8422087	EMD2
AAAGGATAGTGAGCCA-1	singlet	1	-977.2890836	-397.1848782	-829.1713108	EMD2
AAAGGATAGTGGCGAT-1	singlet	0	-1108.857289	-3998.756546	-3733.972004	EMD2
AAAGGATAGTTGCTGT-1	singlet	1	-4338.163969	-1375.236721	-3577.785927	EMD2
AAAGGATCAGCACAAG-1	singlet	0	-869.0664225	-2281.709085	-2230.59953	EMD2
AAAGGATGTAATTGGA-1	singlet	0	-351.0276683	-1014.739742	-850.5216117	EMD2
AAAGGATGTAGACGTG-1	singlet	0	-895.2731682	-2587.904647	-2653.688556	EMD2
AAAGGATGTCCTCATC-1	singlet	0	-1903.870208	-6621.02004	-6322.396025	EMD2
AAAGGATGTGAATTGA-1	singlet	0	-2150.250319	-7789.563866	-7281.932992	EMD2
AAAGGATGTGACAGGT-1	singlet	0	-209.7952643	-528.1477247	-587.7781996	EMD2
AAAGGATTCGACCAAT-1	singlet	1	-1963.765969	-563.8336249	-1647.486283	EMD2
AAAGGATTCTAAACGC-1	singlet	1	-995.4166473	-379.1604524	-874.2218533	EMD2
AAAGGATTCTTAGGAC-1	singlet	0	-288.2799489	-927.5754768	-860.5773029	EMD2
AAAGGGCAGCGGTAAC-1	singlet	0	-1008.929847	-2971.081118	-2882.994302	EMD2
AAAGGGCCAAAGTATG-1	singlet	1	-489.2655264	-180.9726062	-431.3320112	EMD2
AAAGGGCCACACGGAA-1	singlet	1	-597.0701528	-208.8107815	-523.4699778	EMD2
AAAGGGCCAGACGGAT-1	singlet	1	-482.7996517	-204.0804786	-367.5514607	EMD2
AAAGGGCGTAGATCGG-1	singlet	1	-86.62840066	-42.16489887	-77.82946403	EMD2
AAAGGGCTCGTTCATT-1	singlet	1	-2346.261881	-751.6855912	-1957.242875	EMD2
AAAGGTAAGACCCGCT-1	singlet	1	-2233.109087	-735.5531488	-1788.736809	EMD2
AAAGGTAAGTAACCTC-1	singlet	1	-3499.871937	-1145.160878	-2817.228469	EMD2
AAAGGTAAGTCATACC-1	singlet	0	-232.9572514	-719.9095841	-702.8403489	EMD2
AAAGGTACAGAGGCAT-1	singlet	1	-1246.804069	-430.0803639	-1039.149815	EMD2
AAAGGTACAGTTGCGC-1	singlet	1	-2169.95687	-705.2379827	-1605.013794	EMD2
AAAGGTACATCGCCTT-1	singlet	2	-3010.331877	-2758.67175	-926.6747188	EMD2
AAAGGTAGTACAAAGT-1	singlet	1	-301.5654923	-148.2183431	-275.6799754	EMD2
AAAGGTAGTATGGTAA-1	singlet	0	-310.1034613	-1231.500036	-1029.784437	EMD2
AAAGGTAGTCGTGATT-1	singlet	2	-1407.776618	-1211.174574	-477.8381413	EMD2
AAAGGTAGTTATGACC-1	singlet	2	-215.746618	-246.9393502	-103.5311127	EMD2
AAAGGTATCCAACTAG-1	singlet	1	-101.4630657	-25.97240482	-69.33788136	EMD2
AAAGGTATCTCCTGTG-1	singlet	2	-206.958107	-136.3192981	-86.7483127	EMD2
AAAGGTATCTCTCTAA-1	singlet	1	-1944.735126	-674.6607714	-1762.071186	EMD2
AAAGTCCCAAGTATAG-1	singlet	1	-2101.079315	-787.0361804	-1858.674571	EMD2
AAAGTCCCACAGCCAC-1	singlet	1	-238.5266815	-74.3875015	-161.710706	EMD2
AAAGTCCCATTGCTGA-1	singlet	0	-1300.595961	-5344.611142	-5364.060827	EMD2
AAAGTCCGTAACTTCG-1	singlet	1	-32.5959461	-18.4602148	-28.55858311	EMD2
AAAGTCCGTTCGGTCG-1	singlet	2	-2931.98462	-2428.049305	-895.5555554	EMD2
AAAGTCCTCACCGGGT-1	singlet	0	-478.5556684	-1234.972251	-1143.964774	EMD2
AAAGTCCTCCAGCACG-1	singlet	1	-469.2153433	-177.5959847	-354.2900613	EMD2
AAAGTCCTCCTTGACC-1	singlet	1	-608.2101887	-266.281823	-514.3111146	EMD2
AAAGTGAAGATTGTGA-1	singlet	0	-659.0872085	-1845.337971	-1872.392959	EMD2
AAAGTGAAGCCTGGAA-1	singlet	1	-265.5245216	-119.1911063	-246.7520114	EMD2
AAAGTGACACCGCTAG-1	singlet	0	-1549.807715	-7045.538322	-6540.8771	EMD2
AAAGTGACAGCCGTCA-1	singlet	0	-284.8030194	-781.5234143	-725.9759417	EMD2
AAAGTGACATGTGACT-1	singlet	1	-383.2589419	-125.981332	-315.058955	EMD2
AAAGTGAGTCTCGGGT-1	singlet	1	-2275.696685	-580.4299105	-1850.217524	EMD2
AAAGTGATCCATACTT-1	singlet	1	-2639.641058	-886.1193804	-2035.25806	EMD2
AAAGTGATCGCAGTGC-1	singlet	1	-1134.282238	-332.3650692	-1043.129112	EMD2
AAAGTGATCGCTCATC-1	unassigned	1/0	-7.216270791	-4.075531518	-7.855150733	EMD2
AAAGTGATCTTAGCTT-1	singlet	1	-1243.001854	-408.8318129	-987.1935706	EMD2
AAATGGAAGGCCTGCT-1	singlet	1	-4067.947545	-1314.682173	-2984.921186	EMD2
AAATGGACAACACTAC-1	singlet	0	-472.4038954	-1260.509144	-1199.836285	EMD2
AAATGGACACTCCTTG-1	singlet	1	-2251.974769	-686.6349177	-1798.615532	EMD2
AAATGGACAGCGCTTG-1	singlet	1	-2336.110326	-681.0281226	-1912.046469	EMD2
AAATGGAGTACGAGTG-1	singlet	1	-1561.282953	-531.0467483	-1255.56821	EMD2
AAATGGAGTGATTGGG-1	singlet	1	-1118.997672	-363.3852079	-918.8611448	EMD2
AAATGGAGTTCGGCGT-1	singlet	2	-790.7093611	-757.6678462	-298.0740841	EMD2
AAATGGATCAAGGCTT-1	singlet	2	-457.3234279	-539.0095122	-163.9685001	EMD2

AAATGGATCCCGGTAG-1	singlet	1	-842.1278596	-271.4783533	-702.7775765	EMD2
AACAAAGAGAGAGCAA-1	singlet	2	-2995.201683	-3560.095082	-926.6828893	EMD2
AACAAAGAGGTTTGAA-1	singlet	0	-583.1639511	-2104.333119	-1982.385127	EMD2
AACAAAGAGTTTAGGA-1	singlet	2	-1170.605312	-1132.153561	-382.3088336	EMD2
AACAAAGCAGCTGTAT-1	singlet	2	-600.8497431	-517.3271171	-228.7592703	EMD2
AACAAAGGTACAGGTG-1	singlet	2	-4855.821321	-4444.763359	-2022.496182	EMD2
AACAAAGGTATTCTCT-1	singlet	0	-1220.294037	-3397.138437	-3404.648822	EMD2
AACAAAGGTGTCTTCC-1	singlet	2	-2481.582133	-2217.805135	-785.1045225	EMD2
AACAAAGGTTCTTAGG-1	singlet	2	-954.9524193	-918.8697894	-354.9234828	EMD2
AACAAAGTCCGCACTT-1	singlet	1	-1957.926432	-585.442268	-1509.458428	EMD2
AACAAAGTCGGACTTA-1	singlet	2	-3270.393039	-2839.988203	-1065.077638	EMD2
AACAAAGTCTCGGGAC-1	singlet	1	-567.2262025	-226.2691877	-529.1897574	EMD2
AACAAAGTCTGTCCGT-1	singlet	1	-3888.17565	-1070.121425	-3113.031287	EMD2
AACAACCAGACCTGGA-1	singlet	2	-858.1923568	-800.2378388	-329.3224833	EMD2
AACAACCAGAGCTGCA-1	singlet	1	-385.1039861	-136.5703458	-331.7963239	EMD2
AACAACCAGATTGCGG-1	singlet	2	-183.5442323	-137.1252483	-67.92036281	EMD2
AACAACCAGGAGTCTG-1	singlet	1	-301.9513487	-99.86035823	-222.0041695	EMD2
AACAACCAGGTAAGAG-1	unassigned	1/0	-10.4206433	-7.250980321	-11.2493699	EMD2
AACAACCCAGCTGTCG-1	singlet	1	-1099.749982	-348.8679304	-900.6328615	EMD2
AACAACCCATCTTCGC-1	singlet	1	-544.6684615	-222.4667593	-474.7566631	EMD2
AACAACCGTATTGGCT-1	singlet	2	-674.1998218	-600.8345491	-221.179965	EMD2
AACAACCGTCGGAACA-1	singlet	1	-2810.000899	-803.7286949	-2218.092993	EMD2
AACAACCGTGGGCTCT-1	singlet	1	-3704.897166	-1117.964435	-2905.379772	EMD2
AACAACCGTTGGAGGT-1	singlet	1	-1262.149637	-373.5726025	-966.7034136	EMD2
AACAACCTCAAAGGAT-1	singlet	1	-2877.94484	-847.4002449	-2247.925069	EMD2
AACAACCTCACACCGG-1	singlet	2	-1207.48603	-1079.228602	-473.5133365	EMD2
AACAACCTCTGCGGAC-1	singlet	0	-1173.010342	-3436.936702	-3261.794034	EMD2
AACAAGAAGAGCCATG-1	singlet	1	-1343.972421	-387.1298805	-1263.20406	EMD2
AACAAGACAACAGTGG-1	unassigned	45323	-21.86162455	-21.51975984	-12.37352951	EMD2
AACAAGACATCCGATA-1	singlet	1	-60.88692845	-25.24969658	-48.56792878	EMD2
AACAAGAGTTAGGGAC-1	singlet	1	-3113.426284	-1090.65999	-2754.135102	EMD2
AACAAGAGTTTGGGAG-1	singlet	1	-348.1266594	-134.3970052	-262.9804092	EMD2
AACACACAGAATCGTA-1	singlet	0	-48.377834	-195.6858175	-218.163884	EMD2
AACACACAGCCATTTG-1	singlet	0	-192.1636558	-451.3395611	-438.8812602	EMD2
AACACACAGCGACTGA-1	doublet	45293	-3146.236571	-1426.758077	-1924.626868	EMD2
AACACACAGTAACCTC-1	singlet	0	-25.70728913	-60.73866037	-66.52162554	EMD2
AACACACGTCAGTCTA-1	singlet	1	-2224.671724	-621.0882042	-1625.370809	EMD2
AACACACTCAATGCAC-1	singlet	2	-79.19081542	-65.63260876	-37.58528246	EMD2
AACACACTCATTCGTT-1	singlet	1	-706.1535284	-237.2068433	-584.448132	EMD2
AACACACTCCATCTCG-1	singlet	1	-1012.026715	-341.5367086	-882.1983932	EMD2
AACAGGGAGCATATGA-1	singlet	2	-533.2076727	-469.9197906	-203.0268088	EMD2
AACAGGGAGCGGACAT-1	singlet	1	-1361.675443	-410.0981021	-971.9941833	EMD2
AACAGGGCAATGCAGG-1	singlet	1	-363.2721251	-138.5285746	-284.9800882	EMD2
AACAGGGCACGGTGCT-1	singlet	1	-588.0527141	-214.3392647	-476.7541015	EMD2
AACAGGGGTAACATCC-1	singlet	0	-181.7718479	-441.7552952	-402.7248223	EMD2
AACAGGGTCCTCTAAT-1	singlet	1	-1711.552308	-445.061165	-1366.294184	EMD2
AACCAACAGCGAATGC-1	singlet	0	-698.7094898	-1824.904337	-1721.554887	EMD2
AACCAACAGCTGAGCA-1	singlet	0	-2753.514361	-8999.49541	-9216.842139	EMD2
AACCAACAGGCTAGCA-1	singlet	2	-2107.913903	-2103.294692	-703.4020836	EMD2
AACCAACAGGTTCATC-1	singlet	2	-2521.815342	-2268.280392	-956.3220469	EMD2
AACCAACAGTGCTAGG-1	singlet	0	-1073.881235	-3402.629913	-3109.49376	EMD2
AACCAACCACATTGTG-1	singlet	2	-3127.922846	-2558.828876	-942.2529539	EMD2
AACCAACCAGAGGTAC-1	singlet	2	-110.7214494	-67.03159965	-40.32296876	EMD2
AACCAACCAGCAATTC-1	singlet	2	-2469.155136	-2064.274298	-850.7985761	EMD2
AACCAACCATGGCTGC-1	singlet	0	-35.46834848	-75.35245173	-66.73630946	EMD2
AACCAACGTATCCCAA-1	singlet	2	-360.7053773	-296.311574	-116.7425057	EMD2
AACCAACGTTGAATCC-1	singlet	0	-312.8581879	-924.1412933	-805.4062839	EMD2
AACCACAAGACCTTTG-1	unassigned	0	-2.253441491	-2.713157256	-2.835783365	EMD2
AACCACAAGATTGTGA-1	singlet	1	-1874.076409	-673.856127	-1675.437401	EMD2
AACCACAAGCACGGAT-1	singlet	1	-2969.705033	-841.8969922	-2281.434196	EMD2
AACCACAAGGTCCGAA-1	singlet	2	-2884.074571	-2544.411909	-908.6192801	EMD2
AACCACAAGTTGTAAG-1	singlet	1	-1325.493558	-415.7428848	-1231.634628	EMD2
AACCACACAAACGGCA-1	singlet	2	-2038.765284	-1670.645482	-672.6656857	EMD2
AACCACACAGCTTCCT-1	singlet	2	-2896.062315	-2693.696932	-1076.235475	EMD2
AACCACAGTCACCACG-1	singlet	0	-830.0307442	-2568.216587	-2473.680351	EMD2
AACCACAGTGTTCCAA-1	singlet	1	-120.8658141	-51.72222047	-104.7763732	EMD2
AACCACATCATTTGGG-1	doublet	1/0	-1060.697283	-888.3583968	-1241.458845	EMD2
AACCATGAGGCCGCTT-1	singlet	1	-2614.339669	-906.129288	-2161.392712	EMD2
AACCATGAGTGCTAGG-1	singlet	1	-323.8810683	-112.2656747	-255.5750656	EMD2
AACCATGGTGCACATT-1	singlet	2	-2907.7772	-2256.983113	-938.5263523	EMD2
AACCATGGTGCGTTTA-1	singlet	0	-2079.874489	-7474.91084	-7255.82177	EMD2
AACCATGTCGCCGATG-1	singlet	1	-360.6779189	-154.5451539	-298.549154	EMD2
AACCCAAAGCAAATGT-1	singlet	1	-760.518713	-304.4905659	-618.4428902	EMD2
AACCCAAAGGCATCGA-1	singlet	1	-2296.221598	-680.4823182	-2002.714078	EMD2
AACCCAAAGTAGCCAG-1	singlet	2	-2551.116272	-2199.013004	-753.0259491	EMD2
AACCCAAAGTGTGTTC-1	singlet	1	-932.4092402	-345.4238321	-779.1245342	EMD2
AACCCAACAAACCATC-1	singlet	1	-747.8639169	-242.3159515	-520.9962658	EMD2
AACCCAACAAGCCATT-1	singlet	2	-624.0430627	-525.7088812	-248.679858	EMD2
AACCCAACACCTCTAC-1	singlet	1	-568.3365508	-217.9815181	-494.0720254	EMD2
AACCCAACAGAGGTTG-1	singlet	1	-2764.785481	-865.0586711	-2031.578784	EMD2
AACCCAACAGCGGTCT-1	singlet	0	-175.8567315	-682.4022413	-491.5297436	EMD2
AACCCAATCACCATCC-1	singlet	0	-250.4949937	-860.2327807	-740.0034448	EMD2
AACCCAATCACGATAC-1	singlet	0	-461.1791098	-1410.079449	-1425.053333	EMD2
AACCCAATCTGCGGCA-1	singlet	0	-275.5332132	-930.7940824	-828.0145913	EMD2
AACCCAATCTGTGTGA-1	singlet	1	-860.0290968	-264.2869998	-665.5255819	EMD2
AACCTGAAGACCCTTA-1	singlet	2	-976.9739349	-843.3727403	-342.8543888	EMD2
AACCTGAAGAGAGGTA-1	singlet	1	-3843.030657	-1004.28394	-2820.035256	EMD2
AACCTGAAGTAGACCG-1	singlet	1	-1741.986007	-457.3614993	-1333.153158	EMD2
AACCTGACAGACCCGT-1	singlet	1	-88.28935206	-25.02138866	-60.33270808	EMD2
AACCTGAGTCCATACA-1	singlet	1	-1670.633479	-621.1816214	-1320.993315	EMD2
AACCTGAGTGAAGCGT-1	singlet	0	-140.9004568	-361.5124249	-364.4472795	EMD2
AACCTGAGTGCCGTAC-1	singlet	1	-999.6405823	-341.3318537	-784.2736041	EMD2
AACCTGATCAGAGTGG-1	singlet	1	-255.6660589	-83.79891543	-179.5189088	EMD2
AACCTGATCATTCACT-1	singlet	1	-3533.358826	-838.6471815	-2425.600197	EMD2
AACCTGATCCCAAGTA-1	singlet	0	-1127.464719	-3809.95057	-3766.29077	EMD2
AACCTTTAGCCTTCTC-1	singlet	1	-3557.519278	-938.3753401	-2327.818532	EMD2
AACCTTTAGGTACTGG-1	singlet	1	-585.451612	-199.7724773	-426.0980065	EMD2
AACCTTTAGTCAAGCG-1	singlet	1	-4735.504837	-1750.392456	-4116.844623	EMD2
AACCTTTCACACGCCA-1	doublet	0/1	-431.0238986	-617.0834787	-627.3963494	EMD2
AACCTTTGTATGCAAA-1	singlet	1	-3924.646412	-1198.047567	-3177.306258	EMD2
AACCTTTGTTCTCACC-1	singlet	1	-1976.664724	-689.3480885	-1735.792552	EMD2
AACCTTTGTTTGCCGG-1	singlet	1	-278.5461962	-136.3631903	-274.1826982	EMD2
AACCTTTTCGTTGCCT-1	singlet	2	-3854.261357	-3010.348615	-1227.509819	EMD2
AACCTTTTCTCGCTTG-1	singlet	1	-2213.920752	-699.4296056	-1634.321569	EMD2
AACGAAAAGAAGTCTA-1	singlet	1	-382.224186	-132.3453242	-320.5666782	EMD2
AACGAAAAGCGACAGT-1	singlet	1	-3309.358638	-1032.154158	-2668.559962	EMD2
AACGAAACACAGTGTT-1	doublet	0/2	-2082.259553	-3486.333198	-2722.660152	EMD2
AACGAAACAGCGCGTT-1	singlet	1	-602.1213051	-214.553768	-490.30235	EMD2
AACGAAACAGCGTACC-1	singlet	1	-305.735787	-123.4418946	-251.5566547	EMD2
AACGAAAGTACCCACG-1	singlet	1	-1437.113075	-534.6293611	-1302.819493	EMD2

AACGAAAGTATCGTAC-1	singlet	1	-925.4635005	-313.8774402	-675.5577803	EMD2
AACGAAAGTCATCTAG-1	singlet	1	-3033.470587	-1052.002722	-2682.617311	EMD2
AACGAAAGTCCTTGTC-1	singlet	1	-3647.0547	-1268.282349	-3037.984461	EMD2
AACGAAAGTCGTGCCA-1	singlet	1	-468.669113	-146.5282075	-321.083098	EMD2
AACGAAAGTGGAAATT-1	singlet	0	-109.3002954	-222.0364781	-217.2270645	EMD2
AACGAAATCGGTCAGC-1	singlet	0	-127.0407128	-380.8246881	-322.095479	EMD2
AACGGGAAGTGAGGTC-1	singlet	0	-1384.419889	-5411.518534	-5155.005981	EMD2
AACGGGAAGTTAGTAG-1	singlet	2	-3492.608915	-2724.897306	-1115.508612	EMD2
AACGGGACAATCACGT-1	singlet	1	-3380.798715	-1043.312966	-2722.508155	EMD2
AACGGGACACCAGTAT-1	singlet	2	-4520.284647	-3610.195625	-1528.204434	EMD2
AACGGGACACTGATTG-1	singlet	1	-2031.836127	-558.2010343	-1751.240825	EMD2
AACGGGACAGGCATTT-1	singlet	0	-752.0628973	-1627.299197	-1700.970125	EMD2
AACGGGAGTACAGTAA-1	singlet	0	-677.5758842	-2087.550447	-1960.073139	EMD2
AACGGGAGTATCCCAA-1	singlet	2	-3863.025063	-3027.529096	-1139.977481	EMD2
AACGGGAGTTCCTACC-1	singlet	1	-105.6364859	-25.74449179	-80.54573264	EMD2
AACGGGAGTTGGGTAG-1	singlet	1	-138.2735193	-55.25025366	-121.8941115	EMD2
AACGGGATCAGGTAAA-1	singlet	1	-385.7742973	-155.6279294	-335.7275286	EMD2
AACGGGATCGACGACC-1	singlet	0	-1687.183646	-7097.722085	-6423.152508	EMD2
AACGGGATCGCAGATT-1	singlet	0	-238.8795907	-761.4817612	-733.3267753	EMD2
AACGGGATCGTCTACC-1	singlet	1	-476.0758132	-152.4151271	-341.9498044	EMD2
AACGTCAAGCACTGGA-1	singlet	0	-1231.594138	-5985.955789	-5541.128357	EMD2
AACGTCAAGCTACGTT-1	singlet	1	-4415.365193	-1172.872104	-3286.328294	EMD2
AACGTCAAGGACACTG-1	singlet	1	-227.9796496	-101.0328899	-193.4772885	EMD2
AACGTCACAAACCGGA-1	singlet	0	-481.2267606	-1412.672077	-1377.814463	EMD2
AACGTCACAAGATGGC-1	singlet	1	-2650.541839	-680.7125853	-2065.977679	EMD2
AACGTCACACAAATCC-1	singlet	1	-461.2629893	-186.364913	-428.6649558	EMD2
AACGTCACACACGTGC-1	singlet	2	-1250.5945	-1177.886624	-395.1297304	EMD2
AACGTCACATGCCGGT-1	singlet	1	-69.19551271	-34.65426521	-60.22801321	EMD2
AACGTCACATTGAGCT-1	singlet	1	-409.2804178	-168.7276023	-359.3382376	EMD2
AACGTCAGTCCTATAG-1	singlet	0	-504.389289	-1221.535008	-1096.597931	EMD2
AACGTCAGTTGCAAGG-1	singlet	1	-237.3181266	-117.6365554	-232.2446662	EMD2
AACGTCATCACGGACC-1	singlet	1	-872.5342073	-318.1557275	-875.5598022	EMD2
AACGTCATCGAAACAA-1	singlet	1	-2963.520978	-991.9419068	-2249.392802	EMD2
AACGTCATCGAAGAAT-1	singlet	0	-84.08012089	-183.2414174	-167.0075706	EMD2
AACGTCATCGTTGTAG-1	singlet	1	-238.3738176	-95.15502954	-247.6177997	EMD2
AACTTCTAGGACCCAA-1	singlet	1	-1401.383067	-378.8094577	-1053.674704	EMD2
AACTTCTAGGTCATAA-1	singlet	2	-5071.025806	-4382.986929	-1407.332357	EMD2
AACTTCTCAAGGTCTT-1	singlet	2	-1374.634087	-1386.479674	-450.0921948	EMD2
AACTTCTCAATGACCT-1	singlet	2	-2803.06493	-2733.9474	-958.3588499	EMD2
AACTTCTCACGCCACA-1	singlet	1	-373.4191024	-166.5987399	-372.8088858	EMD2
AACTTCTCAGAGGCTA-1	unassigned	1	-3.878658865	-2.932809687	-3.816677391	EMD2
AACTTCTCAGGCATTT-1	singlet	1	-472.2123314	-167.3090732	-388.5974879	EMD2
AACTTCTCAGGGAATC-1	singlet	1	-1180.690368	-446.1011704	-1050.848724	EMD2
AACTTCTGTATCTCTT-1	singlet	2	-3336.178235	-3129.313157	-1029.979364	EMD2
AACTTCTGTGGCTAGA-1	unassigned	1	-10.0126547	-4.316017257	-9.37056804	EMD2
AACTTCTTCATGGATC-1	singlet	1	-247.1580658	-121.848885	-227.1323155	EMD2
AACTTCTTCCACCCTA-1	singlet	0	-303.1283086	-735.9498518	-661.1660501	EMD2
AACTTCTTCCATACAG-1	singlet	2	-1471.613038	-1347.119736	-430.1513325	EMD2
AACTTCTTCTGGCTGG-1	singlet	1	-837.9316415	-260.3878165	-581.6131201	EMD2
AAGAACAAGACCACGA-1	singlet	1	-3934.684455	-1024.621211	-2929.758807	EMD2
AAGAACAAGGGAACAA-1	doublet	45323	-1307.51789	-788.8994602	-691.3951991	EMD2
AAGAACAAGTCGAATA-1	singlet	2	-531.5100113	-497.2799747	-198.1695882	EMD2
AAGAACACAAATAGCA-1	singlet	1	-1789.765952	-636.4252105	-1468.688143	EMD2
AAGAACACAAATGATG-1	singlet	1	-488.3668054	-204.8820299	-385.73801	EMD2
AAGAACACATACAGGG-1	singlet	1	-3759.428921	-1319.393971	-3013.297632	EMD2
AAGAACACATTGTCGA-1	singlet	1	-1415.720975	-569.3484032	-1174.909242	EMD2
AAGAACAGTACGATCT-1	singlet	1	-68.72175535	-34.84268287	-128.5557769	EMD2
AAGAACAGTAGCGTTT-1	singlet	0	-1998.645375	-7908.204512	-7431.422023	EMD2
AAGAACAGTGGTAATA-1	singlet	1	-361.6388045	-150.8388893	-309.5584504	EMD2
AAGAACATCATCTCTA-1	singlet	1	-1001.501102	-371.439149	-913.8287975	EMD2
AAGAACATCGACCAAT-1	singlet	0	-483.6699052	-1648.643984	-1386.67355	EMD2
AAGAACATCTCATGCC-1	singlet	1	-126.4087083	-47.97733676	-113.5033064	EMD2
AAGAACATCTTTCTAG-1	singlet	2	-1099.035885	-1015.695732	-415.5153618	EMD2
AAGACAAAGGCTCAAG-1	singlet	1	-1018.037562	-302.4993886	-830.1581615	EMD2
AAGACAAAGTAAAGCT-1	singlet	2	-359.1098191	-301.9378323	-143.7880806	EMD2
AAGACAAAGTCACGCC-1	singlet	1	-204.5747507	-64.84974507	-172.6468492	EMD2
AAGACAAAGTGCACCC-1	singlet	1	-1816.752983	-590.6865677	-1476.667054	EMD2
AAGACAACACGACGTC-1	singlet	0	-938.2265874	-3620.759484	-3184.51031	EMD2
AAGACAACAGTCAGCC-1	singlet	2	-283.8166355	-236.9950703	-119.2960763	EMD2
AAGACAAGTACGGGAT-1	singlet	1	-514.689326	-212.0054148	-454.7199228	EMD2
AAGACAAGTATTTCTC-1	singlet	1	-379.5320376	-154.1723666	-254.3784365	EMD2
AAGACAAGTTTACGTG-1	singlet	2	-1720.832327	-1531.611583	-540.900177	EMD2
AAGACAAGTTTAGACC-1	singlet	0	-1915.614565	-7177.079446	-6563.518094	EMD2
AAGACAATCATCGCTC-1	singlet	1	-2287.585164	-735.6216326	-1915.951346	EMD2
AAGACAATCCCTCGAT-1	singlet	2	-1210.278584	-1129.991844	-452.8807529	EMD2
AAGACAATCCCTTGTG-1	singlet	2	-3099.415821	-2507.988835	-1061.982775	EMD2
AAGACAATCCTGGTCT-1	singlet	0	-52.83033854	-159.5937632	-136.7393716	EMD2
AAGACTCAGAGGTATT-1	singlet	1	-1866.31306	-592.2690035	-1543.647622	EMD2
AAGACTCCAAGCCATT-1	singlet	2	-388.4471112	-296.8588415	-197.9260418	EMD2
AAGACTCCAAGTGGGT-1	singlet	0	-905.8978352	-2691.298282	-2718.038357	EMD2
AAGACTCCATAGGTTC-1	singlet	0	-502.7389447	-1428.214638	-1274.786163	EMD2
AAGACTCGTCACTCAA-1	singlet	1	-449.2360711	-185.0536758	-426.083341	EMD2
AAGACTCTCATTCTTG-1	singlet	0	-107.476187	-336.0479723	-286.7991157	EMD2
AAGACTCTCCCTGGTT-1	singlet	1	-256.3929638	-108.1896851	-222.9437887	EMD2
AAGACTCTCCGTAATG-1	singlet	2	-2582.699419	-1993.306188	-731.4203537	EMD2
AAGACTCTCGACCTAA-1	singlet	2	-1280.219171	-1019.466947	-419.7650893	EMD2
AAGACTCTCTCTCTAA-1	singlet	2	-3597.132989	-3013.50821	-1269.984886	EMD2
AAGACTCTCTGCGATA-1	singlet	1	-2001.688429	-511.7840821	-1752.094866	EMD2
AAGATAGAGCAAGGAA-1	singlet	1	-1493.42036	-410.6539644	-1039.051814	EMD2
AAGATAGAGCATTGAA-1	singlet	2	-1179.705005	-1050.106804	-373.8286568	EMD2
AAGATAGAGCGGTATG-1	singlet	0	-1007.581424	-3242.620854	-3123.253511	EMD2
AAGATAGAGTAAACAC-1	singlet	0	-1150.639451	-4525.980817	-3676.494099	EMD2
AAGATAGCAAAGGGCT-1	singlet	1	-295.1020911	-125.4821866	-283.7760336	EMD2
AAGATAGCAATTGGTC-1	singlet	2	-2485.120327	-2141.047375	-876.1314646	EMD2
AAGATAGGTATTTCGG-1	singlet	1	-4276.265636	-1455.926884	-3358.09881	EMD2
AAGATAGGTCGCATCG-1	singlet	0	-678.3762414	-1863.335134	-1950.671033	EMD2
AAGATAGTCCCAGGAC-1	singlet	1	-1891.346494	-640.7427899	-1797.328088	EMD2
AAGCATCAGAAACTAC-1	singlet	0	-2374.775658	-8934.396658	-8393.705069	EMD2
AAGCATCAGCGGGTAT-1	singlet	1	-3259.128375	-1183.669276	-2896.337508	EMD2
AAGCATCAGGCACCAA-1	singlet	1	-326.3147248	-130.6775222	-248.0107029	EMD2
AAGCATCAGTATCCTG-1	singlet	2	-4493.797197	-4061.200021	-1514.351494	EMD2
AAGCATCCAACGTTAC-1	singlet	1	-99.11007478	-26.61322568	-59.4316992	EMD2
AAGCATCCAATACGAA-1	singlet	1	-1032.643066	-279.6496096	-808.7579961	EMD2
AAGCATCGTAACGTTC-1	singlet	0	-1191.136566	-3821.751312	-3505.823667	EMD2
AAGCATCGTACGCGTC-1	singlet	1	-185.1039561	-79.50104494	-145.3590032	EMD2
AAGCATCGTCCGACGT-1	singlet	2	-765.9756751	-715.5146747	-255.4277193	EMD2
AAGCATCGTGGACAGT-1	singlet	2	-2427.921444	-2250.556494	-682.0468195	EMD2
AAGCCATAGCGCCCAT-1	singlet	1	-352.4856681	-142.3159853	-322.4793978	EMD2
AAGCCATAGTTCATGC-1	singlet	0	-131.7157778	-374.3190207	-344.1871022	EMD2
AAGCCATCAAAGCACG-1	doublet	1/0	-2276.585963	-1268.47996	-2300.357788	EMD2

AAGCCATCAGTAGAAT-1	singlet	1	-508.7271608	-196.952523	-406.1769359	EMD2
AAGCCATCATAGATCC-1	singlet	1	-348.8532561	-146.9878295	-305.1466653	EMD2
AAGCCATCATTGGCAT-1	singlet	0	-173.0097624	-496.0908713	-485.6816058	EMD2
AAGCCATGTAGGAGGG-1	singlet	1	-760.2226146	-256.9263277	-688.2903684	EMD2
AAGCCATGTCCTGAAT-1	singlet	1	-69.88416674	-35.13226081	-57.0624431	EMD2
AAGCCATGTGAGTTGG-1	singlet	1	-153.0931894	-77.20369307	-138.8053538	EMD2
AAGCCATGTGCACAAG-1	singlet	0	-500.9336862	-1918.444558	-1608.415851	EMD2
AAGCCATGTTGACTGT-1	singlet	1	-708.9607074	-270.1446402	-616.6359744	EMD2
AAGCCATGTTTACTGG-1	singlet	1	-121.5545364	-34.52177192	-103.8238867	EMD2
AAGCCATTCACTAGCA-1	singlet	1	-1465.83505	-432.4439795	-1186.931794	EMD2
AAGCCATTCATCTATC-1	singlet	2	-4884.214186	-4019.901606	-1766.795967	EMD2
AAGCCATTCCGATCTC-1	singlet	1	-2389.381012	-572.8796433	-1906.439515	EMD2
AAGCCATTCGGCACTG-1	singlet	1	-5303.946547	-1557.886865	-4055.864444	EMD2
AAGCCATTCTCACGAA-1	singlet	2	-822.7442662	-737.2961663	-367.5090881	EMD2
AAGCCATTCTGTACAG-1	singlet	1	-2056.789888	-696.7000429	-2125.157841	EMD2
AAGCGAGAGCTCGAAG-1	singlet	1	-170.031392	-59.52767571	-129.5572018	EMD2
AAGCGAGCACATATCG-1	singlet	1	-2182.186914	-786.2521303	-2032.131588	EMD2
AAGCGAGGTTAGAAGT-1	singlet	2	-3322.929227	-2745.470165	-976.3739206	EMD2
AAGCGAGTCAAATGCC-1	singlet	0	-444.8800865	-1527.926243	-1257.868576	EMD2
AAGCGAGTCATGCTAG-1	singlet	0	-168.0940195	-434.1181631	-418.4296307	EMD2
AAGCGAGTCGCTGTTC-1	singlet	0	-377.8033347	-1161.576063	-1133.126939	EMD2
AAGCGAGTCGTTCAGA-1	singlet	1	-208.9730959	-69.88339426	-183.5369474	EMD2
AAGCGAGTCGTTTACT-1	singlet	1	-248.1019859	-99.74566526	-210.5826376	EMD2
AAGCGAGTCTACCACC-1	singlet	1	-1094.206512	-302.1682214	-752.6859918	EMD2
AAGCGAGTCTCCCAAC-1	singlet	1	-141.8804121	-61.16888053	-132.2696381	EMD2
AAGCGTTAGAGACAAG-1	singlet	1	-2100.299339	-568.553083	-1636.860892	EMD2
AAGCGTTAGCAGGGAG-1	singlet	0	-53.21045264	-184.5528956	-138.9762424	EMD2
AAGCGTTCAGCCGTTG-1	singlet	2	-4343.537299	-4240.138889	-1420.193115	EMD2
AAGCGTTCAGTGGGTA-1	singlet	0	-653.6209315	-1731.703316	-1689.090116	EMD2
AAGCGTTGTATCACGT-1	singlet	0	-211.5959365	-642.3088006	-640.1240764	EMD2
AAGCGTTGTATGAAGT-1	singlet	0	-703.5088695	-2207.471108	-1890.285725	EMD2
AAGCGTTTCGCCCAGA-1	singlet	2	-858.7499479	-894.8103236	-313.9389535	EMD2
AAGCGTTTCGTGCATA-1	singlet	1	-2083.476451	-665.5475251	-1608.542303	EMD2
AAGGAATAGACCAAAT-1	singlet	0	-1361.697136	-4744.862752	-4668.458848	EMD2
AAGGAATAGGCCTAGA-1	singlet	1	-1018.898255	-359.3163345	-755.3210118	EMD2
AAGGAATAGGCGTCCT-1	singlet	2	-2937.243693	-2124.793204	-990.4921329	EMD2
AAGGAATAGTTAACAG-1	singlet	0	-730.0082829	-2199.000107	-2188.978078	EMD2
AAGGAATCAAAGGGTC-1	singlet	0	-158.6222936	-676.3398611	-508.4758357	EMD2
AAGGAATCACAAGTTC-1	singlet	1	-2459.779877	-780.6224789	-2272.082422	EMD2
AAGGAATGTAATGATG-1	singlet	1	-688.9419544	-273.2530863	-645.2342782	EMD2
AAGGAATGTAGGATAT-1	singlet	1	-1679.50399	-442.9500065	-1087.028331	EMD2
AAGGAATGTTCGTGCG-1	singlet	1	-2272.434215	-615.0884343	-1762.87171	EMD2
AAGGAATTCCACGGAC-1	singlet	0	-394.2652941	-1184.878164	-1093.431246	EMD2
AAGGAATTCTCTGCCA-1	singlet	1	-512.6222789	-209.9195455	-447.4968914	EMD2
AAGGAATTCTTGCAAG-1	singlet	2	-5711.742799	-4731.991889	-1751.420635	EMD2
AAGGTAACAAATGGCG-1	singlet	1	-2348.01025	-815.9224786	-2151.358372	EMD2
AAGGTAACAAGCGAAC-1	singlet	1	-2069.607574	-704.1505701	-1856.690004	EMD2
AAGGTAACACTCTCGT-1	singlet	1	-2783.816713	-859.0402809	-2065.641169	EMD2
AAGGTAACAGAAGCGT-1	singlet	1	-361.6418181	-168.571237	-349.4344389	EMD2
AAGGTAACAGCTGTCG-1	singlet	0	-1041.74975	-2860.967036	-2627.067794	EMD2
AAGGTAACATATACCG-1	singlet	1	-2248.407165	-731.8848217	-1819.032606	EMD2
AAGGTAACATTGAAGA-1	singlet	1	-2036.345174	-633.4510396	-1715.205778	EMD2
AAGGTAAGTACCGGAA-1	singlet	0	-112.5579599	-239.8854981	-237.6790283	EMD2
AAGGTAAGTCGTCTCT-1	singlet	1	-1706.970977	-550.6676956	-1228.757073	EMD2
AAGGTAAGTGTGCCTG-1	singlet	1	-221.4283919	-85.00020084	-198.2414866	EMD2
AAGGTAATCCATCGTC-1	singlet	1	-175.2336861	-56.43278373	-136.070475	EMD2
AAGGTAATCCTACCGT-1	doublet	1/0	-2165.026863	-1107.185935	-2099.841199	EMD2
AAGGTAATCGGATACT-1	singlet	0	-548.9989428	-1556.880115	-1431.989545	EMD2
AAGTACCAGGTAGCCA-1	singlet	1	-369.4039628	-116.3767299	-271.4806238	EMD2
AAGTACCAGGTTGCCC-1	singlet	2	-1835.939604	-1513.042088	-783.5865593	EMD2
AAGTACCCAAATCCCA-1	singlet	1	-1739.565894	-708.7560941	-1566.51177	EMD2
AAGTACCGTCATGCAT-1	singlet	1	-499.0604473	-159.6109282	-362.3746376	EMD2
AAGTACCTCACCTTAT-1	singlet	0	-845.6563105	-2782.699824	-2351.470204	EMD2
AAGTACCTCATGGCCG-1	singlet	1	-1159.94615	-426.1430641	-944.8606729	EMD2
AAGTACCTCATTGTTC-1	singlet	0	-860.7468778	-2481.888902	-2430.541764	EMD2
AAGTACCTCCGGACTG-1	singlet	1	-3373.623275	-1149.975306	-2602.841984	EMD2
AAGTACCTCCTGGCTT-1	singlet	0	-378.5695021	-1063.509622	-996.1600304	EMD2
AAGTACCTCGCAATTG-1	singlet	2	-321.2374281	-254.3767794	-120.5282576	EMD2
AAGTACCTCGCTCCTA-1	singlet	1	-817.9454278	-317.6868134	-689.7027397	EMD2
AAGTACCTCGCTGACG-1	singlet	1	-332.8625651	-142.9538257	-334.1445921	EMD2
AAGTACCTCTCAATCT-1	singlet	2	-311.7210215	-301.9009617	-123.8815097	EMD2
AAGTACCTCTGATTCT-1	singlet	1	-1184.814249	-412.4430238	-1025.514441	EMD2
AAGTACCTCTTAAGGC-1	singlet	0	-235.4428382	-698.7789425	-577.3019139	EMD2
AAGTCGTAGATTGACA-1	singlet	1	-266.2266196	-113.9842683	-252.4516971	EMD2
AAGTCGTAGCGCCATC-1	singlet	2	-577.6717097	-602.4313999	-254.0800658	EMD2
AAGTCGTAGCTTTCCC-1	singlet	1	-2920.936604	-994.1614338	-2373.647706	EMD2
AAGTCGTAGGAGGTTC-1	singlet	2	-2344.997618	-2413.456544	-785.5728404	EMD2
AAGTCGTAGTTACGGG-1	singlet	1	-4071.314106	-1438.462632	-3190.763456	EMD2
AAGTCGTCACAGCCTG-1	singlet	1	-347.9023759	-137.2414757	-279.0281137	EMD2
AAGTCGTCACTGCGTG-1	singlet	1	-347.1241502	-132.1173613	-278.7633467	EMD2
AAGTCGTGTACGTTCA-1	singlet	1	-524.0785778	-173.6441498	-473.5346213	EMD2
AAGTCGTGTCAGTCGC-1	singlet	1	-549.7693052	-216.9239776	-399.7098253	EMD2
AAGTCGTGTTCAGCTA-1	singlet	0	-960.4224081	-4140.327252	-3650.230147	EMD2
AAGTCGTGTTTCCAAG-1	doublet	0/1	-1219.082077	-1798.030041	-1948.270834	EMD2
AAGTCGTGTTTCCCAC-1	singlet	0	-363.2258775	-1413.262222	-1252.425616	EMD2
AAGTCGTTCACTTGTT-1	singlet	1	-2466.2026	-914.8528917	-2016.423142	EMD2
AAGTCGTTCATGCCAA-1	singlet	2	-1191.493505	-1118.382346	-410.2726102	EMD2
AAGTCGTTCCAATCCC-1	singlet	1	-1840.426203	-576.870751	-1384.618218	EMD2
AAGTCGTTCTACACTT-1	singlet	2	-5800.16409	-4524.11614	-1478.139585	EMD2
AAGTGAAAGCTACGTT-1	singlet	1	-1230.056205	-495.2226783	-1046.614074	EMD2
AAGTGAAAGGGACTGT-1	singlet	1	-444.0023855	-194.4541746	-376.5235172	EMD2
AAGTGAAAGTGCCAGA-1	singlet	1	-1839.694568	-570.1967454	-1636.743763	EMD2
AAGTGAACACCTGCGA-1	singlet	2	-2885.141552	-2339.973508	-1047.07897	EMD2
AAGTGAAGTAACCCGC-1	singlet	1	-2271.230322	-789.7935689	-2005.910178	EMD2
AAGTGAAGTGGATACG-1	singlet	1	-537.3869515	-156.1552294	-436.4856643	EMD2
AAGTGAAGTGTCTTCC-1	singlet	1	-1209.007167	-406.9500199	-1033.340861	EMD2
AAGTGAATCATAAGGA-1	singlet	1	-2218.836788	-703.6059944	-1808.026972	EMD2
AAGTGAATCATGAGTC-1	singlet	1	-539.853901	-200.3495158	-402.8605056	EMD2
AAGTGAATCCAGCTCT-1	singlet	1	-1315.92441	-503.3553846	-1171.212261	EMD2
AAGTGAATCTGTCGCT-1	singlet	1	-1380.047844	-537.6414322	-1312.761743	EMD2
AAGTTCGAGAATTTGG-1	doublet	45323	-2940.671732	-1674.951704	-1601.475006	EMD2
AAGTTCGAGTATTCCG-1	singlet	2	-56.32730396	-48.91863152	-21.83766715	EMD2
AAGTTCGCAGAGTGAC-1	singlet	1	-462.7012172	-169.9252289	-369.2346406	EMD2
AAGTTCGCAGCGGATA-1	singlet	0	-562.3226884	-2050.878581	-1904.649224	EMD2
AAGTTCGCAGCGTTTA-1	singlet	0	-5.055653579	-8.644665191	-16.10622348	EMD2
AAGTTCGGTCTACACA-1	singlet	0	-437.7782015	-1524.313782	-1420.352034	EMD2
AAGTTCGGTCTGCGCA-1	singlet	1	-607.4551962	-177.9493271	-552.3028944	EMD2
AAGTTCGGTGCCTGAC-1	singlet	1	-3021.485999	-888.6163835	-2529.246665	EMD2
AAGTTCGTCACCATCC-1	singlet	1	-685.8998709	-261.1229913	-534.3782393	EMD2
AAGTTCGTCAGCTGAT-1	singlet	0	-180.480485	-484.6374234	-494.1412345	EMD2

AAGTTCGTCTGTGTGA-1	singlet	0	-22.52298248	-102.0181777	-84.04542327	EMD2
AATAGAGAGAGGATGA-1	singlet	2	-1234.851923	-1020.562929	-452.13041	EMD2
AATAGAGAGGCTGTAG-1	singlet	0	-147.2575113	-492.8728125	-417.1089831	EMD2
AATAGAGCAAGACCTT-1	singlet	1	-839.9413375	-335.3673524	-753.9215914	EMD2
AATAGAGCAAGGGTCA-1	singlet	1	-1451.3011	-412.5082826	-1267.825709	EMD2
AATAGAGCACTGTGAT-1	singlet	1	-626.5583774	-171.3712991	-442.4574782	EMD2
AATAGAGCAGACACAG-1	singlet	1	-317.7542006	-125.7342085	-291.7060587	EMD2
AATAGAGCAGGAATAT-1	singlet	1	-561.9824044	-234.8867988	-478.1388238	EMD2
AATAGAGCATATGCGT-1	singlet	1	-403.1412758	-123.3716398	-307.5860714	EMD2
AATAGAGCATGATCTG-1	singlet	0	-867.8373922	-1899.911882	-1938.440011	EMD2
AATAGAGGTAAGTTAG-1	singlet	0	-111.8231874	-229.4064562	-210.0428837	EMD2
AATAGAGGTGAGACCA-1	singlet	0	-66.05991687	-228.449296	-170.9960491	EMD2
AATAGAGGTTTACACG-1	singlet	2	-289.3591899	-234.6296598	-131.2602663	EMD2
AATAGAGTCAAGGCTT-1	singlet	0	-1523.061596	-5894.935936	-6075.035536	EMD2
AATAGAGTCGTTGCCT-1	singlet	1	-369.7033887	-122.3389566	-304.3941418	EMD2
AATCACGAGACAGTCG-1	singlet	1	-2807.090315	-697.625702	-2312.674067	EMD2
AATCACGAGATGTTAG-1	singlet	1	-2856.901223	-930.8602326	-2222.949885	EMD2
AATCACGAGTAAGGGA-1	singlet	1	-406.8920156	-152.6799335	-310.774461	EMD2
AATCACGCAGCGACAA-1	singlet	1	-2513.765267	-753.4545638	-2199.268957	EMD2
AATCACGCATGCGGTC-1	singlet	0	-70.49057531	-170.5850043	-144.8211556	EMD2
AATCACGCATTGCTTT-1	singlet	1	-1035.595775	-385.9952251	-788.6145938	EMD2
AATCACGGTGCAACAG-1	singlet	2	-405.699354	-385.5253522	-164.1979827	EMD2
AATCACGTCGCATTAG-1	singlet	1	-1662.827135	-405.3683085	-1246.55124	EMD2
AATCACGTCGCTGTCT-1	singlet	0	-960.8116413	-3896.384088	-3888.148316	EMD2
AATCGACAGGGATCGT-1	singlet	0	-880.4581344	-2447.222442	-2616.204383	EMD2
AATCGACCAATTGCTG-1	singlet	0	-2027.334011	-5751.18903	-5854.263261	EMD2
AATCGACCACGGCTAC-1	singlet	1	-336.5427062	-125.1262013	-297.8550612	EMD2
AATCGACCATTACTCT-1	singlet	1	-1857.979012	-439.8472357	-1260.052853	EMD2
AATCGACTCGAACGGA-1	singlet	1	-284.9903751	-107.2855267	-230.46518	EMD2
AATCGACTCGCAAGAG-1	singlet	1	-764.0493242	-281.7923516	-590.2219308	EMD2
AATCGACTCTTTGCAT-1	singlet	2	-3173.430295	-2609.880876	-744.8305351	EMD2
AATCGTGAGAGAGAAC-1	singlet	1	-1123.255871	-365.9555079	-960.2759437	EMD2
AATCGTGAGCAATTCC-1	singlet	0	-797.0227533	-2228.468143	-2198.070334	EMD2
AATCGTGAGTCCGCCA-1	singlet	0	-393.3964408	-1524.022836	-1408.118614	EMD2
AATCGTGCACAGAAGC-1	singlet	1	-92.56744379	-34.02356994	-66.84985375	EMD2
AATCGTGCAGTCACGC-1	singlet	1	-33.89064655	-16.94959017	-22.7206726	EMD2
AATCGTGGTATAATGG-1	singlet	2	-1874.05647	-1570.640021	-683.99584	EMD2
AATCGTGGTGTTATCG-1	singlet	1	-915.3413592	-395.2653301	-855.7143613	EMD2
AATCGTGGTTGGAGAC-1	singlet	1	-2875.762689	-955.0505921	-2172.735254	EMD2
AATCGTGTCCGAGGCT-1	singlet	2	-350.7525485	-287.0098884	-128.1643272	EMD2
AATCGTGTCGTGGCTG-1	singlet	1	-1086.843576	-327.529776	-809.0596595	EMD2
AATGAAGAGAAGGTAG-1	singlet	0	-92.89139709	-218.6904115	-231.2806107	EMD2
AATGAAGAGCGTTACT-1	singlet	2	-1178.168955	-900.2034908	-375.0938216	EMD2
AATGAAGAGGCCACCT-1	singlet	2	-2582.388544	-2028.724416	-812.3517395	EMD2
AATGAAGCAAGCTACT-1	singlet	2	-3049.636282	-2438.4552	-1000.750493	EMD2
AATGAAGCACAGTATC-1	singlet	2	-999.9292835	-993.2023999	-362.5389118	EMD2
AATGAAGCACTGCGAC-1	singlet	1	-2151.54381	-770.0346999	-1867.831575	EMD2
AATGAAGCAGCATGCC-1	singlet	1	-855.200154	-297.4663917	-660.7016287	EMD2
AATGAAGGTCGATGCC-1	singlet	2	-185.6156836	-157.268029	-61.25821933	EMD2
AATGAAGGTCGTCAGC-1	singlet	1	-5751.16678	-2688.34231	-5168.223434	EMD2
AATGAAGTCCTACCAC-1	singlet	2	-2127.313148	-1749.393977	-778.5849449	EMD2
AATGAAGTCGAGAAGC-1	singlet	1	-3075.91551	-911.5947834	-2488.008451	EMD2
AATGAAGTCGGTCTGG-1	singlet	1	-299.2345054	-154.3904146	-270.7585571	EMD2
AATGACCCACCTCAGG-1	singlet	1	-449.4710367	-166.7269189	-344.935438	EMD2
AATGACCCACTAACGT-1	singlet	1	-879.4172964	-259.4251371	-733.4995532	EMD2
AATGACCCAGCGTTGC-1	singlet	0	-111.1629427	-341.9002348	-321.3973236	EMD2
AATGACCCAGTCCGTG-1	singlet	1	-1017.085079	-368.8258696	-858.694963	EMD2
AATGACCCAGTCGGAA-1	singlet	0	-189.0635063	-534.2850828	-381.7642967	EMD2
AATGACCCATCCGAGC-1	singlet	1	-261.5599654	-98.96269072	-228.760374	EMD2
AATGACCCATGGGTCC-1	singlet	1	-1495.202728	-491.4010192	-1316.184881	EMD2
AATGACCGTGTAAACA-1	singlet	1	-369.940871	-170.6475869	-334.852055	EMD2
AATGACCTCCACACAA-1	singlet	0	-191.6144527	-492.4074291	-434.8717455	EMD2
AATGACCTCGAAGCAG-1	singlet	0	-180.2494471	-457.2187675	-439.5894275	EMD2
AATGCCAAGCATGGGT-1	singlet	1	-987.3264912	-363.9330848	-777.0562849	EMD2
AATGCCAAGCCGATTT-1	singlet	0	-1078.156507	-3107.838114	-2876.991283	EMD2
AATGCCAAGGAGCTGT-1	singlet	1	-1803.35654	-619.5542903	-1667.923039	EMD2
AATGCCAAGGCCTAGA-1	singlet	2	-278.6291883	-230.3085746	-80.44842722	EMD2
AATGCCAAGTGATTCC-1	singlet	1	-3768.118556	-1254.409226	-3082.546473	EMD2
AATGCCACACGGTGCT-1	doublet	1/0	-1569.912475	-848.6789079	-1416.280812	EMD2
AATGCCACACTTGGGC-1	singlet	1	-209.180871	-89.67930267	-201.1796345	EMD2
AATGCCACAGCCATTA-1	singlet	2	-1458.191152	-1198.159187	-493.2092284	EMD2
AATGCCACAGCGTAGA-1	singlet	0	-1346.49709	-5028.524783	-4661.427503	EMD2
AATGCCACAGCTCCTT-1	singlet	0	-235.4284717	-827.1899043	-687.5345502	EMD2
AATGCCACAGTCAGCC-1	singlet	1	-631.1645884	-255.4923408	-516.728648	EMD2
AATGCCAGTTATCTTC-1	singlet	0	-35.8531327	-74.64979827	-82.54352125	EMD2
AATGCCAGTTCCACGG-1	singlet	0	-173.8239797	-712.4797341	-629.0218229	EMD2
AATGCCATCATCACCC-1	doublet	0/1	-2807.922715	-3711.129082	-4256.600119	EMD2
AATGCCATCATTTCCA-1	singlet	2	-1675.51823	-1518.710404	-571.377952	EMD2
AATGGAAAGGTGCATG-1	singlet	1	-1493.596814	-620.9336938	-1215.610899	EMD2
AATGGAAAGTCACTGT-1	singlet	2	-2051.254528	-1587.100627	-707.0548145	EMD2
AATGGAACACAGCTTA-1	singlet	0	-261.4498245	-807.2471453	-743.9160424	EMD2
AATGGAACAGCGGATA-1	singlet	2	-205.5219783	-264.722653	-119.684743	EMD2
AATGGAAGTATGAGCG-1	singlet	1	-2311.704462	-834.660117	-2141.007208	EMD2
AATGGAAGTCCACACG-1	unassigned	1	-1.939583161	-0.783055264	-2.270689622	EMD2
AATGGAAGTCGAGCAA-1	singlet	1	-3949.603223	-1118.579386	-3501.705433	EMD2
AATGGAATCAAAGAAC-1	singlet	1	-1914.429449	-700.3125834	-1599.396903	EMD2
AATGGAATCAGCTAGT-1	singlet	1	-391.0243013	-118.4817039	-347.0638942	EMD2
AATGGAATCCTTGACC-1	singlet	0	-77.19576756	-192.755016	-207.9482606	EMD2
AATGGAATCGCTTTAT-1	singlet	1	-1530.66219	-563.2455284	-1158.434007	EMD2
AATGGAATCTTGTGCC-1	singlet	1	-485.2554883	-219.3249662	-466.5309014	EMD2
AATGGCTAGAGTTCGG-1	singlet	2	-711.8925294	-722.9254491	-244.4108655	EMD2
AATGGCTAGCCTTTCC-1	singlet	1	-1144.163675	-390.0487365	-945.4100114	EMD2
AATGGCTAGTGCACAG-1	singlet	0	-154.4338638	-396.5254131	-336.1485489	EMD2
AATGGCTCAGAGACTG-1	singlet	0	-962.3828833	-2324.622707	-2398.121822	EMD2
AATGGCTGTATTTCGG-1	singlet	0	-797.0776819	-2459.404099	-2305.889911	EMD2
AATGGCTGTCCGACGT-1	singlet	1	-1406.172945	-484.6561891	-1140.423715	EMD2
AATGGCTGTCTAGGCC-1	doublet	45323	-6554.422915	-4350.606876	-2963.066895	EMD2
AATGGCTGTGGACCAA-1	singlet	2	-2663.47738	-2208.108219	-949.2422161	EMD2
AATGGCTGTGTATTGC-1	singlet	0	-1135.573872	-3487.242272	-3347.962427	EMD2
AATGGCTTCACTGGGC-1	singlet	0	-3672.929643	-13308.83084	-12814.72502	EMD2
AATGGCTTCCACGTCT-1	singlet	0	-1054.350906	-3139.308411	-2925.952144	EMD2
AATGGCTTCCATCAGA-1	singlet	0	-23.23797277	-92.41453836	-84.37017544	EMD2
AATTCCTAGGTCTTTG-1	singlet	1	-1042.42208	-402.829118	-856.8996232	EMD2
AATTCCTCAAGTGGTG-1	singlet	2	-590.6766496	-544.8580606	-229.6100031	EMD2
AATTCCTCACTGCTTC-1	singlet	1	-128.4797495	-47.53677765	-116.8761786	EMD2
AATTCCTCAGGTCCGT-1	singlet	1	-100.905489	-44.58395695	-86.81450139	EMD2
AATTCCTCATGGCCAC-1	singlet	2	-7568.115311	-6023.276316	-1870.644697	EMD2
AATTCCTGTAACCCTA-1	singlet	1	-845.9878772	-345.6929398	-718.1390571	EMD2
AATTCCTGTCCGATCG-1	singlet	1	-324.176974	-138.1912671	-291.4120664	EMD2
AATTCCTGTCTGATAC-1	singlet	1	-4647.886184	-1368.712919	-3459.330461	EMD2

AATTCCTTCGGTAAGG-1	singlet	0	-374.6704625	-1123.102913	-1047.512739	EMD2
AATTCCTTCTATTCGT-1	singlet	0	-1088.791147	-3493.470015	-3137.123676	EMD2
AATTCCTTCTGGCCAG-1	doublet	45293	-5726.597137	-2592.912775	-3784.524273	EMD2
AATTTCCAGAGGTCGT-1	singlet	1	-538.263371	-186.188791	-435.1986273	EMD2
AATTTCCAGCCTCTTC-1	singlet	0	-1648.0162	-5424.74255	-5231.579516	EMD2
AATTTCCCAAGCAATA-1	singlet	1	-355.6086358	-118.8465165	-272.4405257	EMD2
AATTTCCCAATAGGAT-1	singlet	0	-608.4890722	-1642.52656	-1771.682872	EMD2
AATTTCCCACCAGCTG-1	singlet	1	-635.1348015	-224.9262648	-461.9031063	EMD2
AATTTCCCATGGCCAC-1	singlet	1	-2433.668968	-842.1138589	-1886.05315	EMD2
AATTTCCCATTGTAGC-1	singlet	1	-2828.086859	-944.1648045	-2306.819093	EMD2
AATTTCCGTAGACACG-1	singlet	1	-558.6766375	-208.4191138	-451.385531	EMD2
AATTTCCGTTCTAAGC-1	singlet	1	-477.9372436	-165.1880218	-325.1846581	EMD2
AATTTCCGTTGAGTCT-1	singlet	2	-3631.125476	-2849.775278	-1168.83035	EMD2
AATTTCCTCCACAGGC-1	singlet	1	-431.4376395	-150.532742	-315.9133462	EMD2
AATTTCCTCTACGGTA-1	singlet	2	-2041.355551	-1667.081778	-664.4040195	EMD2
ACAAAGAAGATGCTAA-1	singlet	1	-2275.079923	-731.5692181	-1824.457238	EMD2
ACAAAGAAGGTTGCCC-1	singlet	1	-1073.061656	-444.1116027	-894.3195672	EMD2
ACAAAGAAGTAGACAT-1	singlet	1	-330.2157043	-137.062908	-314.8729553	EMD2
ACAAAGAAGTTGTAAG-1	singlet	0	-680.3957064	-2555.038921	-2381.631971	EMD2
ACAAAGACAAACGGCA-1	singlet	0	-915.8436667	-2742.593751	-2622.858483	EMD2
ACAAAGAGTAGTCCTA-1	singlet	1	-795.2543341	-264.7537031	-776.1317344	EMD2
ACAAAGATCCTGTAGA-1	singlet	1	-487.0588279	-182.5105651	-333.08665	EMD2
ACAAAGATCCTTCAGC-1	singlet	2	-952.4658158	-770.7350524	-370.6064665	EMD2
ACAAAGATCGACCATA-1	singlet	1	-1282.510974	-359.8354815	-1018.968735	EMD2
ACAACCAAGATGGCGT-1	singlet	2	-955.7783377	-858.9085507	-334.7398308	EMD2
ACAACCAAGCTCAGAG-1	singlet	1	-4371.1031	-1453.568252	-3831.369332	EMD2
ACAACCAAGGCTTAGG-1	singlet	0	-579.6764349	-1694.085567	-1668.25345	EMD2
ACAACCAAGTTATGGA-1	singlet	2	-3431.629551	-2814.352602	-990.9776312	EMD2
ACAACCACAGCGAGTA-1	singlet	0	-546.3941513	-1668.847134	-1583.753378	EMD2
ACAACCACATACCAGT-1	singlet	1	-99.91885384	-33.86228983	-80.31822984	EMD2
ACAACCAGTAAGATAC-1	singlet	0	-482.4023121	-1844.049742	-1539.759364	EMD2
ACAACCAGTTGACTGT-1	singlet	2	-124.3867413	-87.82430052	-44.73626389	EMD2
ACAACCATCAGAATAG-1	singlet	1	-1107.352838	-394.3889138	-865.7782889	EMD2
ACAAGCTAGAGGATCC-1	singlet	1	-643.6802713	-241.5011023	-574.162348	EMD2
ACAAGCTAGCAAGTCG-1	singlet	1	-1608.222237	-457.566431	-1200.641793	EMD2
ACAAGCTAGGATATAC-1	singlet	1	-2695.047259	-978.5041709	-2152.824183	EMD2
ACAAGCTAGGCTCTAT-1	singlet	1	-1564.608848	-554.1508617	-1347.566342	EMD2
ACAAGCTCAAGCTGTT-1	singlet	1	-170.084209	-59.28784272	-157.2828637	EMD2
ACAAGCTCAATCGCCG-1	singlet	1	-827.4901862	-333.6495296	-798.6754196	EMD2
ACAAGCTGTACTCAAC-1	doublet	1/0	-3311.565416	-2194.253203	-3272.952057	EMD2
ACAAGCTGTCGTGATT-1	singlet	2	-1488.267857	-1305.117752	-416.611672	EMD2
ACAAGCTGTGAACGGT-1	singlet	1	-679.4337988	-269.8042546	-544.2224097	EMD2
ACAAGCTGTGAGATCG-1	singlet	1	-668.8498722	-254.1378811	-486.1498735	EMD2
ACAAGCTGTTAGGCCC-1	singlet	1	-689.60412	-308.2418972	-641.4919163	EMD2
ACAAGCTGTTCCTAAG-1	singlet	1	-1505.172853	-515.8492701	-1237.939859	EMD2
ACAAGCTTCACGGTCG-1	singlet	1	-2675.210818	-1051.750609	-2135.157535	EMD2
ACAAGCTTCGAGTTGT-1	singlet	1	-778.8682709	-277.9124448	-643.131964	EMD2
ACAAGCTTCGGTTAGT-1	singlet	1	-384.1439203	-172.0435054	-290.4780475	EMD2
ACAAGCTTCGTTTACT-1	singlet	1	-1072.6578	-339.3572937	-908.7829524	EMD2
ACAAGCTTCTAACGCA-1	singlet	1	-720.6885572	-253.2091138	-581.2875934	EMD2
ACAAGCTTCTTTGGAG-1	doublet	45293	-2278.343971	-1115.572628	-1637.498382	EMD2
ACACAGTAGCAACTCT-1	singlet	0	-1382.61093	-3910.226961	-3731.566914	EMD2
ACACAGTAGGCTCAAG-1	doublet	1/0	-5154.542561	-3893.519487	-6233.634376	EMD2
ACACAGTAGGGAGTGG-1	singlet	1	-2306.042043	-711.9264513	-1984.175049	EMD2
ACACAGTAGTCGCTAT-1	doublet	45293	-5871.651047	-3363.850315	-3882.103518	EMD2
ACACAGTCAACCACAT-1	doublet	0/1	-5231.719973	-7660.416435	-9182.165387	EMD2
ACACAGTCAATCGCAT-1	singlet	1	-311.7182425	-127.0458906	-297.7681392	EMD2
ACACAGTCATGACTGT-1	singlet	1	-392.947086	-154.5029163	-313.8894073	EMD2
ACACAGTCATGGCCAC-1	singlet	0	-1267.109811	-5211.959523	-5216.236964	EMD2
ACACAGTGTAATTGGA-1	singlet	1	-1731.291784	-657.5662506	-1566.057569	EMD2
ACACAGTGTCCAGAAG-1	singlet	1	-485.8286759	-162.5214648	-367.4092264	EMD2
ACACAGTGTTAGGGAC-1	doublet	0/2	-3360.114033	-6807.386264	-5462.858091	EMD2
ACACAGTTCCAACTAG-1	singlet	1	-1329.542405	-465.7057031	-1173.093719	EMD2
ACACAGTTCGACGATT-1	singlet	1	-379.0770877	-130.7937653	-306.5170449	EMD2
ACACAGTTCTGGAAGG-1	singlet	2	-968.4752735	-950.2961844	-328.6551316	EMD2
ACACCAAAGCTCACTA-1	singlet	2	-5650.663553	-4412.132451	-1800.36357	EMD2
ACACCAACAACCGCTG-1	singlet	1	-74.40618923	-31.68142983	-67.24243679	EMD2
ACACCAACATCGATCA-1	singlet	2	-1133.647241	-1138.240041	-446.1797864	EMD2
ACACCAACATGAATAG-1	singlet	1	-441.1013154	-185.6640509	-445.5546877	EMD2
ACACCAACATGGAACG-1	singlet	0	-326.5121604	-818.5939244	-797.4401729	EMD2
ACACCAAGTACGTGTT-1	singlet	2	-1965.749597	-1731.444716	-735.2816448	EMD2
ACACCAAGTCACTACA-1	singlet	0	-981.8313049	-5224.78736	-4053.620698	EMD2
ACACCAAGTCCTATAG-1	doublet	45293	-273.1509147	-121.9438407	-214.873762	EMD2
ACACCAAGTCCTGTCT-1	singlet	2	-2103.219604	-1848.91197	-785.9137455	EMD2
ACACCAAGTCGAATTC-1	singlet	1	-418.7740631	-150.4416477	-308.9775227	EMD2
ACACCAATCCATTGCC-1	singlet	1	-425.7618193	-160.3025017	-358.0284528	EMD2
ACACCAATCGAAGTGG-1	singlet	1	-306.2399047	-109.6762845	-275.1419516	EMD2
ACACCAATCTGAATGC-1	singlet	1	-1776.910422	-594.89396	-1571.415257	EMD2
ACACGCGAGAGAGGTA-1	singlet	2	-1548.011587	-1176.350693	-486.2984736	EMD2
ACACGCGAGATGAAGG-1	singlet	0	-733.6169479	-2847.690388	-2732.69672	EMD2
ACACGCGAGCCGTCGT-1	singlet	1	-243.3042457	-85.18140297	-201.5820201	EMD2
ACACGCGGTACCACGC-1	singlet	1	-1404.782795	-505.0514498	-1275.754184	EMD2
ACACGCGGTGATGAAT-1	singlet	1	-1653.699435	-451.8469901	-1377.820735	EMD2
ACACGCGGTGCAACAG-1	singlet	0	-701.7304439	-2835.991628	-2679.623726	EMD2
ACACGCGTCGTTGCCT-1	singlet	2	-492.5835227	-460.8391177	-184.5114016	EMD2
ACACGCGTCTCCACTG-1	singlet	1	-1144.362773	-437.771063	-1037.776982	EMD2
ACACTGAAGTGGAATT-1	singlet	1	-360.2028417	-122.2107659	-269.6835639	EMD2
ACACTGACAGTGACCC-1	singlet	2	-2904.94795	-2230.625529	-903.66147	EMD2
ACACTGACATAGGCGA-1	singlet	0	-653.7604462	-2530.950467	-2003.994913	EMD2
ACACTGAGTACTGCGC-1	singlet	2	-1199.402953	-1002.008869	-442.2089629	EMD2
ACACTGAGTCTCAGAT-1	singlet	1	-353.9107353	-127.1532647	-295.7148335	EMD2
ACACTGAGTGGCTGAA-1	singlet	1	-295.6201802	-106.4748834	-253.4350185	EMD2
ACACTGAGTGGTTCTA-1	singlet	1	-3006.21737	-961.2410061	-2391.293709	EMD2
ACACTGATCACTTCTA-1	singlet	0	-342.29405	-1048.445151	-992.7977581	EMD2
ACAGAAAAGATTCGCT-1	doublet	45323	-957.9855775	-742.802547	-663.5673312	EMD2
ACAGAAAAGTAGTCAA-1	singlet	1	-3492.023975	-868.5459277	-2588.678856	EMD2
ACAGAAACAATCTGCA-1	singlet	2	-1006.276012	-756.8934552	-376.2408886	EMD2
ACAGAAACACGACCTG-1	singlet	1	-2575.310324	-798.338211	-2223.572069	EMD2
ACAGAAACAGCTGTGC-1	singlet	0	-649.4852483	-2163.796591	-2008.406765	EMD2
ACAGAAACATCTATCT-1	singlet	1	-2572.862634	-889.4152466	-2094.233357	EMD2
ACAGAAACATGACGAG-1	singlet	0	-1352.949304	-4382.163337	-4088.06445	EMD2
ACAGAAAGTTAGCTAC-1	singlet	1	-449.9621883	-164.415962	-388.5128292	EMD2
ACAGAAATCAGCCCAG-1	singlet	1	-2991.736061	-889.052459	-2662.114423	EMD2
ACAGAAATCCATACTT-1	singlet	2	-624.2943227	-606.0829028	-261.3202075	EMD2
ACAGCCGAGAAGCGGG-1	singlet	0	-844.1055884	-2299.550559	-2206.933953	EMD2
ACAGCCGAGCCTAGGA-1	singlet	1	-705.3435579	-236.7894862	-497.9468947	EMD2
ACAGCCGCAGACATCT-1	singlet	1	-360.2867387	-129.4265935	-234.9276441	EMD2
ACAGCCGCAGCGCGTT-1	singlet	0	-417.1747945	-1187.672691	-1176.433216	EMD2
ACAGCCGCATAACAGA-1	singlet	2	-1598.100696	-1548.36589	-535.289474	EMD2
ACAGCCGCATAGCACT-1	singlet	1	-1097.61603	-332.7188907	-907.9710593	EMD2

ACAGCCGGTACAGTAA-1	singlet	2	-3370.775501	-2541.212241	-1345.563253	EMD2
ACAGCCGGTACAGTCT-1	singlet	2	-8385.722541	-6949.905208	-2874.982761	EMD2
ACAGCCGGTAGATTGA-1	singlet	0	-244.6495351	-674.453108	-641.3909305	EMD2
ACAGCCGGTCCGCAGT-1	singlet	1	-2442.108458	-888.5848507	-1870.126301	EMD2
ACAGCCGGTCGCTGCA-1	singlet	1	-444.224633	-187.643663	-439.903925	EMD2
ACAGCCGGTGGCGCTT-1	singlet	2	-524.3012688	-476.7519432	-153.8926139	EMD2
ACAGCCGGTTAGGCTT-1	singlet	0	-366.3927702	-1327.49804	-1145.291525	EMD2
ACAGCCGTCCATCGTC-1	singlet	0	-191.300426	-507.9846357	-400.3924005	EMD2
ACAGCCGTCCTCTCGA-1	singlet	0	-490.826461	-1371.076042	-1350.146294	EMD2
ACAGCCGTCCTGTACC-1	singlet	0	-146.1303552	-364.1380646	-358.6685133	EMD2
ACAGCCGTCTAGTCAG-1	singlet	1	-459.7980702	-172.7759455	-368.8692828	EMD2
ACAGCCGTCTCGCTTG-1	singlet	0	-335.5389087	-861.0093967	-861.2547815	EMD2
ACAGCCGTCTGGCCTT-1	singlet	2	-477.9407629	-543.3176108	-216.5307004	EMD2
ACAGGGAAGATTAGTG-1	singlet	1	-2307.791828	-677.1857046	-1803.214889	EMD2
ACAGGGAAGGCATGGT-1	singlet	1	-1517.026423	-436.8710013	-1391.148927	EMD2
ACAGGGACACCAACAT-1	singlet	0	-1065.81766	-3717.16512	-3667.913078	EMD2
ACAGGGAGTGACACGA-1	singlet	2	-209.8623467	-213.1945017	-66.58438333	EMD2
ACAGGGATCAAACTGC-1	singlet	1	-1952.303024	-579.0610588	-1854.9139	EMD2
ACAGGGATCTCACCCA-1	singlet	0	-898.9266483	-2424.483155	-2335.579029	EMD2
ACAGGGATCTGCGATA-1	singlet	1	-428.796358	-168.1335209	-309.793683	EMD2
ACATCCCAGGATCATA-1	singlet	1	-1672.794501	-493.3876423	-1356.748855	EMD2
ACATCCCAGTAGCATA-1	singlet	1	-1345.012556	-495.348898	-1141.347649	EMD2
ACATCCCAGTCGAAGC-1	singlet	1	-872.3391992	-305.1936778	-644.8864477	EMD2
ACATCCCCAGAGAATT-1	doublet	0/2	-2550.087504	-4912.401006	-3635.169554	EMD2
ACATCCCGTACGAAAT-1	singlet	1	-3368.609662	-874.7300319	-2583.686421	EMD2
ACATCCCGTCAATCTG-1	singlet	2	-1404.483275	-1354.956525	-543.4316404	EMD2
ACATCCCGTTCGAACT-1	singlet	1	-2027.200502	-561.9004819	-1632.64495	EMD2
ACATCCCTCACCCATC-1	singlet	1	-3835.849516	-1252.990705	-3345.401625	EMD2
ACATCCCTCCCGAACG-1	singlet	1	-6040.314304	-1984.519818	-5055.316651	EMD2
ACATCCCTCTTCCGTG-1	singlet	1	-1668.301264	-569.4429287	-1406.527711	EMD2
ACATCGAAGATCCGAG-1	singlet	1	-3562.726272	-1042.769113	-2588.719489	EMD2
ACATCGAAGCCACAAG-1	singlet	1	-381.9787708	-153.6762631	-340.9134066	EMD2
ACATCGACACATAGCT-1	singlet	2	-581.430767	-469.1000518	-247.8868482	EMD2
ACATCGAGTCAGACTT-1	singlet	0	-1176.449589	-3273.840255	-3175.533762	EMD2
ACATCGAGTGGACAGT-1	singlet	2	-1261.10265	-1246.156841	-424.0174734	EMD2
ACATCGAGTTAGAGTA-1	singlet	1	-216.8181214	-84.49462855	-150.2496677	EMD2
ACATCGATCCATTTGT-1	singlet	2	-361.3133545	-301.6718938	-100.7778512	EMD2
ACATCGATCCTACGAA-1	singlet	0	-170.9268661	-594.4028677	-442.5101484	EMD2
ACATCGATCTACTGAG-1	singlet	1	-326.9438666	-139.2350932	-256.5016464	EMD2
ACATCGATCTGACAGT-1	singlet	1	-356.8361701	-155.6868801	-358.0941643	EMD2
ACATCGATCTGCTAGA-1	singlet	2	-4074.534827	-3287.37835	-1081.650609	EMD2
ACATCGATCTGTTGGA-1	singlet	1	-304.5541594	-152.0930915	-279.142734	EMD2
ACATGCAAGCCGTTGC-1	unassigned	45293	-48.02538732	-28.35331967	-35.27269784	EMD2
ACATGCACAACCGGAA-1	singlet	1	-316.4817619	-129.2277065	-246.0289537	EMD2
ACATGCACACAACATC-1	singlet	1	-5261.169617	-1612.696285	-3882.732672	EMD2
ACATGCACACATAGCT-1	singlet	1	-1032.548691	-307.2249597	-833.3121068	EMD2
ACATGCACACGTCGTG-1	singlet	1	-1531.477743	-455.7873212	-1204.888219	EMD2
ACATGCACACTACAGT-1	singlet	2	-5929.385157	-5425.961244	-1445.497794	EMD2
ACATGCACAGCGACCT-1	singlet	1	-1204.034529	-481.310944	-985.8236673	EMD2
ACATGCACATTGGGAG-1	singlet	2	-245.2450792	-186.2785757	-98.54189198	EMD2
ACATGCAGTATTCTCT-1	singlet	1	-2367.319573	-679.4475354	-2009.132012	EMD2
ACATGCAGTGACCGAA-1	singlet	2	-28.70345256	-20.47266844	-10.67609832	EMD2
ACATGCAGTTCTATCT-1	singlet	2	-399.3372706	-404.5337977	-156.3755978	EMD2
ACATGCAGTTGCTCGG-1	singlet	1	-354.6312746	-128.458825	-294.2734648	EMD2
ACATGCATCACGGGAA-1	singlet	2	-4495.932779	-3337.031081	-1085.241645	EMD2
ACATGCATCGAAGCCC-1	singlet	1	-322.7418309	-110.137345	-324.8029067	EMD2
ACATGCATCGAGATAA-1	doublet	1/0	-2493.881564	-1932.337085	-2774.403843	EMD2
ACATTTCAGGGAGAAT-1	singlet	1	-307.7141926	-97.79780053	-211.2848635	EMD2
ACATTTCAGTCTGGAG-1	singlet	0	-614.4461442	-2144.339344	-1946.869043	EMD2
ACATTTCAGTGGCAGT-1	unassigned	1/0	-18.95976859	-16.16802211	-11.94682109	EMD2
ACATTTCGTACAGTCT-1	singlet	2	-594.4729747	-535.6625211	-267.0534964	EMD2
ACATTTCGTATGGGAC-1	singlet	2	-123.1277743	-97.98663823	-47.38779576	EMD2
ACATTTCTCAAGCTGT-1	singlet	0	-2198.232986	-7420.201119	-6707.727576	EMD2
ACATTTCTCCGGTAGC-1	singlet	2	-3957.645136	-3308.343206	-1261.772204	EMD2
ACCAAACAGCAAATCA-1	singlet	1	-312.2937461	-131.5701112	-256.1424537	EMD2
ACCAAACGTCATCCCT-1	singlet	1	-316.3919133	-126.9251186	-306.5893704	EMD2
ACCAAACGTCCACGCA-1	singlet	2	-42.27184272	-37.26446529	-19.82596247	EMD2
ACCAAACTCATCCTAT-1	singlet	2	-1018.899183	-942.720753	-382.6665344	EMD2
ACCAACAAGGAAGTCC-1	singlet	1	-1777.032275	-564.8204967	-1450.398622	EMD2
ACCAACAAGGCATGGT-1	singlet	1	-1086.096202	-355.678908	-840.7072373	EMD2
ACCAACACAATGCTCA-1	doublet	45323	-5427.319287	-3643.201328	-3328.722366	EMD2
ACCAACACACAGACGA-1	singlet	1	-1144.264839	-388.2871474	-835.1705724	EMD2
ACCAACACACAGGATG-1	singlet	1	-7264.929132	-2484.974764	-5884.710511	EMD2
ACCAACACATGGGAAC-1	singlet	2	-3103.566261	-2588.946156	-1086.853968	EMD2
ACCAACAGTCGATTTG-1	singlet	1	-2207.942758	-506.3988282	-1430.345382	EMD2
ACCAACAGTGAATAAC-1	singlet	0	-558.4070727	-1664.786562	-1505.578837	EMD2
ACCAACAGTGCTGCAC-1	singlet	0	-911.4380635	-2510.190781	-2389.21777	EMD2
ACCAACAGTTTGTTGG-1	singlet	1	-271.5618176	-109.8741137	-220.5276523	EMD2
ACCAACATCTTACGTT-1	singlet	1	-592.0905958	-210.4131138	-485.9463584	EMD2
ACCACAAAGAGAGAAC-1	singlet	1	-291.5366907	-107.9484229	-217.9791321	EMD2
ACCACAAAGAGTCGAC-1	singlet	0	-537.6762787	-1708.414758	-1642.654538	EMD2
ACCACAAAGATGGGCT-1	singlet	2	-3943.473314	-3225.901526	-1257.876937	EMD2
ACCACAAAGGGCAACT-1	singlet	0	-148.787616	-383.6882878	-386.5159151	EMD2
ACCACAACAGTTGGTT-1	singlet	0	-774.331103	-2703.696503	-2235.458729	EMD2
ACCACAACATAGAAAC-1	singlet	1	-1892.206955	-704.600015	-1534.651961	EMD2
ACCACAACATGTTTGG-1	singlet	0	-523.0576634	-1662.331938	-1478.918295	EMD2
ACCACAATCCCTTGGT-1	doublet	1/0	-2728.235595	-2163.781727	-3110.441903	EMD2
ACCACAATCTAGCAAC-1	singlet	1	-4041.341601	-1010.191309	-3234.439027	EMD2
ACCACAATCTTGCAAG-1	singlet	0	-2693.425221	-11098.0071	-11140.09887	EMD2
ACCATTTAGCGGTAAC-1	doublet	1/0	-3816.045122	-2207.150243	-3664.688295	EMD2
ACCATTTCAAGAGAGA-1	singlet	2	-3125.005837	-2901.672933	-1114.736418	EMD2
ACCATTTCACTACACA-1	singlet	0	-1177.835013	-3626.537701	-3304.44631	EMD2
ACCATTTGTCAACCTA-1	singlet	1	-677.5936036	-246.097875	-532.5867855	EMD2
ACCATTTTCACCTCGT-1	singlet	0	-569.8540838	-1591.600291	-1489.360057	EMD2
ACCATTTTCCCTGTTG-1	singlet	1	-3088.498446	-1043.552654	-2359.571771	EMD2
ACCATTTTCCGATGCG-1	singlet	1	-1373.525256	-628.185404	-1292.20756	EMD2
ACCATTTTCCGCGATG-1	singlet	2	-3011.734395	-2226.017999	-949.8351426	EMD2
ACCCAAAAGAGGCGTT-1	singlet	1	-394.7818851	-163.7168672	-304.1705547	EMD2
ACCCAAACAAAGGCTG-1	singlet	1	-1281.303606	-444.4226611	-968.9631999	EMD2
ACCCAAACAAGTTCCA-1	singlet	0	-890.4256906	-2501.354814	-2375.14412	EMD2
ACCCAAACACGAGGTA-1	singlet	1	-2480.567065	-470.3327823	-1518.64496	EMD2
ACCCAAACATTGCCGG-1	doublet	0/1	-308.7408022	-317.3625338	-331.1262503	EMD2
ACCCAAAGTATCGTAC-1	singlet	2	-7527.249144	-6374.47468	-2273.638957	EMD2
ACCCAAAGTCGGTAAG-1	singlet	0	-3.189107879	-6.91593463	-8.088754842	EMD2
ACCCAAAGTTTGAACC-1	singlet	1	-351.9275159	-165.0223347	-290.6849835	EMD2
ACCCAAATCCGGTTCT-1	singlet	1	-350.1910734	-131.6316937	-262.9446116	EMD2
ACCCAAATCGACATCA-1	singlet	1	-374.053887	-123.2054797	-315.8463083	EMD2
ACCCAAATCGTGTTCC-1	singlet	2	-1833.400653	-1442.610488	-573.2700603	EMD2
ACCCTCAAGAGTCGAC-1	singlet	1	-158.037544	-62.38558282	-128.0380748	EMD2
ACCCTCAAGCATCAAA-1	singlet	0	-255.0256645	-720.8635141	-612.9916698	EMD2

ACCCTCAAGTAAACAC-1	singlet	1	-61.27009089	-25.66806739	-48.44115771	EMD2
ACCCTCAAGTATAGAC-1	singlet	1	-554.2992441	-255.6590329	-515.3694756	EMD2
ACCCTCAAGTTGTCAC-1	singlet	1	-3675.593013	-1209.169776	-3315.596037	EMD2
ACCCTCACACCCTTGT-1	singlet	0	-2214.533811	-7364.851843	-7067.097586	EMD2
ACCCTCACAGCTATAC-1	singlet	0	-965.2406326	-3355.926098	-2826.397803	EMD2
ACCCTCACATACTGAC-1	singlet	2	-4962.587308	-3882.280494	-1590.383256	EMD2
ACCCTCAGTACCGGAA-1	singlet	2	-5706.247197	-4200.124009	-1524.258083	EMD2
ACCCTCAGTACTCCGG-1	singlet	1	-365.8082459	-170.0933571	-333.3359429	EMD2
ACCCTCAGTGAGTTGG-1	doublet	0/1	-695.80055	-855.1142912	-949.2461053	EMD2
ACCCTCAGTGTTGATC-1	singlet	2	-1826.888289	-1831.063561	-665.0160852	EMD2
ACCCTCAGTTCTCAGA-1	singlet	1	-904.8660022	-340.0864143	-781.3723842	EMD2
ACCCTCAGTTGCGGCT-1	singlet	1	-389.3696737	-154.8482179	-273.2770387	EMD2
ACCCTCATCAATCTTC-1	singlet	2	-3757.644304	-2780.895691	-1417.243592	EMD2
ACCCTCATCGTGTGGC-1	singlet	2	-1411.971403	-1416.447028	-553.6767159	EMD2
ACCCTCATCTAACACG-1	singlet	0	-677.5724488	-2096.359259	-1904.872438	EMD2
ACCCTCATCTATTTCG-1	singlet	0	-741.442647	-1992.942092	-2025.245553	EMD2
ACCCTTGAGGTCCAGA-1	singlet	1	-1090.994328	-372.8083829	-941.6667367	EMD2
ACCCTTGCATAGAAAC-1	doublet	45293	-4021.611716	-1820.179584	-2641.688428	EMD2
ACCCTTGGTACGGCAA-1	singlet	1	-329.9122169	-112.4733093	-232.8841571	EMD2
ACCCTTGGTCTTCGAA-1	singlet	0	-137.4841553	-483.0667592	-312.4968676	EMD2
ACCCTTGGTTACCCAA-1	singlet	1	-435.17796	-172.7454074	-330.5855983	EMD2
ACCCTTGTCACCTGGG-1	singlet	1	-102.1496623	-46.53168647	-125.1817655	EMD2
ACCCTTGTCAGTGGGA-1	singlet	1	-169.2017026	-74.43852991	-137.8113175	EMD2
ACCCTTGTCCCAGTGG-1	singlet	1	-567.0395683	-198.2239533	-466.7386674	EMD2
ACCCTTGTCCTAGCGG-1	singlet	1	-615.749244	-192.0762162	-442.4571414	EMD2
ACCCTTGTCGCGTCGA-1	singlet	1	-669.4153478	-228.6798483	-532.6108966	EMD2
ACCCTTGTCTCTGCTG-1	unassigned	1	-2.432432791	-0.93960428	-1.379348374	EMD2
ACCGTTCAGACATCCT-1	singlet	0	-124.0167634	-399.9613707	-328.5697801	EMD2
ACCGTTCAGAGAGGGC-1	singlet	1	-2782.076861	-921.2498149	-2262.943665	EMD2
ACCGTTCAGCCGCACT-1	singlet	1	-1199.671229	-388.4145188	-996.6030602	EMD2
ACCGTTCAGCTCCGAC-1	singlet	1	-280.5767928	-64.98964524	-185.832144	EMD2
ACCGTTCAGCTGCCTG-1	singlet	2	-3680.092559	-3150.124553	-1205.030434	EMD2
ACCGTTCAGGGAACAA-1	singlet	0	-1206.751168	-3568.486826	-3204.007108	EMD2
ACCGTTCCACGACAGA-1	singlet	0	-428.1262394	-1551.407593	-1334.874997	EMD2
ACCGTTCGTAACACCT-1	singlet	1	-351.704841	-159.3055982	-337.5470369	EMD2
ACCGTTCGTACTGCGC-1	singlet	1	-910.3033834	-312.3862601	-791.0154908	EMD2
ACCGTTCGTCCAGCAC-1	singlet	1	-3858.234915	-1005.671931	-3215.182181	EMD2
ACCGTTCGTCGTTATG-1	singlet	1	-273.40395	-107.5786953	-234.9664384	EMD2
ACCGTTCTCAACCCGG-1	singlet	0	-284.1827755	-706.1402115	-640.7544126	EMD2
ACCGTTCTCCTGGGAC-1	singlet	0	-46.97011186	-180.5394498	-148.8993278	EMD2
ACCGTTCTCGACGACC-1	singlet	0	-2450.609593	-8849.040216	-8531.400384	EMD2
ACCTACCAGACTTCGT-1	singlet	2	-349.2129761	-345.4043793	-144.2246961	EMD2
ACCTACCAGCTATCTG-1	singlet	1	-2366.984386	-697.953029	-2102.5407	EMD2
ACCTACCAGGGTACGT-1	singlet	0	-1210.201904	-3535.219537	-3321.487752	EMD2
ACCTACCAGGTGCTGA-1	singlet	1	-565.5792973	-170.315838	-440.5398771	EMD2
ACCTACCCAGCTGAAG-1	singlet	0	-712.5343478	-2375.678322	-2169.273516	EMD2
ACCTACCGTATACAGA-1	singlet	1	-322.6163368	-115.9676338	-267.780668	EMD2
ACCTACCGTATACGGG-1	singlet	0	-913.6249319	-1969.075629	-2036.180562	EMD2
ACCTACCGTGTTAAAG-1	doublet	0/1	-1406.055127	-1515.318534	-1840.503862	EMD2
ACCTACCGTTGATGTC-1	singlet	0	-618.0471352	-1887.720879	-1703.372324	EMD2
ACCTACCTCATCGGGC-1	singlet	1	-385.6082984	-128.0250671	-254.8623185	EMD2
ACCTACCTCTCGACGG-1	singlet	2	-1399.115123	-1638.407781	-538.2442104	EMD2
ACCTACCTCTCGCTTG-1	singlet	0	-805.4240041	-2408.642284	-2314.719732	EMD2
ACCTACCTCTGAGGCC-1	singlet	0	-1791.807972	-5369.612901	-5133.403741	EMD2
ACCTACCTCTGTGCGG-1	singlet	1	-446.6365864	-171.927663	-358.481247	EMD2
ACCTGAAAGATAACAC-1	singlet	1	-3266.375179	-1125.648617	-2439.956752	EMD2
ACCTGAAAGCGTTAGG-1	singlet	1	-1217.743498	-407.9870704	-1036.499002	EMD2
ACCTGAAAGCTGGCTC-1	singlet	1	-265.2402191	-54.44674628	-179.926438	EMD2
ACCTGAACAACCGGAA-1	unassigned	1	-7.023821254	-3.079264469	-3.793359435	EMD2
ACCTGAACACCTATCC-1	singlet	1	-1797.945719	-502.1794802	-1278.993083	EMD2
ACCTGAAGTACCGTGC-1	singlet	1	-1078.601478	-383.2686025	-943.8710261	EMD2
ACCTGAAGTTGTCAGT-1	singlet	0	-178.6102666	-530.0367088	-526.3457186	EMD2
ACCTGAATCACCTCGT-1	singlet	1	-1166.840831	-374.551402	-976.4180936	EMD2
ACCTGAATCAGAGCAG-1	singlet	1	-2353.4349	-574.2411554	-2079.055297	EMD2
ACCTGAATCCTACCAC-1	singlet	2	-398.7393315	-271.9323075	-86.59050436	EMD2
ACCTGAATCGTGCTCT-1	singlet	2	-956.3254555	-718.4379241	-316.2349898	EMD2
ACCTGAATCTAGATCG-1	singlet	1	-2495.029651	-582.1962723	-1887.053914	EMD2
ACCTGAATCTGTGTGA-1	singlet	2	-5421.575197	-4340.97351	-1437.348864	EMD2
ACCTGTCAGACCAAAT-1	singlet	0	-184.6450414	-562.5203067	-579.6749374	EMD2
ACCTGTCAGCGTCTGC-1	singlet	1	-1222.156414	-433.1274295	-944.6518402	EMD2
ACCTGTCCAACGAGGT-1	singlet	1	-6316.782866	-1937.091855	-4890.483517	EMD2
ACCTGTCCAGACACCC-1	singlet	2	-1435.143975	-1565.766285	-497.1463027	EMD2
ACCTGTCCATACCACA-1	singlet	1	-798.0369816	-241.5211361	-577.1733174	EMD2
ACCTGTCCATCGTCCT-1	singlet	2	-434.1229822	-443.833323	-153.9491144	EMD2
ACCTGTCGTACATTGC-1	singlet	0	-3.431550736	-12.81764387	-21.71611719	EMD2
ACCTGTCGTATCCCAA-1	doublet	0/1	-9.327739546	-10.25350362	-15.54947933	EMD2
ACCTGTCGTCGATTTG-1	singlet	0	-127.6656908	-377.9789662	-378.1377088	EMD2
ACCTGTCGTGGTACAG-1	singlet	0	-110.8620624	-372.0630023	-346.9267821	EMD2
ACCTGTCGTTAAGACA-1	singlet	0	-770.2416725	-2105.3946	-2139.99576	EMD2
ACCTGTCTCACCCTTG-1	singlet	1	-1992.943904	-716.3189709	-1839.988963	EMD2
ACCTGTCTCACTGTCC-1	singlet	1	-111.6012649	-35.47308995	-71.15022781	EMD2
ACCTGTCTCCGAGTGC-1	singlet	0	-1303.722732	-4637.860339	-4603.907628	EMD2
ACCTGTCTCTCATTTG-1	singlet	0	-1005.405041	-3249.568989	-3246.964994	EMD2
ACCTGTCTCTCTCAAT-1	doublet	45323	-5203.441324	-2678.910156	-2659.476126	EMD2
ACGATCAAGACGATAT-1	singlet	0	-262.2535433	-695.249734	-709.2154453	EMD2
ACGATCAAGCCACAAG-1	singlet	1	-48.17722202	-21.05620094	-44.70688551	EMD2
ACGATCAAGGGTATAT-1	singlet	0	-206.561265	-661.8526797	-592.4913996	EMD2
ACGATCAAGGTCCAGA-1	singlet	1	-458.4760719	-166.5190828	-372.9840565	EMD2
ACGATCAAGTAGGATT-1	singlet	2	-788.462722	-737.9295893	-266.5696594	EMD2
ACGATCAAGTTGGACG-1	singlet	1	-1038.694351	-343.2804582	-823.4398204	EMD2
ACGATCACAATCTCGA-1	singlet	2	-2601.342475	-2094.221641	-837.067738	EMD2
ACGATCACAATGAACA-1	singlet	1	-1176.601577	-448.0230796	-956.9061035	EMD2
ACGATCACACAAGCTT-1	singlet	0	-68.87385498	-174.7620365	-164.9511244	EMD2
ACGATCAGTAATTGGA-1	singlet	1	-2229.280378	-789.0799057	-1912.790183	EMD2
ACGATCAGTATTTCTC-1	singlet	0	-1275.689083	-4881.932747	-4769.909129	EMD2
ACGATCAGTCGCAACC-1	singlet	2	-55.01886563	-40.54866712	-22.20221052	EMD2
ACGATCAGTCTTTATC-1	singlet	1	-4857.393674	-1249.942545	-3643.4051	EMD2
ACGATCAGTGAGTTGG-1	singlet	1	-84.45823274	-33.71849648	-71.61580059	EMD2
ACGATCAGTTCGAGCC-1	singlet	1	-214.6944561	-69.11144185	-182.8584587	EMD2
ACGATGTCAATTGTGC-1	singlet	1	-283.2971886	-159.6293719	-254.8496651	EMD2
ACGATGTCACTCACTC-1	singlet	1	-1533.78402	-546.0344019	-1277.61748	EMD2
ACGATGTGTCGTGATT-1	singlet	2	-3670.140165	-3042.109292	-1219.455256	EMD2
ACGATGTGTTAAAGTG-1	singlet	1	-1362.614637	-365.6178248	-1033.858476	EMD2
ACGATGTTCACCCTGT-1	singlet	2	-220.8722455	-192.4526082	-66.68849943	EMD2
ACGATGTTCATTACGG-1	singlet	1	-341.6125366	-147.4894661	-273.8692228	EMD2
ACGATGTTCCAGCCTT-1	singlet	1	-589.8136308	-229.1609308	-478.2597243	EMD2
ACGATGTTCGAAGCAG-1	singlet	1	-2022.429198	-658.4537497	-1701.581578	EMD2
ACGATGTTCTAGGCCG-1	singlet	1	-1321.92904	-399.0615201	-1132.625782	EMD2
ACGATGTTCTGAGAGG-1	singlet	1	-374.6491636	-147.1590582	-329.9720553	EMD2
ACGATGTTCTGGGATT-1	singlet	1	-1960.055002	-626.7549275	-1560.229548	EMD2

ACGCACGAGACAAGCC-1	singlet	2	-2086.673014	-2050.52246	-725.7637335	EMD2
ACGCACGCAAATGATG-1	singlet	1	-446.1473634	-160.5469176	-377.9147723	EMD2
ACGCACGCACGCACCA-1	singlet	1	-1018.96225	-275.4285867	-673.8562863	EMD2
ACGCACGCAGACGATG-1	singlet	2	-2244.796255	-1711.027224	-779.6351022	EMD2
ACGCACGCAGGACAGT-1	singlet	1	-140.3528737	-54.57205673	-106.6664752	EMD2
ACGCACGCATGGGTTT-1	singlet	1	-75.25270505	-21.99631712	-58.46794479	EMD2
ACGCACGGTTAAGGGC-1	singlet	1	-2511.005909	-736.9282424	-2082.317102	EMD2
ACGCACGGTTAGAAAC-1	singlet	1	-475.4655057	-161.5056366	-349.9559635	EMD2
ACGCACGTCAGAGCGA-1	singlet	1	-265.0061883	-84.33157756	-173.4834486	EMD2
ACGGAAGAGACATGCG-1	singlet	1	-219.3870935	-91.70122111	-186.0308009	EMD2
ACGGAAGAGCCTGACC-1	singlet	2	-96.86874751	-88.92885751	-27.04018469	EMD2
ACGGAAGAGGGAGATA-1	singlet	1	-218.9065134	-84.68729376	-183.3574586	EMD2
ACGGAAGAGTTGAAGT-1	singlet	0	-949.9277865	-3294.642971	-3266.76351	EMD2
ACGGAAGCAACTCGTA-1	singlet	0	-2169.080789	-6987.401194	-6656.197796	EMD2
ACGGAAGGTCAGGAGT-1	singlet	2	-1223.761626	-943.5334942	-343.4141682	EMD2
ACGGAAGGTCGACGCT-1	singlet	0	-63.64721247	-301.535391	-240.2870512	EMD2
ACGGAAGGTTCACGAT-1	singlet	1	-1408.672508	-500.9042164	-1181.523089	EMD2
ACGGAAGGTTCCGCGA-1	unassigned	0	-2.536675003	-3.76763939	-2.670211404	EMD2
ACGGAAGGTTGCATTG-1	singlet	1	-516.9867562	-216.59424	-391.1947982	EMD2
ACGGAAGGTTGTCTAG-1	singlet	2	-3114.126072	-2384.986449	-987.9982029	EMD2
ACGGAAGTCACGACTA-1	singlet	2	-903.2147956	-957.4639019	-308.9053663	EMD2
ACGGAAGTCATGTCTT-1	singlet	1	-3346.410131	-912.4184637	-2635.661641	EMD2
ACGGAAGTCCATCGTC-1	singlet	1	-227.6824824	-81.29967504	-158.1210687	EMD2
ACGGAAGTCCCTATTA-1	singlet	2	-1299.015663	-1378.696658	-452.8577881	EMD2
ACGGAAGTCTGGGCAC-1	singlet	0	-1166.773579	-4045.273395	-3777.468895	EMD2
ACGGGTCAGAGCATAT-1	doublet	0/1	-3983.364796	-5129.696022	-6164.820662	EMD2
ACGGGTCAGGTCATTC-1	singlet	0	-192.3813892	-547.9797249	-485.4680307	EMD2
ACGGGTCAGTAGACCG-1	singlet	1	-1130.114647	-423.1481772	-1018.761997	EMD2
ACGGGTCCAAATGGAT-1	singlet	1	-1836.71137	-599.3114961	-1413.445755	EMD2
ACGGGTCCACAAGCTT-1	singlet	1	-374.5531018	-151.7415174	-347.8124509	EMD2
ACGGGTCCACAATGTC-1	singlet	1	-492.882864	-215.5514102	-418.2802461	EMD2
ACGGGTCCACACCTAA-1	singlet	0	-179.9118831	-448.1931829	-411.5187149	EMD2
ACGGGTCCACAGTCAT-1	singlet	0	-115.3291357	-343.1157388	-281.3103067	EMD2
ACGGGTCCAGGTTCCG-1	singlet	2	-7282.006228	-5962.581005	-2306.237454	EMD2
ACGGGTCCATAGATGA-1	singlet	1	-528.6733097	-228.0166743	-456.2016618	EMD2
ACGGGTCGTCACCGAC-1	singlet	2	-1072.147433	-1204.80627	-438.8591912	EMD2
ACGGGTCGTCGTGGTC-1	singlet	0	-1392.689656	-4921.516591	-4999.137772	EMD2
ACGGGTCGTCTTTCAT-1	singlet	2	-3897.458062	-3196.745713	-1259.200425	EMD2
ACGGGTCGTGTCCGTG-1	singlet	0	-259.8872341	-952.573926	-860.9714482	EMD2
ACGGGTCTCCTGATAG-1	singlet	1	-2095.474579	-530.3401071	-1731.186535	EMD2
ACGGGTCTCTGGGTCG-1	singlet	1	-296.3605793	-129.2463062	-272.1482487	EMD2
ACGGGTCTCTTGGTGA-1	singlet	1	-1017.169136	-373.814867	-905.525903	EMD2
ACGGTCGAGACTACCT-1	singlet	1	-405.6930257	-158.5051797	-318.4825732	EMD2
ACGGTCGAGATTACCC-1	singlet	1	-4027.165526	-1278.930282	-3354.312847	EMD2
ACGGTCGAGGAAAGGT-1	singlet	0	-1753.54822	-6614.697271	-5952.125293	EMD2
ACGGTCGAGGAGGCAG-1	unassigned	45323	-15.13518197	-5.767320539	-5.06798282	EMD2
ACGGTCGAGGTAGTAT-1	singlet	0	-196.445714	-531.5217769	-443.0268692	EMD2
ACGGTCGAGGTCACCC-1	singlet	2	-5963.41438	-5048.75654	-1909.475964	EMD2
ACGGTCGAGTCGAATA-1	singlet	1	-353.8904592	-124.6546568	-297.7052209	EMD2
ACGGTCGGTAGCGCTC-1	singlet	2	-1035.827862	-881.5761808	-383.3817993	EMD2
ACGGTCGGTCAGATTC-1	singlet	0	-17.66584099	-67.0813318	-57.31126743	EMD2
ACGGTCGGTGACAACG-1	singlet	0	-1488.876575	-4825.692654	-4328.930991	EMD2
ACGGTCGGTGGAAATT-1	singlet	1	-2068.746247	-691.8685372	-1933.853845	EMD2
ACGGTCGGTTGGGCCT-1	singlet	1	-522.41653	-161.1213496	-345.6224462	EMD2
ACGGTCGTCCACGTGG-1	singlet	1	-332.6520685	-140.7876263	-262.9921623	EMD2
ACGGTCGTCGCAAGAG-1	singlet	2	-3669.550628	-2919.126263	-796.3494908	EMD2
ACGGTTAAGAATTGCA-1	doublet	0/2	-1974.408753	-4415.251831	-3732.136862	EMD2
ACGGTTAAGAGGTTAT-1	singlet	0	-1065.054806	-3346.074567	-3085.871249	EMD2
ACGGTTAAGCAACAAT-1	singlet	0	-280.0301315	-824.4867588	-669.4519699	EMD2
ACGGTTAAGCATAGGC-1	singlet	1	-249.7593181	-99.21703067	-198.6214887	EMD2
ACGGTTAAGGCGTCCT-1	singlet	1	-868.6557706	-301.0354309	-718.4326222	EMD2
ACGGTTAAGTAGGTTA-1	singlet	2	-2328.109348	-2130.895592	-912.6949662	EMD2
ACGGTTACATTAAGCC-1	singlet	2	-324.3781371	-398.0472541	-154.2529185	EMD2
ACGGTTAGTAGTACGG-1	singlet	1	-743.5840581	-226.9086961	-586.7675096	EMD2
ACGGTTAGTATGCTTG-1	doublet	45293	-262.5483654	-146.7820917	-205.252299	EMD2
ACGGTTAGTCGGCACT-1	singlet	1	-2121.364374	-718.8575024	-1637.047882	EMD2
ACGGTTAGTGCTCTCT-1	singlet	2	-572.10148	-522.309236	-95.73922022	EMD2
ACGGTTAGTTGGTGTT-1	singlet	1	-34.85612307	-17.02732221	-38.09502853	EMD2
ACGGTTATCGTACACA-1	singlet	2	-1361.012974	-1223.39411	-564.1743342	EMD2
ACGGTTATCTCTGCTG-1	singlet	1	-2155.623346	-660.9721719	-1764.542164	EMD2
ACGTAACAGAAGTATC-1	singlet	1	-392.8029491	-144.7444358	-346.5626325	EMD2
ACGTAACAGAGTCGAC-1	singlet	1	-1647.448984	-527.053645	-1269.604404	EMD2
ACGTAACAGCGGATCA-1	singlet	0	-132.234868	-504.5710959	-439.182119	EMD2
ACGTAACAGTTCCAGT-1	singlet	1	-3159.857061	-1041.784449	-2907.950823	EMD2
ACGTAACCACAAGCAG-1	singlet	2	-819.6821894	-869.7505057	-303.5306719	EMD2
ACGTAACTCAGACATC-1	singlet	1	-3631.546643	-1170.17493	-2949.245862	EMD2
ACGTAACTCATCGACA-1	singlet	0	-123.3765753	-418.0041049	-401.8141366	EMD2
ACGTAACTCGCCTAGG-1	singlet	1	-1873.252954	-660.2032385	-1504.72956	EMD2
ACGTACAAGAAGAACG-1	singlet	1	-928.0892498	-278.1533798	-820.0600907	EMD2
ACGTACAAGTCTAACC-1	singlet	1	-261.7028063	-110.4932121	-233.2773691	EMD2
ACGTACACACACGGAA-1	singlet	0	-1078.918399	-3535.442771	-3206.745539	EMD2
ACGTACAGTGTGAGCA-1	singlet	2	-914.1299109	-854.8892238	-307.8088686	EMD2
ACGTACAGTTTGACAC-1	singlet	2	-5124.854573	-4107.812674	-1451.576319	EMD2
ACGTACATCAAACGAA-1	singlet	1	-1462.910621	-506.4637179	-1234.428936	EMD2
ACGTACATCAATGTCG-1	singlet	1	-4718.350862	-1401.253397	-3600.696745	EMD2
ACGTACATCGGAAGGT-1	singlet	0	-116.8628357	-456.4744934	-367.9591832	EMD2
ACGTAGTAGATCGCCC-1	singlet	1	-192.0193936	-41.04552676	-140.9432051	EMD2
ACGTAGTAGGCGACAT-1	singlet	2	-1690.428912	-1390.877312	-565.1010952	EMD2
ACGTAGTAGTCCGCCA-1	singlet	0	-725.92304	-2127.720928	-1916.900554	EMD2
ACGTAGTCAAGCCATT-1	singlet	1	-1102.757435	-315.2399142	-886.5633725	EMD2
ACGTAGTCACCCAACG-1	singlet	1	-1437.487928	-436.3881081	-1244.013974	EMD2
ACGTAGTGTAAGACCG-1	singlet	0	-325.1799812	-1008.806032	-938.4563565	EMD2
ACGTAGTGTACGCTTA-1	singlet	1	-567.3396574	-204.8690179	-465.2078908	EMD2
ACGTAGTGTAGGAGGG-1	singlet	1	-848.8048584	-260.5310131	-705.6255622	EMD2
ACGTAGTGTGATACTC-1	singlet	1	-2122.024959	-624.5179508	-1754.496301	EMD2
ACGTAGTGTGGAGGTT-1	unassigned	0/2	-4.39013544	-6.697598563	-7.002194107	EMD2
ACGTAGTTCAACGCTA-1	singlet	0	-267.3652572	-791.1674865	-794.619388	EMD2
ACGTCCTAGCGTGAAC-1	singlet	1	-522.789275	-196.5326177	-436.8771502	EMD2
ACGTCCTAGGCCTAGA-1	singlet	1	-372.5993077	-145.7767731	-289.0838703	EMD2
ACGTCCTAGTTCCGGC-1	singlet	1	-1683.279037	-570.4209806	-1418.157324	EMD2
ACGTCCTCATCCTCAC-1	singlet	2	-1157.445806	-1160.319745	-402.3673837	EMD2
ACGTCCTGTCACCGAC-1	singlet	1	-286.9854461	-120.8747492	-229.0896524	EMD2
ACGTCCTGTCCCTCAT-1	singlet	2	-1583.418937	-1268.000502	-549.3308798	EMD2
ACGTCCTGTCGCTCGA-1	doublet	2/0	-4493.011478	-4946.388847	-2773.999139	EMD2
ACGTCCTTCAGCGGAA-1	singlet	0	-190.3219053	-511.3087837	-453.1066011	EMD2
ACGTCCTTCATCTATC-1	singlet	2	-5014.721309	-4715.880227	-1641.316077	EMD2
ACGTCCTTCCCATAAG-1	singlet	0	-169.8750194	-795.125005	-464.1445301	EMD2
ACGTTCCAGCACTCTA-1	doublet	1/0	-411.0801593	-228.9352181	-407.5445762	EMD2
ACGTTCCAGTAACCTC-1	singlet	1	-163.7035389	-68.12528658	-152.5744008	EMD2
ACGTTCCCAACAGCTT-1	singlet	2	-324.7368351	-252.2830676	-106.391446	EMD2

ACGTTCCCACTAACGT-1	singlet	0	-52.27810863	-158.3412113	-174.6567098	EMD2
ACGTTCCGTAAGTCAA-1	singlet	1	-296.3194757	-98.65504573	-213.3048533	EMD2
ACGTTCCGTCAAGGCA-1	singlet	1	-261.0355829	-84.33750448	-210.8603543	EMD2
ACGTTCCGTGCATGAG-1	singlet	0	-805.4702753	-3235.02233	-3019.244114	EMD2
ACGTTCCTCGATTGAC-1	singlet	1	-4895.650657	-1453.134967	-3669.398537	EMD2
ACTACGAAGATCGCCC-1	singlet	2	-3190.679482	-2841.349646	-955.3182058	EMD2
ACTACGAAGGAACGAA-1	singlet	1	-386.4175613	-151.3397754	-313.9816077	EMD2
ACTACGACACGAGGTA-1	singlet	1	-2565.450864	-685.9910516	-2029.486628	EMD2
ACTACGACATGATCTG-1	singlet	0	-23.20739877	-50.18300633	-54.46639947	EMD2
ACTACGAGTAGGGTAC-1	singlet	1	-214.2898782	-102.8074151	-205.7564325	EMD2
ACTACGAGTCAGATTC-1	singlet	1	-330.8565016	-133.2002759	-315.2682804	EMD2
ACTATCTAGCCAGTAG-1	singlet	1	-1595.23082	-546.9830989	-1199.002016	EMD2
ACTATCTAGTGATTCC-1	singlet	1	-98.17495129	-47.55543443	-92.1727715	EMD2
ACTATCTCAATGGCAG-1	singlet	1	-406.0784359	-163.7795698	-339.8173557	EMD2
ACTATCTGTAGCGTAG-1	singlet	1	-300.1444878	-120.4253462	-290.9010299	EMD2
ACTATCTGTATGTGTC-1	singlet	0	-2167.766674	-7246.409489	-6731.932803	EMD2
ACTATCTTCGTAGGAG-1	singlet	2	-3702.577522	-2904.574476	-1383.28754	EMD2
ACTATCTTCTCATTTG-1	singlet	1	-3708.849918	-1305.178196	-3319.781829	EMD2
ACTATGGAGTTCTACG-1	singlet	1	-2893.779566	-692.16699	-2113.079687	EMD2
ACTATGGTCATGTCAG-1	singlet	1	-1002.94593	-435.2616763	-844.4275874	EMD2
ACTATGGTCATTGTTC-1	singlet	0	-135.7866427	-293.1176991	-257.929676	EMD2
ACTATGGTCTGCGGCA-1	singlet	1	-5679.60325	-1732.077494	-4683.830282	EMD2
ACTATTCAGAGAGTTT-1	singlet	0	-1045.819125	-3877.271156	-3900.13132	EMD2
ACTATTCAGCTAGTTC-1	singlet	1	-405.9092244	-151.9961454	-368.9493091	EMD2
ACTATTCAGTAGGAAG-1	singlet	1	-3479.696992	-957.8822955	-2647.620104	EMD2
ACTATTCCAATCGCCG-1	singlet	2	-132.8302571	-111.5358222	-43.33211874	EMD2
ACTATTCGTAACTGCT-1	singlet	0	-1666.259416	-6057.117266	-5497.077915	EMD2
ACTATTCGTACTAGCT-1	singlet	1	-400.302901	-176.7897055	-318.4832142	EMD2
ACTATTCGTCGCGTTG-1	singlet	1	-4581.381718	-1317.74509	-3574.088682	EMD2
ACTATTCGTCTGTCCT-1	singlet	0	-706.310197	-2082.996694	-1988.131229	EMD2
ACTATTCTCATAGCAC-1	singlet	2	-2673.153858	-2060.96263	-1198.124528	EMD2
ACTATTCTCCAATGCA-1	singlet	0	-210.7821283	-604.5467399	-512.8439098	EMD2
ACTATTCTCCCGTTCA-1	singlet	0	-238.5054909	-726.9437269	-640.7882919	EMD2
ACTATTCTCGCTTTAT-1	singlet	1	-2937.466328	-918.2262994	-2041.739466	EMD2
ACTATTCTCTGGGCCA-1	singlet	1	-4355.475142	-1179.427233	-3100.00512	EMD2
ACTCCCAAGAATCCCT-1	singlet	1	-190.0875432	-40.20843593	-108.4900702	EMD2
ACTCCCAAGATCACTC-1	singlet	1	-1419.945259	-597.9018816	-1124.988068	EMD2
ACTCCCAAGGTTACCT-1	singlet	1	-1578.659918	-648.2537341	-1177.032302	EMD2
ACTCCCAAGGTTGTTC-1	singlet	2	-10085.97496	-9011.710761	-3929.615784	EMD2
ACTCCCACAATAGGGC-1	singlet	1	-949.7516856	-340.351266	-797.3278027	EMD2
ACTCCCACACGGTGCT-1	singlet	1	-269.886907	-115.030983	-225.1080448	EMD2
ACTCCCACAGAGTCAG-1	singlet	0	-80.58108767	-271.1725626	-275.4659927	EMD2
ACTCCCAGTGGGTCAA-1	singlet	2	-239.7117277	-217.366963	-90.4993713	EMD2
ACTCCCAGTTAGAAAC-1	singlet	0	-394.7301223	-1145.397906	-1134.859687	EMD2
ACTCCCAGTTGATGTC-1	singlet	1	-659.3252815	-185.5391825	-423.7688483	EMD2
ACTCCCATCAAGCCCG-1	singlet	1	-915.9363397	-273.8694786	-802.2077212	EMD2
ACTCCCATCCGGACGT-1	singlet	2	-2169.057526	-1591.171113	-598.0164653	EMD2
ACTCCCATCGTGTCAA-1	singlet	1	-2909.363491	-1016.747652	-2509.171719	EMD2
ACTCTCGAGCGGGTAT-1	singlet	0	-712.3744523	-1865.51886	-1794.685308	EMD2
ACTCTCGAGGGACCAT-1	singlet	0	-619.3875794	-1802.723263	-1715.582695	EMD2
ACTCTCGAGGGAGGAC-1	singlet	1	-428.0612984	-168.6586698	-346.2037135	EMD2
ACTCTCGCACTATGTG-1	singlet	1	-459.6403282	-178.7752479	-348.844415	EMD2
ACTCTCGCAGACCTAT-1	singlet	2	-1762.901061	-1457.573128	-571.9442875	EMD2
ACTCTCGCAGGTTCGC-1	singlet	1	-1406.873818	-477.9015021	-1222.372218	EMD2
ACTCTCGGTATGACAA-1	singlet	1	-2772.516248	-800.1192055	-2389.154094	EMD2
ACTCTCGGTGATTCAC-1	singlet	1	-150.2965273	-51.53269578	-89.23233295	EMD2
ACTCTCGGTTACACTG-1	singlet	1	-4010.061904	-1135.324	-3197.16514	EMD2
ACTCTCGTCAAACGTC-1	singlet	0	-103.617115	-241.3853301	-182.1688584	EMD2
ACTCTCGTCCAAGGGA-1	singlet	0	-152.4948165	-404.5736921	-397.6593142	EMD2
ACTGATGAGACGACTG-1	singlet	2	-691.4592852	-588.5293578	-264.9340819	EMD2
ACTGATGAGCTCCACG-1	singlet	0	-1118.629424	-3840.607066	-3714.544664	EMD2
ACTGATGCACCATATG-1	singlet	1	-2372.94549	-831.5770189	-2123.370577	EMD2
ACTGATGCACCTGCGA-1	singlet	2	-499.2019727	-452.5036813	-232.0383682	EMD2
ACTGATGCATCTCAAG-1	singlet	1	-958.8936867	-307.8689421	-688.5191442	EMD2
ACTGATGGTAACATGA-1	singlet	1	-468.9233693	-199.2180435	-436.0730317	EMD2
ACTGATGGTGTTAGCT-1	singlet	1	-791.3686604	-295.2763107	-690.9836098	EMD2
ACTGATGTCATGCGGC-1	singlet	1	-365.6041927	-123.5559314	-305.2924067	EMD2
ACTGCAAAGCTCGACC-1	singlet	0	-1127.745177	-3443.559846	-3338.04311	EMD2
ACTGCAAAGGCTCACC-1	singlet	2	-1045.765121	-922.7268483	-334.7612813	EMD2
ACTGCAACACACGGAA-1	singlet	2	-8577.607693	-7084.159771	-2559.374476	EMD2
ACTGCAACAGAGGTAC-1	singlet	1	-603.5148352	-237.4192443	-554.3027434	EMD2
ACTGCAACAGGCGAAT-1	singlet	0	-689.9814771	-2244.383562	-2281.145753	EMD2
ACTGCAACATGGCACC-1	singlet	0	-569.9831222	-1940.212865	-1784.030293	EMD2
ACTGCAACATTCACCC-1	singlet	2	-630.7062545	-638.1184751	-255.1475387	EMD2
ACTGCAAGTCGAACGA-1	doublet	45323	-7596.084367	-4704.195771	-3596.188453	EMD2
ACTGCAATCACAGTGT-1	singlet	1	-139.1950501	-71.43297102	-133.4723021	EMD2
ACTGCAATCACCACAA-1	singlet	2	-1081.154905	-1029.82152	-421.1851619	EMD2
ACTGCAATCGGTAAGG-1	singlet	2	-6717.751434	-5446.314779	-2123.384709	EMD2
ACTGCAATCTATACGG-1	singlet	0	-187.9519999	-655.5016628	-525.5749984	EMD2
ACTGTCCAGCTCGCAC-1	singlet	1	-4602.447637	-1269.718539	-3426.597313	EMD2
ACTGTCCAGGACAACC-1	singlet	1	-650.4341801	-257.1772353	-511.7071721	EMD2
ACTGTCCCAAGCCATT-1	singlet	1	-2101.667252	-695.9626418	-1596.763408	EMD2
ACTGTCCCAGGTCTCG-1	singlet	1	-873.0946436	-317.7815639	-671.6200503	EMD2
ACTGTCCGTGTCGCTG-1	singlet	1	-291.6689334	-110.7114094	-239.5013531	EMD2
ACTGTCCGTTGAGGAC-1	singlet	1	-2587.470823	-716.8857698	-2290.326403	EMD2
ACTGTCCTCACACGAT-1	singlet	1	-451.4075185	-154.4213107	-351.3712099	EMD2
ACTGTCCTCACTGGGC-1	singlet	1	-2111.067608	-766.870028	-1748.235379	EMD2
ACTGTCCTCCCGAATA-1	singlet	1	-2779.755981	-619.8674735	-1936.060075	EMD2
ACTGTCCTCGTCACCT-1	singlet	1	-541.4776095	-208.8468726	-440.1467647	EMD2
ACTGTCCTCGTTCCTG-1	singlet	1	-59.3090672	-15.75315875	-29.38789225	EMD2
ACTGTCCTCTCTCTAA-1	singlet	1	-1901.951616	-714.8747568	-1599.040142	EMD2
ACTGTGAAGACTCATC-1	singlet	2	-955.586425	-826.7924628	-362.1549416	EMD2
ACTGTGAAGAGAGTTT-1	singlet	1	-402.987452	-171.823935	-332.1612571	EMD2
ACTGTGAAGCATGCAG-1	singlet	0	-1240.671921	-4183.163337	-3761.490793	EMD2
ACTGTGAAGCGCCTTG-1	singlet	1	-520.8761882	-164.8149463	-408.732426	EMD2
ACTGTGAAGCTTCGTA-1	singlet	2	-1101.740432	-1288.020571	-424.6758962	EMD2
ACTGTGACAACTGTGT-1	singlet	0	-500.3579231	-1514.647243	-1389.452942	EMD2
ACTGTGACAGCTGGTC-1	singlet	1	-237.8421873	-83.56195227	-167.5131015	EMD2
ACTGTGACATCAGCAT-1	singlet	1	-2361.356504	-689.3858381	-1727.75679	EMD2
ACTGTGACATCGAAGG-1	singlet	0	-530.4806514	-1710.414467	-1571.790688	EMD2
ACTGTGACATTGTCGA-1	singlet	1	-205.0739876	-95.02375028	-185.2972093	EMD2
ACTGTGAGTAGTGCGA-1	singlet	0	-667.1330761	-2246.246513	-1945.579255	EMD2
ACTGTGAGTCCAGCAC-1	singlet	1	-471.9621959	-192.4490791	-412.4497517	EMD2
ACTGTGATCCACCCTA-1	singlet	0	-399.4114029	-1098.13768	-965.24018	EMD2
ACTGTGATCGAAGTGG-1	singlet	0	-202.0414631	-582.8646772	-545.941141	EMD2
ACTGTGATCGATGCAT-1	singlet	1	-2394.809664	-690.3154958	-1795.08152	EMD2
ACTTAGGAGCATACTC-1	singlet	0	-142.1732608	-373.7103663	-381.4621366	EMD2
ACTTAGGAGGACTTCT-1	singlet	2	-282.8287561	-347.6599601	-111.6581162	EMD2
ACTTAGGAGGATATGT-1	singlet	1	-110.962704	-32.07308103	-75.4577231	EMD2
ACTTAGGAGGTAAGAG-1	singlet	1	-8.46573458	-3.798670817	-10.48489641	EMD2

ACTTAGGAGTTCACTG-1	singlet	0	-2240.060677	-6979.77703	-6830.489023	EMD2
ACTTAGGCAACTGCCG-1	singlet	1	-2196.427404	-708.412314	-1751.608249	EMD2
ACTTAGGCAATATCCG-1	singlet	0	-110.0986371	-314.9799761	-255.9721116	EMD2
ACTTAGGCACAGTATC-1	singlet	2	-2911.552801	-2341.81803	-1028.522373	EMD2
ACTTAGGCACGCAAAG-1	singlet	1	-512.3019439	-167.7666202	-343.5859138	EMD2
ACTTAGGGTACCCACG-1	singlet	0	-1096.969319	-3618.006402	-3294.910888	EMD2
ACTTAGGGTAGATTAG-1	singlet	1	-230.5459749	-103.82993	-205.0589898	EMD2
ACTTAGGTCAGGACAG-1	singlet	0	-589.6859282	-1735.752036	-1651.01909	EMD2
ACTTAGGTCCCACAGG-1	singlet	0	-2113.695033	-6464.84417	-6034.619691	EMD2
ACTTAGGTCGATCCAA-1	singlet	0	-411.7228579	-1532.11355	-1267.922868	EMD2
ACTTAGGTCGCGTGAC-1	singlet	1	-344.9075076	-150.3290828	-267.7071787	EMD2
ACTTAGGTCGGCTGAC-1	singlet	1	-4691.968662	-1492.273127	-4064.371433	EMD2
ACTTATCAGATTTGCC-1	unassigned	1	-19.31624477	-3.605294129	-6.193012817	EMD2
ACTTATCAGGATCATA-1	singlet	2	-3489.33636	-2716.201055	-1430.723239	EMD2
ACTTATCCACTGCTTC-1	singlet	1	-827.2036033	-324.8936198	-697.6871996	EMD2
ACTTATCCAGGAATCG-1	singlet	1	-1721.459221	-521.8993136	-1449.511512	EMD2
ACTTATCCATGTACGT-1	singlet	2	-1877.066056	-2004.813539	-545.6597379	EMD2
ACTTATCGTAACTAAG-1	singlet	2	-1529.374655	-1495.732023	-644.1794078	EMD2
ACTTATCGTACTCCGG-1	singlet	1	-685.9931355	-265.3567428	-546.6592439	EMD2
ACTTATCGTATCCTTT-1	singlet	1	-728.3283763	-247.5583954	-634.4213588	EMD2
ACTTATCTCCGTGGCA-1	singlet	0	-242.484228	-1231.548027	-989.2656186	EMD2
ACTTATCTCGGAGTGA-1	singlet	1	-1078.445956	-379.7860394	-782.1859303	EMD2
ACTTCCGAGCAATAAC-1	singlet	1	-383.9594061	-163.1939178	-336.6825556	EMD2
ACTTCCGAGGAGTCTG-1	singlet	2	-480.1075028	-475.0107979	-194.5886396	EMD2
ACTTCCGAGGTCTGGA-1	singlet	1	-346.7947817	-133.4552467	-299.6190374	EMD2
ACTTCCGCAGAACTTC-1	singlet	1	-600.2745322	-234.680742	-500.3176048	EMD2
ACTTCCGCAGCACGAA-1	singlet	0	-784.9695791	-2583.597863	-2481.26668	EMD2
ACTTCCGCAGCTGAAG-1	singlet	1	-309.8279266	-140.564764	-319.770922	EMD2
ACTTCCGCATTCTCTA-1	singlet	2	-201.5246797	-168.3172469	-86.80447091	EMD2
ACTTCCGGTAAGAACT-1	singlet	1	-227.7028596	-82.77103024	-194.3409075	EMD2
ACTTCCGGTCACCGCA-1	singlet	1	-1182.181124	-354.9013322	-1087.648241	EMD2
ACTTCCGGTGAATTAG-1	singlet	0	-371.7733256	-1150.150622	-1070.240775	EMD2
ACTTCCGGTGCTTATG-1	singlet	1	-1119.122544	-345.2981586	-857.6298483	EMD2
ACTTCCGTCGCCAATA-1	unassigned	2	-3.422153511	-2.029334456	-0.999055389	EMD2
ACTTCCGTCGTTGCCT-1	singlet	2	-1295.440441	-1537.817571	-409.5820557	EMD2
ACTTCGCAGCTAATCC-1	singlet	1	-1808.873558	-579.452394	-1389.13331	EMD2
ACTTCGCAGGCTCACC-1	singlet	1	-921.0615995	-279.4173162	-767.4020033	EMD2
ACTTCGCAGTAGATCA-1	singlet	1	-1431.236122	-455.1682386	-1246.741838	EMD2
ACTTCGCCAAATAGCA-1	singlet	1	-325.5822525	-146.1699811	-297.705816	EMD2
ACTTCGCCAGCAGACA-1	singlet	1	-219.2876119	-91.58209783	-160.4089895	EMD2
ACTTCGCCATCATTTC-1	singlet	1	-2183.515131	-773.0210548	-1697.979051	EMD2
ACTTCGCGTAGAGTTA-1	singlet	1	-103.6645417	-39.50935696	-64.28977346	EMD2
ACTTCGCGTGCGACAA-1	singlet	2	-1125.695942	-1011.85528	-324.9709186	EMD2
ACTTCGCGTGGGACAT-1	singlet	1	-945.7876155	-297.76651	-753.3271579	EMD2
ACTTCGCGTTACTCAG-1	singlet	0	-546.0271962	-1803.331957	-1585.218676	EMD2
ACTTCGCGTTGTATGC-1	singlet	0	-674.1616964	-2335.797641	-2305.76929	EMD2
ACTTCGCTCATCGTAG-1	singlet	1	-1573.268916	-546.7490804	-1416.335226	EMD2
ACTTCGCTCCATCTCG-1	singlet	1	-1547.413843	-557.0571966	-1368.346677	EMD2
ACTTCGCTCTTAGCCC-1	singlet	2	-2655.581422	-2548.863381	-944.7954674	EMD2
ACTTCGCTCTTTCGAT-1	singlet	1	-918.2478567	-330.9037047	-814.8476138	EMD2
ACTTTCAAGAGTATAC-1	singlet	1	-1369.221437	-410.428214	-1239.524384	EMD2
ACTTTCAAGATTGACA-1	singlet	2	-1641.444375	-1510.952213	-616.6078894	EMD2
ACTTTCAAGTACCATC-1	singlet	1	-90.0696568	-38.06086233	-85.15162171	EMD2
ACTTTCACAACACGAG-1	singlet	0	-54.50088168	-159.0233756	-145.1835343	EMD2
ACTTTCACAAGAGGCT-1	singlet	1	-2723.968511	-953.9609038	-2301.423455	EMD2
ACTTTCAGTACATTGC-1	singlet	1	-3075.900099	-944.548171	-2433.511521	EMD2
ACTTTCAGTCCGAAGA-1	singlet	0	-205.4218438	-562.5288414	-443.2209676	EMD2
ACTTTCATCGCTTACC-1	singlet	1	-1112.88543	-368.948081	-806.8095968	EMD2
ACTTTCATCTTTCTAG-1	singlet	1	-2475.643252	-811.0818802	-2130.02317	EMD2
ACTTTGTAGAGTCAGC-1	singlet	0	-38.04402095	-73.39297193	-104.0658252	EMD2
ACTTTGTAGGTGCAGT-1	singlet	1	-89.92251648	-28.78586821	-67.79776904	EMD2
ACTTTGTAGGTTCCAT-1	singlet	2	-2272.456512	-2104.592726	-912.2711317	EMD2
ACTTTGTAGTTGTCGT-1	singlet	0	-407.3062988	-1082.456926	-1074.92573	EMD2
ACTTTGTCAACCGCTG-1	singlet	1	-2293.381129	-748.8963391	-1953.350549	EMD2
ACTTTGTCAATGGGTG-1	singlet	1	-1755.807952	-597.3685613	-1447.455626	EMD2
ACTTTGTGTCACCCTT-1	singlet	1	-3546.853142	-1097.546885	-2987.15145	EMD2
ACTTTGTGTTGCGGCT-1	singlet	0	-966.4936981	-3738.197502	-3619.08273	EMD2
ACTTTGTTCTCAGTCC-1	singlet	1	-262.6592967	-98.75229691	-179.6021948	EMD2
ACTTTGTTCTCTCGCA-1	singlet	1	-1355.309772	-467.5491293	-1038.72087	EMD2
AGAAATGAGGCCCAAA-1	singlet	1	-2503.205893	-782.5882259	-2206.314957	EMD2
AGAAATGCATGGGAAC-1	singlet	2	-8103.741385	-5919.414337	-1513.795312	EMD2
AGAAATGGTATTCCTT-1	singlet	1	-439.8198316	-149.6531491	-345.1480999	EMD2
AGAAATGTCGTAGTGT-1	singlet	1	-184.6816395	-103.4348022	-198.6473759	EMD2
AGAACAAAGCAATAGT-1	singlet	0	-1071.870808	-2833.308319	-2772.121244	EMD2
AGAACAAAGGAACTCG-1	singlet	0	-769.5267079	-2216.009048	-2195.572253	EMD2
AGAACAAAGGCAGGGA-1	singlet	1	-2181.851436	-516.694967	-1456.361381	EMD2
AGAACAAAGTAATCCC-1	singlet	1	-3608.909265	-1108.387	-3398.311348	EMD2
AGAACAACAATTGAAG-1	singlet	1	-209.4704578	-88.11518411	-215.0881165	EMD2
AGAACAACACCGTCGA-1	singlet	2	-54.64943328	-73.35363211	-14.96321815	EMD2
AGAACAACATTGCAAC-1	singlet	1	-596.1863097	-199.9753148	-420.3671973	EMD2
AGAACCTAGACGCCAA-1	singlet	2	-97.72641882	-55.31757296	-26.58269111	EMD2
AGAACCTAGGCTAACG-1	singlet	1	-439.7190338	-199.5706232	-385.5400169	EMD2
AGAACCTAGGGTCAAC-1	singlet	0	-799.3913596	-2046.980406	-2123.470324	EMD2
AGAACCTCACAGTATC-1	singlet	1	-488.2193771	-200.2410383	-408.7331873	EMD2
AGAACCTCAGTGTACT-1	singlet	1	-538.019281	-203.3221341	-454.2078586	EMD2
AGAACCTCATTCTCCG-1	singlet	1	-692.8082618	-259.7318673	-629.4936796	EMD2
AGAACCTGTAATGATG-1	singlet	2	-1340.850862	-1181.903866	-442.9219827	EMD2
AGAACCTGTGCGAACA-1	singlet	1	-473.715712	-174.1200275	-354.2649492	EMD2
AGAACCTTCGAACCTA-1	singlet	1	-492.09007	-217.3032822	-418.4469671	EMD2
AGAACCTTCGAGCACC-1	singlet	2	-439.1681513	-348.3400013	-215.135937	EMD2
AGAACCTTCGCTGACG-1	singlet	1	-239.3062176	-87.44927918	-167.6051836	EMD2
AGAACCTTCTAGAGCT-1	singlet	2	-293.4561682	-292.0524144	-110.6125512	EMD2
AGAAGCGAGAATCGCG-1	singlet	0	-1132.186127	-3863.236079	-3512.226193	EMD2
AGAAGCGAGACACACG-1	singlet	0	-214.3593701	-524.1068069	-500.4085813	EMD2
AGAAGCGAGCACTCCG-1	singlet	1	-77.91070148	-32.37657052	-56.08117561	EMD2
AGAAGCGAGGGTTTCT-1	singlet	2	-382.263002	-367.1669774	-145.8636191	EMD2
AGAAGCGAGGTGGGTT-1	singlet	1	-457.9950844	-170.9251279	-407.0929624	EMD2
AGAAGCGCACAACCGC-1	singlet	1	-288.356249	-80.16786017	-217.3870793	EMD2
AGAAGCGCACATGTTG-1	singlet	1	-5656.878053	-1748.780044	-4395.069262	EMD2
AGAAGCGCAGTCGCAC-1	singlet	1	-1877.969164	-536.0831509	-1583.96819	EMD2
AGAAGCGCATCGAACT-1	singlet	1	-408.6146996	-144.78652	-328.9586661	EMD2
AGAAGCGGTGAGGAAA-1	singlet	2	-1639.244464	-1744.261796	-522.4288658	EMD2
AGAAGCGGTGGGTATG-1	singlet	0	-2811.712168	-9884.662845	-9738.605621	EMD2
AGAAGCGGTGTACAGG-1	singlet	1	-259.3549708	-97.53457282	-214.1297596	EMD2
AGAAGCGTCCGGACTG-1	singlet	1	-94.42841575	-32.04289395	-70.11812222	EMD2
AGAAGCGTCTCCAAGA-1	singlet	0	-159.994786	-435.2610153	-413.1566206	EMD2
AGAAGTAAGAAACTGT-1	singlet	2	-1481.932052	-1202.083729	-491.5553989	EMD2
AGAAGTAAGAGATTCA-1	singlet	1	-2301.473387	-825.3323925	-2179.69612	EMD2
AGAAGTAAGCAAGGAA-1	singlet	1	-6494.116601	-1948.370274	-4963.469805	EMD2
AGAAGTAAGTAGGCCA-1	singlet	1	-2630.045415	-961.6427437	-2154.486378	EMD2

AGAAGTACACTACTTT-1	singlet	2	-2980.504893	-2238.857241	-914.1218913	EMD2
AGAAGTACACTATCGA-1	singlet	1	-2918.899885	-1034.950958	-2518.05622	EMD2
AGAAGTACAGGGACTA-1	singlet	2	-798.3956471	-729.0548366	-352.8925622	EMD2
AGAAGTACATCTGCGG-1	singlet	0	-268.3375142	-952.2343322	-803.5863463	EMD2
AGAAGTACATGACAAA-1	doublet	0/1	-981.0685599	-1581.385031	-1684.244205	EMD2
AGAAGTACATTCGATG-1	singlet	1	-2066.24555	-639.5893272	-1692.527828	EMD2
AGAAGTAGTCGAGTTT-1	unassigned	45293	-313.2854585	-129.951883	-249.5816754	EMD2
AGAAGTAGTCGCGTTG-1	singlet	0	-424.7124687	-1558.718786	-1342.123121	EMD2
AGAAGTAGTGCTATTG-1	singlet	1	-3636.306454	-1254.747883	-3144.489617	EMD2
AGAAGTATCCGCTAGG-1	singlet	2	-2927.763654	-2432.697158	-908.4073785	EMD2
AGAAGTATCTAACACG-1	singlet	0	-915.346442	-3379.784985	-2961.459266	EMD2
AGACAAAAGCAGATAT-1	singlet	1	-154.8818955	-75.74707104	-152.9263944	EMD2
AGACAAAAGGCTTCCG-1	singlet	1	-116.6488011	-33.36854514	-93.29981027	EMD2
AGACAAAAGGGTGGGA-1	singlet	0	-206.3358619	-692.4437201	-642.6772681	EMD2
AGACAAAAGTTGCGAG-1	singlet	1	-338.5025979	-143.699415	-334.5667509	EMD2
AGACAAACAGCTTCGG-1	unassigned	45293	-44.14278756	-20.68131488	-37.91813113	EMD2
AGACAAACATTGGGAG-1	singlet	1	-1640.886204	-579.0872649	-1405.983683	EMD2
AGACAAATCAGGCGAA-1	singlet	1	-451.8319663	-207.0837137	-401.3485407	EMD2
AGACAAATCGGAGTGA-1	unassigned	2	-4.074784613	-1.385820095	-1.223371969	EMD2
AGACAAATCTATTCGT-1	singlet	1	-352.5744545	-145.0645601	-324.9190769	EMD2
AGACAAATCTCCAATT-1	singlet	1	-409.7459538	-159.2990673	-315.5939372	EMD2
AGACAAATCTTGAGCA-1	singlet	2	-623.0351242	-678.7569846	-291.1705391	EMD2
AGACACTCAAACTAAG-1	singlet	1	-2993.448163	-794.5678617	-2171.426607	EMD2
AGACACTCACACCGCA-1	singlet	0	-402.3623698	-1143.709108	-940.6604298	EMD2
AGACACTCAGGCACAA-1	singlet	1	-149.5177716	-61.67139453	-117.3413392	EMD2
AGACACTGTACAGCGA-1	singlet	1	-1968.189398	-698.8304216	-1450.988347	EMD2
AGACACTGTTATAGCC-1	singlet	2	-1278.293428	-1223.419532	-444.281926	EMD2
AGACAGGAGCACCCAC-1	singlet	1	-3402.251419	-926.1323596	-2806.830941	EMD2
AGACAGGCATCGGCCA-1	singlet	2	-979.0509253	-930.2545298	-325.9882671	EMD2
AGACAGGGTCGTCTCT-1	singlet	2	-696.4087725	-555.693598	-226.0546187	EMD2
AGACAGGGTCTTCATT-1	singlet	1	-622.2643689	-268.6376191	-562.1251753	EMD2
AGACAGGTCGCTAAAC-1	singlet	0	-917.8046661	-2420.396751	-2379.753394	EMD2
AGACAGGTCTTTCAGT-1	singlet	0	-320.9762258	-1017.766041	-953.4288502	EMD2
AGACCATCAAACTAGA-1	singlet	0	-621.6655986	-2274.670342	-1959.565269	EMD2
AGACCATCAATGCTCA-1	singlet	1	-1616.558919	-557.4320555	-1273.607974	EMD2
AGACCATCACGCAAAG-1	singlet	1	-2173.305663	-603.7190967	-1864.972463	EMD2
AGACCATGTGCGAACA-1	singlet	1	-356.7483192	-133.0912938	-248.8374327	EMD2
AGACCATGTGCGTTTA-1	singlet	1	-1854.494778	-675.7801288	-1502.793141	EMD2
AGACCATGTGTGATGG-1	singlet	2	-3131.881181	-2577.605304	-1021.077997	EMD2
AGACCCGCAAGAGGCT-1	singlet	2	-259.6162294	-199.3520059	-74.18317112	EMD2
AGACCCGCACCTCTGT-1	singlet	1	-2742.095201	-864.39371	-2214.062954	EMD2
AGACCCGGTCCTACGG-1	singlet	2	-400.2773512	-468.961475	-166.3764243	EMD2
AGACCCGGTGACGTCC-1	singlet	1	-396.6574874	-181.0656987	-344.3810092	EMD2
AGACCCGGTGATGAAT-1	singlet	1	-1271.508785	-479.015919	-1027.004626	EMD2
AGACCCGTCGCATGAT-1	singlet	1	-1299.110716	-427.3181019	-1000.963861	EMD2
AGACTCAAGAGTTGTA-1	singlet	1	-96.04391039	-34.95139367	-90.70106863	EMD2
AGACTCAAGATGCCGA-1	singlet	2	-2347.955229	-2160.535919	-600.8068151	EMD2
AGACTCAAGCGGTATG-1	singlet	1	-1639.52416	-622.7632661	-1505.428233	EMD2
AGACTCAAGGCACTCC-1	singlet	1	-682.0738388	-175.3488989	-453.4115539	EMD2
AGACTCAAGGTCCAGA-1	singlet	1	-812.9290172	-299.8780298	-622.7946018	EMD2
AGACTCAAGTAAGCAT-1	singlet	1	-173.8976714	-86.482011	-177.2918622	EMD2
AGACTCAAGTTGGGAC-1	singlet	1	-515.2295422	-201.3784225	-449.0839128	EMD2
AGACTCACAACGCATT-1	singlet	1	-1933.584243	-611.0115323	-1862.698263	EMD2
AGACTCACAAGTCGTT-1	singlet	1	-1042.426175	-396.8560154	-948.6032413	EMD2
AGACTCACAATTGTGC-1	singlet	0	-1055.373593	-3008.414778	-2925.885134	EMD2
AGACTCACATACAGAA-1	singlet	0	-1256.268938	-3694.187415	-3670.912381	EMD2
AGACTCAGTCGTTGGC-1	singlet	2	-528.5236873	-509.8028967	-195.7171626	EMD2
AGACTCATCAACCTCC-1	singlet	0	-988.0526261	-4139.887996	-3654.842099	EMD2
AGAGAATAGCTAAACA-1	singlet	1	-2212.545452	-583.5893861	-2003.050052	EMD2
AGAGAATAGTTTCTTC-1	singlet	0	-1636.311821	-4371.341429	-3852.181907	EMD2
AGAGAATCAAGATTGA-1	singlet	1	-638.6773209	-249.7710157	-562.0263979	EMD2
AGAGAATCACAAGCAG-1	singlet	1	-678.3565173	-241.0445492	-552.6698866	EMD2
AGAGAATCACACACGC-1	singlet	0	-921.5406618	-3199.208234	-3014.415184	EMD2
AGAGAATCACCTGCTT-1	singlet	1	-833.7273114	-294.6388825	-662.5451062	EMD2
AGAGAATCACTGGACC-1	singlet	1	-315.9413025	-122.0627987	-237.5372459	EMD2
AGAGAATCATCAGTCA-1	singlet	0	-725.1993006	-4406.948251	-3341.84117	EMD2
AGAGAATGTATCAGCT-1	singlet	1	-257.9306723	-108.9085232	-264.7419977	EMD2
AGAGAATGTCCTCCTA-1	singlet	1	-1375.943453	-456.8902766	-1067.964243	EMD2
AGAGAATGTGGGTATG-1	singlet	0	-642.3123261	-1714.899453	-1781.015301	EMD2
AGAGAATGTTTCGTGA-1	singlet	0	-219.5587692	-645.1429134	-653.419223	EMD2
AGAGAATTCAAGTCGT-1	singlet	1	-4169.130397	-1417.984095	-3229.840902	EMD2
AGAGAATTCCCATAGA-1	singlet	1	-157.7616096	-43.85594722	-174.0811745	EMD2
AGAGAATTCCCATTCG-1	singlet	1	-1659.19675	-534.8975488	-1444.963282	EMD2
AGAGAATTCGATCCAA-1	singlet	0	-159.1045972	-330.099326	-296.3918359	EMD2
AGAGAATTCGTGCTCT-1	unassigned	2/0	-46.46579262	-47.91132388	-29.57018027	EMD2
AGAGAGCAGAATTTGG-1	singlet	0	-532.0489649	-1539.171102	-1530.088586	EMD2
AGAGAGCAGGGTGAAA-1	singlet	0	-174.9209068	-551.9856786	-473.9306689	EMD2
AGAGAGCAGTCTACCA-1	singlet	1	-483.2796581	-180.7750572	-434.3570479	EMD2
AGAGAGCCAGACCAAG-1	singlet	0	-152.782134	-364.330322	-341.829122	EMD2
AGAGAGCCAGGACTTT-1	singlet	1	-359.5431631	-168.5564149	-378.3341541	EMD2
AGAGAGCTCATTCGTT-1	singlet	1	-3335.286474	-829.994071	-2405.482641	EMD2
AGAGAGCTCCTCTAAT-1	singlet	2	-1076.988926	-848.1692268	-270.7643321	EMD2
AGAGAGCTCGCAATTG-1	singlet	1	-736.9299794	-247.8975612	-485.5103581	EMD2
AGAGAGCTCGTCACCT-1	singlet	0	-700.7700178	-2984.25745	-2487.267469	EMD2
AGAGCAGCAAGCTACT-1	singlet	1	-1006.184695	-388.2520987	-912.7749905	EMD2
AGAGCAGCACAAGCTT-1	singlet	1	-1970.106784	-548.7495416	-1954.965613	EMD2
AGAGCAGCACGGAAGT-1	singlet	1	-592.0554204	-224.4427737	-434.4950625	EMD2
AGAGCAGCAGACATCT-1	singlet	1	-176.0302573	-74.69747942	-150.3363863	EMD2
AGAGCAGCATGACTTG-1	singlet	1	-1053.286023	-280.8270059	-770.365616	EMD2
AGAGCAGGTAAGATAC-1	doublet	45323	-5656.845882	-3512.046343	-2931.740728	EMD2
AGAGCAGGTATCCCAA-1	doublet	0/1	-857.83768	-1071.061636	-1401.305655	EMD2
AGAGCAGGTCACTCTC-1	singlet	1	-1227.227482	-398.3742066	-1053.315662	EMD2
AGAGCAGGTCAGTCGC-1	singlet	0	-689.5860735	-2422.40969	-2457.435871	EMD2
AGAGCAGTCATGTCTT-1	doublet	45323	-4646.206428	-2857.395291	-1886.071061	EMD2
AGAGCAGTCTGTCGCT-1	singlet	2	-897.849832	-825.4985849	-255.6055643	EMD2
AGAGCCCAGCTGACTT-1	singlet	2	-3426.341085	-2584.890971	-1106.053665	EMD2
AGAGCCCAGTGATAAC-1	singlet	1	-2025.953074	-654.6385064	-1675.542509	EMD2
AGAGCCCCAACTGTGT-1	singlet	2	-6137.613712	-5351.723812	-1916.5705	EMD2
AGAGCCCCACACCGCA-1	singlet	1	-475.4568026	-202.3668709	-488.2102613	EMD2
AGAGCCCCACAGTCCG-1	singlet	1	-407.0330001	-146.1329692	-305.2211066	EMD2
AGAGCCCCAGTAGGAC-1	singlet	1	-5247.306979	-1117.669175	-3716.730454	EMD2
AGAGCCCTCAAACCCA-1	singlet	1	-1006.198332	-296.2692924	-813.6962614	EMD2
AGAGCCCTCAGACCTA-1	singlet	0	-702.1714175	-1837.268967	-1796.522185	EMD2
AGAGCCCTCAGCGCAC-1	singlet	2	-1227.970818	-1219.217521	-426.9933445	EMD2
AGAGCCCTCATGGAGG-1	singlet	2	-334.9254128	-308.6546947	-126.5286366	EMD2
AGAGCCCTCTCAACGA-1	singlet	2	-209.9739342	-165.56493	-78.27424196	EMD2
AGAGCCCTCTGATTCT-1	singlet	2	-3148.410595	-2880.602229	-1147.19064	EMD2
AGATAGAAGCATGTTC-1	singlet	1	-2954.786356	-818.121955	-2380.766013	EMD2
AGATAGAAGCGTTAGG-1	singlet	0	-246.4697343	-682.1184656	-577.0143614	EMD2
AGATAGAAGGTATTGA-1	singlet	1	-2016.407712	-601.1037735	-1610.55199	EMD2

AGATAGAAGTCATCCA-1	doublet	1/0	-2729.702653	-2480.519457	-3559.302728	EMD2
AGATAGACACAATGAA-1	singlet	1	-3235.519564	-1162.220927	-2516.423482	EMD2
AGATAGACACTTCAAG-1	singlet	2	-3143.232667	-2720.142108	-1172.323883	EMD2
AGATAGACAGTATACC-1	singlet	0	-37.83450133	-64.10024815	-56.64959273	EMD2
AGATAGACATCAGTCA-1	singlet	0	-953.1359507	-3523.818783	-3329.770121	EMD2
AGATAGACATCTCGTC-1	singlet	1	-10000.00017	-3156.271987	-8042.133484	EMD2
AGATAGAGTAACATAG-1	singlet	2	-339.8043782	-270.4636337	-140.6568497	EMD2
AGATAGAGTACTCGAT-1	singlet	0	-255.4292968	-743.981634	-687.5564543	EMD2
AGATAGAGTATATGGA-1	singlet	1	-254.8276528	-129.8972858	-218.3153855	EMD2
AGATAGAGTCATCACA-1	singlet	2	-1280.216818	-1199.097121	-506.2984051	EMD2
AGATAGAGTCATTGCA-1	singlet	2	-864.3486554	-721.3761712	-287.3773362	EMD2
AGATAGAGTGGATGAC-1	singlet	1	-1321.579976	-408.0673559	-934.9801176	EMD2
AGATAGAGTGGTGATG-1	singlet	1	-401.0516733	-159.476488	-325.644037	EMD2
AGATAGATCTACGCGG-1	singlet	0	-146.231927	-382.8029802	-316.9776297	EMD2
AGATAGATCTTAAGGC-1	singlet	2	-141.9273169	-131.0616784	-60.69710887	EMD2
AGATAGATCTTCCGTG-1	singlet	1	-422.3201068	-144.9764811	-311.6710916	EMD2
AGATCCAAGAAATTGC-1	doublet	0/2	-5153.40808	-7323.719939	-5357.745846	EMD2
AGATCCAAGAACCCGA-1	singlet	2	-1579.290326	-1394.825868	-651.6862657	EMD2
AGATCCAAGCAATTAG-1	singlet	1	-249.6260164	-97.99339711	-219.537494	EMD2
AGATCCAAGGGATCTG-1	singlet	1	-1707.446166	-494.5031099	-1268.004499	EMD2
AGATCCACACAGGATG-1	singlet	1	-126.3432528	-46.2251825	-100.4319484	EMD2
AGATCCAGTCTCCCTA-1	singlet	1	-713.2659941	-267.2295652	-548.8806586	EMD2
AGATCCAGTGCAGTGA-1	doublet	1/0	-2093.314032	-1492.202049	-2453.701725	EMD2
AGATCCAGTGTTCGAT-1	singlet	0	-429.3715147	-1271.314247	-1256.599558	EMD2
AGATCCATCACTGTCC-1	singlet	1	-625.3078331	-276.8952336	-630.6897188	EMD2
AGATCCATCTAACGGT-1	singlet	1	-724.124686	-295.2021758	-631.9907093	EMD2
AGATCGTAGGCTTAGG-1	singlet	0	-26.8987415	-56.63373725	-60.77462943	EMD2
AGATCGTCAAATGCGG-1	singlet	1	-2070.104526	-559.1673359	-1791.395126	EMD2
AGATCGTGTGACCGTC-1	singlet	2	-158.1196446	-129.3178626	-42.0404298	EMD2
AGATGAAAGAAGTCTA-1	singlet	0	-468.41903	-1326.071206	-1305.932048	EMD2
AGATGAAAGAATCGCG-1	singlet	1	-2517.325134	-828.7834434	-2006.154838	EMD2
AGATGAAAGACAGCGT-1	singlet	2	-3454.537923	-2691.616048	-1183.2352	EMD2
AGATGAAAGATTACCC-1	singlet	1	-388.3820841	-179.3407788	-306.6782381	EMD2
AGATGAAAGGGCCCTT-1	singlet	0	-203.9450421	-604.709874	-607.134592	EMD2
AGATGAAAGTGAACAT-1	singlet	1	-2194.821874	-741.3550015	-1818.937849	EMD2
AGATGAACAGCACAAG-1	singlet	0	-461.7238741	-1385.333996	-1340.478078	EMD2
AGATGAACAGCGTAGA-1	singlet	1	-332.0106349	-129.5832858	-323.7626548	EMD2
AGATGAACATCTCATT-1	singlet	1	-2566.338376	-999.1196677	-2278.46099	EMD2
AGATGAACATGTGACT-1	singlet	2	-2376.434565	-2387.893807	-813.6841466	EMD2
AGATGAAGTACAGCGA-1	singlet	1	-281.3577373	-117.3907131	-298.9297345	EMD2
AGATGAAGTCGAGATG-1	singlet	2	-120.7311999	-46.96426338	-33.21352401	EMD2
AGATGAAGTTACCCTC-1	singlet	1	-5006.135073	-1777.434522	-4098.394707	EMD2
AGATGAAGTTTCTTAC-1	singlet	2	-389.7386604	-365.6886476	-181.8850585	EMD2
AGATGAATCCACGGGT-1	singlet	1	-328.5186099	-109.4486669	-244.1794558	EMD2
AGATGAATCCTGCCAT-1	singlet	1	-349.1639395	-129.315333	-294.7896265	EMD2
AGATGAATCTTTGCGC-1	singlet	2	-2946.243978	-3192.687383	-924.2898184	EMD2
AGATGCTAGCACACCC-1	singlet	2	-797.7206005	-758.6460561	-311.3668304	EMD2
AGATGCTAGGTGCCTC-1	singlet	1	-492.2593432	-226.6227722	-429.3091815	EMD2
AGATGCTAGTTGCTGT-1	singlet	0	-608.6056968	-1990.214101	-1806.059278	EMD2
AGATGCTCACGAGGTA-1	singlet	1	-267.5347004	-111.5228478	-227.9669049	EMD2
AGATGCTGTCATTGCA-1	singlet	1	-855.1468937	-301.6073301	-802.1220822	EMD2
AGATGCTTCTTACCGC-1	singlet	1	-100.7029578	-39.17068389	-80.92957997	EMD2
AGATGCTTCTTTCGAT-1	singlet	0	-605.5682754	-1619.31199	-1543.011498	EMD2
AGCATCAAGCCTGTCG-1	singlet	1	-813.1139796	-271.2225886	-629.261872	EMD2
AGCATCACAGGTTTAC-1	singlet	2	-1671.602807	-1361.850913	-570.4424014	EMD2
AGCATCACATGCAGGA-1	singlet	2	-2830.927981	-2266.042241	-988.0540752	EMD2
AGCATCAGTTGCTAGT-1	singlet	1	-140.4700707	-55.81327016	-93.34818692	EMD2
AGCATCATCACCATAG-1	unassigned	1	-5.095929446	-0.870829555	-1.379424961	EMD2
AGCATCATCGGAATGG-1	singlet	0	-526.4497751	-1645.192981	-1604.420365	EMD2
AGCATCATCTCGCCTA-1	singlet	1	-1512.603238	-566.7814384	-1292.342648	EMD2
AGCCAATAGCATGATA-1	singlet	0	-1590.245168	-5865.237084	-5825.12662	EMD2
AGCCAATAGCGCTTCG-1	singlet	1	-1064.929103	-436.1654897	-947.4057153	EMD2
AGCCAATAGGTCCTGC-1	singlet	0	-224.2708947	-618.3061201	-603.0978285	EMD2
AGCCAATAGTGTACAA-1	singlet	2	-2445.933091	-2294.955427	-688.9839158	EMD2
AGCCAATCACAAAGCG-1	singlet	2	-528.5372525	-511.2717521	-229.0134952	EMD2
AGCCAATCACAATGTC-1	singlet	1	-952.6054178	-335.9947939	-823.1834028	EMD2
AGCCAATCATACCATG-1	singlet	1	-1227.285237	-289.4522672	-756.1991416	EMD2
AGCCAATGTATCATGC-1	singlet	1	-3712.179323	-1185.065164	-3151.60922	EMD2
AGCCAATGTGGTCTCG-1	singlet	1	-448.0066462	-151.5855898	-394.8289201	EMD2
AGCCAATGTTTCACTT-1	singlet	0	-365.3536355	-795.3386699	-750.3480637	EMD2
AGCCAATTCAGGACAG-1	singlet	1	-2241.413735	-751.9824684	-1746.657045	EMD2
AGCCAATTCATACGAC-1	singlet	1	-303.1539741	-102.5993276	-229.1561662	EMD2
AGCCAATTCTGGCCAG-1	singlet	1	-126.0683563	-25.99774063	-81.25173068	EMD2
AGCCACGAGAGCCCAA-1	singlet	0	-370.3080221	-1540.595429	-1236.466819	EMD2
AGCCACGAGCACAAAT-1	singlet	1	-2763.391329	-936.4328043	-2299.302125	EMD2
AGCCACGAGCGATTCT-1	singlet	0	-516.9906903	-1511.304095	-1389.848888	EMD2
AGCCACGAGGACAAGA-1	singlet	1	-6363.116998	-1575.932684	-5097.318063	EMD2
AGCCACGAGGTAGCAC-1	singlet	2	-1968.125283	-1652.888865	-687.1817141	EMD2
AGCCACGAGGTTAAAC-1	singlet	1	-1063.665511	-336.9154784	-831.5549153	EMD2
AGCCACGCACGCGTGT-1	singlet	0	-505.9303339	-1509.413217	-1484.309141	EMD2
AGCCACGGTATGTCAC-1	singlet	1	-1550.679623	-532.8880617	-1473.400249	EMD2
AGCCACGGTTATCTGG-1	singlet	2	-2404.905827	-2117.127192	-657.2304044	EMD2
AGCCACGGTTCTTCAT-1	singlet	2	-504.60863	-488.536295	-189.3161878	EMD2
AGCCACGTCGCAGAGA-1	singlet	2	-1255.670755	-1186.622895	-384.9053836	EMD2
AGCCAGCAGACGGTCA-1	singlet	2	-3324.567632	-2504.217821	-954.2234515	EMD2
AGCCAGCAGACTACCT-1	singlet	1	-3708.442528	-1215.885002	-2958.760111	EMD2
AGCCAGCAGCAGCCTC-1	singlet	2	-233.2800888	-214.2022049	-120.404557	EMD2
AGCCAGCAGCCAAGTG-1	singlet	0	-1331.493428	-3951.069878	-3781.959669	EMD2
AGCCAGCAGCGAGTCA-1	unassigned	1/0	-15.54772867	-11.61982694	-36.95618941	EMD2
AGCCAGCAGGAAAGAC-1	singlet	1	-355.817052	-147.4963896	-305.7284868	EMD2
AGCCAGCCACCTCTGT-1	singlet	1	-14.91299928	-4.788404404	-13.8148049	EMD2
AGCCAGCCACGGGTAA-1	singlet	1	-426.0231294	-167.8457769	-309.0075273	EMD2
AGCCAGCCAGATAAAC-1	singlet	2	-351.0774341	-302.6066133	-155.3227289	EMD2
AGCCAGCGTACTCAAC-1	singlet	2	-3118.171302	-2754.532145	-882.7029204	EMD2
AGCCAGCGTTGCATTG-1	singlet	0	-181.6649214	-441.7915634	-411.9003565	EMD2
AGCCAGCTCCTGGTCT-1	singlet	0	-1197.147339	-3686.983571	-3432.847678	EMD2
AGCCAGCTCCTGTTGC-1	singlet	1	-1432.163077	-468.0426703	-1170.843926	EMD2
AGCCAGCTCGTCTACC-1	singlet	2	-1736.493724	-1274.752166	-589.2011967	EMD2
AGCCAGCTCTACCACC-1	singlet	1	-4394.093758	-1299.728528	-3308.17076	EMD2
AGCCAGCTCTCACGAA-1	singlet	1	-6380.988577	-1771.288674	-4927.166674	EMD2
AGCCAGCTCTTACCAT-1	singlet	1	-280.5660376	-150.4053886	-246.5795752	EMD2
AGCCAGCTCTTCGCTG-1	singlet	1	-998.2666108	-306.3948503	-780.4218685	EMD2
AGCGATTAGATAACGT-1	singlet	2	-4050.66332	-3300.944404	-1438.006174	EMD2
AGCGATTAGATCACCT-1	singlet	2	-124.256118	-135.7269017	-48.78459078	EMD2
AGCGATTAGCGTTCCG-1	singlet	2	-1545.821322	-1341.913983	-483.4126653	EMD2
AGCGATTAGGACAAGA-1	singlet	1	-265.2515407	-68.11046698	-199.6451272	EMD2
AGCGATTAGGTTCTTG-1	singlet	0	-801.431183	-2452.618266	-2561.25131	EMD2
AGCGATTCACCGTCTT-1	singlet	1	-1270.299232	-498.7286651	-1078.39241	EMD2
AGCGATTCACGCGCTA-1	singlet	0	-1015.165866	-3322.018658	-2912.778689	EMD2
AGCGATTCAGGAGGTT-1	singlet	2	-1060.819043	-1233.671051	-404.9585993	EMD2

AGCGATTGTTACGATC-1	singlet	1	-1977.508359	-625.8259972	-1682.662943	EMD2
AGCGATTTCCCACAGG-1	singlet	1	-425.2343505	-155.5574513	-363.7556271	EMD2
AGCGATTTCCGTAGTA-1	singlet	1	-677.3801301	-238.4556268	-528.5094784	EMD2
AGCGCCAAGAGGCGGA-1	singlet	0	-603.5218885	-3330.836806	-2763.807849	EMD2
AGCGCCAAGGAGAGGC-1	singlet	1	-3274.207139	-918.8729936	-2653.800642	EMD2
AGCGCCACACAGTGTT-1	doublet	2/0	-3929.422498	-3922.343712	-2295.225862	EMD2
AGCGCCACATCGATGT-1	singlet	1	-1646.588073	-611.7335884	-1249.853339	EMD2
AGCGCCACATGCGGTC-1	singlet	2	-1912.561467	-1591.718783	-576.0998539	EMD2
AGCGCCAGTATGTCAC-1	singlet	1	-4155.032147	-1174.038229	-3040.473151	EMD2
AGCGCCAGTTTCACTT-1	singlet	2	-1033.253027	-1285.180418	-317.9092687	EMD2
AGCGCCATCACGATAC-1	singlet	0	-874.7559666	-2427.635374	-2512.62479	EMD2
AGCGCCATCGGTCAGC-1	singlet	0	-382.5709343	-1427.860985	-1240.188592	EMD2
AGCGCCATCTGAGTCA-1	singlet	0	-246.2700416	-869.4440221	-860.6310265	EMD2
AGCGCCATCTTTGCGC-1	singlet	1	-1729.533476	-533.1366114	-1516.136644	EMD2
AGCGCTGAGCCGAATG-1	singlet	1	-774.4879093	-232.1283269	-581.5612672	EMD2
AGCGCTGAGTCACTAC-1	singlet	0	-964.2734269	-3003.599273	-2805.049734	EMD2
AGCGCTGCAAAGGGTC-1	singlet	1	-4716.455913	-739.326463	-3085.102652	EMD2
AGCGCTGCAGAGGAAA-1	singlet	1	-2638.960803	-813.3153144	-2058.000526	EMD2
AGCGCTGCAGGACGAT-1	singlet	1	-77.87395364	-37.14121961	-69.23511651	EMD2
AGCGCTGCATCTCGTC-1	singlet	1	-815.6058483	-245.7527263	-547.8190508	EMD2
AGCGCTGCATGGTACT-1	singlet	2	-588.3517289	-488.0454629	-254.713629	EMD2
AGCGCTGGTACGATGG-1	singlet	1	-742.6725967	-231.5545071	-563.6522233	EMD2
AGCGCTGGTAGAATAC-1	singlet	2	-585.4814168	-450.3509224	-250.5497704	EMD2
AGCGCTGGTAGTAAGT-1	doublet	45293	-3928.995497	-2007.037756	-2337.03618	EMD2
AGCGCTGTCCGCACGA-1	singlet	1	-1691.341215	-504.6473686	-1274.171198	EMD2
AGCGTATAGACCACGA-1	singlet	1	-330.4792221	-115.8021538	-259.9846849	EMD2
AGCGTATAGCTTTCCC-1	singlet	1	-3595.217192	-1216.221313	-2867.5449	EMD2
AGCGTATCAAATGCTC-1	singlet	1	-707.9500892	-216.7196979	-477.7472668	EMD2
AGCGTATCAAGGCGTA-1	singlet	0	-124.8258393	-289.5476144	-276.1796808	EMD2
AGCGTATCACACAGAG-1	singlet	1	-909.9837706	-277.5032668	-626.9301892	EMD2
AGCGTATCAGGTACGA-1	singlet	2	-3865.997595	-3238.319032	-1272.652693	EMD2
AGCGTATCATAAGCAA-1	singlet	1	-463.5348882	-170.9128023	-453.1702195	EMD2
AGCGTATGTATGGTTC-1	singlet	2	-2240.485541	-1822.608128	-786.0933722	EMD2
AGCGTATGTCAACCAT-1	singlet	1	-1711.134411	-542.8314564	-1458.493338	EMD2
AGCGTATGTCACTCAA-1	singlet	1	-1515.240948	-442.8301598	-1340.379013	EMD2
AGCGTATGTCTCAAGT-1	singlet	2	-256.1381585	-285.1942693	-118.6596848	EMD2
AGCGTATGTTCACGAT-1	singlet	0	-101.5479507	-339.8288712	-269.1263628	EMD2
AGCGTATGTTGTGGCC-1	singlet	2	-2332.716799	-1893.399246	-724.3682845	EMD2
AGCGTATTCACCATGA-1	singlet	1	-1521.701465	-537.5713389	-1284.019058	EMD2
AGCGTATTCAGACCTA-1	singlet	1	-1710.914167	-502.4740753	-1385.989644	EMD2
AGCGTATTCCCATGGG-1	singlet	1	-1480.762803	-334.0290896	-1053.403843	EMD2
AGCGTATTCCTCCACA-1	singlet	1	-74.85341569	-30.21995037	-67.43756328	EMD2
AGCGTCGAGAGTACCG-1	singlet	0	-37.48121417	-170.676056	-123.4885761	EMD2
AGCGTCGAGGCTTAAA-1	singlet	1	-234.1675906	-74.34920007	-170.1811795	EMD2
AGCGTCGCACTACTTT-1	singlet	1	-273.6000334	-102.7544478	-223.3605024	EMD2
AGCGTCGCATTGAGGG-1	singlet	1	-185.1709274	-54.30165321	-108.0310096	EMD2
AGCTACACACAAATCC-1	singlet	1	-324.78699	-148.6669284	-280.778098	EMD2
AGCTACACACATGAAA-1	doublet	45323	-124.1142611	-67.92334371	-57.88160137	EMD2
AGCTACACATTACGGT-1	singlet	2	-2670.448595	-2513.910401	-909.8635002	EMD2
AGCTACACATTGGATC-1	singlet	1	-467.7809462	-195.6041182	-381.1301157	EMD2
AGCTACAGTCTTACAG-1	singlet	0	-608.1167919	-1962.001413	-1915.776019	EMD2
AGCTACAGTGGTCTTA-1	singlet	1	-578.1781172	-184.9410216	-499.8343484	EMD2
AGCTACATCCGTGGTG-1	singlet	0	-726.3926385	-1952.513441	-2031.663391	EMD2
AGCTACATCGATAACC-1	singlet	1	-2119.193895	-648.3317056	-1638.975432	EMD2
AGCTACATCGCGGACT-1	singlet	0	-980.5708516	-3108.159537	-2940.907427	EMD2
AGCTACATCGGCATAT-1	singlet	1	-343.7500495	-112.9233205	-280.7388479	EMD2
AGCTACATCTACCAGA-1	singlet	1	-1228.717317	-471.2196459	-1094.130495	EMD2
AGCTACATCTAGCCAA-1	singlet	0	-5.476411644	-9.797186844	-14.34920473	EMD2
AGCTACATCTGATTCT-1	singlet	2	-25.57135643	-20.39833824	-14.12712289	EMD2
AGCTCAACACGTACTA-1	singlet	0	-1804.851359	-6406.56153	-6211.092955	EMD2
AGCTCAAGTACGACTT-1	singlet	1	-136.8925931	-50.00895747	-120.4907051	EMD2
AGCTCAAGTCTTCCGT-1	singlet	1	-17.35133732	-4.221665438	-16.48945382	EMD2
AGCTCAAGTGGTCCGT-1	singlet	1	-1989.44406	-630.8996056	-1682.784767	EMD2
AGCTCAATCACTGTCC-1	singlet	1	-2659.916322	-967.2726138	-2134.514161	EMD2
AGCTCAATCAGCTTCC-1	singlet	1	-2109.081145	-757.480821	-1843.938758	EMD2
AGCTCAATCGTCAGAT-1	singlet	1	-1288.258156	-425.4058808	-1175.951007	EMD2
AGCTCAATCTGCACCT-1	singlet	1	-346.5355889	-150.6479944	-296.3401461	EMD2
AGCTTCCAGGATTTCC-1	singlet	2	-4434.739315	-3684.32256	-1416.944482	EMD2
AGCTTCCAGGCCCGTT-1	singlet	2	-68.72136781	-76.58538692	-26.17433977	EMD2
AGCTTCCCAATAAGGT-1	singlet	0	-260.8880313	-955.3274684	-698.3701855	EMD2
AGCTTCCCAGAATTCC-1	singlet	2	-348.0864132	-361.8960105	-120.3795023	EMD2
AGCTTCCCAGCGTATT-1	singlet	1	-123.4672153	-32.58310318	-66.48248548	EMD2
AGCTTCCCAGGGACTA-1	singlet	1	-481.0746933	-156.7259905	-398.512929	EMD2
AGCTTCCCATTCAGCA-1	singlet	1	-372.9489213	-157.7798377	-314.5299748	EMD2
AGCTTCCGTGCGTGCT-1	singlet	1	-1206.550091	-409.5473331	-923.2121273	EMD2
AGCTTCCGTGTCCTAA-1	singlet	2	-2544.020966	-2319.161107	-870.5137564	EMD2
AGCTTCCTCGAGTTGT-1	singlet	2	-637.7062416	-394.6020369	-195.5423119	EMD2
AGCTTCCTCTACCAGA-1	singlet	1	-660.3707085	-213.769584	-479.2784556	EMD2
AGGAAATAGAAGGCTC-1	singlet	1	-6174.052409	-1837.570985	-5156.196989	EMD2
AGGAAATAGAGTGACC-1	singlet	1	-1527.80921	-492.0613274	-1203.008371	EMD2
AGGAAATAGGGCGAAG-1	singlet	2	-3706.111577	-2973.513791	-1101.164441	EMD2
AGGAAATAGGTGGTTG-1	singlet	1	-379.7539642	-127.2078232	-296.5804793	EMD2
AGGAAATCAAATCCCA-1	singlet	1	-968.7992936	-308.3572076	-660.4542958	EMD2
AGGAAATTCGCCTAGG-1	singlet	0	-582.1910979	-1881.3137	-1706.927293	EMD2
AGGAATAAGAAGTATC-1	singlet	1	-354.8789375	-109.6146429	-302.8607284	EMD2
AGGAATAAGCATCAGG-1	singlet	1	-1492.36182	-506.37308	-1113.949679	EMD2
AGGAATAAGGCAGCTA-1	singlet	1	-1927.994291	-577.4734716	-1690.582046	EMD2
AGGAATAGTTGCGGAA-1	singlet	1	-935.8312441	-344.088492	-766.5407846	EMD2
AGGAATATCAGTAGGG-1	doublet	0/1	-2339.485227	-2739.785615	-3569.954934	EMD2
AGGAATATCCTAGAGT-1	singlet	0	-437.6864797	-1558.440546	-1343.637256	EMD2
AGGAATATCGCCTTGT-1	singlet	1	-464.0928164	-158.4383751	-365.9030861	EMD2
AGGAATATCTTGAACG-1	singlet	2	-1150.009925	-1182.383701	-317.8995154	EMD2
AGGACGAAGAAACCCG-1	singlet	2	-164.2239506	-139.4032544	-80.16503617	EMD2
AGGACGAAGACATAGT-1	singlet	1	-1101.594996	-371.2803954	-894.5274432	EMD2
AGGACGAAGCTCGACC-1	singlet	2	-3314.103036	-2785.193373	-1346.910839	EMD2
AGGACGAAGGTGGGTT-1	singlet	2	-3846.349873	-3263.571937	-1286.224377	EMD2
AGGACGACAATTTCCT-1	singlet	1	-445.5268189	-168.3122272	-395.9030788	EMD2
AGGACGACAGACTCTA-1	singlet	1	-1995.638618	-724.0574645	-1670.922469	EMD2
AGGACGACAGTTAGGG-1	doublet	0/1	-271.7251743	-341.7471628	-397.9143947	EMD2
AGGACGATCCTGGGAC-1	singlet	1	-1992.357236	-545.606876	-1375.684702	EMD2
AGGACGATCGTCTCAC-1	singlet	0	-1238.255175	-5307.429564	-5037.566037	EMD2
AGGACTTCACTTGAGT-1	singlet	2	-874.4227482	-708.7630496	-288.5190061	EMD2
AGGACTTCAGTTCCAA-1	singlet	1	-1284.53106	-444.5550898	-1147.037999	EMD2
AGGACTTCATAGAAAC-1	singlet	1	-290.6107703	-138.0688124	-271.0976763	EMD2
AGGACTTGTCGGTGTC-1	singlet	2	-2455.031917	-2210.676071	-835.7298814	EMD2
AGGACTTGTCTCAGAT-1	singlet	0	-916.8974576	-2546.141199	-2804.053773	EMD2
AGGACTTGTTCAAGTC-1	singlet	1	-3781.962534	-1108.600179	-2983.359378	EMD2
AGGACTTGTTCATCTT-1	singlet	1	-361.2628162	-146.7012926	-287.6765456	EMD2
AGGACTTGTTCCTAGA-1	singlet	1	-808.9840312	-293.7094147	-642.0104954	EMD2
AGGACTTTCCATGATG-1	singlet	0	-89.02022285	-323.7143825	-288.054948	EMD2

AGGACTTTCGACATCA-1	singlet	2	-1026.569579	-794.7452123	-294.7110939	EMD2
AGGAGGTAGAGGTTTA-1	singlet	1	-2408.845629	-768.6510635	-2031.437612	EMD2
AGGAGGTAGCCGTTAT-1	singlet	1	-5295.860805	-1896.480807	-4495.251411	EMD2
AGGAGGTAGTTGGACG-1	singlet	1	-366.238958	-148.4589031	-325.4562666	EMD2
AGGAGGTAGTTTGCTG-1	singlet	2	-517.2532855	-480.9437392	-222.9773514	EMD2
AGGAGGTCAAGCGAGT-1	singlet	1	-1794.855924	-502.1787386	-1481.658497	EMD2
AGGAGGTCACCAGCTG-1	singlet	2	-982.9362664	-992.2095522	-347.9778655	EMD2
AGGAGGTCAGGATTCT-1	singlet	0	-1875.655946	-5849.081484	-5540.185822	EMD2
AGGAGGTCATACAGAA-1	singlet	2	-1752.159668	-1400.215751	-703.3746286	EMD2
AGGAGGTCATACTTTC-1	singlet	2	-83.44252868	-111.1237154	-38.59308323	EMD2
AGGAGGTCATATGAAG-1	singlet	1	-675.5504273	-209.6319195	-557.3724528	EMD2
AGGAGGTCATCATGAC-1	singlet	2	-1662.842732	-1544.515993	-527.6750565	EMD2
AGGAGGTCATCCGTGG-1	singlet	1	-19.40842307	-9.688336608	-23.76884905	EMD2
AGGAGGTTCTTTGGAG-1	singlet	2	-547.7908568	-527.9240852	-227.2160105	EMD2
AGGATAAAGGGCGAGA-1	singlet	1	-437.8367312	-155.8747346	-325.1056039	EMD2
AGGATAAAGTAGGTTA-1	singlet	2	-3395.927263	-2992.073001	-1032.039822	EMD2
AGGATAACACGACGAA-1	singlet	1	-332.8310637	-136.5660768	-266.9582619	EMD2
AGGATAACAGTAACAA-1	singlet	1	-8.691160321	-1.739422561	-7.269099789	EMD2
AGGATAAGTACTAAGA-1	singlet	1	-1209.261244	-432.0997219	-1058.149617	EMD2
AGGATAATCTTGGTCC-1	singlet	0	-178.637653	-431.7100172	-415.2834251	EMD2
AGGATCTAGGTCATTC-1	singlet	2	-741.1217353	-766.041671	-288.81257	EMD2
AGGATCTAGTCCTGCG-1	singlet	1	-1431.681831	-383.5651144	-1100.984126	EMD2
AGGATCTAGTTGCCTA-1	singlet	0	-275.2594469	-875.6217811	-845.3076604	EMD2
AGGATCTCAAAGTGTA-1	singlet	0	-645.769374	-2422.353181	-1973.343037	EMD2
AGGATCTCAGCATGCC-1	singlet	1	-929.5852403	-275.5182023	-620.534881	EMD2
AGGATCTCATCCCACT-1	singlet	2	-5477.649506	-4237.18622	-1739.003546	EMD2
AGGATCTCATTGCTTT-1	singlet	1	-1960.051106	-668.4178844	-1797.945385	EMD2
AGGATCTTCACTTATC-1	singlet	0	-26.12903623	-76.86638936	-88.04329326	EMD2
AGGATCTTCAGTGTCA-1	singlet	0	-161.0397974	-551.4218326	-509.3574507	EMD2
AGGCATTAGAGAGCGG-1	singlet	2	-591.3717826	-582.2504624	-271.0883168	EMD2
AGGCATTAGTTCTACG-1	singlet	2	-796.2789666	-685.5312847	-282.6149597	EMD2
AGGCATTCACATACTG-1	singlet	1	-1795.072037	-571.2684595	-1469.558826	EMD2
AGGCATTCATGGGATG-1	singlet	1	-2324.197125	-632.9841487	-1840.621707	EMD2
AGGCATTCATGTCAGT-1	singlet	0	-402.242438	-1150.78043	-1093.37473	EMD2
AGGCATTGTTCTCGCT-1	singlet	1	-202.3718185	-72.30791377	-165.8241687	EMD2
AGGCATTTCTGCTGAA-1	singlet	1	-2499.082783	-893.6486642	-2141.729684	EMD2
AGGCCACAGCCGTCGT-1	singlet	2	-2182.214616	-1927.557408	-753.4146916	EMD2
AGGCCACAGCTGACTT-1	singlet	1	-700.2993257	-236.9516926	-499.6651211	EMD2
AGGCCACGTCATGGCC-1	singlet	1	-8710.627414	-2529.62468	-6573.553007	EMD2
AGGCCACGTGGAGAAA-1	singlet	2	-5150.574829	-4062.016983	-1408.727594	EMD2
AGGCCACGTTGCATGT-1	singlet	1	-2101.676639	-636.9342788	-1819.570986	EMD2
AGGCCACTCACTTTGT-1	singlet	1	-4982.276174	-1064.877057	-4118.042914	EMD2
AGGCCACTCCATCTGC-1	singlet	0	-217.2735753	-672.9554944	-726.2235853	EMD2
AGGCTGCAGAATCGTA-1	singlet	0	-570.45969	-1842.267302	-1709.554865	EMD2
AGGCTGCAGATACGAT-1	singlet	2	-2120.858955	-1654.933243	-694.9112927	EMD2
AGGCTGCAGATCCGAG-1	singlet	2	-1877.046031	-1539.140465	-621.9431809	EMD2
AGGCTGCAGCACGTCC-1	singlet	2	-294.4659195	-137.7590974	-76.92723907	EMD2
AGGCTGCAGCTATCCA-1	singlet	0	-1063.807039	-3153.151478	-2929.566547	EMD2
AGGCTGCAGGGCTTCC-1	singlet	1	-2845.808981	-895.0277901	-2249.814738	EMD2
AGGCTGCAGGTGCTAG-1	singlet	1	-2595.856576	-846.4591041	-2090.128264	EMD2
AGGCTGCAGGTTACAA-1	singlet	1	-248.7417281	-110.241774	-237.6220594	EMD2
AGGCTGCAGGTTGAGC-1	unassigned	2	-39.06647773	-17.03451398	-16.43761125	EMD2
AGGCTGCCACACACGC-1	singlet	0	-348.637761	-1136.799765	-1030.929387	EMD2
AGGCTGCCACATACGT-1	singlet	0	-459.93913	-1276.207725	-1163.475359	EMD2
AGGCTGCCACTGCTTC-1	singlet	1	-308.4231609	-96.37247764	-229.5397407	EMD2
AGGCTGCCAGCTCATA-1	singlet	1	-1546.584437	-483.6590049	-1530.636916	EMD2
AGGCTGCCAGGTCAAG-1	singlet	1	-2433.631569	-923.6900829	-2003.508544	EMD2
AGGCTGCGTACCCGCA-1	singlet	1	-777.6723038	-245.6519707	-526.8180983	EMD2
AGGCTGCGTATACGGG-1	singlet	0	-184.8425001	-502.3234124	-511.2953084	EMD2
AGGCTGCGTGTCCGGT-1	singlet	1	-384.8809986	-170.2515818	-363.7761882	EMD2
AGGCTGCGTTCAAAGA-1	singlet	1	-341.3457671	-137.5332846	-286.1149783	EMD2
AGGCTGCTCCACGTGG-1	singlet	1	-325.0622446	-110.2017287	-262.8883277	EMD2
AGGCTGCTCTCAAAGC-1	singlet	2	-3109.771589	-2497.698178	-1143.329908	EMD2
AGGGAGTCAGAAACCG-1	singlet	1	-2925.196776	-943.8215323	-2304.838709	EMD2
AGGGAGTCAGACCCGT-1	singlet	1	-1266.926664	-426.0026402	-997.8462146	EMD2
AGGGAGTCAGATTCGT-1	singlet	0	-204.9999336	-620.0631629	-577.7948264	EMD2
AGGGAGTCAGTAACCT-1	singlet	1	-5215.192138	-1478.492633	-4062.064916	EMD2
AGGGAGTCATCGGAGA-1	singlet	0	-1001.278888	-2845.445064	-2716.589424	EMD2
AGGGAGTGTATTGACC-1	singlet	1	-256.0654564	-126.91155	-214.3501219	EMD2
AGGGAGTGTGAGAGGG-1	singlet	2	-1162.174151	-1111.768539	-420.0042765	EMD2
AGGGAGTGTTAAACAG-1	singlet	1	-3202.862356	-796.2429883	-2468.75855	EMD2
AGGGAGTTCAGGACAG-1	singlet	1	-396.5467076	-147.7089053	-329.3942437	EMD2
AGGGAGTTCGCTGTTC-1	singlet	2	-249.4241553	-192.6703631	-74.07734594	EMD2
AGGGAGTTCGGAGCAA-1	singlet	1	-563.8511527	-202.6748783	-452.005442	EMD2
AGGGAGTTCTCAACGA-1	singlet	0	-1268.757942	-4807.745728	-4892.450658	EMD2
AGGGAGTTCTGACCCT-1	singlet	1	-4220.27022	-1152.054964	-3400.451127	EMD2
AGGGCCTCACGTAGAG-1	singlet	1	-1649.61896	-412.9720053	-1247.290805	EMD2
AGGGCCTCAGCACGAA-1	singlet	0	-63.71046367	-218.1906429	-204.9674007	EMD2
AGGGCCTGTTAAGTCC-1	singlet	2	-1797.518692	-1484.433619	-514.3778438	EMD2
AGGGCCTTCATAGAGA-1	singlet	1	-1399.162293	-400.9552626	-1049.366987	EMD2
AGGGCCTTCGGTTGTA-1	singlet	2	-2161.43255	-1583.919695	-689.6479504	EMD2
AGGGCCTTCTCCTGCA-1	singlet	2	-542.7486852	-599.8083036	-220.5880608	EMD2
AGGGCTCAGCAATTAG-1	singlet	1	-603.5858247	-192.6866307	-418.445821	EMD2
AGGGCTCAGGAAAGTG-1	singlet	1	-3673.691611	-987.6906328	-2807.626082	EMD2
AGGGCTCAGTTCGCAT-1	doublet	1/0	-2143.266665	-1099.809761	-1791.134011	EMD2
AGGGCTCCAAAGGTTA-1	singlet	1	-767.1668345	-251.5119406	-530.0799798	EMD2
AGGGCTCCAATTGCAC-1	singlet	1	-2415.709689	-745.2914356	-1805.698001	EMD2
AGGGCTCCAGCGATTT-1	singlet	0	-233.8954379	-867.7090125	-655.3753833	EMD2
AGGGCTCCAGTCCGTG-1	singlet	1	-1070.308309	-354.1210402	-765.314757	EMD2
AGGGCTCCATTAGGCT-1	singlet	1	-1207.09621	-450.9601996	-1051.371196	EMD2
AGGGCTCGTCCAGCGT-1	singlet	0	-51.53685743	-125.979307	-146.5183364	EMD2
AGGGCTCGTGGCATCC-1	singlet	0	-841.6296153	-2596.478866	-2512.124075	EMD2
AGGGCTCTCCGAAATC-1	singlet	2	-1794.265381	-1384.834768	-565.7414747	EMD2
AGGGTCCAGCGTGAAC-1	singlet	1	-2218.979349	-630.7893707	-1759.276112	EMD2
AGGGTCCAGGTCACCC-1	singlet	1	-646.0904231	-252.9677774	-582.618574	EMD2
AGGGTCCAGTGAGGTC-1	singlet	1	-1763.009466	-492.8550369	-1410.336313	EMD2
AGGGTCCAGTGCCAGA-1	singlet	0	-1090.035981	-3355.450358	-3194.84224	EMD2
AGGGTCCCAATCTAGC-1	singlet	1	-1265.460208	-436.4900246	-1178.751288	EMD2
AGGGTCCCAATTGAAG-1	singlet	2	-427.0960056	-341.0229741	-103.0654219	EMD2
AGGGTCCCACAGCTTA-1	singlet	0	-1377.999324	-4463.826412	-4204.381628	EMD2
AGGGTCCCACGTGTGC-1	singlet	1	-1515.951448	-333.6700146	-916.9258888	EMD2
AGGGTCCGTCTAGATC-1	singlet	2	-1251.61313	-1012.805925	-389.1915853	EMD2
AGGGTCCGTTGCAACT-1	singlet	1	-1108.337417	-398.0376825	-922.5288334	EMD2
AGGGTCCTCGCGATCG-1	singlet	1	-1176.445446	-448.6415569	-1037.680635	EMD2
AGGGTCCTCTTCTTCC-1	singlet	1	-155.1253601	-58.63846979	-149.9276717	EMD2
AGGGTGAAGTAAACAC-1	singlet	1	-94.87199084	-31.08066577	-47.44826799	EMD2
AGGGTGAAGTGCGCTC-1	singlet	2	-527.1249999	-512.2127591	-171.0592266	EMD2
AGGGTGACAAACGTGG-1	singlet	1	-1369.320484	-436.1693326	-1024.474736	EMD2
AGGGTGACAAATGCGG-1	singlet	1	-451.2584383	-164.1629318	-332.4726369	EMD2
AGGGTGACAGAGCGTA-1	singlet	1	-106.3143145	-57.27674768	-118.8180513	EMD2

AGGGTGAGTAGTTCCA-1	singlet	0	-940.2565625	-3222.878985	-3096.651622	EMD2
AGGGTGAGTCACGACC-1	singlet	1	-1761.749176	-385.17274	-1898.695739	EMD2
AGGGTGAGTGTTACTG-1	singlet	1	-96.68315465	-31.53962108	-68.93282801	EMD2
AGGGTGAGTTCATCGA-1	singlet	2	-3822.213108	-3239.861819	-1392.550167	EMD2
AGGGTGATCGTGCGAC-1	singlet	1	-209.0334751	-79.24672861	-180.907332	EMD2
AGGGTTTAGCCATTGT-1	singlet	1	-194.3389518	-80.5529434	-196.1823312	EMD2
AGGGTTTAGCTATCCA-1	singlet	1	-2936.573076	-857.6076412	-2454.201865	EMD2
AGGGTTTAGTACTGTC-1	singlet	1	-1355.109149	-474.6790889	-1159.108349	EMD2
AGGGTTTCACCGTCGA-1	singlet	2	-4176.35408	-3275.023605	-1507.96512	EMD2
AGGGTTTCAGAGGACT-1	singlet	1	-1159.085711	-431.3314185	-1037.355871	EMD2
AGGGTTTCATGAGAAT-1	singlet	1	-785.7495652	-293.0572486	-721.7185634	EMD2
AGGGTTTGTCAATCTG-1	singlet	1	-140.6463753	-48.69337286	-121.3052108	EMD2
AGGGTTTGTCATAGTC-1	singlet	1	-392.7466924	-151.5057933	-289.0869274	EMD2
AGGGTTTGTGATCGTT-1	singlet	1	-302.3334461	-128.3581515	-232.0588643	EMD2
AGGGTTTGTTCTAACG-1	singlet	1	-1213.344893	-423.5922201	-971.3927839	EMD2
AGGGTTTTCGAAGCCC-1	singlet	2	-471.5395356	-301.3493617	-126.716492	EMD2
AGGGTTTTCTCGTGGG-1	singlet	1	-1054.447393	-397.1424391	-894.546556	EMD2
AGGGTTTTCTCTATAC-1	singlet	1	-374.5621707	-184.3411043	-377.5777243	EMD2
AGGTAGGAGATTCGCT-1	singlet	0	-832.0839943	-2909.578807	-2653.696648	EMD2
AGGTAGGAGATTGACA-1	singlet	2	-939.3778755	-913.9964705	-369.9292452	EMD2
AGGTAGGAGCTCGCAC-1	doublet	2/0	-5264.797601	-6166.785801	-3672.545164	EMD2
AGGTAGGCACGAAGAC-1	singlet	1	-3304.558736	-998.4029917	-2572.834188	EMD2
AGGTAGGGTGTGTCGC-1	singlet	1	-2269.017166	-735.4250521	-1840.508908	EMD2
AGGTAGGGTTGACTGT-1	singlet	1	-4468.962294	-1545.047492	-3609.673077	EMD2
AGGTAGGTCCCTGTTG-1	singlet	2	-105.2308531	-102.7581234	-50.44802654	EMD2
AGGTAGGTCGCATTAG-1	singlet	0	-784.6541164	-3311.491923	-3088.025003	EMD2
AGGTAGGTCTACCACC-1	singlet	2	-260.4409164	-186.2298611	-98.5285365	EMD2
AGGTCATAGAGGTCGT-1	singlet	2	-1116.545897	-1067.950076	-408.7558215	EMD2
AGGTCATAGCGTCTGC-1	singlet	2	-2698.982776	-2032.211818	-888.3580143	EMD2
AGGTCATAGGCCCGTT-1	singlet	2	-706.6155349	-623.9466567	-257.7954701	EMD2
AGGTCATAGGTCGCCT-1	singlet	0	-1578.20529	-4156.252801	-3936.774832	EMD2
AGGTCATCAATCAGCT-1	singlet	1	-756.060096	-223.7114504	-595.1125067	EMD2
AGGTCATCAGCTGTTA-1	singlet	1	-127.7690973	-39.17636029	-91.84357764	EMD2
AGGTCATCAGGATCTT-1	singlet	0	-510.1293141	-1608.325674	-1594.457027	EMD2
AGGTCATCATGGCTAT-1	singlet	2	-718.5454499	-749.5948316	-313.3769081	EMD2
AGGTCATGTAAGCTCT-1	singlet	1	-2218.610221	-710.2218226	-1649.662465	EMD2
AGGTCATGTACCGCGT-1	singlet	2	-564.5025972	-583.0904478	-189.6953791	EMD2
AGGTCATGTCCCGCAA-1	singlet	0	-281.9369075	-838.3263465	-841.3082311	EMD2
AGGTCATGTCCTACGG-1	singlet	1	-152.0995175	-66.18994394	-134.808984	EMD2
AGGTCATTCCTAGCTC-1	singlet	1	-306.0030937	-120.1278698	-262.5993884	EMD2
AGGTCATTCGGAAGGT-1	singlet	1	-1040.428611	-304.1760705	-764.7297702	EMD2
AGGTCATTCTCCCAAC-1	singlet	1	-36.67400294	-6.703449782	-26.63023679	EMD2
AGGTCATTCTGTCTCG-1	singlet	2	-2162.22948	-2325.666224	-1140.831061	EMD2
AGGTCTAAGAAGTATC-1	singlet	2	-3987.337262	-3205.856079	-1311.107563	EMD2
AGGTCTAAGAGTTGAT-1	singlet	1	-1489.695995	-509.4734095	-1293.230388	EMD2
AGGTCTAAGTATGATG-1	singlet	2	-7357.668361	-5992.635135	-2208.703073	EMD2
AGGTCTAAGTTGAATG-1	singlet	2	-10.09529011	-8.008091791	-3.621971633	EMD2
AGGTCTACAATGCAAA-1	singlet	1	-1585.511207	-536.3001101	-1302.955895	EMD2
AGGTCTACACCAGCGT-1	singlet	2	-2327.557825	-1860.028867	-797.5448384	EMD2
AGGTCTACATACAGCT-1	singlet	1	-50.10949776	-13.56447522	-33.96257377	EMD2
AGGTCTAGTCTGTCAA-1	singlet	1	-3488.976797	-867.6825542	-2392.997858	EMD2
AGGTCTATCGGACGTC-1	singlet	0	-785.5512708	-2724.95943	-2577.294419	EMD2
AGGTCTATCGGTCGAC-1	singlet	2	-4344.734655	-3810.716356	-1450.196771	EMD2
AGGTGTTAGCCATTGT-1	unassigned	2/0	-10.89062359	-11.71056218	-7.100325386	EMD2
AGGTGTTAGCTCATAC-1	singlet	1	-423.9005831	-194.4066599	-375.625409	EMD2
AGGTGTTCAAGCACAG-1	singlet	1	-555.8833001	-218.438517	-436.1187098	EMD2
AGGTGTTCACCTTCCA-1	singlet	1	-1216.567857	-452.7025263	-1001.547724	EMD2
AGGTGTTGTACGTTCA-1	singlet	0	-1660.390196	-3681.623039	-3835.560227	EMD2
AGGTGTTGTCGGTAAG-1	singlet	1	-1571.492764	-326.4513994	-928.8918582	EMD2
AGGTGTTTCCATCACC-1	singlet	1	-3134.723927	-978.6148339	-2532.667861	EMD2
AGGTGTTTCCTAAGTG-1	singlet	0	-230.6972332	-770.3907785	-670.3639095	EMD2
AGGTGTTTCTCCTGTG-1	singlet	1	-3403.704722	-979.1410774	-2643.575358	EMD2
AGGTGTTTCTCGAGTA-1	singlet	1	-1192.550333	-380.5157602	-1061.168601	EMD2
AGGTTACAGAAGGTAG-1	singlet	1	-1601.94475	-503.367099	-1351.00748	EMD2
AGGTTACAGAGCAGCT-1	singlet	1	-2828.373204	-713.0913291	-2571.012655	EMD2
AGGTTACAGGATTTAG-1	singlet	1	-3639.668798	-1226.684314	-3002.029329	EMD2
AGGTTACCAATTTCTC-1	singlet	0	-344.7004157	-1066.734648	-918.0440515	EMD2
AGGTTACCACGACTAT-1	singlet	2	-365.658207	-405.5408583	-158.0444932	EMD2
AGGTTACCACGAGGAT-1	singlet	1	-2960.771877	-958.078579	-2337.174786	EMD2
AGGTTACGTAGGTACG-1	singlet	1	-1224.737666	-367.0846648	-986.249738	EMD2
AGGTTACTCAATCCGA-1	singlet	1	-6913.140478	-2025.164811	-5625.99477	EMD2
AGGTTACTCAGCTTCC-1	singlet	1	-2694.723556	-698.5162822	-1936.28179	EMD2
AGGTTGTAGACCATGG-1	singlet	1	-3707.294252	-941.8813332	-2679.724095	EMD2
AGGTTGTAGAGAACCC-1	singlet	1	-3656.356359	-1088.505997	-2717.368838	EMD2
AGGTTGTAGTAGGATT-1	singlet	0	-384.0658821	-968.4492398	-962.8897361	EMD2
AGGTTGTAGTGGAATT-1	singlet	0	-1112.104299	-4262.531501	-4272.829453	EMD2
AGGTTGTCACCAGTTA-1	singlet	2	-834.9483785	-797.1379392	-271.9793855	EMD2
AGGTTGTCACTCGATA-1	singlet	2	-8659.356043	-6792.815978	-2626.501238	EMD2
AGGTTGTGTACTGCGC-1	singlet	0	-491.3789573	-1771.487484	-1732.397824	EMD2
AGGTTGTGTTCAACGT-1	singlet	1	-733.527403	-211.7693935	-616.0802167	EMD2
AGGTTGTTCCACGGGT-1	singlet	1	-163.0371409	-75.81999444	-142.713316	EMD2
AGGTTGTTCCAGCTCT-1	singlet	0	-230.2408278	-868.2377647	-683.7183589	EMD2
AGGTTGTTCCCTCTAG-1	singlet	1	-471.0914741	-179.942068	-384.7389591	EMD2
AGGTTGTTCGGCATAT-1	singlet	2	-1655.53934	-1413.969444	-587.9630036	EMD2
AGTAACCAGAGTGTGC-1	singlet	2	-962.2540396	-862.6694351	-338.9105548	EMD2
AGTAACCAGATGATTG-1	singlet	0	-1584.006107	-5209.33909	-5261.931036	EMD2
AGTAACCCAAATCGTC-1	singlet	1	-813.0165326	-276.4563843	-476.7126812	EMD2
AGTAACCCACATCCCT-1	singlet	1	-414.5082624	-140.7258585	-305.7716542	EMD2
AGTAACCGTGCAATAA-1	singlet	1	-622.8212897	-248.861267	-560.3157874	EMD2
AGTAACCTCGTCGACG-1	doublet	0/2	-503.4391162	-1104.522398	-742.5854876	EMD2
AGTACCACAAACTAGA-1	singlet	1	-595.8104287	-201.7421644	-474.1897648	EMD2
AGTACCACAATTGTGC-1	singlet	1	-1592.728849	-554.7024508	-1391.580345	EMD2
AGTACCACACAAAGCG-1	doublet	45323	-1258.782354	-950.2696802	-598.1548033	EMD2
AGTACCACACTGCATA-1	singlet	1	-295.694229	-130.2470137	-299.3200031	EMD2
AGTACCACATTCTGTT-1	singlet	0	-306.4299187	-936.359874	-819.6168829	EMD2
AGTACCAGTAACGCGA-1	singlet	2	-1484.877267	-1310.925708	-557.5607358	EMD2
AGTACCAGTCTGATAC-1	singlet	1	-798.7554183	-276.6045811	-532.2227855	EMD2
AGTACCAGTGAAAGTT-1	singlet	1	-342.5751169	-130.1577945	-289.2980103	EMD2
AGTACCAGTGGACTAG-1	singlet	1	-501.7121786	-215.5588099	-451.3268552	EMD2
AGTACCATCATTGCTT-1	singlet	1	-479.2354879	-184.7281671	-359.7817931	EMD2
AGTACCATCCAGTTCC-1	singlet	1	-1515.304789	-519.0076094	-1380.185171	EMD2
AGTACCATCGTCGACG-1	singlet	0	-695.9394254	-2116.073104	-2017.214552	EMD2
AGTACTGAGCCTTTGA-1	doublet	45293	-470.2284792	-195.3215788	-315.5068579	EMD2
AGTACTGAGGTTGTTC-1	singlet	0	-248.9359044	-915.7153125	-873.7139203	EMD2
AGTACTGCAAATGGAT-1	singlet	1	-482.6815984	-186.6595576	-428.6619001	EMD2
AGTACTGCAACTCATG-1	singlet	2	-245.7550369	-199.7344713	-86.01968963	EMD2
AGTACTGCACGATTCA-1	unassigned	45293	-19.31107506	-4.630154304	-6.450837767	EMD2
AGTACTGGTAGCTGCC-1	singlet	1	-553.8288712	-259.6069084	-499.2308639	EMD2
AGTACTGGTGGAACAC-1	unassigned	0/1	-2.485714578	-2.768092297	-3.112061998	EMD2
AGTACTGGTTGGGTAG-1	singlet	0	-1478.542864	-4599.876672	-4426.699663	EMD2

AGTACTGTCCTTGAAG-1	singlet	2	-3875.929464	-3104.021465	-1308.182142	EMD2
AGTACTGTCGGATAAA-1	singlet	0	-86.11183017	-285.0377958	-247.9828685	EMD2
AGTACTGTCTGCGTCT-1	singlet	2	-1357.082256	-1142.25228	-452.9085143	EMD2
AGTAGCTAGATGTTGA-1	singlet	1	-368.396162	-129.2519642	-295.4188136	EMD2
AGTAGCTAGGTCCCGT-1	singlet	1	-359.0350272	-137.3889399	-305.9686833	EMD2
AGTAGCTCAGAGAAAG-1	singlet	0	-311.4411816	-1014.092778	-848.1409785	EMD2
AGTAGCTCAGCTTTCC-1	singlet	1	-462.0617865	-165.3104166	-443.1172971	EMD2
AGTAGCTGTACGTTCA-1	singlet	1	-1567.959618	-379.5142956	-1534.637669	EMD2
AGTAGCTGTAGCGCCT-1	singlet	0	-211.6804789	-568.2924857	-557.6766847	EMD2
AGTAGCTGTATGCTTG-1	singlet	0	-712.183752	-2559.372341	-2369.90131	EMD2
AGTAGCTGTCGGTACC-1	singlet	1	-277.6347747	-93.75447136	-218.2853747	EMD2
AGTAGCTGTGATTGGG-1	doublet	45293	-712.1885232	-347.0535401	-684.2376569	EMD2
AGTAGCTTCCTGCTAC-1	singlet	0	-1399.646379	-5173.315255	-4932.219447	EMD2
AGTAGCTTCGCAATTG-1	singlet	1	-520.6231068	-209.8792221	-444.2388798	EMD2
AGTAGCTTCTTAGTTC-1	singlet	2	-313.2643265	-244.4143086	-102.1594445	EMD2
AGTAGTCAGAAGTCAT-1	singlet	1	-4102.116357	-1163.429568	-3415.936191	EMD2
AGTAGTCAGACCAAAT-1	singlet	2	-4694.826413	-3898.048511	-1611.698694	EMD2
AGTAGTCAGACGGTCA-1	singlet	1	-354.7087315	-145.7215165	-288.865045	EMD2
AGTAGTCAGCAGCGAT-1	singlet	1	-343.286024	-128.0071638	-264.3168727	EMD2
AGTAGTCAGGGCTTCC-1	singlet	2	-390.9263547	-270.1368794	-153.4207765	EMD2
AGTAGTCAGTCTGTAC-1	singlet	1	-1423.332798	-430.469705	-982.3067675	EMD2
AGTAGTCCAAACTCGT-1	doublet	1/0	-969.6980756	-526.3111974	-949.9867125	EMD2
AGTAGTCGTTAGGACG-1	singlet	1	-614.1760773	-226.9482764	-506.5276593	EMD2
AGTAGTCGTTCAGCTA-1	singlet	2	-1855.879323	-1449.148099	-412.528661	EMD2
AGTAGTCGTTCTGACA-1	singlet	1	-906.989772	-271.0759361	-667.1437315	EMD2
AGTAGTCTCACATACG-1	singlet	1	-677.3376521	-201.3197548	-489.0937694	EMD2
AGTAGTCTCCATACTT-1	singlet	2	-1237.715063	-1224.270759	-456.4676359	EMD2
AGTAGTCTCCGACAGC-1	singlet	2	-3557.340387	-2751.752689	-1065.146602	EMD2
AGTAGTCTCTGGGTCG-1	singlet	2	-495.6103108	-421.9969894	-201.8647166	EMD2
AGTCAACAGACGCTCC-1	singlet	0	-344.5927775	-966.4711856	-830.9987357	EMD2
AGTCAACAGCGATTCT-1	singlet	1	-503.9432761	-203.8281473	-378.5474608	EMD2
AGTCAACAGGAGGCAG-1	singlet	0	-686.7003061	-2125.651197	-2136.294592	EMD2
AGTCAACAGGCAGGGA-1	singlet	0	-1630.931436	-5454.948283	-5274.160953	EMD2
AGTCAACAGGTTCATC-1	singlet	1	-2140.375582	-657.3522239	-1845.043939	EMD2
AGTCAACAGTCTTCCC-1	singlet	1	-468.8384087	-171.505275	-424.9095621	EMD2
AGTCAACCAACCTAAC-1	singlet	0	-1191.259569	-4255.109593	-4315.178785	EMD2
AGTCAACCAGCTGTAT-1	singlet	1	-1807.876971	-593.2847467	-1536.494148	EMD2
AGTCAACCAGCTTTGA-1	singlet	1	-4899.96125	-1414.222132	-3663.901986	EMD2
AGTCAACCAGTGTGCC-1	singlet	0	-808.1496646	-2007.430005	-1978.407932	EMD2
AGTCAACCATCTGTTT-1	singlet	1	-464.1658422	-181.3390018	-429.6814755	EMD2
AGTCAACCATTCTTCA-1	singlet	0	-833.0195004	-2450.54499	-2374.409263	EMD2
AGTCAACGTCAATGGG-1	singlet	1	-356.8137392	-122.8126897	-318.8586732	EMD2
AGTCAACGTGGCGTAA-1	singlet	1	-2938.945157	-1156.521841	-2533.323992	EMD2
AGTCAACTCCATCTAT-1	singlet	1	-449.1443916	-170.0250681	-406.8041016	EMD2
AGTCACAAGTAGAGTT-1	singlet	2	-1802.273921	-1575.412988	-727.1604509	EMD2
AGTCACACACAAATAG-1	singlet	1	-2697.663541	-927.2922841	-2124.341646	EMD2
AGTCACACAGCGTATT-1	singlet	0	-794.554123	-2566.581851	-2456.305092	EMD2
AGTCACAGTAGGGTAC-1	singlet	1	-101.9060381	-39.09108845	-73.78684771	EMD2
AGTCACAGTGGCTAGA-1	singlet	0	-1273.815225	-3954.414334	-3754.833422	EMD2
AGTCACAGTGGTCTGC-1	doublet	0/1	-5823.322637	-7424.843049	-9465.649703	EMD2
AGTCACATCTCACTCG-1	singlet	1	-3990.894147	-1345.37957	-3258.273407	EMD2
AGTCATGAGACGAGCT-1	singlet	2	-476.5934919	-403.5288121	-161.3946097	EMD2
AGTCATGAGCATTGAA-1	singlet	1	-2509.460917	-971.2851969	-2121.348646	EMD2
AGTCATGAGTTTAGGA-1	singlet	1	-1008.834772	-385.6699717	-920.2847083	EMD2
AGTCATGCACCCTCTA-1	singlet	1	-369.505334	-138.7839178	-323.3657876	EMD2
AGTCATGCATAGAAAC-1	singlet	1	-920.7989523	-328.1964255	-728.6458762	EMD2
AGTCATGCATGGAAGC-1	singlet	1	-3839.391355	-1219.304611	-2945.046992	EMD2
AGTCATGGTAGGAGGG-1	singlet	2	-1372.825866	-1142.94297	-464.5307534	EMD2
AGTCATGGTCTCGCGA-1	singlet	2	-1858.017753	-1643.854855	-679.2837843	EMD2
AGTCATGGTGATATAG-1	singlet	1	-696.7099839	-256.738242	-515.8879808	EMD2
AGTCATGTCAAGAGGC-1	singlet	1	-2901.207713	-895.5044167	-2162.205709	EMD2
AGTCATGTCATCACAG-1	singlet	1	-200.6981442	-73.62250728	-186.0263727	EMD2
AGTCATGTCCCTGTTG-1	singlet	0	-975.1211704	-2835.071489	-2730.837719	EMD2
AGTCATGTCCGTACGG-1	singlet	1	-2833.412073	-907.1289294	-2326.134934	EMD2
AGTCATGTCGAGTCTA-1	singlet	1	-680.9769948	-241.9198887	-565.6755244	EMD2
AGTCATGTCGCACTCT-1	singlet	1	-2847.717942	-874.0035974	-2548.172575	EMD2
AGTCATGTCGTTATCT-1	singlet	1	-2357.166494	-750.8645988	-2022.019821	EMD2
AGTCATGTCTGAACGT-1	singlet	1	-3756.000328	-1325.964788	-3175.077999	EMD2
AGTCTCCAGGAGCTGT-1	singlet	0	-410.9631511	-1060.858108	-1080.411912	EMD2
AGTCTCCAGTCGGCCT-1	singlet	2	-1222.644171	-1082.632041	-434.6338743	EMD2
AGTCTCCGTCGAAGCA-1	singlet	0	-38.85759908	-158.5276957	-138.779036	EMD2
AGTCTCCGTTAAACAG-1	singlet	2	-103.310586	-100.3809855	-44.26635903	EMD2
AGTCTCCTCGACGACC-1	singlet	0	-421.7336554	-1497.553798	-1222.098013	EMD2
AGTCTCCTCGTGTGGC-1	singlet	1	-700.8833217	-205.4943378	-535.9436084	EMD2
AGTCTCCTCTAGCCAA-1	singlet	1	-158.2651611	-68.21693768	-105.009964	EMD2
AGTGACTAGTTAACAG-1	singlet	0	-729.8337534	-2224.573456	-2186.670126	EMD2
AGTGACTCAAGATTGA-1	singlet	2	-211.408965	-215.864543	-103.6869078	EMD2
AGTGACTCAGAGTCTT-1	singlet	1	-845.5709789	-290.6604867	-630.4320269	EMD2
AGTGACTGTGACTCGC-1	singlet	1	-1591.970421	-536.8837588	-1274.536268	EMD2
AGTGACTTCGCTAAAC-1	singlet	1	-1181.2228	-361.303645	-937.0127626	EMD2
AGTGATCAGGCTCACC-1	singlet	1	-1206.725949	-270.0038006	-747.814699	EMD2
AGTGATCAGTAAGACT-1	doublet	0/1	-2964.978939	-5533.317203	-6397.823387	EMD2
AGTGATCAGTAGGTTA-1	doublet	0/1	-643.6696438	-1097.924812	-1027.352876	EMD2
AGTGATCAGTGGAATT-1	singlet	0	-326.7381545	-901.3931421	-784.3088506	EMD2
AGTGATCCAACACAGG-1	singlet	1	-174.774999	-77.60285539	-162.2472536	EMD2
AGTGATCCAAGGTCGA-1	singlet	1	-487.1113373	-218.8396345	-467.3172757	EMD2
AGTGATCCAATGCTCA-1	singlet	2	-1690.040053	-1766.626113	-518.4437659	EMD2
AGTGATCCATGACTGT-1	singlet	1	-1254.362831	-471.8653493	-1029.274157	EMD2
AGTGATCGTAGGGAGG-1	doublet	0/2	-2016.809556	-4191.872312	-3358.828922	EMD2
AGTGATCGTCACTCTC-1	singlet	2	-667.4401658	-671.5876133	-233.1486836	EMD2
AGTGATCTCCTGCTAC-1	singlet	2	-1485.744157	-1489.52877	-507.5032047	EMD2
AGTGCCGAGACCATGG-1	singlet	1	-5611.895124	-1656.653786	-4232.938948	EMD2
AGTGCCGCAAATACAG-1	unassigned	45293	-11.46552074	-6.125880541	-7.582578482	EMD2
AGTGCCGCAATGGCCC-1	singlet	1	-4518.842182	-1454.564312	-3757.882066	EMD2
AGTGCCGCACCCTGAG-1	singlet	2	-933.3348515	-763.0589388	-304.2062607	EMD2
AGTGCCGCATCATCTT-1	singlet	2	-275.3448013	-227.9634047	-120.4463728	EMD2
AGTGCCGGTGAGATCG-1	singlet	1	-1408.391927	-411.6733086	-1052.091706	EMD2
AGTGCCGGTGAGCCAA-1	singlet	1	-3322.906992	-957.700268	-3042.426535	EMD2
AGTGCCGGTTGTGCCG-1	singlet	1	-292.1537602	-146.5982392	-312.5551094	EMD2
AGTGCCGTCACAATGC-1	singlet	1	-2771.906747	-811.7362683	-2133.255027	EMD2
AGTGCCGTCATGCCGG-1	singlet	1	-2017.43683	-541.5323157	-1846.769381	EMD2
AGTGCCGTCCACCTGT-1	singlet	0	-575.438841	-2345.328573	-2174.569651	EMD2
AGTGCCGTCCATAAGC-1	singlet	2	-4443.181169	-3897.42273	-1472.581169	EMD2
AGTGCCGTCCGGCAGT-1	singlet	1	-277.4795606	-110.6057272	-249.3040495	EMD2
AGTGCCGTCCGTGGGT-1	singlet	0	-152.7518198	-451.4708278	-343.0510951	EMD2
AGTGCCGTCTTAAGGC-1	singlet	1	-345.2296411	-147.6634853	-313.5134232	EMD2
AGTGTTGAGTTCACTG-1	singlet	2	-338.2595044	-295.9168456	-148.1572991	EMD2
AGTGTTGCACAGTGTT-1	singlet	0	-1265.539244	-4402.602808	-4068.313796	EMD2
AGTGTTGGTGCCTATA-1	singlet	2	-2813.060689	-2354.128026	-799.6276876	EMD2
AGTGTTGGTGGCAGAT-1	singlet	0	-549.1028689	-1638.822677	-1459.674049	EMD2

AGTGTTGTCGCTAATG-1	singlet	1	-753.8331636	-232.0714904	-532.4238061	EMD2
AGTTAGCAGCTGTCCG-1	singlet	1	-867.0104687	-305.4674868	-690.7104301	EMD2
AGTTAGCAGGCGTCCT-1	singlet	2	-2402.959584	-2085.033178	-839.5398549	EMD2
AGTTAGCAGGCTTAGG-1	singlet	1	-1466.681017	-560.9606889	-1201.578185	EMD2
AGTTAGCAGTGAGGCT-1	doublet	0/2	-28.51436984	-32.48421058	-29.05296832	EMD2
AGTTAGCCAAACTCGT-1	singlet	0	-1013.034859	-2859.578798	-2724.911204	EMD2
AGTTAGCCACTGCATA-1	singlet	1	-579.0202603	-184.2266618	-396.1232413	EMD2
AGTTAGCCAGAGGAAA-1	singlet	1	-1479.27645	-424.3177935	-1097.220014	EMD2
AGTTAGCCAGCCTTCT-1	singlet	1	-2774.709173	-818.3525127	-2124.060691	EMD2
AGTTAGCCAGTGGCTC-1	singlet	2	-2299.501247	-1859.922085	-724.0579716	EMD2
AGTTAGCCATGGAACG-1	unassigned	1	-2.458100735	-1.893045146	-5.789439693	EMD2
AGTTAGCGTAATGATG-1	singlet	1	-406.3020898	-139.5558441	-372.2231506	EMD2
AGTTAGCTCTCCACTG-1	singlet	0	-617.8812582	-2139.403107	-1726.741177	EMD2
AGTTAGCTCTCTGAGA-1	singlet	1	-3742.473867	-1234.206736	-2911.219042	EMD2
AGTTCCCAGACTCGAG-1	singlet	0	-1319.124051	-4218.756453	-3980.416308	EMD2
AGTTCCCAGCGACTGA-1	singlet	1	-1273.851033	-349.7137245	-957.4882164	EMD2
AGTTCCCAGCGCTGAA-1	singlet	1	-2482.686323	-932.3277174	-2094.704267	EMD2
AGTTCCCAGCTAGATA-1	singlet	1	-1236.360025	-444.078346	-907.6527917	EMD2
AGTTCCCAGGATTCCT-1	singlet	0	-295.9344397	-717.7592585	-706.8567527	EMD2
AGTTCCCCATAACCCA-1	singlet	0	-146.1193595	-410.9236976	-333.4998408	EMD2
AGTTCCCGTCATGACT-1	singlet	0	-51.60494383	-164.2649506	-172.914219	EMD2
AGTTCCCTCCAAGCTA-1	singlet	1	-2981.153249	-981.6683232	-2247.266031	EMD2
AGTTCCCTCCGCAACG-1	singlet	1	-473.6364107	-145.167878	-318.3480131	EMD2
AGTTCGAAGAGGTATT-1	singlet	1	-268.6848117	-105.7548334	-207.4585327	EMD2
AGTTCGAAGATCGGTG-1	singlet	1	-86.4155885	-35.1417109	-81.55839033	EMD2
AGTTCGAAGCCGCACT-1	singlet	0	-2148.762109	-6978.027269	-6888.79618	EMD2
AGTTCGAAGTTAGAAC-1	singlet	2	-358.4123948	-355.9662161	-145.0362427	EMD2
AGTTCGAAGTTGCATC-1	singlet	1	-2153.013778	-632.9938015	-1747.463006	EMD2
AGTTCGACACCAGCGT-1	singlet	1	-1997.741844	-562.262355	-1814.565717	EMD2
AGTTCGACATAGATCC-1	singlet	1	-433.7494209	-197.1488844	-410.3867914	EMD2
AGTTCGAGTACGTAGG-1	singlet	1	-273.7077977	-93.85347632	-255.3974792	EMD2
AGTTCGATCAACGAGG-1	singlet	0	-376.583162	-1070.432905	-1016.467264	EMD2
AGTTCGATCGTGCTCT-1	singlet	1	-2107.45508	-649.0548848	-1577.643193	EMD2
AGTTCGATCTAGATCG-1	singlet	1	-759.9733892	-251.8423612	-597.1520052	EMD2
ATACCGAAGAATAACC-1	singlet	1	-8949.730636	-2735.915866	-6620.28119	EMD2
ATACCGAAGATTGTGA-1	singlet	1	-566.9398684	-204.2969966	-439.9843606	EMD2
ATACCGAAGCAAATGT-1	singlet	1	-2447.673281	-810.9575372	-1885.6079	EMD2
ATACCGAAGCCTCAAT-1	singlet	2	-8429.672548	-6305.72741	-2475.69602	EMD2
ATACCGAAGCGAATGC-1	singlet	1	-404.9573585	-147.363421	-369.4924389	EMD2
ATACCGAAGCTGTACT-1	singlet	1	-3512.824321	-939.5455005	-2333.829308	EMD2
ATACCGAAGGATAATC-1	doublet	45293	-252.3274924	-130.1717121	-169.4218776	EMD2
ATACCGAAGGTGGCTA-1	singlet	2	-324.6971828	-293.3192405	-126.3220687	EMD2
ATACCGACAAACGAGC-1	singlet	1	-1899.978913	-650.2317749	-1473.371575	EMD2
ATACCGACACATATCG-1	singlet	1	-2688.659773	-752.7916407	-2070.3835	EMD2
ATACCGAGTGAGTAGC-1	singlet	2	-2055.149467	-1616.818126	-630.1049786	EMD2
ATACCGAGTGTCTTAG-1	singlet	2	-2135.287007	-2107.839272	-464.5411827	EMD2
ATACCGAGTTGCATAC-1	singlet	1	-978.8582216	-295.0807708	-842.537605	EMD2
ATACCGAGTTTAAGGA-1	singlet	2	-1197.812825	-931.1114667	-425.2573909	EMD2
ATACCGATCGGTGTAT-1	singlet	1	-243.6711814	-90.03007443	-232.5519489	EMD2
ATACCTTAGCTAATCC-1	singlet	1	-385.7178006	-171.0323412	-333.9487167	EMD2
ATACCTTAGGTCGTAG-1	singlet	0	-209.5051749	-670.6442671	-529.1861398	EMD2
ATACCTTCAAAGACTA-1	singlet	2	-2138.401931	-1769.728696	-831.016316	EMD2
ATACCTTCACATATGC-1	singlet	1	-2778.433361	-1057.592497	-2158.809668	EMD2
ATACCTTCAGATCCAT-1	singlet	1	-1561.646168	-495.6834845	-1238.063207	EMD2
ATACCTTCATAGTCGT-1	singlet	2	-85.62673206	-53.19374854	-24.32213025	EMD2
ATACCTTCATCTGGGC-1	singlet	1	-543.7655348	-182.5225446	-414.104217	EMD2
ATACCTTGTTAAAGTG-1	singlet	1	-947.7154357	-344.7543985	-796.7274707	EMD2
ATACCTTTCCTATTTG-1	singlet	1	-2005.115479	-823.4936982	-1681.905984	EMD2
ATACCTTTCTCCGCAT-1	singlet	1	-589.7891957	-187.5099943	-459.340802	EMD2
ATACTTCAGAAGTCTA-1	singlet	0	-30.07231456	-56.75177038	-63.12652834	EMD2
ATACTTCAGAGTTGAT-1	singlet	1	-339.1190063	-108.5271828	-221.0390389	EMD2
ATACTTCCAAAGGAGA-1	singlet	0	-626.5084888	-1924.193411	-1811.701436	EMD2
ATACTTCCACAACGTT-1	singlet	0	-854.2995119	-2310.322717	-2287.672257	EMD2
ATACTTCCACGACGCT-1	singlet	0	-1301.521247	-4227.74365	-4076.801895	EMD2
ATACTTCCAGTCTTCC-1	singlet	2	-1639.775885	-1442.332285	-674.7536094	EMD2
ATACTTCCATCCTAAG-1	singlet	1	-2125.741589	-750.8958937	-1772.799636	EMD2
ATACTTCCATGACTTG-1	singlet	1	-1936.75962	-564.2564498	-1444.034126	EMD2
ATACTTCGTCTAATCG-1	singlet	1	-2137.794157	-764.3510458	-1781.150488	EMD2
ATACTTCGTCTTCGAA-1	singlet	1	-2517.301534	-693.7809484	-2175.453766	EMD2
ATACTTCGTTACTCAG-1	singlet	1	-413.1545715	-191.7867505	-393.9168332	EMD2
ATACTTCTCAAGGCTT-1	singlet	1	-400.98527	-136.9921388	-289.2369744	EMD2
ATACTTCTCAGGAAAT-1	singlet	0	-316.756435	-874.4879485	-876.9438511	EMD2
ATACTTCTCCACGGGT-1	singlet	1	-3780.366524	-1079.322713	-2782.920475	EMD2
ATACTTCTCTCCCATG-1	unassigned	0/2	-22.34390316	-27.45182117	-23.28209624	EMD2
ATAGACCAGGAGTATT-1	singlet	1	-699.6003534	-289.0089418	-589.1836815	EMD2
ATAGACCAGTCGCTAT-1	singlet	0	-587.5087019	-1802.509326	-1727.983245	EMD2
ATAGACCAGTCGGGAT-1	singlet	1	-1866.486805	-567.0412636	-1442.942692	EMD2
ATAGACCCAATCACGT-1	singlet	1	-289.2416393	-114.1848066	-301.558489	EMD2
ATAGACCCACCTGAAT-1	singlet	1	-2796.581894	-949.5753083	-2327.329034	EMD2
ATAGACCCACTAACGT-1	singlet	1	-952.4433145	-359.1195625	-806.1887459	EMD2
ATAGACCGTATGAAAC-1	singlet	1	-4159.744629	-1268.494727	-3174.467943	EMD2
ATAGACCGTCGTATTG-1	singlet	0	-460.3488817	-1325.137055	-1314.863472	EMD2
ATAGACCGTGTTTGCA-1	singlet	1	-937.3000178	-328.6839629	-805.0192478	EMD2
ATAGACCTCGGACGTC-1	singlet	2	-5451.431322	-4511.231492	-1453.822189	EMD2
ATAGACCTCGTCAAAC-1	singlet	1	-677.6544059	-253.0911121	-649.4060798	EMD2
ATAGAGAAGCGTGAAC-1	singlet	1	-879.5064627	-362.3292954	-739.216247	EMD2
ATAGAGAAGTGGTGAC-1	singlet	0	-174.6006326	-406.0773567	-437.9711711	EMD2
ATAGAGACAAGGCGTA-1	singlet	1	-2561.257686	-735.6281309	-2046.964419	EMD2
ATAGAGACAAGTATCC-1	unassigned	0/2	-70.64169747	-156.9582455	-113.2305645	EMD2
ATAGAGACAGAGTTGG-1	singlet	2	-254.0649027	-227.0114652	-96.23173661	EMD2
ATAGAGAGTATTGCCA-1	singlet	2	-2316.583549	-1852.321128	-614.5623244	EMD2
ATAGAGAGTCCACGCA-1	singlet	2	-399.4667505	-319.4902029	-94.2533364	EMD2
ATAGAGAGTGCTTATG-1	singlet	1	-79.42216583	-37.23878672	-96.0667104	EMD2
ATAGAGATCGACATTG-1	singlet	0	-759.2925952	-2058.927721	-1939.106196	EMD2
ATAGAGATCTACGCGG-1	singlet	1	-4792.928256	-1486.116935	-4146.570629	EMD2
ATAGAGATCTTGCAGA-1	singlet	1	-523.4968976	-161.8127866	-334.9035936	EMD2
ATAGGCTAGTGTACAA-1	singlet	1	-109.0557749	-33.86001577	-78.6911927	EMD2
ATAGGCTCATAAGATG-1	singlet	2	-472.7595873	-475.1261984	-218.4690282	EMD2
ATAGGCTGTATACAGA-1	singlet	1	-320.171641	-119.0793767	-255.757313	EMD2
ATAGGCTGTGCGTGCT-1	singlet	1	-868.5619481	-299.1075582	-790.1763487	EMD2
ATAGGCTTCAAGAAAC-1	singlet	1	-1255.802467	-355.2852611	-972.0732932	EMD2
ATAGGCTTCCTAACAG-1	singlet	1	-6846.507356	-2141.238974	-5423.303939	EMD2
ATAGGCTTCCTTCGAC-1	singlet	1	-242.9429503	-98.45388023	-218.4204085	EMD2
ATAGGCTTCTTCCTAA-1	singlet	2	-108.3084614	-107.5351724	-34.59717074	EMD2
ATATCCTAGCCGGAAT-1	singlet	1	-4326.503893	-1283.786378	-3441.321521	EMD2
ATATCCTAGCTGCCAC-1	singlet	2	-1426.569421	-1341.225783	-477.2659622	EMD2
ATATCCTAGGCACCAA-1	unassigned	2/0	-14.47686216	-15.64847827	-9.593791368	EMD2
ATATCCTAGGCACTAG-1	singlet	1	-3297.083712	-907.1637472	-2685.040479	EMD2
ATATCCTAGGCCTGCT-1	singlet	1	-3245.609173	-1107.951198	-2415.198452	EMD2
ATATCCTAGGTAAGGA-1	singlet	1	-512.4826682	-176.8334436	-438.1132617	EMD2

ATATCCTAGGTAGCAC-1	singlet	1	-2037.275847	-543.41963	-1660.009458	EMD2
ATATCCTAGTTGCTCA-1	singlet	2	-2090.635459	-1984.552377	-772.6296954	EMD2
ATATCCTCACGGCTAC-1	singlet	1	-7503.205822	-2381.616402	-6101.014793	EMD2
ATATCCTCATACTGTG-1	singlet	1	-80.05882281	-26.17379489	-73.93374122	EMD2
ATATCCTCATGCAGCC-1	doublet	1/0	-535.4697021	-342.5244918	-553.6139795	EMD2
ATATCCTGTGCGCTCA-1	singlet	1	-4590.502675	-1480.160835	-3859.196123	EMD2
ATATCCTGTGTATTGC-1	singlet	1	-2475.159028	-808.9992605	-1912.055825	EMD2
ATATCCTTCAGCCTCT-1	singlet	1	-874.3898905	-328.7352627	-793.2177763	EMD2
ATATCCTTCGCGTTTC-1	singlet	0	-607.4560553	-2234.246635	-2024.909834	EMD2
ATCACAGAGACTCATC-1	singlet	1	-805.6422681	-286.3468612	-743.3210421	EMD2
ATCACAGAGCGACATG-1	singlet	1	-2133.273693	-729.3524296	-1656.411862	EMD2
ATCACAGAGGCGACAT-1	singlet	1	-381.4372687	-162.832874	-341.7915441	EMD2
ATCACAGAGTGATTCC-1	singlet	2	-553.3650407	-500.5177981	-259.0792662	EMD2
ATCACAGCACACCTGG-1	singlet	1	-812.7255063	-220.8114619	-502.4698628	EMD2
ATCACAGCAGGACTTT-1	singlet	1	-3071.371862	-900.0140599	-2665.206455	EMD2
ATCACAGCATACATCG-1	singlet	0	-530.9668648	-1571.890344	-1368.982159	EMD2
ATCACAGCATGCACTA-1	singlet	1	-916.9543065	-355.2167062	-813.2682317	EMD2
ATCACAGGTATAATGG-1	singlet	2	-1796.841472	-1627.010716	-568.7650144	EMD2
ATCACAGGTCGACTTA-1	singlet	1	-582.9501347	-244.8486781	-517.8232585	EMD2
ATCACAGTCGCAGTTA-1	singlet	1	-1140.270966	-293.0069255	-957.0516082	EMD2
ATCACAGTCGTTGCCT-1	singlet	0	-1154.270989	-3902.036526	-3600.882761	EMD2
ATCACAGTCGTTTACT-1	singlet	1	-1015.71458	-321.0214829	-738.5820193	EMD2
ATCACAGTCTATACTC-1	singlet	2	-3620.834047	-2890.523518	-1236.932581	EMD2
ATCACGAAGGCTCTAT-1	singlet	1	-2291.610438	-727.3637095	-1740.056388	EMD2
ATCACGACAGGTATGG-1	singlet	1	-1377.863126	-499.9676442	-1186.614664	EMD2
ATCACGAGTAACCCTA-1	singlet	1	-1565.977921	-563.9022094	-1296.105325	EMD2
ATCACGAGTCTTAGTG-1	singlet	2	-2697.067824	-2012.663421	-809.913754	EMD2
ATCACGATCTTACGTT-1	singlet	0	-238.8199785	-704.9462033	-760.9823869	EMD2
ATCACTTAGTGGAATT-1	singlet	1	-972.855523	-345.3089972	-786.2762689	EMD2
ATCACTTCAAGGTCAG-1	singlet	1	-1850.548588	-495.9655971	-1578.383497	EMD2
ATCACTTCAGAAATCA-1	singlet	1	-231.5307114	-115.0350627	-210.6449779	EMD2
ATCACTTCAGAGACTG-1	singlet	0	-681.2009835	-2316.588799	-2174.094199	EMD2
ATCACTTCAGAGGCAT-1	unassigned	1	-3.812633678	-1.958138638	-2.370188139	EMD2
ATCACTTGTAGTGATA-1	singlet	1	-549.506093	-199.4867167	-461.0609771	EMD2
ATCACTTGTCATGACT-1	singlet	1	-332.2903771	-138.8125631	-278.0930606	EMD2
ATCACTTGTGACATCT-1	singlet	2	-228.9395141	-226.2837389	-85.61030823	EMD2
ATCACTTGTGGTCCGT-1	singlet	1	-1236.46504	-343.1416834	-1081.113962	EMD2
ATCAGGTAGAAATTCG-1	unassigned	45293	-49.26421647	-20.77835408	-22.34166923	EMD2
ATCAGGTAGCCGATAG-1	doublet	1/0	-2085.933817	-1162.877816	-1885.026903	EMD2
ATCAGGTAGGTACTGG-1	singlet	1	-404.23743	-189.1250857	-348.9069132	EMD2
ATCAGGTAGTATTCCG-1	singlet	2	-317.066056	-271.9066219	-135.0190595	EMD2
ATCAGGTCACAGTCCG-1	singlet	1	-1025.411988	-398.7787086	-821.6404129	EMD2
ATCAGGTCATCACGGC-1	singlet	0	-1210.087041	-4151.158868	-3906.756182	EMD2
ATCAGGTCATCAGCGC-1	singlet	1	-2265.490563	-679.04826	-1601.029307	EMD2
ATCAGGTCATCCTATT-1	singlet	0	-1009.531616	-2800.987084	-2756.387387	EMD2
ATCAGGTGTAGAGGAA-1	singlet	1	-883.8349668	-319.2493341	-707.5531349	EMD2
ATCAGGTGTGGTTTAC-1	singlet	1	-2986.188554	-996.965841	-2464.537593	EMD2
ATCAGGTTCCGAGATT-1	singlet	1	-2045.028207	-704.5436455	-1756.628174	EMD2
ATCAGGTTCGGAGATG-1	unassigned	0/2	-8.514458477	-16.38835166	-15.63289467	EMD2
ATCAGGTTCGTCACCT-1	singlet	1	-409.2905219	-120.5387299	-269.4419022	EMD2
ATCATTCAGGAATTAC-1	singlet	2	-2274.582151	-1938.962573	-820.7584334	EMD2
ATCATTCAGTGATTCC-1	singlet	1	-2908.258113	-1000.348871	-2346.209344	EMD2
ATCATTCCACAGCCTG-1	singlet	1	-483.8364592	-178.9022539	-364.9124511	EMD2
ATCATTCCATGGTACT-1	singlet	1	-318.9877967	-130.6858107	-212.3757155	EMD2
ATCATTCGTACGATCT-1	singlet	1	-1628.047444	-455.8968122	-1572.199314	EMD2
ATCATTCGTACTCGTA-1	singlet	0	-298.7692024	-848.5216125	-740.547728	EMD2
ATCATTCGTCTGCAAT-1	singlet	1	-1264.83818	-393.6084368	-994.314923	EMD2
ATCATTCTCGTGCGAC-1	singlet	1	-404.8453243	-192.9242494	-358.6300277	EMD2
ATCATTCTCGTTACCC-1	singlet	1	-682.8136118	-209.5972942	-517.1955563	EMD2
ATCATTCTCTTTCCGG-1	singlet	1	-612.1633726	-211.9974449	-493.550476	EMD2
ATCCACCAGAAGAACG-1	singlet	1	-510.2139739	-202.9712106	-509.6740804	EMD2
ATCCACCAGCGAGAAA-1	singlet	1	-217.421682	-101.2758888	-202.1590154	EMD2
ATCCACCAGGGAGTGG-1	singlet	1	-1847.698391	-536.5932013	-1456.424872	EMD2
ATCCACCAGTGCACCC-1	singlet	0	-302.3393507	-907.6321243	-874.5499036	EMD2
ATCCACCCACTTGTCC-1	singlet	1	-144.0652621	-46.79519078	-110.0435099	EMD2
ATCCACCCATGACGTT-1	unassigned	45293	-119.4801967	-47.07586216	-93.12832435	EMD2
ATCCACCGTACGAGCA-1	singlet	2	-4255.155334	-3474.619516	-1329.149109	EMD2
ATCCACCGTACTGCCG-1	singlet	1	-1084.493135	-427.3124878	-931.9677698	EMD2
ATCCACCGTATTTCGG-1	singlet	2	-588.4878535	-508.8524928	-217.9723456	EMD2
ATCCACCTCACCCATC-1	singlet	2	-488.444653	-481.9641095	-199.7570137	EMD2
ATCCACCTCAGCAATC-1	singlet	1	-719.0585173	-255.8118985	-565.2520801	EMD2
ATCCACCTCCACACCT-1	singlet	2	-3534.61016	-3769.04333	-1079.197832	EMD2
ATCCACCTCCACTGGG-1	singlet	2	-1447.942654	-1248.719808	-532.0646648	EMD2
ATCCACCTCTGCCCTA-1	singlet	1	-470.640959	-162.7870588	-370.1696438	EMD2
ATCCATTAGGGAGGGT-1	singlet	1	-298.8055065	-99.29902857	-201.1192858	EMD2
ATCCATTAGTGTCATC-1	singlet	1	-2169.680926	-789.3951325	-1730.02902	EMD2
ATCCATTAGTTTAGGA-1	singlet	1	-8622.104744	-2717.953071	-6247.662184	EMD2
ATCCATTCAAGTACCT-1	singlet	1	-31.06216091	-3.707656198	-8.294811658	EMD2
ATCCATTCAAGTATCC-1	doublet	0/1	-1217.209107	-1396.096958	-1737.445429	EMD2
ATCCATTCAATAGTAG-1	singlet	0	-346.7790658	-1268.930325	-1049.084771	EMD2
ATCCATTCAATTGTGC-1	singlet	1	-5014.581031	-1709.176448	-4034.668705	EMD2
ATCCATTCACCCGTAG-1	singlet	1	-467.0835188	-196.8557448	-449.675816	EMD2
ATCCATTCACGACAAG-1	singlet	2	-1338.437155	-1169.331952	-548.7961425	EMD2
ATCCATTCAGACGGAT-1	doublet	0/2	-3735.713142	-4874.923402	-4005.95828	EMD2
ATCCATTGTATGAGCG-1	singlet	2	-2208.048679	-1998.681692	-746.1037003	EMD2
ATCCATTTCACCTTGC-1	singlet	1	-739.2958167	-235.7113334	-573.6832953	EMD2
ATCCATTTCGTTCTGC-1	singlet	0	-1654.057154	-6057.035042	-5904.753811	EMD2
ATCCATTTCTCATTAC-1	singlet	1	-1728.889524	-616.15346	-1388.660946	EMD2
ATCCCTGAGTGCTCGC-1	singlet	2	-541.0533398	-542.2460813	-192.2019081	EMD2
ATCCCTGCACACGGTC-1	singlet	1	-1309.414946	-482.1300286	-1117.6647	EMD2
ATCCCTGCAGCCTATA-1	singlet	1	-694.6154671	-255.7601503	-539.062915	EMD2
ATCCCTGGTCGTACTA-1	singlet	1	-810.9471714	-311.6800914	-691.4301112	EMD2
ATCCCTGGTGGACCTC-1	singlet	2	-1731.303447	-1499.074775	-633.1680742	EMD2
ATCCCTGTCAAGATAG-1	singlet	0	-997.0356621	-3113.39336	-2983.324855	EMD2
ATCCCTGTCATCCTAT-1	singlet	1	-3592.093221	-1336.616379	-3078.727886	EMD2
ATCCCTGTCTGGTGCG-1	singlet	1	-805.5791786	-262.5111716	-555.0201718	EMD2
ATCCGTCAGATACATG-1	singlet	1	-90.24191212	-46.03540832	-72.57371746	EMD2
ATCCGTCAGGTGATAT-1	singlet	1	-4000.626796	-1203.402488	-3195.407865	EMD2
ATCCGTCAGGTGCATG-1	singlet	1	-1119.186612	-381.7439554	-1057.265294	EMD2
ATCCGTCAGTTGCTCA-1	singlet	1	-766.9422333	-303.8728924	-604.4398621	EMD2
ATCCGTCCAAATGATG-1	singlet	1	-423.4428095	-175.2146487	-349.6056969	EMD2
ATCCGTCCACTTCAAG-1	singlet	1	-453.1218382	-159.8116179	-367.223041	EMD2
ATCCGTCCAGGTGGAT-1	singlet	1	-263.5779976	-95.06479146	-162.2583678	EMD2
ATCCGTCCATCTTTCA-1	singlet	0	-888.5237431	-2650.766932	-2569.282999	EMD2
ATCCGTCCATGCTGCG-1	singlet	2	-515.3062849	-452.6079563	-187.7444213	EMD2
ATCCGTCGTCATCTAG-1	singlet	0	-439.2719359	-1528.725238	-1460.383663	EMD2
ATCCGTCGTCTGTGAT-1	doublet	1/0	-2259.408006	-1439.718133	-2272.990222	EMD2
ATCCGTCGTGAAGCGT-1	singlet	2	-134.8082105	-109.2158257	-45.64934081	EMD2
ATCCGTCGTGTCGCTG-1	singlet	0	-104.5089184	-291.0566917	-240.8827661	EMD2
ATCCTATAGTAATCCC-1	singlet	0	-601.8344166	-1861.267075	-1698.865911	EMD2

ATCCTATCAACGAGGT-1	singlet	1	-1000.834216	-441.7937883	-884.0930262	EMD2
ATCCTATCATACAGAA-1	singlet	2	-1610.849092	-1727.618215	-798.1707974	EMD2
ATCCTATGTCTTGAAC-1	singlet	1	-996.2622935	-342.6988935	-890.2602301	EMD2
ATCCTATGTGAACTAA-1	singlet	0	-365.1203791	-987.2401249	-1108.872858	EMD2
ATCCTATGTGAATAAC-1	singlet	0	-224.6914948	-654.4606805	-633.1788944	EMD2
ATCCTATGTGAGTGAC-1	singlet	2	-5215.007102	-4180.081189	-1690.935768	EMD2
ATCCTATTCATTGCGA-1	singlet	2	-68.60461555	-91.51075148	-36.75334584	EMD2
ATCCTATTCGATCCAA-1	singlet	2	-2194.991008	-1814.107959	-898.6822327	EMD2
ATCGATGAGATAGTCA-1	singlet	0	-91.01629299	-330.0409442	-292.3348047	EMD2
ATCGATGAGGGATCTG-1	singlet	2	-2070.208529	-1691.039017	-728.2949503	EMD2
ATCGATGCAGTCTGGC-1	singlet	2	-2106.378106	-1911.717969	-688.1580715	EMD2
ATCGATGGTCACCACG-1	singlet	0	-657.0125025	-2018.544284	-1871.608788	EMD2
ATCGATGGTCTTACAG-1	singlet	0	-752.1379933	-1992.34467	-2004.16747	EMD2
ATCGCCTAGACCTCAT-1	singlet	1	-259.4983921	-99.83860652	-210.7378724	EMD2
ATCGCCTAGTGAGGTC-1	singlet	1	-1064.193635	-344.7984912	-843.5560143	EMD2
ATCGCCTAGTGGATAT-1	singlet	0	-774.1797902	-2122.546266	-2147.048823	EMD2
ATCGCCTCACCTAAAC-1	singlet	1	-1722.79296	-579.7496708	-1374.277364	EMD2
ATCGCCTCACGGAAGT-1	singlet	0	-606.0919934	-1826.694398	-1801.425322	EMD2
ATCGCCTGTATCACGT-1	singlet	1	-1056.52884	-368.8640777	-741.3242374	EMD2
ATCGCCTGTCGTTGCG-1	singlet	1	-171.0369996	-61.81627721	-123.1802452	EMD2
ATCGCCTGTGAGACCA-1	singlet	1	-2122.685391	-658.1555249	-1583.899246	EMD2
ATCGCCTGTTGAGAGC-1	unassigned	45293	-41.07836912	-22.87947425	-31.67082252	EMD2
ATCGCCTTCATTGAGC-1	singlet	1	-174.7048443	-108.0150863	-187.8848985	EMD2
ATCGCCTTCCATTCGC-1	singlet	1	-390.1004771	-132.4221197	-282.9421529	EMD2
ATCGCCTTCCCTGGTT-1	unassigned	45293	-152.6506405	-63.99442446	-106.1051975	EMD2
ATCGCCTTCGGTAGAG-1	doublet	2/0	-22.15478708	-22.81385608	-20.60372924	EMD2
ATCGGATCAAACAGGC-1	singlet	1	-378.3432512	-150.4353425	-324.8799156	EMD2
ATCGGATCAATCAAGA-1	singlet	2	-1050.438726	-815.0181436	-432.6237716	EMD2
ATCGGATCATCCAACA-1	singlet	1	-252.0420997	-92.27247463	-196.8707704	EMD2
ATCGGATCATTCACCC-1	singlet	1	-476.9486896	-211.1737414	-456.451735	EMD2
ATCGGATCATTGGGAG-1	singlet	1	-1785.250153	-620.3394132	-1445.101028	EMD2
ATCGGATGTCAAACGG-1	singlet	1	-2496.939099	-787.3104074	-2030.83332	EMD2
ATCGGATGTCAATCTG-1	singlet	1	-1802.902978	-518.7179942	-1533.053765	EMD2
ATCGGATGTCCCTCAT-1	singlet	1	-3700.775885	-1361.590876	-3114.187002	EMD2
ATCGGATGTGGAATGC-1	singlet	1	-467.7642516	-195.4601945	-424.7374275	EMD2
ATCGGATTCAAATGAG-1	singlet	0	-279.3351845	-782.1813143	-755.4124425	EMD2
ATCGGATTCGCTTGCT-1	singlet	1	-950.8078751	-339.0716476	-831.2464472	EMD2
ATCGGATTCGGAATGG-1	singlet	2	-325.8477673	-224.0793877	-128.9973523	EMD2
ATCGGATTCTAGATCG-1	singlet	1	-291.611383	-114.0687957	-230.1287178	EMD2
ATCGGCGAGACTGGGT-1	singlet	1	-444.2729589	-213.569402	-388.1624758	EMD2
ATCGGCGAGCTGGAGT-1	singlet	1	-250.3831987	-86.22585021	-220.5627851	EMD2
ATCGGCGAGGCGCTTC-1	singlet	0	-434.9746258	-1535.774077	-1370.892524	EMD2
ATCGGCGAGTTTCAGC-1	singlet	1	-544.3954404	-176.1506566	-440.1221745	EMD2
ATCGGCGCACATCATG-1	singlet	1	-550.4684079	-194.851491	-484.9663417	EMD2
ATCGGCGCAGCAATTC-1	singlet	1	-1743.122383	-555.927882	-1465.084115	EMD2
ATCGGCGCATGACGAG-1	singlet	1	-1238.133475	-497.606506	-1111.596288	EMD2
ATCGGCGCATGGCGCT-1	singlet	1	-488.4707574	-180.5586403	-405.3643798	EMD2
ATCGGCGGTCCACACG-1	singlet	1	-399.093671	-170.7861347	-389.1770017	EMD2
ATCGGCGGTGATCATC-1	singlet	1	-2512.576737	-718.979078	-2008.447457	EMD2
ATCGGCGTCCGTTGGG-1	singlet	2	-2086.829669	-1611.487656	-631.7840555	EMD2
ATCGGCGTCGTTGTGA-1	singlet	0	-223.2678004	-968.5185468	-889.7430331	EMD2
ATCGGCGTCTGCTTAT-1	singlet	0	-139.0963063	-429.7489104	-358.4478998	EMD2
ATCGTAGAGAAAGCGA-1	singlet	1	-1740.755358	-520.0373857	-1418.458748	EMD2
ATCGTAGAGACGGAAA-1	singlet	1	-2212.606717	-702.2944998	-1859.915138	EMD2
ATCGTAGAGCAAGTCG-1	singlet	2	-239.6562407	-200.2152637	-65.95959041	EMD2
ATCGTAGAGCGGGTAT-1	singlet	0	-126.3444284	-493.4336139	-475.5548194	EMD2
ATCGTAGCACACTTAG-1	singlet	2	-1999.922237	-1928.095241	-571.0594566	EMD2
ATCGTAGCACAGCGCT-1	singlet	0	-28.18204161	-90.93368611	-99.44093946	EMD2
ATCGTAGCACTTCCTG-1	singlet	0	-1490.942049	-6501.992282	-6456.32634	EMD2
ATCGTAGCATACAGGG-1	singlet	1	-945.0611461	-373.8403807	-856.684804	EMD2
ATCGTAGGTGTCGCTG-1	singlet	1	-187.8832289	-70.08031567	-176.1721966	EMD2
ATCGTAGGTGTCTTGA-1	singlet	0	-265.62542	-802.3167797	-769.0479567	EMD2
ATCGTAGGTTAAGGAT-1	singlet	1	-3345.103126	-1135.515099	-2831.9873	EMD2
ATCGTAGTCGTGCAGC-1	singlet	1	-183.9786114	-80.70668241	-146.3493916	EMD2
ATCGTAGTCTTCCGTG-1	singlet	0	-114.8109733	-271.0179361	-262.8482064	EMD2
ATCGTCCAGAGCGACT-1	singlet	2	-1048.188236	-872.2914767	-332.0631526	EMD2
ATCGTCCAGCAGCAGT-1	singlet	1	-2142.193651	-729.9680892	-1843.105841	EMD2
ATCGTCCCAATTCGTG-1	singlet	1	-246.383003	-85.22683998	-227.5820234	EMD2
ATCGTCCCATCATCTT-1	singlet	1	-2582.777607	-801.7886747	-2173.676864	EMD2
ATCGTCCCATCGAGCC-1	singlet	1	-97.75673048	-26.63980084	-56.405286	EMD2
ATCGTCCCATGAATCC-1	singlet	0	-1068.572117	-3294.290492	-3250.672982	EMD2
ATCGTCCGTCCACACG-1	singlet	1	-477.9051789	-162.6851686	-390.2237902	EMD2
ATCGTCCGTTGTCCCT-1	singlet	2	-229.101264	-209.5948623	-87.19795027	EMD2
ATCGTGAAGCATTTGC-1	singlet	2	-144.7606497	-117.8325743	-37.69169004	EMD2
ATCGTGAAGGAACATT-1	singlet	1	-958.7412241	-286.7954909	-739.6778366	EMD2
ATCGTGACAATGAGCG-1	singlet	2	-1038.116953	-887.3149129	-252.2326003	EMD2
ATCGTGACATCCAACA-1	singlet	1	-2465.010714	-700.2655435	-1749.252084	EMD2
ATCGTGACATGGATCT-1	singlet	1	-704.3125645	-261.6684231	-638.9913741	EMD2
ATCGTGAGTACGAAAT-1	singlet	2	-1170.253297	-1108.506489	-509.6782329	EMD2
ATCGTGAGTAGACGTG-1	singlet	1	-2078.751659	-566.0968007	-1892.851159	EMD2
ATCGTGAGTGAATGAT-1	singlet	2	-9523.784881	-6926.622402	-2109.395346	EMD2
ATCGTGAGTTTGCAGT-1	singlet	0	-174.9725366	-641.8740428	-586.6602301	EMD2
ATCGTGATCAACACGT-1	singlet	2	-2680.591748	-2290.34307	-992.0614599	EMD2
ATCGTGATCAGAACCT-1	singlet	1	-662.2613348	-209.6701419	-469.9340285	EMD2
ATCGTGATCAGAGCGA-1	singlet	1	-1787.889112	-561.6070203	-1354.435857	EMD2
ATCGTGATCCCGTAAA-1	singlet	2	-3321.520701	-2798.155176	-1011.029051	EMD2
ATCTCTAAGATCCGAG-1	singlet	0	-610.6001634	-2160.550941	-1883.10578	EMD2
ATCTCTACAAATCGTC-1	singlet	1	-2262.102748	-814.8200633	-1840.963066	EMD2
ATCTCTACACTCCTGT-1	singlet	1	-581.7952016	-243.182365	-490.5454672	EMD2
ATCTCTACAGAACTAA-1	singlet	0	-264.573886	-1016.659546	-932.0505739	EMD2
ATCTCTAGTTAAGGGC-1	singlet	1	-437.5551917	-177.4610252	-406.5098503	EMD2
ATCTCTATCATCGCAA-1	singlet	1	-81.1122471	-20.05528858	-67.37921831	EMD2
ATCTCTATCCATTGGA-1	singlet	2	-3755.000522	-3058.63315	-1338.487522	EMD2
ATCTTCAAGCGGGTTA-1	singlet	1	-97.32652631	-29.91988451	-90.15925941	EMD2
ATCTTCAAGGACGCTA-1	singlet	1	-1534.007207	-503.95184	-1353.696329	EMD2
ATCTTCAAGGCTAACG-1	singlet	1	-3298.280514	-1159.584423	-2760.272804	EMD2
ATCTTCACACCAGACC-1	singlet	0	-18.22171547	-52.88622872	-42.37869517	EMD2
ATCTTCACACCAGTAT-1	singlet	1	-375.0241355	-119.4305291	-322.44492	EMD2
ATCTTCACACTTTAGG-1	singlet	1	-1668.29571	-663.7842513	-1549.703195	EMD2
ATCTTCATCACCTACC-1	singlet	1	-487.1447346	-194.6828998	-395.6242614	EMD2
ATCTTCATCCTTGACC-1	singlet	1	-110.849351	-56.1459698	-142.5608206	EMD2
ATCTTCATCTCGTGAA-1	doublet	45323	-191.0736908	-131.4415957	-121.5998274	EMD2
ATGAAAGAGAGGACTC-1	singlet	1	-237.0736596	-112.9823751	-222.1444143	EMD2
ATGAAAGCAAAGGATT-1	singlet	1	-628.719618	-224.9864742	-539.8912669	EMD2
ATGAAAGGTACTAGCT-1	singlet	2	-551.3511031	-596.9233172	-214.1786954	EMD2
ATGAAAGGTGTGACCC-1	singlet	1	-245.7429973	-70.23536331	-183.470403	EMD2
ATGACCAAGAAGGTAG-1	singlet	1	-1471.220274	-550.4942942	-1180.43214	EMD2
ATGACCAAGAGTCAAT-1	singlet	0	-3335.340753	-9626.390831	-8235.063901	EMD2
ATGACCAAGCGAATGC-1	singlet	1	-4985.036056	-1337.876285	-3835.04261	EMD2
ATGACCAAGCGTTAGG-1	singlet	2	-1930.081849	-1431.028274	-677.3401368	EMD2

ATGACCAAGGACAACC-1	singlet	1	-2231.983886	-950.9329273	-1918.685573	EMD2
ATGACCAAGTAACAGT-1	singlet	1	-2760.432429	-927.92391	-2391.324539	EMD2
ATGACCACAAGATTGA-1	singlet	1	-1068.915342	-296.5282138	-702.8093538	EMD2
ATGACCACAAGGTACG-1	singlet	2	-3510.757955	-3049.880591	-1216.186098	EMD2
ATGACCACAGAGAAAG-1	singlet	1	-993.9619928	-319.8064402	-705.6726756	EMD2
ATGACCACATCGCTAA-1	singlet	1	-347.5586202	-168.4621624	-339.4357159	EMD2
ATGACCACATTCACCC-1	singlet	1	-509.5398405	-198.2580162	-407.5893169	EMD2
ATGACCAGTCTGTGAT-1	singlet	1	-420.6484977	-155.2153686	-320.8059181	EMD2
ATGACCAGTGCCGTTG-1	singlet	1	-1671.279587	-543.9712415	-1408.678183	EMD2
ATGACCATCCACACCT-1	singlet	1	-341.36451	-108.9216867	-266.7066797	EMD2
ATGACCATCGCGCCAA-1	singlet	2	-4152.229931	-3735.337291	-1366.542695	EMD2
ATGACCATCTGTGTGA-1	singlet	1	-531.7728841	-219.2717011	-415.0105153	EMD2
ATGAGGGAGGTTACCT-1	singlet	1	-303.6340362	-124.5354266	-294.7095183	EMD2
ATGAGGGAGTGGCGAT-1	singlet	1	-775.9773019	-311.1479082	-645.0234343	EMD2
ATGAGGGCACATATGC-1	singlet	1	-103.7629883	-49.96058143	-119.4420361	EMD2
ATGAGGGCACTTCATT-1	singlet	0	-1007.221192	-2559.581028	-2528.767258	EMD2
ATGAGGGGTAATACCC-1	singlet	1	-932.8821354	-315.1589729	-735.7856151	EMD2
ATGAGGGGTCCGACGT-1	singlet	0	-27.22111004	-87.40621724	-91.75465678	EMD2
ATGAGGGGTGAGCAGT-1	singlet	2	-1219.34603	-1433.560706	-400.4586491	EMD2
ATGAGGGTCGTTGTTT-1	singlet	2	-312.020248	-350.5529943	-139.0491539	EMD2
ATGAGGGTCTCGGGAC-1	singlet	1	-1442.395642	-410.3339179	-1169.73559	EMD2
ATGAGGGTCTTAGTTC-1	singlet	1	-797.9815821	-320.7314568	-677.2410253	EMD2
ATGAGTCAGAATCGCG-1	singlet	1	-2120.821922	-670.34357	-1745.167406	EMD2
ATGAGTCAGAGTTGAT-1	singlet	1	-1835.941205	-685.779332	-1624.903265	EMD2
ATGAGTCAGTTTGTCG-1	singlet	2	-327.1766146	-278.6965613	-130.7266028	EMD2
ATGAGTCCAACCAACT-1	singlet	1	-238.1352998	-96.6920812	-193.2002987	EMD2
ATGAGTCCAAGGACAC-1	singlet	1	-3836.485403	-1262.532022	-3228.387592	EMD2
ATGAGTCCACAGTCAT-1	singlet	1	-3142.651005	-847.0502156	-2753.189011	EMD2
ATGAGTCCATGCAGGA-1	singlet	1	-527.4357343	-187.8954328	-496.0728005	EMD2
ATGAGTCGTCATTGCA-1	singlet	1	-205.3591263	-89.64112884	-164.5080428	EMD2
ATGAGTCGTCCGACGT-1	singlet	1	-1121.368883	-351.2700851	-793.4636563	EMD2
ATGAGTCGTCTGTGTA-1	singlet	2	-1293.392635	-1431.037903	-488.263299	EMD2
ATGAGTCGTTCATCGA-1	singlet	1	-824.2533663	-276.8423724	-682.3182487	EMD2
ATGAGTCTCAAGTGTC-1	singlet	1	-1393.602744	-462.1293826	-1104.341385	EMD2
ATGATCGAGCACTGGA-1	singlet	1	-312.3752097	-74.98806055	-221.1008275	EMD2
ATGATCGAGCTTCTAG-1	singlet	1	-1621.032091	-516.0984046	-1313.51059	EMD2
ATGATCGAGGTAATCA-1	singlet	0	-41.3823835	-163.7159593	-205.9432634	EMD2
ATGATCGAGGTGGTTG-1	unassigned	1/0	-9.611587043	-6.384909393	-17.4121568	EMD2
ATGATCGAGTCTCGTA-1	singlet	1	-1608.590125	-479.9910893	-1407.683249	EMD2
ATGATCGCAACTGAAA-1	singlet	0	-362.9685538	-1052.233479	-925.8938755	EMD2
ATGATCGCACGAAGAC-1	singlet	0	-511.4776453	-1489.24134	-1379.90485	EMD2
ATGATCGCAGTCGTTA-1	singlet	1	-3527.052925	-1128.052337	-3053.022704	EMD2
ATGATCGCATAGATGA-1	singlet	1	-1502.738361	-556.9698371	-1280.798898	EMD2
ATGATCGGTACCAGAG-1	singlet	1	-530.8080147	-209.5582199	-469.6235286	EMD2
ATGATCGGTACTTGTG-1	singlet	1	-1015.373538	-391.6749225	-924.5131027	EMD2
ATGATCGGTCAGTTTG-1	singlet	0	-40.55925809	-90.75503043	-84.4687947	EMD2
ATGATCGGTGATGGCA-1	singlet	2	-2363.797227	-2060.068292	-794.5666103	EMD2
ATGATCGGTGGGCTTC-1	doublet	45323	-1442.334844	-1049.874463	-744.4981362	EMD2
ATGATCGTCATTTACC-1	singlet	0	-1370.921492	-5036.511718	-4695.534909	EMD2
ATGATCGTCCTGTAAG-1	singlet	2	-1081.586472	-905.2754633	-344.8519931	EMD2
ATGATCGTCGTTCTGC-1	singlet	0	-130.3022917	-445.0006133	-400.6046519	EMD2
ATGCATGAGAGCAACC-1	singlet	2	-698.7571481	-692.0016392	-282.0431552	EMD2
ATGCATGAGCATGGGT-1	singlet	1	-1589.202291	-491.427547	-1224.695	EMD2
ATGCATGAGGTTCATC-1	singlet	2	-827.108559	-723.0305734	-315.3734836	EMD2
ATGCATGCAAGTGATA-1	singlet	1	-631.412379	-204.6573905	-428.0685239	EMD2
ATGCATGCAGAGCTAG-1	singlet	0	-877.5344999	-2554.016539	-2339.734436	EMD2
ATGCATGCATCGATCA-1	singlet	1	-703.6929886	-228.016915	-512.3983569	EMD2
ATGCATGGTACGCGTC-1	singlet	1	-3993.952318	-1260.6092	-3359.824135	EMD2
ATGCATGGTATGCTAC-1	singlet	0	-1078.658444	-2896.613371	-2838.517844	EMD2
ATGCATGGTCCCGCAA-1	singlet	1	-2518.578018	-815.6793923	-1946.271333	EMD2
ATGCATGGTCGCAACC-1	doublet	1/0	-2537.123501	-2222.763847	-3290.580519	EMD2
ATGCATGGTGGTACAG-1	singlet	2	-873.962448	-836.0140534	-348.8761825	EMD2
ATGCATGTCGAGCACC-1	singlet	1	-922.8561817	-323.8724709	-794.1121934	EMD2
ATGCATGTCTTCTTCC-1	singlet	1	-2104.048854	-749.6465763	-1726.074105	EMD2
ATGCCTCAGCTCCACG-1	singlet	0	-1378.403827	-4234.833779	-4168.841652	EMD2
ATGCCTCCAGGTTACT-1	singlet	1	-4353.672357	-1485.66781	-3769.801361	EMD2
ATGCCTCGTACCCGAC-1	singlet	0	-1016.013305	-4444.521465	-4253.324774	EMD2
ATGCCTCGTCCTCCTA-1	singlet	1	-448.2973336	-138.7304814	-331.4382701	EMD2
ATGCCTCGTCTGCATA-1	unassigned	0/1	-9.655432548	-10.512307	-10.25576475	EMD2
ATGCCTCGTGTCCAAT-1	singlet	1	-772.998339	-253.596746	-672.8518464	EMD2
ATGCCTCTCAAGCGTT-1	singlet	2	-3912.166463	-3078.198526	-1348.543338	EMD2
ATGCCTCTCCATTCAT-1	singlet	1	-630.1844925	-234.4487859	-522.1213329	EMD2
ATGCCTCTCCCGTGTT-1	singlet	1	-461.188983	-165.6153828	-374.5079771	EMD2
ATGCGATAGCAAATCA-1	singlet	1	-248.5362904	-65.7969001	-208.592286	EMD2
ATGCGATAGGATATAC-1	singlet	1	-642.8226228	-228.9827296	-606.8054969	EMD2
ATGCGATAGGCTCTCG-1	singlet	1	-1652.061755	-522.4583714	-1450.663463	EMD2
ATGCGATCAACTCGAT-1	singlet	1	-1014.16725	-370.8060409	-817.8559399	EMD2
ATGCGATCAACTGAAA-1	singlet	1	-1770.993848	-616.5179263	-1723.536868	EMD2
ATGCGATCAGCCTACG-1	singlet	1	-188.9937924	-64.26316158	-178.6572412	EMD2
ATGCGATCAGGTCTCG-1	singlet	1	-2615.401754	-833.6623302	-2208.831259	EMD2
ATGCGATCATACCAGT-1	singlet	2	-2566.749291	-2175.102577	-737.3155044	EMD2
ATGCGATCATAGAATG-1	singlet	1	-377.0832652	-160.3455386	-347.5486512	EMD2
ATGCGATCATGTGGTT-1	singlet	2	-501.0708612	-514.3626029	-212.6750824	EMD2
ATGCGATGTAGGGAGG-1	singlet	1	-187.8737649	-44.81304277	-117.6703517	EMD2
ATGCGATGTTAGAAGT-1	singlet	1	-1695.142479	-566.8669188	-1290.118515	EMD2
ATGCGATGTTGCCAAT-1	singlet	0	-92.16698845	-328.3009087	-283.5722943	EMD2
ATGCGATTCAAATGCC-1	singlet	1	-1380.082557	-437.912598	-1103.7623	EMD2
ATGCGATTCCAATCCC-1	singlet	2	-5141.158937	-5197.462954	-1542.829836	EMD2
ATGCGATTCCATTCGC-1	singlet	1	-261.7595622	-107.8012576	-202.4550502	EMD2
ATGGAGGAGAGTAACT-1	singlet	0	-102.6175444	-276.281156	-281.9395472	EMD2
ATGGAGGAGATCACCT-1	singlet	1	-2493.706849	-825.4425626	-2107.959093	EMD2
ATGGAGGCAAGGAGTC-1	singlet	1	-4031.249568	-1314.978113	-3247.766893	EMD2
ATGGAGGCAATTAGGA-1	singlet	2	-1168.793678	-1162.347393	-398.4248056	EMD2
ATGGAGGCATATCGGT-1	singlet	1	-374.4019339	-137.0819888	-297.4311157	EMD2
ATGGAGGGTCAGGAGT-1	doublet	0/1	-4394.091314	-5029.545757	-6705.113333	EMD2
ATGGAGGGTGACTAAA-1	singlet	2	-5931.970971	-4737.581253	-1221.049225	EMD2
ATGGAGGGTGCGGTAA-1	singlet	0	-40.73507219	-79.11077456	-93.60075541	EMD2
ATGGAGGGTGGCGCTT-1	singlet	2	-2620.480889	-2218.818748	-847.9664811	EMD2
ATGGAGGTCATGCGGC-1	singlet	1	-1915.653092	-545.1852821	-1368.508037	EMD2
ATGGATCAGAGAGCCT-1	singlet	1	-1053.619263	-317.5740894	-807.4552867	EMD2
ATGGATCAGCTGACCC-1	singlet	1	-1497.645974	-492.0386151	-1219.65549	EMD2
ATGGATCAGGTAATCA-1	singlet	1	-2978.453793	-914.0300611	-2403.16359	EMD2
ATGGATCAGTTCGCAT-1	singlet	2	-8306.179728	-6699.587005	-2534.683075	EMD2
ATGGATCCAAATACGA-1	singlet	2	-388.5197949	-340.8197415	-158.7401403	EMD2
ATGGATCCAAGGAGTC-1	singlet	1	-600.4303312	-203.6978665	-485.1651574	EMD2
ATGGATCCAGAGTGAC-1	singlet	1	-3060.586493	-1010.420769	-2614.394789	EMD2
ATGGATCCAGGACGAT-1	singlet	1	-3027.14305	-974.4902591	-2297.055326	EMD2
ATGGATCGTGCCTTCT-1	singlet	1	-3955.564057	-1213.232038	-2928.103789	EMD2
ATGGATCGTTCGCGTG-1	doublet	1/0	-1986.427533	-1663.402166	-2371.672968	EMD2
ATGGATCGTTGTGTTG-1	singlet	1	-760.9882176	-234.5995396	-585.1422931	EMD2

ATGGGAGAGTAGCAAT-1	singlet	0	-354.5081084	-1108.506039	-1182.669273	EMD2
ATGGGAGCAAGGGCAT-1	singlet	1	-256.210793	-108.0060157	-206.4976627	EMD2
ATGGGAGCACCGTGAC-1	singlet	0	-814.7336287	-2988.935472	-3112.302249	EMD2
ATGGGAGCAGGGTCTC-1	singlet	1	-520.0162141	-180.3486272	-376.1459292	EMD2
ATGGGAGCATGACTTG-1	doublet	45323	-10474.21537	-7106.905589	-4369.986824	EMD2
ATGGGAGTCAAACGTC-1	singlet	1	-1287.171169	-470.9244497	-1054.312631	EMD2
ATGGGAGTCCCAGCGA-1	singlet	1	-851.2850834	-322.4056999	-776.1572345	EMD2
ATGGGAGTCCGACAGC-1	singlet	0	-1433.865098	-4590.576552	-4381.979341	EMD2
ATGGGAGTCTCGTGGG-1	singlet	1	-790.0161436	-351.6467821	-684.9275824	EMD2
ATGGGAGTCTGTCGCT-1	singlet	2	-804.7380505	-768.3218922	-325.2527358	EMD2
ATGGGAGTCTTCGACC-1	doublet	45293	-7342.726319	-4065.834733	-4494.732008	EMD2
ATGGGTTAGCTTGTGT-1	singlet	2	-1143.958155	-864.9212167	-345.1673715	EMD2
ATGGGTTAGGATTACA-1	singlet	1	-1612.228006	-547.2473666	-1278.372383	EMD2
ATGGGTTAGGTCACCC-1	singlet	1	-1794.821952	-567.2558392	-1498.397962	EMD2
ATGGGTTCAAAGAGTT-1	singlet	2	-2999.308695	-2496.2501	-942.3238062	EMD2
ATGGGTTCAGACCTGC-1	singlet	1	-605.9037641	-231.0924339	-512.3523386	EMD2
ATGGGTTCAGTCTTCC-1	singlet	2	-3243.850976	-2616.513989	-1276.960364	EMD2
ATGGGTTCATGAGATA-1	singlet	0	-55.57112988	-181.8081345	-129.012014	EMD2
ATGGGTTCATGCGTGC-1	singlet	1	-4139.225554	-1208.934812	-3088.668173	EMD2
ATGGGTTTCGTAGTCA-1	singlet	2	-2821.452866	-2297.845868	-959.3046428	EMD2
ATGGGTTTCTCAACCC-1	singlet	2	-785.265618	-650.4641357	-257.6041577	EMD2
ATGGTTGAGACGGAAA-1	singlet	1	-1024.209547	-383.6468491	-837.5479758	EMD2
ATGGTTGAGGTAACTA-1	singlet	1	-523.0750412	-177.4966646	-411.7007127	EMD2
ATGGTTGCAAAGCAAT-1	singlet	0	-817.446382	-2489.320621	-2327.058812	EMD2
ATGGTTGCAAATGGTA-1	singlet	1	-1515.176896	-546.4972811	-1322.882224	EMD2
ATGGTTGCACAAGTGG-1	singlet	2	-224.4856079	-202.1923569	-74.60902595	EMD2
ATGGTTGCACCAGTTA-1	singlet	1	-412.1021121	-146.0678923	-351.1858093	EMD2
ATGGTTGCAGAAATTG-1	singlet	1	-4187.703494	-1339.495063	-4148.124895	EMD2
ATGGTTGCATGCCATA-1	singlet	2	-1485.564117	-1414.46275	-517.2190533	EMD2
ATGGTTGGTACCGGCT-1	singlet	0	-200.0374797	-571.9075176	-533.9224351	EMD2
ATGGTTGGTCACCTTC-1	singlet	1	-4360.568774	-1463.336743	-3424.838457	EMD2
ATGGTTGGTGACTGTT-1	singlet	2	-185.1434734	-205.9420715	-58.16377546	EMD2
ATGGTTGGTGCAATAA-1	singlet	1	-2150.617904	-791.751683	-1822.537544	EMD2
ATGGTTGGTTGCGGCT-1	singlet	0	-743.7042833	-2351.046651	-2170.844799	EMD2
ATGGTTGTCACCTCAC-1	singlet	0	-1017.130489	-2832.830719	-2697.426963	EMD2
ATGGTTGTCAGCCTTC-1	singlet	0	-520.9253711	-1499.18435	-1578.707163	EMD2
ATGTCCCAGAGAGTGA-1	singlet	0	-332.8373696	-1675.734501	-1535.501864	EMD2
ATGTCCCAGCACCCAC-1	singlet	0	-196.3563972	-540.7288153	-599.8567874	EMD2
ATGTCCCCAGCGAGTA-1	singlet	1	-371.2171492	-114.268749	-310.1625304	EMD2
ATGTCCCGTCAATGGG-1	singlet	1	-359.6964267	-138.684971	-332.1281277	EMD2
ATGTCCCGTGTCTTAG-1	doublet	2/0	-3742.007814	-5029.426459	-3485.671769	EMD2
ATGTCCCGTGTGGTCC-1	singlet	0	-943.0774482	-2875.711172	-2700.488215	EMD2
ATGTCCCGTTGGTAGG-1	singlet	1	-362.3165015	-177.0081453	-321.679894	EMD2
ATGTCCCTCAAGCCCG-1	singlet	1	-578.0282908	-211.6572153	-514.5079466	EMD2
ATGTCCCTCATGCAGT-1	singlet	1	-2361.675531	-573.7167054	-2157.133199	EMD2
ATGTCCCTCTTCGTAT-1	singlet	1	-412.6265684	-149.7456331	-391.0618192	EMD2
ATGTCTTAGAATTTGG-1	singlet	2	-941.8432859	-907.3074159	-416.3297929	EMD2
ATGTCTTAGCTAGTTC-1	singlet	1	-2266.992841	-779.1728686	-1871.20076	EMD2
ATGTCTTCACTTGAAC-1	singlet	2	-2400.788873	-2086.876358	-793.5272361	EMD2
ATGTCTTCAGAGTGAC-1	singlet	1	-333.1650659	-111.0881198	-271.0586095	EMD2
ATGTCTTCATCAACCA-1	singlet	0	-2358.140175	-8428.680378	-7998.50325	EMD2
ATGTCTTGTCCGGTCA-1	singlet	0	-1466.015755	-5240.76482	-5146.335798	EMD2
ATGTCTTGTGAGGAAA-1	singlet	1	-358.5398921	-140.5294332	-312.9314139	EMD2
ATGTCTTTCACACCGG-1	singlet	0	-1079.23813	-3190.701767	-3127.8248	EMD2
ATGTCTTTCACGGAGA-1	singlet	2	-221.2575138	-175.6232104	-87.55055534	EMD2
ATGTCTTTCCACAAGT-1	singlet	1	-388.3686166	-160.215595	-338.229101	EMD2
ATGTCTTTCCTCAGAA-1	singlet	1	-1406.3983	-579.9010819	-1166.905983	EMD2
ATGTCTTTCTGTCGCT-1	singlet	1	-2399.64968	-779.8955108	-2122.052714	EMD2
ATGTCTTTCTTGGAAC-1	singlet	0	-1568.163905	-6301.467522	-5852.392898	EMD2
ATTACCTAGCATACTC-1	singlet	1	-2359.263231	-792.9591734	-1864.57498	EMD2
ATTACCTAGCATGAAT-1	singlet	1	-468.8809638	-186.999448	-406.7015192	EMD2
ATTACCTAGCCGATAG-1	singlet	1	-1101.921649	-363.9109227	-909.3389179	EMD2
ATTACCTCAGATCACT-1	singlet	1	-691.0791455	-257.7870836	-610.6697368	EMD2
ATTACCTGTACAGTCT-1	singlet	2	-153.9127857	-103.9999386	-49.25141418	EMD2
ATTACCTGTATACCCA-1	singlet	0	-1029.018497	-2808.59654	-2709.687946	EMD2
ATTACCTGTATTAAGG-1	singlet	1	-1672.340128	-596.4408839	-1289.953162	EMD2
ATTACCTGTGATTAGA-1	singlet	0	-792.6342554	-2399.16536	-2405.652091	EMD2
ATTACCTTCTAACACG-1	singlet	0	-240.7525641	-845.1244421	-729.4357562	EMD2
ATTACCTTCTGTCGTC-1	singlet	2	-1606.528926	-1471.810883	-682.8470841	EMD2
ATTACTCAGAATTCAG-1	singlet	1	-5444.572939	-2010.348218	-4525.59923	EMD2
ATTACTCAGCAACAGC-1	singlet	1	-993.8222324	-378.1138706	-807.8848635	EMD2
ATTACTCAGCCTCGTG-1	singlet	1	-588.2021203	-169.9027977	-419.0083264	EMD2
ATTACTCCAACGGGTA-1	singlet	1	-1178.371135	-299.9235454	-1056.769143	EMD2
ATTACTCCATGTTACG-1	singlet	0	-1207.13595	-3977.805445	-3650.053929	EMD2
ATTACTCGTACCGTGC-1	singlet	2	-516.516642	-498.8042686	-226.0611041	EMD2
ATTACTCTCCGGACTG-1	singlet	0	-600.2297328	-2142.740393	-1832.304523	EMD2
ATTACTCTCGATCCAA-1	singlet	2	-353.9985611	-295.813966	-139.9036239	EMD2
ATTATCCAGCGAAACC-1	singlet	2	-556.3972396	-459.8675484	-215.452051	EMD2
ATTATCCAGCTTGTTG-1	singlet	1	-315.5192675	-116.7946031	-265.5024886	EMD2
ATTATCCAGTCATGGG-1	doublet	2/0	-11.31681448	-10.40206162	-11.08721135	EMD2
ATTATCCCATCCTCAC-1	singlet	1	-280.0415506	-126.9873122	-267.7832791	EMD2
ATTATCCCATTCACCC-1	singlet	1	-180.1962266	-94.60958536	-222.422711	EMD2
ATTATCCGTAAGGCTG-1	singlet	0	-1242.334768	-4669.177958	-4703.953518	EMD2
ATTATCCGTCAAGTTC-1	singlet	1	-1365.511819	-504.242391	-1244.884353	EMD2
ATTATCCGTGAGGATC-1	singlet	0	-299.4218369	-934.8085117	-911.1779827	EMD2
ATTATCCGTGCCTTCT-1	singlet	1	-3505.688653	-1065.857167	-3050.752218	EMD2
ATTATCCTCAGACAAA-1	singlet	1	-337.4962856	-152.2725722	-268.0232645	EMD2
ATTATCCTCTTCTGGC-1	singlet	2	-3075.375612	-2577.058171	-1143.945525	EMD2
ATTCACTAGTAGACAT-1	singlet	1	-381.9864999	-136.0477536	-291.9354285	EMD2
ATTCACTAGTCGCCAC-1	singlet	1	-223.1707244	-50.12121483	-119.1670379	EMD2
ATTCACTCAAGTGACG-1	singlet	2	-1297.89235	-1304.161209	-539.5484042	EMD2
ATTCACTCACCTCAGG-1	singlet	1	-400.5738608	-177.7690353	-345.7347137	EMD2
ATTCACTGTACTTCCC-1	singlet	1	-2556.101418	-865.5356215	-2107.234098	EMD2
ATTCACTGTAGAGCTG-1	singlet	2	-2905.840354	-2516.927135	-867.9286053	EMD2
ATTCACTGTGAACGGT-1	singlet	0	-90.09610812	-170.7148651	-184.9729744	EMD2
ATTCACTGTGGCTAGA-1	singlet	0	-2384.974079	-6928.36509	-6743.80694	EMD2
ATTCACTGTGGCTTAT-1	singlet	1	-815.4217509	-218.2012618	-739.1279088	EMD2
ATTCACTTCATGGTAC-1	singlet	1	-1907.743614	-451.5775649	-1655.475931	EMD2
ATTCACTTCTGCCCTA-1	singlet	1	-1548.721817	-498.8044251	-1229.053822	EMD2
ATTCAGGAGACATAGT-1	singlet	1	-1249.528194	-443.6187832	-1053.671172	EMD2
ATTCAGGAGCTTCGTA-1	singlet	1	-997.076361	-302.669416	-889.494602	EMD2
ATTCAGGAGTTATGGA-1	singlet	1	-3010.672548	-1070.057282	-2502.709277	EMD2
ATTCAGGCACTCTGCT-1	singlet	1	-3712.819834	-1112.460973	-2896.585144	EMD2
ATTCAGGCAGGTGACA-1	singlet	0	-2353.729392	-8755.019975	-8215.023754	EMD2
ATTCAGGGTCCTTGTC-1	singlet	0	-61.60783187	-198.8431602	-157.6383401	EMD2
ATTCAGGGTCGCGGTT-1	singlet	0	-172.9220881	-521.5246247	-518.9262421	EMD2
ATTCAGGGTGAGCCAA-1	singlet	1	-592.979234	-253.0431984	-512.5132404	EMD2
ATTCAGGTCAGGAACG-1	singlet	1	-347.8745646	-154.2890399	-351.6419092	EMD2
ATTCAGGTCGTAACTG-1	singlet	1	-1418.224059	-483.0253797	-1072.450502	EMD2
ATTCAGGTCGTCGCTT-1	singlet	1	-2112.319724	-711.3943292	-1734.09492	EMD2

ATTCAGGTCTGGGAGA-1	singlet	0	-812.7817826	-2237.640514	-2087.403841	EMD2
ATTCATCAGCGCTGCT-1	singlet	1	-198.4133893	-99.32197162	-154.8612892	EMD2
ATTCATCAGGGCCTCT-1	singlet	1	-3676.623657	-1201.564822	-3117.887589	EMD2
ATTCATCCACTAACCA-1	singlet	0	-288.9485467	-908.6102821	-879.5162061	EMD2
ATTCATCCACTTCCTG-1	singlet	1	-1399.238832	-543.0732606	-1276.402702	EMD2
ATTCATCCATGGAACG-1	singlet	1	-2003.422588	-711.8087331	-1868.061326	EMD2
ATTCATCGTCGCGTCA-1	singlet	1	-1535.608958	-484.9495059	-1328.283494	EMD2
ATTCATCTCAAGCTTG-1	singlet	1	-2817.782599	-993.5087941	-2443.85785	EMD2
ATTCATCTCCAACTGA-1	singlet	1	-147.7303259	-70.14711908	-120.3563634	EMD2
ATTCCATAGATGCAGC-1	singlet	2	-3905.94753	-3268.946027	-1296.209058	EMD2
ATTCCATAGTGACCTT-1	singlet	1	-1058.878033	-397.5483192	-896.5246276	EMD2
ATTCCATAGTTGAAGT-1	singlet	0	-108.8283614	-296.4033633	-242.5172164	EMD2
ATTCCATCACATGGTT-1	singlet	2	-986.1356526	-789.6867676	-369.4741256	EMD2
ATTCCATCACCAGCCA-1	singlet	1	-2041.757212	-630.7415353	-1657.714592	EMD2
ATTCCATGTATCCCTC-1	singlet	1	-1454.076201	-394.7164705	-1270.399932	EMD2
ATTCCATGTCACTAGT-1	doublet	0/2	-2435.564697	-4560.66176	-3509.908783	EMD2
ATTCCATGTCCAAATC-1	singlet	0	-810.0765784	-2435.771039	-2451.559587	EMD2
ATTCCATGTTGTTGTG-1	singlet	0	-671.9017596	-1708.348014	-1735.25676	EMD2
ATTCCATTCGACATAC-1	singlet	2	-668.8125665	-592.0492506	-287.9764764	EMD2
ATTCCATTCTCTTCAA-1	singlet	1	-4062.427224	-909.4363396	-2782.693917	EMD2
ATTCCATTCTTCGGAA-1	singlet	1	-1756.969033	-413.9765755	-1590.0558	EMD2
ATTCCCGAGACCTTTG-1	singlet	0	-408.2910342	-1221.156713	-1085.801623	EMD2
ATTCCCGAGATGACAT-1	singlet	2	-1823.053505	-1658.242171	-578.5510798	EMD2
ATTCCCGAGCCATCCG-1	singlet	1	-1366.338211	-346.1336491	-910.1098692	EMD2
ATTCCCGAGTACCATC-1	singlet	2	-2371.6336	-2235.713387	-840.3229607	EMD2
ATTCCCGCACTTGTCC-1	singlet	1	-1993.644601	-728.0590012	-1565.61561	EMD2
ATTCCCGCAGCAGAAC-1	singlet	1	-2433.539546	-865.8992144	-2097.626896	EMD2
ATTCCCGGTGGCTTGC-1	singlet	2	-328.0396445	-287.3994066	-138.165659	EMD2
ATTCCCGGTGGTAACG-1	singlet	1	-2759.412478	-948.4030384	-2351.918606	EMD2
ATTCCCGGTTGCCATA-1	singlet	1	-2208.137808	-733.9890878	-1882.945047	EMD2
ATTCCCGGTTGGGTAG-1	singlet	1	-279.3172686	-132.8341632	-277.2336112	EMD2
ATTCCCGTCATTCACT-1	singlet	0	-1298.478899	-4019.326376	-3798.839543	EMD2
ATTCCCGTCGCGATCG-1	singlet	1	-1878.882193	-628.5771938	-1422.92455	EMD2
ATTCCCGTCGGAGATG-1	singlet	1	-1027.258113	-361.7598783	-884.5490875	EMD2
ATTCCCGTCGTTCGCT-1	singlet	0	-476.5406622	-1394.989632	-1374.966897	EMD2
ATTCCCGTCTAGTCAG-1	singlet	1	-2851.410544	-881.2102793	-2147.63377	EMD2
ATTCCCGTCTCCGAGG-1	singlet	1	-1795.181397	-542.5102706	-1523.638562	EMD2
ATTCCTAAGAGGTTTA-1	singlet	0	-1107.08723	-3236.098379	-3075.243444	EMD2
ATTCCTAAGCTACAAA-1	singlet	1	-665.7100323	-254.4790534	-502.5266165	EMD2
ATTCCTAAGCTTCGTA-1	singlet	1	-330.7922099	-150.1125119	-303.5177016	EMD2
ATTCCTACAAAGCAAT-1	singlet	2	-319.4260651	-304.6807111	-151.8980164	EMD2
ATTCCTAGTTCCGCGA-1	singlet	1	-23.84401057	-10.50515091	-15.57154687	EMD2
ATTCCTATCATGCAGT-1	singlet	1	-354.0257268	-154.6652146	-346.217576	EMD2
ATTCCTATCGAAGTGG-1	singlet	0	-346.7021124	-1131.214728	-992.5224783	EMD2
ATTCGTTAGGTCGCCT-1	singlet	0	-1458.853693	-5511.957527	-5908.414276	EMD2
ATTCGTTCAAGGTACG-1	singlet	1	-5297.390704	-1551.639249	-4261.095132	EMD2
ATTCGTTCAATTTCCT-1	singlet	1	-800.4815924	-351.1698657	-764.3365254	EMD2
ATTCGTTCACAAGCCC-1	singlet	1	-1707.655588	-578.6076105	-1270.988587	EMD2
ATTCGTTCAGAGGGTT-1	singlet	1	-420.6422668	-120.4694401	-248.2574477	EMD2
ATTCGTTCAGCGGTCT-1	singlet	0	-109.3865903	-390.5764573	-345.3661606	EMD2
ATTCGTTGTGTTGATC-1	singlet	2	-1162.922761	-906.7829994	-377.2119418	EMD2
ATTCGTTGTTCCTTGC-1	singlet	1	-1207.028632	-438.3067248	-1155.442341	EMD2
ATTCGTTGTTCTGACA-1	singlet	0	-618.3351643	-1792.313791	-1716.09774	EMD2
ATTCTACAGGCATCAG-1	singlet	1	-2041.067226	-703.6165327	-1604.241931	EMD2
ATTCTACAGGCTCAAG-1	singlet	0	-900.5848778	-2986.191474	-2776.092612	EMD2
ATTCTACAGGCTCCCA-1	singlet	1	-713.8955416	-318.2791252	-633.1713045	EMD2
ATTCTACCACGCGTCA-1	singlet	1	-2810.464824	-749.5360436	-2227.912476	EMD2
ATTCTACGTACAAAGT-1	singlet	2	-2031.396197	-1833.816454	-774.6861163	EMD2
ATTCTACGTACCTGTA-1	singlet	1	-447.8718656	-199.0130281	-401.9283386	EMD2
ATTCTACGTTATGTCG-1	singlet	2	-386.7999397	-317.2261384	-133.5681629	EMD2
ATTCTACTCCCATAAG-1	singlet	2	-3862.513808	-3044.101326	-1254.850559	EMD2
ATTCTACTCCCGTAAA-1	singlet	0	-1341.607055	-4950.036937	-4575.309575	EMD2
ATTCTACTCTATGTGG-1	singlet	2	-3691.500492	-3492.299434	-1236.345957	EMD2
ATTCTACTCTTCCCGA-1	singlet	0	-1707.082825	-3596.156834	-3654.525593	EMD2
ATTCTTGAGATGATTG-1	singlet	1	-523.8607756	-238.628126	-438.5056538	EMD2
ATTCTTGAGGATTCAA-1	singlet	2	-342.4906299	-338.6999366	-149.1104381	EMD2
ATTCTTGCAAACCGGA-1	singlet	2	-2454.407037	-2027.498747	-786.8191373	EMD2
ATTCTTGCACGGTGTC-1	singlet	1	-1545.443095	-449.8832315	-1327.220368	EMD2
ATTCTTGCACTACTTT-1	singlet	2	-728.4649265	-678.7958625	-282.3209925	EMD2
ATTCTTGGTCTAATCG-1	singlet	2	-4243.265679	-3385.705677	-1269.187893	EMD2
ATTCTTGGTTATGTGC-1	singlet	2	-3095.53746	-2500.496022	-1046.286242	EMD2
ATTGGGTAGTTACGAA-1	singlet	1	-436.8231818	-172.5401518	-361.1522933	EMD2
ATTGGGTAGTTTGGCT-1	singlet	1	-461.2879407	-180.5614311	-397.1660334	EMD2
ATTGGGTCACACTTAG-1	singlet	1	-1157.11944	-435.4842832	-943.7214077	EMD2
ATTGGGTCACCCTAGG-1	singlet	1	-4502.114841	-1409.900479	-3243.675791	EMD2
ATTGGGTCAGTACTAC-1	singlet	0	-1266.406512	-3517.449341	-3416.160785	EMD2
ATTGGGTGTTACCGTA-1	doublet	0/2	-5234.501641	-9077.768786	-7427.710728	EMD2
ATTGGGTTCACCATCC-1	singlet	2	-8761.193961	-6943.944772	-1814.487104	EMD2
ATTGGGTTCCAACACA-1	singlet	1	-560.719959	-189.5705397	-435.844467	EMD2
ATTGGGTTCCCATAAG-1	singlet	1	-695.3700374	-246.5512178	-489.537455	EMD2
ATTGGGTTCCGAGAAG-1	singlet	2	-1221.325124	-1259.871846	-416.8134799	EMD2
ATTGGGTTCGTTCAGA-1	singlet	2	-891.1164511	-885.3927414	-327.446736	EMD2
ATTGGGTTCTTAGGAC-1	doublet	45293	-3776.458165	-2214.136558	-2663.960516	EMD2
ATTGTTCAGTATGTAG-1	singlet	1	-406.3345968	-128.9246139	-281.8421274	EMD2
ATTGTTCCACCTGCGA-1	singlet	1	-800.7487382	-285.9760576	-665.1321004	EMD2
ATTGTTCGTAACGCGA-1	singlet	1	-1358.149683	-457.3351576	-1224.706574	EMD2
ATTGTTCGTTGCCATA-1	singlet	2	-5510.911868	-3917.375031	-2306.451301	EMD2
ATTGTTCTCCGTCCTA-1	singlet	0	-653.1291036	-2054.805508	-2044.497673	EMD2
ATTTACCAGAAATGGG-1	singlet	1	-1150.721182	-418.3458947	-1020.65401	EMD2
ATTTACCAGGTCATAA-1	singlet	2	-1501.171101	-1135.956624	-465.9760236	EMD2
ATTTACCAGTTGAATG-1	singlet	2	-1935.541643	-1827.029751	-537.2014137	EMD2
ATTTACCCAATTGAGA-1	singlet	2	-8217.355956	-6995.507834	-2664.590191	EMD2
ATTTACCCACATCATG-1	doublet	45323	-3169.862609	-1987.102788	-1439.896754	EMD2
ATTTACCCAGCGAGTA-1	singlet	1	-3350.64525	-1181.045616	-2884.653889	EMD2
ATTTACCGTAACAGGC-1	singlet	2	-767.6100099	-628.0124877	-245.3648648	EMD2
ATTTACCGTCGCATCG-1	singlet	2	-98.46291031	-79.91269275	-24.39692279	EMD2
ATTTACCGTGTTCATG-1	singlet	1	-1039.206924	-332.7898005	-850.5813282	EMD2
ATTTACCTCGAACCTA-1	singlet	2	-373.4319259	-320.1904719	-120.3817106	EMD2
ATTTACCTCGCTACAA-1	singlet	0	-483.0539327	-1260.846919	-1218.331289	EMD2
ATTTACCTCTCCGATC-1	singlet	1	-2105.290691	-721.1848494	-1951.902135	EMD2
ATTTCACAGCCGTAAG-1	doublet	1/0	-1624.606445	-1455.569485	-1999.748813	EMD2
ATTTCACAGGACTTCT-1	singlet	2	-5660.067663	-4164.089145	-1233.126867	EMD2
ATTTCACAGTTACGGG-1	singlet	1	-1750.426837	-577.7983668	-1482.660523	EMD2
ATTTCACAGTTCCGGC-1	singlet	1	-1918.955738	-668.7980648	-1455.041971	EMD2
ATTTCACCACGGTGCT-1	singlet	2	-3704.939672	-2999.648466	-1250.751923	EMD2
ATTTCACCACTACTTT-1	singlet	2	-2028.298502	-1829.687654	-737.4114806	EMD2
ATTTCACCAGTGCCTG-1	singlet	0	-573.9539237	-1947.190564	-1750.474081	EMD2
ATTTCACCATCCGAAT-1	singlet	1	-490.3002275	-160.9750244	-358.883733	EMD2
ATTTCACCATCTTCGC-1	singlet	1	-1528.989834	-459.5425518	-1299.004393	EMD2
ATTTCACGTAATGATG-1	singlet	1	-623.7186827	-211.5336884	-513.0614853	EMD2

ATTTCACGTAGCTCGC-1	singlet	0	-70.44391669	-241.7695836	-192.537792	EMD2
ATTTCACGTCTCGACG-1	singlet	1	-370.0105077	-144.1148303	-304.6782526	EMD2
ATTTCACGTGATAGTA-1	singlet	1	-183.0089692	-60.60819786	-137.2395054	EMD2
ATTTCACGTGCGAACA-1	singlet	0	-63.25681404	-238.2967537	-213.0116631	EMD2
ATTTCACTCAACCTCC-1	singlet	1	-3304.902514	-1081.3346	-2502.40706	EMD2
ATTTCACTCGTGTTCC-1	singlet	1	-2512.525622	-673.5368622	-1955.120268	EMD2
ATTTCTGAGTGGGAAA-1	singlet	2	-2393.110303	-1863.493153	-777.245563	EMD2
ATTTCTGCAATAAGGT-1	singlet	1	-456.4946516	-204.5366993	-411.036064	EMD2
ATTTCTGCAATAGTGA-1	singlet	1	-847.4429422	-300.2062404	-732.9684785	EMD2
ATTTCTGCACACGGTC-1	singlet	1	-1239.336181	-458.4016237	-1073.931888	EMD2
ATTTCTGCATTATGCG-1	singlet	1	-28.60501914	-7.563994193	-20.13729961	EMD2
ATTTCTGGTAGAGATT-1	singlet	0	-254.4822343	-756.1422973	-646.8672305	EMD2
ATTTCTGGTCAGGCAA-1	singlet	2	-1993.923464	-1716.560363	-696.1075819	EMD2
ATTTCTGTCCGTATAG-1	singlet	1	-1077.282664	-317.980689	-709.9429563	EMD2
ATTTCTGTCGAGTCTA-1	singlet	1	-2805.03342	-781.0844261	-2084.760309	EMD2
ATTTCTGTCGCGTGAC-1	singlet	1	-1112.096095	-330.0244856	-866.6308624	EMD2
ATTTCTGTCGGACCAC-1	singlet	1	-370.6960842	-167.8278957	-374.6147895	EMD2
CAAAGAAAGGCTTCCG-1	doublet	1/0	-746.7688842	-625.4703877	-866.6939863	EMD2
CAAAGAACAGCGTTGC-1	singlet	1	-1006.116561	-292.4807312	-803.5771979	EMD2
CAAAGAACATGCCGAC-1	singlet	1	-2082.073457	-687.7084482	-1696.646574	EMD2
CAAAGAAGTAGCCCTG-1	singlet	2	-2634.839236	-2180.484022	-928.708718	EMD2
CAAAGAAGTGGTGATG-1	singlet	1	-262.4639758	-123.2946735	-223.6558009	EMD2
CAAAGAATCAAGCGTT-1	singlet	1	-4222.40916	-1331.317671	-3173.527048	EMD2
CAAAGAATCGGAGTAG-1	singlet	1	-1055.06406	-300.7663206	-905.6472587	EMD2
CAACAACAGAAACACT-1	singlet	2	-2974.980355	-2778.542802	-1042.19284	EMD2
CAACAACAGAAGTGTT-1	singlet	1	-2363.132665	-861.6945854	-2047.723228	EMD2
CAACAACAGGATGGCT-1	singlet	2	-3083.085835	-2592.7869	-1062.790772	EMD2
CAACAACAGGCTTAGG-1	singlet	1	-532.7510743	-206.3906672	-453.6243132	EMD2
CAACAACAGTATGACA-1	singlet	0	-232.6480376	-609.481804	-582.5890876	EMD2
CAACAACCAAGTTCCA-1	singlet	1	-523.3253742	-251.1336301	-489.9447033	EMD2
CAACAACCACGGTCTG-1	singlet	1	-3103.154076	-1050.600684	-2652.684729	EMD2
CAACAACTCCATCTGC-1	singlet	2	-2529.44056	-1943.396228	-713.6324579	EMD2
CAACAACTCTCCCATG-1	singlet	2	-2246.431801	-1787.096177	-674.7970198	EMD2
CAACAACTCTGGCCTT-1	singlet	1	-515.2679291	-181.2541553	-403.4885493	EMD2
CAACAGTAGCACCAGA-1	singlet	0	-1502.967426	-5167.529548	-4741.760935	EMD2
CAACAGTAGGCGAAGG-1	singlet	0	-1406.736778	-3857.834573	-3711.664533	EMD2
CAACAGTAGTACCGGA-1	singlet	1	-751.1872983	-294.2417209	-589.0123768	EMD2
CAACAGTCAATTCTCT-1	singlet	1	-3507.393236	-1087.903999	-3058.651811	EMD2
CAACAGTCAGTAACAA-1	singlet	2	-5028.759093	-4065.910956	-1695.136892	EMD2
CAACAGTCATAGGTAA-1	singlet	1	-3841.408917	-1067.433342	-2908.370208	EMD2
CAACAGTGTACGATCT-1	singlet	0	-1181.058673	-3583.599172	-3468.707423	EMD2
CAACAGTGTAGGCTCC-1	singlet	2	-384.411196	-363.6673559	-152.4732289	EMD2
CAACAGTGTCTAGATC-1	singlet	0	-148.0269386	-475.5939632	-413.1622565	EMD2
CAACAGTTCCTACGGG-1	singlet	1	-2271.791757	-779.2284937	-1988.985325	EMD2
CAACAGTTCGAGCACC-1	doublet	2/0	-229.1881867	-242.5395675	-165.259095	EMD2
CAACAGTTCGTAGAGG-1	singlet	1	-1083.776851	-393.1861002	-852.6790453	EMD2
CAACAGTTCTCGCCTA-1	singlet	1	-1654.710143	-525.8555775	-1341.602433	EMD2
CAACAGTTCTGCAGCG-1	singlet	0	-303.3458001	-993.0714977	-839.4986608	EMD2
CAACCAAAGAGAAGGT-1	singlet	1	-412.6557973	-157.6334457	-339.7827605	EMD2
CAACCAAAGCATCAAA-1	singlet	1	-1544.589364	-546.2119756	-1356.392353	EMD2
CAACCAAAGCGCGTTC-1	unassigned	0/2	-4.406907112	-5.398181185	-4.605398405	EMD2
CAACCAACATCCCGTT-1	singlet	1	-2636.994815	-1063.193832	-2251.827072	EMD2
CAACCAAGTCGCTTGG-1	singlet	1	-3646.271149	-1109.987636	-2849.16517	EMD2
CAACCAAGTGTCTTGA-1	unassigned	1	-4.325313951	-1.549395288	-1.785271738	EMD2
CAACCAAGTTATGTCG-1	singlet	0	-922.6929759	-3445.35681	-3281.351962	EMD2
CAACCAAGTTTATGCG-1	singlet	1	-411.2036518	-167.705712	-355.9952959	EMD2
CAACCAATCAAGGCTT-1	singlet	0	-900.1078089	-2981.254491	-2766.574833	EMD2
CAACCAATCGTTGTGA-1	singlet	0	-441.0799876	-1170.986779	-1201.198449	EMD2
CAACCAATCTAGTGAC-1	singlet	1	-4434.638767	-1342.004833	-3620.589517	EMD2
CAACCAATCTCTATGT-1	singlet	2	-536.3566543	-500.7058009	-218.3728064	EMD2
CAACCAATCTGCCCTA-1	singlet	2	-5687.460795	-4435.22187	-1572.877192	EMD2
CAACCAATCTGGCCAG-1	singlet	0	-495.1421643	-1515.676776	-1298.197528	EMD2
CAACCTCCAGCCGTCA-1	singlet	0	-819.8375772	-2507.74256	-2349.875098	EMD2
CAACCTCCATCATCTT-1	singlet	1	-2091.302757	-677.9514829	-1628.724959	EMD2
CAACCTCCATCTCCCA-1	singlet	1	-3455.01456	-1031.749513	-2699.816486	EMD2
CAACCTCCATGACTAC-1	singlet	2	-1840.448121	-1777.755649	-693.0590647	EMD2
CAACCTCGTCCCTGTT-1	singlet	0	-73.7640053	-166.2094989	-196.7532063	EMD2
CAACCTCGTCTCACGG-1	singlet	1	-776.3788562	-301.2411487	-650.3935526	EMD2
CAACCTCGTGCTAGCC-1	singlet	1	-1733.612539	-623.2564322	-1394.268533	EMD2
CAACCTCGTGCTGATT-1	singlet	1	-307.9363092	-110.6682446	-231.5522921	EMD2
CAACCTCTCGAACCTA-1	singlet	2	-1267.979313	-1211.04886	-453.9779708	EMD2
CAACGATAGACGCCCT-1	singlet	1	-435.2008431	-188.4816275	-357.7976291	EMD2
CAACGATAGCGCCGTT-1	singlet	1	-306.1395513	-133.8516182	-284.377651	EMD2
CAACGATAGGCCACTC-1	singlet	1	-1520.940269	-492.6218063	-1178.58699	EMD2
CAACGATAGTTTGAGA-1	singlet	1	-1246.007004	-445.0414154	-1017.577741	EMD2
CAACGATCACCGTACG-1	singlet	1	-438.8583443	-182.4767911	-407.1747003	EMD2
CAACGATCACTGAGTT-1	singlet	1	-3429.038252	-869.953775	-2511.623945	EMD2
CAACGATCATTAAAGG-1	singlet	0	-327.0506829	-869.4951143	-813.9866868	EMD2
CAACGATGTATCCTCC-1	singlet	1	-395.3778536	-155.5211577	-316.8153947	EMD2
CAACGATGTCGCACAC-1	singlet	1	-1786.591266	-588.262428	-1641.83376	EMD2
CAACGATGTGGTATGG-1	singlet	1	-317.4537511	-110.7262152	-246.8209507	EMD2
CAACGATGTTGTCATG-1	singlet	2	-3535.996303	-2942.888386	-837.2060522	EMD2
CAACGATTCCTGCCAT-1	singlet	2	-1111.876776	-882.6666584	-445.0977007	EMD2
CAACGATTCTAAACGC-1	singlet	1	-781.531324	-338.7079109	-630.3701587	EMD2
CAACGGCAGACAACAT-1	singlet	2	-2468.480252	-2058.069327	-808.7722669	EMD2
CAACGGCAGCTACAAA-1	singlet	1	-240.5503989	-113.9529355	-201.5362813	EMD2
CAACGGCAGTGCACTT-1	singlet	1	-173.4665324	-67.17317768	-168.8199123	EMD2
CAACGGCCAAGATGGC-1	singlet	1	-1662.933203	-483.4215557	-1353.152107	EMD2
CAACGGCCAGCGTGAA-1	singlet	2	-2257.155736	-1781.933535	-685.4854076	EMD2
CAACGGCCAGTGTATC-1	singlet	2	-48.09799319	-44.31025331	-16.30580693	EMD2
CAACGGCCATGCAGCC-1	singlet	1	-340.427657	-138.2456455	-253.4756318	EMD2
CAACGGCGTAAGTCAA-1	singlet	2	-176.2050408	-110.2027304	-61.85397097	EMD2
CAACGGCTCATTGCTT-1	singlet	0	-464.2921301	-1579.592712	-1477.338402	EMD2
CAAGACTAGGTAGATT-1	singlet	1	-283.9330168	-106.7092315	-208.448298	EMD2
CAAGACTAGTGAGGCT-1	singlet	2	-2774.023026	-2054.221343	-749.6044773	EMD2
CAAGACTCACCTAAAC-1	singlet	1	-3787.91135	-1167.75898	-3363.410255	EMD2
CAAGACTCAGACGGAT-1	singlet	0	-394.6929001	-1059.995003	-1001.628527	EMD2
CAAGACTCAGGTGGAT-1	singlet	1	-2540.409694	-851.1779971	-2160.839207	EMD2
CAAGACTGTATGACAA-1	singlet	1	-2959.762923	-1035.820871	-2496.911687	EMD2
CAAGACTGTCACATTG-1	singlet	2	-361.78982	-410.8744693	-210.4972689	EMD2
CAAGACTGTTAGGGTG-1	unassigned	1/0	-2.173156714	-1.203228582	-2.650678066	EMD2
CAAGACTTCAAGCCTA-1	singlet	0	-915.6279343	-2370.213871	-2471.975701	EMD2
CAAGACTTCCCATTCG-1	singlet	1	-140.0404403	-35.83098038	-96.03626944	EMD2
CAAGACTTCGAGTCCG-1	singlet	1	-189.6356944	-69.05714191	-159.8239179	EMD2
CAAGAGGCAGAGAAAG-1	singlet	0	-934.3242923	-2762.228547	-2808.795895	EMD2
CAAGAGGGTAGAGCTG-1	singlet	2	-2186.611648	-1849.75609	-839.595242	EMD2
CAAGAGGGTATCGTTG-1	singlet	1	-2270.235217	-715.3453473	-1743.365607	EMD2
CAAGAGGGTCTGCAAT-1	singlet	2	-1015.981239	-908.2594194	-357.3862162	EMD2
CAAGAGGTCATCGGGC-1	singlet	2	-835.004383	-838.9636558	-319.2733664	EMD2
CAAGAGGTCCCGAGAC-1	singlet	2	-4276.847156	-3370.740949	-1298.296736	EMD2

CAAGAGGTCGACGTCG-1	singlet	0	-122.9849682	-436.0205095	-379.9700563	EMD2
CAAGAGGTCGTGCACG-1	singlet	0	-140.9186657	-419.1633263	-385.9819787	EMD2
CAAGCTAAGCCTCTGG-1	singlet	0	-414.9198342	-1300.447013	-1172.331236	EMD2
CAAGCTAAGTCACGCC-1	singlet	1	-2530.754154	-781.1371888	-2193.682182	EMD2
CAAGCTACAACGGCCT-1	singlet	0	-279.7105304	-917.2951194	-771.662097	EMD2
CAAGCTACAATAGTCC-1	singlet	2	-224.0363751	-178.4568236	-62.76323649	EMD2
CAAGCTAGTATGAAAC-1	singlet	1	-2134.551199	-628.4173516	-1849.626392	EMD2
CAAGCTAGTCCATACA-1	singlet	1	-249.1905416	-85.2030875	-243.1470495	EMD2
CAAGCTAGTTCGTAAC-1	singlet	2	-904.8081809	-867.8249709	-383.2122614	EMD2
CAAGCTATCAGAGCAG-1	singlet	1	-265.6768491	-104.5451467	-213.0202299	EMD2
CAAGCTATCGCGCCAA-1	singlet	1	-4750.720335	-1227.581456	-3723.129299	EMD2
CAAGGGACACGCGCTA-1	singlet	1	-3423.535287	-1057.503462	-2588.058851	EMD2
CAAGGGACACTACCGG-1	singlet	1	-336.117148	-125.991523	-315.6334398	EMD2
CAAGGGACAGCGTACC-1	singlet	1	-919.2644582	-305.9939719	-789.1734384	EMD2
CAAGGGACAGTATACC-1	singlet	0	-292.219546	-631.3440217	-687.8254671	EMD2
CAAGGGACAGTGCCTG-1	unassigned	2	-5.229141625	-5.695885847	-3.391105815	EMD2
CAAGGGAGTGAACGGT-1	singlet	1	-279.5125722	-103.05242	-224.967214	EMD2
CAAGGGAGTTGGAGGT-1	singlet	1	-2679.945427	-798.054327	-1981.296921	EMD2
CAAGGGATCGGACTGC-1	singlet	1	-545.8596971	-195.4400489	-397.2592135	EMD2
CAATACGAGCTAATCC-1	doublet	45293	-20.70098237	-15.22873981	-17.52750273	EMD2
CAATACGCAACAGCTT-1	singlet	0	-742.1754011	-1785.475472	-1782.789275	EMD2
CAATACGCAAGTGCTT-1	singlet	1	-832.8512981	-287.5558553	-588.9091098	EMD2
CAATACGCAGTCTACA-1	singlet	1	-2210.037307	-689.2158025	-1695.62619	EMD2
CAATACGGTCCTGGTG-1	singlet	1	-2059.544703	-739.5656281	-1756.728217	EMD2
CAATACGGTTCGGTAT-1	singlet	1	-426.66117	-154.5087139	-364.9238347	EMD2
CAATACGTCCGATCTC-1	singlet	1	-505.8961901	-208.1742995	-439.5355335	EMD2
CAATACGTCGAGATGG-1	singlet	1	-1726.731079	-550.1364893	-1344.197936	EMD2
CAATACGTCGGTCACG-1	doublet	0/1	-3642.239875	-5532.732765	-6825.511439	EMD2
CAATACGTCTGGAAGG-1	singlet	0	-967.1864623	-3002.676473	-2883.832055	EMD2
CAATCGAAGCCGAATG-1	singlet	0	-944.7893323	-2474.91492	-2434.726737	EMD2
CAATCGAAGTCAAGCG-1	singlet	0	-468.6149129	-1312.483893	-1271.738422	EMD2
CAATCGAAGTCGCTAT-1	singlet	1	-1183.323341	-438.2033649	-946.129776	EMD2
CAATCGACAAGGTACG-1	singlet	1	-307.2556543	-116.6957385	-259.0800127	EMD2
CAATCGACATAGTCAC-1	singlet	2	-2802.007829	-2378.860165	-873.8706193	EMD2
CAATCGAGTATGAGGC-1	singlet	2	-2144.58889	-1830.636404	-715.6837333	EMD2
CAATCGATCACCGGTG-1	singlet	1	-1004.463057	-319.9468866	-670.170426	EMD2
CAATCGATCATGCCAA-1	singlet	1	-358.9636889	-156.7507337	-264.995295	EMD2
CAATCGATCCAACTAG-1	singlet	2	-388.3445193	-342.0578165	-171.5498253	EMD2
CAATCGATCCTAGAGT-1	doublet	0/1	-2283.962834	-3681.496193	-4278.098874	EMD2
CAATCGATCTAGTGAC-1	singlet	2	-2307.622949	-1769.465578	-621.2099575	EMD2
CAATGACAGACTTCAC-1	singlet	1	-2069.671854	-650.2372292	-1752.917671	EMD2
CAATGACAGGTTAAAC-1	singlet	1	-142.2910041	-47.98265158	-90.38595631	EMD2
CAATGACCAAATGGCG-1	singlet	0	-175.0945774	-528.4415517	-509.2122681	EMD2
CAATGACCATCCGTGG-1	singlet	0	-372.6328378	-1126.235457	-967.5671127	EMD2
CAATGACGTCGACTTA-1	singlet	2	-2842.426174	-2471.9686	-887.0327518	EMD2
CAATGACGTGCGCTCA-1	singlet	2	-1693.570181	-1457.486703	-792.0728326	EMD2
CAATGACTCAGGTAAA-1	singlet	2	-2282.648647	-2043.198532	-794.9238618	EMD2
CAATGACTCATGAAAG-1	singlet	2	-1091.218812	-917.376171	-406.9058615	EMD2
CAATGACTCTATACTC-1	singlet	1	-4856.35092	-1656.669218	-3992.916151	EMD2
CAATTTCAGAGCATTA-1	singlet	0	-529.1934527	-1426.639975	-1378.858918	EMD2
CAATTTCAGCACTCAT-1	singlet	1	-3348.868771	-992.7483547	-2697.86794	EMD2
CAATTTCAGCCTTGAT-1	singlet	0	-649.0088767	-2152.360772	-1911.376743	EMD2
CAATTTCAGGGAGATA-1	singlet	0	-40.41065802	-110.7163191	-118.6554781	EMD2
CAATTTCAGTACAGAT-1	singlet	1	-537.6709354	-197.2767912	-434.4837982	EMD2
CAATTTCAGTGAGGTC-1	singlet	0	-708.787261	-2202.864575	-1959.224932	EMD2
CAATTTCCAGAGTTCT-1	singlet	2	-2014.524613	-1676.731292	-622.5948916	EMD2
CAATTTCCAGGTACGA-1	singlet	0	-303.0547442	-1133.295883	-1106.412937	EMD2
CAATTTCCATCCTAAG-1	singlet	0	-446.6977202	-1233.400912	-1113.148282	EMD2
CAATTTCGTAGCTGTT-1	singlet	2	-57.98910628	-47.02824643	-23.70183091	EMD2
CAATTTCGTTTAGACC-1	singlet	2	-1639.223681	-1648.450886	-512.0674395	EMD2
CAATTTCTCCTACCGT-1	singlet	1	-3822.038572	-1166.995717	-3001.784848	EMD2
CACAACAAGATAGTGT-1	singlet	1	-289.0197969	-104.6918297	-191.9146081	EMD2
CACAACAAGCTGACTT-1	singlet	2	-1084.195693	-1065.328019	-466.7454511	EMD2
CACAACAAGGCACTAG-1	singlet	0	-404.1541291	-2348.748532	-1926.481187	EMD2
CACAACAAGGTGCAGT-1	singlet	1	-84.94308193	-33.72045756	-70.09704422	EMD2
CACAACACACCCGTAG-1	singlet	0	-188.192282	-694.4510161	-538.5324642	EMD2
CACAACACAGGGAATC-1	singlet	2	-3279.067786	-2682.14349	-1165.242167	EMD2
CACAACAGTACGGTTT-1	singlet	2	-1194.230758	-1100.831122	-496.0863705	EMD2
CACAACAGTATGATCC-1	singlet	1	-2896.195007	-835.9683296	-2618.422063	EMD2
CACAACAGTGATACTC-1	singlet	1	-413.5746834	-178.6128913	-367.3229708	EMD2
CACAACAGTTCAGCTA-1	singlet	1	-1205.772971	-389.5905067	-929.7154818	EMD2
CACAACAGTTTCACAG-1	singlet	1	-229.3323844	-115.5601774	-253.6373154	EMD2
CACAACATCCTATGGA-1	singlet	0	-561.5887945	-1781.854498	-1717.69204	EMD2
CACAACATCGATTCCC-1	singlet	0	-747.0366674	-2483.820059	-2358.475659	EMD2
CACAACATCGCTTAAG-1	singlet	2	-4830.406699	-3966.583875	-1295.508996	EMD2
CACACAACACACCTTC-1	singlet	1	-2743.042867	-901.1760817	-2171.29563	EMD2
CACACAACACGCGTGT-1	singlet	1	-1166.513723	-363.4729812	-862.2103042	EMD2
CACACAACATTACGGT-1	singlet	2	-620.8159317	-577.1931204	-262.8775527	EMD2
CACACAAGTGTTCGAT-1	singlet	1	-1520.163786	-518.5930923	-1397.592372	EMD2
CACACAAGTTACGATC-1	singlet	2	-1490.054493	-1392.721188	-520.9477319	EMD2
CACAGATAGAAACACT-1	singlet	0	-705.3600049	-1847.706118	-1929.097175	EMD2
CACAGATAGAAACCAT-1	singlet	2	-12380.37014	-10713.81499	-4048.537457	EMD2
CACAGATAGCACTCTA-1	singlet	1	-3782.768393	-1183.823744	-3015.937053	EMD2
CACAGATAGTAAATGC-1	singlet	0	-1019.602793	-3175.27879	-2920.070119	EMD2
CACAGATAGTCGGCCT-1	singlet	1	-941.9036301	-340.7690863	-812.5703781	EMD2
CACAGATCACCAGCGT-1	singlet	0	-165.0502056	-349.1689366	-344.0842183	EMD2
CACAGATGTAGGTGCA-1	singlet	1	-3270.354823	-963.8438731	-2534.200866	EMD2
CACAGATGTATGGTAA-1	singlet	0	-1126.990024	-4645.638507	-4576.744742	EMD2
CACAGATGTCTTGAGT-1	singlet	1	-959.3944394	-331.317163	-761.9309979	EMD2
CACAGATGTGCTCTTC-1	singlet	2	-230.5998959	-208.4292512	-99.72064318	EMD2
CACAGATTCCCGTTGT-1	singlet	0	-394.3944332	-977.3567372	-959.7892332	EMD2
CACAGATTCCGTGGCA-1	singlet	2	-51.50877464	-46.73876322	-18.22667623	EMD2
CACAGATTCCTGATAG-1	singlet	1	-477.2358645	-184.3243337	-447.7065797	EMD2
CACAGATTCTAAACGC-1	singlet	2	-133.9097429	-126.7349896	-65.1784686	EMD2
CACAGATTCTTGCAGA-1	singlet	0	-349.675589	-589.6686993	-581.9000872	EMD2
CACAGGCAGCGCGTTC-1	singlet	2	-2014.703555	-1822.861356	-576.1376267	EMD2
CACAGGCAGCGTATGG-1	singlet	1	-250.5355726	-107.2693303	-253.7769717	EMD2
CACAGGCAGGTTTGAA-1	singlet	2	-1431.39626	-1148.172972	-421.2702818	EMD2
CACAGGCAGTGATAAC-1	singlet	2	-5626.167403	-4759.205299	-1717.924927	EMD2
CACAGGCCAAAGGAGA-1	singlet	1	-229.5694155	-105.6010196	-182.6591898	EMD2
CACAGGCCAGAGGACT-1	singlet	1	-3079.991204	-759.5953967	-2235.036949	EMD2
CACAGGCGTAACCAGG-1	singlet	1	-386.4922981	-146.6118358	-320.4264128	EMD2
CACAGGCGTCGCTTAA-1	singlet	1	-614.9129346	-198.6167046	-444.7014885	EMD2
CACAGGCGTCTAATCG-1	singlet	0	-38.25575901	-73.72266714	-70.23653722	EMD2
CACAGGCGTGCGCTCA-1	singlet	2	-774.2588813	-583.7019596	-322.752095	EMD2
CACAGGCTCAAGCGTT-1	singlet	1	-1732.905692	-532.584579	-1569.659418	EMD2
CACAGGCTCCACGGGT-1	singlet	1	-3362.800175	-915.0951088	-2697.438271	EMD2
CACAGGCTCCCACAGG-1	singlet	1	-716.1075017	-287.4686355	-584.8128887	EMD2
CACAGGCTCCGGTAGC-1	singlet	1	-2563.446716	-933.5795984	-2070.917197	EMD2
CACAGGCTCGAGAAGC-1	singlet	2	-390.5401813	-431.4015136	-145.5606704	EMD2

CACAGGCTCGAGTGAG-1	singlet	1	-2342.107919	-847.8514801	-1917.105755	EMD2
CACAGGCTCGCGTGAC-1	singlet	0	-235.2498441	-704.222879	-724.4615477	EMD2
CACAGGCTCGTCGCTT-1	singlet	1	-1769.184994	-538.4330377	-1441.421847	EMD2
CACAGGCTCGTTGTTT-1	singlet	0	-1132.367256	-3821.372862	-3714.822179	EMD2
CACAGGCTCTCCCTAG-1	singlet	0	-568.6368196	-1952.647634	-1833.397481	EMD2
CACATGAAGACGACGT-1	singlet	1	-454.2761454	-176.4669019	-368.4894206	EMD2
CACATGAAGAGCAGAA-1	singlet	2	-3779.696941	-3017.13351	-1195.534532	EMD2
CACATGAAGCGCGTTC-1	unassigned	45323	-5.361716697	-2.337459479	-2.091369437	EMD2
CACATGACAAATCCCA-1	singlet	1	-3677.109305	-981.6118123	-2839.106427	EMD2
CACATGACAATTAGGA-1	singlet	1	-449.4092774	-198.4839336	-437.6583399	EMD2
CACATGACATAGACTC-1	singlet	1	-489.3620145	-211.9619147	-432.0215082	EMD2
CACATGAGTAACAGGC-1	singlet	0	-586.4828231	-1890.295745	-1759.83913	EMD2
CACATGAGTACTGCCG-1	singlet	1	-4497.893583	-1321.058895	-3736.852584	EMD2
CACATGAGTTGTGCAT-1	doublet	45293	-97.45542491	-57.71496999	-94.95784698	EMD2
CACATGATCAGACCGC-1	singlet	1	-581.3850667	-262.4412655	-547.5186328	EMD2
CACATGATCGGAGATG-1	singlet	1	-1199.375495	-417.6293085	-830.5568686	EMD2
CACCAAAAGATTGTGA-1	singlet	0	-6.469837551	-15.18530717	-11.00238678	EMD2
CACCAAAAGCTAGATA-1	singlet	0	-694.4065814	-1978.394332	-1828.510637	EMD2
CACCAAACAAGATTGA-1	singlet	1	-2299.404932	-734.2958795	-1829.736796	EMD2
CACCAAACAAGCGAGT-1	singlet	0	-1117.89211	-4044.581115	-3884.556231	EMD2
CACCAAACACTCTGCT-1	singlet	1	-107.1936251	-36.67935217	-83.43282468	EMD2
CACCAAACATAACTCG-1	singlet	0	-488.8024563	-1440.182892	-1290.102296	EMD2
CACCAAACATGAAAGT-1	singlet	0	-211.2445858	-700.5878055	-569.0942794	EMD2
CACCAAAGTATCGGTT-1	singlet	2	-683.7124491	-597.8944568	-258.370591	EMD2
CACCAAAGTCTACACA-1	singlet	2	-3796.935096	-3403.617814	-1398.081284	EMD2
CACCAAATCGTAACTG-1	singlet	1	-1677.819405	-543.3445563	-1328.01268	EMD2
CACCGTTAGGGTGAAA-1	singlet	1	-1145.364769	-319.9459426	-936.0652909	EMD2
CACCGTTAGTCAACAA-1	singlet	0	-696.5706848	-2095.36775	-1865.617544	EMD2
CACCGTTCACACTGGC-1	singlet	2	-1941.735656	-1546.875584	-678.0142883	EMD2
CACCGTTGTACAGAGC-1	singlet	2	-6483.689539	-5296.784074	-1834.870551	EMD2
CACCGTTGTCCCGTGA-1	singlet	1	-382.1508042	-121.9376214	-270.3380161	EMD2
CACCGTTGTCGAACGA-1	doublet	45293	-4472.288537	-1845.075373	-3184.381386	EMD2
CACCGTTGTGATTCTG-1	singlet	1	-352.7482192	-153.2077177	-308.0140132	EMD2
CACCGTTGTGGTCCCA-1	singlet	0	-1236.099409	-4630.555509	-4675.81779	EMD2
CACCGTTTCCGCAGTG-1	singlet	1	-2856.982783	-856.1701396	-2232.359265	EMD2
CACCGTTTCGGCTATA-1	singlet	1	-4034.957335	-1305.478395	-3427.393754	EMD2
CACCGTTTCTGGTCAA-1	singlet	1	-1732.897206	-503.1443405	-1271.762014	EMD2
CACCGTTTCTGTTCAT-1	singlet	0	-802.8876256	-2412.220449	-2392.227045	EMD2
CACGAATAGAGCAACC-1	singlet	1	-2224.295529	-729.1713458	-1818.478721	EMD2
CACGAATAGTAAACTG-1	singlet	0	-1314.217408	-4670.277687	-4320.222664	EMD2
CACGAATAGTACCGGA-1	singlet	0	-342.5758344	-1094.065366	-1065.297228	EMD2
CACGAATCAAGTACCT-1	singlet	1	-74.19374783	-22.39471389	-85.79011908	EMD2
CACGAATCACGAGAAC-1	singlet	1	-1047.239709	-262.0625915	-981.6503928	EMD2
CACGAATCACTGCGTG-1	singlet	2	-5526.071292	-4357.080257	-1425.317266	EMD2
CACGAATCATGCCGAC-1	singlet	1	-1941.743318	-590.0879938	-1631.506588	EMD2
CACGAATCATTACGGT-1	singlet	1	-1061.536615	-404.6438747	-924.1438656	EMD2
CACGAATGTAGTTCCA-1	singlet	1	-313.5670074	-140.1875163	-263.9232257	EMD2
CACGAATGTCATAACC-1	singlet	1	-924.4837842	-333.8495559	-805.6442636	EMD2
CACGAATGTGCTGTCG-1	singlet	1	-724.6349711	-227.876862	-587.7062109	EMD2
CACGAATGTGTACAGG-1	singlet	1	-3959.186024	-1054.009492	-2933.577664	EMD2
CACGAATTCCTAGAGT-1	singlet	1	-97.53990207	-36.4903132	-84.24468548	EMD2
CACGAATTCGCTCCTA-1	singlet	0	-859.2475364	-2678.499325	-2661.961131	EMD2
CACGAATTCTGCGAGC-1	singlet	1	-303.0175999	-132.528949	-283.7661192	EMD2
CACGGGTAGAACCCGA-1	singlet	2	-1877.205985	-1784.239349	-678.0175356	EMD2
CACGGGTAGACCATTC-1	singlet	2	-159.3818617	-106.6650818	-46.08501725	EMD2
CACGGGTAGCCTCTTC-1	singlet	1	-1752.157333	-610.5805472	-1370.656478	EMD2
CACGTGGAGAACCCGA-1	singlet	0	-698.4168405	-1801.626473	-1894.600332	EMD2
CACGTGGAGGTACCTT-1	singlet	1	-233.7892056	-93.40667178	-193.3289407	EMD2
CACGTGGAGTAAACTG-1	singlet	2	-263.5540117	-244.8675712	-133.3033315	EMD2
CACGTGGAGTTTGTCG-1	singlet	1	-232.055605	-51.63370936	-191.553938	EMD2
CACGTGGGTATCCCTC-1	singlet	1	-2493.118967	-806.9332727	-2089.824202	EMD2
CACGTGGGTATTGCCA-1	singlet	1	-2345.047751	-709.9745569	-1721.296854	EMD2
CACGTGGGTCAAAGTA-1	singlet	1	-534.8218791	-206.1601273	-398.2156226	EMD2
CACGTGGGTTCGGTCG-1	singlet	2	-3130.880306	-2821.62065	-971.8466603	EMD2
CACGTGGTCCTTACCG-1	singlet	0	-131.0440961	-346.1278465	-337.6483853	EMD2
CACGTTCAGGTGCCAA-1	singlet	0	-535.5777071	-1492.571219	-1407.386349	EMD2
CACGTTCCAAGCGCAA-1	singlet	2	-114.3546778	-96.44795473	-51.70344452	EMD2
CACGTTCCAGTAACCT-1	singlet	1	-3124.460978	-896.4036151	-2326.525042	EMD2
CACGTTCCATGGGATG-1	singlet	1	-2895.422781	-1053.441979	-2272.846216	EMD2
CACGTTCGTACGACAG-1	singlet	1	-767.0712768	-236.525527	-623.3324325	EMD2
CACGTTCGTCGATGCC-1	singlet	1	-1913.998488	-556.8138024	-1697.873646	EMD2
CACGTTCGTGCATGTT-1	singlet	1	-436.2287056	-189.1028718	-324.4293293	EMD2
CACGTTCTCTCACCCA-1	singlet	1	-1552.259621	-556.157689	-1232.319127	EMD2
CACTAAGAGATGACAT-1	singlet	1	-1218.691078	-358.5815809	-830.483008	EMD2
CACTAAGAGCAAGCCA-1	singlet	1	-21.53980061	-9.676192078	-17.09049192	EMD2
CACTAAGAGGAATTAC-1	singlet	1	-499.0080611	-243.1505639	-438.2837391	EMD2
CACTAAGGTAATTGGA-1	singlet	1	-1804.450789	-685.5786769	-1386.386449	EMD2
CACTAAGGTGGACCAA-1	singlet	0	-31.96678579	-98.97338091	-84.43308126	EMD2
CACTAAGGTGGTCCGT-1	singlet	1	-307.7225849	-114.7735558	-235.6428575	EMD2
CACTAAGGTGTTGCCG-1	singlet	1	-2737.962363	-450.8952752	-1530.743429	EMD2
CACTAAGTCATGCAGT-1	singlet	1	-384.2361985	-181.3192716	-342.7674769	EMD2
CACTAAGTCATTCATC-1	singlet	1	-4488.135222	-1400.323423	-3670.835566	EMD2
CACTAAGTCCGAGATT-1	singlet	1	-231.1595734	-90.80778444	-177.141833	EMD2
CACTAAGTCGTCCTTG-1	singlet	0	-907.5618844	-2634.442879	-2582.028014	EMD2
CACTAAGTCGTCGCTT-1	doublet	45323	-7873.324588	-4685.740394	-3216.970881	EMD2
CACTAAGTCTCGTGAA-1	singlet	0	-586.9334607	-1756.550368	-1840.615076	EMD2
CACTGAAAGGCTATCT-1	singlet	2	-840.1357278	-747.63714	-249.809319	EMD2
CACTGAAGTGGACTGA-1	singlet	1	-912.3420032	-363.9330105	-779.8931899	EMD2
CACTGAATCAAAGACA-1	singlet	2	-1482.890446	-1331.945962	-503.1439778	EMD2
CACTGAATCACTAGCA-1	singlet	1	-1352.585006	-505.0067564	-1164.839963	EMD2
CACTGAATCAGCCTTC-1	singlet	1	-4690.807495	-1478.802689	-3975.443204	EMD2
CACTGAATCATCACCC-1	singlet	2	-4497.908124	-3730.463124	-1304.04356	EMD2
CACTGAATCCCATTCG-1	singlet	1	-393.2514729	-165.6267286	-397.7952211	EMD2
CACTGAATCGTTCCTG-1	singlet	1	-1152.967322	-389.8594101	-1037.710136	EMD2
CACTGGGAGAGGTATT-1	singlet	2	-1712.567101	-1830.675963	-437.4411785	EMD2
CACTGGGAGATGGGCT-1	singlet	1	-4243.158002	-1372.724102	-3378.345194	EMD2
CACTGGGAGATTGTGA-1	singlet	0	-81.55668891	-213.8882887	-206.5213982	EMD2
CACTGGGAGCGCCTCA-1	singlet	1	-514.9173211	-166.7677345	-380.2335377	EMD2
CACTGGGAGGAACTAT-1	singlet	2	-960.997224	-917.7089741	-306.2846692	EMD2
CACTGGGCAATACCTG-1	singlet	1	-562.4087533	-222.3165526	-430.7928773	EMD2
CACTGGGGTGGACAGT-1	singlet	1	-5736.991133	-1758.501949	-4749.128473	EMD2
CACTGGGGTGGCTGCT-1	singlet	0	-65.17281761	-167.9881975	-167.5512245	EMD2
CACTGGGGTTTGTTCT-1	singlet	1	-706.7771743	-206.3442126	-469.6907038	EMD2
CACTGGGTCCGGTTCT-1	singlet	1	-2599.296877	-749.6059332	-1961.118532	EMD2
CACTGGGTCGTTGTTT-1	singlet	1	-412.1099695	-168.9091043	-353.9184405	EMD2
CACTGGGTCTGGCCAG-1	singlet	0	-554.9711502	-1311.275207	-1317.429508	EMD2
CACTGTCAGCCACCGT-1	doublet	1/0	-99.36627228	-80.71005582	-95.44403569	EMD2
CACTGTCAGCGTGTCC-1	singlet	1	-886.4259555	-318.3278332	-647.1327739	EMD2
CACTGTCAGGTCACCC-1	singlet	1	-2584.371675	-730.4790881	-2120.615462	EMD2
CACTGTCAGTAACGTA-1	singlet	1	-3310.151508	-1030.695625	-2641.098495	EMD2

CACTGTCCAATACAGA-1	singlet	1	-387.521327	-156.6708123	-303.093938	EMD2
CACTGTCCACGGCGTT-1	singlet	2	-135.3317015	-96.40972918	-46.72473844	EMD2
CACTGTCCATATGAAG-1	singlet	1	-447.4163469	-214.1988292	-466.2696417	EMD2
CACTGTCGTCGTATTG-1	singlet	0	-544.142919	-1334.616774	-1308.303815	EMD2
CACTGTCGTCTGTGAT-1	singlet	1	-484.0331426	-157.2416648	-376.0681771	EMD2
CACTGTCGTTCAAGTC-1	singlet	1	-1044.470245	-303.0455907	-863.4515961	EMD2
CACTGTCTCACATACG-1	singlet	0	-1089.917069	-3087.003802	-2990.382704	EMD2
CACTGTCTCATCACCC-1	singlet	1	-462.4638653	-188.9943692	-429.4984689	EMD2
CACTGTCTCTCATTTG-1	singlet	2	-4226.956511	-3559.371648	-1589.259665	EMD2
CACTTCGAGACCAGAC-1	singlet	2	-1722.956187	-1508.309801	-388.7179046	EMD2
CACTTCGAGGACGCTA-1	singlet	0	-546.267325	-1961.684681	-1751.261683	EMD2
CACTTCGAGGCGAAGG-1	singlet	2	-4479.723915	-3812.43979	-1432.303504	EMD2
CACTTCGAGGTAGTAT-1	singlet	1	-1535.246858	-597.2828257	-1347.651831	EMD2
CACTTCGCAAATAAGC-1	singlet	1	-352.4016725	-156.8894075	-345.6824469	EMD2
CACTTCGCAACAAGTA-1	singlet	1	-92.22082914	-30.00790999	-84.65775025	EMD2
CACTTCGCAATATCCG-1	singlet	1	-1186.869733	-402.2514088	-1028.131909	EMD2
CACTTCGCAATCCTTT-1	singlet	0	-1930.435017	-5376.895702	-4947.992567	EMD2
CACTTCGCACGTCATA-1	singlet	1	-357.5360148	-147.1483081	-288.8711322	EMD2
CACTTCGGTCAGTCGC-1	singlet	2	-54.78248754	-79.38223806	-24.57056837	EMD2
CACTTCGGTCTTCAAG-1	singlet	1	-74.68737184	-31.63382359	-53.00548366	EMD2
CACTTCGGTTCCGCAG-1	singlet	1	-1234.44509	-456.2834852	-1073.802653	EMD2
CACTTCGTCTTGTTAC-1	singlet	0	-222.9604354	-711.6163278	-638.9634922	EMD2
CAGAGCCAGACTTCGT-1	singlet	2	-4396.381089	-3391.768349	-1095.341662	EMD2
CAGAGCCAGAGCATCG-1	singlet	1	-456.0051143	-197.2575499	-411.2112346	EMD2
CAGAGCCCACTAAACC-1	singlet	1	-4533.644504	-1418.813158	-3820.369284	EMD2
CAGAGCCCATATCTCT-1	singlet	1	-143.4109335	-52.84952213	-97.90755665	EMD2
CAGAGCCGTGAGCGAT-1	singlet	2	-344.3254868	-336.6727914	-120.5503243	EMD2
CAGAGCCGTGGATACG-1	doublet	1/0	-1502.181905	-986.024909	-1591.289183	EMD2
CAGAGCCGTTCTAACG-1	singlet	2	-1677.966335	-1650.354597	-526.3662236	EMD2
CAGAGCCTCATACGGT-1	singlet	0	-163.3601517	-482.6067853	-404.187194	EMD2
CAGAGCCTCTCTATAC-1	singlet	1	-159.7623494	-64.22648145	-135.7261628	EMD2
CAGATACAGTCAGGGT-1	singlet	1	-1820.222204	-597.588922	-1444.978173	EMD2
CAGATACCAAATCGTC-1	singlet	2	-541.5821666	-473.3854464	-242.0577256	EMD2
CAGATACGTAGCGAGT-1	singlet	0	-1221.178632	-5052.611504	-4809.865348	EMD2
CAGATACGTCGGCCTA-1	singlet	0	-164.8122868	-534.4073962	-422.0929643	EMD2
CAGATACGTTGCGGAA-1	singlet	0	-2051.013327	-6912.523555	-6462.689131	EMD2
CAGATACTCACCTCTG-1	singlet	1	-359.972557	-125.5522938	-301.2173183	EMD2
CAGATACTCATTATCC-1	singlet	2	-1370.217759	-1169.989972	-642.0302607	EMD2
CAGATACTCTCTATGT-1	unassigned	0/1	-14.02247492	-20.38276009	-22.88138759	EMD2
CAGATCAAGAAACTCA-1	singlet	1	-1211.721783	-330.8750425	-1026.914362	EMD2
CAGATCAAGAGGTGCT-1	singlet	1	-1718.848529	-517.2544599	-1427.475482	EMD2
CAGATCAAGATTGCGG-1	singlet	0	-110.4188288	-393.5569111	-281.5794992	EMD2
CAGATCACATACTGAC-1	singlet	2	-2870.882879	-2358.944245	-1007.639085	EMD2
CAGATCACATATCGGT-1	singlet	2	-14.51170431	-13.42806273	-7.849788682	EMD2
CAGATCACATCACGGC-1	singlet	1	-258.3107091	-90.06447166	-231.1048609	EMD2
CAGATCAGTAGTATAG-1	singlet	2	-426.6618082	-388.6274107	-159.4882717	EMD2
CAGATCAGTCATGACT-1	singlet	2	-2276.649822	-2040.418696	-672.7656165	EMD2
CAGATCAGTGAACTAA-1	singlet	1	-862.3940069	-307.1836392	-659.6024235	EMD2
CAGATCAGTGAGGATC-1	singlet	1	-308.3544087	-131.5515453	-286.8573979	EMD2
CAGATCAGTGGGTCAA-1	singlet	1	-297.3092954	-127.0473571	-238.87619	EMD2
CAGATCATCATGCAGT-1	singlet	1	-485.2415924	-177.4747121	-441.1653048	EMD2
CAGATCATCCTACTGC-1	singlet	0	-3282.072257	-10193.06752	-10024.74471	EMD2
CAGATCATCGATGCTA-1	singlet	1	-1008.869312	-348.550371	-875.0020702	EMD2
CAGATTGAGTCGCTAT-1	singlet	1	-497.235661	-158.4468811	-363.3977187	EMD2
CAGATTGCAAAGGCTG-1	singlet	1	-680.3379261	-206.2215544	-570.2903913	EMD2
CAGATTGGTAGGAGGG-1	singlet	1	-2549.583474	-843.2568459	-2060.846222	EMD2
CAGATTGGTCGCGTCA-1	singlet	1	-111.5807724	-48.73935596	-118.2960946	EMD2
CAGCAATAGAAGCCAC-1	singlet	0	-220.3491531	-692.7611691	-572.4324199	EMD2
CAGCAATAGACTCGAG-1	singlet	2	-1412.915301	-1298.061568	-509.8182055	EMD2
CAGCAATCAACCCTAA-1	singlet	1	-620.6373774	-212.2999823	-471.5785304	EMD2
CAGCAATGTAACACGG-1	singlet	1	-2324.686785	-789.3764941	-2067.180677	EMD2
CAGCAATGTACGCGTC-1	singlet	1	-404.371509	-145.6308876	-376.7861532	EMD2
CAGCAATGTATGAGAT-1	singlet	2	-1175.680365	-1009.511876	-380.7164065	EMD2
CAGCAATTCATCTGTT-1	singlet	0	-36.03702916	-68.69215168	-76.89645766	EMD2
CAGCAATTCGGTCGGT-1	singlet	1	-449.5127333	-183.6555389	-362.6416613	EMD2
CAGCACGAGCGTCGAA-1	doublet	45323	-201.1001518	-108.4063398	-106.8074452	EMD2
CAGCACGAGGGCTGAT-1	singlet	1	-471.7715317	-224.0111384	-485.3421779	EMD2
CAGCACGAGTAGCCAG-1	singlet	1	-274.7450765	-101.175471	-212.0638362	EMD2
CAGCACGCAAGAGTGC-1	singlet	1	-259.1942606	-94.11582476	-204.9625234	EMD2
CAGCACGCACTCGATA-1	singlet	1	-3950.500708	-1198.318637	-2930.898609	EMD2
CAGCACGGTAGTATAG-1	singlet	0	-88.72258571	-464.0884549	-245.3485972	EMD2
CAGCACGGTCGTTCAA-1	singlet	0	-1469.471825	-5701.532217	-5416.120629	EMD2
CAGCACGGTGCGTGCT-1	singlet	1	-144.5732768	-53.69807337	-99.29727555	EMD2
CAGCACGGTTGGCTAT-1	singlet	1	-735.5921693	-350.1954478	-573.6770937	EMD2
CAGCACGTCATCTCTA-1	singlet	1	-1636.480831	-513.8650936	-1449.041058	EMD2
CAGCACGTCCACTAGA-1	singlet	1	-3975.464488	-1036.851453	-3126.998958	EMD2
CAGCACGTCCAGCTCT-1	singlet	1	-610.80881	-209.5250666	-539.6996214	EMD2
CAGCACGTCCGCAAAT-1	singlet	1	-82.96494652	-35.95397278	-58.49385134	EMD2
CAGCACGTCGGAGCAA-1	singlet	0	-41.36568482	-111.935285	-87.89357188	EMD2
CAGCAGCAGAGTGAAG-1	singlet	0	-477.6736862	-1607.013231	-1536.223623	EMD2
CAGCAGCAGTGCTAGG-1	singlet	1	-1854.508062	-603.0156711	-1403.699021	EMD2
CAGCAGCGTCGCTCGA-1	singlet	0	-145.1037255	-555.4704938	-497.6752866	EMD2
CAGCAGCGTGATCATC-1	singlet	0	-116.6817273	-342.2864823	-285.0933133	EMD2
CAGCAGCGTGTGGACA-1	singlet	0	-682.197165	-2240.525558	-2154.618542	EMD2
CAGCAGCTCATGCTAG-1	singlet	1	-1454.191441	-458.3412347	-1344.717113	EMD2
CAGCAGCTCGTCGCTT-1	singlet	2	-644.5723465	-646.043607	-226.1611503	EMD2
CAGCCAGAGAATTCAG-1	singlet	1	-311.2011698	-115.5202722	-252.3607276	EMD2
CAGCCAGAGAGATCGC-1	singlet	0	-912.4309536	-2946.913884	-2940.958773	EMD2
CAGCCAGAGCGTTACT-1	singlet	1	-289.9562757	-128.1458892	-262.6677088	EMD2
CAGCCAGAGTGAGCCA-1	singlet	1	-2157.983486	-690.4686342	-2199.966193	EMD2
CAGCCAGCAAAGGCGT-1	singlet	0	-1053.903051	-3115.797605	-3053.981187	EMD2
CAGCCAGCAAGGGTCA-1	singlet	0	-1028.380143	-3008.988014	-2836.328342	EMD2
CAGCCAGCACAACGAG-1	singlet	2	-1664.426608	-1642.062704	-710.7469917	EMD2
CAGCCAGCACCTCAGG-1	singlet	1	-2574.425321	-890.5011952	-2148.745846	EMD2
CAGCCAGCAGCACAAG-1	singlet	2	-5240.239729	-4501.914962	-1614.801673	EMD2
CAGCCAGGTGCCCACA-1	singlet	1	-363.6132655	-142.0151473	-321.6270973	EMD2
CAGCCAGGTGTTGATC-1	singlet	2	-262.7243731	-229.0135252	-124.2159979	EMD2
CAGCCAGGTTCTTGCC-1	singlet	1	-242.124253	-66.17540891	-209.7499171	EMD2
CAGCCAGTCGACACTA-1	singlet	1	-1057.334677	-400.5787237	-852.1910064	EMD2
CAGCGTGAGAGCAGAA-1	singlet	0	-1892.837499	-8122.489124	-7900.124917	EMD2
CAGCGTGAGCTCGCAC-1	singlet	1	-1643.679261	-597.5742947	-1566.078125	EMD2
CAGCGTGAGGATTCAA-1	doublet	0/1	-1879.362466	-1892.908262	-2720.141121	EMD2
CAGCGTGCACATTACG-1	singlet	0	-671.1175374	-2652.641211	-2268.527576	EMD2
CAGCGTGCACTTTATC-1	singlet	1	-733.9227786	-323.3254936	-627.9124437	EMD2
CAGCGTGCATAATCCG-1	singlet	1	-1231.568114	-401.1118018	-992.8302228	EMD2
CAGCGTGGTCAAGCGA-1	singlet	1	-588.4201581	-197.7168575	-469.7295307	EMD2
CAGCGTGGTCCCTAAA-1	singlet	2	-1198.48597	-1263.174405	-319.4661962	EMD2
CAGCGTGGTTCGAAGG-1	singlet	2	-3708.279993	-2755.520418	-1286.947808	EMD2
CAGCGTGTCAACTCTT-1	singlet	2	-1442.814865	-1120.261705	-525.9042349	EMD2
CAGGCCAAGACCGCCT-1	singlet	1	-1917.132109	-590.4506283	-1669.057812	EMD2

CAGGCCAAGCGTTCAT-1	singlet	1	-363.5574218	-125.3410589	-299.538483	EMD2
CAGGCCAAGGGTTTCT-1	singlet	0	-177.7778848	-471.4850921	-512.6123382	EMD2
CAGGCCAAGTTTCGAC-1	singlet	1	-425.6176747	-177.9132716	-373.2249256	EMD2
CAGGCCACAACATCGT-1	singlet	1	-626.8601497	-256.5373296	-541.9277642	EMD2
CAGGCCACAAGCCTGC-1	singlet	1	-2863.690836	-697.847126	-1822.941732	EMD2
CAGGCCACACTCAGAT-1	singlet	0	-426.7537861	-1248.304713	-1142.091593	EMD2
CAGGCCAGTCGACTTA-1	singlet	2	-98.12202596	-72.60337052	-34.70008565	EMD2
CAGGCCAGTCTTTCAT-1	singlet	2	-2592.310159	-2194.932318	-921.7408303	EMD2
CAGGCCAGTTGTCAGT-1	unassigned	45323	-19.43882593	-12.13013688	-9.751657238	EMD2
CAGGGCTAGACAGCTG-1	singlet	1	-2893.319299	-842.7189529	-2273.425073	EMD2
CAGGGCTCACTTGGCG-1	singlet	0	-13.07109513	-26.22393178	-25.65887123	EMD2
CAGGGCTCAGAGTTGG-1	singlet	1	-564.3358839	-218.0357867	-482.3542374	EMD2
CAGGGCTCAGCGGTTC-1	singlet	0	-101.0734718	-234.9635742	-230.4775627	EMD2
CAGGGCTCATGGCTGC-1	singlet	0	-1634.056843	-5383.672489	-5078.041369	EMD2
CAGGGCTGTAACATGA-1	singlet	1	-3292.114104	-870.9606779	-2612.03546	EMD2
CAGGGCTGTCAGGTAG-1	singlet	1	-480.471674	-193.4161253	-419.2193173	EMD2
CAGGGCTGTGTTTACG-1	singlet	0	-330.9932543	-1058.765687	-953.0617381	EMD2
CAGGGCTGTTATGTCG-1	singlet	2	-3999.57207	-3217.834678	-1309.372208	EMD2
CAGGGCTGTTCCGCGA-1	singlet	1	-1165.405494	-393.7613589	-869.5198962	EMD2
CAGGGCTTCCGTGACG-1	singlet	0	-731.0247805	-2537.245955	-2230.939371	EMD2
CAGGGCTTCTTTGATC-1	singlet	0	-897.7351148	-2632.632776	-2574.147271	EMD2
CAGGTATAGCCATTTG-1	singlet	2	-5501.326718	-4497.441127	-1719.549342	EMD2
CAGGTATAGGAACGAA-1	singlet	1	-569.8436125	-222.5057065	-440.8758443	EMD2
CAGGTATCACGGATCC-1	singlet	1	-5962.676964	-2006.167987	-4819.178919	EMD2
CAGGTATCAGCAGTGA-1	singlet	1	-2826.000685	-824.3885058	-2183.119946	EMD2
CAGGTATCAGCTATTG-1	singlet	2	-1226.66757	-1202.048301	-535.1032995	EMD2
CAGGTATCATACCAGT-1	singlet	1	-3212.502631	-864.3611399	-2945.60081	EMD2
CAGGTATCATTCACCC-1	singlet	0	-2073.152025	-7706.226461	-7137.461877	EMD2
CAGGTATTCATAGCAC-1	singlet	0	-9.933427406	-29.79643388	-17.88396572	EMD2
CAGTGCGAGCACCGAA-1	singlet	2	-1908.397281	-1762.660028	-702.9086084	EMD2
CAGTGCGAGCGTTAGG-1	singlet	0	-863.2786072	-2749.138871	-2702.886055	EMD2
CAGTGCGCAAGTCCAT-1	singlet	1	-2912.43959	-917.4333469	-2589.827602	EMD2
CAGTGCGCAAGTCGTT-1	singlet	2	-953.0532142	-810.0265722	-311.2966511	EMD2
CAGTGCGGTACTCGCG-1	singlet	1	-2820.714322	-833.0660168	-2318.197435	EMD2
CAGTGCGGTGCTCTTC-1	singlet	0	-1101.220139	-3642.8605	-3727.445119	EMD2
CAGTTAGAGATAACGT-1	singlet	2	-888.0897778	-779.2758627	-329.1814827	EMD2
CAGTTAGAGCGCCTTG-1	singlet	2	-2213.23946	-1992.51101	-761.7733298	EMD2
CAGTTAGAGTAATACG-1	singlet	1	-1056.465158	-351.1028584	-865.2423254	EMD2
CAGTTAGAGTGCTCGC-1	singlet	1	-338.0270008	-94.99753283	-311.3021531	EMD2
CAGTTAGCAAGCGCAA-1	singlet	2	-644.2873727	-436.1113404	-228.4091228	EMD2
CAGTTAGGTCAGACGA-1	singlet	0	-193.4251472	-542.1393124	-443.619778	EMD2
CAGTTAGGTCCGAAGA-1	doublet	2/0	-505.9692918	-585.1764562	-319.0997626	EMD2
CAGTTAGGTCTCGCGA-1	singlet	0	-93.51624603	-288.1988338	-246.0448684	EMD2
CAGTTAGGTTGTTGTG-1	singlet	1	-139.835041	-63.94480742	-112.475736	EMD2
CAGTTAGTCTATGCCC-1	singlet	1	-2761.783875	-904.9399896	-2174.684433	EMD2
CAGTTCCAGACGACTG-1	singlet	0	-151.655122	-535.6962971	-604.0075406	EMD2
CAGTTCCCAGTGCGCT-1	singlet	0	-109.8779069	-294.9256147	-264.8370794	EMD2
CAGTTCCGTACACGTT-1	singlet	1	-353.4826638	-140.971749	-313.0060413	EMD2
CAGTTCCGTAGGAGGG-1	singlet	1	-472.9022645	-170.1653609	-366.7627527	EMD2
CAGTTCCTCATTTGGG-1	singlet	1	-348.5134282	-122.4983546	-283.8707886	EMD2
CAGTTCCTCCCAGCGA-1	singlet	1	-1781.692103	-582.4052491	-1486.516689	EMD2
CAGTTCCTCCGTAATG-1	singlet	2	-1379.152294	-1441.400365	-568.8149499	EMD2
CAGTTCCTCGTAGCTA-1	singlet	1	-366.3317987	-153.8832845	-294.3919897	EMD2
CAGTTCCTCGTCGGGT-1	singlet	0	-457.3448909	-1875.668371	-1693.793693	EMD2
CAGTTCCTCGTGCAGC-1	singlet	0	-731.4468382	-2647.18517	-2473.590185	EMD2
CAGTTCCTCTGCGATA-1	singlet	1	-433.1229907	-172.145687	-419.4783826	EMD2
CATAAGCAGCGATGAC-1	singlet	2	-787.6203741	-771.6338652	-351.3218418	EMD2
CATAAGCAGCTAGTTC-1	doublet	45323	-4675.129794	-3201.914455	-2282.621864	EMD2
CATAAGCAGGGTGGGA-1	singlet	1	-218.8819023	-81.67755887	-180.2297834	EMD2
CATAAGCAGTGGACTG-1	singlet	2	-2861.131304	-2158.423893	-933.1089674	EMD2
CATAAGCAGTGTAGTA-1	singlet	0	-502.5736864	-1456.430246	-1439.087974	EMD2
CATAAGCCAACAGTGG-1	singlet	1	-748.9687841	-251.1266527	-560.8006743	EMD2
CATAAGCCAAGTTCGT-1	singlet	1	-351.9113284	-137.1497955	-235.1301683	EMD2
CATAAGCCAGCCTATA-1	singlet	1	-1979.779455	-579.9744785	-1696.508588	EMD2
CATAAGCCATCTGGGC-1	doublet	45323	-2583.683875	-1899.176754	-1366.29303	EMD2
CATAAGCGTCCCGGTA-1	singlet	2	-2908.267533	-2304.139495	-789.5789699	EMD2
CATAAGCGTCCCGTGA-1	singlet	1	-198.7078998	-83.04612352	-136.8513698	EMD2
CATAAGCGTCCGTACG-1	singlet	1	-426.2253714	-153.2645079	-343.8104807	EMD2
CATAAGCGTTATAGAG-1	singlet	0	-218.5869966	-604.6243841	-587.5949391	EMD2
CATAAGCGTTCTCGTC-1	singlet	0	-684.7917971	-2101.500162	-1980.662308	EMD2
CATAAGCTCGGACAAG-1	singlet	1	-1728.821597	-603.1047706	-1577.005774	EMD2
CATAAGCTCTTGGTGA-1	singlet	1	-1902.657474	-608.716181	-1510.804998	EMD2
CATACAGAGGATATAC-1	singlet	1	-274.7240682	-108.333827	-237.306643	EMD2
CATACAGCACCCTTGT-1	singlet	2	-2295.376533	-2376.818795	-710.7304384	EMD2
CATACAGGTAAGAACT-1	singlet	1	-450.8260083	-201.9077099	-366.88503	EMD2
CATACAGGTGGCTCTG-1	singlet	1	-3067.808793	-900.5255996	-2297.445492	EMD2
CATACAGGTGTTCATG-1	singlet	2	-1413.780068	-1445.969052	-468.3175312	EMD2
CATACAGTCAGTGGGA-1	singlet	1	-1175.753397	-353.8637285	-1008.815198	EMD2
CATACCCAGGCAATGC-1	singlet	1	-1502.344734	-484.844513	-1310.03136	EMD2
CATACCCAGTTTGTCG-1	singlet	1	-321.0417314	-109.2287495	-351.5736779	EMD2
CATACCCCACTCCTTG-1	singlet	1	-908.8096656	-309.7304837	-632.9206448	EMD2
CATACCCGTCGTTGGC-1	singlet	1	-1608.946745	-533.5393954	-1230.930689	EMD2
CATACCCTCATGAGAA-1	singlet	0	-656.6222077	-1892.37921	-1662.83524	EMD2
CATACCCTCTCAATCT-1	singlet	1	-3710.83535	-1112.560269	-2714.134941	EMD2
CATACCCTCTGAGGCC-1	singlet	1	-454.516632	-146.2188284	-334.4741348	EMD2
CATACTTAGGACTGGT-1	singlet	1	-584.6340821	-250.0350437	-499.8395418	EMD2
CATACTTAGGCGTCCT-1	singlet	2	-1526.717852	-1336.447318	-570.1894639	EMD2
CATACTTAGGTTGCCC-1	singlet	1	-470.7378264	-154.9490333	-343.1390666	EMD2
CATACTTAGTAAGACT-1	singlet	1	-226.6770984	-108.0579188	-234.709922	EMD2
CATACTTGTCAATGGG-1	singlet	2	-1156.052219	-1025.771118	-440.7703386	EMD2
CATACTTGTCGATTTG-1	singlet	1	-2561.172761	-804.7217433	-2023.971956	EMD2
CATACTTTCGTCGGGT-1	singlet	2	-4232.913446	-3606.036378	-1177.582392	EMD2
CATACTTTCGTGCGAC-1	doublet	1/0	-1788.560055	-1111.409493	-1817.245886	EMD2
CATACTTTCTAGCCAA-1	singlet	1	-298.8911406	-119.515851	-269.1147336	EMD2
CATAGACAGGACAGCT-1	singlet	1	-1844.976941	-613.2609778	-1694.18675	EMD2
CATAGACAGTGCAGGT-1	singlet	1	-383.2744206	-164.4333371	-386.6675298	EMD2
CATAGACCAACCGACC-1	singlet	1	-1380.654954	-374.8772367	-872.5696333	EMD2
CATAGACCAAGGACAC-1	singlet	2	-128.1643799	-120.4181932	-55.28726825	EMD2
CATAGACCAGAAGCTG-1	singlet	0	-1094.340125	-3186.75395	-3042.248224	EMD2
CATAGACCAGCAGTCC-1	singlet	1	-993.9613002	-382.1224728	-786.8115772	EMD2
CATAGACCAGTCAGAG-1	singlet	1	-332.5240641	-130.7437574	-273.2260279	EMD2
CATAGACCAGTTCACA-1	singlet	0	-2441.814611	-9520.79674	-9740.605296	EMD2
CATAGACGTCGAAACG-1	singlet	1	-10.84402869	-2.744919041	-11.59528076	EMD2
CATAGACTCATACGGT-1	singlet	1	-1767.112918	-624.420431	-1527.420261	EMD2
CATAGACTCATCAGTG-1	singlet	1	-121.7769075	-59.89164775	-126.4425127	EMD2
CATAGACTCGTTGTGA-1	singlet	1	-203.8366385	-55.45724707	-138.2363757	EMD2
CATCAAGAGGAGAGTA-1	singlet	0	-482.7637635	-2071.170126	-1740.644891	EMD2
CATCAAGAGGCAGGGA-1	singlet	0	-112.4053152	-213.6539688	-204.8240961	EMD2
CATCAAGAGGCATGCA-1	singlet	1	-688.1760609	-237.5543759	-494.8739934	EMD2
CATCAAGAGGGACAGG-1	singlet	1	-354.9428092	-115.5273545	-304.1444812	EMD2

CATCAAGAGGGTCTTT-1	singlet	0	-138.6932816	-324.691227	-335.8338931	EMD2
CATCAAGAGTACTGGG-1	singlet	1	-4494.437842	-1346.437656	-3447.843953	EMD2
CATCAAGAGTAGGTTA-1	doublet	1/0	-2441.241395	-2086.198044	-3079.270357	EMD2
CATCAAGCAACAACAA-1	singlet	1	-419.6993705	-159.0872961	-387.5527061	EMD2
CATCAAGCAACGTTAC-1	singlet	2	-442.3613523	-356.5790979	-175.9040243	EMD2
CATCAAGCACGACGCT-1	singlet	2	-1407.801041	-1058.048903	-463.1404528	EMD2
CATCAAGCACTGAGGA-1	singlet	1	-596.717839	-198.4922352	-460.73438	EMD2
CATCAAGCATCCTAAG-1	singlet	0	-601.0687218	-1793.963756	-1685.032392	EMD2
CATCAAGGTACTGGGA-1	singlet	1	-1595.370027	-517.1914201	-1333.456334	EMD2
CATCAAGGTGACGCCT-1	singlet	1	-283.6577478	-128.1968817	-248.1824398	EMD2
CATCAAGTCAACTCTT-1	singlet	2	-2355.685261	-2126.962211	-715.2523262	EMD2
CATCAAGTCAGATTGC-1	singlet	2	-522.6057919	-621.566748	-190.7033449	EMD2
CATCAAGTCATTACCT-1	singlet	1	-1425.404314	-585.0476489	-1324.380518	EMD2
CATCAAGTCGAGCCTG-1	singlet	1	-1841.999195	-684.5225708	-1616.823263	EMD2
CATCAAGTCGATTGGT-1	singlet	0	-443.7276699	-1268.39026	-1266.632457	EMD2
CATCAAGTCGTTATCT-1	doublet	2/0	-139.25177	-197.5383761	-113.7381392	EMD2
CATCCACAGAAATCCA-1	singlet	0	-33.1822003	-70.7881372	-68.22686537	EMD2
CATCCACAGACCAGAC-1	singlet	1	-3166.523409	-1074.196695	-2405.607033	EMD2
CATCCACAGCAGAAAG-1	singlet	2	-1020.366528	-1046.413009	-373.0187659	EMD2
CATCCACCAACACAAA-1	singlet	1	-4301.928822	-1226.69735	-3558.567261	EMD2
CATCCACCAACGGTAG-1	singlet	2	-391.1110608	-350.6067249	-158.1555874	EMD2
CATCCACCACGTATAC-1	singlet	1	-175.0551079	-73.46681469	-142.8606905	EMD2
CATCCACCAGTCAGTT-1	unassigned	0	-7.993770599	-10.09360684	-8.427830404	EMD2
CATCCACCAGTCGCTG-1	singlet	2	-158.9162472	-136.6936239	-61.84998719	EMD2
CATCCACCATACCACA-1	singlet	2	-2265.55052	-2402.781847	-680.8197614	EMD2
CATCCACGTAACGGTG-1	singlet	0	-111.7578862	-504.3035743	-359.0362139	EMD2
CATCCACGTACACGTT-1	singlet	1	-438.0274061	-157.8800433	-362.5547743	EMD2
CATCCACGTGTTCCTC-1	singlet	1	-4734.318603	-1634.527825	-3964.881242	EMD2
CATCCACTCCCAAGCG-1	singlet	1	-2929.384933	-891.2117107	-2417.986395	EMD2
CATCCACTCGGAAGGT-1	singlet	1	-2288.879443	-567.9387862	-1672.4294	EMD2
CATCCACTCTGAGGCC-1	singlet	2	-932.5040255	-784.4524491	-296.7536417	EMD2
CATCCCAAGTTACTCG-1	singlet	0	-634.4771823	-2203.983613	-2088.18277	EMD2
CATCCCACAAATCAGA-1	singlet	1	-630.7216003	-295.2515991	-526.3354358	EMD2
CATCCCACAACTTGCA-1	singlet	2	-2752.236357	-2338.42776	-969.9181615	EMD2
CATCCCACAAGTTCGT-1	singlet	0	-19.48302293	-39.35567446	-47.39328744	EMD2
CATCCCAGTAACATAG-1	singlet	1	-768.4458203	-320.1926046	-679.7095748	EMD2
CATCCCAGTAGGCAAC-1	singlet	1	-321.9884441	-136.5999452	-295.8264343	EMD2
CATCCCAGTATCGTGT-1	singlet	1	-4755.960411	-1545.383312	-3753.633987	EMD2
CATCCCAGTTAGGCTT-1	singlet	0	-183.6976984	-602.0418696	-467.307174	EMD2
CATCCGTAGACCAGCA-1	singlet	1	-1740.317148	-584.3518211	-1459.602663	EMD2
CATCCGTAGAGAAGGT-1	singlet	0	-225.9687593	-483.2765171	-451.3973058	EMD2
CATCCGTAGCGCTGCT-1	singlet	0	-172.0093023	-483.9711355	-518.0778753	EMD2
CATCCGTAGTAGTCAA-1	singlet	1	-2381.413847	-756.5252927	-1826.400022	EMD2
CATCCGTAGTATAACG-1	singlet	1	-184.2436424	-52.79372951	-122.3629976	EMD2
CATCCGTCAAAGCACG-1	singlet	0	-340.4096275	-1156.664691	-1065.143952	EMD2
CATCCGTCAGGACAGT-1	singlet	2	-934.4468254	-867.8773114	-364.0092734	EMD2
CATCCGTGTACCAGAG-1	singlet	0	-410.1526894	-1418.252375	-1221.27478	EMD2
CATCCGTTCAACACGT-1	singlet	1	-2364.027457	-740.6657967	-1962.199865	EMD2
CATCCGTTCACCCATC-1	singlet	2	-239.2546781	-233.8636489	-95.20315679	EMD2
CATCCGTTCTCGCTCA-1	singlet	1	-40.8663344	-12.16488474	-53.7275234	EMD2
CATCCGTTCTCTAAGG-1	singlet	1	-1120.47848	-390.3137945	-898.4703591	EMD2
CATCCGTTCTGCGGGT-1	singlet	1	-174.0928522	-64.39421732	-173.9946073	EMD2
CATCGCTAGGACACTG-1	singlet	0	-713.0061499	-2331.568696	-2164.118375	EMD2
CATCGCTGTCGCATTA-1	singlet	1	-3732.603079	-1213.189781	-3073.758292	EMD2
CATCGCTGTCTTCAAG-1	singlet	1	-1456.815147	-374.5467014	-1096.404045	EMD2
CATCGCTGTTGCGAAG-1	singlet	1	-938.1956627	-301.4857964	-728.4930066	EMD2
CATCGCTTCACATACG-1	singlet	0	-54.19087765	-123.8975939	-132.6517238	EMD2
CATCGCTTCATGCTAG-1	singlet	1	-901.773219	-318.5411096	-751.9287619	EMD2
CATCGCTTCGTAATGC-1	singlet	1	-1642.086007	-469.904577	-1490.322315	EMD2
CATCGCTTCGTACCTC-1	singlet	1	-299.9181277	-145.5974814	-267.0847083	EMD2
CATCGGGAGAACGTGC-1	singlet	2	-2344.375829	-1988.854902	-849.5737797	EMD2
CATCGGGAGAGAGAAC-1	singlet	1	-2495.526765	-671.8971722	-1921.592947	EMD2
CATCGGGAGTCGTTAC-1	singlet	1	-51.26672769	-24.6978774	-46.65399489	EMD2
CATCGGGAGTCTTCGA-1	singlet	2	-38.89585721	-41.25140165	-13.6202754	EMD2
CATCGGGAGTTCCGTA-1	singlet	0	-819.0529057	-2373.680846	-2394.817731	EMD2
CATCGGGCACCATAAC-1	singlet	1	-1270.795025	-466.2499212	-1161.306612	EMD2
CATCGGGCACGTCATA-1	singlet	2	-422.6872416	-375.7815733	-164.0390647	EMD2
CATCGGGCACTGGAAG-1	singlet	2	-1290.039107	-1214.692179	-484.3015971	EMD2
CATCGGGCATGCCGAC-1	singlet	0	-48.2636725	-208.4768875	-195.2354777	EMD2
CATCGGGGTCGAACAG-1	doublet	0/1	-553.8598405	-916.0464609	-1010.719022	EMD2
CATCGGGGTCTCAAGT-1	singlet	1	-663.0978679	-225.6276329	-569.2777537	EMD2
CATCGGGGTTAGTCGT-1	singlet	1	-217.0588376	-95.13289984	-200.607131	EMD2
CATCGGGTCACCCTTG-1	doublet	2/0	-2366.197461	-2940.508319	-1754.749462	EMD2
CATCGGGTCGTTCATT-1	singlet	1	-1805.851954	-458.0486791	-1227.23875	EMD2
CATCGTCCAAGACCGA-1	singlet	1	-3393.302052	-1119.833831	-2866.770002	EMD2
CATCGTCCACGCAAAG-1	singlet	1	-3009.977334	-968.0295932	-2667.94345	EMD2
CATCGTCCACTAGTAC-1	singlet	0	-1388.068286	-4684.816266	-4516.258742	EMD2
CATCGTCGTACGAGCA-1	singlet	0	-31.75201234	-96.41952245	-87.39893271	EMD2
CATCGTCGTAGCACGA-1	singlet	1	-2999.071325	-920.2579401	-2529.607141	EMD2
CATCGTCGTATGTGTC-1	singlet	1	-232.2801855	-115.4428698	-218.8867972	EMD2
CATCGTCGTGGATCGA-1	singlet	1	-855.5936465	-273.6058538	-661.6547957	EMD2
CATCGTCTCATGGATC-1	singlet	1	-182.9894338	-80.58226219	-159.0105438	EMD2
CATCGTCTCATTTGTC-1	singlet	1	-591.8420817	-213.6053283	-472.701057	EMD2
CATCGTCTCGTAGTCA-1	singlet	1	-1520.792629	-518.4947893	-1242.152022	EMD2
CATCGTCTCTTCGCTG-1	singlet	2	-307.8277433	-251.355763	-116.8970229	EMD2
CATGAGTAGATGCCGA-1	singlet	1	-3293.764116	-1045.571697	-2575.172465	EMD2
CATGAGTAGCTCCACG-1	singlet	1	-5078.699016	-1635.413462	-4204.89448	EMD2
CATGAGTAGCTGACTT-1	singlet	1	-1190.687111	-525.1923142	-1067.13933	EMD2
CATGAGTAGTGCAGCA-1	singlet	1	-646.1438215	-194.4548131	-484.2825918	EMD2
CATGAGTAGTGCCCGT-1	singlet	2	-3487.190461	-3279.144885	-915.516166	EMD2
CATGAGTCAAAGGAGA-1	singlet	1	-742.9511706	-272.7549799	-715.21663	EMD2
CATGAGTCACCCAACG-1	singlet	2	-3597.511841	-3213.131823	-1206.899926	EMD2
CATGAGTCAGCCTACG-1	singlet	1	-210.1866554	-66.35636104	-116.6965032	EMD2
CATGAGTCATGTGGTT-1	singlet	2	-6.301620096	-7.554074012	-2.94938169	EMD2
CATGAGTGTACCTATG-1	singlet	2	-925.9179372	-738.786945	-255.1864476	EMD2
CATGAGTTCATACGAC-1	doublet	45323	-5274.278919	-3234.095167	-2623.841718	EMD2
CATGAGTTCTGCATGA-1	singlet	2	-3337.925522	-3205.677938	-996.6886392	EMD2
CATGCAAAGTTTAGGA-1	singlet	1	-2144.093364	-624.2444914	-1564.897198	EMD2
CATGCAACACAAATCC-1	singlet	1	-1464.276904	-497.2660982	-1242.027576	EMD2
CATGCAACAGAACTAA-1	singlet	1	-2563.73576	-731.1144458	-1952.21803	EMD2
CATGCAACAGACCAAG-1	singlet	1	-2382.319496	-805.0673109	-2057.181178	EMD2
CATGCAACAGAGTGAC-1	singlet	0	-449.8949454	-1180.167227	-1060.313359	EMD2
CATGCAACAGGGTCTC-1	singlet	2	-2770.553838	-2425.646387	-785.9370854	EMD2
CATGCAAGTAGATGTA-1	singlet	1	-1265.429531	-393.8605638	-1231.41354	EMD2
CATGCAAGTCGGCTAC-1	doublet	0/1	-507.5304602	-683.5994751	-802.6717005	EMD2
CATGCAAGTGCGTTTA-1	singlet	1	-1197.333952	-417.4935164	-933.1071358	EMD2
CATGCAAGTGGCACTC-1	singlet	0	-893.0816893	-2825.66109	-2773.602053	EMD2
CATGCAAGTTCAGCTA-1	singlet	1	-1284.130508	-459.0464781	-923.676553	EMD2
CATGCAAGTTCTCGCT-1	singlet	1	-311.5949225	-116.5349914	-298.3312186	EMD2
CATGCAATCACAGAGG-1	singlet	0	-969.5844992	-2648.332865	-2579.244052	EMD2

CATGCAATCGAAGCCC-1	singlet	1	-302.3041882	-114.1081665	-249.2591642	EMD2
CATGCAATCTATTCGT-1	singlet	1	-1545.603696	-621.3027493	-1301.12654	EMD2
CATGCCTAGCATCCCG-1	singlet	1	-1028.217505	-343.5846689	-964.1281993	EMD2
CATGCCTCAACTGGTT-1	singlet	1	-783.1458889	-290.7709101	-628.8448455	EMD2
CATGCCTCAGAGTAAT-1	singlet	1	-1059.39641	-355.5290199	-969.9034551	EMD2
CATGCCTCATATCTGG-1	singlet	2	-3162.283779	-2578.412349	-1180.329852	EMD2
CATGCCTGTCCTACAA-1	doublet	45323	-2392.489737	-1563.702274	-1174.230127	EMD2
CATGCCTGTTCGGGTC-1	singlet	0	-181.0111137	-541.0617855	-439.2451567	EMD2
CATGCCTTCGAAATCC-1	singlet	1	-2255.941322	-748.1346101	-1896.529414	EMD2
CATGCCTTCGAGAATA-1	singlet	1	-2687.378862	-875.3786582	-2160.611363	EMD2
CATGCCTTCGTCGACG-1	unassigned	1	-5.147466388	-3.01121193	-14.34093827	EMD2
CATGCGGAGATGGCAC-1	singlet	1	-518.5192789	-182.8696414	-377.0556218	EMD2
CATGCGGAGGGAGAAT-1	singlet	1	-1248.771012	-371.2392899	-959.3770337	EMD2
CATGCGGAGTCAGCCC-1	singlet	0	-1212.516565	-4134.274762	-3923.07223	EMD2
CATGCGGCATCGATCA-1	singlet	1	-1295.29415	-503.5557495	-1199.2269	EMD2
CATGCGGGTCACCACG-1	singlet	2	-2097.148445	-1762.28297	-594.3180285	EMD2
CATGCGGGTGCATTAC-1	singlet	1	-790.9417696	-259.9595178	-635.5104805	EMD2
CATGCGGGTGCCGTTG-1	singlet	1	-452.2195891	-205.7279812	-381.386545	EMD2
CATGCGGGTTGTTGTG-1	singlet	1	-1129.183197	-337.6236259	-993.0473365	EMD2
CATGCGGGTTTGTGGT-1	singlet	0	-634.1387016	-1794.929718	-1786.707407	EMD2
CATGCGGTCATTACGG-1	singlet	2	-1432.704065	-1363.074383	-507.6201518	EMD2
CATGCGGTCCGGCAAC-1	doublet	2/0	-2571.376923	-3412.802438	-2194.096861	EMD2
CATGCGGTCTCGTCAC-1	singlet	1	-353.0965185	-112.3345352	-281.4402406	EMD2
CATGCTCAGTCTGCAT-1	singlet	2	-3652.1811	-2876.51183	-837.4845927	EMD2
CATGCTCCAACAGATA-1	singlet	2	-9634.554151	-7344.926004	-2443.686426	EMD2
CATGCTCCACCTCGTT-1	singlet	1	-4492.586534	-1412.315078	-3999.595711	EMD2
CATGCTCGTACACGTT-1	singlet	0	-944.7129901	-3486.812271	-3393.493076	EMD2
CATGCTCGTTAGCTAC-1	singlet	1	-273.6936925	-109.868508	-226.2026174	EMD2
CATGCTCGTTCAAGTC-1	singlet	0	-428.8481039	-1089.539268	-1040.828747	EMD2
CATGCTCTCTCAACCC-1	singlet	1	-167.2834642	-56.21271734	-114.5730361	EMD2
CATGGATAGTAGAGTT-1	singlet	1	-1661.44244	-492.9002577	-1520.233986	EMD2
CATGGATAGTCAGAGC-1	singlet	0	-126.7546018	-349.4707811	-275.9435692	EMD2
CATGGATCAAATGATG-1	unassigned	1/0	-20.4492974	-14.0326145	-22.94844324	EMD2
CATGGATCAGACACCC-1	singlet	1	-405.1978193	-191.9596906	-396.9291766	EMD2
CATGGATGTAGGTAGC-1	singlet	1	-222.0292277	-75.44217029	-193.8353544	EMD2
CATGGATGTTGTGGCC-1	singlet	2	-10.88699577	-8.365621891	-2.835802864	EMD2
CATGGATTCGTGTTCC-1	singlet	1	-1148.20971	-435.0831581	-971.7159835	EMD2
CATGGATTCTTCCCGA-1	singlet	2	-3574.706599	-3127.969142	-1263.046652	EMD2
CATGGTAAGTGGTGGT-1	singlet	1	-386.7651806	-148.5852464	-294.9208956	EMD2
CATGGTACAGGCTATT-1	singlet	0	-826.415708	-2379.880131	-2193.737306	EMD2
CATGGTACATGACGGA-1	singlet	2	-476.8201324	-475.2991273	-171.5475953	EMD2
CATGGTAGTCGAGATG-1	doublet	0/1	-2917.393057	-4790.275503	-5491.487732	EMD2
CATGGTATCACTTGGA-1	singlet	2	-3375.743245	-2772.581468	-1131.886501	EMD2
CATGGTATCTCTTGCG-1	singlet	2	-1767.418262	-1683.608376	-625.1189009	EMD2
CATTCATAGAGTCAGC-1	singlet	1	-2293.638985	-869.6088172	-1971.97662	EMD2
CATTCATAGCAAATCA-1	singlet	0	-1439.288724	-4451.235047	-4113.813473	EMD2
CATTCATAGCGCCTTG-1	unassigned	1	-6.49795583	-2.44485686	-4.461600255	EMD2
CATTCATAGGCTTAAA-1	unassigned	0/1	-7.295186973	-11.89567197	-12.05513688	EMD2
CATTCATCACAAGCAG-1	singlet	0	-1642.751789	-5746.738145	-5579.134631	EMD2
CATTCATCACCTCTGT-1	singlet	1	-2015.721133	-594.9073673	-1745.099167	EMD2
CATTCATCACTCAGAT-1	singlet	2	-520.8402597	-453.4956842	-228.9515418	EMD2
CATTCATCATACTTTC-1	singlet	2	-1803.004401	-1787.078384	-686.6027692	EMD2
CATTCATCATATACCG-1	singlet	1	-318.9833544	-130.9240779	-307.6150092	EMD2
CATTCATCATTGTACG-1	singlet	1	-59.79621518	-21.33041523	-46.72138587	EMD2
CATTCATGTAGCCAGA-1	singlet	1	-329.8134649	-135.4080711	-257.054342	EMD2
CATTCATTCAGCCTCT-1	singlet	0	-179.9557889	-516.5821538	-385.8399157	EMD2
CATTCATTCGTACACA-1	singlet	1	-307.5017971	-135.0164455	-329.7011754	EMD2
CATTCATTCTTAGGAC-1	singlet	0	-1742.424729	-5453.999492	-5048.445793	EMD2
CATTCCGAGGAGCAAA-1	singlet	1	-343.0497025	-118.7346634	-285.9587916	EMD2
CATTCCGAGGGATCTG-1	singlet	1	-422.0468214	-145.1030673	-337.3753051	EMD2
CATTCCGAGGGTCACA-1	singlet	1	-1199.713931	-414.8018018	-972.2089003	EMD2
CATTCCGCAACAGATA-1	singlet	1	-463.3325682	-181.7391071	-405.1068588	EMD2
CATTCCGCAACCAACT-1	singlet	1	-2579.31566	-779.1100096	-2035.361522	EMD2
CATTCCGCAATCAAGA-1	singlet	2	-4499.20281	-3638.439422	-1305.231119	EMD2
CATTCCGCACATACTG-1	singlet	1	-2739.889219	-742.9497681	-2142.879602	EMD2
CATTCCGTCCTCTCGA-1	singlet	1	-502.5204757	-186.6780539	-380.0778143	EMD2
CATTCCGTCGTCGACG-1	singlet	0	-1136.13458	-3867.649347	-3708.368403	EMD2
CATTCCGTCTTCGTGC-1	singlet	2	-2352.608137	-2531.9956	-656.3500695	EMD2
CATTCTAAGCAACAAT-1	singlet	1	-241.618467	-125.7649929	-255.8060379	EMD2
CATTCTAAGCGTACAG-1	singlet	1	-498.0927008	-173.8958689	-351.5371031	EMD2
CATTCTAAGGCAGCTA-1	singlet	2	-4721.57958	-3950.009304	-1650.073588	EMD2
CATTCTACACGCAAAG-1	singlet	0	-1923.210555	-7824.333828	-7766.664883	EMD2
CATTCTACACTGTCGG-1	singlet	1	-403.5337104	-153.4378457	-314.6078	EMD2
CATTCTACAGCAAGAC-1	singlet	1	-1839.660673	-610.6976217	-1563.465182	EMD2
CATTCTAGTCATCAGT-1	singlet	1	-205.6738808	-87.79722234	-170.145458	EMD2
CATTCTATCAACACCA-1	singlet	2	-3747.252235	-3299.268569	-1293.326085	EMD2
CATTCTATCGACGATT-1	singlet	1	-327.1800769	-173.6330198	-323.0981843	EMD2
CATTCTATCGGAGTAG-1	singlet	1	-798.9307473	-234.8820195	-570.8583721	EMD2
CATTCTATCGGTGAAG-1	singlet	1	-2317.55461	-712.1166673	-1843.945469	EMD2
CATTGAGAGCTGAGTG-1	doublet	45293	-142.7093861	-77.08947545	-115.6148565	EMD2
CATTGAGCATGGAGAC-1	singlet	0	-1228.261935	-6336.711345	-5980.151915	EMD2
CATTGAGGTGTTCAGT-1	doublet	2/0	-29.86084233	-21.57915083	-18.10902496	EMD2
CATTGAGGTTGCAACT-1	singlet	0	-941.655803	-2942.341727	-2808.264799	EMD2
CATTGAGTCTATCGGA-1	singlet	0	-2343.397481	-8715.908051	-8346.06474	EMD2
CATTGAGTCTTCGATT-1	singlet	0	-318.0099164	-909.3008174	-825.9746003	EMD2
CATTGAGTCTTCTCAA-1	singlet	0	-134.8101048	-466.7796294	-410.4150555	EMD2
CATTGCCAGGTGCTAG-1	singlet	1	-956.3366044	-372.4866633	-808.3706804	EMD2
CATTGCCAGTCTGGAG-1	singlet	1	-414.8606868	-182.7369844	-333.1694422	EMD2
CATTGCCCAGCAGTAG-1	singlet	1	-1915.931544	-548.0295792	-1593.828824	EMD2
CATTGCCGTAACGATA-1	unassigned	2	-3.500166518	-1.587005081	-0.854437225	EMD2
CATTGCCGTATAATGG-1	singlet	1	-3871.44671	-1099.97952	-2875.189345	EMD2
CATTGCCGTGAATAAC-1	singlet	2	-2081.144305	-1926.92827	-760.8119185	EMD2
CATTGCCGTTGCCGAC-1	singlet	1	-622.336252	-204.7497803	-507.4656268	EMD2
CATTGCCTCCACGGAC-1	singlet	1	-1082.167526	-335.8734699	-822.1410065	EMD2
CATTGTTAGACATAAC-1	singlet	1	-1653.473403	-658.7872912	-1535.573312	EMD2
CATTGTTAGCTGGCCT-1	singlet	1	-3447.930231	-1001.901913	-2791.252306	EMD2
CATTGTTAGGCCATAG-1	singlet	2	-678.709041	-638.3261542	-330.5365039	EMD2
CATTGTTAGTCCCAGC-1	singlet	2	-3852.313894	-2958.822934	-1081.445282	EMD2
CATTGTTCACTATGTG-1	singlet	1	-1353.013762	-500.9599311	-1015.126078	EMD2
CATTGTTGTCGCAGTC-1	singlet	0	-357.3099916	-1008.253852	-845.2888426	EMD2
CATTGTTGTGCGTTTA-1	singlet	0	-184.7445907	-503.3016899	-468.7280071	EMD2
CATTGTTGTTCTCGTC-1	singlet	0	-839.6889701	-3199.017444	-3178.848525	EMD2
CATTGTTTCATGACAC-1	singlet	0	-609.093292	-1755.11916	-1857.586124	EMD2
CATTGTTTCCTCACCA-1	singlet	0	-3666.189833	-9141.307805	-9456.360111	EMD2
CATTGTTTCGTGGCGT-1	singlet	0	-161.7543115	-578.4773127	-511.7879524	EMD2
CATTTCAAGAGTTCGG-1	singlet	2	-822.1054174	-803.6564582	-289.861241	EMD2
CATTTCAAGCGTCTCG-1	singlet	1	-365.8900586	-111.1606428	-268.975568	EMD2
CATTTCACAATAGTGA-1	singlet	1	-431.7061368	-202.2740956	-330.1902634	EMD2
CATTTCACATGCTGCG-1	singlet	1	-1456.180913	-472.9385598	-1106.809343	EMD2
CATTTCAGTCTACAGT-1	singlet	1	-3158.105636	-760.1060545	-2016.25861	EMD2

CATTTCAGTTATGGTC-1	singlet	1	-3386.46625	-869.2486595	-2221.897477	EMD2
CATTTCATCACCTGTC-1	singlet	0	-49.84394112	-120.9947157	-110.0561354	EMD2
CATTTCATCTCGACCT-1	doublet	1/0	-2276.765779	-2050.657096	-3122.323094	EMD2
CATTTCATCTGGGATT-1	singlet	1	-660.6122513	-258.5743578	-541.1393118	EMD2
CCAAGCGAGGGTTAGC-1	singlet	1	-2841.480233	-926.7614234	-2200.127122	EMD2
CCAAGCGAGTGGTGAC-1	singlet	1	-257.2045127	-111.9620083	-239.4031875	EMD2
CCAAGCGCACACTTAG-1	singlet	1	-1443.017072	-496.4680076	-1044.286436	EMD2
CCAAGCGCACGCAGTC-1	singlet	2	-2358.039128	-2116.3479	-782.488928	EMD2
CCAAGCGCAGTCTTCC-1	singlet	1	-335.0856671	-124.8277451	-286.7093768	EMD2
CCAAGCGGTGAAGCTG-1	singlet	1	-210.5939241	-64.71467041	-152.1053254	EMD2
CCAAGCGGTTCCGCAG-1	singlet	1	-637.0394139	-257.5860712	-558.3772318	EMD2
CCAAGCGTCATTCGTT-1	singlet	2	-4432.522246	-3550.067898	-1107.135326	EMD2
CCAAGCGTCATTGTTC-1	singlet	1	-790.0493282	-251.8001884	-594.8293127	EMD2
CCAAGCGTCCAACTAG-1	singlet	1	-123.3360355	-48.40486544	-78.35887035	EMD2
CCAAGCGTCCAGTGCG-1	singlet	1	-862.4273705	-282.7126573	-694.8269274	EMD2
CCAAGCGTCGGCTGAC-1	singlet	1	-1030.752949	-364.1186535	-844.5693107	EMD2
CCAAGCGTCTGTGCAA-1	singlet	1	-1383.151783	-434.4104985	-1164.35955	EMD2
CCAATGAAGAGAGCAA-1	singlet	1	-2582.821749	-930.4050222	-2216.592961	EMD2
CCAATGAAGCGTTGTT-1	singlet	1	-4634.700732	-1429.515085	-4040.794742	EMD2
CCAATGAAGTACAGAT-1	singlet	1	-410.0381901	-120.2867603	-271.4217912	EMD2
CCAATGAAGTCATACC-1	singlet	0	-1001.557135	-3621.618686	-3481.011278	EMD2
CCAATGAAGTGCACCC-1	singlet	0	-213.6373603	-851.8176241	-776.1426769	EMD2
CCAATGAAGTGCTAGG-1	doublet	0/2	-143.186307	-212.8160305	-187.0096915	EMD2
CCAATGACACAGAGCA-1	singlet	0	-387.0666865	-1272.813665	-1143.702031	EMD2
CCAATGAGTCACTAGT-1	singlet	1	-444.459761	-147.7475067	-404.1395458	EMD2
CCAATGAGTGTCATTG-1	singlet	1	-676.2078112	-231.7247136	-662.1834241	EMD2
CCAATGATCCTTCTAA-1	singlet	1	-11.64684715	-6.650088273	-15.95596273	EMD2
CCAATTTAGCGATCGA-1	singlet	2	-4222.189409	-3610.783875	-1658.16014	EMD2
CCAATTTAGGTTGAGC-1	singlet	0	-302.1653509	-1218.690198	-1024.795821	EMD2
CCAATTTCAACCGGAA-1	singlet	1	-2714.619612	-863.7516558	-2341.474745	EMD2
CCAATTTCACTGGAAG-1	singlet	0	-681.7374922	-2160.194726	-1983.302083	EMD2
CCAATTTCAGAGGGTT-1	singlet	1	-3030.444189	-844.1855141	-2132.097435	EMD2
CCAATTTCAGAGTCTT-1	doublet	45293	-676.6318496	-396.0890473	-418.7883658	EMD2
CCAATTTTCAAACCCA-1	singlet	1	-1139.21618	-365.9529669	-857.2292417	EMD2
CCAATTTTCATTTACC-1	singlet	1	-501.952079	-224.4372219	-442.8505138	EMD2
CCAATTTTCCACTTCG-1	singlet	1	-116.1432683	-74.29095209	-142.9867624	EMD2
CCACAAAAGTCAGCGA-1	singlet	1	-245.7195121	-102.6058108	-216.7373163	EMD2
CCACAAAAGTCATACC-1	singlet	0	-388.0411475	-1182.124494	-1158.368375	EMD2
CCACAAACAGTAGGAC-1	singlet	1	-1022.957743	-379.9473327	-877.202549	EMD2
CCACAAACAGTCGCAC-1	doublet	45293	-83.01810359	-48.10591049	-70.42258438	EMD2
CCACAAAGTCCCGGTA-1	singlet	0	-1504.14859	-5302.970504	-5306.027729	EMD2
CCACAAAGTCGTTCAA-1	singlet	1	-346.4555389	-134.6167101	-306.5892713	EMD2
CCACAAAGTGAGGAAA-1	singlet	1	-1247.003753	-475.1522522	-1128.844728	EMD2
CCACAAAGTTCGGACC-1	singlet	1	-609.5967615	-176.501217	-459.558331	EMD2
CCACAAATCTCATAGG-1	singlet	2	-470.9729453	-379.5599504	-189.5771769	EMD2
CCACACTAGTTTGGCT-1	singlet	1	-2088.104534	-739.4014679	-1770.387383	EMD2
CCACACTCAATTGCCA-1	singlet	1	-55.32961954	-22.95644997	-40.74964491	EMD2
CCACACTCAGCTTTCC-1	singlet	1	-752.8903725	-315.584441	-676.2759624	EMD2
CCACACTGTACAGCGA-1	singlet	1	-3112.331376	-1148.649833	-2601.34374	EMD2
CCACACTGTAGTTACC-1	singlet	2	-383.0735371	-303.3105008	-163.1337538	EMD2
CCACACTGTCACTTAG-1	singlet	0	-1180.464924	-4128.556687	-3929.27429	EMD2
CCACCATAGAAACTGT-1	singlet	0	-943.5809495	-3303.461432	-3175.784641	EMD2
CCACCATAGAATCCCT-1	unassigned	1/0	-130.7341527	-58.37913758	-165.7986509	EMD2
CCACCATAGACTACGG-1	doublet	45293	-7082.915344	-3141.350024	-5139.426924	EMD2
CCACCATAGCCTCTTC-1	singlet	2	-82.78351307	-80.77327942	-30.54227763	EMD2
CCACCATAGGAACTAT-1	singlet	1	-374.7607661	-162.8643437	-377.2618613	EMD2
CCACCATAGGACGGAG-1	singlet	1	-292.4743267	-117.2855175	-268.8406519	EMD2
CCACCATAGGGAGAAT-1	singlet	1	-664.5431909	-241.5487601	-523.2237344	EMD2
CCACCATAGGGCAGGA-1	singlet	1	-692.9012074	-221.9612884	-557.502493	EMD2
CCACCATAGGGTCACA-1	singlet	2	-1072.115826	-1013.21514	-423.023305	EMD2
CCACCATGTAGACAGC-1	singlet	1	-2956.688898	-1375.012202	-2792.154579	EMD2
CCACCATGTATGATCC-1	singlet	1	-623.8192141	-256.7438649	-563.6055229	EMD2
CCACCATGTGAAAGTT-1	unassigned	1/0	-55.32955732	-27.37844582	-32.33658305	EMD2
CCACCATGTGCCGGTT-1	singlet	1	-3309.555032	-1029.300788	-2707.87797	EMD2
CCACCATGTGCGTTTA-1	singlet	1	-1666.936246	-508.2491463	-1417.49088	EMD2
CCACCATTCAGACCTA-1	singlet	1	-5616.391491	-1710.020562	-4381.504785	EMD2
CCACCATTCCATTTAC-1	singlet	1	-451.4641707	-183.4479857	-356.1337265	EMD2
CCACCATTCCTACGGG-1	singlet	1	-284.4722363	-134.383172	-232.1038617	EMD2
CCACCATTCGAGAAAT-1	singlet	1	-1986.489137	-609.6549354	-1488.133003	EMD2
CCACGAGAGAAGCTGC-1	singlet	2	-1151.477359	-1053.812893	-300.7504258	EMD2
CCACGAGAGAATTCAG-1	singlet	2	-85.97788198	-74.98722073	-25.10507993	EMD2
CCACGAGAGACAGCGT-1	singlet	2	-2094.852832	-2022.008676	-655.0507629	EMD2
CCACGAGAGAGCATCG-1	singlet	1	-1498.813919	-443.969065	-1071.409153	EMD2
CCACGAGAGAGTCAAT-1	singlet	1	-3356.120994	-823.5079102	-2639.610015	EMD2
CCACGAGCAACGACTT-1	singlet	2	-2129.594937	-1932.806743	-749.2979595	EMD2
CCACGAGCACAATCTG-1	singlet	1	-1194.347911	-420.4071193	-1036.752273	EMD2
CCACGAGGTACTGTTG-1	singlet	1	-3343.038705	-998.787037	-2521.771468	EMD2
CCACGAGGTATCGAAA-1	singlet	1	-4702.8233	-1488.512914	-3616.231911	EMD2
CCACGAGGTCAATGGG-1	singlet	1	-167.5034574	-46.97583168	-116.8281847	EMD2
CCACGAGGTGACTCTA-1	singlet	1	-2211.204019	-759.286115	-1720.973561	EMD2
CCACGAGGTTGAGGAC-1	singlet	0	-238.7606969	-887.896807	-789.9329601	EMD2
CCACGAGTCATGACAC-1	singlet	1	-5558.101397	-1355.033587	-4416.845323	EMD2
CCACGAGTCGCTGTCT-1	singlet	0	-275.7116377	-826.3741752	-743.3798255	EMD2
CCACGTTCACATGACT-1	singlet	0	-661.2108836	-1685.817388	-1764.528575	EMD2
CCACGTTCACCGGTCA-1	singlet	1	-5333.661331	-1670.52121	-4009.54054	EMD2
CCACGTTCATACAGCT-1	singlet	0	-715.1314857	-2418.489869	-2306.214183	EMD2
CCACGTTCATATGAAG-1	singlet	0	-142.5810519	-276.4946826	-275.7792407	EMD2
CCACGTTGTCATACCA-1	singlet	1	-2035.790951	-581.8502466	-1332.894675	EMD2
CCACGTTGTGCAGGAT-1	singlet	0	-59.26325821	-213.4005462	-173.6600076	EMD2
CCACGTTGTTTGCCGG-1	singlet	1	-718.6675494	-222.9435702	-449.3425741	EMD2
CCACGTTTCCAAGCCG-1	singlet	2	-4215.599131	-3469.354629	-1379.437314	EMD2
CCACGTTTCGCCTAGG-1	singlet	1	-473.1486349	-221.392139	-460.7547025	EMD2
CCACGTTTCGGCGATC-1	singlet	0	-1796.473752	-4451.438595	-3907.986766	EMD2
CCACGTTTCGTGCACG-1	singlet	1	-492.1862186	-215.3909944	-427.4273055	EMD2
CCACTTGAGGATTCCT-1	singlet	0	-107.2973411	-357.159296	-310.4059126	EMD2
CCACTTGAGTGCAGCA-1	singlet	1	-642.0186621	-189.1289749	-445.569972	EMD2
CCACTTGCATTGACTG-1	singlet	1	-1922.559744	-625.4546362	-1645.252634	EMD2
CCACTTGGTATGCGGA-1	singlet	1	-258.6326221	-109.6186277	-258.2321017	EMD2
CCACTTGGTTGGTGTT-1	singlet	1	-2336.58283	-430.760601	-1441.039487	EMD2
CCACTTGTCAACCGAT-1	singlet	2	-2856.700918	-2526.389923	-1082.264661	EMD2
CCACTTGTCGTGCTCT-1	singlet	1	-4888.55937	-1356.446379	-3750.094579	EMD2
CCACTTGTCTACTTCA-1	singlet	2	-2809.578908	-2824.20159	-1041.45689	EMD2
CCATAAGAGCAACCAG-1	singlet	1	-402.2992351	-186.1448127	-379.7018167	EMD2
CCATAAGCAAATGGCG-1	unassigned	45293	-90.67611232	-37.75147621	-59.26476936	EMD2
CCATAAGCACAACGTT-1	singlet	1	-381.6123932	-143.4068513	-270.4712559	EMD2
CCATAAGCATTACTCT-1	singlet	1	-292.6890305	-113.1461451	-230.8510863	EMD2
CCATAAGGTCCTTGTC-1	singlet	1	-10121.37351	-2706.845524	-7917.685477	EMD2
CCATAAGGTGACACAG-1	unassigned	0/1	-11.00219772	-13.46790557	-22.83716257	EMD2
CCATAAGGTGATTCAC-1	singlet	0	-1069.820203	-3179.742042	-2987.633221	EMD2
CCATAAGGTGGTACAG-1	singlet	1	-898.3966848	-264.693445	-619.830561	EMD2

CCATAAGGTTCCCAAA-1	singlet	1	-2811.62474	-927.635726	-2297.303159	EMD2
CCATAAGTCAAAGGTA-1	singlet	2	-2512.573769	-2514.112932	-995.8549749	EMD2
CCATAAGTCAGACATC-1	singlet	0	-201.3291047	-706.4621795	-693.1097878	EMD2
CCATAAGTCATTGCCC-1	singlet	2	-997.6493348	-852.6609994	-416.7351349	EMD2
CCATAAGTCCAACACA-1	singlet	1	-2040.966865	-706.0879314	-1816.242075	EMD2
CCATAAGTCCCGTTCA-1	singlet	1	-1638.342601	-401.1673666	-1281.699166	EMD2
CCATAAGTCCCTTGTG-1	singlet	1	-1314.529044	-414.5011877	-1037.447215	EMD2
CCATAAGTCCGTCCTA-1	singlet	0	-2623.201849	-8679.845537	-8547.994905	EMD2
CCATAAGTCGTCCTTG-1	singlet	1	-2020.314072	-679.5581803	-1642.891512	EMD2
CCATCACAGATGACAT-1	singlet	1	-403.3098617	-177.6901433	-377.3357432	EMD2
CCATCACAGCATCTTG-1	singlet	0	-147.2849212	-291.9545296	-283.9789985	EMD2
CCATCACAGTACTGGG-1	singlet	1	-2237.125004	-683.9012333	-1949.024095	EMD2
CCATCACAGTCGCCCA-1	singlet	1	-979.1569667	-297.0965935	-745.7117856	EMD2
CCATCACAGTTGGAAT-1	singlet	2	-193.2541454	-142.0495572	-63.71762892	EMD2
CCATCACCAAGACAAT-1	singlet	1	-250.3201302	-129.5936041	-243.3044868	EMD2
CCATCACCATACAGGG-1	singlet	1	-873.9283331	-382.266908	-787.3540369	EMD2
CCATCACGTAGGGTAC-1	singlet	1	-3554.480349	-1147.250092	-2815.56967	EMD2
CCATCACGTTATAGCC-1	singlet	2	-3218.732005	-2658.076205	-1072.902346	EMD2
CCATCACGTTCGGCCA-1	singlet	0	-1060.118785	-2850.501801	-2837.708244	EMD2
CCATCACTCGAGATGG-1	singlet	0	-206.8577031	-514.0373811	-497.211658	EMD2
CCATCACTCTCGCAGG-1	singlet	0	-249.7933592	-664.4154417	-602.8584791	EMD2
CCATCACTCTTTGATC-1	singlet	1	-2467.227587	-884.1479289	-2327.701534	EMD2
CCCAACTAGCATTTGC-1	singlet	1	-2486.971846	-882.6031581	-2143.534216	EMD2
CCCAACTAGTCGGCCT-1	singlet	1	-440.4794972	-171.8533111	-367.552252	EMD2
CCCAACTCAAAGACGC-1	singlet	1	-1517.120158	-545.6703343	-1335.550003	EMD2
CCCAACTCATTCATCT-1	singlet	0	-1051.980279	-3937.581881	-3436.143468	EMD2
CCCAACTGTCTGCGCA-1	singlet	1	-354.5517924	-164.0199493	-315.9263168	EMD2
CCCAACTGTGAAGCGT-1	singlet	1	-1305.396895	-413.1155055	-1063.467717	EMD2
CCCAACTGTGAGTAGC-1	singlet	1	-323.0925698	-126.807054	-247.4366739	EMD2
CCCAACTGTGGCTTAT-1	singlet	1	-292.5012423	-126.6281817	-321.67509	EMD2
CCCAACTGTTCGTTCC-1	singlet	2	-753.1205052	-700.9884456	-267.6084381	EMD2
CCCAACTTCCGTGTGG-1	singlet	1	-3978.544149	-1105.268883	-2938.877241	EMD2
CCCAACTTCGAGCCAC-1	singlet	1	-3175.525759	-775.3640367	-2580.044871	EMD2
CCCAACTTCTACTGCC-1	singlet	0	-2596.131287	-8952.591493	-8429.858748	EMD2
CCCAACTTCTGTGCAA-1	singlet	0	-115.0419921	-338.6505003	-252.2515945	EMD2
CCCATTGAGTGATAAC-1	singlet	0	-201.9775233	-498.0322821	-513.1518947	EMD2
CCCATTGCAATCGTCA-1	unassigned	1/0	-6.932913611	-5.135304444	-10.27440888	EMD2
CCCATTGGTCCGAAGA-1	singlet	0	-104.3498689	-325.1011483	-295.9127226	EMD2
CCCATTGGTGGAGGTT-1	singlet	1	-729.8952841	-242.3123107	-671.8809583	EMD2
CCCATTGGTTACACAC-1	singlet	0	-757.789664	-2241.146805	-1937.23289	EMD2
CCCATTGTCCTGATAG-1	singlet	1	-1405.081784	-480.2628501	-1181.190855	EMD2
CCCATTGTCGTTGCCT-1	doublet	45323	-64.17971923	-47.27412705	-43.76806624	EMD2
CCCATTGTCGTTGTGA-1	singlet	1	-940.4609804	-316.1744209	-694.7773398	EMD2
CCCATTGTCTGCGGAC-1	singlet	2	-1586.82551	-1400.526588	-436.3910301	EMD2
CCCGAAGAGATGTTCC-1	singlet	1	-329.7883072	-140.1577252	-288.0372658	EMD2
CCCGAAGAGCAACAAT-1	singlet	1	-141.3751196	-42.65312909	-107.113599	EMD2
CCCGAAGAGCTTCTAG-1	singlet	1	-241.7809749	-75.34086523	-212.5657559	EMD2
CCCGAAGAGGATATGT-1	singlet	1	-698.9954548	-250.8318452	-486.7246168	EMD2
CCCGAAGAGGTCTTTG-1	singlet	1	-1260.003863	-414.320237	-933.1004343	EMD2
CCCGAAGAGTTTGGCT-1	singlet	1	-657.0709755	-220.8045138	-455.5776988	EMD2
CCCGAAGCAAGACCGA-1	singlet	1	-451.7181898	-185.372676	-388.3014842	EMD2
CCCGAAGCAATCTAGC-1	singlet	1	-980.5199178	-396.3028448	-893.4138083	EMD2
CCCGAAGCAGCACACC-1	singlet	1	-1506.109894	-518.996303	-1293.26539	EMD2
CCCGAAGCAGTAACGG-1	singlet	1	-1077.081922	-395.9507374	-921.4485421	EMD2
CCCGAAGTCGGAGCAA-1	singlet	1	-1522.616931	-533.3732822	-1215.554799	EMD2
CCCGAAGTCTAGACAC-1	singlet	1	-113.0598132	-45.21738523	-113.0538777	EMD2
CCCGGAAAGCTAATCC-1	singlet	1	-263.5901638	-87.71288031	-196.4702122	EMD2
CCCGGAAAGGTCACTT-1	singlet	1	-2489.251465	-800.7727592	-1930.166708	EMD2
CCCGGAACAACTAGAA-1	singlet	1	-464.742123	-168.5225074	-395.7091681	EMD2
CCCGGAACACGTACAT-1	doublet	45293	-29.82359431	-13.85072772	-14.70618344	EMD2
CCCGGAACACTCATAG-1	singlet	1	-31.48704981	-19.80788704	-25.12332069	EMD2
CCCGGAAGTAGAGCTG-1	singlet	0	-428.8921746	-1314.798563	-1167.603235	EMD2
CCCGGAAGTGTGTACT-1	singlet	2	-492.7115444	-421.0451358	-194.7189804	EMD2
CCCGGAAGTGTTCCTC-1	singlet	2	-3540.686331	-2794.001119	-1123.89845	EMD2
CCCGGAAGTTTGGGAG-1	singlet	2	-352.5593112	-372.2850463	-134.0750712	EMD2
CCCGGAATCAGAATAG-1	singlet	1	-1459.663459	-479.4689305	-1257.285169	EMD2
CCCGGAATCCCATTCG-1	singlet	1	-77.90538426	-25.86618838	-89.89342497	EMD2
CCCGGAATCCGATGTA-1	singlet	0	-289.8114797	-1013.611303	-848.1725453	EMD2
CCCTAACAGACCAGAC-1	singlet	1	-1095.006723	-396.5222525	-905.7286933	EMD2
CCCTAACAGGCCATAG-1	singlet	1	-486.3565465	-184.7348993	-380.8951239	EMD2
CCCTAACCACTGCATA-1	singlet	1	-1599.159402	-528.803844	-1386.820805	EMD2
CCCTAACCATAGCTGT-1	singlet	1	-513.0941411	-187.9870006	-344.6705864	EMD2
CCCTAACGTGGTCCGT-1	singlet	2	-757.2654511	-734.1663112	-310.7737664	EMD2
CCCTAACTCCATAGAC-1	singlet	2	-298.5459161	-385.9683818	-144.1614425	EMD2
CCCTAACTCCTGGGTG-1	singlet	1	-1105.879359	-410.4724154	-888.5365136	EMD2
CCCTAACTCGTCGATA-1	singlet	1	-1638.090668	-514.9395877	-1467.898752	EMD2
CCCTAACTCTGCCTGT-1	singlet	1	-1835.801744	-499.3638899	-1277.236936	EMD2
CCCTAACTCTTCCAGC-1	singlet	1	-2868.095891	-941.9289205	-2428.225527	EMD2
CCCTCAAAGATGGTCG-1	singlet	0	-416.0606255	-1072.630415	-1057.105911	EMD2
CCCTCAACAAGTGCAG-1	singlet	1	-1853.400084	-621.5320445	-1406.665417	EMD2
CCCTCAACACAAACGG-1	singlet	1	-2866.401016	-754.3205759	-2368.311588	EMD2
CCCTCAACAGACTCTA-1	singlet	1	-187.2529647	-55.00694673	-143.4576036	EMD2
CCCTCAAGTTAAGCAA-1	singlet	1	-1534.608546	-551.6894889	-1305.326919	EMD2
CCCTCAATCGCCCAGA-1	singlet	1	-2200.145266	-739.558267	-1837.347878	EMD2
CCCTCTCAGGGCAATC-1	singlet	0	-41.85954749	-138.6391567	-121.5470471	EMD2
CCCTCTCCAACCCGCA-1	singlet	1	-6743.370221	-1937.716475	-5299.865375	EMD2
CCCTCTCCATGCCATA-1	singlet	2	-1262.726835	-1267.502795	-489.8219443	EMD2
CCCTCTCGTCATAAAG-1	singlet	2	-2331.413967	-2245.58156	-821.5487603	EMD2
CCCTCTCGTCCTCCAT-1	singlet	1	-1007.205697	-393.5163176	-916.2195531	EMD2
CCCTCTCGTTCAAAGA-1	singlet	1	-298.2839832	-131.1706542	-254.9441449	EMD2
CCCTCTCGTTGGAGAC-1	singlet	1	-1285.679634	-436.7706047	-995.9496245	EMD2
CCCTCTCTCTAGCCAA-1	singlet	2	-1816.683778	-1667.147428	-757.4263529	EMD2
CCCTCTCTCTCCGAAA-1	singlet	1	-528.3623517	-198.8670684	-525.7179571	EMD2
CCCTGATAGATCCCAT-1	singlet	1	-2868.805281	-883.998781	-2619.939731	EMD2
CCCTGATCAATAAGGT-1	singlet	0	-205.9250277	-539.2635132	-452.884449	EMD2
CCCTGATCATAACAGA-1	singlet	1	-3185.279037	-1027.258211	-2545.537586	EMD2
CCCTGATGTATGAAGT-1	singlet	1	-5264.507175	-1682.484353	-3973.75091	EMD2
CCCTGATGTCACTTAG-1	singlet	1	-4111.953351	-1366.100514	-3289.721202	EMD2
CCCTGATGTCTTGCTC-1	singlet	1	-322.5867537	-137.1443369	-271.3789301	EMD2
CCCTGATGTGACATCT-1	singlet	1	-1108.775183	-412.8463482	-937.0186347	EMD2
CCCTGATGTGCAGGAT-1	singlet	1	-357.4574569	-154.2856368	-289.5941287	EMD2
CCCTGATGTTAAGGGC-1	singlet	1	-477.7717281	-164.7154348	-387.5373115	EMD2
CCCTGATGTTCCATTT-1	singlet	1	-637.9297774	-255.567256	-548.4756253	EMD2
CCCTGATTCAATGTCG-1	singlet	2	-457.938481	-375.0326441	-165.6644805	EMD2
CCCTGATTCCGTGCGA-1	singlet	1	-867.4633449	-286.7013987	-742.2887833	EMD2
CCCTTAGAGATTCGCT-1	singlet	1	-3705.73057	-1201.069704	-2786.398676	EMD2
CCCTTAGAGTAGGGTC-1	singlet	2	-992.2598816	-1044.870868	-409.2717856	EMD2
CCCTTAGAGTATCTGC-1	singlet	2	-1954.608781	-1735.919514	-665.6823577	EMD2
CCCTTAGAGTTGCGCC-1	singlet	1	-252.0623981	-98.87318422	-213.2189432	EMD2
CCCTTAGCAAGTGTCT-1	singlet	2	-310.8170413	-278.9002696	-128.1323437	EMD2

CCCTTAGCACCCTGAG-1	singlet	0	-1133.219807	-3337.642403	-3363.385774	EMD2
CCCTTAGCACTAGGTT-1	singlet	2	-472.9898952	-499.618466	-205.8542967	EMD2
CCCTTAGCAGCGTATT-1	singlet	1	-778.7864561	-278.7656892	-625.8351084	EMD2
CCCTTAGGTAGGGTAC-1	doublet	1/0	-382.2035068	-374.7211395	-464.1050474	EMD2
CCCTTAGGTTAAACCC-1	unassigned	1/0	-3.78103859	-2.014203298	-2.184247029	EMD2
CCCTTAGTCAGCGTCG-1	singlet	2	-463.7651361	-352.3271548	-138.4041777	EMD2
CCCTTAGTCCGAACGC-1	singlet	1	-285.8766773	-117.293919	-206.0188129	EMD2
CCGAACGAGGATCACG-1	singlet	0	-518.0181729	-2067.284519	-1778.533318	EMD2
CCGAACGAGTCAAGCG-1	singlet	0	-1226.236513	-3436.411059	-3140.072052	EMD2
CCGAACGAGTGTAGAT-1	singlet	1	-1183.429182	-383.2844052	-985.193205	EMD2
CCGAACGCAAATTAGG-1	singlet	0	-612.54003	-1496.418211	-1546.786683	EMD2
CCGAACGCAATACCCA-1	singlet	2	-1484.344235	-1243.00268	-537.5034927	EMD2
CCGAACGCATGATGCT-1	singlet	1	-1502.481939	-512.3901866	-1252.417672	EMD2
CCGAACGGTCTTCCGT-1	singlet	1	-3997.40383	-1277.261065	-3239.389346	EMD2
CCGAACGGTGGCGTAA-1	singlet	0	-275.2173523	-862.184814	-874.9509353	EMD2
CCGAACGGTTTATGCG-1	singlet	0	-1772.245123	-5751.593593	-5768.179312	EMD2
CCGAACGTCACAGAGG-1	singlet	0	-619.0006542	-1731.71243	-1747.874801	EMD2
CCGAACGTCGATCCCT-1	singlet	2	-478.0668527	-376.0968979	-180.1786072	EMD2
CCGATCTAGAATCGAT-1	singlet	0	-383.2487269	-1192.09563	-1177.015503	EMD2
CCGATCTAGGAACGCT-1	singlet	0	-268.6181243	-734.6594587	-720.5052418	EMD2
CCGATCTCAAGCCATT-1	singlet	1	-683.7739016	-258.9672751	-557.0580438	EMD2
CCGATCTCACAAGGTG-1	singlet	0	-20.00412291	-29.15371406	-33.80212443	EMD2
CCGATCTGTCGAGCTC-1	singlet	0	-36.61088664	-125.3247668	-114.3791041	EMD2
CCGATCTGTGTATTGC-1	singlet	1	-1112.279243	-390.8240332	-851.0216219	EMD2
CCGATCTTCATGGTAC-1	doublet	0/2	-3606.522447	-5811.371988	-3911.495968	EMD2
CCGATCTTCGTGTCAA-1	singlet	1	-196.3226805	-90.7910332	-163.0863799	EMD2
CCGATCTTCTACGGTA-1	singlet	1	-270.3208423	-129.8984728	-262.8198257	EMD2
CCGATGGAGACATATG-1	singlet	0	-74.3959005	-175.2273045	-144.1439252	EMD2
CCGATGGAGTCGAAGC-1	singlet	2	-1018.984368	-928.3582897	-374.7279599	EMD2
CCGATGGCAAGTGCTT-1	singlet	1	-2318.585874	-812.4736721	-1816.01261	EMD2
CCGATGGCACCACATA-1	singlet	0	-544.4047827	-1511.053115	-1308.933554	EMD2
CCGATGGGTTAATGAG-1	singlet	0	-2200.74942	-7414.223905	-7204.8317	EMD2
CCGATGGGTTGCTCCT-1	singlet	1	-988.5000788	-351.6931012	-853.6953595	EMD2
CCGATGGTCCGTGGTG-1	singlet	1	-3053.441305	-827.0303459	-2432.438312	EMD2
CCGATGGTCGCTACAA-1	singlet	0	-163.9892017	-576.2100149	-469.9100032	EMD2
CCGCAAGAGAGGGCGA-1	singlet	1	-1625.705475	-507.2127845	-1513.71735	EMD2
CCGCAAGAGCGGTATG-1	singlet	0	-886.2111729	-3094.175747	-2902.895845	EMD2
CCGCAAGAGGACAGTC-1	singlet	2	-49.99071243	-29.39460848	-17.95048175	EMD2
CCGCAAGAGGAGGCAG-1	singlet	2	-379.8427154	-319.7147174	-142.9994441	EMD2
CCGCAAGAGGGTATAT-1	singlet	1	-577.1477928	-190.6295996	-417.7972561	EMD2
CCGCAAGCACAGCCTG-1	singlet	2	-4376.139735	-3692.840388	-1355.671596	EMD2
CCGCAAGCACTGAGGA-1	singlet	0	-813.0410083	-3265.54818	-3131.259347	EMD2
CCGCAAGCACTGATTG-1	singlet	1	-2604.648013	-891.3633974	-2111.694489	EMD2
CCGCAAGCATCCGATA-1	singlet	1	-368.8074227	-121.4044125	-316.6162	EMD2
CCGCAAGCATGTTCAG-1	singlet	1	-4224.77902	-1464.727093	-3381.415391	EMD2
CCGCAAGGTACGTGAG-1	singlet	1	-2299.978996	-657.5334786	-1810.957598	EMD2
CCGCAAGTCACCTTGC-1	singlet	0	-1071.908081	-2996.758741	-2812.84224	EMD2
CCGCAAGTCACTGCTC-1	doublet	0/1	-2012.164183	-2569.245241	-3131.287908	EMD2
CCGCAAGTCCAGGACC-1	singlet	1	-163.059395	-58.31698638	-125.1318224	EMD2
CCGCAAGTCGGTAACT-1	singlet	1	-445.1955068	-182.7235079	-335.627211	EMD2
CCGCAAGTCTGTCGCT-1	singlet	1	-8150.349994	-2406.26421	-6264.985666	EMD2
CCGGACAAGGACAGCT-1	singlet	1	-2335.016574	-777.3098009	-1883.169991	EMD2
CCGGACACAGGCGATA-1	singlet	1	-218.2159802	-108.4455066	-211.8844346	EMD2
CCGGACAGTACGTGTT-1	singlet	1	-4318.068675	-1109.492832	-3268.6506	EMD2
CCGGACAGTCGTGGAA-1	singlet	0	-394.5038238	-1260.300652	-1253.522812	EMD2
CCGGACAGTGGCGCTT-1	singlet	1	-97.60630637	-35.47902206	-58.21633192	EMD2
CCGGACATCCCATTTA-1	singlet	1	-358.2903464	-99.81265905	-265.6234769	EMD2
CCGGACATCCCTTGTG-1	singlet	2	-719.3314817	-632.8994615	-254.5531118	EMD2
CCGGACATCGCCAGTG-1	singlet	1	-1253.441217	-471.2367509	-1090.605122	EMD2
CCGGACATCTCGCGTT-1	singlet	0	-208.8992628	-637.9962156	-636.7533835	EMD2
CCGGGTAAGAATCGCG-1	singlet	1	-3221.711975	-895.2876254	-2584.981839	EMD2
CCGGGTACAAACGGCA-1	singlet	0	-281.8001152	-873.9693801	-854.3497118	EMD2
CCGGGTACAAATAGCA-1	singlet	0	-335.5930472	-976.414255	-898.245815	EMD2
CCGGGTACAATTTCTC-1	singlet	1	-2042.440776	-670.1903372	-1636.743503	EMD2
CCGGGTACACGAGGTA-1	singlet	0	-696.4127467	-2393.923303	-2012.560891	EMD2
CCGGGTACAGTTAAAG-1	singlet	1	-3559.004731	-1005.951337	-2823.602064	EMD2
CCGGGTAGTAACAAGT-1	singlet	1	-1141.406887	-349.4286293	-1028.681449	EMD2
CCGGGTAGTAAGCTCT-1	singlet	0	-733.9479451	-1846.288066	-1655.125267	EMD2
CCGGGTAGTACTAGCT-1	singlet	0	-48.45621156	-177.0922791	-128.8416318	EMD2
CCGGGTAGTATGGTAA-1	singlet	0	-25.06391269	-55.74610201	-56.0360287	EMD2
CCGGGTAGTCGGTGAA-1	singlet	1	-224.8029017	-87.66204505	-202.0702073	EMD2
CCGGGTAGTGATTCTG-1	singlet	0	-339.6515508	-1250.580294	-1073.598668	EMD2
CCGGGTATCCGGCAAC-1	singlet	0	-1505.652915	-4602.09645	-4452.591138	EMD2
CCGGGTATCCGTGCGA-1	singlet	1	-1915.037431	-632.1487062	-1402.012412	EMD2
CCGGGTATCGCGTGCA-1	singlet	1	-306.7027033	-143.4093164	-292.1310021	EMD2
CCGGTAGAGGGACAGG-1	singlet	1	-242.8396981	-119.1601789	-227.4391988	EMD2
CCGGTAGCAATAGTGA-1	singlet	1	-3125.208537	-1041.196482	-2626.113146	EMD2
CCGGTAGCAATTGCTG-1	singlet	1	-421.2778842	-179.423765	-359.2122256	EMD2
CCGGTAGCACAGTATC-1	singlet	1	-3729.413225	-1173.299356	-2894.134238	EMD2
CCGGTAGCAGGACTTT-1	singlet	1	-143.6237302	-70.6754706	-152.699714	EMD2
CCGGTAGCATGACTCA-1	singlet	0	-2.901535216	-6.25759187	-6.913914956	EMD2
CCGGTAGGTAATTAGG-1	singlet	1	-843.0687448	-296.9362757	-695.9624367	EMD2
CCGGTAGGTCAGATTC-1	singlet	1	-1251.768301	-313.947176	-814.0714916	EMD2
CCGGTAGGTCAGTTTG-1	singlet	1	-2320.382643	-897.6417056	-1871.029934	EMD2
CCGGTAGGTTCGTTCC-1	singlet	0	-171.9517188	-588.8156135	-543.7688522	EMD2
CCGGTAGTCACTGCTC-1	singlet	1	-347.961535	-143.9256572	-331.4489464	EMD2
CCGGTAGTCCTATTTG-1	unassigned	1/0	-15.09258132	-11.62054922	-9.330483819	EMD2
CCGGTAGTCGGACTTA-1	singlet	0	-895.2091488	-1940.184141	-2206.878457	EMD2
CCGGTAGTCTATTCGT-1	singlet	1	-3041.677437	-1049.543971	-2535.719775	EMD2
CCGGTAGTCTGCGAGC-1	singlet	1	-891.5875948	-308.5736735	-733.2343349	EMD2
CCGGTGAAGACTGTTC-1	singlet	1	-1712.98544	-610.5288991	-1495.407839	EMD2
CCGGTGAAGCAATTCC-1	singlet	2	-2555.050454	-2004.744845	-832.4305227	EMD2
CCGGTGAAGCCACAAG-1	singlet	0	-470.8784995	-1212.504674	-1251.308727	EMD2
CCGGTGAAGCTCTGTA-1	singlet	1	-1538.978749	-480.3462594	-1246.944701	EMD2
CCGGTGACACAGAGCA-1	singlet	1	-135.4964186	-70.32711598	-128.9905892	EMD2
CCGGTGACACTCTCGT-1	singlet	0	-517.0648359	-1397.180082	-913.9663348	EMD2
CCGGTGACACTGGACC-1	singlet	1	-548.5715763	-231.286446	-504.9986915	EMD2
CCGGTGACAGTGGTGA-1	singlet	1	-6192.79461	-1726.897212	-4661.623364	EMD2
CCGGTGACATTGACAC-1	singlet	1	-252.0707857	-102.7120445	-244.4967434	EMD2
CCGGTGAGTCGACTTA-1	singlet	1	-454.4875414	-161.1412058	-340.84979	EMD2
CCGGTGAGTCTGTGTA-1	singlet	1	-3252.026055	-1019.521363	-2462.680313	EMD2
CCGGTGAGTGACCGTC-1	singlet	2	-500.5025891	-491.343839	-209.1228934	EMD2
CCGGTGAGTGTCCTAA-1	singlet	1	-645.8510086	-188.6821864	-613.652146	EMD2
CCGGTGAGTGTCTAAC-1	singlet	1	-284.6297769	-116.0618224	-250.5829412	EMD2
CCGGTGAGTTATCTTC-1	singlet	0	-996.9990656	-2951.162599	-2930.444559	EMD2
CCGGTGATCACCACAA-1	singlet	2	-29.91436607	-11.62965726	-9.001774532	EMD2
CCGGTGATCCCAGGAC-1	singlet	2	-2167.684762	-1790.57807	-737.6987684	EMD2
CCGGTGATCTGGAAGG-1	singlet	2	-5973.258373	-4505.079795	-1413.158252	EMD2
CCGTAGGAGAAACACT-1	singlet	0	-27.72768643	-91.22613787	-86.38955064	EMD2
CCGTAGGAGGATTTCC-1	singlet	1	-2332.737338	-606.102702	-1784.266485	EMD2

CCGTAGGAGGGCAACT-1	singlet	0	-2678.557882	-6366.388807	-6535.872948	EMD2
CCGTAGGAGGTGGTTG-1	singlet	1	-1886.697627	-492.3937493	-1209.398789	EMD2
CCGTAGGCACGCCAGT-1	singlet	1	-2848.136756	-925.9056576	-2281.84541	EMD2
CCGTAGGCAGAGAATT-1	singlet	1	-1980.752267	-674.556685	-1782.860136	EMD2
CCGTAGGCAGTCGCAC-1	singlet	1	-1328.082621	-418.5446566	-1019.997198	EMD2
CCGTAGGGTAGATTGA-1	singlet	0	-902.9886843	-3839.590238	-3576.40138	EMD2
CCGTAGGGTGACAACG-1	singlet	0	-489.0914831	-1165.037898	-1097.828747	EMD2
CCGTAGGGTTCAACGT-1	singlet	1	-2116.022632	-846.4440002	-1937.366539	EMD2
CCGTAGGTCAATGTCG-1	singlet	1	-2002.761725	-575.4045703	-1515.362218	EMD2
CCGTAGGTCGCCTCTA-1	singlet	1	-261.1564116	-121.2372252	-252.0668703	EMD2
CCGTAGGTCGTCAAAC-1	singlet	1	-262.2551237	-99.18793383	-202.3259641	EMD2
CCGTGAGAGTAGTGCG-1	singlet	1	-320.9825775	-102.7459197	-260.9640149	EMD2
CCGTGAGCAAACCGGA-1	singlet	0	-574.3458663	-1997.340782	-1927.897364	EMD2
CCGTGAGCAAGCACCC-1	singlet	0	-386.5429297	-1144.293506	-1123.031251	EMD2
CCGTGAGCACACGTGC-1	singlet	1	-307.0168951	-131.6691337	-319.4719854	EMD2
CCGTGAGCACAGCTGC-1	singlet	1	-6.791333665	-2.377029767	-8.343708695	EMD2
CCGTGAGCATGTAACC-1	singlet	0	-328.6730129	-1213.925202	-1040.870608	EMD2
CCGTGAGGTACAGTCT-1	singlet	2	-238.1561237	-157.9465547	-74.51415742	EMD2
CCGTGAGGTTCAATCG-1	singlet	1	-2121.472024	-584.1088186	-1780.894566	EMD2
CCGTTCAAGAAGCTCG-1	singlet	1	-6511.023244	-1850.032942	-5049.242128	EMD2
CCGTTCAAGGGTGAGG-1	singlet	0	-118.2543194	-490.422547	-434.110139	EMD2
CCGTTCAAGGTCTGGA-1	singlet	1	-1793.84084	-592.7363645	-1580.456884	EMD2
CCGTTCACACCCAAGC-1	singlet	1	-334.1431145	-126.2842895	-313.38786	EMD2
CCGTTCACACTATGTG-1	singlet	1	-3087.279906	-1025.727664	-2630.675158	EMD2
CCGTTCAGTACGATTC-1	singlet	2	-703.9030364	-706.542597	-272.1723123	EMD2
CCGTTCAGTGGGAGAG-1	singlet	1	-425.4785227	-153.8802972	-330.3976866	EMD2
CCGTTCAGTTCCATTT-1	singlet	2	-827.7712141	-700.8113978	-323.9782719	EMD2
CCGTTCATCCTTATAC-1	doublet	2/0	-4899.553122	-6125.024598	-3418.983953	EMD2
CCGTTCATCTAGCCTC-1	singlet	2	-345.9664669	-340.1828777	-154.6095363	EMD2
CCTAACCAGATGAATC-1	singlet	1	-1514.61832	-362.6295857	-1158.996312	EMD2
CCTAACCAGCAGCACA-1	singlet	1	-660.5600077	-231.2104762	-468.7468557	EMD2
CCTAACCAGGAATGTT-1	singlet	1	-2862.474379	-998.7235216	-2355.036381	EMD2
CCTAACCAGTCAACAA-1	singlet	0	-2264.862744	-7702.603648	-7314.821553	EMD2
CCTAACCCAAACTCGT-1	singlet	1	-931.8131906	-307.59538	-804.3582074	EMD2
CCTAACCCACTTTAGG-1	singlet	1	-1603.354915	-497.5765379	-1109.122177	EMD2
CCTAACCCAGCTATTG-1	singlet	1	-1569.082725	-516.6947047	-1296.539582	EMD2
CCTAACCCATAGACTC-1	singlet	2	-864.1660737	-886.4519796	-306.5501286	EMD2
CCTAACCCATATGGCT-1	singlet	1	-1088.834189	-355.5103775	-959.4376202	EMD2
CCTAACCTCCTCAGGG-1	singlet	1	-1283.026091	-395.2442854	-1025.682945	EMD2
CCTAACCTCGGCATCG-1	singlet	1	-388.3305236	-154.6192853	-302.0578995	EMD2
CCTAACCTCGGCCAAC-1	singlet	2	-677.961897	-576.5551825	-319.5690095	EMD2
CCTAAGAAGACTACCT-1	singlet	2	-3273.584519	-2676.756479	-1116.506149	EMD2
CCTAAGAAGCCTGGAA-1	singlet	1	-2605.471541	-728.5153248	-1957.607677	EMD2
CCTAAGAAGGAATGTT-1	singlet	1	-2847.19783	-851.8472884	-2291.300199	EMD2
CCTAAGACAAGCAGGT-1	singlet	1	-2101.078273	-751.8088062	-1907.732538	EMD2
CCTAAGACACAATGTC-1	singlet	1	-2111.180528	-645.8888329	-1985.297477	EMD2
CCTAAGAGTACTTCCC-1	singlet	1	-404.8257007	-142.7186853	-344.5439491	EMD2
CCTAAGAGTCAAAGAT-1	singlet	0	-843.0133356	-2525.95485	-2466.723747	EMD2
CCTAAGAGTCTTGGTA-1	singlet	1	-410.4452859	-148.984697	-341.1240221	EMD2
CCTAAGAGTGGCTTAT-1	singlet	1	-177.6447251	-70.92908147	-168.8696791	EMD2
CCTAAGAGTTCTCCCA-1	unassigned	2	-8.064110636	-9.965534308	-5.245388766	EMD2
CCTAAGAGTTTAAGGA-1	singlet	1	-3092.086829	-964.1996863	-2531.112226	EMD2
CCTACGTAGAGTTGCG-1	singlet	1	-2935.658942	-982.991615	-2354.556897	EMD2
CCTACGTAGGCGAACT-1	singlet	0	-796.3820322	-2302.128156	-2302.220431	EMD2
CCTACGTAGGTGCATG-1	singlet	2	-3023.328059	-2862.355222	-866.3620746	EMD2
CCTACGTCACACTTAG-1	singlet	1	-1176.808079	-381.9168119	-1048.681794	EMD2
CCTACGTCAGCGGTTC-1	singlet	2	-128.8901498	-150.5657703	-50.0105562	EMD2
CCTACGTGTTCAAGTC-1	singlet	2	-2264.009701	-2043.244234	-707.6819064	EMD2
CCTATCGAGAGAACCC-1	singlet	1	-573.4201204	-251.368041	-549.5264826	EMD2
CCTATCGAGGTATCTC-1	singlet	1	-1963.576653	-640.126909	-1610.254095	EMD2
CCTATCGCAAGAATAC-1	singlet	1	-970.9189445	-295.3135659	-651.3267063	EMD2
CCTATCGCATAACGGG-1	singlet	1	-1417.675283	-470.5459302	-1239.862437	EMD2
CCTATCGCATCCGTGG-1	singlet	1	-646.9451683	-144.0603795	-501.1355893	EMD2
CCTATCGGTACTCGCG-1	singlet	2	-4445.275357	-3752.066046	-1152.029067	EMD2
CCTATCGGTCGAAACG-1	singlet	1	-246.786397	-84.5112278	-194.4866069	EMD2
CCTATCGGTGTAGGAC-1	singlet	1	-1586.341817	-463.4134757	-1470.966562	EMD2
CCTATCGTCAAACGAA-1	singlet	1	-416.6550716	-159.58201	-355.0631786	EMD2
CCTATCGTCCGCTAGG-1	singlet	0	-26.14327137	-47.14427039	-49.5088066	EMD2
CCTATCGTCGTAATGC-1	singlet	0	-2106.399794	-6937.865843	-6639.390883	EMD2
CCTATCGTCTCCTGTG-1	singlet	1	-325.4346142	-126.2843401	-262.5385932	EMD2
CCTATCGTCTCTGGTC-1	doublet	0/1	-3146.903498	-4032.920036	-4951.957127	EMD2
CCTCAACAGATTTGCC-1	singlet	1	-2306.841856	-771.4106143	-1810.444965	EMD2
CCTCAACAGCACACCC-1	singlet	1	-3634.899968	-1165.706162	-3141.798841	EMD2
CCTCAACAGCTCCATA-1	singlet	0	-984.7451687	-3444.822006	-2846.383008	EMD2
CCTCAACAGGGAGGTG-1	singlet	1	-2561.756193	-877.9435744	-2312.459096	EMD2
CCTCAACCAACAAGTA-1	singlet	0	-1506.161313	-4284.330287	-4148.947579	EMD2
CCTCAACCAGACTCTA-1	singlet	1	-2830.135216	-943.0677014	-2188.436535	EMD2
CCTCAACCAGCTAACT-1	singlet	1	-2200.125555	-683.1313739	-1783.215345	EMD2
CCTCAACGTAGGTAGC-1	singlet	1	-682.8608225	-205.8794407	-549.0990532	EMD2
CCTCAACGTCACAGTT-1	singlet	1	-271.3256617	-125.2519635	-261.7032625	EMD2
CCTCAACGTCGGCACT-1	singlet	1	-1697.903921	-577.8389294	-1321.072061	EMD2
CCTCAACGTGAGCCAA-1	singlet	1	-1023.275666	-372.2431009	-828.4578109	EMD2
CCTCAACGTGGTAATA-1	singlet	2	-2681.709531	-2252.126412	-949.4231431	EMD2
CCTCAACGTGTCCTAA-1	singlet	1	-3182.652354	-1007.852991	-2671.787134	EMD2
CCTCAACTCACCGACG-1	doublet	0/1	-1230.69066	-2103.618658	-2205.535464	EMD2
CCTCAACTCCGGTAGC-1	singlet	0	-1452.782373	-4121.331085	-4127.511192	EMD2
CCTCAACTCGACGTCG-1	singlet	1	-309.3431923	-130.3040018	-285.9667032	EMD2
CCTCAACTCGGTAAGG-1	singlet	2	-1277.902603	-1324.996698	-436.9067629	EMD2
CCTCACAAGACGCATG-1	singlet	1	-118.706523	-42.68654336	-91.61092959	EMD2
CCTCACAAGATGCAGC-1	singlet	0	-727.0195374	-2190.196239	-2154.720391	EMD2
CCTCACAAGATTGATG-1	singlet	1	-5681.648265	-1862.329522	-4561.458196	EMD2
CCTCACAAGGGATCGT-1	singlet	1	-555.8949369	-235.3953495	-487.3051254	EMD2
CCTCACAAGGTTATAG-1	singlet	1	-3663.08345	-1075.843317	-2997.657373	EMD2
CCTCACAAGGTTGGAC-1	singlet	2	-3957.758032	-3078.956988	-1032.682193	EMD2
CCTCACAGTTAGGACG-1	singlet	1	-474.8039388	-204.098192	-436.6638757	EMD2
CCTCACATCCGCACGA-1	singlet	1	-2018.590519	-741.4817378	-1696.237233	EMD2
CCTCACATCTAGGCAT-1	singlet	1	-381.5504854	-159.7321609	-305.4030975	EMD2
CCTCAGTAGAGGGTAA-1	singlet	0	-1769.521655	-5202.889361	-5084.801355	EMD2
CCTCAGTAGCCTATTG-1	singlet	1	-3977.392501	-1266.844874	-3112.24396	EMD2
CCTCAGTAGTAGTCAA-1	singlet	1	-1615.905019	-628.3418905	-1357.536081	EMD2
CCTCAGTAGTCGGCAA-1	singlet	1	-1727.07848	-556.6490596	-1322.772459	EMD2
CCTCAGTAGTTTCGAC-1	singlet	0	-132.5078886	-470.7034562	-393.6241981	EMD2
CCTCAGTGTATGGGAC-1	singlet	2	-3968.558679	-3305.91468	-1272.256868	EMD2
CCTCAGTGTTCTCCAC-1	singlet	1	-328.4341126	-123.8951105	-243.4466578	EMD2
CCTCAGTTCAATGCAC-1	singlet	0	-580.1179952	-1655.388341	-1543.537442	EMD2
CCTCAGTTCATCGGGC-1	singlet	0	-717.5297517	-2075.863314	-1934.874464	EMD2
CCTCAGTTCCGTGGGT-1	singlet	1	-1350.679497	-544.245168	-1172.806727	EMD2
CCTCAGTTCTGGACCG-1	singlet	0	-158.0650353	-408.8543571	-378.4058531	EMD2
CCTCAGTTCTTCACAT-1	singlet	2	-1500.273708	-1177.637141	-555.9045952	EMD2
CCTCATGAGAGGTTAT-1	singlet	2	-215.3252464	-253.03384	-109.4765024	EMD2

CCTCATGAGTGGACGT-1	singlet	2	-4539.426229	-3869.35124	-1451.100294	EMD2
CCTCATGAGTGTTGAA-1	singlet	1	-384.2997944	-182.1972341	-346.2009859	EMD2
CCTCATGCAAACCGGA-1	singlet	1	-1349.023177	-428.6494777	-1051.278762	EMD2
CCTCATGCAACAAAGT-1	singlet	1	-1171.495835	-414.9489274	-866.098911	EMD2
CCTCATGGTGGACTGA-1	singlet	1	-2580.663153	-751.166723	-2028.084442	EMD2
CCTCATGGTTCCACGG-1	singlet	0	-99.85000388	-296.4306433	-235.3089516	EMD2
CCTCATGGTTTCCAAG-1	singlet	1	-186.4901798	-89.68911845	-151.0917203	EMD2
CCTCATGTCGGAGCAA-1	singlet	1	-2269.727142	-684.7147363	-1926.94784	EMD2
CCTCATGTCGGTGCAC-1	singlet	2	-7.247838979	-6.638832723	-2.705307843	EMD2
CCTCATGTCTACAGGT-1	singlet	1	-878.0306673	-311.7656459	-738.1427267	EMD2
CCTCATGTCTGCGTCT-1	doublet	1/0	-427.3557325	-279.0085632	-462.2373528	EMD2
CCTCCAAAGAACGTGC-1	singlet	0	-618.2796181	-2581.02175	-2089.075858	EMD2
CCTCCAAAGATGAATC-1	singlet	1	-458.4107891	-193.4591358	-409.4049601	EMD2
CCTCCAAAGATTGTGA-1	singlet	2	-2236.145689	-1793.490506	-766.3650854	EMD2
CCTCCAAAGCCTTGAT-1	singlet	1	-469.3542862	-149.3281354	-359.0233268	EMD2
CCTCCAAAGGCATCTT-1	singlet	1	-1060.043223	-450.7240536	-951.8061308	EMD2
CCTCCAAAGGCTGAAC-1	singlet	1	-150.6557313	-47.56324805	-81.60786931	EMD2
CCTCCAAAGTATGGCG-1	singlet	2	-2943.906073	-2413.486786	-938.3871156	EMD2
CCTCCAAAGTTCAACC-1	singlet	1	-610.3371759	-279.5838441	-501.4126472	EMD2
CCTCCAACAAGAGGCT-1	singlet	0	-818.2778521	-2615.171338	-2592.006219	EMD2
CCTCCAACACGGTGCT-1	singlet	1	-581.8305252	-265.6515267	-500.713046	EMD2
CCTCCAACACTTGTCC-1	singlet	2	-8.120816663	-8.338426827	-2.479182345	EMD2
CCTCCAACAGCGGTTC-1	singlet	2	-1134.367825	-971.934377	-325.009795	EMD2
CCTCCAACATCTTCGC-1	singlet	1	-425.6463188	-166.1386136	-431.4763015	EMD2
CCTCCAAGTCCTTAAG-1	singlet	2	-2514.669997	-2210.366824	-763.5234594	EMD2
CCTCCAAGTTAGCTAC-1	singlet	1	-2525.901018	-745.6751772	-1898.327218	EMD2
CCTCCAAGTTCGTACA-1	singlet	1	-2427.850834	-844.8612803	-2030.330327	EMD2
CCTCCAATCCGGTTCT-1	singlet	2	-434.410942	-419.7996772	-145.5413373	EMD2
CCTCCAATCTGTAAGC-1	singlet	1	-3464.82606	-956.7212568	-2462.915845	EMD2
CCTCCAATCTTCCCGA-1	singlet	2	-8101.918832	-5890.328509	-3172.348837	EMD2
CCTCCTCAGAGAGCAA-1	singlet	1	-458.4121556	-162.6664646	-393.8736453	EMD2
CCTCCTCAGGCGTTAG-1	singlet	0	-129.8052876	-349.4834312	-279.7184229	EMD2
CCTCCTCCAAGCGAGT-1	singlet	0	-183.5069486	-562.150236	-406.4750039	EMD2
CCTCCTCCAAGCGCTC-1	singlet	1	-2581.782443	-599.2249445	-1549.765838	EMD2
CCTCCTCCAGGAAGTC-1	singlet	1	-180.9728049	-58.42365166	-166.9450405	EMD2
CCTCCTCCATGTGTCA-1	singlet	1	-3155.778178	-1101.526624	-2420.711475	EMD2
CCTCCTCCATTGCCTC-1	singlet	1	-2922.902334	-999.9340936	-2444.777509	EMD2
CCTCCTCGTACCTAAC-1	singlet	1	-2231.258837	-753.1168041	-1853.582044	EMD2
CCTCCTCGTAGATCCT-1	singlet	1	-843.7914166	-288.1224424	-616.9917128	EMD2
CCTCCTCGTCATCAGT-1	doublet	45323	-6766.079897	-3844.064223	-3597.473128	EMD2
CCTCCTCGTCCAGAAG-1	singlet	1	-516.0625639	-210.9141679	-413.6904409	EMD2
CCTCCTCGTTAAGGGC-1	singlet	0	-1266.694222	-3913.63744	-3834.026818	EMD2
CCTCCTCGTTCAAACC-1	singlet	1	-970.5407631	-366.6266664	-849.5879778	EMD2
CCTCCTCTCATCGCTC-1	singlet	1	-1552.760466	-430.2522813	-1426.627165	EMD2
CCTCCTCTCATGCATG-1	singlet	1	-2087.91092	-771.588473	-1735.237084	EMD2
CCTCCTCTCATGGATC-1	singlet	2	-2266.995557	-1939.0192	-718.8240053	EMD2
CCTCCTCTCCATATGG-1	singlet	1	-4126.522518	-1254.907321	-3330.643737	EMD2
CCTCTAGAGCTTTGTG-1	singlet	1	-663.4225004	-252.7588495	-591.0233781	EMD2
CCTCTAGCAAGCTGTT-1	singlet	1	-1167.266459	-397.9312318	-857.7288697	EMD2
CCTCTAGTCCAAGCCG-1	singlet	0	-287.8199778	-908.2565734	-841.401739	EMD2
CCTCTAGTCCACACCT-1	singlet	1	-5172.294729	-1586.889976	-4513.393078	EMD2
CCTCTAGTCCGATGTA-1	unassigned	0	-0.774764677	-5.607634112	-1.326180947	EMD2
CCTCTAGTCCTTTAGT-1	singlet	0	-785.9997306	-2178.510594	-2171.976412	EMD2
CCTCTCCAGAATAACC-1	singlet	1	-655.4933491	-234.4478896	-535.1187937	EMD2
CCTCTCCAGAGGTATT-1	singlet	2	-1297.95548	-1149.820781	-452.8872908	EMD2
CCTCTCCAGCGCTTCG-1	singlet	2	-1221.378897	-1079.280536	-504.4197733	EMD2
CCTCTCCAGCTGACCC-1	singlet	2	-3404.062328	-2792.595298	-1201.9934	EMD2
CCTCTCCAGGACACTG-1	singlet	2	-3296.593051	-3193.414859	-1107.834004	EMD2
CCTCTCCAGGATGAGA-1	singlet	1	-1899.545545	-536.2966913	-1385.735455	EMD2
CCTCTCCAGGTTGACG-1	singlet	0	-32.73384538	-105.9882118	-63.52550614	EMD2
CCTCTCCAGTCATACC-1	singlet	1	-4123.251458	-1349.370647	-3300.014992	EMD2
CCTCTCCCACTACAGT-1	singlet	2	-5662.029946	-4405.694712	-1839.057955	EMD2
CCTCTCCCATCAGCGC-1	singlet	1	-2767.061956	-742.3287807	-2230.273642	EMD2
CCTCTCCGTGTCGCTG-1	singlet	1	-1762.273331	-546.2997148	-1382.793385	EMD2
CCTCTCCTCATGGAGG-1	singlet	2	-917.1941464	-793.6936175	-309.9535537	EMD2
CCTCTCCTCCGACAGC-1	singlet	1	-334.0705027	-125.893635	-329.4590017	EMD2
CCTCTCCTCGTTCTCG-1	singlet	2	-2103.327353	-1867.113541	-757.2518908	EMD2
CCTGCATAGTTACGAA-1	singlet	0	-529.4393891	-1290.267772	-1305.359293	EMD2
CCTGCATCAAGTGTCT-1	singlet	2	-795.3308816	-708.1949064	-317.9001441	EMD2
CCTGCATCACACCTTC-1	singlet	2	-1076.570694	-866.8148093	-350.3535332	EMD2
CCTGCATCATCATCTT-1	singlet	1	-3828.15549	-956.3832467	-2560.183328	EMD2
CCTGCATGTAGCTTTG-1	singlet	1	-2024.40786	-686.6498839	-1608.297831	EMD2
CCTGCATGTCACAGAG-1	singlet	2	-3047.311608	-2165.839361	-1182.377901	EMD2
CCTGCATGTCATGACT-1	singlet	1	-2642.502723	-715.0145626	-1919.704945	EMD2
CCTGCATGTGAATGTA-1	singlet	1	-2105.082142	-872.9930927	-1890.160346	EMD2
CCTGCATGTGGCATCC-1	singlet	1	-507.422697	-218.3091917	-474.5227707	EMD2
CCTGCATTCAGCAATC-1	singlet	2	-1797.727655	-1938.123903	-583.0436228	EMD2
CCTGCATTCGAGATGG-1	singlet	0	-463.8696501	-1286.443152	-1239.228091	EMD2
CCTGCATTCGAGTACT-1	singlet	0	-2004.024279	-6116.203081	-6113.505315	EMD2
CCTGCATTCGCCTAGG-1	singlet	2	-546.1022166	-463.7307494	-174.1585208	EMD2
CCTGCATTCTCACTCG-1	singlet	1	-1192.244675	-324.5953297	-806.475781	EMD2
CCTGCATTCTCTAGGA-1	singlet	1	-1024.993858	-319.5079575	-796.0854071	EMD2
CCTGTTGAGCGACCCT-1	singlet	2	-1126.623723	-958.832157	-449.5880575	EMD2
CCTGTTGAGGAAGAAC-1	singlet	1	-156.4622636	-27.60483291	-85.93099542	EMD2
CCTGTTGAGGGCCCTT-1	singlet	1	-1394.345704	-448.8989718	-1113.002639	EMD2
CCTGTTGCATTATGCG-1	singlet	1	-489.4888124	-194.7004013	-463.3347978	EMD2
CCTGTTGGTATTCTCT-1	singlet	0	-110.4225777	-293.1476809	-289.5578132	EMD2
CCTGTTGGTTAAGCAA-1	singlet	0	-1219.396481	-3545.085557	-3277.868747	EMD2
CCTGTTGGTTATAGCC-1	singlet	0	-49.72243914	-154.8018779	-152.4779465	EMD2
CCTGTTGTCTAACACG-1	singlet	0	-538.341915	-1899.051402	-1554.192744	EMD2
CCTTCAGAGCGAATGC-1	singlet	1	-262.2048978	-110.672099	-224.6630619	EMD2
CCTTCAGAGTAGCTCT-1	singlet	0	-170.454123	-417.8731866	-343.9407409	EMD2
CCTTCAGAGTTGGACG-1	singlet	0	-468.7119641	-1421.912382	-1308.913414	EMD2
CCTTCAGCAAGCACCC-1	singlet	1	-3163.155406	-1051.923978	-2664.077768	EMD2
CCTTCAGCAAGGTACG-1	singlet	2	-559.0187366	-506.2742454	-233.7343741	EMD2
CCTTCAGCAATAACCC-1	doublet	45323	-302.7687112	-177.5763454	-173.108113	EMD2
CCTTCAGCACCAGGTC-1	singlet	2	-4980.853907	-3621.586893	-1183.535828	EMD2
CCTTCAGCATATGGCT-1	singlet	1	-6.731457303	-1.886223004	-11.26677904	EMD2
CCTTCAGCATGGGATG-1	singlet	0	-130.478307	-435.527365	-435.1260487	EMD2
CCTTCAGCATGTCGTA-1	singlet	1	-1529.285385	-460.1682688	-1287.332915	EMD2
CCTTCAGGTCGACTGC-1	singlet	2	-1311.24458	-1149.835215	-460.664868	EMD2
CCTTCAGGTTACGGAG-1	singlet	2	-3892.683006	-3322.964623	-1293.68106	EMD2
CCTTCAGTCATCGGGC-1	singlet	1	-2186.078876	-725.200923	-1807.997901	EMD2
CCTTCAGTCGTCCATC-1	singlet	2	-6468.865519	-4950.6339	-1834.902825	EMD2
CCTTCAGTCTATACGG-1	singlet	1	-1472.525875	-496.0324578	-1218.546178	EMD2
CCTTCAGTCTGGCCTT-1	singlet	2	-17.34179299	-12.00421516	-5.694747332	EMD2
CCTTGTGAGAACTCCT-1	singlet	1	-2711.254567	-791.2407613	-2465.712676	EMD2
CCTTGTGAGATCACCT-1	singlet	2	-3856.871795	-2851.571559	-1028.33346	EMD2
CCTTGTGAGATGAACT-1	singlet	0	-400.2605348	-1384.896548	-1156.311245	EMD2
CCTTGTGAGGTCACCT-1	singlet	2	-231.1797992	-209.5656667	-57.94457252	EMD2

CCTTGTGAGGTTTGAA-1	singlet	1	-584.0157487	-190.7827579	-503.8305483	EMD2
CCTTGTGCAGAAGCTG-1	singlet	1	-318.5544811	-163.0061348	-319.7427723	EMD2
CCTTGTGGTCGTACAT-1	singlet	1	-3388.27406	-803.2942856	-2578.946159	EMD2
CCTTGTGGTCTTCCGT-1	singlet	0	-1499.040856	-3596.198361	-3485.12446	EMD2
CCTTGTGGTGGACTAG-1	singlet	2	-296.9016713	-264.6636266	-150.5566445	EMD2
CCTTGTGTCACTTATC-1	singlet	0	-1965.276953	-6374.528074	-6092.883282	EMD2
CCTTGTGTCAGGAAGC-1	singlet	0	-106.2515059	-317.5125231	-342.278059	EMD2
CCTTTGGAGAAGTATC-1	singlet	0	-1089.359265	-2910.375696	-2990.126537	EMD2
CCTTTGGAGGACAACC-1	singlet	1	-475.9699661	-163.2652669	-347.3191196	EMD2
CCTTTGGCAGCACAGA-1	singlet	1	-286.4850977	-112.9681452	-232.1400813	EMD2
CCTTTGGGTCTGTCAA-1	singlet	1	-1738.543709	-574.8300578	-1331.757258	EMD2
CCTTTGGGTGCAGGAT-1	singlet	1	-221.5500382	-97.79235612	-200.8806841	EMD2
CCTTTGGGTTACACTG-1	singlet	0	-513.7567872	-1608.825458	-1293.733716	EMD2
CCTTTGGGTTCATCTT-1	singlet	1	-1327.500188	-517.1432945	-1140.618094	EMD2
CCTTTGGTCACCGCTT-1	singlet	1	-400.2129018	-186.233617	-362.537407	EMD2
CCTTTGGTCATGGATC-1	singlet	2	-2329.346563	-1935.077181	-698.8359261	EMD2
CCTTTGGTCCAGCAAT-1	singlet	1	-216.6659766	-69.06863895	-152.6469664	EMD2
CCTTTGGTCCGTATAG-1	singlet	0	-370.3212952	-838.2803377	-784.1676569	EMD2
CGAAGGAAGCGAGTCA-1	singlet	1	-356.198859	-121.3535074	-324.4187015	EMD2
CGAAGGACACCCTGAG-1	singlet	2	-2100.563586	-2118.378154	-568.9351445	EMD2
CGAAGGACATGACTTG-1	singlet	1	-1717.139167	-534.1206905	-1488.634295	EMD2
CGAAGGAGTACTCCCT-1	doublet	0/2	-1373.601916	-2269.570559	-1679.025052	EMD2
CGAAGGAGTACTCGCG-1	singlet	1	-422.6509434	-163.2071706	-352.7718722	EMD2
CGAAGGAGTCTAGATC-1	singlet	1	-989.5876108	-367.4882129	-824.7871466	EMD2
CGAAGGAGTGCAACGA-1	singlet	2	-2218.451495	-1927.280686	-709.3336986	EMD2
CGAAGGAGTGTATACC-1	singlet	2	-5099.107048	-3913.259243	-1350.481815	EMD2
CGAAGGAGTTCTCTAT-1	singlet	0	-781.7594151	-2355.894566	-2142.758047	EMD2
CGAAGGAGTTGTGCCG-1	singlet	0	-1525.999544	-5504.03713	-5232.866914	EMD2
CGAAGGATCAAGCGTT-1	singlet	0	-191.185023	-349.9377753	-392.0022652	EMD2
CGAAGGATCATGGAGG-1	singlet	1	-329.2106798	-169.9409787	-295.7852589	EMD2
CGAAGGATCCAACCAA-1	singlet	2	-1580.143401	-1290.337426	-558.2776538	EMD2
CGAAGGATCGGTGCAC-1	singlet	2	-2372.701046	-1835.733246	-998.5350846	EMD2
CGAAGTTAGAACCCGA-1	singlet	0	-1661.631443	-4975.870957	-4862.036893	EMD2
CGAAGTTAGATTACCC-1	singlet	1	-2365.585435	-841.3884437	-1968.056774	EMD2
CGAAGTTAGCGCATCC-1	singlet	1	-1960.774573	-666.7673702	-1577.190386	EMD2
CGAAGTTAGGTAAAGG-1	singlet	1	-3192.992854	-911.3979673	-2934.266785	EMD2
CGAAGTTCATAATGCC-1	singlet	1	-455.0524698	-183.8226323	-426.7579283	EMD2
CGAAGTTGTCAATCTG-1	singlet	0	-691.3024274	-2238.830548	-2195.754603	EMD2
CGAAGTTGTGCCTGAC-1	singlet	2	-4574.383912	-3765.921056	-1344.598305	EMD2
CGAAGTTTCCTAGAGT-1	singlet	2	-140.4776564	-105.1832339	-47.27395837	EMD2
CGAAGTTTCGATAACC-1	unassigned	0	-3.623775985	-3.678550653	-11.01073284	EMD2
CGAAGTTTCGGAATTC-1	singlet	1	-339.3014554	-126.1753585	-335.9376118	EMD2
CGAAGTTTCTCGCTCA-1	singlet	1	-389.2992856	-185.3046083	-326.1583108	EMD2
CGAATTGAGAGCATTA-1	singlet	1	-421.5047199	-170.4937591	-351.5336204	EMD2
CGAATTGAGCGGCTCT-1	singlet	0	-1146.842017	-3781.881496	-3404.597521	EMD2
CGAATTGCAACTCGAT-1	singlet	0	-773.0794936	-2249.941402	-2103.3665	EMD2
CGAATTGCACGCGTGT-1	singlet	2	-817.0398513	-697.5923729	-284.0923501	EMD2
CGAATTGCAGAGGGTT-1	singlet	2	-1253.806581	-1277.819142	-479.0743408	EMD2
CGAATTGCAGCAGATG-1	singlet	1	-188.6416729	-82.24151722	-183.5606853	EMD2
CGAATTGGTACGCTAT-1	singlet	2	-1714.560239	-1605.62486	-520.5855873	EMD2
CGAATTGGTCAGACTT-1	singlet	0	-123.1222162	-270.7559289	-277.6602975	EMD2
CGAATTGGTGATGTAA-1	singlet	0	-425.4879832	-1197.473884	-1245.374221	EMD2
CGAATTGGTGGCACTC-1	singlet	1	-210.7991798	-92.12168726	-198.6469296	EMD2
CGAATTGTCCGTATAG-1	singlet	2	-499.0775427	-396.7176796	-119.3129921	EMD2
CGAATTGTCGAAGCCC-1	singlet	2	-2003.513742	-1633.533217	-714.7157638	EMD2
CGACAGCAGAGTGGCT-1	singlet	2	-1609.419411	-1397.824205	-547.0086328	EMD2
CGACAGCAGATAGTCA-1	singlet	1	-1147.172169	-357.8248436	-918.9887838	EMD2
CGACAGCCAGCTGTAT-1	singlet	0	-843.3434798	-3201.215436	-2911.166952	EMD2
CGACAGCGTACGAGTG-1	singlet	1	-2130.331667	-620.1229169	-1736.144289	EMD2
CGACAGCGTAGGTACG-1	singlet	1	-664.1380107	-261.6189745	-569.4358772	EMD2
CGACAGCGTCTCCCTA-1	singlet	1	-1281.865194	-348.6916644	-1232.731068	EMD2
CGACAGCTCAACGAGG-1	singlet	0	-1140.1032	-3692.881573	-3308.636108	EMD2
CGACAGCTCATCCCGT-1	singlet	1	-221.3119905	-91.17473493	-214.9478634	EMD2
CGACAGCTCCTAACAG-1	singlet	2	-123.6995926	-70.83389834	-29.82119341	EMD2
CGACAGCTCTGGACTA-1	singlet	1	-3582.128193	-1093.76211	-3214.043586	EMD2
CGACAGCTCTTGGAAC-1	singlet	1	-4897.729089	-1468.527003	-3922.935872	EMD2
CGAGAAGAGACAACAT-1	singlet	1	-2360.379485	-655.7677937	-1913.498234	EMD2
CGAGAAGAGAGACAAG-1	singlet	1	-345.4361333	-152.2413006	-371.3505325	EMD2
CGAGAAGAGATGTTAG-1	singlet	1	-1058.289394	-388.150171	-941.1547933	EMD2
CGAGAAGAGCCTATTG-1	singlet	2	-432.2932032	-401.1682745	-168.3191513	EMD2
CGAGAAGAGTAGCATA-1	singlet	1	-1187.487048	-283.7461519	-999.2221354	EMD2
CGAGAAGCAGTAGATA-1	singlet	1	-557.947933	-199.5590878	-446.2880205	EMD2
CGAGAAGGTCGAGCAA-1	singlet	1	-831.1426834	-263.705115	-753.2755482	EMD2
CGAGAAGGTGATTGGG-1	singlet	2	-6483.794009	-4987.310488	-1462.88157	EMD2
CGAGAAGGTGTCATGT-1	singlet	1	-539.5796706	-173.938183	-369.2404866	EMD2
CGAGAAGGTTGCACGC-1	singlet	2	-1241.339254	-1030.416961	-400.5719626	EMD2
CGAGAAGGTTGTGCCG-1	singlet	1	-707.6819562	-252.8865492	-559.9338294	EMD2
CGAGAAGGTTTGATCG-1	singlet	2	-677.1427703	-592.3819187	-261.9632864	EMD2
CGAGAAGTCATGAAAG-1	singlet	1	-1205.517393	-401.8832035	-977.9406819	EMD2
CGAGAAGTCCCAAGCG-1	singlet	0	-562.2280058	-1228.786156	-1212.774439	EMD2
CGAGAAGTCTACTGCC-1	singlet	1	-662.0555024	-273.8284568	-576.3158636	EMD2
CGAGGAAAGAATCTAG-1	singlet	1	-4841.611626	-1536.645253	-4091.312753	EMD2
CGAGGAAAGCTTAAGA-1	singlet	1	-1951.984711	-588.1162223	-1746.841271	EMD2
CGAGGAAAGTGGTCAG-1	singlet	1	-549.3961347	-151.5396469	-437.0108819	EMD2
CGAGGAACAATACGCT-1	singlet	0	-479.8638531	-1216.006114	-1071.702216	EMD2
CGAGGAACAGCACCCA-1	singlet	1	-2140.657128	-653.5417648	-1757.355631	EMD2
CGAGGAATCCACAGCG-1	singlet	1	-267.9406327	-108.512325	-276.4789178	EMD2
CGAGGAATCCCATGGG-1	singlet	0	-1733.690226	-6192.393394	-5895.285748	EMD2
CGAGGAATCGGTTCAA-1	singlet	1	-3803.215732	-1087.506217	-2848.325083	EMD2
CGAGGCTAGACCTTTG-1	singlet	1	-777.8729415	-246.5803443	-580.6387309	EMD2
CGAGGCTAGTCTGTAC-1	singlet	1	-1752.541032	-472.3101753	-1370.507618	EMD2
CGAGGCTAGTGAGGTC-1	singlet	1	-310.1069582	-91.35552769	-264.7434993	EMD2
CGAGGCTAGTGCCTCG-1	singlet	0	-522.1416632	-1869.168277	-1573.861319	EMD2
CGAGGCTAGTTACGAA-1	singlet	1	-1519.093049	-482.3437189	-1245.28225	EMD2
CGAGGCTCACCGTGAC-1	singlet	1	-2169.245438	-713.6697333	-1600.530702	EMD2
CGAGGCTCATCCCGTT-1	singlet	1	-949.5078058	-338.1571593	-937.230573	EMD2
CGAGGCTTCAGCGCGT-1	singlet	1	-1875.810436	-541.8336584	-1485.493787	EMD2
CGAGGCTTCGATCCCT-1	singlet	1	-657.7385011	-227.4205868	-549.8559945	EMD2
CGAGGCTTCGTCAAAC-1	singlet	1	-3106.750822	-990.2513946	-2241.857773	EMD2
CGAGTGCAGAGCCCAA-1	singlet	0	-1349.334603	-4507.623493	-4295.572366	EMD2
CGAGTGCAGCTGAGCA-1	singlet	0	-48.05985064	-86.46090151	-87.86903571	EMD2
CGAGTGCAGGATCACG-1	singlet	1	-1659.820083	-495.1117143	-1458.099579	EMD2
CGAGTGCCATAATCCG-1	singlet	1	-171.478536	-83.74915396	-146.9972599	EMD2
CGAGTGCGTCGAGATG-1	singlet	0	-333.5918994	-921.9310522	-892.2923747	EMD2
CGAGTGCTCGAGTTGT-1	singlet	1	-223.9232853	-88.96628844	-191.6334854	EMD2
CGAGTGCTCGCTAATG-1	singlet	1	-11.30052034	-5.095494315	-10.19586255	EMD2
CGAGTGCTCGTTAGTG-1	singlet	2	-2251.79285	-2204.219885	-591.7976279	EMD2
CGAGTGCTCTACCAGA-1	singlet	1	-1630.267972	-490.3346056	-1543.721168	EMD2
CGAGTGCTCTCGTCGT-1	singlet	2	-310.6853868	-325.8382435	-152.3677087	EMD2
CGAGTTAAGACCATGG-1	singlet	1	-347.5635313	-109.4753022	-287.0588962	EMD2

CGAGTTAAGTCACACT-1	singlet	1	-1019.743678	-286.385476	-743.9824596	EMD2
CGAGTTACAAGCTCTA-1	singlet	1	-1018.772318	-316.2006427	-741.5714942	EMD2
CGAGTTACAATCACGT-1	singlet	0	-873.0852536	-2252.686258	-2226.218873	EMD2
CGAGTTACACCGCTAG-1	singlet	2	-1059.825009	-846.6033716	-393.7006229	EMD2
CGAGTTACAGTAGAAT-1	singlet	1	-180.9710077	-59.57351677	-132.2618368	EMD2
CGAGTTAGTAAGATTG-1	singlet	1	-2100.472695	-692.1126031	-1600.249207	EMD2
CGAGTTAGTATCCCAA-1	singlet	0	-303.7116535	-1088.963489	-800.4310985	EMD2
CGAGTTAGTCCACTCT-1	singlet	0	-540.8645162	-1619.768416	-1688.143006	EMD2
CGAGTTAGTCGTAATC-1	singlet	2	-488.2366243	-427.3130194	-147.6337418	EMD2
CGAGTTAGTCGTCGGT-1	singlet	0	-220.3954495	-765.9973601	-729.545127	EMD2
CGAGTTAGTTTCCATT-1	singlet	0	-401.0679612	-1159.860777	-1032.145969	EMD2
CGAGTTATCCCGATCT-1	singlet	1	-820.3704487	-292.2204984	-682.5038991	EMD2
CGAGTTATCGCTGTTC-1	singlet	1	-1018.812764	-389.6380749	-847.4389401	EMD2
CGAGTTATCTCAACCC-1	singlet	1	-1758.271761	-636.9335545	-1539.39079	EMD2
CGATCGGCACAATGAA-1	singlet	1	-1699.530842	-592.3462628	-1371.583596	EMD2
CGATCGGCAGAACTAA-1	singlet	2	-367.7096159	-344.9797001	-143.205268	EMD2
CGATCGGGTATCTTCT-1	singlet	2	-1916.778761	-1627.800666	-691.3596772	EMD2
CGATCGGGTCTAATCG-1	singlet	1	-692.0544786	-269.197747	-690.8458914	EMD2
CGATCGGGTCTTCTAT-1	singlet	1	-250.7202627	-110.7518474	-215.7570849	EMD2
CGATCGGGTGTGTACT-1	singlet	2	-2126.012897	-1761.053792	-681.1295846	EMD2
CGATCGGGTTCGAACT-1	singlet	0	-406.0073495	-1242.214058	-1231.361535	EMD2
CGATCGGTCTGAGAGG-1	singlet	2	-3069.593242	-2550.836055	-901.6894007	EMD2
CGATGCGAGTCTTCCC-1	singlet	1	-10.72344743	-4.325537462	-11.1794213	EMD2
CGATGCGCAACTCGTA-1	singlet	1	-164.3930282	-44.34113901	-111.8631856	EMD2
CGATGCGCAAGAGATT-1	singlet	0	-300.1155267	-1015.784335	-797.7102639	EMD2
CGATGCGCAAGCACAG-1	singlet	1	-371.3809979	-164.610502	-344.9018542	EMD2
CGATGCGCACAAATAG-1	singlet	2	-2337.318991	-1745.778174	-750.5572807	EMD2
CGATGCGGTAGTGGCA-1	singlet	0	-814.1395063	-3306.535613	-3413.639561	EMD2
CGATGCGGTGACATCT-1	singlet	0	-767.2892807	-2023.995532	-2055.708996	EMD2
CGATGCGTCGCAAGAG-1	unassigned	45293	-61.60006194	-34.94091504	-41.74069042	EMD2
CGATGCGTCGCTCATC-1	singlet	2	-335.9915681	-306.8675458	-140.6332555	EMD2
CGATGCGTCGCTTTAT-1	singlet	1	-1075.427833	-346.7899608	-998.8618291	EMD2
CGATGGCAGCACGATG-1	singlet	2	-2757.396973	-2227.404231	-876.6236408	EMD2
CGATGGCAGCATTGAA-1	singlet	2	-550.8508425	-490.4249486	-216.1059618	EMD2
CGATGGCAGCTCACTA-1	singlet	0	-1352.57425	-4173.64644	-3806.648601	EMD2
CGATGGCAGGCGATAC-1	singlet	2	-202.1861455	-186.3853768	-98.03193771	EMD2
CGATGGCAGTACGTCT-1	singlet	0	-604.0494543	-1690.684957	-1624.952473	EMD2
CGATGGCAGTTTGAGA-1	singlet	2	-1613.586922	-1702.118937	-814.7794985	EMD2
CGATGGCTCAAGTGTC-1	singlet	1	-341.5454993	-158.4157512	-269.8125514	EMD2
CGATGGCTCATAAGGA-1	singlet	1	-1715.464814	-615.6484743	-1404.678431	EMD2
CGATGGCTCATAGACC-1	singlet	0	-125.184735	-436.9612275	-379.7891594	EMD2
CGATGGCTCGATTCCC-1	singlet	2	-9177.594207	-6778.096774	-2176.174048	EMD2
CGATGGCTCTACTATC-1	singlet	1	-356.7417117	-131.1691315	-292.5745208	EMD2
CGATGGCTCTCCTGTG-1	singlet	1	-1022.84468	-302.6906489	-947.9672569	EMD2
CGCAGGTCAAATGAAC-1	singlet	1	-3248.282482	-1016.996819	-2504.893215	EMD2
CGCAGGTCAACGATCT-1	singlet	0	-697.3527699	-1774.978313	-1714.419681	EMD2
CGCAGGTCACCCATAA-1	singlet	1	-291.894622	-128.1322729	-276.0837561	EMD2
CGCAGGTCATCAGCAT-1	unassigned	45293	-3.86776379	-1.916285648	-2.315943922	EMD2
CGCAGGTGTACGATCT-1	singlet	0	-736.3270641	-2514.077493	-2415.360497	EMD2
CGCAGGTGTAGGGTAC-1	singlet	2	-199.9817671	-155.52392	-70.4550081	EMD2
CGCAGGTGTTGTGTTG-1	singlet	0	-38.49063921	-120.5311351	-101.1366385	EMD2
CGCAGGTTCATGCGGC-1	singlet	2	-2714.53883	-2011.706378	-892.4783451	EMD2
CGCAGGTTCCTGGTCT-1	singlet	2	-2355.313865	-2101.236325	-694.9094459	EMD2
CGCAGGTTCTCCAAGA-1	doublet	1/0	-1356.998934	-942.1741936	-1371.177956	EMD2
CGCAGGTTCTCCACTG-1	singlet	2	-2606.814757	-2245.302605	-1004.620726	EMD2
CGCATAAAGCGGTATG-1	singlet	1	-1011.783723	-354.5901543	-876.8635168	EMD2
CGCATAAAGCTCGGCT-1	singlet	1	-1195.490698	-351.5063046	-902.5729319	EMD2
CGCATAACACGTAACT-1	singlet	1	-1861.999265	-620.3329757	-1617.409891	EMD2
CGCATAACACGTAGTT-1	singlet	2	-1239.019033	-891.4962779	-281.2089178	EMD2
CGCATAACAGAACATA-1	singlet	1	-398.0205562	-138.1303602	-313.4889151	EMD2
CGCATAACATAGAGGC-1	singlet	1	-300.4987562	-148.6448042	-260.4714447	EMD2
CGCATAACATGTTCAG-1	singlet	1	-638.9121969	-250.7136224	-523.8611123	EMD2
CGCATAAGTTGCTTGA-1	singlet	0	-446.3651015	-1498.620724	-1308.376403	EMD2
CGCATAATCCAAATGC-1	singlet	1	-731.3357999	-305.1359951	-572.5169665	EMD2
CGCATAATCCGGACTG-1	singlet	1	-1296.813191	-448.7450329	-1127.352786	EMD2
CGCATGGAGAGCATAT-1	singlet	1	-1197.851809	-418.0071456	-958.1391435	EMD2
CGCATGGCAACTGATC-1	singlet	1	-2846.658064	-688.7174751	-2027.547182	EMD2
CGCATGGCAAGACGGT-1	singlet	2	-2519.35098	-2155.261751	-891.0294133	EMD2
CGCATGGCACTCTCGT-1	unassigned	1/0	-20.7799869	-12.07093058	-15.91564156	EMD2
CGCATGGCAGAGCCCT-1	singlet	1	-1550.539108	-423.4107994	-1367.779469	EMD2
CGCATGGCATAGAAAC-1	singlet	2	-927.8566122	-984.9453625	-338.959135	EMD2
CGCATGGCATCCCGTT-1	singlet	1	-13.58598169	-5.053394893	-11.65428879	EMD2
CGCATGGGTACTGCCG-1	singlet	1	-870.8892522	-384.6056896	-708.9915271	EMD2
CGCATGGGTATCAGGG-1	singlet	1	-1912.572947	-593.563694	-1743.353103	EMD2
CGCATGGGTCGCATGC-1	doublet	1/0	-1455.45572	-1282.97732	-1754.437348	EMD2
CGCATGGGTCTCAGGC-1	singlet	1	-329.4934998	-112.3002717	-250.8395737	EMD2
CGCATGGTCATCACCC-1	singlet	2	-439.3772676	-381.2883885	-183.5815531	EMD2
CGCATGGTCGCATTAG-1	singlet	2	-2300.54993	-1922.908236	-715.2604476	EMD2
CGCCAGAAGACAGCTG-1	singlet	1	-867.0325693	-346.4678027	-743.1509481	EMD2
CGCCAGACAACCACGC-1	singlet	1	-3259.166286	-1040.901634	-2767.18476	EMD2
CGCCAGACAATAACCC-1	singlet	1	-375.8350368	-134.6517031	-313.925804	EMD2
CGCCAGACACCTGCTT-1	singlet	0	-114.1475772	-280.0516492	-299.4839228	EMD2
CGCCAGACAGACGCTC-1	unassigned	1/0	-68.7825614	-38.87241928	-63.71303047	EMD2
CGCCAGAGTCAGTCCG-1	singlet	1	-508.768613	-145.9905489	-333.7856782	EMD2
CGCCAGAGTCTCCCTA-1	singlet	1	-1655.953322	-518.943109	-1500.842644	EMD2
CGCCAGAGTGCCTATA-1	singlet	2	-1161.182956	-1210.669978	-367.6061596	EMD2
CGCCAGATCTATACTC-1	singlet	0	-180.0635173	-612.0201206	-496.7133126	EMD2
CGCCATTAGAGCTTTC-1	unassigned	2/0	-43.79091865	-35.60518928	-30.53086855	EMD2
CGCCATTAGGAAGAAC-1	singlet	1	-370.2703524	-114.2223557	-380.8909681	EMD2
CGCCATTAGTTCACTG-1	singlet	1	-2581.986927	-844.4176853	-1999.55945	EMD2
CGCCATTGTAATGATG-1	singlet	2	-993.8393022	-827.2428469	-360.0982491	EMD2
CGCCATTGTGCATGAG-1	unassigned	1/0	-331.3605694	-173.9822436	-334.1072167	EMD2
CGCCATTGTTGCTTGA-1	singlet	1	-438.8919493	-65.55475781	-231.3247231	EMD2
CGCCATTTCACTCACC-1	singlet	1	-25.65657104	-13.39233529	-17.94596345	EMD2
CGCCATTTCCGGACTG-1	singlet	1	-2007.047516	-640.9963419	-1529.147756	EMD2
CGCCATTTCTCTGAGA-1	singlet	1	-871.5164636	-278.3893179	-750.0435589	EMD2
CGCGTGAAGGTGAGAA-1	singlet	1	-820.0612627	-248.9400458	-719.4170692	EMD2
CGCGTGACAATCTAGC-1	singlet	1	-1779.629373	-529.8012222	-1414.085329	EMD2
CGCGTGACACATATGC-1	singlet	1	-719.8447338	-247.1576112	-567.5180842	EMD2
CGCGTGACACGAGAAC-1	singlet	1	-870.9147271	-285.4658815	-656.1111765	EMD2
CGCGTGACAGGACGAT-1	singlet	2	-1582.343598	-1409.154259	-483.3032961	EMD2
CGCGTGAGTACTCCGG-1	singlet	1	-4400.729168	-1159.387471	-3051.458021	EMD2
CGCGTGATCGACGATT-1	doublet	0/2	-541.7677761	-1197.516828	-870.0831779	EMD2
CGCGTGATCGGACCAC-1	singlet	1	-1290.320796	-390.8806575	-1017.086999	EMD2
CGCGTGATCTTAGCAG-1	singlet	2	-1732.644173	-1537.049658	-614.5779933	EMD2
CGGAACCAGGGACTGT-1	singlet	1	-2718.402357	-890.5894569	-2159.219595	EMD2
CGGAACCCAACTCGAT-1	singlet	1	-1521.615107	-488.9983125	-1221.167222	EMD2
CGGAACCCAACTGCTA-1	singlet	1	-2375.810458	-621.3942988	-1783.601031	EMD2
CGGAACCCACCAGCGT-1	singlet	2	-185.0334958	-181.9341699	-81.97111476	EMD2
CGGAACCGTCAACGCC-1	singlet	2	-1232.421414	-1242.660845	-379.485395	EMD2

CGGAACCGTGGCTTGC-1	singlet	1	-305.7118446	-136.3576992	-254.7893053	EMD2
CGGAACCGTTGAATCC-1	singlet	1	-301.0029527	-143.1915297	-264.3262723	EMD2
CGGAACCTCAGACCGC-1	singlet	1	-1185.968555	-352.2020593	-972.655676	EMD2
CGGAACCTCCCTAGGG-1	singlet	0	-592.9233376	-1838.303687	-1786.824438	EMD2
CGGAACCTCGCTGACG-1	singlet	1	-484.6186878	-190.5825693	-386.4299315	EMD2
CGGAACCTCGTCGACG-1	singlet	0	-100.1702867	-239.3319241	-269.1354332	EMD2
CGGAACCTCTGAGCAT-1	singlet	0	-138.9807577	-469.7502809	-337.6524691	EMD2
CGGAACCTCTGTAAGC-1	unassigned	1/0	-2.759865086	-2.540185218	-6.219948435	EMD2
CGGAATTAGAAATTGC-1	doublet	0/1	-2575.681494	-3161.71761	-3648.804004	EMD2
CGGAATTAGATGACCG-1	singlet	1	-2179.011947	-662.1869205	-1930.501641	EMD2
CGGAATTAGGTCGTAG-1	singlet	1	-323.1683429	-135.4162823	-279.0003836	EMD2
CGGAATTAGTGTTCAC-1	singlet	0	-59.89384323	-169.9215808	-168.4252612	EMD2
CGGAATTCACTGGACC-1	singlet	0	-168.6389616	-464.1040052	-392.208936	EMD2
CGGAATTCAGCGGATA-1	singlet	2	-478.3399279	-353.9032149	-156.3373884	EMD2
CGGAATTCATAACGGG-1	singlet	2	-906.0004975	-789.0392375	-346.7753634	EMD2
CGGAATTGTAACGTTC-1	singlet	2	-4808.421786	-3738.031698	-1214.242144	EMD2
CGGAATTGTACCTTCC-1	singlet	1	-3558.315941	-1255.675591	-2734.689579	EMD2
CGGAATTGTAGTGTGG-1	singlet	0	-67.52365627	-177.6747549	-149.2309537	EMD2
CGGAATTGTGACGTCC-1	singlet	1	-294.8546023	-106.0469881	-231.0403214	EMD2
CGGAATTTCAACACCA-1	singlet	1	-338.5065555	-125.9603791	-303.2592756	EMD2
CGGAATTTCCGCTAGG-1	singlet	1	-509.7718334	-196.3172998	-421.5956003	EMD2
CGGAATTTCGCTTGCT-1	singlet	1	-378.0923693	-146.8091759	-315.4695618	EMD2
CGGAATTTCGTAGTCA-1	singlet	1	-335.5114778	-147.5923766	-295.9376669	EMD2
CGGACACAGGCTTCCG-1	unassigned	2/0	-31.24214565	-36.73121505	-27.14009717	EMD2
CGGACACAGGTGCTAG-1	singlet	2	-1785.988683	-1839.205659	-518.1863573	EMD2
CGGACACAGTTTGAGA-1	singlet	1	-290.3044489	-128.5419316	-255.4381776	EMD2
CGGACACCACTTGGCG-1	unassigned	2	-2.952377082	-3.220225838	-1.929351541	EMD2
CGGACACCAGGCGAAT-1	singlet	1	-309.2691203	-132.1788648	-274.4668547	EMD2
CGGACACCATCGGAGA-1	singlet	1	-2828.669245	-927.9776263	-2271.538116	EMD2
CGGACACGTAGGAAAG-1	singlet	2	-103.9200478	-64.22696069	-30.1223669	EMD2
CGGACACGTTAGGACG-1	singlet	2	-1136.17978	-1152.67756	-396.5272539	EMD2
CGGACACGTTCAAAGA-1	singlet	0	-376.6887995	-1165.263057	-1095.732038	EMD2
CGGACACTCAGCGCAC-1	singlet	0	-410.0763747	-1382.71513	-1099.749528	EMD2
CGGACACTCCCTTTGG-1	singlet	1	-359.81746	-140.9562703	-294.4113536	EMD2
CGGACACTCCGATGCG-1	singlet	0	-212.5992346	-635.4161487	-469.3302064	EMD2
CGGACACTCCTCACGT-1	singlet	0	-455.7521461	-1283.277339	-1088.567598	EMD2
CGGACACTCTGACAGT-1	singlet	2	-2214.070346	-1681.97289	-712.8416987	EMD2
CGGAGAACAACTAGAA-1	singlet	1	-1952.200455	-591.43262	-1443.063744	EMD2
CGGAGAACACCTGCAG-1	singlet	1	-333.6131228	-116.0859993	-255.9198102	EMD2
CGGAGAAGTACGTTCA-1	singlet	2	-491.4327853	-505.3125737	-190.3920087	EMD2
CGGAGAAGTATTCTCT-1	singlet	1	-4229.017276	-1119.844702	-3101.479072	EMD2
CGGAGAATCCGCAACG-1	singlet	2	-60.62269192	-36.19805847	-21.70375944	EMD2
CGGAGAATCTTAATCC-1	singlet	2	-4886.493477	-4448.151799	-1947.849563	EMD2
CGGCAGTAGAGATGCC-1	singlet	1	-2272.026252	-673.237256	-2035.507783	EMD2
CGGCAGTCAAGCCATT-1	singlet	1	-929.6032778	-301.7909767	-729.515284	EMD2
CGGCAGTCAATAGTGA-1	singlet	2	-675.5798543	-547.2663366	-250.0727187	EMD2
CGGCAGTCAGCCTATA-1	singlet	0	-620.762823	-2002.343092	-1859.051715	EMD2
CGGCAGTGTCATGCAT-1	singlet	2	-535.062296	-558.6159081	-182.1658324	EMD2
CGGCAGTGTCCAGCAC-1	singlet	2	-330.721585	-303.5120776	-135.1754603	EMD2
CGGCAGTGTCGATTTG-1	singlet	1	-1823.781077	-484.6870591	-1505.824602	EMD2
CGGCAGTGTGTGTCGC-1	singlet	1	-1020.597447	-374.534395	-850.6249474	EMD2
CGGCAGTTCGCATGAT-1	singlet	2	-209.364712	-208.6415679	-90.65948184	EMD2
CGGCAGTTCTGGGTCG-1	singlet	1	-1526.351849	-454.904333	-1237.044673	EMD2
CGGGACTAGCGTTCCG-1	singlet	1	-1327.086955	-379.9905634	-1060.707622	EMD2
CGGGACTAGTCTTCCC-1	singlet	2	-3033.775979	-2658.52976	-1109.892216	EMD2
CGGGACTCATCGCCTT-1	singlet	1	-343.9628376	-138.9641195	-304.9063878	EMD2
CGGGACTGTTCGTAAC-1	singlet	2	-2727.546996	-2139.638059	-871.0740151	EMD2
CGGGACTTCCGAGATT-1	doublet	0/1	-2434.491029	-3737.444297	-4226.890974	EMD2
CGGGACTTCGAAGAAT-1	singlet	1	-1335.305871	-400.466796	-1158.634107	EMD2
CGGGACTTCTCTCGAC-1	singlet	1	-581.6956317	-126.8481327	-334.0157379	EMD2
CGGGCATAGGCATCTT-1	singlet	1	-778.4779322	-262.3742563	-577.1837228	EMD2
CGGGCATAGTCCCAAT-1	singlet	1	-736.5687075	-221.793815	-660.4348243	EMD2
CGGGCATAGTGCTCGC-1	singlet	0	-161.8178348	-544.9674491	-536.3646404	EMD2
CGGGCATCAACGAGGT-1	singlet	1	-425.1449674	-168.2723411	-331.0225259	EMD2
CGGGCATCAACTCGTA-1	singlet	0	-229.9890733	-647.0936257	-625.3379774	EMD2
CGGGCATCACTGATTG-1	singlet	2	-3800.204578	-3207.518937	-1374.374608	EMD2
CGGGCATCATGACTCA-1	singlet	1	-420.8589509	-173.4284507	-346.7335078	EMD2
CGGGCATCATTGACCA-1	singlet	0	-295.3822289	-951.4833773	-877.0385775	EMD2
CGGGCATGTACTAGCT-1	singlet	2	-2002.352436	-1584.509428	-603.2150138	EMD2
CGGGCATGTAGAAACT-1	singlet	1	-740.7063502	-243.3532922	-564.5716397	EMD2
CGGGCATGTAGGAGTC-1	singlet	1	-960.2621491	-320.5563428	-733.3834938	EMD2
CGGGCATGTGACCGAA-1	singlet	2	-3947.317304	-3734.239145	-1247.151661	EMD2
CGGGCATTCGCGGTAC-1	singlet	1	-3261.088865	-1230.647282	-2378.607518	EMD2
CGGGTCACAATGTGGG-1	singlet	0	-669.8518112	-2032.646649	-1918.918408	EMD2
CGGGTCACAGATTTCG-1	singlet	1	-162.4001767	-72.60702524	-116.3119293	EMD2
CGGGTCACATCGTTCC-1	singlet	1	-479.8713986	-186.4781473	-426.1120167	EMD2
CGGGTCACATTCACCC-1	singlet	0	-185.4396513	-509.6516476	-396.9462471	EMD2
CGGGTCACATTGGCAT-1	doublet	45323	-4813.702075	-2752.468352	-2669.226313	EMD2
CGGGTCAGTACGCTTA-1	doublet	0/1	-1158.077041	-1884.231693	-2093.768436	EMD2
CGGGTCATCACCCTCA-1	singlet	1	-1244.471787	-441.9308855	-1055.330291	EMD2
CGGGTCATCGACACCG-1	singlet	1	-834.7766847	-209.9393476	-550.8195124	EMD2
CGGGTCATCGCCTATC-1	singlet	0	-640.9323444	-1791.132983	-1842.824112	EMD2
CGGGTGTAGACCATTC-1	singlet	2	-534.0984328	-560.6195031	-245.940779	EMD2
CGGGTGTAGACTCTAC-1	singlet	2	-1105.020201	-859.5027389	-393.5018264	EMD2
CGGGTGTAGAGTATAC-1	singlet	1	-703.501569	-231.9449211	-579.4651193	EMD2
CGGGTGTAGAGTTCGG-1	singlet	1	-984.7015763	-360.3590819	-787.4756039	EMD2
CGGGTGTAGCAACAAT-1	singlet	1	-2202.775098	-809.0256588	-1853.867628	EMD2
CGGGTGTAGGTGCTGA-1	singlet	2	-2920.946817	-2454.81061	-941.0660435	EMD2
CGGGTGTCAAGCACCC-1	singlet	2	-2485.583007	-2151.340324	-949.263756	EMD2
CGGGTGTCAGCTTTCC-1	singlet	2	-537.0966965	-545.2453565	-233.5848884	EMD2
CGGGTGTGTAGACACG-1	doublet	0/1	-90.93617305	-143.0764775	-141.0767554	EMD2
CGGGTGTGTTCAACGT-1	singlet	2	-1730.65284	-1508.418339	-649.7122104	EMD2
CGGGTGTTCGTCCTTG-1	singlet	2	-112.1317178	-84.76600535	-24.62995939	EMD2
CGGGTGTTCTATCGGA-1	singlet	2	-1077.20551	-1017.983213	-384.2950315	EMD2
CGGGTGTTCTATGTGG-1	singlet	1	-1755.848094	-486.3639482	-1168.105649	EMD2
CGGTCAGAGACCGCCT-1	singlet	0	-76.87652032	-152.2484774	-144.2383951	EMD2
CGGTCAGAGTATGATG-1	singlet	1	-287.9718431	-113.9875329	-211.0550655	EMD2
CGGTCAGGTAACACCT-1	singlet	1	-1851.435492	-565.60066	-1559.12926	EMD2
CGGTCAGGTAAGGCTG-1	singlet	1	-5082.201711	-1657.142602	-3899.850426	EMD2
CGGTCAGGTAGACGTG-1	singlet	0	-133.0578406	-373.7413699	-294.0083359	EMD2
CGGTCAGGTTCAATCG-1	singlet	1	-7172.057618	-2233.132109	-5583.772026	EMD2
CGTAAGTAGCTGGCCT-1	singlet	1	-1382.193028	-453.9933095	-1035.546016	EMD2
CGTAAGTAGCTTGTTG-1	doublet	45293	-6045.998261	-3088.098884	-3582.991816	EMD2
CGTAAGTCAAATACGA-1	singlet	2	-1611.725864	-1536.701025	-567.5466745	EMD2
CGTAAGTCACCCAACG-1	singlet	1	-319.9277833	-180.2466562	-325.1210899	EMD2
CGTAAGTCAGTTGAAA-1	singlet	2	-2361.968996	-1907.999102	-847.7848643	EMD2
CGTAAGTGTACAGGTG-1	singlet	1	-867.8814653	-272.3074795	-727.873215	EMD2
CGTAAGTGTCCATAGT-1	singlet	1	-125.8547394	-48.07415655	-120.923469	EMD2
CGTAAGTGTTCAATCG-1	singlet	2	-1122.962138	-902.1490728	-381.9120092	EMD2
CGTAAGTTCAGACATC-1	singlet	1	-318.7648724	-139.0854145	-250.8234348	EMD2

CGTAAGTTCGTTCTGC-1	singlet	2	-5891.588188	-4289.96642	-1184.436226	EMD2
CGTAATGAGGGCAATC-1	singlet	1	-782.3830752	-260.5833547	-523.9032508	EMD2
CGTAATGAGTCCTACA-1	singlet	1	-480.3880776	-218.1194933	-434.7958307	EMD2
CGTAATGCAAATTGGA-1	singlet	1	-690.4280379	-235.4558716	-662.6013428	EMD2
CGTAATGCAAGTTCCA-1	singlet	0	-495.7581503	-1439.818004	-1435.521148	EMD2
CGTAATGCACAGTCGC-1	singlet	2	-1565.95008	-1463.630854	-486.9749914	EMD2
CGTAATGCACATTCTT-1	singlet	0	-382.8139748	-1214.155182	-1097.647085	EMD2
CGTAATGGTGCACATT-1	singlet	1	-867.2603858	-315.7880548	-654.300335	EMD2
CGTAATGGTTCCGCAG-1	doublet	2/0	-4390.090737	-5261.27447	-3239.093455	EMD2
CGTAATGTCAGGAGAC-1	singlet	0	-1474.910311	-4954.93557	-4629.55031	EMD2
CGTAATGTCCGTATAG-1	singlet	1	-1957.333295	-603.1088329	-1545.415804	EMD2
CGTAATGTCTAGTGAC-1	singlet	1	-305.8239693	-120.7125514	-240.7130309	EMD2
CGTAATGTCTTCTGTA-1	singlet	0	-115.1736468	-272.5436818	-310.1948399	EMD2
CGTAGTAAGCTGGCCT-1	singlet	2	-2163.88816	-1765.76944	-751.8517971	EMD2
CGTAGTAAGGTAGACC-1	singlet	1	-5124.025877	-1600.697491	-4042.574942	EMD2
CGTAGTAGTAACGATA-1	singlet	1	-122.1196825	-68.85142776	-124.7130706	EMD2
CGTAGTAGTGTATTCG-1	singlet	1	-1034.36578	-312.1015419	-958.9532253	EMD2
CGTAGTAGTTCTCACC-1	singlet	1	-523.7240828	-219.0909671	-464.9675198	EMD2
CGTAGTATCCACTGGG-1	singlet	0	-747.811572	-1914.023355	-1753.982473	EMD2
CGTAGTATCCGGACGT-1	singlet	2	-1837.679345	-1339.429993	-590.1093703	EMD2
CGTAGTATCCTCTAGC-1	doublet	45323	-1743.972215	-1221.32794	-990.4372579	EMD2
CGTAGTATCGCTCTAC-1	singlet	1	-707.597257	-234.4537797	-478.3461959	EMD2
CGTAGTATCGGCCCAA-1	singlet	2	-650.7326304	-656.614774	-247.0132043	EMD2
CGTAGTATCTAGGCCG-1	singlet	1	-1269.150501	-464.5651673	-1116.940384	EMD2
CGTAGTATCTTACTGT-1	singlet	1	-1224.906327	-446.9752882	-1156.764366	EMD2
CGTCAAAAGACGAGCT-1	singlet	1	-1848.784134	-544.3694924	-1913.401344	EMD2
CGTCAAAAGCGCACAA-1	singlet	2	-695.0378687	-1161.111919	-404.5670248	EMD2
CGTCAAAAGCGGCTCT-1	singlet	1	-2749.070055	-835.8169069	-2006.115	EMD2
CGTCAAAAGGTAAGAG-1	singlet	1	-197.0803183	-62.79948043	-224.4731405	EMD2
CGTCAAAAGTGCCAGA-1	singlet	1	-729.1943077	-252.0255584	-538.342957	EMD2
CGTCAAAAGTGGATTA-1	singlet	0	-106.9221411	-451.886342	-301.7840894	EMD2
CGTCAAACACCGTGCA-1	doublet	1/0	-895.8252301	-628.9054442	-964.3681727	EMD2
CGTCAAACACTAACCA-1	singlet	1	-1978.193113	-689.9803596	-1719.363831	EMD2
CGTCAAACAGACGGAT-1	singlet	1	-930.7206948	-340.2668722	-642.2832313	EMD2
CGTCAAAGTGTAAACA-1	singlet	0	-451.8896713	-1386.847852	-1374.307494	EMD2
CGTCAAAGTTTCCCAC-1	singlet	0	-261.8360048	-923.6680815	-656.6384675	EMD2
CGTCAAATCAGCCTTC-1	singlet	2	-267.8618342	-260.3745026	-77.57429413	EMD2
CGTCCATAGACCCTTA-1	singlet	2	-4222.339926	-3434.115452	-1516.69717	EMD2
CGTCCATCAATCAAGA-1	singlet	2	-1955.527285	-1750.716962	-635.3725614	EMD2
CGTCCATCACATGAAA-1	singlet	0	-547.3855615	-1695.398035	-1602.325952	EMD2
CGTCCATCAGACTGCC-1	singlet	1	-2686.439797	-905.1542088	-2025.075157	EMD2
CGTCCATCAGCTGTAT-1	singlet	2	-85.12701583	-68.86679426	-26.02340724	EMD2
CGTCCATTCCTGCCAT-1	singlet	1	-344.0044514	-136.2584603	-274.7942589	EMD2
CGTCCATTCTGAGAAA-1	singlet	0	-115.4288284	-317.8838404	-321.8917467	EMD2
CGTGAATAGAAACTAC-1	singlet	1	-3774.953448	-1207.745152	-3000.080042	EMD2
CGTGAATAGTAACAGT-1	singlet	0	-907.2166887	-2811.029774	-2743.443118	EMD2
CGTGAATAGTCGGCAA-1	singlet	0	-587.2879263	-1911.276128	-1857.80583	EMD2
CGTGAATCAATAACGA-1	singlet	2	-231.0277216	-288.3053508	-111.1648071	EMD2
CGTGAATCAGTAGATA-1	singlet	0	-153.9831646	-325.8575019	-297.9197244	EMD2
CGTGAATCATATGGCT-1	singlet	1	-1397.375849	-459.1822164	-1219.119048	EMD2
CGTGAATGTCCGGACT-1	singlet	2	-286.9493449	-271.094236	-117.0648891	EMD2
CGTGAATGTGCGACAA-1	singlet	1	-1576.325945	-449.8407481	-1325.993351	EMD2
CGTGAATGTGTGTGGA-1	singlet	1	-1896.665803	-605.2892187	-1522.311867	EMD2
CGTGAATTCCGGGACT-1	doublet	45293	-595.9527653	-258.851967	-473.6067888	EMD2
CGTGATAAGACCAGAC-1	singlet	0	-218.8463367	-631.3888171	-508.3628657	EMD2
CGTGATAAGCCTATTG-1	singlet	1	-1671.171432	-527.3329895	-1212.521413	EMD2
CGTGATAAGCTCGTGC-1	singlet	0	-842.5917859	-2763.230727	-2612.165414	EMD2
CGTGATAAGGGACACT-1	singlet	0	-366.4161408	-1441.541013	-1349.255853	EMD2
CGTGATAAGGTAAAGG-1	unassigned	2	-12.29772617	-3.166665435	-2.054887149	EMD2
CGTGATAAGGTACATA-1	doublet	45293	-1012.184782	-434.0334926	-642.1035771	EMD2
CGTGATACAAATACAG-1	singlet	2	-332.3791314	-326.2939111	-181.6072922	EMD2
CGTGATACACCTAAAC-1	singlet	1	-2061.130318	-562.5903465	-1701.73675	EMD2
CGTGATACACTACACA-1	singlet	1	-3246.391582	-890.5734646	-2790.489297	EMD2
CGTGATAGTGTGATGG-1	singlet	1	-2229.690014	-713.6992947	-1707.405769	EMD2
CGTGATATCATCCTGC-1	singlet	1	-265.8326249	-103.7718226	-241.6898083	EMD2
CGTGATATCTGATGGT-1	singlet	1	-1610.981046	-518.7168915	-1282.294801	EMD2
CGTGCTTAGAGCCGTA-1	unassigned	0	-14.60996733	-20.2786619	-16.8201806	EMD2
CGTGCTTAGAGTCACG-1	singlet	2	-1635.449282	-1432.513428	-489.5111879	EMD2
CGTGCTTAGCCGGATA-1	singlet	0	-169.6496285	-601.8707706	-491.6159798	EMD2
CGTGCTTAGTAACAGT-1	singlet	0	-711.0630608	-2430.141381	-2354.83372	EMD2
CGTGCTTAGTTATGGA-1	singlet	2	-411.5414056	-372.7963019	-150.8488143	EMD2
CGTGCTTAGTTGAAGT-1	singlet	0	-764.2511837	-2388.800181	-2190.678326	EMD2
CGTGCTTCACCATTCC-1	singlet	1	-361.8476535	-166.3094373	-339.6746029	EMD2
CGTGCTTCAGTCGGAA-1	singlet	1	-3312.808774	-995.8229373	-2412.53029	EMD2
CGTGCTTCATGACGAG-1	singlet	2	-1427.933791	-1197.208747	-462.2632076	EMD2
CGTGCTTCATTGACTG-1	singlet	1	-252.219467	-97.51747622	-227.674547	EMD2
CGTGCTTGTCATCACA-1	singlet	0	-515.0136676	-1470.473439	-1376.622389	EMD2
CGTGCTTGTCCACTTC-1	singlet	1	-1009.12138	-396.8608103	-836.8261014	EMD2
CGTGCTTGTGCGAGTA-1	singlet	1	-1739.582484	-504.5336639	-1475.334542	EMD2
CGTGCTTTCCCGAATA-1	singlet	1	-73.95131952	-26.63834777	-59.34138448	EMD2
CGTGCTTTCCTCAGGG-1	singlet	1	-369.6663095	-142.2349129	-351.0282862	EMD2
CGTGCTTTCGCCAGAC-1	singlet	2	-2175.739525	-1843.411046	-765.7455887	EMD2
CGTGTCTAGATGCTAA-1	singlet	1	-1049.305828	-305.1120078	-1058.556226	EMD2
CGTGTCTCACTGATTG-1	singlet	1	-194.9837097	-82.25626997	-186.5435518	EMD2
CGTGTCTCATGAAGGC-1	singlet	1	-571.3734275	-210.7225272	-426.760304	EMD2
CGTGTCTGTTGGACTT-1	singlet	1	-85.95206615	-26.02817721	-71.9936021	EMD2
CGTTAGACAACTGCCG-1	singlet	0	-555.4660565	-1963.688051	-1878.838529	EMD2
CGTTAGACACGTTCGG-1	singlet	1	-2819.904072	-707.0445032	-2494.578748	EMD2
CGTTAGACACTTTATC-1	singlet	2	-4872.599058	-4240.325451	-1702.891582	EMD2
CGTTAGAGTACTAAGA-1	singlet	1	-807.5431337	-309.0030489	-687.8594561	EMD2
CGTTAGAGTGAGACGT-1	singlet	2	-1601.362805	-1271.73788	-541.0947994	EMD2
CGTTAGAGTTTAGTCG-1	singlet	1	-364.0255042	-116.7305355	-309.8466486	EMD2
CGTTAGATCCTTGAAG-1	singlet	0	-676.3689975	-1961.639002	-1894.217386	EMD2
CGTTAGATCGTCCTTG-1	singlet	1	-517.939678	-167.8855035	-495.0141226	EMD2
CGTTCTGAGAAGTATC-1	singlet	0	-167.2976966	-542.6034213	-443.3214599	EMD2
CGTTCTGAGGATACCG-1	singlet	2	-1210.200655	-1125.744564	-407.6752462	EMD2
CGTTCTGAGGGTCAAC-1	singlet	0	-197.5587251	-696.0240835	-651.1043395	EMD2
CGTTCTGCAACAGAGC-1	singlet	2	-660.1110145	-599.4504597	-261.7614266	EMD2
CGTTCTGCAAGTAGTA-1	singlet	1	-2623.025454	-867.875539	-1919.811219	EMD2
CGTTCTGGTGTTGATC-1	singlet	0	-561.0080087	-1764.532856	-1596.080807	EMD2
CGTTCTGGTTATGTCG-1	singlet	1	-285.7672664	-106.4632703	-258.879233	EMD2
CGTTCTGGTTGCTGAT-1	singlet	0	-607.1959498	-2209.366009	-2146.773486	EMD2
CGTTCTGTCCATCACC-1	singlet	1	-333.4983443	-132.8723809	-229.3098488	EMD2
CGTTGGGAGGGCTAAC-1	singlet	1	-2229.740153	-657.0015337	-1829.093196	EMD2
CGTTGGGCATATAGCC-1	singlet	1	-468.9619518	-191.6744512	-408.4194446	EMD2
CGTTGGGGTAACGATA-1	singlet	0	-430.9553366	-3218.373892	-2547.533926	EMD2
CGTTGGGTCATGAGGG-1	singlet	1	-1428.485818	-497.4975594	-1179.399777	EMD2
CGTTGGGTCGCGAAGA-1	singlet	0	-425.7574358	-1221.889651	-1185.00535	EMD2
CGTTGGGTCGGACGTC-1	singlet	1	-2235.119054	-677.247163	-1612.460735	EMD2
CGTTGGGTCTGCCTGT-1	singlet	1	-595.8601359	-217.8845331	-472.334497	EMD2

CTAACCCAGAATCGAT-1	singlet	0	-31.73093149	-112.4216646	-89.14793661	EMD2
CTAACCCAGTAACCGG-1	singlet	1	-2464.930158	-877.2150181	-1946.755767	EMD2
CTAACCCAGTGTGTTC-1	singlet	1	-2678.291751	-534.577553	-1864.288631	EMD2
CTAACCCCAAGACTGG-1	singlet	1	-2327.453949	-750.550821	-1808.740663	EMD2
CTAACCCCACTCCTGT-1	singlet	1	-519.044646	-191.5757998	-390.4615699	EMD2
CTAACCCCAGGATTCT-1	singlet	1	-2447.605486	-728.6395505	-1878.387749	EMD2
CTAACCCCAGTGCCTG-1	singlet	0	-252.4952012	-710.5294977	-668.3001977	EMD2
CTAACCCGTGATGAAT-1	singlet	1	-488.9126844	-189.5264655	-440.9284234	EMD2
CTAACCCGTTATAGAG-1	singlet	2	-1947.490089	-1831.665071	-764.5919396	EMD2
CTAACCCGTTCTCGTC-1	singlet	1	-1345.913776	-500.9297105	-1169.628424	EMD2
CTAACCCTCGGACCAC-1	singlet	0	-534.2206362	-1239.830427	-1259.398954	EMD2
CTAACCCTCGGCCCAA-1	singlet	1	-397.8173953	-166.0037054	-377.3963185	EMD2
CTAACCCTCTCCGAAA-1	singlet	2	-453.8050643	-509.3527152	-216.8183186	EMD2
CTAACTTAGATACATG-1	singlet	0	-760.428222	-2620.00869	-2265.87014	EMD2
CTAACTTAGGAATTAC-1	singlet	2	-842.5634062	-707.7470337	-337.9220306	EMD2
CTAACTTAGTAGATCA-1	singlet	0	-682.6692348	-4664.984886	-3575.826282	EMD2
CTAACTTGTACCAGAG-1	singlet	1	-2722.22146	-879.8357292	-2087.487686	EMD2
CTAACTTGTAGGAAAG-1	singlet	1	-665.7782382	-254.039588	-495.0046394	EMD2
CTAACTTGTATCCTCC-1	singlet	0	-384.0180947	-844.0348093	-811.3135346	EMD2
CTAACTTGTCCACATA-1	singlet	2	-2041.361014	-1822.640503	-736.7187353	EMD2
CTAACTTGTCCTGTCT-1	doublet	1/0	-2466.729734	-1779.453924	-2769.961825	EMD2
CTAACTTTCGAGATGG-1	singlet	1	-3136.26383	-912.6873243	-2271.175306	EMD2
CTAACTTTCTTACCGC-1	singlet	1	-543.8133538	-207.5137907	-456.225426	EMD2
CTAAGTGAGCTCGGCT-1	singlet	1	-3529.036738	-1020.190365	-2827.038302	EMD2
CTAAGTGAGGTGCTTT-1	singlet	1	-306.4489126	-113.152406	-266.0562464	EMD2
CTAAGTGCAGAGGCTA-1	singlet	1	-1553.64082	-409.5502801	-1203.492608	EMD2
CTAAGTGCAGTCAGTT-1	singlet	0	-1112.887353	-3715.67207	-3393.377959	EMD2
CTAAGTGGTGCAGATG-1	singlet	1	-1630.316984	-514.0246441	-1099.737112	EMD2
CTAAGTGTCAGATGCT-1	singlet	1	-2404.473256	-945.860499	-1957.391782	EMD2
CTAAGTGTCCCAAGTA-1	singlet	1	-1351.406302	-371.4636003	-912.5130784	EMD2
CTAAGTGTCGAGAAAT-1	singlet	1	-752.5033444	-238.2181929	-554.049827	EMD2
CTAAGTGTCGTTCCCA-1	singlet	2	-779.374408	-768.1626668	-269.5945076	EMD2
CTAAGTGTCTCCAATT-1	singlet	0	-631.1317441	-1689.227404	-1653.839757	EMD2
CTACAGAAGACGCAGT-1	singlet	2	-4025.461874	-3138.604708	-1238.194449	EMD2
CTACAGAAGCGACATG-1	singlet	1	-1652.432826	-494.4102859	-1195.033273	EMD2
CTACAGAAGGGCATGT-1	singlet	1	-4190.092384	-1339.866019	-3217.207459	EMD2
CTACAGAAGGTGCAGT-1	singlet	0	-1860.119119	-6361.212759	-6143.513899	EMD2
CTACAGAAGTTGGACG-1	singlet	1	-2518.631701	-797.5029771	-2085.45232	EMD2
CTACAGACAAGGTACG-1	singlet	1	-2060.184178	-758.1111929	-1875.33055	EMD2
CTACAGACACAAGTGG-1	singlet	0	-671.5934595	-2642.867829	-1947.94048	EMD2
CTACAGACACGCTGCA-1	singlet	1	-133.3988325	-61.92084046	-137.6690099	EMD2
CTACAGAGTCCTACGG-1	singlet	0	-516.0576041	-1589.246488	-1484.325034	EMD2
CTACAGATCAACACCA-1	singlet	1	-1951.175432	-645.7142138	-1638.616633	EMD2
CTACAGATCACTAGCA-1	singlet	1	-735.3191323	-243.3656189	-705.3052185	EMD2
CTACAGATCGGTCTAA-1	singlet	1	-2246.149617	-774.9419842	-1968.227051	EMD2
CTACAGATCTTCGCTG-1	singlet	1	-5400.71189	-1392.73653	-3832.018645	EMD2
CTACATTCAAATGGTA-1	singlet	0	-1224.970377	-3566.75036	-3482.959594	EMD2
CTACATTCAGAACATA-1	singlet	1	-287.8452912	-109.6273947	-243.1670274	EMD2
CTACATTCAGCGTTGC-1	singlet	2	-1034.952385	-876.0681681	-363.2435445	EMD2
CTACATTCAGTTCACA-1	singlet	1	-503.5786083	-203.2086712	-456.8869538	EMD2
CTACATTGTAAGCAAT-1	singlet	1	-1413.445143	-452.7295216	-1159.959705	EMD2
CTACATTGTGAGTAGC-1	doublet	45323	-4872.465947	-2993.77195	-2306.903142	EMD2
CTACATTTCAGCAATC-1	singlet	1	-717.9535743	-279.2146641	-584.897284	EMD2
CTACATTTCCGCACGA-1	singlet	1	-427.1117503	-217.1014067	-435.6156593	EMD2
CTACCCAAGAGGTTTA-1	singlet	1	-597.9355849	-181.2866853	-418.2845766	EMD2
CTACCCAAGCCGATTT-1	singlet	2	-1330.47477	-1171.865005	-479.5478129	EMD2
CTACCCAAGCTGTCCG-1	doublet	0/2	-2742.980801	-5122.703389	-4283.290391	EMD2
CTACCCACACCAAATC-1	doublet	0/1	-2258.752018	-2452.959065	-3190.564297	EMD2
CTACCCACATACGCAT-1	singlet	0	-1329.627671	-4123.430201	-3790.921288	EMD2
CTACCCACATTGAGGG-1	singlet	1	-1603.025249	-464.2856436	-1270.31414	EMD2
CTACCCATCACCGGGT-1	unassigned	2	-5.248923236	-1.910816197	-1.717498266	EMD2
CTACCCATCCTTCTAA-1	singlet	0	-107.6292462	-285.5725062	-210.644354	EMD2
CTACCCATCGAAGCAG-1	singlet	2	-2421.690656	-2150.524235	-944.7490141	EMD2
CTACCCATCGGTCAGC-1	singlet	2	-831.6544269	-771.3411913	-329.055056	EMD2
CTACCCATCTCTGCCA-1	singlet	0	-337.4855765	-995.0065812	-926.1023559	EMD2
CTACCCATCTGGTTGA-1	singlet	0	-204.6375877	-567.9055672	-537.9181338	EMD2
CTACCCATCTTGGGCG-1	singlet	1	-2704.498676	-902.6649654	-2193.003757	EMD2
CTACCTGAGGGTGAAA-1	singlet	0	-168.1691757	-520.0557011	-437.4426683	EMD2
CTACCTGAGTACTGTC-1	singlet	1	-421.7155561	-179.9155103	-338.7242147	EMD2
CTACCTGAGTATGACA-1	singlet	1	-5616.331513	-1856.866428	-4518.203593	EMD2
CTACCTGAGTGTTCCA-1	singlet	1	-1669.807995	-588.5161568	-1315.609725	EMD2
CTACCTGCAACCGCTG-1	singlet	1	-641.9295215	-244.4432086	-508.6619999	EMD2
CTACCTGCAGACATCT-1	singlet	2	-3758.170335	-3021.791717	-1179.536532	EMD2
CTACCTGCAGTGTATC-1	singlet	0	-1771.055591	-6197.95839	-5893.504949	EMD2
CTACCTGCAGTTAAAG-1	singlet	0	-1337.354364	-5102.622112	-4522.970675	EMD2
CTACCTGGTGAAAGTT-1	singlet	2	-5072.086401	-4006.600857	-1720.670416	EMD2
CTACCTGGTTCAGTAC-1	singlet	1	-246.3862094	-103.0303417	-258.6404886	EMD2
CTACCTGTCGCTACGG-1	singlet	0	-706.2322644	-2665.901001	-2503.997966	EMD2
CTACCTGTCTAAGGAA-1	singlet	0	-765.0582749	-2576.217949	-2312.807525	EMD2
CTACGGGAGAAGGCTC-1	doublet	0/2	-4368.430605	-7579.570395	-5195.487414	EMD2
CTACGGGAGCCAGAGT-1	singlet	2	-3932.588157	-2927.703641	-1599.858612	EMD2
CTACGGGAGCCTATTG-1	singlet	1	-205.9309486	-54.4092204	-146.6957405	EMD2
CTACGGGAGCGAATGC-1	singlet	0	-449.2063815	-1425.617102	-1288.691929	EMD2
CTACGGGAGCTTTCTT-1	singlet	1	-1141.201725	-480.009632	-1110.484071	EMD2
CTACGGGAGGGCAGGA-1	singlet	1	-250.7423495	-99.41654503	-231.5381565	EMD2
CTACGGGAGTCGTTAC-1	singlet	0	-79.21473133	-184.1727907	-185.9214747	EMD2
CTACGGGAGTTGCGAG-1	singlet	0	-893.1496981	-3168.167223	-3129.27479	EMD2
CTACGGGCACAACGCC-1	singlet	1	-452.7949937	-200.9089664	-423.7436292	EMD2
CTACGGGCACTGGCGT-1	singlet	1	-1021.435747	-315.1861699	-899.5574692	EMD2
CTACGGGCAGAGTTGG-1	singlet	2	-899.319655	-826.5926463	-368.1662925	EMD2
CTACGGGCATGACACT-1	singlet	2	-5838.899179	-5684.588549	-2054.912355	EMD2
CTACGGGGTATCGTTG-1	singlet	1	-1124.475801	-364.3198933	-925.1496949	EMD2
CTACGGGGTCCTGGGT-1	singlet	1	-5979.400108	-1809.314914	-4634.637389	EMD2
CTACGGGGTCTGTCAA-1	doublet	0/1	-7.086147069	-9.522335055	-11.56307439	EMD2
CTACGGGGTTCTGAGT-1	singlet	2	-124.1185397	-108.8906115	-50.8708809	EMD2
CTACGGGGTTTCGGCG-1	singlet	2	-413.1920217	-338.2724001	-136.9880941	EMD2
CTACGGGTCCGACGGT-1	singlet	0	-659.5931194	-2662.171538	-2549.533471	EMD2
CTACGGGTCGATAACC-1	singlet	1	-2568.707201	-887.6280606	-2097.875015	EMD2
CTACTATAGAGTTGTA-1	singlet	1	-299.7319519	-133.181578	-254.1801184	EMD2
CTACTATCACGACGTC-1	singlet	1	-5775.472234	-1263.254983	-4268.559766	EMD2
CTACTATCACGAGAAC-1	singlet	1	-823.1197843	-339.2966692	-701.3591143	EMD2
CTACTATCAGTGTGGA-1	singlet	1	-2351.918207	-555.7043903	-2014.836722	EMD2
CTACTATGTAATGTGA-1	singlet	2	-5489.308616	-4410.145246	-1626.446671	EMD2
CTACTATTCAAGCTGT-1	unassigned	1/0	-14.34792374	-13.59280785	-20.28401265	EMD2
CTACTATTCCCTAGGG-1	singlet	2	-2721.697444	-2610.871055	-1020.128628	EMD2
CTACTATTCGACACCG-1	singlet	0	-753.3600741	-1872.299531	-1886.555647	EMD2
CTACTATTCTGTTCAT-1	singlet	1	-1054.218799	-380.4975027	-818.5189402	EMD2
CTAGACAAGGGCGAGA-1	singlet	0	-52.32782954	-164.5690883	-131.2872784	EMD2
CTAGACAAGGTTACCT-1	singlet	1	-2327.204224	-676.6655256	-1994.480611	EMD2
CTAGACACAAGAATAC-1	singlet	2	-2373.454861	-1981.917774	-886.50053	EMD2

CTAGACACATTAGGCT-1	singlet	1	-4301.966629	-1318.484935	-3418.507685	EMD2
CTAGACAGTATCCTCC-1	singlet	1	-3323.836767	-1382.514323	-2332.793673	EMD2
CTAGACAGTGTATTGC-1	singlet	1	-683.2226837	-257.2506732	-590.3128003	EMD2
CTAGACAGTTCAAGTC-1	singlet	1	-1101.832611	-340.8455536	-964.5175927	EMD2
CTAGACATCAGCCCAG-1	singlet	0	-454.3090201	-1394.001406	-1484.333116	EMD2
CTAGACATCGCTTAAG-1	singlet	0	-410.9166485	-1173.846353	-1085.98426	EMD2
CTAGACATCTAGAGCT-1	singlet	0	-177.2723665	-710.9504807	-569.4392305	EMD2
CTAGACATCTCTGCCA-1	singlet	2	-2298.345804	-2365.717754	-879.0879897	EMD2
CTAGGTAAGCCTCAGC-1	singlet	0	-211.5041542	-888.7112623	-714.2575414	EMD2
CTAGGTAAGGTTAAAC-1	singlet	1	-3490.847044	-1054.215412	-3015.446302	EMD2
CTAGGTACAAGTACCT-1	singlet	1	-562.8262683	-193.8650599	-459.781612	EMD2
CTAGGTACAAGTCCAT-1	singlet	0	-970.6536956	-3082.105623	-2850.143327	EMD2
CTAGGTAGTGCCGAAA-1	singlet	1	-1849.811705	-606.5818544	-1496.56529	EMD2
CTAGGTATCACACCCT-1	singlet	1	-729.3771098	-251.5108406	-539.4539692	EMD2
CTAGGTATCGCTAGCG-1	singlet	1	-617.4836374	-179.8413876	-464.5370263	EMD2
CTAGGTATCTGAGATC-1	singlet	2	-426.8995712	-430.1598155	-226.7934114	EMD2
CTATAGGAGCGTGAGT-1	singlet	0	-757.2750853	-2282.721823	-2259.73625	EMD2
CTATAGGCAACTGCTA-1	singlet	1	-577.802649	-223.7134384	-496.8818449	EMD2
CTATAGGCAAGATGTA-1	singlet	2	-624.9824422	-542.7040788	-226.5700451	EMD2
CTATAGGCAGGAATAT-1	singlet	1	-277.5013072	-125.8322278	-256.322581	EMD2
CTATAGGCATATCTCT-1	singlet	0	-257.3053243	-781.1015201	-663.919556	EMD2
CTATAGGGTATTTCGG-1	singlet	0	-713.6809412	-1889.735479	-1937.166862	EMD2
CTATAGGGTGTGCTTA-1	singlet	0	-1061.909772	-4431.529794	-4223.778188	EMD2
CTATAGGGTTGAGAGC-1	singlet	1	-969.3295638	-373.5247288	-803.6714344	EMD2
CTATAGGTCCAACACA-1	singlet	0	-330.3318212	-983.483002	-909.5132333	EMD2
CTATAGGTCCACACAA-1	singlet	1	-3125.653684	-1157.394476	-2687.045405	EMD2
CTATAGGTCCCGAATA-1	singlet	1	-451.0212098	-154.55263	-372.7589055	EMD2
CTATAGGTCCCGTGAG-1	singlet	0	-540.4307746	-1606.224666	-1466.162229	EMD2
CTATCCGAGACGGTTG-1	singlet	1	-301.5696892	-113.07793	-248.6275245	EMD2
CTATCCGCAATAGGGC-1	singlet	1	-309.8563735	-99.7350023	-225.9417376	EMD2
CTATCCGGTAGAGACC-1	singlet	1	-271.7199197	-97.89897285	-241.3932343	EMD2
CTATCCGGTATGGAAT-1	singlet	1	-2765.083509	-853.1962708	-2169.281761	EMD2
CTATCCGGTCTGTAGT-1	singlet	2	-2778.643329	-2111.09797	-1010.753069	EMD2
CTATCCGGTTAACCTG-1	singlet	1	-2524.023036	-696.2390332	-1806.025194	EMD2
CTATCCGGTTTCAGAC-1	singlet	1	-776.3897137	-248.7887068	-589.7483643	EMD2
CTATCCGTCCGAGGCT-1	singlet	1	-5192.785304	-1551.602258	-4182.064381	EMD2
CTATCCGTCGGACGTC-1	singlet	1	-260.4685815	-81.52454138	-213.7897937	EMD2
CTATCCGTCTCCGAGG-1	singlet	1	-1061.670247	-380.6811187	-911.4974715	EMD2
CTATCCGTCTTTGATC-1	singlet	1	-1037.682116	-397.2462994	-813.0946833	EMD2
CTATCTAAGCGACTAG-1	singlet	0	-2872.274466	-8126.352745	-8178.79906	EMD2
CTATCTACAACATCGT-1	singlet	0	-26.87800824	-71.85349603	-77.05549693	EMD2
CTATCTACAATAGTGA-1	singlet	1	-332.2092065	-141.3534742	-235.9294495	EMD2
CTATCTACAGTAACCT-1	singlet	1	-740.1660297	-284.9308775	-566.445823	EMD2
CTATCTATCGTACACA-1	singlet	1	-2107.08815	-813.8487474	-1755.866713	EMD2
CTATCTATCTCATGCC-1	singlet	2	-1143.364449	-1083.537047	-412.3871816	EMD2
CTCAACCAGAATTCAG-1	singlet	2	-3352.332504	-2882.384524	-1253.564867	EMD2
CTCAACCAGAGTGTGC-1	singlet	1	-895.7629335	-325.9421631	-695.1814755	EMD2
CTCAACCAGCGTGAAC-1	singlet	1	-3140.250425	-1130.090575	-2823.828157	EMD2
CTCAACCAGGATAATC-1	singlet	1	-2206.933476	-800.6144871	-1870.225876	EMD2
CTCAACCCAAACGTGG-1	singlet	1	-1044.365345	-408.6110546	-974.7264462	EMD2
CTCAACCCACAATTCG-1	singlet	1	-344.6563383	-153.1038295	-323.6550542	EMD2
CTCAACCCACAGCCAC-1	singlet	0	-31.37494391	-104.5277142	-91.72956072	EMD2
CTCAACCCAGAAGCTG-1	singlet	1	-2212.488221	-624.9301564	-1685.972449	EMD2
CTCAACCCATAACGGG-1	singlet	1	-1308.130973	-473.6769676	-1134.40172	EMD2
CTCAACCGTCCTCAGG-1	singlet	2	-3743.905127	-3081.890543	-1361.786987	EMD2
CTCAACCTCATCGCTC-1	singlet	0	-2464.562366	-5990.638725	-6087.976808	EMD2
CTCAACCTCTATCGTT-1	singlet	1	-587.2732428	-223.6167791	-553.8649776	EMD2
CTCAAGAAGATCGGTG-1	singlet	0	-67.23680872	-178.1858824	-192.9238625	EMD2
CTCAAGAAGATGCTGG-1	singlet	0	-139.4158948	-333.3826426	-296.5990083	EMD2
CTCAAGAAGCACGATG-1	singlet	1	-1880.724485	-385.629583	-1284.806897	EMD2
CTCAAGAAGGGTGAAA-1	singlet	1	-419.3423691	-183.1020531	-386.2150588	EMD2
CTCAAGAAGGGTTAGC-1	singlet	1	-664.6466206	-229.4429374	-489.6547557	EMD2
CTCAAGACAAGGATGC-1	singlet	0	-45.09863928	-131.8704773	-121.6677356	EMD2
CTCAAGACAGAAATTG-1	singlet	1	-390.1058582	-160.2115536	-320.9886173	EMD2
CTCAAGACATGTGCCG-1	singlet	1	-676.8565016	-252.2748478	-511.0887571	EMD2
CTCAAGAGTAACGGTG-1	singlet	1	-1941.093527	-626.8373324	-1660.54481	EMD2
CTCAAGAGTACCTAAC-1	singlet	1	-594.8606346	-231.9785732	-484.4561315	EMD2
CTCAAGAGTCCTTTGC-1	singlet	1	-406.8600861	-167.0325383	-304.4658269	EMD2
CTCAAGAGTCGGCACT-1	singlet	1	-414.6621017	-173.6886963	-393.4675328	EMD2
CTCAAGAGTCGGTGTC-1	singlet	1	-2563.769183	-764.6221104	-2024.427541	EMD2
CTCAAGAGTGTAGTGG-1	unassigned	1/0	-7.679813038	-4.849873234	-6.667801651	EMD2
CTCAAGAGTTATCTTC-1	singlet	1	-2094.538985	-611.0365572	-1754.52968	EMD2
CTCAAGATCAGACATC-1	singlet	2	-1937.839069	-1620.137341	-620.8352494	EMD2
CTCAATTAGAAGTCTA-1	singlet	1	-424.9283279	-193.8464732	-398.0526268	EMD2
CTCAATTAGATGGTCG-1	singlet	2	-5203.257571	-4303.546419	-1670.686974	EMD2
CTCAATTAGCGATGGT-1	singlet	2	-963.3031739	-829.2092687	-362.4786342	EMD2
CTCAATTAGCTCGTGC-1	singlet	2	-2675.066511	-2612.894448	-823.3400573	EMD2
CTCAATTAGGACTAAT-1	doublet	45323	-6684.945514	-4452.224806	-2838.889492	EMD2
CTCAATTAGGTCGAGT-1	singlet	1	-1166.997503	-405.8809646	-1034.115238	EMD2
CTCAATTCACGGCACT-1	singlet	2	-5512.531716	-4136.029473	-1654.426314	EMD2
CTCAATTCAGCGCTTG-1	singlet	2	-430.9165327	-361.6568898	-169.0532064	EMD2
CTCAATTGTTCTCGTC-1	singlet	1	-2785.242828	-948.9285285	-2468.269239	EMD2
CTCAATTTCAGCATTG-1	singlet	2	-533.1910755	-455.9904392	-205.2448133	EMD2
CTCAATTTCCATTTCA-1	singlet	1	-623.5080325	-194.7566168	-497.8827301	EMD2
CTCACTGAGACCATAA-1	singlet	1	-1020.236399	-308.7069518	-958.4191445	EMD2
CTCACTGAGACTGTTC-1	singlet	1	-1123.580002	-387.57496	-869.7399488	EMD2
CTCACTGAGATAGGGA-1	singlet	1	-1557.719262	-475.7694736	-1281.606327	EMD2
CTCACTGAGGTATTGA-1	singlet	1	-1049.707724	-405.5107225	-942.4321744	EMD2
CTCACTGAGTCCCGGT-1	singlet	1	-2447.954831	-730.0605834	-2256.80815	EMD2
CTCACTGAGTTCCGGC-1	singlet	1	-500.5138535	-181.4917174	-351.2079826	EMD2
CTCACTGCAGTAGAAT-1	singlet	2	-322.2291715	-272.3183773	-121.2605135	EMD2
CTCACTGCATCCTATT-1	singlet	0	-318.8383605	-1072.338078	-864.8059601	EMD2
CTCACTGGTGCTCGTG-1	unassigned	1	-3.553353463	-1.564800854	-2.500327537	EMD2
CTCACTGGTTAGTTCG-1	singlet	2	-1093.25836	-1197.766657	-421.0884165	EMD2
CTCACTGTCGCGTCGA-1	singlet	0	-635.2092831	-1889.044702	-1735.824476	EMD2
CTCAGAACACCCTAGG-1	singlet	0	-306.3787771	-868.2006047	-886.4282516	EMD2
CTCAGAACACTAGTAC-1	singlet	1	-420.4953622	-184.6030959	-355.6354716	EMD2
CTCAGAACAGGACTAG-1	singlet	1	-457.7990023	-167.3057678	-394.5925163	EMD2
CTCAGAAGTACAGAGC-1	singlet	1	-384.9756142	-151.4974842	-328.0400394	EMD2
CTCAGAAGTCTTACTT-1	singlet	1	-1093.804221	-440.6139056	-937.4706227	EMD2
CTCAGAAGTGACTCGC-1	singlet	1	-306.9447515	-143.2962768	-345.3753533	EMD2
CTCAGAAGTTAGAGAT-1	singlet	0	-1482.77569	-4515.068949	-4237.715027	EMD2
CTCAGAATCTCCGAGG-1	singlet	1	-348.9079368	-165.6832391	-330.0503559	EMD2
CTCAGGGAGAATAGTC-1	singlet	1	-290.5241984	-116.9287698	-249.6375989	EMD2
CTCAGGGAGACAAGCC-1	singlet	1	-632.751012	-238.7482295	-481.074292	EMD2
CTCAGGGAGGATTTCC-1	singlet	1	-1939.08596	-515.9653659	-1492.826435	EMD2
CTCAGGGAGGTCTACT-1	singlet	1	-449.2089081	-153.1934348	-354.1949745	EMD2
CTCAGGGAGTAATACG-1	singlet	1	-440.6774133	-183.9476942	-369.2578052	EMD2
CTCAGGGAGTTCTACG-1	singlet	1	-2872.423025	-992.9018692	-2506.769388	EMD2
CTCAGGGCACGTATAC-1	singlet	1	-449.318277	-149.2438464	-310.0402948	EMD2

CTCAGGGGTAGCTGAG-1	singlet	1	-1207.624012	-319.3640229	-1087.356844	EMD2
CTCAGGGGTATCGAGG-1	singlet	1	-1600.309095	-585.1530943	-1186.188142	EMD2
CTCAGGGGTCAGATTC-1	singlet	2	-1344.364638	-1197.432819	-464.8895525	EMD2
CTCAGGGGTCTACACA-1	singlet	0	-1020.244402	-2183.249492	-2121.440214	EMD2
CTCAGGGTCATCGTAG-1	singlet	1	-217.5841524	-73.54149847	-177.1696471	EMD2
CTCAGGGTCCACCTGT-1	singlet	1	-585.5448985	-206.7688627	-480.5859368	EMD2
CTCAGTCAGTTTAGGA-1	singlet	1	-2109.930873	-695.607757	-1568.631931	EMD2
CTCAGTCCACCACATA-1	singlet	1	-625.1557512	-268.0964845	-584.6049241	EMD2
CTCAGTCCAGGTGAGT-1	singlet	1	-6734.961705	-1949.636495	-5253.184919	EMD2
CTCAGTCGTCGCACAC-1	singlet	1	-269.9279726	-115.0401938	-213.5541287	EMD2
CTCAGTCGTGACAGCA-1	singlet	1	-537.4508118	-200.0013002	-542.2044711	EMD2
CTCAGTCGTGACCGAA-1	singlet	1	-1385.619271	-504.4188885	-1354.938709	EMD2
CTCAGTCTCACTGCTC-1	doublet	45293	-1900.778547	-1095.049834	-1478.392056	EMD2
CTCAGTCTCCTCAGGG-1	singlet	1	-2234.366392	-521.5965646	-1947.879422	EMD2
CTCAGTCTCGGCTATA-1	singlet	1	-527.060916	-180.4997169	-404.6368805	EMD2
CTCAGTCTCTGCGGGT-1	singlet	2	-3592.634085	-2794.279867	-1099.310878	EMD2
CTCATCGAGATCGACG-1	singlet	1	-639.3960735	-273.9971665	-582.4110046	EMD2
CTCATCGAGTGAGGCT-1	singlet	2	-2383.220362	-1857.250726	-750.7064265	EMD2
CTCATCGCACAGTATC-1	singlet	2	-212.2048731	-207.2640623	-83.14633023	EMD2
CTCATCGGTAGGAGGG-1	singlet	1	-699.1852711	-222.3922147	-476.8051038	EMD2
CTCATCGGTTCTTGCC-1	singlet	2	-840.2832556	-772.7269404	-243.7330351	EMD2
CTCATCGGTTTACCAG-1	singlet	1	-2062.250316	-601.8939836	-1417.432689	EMD2
CTCATCGTCCGTTGAA-1	singlet	2	-3818.895452	-3322.164744	-1255.223122	EMD2
CTCATGCAGCACCGAA-1	singlet	1	-3229.848351	-1066.031891	-2618.164165	EMD2
CTCATGCAGCTACTAC-1	singlet	2	-1131.747574	-1091.17086	-413.8309041	EMD2
CTCATGCAGGTCCCTG-1	singlet	2	-1893.790481	-1814.473337	-664.7416419	EMD2
CTCATGCAGGTTAAAC-1	singlet	1	-505.0256078	-189.7335397	-372.1958202	EMD2
CTCATGCCAATCGCAT-1	singlet	1	-643.8932306	-240.6414586	-564.5010306	EMD2
CTCATGCCACATTGTG-1	singlet	1	-2058.341946	-636.2052745	-1772.941191	EMD2
CTCATGCCACCAGCTG-1	singlet	2	-1447.73011	-1340.775339	-445.716387	EMD2
CTCATGCCAGAACGCA-1	singlet	0	-324.3185516	-1130.365291	-1048.742916	EMD2
CTCATGCCAGCTCTGG-1	singlet	0	-886.8807006	-1802.45538	-1873.969015	EMD2
CTCATGCGTTGAGTCT-1	singlet	1	-1033.295286	-385.8198018	-814.316878	EMD2
CTCATGCTCCAACCAA-1	singlet	1	-432.1039527	-202.2586077	-378.4711178	EMD2
CTCATTAAGCGCAATG-1	singlet	0	-2201.921222	-7471.058911	-6728.297921	EMD2
CTCATTACACAGCTTA-1	singlet	1	-412.3212724	-157.2859932	-300.6388969	EMD2
CTCATTACACATACGT-1	singlet	1	-1980.872652	-641.00298	-1719.548372	EMD2
CTCATTACACGGCACT-1	singlet	2	-252.6054959	-240.2008052	-108.5874373	EMD2
CTCATTACATTCAGCA-1	singlet	1	-744.2518397	-376.6714633	-651.959482	EMD2
CTCATTAGTACGGATG-1	singlet	0	-889.1453276	-2549.286876	-2497.700904	EMD2
CTCATTAGTCGGTGAA-1	singlet	1	-525.375922	-211.9207941	-536.1035913	EMD2
CTCATTATCGGTCGGT-1	singlet	1	-906.8974451	-290.0150209	-754.2953946	EMD2
CTCATTATCTTTGATC-1	singlet	1	-389.2788196	-174.5995768	-331.0014785	EMD2
CTCCAACAGAGCTGCA-1	singlet	2	-2206.937562	-1667.575017	-837.4347301	EMD2
CTCCAACAGGTTTACC-1	singlet	1	-3822.51638	-1268.169749	-3161.649803	EMD2
CTCCAACAGTAGCAAT-1	singlet	1	-618.542844	-184.4926439	-453.0397734	EMD2
CTCCAACAGTATGATG-1	singlet	1	-1865.516697	-519.3048026	-2096.496193	EMD2
CTCCAACCAATTAGGA-1	singlet	1	-2217.242832	-672.9430904	-1870.141509	EMD2
CTCCAACCACGGTGCT-1	singlet	1	-521.1831184	-222.9643319	-526.4187559	EMD2
CTCCAACTCCAACTAG-1	singlet	2	-5441.540272	-4387.639666	-1469.609764	EMD2
CTCCAACTCGCAGTCG-1	singlet	2	-388.7289977	-424.9286457	-145.4412058	EMD2
CTCCAACTCGCGTGAC-1	singlet	1	-306.9210915	-151.2196626	-251.0930738	EMD2
CTCCAACTCTGGGAGA-1	doublet	1/0	-371.1904174	-175.5888595	-297.2303047	EMD2
CTCCACAAGAAGTGTT-1	singlet	1	-231.4111207	-92.92154545	-240.3549079	EMD2
CTCCACAAGAGTCACG-1	singlet	1	-1286.712801	-432.6421527	-1185.280742	EMD2
CTCCACAAGGAACGAA-1	singlet	1	-2637.123858	-761.2065817	-2160.788331	EMD2
CTCCACAAGGTCACCC-1	singlet	1	-992.2056462	-402.7666032	-832.1818239	EMD2
CTCCACAAGTGCAGGT-1	singlet	1	-5490.524705	-1632.490386	-4384.785459	EMD2
CTCCACAAGTTGCCCG-1	singlet	2	-39.27885531	-39.15522497	-30.65972245	EMD2
CTCCACACAACGAGGT-1	singlet	1	-259.3525366	-84.54638901	-176.8405054	EMD2
CTCCACACAGCTTCCT-1	singlet	1	-704.2407191	-257.683677	-478.0928412	EMD2
CTCCACAGTACCTGTA-1	singlet	1	-1321.737274	-444.0548279	-1056.37214	EMD2
CTCCACAGTATGGAGC-1	singlet	2	-638.504625	-521.9350005	-229.5928894	EMD2
CTCCACAGTGATTGGG-1	singlet	1	-715.9305149	-184.3626627	-478.2978909	EMD2
CTCCACAGTTCGTAAC-1	singlet	2	-484.391073	-441.567341	-244.476701	EMD2
CTCCACATCCATCAGA-1	singlet	1	-2325.833745	-859.7956091	-2165.121169	EMD2
CTCCACATCTATCGTT-1	singlet	2	-525.6682567	-438.0249607	-182.8646658	EMD2
CTCCACATCTTGCAAG-1	singlet	2	-3638.106291	-3033.312065	-1022.795526	EMD2
CTCCATGCACAGTGAG-1	singlet	0	-103.6335726	-299.6208636	-289.4953758	EMD2
CTCCATGGTACACGTT-1	singlet	0	-473.7749472	-1477.530415	-1390.760558	EMD2
CTCCATGGTCAATCTG-1	singlet	1	-1363.515901	-421.8352417	-1024.67264	EMD2
CTCCATGGTGACCGTC-1	singlet	1	-299.1270207	-102.1397851	-268.8120244	EMD2
CTCCATGGTTAGTTCG-1	singlet	1	-714.1585436	-237.15358	-511.6236221	EMD2
CTCCATGTCCAACTGA-1	singlet	0	-1087.552484	-3023.498302	-3054.025227	EMD2
CTCCATGTCCCACAGG-1	singlet	2	-1127.121637	-1216.966904	-462.876869	EMD2
CTCCATGTCGCAGTTA-1	singlet	1	-3612.479231	-1177.696014	-2984.903871	EMD2
CTCCATGTCGCATTAG-1	singlet	1	-1027.947485	-329.6254317	-861.1746289	EMD2
CTCCCAAAGAAGGCTC-1	singlet	2	-2535.317348	-2580.592785	-756.0932615	EMD2
CTCCCAAAGCACCCAC-1	singlet	0	-1821.430725	-5241.526823	-5164.074422	EMD2
CTCCCAAAGGTAGTAT-1	singlet	1	-1249.142627	-498.6857722	-1201.258036	EMD2
CTCCCAACAAGACGGT-1	singlet	0	-290.0644448	-933.1254733	-728.7430186	EMD2
CTCCCAACACAAGCCC-1	singlet	2	-5547.625708	-4538.728787	-1906.168194	EMD2
CTCCCAACACGACCTG-1	singlet	1	-1591.251028	-504.3531564	-1361.636822	EMD2
CTCCCAACACGTCGTG-1	doublet	45323	-6985.46229	-4777.734222	-3172.439927	EMD2
CTCCCAACACTAGGCC-1	singlet	1	-3307.588867	-1112.175737	-2791.220368	EMD2
CTCCCAACAGTAACCT-1	singlet	1	-1703.972692	-639.1666055	-1414.806527	EMD2
CTCCCAACATGGGAAC-1	singlet	2	-2563.893814	-2261.987267	-818.000708	EMD2
CTCCCAAGTTGCGGCT-1	singlet	0	-266.0992384	-696.0129548	-659.7658227	EMD2
CTCCCAATCCCTGGTT-1	singlet	0	-99.41817057	-364.7890878	-260.0445943	EMD2
CTCCCAATCTTAAGGC-1	singlet	2	-4121.448832	-3386.821981	-1187.523504	EMD2
CTCCCTCAGCACTCAT-1	singlet	1	-175.6136247	-85.36332687	-180.7664329	EMD2
CTCCCTCAGCATGAAT-1	singlet	2	-148.3272108	-105.4599786	-46.98713306	EMD2
CTCCCTCAGGTGATCG-1	singlet	1	-2685.302864	-818.2988381	-2240.284054	EMD2
CTCCCTCAGTGCAAAT-1	singlet	1	-452.6780077	-180.146328	-421.473953	EMD2
CTCCCTCCAAATGCTC-1	singlet	2	-3681.495377	-2800.063167	-1367.786356	EMD2
CTCCCTCCAACACAGG-1	singlet	1	-4258.528588	-1439.290147	-3263.146955	EMD2
CTCCCTCCAATCAAGA-1	singlet	2	-382.8582506	-355.1735394	-160.748447	EMD2
CTCCCTCCACCGTCTT-1	singlet	1	-1274.167886	-345.2112941	-1004.255281	EMD2
CTCCCTCGTTGGATCT-1	singlet	1	-3344.213356	-947.7705685	-2416.712249	EMD2
CTCCCTCTCACTTATC-1	singlet	1	-836.6018458	-327.5143888	-674.0226721	EMD2
CTCCGATAGAGAGTTT-1	singlet	1	-952.0853957	-320.3515009	-811.6328325	EMD2
CTCCGATAGAGTCCGA-1	singlet	2	-297.4386072	-338.2344655	-105.3126654	EMD2
CTCCGATAGGGCGAAG-1	singlet	2	-2365.080249	-1813.151175	-724.9255119	EMD2
CTCCGATAGGTGATCG-1	singlet	2	-1947.384156	-1946.316186	-645.2524667	EMD2
CTCCGATAGTACGTCT-1	singlet	2	-546.7538716	-519.960323	-193.8065104	EMD2
CTCCGATCAGACGGAT-1	singlet	0	-492.321525	-1898.62672	-1790.713824	EMD2
CTCCGATCAGAGGCAT-1	singlet	0	-1035.676779	-3644.091857	-3499.315559	EMD2
CTCCGATCAGCAGATG-1	singlet	0	-478.6043321	-1752.139859	-1689.23718	EMD2
CTCCGATCATCTCGTC-1	singlet	1	-113.7942851	-54.29957754	-97.08050215	EMD2
CTCCGATCATGCAGCC-1	singlet	0	-122.617242	-344.0330271	-298.0662322	EMD2

CTCCGATGTAGCTTGT-1	singlet	1	-732.1699732	-210.7161916	-662.1098838	EMD2
CTCCGATGTCAACCAT-1	singlet	2	-2009.712505	-1776.230501	-789.924557	EMD2
CTCCGATGTTATCCAG-1	singlet	1	-2605.346043	-751.983635	-1895.58955	EMD2
CTCCGATGTTGTCCCT-1	singlet	1	-972.1517883	-340.6969815	-807.0197574	EMD2
CTCCGATTCAGCATTG-1	singlet	2	-2191.447186	-1695.059549	-727.9158136	EMD2
CTCCGATTCCACGTGG-1	singlet	2	-677.4297974	-524.9951417	-241.351833	EMD2
CTCCGATTCCTTTAGT-1	singlet	2	-2409.698098	-2137.357932	-878.2360534	EMD2
CTCCGATTCTGACAGT-1	unassigned	1	-0.853607542	-0.851633337	-0.856930988	EMD2
CTCCTCCAGTTGAAGT-1	singlet	0	-180.482334	-561.1966507	-523.0477181	EMD2
CTCCTCCAGTTGTCAC-1	doublet	0/2	-2397.664397	-3998.10654	-2699.185319	EMD2
CTCCTCCCAATCTCTT-1	singlet	1	-1254.157372	-506.0187498	-1129.125411	EMD2
CTCCTCCGTCGTGTTA-1	singlet	1	-844.0168222	-260.1143713	-717.7271416	EMD2
CTCCTCCGTTTAGACC-1	singlet	1	-1312.250676	-491.4373837	-1166.244091	EMD2
CTCCTCCGTTTCGTTT-1	singlet	2	-726.6089137	-691.152935	-305.0847643	EMD2
CTCCTCCTCCTTGACC-1	singlet	0	-364.3657516	-1151.730284	-1073.050444	EMD2
CTCCTCCTCGTCCTTG-1	singlet	1	-3280.076808	-1117.798871	-2896.725174	EMD2
CTCCTCCTCTCATGCC-1	singlet	0	-894.4398294	-2054.343312	-2158.920791	EMD2
CTCCTTTAGCTGACTT-1	singlet	1	-2113.45103	-802.3987762	-1925.137145	EMD2
CTCCTTTAGTCTCCTC-1	singlet	1	-340.2711057	-137.5546174	-323.962811	EMD2
CTCCTTTAGTGATCGG-1	singlet	1	-3393.276935	-1085.035174	-2716.147407	EMD2
CTCCTTTCAAAGGAGA-1	singlet	1	-2433.564347	-839.6446006	-2027.929734	EMD2
CTCCTTTCAAGGAGTC-1	singlet	2	-2672.276587	-2555.165227	-996.5107957	EMD2
CTCCTTTCACTTGGCG-1	singlet	1	-1227.73736	-515.2093456	-998.8871462	EMD2
CTCCTTTGTAACGCGA-1	singlet	1	-566.8434414	-215.4350236	-463.9967859	EMD2
CTCCTTTGTAGCTTTG-1	singlet	1	-2739.91738	-860.5561079	-2267.867926	EMD2
CTCCTTTGTGCCGTTG-1	singlet	1	-1766.776756	-626.227476	-1398.512209	EMD2
CTCCTTTGTGCTTCAA-1	singlet	2	-693.1136415	-597.2350247	-203.7398499	EMD2
CTCCTTTTCAGCCTCT-1	singlet	2	-1078.494358	-999.4415764	-349.1460493	EMD2
CTCCTTTTCCACGGGT-1	singlet	1	-4215.402184	-1403.12445	-3419.100162	EMD2
CTCCTTTTCCCAGCGA-1	singlet	1	-980.4854599	-297.9649443	-686.8762266	EMD2
CTCGAGGAGAACTTCC-1	singlet	1	-2262.692	-684.5168722	-1754.641378	EMD2
CTCGAGGAGGGTCACA-1	singlet	2	-2083.428493	-1904.15222	-765.7305107	EMD2
CTCGAGGAGTCCTGTA-1	singlet	1	-1951.859289	-528.2662979	-1460.360371	EMD2
CTCGAGGCACACTTAG-1	singlet	1	-1078.957537	-351.0092399	-832.5003754	EMD2
CTCGAGGCAGCATACT-1	singlet	2	-3587.932402	-3026.06467	-1205.951577	EMD2
CTCGAGGCAGTCCCGA-1	singlet	0	-134.494957	-407.7292319	-353.4690319	EMD2
CTCGAGGGTATGGTAA-1	singlet	2	-666.787386	-530.0877596	-182.796428	EMD2
CTCGAGGGTGGCTAGA-1	singlet	2	-1483.859688	-1126.451398	-504.1016605	EMD2
CTCGAGGGTTGTTGTG-1	doublet	45323	-6716.882152	-4154.135241	-3310.751214	EMD2
CTCGAGGGTTTATGCG-1	singlet	1	-288.9208546	-106.5355319	-243.8907956	EMD2
CTCGAGGTCTGCAGCG-1	singlet	2	-170.2570233	-203.8729513	-73.28414506	EMD2
CTCGAGGTCTGGGCAC-1	singlet	1	-2289.055704	-750.7133348	-1837.490837	EMD2
CTCTCAGAGCGTGAGT-1	singlet	2	-3039.579051	-2364.00269	-862.3510027	EMD2
CTCTCAGAGCGTTACT-1	singlet	1	-1167.331665	-353.1136825	-964.5169826	EMD2
CTCTCAGAGCTCGTGC-1	singlet	1	-2025.971722	-553.3920274	-1682.597707	EMD2
CTCTCAGAGGTATCTC-1	singlet	2	-1060.723849	-938.6390217	-376.7002722	EMD2
CTCTCAGCAGAATCGG-1	singlet	1	-977.4757867	-326.4695439	-747.4113648	EMD2
CTCTCAGCAGCGTGAA-1	singlet	1	-414.5496061	-119.5005986	-304.2245723	EMD2
CTCTCAGCAGTTGCGC-1	singlet	2	-865.3834166	-840.3282024	-321.5262265	EMD2
CTCTCAGGTAGGCTCC-1	singlet	0	-133.6770188	-483.1364772	-408.8146329	EMD2
CTCTCAGGTCACTAGT-1	singlet	2	-1991.649933	-1875.094725	-538.1978574	EMD2
CTCTCAGGTGATACCT-1	singlet	1	-1492.722641	-433.6564064	-1035.208372	EMD2
CTCTCAGTCTCGACCT-1	singlet	1	-2390.559983	-902.0795853	-2072.597464	EMD2
CTCTCGAAGCAGTAAT-1	singlet	1	-3013.577428	-780.549442	-2515.920218	EMD2
CTCTCGACAAACAGGC-1	singlet	1	-5595.522851	-1455.315173	-4484.244232	EMD2
CTCTCGACAACCAACT-1	singlet	2	-4886.183072	-3728.529631	-1383.93249	EMD2
CTCTCGACAGAGCTAG-1	singlet	2	-1549.458372	-1183.153137	-537.6575381	EMD2
CTCTCGACATAGACTC-1	singlet	2	-3826.152749	-2964.546702	-1295.460128	EMD2
CTCTCGACATATAGCC-1	singlet	0	-24.70456159	-75.43615081	-96.43222122	EMD2
CTCTCGACATGACACT-1	singlet	1	-1911.962251	-875.4460544	-1794.802371	EMD2
CTCTCGAGTCAACGCC-1	singlet	1	-371.2821859	-143.4962244	-318.4910556	EMD2
CTCTCGAGTTTGGAAA-1	singlet	1	-1908.061798	-589.1875977	-1752.311174	EMD2
CTCTCGATCCTTCTAA-1	singlet	0	-481.9978675	-1261.29045	-1230.337917	EMD2
CTCTCGATCTCTAGGA-1	singlet	0	-1186.113102	-3263.640258	-3371.318838	EMD2
CTCTCGATCTGTTCAT-1	singlet	1	-3277.846071	-986.0596405	-2851.230097	EMD2
CTCTGGTAGCGAGGAG-1	singlet	0	-88.18740035	-233.6913149	-220.1936241	EMD2
CTCTGGTAGGTAATCA-1	singlet	1	-1314.113184	-439.4516108	-1219.005386	EMD2
CTCTGGTAGGTTGCCC-1	singlet	0	-237.5355147	-544.76684	-623.2152719	EMD2
CTCTGGTAGTGTCATC-1	singlet	2	-615.8527649	-639.7960856	-221.1101722	EMD2
CTCTGGTCAACCGTAT-1	singlet	0	-1206.696886	-3675.500939	-3366.026081	EMD2
CTCTGGTCATCTCAAG-1	singlet	2	-966.2091535	-792.0530477	-344.7333298	EMD2
CTCTGGTCATGTCAGT-1	singlet	1	-101.6508832	-38.7241098	-110.3895788	EMD2
CTCTGGTGTAACGTTC-1	singlet	0	-58.82371212	-200.6076256	-141.5458419	EMD2
CTCTGGTGTCAACCTA-1	singlet	1	-680.1768126	-229.4432416	-601.7252796	EMD2
CTCTGGTGTGTGTCGC-1	singlet	1	-871.9832047	-288.0228247	-703.8293456	EMD2
CTCTGGTGTTGAATCC-1	singlet	1	-243.7958566	-141.2514072	-235.4502883	EMD2
CTCTGGTTCAATCAGC-1	singlet	0	-1053.669063	-4336.763361	-4402.41286	EMD2
CTCTGGTTCTGGCCGA-1	singlet	0	-192.927166	-503.5795658	-400.2074601	EMD2
CTGAATGAGCAACAAT-1	singlet	1	-747.2313983	-240.1180842	-576.0777616	EMD2
CTGAATGAGGTTCCAT-1	singlet	2	-1152.377753	-974.5151177	-454.9603903	EMD2
CTGAATGGTCCACACG-1	singlet	1	-1474.494542	-523.5025973	-1162.710632	EMD2
CTGAATGGTTACGATC-1	singlet	0	-508.9813647	-1530.81625	-1389.265862	EMD2
CTGAATGGTTCTCACC-1	singlet	0	-330.2282972	-1095.324096	-989.6273701	EMD2
CTGAATGGTTTCGGCG-1	singlet	0	-27.98246886	-101.7176919	-89.01689443	EMD2
CTGAATGTCACCATAG-1	singlet	1	-3907.889395	-1217.222003	-2972.416208	EMD2
CTGAATGTCCAACTGA-1	singlet	1	-2451.39791	-844.9010056	-2082.066377	EMD2
CTGAATGTCGCAGTGC-1	singlet	1	-3026.153943	-916.7532409	-2243.767479	EMD2
CTGAATGTCGCCTTTG-1	singlet	0	-672.7037662	-2261.968781	-1883.736856	EMD2
CTGAATGTCGTTAGTG-1	singlet	2	-5323.62554	-4267.113307	-1835.953638	EMD2
CTGAATGTCTCCTGCA-1	singlet	2	-606.4107391	-569.9108327	-240.9656723	EMD2
CTGAGCGAGAGAGTTT-1	singlet	1	-3455.284927	-1017.555891	-2899.810787	EMD2
CTGAGCGAGCGTCTGC-1	singlet	0	-81.624727	-189.6216019	-245.5599023	EMD2
CTGAGCGAGGACAGCT-1	singlet	1	-456.142902	-123.6143317	-305.777231	EMD2
CTGAGCGAGTATGGCG-1	singlet	1	-1446.679974	-506.5698921	-1306.822559	EMD2
CTGAGCGCATACAGGG-1	singlet	0	-198.5840045	-392.9311189	-397.6387609	EMD2
CTGAGCGGTAACGGTG-1	singlet	0	-739.5759112	-2007.965144	-2026.839185	EMD2
CTGAGCGGTTAGTCGT-1	singlet	1	-3239.493164	-912.4209559	-2690.961434	EMD2
CTGAGCGGTTGTGGCC-1	singlet	0	-788.5419833	-2841.902485	-2672.107434	EMD2
CTGAGGCAGCCTCAAT-1	singlet	0	-484.3185163	-1379.301173	-1128.535771	EMD2
CTGAGGCAGCGGATCA-1	singlet	1	-425.0006075	-135.9645372	-306.8160141	EMD2
CTGAGGCAGCTGCGAA-1	singlet	0	-324.1007517	-962.4723425	-894.8656986	EMD2
CTGAGGCAGGCTTTCA-1	singlet	0	-1011.832347	-4014.684614	-3561.756028	EMD2
CTGAGGCCAACAGCCC-1	singlet	1	-180.1708096	-64.61625792	-135.8195096	EMD2
CTGAGGCCACTGGATT-1	singlet	1	-1408.789774	-456.8484801	-1112.630457	EMD2
CTGAGGCCAGATAAAC-1	doublet	1/0	-3100.727306	-1507.299799	-3245.420733	EMD2
CTGAGGCCAGATCCTA-1	singlet	1	-3253.596833	-998.1753882	-2398.596734	EMD2
CTGAGGCCATACCATG-1	singlet	1	-1511.859936	-517.8750355	-1234.442902	EMD2
CTGAGGCGTAGAGATT-1	singlet	1	-372.4259401	-113.8753375	-287.8417909	EMD2
CTGAGGCGTCTCTCCA-1	singlet	1	-444.0724399	-177.5481327	-374.6944962	EMD2
CTGAGGCTCAAGAAAC-1	singlet	1	-2195.754298	-679.8257482	-1913.607239	EMD2

CTGAGGCTCATCTACT-1	singlet	1	-1255.718727	-323.9502572	-806.7947315	EMD2
CTGAGGCTCCATGATG-1	singlet	1	-2967.995906	-665.5038354	-1977.754415	EMD2
CTGAGGCTCGACCTAA-1	singlet	2	-2660.19507	-1998.091434	-820.6428245	EMD2
CTGATCCAGACAGCGT-1	singlet	0	-1021.315983	-2996.839188	-2912.805464	EMD2
CTGATCCAGATCCCAT-1	singlet	1	-520.479751	-245.485861	-471.9777605	EMD2
CTGATCCAGATGACAT-1	singlet	1	-110.3808839	-39.3364517	-75.33825565	EMD2
CTGATCCAGCGTGAAC-1	singlet	1	-772.9590794	-260.9083829	-596.3363093	EMD2
CTGATCCAGTAAACTG-1	singlet	0	-984.8362941	-3195.466828	-3096.979932	EMD2
CTGATCCAGTCTTCCC-1	singlet	0	-68.01779539	-172.0587469	-163.6256076	EMD2
CTGATCCAGTTGCATC-1	singlet	1	-2841.467165	-756.8233925	-2226.265527	EMD2
CTGATCCCAAATCAAG-1	singlet	1	-738.5976388	-318.5091894	-607.8295372	EMD2
CTGATCCCAATGGCCC-1	singlet	0	-128.6993195	-483.9428685	-527.9742859	EMD2
CTGATCCCAGGTGGAT-1	singlet	1	-454.599917	-191.5406955	-411.5284881	EMD2
CTGATCCTCAATCGGT-1	singlet	1	-427.7658454	-157.905726	-366.1108807	EMD2
CTGCAGGAGATTGAGT-1	singlet	1	-2749.646269	-947.706957	-2480.287017	EMD2
CTGCAGGAGTGCAGGT-1	singlet	2	-2698.609503	-2598.957847	-1318.644007	EMD2
CTGCAGGCAGCTTTGA-1	singlet	1	-2537.261583	-778.7937476	-2235.276917	EMD2
CTGCAGGGTCGCAACC-1	singlet	1	-760.1347182	-294.7136775	-601.9174008	EMD2
CTGCAGGGTGGTTTAC-1	singlet	1	-1910.373739	-637.8500058	-1753.939747	EMD2
CTGCAGGGTGTCATGT-1	singlet	1	-240.031283	-111.7611046	-221.3122797	EMD2
CTGCAGGTCAACGTGT-1	singlet	0	-1698.269711	-5664.847317	-5755.960633	EMD2
CTGCAGGTCCCATAGA-1	doublet	1/0	-280.7136188	-146.817399	-262.3593778	EMD2
CTGCATCAGAAGCCTG-1	singlet	1	-1396.189676	-456.560143	-1213.998718	EMD2
CTGCATCAGATAGTGT-1	singlet	1	-1183.398173	-343.1614263	-987.9234563	EMD2
CTGCATCAGCTCTATG-1	singlet	1	-119.4643266	-37.81926118	-100.956627	EMD2
CTGCATCAGGTACAAT-1	doublet	45293	-2552.045558	-1580.115135	-1616.05287	EMD2
CTGCATCAGTTCCAGT-1	singlet	2	-252.3525455	-255.5028785	-137.1451952	EMD2
CTGCATCCAACCGTGC-1	singlet	1	-3370.572035	-952.5180241	-2334.363052	EMD2
CTGCATCCAAGTCATC-1	singlet	0	-17.70148838	-80.39891028	-46.62909169	EMD2
CTGCATCCACAGCTGC-1	singlet	1	-798.131552	-290.6353301	-644.3192159	EMD2
CTGCATCCAGCGGTTC-1	singlet	2	-1046.759013	-1025.566237	-405.3069092	EMD2
CTGCATCGTAATGCGG-1	singlet	1	-13.28233006	-6.543243653	-11.25263071	EMD2
CTGCATCGTATGATCC-1	singlet	1	-687.4580356	-248.3572192	-585.0279775	EMD2
CTGCATCGTCGGTGAA-1	singlet	1	-1022.110229	-324.3296937	-852.2533777	EMD2
CTGCATCTCCATCCGT-1	singlet	1	-1305.979426	-520.7261	-1166.984098	EMD2
CTGCCATAGAATAACC-1	doublet	45323	-8974.109274	-5357.138561	-4034.097813	EMD2
CTGCCATAGACTCATC-1	singlet	2	-6697.474276	-5609.761876	-1945.170525	EMD2
CTGCCATAGCGAGTAC-1	singlet	1	-870.1035696	-356.0494402	-792.4017208	EMD2
CTGCCATAGGGACACT-1	singlet	2	-1718.076303	-1307.408109	-543.2164522	EMD2
CTGCCATCAAACGGCA-1	singlet	0	-210.0907768	-597.8781085	-548.4499402	EMD2
CTGCCATCATTGCTTT-1	singlet	1	-1088.139949	-359.420646	-900.9107281	EMD2
CTGCCATGTATGCGGA-1	singlet	1	-1068.840509	-323.8852161	-827.7578914	EMD2
CTGCCATGTGATCATC-1	singlet	0	-1864.662156	-6643.837832	-6147.363984	EMD2
CTGCCATGTTCGGTAT-1	singlet	1	-838.4925569	-269.2121303	-562.4460768	EMD2
CTGCCATGTTCTCACC-1	singlet	1	-309.5276089	-108.9257371	-232.2603584	EMD2
CTGCCATTCCGAGGCT-1	singlet	0	-895.0137826	-3522.539767	-3161.007108	EMD2
CTGCCATTCGACCACG-1	singlet	2	-1133.714858	-1045.884002	-447.9125976	EMD2
CTGCCATTCGCTGCGA-1	singlet	1	-1172.506844	-351.7492972	-998.2795638	EMD2
CTGCCTAAGCAATTAG-1	singlet	1	-80.10391375	-24.67854921	-76.7928308	EMD2
CTGCCTAAGCACGGAT-1	singlet	0	-816.4309111	-2630.58089	-2530.50072	EMD2
CTGCCTAAGCGGTAGT-1	doublet	1/0	-3537.673157	-3369.200823	-4710.403061	EMD2
CTGCCTAAGGTCCTGC-1	singlet	1	-1372.289232	-388.5685754	-1172.152739	EMD2
CTGCCTAAGTTGGAAT-1	singlet	1	-3501.191067	-984.7058208	-2988.588238	EMD2
CTGCCTACACAATGAA-1	singlet	1	-1367.466921	-249.9051352	-818.0863626	EMD2
CTGCCTACAGCGAGTA-1	singlet	1	-105.5541832	-36.52787399	-99.85243061	EMD2
CTGCCTACAGTGGCTC-1	singlet	1	-2432.053326	-820.347907	-1919.336767	EMD2
CTGCCTAGTATCTCGA-1	doublet	0/1	-6014.320999	-9746.930668	-11026.15777	EMD2
CTGCCTAGTCCCTGTT-1	singlet	1	-1832.308861	-610.2244342	-1625.179136	EMD2
CTGCCTAGTGTTCAGT-1	singlet	1	-1721.690695	-478.1676299	-1282.61075	EMD2
CTGCCTATCTATTGTC-1	singlet	1	-2239.280988	-688.2553718	-1766.117507	EMD2
CTGCCTATCTCATAGG-1	singlet	0	-70.56374835	-183.4126493	-192.0804754	EMD2
CTGCCTATCTGAGATC-1	singlet	0	-95.21244629	-229.2805236	-213.8610908	EMD2
CTGCGAGAGACCTCAT-1	singlet	1	-2068.193242	-643.7753729	-1748.509863	EMD2
CTGCGAGAGACGAAGA-1	singlet	1	-1246.502323	-348.2912519	-891.5369465	EMD2
CTGCGAGAGAGAGCGG-1	singlet	1	-479.3667488	-144.2291141	-342.3244444	EMD2
CTGCGAGAGTATTAGG-1	singlet	2	-1543.88304	-1512.146801	-480.7310958	EMD2
CTGCGAGAGTCCTGTA-1	singlet	1	-2011.746021	-619.3043664	-1579.219262	EMD2
CTGCGAGCAGTCAGCC-1	singlet	0	-1886.673809	-5695.121094	-5424.706568	EMD2
CTGCGAGCATGGGTCC-1	singlet	1	-3493.766847	-1004.136787	-2567.815474	EMD2
CTGCGAGGTAGCTCGC-1	singlet	1	-5379.598794	-1695.32145	-4224.780459	EMD2
CTGCGAGGTAGGCAGT-1	singlet	1	-333.0769219	-125.5774593	-271.3261264	EMD2
CTGCGAGTCAACGCTA-1	singlet	1	-195.7935332	-56.80681828	-147.8346075	EMD2
CTGCGAGTCAACTACG-1	singlet	2	-112.3233373	-72.36921616	-39.299509	EMD2
CTGCGAGTCTTCCTAA-1	singlet	1	-162.955378	-67.54405306	-122.5866354	EMD2
CTGCTCAAGAGCAGTC-1	singlet	1	-792.7781125	-256.1073653	-585.6825375	EMD2
CTGCTCAAGATTTGCC-1	singlet	1	-439.5429413	-156.8652039	-338.0424073	EMD2
CTGCTCAAGGATATGT-1	singlet	2	-5483.795042	-4690.13129	-2042.510989	EMD2
CTGCTCAAGGTACCTT-1	singlet	1	-1772.757812	-504.4191935	-1373.966357	EMD2
CTGCTCACAGGTGAGT-1	singlet	1	-2819.884248	-858.4627907	-2203.466673	EMD2
CTGCTCACAGGTTTAC-1	singlet	1	-190.0293325	-53.9998658	-144.4260611	EMD2
CTGCTCACATTGCTTT-1	singlet	1	-285.9644231	-89.94659265	-240.171683	EMD2
CTGCTCAGTACACGCC-1	singlet	1	-1399.537308	-489.6686315	-1169.029757	EMD2
CTGCTCAGTATGGTTC-1	singlet	1	-59.23253626	-22.57267297	-55.17540706	EMD2
CTGCTCAGTGCAGGAT-1	singlet	2	-1335.258697	-939.3760179	-398.4233091	EMD2
CTGCTCATCACCGGTG-1	singlet	1	-852.4717244	-333.2198018	-639.7335179	EMD2
CTGCTCATCGCAGTTA-1	singlet	2	-1498.06337	-1614.5555	-352.4707735	EMD2
CTGCTCATCGGACGTC-1	singlet	2	-1432.953486	-1220.730401	-555.2041144	EMD2
CTGGACGAGACGGAAA-1	singlet	1	-1087.955423	-364.2111386	-905.9308379	EMD2
CTGGACGAGGGTACGT-1	singlet	1	-497.1201726	-179.6062406	-384.0605011	EMD2
CTGGACGCAATCCTTT-1	singlet	1	-2550.835691	-865.2742346	-2315.370874	EMD2
CTGGACGCACAGCCTG-1	singlet	2	-983.3030066	-788.9608966	-365.2985727	EMD2
CTGGACGCACGCGTCA-1	singlet	2	-2768.386349	-2126.746942	-901.2303191	EMD2
CTGGACGCACGTACTA-1	singlet	0	-1976.903227	-6986.475752	-6540.276071	EMD2
CTGGACGCAGAGTGTG-1	singlet	1	-489.5095807	-161.0407202	-382.9699831	EMD2
CTGGACGCAGGCACTC-1	singlet	0	-293.1194578	-922.7866598	-848.6702571	EMD2
CTGGACGGTAGTCACT-1	singlet	0	-770.3096401	-2732.668057	-2598.056776	EMD2
CTGGACGGTTCGCGTG-1	singlet	1	-416.0956217	-125.09741	-369.0802127	EMD2
CTGGACGTCAGGAAGC-1	singlet	1	-786.2679042	-264.4417928	-609.9694869	EMD2
CTGGACGTCGTTATCT-1	singlet	2	-1013.797017	-893.3342263	-253.778245	EMD2
CTGGACGTCTCACGAA-1	singlet	2	-1287.345535	-1447.001587	-450.8967915	EMD2
CTGGCAGAGAGTGTGC-1	singlet	1	-174.986265	-77.2123002	-174.3704902	EMD2
CTGGCAGAGCTCTTCC-1	singlet	1	-272.7711868	-121.2329383	-256.1082025	EMD2
CTGGCAGAGTAAGGGA-1	singlet	1	-1149.453403	-304.0516302	-994.2667308	EMD2
CTGGCAGCAAGACGAC-1	singlet	1	-464.0241139	-192.4620143	-443.9305766	EMD2
CTGGCAGCAGGTCAAG-1	singlet	0	-280.7181342	-905.0433986	-758.8086137	EMD2
CTGGCAGGTTTCCAAG-1	singlet	0	-240.499209	-901.4816502	-803.0291228	EMD2
CTGGCAGTCCGAGGCT-1	singlet	0	-507.7514224	-1570.213861	-1643.407948	EMD2
CTGGCAGTCTAGAGCT-1	unassigned	0	-8.558152587	-10.10352973	-11.62584333	EMD2
CTGGTCTAGGTTCACT-1	singlet	1	-2364.738593	-790.7762405	-2006.736681	EMD2
CTGGTCTCAATCAAGA-1	singlet	1	-1612.468486	-525.6561291	-1204.418182	EMD2

CTGGTCTCACAACGCC-1	doublet	0/1	-2673.607722	-3281.921649	-4182.219041	EMD2
CTGGTCTCACTCCACT-1	singlet	1	-708.2684171	-250.8611821	-515.9917702	EMD2
CTGGTCTCACTCCTGT-1	singlet	2	-1880.775146	-1482.661869	-722.0679208	EMD2
CTGGTCTCAGTCACGC-1	singlet	1	-1185.647301	-448.255842	-1007.923947	EMD2
CTGGTCTCATCCGTTC-1	singlet	1	-2001.675693	-596.0297073	-1673.658503	EMD2
CTGGTCTCATCCTATT-1	singlet	1	-344.6308582	-122.5631461	-247.2664883	EMD2
CTGGTCTGTCGTCAGC-1	singlet	2	-2034.886534	-1756.317543	-644.1950147	EMD2
CTGGTCTGTCGTGTTA-1	singlet	1	-1728.957814	-593.0439757	-1315.568234	EMD2
CTGGTCTGTGTACGCC-1	singlet	1	-3667.597311	-1124.462929	-2980.318338	EMD2
CTGGTCTTCCGCTTAC-1	singlet	1	-564.9670228	-213.9945818	-509.0613317	EMD2
CTGGTCTTCGAGATGG-1	singlet	1	-998.4394561	-343.2680587	-896.2158134	EMD2
CTGGTCTTCTCCGTGT-1	singlet	2	-1228.527536	-921.6120756	-395.8609797	EMD2
CTGTACCAGCATTGTC-1	singlet	1	-3855.61697	-1261.633998	-3039.304569	EMD2
CTGTACCAGTGCACTT-1	singlet	1	-3904.607363	-1215.211312	-3256.781401	EMD2
CTGTACCCACCGGTCA-1	singlet	2	-2171.409441	-2086.671569	-654.4073749	EMD2
CTGTACCCAGAAGTTA-1	singlet	1	-3524.480288	-1102.309257	-3030.056389	EMD2
CTGTACCGTATCAGCT-1	singlet	0	-411.9981661	-1530.702507	-1450.176385	EMD2
CTGTACCTCCAAGCAT-1	singlet	0	-484.0762753	-1389.901839	-1469.031233	EMD2
CTGTACCTCGACCTAA-1	doublet	45293	-6032.346045	-2679.637298	-3555.164807	EMD2
CTGTACCTCGGTTAGT-1	singlet	1	-516.4397052	-177.4979884	-481.7086043	EMD2
CTGTACCTCTACCCAC-1	singlet	1	-1565.912127	-483.1396292	-1170.619688	EMD2
CTGTACCTCTAGACCA-1	singlet	1	-2100.237961	-746.5023471	-1729.697508	EMD2
CTGTACCTCTCTCCGA-1	singlet	2	-5516.741615	-4795.601555	-1746.727242	EMD2
CTGTACCTCTGATGGT-1	singlet	2	-274.7857025	-268.0817674	-77.83985795	EMD2
CTGTACCTCTTTCCAA-1	singlet	1	-517.6698499	-204.170702	-372.8196618	EMD2
CTGTAGAAGCACCTGC-1	singlet	1	-996.7514419	-304.1957097	-725.1146753	EMD2
CTGTAGAAGCCATCCG-1	singlet	0	-383.2333144	-1138.495822	-1122.080827	EMD2
CTGTAGAAGCGCGTTC-1	singlet	1	-407.771277	-162.1733621	-322.4708689	EMD2
CTGTAGAAGGGACACT-1	singlet	2	-412.9718749	-409.0676395	-155.4778575	EMD2
CTGTAGACAGCGACAA-1	singlet	2	-1390.456005	-1330.447954	-530.3813617	EMD2
CTGTAGACAGGATCTT-1	singlet	0	-2.100457842	-11.80240121	-11.67622672	EMD2
CTGTAGATCACTACGA-1	unassigned	2	-9.491378058	-3.972727891	-2.991800019	EMD2
CTGTAGATCTACTATC-1	singlet	0	-289.2532948	-1110.319905	-902.3470457	EMD2
CTGTAGATCTAGAGCT-1	singlet	1	-1491.801648	-425.9918409	-1279.370731	EMD2
CTGTAGATCTGGGCCA-1	singlet	0	-315.0102997	-896.9238281	-844.66199	EMD2
CTGTATTAGACTACCT-1	singlet	0	-169.7151456	-596.6878901	-525.7472341	EMD2
CTGTATTAGCACTCTA-1	doublet	45293	-5099.612926	-2632.595366	-2999.462586	EMD2
CTGTATTAGCCAAGCA-1	singlet	1	-441.5141274	-175.6988191	-402.0975698	EMD2
CTGTATTAGCTCGCAC-1	singlet	1	-838.9901247	-267.9002705	-657.5580088	EMD2
CTGTATTAGGTTGAGC-1	singlet	0	-1041.968719	-3933.779336	-3734.267062	EMD2
CTGTATTCAAGATTGA-1	singlet	1	-1359.772941	-438.9929814	-1124.785797	EMD2
CTGTATTGTCCGAAAG-1	singlet	2	-836.2405057	-730.5082542	-327.1826271	EMD2
CTGTATTGTCCTGAAT-1	singlet	1	-2281.393654	-673.5364491	-1626.010376	EMD2
CTGTATTGTCGTCTCT-1	doublet	0/1	-3090.703434	-3197.816277	-4515.571692	EMD2
CTGTATTTCGATCCCT-1	singlet	1	-490.6359168	-189.8158841	-461.3089768	EMD2
CTGTCGTAGCAGCCTC-1	singlet	0	-1158.596457	-2906.443777	-3149.684211	EMD2
CTGTCGTAGGAAAGTG-1	singlet	1	-2448.433421	-735.5569154	-1883.005375	EMD2
CTGTCGTAGGAACGTC-1	singlet	0	-305.0692312	-995.6887813	-797.0221165	EMD2
CTGTCGTAGTGAGTGC-1	singlet	1	-1204.047218	-436.7343629	-1039.883564	EMD2
CTGTCGTAGTGCCAGA-1	singlet	0	-755.0592045	-2404.583629	-2085.579902	EMD2
CTGTCGTCACTTCTCG-1	singlet	0	-46.02505029	-133.0350099	-110.2916007	EMD2
CTGTCGTCAGTTGTCA-1	singlet	1	-1855.969736	-635.3811025	-1395.988409	EMD2
CTGTCGTGTGCAATGG-1	singlet	1	-98.39390819	-41.06817177	-83.21907953	EMD2
CTGTGAAAGACGCAGT-1	singlet	1	-385.0250798	-132.1892017	-372.4300178	EMD2
CTGTGAAAGGAACGCT-1	unassigned	45323	-12.85006172	-6.37119785	-3.964111917	EMD2
CTGTGAAAGGAATTAC-1	singlet	0	-185.5913764	-528.1588373	-472.0132538	EMD2
CTGTGAAAGGCATGCA-1	singlet	0	-360.2628467	-713.4682891	-733.4691382	EMD2
CTGTGAAAGGGAGAAT-1	singlet	1	-1050.111844	-394.6078166	-815.3088934	EMD2
CTGTGAACAGCTACAT-1	singlet	1	-1166.806662	-446.0536205	-924.7188474	EMD2
CTGTGAAGTGATTCAC-1	singlet	1	-2582.70279	-739.6978544	-1929.729178	EMD2
CTGTGAAGTTTCGGCG-1	singlet	1	-299.9445615	-137.5007936	-252.0714895	EMD2
CTGTGAATCCCTCAAC-1	doublet	45293	-6295.568114	-3042.605085	-3791.104658	EMD2
CTGTGAATCCTCACTG-1	singlet	1	-861.8378927	-318.6363446	-731.1051868	EMD2
CTGTGAATCTGTCCCA-1	singlet	1	-1215.598066	-416.3544019	-1020.781427	EMD2
CTGTGGGAGACGACTG-1	unassigned	2	-1.102078573	-2.759195596	-1.093761673	EMD2
CTGTGGGAGGTACCTT-1	singlet	1	-349.7956853	-122.0471147	-273.8742356	EMD2
CTGTGGGAGGTGAGAA-1	singlet	0	-68.35377636	-194.1603406	-154.1882988	EMD2
CTGTGGGCACAGACGA-1	singlet	1	-461.7091864	-188.3902305	-398.1600646	EMD2
CTGTGGGCACATGGTT-1	singlet	1	-429.5699783	-185.422229	-448.0221491	EMD2
CTGTGGGCATAACAGA-1	singlet	2	-616.7173636	-634.3939355	-286.8068132	EMD2
CTGTGGGTCATCTACT-1	singlet	1	-1577.022872	-403.0027169	-1018.656012	EMD2
CTGTGGGTCGTTCATT-1	singlet	1	-340.8337773	-142.1971368	-286.8046328	EMD2
CTTACCGAGGCTGTAG-1	singlet	0	-97.90560877	-472.6973801	-319.4810329	EMD2
CTTACCGCACAGGATG-1	singlet	1	-89.37987191	-27.92128773	-62.9295712	EMD2
CTTACCGCACCAGTTA-1	singlet	1	-326.2206798	-123.5066501	-270.6025042	EMD2
CTTACCGCAGATCATC-1	singlet	1	-301.9560766	-136.0180426	-266.4936658	EMD2
CTTACCGCATATGAAG-1	singlet	1	-4668.310846	-1274.235616	-3361.694386	EMD2
CTTACCGCATTGTACG-1	singlet	2	-496.851644	-532.1614314	-164.4493597	EMD2
CTTACCGCATTGTAGC-1	singlet	0	-818.2937859	-3036.154056	-2773.945824	EMD2
CTTACCGGTCTGATCA-1	singlet	0	-71.44305567	-185.5387279	-188.9292148	EMD2
CTTACCGGTGAAGCGT-1	singlet	1	-1118.462008	-359.1071923	-967.3394363	EMD2
CTTACCGGTGTCCGGT-1	singlet	2	-704.5911385	-835.6652422	-278.3737424	EMD2
CTTACCGGTTAAGGAT-1	singlet	1	-375.1575913	-121.9282147	-232.7765183	EMD2
CTTACCGTCCTGGCTT-1	singlet	0	-952.0974014	-2876.463149	-2719.266523	EMD2
CTTAGGAAGCGCCTTG-1	singlet	1	-2079.779174	-732.3314067	-1701.818407	EMD2
CTTAGGAAGCTACAAA-1	singlet	1	-3605.758292	-1130.858153	-3205.854101	EMD2
CTTAGGAAGGAAAGGT-1	singlet	1	-2653.230768	-967.4816573	-2167.393553	EMD2
CTTAGGAAGTAGTCTC-1	singlet	1	-84.71137126	-28.15399434	-53.25139976	EMD2
CTTAGGACAGAAATCA-1	singlet	2	-284.3899659	-281.205508	-115.7492975	EMD2
CTTAGGACATAAGCGG-1	singlet	0	-728.3612013	-2048.831518	-2039.512207	EMD2
CTTAGGACATCATTGG-1	singlet	1	-475.2305964	-159.6646845	-349.232405	EMD2
CTTAGGAGTCCAAGAG-1	singlet	0	-734.6598771	-2420.971146	-2179.136316	EMD2
CTTAGGATCCATAGAC-1	singlet	1	-2395.623299	-790.8853866	-1809.096531	EMD2
CTTAGGATCCCTATTA-1	singlet	2	-3219.784181	-2491.976232	-1119.41211	EMD2
CTTAGGATCCGTTGGG-1	singlet	1	-207.5940062	-56.43438921	-166.9906448	EMD2
CTTAGGATCGTAATGC-1	singlet	1	-1992.556612	-639.7777322	-1669.928948	EMD2
CTTCAATAGCTATCTG-1	singlet	1	-2234.413351	-671.6325146	-1943.579487	EMD2
CTTCAATAGGCAGGTT-1	singlet	0	-851.0303395	-2298.685823	-2275.008385	EMD2
CTTCAATAGGGACCAT-1	singlet	2	-573.8075641	-502.3836055	-215.3221789	EMD2
CTTCAATAGGTCGTGA-1	singlet	2	-967.6619908	-814.3949337	-392.6458215	EMD2
CTTCAATAGTCCGCGT-1	singlet	1	-358.7555618	-143.10095	-288.3416443	EMD2
CTTCAATCAACATCGT-1	doublet	0/1	-366.4285444	-395.9079486	-517.5791198	EMD2
CTTCAATCAACTGAAA-1	singlet	1	-7417.525703	-2341.524992	-5933.981484	EMD2
CTTCAATCAGATACCT-1	singlet	0	-769.2156037	-2254.579069	-2227.012781	EMD2
CTTCAATCAGGAATCG-1	singlet	2	-619.6674865	-631.6941752	-205.6880968	EMD2
CTTCAATCAGGCGAAT-1	singlet	0	-786.1742173	-2537.113921	-2256.866299	EMD2
CTTCAATGTATCACCA-1	singlet	1	-747.4012356	-282.7783229	-600.0126498	EMD2
CTTCAATTCCACAGCG-1	doublet	45293	-1270.753273	-760.0527896	-865.959914	EMD2
CTTCAATTCCCTTGTG-1	singlet	0	-369.0693144	-925.2296631	-843.0543113	EMD2
CTTCAATTCTGGGCGT-1	singlet	0	-344.8468984	-994.7279799	-923.3249685	EMD2

CTTCAATTCTTCCTAA-1	singlet	1	-4888.166259	-1669.31784	-4138.391658	EMD2
CTTCCGACACAAACGG-1	singlet	1	-120.0121016	-62.48312859	-139.8381224	EMD2
CTTCCGACATACAGCT-1	singlet	0	-1964.348206	-5967.751846	-5678.841455	EMD2
CTTCCGAGTAACGGTG-1	singlet	1	-435.0552053	-200.9060834	-378.3425537	EMD2
CTTCCGAGTGAACCGA-1	singlet	1	-297.8618874	-120.272452	-257.2764094	EMD2
CTTCCGATCCGTGGTG-1	singlet	2	-208.6261014	-265.7291203	-117.550428	EMD2
CTTCCTTAGATGGTCG-1	singlet	2	-180.5228739	-173.0988081	-69.66333745	EMD2
CTTCCTTCAATGTGGG-1	singlet	1	-270.2611626	-104.3207615	-204.3804021	EMD2
CTTCCTTCACCAAATC-1	singlet	0	-198.585087	-518.0251318	-405.3549493	EMD2
CTTCCTTCAGGTACGA-1	singlet	0	-443.4818944	-1274.132841	-1365.032948	EMD2
CTTCCTTCATCCGATA-1	singlet	1	-296.6148458	-109.5022525	-226.0676467	EMD2
CTTCCTTCATTGACAC-1	singlet	1	-5875.052954	-1793.190126	-4725.290978	EMD2
CTTCCTTGTCGTCAGC-1	singlet	2	-259.7690281	-241.5411008	-118.748782	EMD2
CTTCCTTGTCTCTCTG-1	singlet	1	-3792.8989	-959.9219499	-2838.110514	EMD2
CTTCCTTGTGGTATGG-1	singlet	1	-354.8000701	-130.045834	-300.4375178	EMD2
CTTCCTTGTTGGATCT-1	singlet	2	-3311.810024	-2626.691915	-840.7622501	EMD2
CTTCCTTTCTCTCGAC-1	singlet	1	-380.8772581	-147.061695	-363.8676878	EMD2
CTTCGGTCACATACGT-1	singlet	1	-3385.50608	-962.2687838	-2652.404462	EMD2
CTTCGGTCATCTGTTT-1	singlet	1	-234.2438814	-87.35276258	-186.2915094	EMD2
CTTCGGTGTTGTTGCA-1	singlet	1	-731.4822139	-278.7575823	-666.7720478	EMD2
CTTCGGTTCGCTAAAC-1	singlet	0	-246.4914238	-772.7266698	-712.8633299	EMD2
CTTCTAAAGGAAAGGT-1	singlet	2	-1228.518989	-1359.194826	-445.1691205	EMD2
CTTCTAACAGCCGTTG-1	singlet	2	-88.28528729	-86.56363854	-23.39951136	EMD2
CTTCTAACATTCCTAT-1	singlet	0	-88.01360418	-241.3357993	-230.7964325	EMD2
CTTCTAAGTGTTCGAT-1	singlet	1	-1516.798382	-476.5399858	-1090.202957	EMD2
CTTCTCTAGGAGCTGT-1	singlet	2	-1156.621477	-1100.887652	-451.0718411	EMD2
CTTCTCTAGGCAGCTA-1	singlet	0	-637.4812636	-1782.133296	-1619.869564	EMD2
CTTCTCTAGTTCATGC-1	singlet	1	-1312.71136	-462.6086313	-1098.920606	EMD2
CTTCTCTCAGGTGAGT-1	singlet	1	-527.9670448	-198.8497163	-452.7952811	EMD2
CTTCTCTGTGTTACTG-1	singlet	1	-448.6376742	-154.8336684	-312.7704967	EMD2
CTTCTCTTCGGCATAT-1	singlet	2	-557.3538587	-485.4932012	-226.6969681	EMD2
CTTGAGAAGAGAGCAA-1	singlet	0	-401.6663207	-1260.954702	-1078.619701	EMD2
CTTGAGAAGGACAGTC-1	singlet	1	-1890.27068	-537.7679502	-1697.58024	EMD2
CTTGAGAAGTGACCTT-1	singlet	1	-192.418151	-67.60641427	-145.3287442	EMD2
CTTGAGACAACCTAAC-1	singlet	2	-2258.058946	-2134.564293	-787.5129497	EMD2
CTTGAGACAGTCGCAC-1	doublet	45323	-160.8277513	-112.3954255	-110.8242023	EMD2
CTTGAGACATCTGCGG-1	singlet	1	-768.5373037	-226.0864663	-517.8308959	EMD2
CTTGAGAGTATCTCTT-1	singlet	1	-1942.163133	-604.73598	-1682.731718	EMD2
CTTGAGATCTTCTGTA-1	singlet	1	-1280.36031	-464.3999472	-1024.760626	EMD2
CTTGATTAGTAAACGT-1	singlet	1	-291.6625262	-115.8331363	-276.2338459	EMD2
CTTGATTCAATGTTGC-1	singlet	1	-486.8546108	-159.8233391	-376.1006629	EMD2
CTTGATTCAGGCCTGT-1	singlet	1	-421.7458926	-133.9848854	-316.2422949	EMD2
CTTGATTCAGTCTCTC-1	singlet	1	-109.525402	-42.07698139	-104.3807494	EMD2
CTTGATTCATCAGCAT-1	unassigned	0/2	-16.86063979	-34.22738912	-39.83008032	EMD2
CTTGATTCATCGATAC-1	singlet	1	-1992.864846	-655.1026392	-1747.448843	EMD2
CTTGATTGTCCACATA-1	singlet	1	-3037.404541	-893.2853923	-2403.23574	EMD2
CTTGATTTCGATTGGT-1	singlet	1	-1449.348955	-523.6213246	-1182.526703	EMD2
CTTGATTTCGGCCAAC-1	singlet	1	-1891.297563	-545.524066	-1446.828834	EMD2
CTTGATTTCTATCACT-1	singlet	1	-4252.741172	-1405.856243	-3362.012399	EMD2
CTTGATTTCTCCATAT-1	singlet	1	-1674.282645	-635.7503808	-1492.559216	EMD2
CTTTCAAAGACTTGTC-1	singlet	2	-2409.121597	-2242.642491	-903.5883428	EMD2
CTTTCAAAGCAGGTCA-1	singlet	1	-982.5308234	-270.3896153	-679.1294148	EMD2
CTTTCAAAGCTTGTTG-1	singlet	0	-771.6474241	-2104.329436	-2096.115853	EMD2
CTTTCAAAGGTCGTGA-1	singlet	1	-760.5109921	-279.2302016	-669.276922	EMD2
CTTTCAAAGTCATCCA-1	singlet	0	-941.3131032	-2500.436853	-2548.42707	EMD2
CTTTCAACACTAGGCC-1	singlet	0	-8523.871927	-23096.30584	-23342.92343	EMD2
CTTTCAACAGCACAAG-1	singlet	0	-1365.610746	-5302.791628	-5151.364389	EMD2
CTTTCAACATACCAGT-1	singlet	1	-506.2242485	-234.049887	-474.189809	EMD2
CTTTCAAGTGCGCTCA-1	singlet	1	-831.2338533	-269.9309633	-641.421063	EMD2
CTTTCAAGTGTCCACG-1	singlet	1	-475.2861612	-214.4280808	-408.0205195	EMD2
CTTTCAATCGCTACAA-1	singlet	0	-398.1934297	-958.2644776	-988.2760511	EMD2
CTTTCAATCGTAATGC-1	singlet	0	-1847.804663	-5467.731992	-5383.302934	EMD2
CTTTCAATCTAGACAC-1	singlet	2	-4293.003504	-3599.556203	-1379.106863	EMD2
CTTTCGGAGCACTCGC-1	singlet	1	-2330.582877	-635.4199844	-1713.13878	EMD2
CTTTCGGCAAAGGCTG-1	singlet	2	-4845.471855	-3803.369781	-1236.408625	EMD2
CTTTCGGCAGGCATTT-1	singlet	2	-779.558585	-747.4553922	-250.7046809	EMD2
CTTTCGGCAGGTGACA-1	singlet	0	-1025.773154	-3665.103002	-3259.039621	EMD2
CTTTCGGGTAAGCAAT-1	singlet	1	-394.8321589	-139.2105866	-318.2074294	EMD2
CTTTCGGGTGAGATAT-1	unassigned	2	-8.688155097	-4.878080351	-4.057297142	EMD2
CTTTCGGTCGCTAGCG-1	singlet	1	-1640.854172	-573.99818	-1350.386719	EMD2
CTTTCGGTCGGCTGAC-1	doublet	45323	-4265.943879	-2385.667807	-1997.771619	EMD2
CTTTCGGTCGTCCTCA-1	singlet	2	-1045.299498	-793.7289333	-353.8082122	EMD2
GAAACCTAGACCCTTA-1	singlet	1	-3356.217403	-1101.544062	-2643.869811	EMD2
GAAACCTAGCTTAGTC-1	singlet	1	-4115.841877	-1371.66884	-3381.570322	EMD2
GAAACCTCAGAGCGTA-1	singlet	2	-1986.98835	-1487.19852	-491.3292293	EMD2
GAAACCTGTAGGTGCA-1	singlet	1	-1292.373793	-453.3219995	-1045.590383	EMD2
GAAACCTGTCGTAATC-1	singlet	2	-4256.514818	-3338.288007	-1344.878468	EMD2
GAAACCTGTGCAACGA-1	singlet	2	-8526.830198	-7717.557154	-2390.04976	EMD2
GAAACCTGTGCTCGTG-1	singlet	1	-917.8069718	-297.6159176	-725.0140302	EMD2
GAAACCTTCACGTCCT-1	singlet	2	-6718.443524	-5645.585139	-2112.067581	EMD2
GAAACCTTCATGAGGG-1	singlet	0	-1006.082595	-3157.363019	-2895.288235	EMD2
GAAACCTTCGACATTG-1	singlet	1	-1957.981014	-575.8099957	-1631.128942	EMD2
GAAACCTTCTAGTGTG-1	singlet	2	-729.7401406	-594.6392054	-263.7363406	EMD2
GAAATGAAGAGGTGCT-1	singlet	1	-284.4926804	-100.9627187	-231.385904	EMD2
GAAATGAAGCTACTGT-1	singlet	2	-2736.878293	-2647.116972	-852.4807152	EMD2
GAAATGAAGGTACATA-1	singlet	0	-279.9935847	-872.9200849	-865.1296156	EMD2
GAAATGACAGAATTCC-1	singlet	1	-1500.435511	-500.4739503	-1131.332115	EMD2
GAAATGAGTATGAAGT-1	singlet	1	-524.9618258	-232.6965803	-472.1881767	EMD2
GAAATGAGTTCAAACC-1	singlet	2	-528.4108826	-586.7213959	-253.3996024	EMD2
GAAATGAGTTCTAAGC-1	singlet	0	-43.4695367	-91.14735894	-88.2152863	EMD2
GAAATGAGTTCTTCAT-1	singlet	1	-2379.414591	-794.584154	-1977.604197	EMD2
GAAATGATCTGTGTGA-1	singlet	0	-66.52247011	-192.5721553	-187.0595919	EMD2
GAACACTAGCGTTAGG-1	doublet	45323	-1172.769339	-863.0969979	-650.4633613	EMD2
GAACACTAGGCACTCC-1	singlet	1	-2402.911053	-886.1683952	-1928.161446	EMD2
GAACACTTCCGACGGT-1	singlet	0	-333.0668668	-941.5823924	-924.127062	EMD2
GAACACTTCCGAGTGC-1	doublet	45293	-4477.829222	-1900.271366	-2865.730423	EMD2
GAACGTTAGCCGGAAT-1	singlet	1	-968.7554886	-326.5339186	-859.6681869	EMD2
GAACGTTAGGAGAATG-1	singlet	1	-2689.429965	-791.634283	-2381.770757	EMD2
GAACGTTAGTTGCCTA-1	singlet	1	-791.8422197	-293.3629016	-633.3517044	EMD2
GAACGTTCAATCGTCA-1	singlet	1	-1864.380991	-552.3906805	-1392.165557	EMD2
GAACGTTCACCTGAAT-1	singlet	0	-786.0279943	-2600.037705	-2398.300092	EMD2
GAACGTTCAGGTAGTG-1	singlet	1	-1066.409965	-363.9040747	-779.1443468	EMD2
GAACGTTGTAGCGAGT-1	singlet	1	-265.2495151	-142.5834628	-288.8556522	EMD2
GAACGTTGTCCGGACT-1	singlet	1	-2292.999645	-676.1579733	-1747.585218	EMD2
GAACGTTGTCTCGCGA-1	singlet	0	-551.9905537	-1757.589355	-1534.502188	EMD2
GAACGTTGTGTTCATG-1	singlet	1	-1065.25365	-362.2637817	-917.0108503	EMD2
GAACGTTGTTCGGTAT-1	singlet	1	-371.0977065	-118.7317574	-313.3893069	EMD2
GAACGTTTCAGCGCGT-1	singlet	0	-265.6278678	-806.9844621	-721.5463592	EMD2
GAACGTTTCATCGACA-1	singlet	2	-3370.775365	-2519.971283	-872.5930087	EMD2
GAACTGTAGCAACCAG-1	singlet	0	-100.5926844	-275.1623928	-244.1246044	EMD2

GAACTGTAGGCGTTGA-1	singlet	1	-1932.704843	-653.4743826	-1641.161947	EMD2
GAACTGTCAAGAATGT-1	singlet	1	-579.8550344	-208.2018082	-514.8983978	EMD2
GAACTGTCACTGAGGA-1	singlet	0	-837.29927	-2386.718961	-2261.615206	EMD2
GAACTGTCAGGAGGAG-1	singlet	1	-1514.79435	-415.4182938	-1245.202813	EMD2
GAACTGTCATGGAGAC-1	doublet	1/0	-70.57517031	-36.94515251	-72.04652739	EMD2
GAACTGTGTCTAGGCC-1	singlet	1	-1283.758716	-476.8590322	-1122.598242	EMD2
GAAGAATAGCTCTTCC-1	singlet	0	-111.2367265	-220.4914258	-214.1628659	EMD2
GAAGAATAGGAACTCG-1	singlet	2	-1199.004983	-982.8267373	-499.2499325	EMD2
GAAGAATCAGAGTTGG-1	singlet	0	-183.9375194	-573.3373533	-579.6684094	EMD2
GAAGAATCATTGGATC-1	singlet	1	-1695.856266	-426.5947298	-1397.22655	EMD2
GAAGAATGTATGGTAA-1	singlet	2	-789.8382736	-735.3086447	-312.4731667	EMD2
GAAGAATGTGTAGGAC-1	singlet	1	-157.790137	-59.41214738	-125.3364733	EMD2
GAAGAATGTTCGTACA-1	singlet	1	-689.0936925	-169.4477579	-555.5436292	EMD2
GAAGAATTCAGTGTTG-1	singlet	1	-323.4858057	-124.9616208	-301.7801556	EMD2
GAAGAATTCATTTACC-1	singlet	0	-516.2148809	-1465.397317	-1449.327779	EMD2
GAAGCCCAGGTCACCC-1	singlet	0	-2174.2588	-7379.335169	-7667.073008	EMD2
GAAGCCCAGGTTCTAC-1	singlet	2	-751.8911596	-660.9640457	-355.656922	EMD2
GAAGCCCAGTCACTGT-1	doublet	1/0	-1515.118917	-1015.813588	-1684.446806	EMD2
GAAGCCCAGTCCCTAA-1	singlet	1	-1245.134128	-443.2884911	-960.3085165	EMD2
GAAGCCCCACAAGCCC-1	singlet	2	-1687.754546	-1611.82128	-577.7862113	EMD2
GAAGCCCCAGCAGTCC-1	singlet	1	-2646.869676	-819.5244723	-2007.727918	EMD2
GAAGCCCGTAGTCCTA-1	singlet	1	-741.4366584	-249.1731732	-685.1391026	EMD2
GAAGCCCGTTTCGTTT-1	singlet	1	-421.7308125	-135.6806904	-296.9644533	EMD2
GAAGCCCTCACTGTCC-1	singlet	1	-351.0281753	-168.8721675	-304.6716071	EMD2
GAAGCCCTCATAAGGA-1	singlet	1	-321.1725917	-151.6995751	-289.7539148	EMD2
GAAGCCCTCGCGGTAC-1	singlet	0	-506.3277294	-1790.699032	-1689.36951	EMD2
GAAGCCCTCTCCGCAT-1	singlet	0	-1431.64191	-6441.574899	-6094.849779	EMD2
GAAGCGAAGCCGTTAT-1	singlet	0	-204.592944	-628.5389307	-596.3509615	EMD2
GAAGCGAAGCTGACCC-1	doublet	45293	-72.81932226	-36.03385799	-56.8079618	EMD2
GAAGCGACAACACAGG-1	singlet	1	-237.1492384	-82.41244555	-207.8556423	EMD2
GAAGCGACACACGTGC-1	singlet	0	-70.14203254	-181.5094828	-210.4600032	EMD2
GAAGCGACACCATTCC-1	singlet	1	-591.5919568	-224.2567317	-571.1612259	EMD2
GAAGCGACACTAACGT-1	singlet	1	-663.6381768	-245.9297787	-603.1204721	EMD2
GAAGCGACAGCGTTGC-1	singlet	1	-1413.875566	-358.0612732	-1186.522676	EMD2
GAAGCGACAGGTCCGT-1	singlet	2	-568.378632	-662.3721374	-183.5508205	EMD2
GAAGCGATCACACGAT-1	singlet	1	-2220.331644	-602.6453244	-1951.094786	EMD2
GAAGCGATCAGTAGGG-1	singlet	0	-1248.107466	-4385.651185	-4232.215385	EMD2
GAAGCGATCATCACAG-1	singlet	2	-3597.580507	-2768.605728	-822.3853986	EMD2
GAAGCGATCCACATAG-1	singlet	1	-4271.936457	-1335.799203	-3174.397436	EMD2
GAAGCGATCCTAGCCT-1	unassigned	1/0	-9.766064153	-4.871794257	-6.60850614	EMD2
GAAGCGATCGATACAC-1	singlet	0	-1725.933643	-4046.422831	-3636.401298	EMD2
GAAGGACAGAGAATCT-1	singlet	0	-169.6528325	-608.5877176	-447.4106214	EMD2
GAAGGACAGCTACAAA-1	singlet	1	-1325.806876	-452.5104289	-1148.558777	EMD2
GAAGGACAGGGCGAAG-1	singlet	2	-1128.756735	-1043.852637	-372.8783421	EMD2
GAAGGACCAAGACGAC-1	unassigned	0	-1.865872153	-7.632325104	-3.992409434	EMD2
GAAGGACCAATACAGA-1	singlet	1	-951.2593644	-353.4867136	-808.374821	EMD2
GAAGGACGTTAGGACG-1	singlet	2	-1398.310144	-1259.747481	-463.0689398	EMD2
GAAGGACTCCTACGGG-1	singlet	2	-384.0390149	-359.1520576	-158.4224165	EMD2
GAAGGACTCTAAGGAA-1	singlet	1	-67.54040811	-31.54450826	-52.51786295	EMD2
GAAGGACTCTTAAGGC-1	singlet	2	-458.906831	-414.6515586	-182.6929765	EMD2
GAAGGACTCTTCCTAA-1	singlet	1	-3979.104109	-1348.14844	-3328.553093	EMD2
GAAGGGTAGAGCAACC-1	singlet	1	-236.5967841	-90.59840485	-209.5128455	EMD2
GAAGGGTAGCCTGTCG-1	singlet	1	-2121.098943	-711.9031879	-1736.661384	EMD2
GAAGGGTAGTTACGGG-1	singlet	0	-71.94921474	-264.7598731	-194.6657583	EMD2
GAAGGGTCACCTGCGA-1	singlet	2	-165.2339923	-164.2423331	-59.76412105	EMD2
GAAGGGTGTACCCAGC-1	singlet	1	-412.3685968	-178.0191112	-361.8263001	EMD2
GAAGGGTGTACTAACC-1	singlet	0	-250.2192053	-932.1455973	-840.3989627	EMD2
GAAGGGTGTTACCCTC-1	singlet	0	-306.3899321	-1056.722962	-842.2519162	EMD2
GAAGGGTTCGTGTGAT-1	singlet	1	-1360.466094	-438.6644805	-1101.151708	EMD2
GAAGTAAAGATCCGAG-1	singlet	1	-2572.108283	-852.3803722	-2383.836074	EMD2
GAAGTAAAGTCTAGAA-1	singlet	1	-446.4993442	-175.0873108	-374.5187531	EMD2
GAAGTAACACAAGGTG-1	singlet	0	-300.4758659	-1071.416013	-977.2865914	EMD2
GAAGTAACACGCGGTT-1	singlet	2	-5184.007222	-4065.556303	-1421.136733	EMD2
GAAGTAACAGGAGGAG-1	singlet	1	-1148.924357	-364.4182004	-908.0367665	EMD2
GAAGTAAGTAAGACCG-1	singlet	1	-687.3739205	-216.1850959	-529.2823681	EMD2
GAAGTAAGTTGAAGTA-1	doublet	1/0	-3606.783828	-2977.195839	-4131.667275	EMD2
GAAGTAAGTTTACGAC-1	unassigned	2/0	-14.87248921	-19.5906757	-7.263569546	EMD2
GAAGTAATCAACACGT-1	singlet	1	-972.7724583	-325.8998084	-841.3845192	EMD2
GAAGTAATCAGTCCGG-1	singlet	2	-2817.374458	-2401.25483	-906.9585401	EMD2
GAAGTAATCGAACGCC-1	singlet	1	-388.8424156	-164.7136458	-335.2577042	EMD2
GAAGTAATCTATTTCG-1	singlet	1	-3498.050966	-1147.27975	-2788.435181	EMD2
GAATAGAAGATAGCAT-1	singlet	0	-1223.751083	-3978.72928	-3654.107016	EMD2
GAATAGAAGCACAAAT-1	singlet	0	-736.3488192	-2018.547644	-1880.525073	EMD2
GAATAGAAGTCAGAGC-1	singlet	1	-4112.379806	-1370.296071	-3170.969422	EMD2
GAATAGAAGTTGTCAC-1	singlet	1	-512.38816	-192.8482454	-447.1174898	EMD2
GAATAGACACAAGCCC-1	singlet	0	-2383.507602	-8181.160079	-7684.754133	EMD2
GAATAGAGTCACTTCC-1	singlet	2	-3882.244527	-3119.483025	-1171.670546	EMD2
GAATAGAGTGACCGTC-1	singlet	2	-883.3766097	-943.8456791	-320.765255	EMD2
GAATAGATCCTTATAC-1	singlet	1	-273.019832	-100.0667226	-207.3372755	EMD2
GAATAGATCGCGTGCA-1	singlet	0	-365.3650882	-1313.561719	-1070.481612	EMD2
GAATAGATCGGAATGG-1	singlet	0	-39.17860465	-104.9242828	-139.1483527	EMD2
GAATAGATCTACTGAG-1	singlet	1	-1972.314887	-678.7671597	-1607.455018	EMD2
GAATCACAGAAACTCA-1	singlet	2	-11551.49872	-9215.493804	-3403.936979	EMD2
GAATCACAGTACCCTA-1	singlet	2	-65.40946339	-73.10746929	-22.51891992	EMD2
GAATCACCAGCGACCT-1	singlet	1	-439.2327847	-169.4346673	-360.3369597	EMD2
GAATCACGTTCAGCTA-1	singlet	1	-1396.630801	-541.5107955	-1130.093353	EMD2
GAATCACGTTGATCGT-1	singlet	2	-3077.584935	-2790.862877	-991.6533965	EMD2
GAATCACTCGACGCTG-1	singlet	2	-2544.903561	-2926.214566	-948.1392754	EMD2
GAATCACTCGCGTCGA-1	singlet	2	-2103.074523	-1864.062699	-726.9332841	EMD2
GAATCACTCGTGTCAA-1	singlet	2	-2618.589645	-2458.386553	-789.5531557	EMD2
GAATCGTAGAACTCCT-1	singlet	2	-1526.979183	-1364.806496	-569.5255056	EMD2
GAATCGTAGGGTACGT-1	singlet	0	-21.57185842	-59.86599202	-51.01608111	EMD2
GAATCGTAGGTCGCCT-1	singlet	0	-2702.421301	-8779.783472	-8199.784319	EMD2
GAATCGTAGTGTTGTC-1	singlet	1	-2853.664864	-861.7318661	-2095.881597	EMD2
GAATCGTCAAATGAAC-1	singlet	2	-250.259431	-157.126607	-64.34185001	EMD2
GAATCGTCAAGTGGTG-1	singlet	1	-2617.700575	-650.4372584	-1775.177155	EMD2
GAATCGTCAGACGATG-1	doublet	1/0	-3743.309893	-3317.354355	-3603.55809	EMD2
GAATCGTCATGTCAGT-1	singlet	1	-1774.236402	-619.9482474	-1521.603451	EMD2
GAATCGTGTGATACAA-1	singlet	1	-377.8794239	-162.6644757	-269.3035547	EMD2
GAATCGTTCCTGATAG-1	singlet	1	-346.9180343	-120.3371794	-288.169621	EMD2
GAATCGTTCTCACGAA-1	doublet	45323	-1372.507792	-1020.928656	-714.0120825	EMD2
GAATCGTTCTTCCTAA-1	singlet	1	-193.4511936	-76.92203017	-191.685237	EMD2
GACACGCAGCTGCCAC-1	singlet	0	-479.8639737	-1529.817364	-1306.082318	EMD2
GACACGCAGGTGCTGA-1	singlet	1	-1041.291321	-329.6072362	-894.3212394	EMD2
GACACGCCACACGTGC-1	singlet	0	-874.379712	-2700.115507	-2392.25494	EMD2
GACACGCCACTTGGGC-1	singlet	1	-461.1312439	-156.9915184	-378.1815565	EMD2
GACACGCCAGAAATTG-1	singlet	1	-2223.77907	-731.2635238	-1892.662341	EMD2
GACACGCGTTCCGCTT-1	singlet	0	-120.7036744	-378.7480361	-332.3909486	EMD2
GACACGCTCGATGCAT-1	singlet	1	-410.4213773	-132.8025735	-298.3615394	EMD2
GACAGCCAGTTGTAAG-1	singlet	1	-1501.648455	-549.7601875	-1275.386335	EMD2

GACAGCCCAACTCATG-1	singlet	0	-251.6300417	-642.1525413	-700.0974216	EMD2
GACAGCCGTACCCGAC-1	singlet	1	-170.4695192	-80.26981085	-174.6755793	EMD2
GACAGCCGTGAGTCAG-1	singlet	0	-465.9373269	-1335.826015	-1330.793782	EMD2
GACAGCCGTGCAATGG-1	singlet	2	-335.1015994	-214.6677265	-110.4161841	EMD2
GACAGCCGTGCATGTT-1	singlet	1	-623.725853	-145.9566265	-384.0241209	EMD2
GACAGCCGTTCGAAGG-1	singlet	1	-386.7484828	-163.8306073	-297.7431139	EMD2
GACAGCCTCAGACTGT-1	doublet	0/1	-2853.812728	-3643.615601	-4481.228639	EMD2
GACAGCCTCCGCGGAT-1	singlet	2	-391.3294431	-395.3427636	-188.3429908	EMD2
GACAGCCTCCGTGGTG-1	singlet	0	-106.2231278	-355.8955702	-362.4182365	EMD2
GACAGCCTCGCATAGT-1	singlet	0	-786.5577002	-2247.489949	-2167.688654	EMD2
GACATCAAGACGGAAA-1	singlet	1	-384.8640653	-173.5827805	-426.4779331	EMD2
GACATCAAGCAAGGAA-1	singlet	1	-1146.037762	-338.312384	-911.4226032	EMD2
GACATCAAGGTCTTTG-1	singlet	1	-1164.737681	-470.7722369	-1044.074478	EMD2
GACATCACACATGGTT-1	singlet	0	-107.8797561	-314.5756592	-285.2386573	EMD2
GACATCACATGGCACC-1	singlet	1	-3546.687367	-961.9005869	-2637.300651	EMD2
GACCAATCAACCGACC-1	singlet	1	-375.3850447	-169.5887007	-304.1735379	EMD2
GACCAATCAAGAGTGC-1	singlet	1	-3199.563347	-1125.275262	-2727.294033	EMD2
GACCAATCAGCTGGTC-1	singlet	2	-480.7378217	-432.2194027	-204.7038148	EMD2
GACCAATCATGAAGCG-1	singlet	2	-5390.410491	-3555.055748	-1628.625812	EMD2
GACCAATGTAGGAGTC-1	singlet	1	-2796.348399	-974.3759371	-2332.204917	EMD2
GACCAATGTGAATATG-1	singlet	2	-1346.579655	-1174.104842	-427.3426526	EMD2
GACCAATGTGAGAACC-1	singlet	0	-1331.615611	-6192.877071	-5936.793511	EMD2
GACCAATGTGGGCTCT-1	singlet	2	-4072.646808	-3076.922529	-1344.058976	EMD2
GACCAATGTTCTCCTG-1	singlet	1	-671.7319508	-266.4074913	-587.7334241	EMD2
GACCAATTCAGAGTGG-1	singlet	0	-165.8348018	-562.0626822	-477.4665872	EMD2
GACCAATTCGGTATGT-1	singlet	1	-133.5074059	-48.27824839	-113.9861427	EMD2
GACCAATTCTCTATGT-1	singlet	0	-260.4135316	-903.6872102	-731.3684229	EMD2
GACCAATTCTTCCAGC-1	singlet	0	-610.7719189	-1796.456528	-1922.287215	EMD2
GACCCAGAGCCATGCC-1	singlet	1	-748.5655087	-276.3925469	-650.6399805	EMD2
GACCCAGAGGGCAAGG-1	singlet	0	-648.6223749	-1776.058834	-1846.553022	EMD2
GACCCAGCAGACAATA-1	singlet	1	-419.6676649	-201.6053197	-411.8399076	EMD2
GACCCAGCATTATGCG-1	singlet	1	-360.4394963	-146.6538533	-293.9618296	EMD2
GACCCAGGTACGCGTC-1	singlet	1	-747.3017784	-244.0379287	-653.1395967	EMD2
GACCCAGGTGTTCGTA-1	singlet	1	-4040.056467	-1089.896752	-3142.571199	EMD2
GACCCAGGTTAAACCC-1	singlet	2	-1167.173075	-1076.464939	-438.2557284	EMD2
GACCCAGGTTGAATCC-1	singlet	0	-242.4060623	-725.0314919	-672.8359334	EMD2
GACCCAGTCACAATGC-1	singlet	1	-431.7163591	-136.6922939	-369.8152628	EMD2
GACCCAGTCCAGTGTA-1	singlet	1	-1192.149926	-455.3491238	-1005.319634	EMD2
GACCCAGTCCGCACGA-1	singlet	0	-447.2003545	-1116.550433	-1117.622392	EMD2
GACCCTTAGAGGTTAT-1	singlet	1	-125.4078344	-46.95884158	-123.2256182	EMD2
GACCCTTCAACCCTCT-1	singlet	0	-101.3241637	-264.371701	-276.1536717	EMD2
GACCCTTGTCACTTAG-1	singlet	1	-326.5138918	-103.5831037	-300.0324785	EMD2
GACCCTTGTGTGACCC-1	singlet	2	-237.8138095	-263.7164638	-110.1619076	EMD2
GACCGTGAGGAACTCG-1	singlet	2	-657.2805339	-581.6302544	-274.9558333	EMD2
GACCGTGCAGTTTCAG-1	singlet	0	-923.7625879	-2779.872489	-2683.965479	EMD2
GACCGTGGTATTTCTC-1	singlet	1	-2845.162072	-822.8942744	-1835.657559	EMD2
GACCGTGGTGTACGCC-1	singlet	1	-310.0963528	-113.6586812	-287.335668	EMD2
GACCGTGGTTAACAGA-1	singlet	1	-2030.760928	-619.0925375	-1537.902051	EMD2
GACCGTGGTTTGTGGT-1	singlet	1	-2642.803117	-703.6205328	-1951.100044	EMD2
GACCGTGTCCGCTTAC-1	singlet	1	-1873.517321	-633.7088696	-1541.693984	EMD2
GACCGTGTCCTTTGAT-1	singlet	2	-1047.116708	-1006.437308	-359.1266251	EMD2
GACCTTCAGGAGAATG-1	singlet	0	-424.84701	-1343.45775	-1284.311737	EMD2
GACCTTCAGGCCACCT-1	singlet	1	-1063.704399	-355.4823119	-838.9282206	EMD2
GACCTTCAGTTCATCG-1	doublet	1/0	-982.6819858	-943.4217441	-1144.809573	EMD2
GACCTTCAGTTTCAGC-1	singlet	2	-1977.330482	-1917.781094	-652.0633171	EMD2
GACCTTCCAATACCCA-1	singlet	0	-210.6568081	-496.5826758	-505.1385413	EMD2
GACCTTCCACTCATAG-1	singlet	0	-1734.345602	-5954.708217	-5622.517617	EMD2
GACCTTCCATCCCACT-1	singlet	2	-452.8181113	-432.9813785	-220.9961935	EMD2
GACCTTCCATCGGCCA-1	singlet	1	-655.3773446	-226.3866902	-524.0931409	EMD2
GACCTTCCATCGTGGC-1	singlet	1	-2001.261772	-727.7334923	-1740.671388	EMD2
GACCTTCCATCTTCGC-1	singlet	2	-1857.453693	-1801.510965	-729.1261997	EMD2
GACCTTCGTCGGAAAC-1	singlet	0	-743.2867007	-2676.580158	-2363.559549	EMD2
GACCTTCGTGCAACGA-1	singlet	1	-2335.342184	-730.9859611	-1787.541029	EMD2
GACCTTCGTTAAGAAC-1	singlet	1	-1840.788267	-617.4769423	-1549.211219	EMD2
GACCTTCTCACCTCGT-1	singlet	1	-16.11090378	-8.843323647	-16.20286635	EMD2
GACCTTCTCTCACTCG-1	singlet	2	-310.4370973	-210.5137418	-130.7471728	EMD2
GACCTTCTCTCTCAAT-1	singlet	1	-3391.040044	-1046.410421	-2839.249692	EMD2
GACGCTGAGAAGGGAT-1	singlet	0	-550.425226	-1520.927927	-1588.183842	EMD2
GACGCTGAGACTTAAG-1	singlet	0	-2007.073892	-8649.242423	-8410.324067	EMD2
GACGCTGAGCCATGCC-1	singlet	1	-2059.494009	-646.5820544	-1958.751808	EMD2
GACGCTGAGGATTTAG-1	singlet	1	-3494.324137	-1025.285867	-2758.603134	EMD2
GACGCTGAGGGTTTCT-1	singlet	1	-2403.373767	-726.843074	-2043.782672	EMD2
GACGCTGAGTGAATAC-1	doublet	0/2	-2005.51433	-3690.588912	-2924.129467	EMD2
GACGCTGCAAATGAGT-1	singlet	1	-1611.403136	-494.2078501	-1449.737661	EMD2
GACGCTGCAAGCACCC-1	singlet	1	-89.38313953	-42.70565457	-98.12628312	EMD2
GACGCTGCAATTAGGA-1	singlet	1	-2280.662794	-663.9943861	-1641.419318	EMD2
GACGCTGCACCAGCTG-1	singlet	1	-376.4722659	-145.8224202	-306.2953927	EMD2
GACGCTGCACGACGCT-1	singlet	2	-662.6068914	-629.224752	-272.5544668	EMD2
GACGCTGCAGAATGTA-1	singlet	0	-287.2629158	-763.2826602	-708.0334565	EMD2
GACGCTGCAGTAACGG-1	singlet	1	-3365.749021	-1085.244307	-2539.697299	EMD2
GACGCTGGTCTTTATC-1	singlet	0	-674.6461617	-2056.847988	-1969.273073	EMD2
GACGCTGTCGTTGTAG-1	singlet	0	-134.7793796	-483.3726523	-395.3535674	EMD2
GACGTTAAGAGGGCGA-1	singlet	0	-446.3184079	-1285.652987	-1304.800234	EMD2
GACGTTAAGCTAATCC-1	singlet	2	-788.6156098	-823.943635	-272.7277214	EMD2
GACGTTAAGGACAAGA-1	singlet	2	-548.2109094	-545.3239911	-217.0755738	EMD2
GACGTTAAGGCAATGC-1	singlet	2	-9198.217112	-7153.166546	-2396.92093	EMD2
GACGTTAAGTGGATAT-1	singlet	1	-5576.954557	-1583.927226	-4434.48381	EMD2
GACGTTACAACATCGT-1	singlet	1	-1540.882006	-500.0521873	-1357.731986	EMD2
GACGTTAGTCACTAGT-1	singlet	0	-334.9986754	-1376.418727	-1246.371591	EMD2
GACGTTAGTCTGTCAA-1	doublet	0/1	-1881.570289	-3394.102988	-3581.905667	EMD2
GACGTTAGTCTTCATT-1	doublet	45293	-2727.352473	-1532.400015	-1665.256501	EMD2
GACGTTAGTGATATAG-1	singlet	2	-706.947523	-700.6382832	-316.3782936	EMD2
GACGTTAGTGTTATCG-1	singlet	1	-2374.504175	-686.1273952	-1792.728682	EMD2
GACGTTAGTTTGGCTA-1	singlet	2	-360.3462947	-407.4865169	-150.8546187	EMD2
GACGTTATCAGCGGAA-1	singlet	1	-1402.011981	-458.8066522	-1192.506784	EMD2
GACGTTATCAGGAACG-1	singlet	1	-519.1348518	-160.0899683	-364.9423085	EMD2
GACGTTATCCACTGGG-1	singlet	1	-71.89161698	-19.75210908	-62.54500277	EMD2
GACGTTATCCTACTGC-1	singlet	1	-2913.371148	-909.1542592	-2470.710533	EMD2
GACGTTATCGTAACAC-1	singlet	1	-397.6006158	-159.5121646	-306.9757272	EMD2
GACTATGCAACAAGTA-1	singlet	0	-221.6435043	-1008.959409	-1156.849516	EMD2
GACTATGCACGATAGG-1	doublet	0/1	-2018.817245	-2628.224295	-3075.422227	EMD2
GACTATGCATGTGCTA-1	singlet	1	-3477.893193	-1122.739932	-2980.827986	EMD2
GACTATGGTGTCATGT-1	singlet	1	-356.4515509	-165.1313497	-307.7384825	EMD2
GACTATGGTTGGAGGT-1	singlet	0	-470.0450362	-1563.006943	-1425.934721	EMD2
GACTATGTCACATTGG-1	singlet	0	-1609.325325	-6417.964308	-6151.907444	EMD2
GACTATGTCATTGCGA-1	singlet	1	-1633.094392	-657.7550395	-1315.922653	EMD2
GACTATGTCTGGAAGG-1	singlet	1	-224.3071384	-88.04152678	-192.9125103	EMD2
GACTCAAAGACGATAT-1	singlet	2	-458.9568793	-408.838133	-189.556668	EMD2
GACTCAACAACCGCTG-1	singlet	1	-1690.950609	-553.3927586	-1460.698174	EMD2
GACTCAACAGCGGATA-1	singlet	1	-432.7365452	-182.7088903	-357.3413086	EMD2

GACTCAAGTATAGCTC-1	singlet	0	-105.7420942	-510.289319	-446.4788642	EMD2
GACTCAAGTCGCGTCA-1	singlet	2	-2041.418269	-1701.041521	-652.4481038	EMD2
GACTCAAGTGGTAACG-1	singlet	0	-719.6318266	-2362.432342	-2257.271214	EMD2
GACTCAAGTTGCTCAA-1	doublet	0/2	-905.3279885	-1649.454331	-1280.942953	EMD2
GACTCAATCACATTGG-1	singlet	1	-2021.148902	-724.8390181	-1797.083791	EMD2
GACTCAATCACCCTCA-1	singlet	2	-427.7321902	-427.3980779	-162.2135617	EMD2
GACTCAATCATTTCGT-1	singlet	0	-1078.780223	-2916.640874	-2842.052772	EMD2
GACTCTCAGACCCGCT-1	singlet	1	-374.3454204	-164.1701046	-323.6052909	EMD2
GACTCTCAGCCATATC-1	singlet	1	-1738.599179	-659.1489417	-1296.141233	EMD2
GACTCTCAGGTGTGAC-1	singlet	1	-2532.407061	-941.4106178	-2089.607465	EMD2
GACTCTCCACACCTTC-1	singlet	1	-2812.128212	-1019.318495	-2368.975637	EMD2
GACTCTCCATGAGGGT-1	singlet	0	-176.9764646	-390.7094922	-343.7999318	EMD2
GACTCTCGTAACATAG-1	unassigned	1/0	-8.8070837	-7.940604703	-10.74068539	EMD2
GACTCTCGTTACGATC-1	singlet	0	-813.3201041	-2383.331571	-2466.739824	EMD2
GACTCTCGTTGCCGAC-1	doublet	0/1	-1996.961785	-3635.021188	-3982.946583	EMD2
GACTCTCTCTTTCCAA-1	singlet	1	-657.3088722	-290.6828722	-561.6586945	EMD2
GACTGATAGGCTGAAC-1	singlet	2	-187.7902834	-185.0765319	-79.2029591	EMD2
GACTGATAGTACAACA-1	singlet	1	-5025.022779	-1716.537408	-4102.195578	EMD2
GACTGATAGTCCTACA-1	singlet	2	-994.5819341	-803.9239544	-357.6959317	EMD2
GACTGATCACGCGCTA-1	singlet	2	-5959.745426	-4441.381778	-1208.831712	EMD2
GACTGATCAGAAGTGC-1	singlet	1	-277.7899297	-93.60611439	-200.1224189	EMD2
GACTGATCATAGAAAC-1	singlet	1	-2620.362521	-860.6976261	-2227.071374	EMD2
GACTGATGTCGCGGTT-1	singlet	1	-1375.354674	-449.8988976	-1078.191356	EMD2
GACTGATGTGCGTGCT-1	singlet	1	-308.1225417	-103.3116743	-223.5281143	EMD2
GACTGATGTTTCTATC-1	singlet	1	-498.4372792	-141.9839382	-348.2440595	EMD2
GACTGATTCAGACAAA-1	singlet	2	-906.4132343	-823.4521906	-309.1715592	EMD2
GACTGATTCCTAACAG-1	singlet	1	-242.3152113	-98.29514802	-214.1658669	EMD2
GACTGATTCGTAGTCA-1	singlet	1	-331.9127726	-106.5225775	-294.8442806	EMD2
GACTGATTCTACGGGC-1	singlet	1	-380.5127053	-180.784219	-321.8863613	EMD2
GACTGATTCTCTGACC-1	singlet	1	-3261.802617	-859.8383375	-2371.712201	EMD2
GACTTCCAGGCATCGA-1	singlet	2	-2155.818472	-1850.172932	-671.373081	EMD2
GACTTCCCACCAGTTA-1	singlet	1	-237.1084452	-62.43335109	-174.466773	EMD2
GACTTCCCATACATCG-1	singlet	0	-658.1678707	-2099.085996	-1794.482213	EMD2
GACTTCCGTCACCCTT-1	singlet	1	-484.3826148	-183.0044616	-403.6848937	EMD2
GACTTCCGTCCACGCA-1	singlet	0	-87.73456005	-218.2385586	-242.3486858	EMD2
GACTTCCGTTGTGTTG-1	singlet	1	-1808.491837	-424.004882	-1651.625859	EMD2
GACTTCCTCAACACGT-1	singlet	1	-216.9435199	-83.48931394	-199.9914317	EMD2
GACTTCCTCACCGACG-1	singlet	1	-505.0972415	-193.7309949	-441.6687945	EMD2
GACTTCCTCCGCACTT-1	singlet	1	-1507.862245	-538.4747709	-1226.405894	EMD2
GACTTCCTCCGTCAAA-1	singlet	1	-49.83183236	-19.3734977	-35.95769753	EMD2
GACTTCCTCGCGTGAC-1	singlet	1	-1688.369539	-606.0916952	-1408.841753	EMD2
GAGAAATAGATGTAGT-1	singlet	1	-2747.27678	-952.7676997	-2276.316267	EMD2
GAGAAATAGGTGGTTG-1	singlet	0	-305.1436752	-767.8537207	-760.4824827	EMD2
GAGAAATCAGTGTATC-1	singlet	0	-43.77760712	-77.4415114	-102.5886047	EMD2
GAGAAATGTCAGACGA-1	singlet	1	-495.5899634	-172.0262047	-423.0120134	EMD2
GAGAAATGTCGTGGTC-1	unassigned	1	-0.839070059	-0.817065165	-1.570862086	EMD2
GAGAAATGTGGCATCC-1	singlet	0	-62.50628268	-188.2311355	-179.698765	EMD2
GAGAAATGTTTACTGG-1	singlet	1	-1389.536266	-482.2245694	-1306.139137	EMD2
GAGAAATTCACTTTGT-1	singlet	1	-3662.759703	-1449.73166	-3153.801059	EMD2
GAGAAATTCGGCATCG-1	singlet	2	-925.1912117	-876.2842862	-410.4226171	EMD2
GAGAAATTCTTCGTAT-1	singlet	0	-981.4074134	-2975.816739	-2657.175052	EMD2
GAGACCCAGCGACAGT-1	singlet	2	-198.2444916	-185.0822857	-45.58233093	EMD2
GAGACCCCAAGGTACG-1	singlet	1	-1979.85901	-658.3515795	-1462.533035	EMD2
GAGACCCCACAAAGTA-1	singlet	1	-394.7873684	-158.897269	-349.9011811	EMD2
GAGACCCTCCATAGGT-1	singlet	1	-1258.935832	-484.087087	-1086.140802	EMD2
GAGACCCTCCTCACTG-1	singlet	0	-367.6613164	-1104.801912	-1068.026343	EMD2
GAGACCCTCCTTGGAA-1	singlet	0	-226.3209537	-707.7649987	-544.7897434	EMD2
GAGACTTAGCATGAAT-1	singlet	1	-472.7021157	-192.0776478	-397.6899206	EMD2
GAGACTTAGCTGTCCG-1	singlet	1	-343.3377744	-117.4087321	-302.8259113	EMD2
GAGACTTAGTATGAAC-1	singlet	1	-214.6601026	-78.37261564	-178.1752859	EMD2
GAGACTTTCTCACCCA-1	singlet	0	-100.0734723	-252.5454311	-225.8662474	EMD2
GAGACTTTCTGTCAGA-1	singlet	0	-268.5759759	-748.7447484	-713.7042729	EMD2
GAGACTTTCTTACGTT-1	singlet	1	-694.2934342	-232.0715191	-555.7025636	EMD2
GAGAGGTAGAGCCGAT-1	singlet	0	-338.8669817	-1300.532439	-928.5989645	EMD2
GAGAGGTAGCCAAGTG-1	singlet	2	-3657.904215	-3087.328348	-1202.150706	EMD2
GAGAGGTAGGGAGAAT-1	singlet	2	-381.115624	-484.7561148	-177.4622166	EMD2
GAGAGGTCACAAGCTT-1	singlet	0	-365.9288836	-1092.002766	-1042.363804	EMD2
GAGAGGTCACCATAAC-1	singlet	1	-2263.339801	-425.8584093	-1461.882319	EMD2
GAGATGGAGCAGCAGT-1	singlet	0	-130.6108713	-380.569501	-328.8254141	EMD2
GAGATGGAGTCTTCCC-1	singlet	1	-2227.317888	-674.2605237	-2082.543491	EMD2
GAGATGGCAAGCTACT-1	singlet	1	-1779.687447	-631.7493123	-1406.598931	EMD2
GAGATGGGTACAGTCT-1	singlet	0	-703.8737057	-2777.815807	-2421.941129	EMD2
GAGATGGGTTGCTCGG-1	singlet	1	-1144.792169	-365.4572538	-989.7430182	EMD2
GAGATGGTCAACGTGT-1	singlet	1	-1598.08726	-600.8128279	-1396.270044	EMD2
GAGATGGTCTAGCATG-1	singlet	0	-66.75488034	-167.0509758	-187.9743367	EMD2
GAGCCTGAGGATTTGA-1	singlet	1	-1828.553003	-465.1250378	-1153.463334	EMD2
GAGCCTGAGTCGAATA-1	singlet	2	-4532.661484	-3792.939039	-1440.748257	EMD2
GAGCCTGCACTCGATA-1	singlet	1	-1082.91744	-328.701411	-740.6714618	EMD2
GAGCCTGCAGAATGTA-1	singlet	1	-859.3252688	-266.0690034	-654.6165757	EMD2
GAGCCTGCATCCCGTT-1	singlet	1	-483.2889412	-193.3300985	-410.5575844	EMD2
GAGCCTGGTTCGAGCC-1	singlet	2	-3824.417656	-3125.679942	-1174.952851	EMD2
GAGCCTGTCAACGTGT-1	singlet	1	-334.8015088	-144.7983286	-278.4366063	EMD2
GAGCCTGTCGTTGTGA-1	singlet	1	-926.7702711	-322.925032	-751.9006493	EMD2
GAGCCTGTCTAGTGTG-1	singlet	0	-288.4240299	-1023.033963	-869.0552953	EMD2
GAGCTGCAGACTTGTC-1	singlet	2	-1603.679497	-1298.916162	-475.817108	EMD2
GAGCTGCAGAGCGACT-1	singlet	1	-359.409087	-141.0585908	-277.878984	EMD2
GAGCTGCAGCATGCAG-1	singlet	1	-198.4814818	-63.09746282	-117.6137186	EMD2
GAGCTGCAGCTCGAAG-1	singlet	2	-354.7605141	-342.9668569	-132.1415796	EMD2
GAGCTGCCAAAGCTCT-1	singlet	1	-413.1086004	-174.0729749	-363.0990723	EMD2
GAGCTGCCACGCTGAC-1	singlet	1	-2315.819646	-593.9633211	-1755.170492	EMD2
GAGCTGCCAGCAGACA-1	singlet	1	-1661.45781	-681.1113149	-1472.056607	EMD2
GAGCTGCCATACAGCT-1	singlet	1	-253.0594497	-89.66601707	-203.076461	EMD2
GAGCTGCCATGTTCGA-1	singlet	1	-1565.278357	-481.115487	-1157.681627	EMD2
GAGCTGCGTCGCTCGA-1	singlet	1	-1761.976289	-551.4321008	-1319.737723	EMD2
GAGCTGCGTCTCTCCA-1	singlet	1	-3290.682685	-912.7855679	-2510.328864	EMD2
GAGCTGCGTGATCATC-1	singlet	2	-499.0379439	-408.0772943	-201.7703344	EMD2
GAGCTGCGTTACACAC-1	singlet	0	-54.13144107	-218.5011693	-169.3016461	EMD2
GAGCTGCTCATTGTGG-1	singlet	1	-1070.236184	-358.9743754	-884.6812723	EMD2
GAGCTGCTCTCAGTCC-1	singlet	0	-749.9096437	-2166.436077	-2067.28051	EMD2
GAGGCAAAGCAGGTCA-1	singlet	1	-292.6094546	-125.3917526	-314.8137129	EMD2
GAGGCAAAGCCTTCTC-1	singlet	0	-1017.351102	-3696.572106	-3663.491028	EMD2
GAGGCAACAATACCCA-1	singlet	0	-1279.30595	-4053.855586	-3751.011166	EMD2
GAGGCAACACCTGCGA-1	singlet	2	-2869.253723	-2222.752063	-916.3966139	EMD2
GAGGCAACACGTGAGA-1	singlet	2	-921.702915	-747.5687641	-363.9280644	EMD2
GAGGCAACAGACCTAT-1	singlet	2	-122.5947881	-101.3814293	-39.48507557	EMD2
GAGGCAACAGCCTATA-1	singlet	1	-87.16591997	-22.19841599	-61.16129501	EMD2
GAGGCAAGTACCAATC-1	singlet	0	-580.0650705	-1916.461742	-1987.934137	EMD2
GAGGCAAGTAGAATAC-1	singlet	1	-5035.43051	-1455.893011	-4081.431871	EMD2
GAGGCAAGTCTAACTG-1	singlet	0	-71.21819478	-186.8550087	-165.352871	EMD2
GAGGCAAGTTCGTTCC-1	singlet	0	-408.8491262	-1035.264171	-1021.092798	EMD2

GAGGCAAGTTGATCGT-1	singlet	1	-2054.34923	-575.3437195	-1459.848028	EMD2
GAGGCAATCGCGCTGA-1	singlet	1	-124.2344303	-60.39446623	-125.3665965	EMD2
GAGGCAATCGCGTAGC-1	doublet	1/0	-12.04328788	-11.58785408	-12.44228305	EMD2
GAGGCAATCGTGGACC-1	singlet	0	-423.050604	-1303.182442	-1231.976173	EMD2
GAGGCAATCTGGCCGA-1	singlet	0	-149.8097376	-434.6586634	-452.4319537	EMD2
GAGGCCTAGATGGGCT-1	singlet	1	-1169.50947	-323.9557199	-752.2992803	EMD2
GAGGCCTAGCATTGAA-1	singlet	1	-3744.481695	-1094.818664	-3202.273942	EMD2
GAGGCCTCAAGGACAC-1	singlet	1	-782.4900314	-246.6064384	-605.6790005	EMD2
GAGGCCTCAGCTACCG-1	singlet	1	-1988.533459	-509.1957567	-1496.185652	EMD2
GAGGCCTCAGGTTACT-1	singlet	0	-71.72717434	-204.9986076	-185.3350589	EMD2
GAGGCCTCAGTCACGC-1	singlet	0	-53.55007834	-163.1370212	-176.66932	EMD2
GAGGCCTCATTGCTGA-1	singlet	2	-484.6506218	-481.4764597	-195.1493095	EMD2
GAGGCCTGTCATCCGG-1	singlet	1	-828.2070439	-359.2956195	-720.354014	EMD2
GAGGCCTGTGAGATTA-1	singlet	0	-1926.742315	-7684.367547	-7422.207249	EMD2
GAGGCCTGTGGTAATA-1	singlet	0	-319.3446382	-818.7078183	-747.3942832	EMD2
GAGGCCTTCACATTGG-1	singlet	2	-86.82869811	-102.0081427	-23.11884664	EMD2
GAGGGATAGAAGTATC-1	singlet	1	-1355.683642	-398.58026	-1120.325174	EMD2
GAGGGATAGATAGCAT-1	singlet	0	-1335.822775	-5046.366497	-5106.758389	EMD2
GAGGGATGTGCGTTTA-1	singlet	0	-852.4306882	-2917.948284	-2843.843928	EMD2
GAGGGATTCGCGATCG-1	singlet	1	-491.0418796	-192.7478308	-428.9199315	EMD2
GAGGGATTCTAGGAAA-1	singlet	1	-3505.779612	-857.9259432	-2518.173433	EMD2
GAGGGATTCTGTAAGC-1	singlet	2	-2859.373057	-2709.467668	-990.250143	EMD2
GAGGGTAAGGGCAGGA-1	singlet	0	-836.0696879	-3108.279134	-2873.978473	EMD2
GAGGGTAAGTCAGAGC-1	singlet	1	-438.3187573	-190.8401081	-394.8498588	EMD2
GAGGGTAAGTCGCCAC-1	singlet	0	-1266.173131	-4294.768649	-3941.940531	EMD2
GAGGGTACACGCCAGT-1	singlet	1	-264.6992142	-128.7144581	-305.4838262	EMD2
GAGGGTACATGAGTAA-1	singlet	1	-2957.668164	-932.3107012	-2384.990601	EMD2
GAGGGTACATGGCTGC-1	singlet	1	-1593.489776	-442.9577116	-1427.977765	EMD2
GAGGGTAGTTAACCTG-1	singlet	1	-1984.780125	-692.8993736	-1651.13125	EMD2
GAGGGTAGTTCTAACG-1	singlet	1	-1599.713528	-440.5574674	-1075.103028	EMD2
GAGGGTAGTTTAGTCG-1	singlet	2	-1859.584239	-1688.517068	-658.5450775	EMD2
GAGGGTATCTGCCTGT-1	unassigned	1	-9.226993803	-4.303812551	-5.013413	EMD2
GAGTCATCACTGCGAC-1	singlet	1	-201.9345132	-74.71094602	-150.2362054	EMD2
GAGTCATCACTTGAAC-1	singlet	1	-2003.933047	-696.3287766	-1618.35966	EMD2
GAGTCATCATACCACA-1	singlet	1	-745.5047493	-218.5633578	-500.3327422	EMD2
GAGTCATCATCGAAGG-1	singlet	1	-150.6366248	-32.24293685	-98.55366892	EMD2
GAGTCATGTGATACAA-1	singlet	1	-340.344131	-134.9626885	-324.3775218	EMD2
GAGTCATTCACCCATC-1	singlet	0	-579.8009473	-1616.60502	-1673.589752	EMD2
GAGTCATTCTAGTGAC-1	singlet	1	-1156.646228	-332.4388486	-856.990986	EMD2
GAGTCATTCTGGGCCA-1	singlet	2	-124.5668381	-138.1117138	-48.80906721	EMD2
GAGTCATTCTTCGGAA-1	singlet	1	-1479.968055	-437.2551366	-1255.408935	EMD2
GAGTCTAAGCTCTTCC-1	singlet	0	-226.3615821	-650.495619	-613.0327879	EMD2
GAGTCTAAGCTGAAAT-1	doublet	1/0	-4993.07813	-2571.964891	-4903.728064	EMD2
GAGTCTAAGGCTATCT-1	singlet	1	-7527.995197	-2048.544248	-5885.489773	EMD2
GAGTCTAAGTGAGGCT-1	singlet	1	-380.2425996	-103.334108	-253.2258648	EMD2
GAGTCTACAAGCACAG-1	singlet	1	-2344.374097	-715.5832512	-1926.784023	EMD2
GAGTCTACAATAGTAG-1	singlet	1	-2123.054065	-749.6027863	-1624.313547	EMD2
GAGTCTAGTACGAGCA-1	singlet	1	-843.2242926	-307.6758331	-665.9629701	EMD2
GAGTCTAGTACTGAGG-1	singlet	0	-38.77816961	-97.68038792	-93.6514016	EMD2
GAGTCTAGTCTCAGAT-1	singlet	1	-149.4055912	-20.89705891	-94.26204295	EMD2
GAGTCTAGTTACCTTT-1	singlet	0	-255.5137197	-605.9375944	-591.9001169	EMD2
GAGTCTATCTCGTGGG-1	singlet	2	-54.60742649	-38.23603904	-22.44737448	EMD2
GAGTCTATCTTTCGAT-1	singlet	0	-2048.105755	-7883.454227	-7767.612327	EMD2
GAGTGAGAGCAACTTC-1	singlet	0	-86.04441756	-362.8478349	-276.6928357	EMD2
GAGTGAGCAAGTATAG-1	singlet	0	-55.66960334	-216.9901606	-170.220438	EMD2
GAGTGAGCACCGCTGA-1	singlet	0	-748.1836127	-2320.633938	-2065.180689	EMD2
GAGTGAGGTAAGGTCG-1	singlet	1	-2335.471013	-638.6755763	-1678.451263	EMD2
GAGTGAGGTACGGCAA-1	singlet	1	-469.1180353	-122.5060387	-295.0863017	EMD2
GAGTGAGGTGGCTTAT-1	doublet	0/1	-102.8975789	-136.1989927	-139.304995	EMD2
GAGTGTTAGAAACTGT-1	singlet	0	-444.2709911	-1310.291834	-1230.599112	EMD2
GAGTGTTAGAAGATCT-1	singlet	0	-307.2253186	-1091.640244	-1082.68784	EMD2
GAGTGTTAGATAGCTA-1	singlet	1	-1457.537894	-507.919264	-1207.048669	EMD2
GAGTGTTCAACACAAA-1	singlet	2	-326.5682603	-312.7892877	-122.2170197	EMD2
GAGTGTTCAAGTAGTA-1	singlet	0	-1805.538778	-7065.325309	-7536.358396	EMD2
GAGTGTTGTATGCTAC-1	doublet	45323	-4035.880686	-2638.972531	-1584.419944	EMD2
GAGTGTTGTCCACATA-1	singlet	1	-1032.896045	-343.1106877	-803.9931113	EMD2
GAGTTACAGAATCGTA-1	singlet	1	-132.7584148	-60.26857219	-115.6931903	EMD2
GAGTTACAGCACACCC-1	singlet	1	-2783.865725	-1061.06541	-2409.074391	EMD2
GAGTTACAGCTAATCC-1	singlet	0	-941.113539	-2586.201492	-2473.166904	EMD2
GAGTTACCAAGAGCTG-1	singlet	1	-163.3278748	-66.83454195	-149.558042	EMD2
GAGTTACCAGCATCTA-1	singlet	1	-872.9898602	-308.3422338	-600.1327671	EMD2
GAGTTACGTTTACGAC-1	singlet	1	-913.8103755	-342.3042201	-846.8138765	EMD2
GAGTTGTAGCTCGAAG-1	singlet	1	-656.5364236	-215.2342278	-513.4785733	EMD2
GAGTTGTAGGAACTCG-1	singlet	2	-2264.135458	-2097.190161	-755.3685136	EMD2
GAGTTGTAGTAGGTTA-1	singlet	2	-391.7534127	-367.7381594	-131.841513	EMD2
GAGTTGTAGTGCCGAA-1	singlet	1	-683.6924299	-197.7831283	-554.8407433	EMD2
GAGTTGTCAAATGCTC-1	singlet	0	-753.8703999	-2192.53428	-2062.774032	EMD2
GAGTTGTCACAACCGC-1	singlet	0	-49.98158509	-200.7393177	-174.2641859	EMD2
GAGTTGTCATAATCGC-1	singlet	1	-122.9590048	-43.3065194	-64.76333291	EMD2
GAGTTGTGTATTGGCT-1	singlet	2	-152.6805406	-141.4666243	-64.7588595	EMD2
GAGTTGTTCAACGCTA-1	singlet	2	-1483.815246	-1158.079034	-468.2510124	EMD2
GAGTTGTTCCGTGTAA-1	singlet	1	-2244.752407	-707.345823	-2010.191548	EMD2
GAGTTGTTCCTGTAAG-1	singlet	2	-335.0426704	-278.2135907	-121.2848539	EMD2
GAGTTGTTCGGAGATG-1	singlet	1	-2995.257529	-1045.837741	-2297.059101	EMD2
GAGTTGTTCGGCCTTT-1	singlet	0	-807.0260562	-2561.432804	-2383.680329	EMD2
GAGTTGTTCTCGCAGG-1	singlet	0	-468.3005072	-1524.218297	-1450.190331	EMD2
GAGTTTGAGCAGATAT-1	singlet	0	-550.7364555	-1398.999392	-1344.133643	EMD2
GAGTTTGAGTATCCTG-1	singlet	1	-1424.257869	-455.1579149	-1162.726355	EMD2
GAGTTTGGTCTCGCGA-1	singlet	0	-128.5465162	-431.4244809	-395.1874512	EMD2
GAGTTTGTCCATTTCA-1	singlet	1	-344.5075984	-143.6598021	-288.8338577	EMD2
GAGTTTGTCCCAGCGA-1	singlet	2	-843.1333581	-796.3371437	-304.2823041	EMD2
GAGTTTGTCGGACAAG-1	singlet	0	-929.8688166	-2989.872879	-2820.085091	EMD2
GAGTTTGTCGTCAGAT-1	singlet	1	-525.6661255	-227.0256467	-438.3109286	EMD2
GAGTTTGTCTGCCTGT-1	singlet	1	-266.6854733	-130.4881188	-287.9381674	EMD2
GAGTTTGTCTTCGCTG-1	doublet	0/1	-1558.074539	-2269.917648	-2616.33494	EMD2
GATAGAAAGAGTATAC-1	singlet	1	-114.1291566	-56.69972098	-101.9049558	EMD2
GATAGAAAGATCACCT-1	singlet	1	-326.9346101	-136.1153087	-288.5963582	EMD2
GATAGAAAGCCTCAAT-1	singlet	1	-1119.681155	-391.0728312	-828.0119313	EMD2
GATAGAAAGGCCATAG-1	singlet	2	-630.5637188	-700.1394011	-282.2131884	EMD2
GATAGAAAGTTCACTG-1	singlet	0	-596.3099725	-1815.609224	-1662.268919	EMD2
GATAGAACAAGTGGCA-1	singlet	0	-1199.491982	-3559.515438	-3397.325678	EMD2
GATAGAAGTAAGAACT-1	singlet	1	-1270.001522	-461.2182297	-955.1055352	EMD2
GATAGAAGTAGTGTGG-1	singlet	0	-365.0233536	-925.234049	-856.4141434	EMD2
GATAGAAGTGTAGTGG-1	singlet	1	-1516.875268	-494.8509832	-1128.890266	EMD2
GATAGAATCTCTAAGG-1	singlet	1	-2929.755148	-978.5153256	-2456.587019	EMD2
GATAGAATCTGCTTAT-1	singlet	1	-1385.471359	-481.208122	-1209.506079	EMD2
GATAGCTAGAGAGAAC-1	singlet	1	-3038.79695	-968.3240528	-2647.515418	EMD2
GATAGCTAGCACTCGC-1	singlet	1	-1947.828732	-516.9773295	-1502.040255	EMD2
GATAGCTAGGACTGGT-1	singlet	1	-1675.548124	-472.7327236	-1217.954266	EMD2
GATAGCTAGGCTCCCA-1	singlet	1	-1648.880504	-415.7358479	-1133.935308	EMD2

GATAGCTAGTCCCTAA-1	singlet	1	-1331.569729	-477.3817097	-1193.338858	EMD2
GATAGCTCAACGTAAA-1	singlet	1	-74.15560537	-31.18167248	-80.93060641	EMD2
GATAGCTCATGGTACT-1	singlet	0	-1171.988235	-4527.775692	-4332.061743	EMD2
GATAGCTTCAGATTGC-1	singlet	1	-2779.602148	-785.1848515	-2270.478113	EMD2
GATAGCTTCCCGAGAC-1	singlet	1	-641.3834406	-226.9391724	-388.1170275	EMD2
GATCACAAGAGCATCG-1	singlet	1	-824.0755249	-293.0793542	-769.0481999	EMD2
GATCACAAGCATGGGT-1	singlet	0	-484.5734384	-1729.213717	-1322.650768	EMD2
GATCACAAGCCGGAAT-1	singlet	0	-580.8941082	-2228.5751	-2041.60516	EMD2
GATCACAAGTCGCTAT-1	singlet	1	-349.3923879	-149.1673825	-318.6609247	EMD2
GATCACAGTAGTTCCA-1	singlet	0	-168.2018099	-558.7920959	-541.5729772	EMD2
GATCACAGTCTACACA-1	singlet	1	-4217.551846	-1376.888566	-3486.138994	EMD2
GATCACAGTGTCTTCC-1	singlet	0	-1385.681544	-4221.100843	-4152.030845	EMD2
GATCACAGTTATGTCG-1	singlet	1	-211.2728777	-71.64101863	-151.0322423	EMD2
GATCACATCCTCACTG-1	singlet	1	-1108.149243	-338.708987	-736.4892509	EMD2
GATCACATCCTCCACA-1	singlet	0	-222.4107093	-569.33873	-469.1532828	EMD2
GATCAGTAGCAAACAT-1	singlet	1	-262.1309121	-109.3151027	-190.9623171	EMD2
GATCAGTAGTAGAATC-1	singlet	1	-21.57375935	-10.34242073	-13.41175099	EMD2
GATCAGTAGTCTCTGA-1	singlet	1	-360.6885705	-171.0204405	-344.5052405	EMD2
GATCAGTAGTTACGGG-1	singlet	0	-284.5255596	-946.7303844	-816.6358996	EMD2
GATCAGTCAATTGCGT-1	singlet	1	-109.1256318	-43.41788453	-76.12838744	EMD2
GATCAGTCACCCAATA-1	singlet	1	-3026.577001	-1048.018599	-2743.358509	EMD2
GATCAGTCATGGAACG-1	singlet	1	-1455.975599	-406.3054203	-1204.982395	EMD2
GATCAGTCATGTCGTA-1	singlet	2	-2533.096141	-1993.635425	-824.5826656	EMD2
GATCAGTGTCCAACGC-1	singlet	1	-1641.684525	-680.0966204	-1332.750485	EMD2
GATCAGTTCCGATAGT-1	singlet	1	-112.1617596	-34.79485337	-76.76194041	EMD2
GATCAGTTCTTGTTAC-1	singlet	1	-868.6400173	-323.2722901	-771.9756573	EMD2
GATCATGAGCGCCCAT-1	singlet	2	-389.8820543	-349.7517569	-127.3219088	EMD2
GATCATGAGGCTTAGG-1	singlet	1	-234.7048369	-96.43162507	-210.3018405	EMD2
GATCATGCAAATGGAT-1	singlet	2	-2284.207241	-1892.287306	-660.6912562	EMD2
GATCATGCACAGAGAC-1	singlet	1	-1646.94964	-608.0662895	-1508.859868	EMD2
GATCATGCACATTGTG-1	singlet	1	-2143.925655	-676.5447337	-1957.717759	EMD2
GATCATGCAGGCAATG-1	singlet	2	-532.0039483	-429.0868389	-188.1477541	EMD2
GATCATGCAGGTGTTT-1	singlet	0	-23.67758258	-90.0892464	-67.25311111	EMD2
GATCATGCATACAGCT-1	singlet	1	-203.9271187	-90.32692391	-177.3930731	EMD2
GATCATGGTCGATGCC-1	singlet	1	-334.6166719	-146.624366	-269.1461635	EMD2
GATCATGGTCGTGGTC-1	singlet	1	-530.6451598	-197.2986808	-472.9506745	EMD2
GATCATGGTGGTCCGT-1	singlet	1	-1595.777357	-427.9229723	-1335.993372	EMD2
GATCCCTAGCGCAATG-1	singlet	2	-4100.700815	-3512.988996	-1465.202153	EMD2
GATCCCTCACCGGAAA-1	singlet	0	-126.6003412	-446.5845585	-430.6284651	EMD2
GATCCCTCAGGAAGTC-1	singlet	1	-190.9731807	-67.83900561	-152.1634792	EMD2
GATCCCTCAGTAGATA-1	singlet	1	-4288.962858	-1306.82143	-3285.985826	EMD2
GATCCCTGTACAGGTG-1	singlet	1	-196.7498736	-64.80833038	-163.1367294	EMD2
GATCCCTGTCATCGCG-1	singlet	1	-996.012977	-406.1141443	-902.5431945	EMD2
GATCCCTTCATTCTTG-1	singlet	0	-853.1628095	-2596.925902	-2466.027706	EMD2
GATCCCTTCGTGGCTG-1	singlet	0	-458.1200203	-1586.998268	-1477.095698	EMD2
GATCGTAAGTAGGTTA-1	singlet	0	-421.9447053	-1440.995349	-1149.313088	EMD2
GATCGTAAGTGCACAG-1	singlet	0	-974.3463352	-2856.375709	-2832.537356	EMD2
GATCGTACAACCGTGC-1	singlet	1	-3126.743081	-1034.724323	-2458.641941	EMD2
GATCGTACAAGTGACG-1	singlet	1	-1699.605808	-502.7911573	-1416.80454	EMD2
GATCGTAGTGACTAAA-1	singlet	1	-103.6330873	-59.3641822	-90.14875566	EMD2
GATCGTAGTTAGAAAC-1	doublet	45293	-1258.357263	-659.3570898	-938.5516125	EMD2
GATCGTATCATTGTTC-1	singlet	1	-272.5671396	-116.7843229	-223.8334149	EMD2
GATCGTATCTGTGCAA-1	singlet	1	-221.0798269	-80.08952052	-204.9467143	EMD2
GATGACTAGACCGTTT-1	doublet	1/0	-501.0190587	-301.6205971	-519.9998332	EMD2
GATGACTAGACGCCCT-1	singlet	2	-2301.55251	-1726.757277	-760.430312	EMD2
GATGACTAGAGTCTTC-1	singlet	2	-3193.82298	-2902.506002	-1171.875026	EMD2
GATGACTCAGTCTACA-1	singlet	1	-230.0145063	-95.46160157	-182.2868006	EMD2
GATGACTGTACTGAGG-1	singlet	1	-1949.888318	-529.5095589	-1497.204177	EMD2
GATGACTGTATACGGG-1	singlet	2	-753.9967067	-774.821733	-298.3391673	EMD2
GATGACTTCAATCCGA-1	singlet	1	-731.5543396	-278.1437526	-581.0351778	EMD2
GATGAGGCAGCAGAAC-1	singlet	2	-499.5341896	-471.031168	-196.1086648	EMD2
GATGAGGCAGGACTTT-1	singlet	2	-1157.516305	-1004.589786	-478.9450756	EMD2
GATGAGGCAGTATTCG-1	singlet	1	-1566.341036	-527.594017	-1196.597489	EMD2
GATGATCAGCCGCTTG-1	singlet	0	-363.2290252	-990.3595816	-895.8772536	EMD2
GATGATCCACACTTAG-1	singlet	1	-268.1902277	-119.6879579	-220.9984255	EMD2
GATGATCGTAGTGCGA-1	singlet	1	-1167.017873	-372.5865636	-870.8872368	EMD2
GATGATCGTCCTTTGC-1	singlet	2	-821.4069852	-709.9607208	-322.2226288	EMD2
GATGATCGTCTCGGGT-1	singlet	0	-267.2759763	-795.937771	-777.6731263	EMD2
GATGATCGTGAGCCAA-1	singlet	1	-289.9086074	-102.6733578	-252.6258897	EMD2
GATGATCTCACCCTGT-1	singlet	0	-1218.702947	-4086.790797	-4019.469971	EMD2
GATGATCTCACCGGGT-1	singlet	2	-2482.265141	-2040.852018	-939.4963245	EMD2
GATGATCTCTCTTGCG-1	singlet	1	-276.6894756	-106.0082645	-233.4054647	EMD2
GATGCTAAGAGACAAG-1	singlet	2	-839.5048732	-811.1682817	-246.8288257	EMD2
GATGCTAAGCCTCTCT-1	singlet	1	-1163.289868	-468.7011043	-1009.896497	EMD2
GATGCTAAGGGAGAAT-1	singlet	0	-770.5940303	-2708.180304	-2278.180862	EMD2
GATGCTAAGGTTAGTA-1	singlet	1	-468.5307237	-169.1652494	-350.8050924	EMD2
GATGCTACACGGTAGA-1	singlet	1	-378.1577537	-174.5369827	-312.4266602	EMD2
GATGCTAGTACAGTTC-1	singlet	2	-600.1581028	-557.7517726	-220.4984219	EMD2
GATGCTAGTCCATAGT-1	singlet	2	-57.61875976	-31.31074272	-19.97946507	EMD2
GATGCTAGTGAGATCG-1	singlet	0	-350.5723219	-1085.143291	-1008.464172	EMD2
GATGCTAGTGCCAAGA-1	singlet	1	-2105.01932	-565.3855896	-1875.325862	EMD2
GATGCTAGTGGCTAGA-1	singlet	1	-367.8190296	-123.9091327	-290.9232039	EMD2
GATGCTATCCAGCAAT-1	singlet	2	-438.6953491	-494.0667709	-192.9657379	EMD2
GATGCTATCGACCCAG-1	singlet	2	-4053.676277	-3280.541783	-1518.146039	EMD2
GATGGAGAGGTTGACG-1	doublet	0/2	-2177.995441	-4176.045908	-3272.654643	EMD2
GATGGAGAGTAACCTC-1	singlet	2	-1052.267734	-892.8357142	-331.9305896	EMD2
GATGGAGAGTGAATAC-1	singlet	2	-1349.712614	-1307.14881	-492.4026101	EMD2
GATGGAGCAAACTGCT-1	singlet	1	-1973.784628	-631.5818042	-1684.866042	EMD2
GATGGAGCACCCTCTA-1	singlet	0	-502.6670617	-1643.300291	-1479.247404	EMD2
GATGGAGCACGATTCA-1	singlet	1	-428.8390005	-157.9434933	-339.8953139	EMD2
GATGGAGCAGTCAACT-1	singlet	0	-96.95581432	-363.8379037	-287.4731411	EMD2
GATGGAGGTATTCCTT-1	unassigned	0	-1.705046493	-3.764528126	-6.184388435	EMD2
GATGGAGGTCCATAGT-1	singlet	1	-321.5850288	-138.5972132	-250.2199017	EMD2
GATGGAGGTGCTCCGA-1	singlet	1	-408.3432172	-88.73983331	-332.6771227	EMD2
GATGTTGAGCACAAAT-1	singlet	2	-1353.325217	-1369.199062	-492.7066452	EMD2
GATGTTGAGCCTGGAA-1	singlet	2	-3277.620014	-2700.511197	-938.5341616	EMD2
GATGTTGAGCGACAGT-1	singlet	1	-305.7678741	-115.1444259	-266.9868664	EMD2
GATGTTGCAATGGCCC-1	singlet	0	-310.8859157	-837.9388989	-814.087582	EMD2
GATGTTGCACTGGCCA-1	singlet	1	-290.7322334	-106.8499267	-239.4355932	EMD2
GATGTTGCATACAGAA-1	singlet	0	-279.7562462	-1102.856027	-935.6050292	EMD2
GATGTTGGTCTAACTG-1	singlet	1	-269.2407001	-96.14225629	-224.7789626	EMD2
GATGTTGTCCGCACGA-1	singlet	0	-110.6596912	-274.6404824	-286.3785261	EMD2
GATGTTGTCGAGAAAT-1	singlet	0	-1470.387288	-5142.651787	-4800.610086	EMD2
GATGTTGTCTTTCGAT-1	singlet	0	-387.15981	-1121.841675	-1027.427304	EMD2
GATTCGAAGAAGCGGG-1	singlet	1	-3252.179445	-1000.102854	-2317.64929	EMD2
GATTCGAAGCCTCAAT-1	singlet	0	-964.1984873	-2907.453725	-2759.080983	EMD2
GATTCGACAGCTCGGT-1	singlet	0	-1696.563066	-5597.263473	-5387.684884	EMD2
GATTCGAGTCCGGATC-1	singlet	1	-5168.160285	-1552.004337	-3910.773229	EMD2
GATTCGAGTTTGGCTA-1	singlet	1	-449.8298466	-168.2712382	-304.0310222	EMD2
GATTCGATCACCATCC-1	singlet	1	-475.2932382	-218.5662935	-425.0244869	EMD2

GATTCGATCCTCATAT-1	singlet	0	-680.7620786	-2150.156915	-1999.955459	EMD2
GATTCGATCGATGGAG-1	singlet	1	-21.82687164	-4.325689577	-16.74458059	EMD2
GATTCGATCGTGCATA-1	doublet	0/1	-337.6135626	-485.9713195	-537.5722616	EMD2
GATTCGATCTCTGACC-1	singlet	0	-1019.228144	-3067.32326	-2958.981606	EMD2
GATTCTTAGAGTACCG-1	singlet	2	-304.3678965	-292.5811165	-115.6876645	EMD2
GATTCTTAGTTGGAAT-1	singlet	1	-2524.33277	-449.9947336	-1468.943757	EMD2
GATTCTTCAACCAGAG-1	singlet	1	-481.1301455	-153.2972765	-399.2751361	EMD2
GATTCTTCAACGACAG-1	singlet	2	-1300.843994	-1163.34392	-438.0418167	EMD2
GATTCTTCAAGTGGAC-1	singlet	2	-687.9202631	-588.5324756	-294.6508697	EMD2
GATTCTTCAATAGTGA-1	singlet	2	-1532.553547	-1345.004351	-468.4295641	EMD2
GATTCTTCACCAAAGG-1	singlet	1	-462.0176974	-219.7016794	-387.2426332	EMD2
GATTCTTCACCCTAGG-1	singlet	1	-462.7248111	-123.1582319	-315.0316494	EMD2
GATTCTTCATTGACTG-1	singlet	1	-9880.756716	-2922.285268	-7542.511366	EMD2
GATTCTTGTCACTAGT-1	singlet	0	-1298.498752	-4423.526533	-4207.389272	EMD2
GATTCTTGTTCCCACT-1	singlet	0	-1034.113877	-3120.681704	-2973.738706	EMD2
GATTCTTTCATCACAG-1	singlet	2	-7158.32322	-7288.563742	-3448.445161	EMD2
GATTCTTTCCAAATGC-1	singlet	1	-3877.121379	-1172.688146	-3122.862846	EMD2
GATTGGTAGGCTTAAA-1	singlet	0	-54.48421806	-192.5493726	-192.5826606	EMD2
GATTGGTCAATTTCTC-1	singlet	1	-564.4362065	-230.2063294	-432.9233023	EMD2
GATTGGTGTAAGATCA-1	singlet	2	-1379.965588	-1364.390824	-512.3872014	EMD2
GATTGGTGTAGTCGGA-1	singlet	1	-4017.843541	-1281.526273	-3106.586778	EMD2
GATTGGTGTCAAGCGA-1	singlet	2	-1144.452879	-1339.970501	-444.1658354	EMD2
GATTGGTGTCGCAGTC-1	singlet	2	-577.4069381	-528.2230007	-252.5336666	EMD2
GATTGGTGTCTTTCAT-1	singlet	1	-1481.093241	-481.9743071	-1031.7221	EMD2
GATTGGTGTGGAGAAA-1	singlet	1	-2220.63934	-742.1020483	-1724.669153	EMD2
GATTGGTGTTCCTAAG-1	singlet	2	-2416.208463	-2142.602784	-919.2320674	EMD2
GATTGGTGTTCGGCGT-1	singlet	1	-382.0541025	-153.0367209	-326.8985745	EMD2
GATTGGTTCACATACG-1	singlet	1	-2674.649343	-814.3969662	-2241.31881	EMD2
GATTGGTTCCGGTAAT-1	singlet	1	-272.4215733	-116.4713688	-235.0167239	EMD2
GATTGGTTCGCGGTAC-1	singlet	1	-556.9117832	-145.9542118	-502.4813542	EMD2
GATTTCTAGATTGACA-1	singlet	1	-1577.429586	-507.5189433	-1365.273352	EMD2
GATTTCTAGTGCGTCC-1	singlet	0	-391.5711153	-1285.544428	-1134.429271	EMD2
GATTTCTAGTTAGTGA-1	singlet	0	-612.3713315	-1764.389077	-1668.387498	EMD2
GATTTCTGTAACCAGG-1	singlet	1	-740.284976	-286.7377513	-587.284544	EMD2
GATTTCTGTAGGCTCC-1	singlet	0	-174.3013307	-412.620994	-417.009494	EMD2
GATTTCTGTCGTCAGC-1	singlet	2	-780.2781852	-673.4923525	-277.6201449	EMD2
GATTTCTGTGCATACT-1	singlet	0	-1185.806928	-3655.223393	-3365.134086	EMD2
GATTTCTGTTCCGCAG-1	singlet	0	-130.5514956	-319.6954996	-283.3977949	EMD2
GATTTCTTCAGCTGAT-1	singlet	1	-515.5398788	-219.3058942	-493.7418949	EMD2
GATTTCTTCATCCCGT-1	singlet	1	-518.9040122	-212.9270733	-425.2842851	EMD2
GATTTCTTCATCCTAT-1	singlet	2	-3227.905931	-2655.771804	-1149.097664	EMD2
GATTTCTTCCGGCAGT-1	singlet	0	-845.1418035	-2599.764856	-2437.982935	EMD2
GATTTCTTCCGTTGGG-1	singlet	0	-275.17509	-742.9917401	-714.2133846	EMD2
GATTTCTTCGAACGGA-1	singlet	1	-394.0247434	-164.8200733	-315.2242647	EMD2
GCAACATAGAGTCGAC-1	singlet	1	-1044.1975	-242.9536381	-816.2772576	EMD2
GCAACATAGATAACAC-1	singlet	2	-1430.367142	-1164.718152	-567.4593029	EMD2
GCAACATAGTGGTTGG-1	singlet	0	-260.5439433	-731.1886102	-694.3128132	EMD2
GCAACATCACACCGAC-1	singlet	2	-1559.29027	-1404.076657	-541.2900321	EMD2
GCAACATCACTATCGA-1	singlet	1	-2581.390433	-779.5169608	-2164.328739	EMD2
GCAACATGTCAATCTG-1	singlet	1	-1059.594443	-386.7403429	-892.3170752	EMD2
GCAACATGTCGTACAT-1	singlet	2	-4164.25893	-3334.615974	-1567.543383	EMD2
GCAACATGTCTAGATC-1	singlet	0	-963.4333645	-2376.605926	-2201.045464	EMD2
GCAACATTCAACCTCC-1	singlet	0	-2313.902971	-5931.960977	-5838.867496	EMD2
GCAACATTCACCCATC-1	singlet	0	-106.9663461	-303.6723198	-292.7681553	EMD2
GCAACCGAGACTGGGT-1	singlet	1	-1696.449094	-444.7833298	-1293.225566	EMD2
GCAACCGAGCATCGAG-1	singlet	2	-517.9228325	-463.7916234	-204.1047337	EMD2
GCAACCGAGCGTGAGT-1	singlet	1	-990.5317766	-314.4901913	-837.5735368	EMD2
GCAACCGAGTGGTCAG-1	doublet	0/2	-885.3110008	-1496.668991	-1123.200308	EMD2
GCAACCGCAGTTTCAG-1	singlet	1	-339.1916717	-114.8345869	-265.5680707	EMD2
GCAACCGGTAACCCTA-1	singlet	1	-1038.29524	-358.4092696	-867.6813348	EMD2
GCAACCGGTACCGTGC-1	singlet	2	-3089.502088	-3164.477182	-744.6129416	EMD2
GCAACCGGTATTGCCA-1	singlet	1	-1289.039152	-396.4152403	-1130.564631	EMD2
GCAACCGGTTAGTCGT-1	singlet	1	-1612.883228	-517.5123081	-1286.760347	EMD2
GCAACCGGTTGCCGAC-1	singlet	1	-27.82573101	-13.59239891	-33.0099529	EMD2
GCAACCGTCAGCGCGT-1	singlet	0	-161.5216609	-451.1454993	-450.5873219	EMD2
GCAACCGTCTGCGGCA-1	singlet	1	-443.5609629	-156.6011574	-317.4248472	EMD2
GCACATAAGACCTCCG-1	singlet	1	-2414.285086	-610.1331143	-1994.625608	EMD2
GCACATAAGAGTAACT-1	singlet	1	-124.5469562	-45.93041634	-81.86560812	EMD2
GCACATAAGCAGCAGT-1	singlet	0	-910.4271971	-2794.209025	-2566.31953	EMD2
GCACATAAGCGCCTCA-1	singlet	1	-245.3040649	-86.83015403	-184.5894228	EMD2
GCACATAAGGTCGTAG-1	singlet	2	-685.5400401	-641.4700111	-301.3767935	EMD2
GCACATAAGTAAATGC-1	singlet	1	-2817.865095	-916.6868359	-2088.799729	EMD2
GCACATAAGTATTGCC-1	singlet	1	-5436.796111	-1552.302066	-3925.635536	EMD2
GCACATACACGTACTA-1	singlet	0	-1851.061425	-7132.368187	-6670.874446	EMD2
GCACATACATACAGAA-1	singlet	1	-2936.843867	-1068.490151	-2456.858205	EMD2
GCACATACATCGTTCC-1	singlet	1	-325.5568863	-101.7346348	-233.4449115	EMD2
GCACATACATGGCCCA-1	singlet	1	-1340.139686	-399.0803951	-1191.27164	EMD2
GCACATAGTGAGAACC-1	singlet	1	-2141.549081	-752.91521	-1576.010494	EMD2
GCACATAGTGAGAGGG-1	singlet	1	-517.7732421	-166.2897328	-339.8926473	EMD2
GCACATAGTGGTACAG-1	singlet	1	-1270.223868	-381.8182129	-956.6255092	EMD2
GCACATAGTTGTGGAG-1	singlet	1	-1021.828194	-354.7343658	-1029.582621	EMD2
GCACATATCAAGTGGG-1	singlet	1	-355.5617449	-110.2799704	-235.765765	EMD2
GCACATATCACTTCTA-1	singlet	1	-391.5250587	-138.2742687	-312.413696	EMD2
GCACATATCAGCCTTC-1	singlet	1	-1434.004658	-498.6980655	-1103.132705	EMD2
GCACATATCAGTCCGG-1	singlet	0	-840.1965799	-2621.586793	-2553.411075	EMD2
GCACATATCGGTATGT-1	singlet	2	-1680.308946	-1422.061234	-505.1312556	EMD2
GCACGGTAGAGCTGCA-1	singlet	0	-873.929911	-4102.252446	-4635.655884	EMD2
GCACGGTAGCAGGCTA-1	singlet	1	-3652.162927	-1002.360235	-3015.788722	EMD2
GCACGGTAGGATTCCT-1	singlet	0	-153.7512706	-411.1138799	-361.8767464	EMD2
GCACGGTCAAGGAGTC-1	singlet	1	-77.95146714	-25.90468354	-60.94428064	EMD2
GCACGGTCACAGTACT-1	singlet	2	-1008.919822	-976.3516003	-390.9775562	EMD2
GCACGGTCACCGTCGA-1	singlet	1	-505.6061778	-174.8154275	-433.4904666	EMD2
GCACGGTCACGAGGTA-1	singlet	1	-969.0283896	-325.4074308	-858.995609	EMD2
GCACGGTCAGAGCTAG-1	singlet	1	-299.1178384	-121.0395421	-293.8626672	EMD2
GCACGGTCAGAGGACT-1	singlet	1	-343.926948	-136.650913	-288.8689043	EMD2
GCACGGTCAGTCTCTC-1	doublet	45293	-2976.765482	-1755.449203	-1850.549326	EMD2
GCACGGTGTGGGAGAG-1	singlet	0	-339.8700147	-1207.523901	-1024.640844	EMD2
GCACGGTTCAGCAGAG-1	singlet	1	-462.9068891	-163.4839291	-386.5631171	EMD2
GCACGGTTCGAGGCAA-1	singlet	1	-2259.042548	-746.7730749	-1730.007304	EMD2
GCACGGTTCTCTCTAA-1	singlet	2	-1070.797743	-964.0997608	-392.4123653	EMD2
GCACGTGAGCATGGGT-1	singlet	1	-1779.851914	-636.4189065	-1493.590331	EMD2
GCACGTGAGCCTCAAT-1	singlet	1	-95.64352454	-22.85884354	-72.3308789	EMD2
GCACGTGAGCCTCAGC-1	singlet	2	-2465.874524	-2168.744461	-884.7462572	EMD2
GCACGTGAGGACTTCT-1	singlet	1	-3260.286194	-1036.740669	-2529.168385	EMD2
GCACGTGCAAACACGG-1	singlet	0	-1808.1224	-7204.53684	-6866.004296	EMD2
GCACGTGCAATTGCCA-1	singlet	1	-2137.681615	-667.2539414	-1822.511414	EMD2
GCACGTGCATCGAACT-1	singlet	1	-1597.961409	-528.039233	-1415.343128	EMD2
GCACGTGCATCTTTCA-1	singlet	1	-1607.009522	-461.7390849	-1173.337854	EMD2
GCACGTGGTCTTCGAA-1	singlet	1	-3126.185766	-894.1673773	-2350.684081	EMD2
GCACGTGGTGGTCTCG-1	singlet	2	-445.5078645	-357.1045274	-185.758081	EMD2

GCACGTGGTTAGGCCC-1	singlet	1	-894.0847325	-304.4881259	-684.5683647	EMD2
GCACGTGTCAAGCCGC-1	singlet	1	-1799.336977	-517.9800449	-1584.563139	EMD2
GCACGTGTCATGTCTT-1	singlet	1	-1225.972206	-359.8793438	-1055.259937	EMD2
GCACGTGTCCTACACC-1	singlet	1	-327.3205272	-88.37894601	-236.3826146	EMD2
GCACGTGTCCTACCGT-1	singlet	1	-272.5069625	-106.395464	-193.3533888	EMD2
GCACGTGTCGATACGT-1	singlet	1	-706.4174432	-263.8118623	-544.7265327	EMD2
GCACTAAAGGGCCTCT-1	doublet	1/0	-420.2061024	-245.3690052	-391.8940601	EMD2
GCACTAAAGTCGTTAC-1	singlet	2	-10920.29224	-9220.597425	-3608.785049	EMD2
GCACTAACAACCGCTG-1	singlet	1	-3822.928525	-862.1053487	-2830.661488	EMD2
GCACTAACAGCTGTTA-1	singlet	1	-264.656783	-104.3214603	-189.9487335	EMD2
GCACTAATCTTACCGC-1	singlet	2	-3896.9222	-3093.651444	-1116.576724	EMD2
GCAGCCAAGCATCAAA-1	singlet	1	-416.258197	-149.4838118	-337.7698007	EMD2
GCAGCCAAGGTGATAT-1	doublet	0/2	-2974.810132	-6188.049374	-4972.34221	EMD2
GCAGCCAAGTCCCGAC-1	singlet	0	-48.71024134	-87.5031674	-134.9400346	EMD2
GCAGCCACAGACCTGC-1	singlet	0	-311.4792128	-817.3739289	-850.360555	EMD2
GCAGCCATCGAGATGG-1	singlet	1	-2424.063991	-756.9294427	-1850.727765	EMD2
GCAGCTGAGATGCTTC-1	singlet	2	-927.2070612	-794.2054323	-349.7709306	EMD2
GCAGCTGAGATGGTAT-1	singlet	1	-490.5201478	-205.6458967	-386.8706762	EMD2
GCAGCTGAGCCACCGT-1	singlet	1	-2206.679545	-711.9181823	-1614.648468	EMD2
GCAGCTGAGGCTGAAC-1	singlet	1	-79.28650955	-38.35045315	-87.03155093	EMD2
GCAGCTGAGGCTGGAT-1	singlet	0	-1348.000948	-5729.378504	-5706.76869	EMD2
GCAGCTGAGGGCCCTT-1	doublet	1/0	-29.53736977	-26.79277103	-28.57972231	EMD2
GCAGCTGCACCTGATA-1	singlet	0	-615.5065338	-1675.364596	-1654.463768	EMD2
GCAGCTGCAGTGGCTC-1	singlet	1	-233.0025993	-89.44302878	-174.2061091	EMD2
GCAGCTGGTAAGCAAT-1	singlet	1	-890.1007484	-327.9165543	-717.5058824	EMD2
GCAGCTGGTACCCGAC-1	singlet	1	-1377.900776	-476.1402048	-1264.0389	EMD2
GCAGCTGGTAGGCAAC-1	singlet	2	-551.4758948	-457.2671042	-217.6514753	EMD2
GCAGCTGGTTACACAC-1	singlet	0	-878.5822787	-3990.025985	-3705.423649	EMD2
GCAGCTGTCAAAGAAC-1	doublet	0/1	-230.6044901	-233.287497	-304.7618207	EMD2
GCAGCTGTCGCAATTG-1	singlet	2	-898.051631	-965.5863671	-370.6048588	EMD2
GCAGCTGTCTTTACAC-1	singlet	0	-496.7183717	-1710.236252	-1658.935849	EMD2
GCAGGCTAGCCTTTGA-1	singlet	1	-1306.198617	-454.5813753	-1076.449909	EMD2
GCAGGCTCATCCGTTC-1	singlet	2	-4587.28318	-3738.373729	-1557.553399	EMD2
GCAGGCTGTACGATGG-1	singlet	1	-486.9956285	-157.1669846	-482.8884283	EMD2
GCAGGCTGTATAATGG-1	singlet	1	-3640.549727	-933.5443934	-2676.881881	EMD2
GCAGGCTGTCCCGCAA-1	singlet	1	-6190.930385	-1794.389899	-4697.256387	EMD2
GCAGGCTGTCGCGGTT-1	singlet	2	-4363.10676	-3530.608528	-1174.015465	EMD2
GCAGGCTGTGCCGTTG-1	singlet	1	-1551.172786	-519.4333822	-1153.483399	EMD2
GCAGGCTGTTGGACCC-1	unassigned	0/2	-5.66427211	-10.00503955	-7.36975416	EMD2
GCAGGCTGTTGGTGTT-1	singlet	0	-1327.158474	-5496.775407	-4993.012279	EMD2
GCAGGCTTCGTTCCCA-1	singlet	0	-908.0606274	-2821.411845	-2662.11288	EMD2
GCAGTTAAGCCGATAG-1	singlet	1	-2265.34258	-638.2293594	-1612.261962	EMD2
GCAGTTAAGGCATCTT-1	singlet	1	-246.3886595	-132.9686839	-199.0157363	EMD2
GCAGTTACAAATGAGT-1	singlet	1	-122.4059918	-36.79906482	-66.49994811	EMD2
GCAGTTACATAGGAGC-1	singlet	1	-1676.27852	-474.3507447	-1351.005195	EMD2
GCAGTTAGTCGAGCAA-1	singlet	1	-1442.765244	-494.410057	-1203.493486	EMD2
GCAGTTAGTTTACGTG-1	singlet	1	-121.4819625	-42.00871775	-102.1136711	EMD2
GCATCGGAGACATCCT-1	singlet	0	-110.0076063	-339.3667307	-327.8203495	EMD2
GCATCGGAGCATATGA-1	singlet	0	-9.205198092	-20.26595225	-14.09794756	EMD2
GCATCGGAGGGCCTCT-1	singlet	1	-2077.078928	-618.2321584	-1721.670897	EMD2
GCATCGGAGTGCACAG-1	singlet	2	-3137.887284	-2717.26223	-1020.563921	EMD2
GCATCGGAGTGCTAGG-1	singlet	2	-3025.069621	-2520.558233	-1120.478105	EMD2
GCATCGGCACCAGTTA-1	singlet	1	-416.749694	-180.5699411	-386.5694518	EMD2
GCATCGGCACGACGCT-1	singlet	0	-184.6802536	-621.9555558	-607.1362948	EMD2
GCATCGGCAGCCGGTT-1	singlet	1	-2098.92017	-636.5093142	-1677.803699	EMD2
GCATCGGCATCCGAGC-1	singlet	1	-2453.542433	-735.0974743	-1808.681507	EMD2
GCATCGGTCGCGTGAC-1	singlet	1	-2525.207861	-757.3055379	-1997.193039	EMD2
GCATCGGTCTACCCAC-1	singlet	0	-787.3194956	-2646.773624	-2508.034053	EMD2
GCATCTCAGACGGAAA-1	singlet	1	-481.0830875	-159.6324259	-402.7664043	EMD2
GCATCTCAGAGTGAAG-1	singlet	2	-2240.960062	-1820.039031	-790.7289801	EMD2
GCATCTCAGATTAGAC-1	singlet	1	-211.7303876	-73.16254952	-189.5398728	EMD2
GCATCTCAGGACGCTA-1	singlet	1	-183.8237226	-84.40701537	-146.7863169	EMD2
GCATCTCAGGTAAGTT-1	singlet	1	-709.1796877	-274.4309696	-604.5095764	EMD2
GCATCTCAGTTGGACG-1	singlet	1	-1978.098138	-627.2490502	-1621.501066	EMD2
GCATCTCCATAAGCGG-1	singlet	1	-912.3908694	-368.0338099	-752.5477956	EMD2
GCATCTCGTAGTCTGT-1	singlet	1	-1094.838847	-359.1804891	-976.6079044	EMD2
GCATCTCGTCTGTGTA-1	singlet	2	-1498.819632	-1279.328692	-538.5789932	EMD2
GCATCTCGTGCGTTTA-1	singlet	1	-3553.01361	-1092.637096	-2661.591232	EMD2
GCATCTCTCCGAGAAG-1	singlet	1	-2018.149885	-576.6584025	-1309.625042	EMD2
GCATCTCTCTATCGTT-1	singlet	1	-2063.338164	-679.8221483	-1923.475783	EMD2
GCATCTCTCTTTGCAT-1	singlet	1	-1460.640315	-427.3291404	-1144.664991	EMD2
GCATGATAGAGGTTAT-1	singlet	2	-339.4812202	-374.9207651	-180.5948594	EMD2
GCATGATAGATTACCC-1	singlet	2	-3090.198643	-2176.320996	-1282.518407	EMD2
GCATGATAGCCAAGTG-1	singlet	1	-1849.96933	-536.8223483	-1516.103673	EMD2
GCATGATCAAACTAAG-1	singlet	1	-2492.065844	-879.7316692	-1958.24368	EMD2
GCATGATCAAGGCCTC-1	singlet	1	-754.45288	-271.923253	-672.4247412	EMD2
GCATGATCACTCATAG-1	singlet	1	-1295.543914	-414.8376553	-1089.473608	EMD2
GCATGATCATGTCAGT-1	singlet	2	-945.0384772	-893.0700192	-312.6749229	EMD2
GCATGATGTGTAAATG-1	singlet	1	-606.0033173	-229.4509317	-467.9932118	EMD2
GCATGATGTTCATCGA-1	singlet	0	-584.1653466	-2179.000537	-2154.191433	EMD2
GCATGATTCAGCGGAA-1	singlet	1	-218.2865098	-92.62118725	-185.9092202	EMD2
GCATGATTCATCCTAT-1	singlet	1	-6256.319471	-2064.418432	-4946.396309	EMD2
GCATTAGAGCAGATAT-1	singlet	1	-3139.916472	-1019.935275	-2538.196296	EMD2
GCATTAGAGCCGAACA-1	singlet	1	-837.4947892	-209.8396732	-678.8979741	EMD2
GCATTAGAGCTAATCC-1	singlet	2	-3132.786353	-2785.98619	-884.1421891	EMD2
GCATTAGAGGTAAAGG-1	singlet	1	-75.96457098	-17.68945544	-42.5412673	EMD2
GCATTAGCAAAGCAAT-1	singlet	1	-955.5474073	-346.1097795	-768.9948147	EMD2
GCATTAGCAGTGCCTG-1	singlet	1	-628.7121224	-203.3570943	-487.0873419	EMD2
GCATTAGGTCACGCTG-1	singlet	1	-285.7799199	-145.9524676	-264.560036	EMD2
GCATTAGGTCCGCAGT-1	singlet	1	-329.8094907	-126.5370842	-274.0544238	EMD2
GCATTAGGTTGCCAAT-1	singlet	1	-161.2994691	-75.44365152	-149.1080965	EMD2
GCATTAGTCAGTCTTT-1	singlet	0	-398.1388268	-1475.195732	-1288.549417	EMD2
GCATTAGTCTCTTAAC-1	singlet	1	-219.3081571	-79.00052661	-178.1688256	EMD2
GCCAACGCAACAAGAT-1	singlet	1	-1470.72935	-526.2601033	-1274.065464	EMD2
GCCAACGCACTTGAGT-1	singlet	1	-2739.901566	-933.6190248	-2151.086659	EMD2
GCCAACGGTTTGGGAG-1	singlet	0	-1589.452686	-5521.082899	-5214.085166	EMD2
GCCAACGTCACTGCTC-1	singlet	1	-1679.049626	-570.4433112	-1349.154844	EMD2
GCCAACGTCATTCGTT-1	singlet	1	-2873.345406	-885.0710932	-2354.25834	EMD2
GCCAGCAAGAGCTGAC-1	unassigned	2/0	-2.516622092	-5.886188264	-2.132231957	EMD2
GCCAGCAAGGAGATAG-1	singlet	1	-745.4788338	-289.6690907	-577.239722	EMD2
GCCAGCAAGGTAAGGA-1	singlet	1	-538.3452187	-186.771257	-493.0772504	EMD2
GCCAGCAAGTGGTTCT-1	singlet	1	-299.409488	-124.9393653	-242.2276305	EMD2
GCCAGCACACCTGCAG-1	singlet	1	-185.2964595	-84.97983076	-212.0009762	EMD2
GCCAGCACAGAACTCT-1	unassigned	2	-10.57777029	-9.356500106	-6.229836867	EMD2
GCCAGCACATAACCCA-1	singlet	1	-2570.891166	-591.0764456	-2048.341089	EMD2
GCCAGCAGTGTCCGTG-1	singlet	0	-1127.966095	-3400.795168	-3280.168169	EMD2
GCCAGCAGTTGGAGGT-1	singlet	2	-1464.247095	-1190.834954	-603.126342	EMD2
GCCAGCATCCGAAATC-1	singlet	1	-637.9845167	-254.8713959	-535.5057153	EMD2
GCCAGCATCTAAGCCA-1	singlet	2	-3150.566259	-2740.835128	-1339.144427	EMD2
GCCAGGTAGAAGCGGG-1	singlet	1	-3701.269127	-1204.152335	-2889.836118	EMD2

GCCAGGTAGACATGCG-1	singlet	1	-507.2134796	-176.6989101	-496.0408249	EMD2
GCCAGGTAGGCCTTGC-1	doublet	45323	-4302.48706	-2658.56021	-1764.06061	EMD2
GCCAGGTAGTTCGGTT-1	singlet	2	-2926.727552	-2364.303363	-956.3152554	EMD2
GCCAGGTCAGAGGAAA-1	singlet	1	-1428.792331	-498.7661923	-1153.548577	EMD2
GCCAGGTCAGGTTACT-1	singlet	0	-212.8256822	-820.035125	-720.6917817	EMD2
GCCAGGTCATGGGATG-1	singlet	2	-2132.071039	-1811.066815	-577.1727456	EMD2
GCCAGGTGTAGCGTAG-1	singlet	1	-507.9658244	-167.0183734	-359.0180263	EMD2
GCCAGGTGTGTTAAAG-1	singlet	1	-1566.407018	-589.7196132	-1405.17932	EMD2
GCCAGGTTCTTACCGC-1	singlet	1	-65.92371218	-27.15927309	-74.04034861	EMD2
GCCAGTGAGAAGTCTA-1	singlet	1	-389.473711	-153.0595082	-339.7589251	EMD2
GCCAGTGAGAGGTTAT-1	doublet	2/0	-6580.448094	-8817.880541	-5539.665113	EMD2
GCCAGTGAGGGAGGAC-1	singlet	1	-1784.581263	-554.8936659	-1334.336328	EMD2
GCCAGTGCAATTGGTC-1	singlet	1	-2754.318613	-787.0706054	-2198.741463	EMD2
GCCAGTGCACAATGTC-1	singlet	0	-20.88010401	-58.77713996	-62.2506647	EMD2
GCCAGTGCACATTCGA-1	singlet	1	-739.5385729	-287.2209743	-676.9475593	EMD2
GCCAGTGCATTCTGTT-1	singlet	1	-354.1068233	-130.3183631	-286.7250799	EMD2
GCCAGTGCATTGACCA-1	singlet	1	-947.4251087	-354.7237941	-782.9512963	EMD2
GCCAGTGGTACAGAAT-1	singlet	0	-501.6392408	-1236.288341	-1242.303269	EMD2
GCCAGTGGTCACCGAC-1	singlet	0	-1283.646123	-4463.187681	-4540.42047	EMD2
GCCAGTGTCCTAGAGT-1	unassigned	45323	-36.1145094	-19.24517106	-14.96555292	EMD2
GCCAGTGTCGATGCAT-1	singlet	1	-414.0531222	-157.8797227	-303.9844948	EMD2
GCCAGTGTCGCTACAA-1	singlet	1	-68.25110432	-33.4224694	-59.87399777	EMD2
GCCAGTGTCGTGGGAA-1	singlet	1	-2657.073487	-787.5998611	-2292.971363	EMD2
GCCAGTGTCTCTGACC-1	singlet	1	-572.2511391	-234.7592554	-511.0526953	EMD2
GCCATGGCAAAGGATT-1	singlet	1	-2420.656511	-832.8408489	-2121.321501	EMD2
GCCATGGCAGCTTTCC-1	singlet	1	-690.5386403	-235.9188693	-619.193965	EMD2
GCCATGGCAGTTGTCA-1	singlet	1	-464.882342	-186.351784	-395.6297061	EMD2
GCCATGGTCCACGTGG-1	doublet	1/0	-3288.005151	-3205.549023	-4131.800819	EMD2
GCCATGGTCGCTTACC-1	singlet	0	-476.7506314	-1564.794686	-1281.868824	EMD2
GCCATGGTCTCGTGGG-1	singlet	0	-211.8554848	-756.4490412	-671.364674	EMD2
GCCATTCAGATGACAT-1	singlet	1	-3161.800444	-1154.07422	-2476.793372	EMD2
GCCATTCAGATTACCC-1	singlet	0	-1789.350848	-4944.327253	-4754.794581	EMD2
GCCATTCAGGGATCGT-1	singlet	1	-317.0819817	-137.8299082	-300.4937952	EMD2
GCCATTCAGTGAGTTA-1	singlet	1	-1292.552488	-417.3090156	-995.3329424	EMD2
GCCATTCCACTTGACA-1	singlet	2	-1822.727267	-1525.257361	-667.6757184	EMD2
GCCATTCGTAGTAAGT-1	singlet	1	-281.9419063	-95.46772133	-227.5006604	EMD2
GCCATTCGTTTCCCAC-1	doublet	45293	-31.76031146	-23.56891373	-30.85866695	EMD2
GCCATTCTCCGGTTCT-1	singlet	0	-1379.525133	-3984.544525	-3761.14468	EMD2
GCCATTCTCGACGAGA-1	singlet	0	-609.5790662	-1900.692982	-1829.581341	EMD2
GCCCAGAAGAGATTCA-1	singlet	2	-834.1322182	-723.068207	-330.8087266	EMD2
GCCCAGAAGATGACCG-1	singlet	1	-1974.513537	-664.6681779	-1608.139201	EMD2
GCCCAGAAGGAACGCT-1	singlet	1	-1818.043257	-525.5364291	-1550.508252	EMD2
GCCCAGAAGGCACCAA-1	singlet	0	-538.5992378	-1627.060509	-1359.120002	EMD2
GCCCAGAAGGCGATAC-1	singlet	1	-2968.474813	-715.8526194	-2063.253296	EMD2
GCCCAGAAGTCCCAGC-1	singlet	1	-1661.261602	-528.845079	-1659.810599	EMD2
GCCCAGACAAAGGGCT-1	singlet	0	-271.6273881	-850.3749647	-751.2706016	EMD2
GCCCAGACATGGCTAT-1	singlet	1	-4791.943559	-1421.885817	-3552.732799	EMD2
GCCCAGATCTGCGGCA-1	singlet	1	-554.8695011	-214.1716342	-490.1214813	EMD2
GCCCGAAAGCAAACAT-1	doublet	1/0	-364.2829852	-198.5080172	-340.7681633	EMD2
GCCCGAAAGGATGTTA-1	singlet	2	-424.363256	-380.6454726	-168.0214386	EMD2
GCCCGAAAGGTTTGAA-1	singlet	0	-119.9700699	-439.1281517	-368.4131172	EMD2
GCCCGAACAAGTGGGT-1	singlet	1	-549.8920395	-224.5865174	-486.1399221	EMD2
GCCCGAACATGTCAGT-1	singlet	1	-800.9623057	-280.2975223	-586.8919938	EMD2
GCCCGAAGTTAGAAAC-1	singlet	0	-28.62820467	-67.41052803	-75.37781749	EMD2
GCCCGAATCATACGGT-1	singlet	1	-2303.065311	-612.5892778	-1869.647921	EMD2
GCCCGAATCTCCTGCA-1	singlet	2	-547.2583229	-449.1582979	-253.1350144	EMD2
GCCCGAATCTCTGAGA-1	singlet	1	-2569.304234	-609.1187254	-1803.142645	EMD2
GCCGATGAGGGTAATT-1	singlet	1	-209.2058956	-85.42864891	-176.6626929	EMD2
GCCGATGAGTAGACAT-1	singlet	1	-792.1855903	-240.1707325	-756.8305328	EMD2
GCCGATGCAAATGCTC-1	singlet	2	-356.3766407	-436.4038137	-176.9143215	EMD2
GCCGATGCAACAAGAT-1	singlet	1	-885.5192965	-307.9632727	-674.7382913	EMD2
GCCGATGCAGACATCT-1	singlet	0	-1078.392031	-3157.291237	-3016.199863	EMD2
GCCGATGGTAACAAGT-1	singlet	2	-1219.543538	-1108.126256	-486.0728343	EMD2
GCCGATGGTAGAATGT-1	singlet	2	-199.5852051	-177.2210564	-75.80539771	EMD2
GCCGTGAAGAAGTGTT-1	singlet	1	-245.2614095	-74.6412892	-194.3004885	EMD2
GCCGTGACACGGCCAT-1	singlet	2	-3186.039756	-2898.125259	-846.1510074	EMD2
GCCGTGACAGCTTTGA-1	singlet	0	-34.51548792	-107.820814	-120.8023385	EMD2
GCCGTGACAGTAGTGG-1	singlet	1	-401.9772373	-151.5039239	-290.2013852	EMD2
GCCGTGAGTGGTCTTA-1	singlet	1	-891.6727166	-304.8110649	-793.7372272	EMD2
GCCGTGAGTTGACTAC-1	singlet	1	-1476.590199	-434.4567876	-1256.482867	EMD2
GCCGTGATCCCGTGTT-1	singlet	2	-390.877152	-410.8293947	-178.4739039	EMD2
GCCGTGATCCTACGGG-1	singlet	1	-2645.777281	-900.6381131	-2173.128441	EMD2
GCCGTGATCTCCTGCA-1	singlet	2	-1468.322753	-1118.124577	-379.4638207	EMD2
GCCTGTTAGACGAAGA-1	doublet	0/2	-1397.218592	-2050.575105	-1711.616415	EMD2
GCCTGTTAGGGAGGTG-1	singlet	1	-4361.022843	-1136.106415	-3570.685486	EMD2
GCCTGTTCAAATACGA-1	singlet	1	-844.7038995	-332.0431754	-607.0621572	EMD2
GCCTGTTCAATACGCT-1	singlet	1	-584.463796	-184.4133372	-442.3840553	EMD2
GCCTGTTCAGAGGCTA-1	singlet	1	-1447.124431	-505.8837173	-1202.012996	EMD2
GCCTGTTCAGGCGTTC-1	singlet	0	-756.6043357	-2169.699918	-2222.904144	EMD2
GCCTGTTCAGTAGTTC-1	singlet	1	-790.1257153	-337.1434311	-741.4497115	EMD2
GCCTGTTCATGTGGCC-1	singlet	1	-1280.930219	-507.3566047	-1147.109064	EMD2
GCCTGTTGTACTCAAC-1	singlet	1	-379.9893072	-138.8684256	-326.598089	EMD2
GCCTGTTTCCCATAGA-1	singlet	1	-3319.779851	-1196.802225	-2813.94372	EMD2
GCCTGTTTCCGTCCTA-1	singlet	1	-11.07788824	-4.188343191	-17.15709285	EMD2
GCCTGTTTCGCGCCAA-1	singlet	0	-520.626971	-1641.237826	-1671.217795	EMD2
GCGAGAAAGGCGTCCT-1	singlet	1	-253.7221694	-104.0997463	-255.7956747	EMD2
GCGAGAAAGTAGCATA-1	singlet	1	-2676.913104	-843.3153654	-2261.659968	EMD2
GCGAGAAAGTATTCCG-1	singlet	0	-673.4905993	-2095.954211	-1995.209037	EMD2
GCGAGAAGTCAAGGCA-1	singlet	1	-357.8031168	-141.0844322	-320.122589	EMD2
GCGAGAATCGAGTGAG-1	singlet	2	-2489.912395	-2409.173539	-695.7022867	EMD2
GCGAGAATCGTACCTC-1	singlet	1	-1202.105662	-399.0408365	-915.0203284	EMD2
GCGAGAATCTAAGGAA-1	singlet	2	-3093.584954	-2926.600692	-940.6712308	EMD2
GCGAGAATCTCTCGAC-1	singlet	2	-371.0853087	-395.8399877	-154.2712739	EMD2
GCGATCGAGACCTTTG-1	singlet	0	-133.402784	-368.7221994	-352.4542227	EMD2
GCGATCGAGCCAGAGT-1	singlet	1	-1751.647714	-631.7413093	-1411.765619	EMD2
GCGATCGAGTGATTCC-1	singlet	2	-524.0973224	-484.4054178	-182.9961166	EMD2
GCGATCGCAAGCTGCC-1	singlet	0	-891.9874765	-2718.237501	-2590.328283	EMD2
GCGATCGCACCGCTAG-1	singlet	2	-341.2259478	-375.6791749	-141.0698768	EMD2
GCGATCGCATCGTGCG-1	singlet	2	-778.7120669	-690.2527648	-337.4560375	EMD2
GCGATCGGTCCTATAG-1	singlet	2	-946.3133752	-970.253856	-272.1426302	EMD2
GCGATCGGTGATGAAT-1	singlet	1	-603.7073218	-210.7795045	-465.3565174	EMD2
GCGATCGTCACGGTCG-1	singlet	1	-477.7748939	-169.4389655	-389.2292983	EMD2
GCGGAAAAGCAGAAAG-1	singlet	1	-293.4576861	-91.05010537	-192.465112	EMD2
GCGGAAAAGGGTGAAA-1	singlet	1	-6406.672649	-1655.541817	-5305.140921	EMD2
GCGGAAACAGCACAAG-1	singlet	2	-4862.550641	-4108.582123	-1363.823313	EMD2
GCGGAAACAGGTAGTG-1	singlet	1	-2928.980538	-767.3887239	-2335.709045	EMD2
GCGGAAAGTAATGATG-1	singlet	1	-4465.087958	-1535.986608	-3379.949591	EMD2
GCGGAAAGTCTACAAC-1	singlet	1	-1183.026212	-460.1157853	-1011.358972	EMD2
GCGGAAATCCTAAGTG-1	singlet	2	-421.688034	-383.0171493	-176.2018679	EMD2
GCGGATCAGAAGCGGG-1	singlet	1	-1643.683626	-557.5283366	-1350.638713	EMD2

GCGGATCAGAGCAGTC-1	singlet	1	-338.5960325	-125.849326	-311.6230703	EMD2
GCGGATCCAGCACAGA-1	singlet	2	-2371.044101	-2287.139235	-489.514617	EMD2
GCGGATCCATCGTTCC-1	singlet	0	-1501.229437	-6557.006519	-6022.347577	EMD2
GCGGATCGTAGACGTG-1	singlet	1	-495.2221447	-194.1563447	-383.0000719	EMD2
GCGGATCGTCATTCCC-1	singlet	2	-4676.872128	-3466.051414	-1382.273869	EMD2
GCGGATCGTCTAATCG-1	singlet	2	-1488.79729	-1413.476703	-488.3450841	EMD2
GCGGATCTCACCTTGC-1	singlet	1	-2798.247169	-881.2449295	-2051.147807	EMD2
GCGGATCTCATTGAGC-1	singlet	0	-19.07276791	-43.44459152	-32.20981776	EMD2
GCGGATCTCCAGTTCC-1	singlet	1	-220.5407915	-85.74667324	-153.6285017	EMD2
GCGGATCTCGAAACAA-1	singlet	1	-1056.555344	-381.7562156	-883.3358772	EMD2
GCGGATCTCTGACAGT-1	singlet	1	-818.6485965	-251.5231484	-603.8274648	EMD2
GCGTGCAAGACCTCAT-1	singlet	2	-869.5074954	-696.1714636	-273.9177331	EMD2
GCGTGCAAGAGAGAAC-1	singlet	1	-312.8322053	-123.5029567	-300.3751862	EMD2
GCGTGCAAGAGCAGTC-1	singlet	0	-1422.618515	-5935.040509	-5666.061598	EMD2
GCGTGCAAGCATAGGC-1	singlet	1	-2523.117638	-922.6061229	-2098.349017	EMD2
GCGTGCAAGCCGAATG-1	singlet	0	-492.5778485	-2797.006249	-2154.948514	EMD2
GCGTGCAAGGCGATAC-1	singlet	1	-1676.851577	-385.3116333	-1219.249704	EMD2
GCGTGCACACAATCTG-1	singlet	1	-1232.443179	-298.4298253	-1178.391173	EMD2
GCGTGCACACTGAGTT-1	singlet	1	-407.6033779	-154.3307719	-338.9242848	EMD2
GCGTGCACATAATCGC-1	singlet	1	-739.1236005	-244.8271327	-524.0176802	EMD2
GCGTGCACATGCCATA-1	singlet	1	-331.6924459	-102.5069557	-293.3238649	EMD2
GCGTGCAGTAGATCGG-1	singlet	0	-225.5932049	-747.5289066	-733.5879803	EMD2
GCGTGCAGTATGTCAC-1	doublet	2/0	-621.0331554	-785.9586275	-423.8403878	EMD2
GCGTGCAGTTCTAAGC-1	singlet	0	-138.787188	-493.0200317	-392.9741131	EMD2
GCGTGCATCAAGAATG-1	singlet	1	-662.2347006	-226.3515601	-467.7468557	EMD2
GCGTGCATCACATCAG-1	singlet	0	-255.5226925	-706.0151473	-672.3119085	EMD2
GCGTGCATCGAGAAGC-1	singlet	1	-1400.614392	-427.2209192	-1093.161681	EMD2
GCGTGCATCGGTAAGG-1	singlet	1	-742.4493465	-278.56067	-668.2299054	EMD2
GCGTGCATCGTAGTCA-1	singlet	2	-2674.876796	-2223.553863	-800.2362469	EMD2
GCGTGCATCTGAGAAA-1	singlet	0	-1425.507377	-4585.414774	-4579.5471	EMD2
GCGTTTCCAAACCATC-1	singlet	0	-5.26037565	-8.602863479	-18.68025676	EMD2
GCGTTTCCAGTGCCTG-1	singlet	2	-1242.4827	-1173.168267	-361.848459	EMD2
GCGTTTCCATCCAACA-1	singlet	1	-618.4076921	-241.6706104	-563.1840571	EMD2
GCGTTTCCATGCCGGT-1	singlet	1	-262.0697297	-124.7600667	-204.4735711	EMD2
GCGTTTCGTAGCTTTG-1	singlet	1	-939.8473898	-307.4848097	-678.7705344	EMD2
GCGTTTCGTCTGTGAT-1	singlet	2	-4381.8663	-3147.427454	-1748.964389	EMD2
GCGTTTCGTTTACGAC-1	singlet	0	-1109.793346	-3897.662744	-3320.799619	EMD2
GCGTTTCTCCTCAGAA-1	singlet	1	-254.1298027	-118.8305538	-251.4649141	EMD2
GCGTTTCTCTCGGTAA-1	singlet	2	-1929.05671	-1778.421143	-683.0261355	EMD2
GCTACAAAGAGTCAGC-1	singlet	1	-2585.13243	-842.0653774	-2029.908593	EMD2
GCTACAACAACCCTAA-1	singlet	1	-1969.998534	-629.82949	-1542.905887	EMD2
GCTACAACACACACTA-1	singlet	2	-357.2484993	-407.7491335	-174.9734904	EMD2
GCTACAACACGATTCA-1	singlet	0	-315.6177868	-880.2581098	-894.1569218	EMD2
GCTACAACATCGAGCC-1	singlet	2	-3135.290558	-2290.734809	-1304.311292	EMD2
GCTACAACATGGCCAC-1	singlet	2	-1124.628363	-869.7980019	-466.3180247	EMD2
GCTACAAGTAGGAGGG-1	singlet	1	-577.4941532	-188.9421114	-488.8206164	EMD2
GCTACAAGTCGTTATG-1	singlet	2	-1778.976572	-1339.412092	-545.6017319	EMD2
GCTACAAGTGAGATTA-1	singlet	2	-2621.770408	-2238.104544	-941.2201998	EMD2
GCTACAAGTGTTGCCG-1	singlet	1	-273.3244366	-100.19792	-221.2555254	EMD2
GCTACAATCAGCGCAC-1	singlet	1	-4450.827993	-1226.692581	-3407.190808	EMD2
GCTACAATCCGAGATT-1	singlet	1	-2723.159773	-915.1494053	-2113.544521	EMD2
GCTACAATCCTCACGT-1	unassigned	0/2	-18.16720626	-24.05939934	-19.56863139	EMD2
GCTACAATCGTCGGGT-1	singlet	1	-594.9910456	-200.7744456	-477.8902748	EMD2
GCTACAATCTGCTTTA-1	singlet	0	-143.8717991	-383.7316937	-370.8142002	EMD2
GCTACCTAGAGAGAAC-1	singlet	0	-124.3190918	-387.659825	-280.1852147	EMD2
GCTACCTCACTAGTAC-1	singlet	2	-952.5962732	-847.5256995	-329.8713393	EMD2
GCTACCTCACTGCGAC-1	singlet	0	-830.487626	-2631.623459	-2363.077206	EMD2
GCTACCTCAGAGTGAC-1	doublet	45323	-2164.185762	-1236.018086	-1234.955789	EMD2
GCTACCTTCATGACAC-1	singlet	1	-1450.822999	-483.07416	-1265.751085	EMD2
GCTACCTTCCATTCAT-1	singlet	2	-1585.463398	-1641.613787	-572.1520988	EMD2
GCTACCTTCCGCGAGT-1	singlet	1	-1013.345679	-330.9259666	-772.5389346	EMD2
GCTACCTTCGTGACTA-1	singlet	1	-1064.219657	-266.8048053	-864.2773367	EMD2
GCTACCTTCTCGTGGG-1	singlet	1	-453.6874852	-168.2660732	-409.2173209	EMD2
GCTCAAAAGGCCTGAA-1	singlet	1	-1605.096554	-696.8148027	-1406.807301	EMD2
GCTCAAAAGTACGAGC-1	singlet	1	-3108.143263	-989.2126114	-2467.152105	EMD2
GCTCAAACACCAAAGG-1	singlet	2	-4265.792649	-3598.53584	-1320.252193	EMD2
GCTCAAACACCTGATA-1	singlet	1	-942.9138124	-372.3527055	-748.4626825	EMD2
GCTCAAACAGAGAAAG-1	unassigned	2	-5.206417243	-6.418943118	-2.776230058	EMD2
GCTCAAACAGTAGGAC-1	singlet	1	-2215.530109	-770.3461683	-1735.307996	EMD2
GCTCAAAGTAGACGTG-1	singlet	0	-467.5905616	-1423.124569	-1454.049042	EMD2
GCTCAAAGTCAGGCAA-1	singlet	1	-1822.232526	-584.8741512	-1742.481415	EMD2
GCTCAAATCCTTCTGG-1	singlet	1	-1421.732626	-349.1223551	-1206.887874	EMD2
GCTCAAATCGTACCTC-1	singlet	1	-1502.163993	-428.4620924	-1272.670322	EMD2
GCTCAAATCTAACGGT-1	singlet	1	-2605.970821	-858.0139717	-2070.028685	EMD2
GCTGAATAGGAACATT-1	singlet	1	-2171.232562	-780.5652209	-1933.999397	EMD2
GCTGAATAGTAGCAAT-1	singlet	2	-682.5728085	-680.1893435	-270.6038872	EMD2
GCTGAATCAGACCTGC-1	singlet	0	-1337.00626	-2455.844941	-2606.25315	EMD2
GCTGAATCAGAGAATT-1	doublet	1/0	-11.97385987	-11.34123334	-21.16985262	EMD2
GCTGAATGTCTCAGGC-1	singlet	1	-2301.875057	-754.7986987	-1707.280704	EMD2
GCTGAATGTTCTCGCT-1	singlet	2	-222.7746871	-222.0985943	-101.9637884	EMD2
GCTGAATTCCTTGAAG-1	singlet	0	-536.0491382	-1844.056266	-1470.927087	EMD2
GCTGAATTCGCAGAGA-1	singlet	1	-493.3170902	-162.7382511	-317.2481801	EMD2
GCTGCAGAGAGCATAT-1	singlet	1	-2059.352629	-644.1308197	-1637.57787	EMD2
GCTGCAGAGCTCTTCC-1	singlet	1	-1874.541403	-564.9753656	-1646.639597	EMD2
GCTGCAGAGGTAGACC-1	singlet	1	-1263.234469	-257.1104185	-979.3576579	EMD2
GCTGCAGCAAGTGCAG-1	singlet	1	-1865.917399	-630.4476728	-1508.41373	EMD2
GCTGCAGCAATATCCG-1	singlet	2	-4018.044596	-3200.340838	-1254.053767	EMD2
GCTGCAGCATATAGCC-1	singlet	1	-1846.288079	-630.6970241	-1531.97883	EMD2
GCTGCAGCATCAACCA-1	singlet	1	-2729.689718	-911.6906595	-2406.912811	EMD2
GCTGCAGGTACAATAG-1	unassigned	1	-8.744385297	-2.825204848	-5.833395996	EMD2
GCTGCAGGTCGAAGCA-1	singlet	0	-732.2506494	-2251.246924	-2083.993099	EMD2
GCTGCAGGTCGTCAGC-1	singlet	2	-502.519914	-420.4743036	-174.0347406	EMD2
GCTGCAGGTTCGTGCG-1	singlet	2	-1650.619051	-1446.224407	-579.0955245	EMD2
GCTGCAGTCACTTCTA-1	singlet	1	-138.7675564	-57.36563673	-123.269398	EMD2
GCTGCAGTCGATCCAA-1	singlet	1	-4417.923667	-1404.238706	-3672.152669	EMD2
GCTGCAGTCTAGTACG-1	singlet	1	-2052.442099	-718.4972871	-1813.913522	EMD2
GCTGGGTAGACCAGAC-1	singlet	0	-1876.593123	-5900.313779	-5693.683787	EMD2
GCTGGGTAGCGACATG-1	singlet	1	-981.0725117	-357.8364431	-862.7001444	EMD2
GCTGGGTAGTCCCGGT-1	singlet	0	-520.4023312	-1430.00711	-1342.506144	EMD2
GCTGGGTAGTGGAATT-1	singlet	1	-505.7834935	-159.1051606	-398.0773399	EMD2
GCTGGGTCAAATGATG-1	doublet	45293	-156.9613406	-81.76370801	-95.13224229	EMD2
GCTGGGTGTTTCGTGA-1	singlet	1	-360.0714605	-137.7212357	-257.4970995	EMD2
GCTGGGTTCCGTGTGG-1	singlet	1	-338.0837265	-116.6051065	-271.1998407	EMD2
GCTTCACAGACATCCT-1	singlet	2	-665.4140822	-581.6088475	-216.5932877	EMD2
GCTTCACAGATTAGTG-1	singlet	0	-603.1693847	-2388.292373	-2352.49502	EMD2
GCTTCACCATGAATAG-1	singlet	1	-304.9593467	-114.6515214	-277.9641855	EMD2
GCTTCACCATTACGGT-1	singlet	2	-394.2593775	-379.5561151	-146.0815567	EMD2
GCTTCACGTAGCCCTG-1	doublet	0/2	-1435.575557	-2179.441195	-1504.262685	EMD2
GCTTCACGTAGTTAGA-1	singlet	2	-1610.995179	-1503.723883	-573.521263	EMD2
GCTTCACGTCCAAAGG-1	singlet	0	-626.7837995	-1886.194692	-1818.158454	EMD2

GCTTCACTCAACGCTA-1	singlet	1	-3427.386613	-999.629671	-2647.25531	EMD2
GCTTCACTCTGTCTCG-1	singlet	2	-570.2386211	-615.3260034	-227.9683501	EMD2
GCTTGGGCAAACCGGA-1	singlet	2	-1868.581401	-1739.924928	-556.7911916	EMD2
GCTTGGGCAACAGTGG-1	singlet	2	-1460.020158	-1322.912166	-554.6539817	EMD2
GCTTGGGCACAAGTTC-1	singlet	1	-290.8390657	-110.7923863	-223.7383783	EMD2
GCTTGGGCACTAACCA-1	singlet	1	-17.58085819	-10.08086921	-24.62656582	EMD2
GCTTGGGCAGCCGTCA-1	doublet	0/1	-687.5482331	-1169.150187	-1324.003672	EMD2
GCTTGGGCATGGAAGC-1	singlet	1	-2356.928089	-773.7228245	-1958.417432	EMD2
GCTTGGGGTCCACATA-1	singlet	2	-492.9175675	-468.2812283	-232.7630839	EMD2
GCTTGGGGTCCTTGTC-1	singlet	1	-459.8596407	-188.2745649	-415.8133384	EMD2
GCTTGGGGTCGAGCAA-1	singlet	2	-2389.362324	-1956.443119	-717.9669716	EMD2
GCTTGGGGTGACTGTT-1	singlet	2	-2260.962348	-1701.026032	-747.4571068	EMD2
GCTTGGGGTGGTCTCG-1	singlet	1	-1103.659357	-320.537377	-823.2554619	EMD2
GCTTGGGTCAGTGTTG-1	singlet	0	-361.2772467	-1491.700783	-1599.812408	EMD2
GCTTGGGTCGGACCAC-1	singlet	1	-1462.135607	-437.332276	-1076.767247	EMD2
GCTTGGGTCTGGGTCG-1	singlet	1	-1772.391256	-605.6170722	-1564.166025	EMD2
GCTTTCGCAAATTAGG-1	singlet	0	-576.433069	-1782.6221	-1753.4263	EMD2
GCTTTCGCAATACCCA-1	singlet	2	-1692.770632	-1402.29947	-647.7783041	EMD2
GCTTTCGCAGACGCTC-1	singlet	2	-494.5317156	-385.3173803	-177.6342168	EMD2
GCTTTCGGTTCTCACC-1	singlet	0	-1064.86827	-3692.079144	-3476.050131	EMD2
GCTTTCGTCTCCTGAC-1	singlet	1	-494.4564939	-189.5332619	-364.9644966	EMD2
GCTTTCGTCTTCCCGA-1	singlet	1	-3500.403151	-1384.426517	-2911.959222	EMD2
GGAACCCAGTCCGTCG-1	singlet	0	-327.7619342	-1206.013002	-1149.938778	EMD2
GGAACCCAGTGTAGTA-1	singlet	1	-3882.296023	-1349.238791	-3358.022026	EMD2
GGAACCCCACCACATA-1	singlet	1	-166.3072137	-68.49954446	-145.9605252	EMD2
GGAACCCCAGCATTGT-1	singlet	1	-210.1416533	-63.06077502	-157.0939778	EMD2
GGAACCCGTACCGCGT-1	singlet	1	-2165.045684	-794.191411	-1892.56685	EMD2
GGAACCCGTATGAGGC-1	singlet	0	-277.336588	-1170.056959	-966.1324295	EMD2
GGAACCCTCAGTGTCA-1	singlet	1	-3494.821147	-1008.702902	-2925.959388	EMD2
GGAACCCTCATCCCGT-1	singlet	1	-2806.814777	-739.3914267	-2046.410335	EMD2
GGAACCCTCCTCGCAT-1	singlet	1	-302.9249631	-144.5212744	-252.9376733	EMD2
GGAACCCTCGTGAGAG-1	unassigned	45293	-4.912103087	-2.925395035	-3.611542474	EMD2
GGAACCCTCGTTAGAC-1	singlet	2	-2919.239175	-2814.949112	-954.3704764	EMD2
GGAAGTGAGACGGTCA-1	singlet	1	-2477.91256	-468.3388013	-1631.207926	EMD2
GGAAGTGAGTCTAGAA-1	singlet	1	-677.191965	-215.7743038	-497.699893	EMD2
GGAAGTGCAAGTGGAC-1	singlet	1	-2153.694804	-723.8752942	-1776.315857	EMD2
GGAAGTGCAGAAGCGT-1	singlet	1	-377.3596159	-129.4504034	-323.6821141	EMD2
GGAAGTGCATACAGGG-1	singlet	0	-1045.806175	-3545.086824	-3283.33499	EMD2
GGAAGTGCATGGTACT-1	singlet	1	-2139.211068	-691.6175843	-1924.892408	EMD2
GGAAGTGGTGGTTCTA-1	singlet	0	-102.2947036	-395.2996328	-374.6735942	EMD2
GGAAGTGTCACAAGGG-1	singlet	1	-275.0392561	-128.6756734	-252.2796046	EMD2
GGAAGTGTCGGAATTC-1	singlet	0	-243.0475961	-626.6556034	-621.2125823	EMD2
GGAAGTGTCTGGTGCG-1	singlet	2	-2485.878284	-2268.98363	-766.5510172	EMD2
GGAATCTAGCGACCCT-1	singlet	2	-799.653065	-724.7953703	-293.7953512	EMD2
GGAATCTAGGCCCAAA-1	singlet	1	-438.4674627	-190.3203651	-374.6807779	EMD2
GGAATCTCAAGAGGCT-1	singlet	1	-4555.116033	-1316.221321	-3690.444579	EMD2
GGAATCTCAAGTATCC-1	singlet	1	-2282.830097	-727.4486549	-1884.510153	EMD2
GGAATCTCACCCAACG-1	singlet	1	-340.0882709	-124.5052462	-292.6774707	EMD2
GGAATCTCACTACTTT-1	singlet	1	-1032.338543	-348.1511589	-838.8890266	EMD2
GGAATCTCAGACGATG-1	singlet	2	-444.6062544	-473.9674062	-171.900933	EMD2
GGAATCTCATTCCTAT-1	singlet	1	-378.7180847	-159.1687951	-325.5838721	EMD2
GGAATCTGTGATCATC-1	singlet	1	-1563.548634	-529.8224048	-1275.596869	EMD2
GGAATCTGTGTACGCC-1	singlet	0	-1311.372668	-2933.934391	-3059.88885	EMD2
GGAATCTGTTAGGCCC-1	singlet	0	-896.5092031	-2390.454225	-2356.886326	EMD2
GGAATCTTCTCGCAGG-1	singlet	0	-174.5804081	-726.9180457	-578.614888	EMD2
GGAATGGAGAGCCGTA-1	singlet	0	-41.11462086	-212.4350595	-171.2111375	EMD2
GGAATGGAGCTTCGTA-1	singlet	2	-2210.940926	-1995.087543	-792.7456075	EMD2
GGAATGGAGGTTTGAA-1	singlet	1	-1566.616866	-401.4092635	-1133.199607	EMD2
GGAATGGCAGGTTCGC-1	singlet	1	-1473.398378	-540.4002416	-1095.343142	EMD2
GGAATGGGTGTGATGG-1	singlet	0	-589.3427622	-1925.455935	-1866.447425	EMD2
GGAATGGTCACCTCTG-1	singlet	0	-927.9983512	-3199.327497	-3304.814851	EMD2
GGAATGGTCCCTGTTG-1	singlet	1	-338.4597403	-123.2689901	-289.3129819	EMD2
GGAATGGTCTCTCAAT-1	singlet	1	-1039.182717	-414.5406976	-975.4001096	EMD2
GGACGTCAGACCAACG-1	singlet	2	-1690.05919	-1930.677154	-602.8756509	EMD2
GGACGTCAGACTGTTC-1	singlet	2	-682.709521	-678.3402413	-249.0378554	EMD2
GGACGTCAGCATGGGT-1	singlet	0	-219.6932644	-578.6685123	-549.4551675	EMD2
GGACGTCAGCGTGTCC-1	singlet	1	-330.7888599	-114.4510137	-245.6640791	EMD2
GGACGTCAGGATCATA-1	singlet	1	-822.2481215	-237.0755093	-580.1842804	EMD2
GGACGTCAGTCCCAAT-1	singlet	1	-3392.703081	-1101.169508	-2808.577714	EMD2
GGACGTCCAAAGGTTA-1	singlet	0	-346.5685054	-1064.700997	-1084.340692	EMD2
GGACGTCCAACAGCCC-1	singlet	2	-731.686051	-716.0813394	-277.4450257	EMD2
GGACGTCCAAGAGGTC-1	singlet	0	-882.1559802	-2608.849143	-2447.043962	EMD2
GGACGTCCAAGGATGC-1	singlet	1	-1109.23382	-364.1697559	-789.7714244	EMD2
GGACGTCCAAGTGGCA-1	doublet	45293	-5660.629867	-2635.064501	-3269.103511	EMD2
GGACGTCCACCTATCC-1	singlet	1	-5001.138947	-1553.448565	-3924.190415	EMD2
GGACGTCCATGCTGCG-1	singlet	0	-1677.508445	-5844.162955	-5343.475368	EMD2
GGACGTCGTCATCCCT-1	singlet	1	-2391.225464	-743.2129252	-1678.462763	EMD2
GGACGTCGTCTGTCAA-1	singlet	1	-137.3633413	-44.90440785	-108.3480345	EMD2
GGACGTCGTGGATTTC-1	singlet	2	-990.1810497	-993.2811202	-346.3392218	EMD2
GGACGTCGTTATGGTC-1	singlet	1	-819.8248302	-272.9358892	-686.6860927	EMD2
GGACGTCGTTCAGTAC-1	singlet	0	-498.7179776	-1690.998109	-1618.046031	EMD2
GGACGTCTCTCTATAC-1	singlet	2	-1276.135599	-1216.767209	-553.5056478	EMD2
GGAGAACAGGTGCTAG-1	singlet	1	-1386.217938	-462.957235	-1160.157517	EMD2
GGAGAACAGTCAGCGA-1	singlet	0	-307.6717356	-895.0226134	-792.5437983	EMD2
GGAGAACAGTCATTGC-1	singlet	0	-725.3300133	-2545.906339	-2236.899476	EMD2
GGAGAACAGTTGGCGA-1	singlet	1	-702.1979409	-257.2234554	-598.8942846	EMD2
GGAGAACCACCGTGGT-1	singlet	1	-2011.738191	-633.7691176	-1708.898263	EMD2
GGAGAACCACGGGTAA-1	singlet	1	-2235.284327	-649.1153312	-1839.748309	EMD2
GGAGAACCACTGAATC-1	unassigned	1	-13.53814326	-3.250056069	-3.986516221	EMD2
GGAGAACCACTGAGGA-1	singlet	1	-641.9680524	-237.9455027	-571.9062375	EMD2
GGAGAACGTTTGCCGG-1	singlet	0	-546.9869271	-1988.530473	-1693.694521	EMD2
GGAGAACTCCCAGCGA-1	singlet	1	-273.7046237	-128.2012804	-287.3796311	EMD2
GGAGAACTCGTCCTTG-1	singlet	2	-353.1126291	-303.0693572	-146.3169516	EMD2
GGAGAACTCTCTGCTG-1	singlet	1	-141.028752	-34.02974576	-119.0162898	EMD2
GGAGATGAGTCATTGC-1	singlet	1	-328.5565616	-130.3409076	-281.6286044	EMD2
GGAGATGCAAATACGA-1	singlet	1	-1394.420145	-455.8758291	-1086.406511	EMD2
GGAGATGCAAGTGGCA-1	doublet	0/2	-2096.669566	-4002.169932	-3001.936662	EMD2
GGAGATGCACCTGTCT-1	singlet	1	-235.256247	-118.2605373	-249.3067877	EMD2
GGAGATGGTACTCCCT-1	singlet	1	-1160.848898	-450.6134902	-969.5122833	EMD2
GGAGATGTCCTTCGAC-1	singlet	1	-464.5319657	-141.4522963	-344.9260823	EMD2
GGAGATGTCGCCAACG-1	singlet	0	-259.6806746	-835.1220579	-819.5111118	EMD2
GGAGCAAAGAGCCGTA-1	singlet	1	-394.5885153	-128.5007231	-352.2846836	EMD2
GGAGCAAAGTGTACAA-1	singlet	2	-3638.364092	-2936.31763	-1109.505875	EMD2
GGAGCAACACATGACT-1	singlet	0	-288.3416714	-716.6213892	-669.839892	EMD2
GGAGCAACACTTTAGG-1	singlet	1	-542.8097508	-133.5178957	-385.1834421	EMD2
GGAGCAACAGCGACAA-1	singlet	2	-13509.95477	-11196.91942	-4227.298566	EMD2
GGAGCAACATCCCGTT-1	singlet	0	-50.47602957	-123.1916163	-124.4058417	EMD2
GGAGCAACATGAGAAT-1	singlet	0	-247.8302827	-837.4903169	-739.6078754	EMD2
GGAGCAACATGGAATA-1	singlet	0	-684.4301113	-2053.743328	-2005.505039	EMD2
GGAGCAAGTAGGACTG-1	singlet	2	-79.21086323	-70.83123878	-26.36404875	EMD2

GGAGCAAGTTAAGTCC-1	singlet	2	-601.9939361	-513.9548298	-226.5910227	EMD2
GGAGCAAGTTGACGGA-1	singlet	2	-605.5400587	-598.6629862	-165.7338211	EMD2
GGAGCAATCCCGAGTG-1	singlet	0	-110.2681862	-319.0076717	-245.1442105	EMD2
GGAGCAATCCTCACTG-1	singlet	1	-160.6654425	-58.70668987	-124.3729179	EMD2
GGAGCAATCCTTATGT-1	singlet	2	-173.3692936	-203.1347406	-64.57168775	EMD2
GGAGCAATCGGCATTA-1	singlet	1	-4376.655907	-1220.141114	-3419.115406	EMD2
GGAGCAATCTCTGCTG-1	singlet	1	-3239.722035	-906.4979944	-2327.268188	EMD2
GGAGGATAGCTGTACT-1	singlet	0	-251.6951489	-572.5729621	-567.5946998	EMD2
GGAGGATAGTCACTGT-1	singlet	2	-454.3020795	-416.5445696	-221.3005301	EMD2
GGAGGATCACCAGCTG-1	singlet	1	-1544.600293	-559.975055	-1253.87562	EMD2
GGAGGATCAGTAGTGG-1	singlet	1	-284.5194536	-127.4552781	-224.2061563	EMD2
GGAGGATCATACTGAC-1	singlet	1	-750.9840809	-313.7137455	-697.7091743	EMD2
GGAGGATGTAGGCTGA-1	singlet	2	-684.0474927	-579.8604311	-271.2012315	EMD2
GGAGGATGTCCGGATC-1	singlet	2	-5983.051765	-5319.822369	-2101.473654	EMD2
GGAGGATGTTCTATCT-1	singlet	2	-2492.13921	-1961.946896	-878.4528513	EMD2
GGAGGATTCCGTGTCT-1	singlet	2	-1651.149963	-1858.985525	-466.1347288	EMD2
GGAGGATTCCTTCTAA-1	singlet	0	-86.06100698	-243.8300137	-255.718989	EMD2
GGAGGTAAGAGGGTCT-1	singlet	0	-333.721569	-1043.762557	-868.2560605	EMD2
GGAGGTAAGCGAGTCA-1	singlet	2	-946.9102014	-890.8939647	-344.8394843	EMD2
GGAGGTAAGCGTCTCG-1	singlet	0	-715.1399838	-2377.612095	-2236.910698	EMD2
GGAGGTAAGGTTCATC-1	singlet	1	-302.2970378	-141.2214688	-293.7918372	EMD2
GGAGGTACACCCAAGC-1	singlet	1	-1004.103129	-304.1058516	-821.3398533	EMD2
GGAGGTACAGACAAGC-1	singlet	0	-776.6091428	-2107.311069	-2051.568582	EMD2
GGAGGTACAGTTAAAG-1	singlet	1	-2716.708492	-706.3530173	-2102.450039	EMD2
GGAGGTAGTTGGGATG-1	singlet	0	-822.8840752	-2456.646913	-2283.019246	EMD2
GGAGGTATCGTAACTG-1	singlet	1	-3461.179562	-1311.087492	-2934.296663	EMD2
GGAGGTATCTCAATCT-1	singlet	1	-427.7216264	-119.8605697	-278.102494	EMD2
GGAGGTATCTTCCACG-1	singlet	1	-252.4031754	-144.8799434	-246.701252	EMD2
GGATCTAAGCACCGAA-1	singlet	1	-2406.618324	-707.2218389	-1865.957426	EMD2
GGATCTAAGCATGCAG-1	singlet	0	-73.45441264	-224.7334371	-208.2956731	EMD2
GGATCTAAGTGGAATT-1	singlet	1	-430.3625064	-166.2835498	-329.0278316	EMD2
GGATCTACAAATTGCC-1	singlet	0	-139.7583591	-459.1038763	-449.8954661	EMD2
GGATCTACACTACGGC-1	singlet	1	-1945.835467	-680.5924225	-1622.239526	EMD2
GGATCTACAGCGAACA-1	singlet	1	-2094.988053	-614.5470837	-1486.279744	EMD2
GGATCTACATCTGCGG-1	singlet	1	-311.3760133	-159.5962417	-308.9258776	EMD2
GGATCTAGTCATAAAG-1	singlet	1	-1300.791662	-463.3387732	-1136.131085	EMD2
GGATCTAGTGGTACAG-1	singlet	1	-1173.673006	-430.5563962	-966.4064782	EMD2
GGATCTAGTGTGTGTT-1	singlet	1	-270.7298452	-113.4160405	-234.0989833	EMD2
GGATCTAGTTACACTG-1	singlet	1	-2977.621777	-841.7222757	-2225.192357	EMD2
GGATCTAGTTGGTAGG-1	singlet	1	-713.4743634	-264.3841276	-562.6609691	EMD2
GGATCTAGTTTCGTGA-1	singlet	0	-771.223802	-1485.200499	-1560.918057	EMD2
GGATCTAGTTTGTTCT-1	singlet	0	-203.5990292	-653.3071333	-523.7878178	EMD2
GGATCTATCTGGAAGG-1	singlet	1	-1406.594648	-537.6958383	-1183.611094	EMD2
GGATGTTCAGTCGAGA-1	singlet	1	-8.597773244	-3.973854613	-9.486256273	EMD2
GGATGTTCATCCGAGC-1	singlet	1	-336.2055873	-84.05881427	-224.5341335	EMD2
GGATGTTGTTCGATTG-1	singlet	0	-781.9207803	-2148.133439	-2262.994863	EMD2
GGATGTTGTTGTCCCT-1	singlet	1	-1109.445317	-298.9530025	-905.0485513	EMD2
GGATGTTTCATCTCTA-1	singlet	0	-529.1517734	-1481.408052	-1349.331675	EMD2
GGATGTTTCGCTGACG-1	singlet	0	-510.6885176	-1668.68399	-1543.60332	EMD2
GGCACGTAGCTCGTGC-1	unassigned	0/2	-40.31415038	-106.5631576	-75.89519523	EMD2
GGCACGTAGGTGCTAG-1	unassigned	45323	-22.53451944	-12.44819137	-9.432619262	EMD2
GGCACGTAGTCCCTAA-1	singlet	0	-44.84639978	-129.8900115	-121.3908826	EMD2
GGCACGTCACCTCTGT-1	singlet	0	-562.4117139	-1671.134521	-1570.767969	EMD2
GGCACGTCATCGCTAA-1	singlet	0	-836.7349571	-2876.575266	-2527.219874	EMD2
GGCACGTGTACAGCGA-1	singlet	0	-1290.558439	-3691.948473	-3521.722012	EMD2
GGCACGTGTCAAAGCG-1	singlet	1	-1185.869396	-411.1238868	-1024.672679	EMD2
GGCACGTGTGAGTTGG-1	singlet	0	-1884.086283	-5664.939108	-5308.660909	EMD2
GGCACGTGTGCATTTG-1	singlet	1	-264.8150427	-81.77853247	-285.8906271	EMD2
GGCACGTGTTGCCTAA-1	singlet	0	-972.2047208	-2939.384611	-2861.157349	EMD2
GGCACGTTCGGCTCTT-1	singlet	0	-744.1665601	-2134.249466	-2247.733035	EMD2
GGCAGTCAGATGGGCT-1	singlet	1	-4651.064174	-1240.209697	-3648.113651	EMD2
GGCAGTCCAGAGTGAC-1	doublet	1/0	-2687.318451	-2138.010868	-3093.753549	EMD2
GGCAGTCCATTCTCTA-1	doublet	0/1	-3463.092775	-3606.077951	-4481.550931	EMD2
GGCAGTCGTCCTCCTA-1	singlet	1	-179.2415133	-75.51160732	-146.8202254	EMD2
GGCAGTCGTGGCTAGA-1	singlet	0	-429.928573	-1076.293691	-1173.316285	EMD2
GGCAGTCGTTCTCCAC-1	singlet	1	-726.6355893	-243.2799661	-527.950026	EMD2
GGCAGTCTCCGCACGA-1	singlet	1	-760.4203549	-247.5427459	-558.0713826	EMD2
GGCAGTCTCGTGCATA-1	singlet	0	-530.7803224	-1917.84573	-1816.414976	EMD2
GGCAGTCTCTTTCCGG-1	singlet	1	-622.9610647	-259.858193	-553.1137703	EMD2
GGCGTCAAGCTAAGTA-1	singlet	1	-269.9106056	-125.642073	-254.0227752	EMD2
GGCGTCAAGGAGTACC-1	singlet	2	-5424.963242	-4224.70685	-1380.61788	EMD2
GGCGTCAAGGTTGCCC-1	singlet	1	-1330.688915	-397.9302173	-998.9579307	EMD2
GGCGTCAAGTCAACAA-1	singlet	2	-1822.699946	-1689.075792	-579.8263506	EMD2
GGCGTCACACACTTAG-1	singlet	0	-245.2683618	-1069.912112	-953.4536558	EMD2
GGCGTCACACGACTAT-1	singlet	2	-2182.973032	-2208.250898	-758.0614402	EMD2
GGCGTCAGTACTAAGA-1	singlet	0	-475.115592	-1765.8843	-1673.056252	EMD2
GGCGTCAGTCAGGCAA-1	singlet	1	-746.9562283	-250.5343344	-529.0146572	EMD2
GGCGTCAGTCCCTCAT-1	singlet	1	-569.712786	-170.8371748	-518.7734798	EMD2
GGCGTCAGTCTCTCCA-1	singlet	1	-2159.760623	-666.5356534	-1871.300485	EMD2
GGCGTCAGTGGGCTCT-1	singlet	1	-317.5544203	-133.0816497	-272.9370501	EMD2
GGCGTCATCACTGCTC-1	singlet	0	-535.1365756	-1800.092632	-1606.807792	EMD2
GGCGTCATCGAAGGAC-1	singlet	2	-1484.50175	-1303.280772	-434.6208672	EMD2
GGCGTCATCGGTAAGG-1	singlet	1	-2353.473913	-681.7667643	-1657.952269	EMD2
GGCTGTGAGAAGCTCG-1	singlet	0	-981.5523916	-3928.914079	-3843.396708	EMD2
GGCTGTGAGACCGCCT-1	singlet	0	-745.5734248	-2205.599546	-2232.317887	EMD2
GGCTGTGAGAGCAGCT-1	singlet	2	-2067.707625	-2258.825439	-643.6494924	EMD2
GGCTGTGAGCAACTTC-1	singlet	1	-296.9960824	-123.5919397	-267.5914083	EMD2
GGCTGTGAGGGTAATT-1	singlet	1	-1851.16604	-574.936685	-1608.829923	EMD2
GGCTGTGAGGTCGACA-1	singlet	2	-14.49380596	-5.589275439	-3.05656877	EMD2
GGCTGTGAGTGCAAAT-1	singlet	1	-1930.44009	-744.3549251	-1642.754141	EMD2
GGCTGTGCAATGTTGC-1	singlet	0	-1154.722021	-4056.811502	-3573.287078	EMD2
GGCTGTGCAGAAATCA-1	singlet	1	-1593.442761	-614.566283	-1351.104047	EMD2
GGCTGTGCAGTTGAAA-1	singlet	1	-546.2150376	-205.4010217	-461.2512411	EMD2
GGCTGTGGTGGGCTCT-1	singlet	0	-145.6648091	-473.0600807	-386.3995208	EMD2
GGCTGTGGTTCTCCTG-1	singlet	2	-2581.127424	-2522.201406	-774.7589935	EMD2
GGCTGTGTCAGACCTA-1	singlet	2	-174.6958591	-188.2522072	-67.75102978	EMD2
GGCTGTGTCTCATTTG-1	singlet	2	-2447.146195	-2029.1036	-722.3477704	EMD2
GGCTTGGCATAGGTTC-1	doublet	1/0	-342.5754845	-177.7115527	-271.3011318	EMD2
GGCTTGGGTAGCGTAG-1	singlet	0	-741.6191614	-2391.956625	-2413.254439	EMD2
GGCTTGGGTGGGTTGA-1	singlet	0	-290.3583054	-751.6409223	-763.0535244	EMD2
GGCTTGGTCGCATGAT-1	singlet	1	-298.5686379	-95.57430634	-212.9615752	EMD2
GGCTTGGTCGCGTGCA-1	singlet	0	-162.0575494	-539.3317175	-494.9288375	EMD2
GGCTTGGTCGCTCCTA-1	singlet	1	-1791.620774	-473.9453934	-1433.17759	EMD2
GGCTTGGTCTACGGTA-1	singlet	1	-1397.148767	-439.239821	-1338.002838	EMD2
GGCTTGGTCTTGGGCG-1	singlet	0	-296.9823502	-1064.875279	-879.4139162	EMD2
GGCTTTCAGGTTTACC-1	singlet	1	-167.9902053	-57.16082556	-106.2511693	EMD2
GGCTTTCAGTATAGGT-1	singlet	2	-4576.295627	-3711.472887	-1519.288844	EMD2
GGCTTTCAGTCACAGG-1	singlet	1	-276.1464328	-119.213823	-253.060847	EMD2
GGCTTTCAGTGGTCAG-1	doublet	1/0	-268.9252026	-173.7647803	-260.9142491	EMD2
GGCTTTCCACAGGATG-1	singlet	1	-2555.394575	-824.2571147	-1989.007629	EMD2

GGCTTTCCATGGCTGC-1	singlet	1	-1004.686773	-340.3117792	-801.5703653	EMD2
GGCTTTCGTAAGATAC-1	singlet	1	-3140.990499	-1144.90676	-2675.427115	EMD2
GGCTTTCGTGAGTAAT-1	singlet	1	-197.762752	-76.33390815	-150.6208052	EMD2
GGCTTTCGTTCTGAGT-1	singlet	1	-1369.487839	-378.2967395	-1052.387476	EMD2
GGCTTTCTCAATGCAC-1	singlet	2	-695.8117701	-567.9840438	-264.2015859	EMD2
GGCTTTCTCCGCAACG-1	singlet	0	-154.3199036	-460.5449196	-398.876261	EMD2
GGCTTTCTCGCCAGTG-1	singlet	1	-652.0982666	-265.240886	-545.2784932	EMD2
GGCTTTCTCGCTACGG-1	singlet	1	-532.2912654	-206.812795	-440.7300619	EMD2
GGCTTTCTCGCTCATC-1	singlet	0	-555.6160565	-1757.56529	-1560.353091	EMD2
GGCTTTCTCTAGTACG-1	singlet	1	-1796.187536	-605.4412218	-1425.6053	EMD2
GGCTTTCTCTTAGGAC-1	singlet	0	-500.3209065	-1701.759457	-1443.58465	EMD2
GGGAAGTAGCTGCCTG-1	singlet	1	-672.1130476	-189.0559054	-509.2469687	EMD2
GGGAAGTAGTACAACA-1	singlet	1	-632.1417144	-220.224187	-528.7898188	EMD2
GGGAAGTCATCCTCAC-1	singlet	2	-6567.306957	-4258.675864	-2018.708744	EMD2
GGGAAGTGTATCGGTT-1	singlet	2	-1053.420478	-911.2490111	-345.3883006	EMD2
GGGAAGTGTATGCAAA-1	singlet	1	-1026.662964	-364.7092124	-917.721583	EMD2
GGGAAGTGTCACAGTT-1	singlet	1	-4009.74076	-617.4502997	-3042.787317	EMD2
GGGAAGTTCAGCGCAC-1	singlet	1	-1438.363986	-450.163738	-1141.79719	EMD2
GGGAAGTTCAGGGTAG-1	singlet	2	-1382.90156	-1239.038338	-558.2596668	EMD2
GGGAAGTTCCAAGGGA-1	singlet	1	-2092.592159	-704.4484944	-1839.041151	EMD2
GGGAAGTTCCCAAGTA-1	doublet	1/0	-2378.336835	-2220.253597	-2967.943334	EMD2
GGGAAGTTCCGGCAAC-1	singlet	1	-538.7276046	-207.8570598	-436.5700129	EMD2
GGGACAAAGCTTAAGA-1	singlet	1	-730.0544401	-242.404356	-556.9773915	EMD2
GGGACAAAGTTCCTGA-1	singlet	1	-501.2114789	-186.7752004	-397.5003351	EMD2
GGGACAACAAGTGGTG-1	doublet	1/0	-2046.85855	-1523.49117	-2505.317499	EMD2
GGGACAAGTAACGTTC-1	doublet	1/0	-788.5008494	-519.5412874	-889.4284519	EMD2
GGGACAAGTCAAACGG-1	singlet	1	-482.599783	-197.9334426	-383.2440515	EMD2
GGGACAATCGTTTACT-1	singlet	0	-850.9389673	-2597.52292	-2568.403807	EMD2
GGGACCTAGAGGTCGT-1	unassigned	45293	-13.22531177	-2.662312524	-3.093384866	EMD2
GGGACCTAGCAGGCTA-1	singlet	1	-2366.913152	-753.0113001	-1889.570472	EMD2
GGGACCTAGCCGAATG-1	singlet	1	-537.015349	-243.1976791	-509.2303182	EMD2
GGGACCTCACGTAACT-1	doublet	45293	-1789.655184	-906.316036	-1420.149193	EMD2
GGGACCTCAGATACTC-1	singlet	1	-1559.775459	-547.4951441	-1405.965514	EMD2
GGGACCTGTTAAGGAT-1	singlet	1	-404.6640022	-157.4613112	-327.3117266	EMD2
GGGACCTTCTGACCCT-1	singlet	1	-742.6762347	-272.1732001	-610.2636101	EMD2
GGGACTCAGACGCCCT-1	singlet	0	-1728.322253	-4215.785463	-4454.409497	EMD2
GGGACTCAGATGGCAC-1	singlet	1	-600.0389118	-206.3557861	-476.4976041	EMD2
GGGACTCAGTTGGCTT-1	singlet	2	-2274.091082	-1871.763956	-772.6672115	EMD2
GGGACTCCAATGAGCG-1	singlet	1	-1612.162909	-482.7708475	-1442.737491	EMD2
GGGACTCCACACGTGC-1	singlet	0	-842.9923585	-2541.647871	-2540.088724	EMD2
GGGACTCGTAAGAACT-1	singlet	0	-1686.87031	-6057.423289	-5474.875613	EMD2
GGGACTCGTATGCAAA-1	singlet	2	-220.6098007	-272.5253884	-118.6821251	EMD2
GGGACTCGTGGAGAAA-1	singlet	2	-582.6155288	-558.2696608	-232.5300286	EMD2
GGGACTCGTTAAGAAC-1	singlet	0	-463.2871328	-1326.171391	-1260.544996	EMD2
GGGACTCTCTTCGGTC-1	singlet	1	-445.4459304	-153.496364	-357.0919337	EMD2
GGGAGATAGATGGGCT-1	doublet	1/0	-2336.25381	-1385.936636	-2213.519192	EMD2
GGGAGATAGTTGAAGT-1	singlet	1	-1718.510418	-582.5064349	-1411.094958	EMD2
GGGAGATAGTTGCTCA-1	doublet	1/0	-2871.905388	-1966.573323	-3068.39717	EMD2
GGGAGATCACCATTCC-1	singlet	1	-458.8465535	-138.248427	-306.5778594	EMD2
GGGAGATCACGCTATA-1	singlet	1	-51.80280957	-21.15846816	-45.17074894	EMD2
GGGAGATCAGAGCTAG-1	singlet	1	-498.2438908	-170.7076158	-421.5474483	EMD2
GGGAGATCATCATCCC-1	unassigned	1/0	-6.173225555	-2.335804517	-2.867017327	EMD2
GGGAGATGTAACACGG-1	doublet	1/0	-1296.186248	-945.7923854	-1398.656359	EMD2
GGGAGATGTAGTGGCA-1	singlet	1	-172.8508712	-62.89411993	-157.092891	EMD2
GGGAGATGTCACCGAC-1	singlet	0	-208.7969132	-772.7395569	-789.7170217	EMD2
GGGAGATGTCTGTCCT-1	singlet	0	-519.9998657	-1529.726353	-1434.23058	EMD2
GGGAGATGTGAGGATC-1	singlet	1	-1448.140439	-392.6253889	-1084.689722	EMD2
GGGAGATTCAAACCCA-1	singlet	1	-181.0246747	-64.72805113	-137.2711107	EMD2
GGGAGATTCATCACCC-1	unassigned	1	-8.472022428	-2.926668925	-3.001134857	EMD2
GGGAGTAAGACTTCGT-1	singlet	1	-1328.681961	-503.7353072	-1190.200742	EMD2
GGGAGTAAGCAACAAT-1	singlet	2	-819.7030532	-817.4594335	-364.354322	EMD2
GGGAGTAAGCCAAGTG-1	singlet	1	-16.238535	-4.189148324	-15.5207546	EMD2
GGGAGTAAGCGATTCT-1	singlet	1	-708.4649996	-300.0788468	-641.1620174	EMD2
GGGAGTAAGTCTGCGC-1	singlet	1	-1783.701537	-616.3347623	-1653.065337	EMD2
GGGAGTACAAGTCCAT-1	singlet	2	-3611.040511	-2617.311875	-1385.771135	EMD2
GGGAGTACACACCGCA-1	singlet	1	-1831.751583	-546.86004	-1540.987647	EMD2
GGGAGTACACCGTCGA-1	singlet	0	-519.47528	-3894.457702	-2912.924328	EMD2
GGGAGTACAGCATGCC-1	singlet	2	-1052.867042	-1007.559308	-418.0613448	EMD2
GGGAGTAGTATGGTTC-1	doublet	45293	-4040.197802	-1581.147964	-2705.551183	EMD2
GGGAGTAGTGATATAG-1	singlet	1	-1844.493366	-518.5903999	-1358.881312	EMD2
GGGAGTAGTGTCCAAT-1	singlet	1	-1071.130752	-420.290879	-938.9968863	EMD2
GGGAGTATCCCGAGGT-1	singlet	2	-2216.867081	-2308.833927	-550.1282734	EMD2
GGGATCCAGGAGGCAG-1	singlet	0	-246.2887264	-878.0918644	-647.3717378	EMD2
GGGATCCAGGGAGGGT-1	singlet	1	-2618.705957	-841.6479003	-2102.611928	EMD2
GGGATCCCACCCTTGT-1	singlet	1	-600.743355	-196.8867741	-460.0734474	EMD2
GGGATCCCAGGCATGA-1	singlet	1	-218.114501	-104.9355631	-190.2106424	EMD2
GGGATCCTCCACACAA-1	singlet	0	-432.2047775	-1213.924906	-1053.133805	EMD2
GGGATCCTCGACACCG-1	singlet	1	-660.6810581	-256.6720062	-561.6551671	EMD2
GGGATCCTCTACCTTA-1	singlet	1	-1331.727418	-443.363916	-1237.308579	EMD2
GGGATGAAGAGTGTGC-1	singlet	2	-5758.990398	-4583.665349	-1577.879173	EMD2
GGGATGAAGATTGAGT-1	singlet	1	-4514.282025	-1648.288326	-3871.912589	EMD2
GGGATGAAGTACTGGG-1	singlet	0	-9.540098854	-22.41757621	-29.69749178	EMD2
GGGATGACAACGCCCA-1	singlet	1	-865.0751107	-264.2609827	-659.5668672	EMD2
GGGATGACATGGCCCA-1	singlet	2	-1923.245414	-1718.688334	-740.8457291	EMD2
GGGATGAGTAGATCGG-1	singlet	2	-771.0055632	-727.5308905	-308.759507	EMD2
GGGATGAGTCATAACC-1	singlet	1	-2270.379216	-804.209543	-1991.608869	EMD2
GGGATGAGTGGCTACC-1	singlet	2	-294.2510761	-317.4030294	-100.7946051	EMD2
GGGATGATCAATCTTC-1	singlet	0	-884.6836644	-2546.402817	-2481.647207	EMD2
GGGATGATCACGGACC-1	singlet	2	-2631.359668	-2099.343861	-886.0227297	EMD2
GGGCCATAGCATAGGC-1	singlet	1	-326.6219362	-124.627625	-278.8632047	EMD2
GGGCCATAGGATGGCT-1	singlet	2	-512.0404455	-382.2834512	-160.5712621	EMD2
GGGCCATCACTCATAG-1	singlet	0	-2049.711899	-7288.567983	-7219.938173	EMD2
GGGCCATCACTGAGTT-1	singlet	2	-2651.41469	-2267.505284	-948.2452042	EMD2
GGGCCATCATAATGAG-1	singlet	1	-1869.794399	-565.9693275	-1818.518269	EMD2
GGGCCATGTATCGCGC-1	singlet	2	-512.6717388	-702.9131286	-224.5047452	EMD2
GGGCCATGTGCTGCAC-1	singlet	0	-202.2994467	-875.2507373	-755.3308939	EMD2
GGGCCATGTTGTGTAC-1	singlet	1	-4363.717381	-1332.223474	-3237.355576	EMD2
GGGCCATTCACTCCGT-1	singlet	1	-798.8406567	-236.267599	-587.4473121	EMD2
GGGCCATTCCATTGCC-1	singlet	2	-761.7845036	-675.3989406	-274.0147037	EMD2
GGGCCATTCGAAGTGG-1	singlet	1	-188.9628877	-71.40261268	-170.6080662	EMD2
GGGCGTTAGACTTCAC-1	singlet	0	-773.1601713	-2399.508056	-2297.588302	EMD2
GGGCGTTAGCCAAGGT-1	singlet	0	-118.4667984	-395.5522608	-306.264802	EMD2
GGGCGTTAGCTAGTTC-1	doublet	1/0	-260.9714743	-171.942301	-301.4815854	EMD2
GGGCGTTCAGCTATTG-1	doublet	45293	-216.1348974	-120.2475818	-162.6353298	EMD2
GGGCGTTGTAGCGTCC-1	singlet	1	-555.914708	-182.3343897	-395.6829123	EMD2
GGGCGTTTCTAGTGTG-1	singlet	0	-1106.610001	-3803.058477	-3663.769462	EMD2
GGGCTACAGGTAGCCA-1	singlet	1	-477.4405952	-131.2128554	-307.28914	EMD2
GGGCTACAGTAAACAC-1	singlet	1	-450.2063089	-189.419052	-396.2420659	EMD2
GGGCTACCAAGATGTA-1	singlet	1	-2451.772676	-829.571221	-1826.261064	EMD2
GGGCTACCAGAAGTTA-1	singlet	1	-669.0516443	-210.7057687	-561.6274855	EMD2

GGGCTACCATGGGTTT-1	singlet	2	-683.2023683	-613.9271792	-224.2239387	EMD2
GGGCTACGTTAATCGC-1	singlet	2	-1141.709339	-1172.166682	-437.7203828	EMD2
GGGCTACGTTTCTTAC-1	singlet	0	-150.4421123	-534.7947729	-506.0951253	EMD2
GGGCTACTCTCTAGGA-1	singlet	1	-1863.204588	-495.7236661	-1607.566792	EMD2
GGGCTCAAGCATCAGG-1	singlet	1	-953.098781	-326.1781819	-813.6774023	EMD2
GGGCTCAAGCCTGGAA-1	singlet	0	-663.515676	-2081.116652	-2003.105737	EMD2
GGGCTCACAGCTACAT-1	singlet	1	-337.0369058	-146.5302986	-273.7959724	EMD2
GGGCTCACATGGCCCA-1	singlet	1	-282.2016833	-118.6041835	-275.2451355	EMD2
GGGCTCAGTGACAGGT-1	singlet	0	-2.54208943	-13.12883856	-9.633566168	EMD2
GGGCTCAGTGGCCACT-1	singlet	1	-2845.469797	-844.9248934	-2259.459278	EMD2
GGGCTCAGTTCTCAGA-1	singlet	1	-4475.142197	-1190.117053	-3078.563281	EMD2
GGGCTCATCATTTGTC-1	singlet	0	-303.9494819	-885.9239446	-770.8128303	EMD2
GGGCTCATCCGTGGCA-1	singlet	1	-3437.893587	-1118.080472	-2440.507444	EMD2
GGGCTCATCGGTATGT-1	singlet	2	-273.7379806	-307.2948349	-116.4165036	EMD2
GGGTAGAAGAGGCTGT-1	singlet	0	-511.3641439	-1394.590544	-1337.482386	EMD2
GGGTAGAAGATACATG-1	doublet	45293	-89.31065792	-33.9075373	-58.02481183	EMD2
GGGTAGACAAGGTACG-1	singlet	0	-226.3585483	-557.7715685	-518.7472722	EMD2
GGGTAGACAAGTGCTT-1	singlet	1	-1143.013673	-374.8028522	-983.8170627	EMD2
GGGTAGACATAACTCG-1	singlet	1	-533.4126604	-193.3455966	-467.2096362	EMD2
GGGTAGACATACTGTG-1	singlet	1	-1044.6844	-348.2408265	-919.5707155	EMD2
GGGTAGACATAGTCAC-1	singlet	1	-615.587153	-196.6609541	-494.1316723	EMD2
GGGTAGAGTAACAGGC-1	singlet	0	-1537.134299	-4429.406419	-4328.850914	EMD2
GGGTAGATCAGTAGGG-1	singlet	1	-432.1225587	-149.2566951	-334.2119378	EMD2
GGGTAGATCCCTCGTA-1	singlet	0	-637.0992503	-2043.865804	-1805.940228	EMD2
GGGTATTAGCACTCCG-1	singlet	0	-63.96957579	-201.7504235	-202.6141203	EMD2
GGGTATTAGTGCGACA-1	singlet	2	-1141.46333	-997.2147623	-400.1643576	EMD2
GGGTATTCACATTCGA-1	singlet	1	-292.7999807	-127.9413635	-265.8372965	EMD2
GGGTATTGTAAGGCTG-1	singlet	0	-263.9164378	-1026.704446	-919.5562624	EMD2
GGGTATTGTTGTCTAG-1	singlet	0	-465.3032057	-1368.578874	-1283.154669	EMD2
GGGTATTTCGATAACC-1	singlet	0	-741.01775	-2681.844014	-2505.998141	EMD2
GGGTATTTCGCTACAA-1	singlet	1	-342.4752614	-124.906423	-285.8093008	EMD2
GGGTATTTCGGCGATC-1	singlet	1	-126.1619528	-45.06732536	-100.0083983	EMD2
GGGTATTTCTGAGAAA-1	singlet	1	-275.6862436	-112.9958877	-229.0818338	EMD2
GGGTCACAGAGGTTTA-1	singlet	1	-1741.446197	-596.5120739	-1399.250912	EMD2
GGGTCACAGATAGCAT-1	singlet	1	-512.9923432	-190.7452728	-421.511919	EMD2
GGGTCACAGGGAGAAT-1	singlet	2	-100.1118368	-126.8556013	-42.86564003	EMD2
GGGTCACCATGGGCAA-1	singlet	2	-1293.431731	-1162.961405	-416.333109	EMD2
GGGTCACTCGCTTTAT-1	singlet	1	-532.2469075	-190.9713765	-366.5436491	EMD2
GGGTCTGAGAACTGAT-1	singlet	2	-1203.726069	-1146.678998	-432.0005458	EMD2
GGGTCTGAGGACTAAT-1	singlet	2	-4870.230474	-4009.485995	-1609.001375	EMD2
GGGTCTGAGTGAGCCA-1	singlet	1	-1611.344145	-474.146478	-1311.233522	EMD2
GGGTCTGCAACGTAAA-1	singlet	1	-366.6297484	-160.1543179	-341.3391697	EMD2
GGGTCTGCAGCTCCTT-1	singlet	1	-3026.748632	-927.1886141	-2603.986814	EMD2
GGGTCTGCAGGCGTTC-1	singlet	1	-206.6625877	-86.93723587	-180.9572928	EMD2
GGGTCTGTCCAAGCAT-1	singlet	1	-2430.589776	-822.7719445	-1907.442125	EMD2
GGGTCTGTCCATACAG-1	singlet	1	-1306.879758	-475.3427576	-1116.563657	EMD2
GGGTCTGTCTCCGCAT-1	singlet	0	-96.10356976	-273.0845661	-233.8117148	EMD2
GGGTGAAAGCTTAGTC-1	singlet	1	-274.3233466	-114.3162771	-252.7360285	EMD2
GGGTGAACAGTCAGAG-1	unassigned	0/2	-2.013355112	-5.6165617	-2.0821266	EMD2
GGGTGAACATCGCTAA-1	singlet	0	-653.496739	-1939.908861	-1724.512999	EMD2
GGGTGAAGTCCAGGTC-1	singlet	0	-660.3964981	-2084.547931	-1975.757146	EMD2
GGGTGAAGTTTCCATT-1	singlet	1	-393.8953757	-182.0916292	-320.7456917	EMD2
GGGTGAATCTACGCAA-1	singlet	1	-386.2609959	-135.5616133	-359.7706427	EMD2
GGGTGTCAGCCGGATA-1	singlet	1	-618.2402402	-178.891513	-402.733792	EMD2
GGGTGTCAGCCTCTGG-1	singlet	0	-302.5776773	-1054.046486	-875.1319788	EMD2
GGGTGTCAGCGTCTCG-1	singlet	1	-1855.866521	-524.6332425	-1466.193733	EMD2
GGGTGTCAGTAAGGGA-1	singlet	1	-2323.254033	-697.4420861	-1577.275778	EMD2
GGGTGTCAGTTCGCAT-1	singlet	1	-904.0381509	-251.5735627	-751.4520021	EMD2
GGGTGTCGTAACTGCT-1	singlet	1	-516.7883407	-189.4286547	-427.9030203	EMD2
GGGTGTCGTAGGACCA-1	singlet	2	-1026.884412	-839.6965915	-372.3306634	EMD2
GGGTGTCGTCATGACT-1	doublet	0/1	-1443.130628	-1682.22873	-1908.07621	EMD2
GGGTGTCGTCTTCATT-1	singlet	1	-321.4710685	-140.2034188	-306.5146404	EMD2
GGGTTATAGACATAAC-1	singlet	0	-449.8666737	-1463.874795	-1412.465209	EMD2
GGGTTATAGCATCTTG-1	singlet	1	-1024.6821	-341.3278529	-819.0121912	EMD2
GGGTTATAGCCGATTT-1	singlet	2	-2211.914077	-1978.349738	-619.943673	EMD2
GGGTTATAGTGGCGAT-1	singlet	1	-385.6138433	-162.8052749	-346.0050136	EMD2
GGGTTATCAAGCGCTC-1	singlet	1	-1607.469079	-446.5354705	-1470.513107	EMD2
GGGTTATCAGCTCTGG-1	singlet	1	-12.16173776	-2.946957484	-17.44449157	EMD2
GGGTTATCAGGTTTAC-1	singlet	1	-423.9125807	-184.4061337	-300.8279263	EMD2
GGGTTATGTCCAGAAG-1	singlet	1	-2712.84532	-859.7135482	-2066.469645	EMD2
GGGTTATTCCTGTTGC-1	singlet	1	-171.6981493	-59.91488675	-162.4303633	EMD2
GGGTTATTCGACGCGT-1	singlet	2	-640.0407649	-466.5196357	-174.7330784	EMD2
GGGTTATTCTTAGCCC-1	singlet	0	-420.991513	-1420.205395	-1282.23606	EMD2
GGGTTTAAGCATTTGC-1	singlet	1	-54.39298591	-20.75450722	-50.26935747	EMD2
GGGTTTACAACGAGGT-1	singlet	1	-719.4924453	-195.1371661	-511.8181147	EMD2
GGGTTTACACTGCGTG-1	singlet	1	-3089.056998	-1060.806761	-2503.310216	EMD2
GGGTTTACATGCAGCC-1	singlet	2	-1588.342681	-1317.371123	-485.7385327	EMD2
GGGTTTAGTACGATTC-1	singlet	2	-410.5892738	-430.7986415	-221.0203066	EMD2
GGGTTTAGTCACTGAT-1	singlet	0	-18.88642706	-88.91512043	-104.9387297	EMD2
GGGTTTAGTCGCACAC-1	singlet	0	-701.9282224	-1874.498063	-1810.717496	EMD2
GGGTTTAGTGAATGTA-1	singlet	2	-360.3431859	-351.695574	-131.7029366	EMD2
GGGTTTAGTGACAGCA-1	singlet	0	-16.44386667	-42.40804392	-35.93963102	EMD2
GGGTTTAGTGGTCTGC-1	singlet	1	-463.0119751	-191.0172746	-387.9762856	EMD2
GGGTTTATCAAAGAAC-1	singlet	2	-2308.146486	-2229.36306	-800.6272338	EMD2
GGGTTTATCACTCCGT-1	singlet	2	-5714.759341	-4536.242648	-1640.208429	EMD2
GGGTTTATCAGCCCAG-1	singlet	0	-1440.57164	-4264.622503	-4457.916307	EMD2
GGGTTTATCCACGTGG-1	singlet	1	-2901.509405	-455.5702718	-1652.784239	EMD2
GGTAACTAGCCAGACA-1	singlet	2	-724.0264197	-653.5194717	-287.2475081	EMD2
GGTAACTCATGTGCCG-1	singlet	0	-495.30488	-1637.101875	-1462.224054	EMD2
GGTAACTCATTAAGCC-1	doublet	1/0	-1978.737741	-1667.742365	-2193.041901	EMD2
GGTAACTGTTACACAC-1	singlet	2	-2869.187575	-2503.096009	-828.4350498	EMD2
GGTAACTGTTATCTTC-1	singlet	2	-838.2247975	-751.7678486	-280.6768515	EMD2
GGTAACTTCATGCGGC-1	singlet	1	-402.3978965	-149.4846614	-314.8027218	EMD2
GGTAACTTCCACTGGG-1	singlet	1	-766.6030636	-294.3371061	-592.2624839	EMD2
GGTAACTTCGAACCAT-1	singlet	0	-239.2250792	-818.3263778	-633.436722	EMD2
GGTAACTTCTGGTGCG-1	singlet	0	-650.3645367	-1771.872187	-1630.48607	EMD2
GGTAATCAGGAAGTGA-1	singlet	1	-948.2771579	-244.3493681	-570.4535936	EMD2
GGTAATCAGGACATCG-1	singlet	1	-1954.934693	-497.0786659	-1442.531005	EMD2
GGTAATCAGGGAGGCA-1	singlet	0	-902.6744134	-1756.465036	-1592.832684	EMD2
GGTAATCAGTCACACT-1	singlet	1	-3209.715259	-1040.852944	-2783.141693	EMD2
GGTAATCAGTGAGTGC-1	singlet	0	-291.6700044	-886.4069385	-752.6721003	EMD2
GGTAATCAGTGTTGAA-1	singlet	1	-288.719496	-103.1114941	-260.6713622	EMD2
GGTAATCCAGAACATA-1	singlet	1	-433.3260165	-173.9081452	-411.8941895	EMD2
GGTAATCCAGAGCTAG-1	singlet	1	-458.4154135	-180.6108127	-367.8889226	EMD2
GGTAATCGTACTCGAT-1	singlet	1	-1547.224295	-569.7601454	-1335.441116	EMD2
GGTAATCGTCTCGACG-1	singlet	1	-2059.32891	-741.6404348	-1659.095356	EMD2
GGTAATCGTGATCGTT-1	singlet	1	-210.274896	-85.79190267	-183.7795948	EMD2
GGTAATCGTGGCGTAA-1	singlet	1	-1846.452732	-651.5442468	-1673.882147	EMD2
GGTAATCGTGGCTGCT-1	singlet	1	-941.4283478	-299.0320861	-728.6718729	EMD2
GGTAATCTCAGAGTTC-1	singlet	1	-866.3614576	-329.6043964	-747.0995413	EMD2

GGTAATCTCGCTAGCG-1	singlet	1	-3657.545542	-1215.097704	-2885.218316	EMD2
GGTAGAGAGAGCCATG-1	singlet	2	-3356.51598	-2605.800897	-1032.593871	EMD2
GGTAGAGAGCTAATGA-1	singlet	1	-1316.77903	-377.2136599	-989.7977522	EMD2
GGTAGAGAGGCGAAGG-1	singlet	1	-409.7087936	-171.7097575	-371.3709844	EMD2
GGTAGAGGTCTTGTCC-1	singlet	1	-292.9046203	-94.44066308	-211.7426004	EMD2
GGTAGAGGTGTCCACG-1	singlet	1	-584.5451834	-196.2992163	-450.2018657	EMD2
GGTAGAGGTTCAAACC-1	singlet	1	-387.772373	-144.2630055	-378.9054319	EMD2
GGTAGAGGTTGCATCA-1	singlet	1	-1092.552709	-370.0878933	-852.344655	EMD2
GGTAGAGTCGGACTTA-1	singlet	0	-33.94169875	-69.13609103	-81.33529323	EMD2
GGTCACGAGCTTGTGT-1	singlet	1	-1677.502704	-487.2230877	-1198.898967	EMD2
GGTCACGCAACTGCCG-1	singlet	0	-878.6225744	-2498.23828	-2385.776111	EMD2
GGTCACGCACATCCCT-1	singlet	1	-2560.46328	-935.8908896	-2261.851681	EMD2
GGTCACGCATCGAAGG-1	singlet	1	-1693.62183	-518.8304845	-1238.513785	EMD2
GGTCACGTCAGGGTAG-1	singlet	0	-1788.160391	-4902.253178	-4410.548757	EMD2
GGTCACGTCGCCTTGT-1	singlet	0	-408.2263782	-1263.303393	-1148.427344	EMD2
GGTCACGTCTCAACGA-1	singlet	0	-70.10525633	-208.6662564	-185.0691663	EMD2
GGTCTGGAGACAGCTG-1	singlet	2	-273.8869917	-218.8575391	-105.6711805	EMD2
GGTCTGGAGAGAGGGC-1	singlet	1	-106.0226186	-40.00387709	-87.49960712	EMD2
GGTCTGGAGTTAGTGA-1	singlet	0	-686.3372934	-1800.494063	-1843.146011	EMD2
GGTCTGGCATCGCTGG-1	singlet	0	-3578.185406	-8647.051909	-9006.38131	EMD2
GGTCTGGGTCTGCATA-1	singlet	0	-375.3319706	-1639.866049	-1455.831111	EMD2
GGTGAAGAGCCAGTAG-1	singlet	1	-523.7872798	-193.9857821	-437.1239681	EMD2
GGTGAAGAGGACTTCT-1	singlet	1	-3222.013687	-959.7441607	-2478.759796	EMD2
GGTGAAGCATCTAGAC-1	unassigned	2	-5.982656323	-2.471097586	-1.861794718	EMD2
GGTGAAGGTATAGGGC-1	singlet	1	-1647.093399	-460.6407575	-1044.900419	EMD2
GGTGAAGTCACCTACC-1	doublet	1/0	-340.315788	-201.7233325	-343.5916934	EMD2
GGTGAAGTCAGACAAA-1	singlet	2	-2860.201385	-2394.908063	-1030.230435	EMD2
GGTGAAGTCTAGATCG-1	singlet	1	-2185.833721	-805.4361312	-1761.858627	EMD2
GGTGATTAGCAAGCCA-1	singlet	2	-5599.01884	-4616.096737	-1899.890188	EMD2
GGTGATTAGGTACTGG-1	singlet	1	-534.2965627	-196.3957485	-410.0967144	EMD2
GGTGATTAGTCAACAA-1	singlet	1	-939.3439358	-295.2112558	-726.2509979	EMD2
GGTGATTGTCTTTCAT-1	singlet	1	-1392.69267	-544.2908689	-1252.449205	EMD2
GGTGATTTCATGAGTC-1	doublet	0/1	-413.9723238	-469.8966366	-519.1892139	EMD2
GGTGATTTCCTTCAGC-1	singlet	1	-349.6723508	-137.533073	-297.054648	EMD2
GGTGATTTCGAGAGAC-1	singlet	2	-196.2212335	-159.7812834	-76.00303686	EMD2
GGTGATTTCGCTCTCA-1	singlet	0	-1186.027434	-3301.53266	-3371.40199	EMD2
GGTGGCTAGATCGGTG-1	singlet	1	-3498.987066	-1016.484052	-2651.64927	EMD2
GGTGGCTAGCTGAAGC-1	singlet	1	-631.3901945	-218.6517014	-500.0540334	EMD2
GGTGGCTCACTCACTC-1	singlet	2	-718.6576409	-835.0166236	-255.6035849	EMD2
GGTGGCTCACTGGAAG-1	singlet	1	-331.1797091	-113.4238701	-269.1889468	EMD2
GGTGGCTCAGTTGAAA-1	singlet	2	-661.4001284	-658.8717026	-222.8486809	EMD2
GGTGGCTCATGCGTGC-1	singlet	2	-3551.046603	-3020.700235	-1193.027652	EMD2
GGTGGCTGTGAGATAT-1	singlet	0	-648.6769996	-1884.848802	-1674.056674	EMD2
GGTGGCTTCATTCCTA-1	singlet	1	-2126.013195	-694.9473591	-1828.38608	EMD2
GGTGGCTTCATTGCTT-1	singlet	1	-660.4452156	-240.7002769	-631.9298646	EMD2
GGTGGCTTCTCGTGGG-1	singlet	1	-2328.324582	-752.8372395	-1862.465264	EMD2
GGTGTCGAGATGCTAA-1	singlet	0	-533.138136	-1363.455362	-1419.734663	EMD2
GGTGTCGAGGAAAGAC-1	singlet	1	-4505.208123	-1300.800748	-3497.486409	EMD2
GGTGTCGAGGTAATCA-1	singlet	1	-1366.354394	-406.2808611	-1043.13978	EMD2
GGTGTCGAGTAGTCCT-1	singlet	1	-1047.942061	-277.3501813	-863.7522658	EMD2
GGTGTCGAGTCCGCGT-1	singlet	2	-453.6185909	-493.0761619	-216.8620264	EMD2
GGTGTCGAGTGGATTA-1	singlet	0	-469.1944657	-1632.785987	-1597.740116	EMD2
GGTGTCGCAATAGTCC-1	singlet	2	-2902.708282	-2140.70881	-1277.029033	EMD2
GGTGTCGGTAAGGTCG-1	singlet	2	-2542.600193	-1975.986306	-615.6313753	EMD2
GGTGTCGGTGGAGAAA-1	singlet	1	-1276.7405	-471.3494908	-1128.216474	EMD2
GGTGTCGGTTCTCGTC-1	singlet	0	-98.32483084	-263.1585041	-295.0835324	EMD2
GGTGTCGTCGCTGTCT-1	singlet	1	-3942.678878	-1195.777483	-3000.306932	EMD2
GGTGTCGTCGTTTACT-1	singlet	1	-373.8409118	-130.2239313	-365.0949915	EMD2
GGTGTTAAGACTGGGT-1	singlet	1	-3319.24639	-991.6543541	-2630.110078	EMD2
GGTGTTACAAAGGGTC-1	unassigned	45293	-36.11168094	-11.40363489	-21.67477941	EMD2
GGTGTTACAAGTGTCT-1	singlet	1	-4334.8757	-1392.374568	-3620.799279	EMD2
GGTGTTACACTATGTG-1	singlet	1	-442.168903	-158.077124	-393.3789841	EMD2
GGTGTTACAGGGTCTC-1	singlet	0	-1115.875909	-5138.257497	-5143.237505	EMD2
GGTGTTACATAGAGGC-1	singlet	1	-1093.106467	-347.7113126	-882.6392836	EMD2
GGTGTTACATAGTCGT-1	doublet	45323	-1581.526843	-1249.886216	-910.6488406	EMD2
GGTGTTACATCCTATT-1	singlet	2	-784.993719	-863.2114394	-295.1615582	EMD2
GGTGTTAGTCTGTTAG-1	singlet	1	-144.9515262	-64.64648098	-121.0159636	EMD2
GGTGTTAGTGCATTAC-1	singlet	1	-140.2282274	-55.11829982	-134.7947444	EMD2
GGTGTTAGTGGAATGC-1	singlet	0	-110.1252501	-364.9610243	-379.9062211	EMD2
GGTGTTATCAAAGAAC-1	singlet	1	-2622.097167	-774.1968612	-2081.0204	EMD2
GGTGTTATCATTGTTC-1	singlet	1	-3405.634539	-964.8419052	-2801.686082	EMD2
GGTGTTATCGGACTGC-1	singlet	1	-1700.673272	-502.8192553	-1393.99441	EMD2
GGTTAACAGACCATAA-1	singlet	0	-522.3544975	-1515.185519	-1317.319651	EMD2
GGTTAACAGAGATTCA-1	singlet	1	-678.6363459	-277.8982834	-645.9163327	EMD2
GGTTAACAGGTTAGTA-1	singlet	1	-957.820162	-329.5618444	-780.2477631	EMD2
GGTTAACAGTTAACAG-1	singlet	1	-900.3263549	-390.8583058	-903.2258852	EMD2
GGTTAACCAACAAAGT-1	singlet	1	-856.2466079	-269.5955776	-679.4206272	EMD2
GGTTAACCACCAAAGG-1	singlet	1	-2893.234272	-799.4262617	-2145.443574	EMD2
GGTTAACCACGTGTGC-1	singlet	1	-201.5729446	-88.2960421	-158.3662419	EMD2
GGTTAACCACTCAGAT-1	singlet	0	-791.0647834	-2916.426903	-2909.624234	EMD2
GGTTAACCAGATACTC-1	singlet	2	-433.6051594	-422.1896529	-178.7642163	EMD2
GGTTAACTCACATACG-1	singlet	1	-125.4403677	-27.97702485	-102.6583309	EMD2
GGTTAACTCCAAGCAT-1	singlet	1	-261.411383	-123.5666079	-239.1719189	EMD2
GGTTAACTCCTGGGAC-1	singlet	0	-1040.258739	-4546.134316	-4991.568512	EMD2
GGTTCTCAGTCATGGG-1	singlet	2	-2147.56612	-1838.069677	-761.6774496	EMD2
GGTTCTCCAATCGCCG-1	singlet	1	-264.7501969	-118.9102685	-218.2693446	EMD2
GGTTCTCCACAATCTG-1	singlet	1	-2235.384395	-708.9269712	-1753.927661	EMD2
GGTTCTCCACGATAGG-1	singlet	1	-1595.616336	-513.203221	-1188.269215	EMD2
GGTTCTCCAGCTATTG-1	singlet	0	-1001.086444	-3148.686965	-3032.772373	EMD2
GGTTCTCCATGCGTGC-1	singlet	1	-4460.76792	-1106.106329	-3559.216298	EMD2
GGTTCTCGTCGATGCC-1	singlet	2	-243.4526823	-224.3776246	-91.45418775	EMD2
GGTTCTCGTCTTAGTG-1	singlet	1	-1200.909373	-427.0440382	-992.1293634	EMD2
GGTTCTCGTGACTGAG-1	singlet	1	-431.3600441	-158.1724795	-383.6580646	EMD2
GGTTCTCGTTGTCATG-1	singlet	1	-2981.591297	-1007.764336	-2389.95565	EMD2
GGTTCTCTCGAGTTGT-1	singlet	1	-1100.144934	-390.4380175	-963.0684552	EMD2
GGTTCTCTCTACGCGG-1	singlet	1	-253.5869198	-106.8933699	-202.1825495	EMD2
GGTTCTCTCTGGGAGA-1	singlet	1	-1720.751582	-623.2049155	-1513.72071	EMD2
GGTTGTAAGGACAAGA-1	singlet	0	-105.8838517	-248.8973954	-235.3683206	EMD2
GGTTGTAAGGTCGTCC-1	singlet	0	-991.7516198	-3705.551243	-3546.338905	EMD2
GGTTGTACAATCGAAA-1	singlet	0	-591.1481563	-1887.754484	-1706.636451	EMD2
GGTTGTACACCGTGCA-1	singlet	1	-896.9407563	-253.3349554	-657.5677594	EMD2
GGTTGTACAGAAACCG-1	singlet	1	-669.9083855	-260.3229621	-683.6024848	EMD2
GGTTGTAGTAAGTCAA-1	singlet	1	-394.9367932	-166.0849064	-356.5521483	EMD2
GGTTGTATCAGTGATC-1	singlet	1	-203.8854841	-75.70333107	-293.2046193	EMD2
GGTTGTATCGACGCGT-1	singlet	1	-2136.110629	-593.7822767	-1832.991636	EMD2
GGTTGTATCTCATTGT-1	singlet	1	-1458.543765	-411.3672364	-1188.962186	EMD2
GTAACACAGTCAGGGT-1	singlet	1	-1676.157555	-574.5728181	-1498.506507	EMD2
GTAACACAGTTAGTAG-1	singlet	2	-6.072812239	-6.041503001	-2.360051217	EMD2
GTAACACAGTTCATGC-1	singlet	1	-2871.822339	-867.3525318	-2376.015467	EMD2
GTAACACCAAATCAGA-1	singlet	1	-12.13209699	-3.989045302	-10.12948756	EMD2

GTAACACCAACCGACC-1	singlet	1	-4541.446498	-1669.640714	-3876.532942	EMD2
GTAACACCACGAGGTA-1	singlet	1	-390.6195428	-133.0131197	-353.3489215	EMD2
GTAACACGTCGCTTGG-1	singlet	2	-8142.133322	-5225.509416	-2813.905625	EMD2
GTAACACGTCTACAGT-1	singlet	1	-2092.444379	-550.3088222	-1517.3907	EMD2
GTAACACTCCATAGGT-1	singlet	0	-305.3131214	-875.3698377	-679.7656712	EMD2
GTAACCAAGGCATCAG-1	singlet	0	-293.2680705	-791.1809089	-705.7936357	EMD2
GTAACCACAAGTGCTT-1	singlet	1	-406.3788942	-142.8232049	-310.4091439	EMD2
GTAACCACACAAACGG-1	singlet	1	-431.885965	-167.9027717	-368.0898505	EMD2
GTAACCACACAGAAGC-1	singlet	0	-155.680245	-401.2805892	-411.6853726	EMD2
GTAACCAGTATCGCTA-1	singlet	1	-599.5478809	-222.7188268	-470.8775173	EMD2
GTAACCAGTGGGTCAA-1	singlet	2	-2162.849886	-1806.978204	-884.3303159	EMD2
GTAACCAGTTCCCAAA-1	doublet	45293	-4890.834183	-1807.199756	-3567.089514	EMD2
GTAACCATCAAGCCAT-1	singlet	0	-692.7833587	-2095.459917	-2111.554915	EMD2
GTAACCATCTTTCAGT-1	singlet	1	-435.1619585	-175.4969036	-325.9086826	EMD2
GTAAGTCAGACTTCAC-1	singlet	2	-2510.422453	-2365.335747	-972.0294701	EMD2
GTAAGTCAGATCGGTG-1	singlet	1	-361.8699876	-167.2969266	-327.8863807	EMD2
GTAAGTCAGATTAGAC-1	singlet	1	-2263.740432	-625.6797801	-1808.919717	EMD2
GTAAGTCAGCTGAAGC-1	singlet	2	-141.1251348	-172.5027038	-39.23168105	EMD2
GTAAGTCAGGCAGCTA-1	singlet	2	-1855.927252	-1521.545856	-647.6591927	EMD2
GTAAGTCAGTGCAGGT-1	singlet	2	-371.8391778	-388.2753132	-153.9828374	EMD2
GTAAGTCCAAATGAGT-1	singlet	1	-174.7116456	-74.36057721	-160.3022173	EMD2
GTAAGTCCAGAGTTCT-1	singlet	1	-2414.21262	-848.1187251	-1862.888937	EMD2
GTAAGTCGTAGGCAAC-1	singlet	1	-1120.478742	-311.7145271	-742.8524257	EMD2
GTAAGTCGTATGCAAA-1	singlet	1	-1559.509533	-487.741283	-1235.578576	EMD2
GTAAGTCGTCGTTGGC-1	singlet	1	-608.7513786	-206.9499147	-522.880435	EMD2
GTAAGTCGTTATCTGG-1	singlet	0	-429.6475422	-1263.533825	-1120.418591	EMD2
GTAAGTCTCCGTGGTG-1	singlet	1	-361.6265538	-163.9720369	-306.5174881	EMD2
GTAAGTCTCGTTATCT-1	singlet	1	-2477.137876	-686.5874883	-2038.43465	EMD2
GTAAGTCTCTACCCAC-1	singlet	1	-346.1656267	-134.0083586	-306.3622531	EMD2
GTAAGTCTCTCGTCGT-1	singlet	0	-535.4494786	-1546.182233	-1501.048607	EMD2
GTAATCGAGCGATCGA-1	singlet	1	-487.3372043	-133.9688481	-353.7816572	EMD2
GTAATCGAGGCCACTC-1	singlet	1	-3728.140851	-920.1964283	-2668.620204	EMD2
GTAATCGAGTGGTCAG-1	singlet	0	-312.9639448	-1113.456308	-1086.594773	EMD2
GTAATCGGTGACAACG-1	doublet	1/0	-3381.016164	-2649.992641	-3889.93544	EMD2
GTAATCGGTGGAATGC-1	singlet	1	-234.2690593	-116.5316403	-204.4566854	EMD2
GTAATCGTCCTCTGCA-1	singlet	1	-3228.291247	-899.0575572	-2410.062239	EMD2
GTAATCGTCGTCCATC-1	singlet	1	-1404.782768	-347.098829	-1327.181139	EMD2
GTAATGCAGGATATGT-1	singlet	1	-1333.147523	-475.7062449	-1106.904535	EMD2
GTAATGCAGGCTATCT-1	singlet	0	-1153.470936	-3239.423343	-3269.936019	EMD2
GTAATGCCACTCCGGA-1	singlet	1	-1040.424711	-380.9563172	-836.2248276	EMD2
GTAATGCCAGTTCTAG-1	singlet	0	-102.6259181	-256.6639388	-230.6303081	EMD2
GTAATGCCATAGTCAC-1	singlet	0	-1851.651697	-4801.645643	-4112.303119	EMD2
GTAATGCGTACAGAAT-1	unassigned	0/1	-22.58691886	-24.01004082	-19.68855605	EMD2
GTAATGCGTATGGTAA-1	singlet	1	-1200.523033	-454.1520003	-1039.86236	EMD2
GTAATGCGTATTGAGA-1	doublet	1/0	-1078.415019	-755.8870261	-1073.073218	EMD2
GTAATGCGTTGCGAAG-1	singlet	1	-1042.94914	-363.8007112	-835.1460551	EMD2
GTAATGCTCCCAGCGA-1	singlet	1	-326.4525087	-131.7454172	-293.5677929	EMD2
GTAATGCTCGGAATTC-1	singlet	1	-402.122037	-159.374585	-295.3673735	EMD2
GTAATGCTCTGAGGTT-1	singlet	1	-3108.582435	-935.3183993	-2535.492694	EMD2
GTACAACAGACCTCAT-1	singlet	1	-284.8472557	-111.1898563	-265.5193523	EMD2
GTACAACAGCACCAGA-1	singlet	1	-2809.883645	-985.7495927	-2355.411951	EMD2
GTACAACAGGTTCCAT-1	singlet	1	-1839.3436	-608.6684558	-1472.758254	EMD2
GTACAACAGTATTGCC-1	singlet	0	-101.1096352	-249.3136705	-225.0916416	EMD2
GTACAACCAGAGTTCT-1	singlet	0	-309.9295288	-933.8281377	-963.6135853	EMD2
GTACAACCATGAAAGT-1	singlet	1	-1607.3945	-603.2874281	-1362.525997	EMD2
GTACAACGTAGTAAGT-1	singlet	1	-582.9558504	-261.4338244	-542.3715163	EMD2
GTACAACGTATCAGGG-1	singlet	1	-712.0074805	-207.5354559	-698.0001892	EMD2
GTACAACGTCTGCATA-1	singlet	1	-395.4429343	-160.6504983	-303.6872876	EMD2
GTACAACTCACGAACT-1	singlet	0	-500.389808	-1639.805308	-1489.117387	EMD2
GTACAACTCCCGAGAC-1	singlet	1	-186.9717396	-92.19987639	-192.9346132	EMD2
GTACAGTAGATACAGT-1	singlet	0	-737.0116907	-1978.877217	-1976.948311	EMD2
GTACAGTAGGATGTTA-1	singlet	0	-87.4860417	-211.7033424	-199.0679125	EMD2
GTACAGTAGTCACTGT-1	singlet	1	-2144.277349	-506.8703801	-1559.496461	EMD2
GTACAGTCAGGTATGG-1	singlet	1	-474.0959764	-180.8531124	-365.0699198	EMD2
GTACAGTCATCGGCCA-1	singlet	2	-3287.408452	-2672.545494	-990.5368494	EMD2
GTACAGTGTATCTCTT-1	singlet	1	-3735.807629	-1115.421682	-2892.408151	EMD2
GTACAGTGTCGCATTA-1	doublet	0/1	-7.935531844	-14.61945556	-12.68384331	EMD2
GTACAGTGTGCAGTGA-1	singlet	1	-775.1946019	-301.1079956	-698.4757773	EMD2
GTACAGTGTGGCTACC-1	singlet	2	-374.2574057	-308.60737	-194.3330327	EMD2
GTACAGTTCCGACAGC-1	singlet	2	-359.2019209	-418.910244	-186.6918885	EMD2
GTACAGTTCGTGGCGT-1	singlet	1	-362.4407638	-143.4363274	-327.1511965	EMD2
GTAGAAAAGACCTTTG-1	singlet	0	-382.958176	-1104.528403	-1085.775176	EMD2
GTAGAAAAGACTACCT-1	singlet	0	-185.5617256	-593.8640153	-493.5887399	EMD2
GTAGAAAAGCGCCTTG-1	singlet	0	-21.75689224	-58.71712877	-57.77072904	EMD2
GTAGAAAAGGCTCCCA-1	singlet	1	-2482.627504	-937.8266794	-2314.848251	EMD2
GTAGAAAAGTGGTTCT-1	singlet	0	-376.3034473	-937.7478303	-1025.115089	EMD2
GTAGAAACAGCACAGA-1	singlet	1	-2931.191963	-942.0910135	-2344.055693	EMD2
GTAGAAACATCACCAA-1	singlet	1	-1436.997799	-543.5085448	-1273.904055	EMD2
GTAGAAAGTTAGGGAC-1	singlet	1	-648.4905435	-232.7916528	-536.6684817	EMD2
GTAGAAAGTTGCACGC-1	singlet	1	-3194.183744	-931.2688639	-2782.588845	EMD2
GTAGAAAGTTGGTGTT-1	singlet	1	-1057.568174	-370.2827177	-924.5955607	EMD2
GTAGAAATCCAGCCTT-1	singlet	1	-850.1027549	-247.8034208	-614.2605789	EMD2
GTAGAAATCCTGTTGC-1	singlet	0	-164.2546894	-408.9547894	-376.5234996	EMD2
GTAGAAATCCTTATGT-1	singlet	2	-1010.654191	-896.759602	-334.2891965	EMD2
GTAGAAATCGGATAAA-1	singlet	1	-429.7772877	-152.317437	-386.5032077	EMD2
GTAGAGGAGACCAGCA-1	singlet	1	-43.4346686	-9.916580186	-21.82017328	EMD2
GTAGAGGAGAGCCATG-1	singlet	1	-1844.260276	-636.1822868	-1667.386008	EMD2
GTAGAGGAGGAGAGGC-1	singlet	1	-1738.583569	-584.2748555	-1419.912512	EMD2
GTAGAGGAGGCAGCTA-1	singlet	2	-2227.433928	-1681.346953	-623.730306	EMD2
GTAGAGGAGTTAACAG-1	singlet	1	-2575.114667	-774.1801978	-1976.163579	EMD2
GTAGAGGCAGGATCTT-1	singlet	1	-1181.07022	-371.4183447	-974.6740344	EMD2
GTAGAGGGTCTCACAA-1	singlet	0	-906.9512426	-3101.820048	-2973.649748	EMD2
GTAGAGGGTGACATCT-1	unassigned	1/0	-6.392993999	-4.108799312	-9.323239003	EMD2
GTAGAGGGTGGACTAG-1	singlet	1	-1230.707532	-496.6663639	-1025.396575	EMD2
GTAGAGGTCGACACTA-1	singlet	1	-2889.102541	-909.0103341	-2061.30831	EMD2
GTAGATCAGACAAGCC-1	singlet	1	-4149.742961	-1043.565825	-3291.003551	EMD2
GTAGATCAGCCGGATA-1	singlet	2	-5248.990622	-4178.970427	-2070.918387	EMD2
GTAGATCCAAGTGGCA-1	doublet	0/2	-1838.733587	-2712.701426	-2016.744201	EMD2
GTAGATCCACTCATAG-1	singlet	1	-2372.254823	-759.8002971	-1858.577378	EMD2
GTAGATCCAGAGTTGG-1	unassigned	1/0	-6.976114386	-5.255764069	-8.204158838	EMD2
GTAGATCCAGCTGTGC-1	singlet	2	-131.989467	-156.4236315	-64.9397119	EMD2
GTAGATCGTCGAAACG-1	singlet	0	-61.14163528	-244.5893721	-191.2812601	EMD2
GTAGATCGTCTGTTAG-1	singlet	2	-2471.732488	-2066.952961	-861.0331288	EMD2
GTAGATCGTGGCTTGC-1	singlet	2	-2938.442993	-2361.295757	-877.8938752	EMD2
GTAGCTAAGCCGATCC-1	singlet	1	-1528.254417	-388.9025181	-1257.097491	EMD2
GTAGCTAAGGCTTAGG-1	singlet	1	-988.5028438	-313.1550935	-671.2837143	EMD2
GTAGCTAAGTCTAGCT-1	singlet	0	-1350.624961	-4118.020408	-3973.39063	EMD2
GTAGCTAAGTGCGACA-1	singlet	0	-815.0657374	-2241.113459	-2387.050101	EMD2
GTAGCTACAAATGGCG-1	singlet	1	-499.5380297	-206.954688	-451.6533306	EMD2
GTAGCTACAGACACAG-1	singlet	0	-942.6483461	-2962.599559	-2896.293021	EMD2

GTAGCTAGTAGGAGTC-1	singlet	1	-428.0590942	-149.5701611	-308.7737719	EMD2
GTAGCTAGTATTGGCT-1	singlet	2	-1424.525989	-1407.55863	-566.3067368	EMD2
GTAGCTAGTGGACCAA-1	singlet	1	-245.0718882	-53.59718976	-151.5037575	EMD2
GTAGCTAGTTAGAGTA-1	unassigned	0	-3.440813344	-3.85142353	-8.257071686	EMD2
GTAGCTATCTGCGATA-1	singlet	1	-313.4749834	-155.6577266	-260.8883826	EMD2
GTAGGAGAGAGAGAAC-1	singlet	1	-5564.609953	-1665.731249	-4377.118308	EMD2
GTAGGAGAGCCGGAAT-1	singlet	1	-1000.625482	-337.7369376	-841.4671391	EMD2
GTAGGAGAGCGTGCCT-1	singlet	1	-2337.616928	-484.6543756	-1376.647566	EMD2
GTAGGAGAGTTGGCTT-1	singlet	1	-483.3247755	-163.1036538	-413.7361266	EMD2
GTAGGAGGTCTCGCGA-1	singlet	1	-1922.881138	-690.6871289	-1409.815961	EMD2
GTAGGAGTCAATCCGA-1	doublet	45293	-6.317570956	-5.444478908	-6.19874545	EMD2
GTAGGAGTCACACCGG-1	singlet	1	-72.03945212	-17.63909935	-59.02481229	EMD2
GTAGGAGTCCGTGTCT-1	singlet	0	-906.6199398	-3718.851535	-3548.450109	EMD2
GTAGGAGTCTAACGCA-1	singlet	0	-194.5647956	-472.9582183	-524.202565	EMD2
GTAGGTTAGAAGTGTT-1	singlet	0	-817.7847661	-2538.259953	-2513.888193	EMD2
GTAGGTTAGACTCATC-1	singlet	1	-2969.844885	-869.2067192	-2243.191771	EMD2
GTAGGTTAGGTATAGT-1	singlet	1	-415.620677	-152.7467224	-366.9246111	EMD2
GTAGGTTAGTGTTGTC-1	unassigned	1	-18.60245523	-8.079012015	-10.02591006	EMD2
GTAGGTTCACCTTCCA-1	singlet	2	-564.1000743	-545.859327	-209.5290633	EMD2
GTAGGTTGTCACGCTG-1	singlet	1	-1686.038347	-534.6092	-1339.586744	EMD2
GTAGGTTGTCACTCAA-1	singlet	0	-360.9820481	-1035.829219	-955.7743551	EMD2
GTAGGTTGTGACTCTA-1	singlet	0	-855.778016	-4006.580442	-4346.959624	EMD2
GTAGGTTGTGGGTATG-1	singlet	0	-687.9430557	-2215.33348	-2044.800098	EMD2
GTAGGTTTCAACGAGG-1	singlet	2	-2224.270499	-2122.887463	-684.4690238	EMD2
GTAGGTTTCCAAAGGG-1	singlet	2	-4418.490365	-3616.111566	-1345.6545	EMD2
GTAGGTTTCCTCTCGA-1	singlet	1	-130.6621396	-45.49522943	-97.87439716	EMD2
GTAGTACAGGGTGAGG-1	singlet	0	-780.9751213	-2322.022173	-2294.927809	EMD2
GTAGTACCAGTGGGTA-1	singlet	2	-326.8315408	-344.0010558	-160.1755768	EMD2
GTAGTACCATGGAACG-1	singlet	0	-140.5297372	-246.0380769	-263.5646334	EMD2
GTAGTACGTAGACGTG-1	singlet	1	-821.794637	-261.6412504	-656.8524345	EMD2
GTAGTACGTCATCCGG-1	singlet	1	-544.1847226	-181.118509	-409.3022504	EMD2
GTAGTACGTCTCCCTA-1	singlet	1	-554.2113857	-196.623993	-477.6289406	EMD2
GTAGTACGTGCTCTTC-1	singlet	1	-1070.74922	-330.2413848	-903.0645408	EMD2
GTAGTACGTTTGTTCT-1	singlet	0	-607.7247329	-1623.89866	-1620.869713	EMD2
GTAGTACTCCTTGGAA-1	singlet	0	-174.1771337	-495.4742262	-423.0074398	EMD2
GTAGTACTCGCATTAG-1	singlet	0	-724.0161732	-2954.015576	-2612.124547	EMD2
GTAGTACTCGTCGGGT-1	singlet	0	-285.3506887	-832.2975313	-870.3835018	EMD2
GTATTGGAGACTGAGC-1	singlet	0	-274.6930661	-772.0310887	-717.9502832	EMD2
GTATTGGAGGAAGTAG-1	singlet	0	-104.3973793	-510.0379954	-422.4152449	EMD2
GTATTGGAGGTCGTAG-1	singlet	1	-486.9352231	-158.2556815	-358.4884225	EMD2
GTATTGGCACAAGCCC-1	singlet	2	-1047.994773	-1001.840685	-369.0320419	EMD2
GTATTGGCATGAAGCG-1	singlet	1	-1694.342457	-544.8573676	-1522.305184	EMD2
GTATTGGGTCCATACA-1	singlet	1	-200.4504521	-87.54715549	-239.0261579	EMD2
GTATTGGGTGACTAAA-1	singlet	1	-2929.812267	-1001.525504	-2340.305309	EMD2
GTATTGGGTGAGATTA-1	doublet	1/0	-55.2024041	-40.52648984	-52.0144497	EMD2
GTATTGGGTTGTTGTG-1	singlet	1	-2500.521734	-755.9479692	-1793.242841	EMD2
GTATTGGTCTGCGGAC-1	singlet	1	-1309.542869	-418.6828288	-1175.133983	EMD2
GTATTTCAGCATTTGC-1	singlet	2	-2914.44686	-2472.969097	-985.2352078	EMD2
GTATTTCAGCGCTGCT-1	singlet	2	-192.6178166	-156.8244651	-85.7136531	EMD2
GTATTTCAGCTAAATG-1	singlet	1	-515.7375687	-214.7861948	-413.4893357	EMD2
GTATTTCAGTGAGCCA-1	singlet	1	-2725.142281	-801.1771563	-2058.657353	EMD2
GTATTTCCAAGGCGTA-1	singlet	2	-4184.071748	-3378.955011	-1172.788103	EMD2
GTATTTCCACCTGCGA-1	singlet	2	-455.8091485	-358.3975503	-172.4190509	EMD2
GTATTTCCACTAGAGG-1	singlet	1	-1503.543986	-607.5639421	-1427.795999	EMD2
GTATTTCCACTCACTC-1	singlet	0	-275.1925422	-787.8771574	-845.0469333	EMD2
GTATTTCGTGACGCCT-1	singlet	1	-454.145329	-172.1346543	-396.1048924	EMD2
GTATTTCGTGCATTAC-1	singlet	1	-349.5123297	-132.8391175	-285.1946985	EMD2
GTATTTCTCACTGAAC-1	singlet	2	-1424.121094	-1286.359134	-596.6545076	EMD2
GTATTTCTCAGCTGAT-1	singlet	2	-1756.130335	-1461.297213	-666.8343897	EMD2
GTATTTCTCCGATAGT-1	singlet	2	-1600.43103	-1445.108689	-547.6680286	EMD2
GTATTTCTCTCATAGG-1	singlet	2	-2930.42524	-2619.968208	-1069.69633	EMD2
GTCAAACAGCTACGTT-1	singlet	2	-1734.903688	-1424.938662	-459.9136936	EMD2
GTCAAACAGGGAGTTC-1	singlet	0	-506.4924104	-1181.805393	-1170.729101	EMD2
GTCAAACAGTGGCAGT-1	singlet	1	-1527.638287	-557.7924012	-1444.272308	EMD2
GTCAAACCAATAACGA-1	singlet	0	-35.34716978	-90.21010494	-100.4233735	EMD2
GTCAAACCAGTGGTGA-1	singlet	1	-264.7157759	-116.9676167	-229.4152821	EMD2
GTCAAACGTACAGTTC-1	singlet	1	-2646.241047	-736.7468168	-1989.23001	EMD2
GTCAAACTCGCAGAGA-1	singlet	0	-530.4084221	-1614.539868	-1547.939005	EMD2
GTCAAACTCTAGTGTG-1	singlet	1	-497.6572463	-180.9765812	-381.6277489	EMD2
GTCAAGTAGCGATTCT-1	singlet	0	-557.0564396	-1623.581667	-1521.731897	EMD2
GTCAAGTAGGTCCAGA-1	singlet	1	-227.0877545	-73.30385571	-177.4875166	EMD2
GTCAAGTCACATGGTT-1	singlet	1	-137.6799302	-60.90134115	-129.8799988	EMD2
GTCAAGTCACTGAATC-1	singlet	1	-2460.099723	-847.4184197	-1938.431157	EMD2
GTCAAGTCAGACCAAG-1	singlet	1	-2780.823637	-513.2643823	-1648.185996	EMD2
GTCAAGTGTAAGCAAT-1	singlet	1	-788.4512691	-364.3403927	-723.713556	EMD2
GTCAAGTGTCAAAGTA-1	singlet	1	-1399.780433	-438.4529637	-1316.728982	EMD2
GTCAAGTGTCGGTACC-1	singlet	1	-130.4345328	-52.34292187	-101.575747	EMD2
GTCAAGTGTGGATACG-1	singlet	1	-618.7941855	-238.0556508	-553.6205476	EMD2
GTCAAGTGTTAGGCCC-1	singlet	1	-1186.973559	-404.9459902	-945.836418	EMD2
GTCAAGTTCATTGTTC-1	singlet	0	-83.27422876	-252.5169039	-234.0507459	EMD2
GTCAAGTTCCGAACGC-1	singlet	2	-491.1128691	-414.7162334	-201.7333095	EMD2
GTCAAGTTCCTTGACC-1	singlet	0	-478.7004695	-1622.709661	-1271.181607	EMD2
GTCACGGAGAAGAACG-1	singlet	1	-471.6747083	-131.571948	-346.9635095	EMD2
GTCACGGAGGGCCTCT-1	singlet	0	-118.8673509	-362.5465402	-313.1921753	EMD2
GTCACGGAGGTAACTA-1	singlet	0	-847.9407262	-2633.838942	-2507.747689	EMD2
GTCACGGCATACTGAC-1	singlet	0	-555.7776535	-1602.243108	-1710.431331	EMD2
GTCACGGGTTGGCTAT-1	singlet	1	-288.4293217	-103.7435138	-222.1020486	EMD2
GTCACGGGTTGTGTTG-1	singlet	0	-53.51360446	-127.4526056	-172.6815234	EMD2
GTCACGGTCATGGATC-1	singlet	1	-184.3033561	-75.7434904	-149.2292903	EMD2
GTCACGGTCATTTCGT-1	singlet	2	-3241.772897	-2929.028799	-1107.038273	EMD2
GTCACGGTCCGTCCTA-1	singlet	0	-160.6701069	-475.8069849	-360.1921078	EMD2
GTCACGGTCGCAGATT-1	singlet	0	-1880.747126	-6762.86611	-6409.577156	EMD2
GTCACTCAGGGTATAT-1	singlet	1	-1216.673207	-392.5738831	-910.0315915	EMD2
GTCACTCAGTACGTCT-1	singlet	2	-453.0579513	-363.7905144	-195.0622306	EMD2
GTCACTCCACGGCACT-1	singlet	0	-171.1617928	-540.9019628	-425.96331	EMD2
GTCACTCGTGCCGGTT-1	singlet	0	-205.0810576	-739.4670521	-609.3572294	EMD2
GTCACTCTCCTTCACG-1	singlet	1	-501.9829722	-207.2618553	-430.3608528	EMD2
GTCACTCTCGGAACTT-1	singlet	1	-268.4181421	-109.414872	-245.7919006	EMD2
GTCACTCTCTCGGTCT-1	singlet	1	-756.4913421	-186.683589	-597.1741188	EMD2
GTCAGCGAGACGTCCC-1	singlet	1	-685.2414613	-208.0207001	-462.2534325	EMD2
GTCAGCGAGCAACAGC-1	singlet	1	-251.5741897	-92.6123803	-158.7124596	EMD2
GTCAGCGAGCAGGGAG-1	singlet	2	-227.1241011	-228.1914349	-81.47240304	EMD2
GTCAGCGAGGGAACAA-1	singlet	0	-41.18138303	-113.0754686	-119.8383221	EMD2
GTCAGCGAGTCATGCT-1	singlet	2	-1635.373084	-1511.138596	-562.8490402	EMD2
GTCAGCGGTACTCCCT-1	singlet	2	-5578.251409	-5065.047816	-2248.311283	EMD2
GTCAGCGTCGGCATCG-1	singlet	1	-80.17359598	-20.59103727	-60.27250185	EMD2
GTCATCCAGAACCCGA-1	singlet	1	-188.1179828	-66.69452472	-157.0066717	EMD2
GTCATCCAGGTATCTC-1	singlet	0	-299.3908035	-939.4861998	-805.4490182	EMD2
GTCATCCAGTGCTACT-1	singlet	2	-2386.550639	-1875.704032	-809.716056	EMD2
GTCATCCCATGTCGTA-1	singlet	1	-326.7121325	-122.728762	-252.4982082	EMD2

GTCATCCGTGTTGACT-1	singlet	1	-294.6242695	-97.76631135	-234.7908944	EMD2
GTCATCCTCATTTCCA-1	singlet	0	-56.32368111	-212.262138	-189.4078179	EMD2
GTCATGAAGTAAGACT-1	singlet	1	-1121.505201	-381.4022183	-1008.470026	EMD2
GTCATGACAAGGGCAT-1	singlet	1	-850.923835	-281.7840227	-832.8824723	EMD2
GTCATGACACCCTATC-1	singlet	2	-2996.539531	-2522.430853	-1084.923805	EMD2
GTCATGACAGGTGTTT-1	singlet	1	-489.2600729	-188.9472705	-374.0148153	EMD2
GTCATGACATTCCTAT-1	singlet	1	-2097.361399	-670.5071847	-1864.182419	EMD2
GTCATGAGTAGAGACC-1	singlet	1	-2806.833046	-908.2495606	-2286.077262	EMD2
GTCATGAGTATGCGTT-1	singlet	0	-217.5280035	-729.8025794	-700.1778761	EMD2
GTCATGAGTCCAAAGG-1	singlet	1	-99.82011242	-20.9697755	-79.7587893	EMD2
GTCATGAGTTGCGTAT-1	singlet	1	-246.8047486	-90.52959143	-216.7983229	EMD2
GTCATGATCAACCCGG-1	singlet	2	-1925.590109	-1506.257301	-604.963628	EMD2
GTCATGATCAATCTCT-1	singlet	1	-2418.499476	-782.8113203	-1938.823095	EMD2
GTCATTTCAAGGCCTC-1	singlet	1	-1673.835122	-467.8379639	-1398.717026	EMD2
GTCATTTCAAGTACCT-1	singlet	2	-1767.162684	-1509.410742	-607.7759526	EMD2
GTCATTTCACCAGACC-1	singlet	1	-2218.242506	-793.2497278	-1807.397038	EMD2
GTCATTTCACGTTGGC-1	singlet	0	-1485.952978	-4489.120296	-4180.250338	EMD2
GTCATTTCATAGTCAC-1	singlet	1	-485.0051713	-232.6946756	-388.4829874	EMD2
GTCATTTGTAGAGCTG-1	singlet	0	-317.6820066	-1165.077204	-916.5362909	EMD2
GTCATTTGTCGCACAC-1	singlet	0	-267.6599204	-717.645288	-727.3791012	EMD2
GTCATTTGTCTTTATC-1	singlet	2	-2567.181411	-2260.685675	-742.4434158	EMD2
GTCATTTGTTTCAGAC-1	singlet	2	-3252.441857	-3079.010156	-947.2153997	EMD2
GTCATTTTCACCGACG-1	singlet	2	-245.8915716	-245.0632811	-71.07064535	EMD2
GTCATTTTCGAACCTA-1	singlet	2	-2290.79811	-1799.164009	-746.2882855	EMD2
GTCATTTTCGCCGAGT-1	singlet	1	-1914.575401	-626.2282332	-1688.170309	EMD2
GTCCACTAGATGAATC-1	singlet	0	-567.6209863	-2123.418876	-1693.720709	EMD2
GTCCACTAGCGTTCAT-1	singlet	1	-762.9553367	-275.0310321	-590.450991	EMD2
GTCCACTAGGCTATCT-1	singlet	1	-2892.87475	-902.7931351	-2051.611311	EMD2
GTCCACTAGGGTCTTT-1	singlet	1	-365.1338168	-154.8782744	-353.8194774	EMD2
GTCCACTAGTATCTGC-1	singlet	2	-316.4967767	-292.724544	-105.4079106	EMD2
GTCCACTCACGGTGTC-1	singlet	1	-281.9037826	-142.0124925	-243.2873205	EMD2
GTCCACTCAGTTAGAA-1	singlet	0	-134.8676839	-326.9648019	-237.8842386	EMD2
GTCCACTCATCAACCA-1	singlet	1	-889.8858048	-333.5532219	-785.8731719	EMD2
GTCCACTGTCACAGTT-1	singlet	1	-161.6785783	-88.20187132	-150.0675517	EMD2
GTCCACTGTCATCCGG-1	singlet	1	-286.9254951	-109.3873851	-280.9328976	EMD2
GTCCACTGTTGATGTC-1	singlet	1	-1216.887011	-414.7778715	-1012.489594	EMD2
GTCCACTTCCGTCCTA-1	singlet	2	-1569.386098	-1434.950501	-623.5929313	EMD2
GTCCACTTCGCTCATC-1	singlet	1	-704.1618158	-281.5453934	-578.7539968	EMD2
GTCCACTTCTACCAGA-1	singlet	1	-353.7605558	-145.4953209	-348.6538074	EMD2
GTCCCATAGAATCTAG-1	singlet	1	-1180.420953	-493.7041164	-1023.982639	EMD2
GTCCCATAGCGCCTCA-1	singlet	1	-1698.872314	-497.2887181	-1357.149764	EMD2
GTCCCATAGGGAACAA-1	singlet	1	-97.61443712	-27.15911865	-74.68206619	EMD2
GTCCCATCATTACTCT-1	singlet	0	-256.3407461	-476.2771745	-532.436928	EMD2
GTCCCATGTGAGATAT-1	singlet	1	-988.9668087	-297.57736	-718.6982035	EMD2
GTCCCATGTTCATCGA-1	singlet	0	-930.3236205	-2966.883402	-2858.950709	EMD2
GTCCCATTCCGGTTCT-1	singlet	0	-1393.281022	-4164.063292	-4134.280244	EMD2
GTCCCATTCTGAGGCC-1	singlet	0	-723.6795009	-2513.443699	-2108.896131	EMD2
GTCCTCAAGACCATTC-1	singlet	1	-1818.264746	-665.8763523	-1502.294073	EMD2
GTCCTCACACAGCGCT-1	singlet	1	-572.9821907	-210.3163048	-490.6737616	EMD2
GTCCTCACAGCGACAA-1	singlet	2	-681.250806	-582.4730403	-248.3871538	EMD2
GTCCTCACAGGCTTGC-1	singlet	2	-1862.088115	-1693.812085	-615.305042	EMD2
GTCCTCACAGGTCCGT-1	singlet	1	-1123.163987	-266.5504009	-926.5640122	EMD2
GTCCTCAGTAGTGTGG-1	singlet	0	-322.0910109	-968.8601861	-923.1966705	EMD2
GTCCTCAGTCCCTAAA-1	singlet	1	-4884.535461	-1532.208385	-3691.623774	EMD2
GTCCTCATCCTCAGAA-1	singlet	2	-2954.637159	-2375.680783	-1025.758242	EMD2
GTCCTCATCCTCTCTT-1	singlet	2	-2298.161386	-2199.805869	-916.7060297	EMD2
GTCGAATAGATTAGTG-1	singlet	1	-191.4637843	-94.66316051	-194.0645404	EMD2
GTCGAATAGGCGAAGG-1	singlet	1	-261.089723	-94.17897278	-219.7288301	EMD2
GTCGAATCAAGATTGA-1	singlet	1	-295.8312475	-128.6710365	-279.4141494	EMD2
GTCGAATCACAGAGCA-1	singlet	1	-345.7592409	-127.8328636	-261.9916348	EMD2
GTCGAATCACATCCCT-1	singlet	1	-3254.509588	-1000.951456	-2681.007234	EMD2
GTCGAATCACCATTCC-1	singlet	1	-1737.876959	-360.0440838	-1063.340633	EMD2
GTCGAATCAGCACACC-1	singlet	0	-556.6025935	-1645.650839	-1534.227488	EMD2
GTCGAATCATACGCAT-1	singlet	0	-77.26327914	-259.5845698	-202.4413412	EMD2
GTCGAATGTACTCGTA-1	singlet	1	-685.746365	-210.6196941	-503.225658	EMD2
GTCGAATTCAAGCCAT-1	singlet	1	-979.0633186	-296.3098865	-819.3472652	EMD2
GTCGAATTCACACGAT-1	singlet	2	-2521.654665	-2458.313384	-853.4206399	EMD2
GTCGAATTCAGCAATC-1	singlet	1	-651.8739171	-208.9487413	-475.5162342	EMD2
GTCGAATTCCACAAGT-1	singlet	1	-48.72993677	-17.08994539	-33.47648425	EMD2
GTCGAATTCCGTGACG-1	singlet	1	-331.523827	-106.5069993	-195.1315121	EMD2
GTCGCGAAGATGGTAT-1	singlet	2	-1358.105169	-1048.629343	-419.6174662	EMD2
GTCGCGAAGCCGGAAT-1	singlet	2	-96.42471272	-77.71379262	-48.07740213	EMD2
GTCGCGAAGCCTCCAG-1	singlet	1	-724.2491459	-274.2711833	-613.5172229	EMD2
GTCGCGAAGGTTCAGG-1	singlet	1	-715.7238731	-238.5441344	-563.6066417	EMD2
GTCGCGAAGTCTTGGT-1	singlet	2	-298.6674925	-314.4690247	-139.5804972	EMD2
GTCGCGAAGTTGAATG-1	singlet	0	-384.6235012	-1141.195268	-1093.654883	EMD2
GTCGCGACACGACAGA-1	singlet	1	-1413.567467	-338.1810779	-1583.980691	EMD2
GTCGCGACAGGGACTA-1	singlet	1	-1287.583182	-409.6204866	-1035.344615	EMD2
GTCGCGACATAATCCG-1	singlet	1	-134.7866031	-40.51679517	-115.3762799	EMD2
GTCGCGACATCTCGTC-1	singlet	1	-2553.042289	-797.8887638	-2182.044476	EMD2
GTCGCGAGTACAGAGC-1	singlet	1	-1085.796881	-345.6966185	-916.0340136	EMD2
GTCGCGAGTGACAACG-1	singlet	1	-614.0758742	-229.1743466	-509.2315401	EMD2
GTCGCGAGTGAGGAAA-1	singlet	2	-2212.735491	-1792.573857	-663.8659212	EMD2
GTCGCGAGTGGACAGT-1	singlet	1	-267.1943938	-111.5942912	-257.402485	EMD2
GTCGCGAGTGGCAACA-1	singlet	1	-725.6189529	-307.9690162	-605.8176129	EMD2
GTCGCGAGTGTATTGC-1	singlet	0	-62.48981237	-192.9776411	-225.9080773	EMD2
GTCGCGAGTTTGAACC-1	singlet	1	-1145.541499	-343.4222432	-808.4017979	EMD2
GTCGCGATCAGATGCT-1	singlet	0	-845.2445283	-2655.266644	-2688.575058	EMD2
GTCGCGATCAGGCGAA-1	singlet	2	-309.9127441	-323.6792308	-139.734827	EMD2
GTCGCGATCGGACTGC-1	singlet	1	-1270.042036	-383.1251275	-1010.186317	EMD2
GTCGTAAAGGAGGTTC-1	singlet	1	-2273.191546	-665.8624719	-1891.382297	EMD2
GTCGTAAAGTTGTAAG-1	doublet	1/0	-1143.373978	-974.5946312	-1297.807421	EMD2
GTCGTAACAAGGCCTC-1	singlet	1	-1513.587668	-464.7378849	-1232.008693	EMD2
GTCGTAACACAACGAG-1	singlet	1	-400.0186772	-160.6241378	-379.9491627	EMD2
GTCGTAACACCCTCTA-1	singlet	1	-120.1380228	-49.8124584	-124.055315	EMD2
GTCGTAACAGCTACCG-1	singlet	0	-908.2148104	-3197.854695	-3175.611475	EMD2
GTCGTAAGTATAATGG-1	singlet	1	-446.9621035	-183.3904472	-391.585575	EMD2
GTCGTAAGTCCCAAAT-1	singlet	0	-164.384023	-540.4252084	-526.3997712	EMD2
GTCGTAAGTGGTTTGT-1	singlet	1	-1424.202396	-424.3503894	-1151.263629	EMD2
GTCGTAAGTTCTAACG-1	singlet	0	-138.2328582	-489.2796208	-337.7419986	EMD2
GTCGTAATCGACGCGT-1	singlet	2	-1878.498632	-1496.008562	-540.2373069	EMD2
GTCGTTCAGCCTTTGA-1	singlet	1	-245.647385	-82.23761279	-208.6662067	EMD2
GTCGTTCAGGAGCAAA-1	singlet	1	-893.6650338	-351.7500881	-797.1907347	EMD2
GTCGTTCAGGTCGTGA-1	singlet	0	-426.1293326	-1367.304359	-1250.179895	EMD2
GTCGTTCCAAGTGCTT-1	singlet	1	-1076.590673	-383.045635	-872.4244448	EMD2
GTCGTTCCAATTGAAG-1	singlet	2	-513.3557527	-484.4459128	-183.6062726	EMD2
GTCGTTCCATCACGGC-1	singlet	2	-562.9465762	-425.6154227	-193.8183603	EMD2
GTCGTTCGTAACGCGA-1	singlet	1	-822.3187634	-297.7358861	-717.1390687	EMD2
GTCGTTCGTCGTGATT-1	doublet	0/1	-144.1360855	-156.0293406	-240.3458571	EMD2
GTCGTTCGTTACCCTC-1	singlet	1	-255.6641154	-86.08752257	-183.2523506	EMD2

GTCTACCAGCAAGTCG-1	singlet	1	-140.2117791	-77.95080569	-128.5589379	EMD2
GTCTACCAGGAAGTGA-1	singlet	0	-665.9535627	-1830.264628	-1841.363866	EMD2
GTCTACCAGGGCAAGG-1	singlet	2	-939.3340571	-814.422401	-303.4138851	EMD2
GTCTACCGTCATACCA-1	singlet	0	-497.1194233	-2121.946783	-1735.819134	EMD2
GTCTACCGTCCAACGC-1	doublet	0/2	-2066.043175	-3521.672243	-2677.266677	EMD2
GTCTACCGTCCGGATC-1	singlet	1	-2173.143555	-710.5691999	-1692.329543	EMD2
GTCTACCGTCGGATTT-1	singlet	1	-602.5330962	-197.2117731	-451.6229209	EMD2
GTCTACCGTCTTCTAT-1	singlet	1	-2240.191833	-617.3542571	-1657.811414	EMD2
GTCTACCGTGTCATCA-1	singlet	2	-544.5552765	-493.6360864	-258.927753	EMD2
GTCTACCTCATGCCGG-1	singlet	2	-729.1271502	-880.0229568	-267.0633457	EMD2
GTCTACCTCGCATTGA-1	singlet	1	-2647.278062	-850.0382591	-1926.857708	EMD2
GTCTACCTCTTACACT-1	singlet	1	-5065.994519	-1540.359382	-3811.816432	EMD2
GTCTAGAAGATACATG-1	singlet	2	-542.1670128	-481.0434508	-233.7477699	EMD2
GTCTAGAAGATGGGCT-1	singlet	1	-168.0720425	-76.64898488	-129.3520319	EMD2
GTCTAGAAGCGGTATG-1	singlet	0	-20.45265321	-34.07308899	-30.50286062	EMD2
GTCTAGAAGGATGTTA-1	singlet	0	-860.7568691	-2476.03903	-2320.695343	EMD2
GTCTAGACAAGGGTCA-1	singlet	1	-1735.345871	-503.8084354	-1427.544485	EMD2
GTCTAGAGTAGACGTG-1	singlet	0	-360.4876973	-1012.482161	-872.1199357	EMD2
GTCTAGAGTGAAAGTT-1	singlet	2	-3458.164833	-2680.643103	-991.9460534	EMD2
GTCTAGAGTGGCAGAT-1	doublet	0/2	-932.8850576	-1576.39291	-1100.44012	EMD2
GTCTAGAGTTGTCAGT-1	singlet	1	-114.3583736	-36.11993506	-75.73237507	EMD2
GTCTAGATCAGTGTTG-1	singlet	2	-3439.842457	-2746.833582	-1079.666851	EMD2
GTCTAGATCCCTCATG-1	singlet	1	-709.3526157	-225.9431254	-530.7677994	EMD2
GTCTCACAGCCTGCCA-1	singlet	0	-131.8270432	-264.6875789	-259.8591145	EMD2
GTCTCACAGGAGATAG-1	singlet	0	-918.8623638	-3276.700985	-2858.685279	EMD2
GTCTCACCAATCTGCA-1	singlet	1	-307.2335115	-132.3317617	-231.7774025	EMD2
GTCTCACCACAAAGCG-1	singlet	1	-770.693039	-297.7977216	-577.7994815	EMD2
GTCTCACCACACAGCC-1	singlet	1	-706.0642522	-295.8046395	-555.0248818	EMD2
GTCTCACCATGTGGCC-1	singlet	0	-755.737001	-3099.271769	-2839.874694	EMD2
GTCTCACGTACCGGAA-1	singlet	2	-1146.929967	-944.2298166	-423.8282286	EMD2
GTCTCACGTATGATCC-1	singlet	0	-647.4122399	-1642.52136	-1812.878234	EMD2
GTCTCACGTCGAGATG-1	singlet	0	-792.4689926	-2684.077184	-2446.270546	EMD2
GTCTCACGTGACGTCC-1	singlet	1	-1862.922832	-709.3851128	-1601.610773	EMD2
GTCTCACTCAGCATTG-1	singlet	1	-1764.84242	-644.5917182	-1637.917162	EMD2
GTCTCACTCCATCTAT-1	singlet	2	-2119.041676	-1948.79968	-805.1137904	EMD2
GTCTGTCAGAGTCTTC-1	singlet	2	-530.1735037	-489.5341231	-241.5893054	EMD2
GTCTGTCCACCTCAGG-1	singlet	1	-728.9812846	-261.0655617	-611.2003352	EMD2
GTCTGTCCAGATTAAG-1	singlet	1	-237.0948248	-99.5321031	-190.8919951	EMD2
GTCTGTCGTACCGTCG-1	singlet	0	-605.6832936	-1933.094111	-1759.932808	EMD2
GTCTGTCGTACTCCGG-1	singlet	1	-1832.18798	-538.9565273	-1434.932992	EMD2
GTCTGTCGTCACAATC-1	singlet	2	-1429.68928	-1207.449855	-485.5619788	EMD2
GTCTGTCGTCACTCTC-1	singlet	0	-548.0846889	-1464.891468	-1441.456559	EMD2
GTCTGTCGTCAGACGA-1	unassigned	1/0	-124.4094221	-57.05416336	-89.46156929	EMD2
GTCTGTCGTCTCCCTA-1	unassigned	2/0	-3.082055813	-11.08410519	-2.934702603	EMD2
GTCTGTCTCAACGAGG-1	doublet	0/1	-2157.828795	-2828.97074	-3082.760879	EMD2
GTCTGTCTCTCTATAC-1	singlet	0	-83.74970262	-208.7411386	-247.1615538	EMD2
GTCTTTAAGAACCCGA-1	singlet	1	-2788.451857	-945.8633755	-2394.827948	EMD2
GTCTTTAAGGTAAGGA-1	singlet	2	-2568.97723	-1933.726931	-1018.170596	EMD2
GTCTTTACACAATGTC-1	singlet	2	-280.5916267	-229.3307245	-103.8172695	EMD2
GTCTTTACACTCCTGT-1	singlet	1	-881.8027286	-321.5572408	-710.833654	EMD2
GTCTTTAGTGAGATAT-1	singlet	0	-1180.115578	-2106.560389	-2252.352	EMD2
GTCTTTAGTGCTGATT-1	singlet	2	-10.58629046	-12.51989244	-5.827643065	EMD2
GTCTTTAGTTCGGCCA-1	doublet	0/1	-798.7213561	-972.4427975	-1086.24881	EMD2
GTCTTTAGTTGACTAC-1	singlet	1	-326.7667586	-137.8664018	-246.4003941	EMD2
GTCTTTATCTGTTGGA-1	singlet	0	-170.8801813	-558.4873183	-489.6524373	EMD2
GTGACGCAGATCGCTT-1	singlet	0	-106.8594346	-321.8441411	-262.8616889	EMD2
GTGACGCAGGGATGTC-1	singlet	2	-293.1709639	-318.6113656	-130.1774931	EMD2
GTGACGCCACGCTATA-1	singlet	1	-1159.08522	-450.0040069	-990.1265034	EMD2
GTGACGCCACTACCCT-1	singlet	0	-207.9020722	-488.7037551	-488.9311638	EMD2
GTGACGCCAGCCTTCT-1	singlet	1	-1087.740182	-347.3739782	-937.9110458	EMD2
GTGACGCCAGGAATCG-1	singlet	1	-1192.396512	-383.0374709	-1152.654715	EMD2
GTGACGCCAGTTGTCA-1	singlet	1	-2384.659999	-758.3436982	-1922.981981	EMD2
GTGACGCGTATCGCTA-1	singlet	0	-67.37619865	-179.1792163	-163.1775927	EMD2
GTGACGCGTGCACATT-1	singlet	0	-235.6729638	-879.6848791	-878.8814617	EMD2
GTGACGCGTGTGAATA-1	singlet	0	-166.2528671	-404.0827779	-355.0738776	EMD2
GTGACGCTCTTGGTGA-1	singlet	1	-261.5197458	-99.3880689	-248.9770646	EMD2
GTGAGCCAGAGTGACC-1	singlet	1	-337.4226007	-139.0036954	-306.1343914	EMD2
GTGAGCCAGGAACATT-1	singlet	0	-817.1023731	-2290.259961	-2038.777127	EMD2
GTGAGCCAGGGAGATA-1	singlet	1	-240.1607042	-103.6637199	-205.784156	EMD2
GTGAGCCAGGGCATGT-1	singlet	1	-2107.854314	-604.79366	-1603.467104	EMD2
GTGAGCCAGTCACGCC-1	singlet	0	-585.239749	-2111.769692	-1958.05597	EMD2
GTGAGCCAGTCCGCGT-1	singlet	2	-570.9728531	-556.3095957	-212.2801599	EMD2
GTGAGCCAGTCGAAAT-1	singlet	1	-958.0723944	-319.3595646	-735.2472211	EMD2
GTGAGCCCAAATCCCA-1	singlet	2	-810.0844459	-753.1689022	-250.8970981	EMD2
GTGAGCCGTTCACGAT-1	singlet	1	-1694.514001	-532.1545705	-1480.368652	EMD2
GTGAGCCTCAACGCTA-1	singlet	1	-248.0225089	-101.1092746	-255.1576088	EMD2
GTGAGCCTCATCGCTC-1	singlet	1	-1182.197118	-308.7183814	-910.5142542	EMD2
GTGAGCCTCTCGACCT-1	singlet	0	-1148.708036	-4137.725471	-4085.325627	EMD2
GTGAGGAAGTAGACCG-1	singlet	2	-5160.268356	-4197.146365	-1581.66318	EMD2
GTGAGGACAATCCTAG-1	singlet	0	-476.2970661	-1700.661336	-1638.123221	EMD2
GTGAGGACACCGCTAG-1	singlet	2	-1125.092691	-1012.607301	-409.9955454	EMD2
GTGAGGACACTACACA-1	singlet	1	-528.8876888	-166.8083028	-436.2080333	EMD2
GTGAGGAGTAACTAAG-1	singlet	2	-2709.662261	-2150.939385	-864.5945173	EMD2
GTGAGGATCCTCACTG-1	singlet	1	-1424.508284	-551.5021655	-1261.981217	EMD2
GTGAGGATCTGAGCAT-1	singlet	2	-2739.219763	-2696.398922	-728.2307724	EMD2
GTGAGTTAGATGAACT-1	singlet	2	-3068.572742	-2860.244989	-972.7081119	EMD2
GTGAGTTAGCGCTTCG-1	singlet	1	-580.7779191	-211.8932156	-498.6878241	EMD2
GTGAGTTAGCTAGCCC-1	singlet	1	-228.1813639	-111.0223459	-191.5075414	EMD2
GTGAGTTAGGACATCG-1	singlet	0	-1181.346373	-3582.712004	-3540.514482	EMD2
GTGAGTTAGTGCACAG-1	singlet	1	-591.8571084	-225.5394372	-478.3740097	EMD2
GTGAGTTCAGGTTCCG-1	singlet	0	-192.9258708	-429.8764805	-456.3104671	EMD2
GTGAGTTGTAGAGACC-1	singlet	1	-322.1290328	-134.8139754	-287.5686714	EMD2
GTGAGTTGTCTGATAC-1	singlet	1	-1902.659349	-605.8109128	-1756.312708	EMD2
GTGAGTTGTGACAGCA-1	singlet	1	-1136.690604	-374.6753543	-978.7776991	EMD2
GTGAGTTTCAATGCAC-1	singlet	2	-2787.334584	-2226.536992	-883.0862026	EMD2
GTGAGTTTCCAGGACC-1	singlet	1	-474.9387266	-180.404747	-333.1438716	EMD2
GTGAGTTTCGACCCAG-1	singlet	1	-240.7946316	-81.06230819	-210.1899849	EMD2
GTGATGTAGACCAGCA-1	singlet	2	-3722.204128	-3005.328699	-1012.073666	EMD2
GTGATGTAGGCAGGTT-1	singlet	0	-1022.008927	-3520.150753	-3389.030174	EMD2
GTGATGTAGTCCGCGT-1	singlet	1	-496.1048768	-179.4831939	-434.8425176	EMD2
GTGATGTAGTTAACGA-1	singlet	1	-421.8007674	-170.9709966	-384.2194085	EMD2
GTGATGTCAATGAACA-1	singlet	0	-252.8869397	-704.5745212	-702.4488382	EMD2
GTGATGTCACTCTCGT-1	singlet	1	-640.2721294	-187.1889119	-403.3938214	EMD2
GTGATGTCAGCAGATG-1	singlet	1	-569.5967792	-188.0944985	-377.3504253	EMD2
GTGATGTCAGCGAACA-1	singlet	2	-5076.121965	-3978.678285	-1481.284117	EMD2
GTGATGTCAGGAAGTC-1	singlet	1	-3303.284428	-983.3167027	-2652.286096	EMD2
GTGATGTCATCCGGCA-1	singlet	2	-263.4700261	-227.0730139	-89.96200499	EMD2
GTGATGTGTACGATCT-1	singlet	0	-1606.329193	-4323.480893	-4361.99099	EMD2
GTGATGTGTCCTACGG-1	singlet	1	-291.5020996	-134.114254	-282.1929579	EMD2
GTGATGTGTGCGACAA-1	singlet	0	-632.8408734	-2247.635249	-2036.491882	EMD2

GTGATGTGTTTCGCTC-1	singlet	1	-1004.278944	-323.9730974	-805.7333422	EMD2
GTGATGTTCACCATGA-1	singlet	1	-2830.826195	-929.8080764	-2292.632327	EMD2
GTGATGTTCCTAACAG-1	singlet	2	-53.3180711	-42.39741167	-24.44160495	EMD2
GTGATGTTCGACCATA-1	singlet	1	-1890.988166	-666.259765	-1532.460445	EMD2
GTGATGTTCTCATGGA-1	singlet	1	-2265.880411	-652.8002904	-1903.902847	EMD2
GTGATGTTCTGTGCTC-1	singlet	0	-36.97282538	-93.88676956	-84.37052861	EMD2
GTGCACGCAACAACAA-1	singlet	0	-154.4625504	-474.7766116	-459.9055681	EMD2
GTGCACGCAGTTAAAG-1	singlet	1	-1181.413574	-432.3571712	-1092.727581	EMD2
GTGCACGCATCACCAA-1	singlet	0	-718.2138916	-2135.233881	-2011.071103	EMD2
GTGCACGCATGTGCCG-1	singlet	0	-1251.222569	-4669.174049	-4222.738824	EMD2
GTGCACGGTATTCTCT-1	singlet	1	-412.1792565	-172.7849021	-337.1740188	EMD2
GTGCACGGTCAATCTG-1	singlet	1	-394.0223477	-164.2787933	-291.6524887	EMD2
GTGCACGGTGAGCCAA-1	singlet	1	-344.8047066	-139.4153602	-271.6350363	EMD2
GTGCACGGTGTAAATG-1	singlet	1	-374.5503083	-171.7429782	-363.3783076	EMD2
GTGCACGTCATCACAG-1	singlet	0	-84.74499333	-302.6986981	-214.954763	EMD2
GTGCACGTCGTCGATA-1	singlet	2	-6180.500993	-4885.281083	-1701.708206	EMD2
GTGCAGCAGGATCATA-1	singlet	1	-268.7842987	-110.7795306	-211.7726195	EMD2
GTGCAGCAGGTCCTGC-1	singlet	0	-541.0019467	-1762.003212	-1628.31907	EMD2
GTGCAGCCAACTCATG-1	doublet	1/0	-1471.723063	-939.3853606	-1464.348643	EMD2
GTGCAGCCAACTGTGT-1	singlet	0	-170.2970153	-450.3792641	-433.3345574	EMD2
GTGCAGCCAATAACGA-1	singlet	0	-593.834688	-1946.666272	-1918.66198	EMD2
GTGCAGCCACAAGCTT-1	singlet	1	-269.5032418	-96.65213872	-282.16521	EMD2
GTGCAGCCACTACCCT-1	doublet	45293	-3963.328217	-2623.752193	-2917.715803	EMD2
GTGCAGCCAGTTGAAA-1	singlet	1	-1108.929305	-339.4003111	-924.5183006	EMD2
GTGCAGCGTCAAGGCA-1	singlet	1	-658.0806819	-225.2093951	-542.7735068	EMD2
GTGCAGCGTTCATCGA-1	singlet	1	-337.7579782	-135.0883425	-261.9112873	EMD2
GTGCAGCGTTGTGTTG-1	singlet	0	-722.9065037	-2082.662079	-1997.404162	EMD2
GTGCAGCTCCGCGGAT-1	singlet	1	-707.9389298	-269.8387578	-620.9677977	EMD2
GTGCAGCTCGCCTATC-1	singlet	2	-1522.615099	-1176.715294	-556.0786956	EMD2
GTGCAGCTCGTTTACT-1	singlet	1	-1447.833128	-484.7650359	-1237.530688	EMD2
GTGCAGCTCTTACCAT-1	singlet	2	-2404.683228	-1970.063414	-991.544293	EMD2
GTGCGTGAGCGATTCT-1	singlet	1	-95.3220872	-32.25347127	-79.88175593	EMD2
GTGCGTGAGCTACTGT-1	singlet	1	-615.7481737	-203.1641186	-521.0089818	EMD2
GTGCGTGAGTATGCAA-1	singlet	2	-2451.314337	-2317.234222	-850.7075778	EMD2
GTGCGTGAGTCGAATA-1	singlet	0	-303.2069575	-873.0879127	-822.8796732	EMD2
GTGCGTGCAATCTCGA-1	singlet	0	-1589.386243	-5851.804047	-5808.71019	EMD2
GTGCGTGCACCAAATC-1	singlet	1	-1084.011777	-375.9533394	-763.6165024	EMD2
GTGCGTGCACTATCGA-1	singlet	1	-667.3670011	-262.6096635	-533.6664048	EMD2
GTGCGTGCAGAACTAA-1	singlet	1	-2764.350751	-861.3784362	-2321.797906	EMD2
GTGCGTGCAGGAACCA-1	singlet	1	-1740.27561	-475.8412359	-1326.07015	EMD2
GTGCGTGCAGGGCTTC-1	singlet	1	-252.2902429	-103.5403792	-203.5729766	EMD2
GTGCGTGGTAGGTGCA-1	singlet	0	-143.9500179	-435.1156844	-415.7327726	EMD2
GTGCGTGGTATTGCCA-1	singlet	1	-420.5031943	-115.3516014	-285.2478925	EMD2
GTGCGTGTCAAGGACG-1	singlet	2	-237.9670236	-280.2281975	-109.2583263	EMD2
GTGCGTGTCCCGATCT-1	singlet	1	-2403.94196	-792.490472	-2128.5909	EMD2
GTGCGTGTCCTTTAGT-1	singlet	1	-1103.574469	-299.3893619	-751.3258432	EMD2
GTGCGTGTCGCATGAT-1	singlet	2	-2271.020679	-1892.756952	-883.5206836	EMD2
GTGCGTGTCTCACCCA-1	singlet	2	-425.6895835	-517.4813766	-182.9511512	EMD2
GTGCGTGTCTTCGATT-1	singlet	1	-1311.269735	-400.4204047	-1163.251097	EMD2
GTGCGTGTCTTCGTGC-1	singlet	0	-601.3180878	-1874.641525	-1629.76371	EMD2
GTGCTGGAGAGGGTCT-1	singlet	0	-567.5538402	-1715.639161	-1640.261515	EMD2
GTGCTGGAGTCGGGAT-1	singlet	1	-204.5592419	-75.23375601	-176.4468766	EMD2
GTGCTGGCAACCCGCA-1	doublet	45323	-2168.379292	-1312.080326	-1154.146183	EMD2
GTGCTGGGTCGTCGGT-1	singlet	1	-1517.865893	-435.2099127	-1348.616867	EMD2
GTGCTGGTCGTTCCTG-1	singlet	1	-477.3206625	-173.5123337	-405.1569478	EMD2
GTGCTTCAGATGTTGA-1	singlet	1	-1895.141718	-635.6168234	-1656.289302	EMD2
GTGCTTCAGGCTATCT-1	singlet	2	-84.63778274	-81.2569968	-40.24917245	EMD2
GTGCTTCAGTCACTAC-1	singlet	0	-1792.462471	-6527.092208	-6214.931119	EMD2
GTGCTTCAGTTTGAGA-1	singlet	1	-420.4179727	-143.388787	-367.8252172	EMD2
GTGCTTCCATCGAAGG-1	singlet	2	-613.291082	-607.7597929	-234.9618007	EMD2
GTGCTTCGTATAATGG-1	singlet	1	-1503.020275	-531.0759253	-1215.284849	EMD2
GTGCTTCGTCATATGC-1	singlet	1	-250.6872391	-93.07009993	-203.7470094	EMD2
GTGCTTCGTCATCACA-1	singlet	0	-555.4352773	-1775.985602	-1711.892518	EMD2
GTGCTTCGTGAGGCAT-1	singlet	1	-275.7826527	-101.691331	-214.1692065	EMD2
GTGCTTCGTGCTGATT-1	singlet	2	-4019.295416	-3548.048397	-1193.365052	EMD2
GTGCTTCTCAAATAGG-1	singlet	1	-289.9989851	-107.2724968	-266.2732821	EMD2
GTGGAAGAGACGGAAA-1	singlet	1	-287.6018535	-131.121921	-274.7965338	EMD2
GTGGAAGAGGATCATA-1	singlet	1	-753.6821937	-246.0350924	-566.8633645	EMD2
GTGGAAGAGGCTCTCG-1	singlet	2	-7773.845593	-5949.686463	-2732.491625	EMD2
GTGGAAGCACTACAGT-1	singlet	2	-1756.210299	-1621.927794	-631.6379039	EMD2
GTGGAAGGTCGCAACC-1	doublet	45323	-1844.487115	-1341.547358	-1036.370607	EMD2
GTGGAAGGTGGTCTTA-1	singlet	0	-842.6991895	-2227.168132	-2286.950581	EMD2
GTGGAAGTCATCTACT-1	singlet	1	-479.2397451	-155.9907746	-395.692191	EMD2
GTGGAAGTCCACTAGA-1	singlet	1	-230.9464284	-99.2829338	-212.508963	EMD2
GTGGAAGTCCATCTGC-1	singlet	2	-1545.401933	-1454.680478	-605.7039298	EMD2
GTGGAGAAGCTGAAAT-1	doublet	0/1	-1193.119381	-1954.007138	-2172.053692	EMD2
GTGGAGACAAATCAGA-1	singlet	0	-911.0138983	-3024.711667	-2887.752407	EMD2
GTGGAGACACATAGCT-1	singlet	0	-1658.492802	-5323.671231	-4877.558339	EMD2
GTGGAGAGTTCGGGTC-1	singlet	1	-574.3947043	-224.680345	-468.4462252	EMD2
GTGGAGATCCTGGGAC-1	singlet	1	-1934.891967	-696.2585085	-1595.903219	EMD2
GTGGAGATCTTACGGA-1	singlet	1	-767.4987848	-238.9104019	-569.1241824	EMD2
GTGGAGATCTTTGCGC-1	singlet	1	-1999.354201	-587.4334468	-1683.413075	EMD2
GTGGCGTCAGTCAACT-1	doublet	45293	-314.9655338	-155.8154222	-245.4715127	EMD2
GTGGCGTCATCCGGTG-1	singlet	1	-352.5367423	-139.2705748	-356.7335269	EMD2
GTGGCGTGTATATGGA-1	singlet	0	-446.8088877	-1322.505378	-1251.876892	EMD2
GTGGCGTGTCCTTGTC-1	singlet	0	-332.3599251	-1326.473227	-973.5563276	EMD2
GTGGCGTGTGATGGCA-1	singlet	0	-597.6099927	-1641.819871	-1753.59093	EMD2
GTGGCGTTCAGTCACA-1	singlet	1	-312.5503976	-122.6256533	-274.8794315	EMD2
GTGGCGTTCATCGGGC-1	singlet	1	-859.6080841	-321.3869556	-635.5203202	EMD2
GTGGCGTTCTCCTGCA-1	singlet	1	-985.1398716	-373.3197271	-869.7202858	EMD2
GTGGGAAAGAGGGTAA-1	unassigned	2/0	-12.67187549	-11.97606714	-7.64299962	EMD2
GTGGGAACAGGGAATC-1	singlet	1	-342.6907261	-138.1260064	-336.201822	EMD2
GTGGGAAGTCATCCCT-1	singlet	2	-741.4499315	-660.6261001	-284.8183421	EMD2
GTGGGAAGTCGAACGA-1	singlet	1	-1404.740281	-348.2160722	-961.9170773	EMD2
GTGGGAAGTTCGAAGG-1	singlet	0	-748.2359765	-2438.250369	-2134.885539	EMD2
GTGGGAATCCCTCTCC-1	singlet	0	-811.7083364	-2458.745219	-2247.930471	EMD2
GTGGGAATCCTACACC-1	singlet	1	-1670.594464	-516.4384516	-1372.576876	EMD2
GTGGGAATCCTGTTGC-1	singlet	0	-977.1999373	-3048.373087	-2963.672363	EMD2
GTGGGAATCGTTAGTG-1	singlet	1	-30.27556628	-10.61158216	-19.41644084	EMD2
GTGGGAATCTCTCGAC-1	singlet	1	-421.5138367	-167.0380631	-335.5832263	EMD2
GTGGGAATCTCTGCCA-1	singlet	0	-624.7486412	-1760.210666	-1826.756859	EMD2
GTGGGAATCTTGTGCC-1	singlet	1	-963.6139936	-309.1860917	-715.0292312	EMD2
GTGGTTAAGCGTTACT-1	singlet	2	-275.72401	-256.6838557	-53.82062124	EMD2
GTGGTTAAGTCTAACC-1	singlet	1	-1064.458659	-425.9193481	-1009.860945	EMD2
GTGGTTACAACACGTT-1	singlet	0	-500.5621084	-1568.77346	-1324.044646	EMD2
GTGGTTACAACGTATC-1	singlet	0	-413.7719272	-1269.541578	-1150.771275	EMD2
GTGGTTACACAGTATC-1	singlet	2	-645.757252	-562.2236383	-250.8194191	EMD2
GTGGTTACACTTCTCG-1	singlet	1	-400.090894	-149.7839831	-347.2677436	EMD2
GTGGTTACATGACCCG-1	doublet	0/1	-1185.055232	-1337.288286	-1554.943696	EMD2
GTGGTTAGTAACACCT-1	singlet	1	-201.0030763	-83.68310112	-192.2391056	EMD2

GTGGTTAGTGCAATGG-1	unassigned	1	-2.207063122	-0.888462369	-2.052603235	EMD2
GTGGTTATCAGACCTA-1	singlet	1	-324.2298877	-173.5173541	-296.8814862	EMD2
GTGGTTATCCGAAATC-1	singlet	0	-505.8269061	-1495.955731	-1332.773343	EMD2
GTGTAACAGAGGCTGT-1	singlet	2	-346.8035256	-373.5031425	-146.3222286	EMD2
GTGTAACAGCGAATGC-1	unassigned	2/0	-11.58368509	-11.06425445	-6.216356765	EMD2
GTGTAACAGGACTAAT-1	singlet	0	-795.8463865	-2627.249914	-2626.388242	EMD2
GTGTAACCAAGCGAAC-1	singlet	0	-268.0403106	-726.7658498	-706.8762183	EMD2
GTGTAACGTACACTCA-1	singlet	0	-764.3925053	-2179.316476	-2260.197085	EMD2
GTGTAACGTCCAGCCA-1	singlet	1	-3642.785352	-1053.480335	-2623.421854	EMD2
GTGTAACGTCGGTAAG-1	singlet	1	-239.6247721	-113.6656757	-241.1985742	EMD2
GTGTAACGTGGCAACA-1	singlet	1	-103.96348	-34.66021355	-79.19292035	EMD2
GTGTAACGTGTTATCG-1	singlet	1	-2290.665754	-643.8366329	-1704.982585	EMD2
GTGTAACTCGCCTAGG-1	singlet	1	-1690.902762	-595.9956192	-1282.800119	EMD2
GTGTAACTCTCCATAT-1	singlet	1	-3627.269543	-1157.750931	-2774.790657	EMD2
GTGTAACTCTTACGTT-1	singlet	1	-282.5400997	-110.0460294	-242.3018994	EMD2
GTGTCCTAGCTGGCTC-1	singlet	2	-3360.407845	-2365.848479	-988.7752432	EMD2
GTGTCCTCAACTCGTA-1	singlet	1	-1722.729854	-523.8971875	-1463.254074	EMD2
GTGTCCTCACATCCCT-1	singlet	2	-474.8061642	-555.190105	-163.8563483	EMD2
GTGTCCTGTACAAAGT-1	singlet	1	-214.4974726	-93.53855032	-198.2144129	EMD2
GTGTCCTGTCTAATCG-1	singlet	0	-207.5706527	-615.4719532	-477.4313586	EMD2
GTGTCCTGTCTCGGAC-1	singlet	0	-591.9894606	-2011.624938	-1831.039626	EMD2
GTGTCCTGTGACTCGC-1	doublet	45293	-2766.126697	-1352.794376	-1596.935573	EMD2
GTGTCCTGTGGTCCGT-1	unassigned	2	-1.422536945	-1.490637414	-0.873255344	EMD2
GTGTCCTTCCTTATAC-1	singlet	0	-111.2875714	-304.645185	-257.1292072	EMD2
GTGTCCTTCGGTATGT-1	singlet	0	-191.6236208	-566.3278913	-623.3680383	EMD2
GTGTCCTTCGTGACTA-1	singlet	2	-1427.391446	-1216.813691	-476.1026911	EMD2
GTGTGATAGACCCTTA-1	singlet	2	-1086.954265	-1247.557692	-324.0512178	EMD2
GTGTGATAGATGCTAA-1	singlet	1	-177.5575983	-51.15324299	-145.6809805	EMD2
GTGTGATAGTAAACAC-1	singlet	1	-1872.930292	-670.9905316	-1564.280146	EMD2
GTGTGATCATAAGCAA-1	singlet	2	-4275.33575	-3361.675403	-1247.312743	EMD2
GTGTGATCATAATCGC-1	singlet	1	-309.9238133	-120.4143772	-317.6900508	EMD2
GTGTGATCATAGATCC-1	singlet	1	-2003.099518	-634.1111142	-1455.331843	EMD2
GTGTGATGTACTCGCG-1	doublet	45293	-5247.83275	-2007.084618	-3455.849421	EMD2
GTGTGATGTAGTGTGG-1	singlet	1	-1511.443869	-420.0343564	-1076.212997	EMD2
GTGTGATTCACCGACG-1	singlet	0	-1128.662946	-4061.722033	-3510.0384	EMD2
GTGTGATTCAGCAATC-1	singlet	1	-292.9754124	-122.1057118	-276.7886435	EMD2
GTGTGGCAGCCTCTTC-1	singlet	0	-1035.358561	-2900.773373	-2879.225143	EMD2
GTGTGGCAGCTGGCTC-1	singlet	1	-328.3133574	-142.4422564	-272.2716908	EMD2
GTGTGGCAGTCAGGGT-1	singlet	2	-509.4678545	-491.7284378	-164.7451183	EMD2
GTGTGGCCAAAGACTA-1	singlet	0	-615.2227266	-1976.575153	-1847.881824	EMD2
GTGTGGCCAAAGTGTA-1	singlet	1	-252.151903	-98.32713601	-203.2122187	EMD2
GTGTGGCCAAGATTGA-1	singlet	2	-1560.711719	-1350.503656	-407.9661242	EMD2
GTGTGGCGTACATTGC-1	singlet	0	-1073.69594	-3560.92989	-3131.762131	EMD2
GTGTGGCGTACGAAAT-1	singlet	1	-550.864563	-192.882578	-433.6722227	EMD2
GTGTGGCGTACGGTTT-1	singlet	1	-188.4412399	-72.15014219	-149.6437731	EMD2
GTGTGGCGTCAGGAGT-1	singlet	1	-519.2286722	-225.0161577	-430.0063445	EMD2
GTGTGGCGTCGGAAAC-1	singlet	1	-335.392787	-110.9749271	-256.5738568	EMD2
GTGTGGCGTTGCTCCT-1	singlet	2	-602.5764911	-612.5404771	-263.2364788	EMD2
GTGTGGCGTTGGGTTT-1	singlet	2	-2783.536934	-2418.728094	-926.3361987	EMD2
GTGTGGCTCAATCCAG-1	singlet	1	-296.1491528	-130.64005	-312.1984103	EMD2
GTGTGGCTCCACTGAA-1	singlet	2	-540.4763864	-537.7650405	-220.1711868	EMD2
GTGTTAGAGAGATTCA-1	singlet	1	-635.5756082	-186.3272045	-503.5551896	EMD2
GTGTTAGAGCTTCGTA-1	singlet	1	-413.587152	-162.7252104	-358.7540584	EMD2
GTGTTAGAGTGCTCAT-1	singlet	1	-1876.11693	-577.5039972	-1531.087627	EMD2
GTGTTAGGTATCATGC-1	singlet	2	-1108.734793	-1132.592027	-417.1590281	EMD2
GTGTTAGGTCGCGTCA-1	singlet	2	-1558.962416	-1554.410415	-511.3140032	EMD2
GTGTTAGGTCTGCAAT-1	singlet	1	-281.3314453	-105.6419759	-231.5802089	EMD2
GTGTTAGGTTTCACAG-1	singlet	0	-328.5983099	-1110.264113	-972.3355619	EMD2
GTGTTAGTCGCCAACG-1	singlet	1	-58.6633801	-22.00532937	-52.9022929	EMD2
GTGTTCCAGACAAGCC-1	singlet	0	-311.2157072	-1143.649064	-953.8662617	EMD2
GTGTTCCAGACGCATG-1	singlet	1	-2259.36761	-701.232623	-1711.107536	EMD2
GTGTTCCAGCGTGTCC-1	singlet	1	-132.9588285	-30.72587082	-93.65942756	EMD2
GTGTTCCAGTAGGTTA-1	singlet	1	-495.2118679	-198.2340605	-443.3860513	EMD2
GTGTTCCCAAACTAGA-1	singlet	1	-296.7449762	-123.7657062	-247.5502222	EMD2
GTGTTCCGTATGAGCG-1	singlet	0	-289.1583803	-888.9510754	-731.8976306	EMD2
GTGTTCCTCTTACGTT-1	singlet	2	-930.5575179	-749.2592378	-309.8710993	EMD2
GTTACAGAGCAACAGC-1	singlet	1	-2485.765805	-797.0202141	-2116.549961	EMD2
GTTACAGCAAGTGTCT-1	singlet	1	-2338.206868	-803.6337763	-1921.207717	EMD2
GTTACAGCAGCGGTCT-1	singlet	1	-1938.693188	-661.6552295	-1552.488977	EMD2
GTTACAGCATTGAGCT-1	singlet	0	-1132.351763	-4088.9212	-3966.54078	EMD2
GTTACAGGTGTATTGC-1	singlet	1	-651.7842657	-257.235259	-696.013367	EMD2
GTTACAGGTTCTTCAT-1	singlet	0	-1407.340183	-4681.198338	-4165.58612	EMD2
GTTACAGTCCGAACGC-1	singlet	0	-154.2705474	-284.6378373	-297.4312292	EMD2
GTTACAGTCCTTCTGG-1	singlet	1	-692.6907382	-275.8869743	-526.199578	EMD2
GTTACAGTCGAACGGA-1	singlet	1	-3149.069321	-873.5834833	-2187.85248	EMD2
GTTACAGTCTGTGTGA-1	singlet	1	-2609.785742	-1004.978066	-2214.609148	EMD2
GTTACCCAGAGAGGTA-1	singlet	1	-456.3210751	-185.5592364	-354.8672984	EMD2
GTTACCCAGGCATGGT-1	singlet	1	-1669.344602	-648.4822818	-1464.854278	EMD2
GTTACCCAGGCTTTCA-1	singlet	1	-3114.583544	-993.2542225	-2630.000752	EMD2
GTTACCCAGGTTGTTC-1	singlet	0	-1340.081408	-4215.823705	-3967.966866	EMD2
GTTACCCAGTATTGCC-1	singlet	1	-1498.637094	-549.6475872	-1295.434433	EMD2
GTTACCCAGTCGTCTA-1	singlet	1	-1178.799495	-387.7772191	-997.0606825	EMD2
GTTACCCCAATTTCCT-1	doublet	0/1	-9.087637264	-10.6945541	-12.02079806	EMD2
GTTACCCCACGGGTAA-1	doublet	45293	-1128.385933	-603.2901941	-886.7302109	EMD2
GTTACCCCAGCGACAA-1	singlet	2	-9368.907591	-7239.80491	-2068.555484	EMD2
GTTACCCCAGCTCTGG-1	singlet	1	-337.3904228	-116.1479571	-298.1384575	EMD2
GTTACCCCAGTGGTGA-1	singlet	1	-434.9016124	-143.8734344	-289.4209212	EMD2
GTTACCCGTCACTAGT-1	singlet	2	-4155.566943	-3364.993725	-1120.401958	EMD2
GTTACCCGTCCTGTCT-1	singlet	0	-1811.5808	-7102.767709	-6596.195405	EMD2
GTTACCCGTCTTAGTG-1	singlet	1	-268.3964333	-121.1745171	-237.3821396	EMD2
GTTACCCGTGTTCAGT-1	singlet	2	-2438.991974	-1990.697513	-678.162702	EMD2
GTTACCCTCCACGTAA-1	singlet	0	-3065.532436	-10715.63285	-10134.41453	EMD2
GTTACCCTCGTACACA-1	singlet	1	-3402.463052	-1287.344299	-2731.870297	EMD2
GTTACGAAGCCGTCGT-1	unassigned	1	-9.323640833	-3.506175357	-6.563470566	EMD2
GTTACGAAGGCACGAT-1	singlet	0	-833.5117782	-2466.509074	-2295.238561	EMD2
GTTACGAAGGGAGGAC-1	singlet	0	-275.1714257	-792.4744154	-764.193709	EMD2
GTTACGACACAATGAA-1	singlet	1	-921.7499219	-360.9579032	-821.9369288	EMD2
GTTACGATCAACTACG-1	singlet	1	-357.3050727	-164.2612996	-333.3404692	EMD2
GTTACGATCCTCTTTC-1	singlet	1	-345.8077919	-141.8398012	-257.6587636	EMD2
GTTAGACAGGTAAGTT-1	singlet	1	-1808.696876	-612.7263481	-1457.635917	EMD2
GTTAGACCAACAGCCC-1	singlet	2	-1836.120005	-1382.262647	-608.6975481	EMD2
GTTAGACGTATTAAGG-1	singlet	0	-355.5769632	-1090.611295	-1035.479086	EMD2
GTTAGACGTCATGGCC-1	singlet	1	-1972.467435	-544.9617014	-1342.252746	EMD2
GTTAGACGTTAGAAAC-1	singlet	1	-3057.826342	-925.8999411	-2311.097795	EMD2
GTTAGACGTTGGACCC-1	singlet	0	-742.4575756	-4328.283871	-3441.263269	EMD2
GTTAGACTCCAACCGG-1	singlet	0	-24.00336135	-78.81366429	-76.93911761	EMD2
GTTAGACTCGATGCAT-1	singlet	2	-2945.084731	-3065.391553	-1076.618633	EMD2
GTTAGACTCGCCGTGA-1	singlet	1	-1988.052922	-542.8209781	-1781.343196	EMD2
GTTAGACTCGCGGACT-1	singlet	2	-2209.017735	-1797.687624	-630.1672201	EMD2
GTTAGTGAGAAGTGTT-1	singlet	0	-734.904894	-2484.094947	-2070.573789	EMD2

GTTAGTGAGACAGCGT-1	singlet	2	-1174.805132	-967.6876621	-424.1953758	EMD2
GTTAGTGCAACAACAA-1	singlet	2	-3596.123671	-2899.709722	-1194.761433	EMD2
GTTAGTGCATAGCACT-1	singlet	0	-267.7964892	-849.2949617	-898.9792374	EMD2
GTTAGTGCATCATTTC-1	singlet	2	-461.3064184	-417.5783074	-209.7104466	EMD2
GTTAGTGGTGCGTCGT-1	singlet	0	-169.2160172	-513.4828483	-531.7004525	EMD2
GTTAGTGGTGTAGCAG-1	singlet	1	-4492.558949	-1475.45081	-3370.464369	EMD2
GTTAGTGGTTGAGTCT-1	singlet	1	-109.9322831	-37.51455967	-74.72491932	EMD2
GTTAGTGGTTGCAACT-1	singlet	2	-915.8284945	-941.4174986	-378.1340652	EMD2
GTTAGTGTCCTGGGTG-1	singlet	1	-278.0769298	-128.6134365	-282.6425755	EMD2
GTTAGTGTCTTTCAGT-1	singlet	0	-603.144526	-2296.609877	-1865.985796	EMD2
GTTATGGAGAGTTCGG-1	unassigned	0	-1.659654655	-5.948008848	-1.854229479	EMD2
GTTATGGAGTTAGTAG-1	singlet	1	-232.3030326	-75.8239302	-162.8411503	EMD2
GTTATGGAGTTGTAGA-1	singlet	1	-1959.429888	-633.9883332	-1623.86067	EMD2
GTTATGGCACTGCGTG-1	singlet	1	-368.972376	-167.1085869	-338.8218338	EMD2
GTTATGGCACTTCAGA-1	singlet	1	-725.7754175	-229.4337831	-546.077048	EMD2
GTTATGGCAGCTGAAG-1	doublet	1/0	-168.0166901	-95.14039608	-191.50846	EMD2
GTTATGGGTACCGGCT-1	singlet	1	-765.7966784	-282.3660236	-588.2147183	EMD2
GTTATGGGTCCCGCAA-1	singlet	0	-125.6104936	-351.8148641	-254.0108637	EMD2
GTTATGGGTTCATCGA-1	singlet	2	-1445.738217	-1402.533129	-570.2696289	EMD2
GTTATGGTCGTAGTGT-1	singlet	1	-281.8677694	-119.7750763	-259.8758002	EMD2
GTTATGGTCTCGTCAC-1	singlet	1	-2851.968457	-1006.549684	-2468.425321	EMD2
GTTCATTAGAACTTCC-1	singlet	1	-499.8061847	-234.0881646	-490.9689187	EMD2
GTTCATTAGCTCGAAG-1	singlet	1	-270.6925142	-106.5145719	-236.6952965	EMD2
GTTCATTAGGAAACGA-1	singlet	1	-872.0109654	-288.8429581	-796.8982513	EMD2
GTTCATTAGGAGTACC-1	singlet	1	-552.4878124	-222.2346244	-439.3471457	EMD2
GTTCATTAGGTCTTTG-1	singlet	1	-1896.762576	-569.2802273	-1580.935107	EMD2
GTTCATTGTAACTAAG-1	singlet	1	-1176.268416	-452.4015382	-892.8450939	EMD2
GTTCATTGTCATAACC-1	singlet	1	-276.4434227	-118.1434081	-289.7414832	EMD2
GTTCATTGTCATGCAT-1	singlet	1	-1067.012998	-365.3973248	-870.607246	EMD2
GTTCATTGTCATTCCC-1	singlet	0	-3338.36514	-9746.632653	-9824.180447	EMD2
GTTCATTGTGCCGTTG-1	singlet	2	-2200.216245	-1848.361222	-753.0298868	EMD2
GTTCATTGTTCTCAGA-1	unassigned	0	-2.169500469	-3.548396363	-2.867221423	EMD2
GTTCATTGTTTCCCAC-1	singlet	0	-1142.124571	-2191.234875	-2239.223815	EMD2
GTTCATTTCGAGAAAT-1	singlet	1	-1241.902954	-405.8920947	-1018.043223	EMD2
GTTCATTTCGGTAGAG-1	singlet	1	-1289.172636	-431.1145662	-953.4597427	EMD2
GTTCCGTAGGAAAGTG-1	singlet	1	-1988.225743	-613.8079046	-1529.41442	EMD2
GTTCCGTAGTTTCGAC-1	singlet	0	-346.0146088	-1262.908426	-1080.826897	EMD2
GTTCCGTCACTCAGAT-1	singlet	1	-568.4775007	-173.9124386	-392.3975403	EMD2
GTTCCGTCAGAGGACT-1	singlet	1	-2309.49191	-774.107617	-1923.55487	EMD2
GTTCCGTGTCCGAAGA-1	singlet	1	-674.0143218	-228.5778194	-613.8943993	EMD2
GTTCCGTGTCGTCGGT-1	singlet	1	-2730.067271	-761.4487475	-2083.009281	EMD2
GTTCCGTGTGGCTCTG-1	singlet	0	-440.9364102	-1188.039809	-1124.977717	EMD2
GTTCCGTGTGTAACGG-1	unassigned	1/0	-27.05743725	-16.01724865	-48.19747284	EMD2
GTTCCGTGTTACACAC-1	singlet	2	-5095.454067	-4282.628397	-1198.095847	EMD2
GTTCCGTTCCGGTAGC-1	singlet	2	-2419.012714	-2042.692809	-877.0717479	EMD2
GTTCCGTTCTTCCCGA-1	singlet	1	-1024.777597	-327.4244179	-730.9592246	EMD2
GTTCGCTAGAGTCTTC-1	singlet	0	-355.0013772	-1467.244204	-1185.233296	EMD2
GTTCGCTAGCTAAATG-1	singlet	1	-246.7497357	-95.71866811	-253.9867613	EMD2
GTTCGCTCACAGTGTT-1	singlet	0	-692.5853538	-2190.387788	-1861.388894	EMD2
GTTCGCTCACGGTGAA-1	singlet	1	-2314.725787	-789.6601541	-1906.310832	EMD2
GTTCGCTCAGATACTC-1	singlet	0	-130.3206361	-378.920197	-416.1474207	EMD2
GTTCGCTCAGCCGTTG-1	singlet	1	-220.0161749	-83.8759086	-181.3769439	EMD2
GTTCGCTCATCGTCCT-1	singlet	1	-2084.936329	-658.5772756	-1664.847974	EMD2
GTTCGCTGTAAGCAAT-1	singlet	1	-986.4415205	-365.9152978	-920.0288966	EMD2
GTTCGCTGTGAACGGT-1	singlet	1	-579.3558143	-242.3992749	-510.8459397	EMD2
GTTCGCTGTTACAGCT-1	singlet	1	-6744.601893	-2069.754329	-5368.522729	EMD2
GTTCGCTTCCTCTCGA-1	singlet	1	-513.4670864	-178.8553971	-376.960685	EMD2
GTTCGCTTCTGTTGGA-1	singlet	0	-55.20155469	-131.1491985	-152.6871812	EMD2
GTTCTATAGCTACAAA-1	singlet	1	-1894.202672	-618.7950511	-1659.767375	EMD2
GTTCTATAGCTGCCAC-1	singlet	1	-5590.84546	-1545.626467	-4220.389498	EMD2
GTTCTATCAAGCCATT-1	singlet	1	-489.9237859	-224.5067816	-408.1548209	EMD2
GTTCTATCAATTCACG-1	singlet	2	-83.80662373	-71.25507547	-30.52716723	EMD2
GTTCTATCACGGATCC-1	singlet	1	-844.6318512	-278.7123324	-705.9721115	EMD2
GTTCTATCACGGCTAC-1	singlet	0	-1522.512686	-3753.090767	-3519.766402	EMD2
GTTCTATCAGCGTACC-1	singlet	1	-885.5433549	-362.0507345	-818.6415881	EMD2
GTTCTATGTTTGACAC-1	singlet	1	-436.0750583	-189.8404833	-379.146331	EMD2
GTTCTATTCCCATACC-1	singlet	1	-1482.133091	-503.6420753	-1276.643029	EMD2
GTTCTATTCGAAACAA-1	singlet	2	-2295.703024	-2017.998247	-691.842639	EMD2
GTTCTATTCGAACGCC-1	singlet	1	-153.5769555	-43.82532396	-133.9627318	EMD2
GTTGAACAGAATCGCG-1	singlet	0	-155.9134884	-608.295241	-520.6307446	EMD2
GTTGAACAGTTAACAG-1	singlet	1	-128.970682	-63.2672775	-118.8883858	EMD2
GTTGAACAGTTGGCTT-1	singlet	0	-419.4422423	-1140.381053	-1132.928139	EMD2
GTTGAACCAAGTGGTG-1	singlet	1	-684.6342731	-227.0040004	-572.136737	EMD2
GTTGAACCACCAAAGG-1	doublet	0/2	-1242.512637	-2266.266701	-1871.932477	EMD2
GTTGAACGTCTGCGCA-1	singlet	2	-922.6217246	-832.0213961	-326.0326646	EMD2
GTTGAACTCATCACAG-1	singlet	1	-1837.920501	-622.129396	-1467.791021	EMD2
GTTGAACTCCTTCACG-1	singlet	0	-1215.681294	-4319.911985	-4185.083006	EMD2
GTTGCGGAGCCATTGT-1	singlet	0	-515.0194476	-1695.912275	-1570.945804	EMD2
GTTGCGGAGGTTTGAA-1	singlet	1	-883.4175529	-335.3860665	-721.9980305	EMD2
GTTGCGGAGTCGGCCT-1	singlet	1	-145.050217	-39.87912524	-85.68999359	EMD2
GTTGCGGAGTTGTAAG-1	singlet	1	-390.8685046	-110.2544619	-315.1078318	EMD2
GTTGCGGCAACATACC-1	singlet	1	-1251.933847	-395.9565207	-1023.832743	EMD2
GTTGCGGCAGAACCGA-1	singlet	1	-745.7104001	-300.2645172	-647.1029047	EMD2
GTTGCGGCAGGTGTTT-1	singlet	2	-996.4317551	-774.4331597	-350.6614221	EMD2
GTTGCGGCATTGCAAC-1	singlet	1	-312.0048189	-142.5940851	-237.394101	EMD2
GTTGCGGGTAATTAGG-1	singlet	2	-829.8151469	-729.6347271	-282.0145376	EMD2
GTTGCGGGTACGTACT-1	singlet	1	-4393.363598	-1268.12921	-3501.958424	EMD2
GTTGCGGGTAGGACTG-1	singlet	2	-393.0918108	-387.593901	-150.3915593	EMD2
GTTGCGGGTGCACATT-1	singlet	0	-61.2731573	-184.9717418	-141.4891566	EMD2
GTTGCGGGTTGTGGCC-1	singlet	1	-1938.677852	-685.946571	-1531.397476	EMD2
GTTGCGGTCCTCGATC-1	singlet	0	-109.1099854	-327.3175419	-245.4751876	EMD2
GTTGCGGTCGAGAGAC-1	singlet	1	-3103.022931	-875.6807479	-2280.434969	EMD2
GTTGCGGTCTCTAAGG-1	singlet	1	-1430.07693	-404.9866189	-1227.197282	EMD2
GTTGCTCAGACAGCTG-1	singlet	0	-489.6217423	-1659.519068	-1382.675224	EMD2
GTTGCTCAGACCTCAT-1	doublet	45293	-593.9941335	-269.6203993	-429.2140039	EMD2
GTTGCTCAGAGAGAAC-1	singlet	1	-1677.956757	-519.9262918	-1251.03367	EMD2
GTTGCTCAGATGTTCC-1	singlet	1	-947.8525557	-353.6823972	-724.0066028	EMD2
GTTGCTCAGTTCATGC-1	doublet	2/0	-2970.102831	-4469.337774	-2336.628149	EMD2
GTTGCTCCAATCCTAG-1	singlet	1	-800.3534049	-305.5560868	-648.4184703	EMD2
GTTGCTCCACCCTATC-1	singlet	2	-715.2576179	-599.6038783	-251.566815	EMD2
GTTGCTCGTCCTGTTC-1	singlet	2	-686.4710508	-545.1869995	-244.5630954	EMD2
GTTGCTCGTTAGGACG-1	singlet	1	-1591.752728	-507.5791708	-1378.286013	EMD2
GTTGCTCTCCCTCTTT-1	singlet	2	-1906.392901	-1690.286663	-595.3219228	EMD2
GTTGCTCTCCGTCAAA-1	singlet	2	-1521.081285	-1400.969548	-517.3327631	EMD2
GTTGCTCTCCTAAGTG-1	singlet	1	-1140.001791	-385.2780098	-930.2156353	EMD2
GTTGTAGCAAAGAACT-1	singlet	0	-126.7339341	-412.5190591	-335.5732918	EMD2
GTTGTAGCAATTCGTG-1	singlet	2	-565.3661555	-503.3683323	-221.6833831	EMD2
GTTGTAGCATAAGCGG-1	singlet	1	-1800.527065	-579.4333341	-1554.564956	EMD2
GTTGTAGGTCCAAATC-1	singlet	0	-1171.625841	-4601.069689	-5268.038686	EMD2
GTTGTAGGTCCAGAAG-1	singlet	0	-206.552996	-822.5189112	-811.7423521	EMD2

GTTGTAGGTGAGATTA-1	singlet	1	-131.4524982	-48.00357293	-109.8760066	EMD2
GTTGTAGGTGGCCCAT-1	singlet	1	-625.3312241	-201.5002898	-435.7117357	EMD2
GTTGTAGGTTAGCGGA-1	singlet	1	-685.956213	-291.4932988	-515.1670277	EMD2
GTTGTAGGTTGTGGAG-1	singlet	1	-1484.788079	-500.8610337	-1155.455858	EMD2
GTTGTAGGTTTGTTGG-1	singlet	1	-2165.710274	-746.2850467	-2128.454238	EMD2
GTTGTAGTCCTACAAG-1	unassigned	45293	-79.38733168	-30.56118646	-51.7558078	EMD2
GTTGTCCAGGTACAAT-1	singlet	0	-264.2037156	-948.8087128	-749.7283587	EMD2
GTTGTCCCACTGAATC-1	singlet	1	-2200.002955	-724.5662798	-1837.243622	EMD2
GTTGTCCGTAAGGCTG-1	singlet	2	-75.22652259	-89.41531943	-38.88098857	EMD2
GTTGTCCGTCAGTTTG-1	singlet	2	-1517.564288	-1348.361503	-565.9313332	EMD2
GTTGTCCGTCCGAAAG-1	singlet	1	-485.1097068	-208.6001717	-428.1636496	EMD2
GTTGTCCGTCTCCCTA-1	singlet	0	-116.1019814	-239.3330195	-205.5001472	EMD2
GTTGTCCTCACTACGA-1	singlet	1	-1456.257495	-438.3995894	-1300.014103	EMD2
GTTGTCCTCAGAGCGA-1	singlet	2	-756.7350786	-685.2881389	-221.2841703	EMD2
GTTGTCCTCATTGTTC-1	singlet	1	-479.9511843	-188.5971701	-355.4149677	EMD2
GTTGTCCTCCGTTGAA-1	doublet	1/0	-1285.503085	-932.8411796	-1386.251422	EMD2
GTTGTCCTCCTCACGT-1	singlet	1	-328.641519	-117.6319073	-279.525882	EMD2
GTTGTCCTCTGCGAGC-1	doublet	0/1	-299.4434364	-453.0635254	-528.5098488	EMD2
GTTGTCCTCTGTTCAT-1	singlet	1	-181.5619312	-79.56386316	-163.1204268	EMD2
GTTGTCCTCTTACCGC-1	singlet	0	-1997.348025	-4602.632503	-4888.885396	EMD2
GTTGTGAAGACGCTCC-1	singlet	1	-965.4704027	-321.7178207	-711.6403492	EMD2
GTTGTGAAGAGGGTAA-1	singlet	2	-2029.639324	-1884.539892	-680.6483556	EMD2
GTTGTGAAGTTACGGG-1	singlet	1	-641.339261	-227.2311261	-480.0525756	EMD2
GTTGTGACAATAGTAG-1	singlet	2	-1683.682023	-1453.783981	-444.6603086	EMD2
GTTGTGACACTCAAGT-1	singlet	1	-4017.331865	-1036.359416	-2943.085034	EMD2
GTTGTGAGTCGGAAAC-1	singlet	1	-1988.957573	-659.6157651	-1626.571592	EMD2
GTTGTGAGTTCAGTAC-1	singlet	1	-2213.995208	-644.3335038	-1681.765893	EMD2
GTTGTGAGTTCGTTCC-1	singlet	1	-2794.202718	-910.678695	-2360.410465	EMD2
GTTGTGATCACTGATG-1	singlet	1	-242.066433	-108.7338577	-215.8388845	EMD2
GTTGTGATCATGTCAG-1	doublet	1/0	-1506.773161	-1433.339988	-1739.198916	EMD2
GTTGTGATCCAACCGG-1	singlet	2	-380.3282171	-405.7080317	-178.0883916	EMD2
GTTGTGATCCTACGAA-1	singlet	1	-343.4300528	-144.1094353	-335.2142071	EMD2
GTTGTGATCCTAGCTC-1	singlet	2	-7011.672232	-5549.578602	-2036.778009	EMD2
GTTGTGATCTATCCAT-1	singlet	2	-3488.509636	-2662.317438	-957.0844886	EMD2
GTTGTGATCTGGTCAA-1	singlet	0	-230.8092429	-1129.49088	-789.8243801	EMD2
GTTTACTCACAAAGCG-1	singlet	2	-237.9357992	-309.6150888	-123.8521333	EMD2
GTTTACTCACGGCACT-1	singlet	1	-2216.477644	-724.8298576	-1668.184179	EMD2
GTTTACTCACTCGATA-1	singlet	2	-467.922909	-477.4010728	-195.4617786	EMD2
GTTTACTGTCGAAGCA-1	singlet	1	-2336.383601	-675.8330027	-2062.527989	EMD2
GTTTACTGTCGTGTTA-1	singlet	2	-298.1168103	-255.4907444	-93.43830347	EMD2
GTTTACTGTGATGAAT-1	singlet	1	-751.4637117	-251.7573401	-584.1106442	EMD2
GTTTACTTCGATTTCT-1	singlet	1	-1272.736236	-420.4098812	-946.4869698	EMD2
GTTTACTTCGGAATGG-1	singlet	2	-542.9710415	-428.2381635	-186.0840671	EMD2
GTTTACTTCTACTGAG-1	singlet	1	-390.0830408	-142.7054799	-290.5885505	EMD2
GTTTGGAAGAGTGGCT-1	singlet	0	-563.5082573	-1755.284651	-1783.433397	EMD2
GTTTGGAAGCAGGCAT-1	singlet	2	-896.7490541	-914.7920893	-297.4138975	EMD2
GTTTGGAAGCTGCCAC-1	singlet	1	-251.6596256	-83.12739695	-179.0364372	EMD2
GTTTGGAAGGTGATAT-1	singlet	2	-1802.771637	-1331.682463	-791.6267212	EMD2
GTTTGGAGTAGACAGC-1	singlet	1	-432.914108	-183.277414	-364.2434644	EMD2
GTTTGGAGTCAACGCC-1	singlet	2	-1045.620611	-900.2629963	-311.5322523	EMD2
GTTTGGAGTCGAAACG-1	doublet	45293	-4950.14791	-2271.65204	-3007.800212	EMD2
GTTTGGAGTCTGTCAA-1	singlet	1	-2306.714291	-725.3907047	-1919.789872	EMD2
GTTTGGATCTATTCGT-1	singlet	2	-2410.108099	-1924.113327	-806.8732241	EMD2
GTTTGGATCTTCCAGC-1	singlet	0	-297.3470404	-907.0261205	-836.2196659	EMD2
TAACACGAGCCTATCA-1	singlet	0	-170.9173693	-463.3784911	-528.3793982	EMD2
TAACACGAGCCTCAGC-1	singlet	1	-3898.170596	-1238.919941	-3215.609608	EMD2
TAACACGAGTCCTGCG-1	singlet	2	-1530.958578	-1626.428824	-571.2564317	EMD2
TAACACGCACCGTACG-1	singlet	1	-3854.283832	-1350.384964	-3089.131688	EMD2
TAACACGCACGACAAG-1	singlet	2	-1495.098228	-1366.452586	-513.3102691	EMD2
TAACACGCACTAGTAC-1	singlet	1	-2051.814205	-606.0329132	-1801.690163	EMD2
TAACACGCAGTCGGTC-1	singlet	1	-1081.627178	-364.3458668	-893.0405651	EMD2
TAACACGGTGCATTAC-1	singlet	1	-716.9237762	-301.745226	-636.4607282	EMD2
TAACACGGTGCGGCTT-1	singlet	1	-451.7244453	-198.6361432	-472.8416861	EMD2
TAACACGGTGGGCTTC-1	singlet	1	-1052.547884	-293.6041397	-895.8942457	EMD2
TAACACGGTTGCCATA-1	singlet	1	-635.87908	-219.4798771	-492.4463976	EMD2
TAACACGTCAAGTCTG-1	singlet	1	-395.938341	-172.02496	-409.7535681	EMD2
TAACACGTCACATACG-1	singlet	1	-2222.813159	-612.6266696	-1613.144859	EMD2
TAACACGTCACCGCTT-1	singlet	1	-228.3492237	-52.71488188	-183.8284669	EMD2
TAACACGTCCACTTTA-1	singlet	1	-2285.18598	-802.0419384	-1752.969432	EMD2
TAACACGTCCTTCTAA-1	singlet	1	-2147.032096	-736.6053728	-1787.888966	EMD2
TAACACGTCGAGATGG-1	singlet	0	-1064.440651	-4088.58712	-3824.180119	EMD2
TAACACGTCGTCACCT-1	singlet	1	-388.0626507	-150.211419	-314.7277112	EMD2
TAACACGTCTCTTGCG-1	singlet	1	-2407.074038	-729.2536894	-1956.233022	EMD2
TAACCAGAGACTTAAG-1	singlet	1	-1617.386415	-583.6294621	-1419.577261	EMD2
TAACCAGAGTAAACGT-1	singlet	1	-2951.085149	-906.0940171	-2177.070371	EMD2
TAACCAGCACTGGCCA-1	singlet	1	-1921.239631	-705.8869542	-1668.440005	EMD2
TAACCAGCAGACCTAT-1	singlet	2	-4344.251569	-3449.784517	-1183.006392	EMD2
TAACCAGCAGAGGGTT-1	singlet	0	-858.4298939	-2630.585339	-2515.048542	EMD2
TAACCAGCATATGAAG-1	singlet	0	-104.5652032	-307.47594	-284.0624056	EMD2
TAACCAGCATGACAAA-1	singlet	0	-306.5438602	-637.8775694	-662.4816166	EMD2
TAACCAGGTAGAGTTA-1	singlet	0	-233.573725	-755.9427213	-659.6166469	EMD2
TAACCAGGTAGCTGCC-1	singlet	2	-820.442039	-836.7369912	-286.5266794	EMD2
TAACCAGGTATCGTAC-1	singlet	1	-803.5675479	-332.9988802	-752.7222868	EMD2
TAACCAGGTCCATACA-1	singlet	1	-290.1124783	-128.5277423	-225.544698	EMD2
TAACCAGGTGCCTTTC-1	singlet	1	-4322.295399	-1774.156639	-3342.059544	EMD2
TAACCAGTCACCACAA-1	singlet	0	-1626.681925	-5760.792294	-5094.053088	EMD2
TAACCAGTCCTAGAGT-1	singlet	2	-4261.013388	-3751.174581	-1528.361193	EMD2
TAACCAGTCCTCACCA-1	singlet	1	-1705.663823	-521.0533802	-1482.958225	EMD2
TAACCAGTCGAACCTA-1	singlet	1	-352.3443322	-157.1735569	-280.3414665	EMD2
TAACCAGTCGACATCA-1	singlet	1	-361.7628549	-151.6026603	-337.2981652	EMD2
TAACCAGTCGCGGTAC-1	singlet	0	-698.0710115	-1996.109594	-2007.834784	EMD2
TAACCAGTCGCTACAA-1	singlet	1	-807.666852	-329.5578891	-762.5217152	EMD2
TAACCAGTCTTTCAGT-1	singlet	1	-556.2463273	-198.9234755	-488.2218957	EMD2
TAACGACAGAGTAACT-1	singlet	1	-1977.349334	-585.3404675	-1534.817869	EMD2
TAACGACAGATGTTCC-1	singlet	1	-1851.954158	-669.5353218	-1602.002785	EMD2
TAACGACAGCCTATTG-1	singlet	2	-1134.630283	-1183.747084	-444.8657931	EMD2
TAACGACCAAGGCTTT-1	singlet	2	-1044.148628	-1266.950499	-331.5384449	EMD2
TAACGACCAATACCCA-1	singlet	0	-34.87074065	-93.01746669	-86.96688057	EMD2
TAACGACCAATACGCT-1	singlet	1	-1683.761967	-382.8536351	-1129.422771	EMD2
TAACGACCACTTTATC-1	singlet	0	-446.0695986	-1200.564477	-1149.388096	EMD2
TAACGACCAGATACTC-1	singlet	0	-179.9476246	-644.5700274	-534.6795316	EMD2
TAACGACCATCTTCGC-1	singlet	1	-819.1861676	-254.4868762	-675.4040352	EMD2
TAACGACGTTGCATGT-1	singlet	1	-724.1234178	-298.6425964	-618.76064	EMD2
TAACGACTCTCTCTAA-1	singlet	1	-239.5314411	-111.3460785	-215.5334967	EMD2
TAACTTCAGCACCCAC-1	singlet	1	-639.5892475	-167.9555132	-416.4258075	EMD2
TAACTTCAGCCGTCGT-1	singlet	1	-12.18015789	-6.828391844	-17.03907736	EMD2
TAACTTCAGCTGAGTG-1	singlet	1	-292.6902825	-107.5556545	-234.9167293	EMD2
TAACTTCAGTGGTCAG-1	singlet	1	-2361.934571	-677.0458072	-2101.008641	EMD2
TAACTTCCAAAGTGTA-1	singlet	2	-1801.279079	-1447.559767	-571.5616779	EMD2
TAACTTCCACCAGGTC-1	singlet	1	-622.0512793	-262.1433075	-518.6653353	EMD2

TAACTTCCACTACACA-1	singlet	1	-644.6826471	-262.7314883	-560.1048648	EMD2
TAACTTCCACTACTTT-1	singlet	1	-1159.849343	-417.9105251	-970.3105637	EMD2
TAACTTCCAGGTGTTT-1	singlet	1	-3096.813026	-1234.003927	-2585.453604	EMD2
TAACTTCGTGCTTCAA-1	unassigned	2	-8.0365112	-12.01730614	-3.730978833	EMD2
TAACTTCTCATGCGGC-1	singlet	0	-882.5536754	-1520.814173	-1608.996592	EMD2
TAACTTCTCCTAACAG-1	singlet	2	-3244.209614	-2687.068005	-1035.82299	EMD2
TAACTTCTCGCATGAT-1	singlet	1	-422.4634115	-177.7675345	-336.8443986	EMD2
TAAGCACAGACGATAT-1	singlet	0	-316.3002024	-1062.804806	-916.3517493	EMD2
TAAGCACAGAGTGGCT-1	singlet	0	-867.5618292	-2417.349393	-2416.543462	EMD2
TAAGCACAGCCTAGGA-1	singlet	2	-2100.861112	-1801.790677	-754.0884338	EMD2
TAAGCACAGCGTGTCC-1	singlet	0	-275.1014007	-912.0056478	-848.3109862	EMD2
TAAGCACAGCTCGCAC-1	doublet	45293	-1456.859658	-612.5519109	-909.8349761	EMD2
TAAGCACAGTAAACGT-1	singlet	2	-1251.831828	-1008.839975	-458.9510812	EMD2
TAAGCACAGTCATACC-1	singlet	1	-950.596104	-319.4420016	-760.8119023	EMD2
TAAGCACCAGGATTCT-1	singlet	1	-239.6420454	-81.61479276	-161.7759807	EMD2
TAAGCACGTAGAGCTG-1	singlet	0	-1281.886795	-4096.328077	-4097.778101	EMD2
TAAGCACGTTTACGTG-1	singlet	1	-1793.132913	-515.0882266	-1436.993001	EMD2
TAAGCCAAGCGGTATG-1	singlet	1	-943.2297143	-367.1246062	-807.1196981	EMD2
TAAGCCAAGTAAGACT-1	singlet	0	-232.798464	-656.9178214	-588.8135525	EMD2
TAAGCCAAGTAAGGGA-1	singlet	2	-7637.100078	-6103.664458	-2158.110001	EMD2
TAAGCCAAGTATTGCC-1	singlet	1	-279.1832625	-130.4013573	-247.2725135	EMD2
TAAGCCAGTCCAGCAC-1	singlet	0	-43.00713336	-127.7095234	-157.5320207	EMD2
TAAGCCAGTCTGCATA-1	singlet	1	-3706.755255	-1103.144038	-2775.098655	EMD2
TAAGCCAGTGCAGATG-1	singlet	1	-2590.514226	-766.258282	-2253.130053	EMD2
TAAGCCAGTTGAGGAC-1	singlet	0	-1180.75215	-3487.232826	-3324.567232	EMD2
TAAGCCATCAGGGTAG-1	singlet	1	-3680.736108	-1129.347008	-2843.566816	EMD2
TAAGCCATCCACGAAT-1	doublet	45293	-4281.745691	-2344.566311	-3087.706021	EMD2
TAAGCCATCCTCTAGC-1	singlet	1	-747.3422281	-310.7507506	-593.7031007	EMD2
TAAGCCATCCTCTGCA-1	unassigned	1	-8.876405371	-3.069998087	-6.536802155	EMD2
TAAGCGTAGCCGATTT-1	singlet	0	-79.83974196	-384.4788877	-307.8254453	EMD2
TAAGCGTAGCCTATTG-1	singlet	1	-106.0134277	-35.08224892	-66.21988901	EMD2
TAAGCGTCATTCTCCG-1	singlet	1	-298.3388578	-110.4297997	-245.9810377	EMD2
TAAGCGTGTCCAACGC-1	singlet	0	-125.5167949	-452.0655955	-322.1739274	EMD2
TAAGCGTGTGACAACG-1	singlet	0	-225.6717246	-733.6558774	-697.0453963	EMD2
TAAGCGTGTGCCTACG-1	singlet	1	-323.0063564	-125.1307857	-265.1908376	EMD2
TAAGCGTGTGTGTGTT-1	singlet	1	-2429.469158	-738.0880737	-2021.521661	EMD2
TAAGCGTGTTGTGCCG-1	singlet	0	-202.9078287	-794.1986255	-721.4654546	EMD2
TAAGCGTTCAAGAATG-1	singlet	1	-2438.808671	-816.9941792	-2007.695532	EMD2
TAAGCGTTCACCCTTG-1	singlet	1	-186.2484282	-89.38668805	-180.6038376	EMD2
TAAGCGTTCATACAGC-1	singlet	1	-502.3415147	-219.7824407	-464.6503831	EMD2
TAAGCGTTCCATAGGT-1	singlet	0	-155.1084347	-451.3221664	-369.9885089	EMD2
TAAGCGTTCCTGGGAC-1	singlet	1	-4587.267971	-1495.557712	-3600.545136	EMD2
TAAGCGTTCGTAACAC-1	singlet	2	-1838.736723	-1376.140131	-600.0125055	EMD2
TAAGTCGAGTTGCGCC-1	singlet	0	-102.0786873	-320.5369322	-322.2091779	EMD2
TAAGTCGCAAGCCCAC-1	singlet	1	-4766.14491	-1275.044798	-3567.047056	EMD2
TAAGTCGCAAGCTGCC-1	singlet	0	-340.2565408	-1268.985174	-1187.455407	EMD2
TAAGTCGCACTAGTAC-1	singlet	1	-2464.751952	-789.3741374	-2120.875431	EMD2
TAAGTCGGTCAATGGG-1	singlet	2	-3391.426954	-2534.319108	-877.3810936	EMD2
TAAGTCGGTCGGTAAG-1	singlet	1	-1432.50037	-437.2863768	-1144.118644	EMD2
TAAGTCGTCCCAGTGG-1	singlet	0	-931.4519293	-2793.317248	-2733.32645	EMD2
TAAGTCGTCCTCAGGG-1	singlet	1	-1061.033026	-348.9824277	-814.3699585	EMD2
TAAGTCGTCTAATTCC-1	singlet	1	-646.1887917	-244.862327	-563.3707699	EMD2
TAAGTCGTCTCGCGTT-1	singlet	1	-140.6562379	-39.31645083	-94.54766397	EMD2
TAATCTCAGAATCGAT-1	singlet	0	-488.1716921	-1265.670537	-1173.170591	EMD2
TAATCTCAGACCGCCT-1	unassigned	0/1	-28.33326323	-46.38776256	-56.61145507	EMD2
TAATCTCAGCACTCTA-1	singlet	1	-996.7362157	-289.3361977	-673.5901683	EMD2
TAATCTCAGTCATTGC-1	singlet	0	-292.1313919	-927.8823676	-866.2627961	EMD2
TAATCTCAGTCTGGAG-1	singlet	1	-4584.790615	-1512.589217	-4007.773917	EMD2
TAATCTCAGTGGCAGT-1	singlet	1	-3858.213836	-1021.465901	-2886.041555	EMD2
TAATCTCCACCCTATC-1	singlet	2	-6887.734432	-5893.622218	-2173.67496	EMD2
TAATCTCCAGCAGACA-1	singlet	1	-1973.690082	-708.4564161	-1760.834738	EMD2
TAATCTCCAGTTAGGG-1	singlet	1	-107.2824033	-50.86947216	-91.56368891	EMD2
TAATCTCGTCCTCCAT-1	singlet	1	-3588.960959	-905.5080275	-2877.199933	EMD2
TAATCTCGTGAGGCAT-1	singlet	0	-1252.002264	-3972.45399	-3937.746189	EMD2
TAATCTCGTTATTCCT-1	singlet	0	-1211.765029	-3600.669197	-3425.006998	EMD2
TAATCTCGTTTCGGCG-1	singlet	0	-968.5882803	-3138.770785	-2971.005347	EMD2
TAATCTCTCATTTCGT-1	singlet	1	-292.3137394	-132.9679588	-241.3558078	EMD2
TAATCTCTCGCTAATG-1	doublet	45323	-5293.89398	-3760.825068	-2450.38903	EMD2
TAATCTCTCGTGCTCT-1	singlet	2	-383.1770131	-354.6909464	-68.47283354	EMD2
TAATTCCAGAGGCGTT-1	singlet	1	-4041.047011	-1189.311215	-3283.363911	EMD2
TAATTCCAGCCTTCTC-1	singlet	0	-1593.618222	-4572.158711	-4344.183703	EMD2
TAATTCCCAGAGACTG-1	singlet	0	-773.9604431	-4406.888965	-3498.636476	EMD2
TAATTCCCATCCGAAT-1	singlet	1	-52.45342759	-24.10957693	-39.53072106	EMD2
TAATTCCCATTGCTTT-1	singlet	1	-1342.887412	-475.0392341	-1011.100188	EMD2
TAATTCCGTCTCTCTG-1	singlet	1	-536.221783	-202.5029615	-408.6811378	EMD2
TAATTCCGTGTGTTTG-1	singlet	1	-2326.546144	-726.958047	-1946.729941	EMD2
TAATTCCGTTAGAGTA-1	singlet	0	-475.161914	-2353.868084	-1860.11821	EMD2
TAATTCCTCACCTTGC-1	singlet	1	-2425.266987	-807.6655818	-2028.58048	EMD2
TACAACGAGGAGCTGT-1	singlet	1	-3331.3137	-1184.072272	-2829.776789	EMD2
TACAACGCACCGTGAC-1	singlet	1	-361.9097293	-135.6387838	-312.8668561	EMD2
TACAACGCATGTAACC-1	singlet	1	-2407.261284	-793.3546137	-1888.344965	EMD2
TACAACGGTCCAGAAG-1	singlet	2	-87.33098044	-84.87510446	-26.80132428	EMD2
TACAACGTCAACCTCC-1	singlet	2	-1724.88371	-1749.236488	-626.6898879	EMD2
TACAACGTCATCGCAA-1	singlet	2	-2895.633218	-2427.867292	-853.1270539	EMD2
TACAACGTCCCGAACG-1	singlet	1	-1052.942782	-353.3248499	-874.1418463	EMD2
TACAACGTCCGACATA-1	singlet	1	-3874.770943	-1332.749321	-3139.234643	EMD2
TACAACGTCGAGAACG-1	singlet	1	-1366.620986	-414.5010129	-1112.899056	EMD2
TACAACGTCTCAGGCG-1	singlet	2	-2423.375721	-1946.346821	-612.9532765	EMD2
TACAACGTCTGCGGCA-1	singlet	1	-1033.853044	-346.9285084	-825.2140466	EMD2
TACACCCAGAAACTGT-1	singlet	1	-861.4429848	-338.1422348	-715.4263294	EMD2
TACACCCAGAAATTGC-1	singlet	2	-9233.277231	-7107.989821	-2844.052998	EMD2
TACACCCAGAGGCCAT-1	singlet	1	-242.4855681	-99.16509188	-212.7997738	EMD2
TACACCCAGATACGAT-1	singlet	1	-1465.675696	-536.948192	-1258.895149	EMD2
TACACCCAGCGTCAGA-1	singlet	0	-607.6388326	-1863.390676	-1620.619886	EMD2
TACACCCAGGCCTTGC-1	singlet	2	-2522.665386	-2268.231606	-749.3461894	EMD2
TACACCCCACACCGAC-1	singlet	0	-547.5593066	-1564.906631	-1543.237426	EMD2
TACACCCCACTCAAGT-1	singlet	1	-1503.100175	-540.2828004	-1143.663722	EMD2
TACACCCCACTTGAAC-1	singlet	1	-7658.405245	-2228.463482	-6367.949888	EMD2
TACACCCCAGATCCAT-1	singlet	1	-3250.126592	-1131.428414	-2762.910264	EMD2
TACACCCCAGGGAGAG-1	singlet	1	-2276.961154	-752.0522285	-1928.690277	EMD2
TACACCCCATGTGGTT-1	singlet	2	-2029.469666	-1530.52423	-655.9713178	EMD2
TACAGGTAGACGCTCC-1	singlet	1	-815.7757916	-266.9105725	-566.6228925	EMD2
TACAGGTAGCGCCTCA-1	singlet	0	-788.6276882	-1977.19843	-1989.144215	EMD2
TACAGGTAGGTGATAT-1	singlet	1	-249.318337	-108.8616314	-252.0726934	EMD2
TACAGGTAGTCGAAAT-1	singlet	1	-508.6574125	-200.3870745	-409.9346627	EMD2
TACAGGTCAAGAGTGC-1	singlet	1	-1575.131213	-458.4559311	-1338.830515	EMD2
TACAGGTCAAGTGGGT-1	singlet	0	-777.4588092	-2617.943775	-2235.512412	EMD2
TACAGGTCACATTACG-1	singlet	2	-1693.197767	-1519.035948	-583.654325	EMD2
TACAGGTCATAGACTC-1	singlet	1	-6732.832638	-1857.394168	-4975.503336	EMD2
TACAGGTCATCCTGTC-1	singlet	1	-1995.464434	-671.9883552	-1533.855233	EMD2

TACAGGTGTAGACGGT-1	singlet	1	-2547.534891	-773.4638061	-2120.691789	EMD2
TACAGGTTCACGAGGA-1	singlet	0	-1207.603274	-4460.689404	-4054.473138	EMD2
TACAGGTTCACTACGA-1	singlet	1	-645.3330147	-267.4712865	-591.7420571	EMD2
TACAGGTTCATTGGTG-1	singlet	2	-153.1446351	-140.5405218	-77.34648827	EMD2
TACAGGTTCCACTGAA-1	singlet	2	-8608.92577	-6635.63469	-1875.821388	EMD2
TACAGGTTCGCGTCGA-1	singlet	0	-103.2487079	-283.7187048	-273.1189664	EMD2
TACAGGTTCGTTGTTT-1	singlet	1	-679.107609	-239.8189534	-551.2061976	EMD2
TACAGGTTCTGGAGAG-1	singlet	1	-148.5827632	-71.20646355	-113.3820341	EMD2
TACATTCAGAGCATAT-1	singlet	0	-1064.655112	-3324.297028	-3316.085642	EMD2
TACATTCAGATACAGT-1	singlet	1	-1113.883017	-385.3312584	-942.19374	EMD2
TACATTCAGCCATCCG-1	singlet	1	-3224.998256	-944.5040799	-2777.7709	EMD2
TACATTCAGTACCATC-1	singlet	1	-738.9574729	-281.961703	-658.3396674	EMD2
TACATTCCACAATGAA-1	singlet	1	-453.6655746	-197.9682367	-416.5768335	EMD2
TACATTCCATGGGAAC-1	singlet	1	-2305.272189	-894.2613109	-2110.614781	EMD2
TACATTCCATTCAGGT-1	singlet	0	-2387.106997	-7971.62277	-7680.132652	EMD2
TACATTCGTCGACTTA-1	singlet	1	-706.9256344	-351.737647	-625.0622392	EMD2
TACATTCGTTCTCAGA-1	singlet	1	-3263.69094	-917.6117989	-2682.10479	EMD2
TACCCACAGATAGCAT-1	singlet	1	-385.9396396	-194.1493627	-337.6322396	EMD2
TACCCACAGATCCGAG-1	singlet	1	-1112.102297	-378.847431	-888.1542731	EMD2
TACCCACAGGTACAAT-1	singlet	1	-1965.164407	-610.7312064	-1802.817211	EMD2
TACCCACAGTACAGCG-1	singlet	0	-181.6163665	-551.6094786	-542.4700938	EMD2
TACCCACAGTACCATC-1	singlet	2	-4110.382048	-3447.611333	-1246.368654	EMD2
TACCCACCAATTCTTC-1	singlet	1	-1748.423005	-495.5121356	-1842.111659	EMD2
TACCCACCATCGTCCT-1	singlet	1	-423.2951102	-175.8804321	-376.1583333	EMD2
TACCCACGTGGTATGG-1	singlet	2	-543.7828636	-446.4647141	-188.2730425	EMD2
TACCCACTCACCGGGT-1	singlet	1	-392.3793679	-183.1761429	-332.2439585	EMD2
TACCCACTCACGATAC-1	singlet	1	-327.8529753	-126.2712548	-275.3500697	EMD2
TACCCACTCGCTATTT-1	singlet	0	-25.37150853	-60.56695657	-54.95495366	EMD2
TACCCACTCTCATAGG-1	doublet	0/2	-193.0674679	-292.2219163	-265.9008144	EMD2
TACCCGTAGACTTCCA-1	singlet	0	-32.93007585	-119.109254	-112.7435654	EMD2
TACCCGTAGAGAGGTA-1	singlet	1	-1306.23653	-432.8695214	-1194.870747	EMD2
TACCCGTAGAGTATAC-1	singlet	1	-421.0860767	-156.2681618	-267.1751313	EMD2
TACCCGTAGGGACTGT-1	singlet	1	-2685.675131	-691.7289623	-1794.128975	EMD2
TACCCGTCAAATACAG-1	singlet	1	-1395.81361	-510.3120278	-1196.093187	EMD2
TACCCGTCACACCTAA-1	singlet	1	-2051.397172	-681.0411045	-1560.702849	EMD2
TACCCGTCAGCGAACA-1	singlet	1	-987.3000852	-341.0710204	-796.6111878	EMD2
TACCCGTGTGGGCTTC-1	singlet	0	-1060.239814	-3617.926313	-3840.226204	EMD2
TACCCGTGTTAAACAG-1	doublet	45293	-747.4637415	-369.2186502	-546.7276495	EMD2
TACCCGTTCGGCTGTG-1	singlet	1	-2203.400735	-714.2327036	-1717.987431	EMD2
TACCGAAAGGATCACG-1	singlet	2	-480.6160041	-440.4574123	-209.9066706	EMD2
TACCGAAAGTTAACGA-1	singlet	1	-7574.241947	-2425.979859	-5962.062245	EMD2
TACCGAACACACAGAG-1	doublet	1/0	-379.8779876	-318.9515225	-471.7121357	EMD2
TACCGAACAGACCTGC-1	singlet	0	-377.5356168	-1048.522384	-996.1191835	EMD2
TACCGAAGTAACACCT-1	singlet	1	-424.6115334	-179.7647119	-404.3098759	EMD2
TACCGAAGTAGGCAAC-1	singlet	1	-499.4239834	-206.2166231	-432.9592703	EMD2
TACCGAATCATGCTAG-1	singlet	1	-2294.663597	-602.7416	-1838.927368	EMD2
TACCGAATCCAGTACA-1	singlet	1	-2453.348154	-734.8012845	-1804.568568	EMD2
TACCGGGAGACGTCGA-1	singlet	1	-232.1915654	-73.25165399	-161.3273617	EMD2
TACCGGGCACACGTGC-1	singlet	0	-586.6729634	-1769.635824	-1775.590062	EMD2
TACCGGGCATCCTTCG-1	singlet	2	-5904.851718	-4942.622107	-2122.59747	EMD2
TACCTCGAGAAGCTGC-1	singlet	1	-613.7278553	-281.5971449	-504.1422784	EMD2
TACCTCGAGGCATCTT-1	singlet	1	-678.062318	-230.8229522	-544.1924188	EMD2
TACCTCGAGTTGTCGT-1	singlet	1	-1671.184879	-523.7422512	-1237.721005	EMD2
TACCTCGCAACAGCCC-1	singlet	0	-550.7182911	-1522.079535	-1430.003439	EMD2
TACCTCGCACACCTAA-1	singlet	2	-134.3224782	-81.24523787	-39.97128169	EMD2
TACCTCGCACCCTAAA-1	singlet	0	-62.14075992	-145.7173207	-164.8724512	EMD2
TACCTCGGTTCTCTAT-1	singlet	1	-374.1230171	-171.4561312	-337.9792947	EMD2
TACCTCGTCCTCTTTC-1	singlet	1	-928.8759612	-346.5625735	-922.0400819	EMD2
TACCTGCAGAAACCAT-1	singlet	0	-695.3969682	-2049.025476	-1895.925903	EMD2
TACCTGCAGACATCAA-1	singlet	1	-304.7613005	-117.4116309	-259.2797858	EMD2
TACCTGCAGAGCACTG-1	singlet	0	-88.16097151	-350.3639752	-315.6474861	EMD2
TACCTGCAGGTAACTA-1	singlet	0	-658.3255873	-1789.354698	-1780.800998	EMD2
TACCTGCAGTAGAGTT-1	doublet	45293	-113.7173089	-57.15724728	-93.03843609	EMD2
TACCTGCCAAACCACT-1	doublet	0/2	-1482.489625	-2840.26981	-2361.461638	EMD2
TACCTGCCAGACTCTA-1	singlet	1	-575.4777085	-220.7449403	-514.6746011	EMD2
TACCTGCCATTCAGGT-1	singlet	0	-201.9110628	-609.7687126	-565.5474708	EMD2
TACCTGCGTAAGTTGA-1	singlet	1	-774.7881484	-235.4433216	-520.5007328	EMD2
TACCTGCGTGAGCAGT-1	singlet	1	-956.6964416	-295.9262449	-687.5197823	EMD2
TACCTGCTCCCAGGCA-1	singlet	1	-313.7538267	-122.3240042	-259.0411231	EMD2
TACCTGCTCCTTATCA-1	singlet	0	-2173.006736	-7534.670254	-6842.703929	EMD2
TACCTGCTCCTTGGAA-1	singlet	2	-1298.938086	-1127.535721	-510.3898223	EMD2
TACGCTCAGAAGGTAG-1	doublet	2/0	-1548.248605	-1821.135775	-1395.248032	EMD2
TACGCTCAGATCGACG-1	singlet	0	-287.9775836	-852.8321643	-922.6897427	EMD2
TACGCTCAGCCATCCG-1	singlet	1	-1138.527714	-382.0399444	-940.6360527	EMD2
TACGCTCAGCCATGCC-1	singlet	0	-216.0134737	-816.0841472	-698.6728012	EMD2
TACGCTCAGGATTTGA-1	singlet	2	-4329.61233	-4156.721517	-1460.213064	EMD2
TACGCTCAGTCAGGGT-1	singlet	1	-933.0987619	-359.8592767	-713.3799891	EMD2
TACGCTCCACAACGCC-1	singlet	1	-417.7552553	-146.6074957	-323.817647	EMD2
TACGCTCCACAAGCCC-1	singlet	1	-3658.629884	-1032.640975	-2747.177491	EMD2
TACGCTCCAGCATCTA-1	singlet	1	-2471.018525	-663.7501826	-1770.973427	EMD2
TACGCTCCAGTCTGGC-1	singlet	2	-935.5679697	-960.3904847	-331.8076394	EMD2
TACGCTCCATTAGGCT-1	singlet	1	-1976.524371	-642.5748034	-1718.115605	EMD2
TACGCTCGTTCTCCCA-1	singlet	2	-1903.785417	-1501.466227	-609.2807828	EMD2
TACGCTCTCACCTTGC-1	singlet	0	-1559.76044	-5070.780747	-5056.700223	EMD2
TACGCTCTCTTCGTAT-1	singlet	1	-2348.633512	-766.4500817	-2048.429887	EMD2
TACGGGCAGCGACATG-1	singlet	2	-2095.237161	-1706.479092	-778.2434658	EMD2
TACGGGCAGTTACGGG-1	singlet	1	-216.4502461	-86.55019269	-165.3845381	EMD2
TACGGGCTCAGCGGAA-1	singlet	1	-3986.55201	-1223.216731	-3238.467185	EMD2
TACGGTAAGCTGGTGA-1	singlet	2	-4229.746867	-3401.668589	-1468.094397	EMD2
TACGGTAAGTATCTGC-1	singlet	1	-6538.695244	-2161.203863	-5217.041657	EMD2
TACGGTACAAGGTCAG-1	singlet	1	-926.724756	-317.3915648	-821.0206714	EMD2
TACGGTACATCATCCC-1	singlet	0	-660.5924879	-2016.669697	-1800.03224	EMD2
TACGGTAGTAGCTGCC-1	singlet	1	-957.0582552	-258.2554689	-854.6650045	EMD2
TACGGTAGTCTCTCCA-1	singlet	1	-368.1484136	-143.5650559	-371.8449104	EMD2
TACGGTAGTTCCGCAG-1	singlet	1	-1239.138576	-349.9236454	-998.7611144	EMD2
TACGGTATCCAGGACC-1	singlet	2	-799.552238	-700.0812463	-356.0373408	EMD2
TACGGTATCCTGTAAG-1	singlet	0	-1187.156413	-3431.450949	-3341.692253	EMD2
TACGGTATCGCGTGCA-1	singlet	1	-208.4366868	-80.18021962	-158.8411022	EMD2
TACGTCCAGAGGTTAT-1	singlet	2	-562.9842906	-609.6624029	-247.6023881	EMD2
TACGTCCCATGCGGTC-1	singlet	1	-456.7294753	-151.3432465	-362.3320607	EMD2
TACGTCCGTAATTGGA-1	singlet	1	-345.0664664	-146.0078575	-320.540388	EMD2
TACGTCCGTGTATACC-1	singlet	2	-2824.108364	-2239.921024	-932.6776494	EMD2
TACGTCCTCGGAGTAG-1	singlet	0	-470.9999704	-1661.263052	-1532.028481	EMD2
TACTGCCAGAAACTAC-1	singlet	1	-496.9541001	-243.8782084	-442.2148014	EMD2
TACTGCCAGACTTCCA-1	singlet	2	-688.5115277	-541.1553268	-240.2109576	EMD2
TACTGCCAGAGTCCGA-1	singlet	1	-1959.14326	-853.7410602	-1862.209924	EMD2
TACTGCCAGCGTGAAC-1	singlet	2	-575.1299863	-569.4245349	-241.7590123	EMD2
TACTGCCAGGGTAATT-1	singlet	1	-472.6761909	-196.6224477	-376.8403353	EMD2
TACTGCCCAAATGAGT-1	singlet	2	-2312.086846	-1820.047839	-832.5392796	EMD2
TACTGCCCAACCTATG-1	singlet	1	-396.5825846	-132.2000557	-303.7261792	EMD2

TACTGCCCACGTTGGC-1	singlet	1	-1031.687462	-378.5467434	-831.0038372	EMD2
TACTGCCCACTATCGA-1	singlet	0	-502.2963795	-1420.333031	-1365.406957	EMD2
TACTGCCCATCCAACA-1	singlet	1	-686.6963566	-227.204027	-582.1398668	EMD2
TACTGCCCATGTTCGA-1	unassigned	0	-1.283951728	-1.30855543	-2.017629073	EMD2
TACTGCCGTATTGAGA-1	singlet	1	-5131.36318	-1920.016016	-4456.470521	EMD2
TACTGCCGTCACTTCC-1	singlet	0	-31.48538135	-65.56900134	-84.42134496	EMD2
TACTGCCGTCAGTCCG-1	singlet	0	-941.0089148	-3243.923261	-3087.23658	EMD2
TACTGCCGTCGTTCAA-1	singlet	1	-887.5234062	-492.3190809	-884.4258052	EMD2
TACTGCCTCGTAACTG-1	singlet	1	-3139.297548	-1093.502456	-2536.798024	EMD2
TACTTACAGGAACGTC-1	singlet	2	-45.07124516	-35.25289815	-28.90440344	EMD2
TACTTACAGGGAGGTG-1	singlet	1	-2074.03228	-609.9082957	-1531.778568	EMD2
TACTTACAGGTTTACC-1	singlet	1	-3303.125137	-1149.095982	-2736.071263	EMD2
TACTTACAGTCATAGA-1	singlet	2	-771.3766081	-661.1325424	-349.7347662	EMD2
TACTTACCAACGATCT-1	singlet	1	-817.5183381	-290.4763475	-679.8941526	EMD2
TACTTACCAACTGCCG-1	singlet	1	-238.0581523	-62.36407561	-154.245057	EMD2
TACTTACCAGAACTAA-1	singlet	0	-1018.4237	-3019.880357	-2783.481887	EMD2
TACTTACGTAGTTACC-1	singlet	1	-871.2901999	-348.5281836	-725.3056039	EMD2
TACTTACGTCCAATCA-1	singlet	1	-6427.222531	-1297.33417	-5118.079877	EMD2
TACTTACGTCGCATTA-1	singlet	2	-1067.489912	-895.717559	-442.2937764	EMD2
TACTTACGTCTTACTT-1	singlet	0	-782.1159722	-2157.738211	-1983.040574	EMD2
TACTTACGTTCAACGT-1	singlet	2	-1218.17695	-1248.232673	-337.8573844	EMD2
TACTTACGTTGCATAC-1	doublet	1/0	-2198.005471	-1825.357535	-2454.682774	EMD2
TACTTACGTTTGGGAG-1	singlet	0	-930.8331845	-2744.80831	-2568.020931	EMD2
TACTTACTCAGCTCTC-1	singlet	1	-571.0436777	-255.0592147	-484.4318164	EMD2
TACTTACTCATGTCAG-1	singlet	0	-363.6155861	-1302.880973	-1332.703625	EMD2
TACTTACTCTGGACTA-1	singlet	0	-361.722382	-1090.021956	-995.0551777	EMD2
TACTTCAAGACCTCCG-1	singlet	1	-2337.299506	-818.4439119	-1807.917391	EMD2
TACTTCAAGACTCAAA-1	singlet	1	-434.5943947	-152.5799099	-385.3354108	EMD2
TACTTCAAGCCTCAAT-1	singlet	0	-1147.818161	-3303.53543	-3169.646562	EMD2
TACTTCAAGCTAGTTC-1	singlet	2	-67.79881691	-72.8777269	-34.50197407	EMD2
TACTTCACAAATGGTA-1	singlet	2	-2141.647985	-1658.108181	-780.0266972	EMD2
TACTTCACAAGAAATC-1	singlet	0	-1948.070761	-6037.97511	-5831.823165	EMD2
TACTTCACAGCTTTCC-1	singlet	0	-521.0959594	-1525.203789	-1460.538607	EMD2
TACTTCACATGACAAA-1	singlet	1	-1940.938107	-624.1671514	-1716.782381	EMD2
TACTTCAGTACAGGTG-1	singlet	2	-2066.748197	-1799.737703	-677.8515225	EMD2
TACTTCAGTCAAACGG-1	singlet	1	-2003.532173	-564.5063221	-1453.998849	EMD2
TACTTCAGTTGTTGTG-1	singlet	1	-3451.389834	-1019.087562	-2682.810395	EMD2
TACTTCATCACTCGAA-1	singlet	2	-3138.058881	-2611.163168	-923.1446597	EMD2
TACTTCATCGAGATGG-1	singlet	1	-1608.939151	-361.1753773	-1303.231051	EMD2
TACTTGTAGGCGTTAG-1	singlet	1	-473.3060475	-158.0082481	-350.1392081	EMD2
TACTTGTCAAAGTGTA-1	singlet	1	-637.3213831	-206.5122236	-488.1031533	EMD2
TACTTGTCACGACGAA-1	singlet	1	-4079.206369	-1027.687245	-3124.981035	EMD2
TACTTGTCACTGATTG-1	singlet	2	-4826.334726	-4753.665261	-2118.766011	EMD2
TACTTGTGTATCCCTC-1	singlet	1	-1304.849501	-500.8113126	-1045.430588	EMD2
TACTTGTGTATGGTAA-1	singlet	1	-348.1519236	-139.3627273	-262.1347363	EMD2
TACTTGTGTCACAGTT-1	singlet	1	-1531.939868	-545.011345	-1305.619706	EMD2
TACTTGTGTCGTCAGC-1	singlet	2	-2429.383687	-2180.422233	-918.4707626	EMD2
TACTTGTGTGGCTGCT-1	singlet	1	-85.34023885	-23.87622191	-75.75160538	EMD2
TACTTGTTCCTACCGT-1	singlet	0	-1036.550523	-4704.567443	-4849.991354	EMD2
TAGACCAAGACGCATG-1	singlet	1	-1854.794251	-616.4026559	-1579.179101	EMD2
TAGACCAAGACGGTTG-1	singlet	0	-338.2859742	-1141.726571	-1101.643557	EMD2
TAGACCAAGCGACAGT-1	singlet	2	-4945.229799	-4209.442926	-1420.690692	EMD2
TAGACCAAGCGTATGG-1	singlet	1	-2074.218311	-582.3490943	-2047.668525	EMD2
TAGACCAAGGATTTCC-1	singlet	1	-425.1578077	-155.0969664	-305.6289517	EMD2
TAGACCAAGGTCCGAA-1	singlet	1	-181.3496831	-67.92264032	-114.6955444	EMD2
TAGACCAAGGTGCTGA-1	singlet	1	-1081.545546	-369.0327481	-999.8211329	EMD2
TAGACCAAGGTTCACT-1	singlet	1	-245.7649746	-67.90012288	-173.8237716	EMD2
TAGACCAAGTACGTCT-1	singlet	2	-1007.099082	-932.955658	-385.263724	EMD2
TAGACCAAGTGAGGTC-1	singlet	1	-2140.567461	-697.5865714	-1525.967659	EMD2
TAGACCACACAATCTG-1	singlet	1	-170.0466698	-61.12544939	-132.1537823	EMD2
TAGACCACAGCGTAGA-1	singlet	1	-2081.680868	-782.0062464	-1808.584119	EMD2
TAGACCACATATCGGT-1	singlet	0	-1275.280086	-4800.285522	-4431.213471	EMD2
TAGACCAGTCTACAGT-1	doublet	0/1	-1901.024056	-2221.386661	-2956.149404	EMD2
TAGACCAGTCTCAGAT-1	singlet	2	-1959.079415	-2019.984424	-539.831772	EMD2
TAGACCATCACAAGAA-1	singlet	1	-3692.319326	-1205.17999	-2852.672435	EMD2
TAGACCATCAGAGTGG-1	singlet	1	-407.7748213	-118.0268113	-382.8560074	EMD2
TAGACCATCATAGAGA-1	singlet	1	-1926.932932	-680.47808	-1630.691872	EMD2
TAGACCATCCTTGAAG-1	singlet	2	-2983.894172	-2602.82467	-1015.630135	EMD2
TAGACTGAGATGCCGA-1	singlet	0	-50.99253088	-161.8332351	-180.5897325	EMD2
TAGACTGAGTCAGCGA-1	singlet	2	-385.4893622	-425.9299649	-176.9714148	EMD2
TAGACTGAGTCCCTAA-1	singlet	2	-4038.293992	-3483.406099	-1277.34259	EMD2
TAGACTGAGTGGTCAG-1	singlet	2	-3792.781453	-3029.145521	-1213.176949	EMD2
TAGACTGCACACAGCC-1	singlet	1	-3046.536854	-1014.996863	-2522.863441	EMD2
TAGACTGCACCAGGTC-1	singlet	1	-251.8087221	-89.34571731	-202.3556155	EMD2
TAGACTGCACCATATG-1	singlet	1	-4118.321378	-1401.878747	-3288.762841	EMD2
TAGACTGGTACCTAGT-1	singlet	2	-738.6993951	-707.3350035	-325.0341305	EMD2
TAGACTGGTAGGAGGG-1	singlet	1	-267.5056439	-108.840593	-221.6263788	EMD2
TAGACTGTCTATGCCC-1	singlet	1	-1731.744687	-562.7158055	-1411.603284	EMD2
TAGAGTCAGAACTGAT-1	singlet	1	-426.1273315	-166.4044248	-360.1301986	EMD2
TAGAGTCAGACGCATG-1	singlet	2	-390.2338235	-461.3609631	-171.0893492	EMD2
TAGAGTCAGCAAGCCA-1	singlet	2	-1232.083623	-1144.60579	-365.7778427	EMD2
TAGAGTCAGCCAAGGT-1	singlet	1	-2383.259383	-821.2678278	-1914.743414	EMD2
TAGAGTCAGCGAGTAC-1	singlet	1	-604.1619593	-246.7236379	-497.1405692	EMD2
TAGAGTCAGCTAAGTA-1	doublet	45293	-562.3792138	-306.0554406	-410.2359187	EMD2
TAGAGTCCAGAGTGTG-1	singlet	1	-1115.96971	-404.6846069	-919.801146	EMD2
TAGAGTCGTCCTCAGG-1	singlet	1	-433.9038265	-141.9184545	-328.4686302	EMD2
TAGAGTCGTCGACGCT-1	singlet	1	-178.082534	-85.49802384	-199.2119456	EMD2
TAGAGTCGTGAGTTTC-1	singlet	1	-23.46068782	-2.784977582	-5.360073588	EMD2
TAGAGTCGTTACCTGA-1	singlet	1	-201.9710213	-81.33165087	-197.1032814	EMD2
TAGAGTCTCCCACAGG-1	singlet	1	-142.776115	-60.8472164	-122.2625016	EMD2
TAGAGTCTCGGCTGAC-1	singlet	0	-1158.481139	-4216.137202	-3588.43146	EMD2
TAGAGTCTCTTAATCC-1	singlet	1	-1305.592038	-469.1605863	-1066.958957	EMD2
TAGATCGAGACCCTTA-1	singlet	2	-1378.03585	-1368.359698	-572.4501589	EMD2
TAGATCGAGAGAGCCT-1	doublet	0/2	-2936.980146	-4907.242098	-3595.939474	EMD2
TAGATCGCATCTTCGC-1	singlet	2	-3274.507832	-2966.720832	-1112.484358	EMD2
TAGATCGCATGAAGGC-1	singlet	0	-26.18850404	-97.15332608	-72.23083765	EMD2
TAGATCGCATTGACCA-1	singlet	1	-1236.976969	-487.1392428	-1049.257629	EMD2
TAGATCGGTAGCGCCT-1	singlet	2	-379.5653559	-310.4382009	-127.1199494	EMD2
TAGATCGGTATGGGAC-1	singlet	1	-649.1399772	-220.8210183	-582.1016308	EMD2
TAGATCGGTGCCTGAC-1	singlet	1	-771.7182136	-238.9085956	-650.5768678	EMD2
TAGATCGGTGGACTAG-1	singlet	0	-946.1241141	-2897.748068	-2806.96213	EMD2
TAGATCGGTGTTTACG-1	singlet	0	-148.9918683	-411.3991534	-372.3385245	EMD2
TAGATCGTCCATGATG-1	singlet	1	-2892.062102	-1043.997366	-2219.093535	EMD2
TAGATCGTCTCCTACG-1	singlet	1	-841.5190793	-294.399441	-755.2764942	EMD2
TAGCACAAGGTACAAT-1	singlet	2	-2980.308273	-2437.467851	-921.9187378	EMD2
TAGCACACAAAGTATG-1	singlet	0	-389.9740185	-1049.368617	-954.587509	EMD2
TAGCACACAAGATGTA-1	singlet	2	-7779.723219	-5936.194852	-1804.154523	EMD2
TAGCACACACCTCTAC-1	singlet	0	-404.4111546	-1053.121033	-980.195306	EMD2
TAGCACACAGTCTTCC-1	doublet	45323	-1125.74938	-810.6151625	-696.695215	EMD2
TAGCACACATGCACTA-1	singlet	1	-2837.408075	-842.1309465	-2123.678463	EMD2

TAGCACAGTACTCGCG-1	singlet	1	-2299.412058	-727.7203393	-2154.179871	EMD2
TAGCACAGTCGTCGGT-1	singlet	1	-556.5912559	-178.8745229	-450.1491629	EMD2
TAGCACAGTCTCAAGT-1	singlet	1	-3179.458039	-1010.939019	-2521.105956	EMD2
TAGCACAGTTACGGAG-1	singlet	1	-1052.838609	-365.0097646	-965.4814387	EMD2
TAGCACATCCATATGG-1	singlet	2	-2258.352136	-1995.052895	-763.433061	EMD2
TAGCACATCCTCACGT-1	singlet	2	-2932.417856	-2483.519717	-921.2882134	EMD2
TAGCACATCGTAGTCA-1	singlet	0	-312.6621473	-859.4754258	-736.4308276	EMD2
TAGGAGGAGACAGCTG-1	singlet	0	-551.3156081	-1692.143409	-1584.471041	EMD2
TAGGAGGAGCATCCTA-1	doublet	1/0	-1359.29182	-1287.492133	-1711.967341	EMD2
TAGGAGGCAGCAGTAG-1	singlet	1	-1952.801393	-631.4275094	-1697.067416	EMD2
TAGGAGGCATAGGTTC-1	singlet	2	-2553.816355	-2332.39183	-702.4480649	EMD2
TAGGAGGGTACCACGC-1	singlet	1	-567.9864486	-213.361969	-441.9408038	EMD2
TAGGAGGGTAGGCTGA-1	singlet	0	-510.6183268	-1455.712445	-1315.80292	EMD2
TAGGAGGGTATGAAAC-1	singlet	1	-2327.578753	-581.4486882	-1790.258221	EMD2
TAGGAGGGTCCTGAAT-1	singlet	2	-2100.847495	-1953.662975	-659.8318964	EMD2
TAGGAGGTCGACACCG-1	singlet	1	-1263.771632	-483.4175357	-1098.116175	EMD2
TAGGAGGTCGCAATGT-1	singlet	1	-317.2401613	-127.2687393	-262.1182608	EMD2
TAGGAGGTCGCTACGG-1	singlet	1	-2461.552698	-773.6900235	-2031.708279	EMD2
TAGGAGGTCTGCAGCG-1	singlet	1	-166.9773851	-56.59593918	-139.5410939	EMD2
TAGGAGGTCTGGACTA-1	singlet	1	-31.28015699	-11.38175994	-17.85206675	EMD2
TAGGGTTAGCGATCGA-1	singlet	1	-873.0329792	-258.8715107	-686.2830885	EMD2
TAGGGTTAGCTTGTTG-1	singlet	1	-380.9757957	-145.1186383	-319.8207219	EMD2
TAGGGTTAGGAGCTGT-1	singlet	1	-268.9164474	-100.0306079	-196.4792517	EMD2
TAGGGTTAGGGAACAA-1	singlet	1	-1888.686318	-478.6355143	-1463.277896	EMD2
TAGGGTTAGGTTATAG-1	singlet	0	-1971.479036	-6323.094788	-5963.79504	EMD2
TAGGGTTAGTGCAACG-1	singlet	0	-292.9611059	-914.3743783	-742.1286909	EMD2
TAGGGTTCACAGCATT-1	singlet	1	-773.223385	-252.7359996	-610.8438233	EMD2
TAGGGTTGTGCCCGTA-1	singlet	1	-1428.811874	-410.2463985	-1089.355402	EMD2
TAGGGTTGTTTGCAGT-1	singlet	1	-310.5628881	-114.2005586	-235.3571161	EMD2
TAGGGTTTCAAACTGC-1	singlet	0	-487.5621194	-1309.269988	-1277.974019	EMD2
TAGGGTTTCAATGTCG-1	singlet	1	-327.3140263	-127.4246613	-269.6917553	EMD2
TAGGGTTTCCAAGCCG-1	singlet	1	-409.7754684	-142.7692898	-325.9973626	EMD2
TAGGGTTTCTCATGCC-1	singlet	1	-538.4177604	-175.9030107	-418.9854705	EMD2
TAGGTACAGAGTCCGA-1	singlet	0	-103.2644187	-235.7721091	-260.9579676	EMD2
TAGGTACAGATGCAGC-1	singlet	1	-920.3358923	-356.7222797	-702.4974293	EMD2
TAGGTACAGCTGGTGA-1	singlet	1	-257.4681176	-106.0878633	-245.374254	EMD2
TAGGTACAGTTTCGAC-1	singlet	1	-381.5945797	-135.2426569	-321.5314401	EMD2
TAGGTACCAACAAAGT-1	singlet	1	-2453.776661	-860.1647278	-2000.746384	EMD2
TAGGTACCAAGACGGT-1	singlet	1	-570.0438528	-243.653476	-474.5759129	EMD2
TAGGTACCACTGCGAC-1	singlet	1	-1480.570167	-432.8397248	-1061.807201	EMD2
TAGGTACCATCGGCCA-1	singlet	0	-50.98289088	-153.9941609	-161.1181915	EMD2
TAGGTACGTATGCTAC-1	singlet	1	-3832.832304	-1262.668166	-3186.850816	EMD2
TAGGTACTCAAGTTGC-1	singlet	1	-372.576385	-162.2649186	-298.5279273	EMD2
TAGGTACTCCGTTTCG-1	singlet	1	-1536.482851	-434.9489294	-1092.494797	EMD2
TAGGTTGAGCTTGTTG-1	singlet	1	-352.0217081	-167.3202348	-301.5400221	EMD2
TAGGTTGAGGACGCAT-1	singlet	1	-2984.400055	-828.4685266	-2209.342327	EMD2
TAGGTTGCAATGCAAA-1	singlet	1	-997.5217714	-423.1124604	-814.9448162	EMD2
TAGGTTGCAGGACATG-1	singlet	0	-938.4681985	-2734.563607	-2908.923365	EMD2
TAGGTTGGTACAAACA-1	singlet	0	-35.47879155	-92.49907102	-106.0592588	EMD2
TAGGTTGGTCTGCATA-1	singlet	1	-3999.52434	-1132.216537	-3451.510362	EMD2
TAGGTTGGTCTTGCGG-1	singlet	2	-341.6679895	-326.4108668	-136.9759373	EMD2
TAGGTTGGTTCCGCGA-1	singlet	1	-378.2609644	-109.574613	-253.6210572	EMD2
TAGGTTGTCACGACTA-1	singlet	1	-1148.614714	-342.2035147	-822.549441	EMD2
TAGGTTGTCATTGTTC-1	singlet	1	-1562.173302	-533.1970861	-1322.115909	EMD2
TAGGTTGTCCACGGAC-1	singlet	1	-825.8965964	-289.182341	-751.3597993	EMD2
TAGGTTGTCCTCAGGG-1	singlet	1	-385.5725415	-144.4289299	-306.5503964	EMD2
TAGGTTGTCTTAAGGC-1	singlet	0	-725.0950941	-1173.177237	-1275.790554	EMD2
TAGTGCAAGCGGCTCT-1	singlet	0	-436.1647914	-1645.910702	-1497.067069	EMD2
TAGTGCAAGGGTGGGA-1	singlet	2	-1589.057075	-1534.448257	-571.1012087	EMD2
TAGTGCACAATAACGA-1	singlet	2	-580.4801969	-514.1676961	-218.9767586	EMD2
TAGTGCACATAGATGA-1	singlet	2	-336.6666572	-364.1496324	-149.7775353	EMD2
TAGTGCACATGTCTAG-1	singlet	2	-2664.114783	-2132.208003	-940.4686888	EMD2
TAGTGCAGTATCCCAA-1	singlet	1	-2957.995229	-1069.325398	-2483.384762	EMD2
TAGTGCATCGTTCAGA-1	singlet	0	-699.7404219	-2199.257712	-2153.655891	EMD2
TATACCTAGCTACGTT-1	singlet	1	-1125.923897	-425.2183592	-915.325183	EMD2
TATACCTAGGAATCGC-1	singlet	2	-383.6786744	-387.0808872	-146.3349122	EMD2
TATACCTAGGTTTACC-1	singlet	1	-2953.905362	-906.7688773	-2437.785613	EMD2
TATACCTCATTGCAAC-1	singlet	1	-3484.662937	-988.8127202	-2591.742759	EMD2
TATACCTGTCTAACTG-1	singlet	1	-1310.116393	-483.7780676	-1115.944465	EMD2
TATACCTGTGACTAAA-1	singlet	0	-1908.672456	-5545.388867	-5407.663778	EMD2
TATACCTGTGGCTTAT-1	singlet	1	-1419.478735	-472.9938332	-1090.089218	EMD2
TATACCTGTGGTCCGT-1	singlet	2	-1247.456288	-1331.286728	-413.6736345	EMD2
TATACCTGTGTCGATT-1	singlet	1	-1586.288299	-477.2874099	-1320.709896	EMD2
TATACCTGTTCCTACC-1	singlet	0	-94.24707531	-344.7751232	-276.4257894	EMD2
TATACCTGTTCTTGTT-1	singlet	1	-321.6617665	-131.0684723	-250.2834303	EMD2
TATACCTGTTGAGGAC-1	singlet	1	-208.2077906	-50.41219115	-152.4211817	EMD2
TATACCTGTTGTAGCT-1	unassigned	1	-15.92252909	-6.53931879	-8.293997701	EMD2
TATACCTTCATGCGGC-1	singlet	1	-524.8580219	-210.49717	-524.6362712	EMD2
TATACCTTCGGATAAA-1	singlet	1	-1393.28784	-475.7473994	-1045.9595	EMD2
TATACCTTCTGCCCTA-1	singlet	1	-1680.643427	-445.3600217	-1435.778118	EMD2
TATATCCAGAAGCGAA-1	singlet	1	-3607.997173	-1105.532332	-2776.351625	EMD2
TATATCCAGAAGCGCT-1	singlet	1	-346.0002575	-160.0357345	-345.6995541	EMD2
TATATCCAGGCTCTCG-1	singlet	0	-909.7224633	-2689.90065	-2428.428891	EMD2
TATATCCAGTCTAGAA-1	singlet	2	-887.7554097	-855.9946989	-342.3475103	EMD2
TATATCCCAACGGCTC-1	singlet	2	-1169.562645	-1233.602643	-448.0551882	EMD2
TATATCCCAATAACGA-1	singlet	1	-2619.014599	-900.3378896	-2281.43298	EMD2
TATATCCCATTGCCGG-1	singlet	1	-448.7387747	-191.1323202	-414.9985911	EMD2
TATATCCGTACCCGCA-1	singlet	1	-2434.710726	-750.4930832	-2093.979201	EMD2
TATATCCGTCCATACA-1	singlet	1	-1455.181915	-582.7779435	-1198.470145	EMD2
TATATCCGTGACACAG-1	singlet	1	-1689.613692	-487.7104937	-1209.661715	EMD2
TATATCCGTGCGCTCA-1	singlet	0	-89.41024104	-200.4940183	-193.8709383	EMD2
TATATCCTCAGTGATC-1	singlet	2	-894.9671935	-940.8419334	-310.4708015	EMD2
TATATCCTCTAGTCAG-1	singlet	1	-435.301889	-180.2395448	-358.653643	EMD2
TATCAGGAGATGAACT-1	singlet	1	-2335.674605	-671.3732219	-1866.325818	EMD2
TATCAGGAGGCGTTAG-1	singlet	0	-320.1949885	-1031.002877	-913.7292789	EMD2
TATCAGGAGTGTACCT-1	singlet	1	-397.8357345	-174.4377634	-386.8805897	EMD2
TATCAGGCATAACGGG-1	singlet	0	-1763.34826	-5449.244686	-5189.23478	EMD2
TATCAGGGTACAGCGA-1	singlet	0	-1649.736706	-4872.074257	-4537.771133	EMD2
TATCAGGGTCACCCTT-1	singlet	1	-504.2349541	-176.9213243	-382.7723718	EMD2
TATCAGGGTCTCGCGA-1	doublet	45293	-1205.02204	-709.0406188	-733.4132676	EMD2
TATCAGGGTGTTTGCA-1	singlet	1	-184.4343829	-51.7044689	-156.5822084	EMD2
TATCAGGGTTCGGTCG-1	singlet	1	-553.8199231	-215.3897147	-437.5569237	EMD2
TATCAGGTCCATCGTC-1	singlet	2	-144.9220296	-152.6981872	-73.36386087	EMD2
TATCAGGTCCCTCTAG-1	singlet	1	-248.59107	-116.3736078	-200.6519117	EMD2
TATCAGGTCCGTGTCT-1	singlet	1	-1695.811745	-529.6541951	-1577.7051	EMD2
TATCAGGTCTACACAG-1	singlet	2	-737.2043621	-669.2907394	-381.0774564	EMD2
TATCAGGTCTACCAGA-1	singlet	1	-1725.699209	-567.7202438	-1314.564655	EMD2
TATCCTAAGCCTGGAA-1	singlet	1	-248.9589837	-117.6914285	-216.0795502	EMD2
TATCCTAAGCGTTCCG-1	singlet	1	-396.2629484	-155.2972298	-358.4528778	EMD2
TATCCTAAGGTTGCCC-1	singlet	1	-651.6981222	-276.3877849	-531.3819332	EMD2

TATCCTAAGTAACCGG-1	singlet	1	-4190.055028	-1383.879851	-3333.149653	EMD2
TATCCTAAGTTTGAGA-1	singlet	1	-510.2616179	-187.8528369	-365.108961	EMD2
TATCCTACAGAACTTC-1	singlet	1	-2468.77177	-784.7598214	-2226.223966	EMD2
TATCCTACAGCAATTC-1	singlet	1	-938.7426539	-315.102641	-762.1751598	EMD2
TATCCTAGTAAGAACT-1	singlet	2	-1292.303623	-938.1351191	-367.2844961	EMD2
TATCCTAGTACCCAGC-1	singlet	2	-411.6500865	-402.6882129	-184.022188	EMD2
TATCCTAGTTCGATTG-1	unassigned	0/1	-3.603430246	-5.899632328	-12.89422153	EMD2
TATCCTATCCTGCTAC-1	singlet	1	-2955.857548	-953.2436869	-2383.275082	EMD2
TATCGCCAGACAACTA-1	singlet	1	-4926.598818	-1373.518793	-3430.33521	EMD2
TATCGCCAGCGGCTCT-1	singlet	1	-2487.243904	-705.5914151	-1875.854203	EMD2
TATCGCCAGTGGTCAG-1	singlet	1	-4437.672322	-1255.371645	-3446.391177	EMD2
TATCGCCAGTTATGGA-1	singlet	1	-1655.574886	-532.4715661	-1318.190051	EMD2
TATCGCCCAAACAGGC-1	singlet	1	-1866.198666	-621.3839409	-1644.140091	EMD2
TATCGCCCAGAACCGA-1	singlet	0	-1187.097752	-3862.453713	-3608.902793	EMD2
TATCGCCGTCCAAATC-1	singlet	1	-2031.366631	-554.2851827	-1418.638589	EMD2
TATCGCCGTTAACAGA-1	singlet	1	-865.4509256	-287.7510956	-674.1847825	EMD2
TATCGCCTCCTAGCGG-1	singlet	1	-1152.906879	-312.2007199	-823.4673161	EMD2
TATCGCCTCCTTCAGC-1	singlet	2	-294.447348	-231.0648169	-108.6383615	EMD2
TATCGCCTCGAACACT-1	singlet	1	-2938.4251	-607.7166498	-1772.146689	EMD2
TATCTGTAGGAGCTGT-1	doublet	45293	-1382.787454	-972.415311	-1148.753912	EMD2
TATCTGTAGGAGGTTC-1	singlet	2	-4533.937243	-3496.17967	-1226.783422	EMD2
TATCTGTAGGTAGCCA-1	singlet	1	-4780.335154	-1352.273458	-3897.799083	EMD2
TATCTGTCAGGTCCCA-1	singlet	0	-892.5994834	-2661.748761	-2551.3804	EMD2
TATCTGTCATCACAGT-1	singlet	1	-1012.555265	-380.1731195	-900.2511738	EMD2
TATCTGTCATCAGCAT-1	singlet	1	-1691.498889	-627.3786279	-1570.49647	EMD2
TATCTGTCATTGACAC-1	singlet	0	-348.6628248	-987.4624163	-801.5474655	EMD2
TATCTGTGTATCGAGG-1	singlet	1	-1840.710289	-521.430035	-1531.182614	EMD2
TATCTGTGTCAGTCCG-1	singlet	2	-2461.70181	-1977.635998	-839.9883202	EMD2
TATCTGTGTCGTTATG-1	singlet	2	-659.3540986	-653.3251202	-247.6749129	EMD2
TATCTGTGTCTAGATC-1	singlet	1	-330.6071818	-99.35581618	-271.4790868	EMD2
TATCTGTGTTTCGTAG-1	singlet	2	-727.4278919	-664.383663	-249.4215521	EMD2
TATCTGTTCAACTGGT-1	singlet	1	-212.2805598	-104.8607517	-181.4448367	EMD2
TATCTGTTCATATGGC-1	singlet	0	-1618.228333	-4268.749613	-3838.24834	EMD2
TATCTGTTCATTACCT-1	doublet	45293	-11.97646003	-7.81728056	-9.577263225	EMD2
TATCTGTTCATTGTTC-1	singlet	1	-2682.390829	-929.5209009	-2130.048806	EMD2
TATCTGTTCGACGACC-1	singlet	1	-4738.547945	-977.3933435	-2941.921526	EMD2
TATCTGTTCGCATGAT-1	singlet	1	-179.3794544	-71.84234336	-115.0428962	EMD2
TATCTGTTCGGATACT-1	singlet	1	-252.8518072	-95.37812084	-220.518851	EMD2
TATCTTGAGAGGTCGT-1	singlet	1	-433.0513377	-165.3228397	-338.8741833	EMD2
TATCTTGAGCCTGTCG-1	singlet	2	-886.0787647	-853.1037273	-282.3775375	EMD2
TATCTTGAGGCACGAT-1	singlet	1	-1270.656937	-413.4991533	-963.2193887	EMD2
TATCTTGAGTGCACAG-1	singlet	1	-273.7652009	-65.15199803	-205.6007017	EMD2
TATCTTGCAAGTATAG-1	singlet	1	-4554.020576	-1103.684441	-3305.235688	EMD2
TATCTTGCAGAAACCG-1	singlet	1	-2174.624291	-688.4915812	-1838.224165	EMD2
TATCTTGCAGAGGTTG-1	singlet	1	-354.3116312	-149.4854523	-301.5175632	EMD2
TATCTTGCAGTCCCGA-1	doublet	0/1	-4737.33585	-5534.916373	-7535.14618	EMD2
TATCTTGCATCCGGTG-1	singlet	1	-545.0560248	-191.9047405	-507.2747231	EMD2
TATCTTGCATCGCTCT-1	singlet	0	-501.2263078	-1751.340293	-1525.062257	EMD2
TATCTTGGTAGGAGTC-1	singlet	0	-116.5441636	-416.555818	-309.8607287	EMD2
TATCTTGGTCGATTTG-1	singlet	1	-1167.300748	-369.0040116	-887.916349	EMD2
TATCTTGGTGAATATG-1	singlet	1	-5564.738924	-1663.076497	-4160.278053	EMD2
TATCTTGGTTTCAGAC-1	singlet	1	-567.1479216	-200.3180173	-498.0982814	EMD2
TATCTTGTCCGCAGTG-1	singlet	1	-3412.199335	-908.1055137	-2520.154327	EMD2
TATGTTCAGAATGTTG-1	singlet	2	-1354.564762	-1405.811755	-525.3704805	EMD2
TATGTTCAGAGAGTGA-1	singlet	1	-99.04455002	-45.1675513	-120.3530746	EMD2
TATGTTCAGAGCAAGA-1	singlet	1	-721.5570036	-243.0128281	-520.6914565	EMD2
TATGTTCAGCTCTGTA-1	singlet	0	-802.5054034	-2526.900261	-2552.16534	EMD2
TATGTTCCAAATCAGA-1	singlet	1	-269.0482294	-87.47104569	-154.8403624	EMD2
TATGTTCCAACGATTC-1	singlet	1	-12028.98318	-3915.603659	-9579.24486	EMD2
TATGTTCCACTTGAAC-1	singlet	0	-866.1071627	-2529.253307	-2470.231602	EMD2
TATGTTCCAGTATACC-1	singlet	0	-19.50089553	-67.02389033	-57.34409872	EMD2
TATGTTCCATATCTGG-1	singlet	1	-254.830819	-64.17852588	-136.7750414	EMD2
TATGTTCCATGTTCAG-1	singlet	1	-474.0831257	-180.6576337	-377.0250408	EMD2
TATGTTCCATTCGGGC-1	singlet	1	-1939.479466	-686.2328017	-1518.353687	EMD2
TATGTTCGTTTGTTCT-1	singlet	2	-1864.193523	-1735.436108	-690.3386623	EMD2
TATTCCACAAATGCGG-1	singlet	1	-1511.397882	-454.9834718	-1144.883293	EMD2
TATTCCACATGAGGGT-1	singlet	1	-1512.224327	-526.6864099	-1302.01105	EMD2
TATTCCAGTACGAGCA-1	singlet	1	-895.2587901	-269.7662165	-716.0013932	EMD2
TATTCCAGTCTTACTT-1	singlet	0	-1752.202106	-6820.488047	-6555.36295	EMD2
TATTCCATCATGCCAA-1	singlet	0	-606.1797331	-1807.682372	-1707.402703	EMD2
TATTCCATCGAAGTGG-1	singlet	0	-82.41910554	-167.3107947	-187.4685868	EMD2
TATTCCATCGGCTCTT-1	doublet	1/0	-45.21018424	-41.82211394	-84.05369559	EMD2
TATTCCATCGTCACCT-1	singlet	1	-2701.75632	-754.406453	-2175.909136	EMD2
TATTCCATCTGCATAG-1	singlet	0	-148.064279	-377.8040595	-347.7267285	EMD2
TATTGCTAGCATCAGG-1	singlet	2	-404.2531217	-363.9325487	-135.5256984	EMD2
TATTGCTAGTTATGGA-1	singlet	1	-181.4501907	-76.73672928	-164.0661962	EMD2
TATTGCTCATTCTGTT-1	singlet	0	-84.56767601	-266.6294264	-249.5987598	EMD2
TATTGCTGTATCCCAA-1	singlet	0	-1362.734628	-4673.949394	-4401.927624	EMD2
TATTGCTGTATCGAAA-1	singlet	1	-459.019484	-191.7911931	-356.9236251	EMD2
TATTGCTGTCGGTAAG-1	singlet	2	-927.9048975	-900.5347148	-420.1288632	EMD2
TATTGCTGTCTAGATC-1	singlet	1	-1473.701048	-560.4413447	-1144.116338	EMD2
TATTGCTTCCACTGGG-1	singlet	1	-283.9677134	-107.1141098	-213.6933224	EMD2
TATTGCTTCGCTCTCA-1	singlet	0	-1600.823377	-5639.358242	-5407.83496	EMD2
TATTGGGAGAGCCGTA-1	singlet	2	-2462.473862	-2060.589167	-861.3400704	EMD2
TATTGGGAGCAATAGT-1	singlet	1	-511.1956592	-192.3018078	-391.9722043	EMD2
TATTGGGAGTACCATC-1	singlet	2	-159.8066423	-122.0503019	-44.70239203	EMD2
TATTGGGGTCTAACTG-1	singlet	1	-268.9266465	-113.7304784	-279.7586578	EMD2
TATTGGGGTCTACAAC-1	doublet	45323	-5056.695662	-2920.64524	-2666.443034	EMD2
TATTGGGGTCTGTCAA-1	singlet	2	-2164.504836	-1924.145382	-829.2463055	EMD2
TATTGGGTCATTATCC-1	singlet	1	-2441.152039	-836.4064959	-2155.386894	EMD2
TATTGGGTCGCCTTTG-1	singlet	1	-1981.355781	-645.919313	-1556.640788	EMD2
TATTGGGTCGGCTGAC-1	singlet	1	-154.0989864	-56.29012921	-124.28206	EMD2
TATTTCGAGAGAGTGA-1	singlet	2	-3370.145209	-3082.754248	-1233.617658	EMD2
TATTTCGAGGATATGT-1	singlet	1	-2700.042239	-848.7110887	-2206.139801	EMD2
TATTTCGAGGATGGCT-1	singlet	1	-2500.344999	-746.8840649	-2188.247788	EMD2
TATTTCGCAGCGTTTA-1	singlet	1	-317.414381	-120.6439156	-266.6108742	EMD2
TATTTCGCAGTGTGCC-1	singlet	2	-593.6851361	-591.9406709	-177.8273614	EMD2
TATTTCGGTCCATAGT-1	doublet	0/1	-3110.41727	-3198.624525	-4438.117717	EMD2
TATTTCGGTCCTACAA-1	singlet	2	-361.7545283	-380.1531994	-173.7155324	EMD2
TATTTCGGTCGAACGA-1	singlet	2	-3898.88918	-3031.079551	-1252.015587	EMD2
TATTTCGGTCTTACAG-1	singlet	1	-382.4947992	-147.9027701	-341.2692947	EMD2
TATTTCGGTGTGTGTT-1	singlet	0	-1323.510774	-3234.841913	-3282.15201	EMD2
TATTTCGGTTCGTGCG-1	singlet	1	-3640.679365	-997.5313399	-2948.245015	EMD2
TATTTCGTCCAAGAGG-1	singlet	0	-862.1447625	-2726.087847	-2515.914752	EMD2
TATTTCGTCGAAGCAG-1	singlet	0	-53.00200034	-136.0118889	-120.697124	EMD2
TCAAGACAGAATCCCT-1	singlet	0	-789.054781	-2432.982422	-2174.878707	EMD2
TCAAGACAGGGTGGGA-1	singlet	1	-2201.971334	-655.6815666	-1793.230033	EMD2
TCAAGACAGTCTAACC-1	doublet	45323	-3152.715983	-2213.340659	-1823.440776	EMD2
TCAAGACCAAATGGAT-1	singlet	1	-442.6398511	-157.5561236	-400.5582866	EMD2
TCAAGACCACTGTGAT-1	singlet	0	-568.8247369	-1710.301761	-1690.01371	EMD2

TCAAGACCAGGCATTT-1	singlet	2	-3559.697325	-2675.172553	-984.1243252	EMD2
TCAAGACCATATTCGG-1	singlet	0	-374.61895	-1028.68695	-965.7024641	EMD2
TCAAGACGTATGGGAC-1	singlet	0	-521.013906	-1396.449665	-1410.660596	EMD2
TCAAGACTCCCTTCCC-1	singlet	2	-403.085341	-447.3074771	-173.9499919	EMD2
TCAAGACTCCTCAGAA-1	singlet	1	-91.87557076	-32.4503142	-65.85299785	EMD2
TCAAGCAAGCGCACAA-1	singlet	1	-597.0085208	-167.5162638	-387.0077159	EMD2
TCAAGCAAGGCCTGCT-1	singlet	1	-1379.085926	-493.7475294	-1144.086175	EMD2
TCAAGCAAGGTTCAGG-1	singlet	2	-1641.712299	-1413.99811	-559.9884156	EMD2
TCAAGCAAGGTTCATC-1	singlet	1	-2125.346687	-754.3488589	-1654.514732	EMD2
TCAAGCACACCAAAGG-1	singlet	1	-1075.061078	-229.1150431	-764.5399796	EMD2
TCAAGCACACCCTATC-1	singlet	1	-527.5371407	-214.1841875	-453.9861484	EMD2
TCAAGCACACCTTCCA-1	singlet	0	-815.2892672	-2415.275832	-2268.721584	EMD2
TCAAGCACACTCCGGA-1	singlet	2	-3940.851013	-3395.569754	-1187.116728	EMD2
TCAAGCACACTGCGAC-1	singlet	0	-2485.088776	-7699.843297	-7275.684553	EMD2
TCAAGCACAGTGACCC-1	singlet	2	-3263.553962	-2665.140884	-966.5110754	EMD2
TCAAGCACAGTTGGTT-1	singlet	1	-337.9995835	-138.2716182	-303.5710238	EMD2
TCAAGCAGTTCGTAAC-1	singlet	1	-1683.146341	-550.6573648	-1286.333834	EMD2
TCAAGCAGTTGGGCCT-1	singlet	2	-1377.169253	-1444.703123	-407.8218081	EMD2
TCAAGCAGTTTACCAG-1	singlet	0	-1570.183556	-4728.444885	-4697.124367	EMD2
TCAAGCATCGCATTAG-1	singlet	0	-131.6126278	-326.0007009	-320.6860672	EMD2
TCAAGTGAGAGAACCC-1	singlet	1	-306.1727943	-111.609473	-235.3889133	EMD2
TCAAGTGAGCGACATG-1	singlet	1	-387.9940197	-141.7302081	-302.4188441	EMD2
TCAAGTGAGGATTACA-1	singlet	1	-3647.75784	-1160.991633	-2820.404205	EMD2
TCAAGTGAGGTTGCCC-1	singlet	1	-75.59117972	-27.81407335	-81.56254842	EMD2
TCAAGTGCAGTGTGCC-1	singlet	1	-3295.190292	-1128.378699	-2799.158246	EMD2
TCAAGTGCATAAGCGG-1	singlet	2	-3149.542603	-2540.066986	-962.6880444	EMD2
TCAAGTGGTCAGTCTA-1	singlet	1	-372.6595754	-133.2503486	-298.8970565	EMD2
TCAAGTGGTGCCGTTG-1	singlet	1	-209.6792443	-105.2057111	-159.3654377	EMD2
TCAAGTGGTGGGCTCT-1	doublet	45323	-6945.241828	-3762.449909	-3078.597745	EMD2
TCAAGTGTCTATTGTC-1	doublet	1/0	-444.9724181	-394.2466525	-527.0649639	EMD2
TCAATCTAGAAGTCAT-1	singlet	0	-48.82026725	-142.006952	-144.0496177	EMD2
TCAATCTAGAGGACTC-1	singlet	1	-3100.728273	-1120.374812	-2446.058667	EMD2
TCAATCTAGCAGGCTA-1	singlet	1	-1972.073206	-788.8174572	-1716.707616	EMD2
TCAATCTAGGCTCACC-1	singlet	1	-2162.13824	-730.2144679	-1865.8263	EMD2
TCAATCTCAACATACC-1	singlet	1	-2489.750161	-890.8375397	-2115.571468	EMD2
TCAATCTGTACTCAAC-1	singlet	2	-254.0531155	-183.7851393	-68.54398642	EMD2
TCAATCTTCACACCCT-1	doublet	2/0	-3676.528221	-4615.307397	-2911.798051	EMD2
TCAATCTTCACCGCTT-1	singlet	0	-1041.40678	-2914.998024	-2824.910649	EMD2
TCAATCTTCACTGTTT-1	singlet	0	-2471.631874	-8037.721498	-7925.729238	EMD2
TCAATCTTCCTGGGAC-1	singlet	1	-1462.776935	-489.9252359	-1143.694227	EMD2
TCAATTCAGAATTTGG-1	singlet	1	-944.5548623	-298.9342204	-692.0258045	EMD2
TCAATTCAGCCAGACA-1	singlet	0	-578.8163889	-1671.241368	-1591.456428	EMD2
TCAATTCAGGCTGTAG-1	singlet	1	-3118.9759	-952.9441154	-2181.180689	EMD2
TCAATTCAGTATGGAT-1	doublet	45323	-2483.313685	-1666.993515	-1606.90893	EMD2
TCAATTCAGTTACGTC-1	singlet	0	-932.7401179	-2969.180023	-2839.234409	EMD2
TCAATTCGTATGAAAC-1	singlet	2	-1154.745199	-884.8788236	-396.5519755	EMD2
TCAATTCTCACAAGAA-1	singlet	1	-738.1886711	-256.5746594	-579.8944343	EMD2
TCAATTCTCTGTCTCG-1	singlet	2	-475.2531109	-498.9052119	-218.4543675	EMD2
TCACAAGAGAAGCCTG-1	singlet	0	-394.5806978	-1237.553476	-1049.056026	EMD2
TCACAAGAGAATTCAG-1	singlet	1	-569.2491273	-178.4254415	-385.043669	EMD2
TCACAAGAGAATTGTG-1	singlet	1	-2892.328676	-1073.857854	-2514.688639	EMD2
TCACAAGAGAGATTCA-1	singlet	2	-38.19474307	-49.99725676	-21.75481707	EMD2
TCACAAGAGATCGGTG-1	singlet	0	-1213.098021	-3793.123315	-3587.265962	EMD2
TCACAAGAGCTCCATA-1	singlet	0	-747.2135526	-2457.12583	-2203.922702	EMD2
TCACAAGAGCTCGCAC-1	singlet	1	-1497.323671	-536.1273514	-1225.595636	EMD2
TCACAAGAGTATGTAG-1	unassigned	0/2	-19.12594069	-24.02705205	-27.74361805	EMD2
TCACAAGAGTCTCGTA-1	singlet	1	-321.0484595	-123.9329613	-301.577868	EMD2
TCACAAGGTCGTGGTC-1	doublet	45293	-102.077415	-53.15323731	-99.4619404	EMD2
TCACAAGTCCACCCTA-1	singlet	1	-934.2671719	-312.4913504	-717.9169306	EMD2
TCACAAGTCTATCGCC-1	singlet	1	-2806.609637	-651.3128102	-2206.379547	EMD2
TCACAAGTCTGCGAGC-1	singlet	1	-3789.246391	-1434.261104	-3070.713793	EMD2
TCACACCAGCACTCCG-1	singlet	0	-584.0865995	-1716.951291	-1550.523235	EMD2
TCACACCAGCTGACAG-1	singlet	1	-2188.971099	-690.9960184	-1595.631994	EMD2
TCACACCAGGACTATA-1	singlet	1	-1234.24803	-490.9047458	-1065.572693	EMD2
TCACACCAGGCTTAAA-1	singlet	1	-550.3752393	-226.942223	-508.2276025	EMD2
TCACACCCAAGACAAT-1	singlet	1	-466.4048429	-170.937273	-359.2295249	EMD2
TCACACCCAAGACTGG-1	singlet	1	-2456.003697	-790.1193272	-2267.40125	EMD2
TCACACCCATGTTACG-1	singlet	1	-1858.392851	-598.2682691	-1540.580248	EMD2
TCACACCGTAGTAAGT-1	singlet	1	-439.2191537	-190.9338448	-324.0777865	EMD2
TCACACCGTGGAAATT-1	singlet	0	-645.5465168	-1968.797038	-1984.390857	EMD2
TCACATTCAAGCACAG-1	doublet	0/2	-33.29282427	-60.57773596	-34.66721017	EMD2
TCACATTGTCAAAGTA-1	singlet	2	-577.9299359	-462.8095813	-260.0300354	EMD2
TCACATTGTCAGTCCG-1	singlet	1	-1475.696957	-531.6715186	-1227.129778	EMD2
TCACATTGTGGTCCGT-1	doublet	45323	-4500.300854	-3104.548441	-2579.565809	EMD2
TCACATTGTTAGCGGA-1	singlet	2	-759.9620636	-704.0568101	-251.0428881	EMD2
TCACATTGTTCCGTTC-1	singlet	1	-1369.273395	-418.4828459	-1215.867698	EMD2
TCACATTGTTGCTGAT-1	singlet	2	-407.0261705	-368.8419512	-179.7645136	EMD2
TCACATTTCCTCGATC-1	singlet	1	-272.9838379	-98.52166033	-239.0361267	EMD2
TCACATTTCTACTCAT-1	singlet	2	-5044.991229	-4171.848073	-1439.041206	EMD2
TCACATTTCTAGAGCT-1	singlet	1	-2401.107577	-644.8440322	-1803.260027	EMD2
TCACATTTCTCATTTG-1	singlet	1	-1186.566122	-479.3864972	-913.8237943	EMD2
TCACGCTAGCAAGTGC-1	singlet	1	-610.0823532	-259.8378426	-514.7354324	EMD2
TCACGCTCAAATGAAC-1	singlet	1	-1275.621137	-466.6032276	-990.8143664	EMD2
TCACGCTCACAGTACT-1	singlet	1	-1468.330437	-376.6829107	-1140.370666	EMD2
TCACGCTCATATTCGG-1	doublet	2/0	-1102.92887	-1374.603415	-613.5720313	EMD2
TCACGCTGTACGATCT-1	singlet	1	-414.2840514	-148.2349973	-340.96541	EMD2
TCACGCTGTCGATTAC-1	singlet	2	-1245.366619	-1058.871993	-416.1765032	EMD2
TCACGCTGTTGGGAAC-1	singlet	2	-3036.032645	-3055.490443	-860.384297	EMD2
TCACGCTGTTTCCATT-1	singlet	1	-495.3532351	-198.1625387	-400.7685383	EMD2
TCACGCTTCTTGAGCA-1	singlet	1	-1163.038662	-425.7137706	-988.7462141	EMD2
TCACGGGAGATGGCGT-1	doublet	45323	-520.6580012	-358.6258521	-305.9691084	EMD2
TCACGGGAGCAGATAT-1	singlet	0	-2038.896553	-6247.676527	-5650.278028	EMD2
TCACGGGAGCCGCACT-1	singlet	1	-400.4102832	-143.9651448	-317.6729733	EMD2
TCACGGGAGCCTGAGA-1	singlet	1	-426.5897846	-159.7843379	-373.3060739	EMD2
TCACGGGCAACCTAAC-1	singlet	1	-330.3026021	-127.4244001	-306.5062165	EMD2
TCACGGGCAGCAATTC-1	singlet	1	-3409.847626	-1132.304792	-2908.770681	EMD2
TCACGGGCAGGGAATC-1	singlet	1	-2069.597865	-669.0027964	-1542.805104	EMD2
TCACGGGCATGACTGT-1	singlet	1	-694.1694138	-238.6615619	-512.7725321	EMD2
TCACGGGGTCACAATC-1	singlet	0	-1737.067933	-6544.320693	-6035.917864	EMD2
TCACGGGTCCATGAGT-1	singlet	0	-964.1877252	-2819.142805	-2803.299179	EMD2
TCACGGGTCCGACGGT-1	singlet	1	-2664.113045	-838.4709985	-2118.758618	EMD2
TCACTATAGCGCGTTC-1	singlet	1	-588.1132648	-242.6620877	-456.4545407	EMD2
TCACTATAGCTCAGAG-1	singlet	1	-1617.698096	-600.2410158	-1402.67325	EMD2
TCACTATAGTAGAGTT-1	singlet	1	-3317.249949	-1020.699205	-2563.49965	EMD2
TCACTATCAGAATGTA-1	unassigned	0	-2.302528988	-2.338339097	-10.09459916	EMD2
TCACTATCAGCTGTTA-1	singlet	0	-1903.391329	-5511.144964	-5300.325626	EMD2
TCACTATGTATGGAAT-1	singlet	1	-247.4525651	-116.1939474	-218.0506226	EMD2
TCACTATGTCATAACC-1	unassigned	1	-5.983241092	-2.63181282	-6.166676802	EMD2
TCACTATGTGAGATCG-1	singlet	1	-1774.285205	-614.5793179	-1346.533469	EMD2
TCACTATGTGCAATAA-1	singlet	0	-277.003304	-764.2791416	-705.1423627	EMD2

TCACTATGTGCTCTCT-1	singlet	0	-2168.501451	-7449.612796	-7232.926169	EMD2
TCACTATTCCTTTAGT-1	singlet	1	-1257.662731	-472.4303984	-1035.133619	EMD2
TCACTATTCGAAATCC-1	singlet	1	-2477.925652	-757.55332	-2007.650237	EMD2
TCACTATTCGAACTCA-1	singlet	1	-3634.235652	-1013.117109	-2714.108296	EMD2
TCACTATTCTATTTCG-1	singlet	0	-360.4081901	-1100.23028	-948.1686931	EMD2
TCACTATTCTGGACTA-1	singlet	1	-124.4976569	-53.44902026	-151.2476272	EMD2
TCACTCGAGATGGTAT-1	singlet	1	-322.795727	-95.38646715	-221.8686137	EMD2
TCACTCGCACGACCTG-1	singlet	0	-1191.429568	-3164.068628	-3250.597021	EMD2
TCACTCGGTACAAAGT-1	unassigned	1/0	-8.322747022	-7.363253816	-8.053710193	EMD2
TCACTCGGTCCAAAGG-1	singlet	0	-811.8934631	-2863.843619	-2697.234764	EMD2
TCACTCGGTTAGGACG-1	singlet	0	-65.85606001	-165.3129758	-156.8364408	EMD2
TCACTCGTCTCTGAGA-1	singlet	0	-145.1346754	-351.8838799	-312.7172199	EMD2
TCAGCAAAGAAGGATG-1	singlet	1	-1046.6752	-383.938155	-835.2705432	EMD2
TCAGCAAAGATAGCTA-1	singlet	0	-1004.624095	-2892.185119	-2666.369704	EMD2
TCAGCAAAGCTGAGTG-1	doublet	0/1	-2724.544591	-4365.130241	-5154.914473	EMD2
TCAGCAAAGGCTTAGG-1	singlet	1	-1007.806749	-330.3104202	-778.6272891	EMD2
TCAGCAAAGGGTCTTT-1	singlet	0	-172.7877585	-841.8115004	-685.2071588	EMD2
TCAGCAAAGTCTGGAG-1	singlet	1	-1335.584451	-342.9557538	-1294.710561	EMD2
TCAGCAACACCCTCTA-1	singlet	2	-3558.606333	-3477.779538	-1328.468901	EMD2
TCAGCAACAGCTGGTC-1	singlet	1	-742.522681	-281.3062866	-541.7097386	EMD2
TCAGCAACATGTGTCA-1	singlet	0	-830.9158215	-1555.01372	-1529.331364	EMD2
TCAGCAAGTACCCACG-1	singlet	1	-2141.016582	-722.7316424	-1914.49138	EMD2
TCAGCAAGTATCCCTC-1	singlet	1	-7585.382464	-1859.08329	-5755.578508	EMD2
TCAGCAAGTCAGTCCG-1	singlet	2	-581.1334046	-564.9984112	-205.9860202	EMD2
TCAGCAAGTGTGTGGA-1	singlet	0	-449.4556227	-1459.765693	-1477.946905	EMD2
TCAGCAAGTGTTGCCG-1	singlet	2	-953.4024126	-852.114523	-303.6066478	EMD2
TCAGCAAGTTCCACAA-1	singlet	0	-558.7386346	-1672.475575	-1705.384615	EMD2
TCAGCAATCAAAGAAC-1	singlet	1	-936.7374754	-309.8508601	-872.0471931	EMD2
TCAGCAATCCCATACC-1	singlet	0	-526.2406107	-1389.47956	-1307.459642	EMD2
TCAGCAATCGACCACG-1	singlet	1	-695.8233759	-226.6766149	-572.246906	EMD2
TCAGCAATCGAGAATA-1	singlet	0	-178.6688028	-953.7123914	-651.1240702	EMD2
TCAGCAATCGCTTAAG-1	singlet	1	-3605.949207	-908.8426577	-2761.885799	EMD2
TCAGCCTAGCGGACAT-1	singlet	2	-8.977004285	-12.76724697	-5.241219631	EMD2
TCAGCCTAGGACGGAG-1	singlet	1	-2710.444091	-793.4984675	-2036.060163	EMD2
TCAGCCTCACATACGT-1	singlet	0	-2401.427707	-7736.484541	-7748.375954	EMD2
TCAGCCTCACTCCCTA-1	singlet	1	-3243.40067	-992.9305666	-2707.337835	EMD2
TCAGCCTCACTGGCGT-1	singlet	1	-334.4894557	-159.9998102	-330.2906685	EMD2
TCAGCCTGTCGCTTAA-1	singlet	1	-2598.999869	-843.0766346	-1938.741292	EMD2
TCAGCCTGTGCCTATA-1	singlet	1	-1282.540805	-258.4612018	-708.1093308	EMD2
TCAGCCTGTGTCATTG-1	singlet	1	-2037.503872	-572.6176527	-1592.265848	EMD2
TCAGCCTTCGCTCATC-1	singlet	1	-752.1909578	-314.4224992	-624.6241146	EMD2
TCAGGGCAGCCAGTAG-1	singlet	2	-1062.645769	-918.5444824	-389.6297261	EMD2
TCAGGGCAGGGTTGCA-1	singlet	1	-140.7894263	-37.8558085	-88.78130616	EMD2
TCAGGGCAGTAGTCTC-1	singlet	1	-1323.2874	-562.0606774	-1165.518524	EMD2
TCAGGGCCAAGTATAG-1	singlet	2	-3040.161686	-2361.182313	-1078.149848	EMD2
TCAGGGCCAATGAAAC-1	singlet	1	-1521.517026	-485.2182214	-1290.135122	EMD2
TCAGGGCCAGCTGAAG-1	singlet	0	-343.1629276	-1160.173491	-1070.749738	EMD2
TCAGGGCCATGAGATA-1	singlet	2	-426.2832273	-319.9407831	-143.4018835	EMD2
TCAGGGCCATGGAATA-1	singlet	2	-609.9775303	-557.2113826	-176.2881471	EMD2
TCAGGGCGTATGGAGC-1	singlet	1	-1228.727575	-366.9701134	-941.7450235	EMD2
TCAGGGCGTCCTGTCT-1	singlet	2	-1006.990069	-786.6664682	-351.5114749	EMD2
TCAGGGCGTTCCTAAG-1	singlet	1	-1771.711382	-513.4914459	-1318.422987	EMD2
TCAGGGCTCGAGTCTA-1	singlet	0	-212.846643	-688.9719297	-665.2295578	EMD2
TCAGGGCTCTACGGGC-1	singlet	0	-884.0990567	-2505.550532	-2528.520657	EMD2
TCAGGGCTCTAGTCAG-1	singlet	1	-3128.058893	-1046.311616	-2349.690176	EMD2
TCAGGGCTCTCGGCTT-1	singlet	1	-3009.201762	-811.8442616	-2164.277202	EMD2
TCAGGGCTCTTTCCGG-1	singlet	0	-845.8692748	-2949.423171	-2853.881784	EMD2
TCAGGTAAGGACAAGA-1	singlet	0	-557.3991875	-2106.900554	-1868.114121	EMD2
TCAGGTACACTGTCGG-1	singlet	0	-634.3083229	-1991.504432	-1883.263876	EMD2
TCAGGTACAGATTCGT-1	singlet	0	-59.99135258	-195.0313495	-180.0221121	EMD2
TCAGGTACAGGCATTT-1	doublet	1/0	-161.7678252	-158.2539263	-214.2482869	EMD2
TCAGGTACATCTAACG-1	singlet	1	-2220.65838	-628.6680931	-1785.748731	EMD2
TCAGGTAGTGCTTATG-1	singlet	1	-324.5738205	-110.7397495	-288.2953787	EMD2
TCAGGTAGTGGCAGAT-1	singlet	1	-319.6982279	-140.6120676	-275.9487236	EMD2
TCAGGTAGTTCTTCAT-1	singlet	1	-2309.576784	-671.9785652	-2225.168883	EMD2
TCAGGTATCACCACAA-1	singlet	1	-1173.846724	-394.9088956	-913.2501098	EMD2
TCAGGTATCAGTGCGC-1	singlet	1	-4752.414789	-1567.386789	-4009.241109	EMD2
TCAGGTATCCATATGG-1	singlet	1	-461.8295884	-146.9637949	-339.9532784	EMD2
TCAGGTATCCTTTGAT-1	singlet	2	-649.5022637	-657.9949021	-288.8495725	EMD2
TCAGGTATCGTCAGAT-1	singlet	1	-2668.012034	-800.191499	-2370.48935	EMD2
TCAGTCCCACACCTGG-1	singlet	1	-2338.452097	-812.6050015	-2056.466603	EMD2
TCAGTCCCACATATCG-1	singlet	0	-864.36969	-2453.075394	-2432.986983	EMD2
TCAGTCCCATATAGCC-1	singlet	1	-671.055074	-220.369354	-515.6573837	EMD2
TCAGTCCCATTCGATG-1	singlet	0	-262.2388025	-719.1854344	-699.3179202	EMD2
TCAGTCCGTTCTTGCC-1	singlet	1	-350.0080601	-159.5683789	-305.2365158	EMD2
TCAGTCCTCCTACCGT-1	singlet	1	-519.3355693	-168.3620665	-373.3880215	EMD2
TCAGTGAAGCAATAAC-1	singlet	2	-72.08975376	-47.02217267	-25.29364025	EMD2
TCAGTGAAGCAGATAT-1	singlet	1	-1891.539897	-653.7111752	-1696.302164	EMD2
TCAGTGAAGCGTGCCT-1	singlet	0	-799.1977628	-2027.153201	-2125.860619	EMD2
TCAGTGAAGGTAAGAG-1	doublet	45323	-4456.826792	-2689.837638	-2327.067405	EMD2
TCAGTGAAGTTTCTTC-1	singlet	1	-403.3891202	-149.8751001	-292.429087	EMD2
TCAGTGACAAGTATAG-1	singlet	0	-543.929388	-1554.203895	-1576.610871	EMD2
TCAGTGACACCAGACC-1	singlet	1	-519.2002959	-222.4404586	-543.4462362	EMD2
TCAGTGACACCTGCTT-1	singlet	1	-776.5671499	-287.6755563	-696.8176351	EMD2
TCAGTGACAGTCCGTG-1	singlet	1	-997.4898186	-367.25645	-852.5268166	EMD2
TCAGTGAGTTAAGCAA-1	singlet	1	-312.3502826	-129.7535825	-233.8012335	EMD2
TCAGTGATCAGCTAGT-1	singlet	0	-904.0264489	-2465.16283	-2416.493899	EMD2
TCAGTGATCATTTCGT-1	singlet	1	-294.7649424	-123.4228875	-246.4726848	EMD2
TCAGTGATCCGCAAAT-1	unassigned	2	-4.495469018	-2.040358014	-1.653849143	EMD2
TCAGTGATCTCGCTCA-1	singlet	2	-1421.568154	-1530.321009	-540.144185	EMD2
TCAGTTTAGAGTTCGG-1	singlet	2	-1144.719051	-1150.757477	-313.8419823	EMD2
TCAGTTTAGCGACTGA-1	singlet	2	-784.5708574	-718.2989162	-264.0901332	EMD2
TCAGTTTAGGCACCAA-1	singlet	2	-477.5605382	-430.4883762	-222.6432977	EMD2
TCAGTTTAGGTCCAGA-1	singlet	0	-31.88212261	-90.45057537	-78.92201965	EMD2
TCAGTTTCAAATCGGG-1	singlet	1	-3126.078973	-1044.993733	-2491.727546	EMD2
TCAGTTTCAACACAAA-1	singlet	2	-93.49354177	-64.5107737	-27.62156407	EMD2
TCAGTTTCAATGGGTG-1	singlet	1	-505.1993722	-225.2781069	-423.3150614	EMD2
TCAGTTTCACACCTTC-1	singlet	1	-2143.525281	-820.2181421	-1892.22582	EMD2
TCAGTTTCACCAGGTC-1	singlet	0	-1980.328043	-9106.981776	-9077.397351	EMD2
TCAGTTTCACTCTCGT-1	singlet	1	-1664.72832	-635.5362957	-1396.356431	EMD2
TCAGTTTCACTGATTG-1	unassigned	1	-35.77174535	-20.61953582	-22.2486658	EMD2
TCAGTTTCAGATTAAG-1	singlet	1	-1464.248813	-463.2821231	-1248.121537	EMD2
TCAGTTTCATGTAACC-1	singlet	1	-829.0156566	-285.3597403	-659.3517333	EMD2
TCAGTTTTCATGGAGG-1	singlet	2	-398.9846558	-486.2138296	-181.5428976	EMD2
TCAGTTTTCATGGCCG-1	singlet	1	-527.5871065	-221.8380111	-448.9702815	EMD2
TCAGTTTTCCACCTGT-1	singlet	1	-1185.222696	-455.0921103	-940.1481504	EMD2
TCAGTTTTCCCGAGAC-1	singlet	1	-3882.179837	-1219.541838	-3029.867509	EMD2
TCAGTTTTCCTCTCGA-1	singlet	0	-799.8254705	-2385.665276	-2290.729459	EMD2
TCATACTCAATGGGTG-1	singlet	2	-385.7997963	-338.099395	-158.9789158	EMD2
TCATACTGTACTTCCC-1	singlet	1	-3828.644226	-1217.623048	-2868.262319	EMD2

TCATACTGTCGAGATG-1	doublet	0/1	-2802.108628	-3710.923453	-4515.08976	EMD2
TCATACTTCTCATTAC-1	singlet	0	-1936.260346	-7708.487445	-7273.123273	EMD2
TCATATCAGAAGCCAC-1	doublet	0/1	-324.4861767	-393.8736126	-394.0477806	EMD2
TCATATCAGTAGCATA-1	singlet	2	-1178.67635	-1377.694213	-493.9911303	EMD2
TCATATCAGTGGCAGT-1	singlet	2	-2539.097082	-2072.123186	-625.0470423	EMD2
TCATATCCAGGCTACC-1	singlet	0	-961.0630925	-2872.504238	-2660.698398	EMD2
TCATATCGTCCACACG-1	singlet	0	-820.94909	-2435.265342	-2470.879362	EMD2
TCATATCTCCTGTTAT-1	singlet	1	-2144.672844	-513.2825224	-1819.684963	EMD2
TCATATCTCGTTGTAG-1	singlet	0	-766.1865655	-1831.762391	-1803.519643	EMD2
TCATATCTCTTGCAAG-1	singlet	0	-2693.928398	-8868.153945	-8696.117164	EMD2
TCATCATAGCGTTCCG-1	singlet	1	-197.3763873	-57.97431938	-145.8841713	EMD2
TCATCATCACCTAAAC-1	singlet	1	-212.2497503	-57.14349347	-141.187463	EMD2
TCATCATCACTGATTG-1	singlet	1	-1703.678738	-580.3762992	-1254.685367	EMD2
TCATCATGTTATAGAG-1	singlet	0	-89.13809907	-232.707676	-208.3192182	EMD2
TCATCATTCACGACTA-1	singlet	1	-806.938258	-312.1556648	-690.9423512	EMD2
TCATCATTCATCTCTA-1	singlet	1	-3978.344605	-1115.266992	-3117.93399	EMD2
TCATCATTCGCGTGAC-1	singlet	1	-1972.716447	-644.3963936	-1570.806059	EMD2
TCATCATTCGGACGTC-1	singlet	1	-770.264124	-254.5817714	-678.443942	EMD2
TCATCATTCGTTATCT-1	singlet	2	-5911.909995	-5212.058813	-1752.817211	EMD2
TCATCCGAGCAGCGAT-1	singlet	1	-1113.827375	-400.4808726	-880.850353	EMD2
TCATCCGAGGCATGCA-1	singlet	0	-501.2847536	-1279.645192	-1266.494481	EMD2
TCATCCGAGGGCCCTT-1	singlet	1	-5808.994594	-2464.960153	-5092.820446	EMD2
TCATCCGGTATTCCGA-1	singlet	1	-292.507991	-112.2517062	-254.1293924	EMD2
TCATCCGGTGGATCGA-1	singlet	0	-252.9605265	-549.0386078	-529.9636885	EMD2
TCATCCGGTTAACCTG-1	singlet	1	-4076.381152	-1487.094915	-3675.581731	EMD2
TCATCCGTCAGTCTTT-1	unassigned	1	-1.850459954	-1.134897689	-1.733624898	EMD2
TCATCCGTCCTAGCTC-1	singlet	1	-453.7995756	-168.942467	-336.296749	EMD2
TCATCCGTCGAGATAA-1	doublet	45293	-2072.873719	-1073.07602	-1429.410631	EMD2
TCATCCGTCTAGCCAA-1	singlet	1	-288.8275926	-77.63277976	-178.5345131	EMD2
TCATCCGTCTCAACCC-1	singlet	2	-3173.854802	-2692.115069	-1012.642462	EMD2
TCATGAGAGAGGATCC-1	singlet	2	-2574.831985	-2146.35451	-913.4914442	EMD2
TCATGAGAGATACTGA-1	doublet	1/0	-2365.397337	-1490.482882	-2411.52018	EMD2
TCATGAGCACAACGAG-1	singlet	1	-5097.726741	-1761.194405	-4164.110486	EMD2
TCATGAGCACACCTAA-1	singlet	1	-758.1265301	-296.0369144	-599.3812863	EMD2
TCATGAGCACAGAGAC-1	singlet	1	-2234.578659	-709.049044	-1699.723462	EMD2
TCATGAGTCGACACTA-1	singlet	0	-915.2065849	-2594.680008	-2594.830982	EMD2
TCATGAGTCTCCGATC-1	singlet	1	-1121.957383	-395.6535777	-818.352888	EMD2
TCATGCCAGAATCTAG-1	singlet	1	-3332.201838	-1086.744299	-2564.112424	EMD2
TCATGCCAGAGTTGTA-1	singlet	1	-2050.764853	-700.7665394	-1727.974431	EMD2
TCATGCCAGCGAATGC-1	singlet	1	-949.8066127	-327.9603748	-734.9447087	EMD2
TCATGCCAGCTAAATG-1	singlet	1	-1433.951049	-446.6066486	-1045.229493	EMD2
TCATGCCAGTACCGGA-1	singlet	0	-1381.561622	-4481.670168	-4277.046557	EMD2
TCATGCCCAACGAGGT-1	singlet	1	-944.9354361	-346.1628138	-786.7305384	EMD2
TCATGCCCAATGGCCC-1	singlet	1	-4158.959869	-1312.060675	-3494.449408	EMD2
TCATGCCGTCCAGGTC-1	singlet	2	-117.9531633	-87.71091192	-42.32344184	EMD2
TCATGCCGTGTATTGC-1	singlet	0	-669.29832	-2054.982567	-1999.960254	EMD2
TCATGCCTCAGTGTTG-1	singlet	1	-2355.050881	-693.8745138	-1712.622064	EMD2
TCATGCCTCCTCTTTC-1	singlet	1	-2403.374103	-815.7429963	-2136.570704	EMD2
TCATGCCTCGAGAATA-1	singlet	1	-426.7635798	-133.3090274	-278.2132793	EMD2
TCATGGAAGATACGAT-1	singlet	1	-315.7926232	-124.9561965	-232.9725977	EMD2
TCATGGAAGGAAAGTG-1	unassigned	1	-5.688428951	-2.120397924	-4.476478761	EMD2
TCATGGAAGTATGCAA-1	singlet	1	-3475.031376	-1183.029947	-2414.915745	EMD2
TCATGGACACACTGGC-1	singlet	2	-528.3448912	-550.7693746	-213.2068963	EMD2
TCATGGACAGGGAGAG-1	singlet	1	-1771.528846	-526.497928	-1430.859294	EMD2
TCATGGAGTCAAACGG-1	singlet	1	-397.9239907	-142.9984243	-293.3467874	EMD2
TCATGGAGTCAAAGAT-1	singlet	1	-2176.547573	-773.7090615	-1701.406249	EMD2
TCATGGAGTTGGTAGG-1	doublet	1/0	-300.0121382	-175.1089472	-281.9456777	EMD2
TCATGGATCGGTAACT-1	singlet	2	-5339.623803	-4973.524396	-1974.324412	EMD2
TCATGGATCTATCCAT-1	singlet	1	-971.9400429	-348.5591106	-776.7524545	EMD2
TCATGTTAGAATACAC-1	singlet	1	-2924.541803	-966.0201396	-2392.24988	EMD2
TCATGTTAGCGACTTT-1	unassigned	45323	-11.48956634	-7.722807719	-5.277097315	EMD2
TCATGTTAGCGTATAA-1	singlet	0	-510.4954316	-1472.513493	-1378.882075	EMD2
TCATGTTAGGTTCACT-1	singlet	1	-223.0544395	-73.99171558	-177.4435326	EMD2
TCATGTTAGTTGGAGC-1	singlet	1	-1650.552492	-573.3683149	-1233.137676	EMD2
TCATGTTCATGGAAGC-1	singlet	1	-289.5698615	-117.4189203	-228.1246909	EMD2
TCATGTTGTTCGTGCG-1	singlet	2	-1684.403559	-1386.009286	-527.9004751	EMD2
TCATGTTTCAAAGGAT-1	singlet	1	-132.9193628	-47.71503424	-110.255202	EMD2
TCATGTTTCACTACGA-1	singlet	0	-221.5227317	-657.6882017	-580.3670328	EMD2
TCATTACCACAGCCTG-1	singlet	2	-1705.499946	-1420.214452	-561.854118	EMD2
TCATTACCAGCTACCG-1	doublet	0/2	-1614.515548	-2828.704498	-2394.49902	EMD2
TCATTACGTAATTGGA-1	singlet	1	-2254.236556	-716.9093123	-1801.977482	EMD2
TCATTACGTACCTAAC-1	singlet	0	-1043.365315	-3266.303696	-2915.083286	EMD2
TCATTACGTTCCACAA-1	singlet	1	-649.2930274	-239.3877372	-545.9986025	EMD2
TCATTACGTTCTGAGT-1	singlet	1	-1500.39557	-392.0835926	-1183.338653	EMD2
TCATTACTCAACCTCC-1	singlet	1	-686.5286098	-250.2530932	-574.7545978	EMD2
TCATTACTCACTTCTA-1	singlet	1	-340.4216745	-117.5422386	-266.7503776	EMD2
TCATTACTCTGCTCTG-1	singlet	0	-698.7249542	-2312.182409	-2187.001304	EMD2
TCATTCAAGAGCAAGA-1	singlet	1	-290.7018764	-117.8423567	-304.3601085	EMD2
TCATTCAAGATTCGAA-1	singlet	2	-560.1130364	-493.9953202	-226.0139909	EMD2
TCATTCACAACCACGC-1	singlet	1	-2502.633357	-798.1730947	-2523.622486	EMD2
TCATTCACACATGTTG-1	singlet	1	-98.50575359	-44.77143908	-99.30151044	EMD2
TCATTCACAGCGTTGC-1	singlet	1	-2600.223772	-816.208203	-1980.961443	EMD2
TCATTCAGTAGTGGCA-1	singlet	0	-32.67525349	-90.66079139	-86.06708757	EMD2
TCATTCAGTCCCTGAG-1	singlet	0	-201.1718832	-618.1387105	-603.2332502	EMD2
TCATTCAGTCGGATTT-1	singlet	2	-539.7181272	-563.7052978	-179.5597664	EMD2
TCATTCAGTGATAGTA-1	singlet	1	-2432.826907	-794.4590052	-1888.995181	EMD2
TCATTCATCAGATGCT-1	singlet	1	-804.3850525	-323.2934212	-745.0421198	EMD2
TCATTCATCCGTATGA-1	singlet	0	-846.8618177	-2920.542482	-2718.210411	EMD2
TCATTCATCGCTGTCT-1	singlet	1	-255.5733709	-101.4682306	-183.5610607	EMD2
TCATTCATCTAACGCA-1	singlet	2	-2872.333233	-2932.931565	-1001.662768	EMD2
TCATTCATCTAGAACC-1	singlet	0	-1369.042651	-4005.470399	-3678.094094	EMD2
TCATTCATCTCTCCGA-1	singlet	1	-4062.81523	-1339.840761	-3376.518752	EMD2
TCATTGTAGATTAGTG-1	doublet	0/2	-2006.251991	-2487.301185	-2216.048224	EMD2
TCATTGTAGCATCCTA-1	singlet	1	-277.5087219	-122.0174637	-230.1351621	EMD2
TCATTGTCATGTGACT-1	singlet	0	-121.9667052	-474.6904852	-321.1733505	EMD2
TCATTGTGTGATGTAA-1	singlet	1	-562.3648224	-231.546874	-494.4979647	EMD2
TCATTGTGTGATTCAC-1	singlet	2	-3038.610522	-2342.606016	-885.6973931	EMD2
TCATTGTGTTCAGTAC-1	singlet	1	-2306.122552	-666.5375876	-2109.40931	EMD2
TCATTGTGTTCCCACT-1	singlet	1	-197.2384383	-49.40950687	-168.5173781	EMD2
TCATTGTGTTGAATCC-1	doublet	45323	-812.7086257	-648.2222375	-513.9324353	EMD2
TCATTGTGTTTACACG-1	singlet	2	-4341.865742	-5158.845063	-1232.638599	EMD2
TCATTGTGTTTCGCTC-1	singlet	2	-412.6178717	-410.0459842	-161.8632098	EMD2
TCATTGTTCCCTCGAT-1	doublet	45293	-6116.978096	-3370.715595	-3450.495303	EMD2
TCATTGTTCCTACAAG-1	singlet	0	-922.1340853	-3148.730091	-2820.18126	EMD2
TCATTGTTCCTTATGT-1	singlet	1	-1451.369989	-517.745273	-1219.072089	EMD2
TCATTGTTCGAATCCA-1	singlet	1	-1472.355214	-532.6130213	-1198.746218	EMD2
TCATTTGAGAGAAGGT-1	singlet	0	-1177.0886	-3910.021445	-3708.327322	EMD2
TCATTTGAGCAGTACG-1	singlet	0	-2555.781999	-7383.109887	-7567.861499	EMD2
TCATTTGAGGATTACA-1	singlet	1	-2675.691652	-917.1310904	-2217.526351	EMD2
TCATTTGAGGGCAAGG-1	singlet	2	-780.1611348	-726.6926217	-225.4811391	EMD2

TCATTTGCAACACGTT-1	singlet	1	-1354.736052	-426.1301254	-1029.792069	EMD2
TCATTTGGTATGAAAC-1	singlet	1	-375.055358	-167.6655197	-342.9765549	EMD2
TCATTTGGTTGTGTTG-1	doublet	1/0	-18.78451917	-14.13131799	-10.74621236	EMD2
TCATTTGTCACAACCA-1	singlet	1	-4651.588793	-1405.163639	-4118.023994	EMD2
TCATTTGTCCATTGCC-1	singlet	1	-430.1295668	-165.0651269	-359.8920108	EMD2
TCATTTGTCTCGTTTA-1	singlet	1	-1698.274071	-677.531387	-1562.466474	EMD2
TCATTTGTCTGATGGT-1	singlet	1	-7768.315986	-2391.094569	-6427.425266	EMD2
TCCACCAAGCATGCGA-1	singlet	2	-2358.388529	-2248.372359	-726.2109543	EMD2
TCCACCAAGCGAGGAG-1	singlet	2	-2176.045692	-1970.611757	-760.4839431	EMD2
TCCACCACAAGTGTCT-1	singlet	1	-731.2348662	-258.5270088	-682.4171393	EMD2
TCCACCACAGTAGAGC-1	singlet	1	-3608.54905	-1076.778242	-2874.679822	EMD2
TCCACCACAGTTTCGA-1	singlet	1	-1356.691504	-378.0123717	-924.2131083	EMD2
TCCACCAGTCGTTATG-1	singlet	2	-6476.057771	-4996.545559	-1600.7447	EMD2
TCCACCATCCATTTCA-1	singlet	1	-888.3921413	-325.2502523	-679.5204091	EMD2
TCCACCATCCGCGATG-1	singlet	2	-193.4406809	-157.4932304	-68.82978161	EMD2
TCCACCATCGAATCCA-1	singlet	1	-1794.155906	-595.3877681	-1486.34201	EMD2
TCCACCATCTATCGGA-1	singlet	1	-1465.735982	-498.2553616	-1252.131577	EMD2
TCCACCATCTGGGTCG-1	singlet	1	-426.2464688	-157.1041156	-386.4554075	EMD2
TCCACGTAGATACAGT-1	singlet	1	-4328.889247	-1344.732316	-3568.084182	EMD2
TCCACGTAGCCAGAGT-1	singlet	2	-404.1369485	-397.2900725	-159.3901845	EMD2
TCCACGTAGTGTTCCA-1	singlet	1	-188.9336542	-74.66610145	-158.1616659	EMD2
TCCACGTCAGAACTAA-1	singlet	1	-827.3396347	-298.6346844	-645.0596773	EMD2
TCCACGTCATATGAAG-1	singlet	1	-522.6745315	-207.0310299	-498.8416684	EMD2
TCCACGTCATTCCTAT-1	singlet	1	-987.9702453	-397.9248909	-817.3389587	EMD2
TCCACGTGTACCGTGC-1	singlet	0	-619.1883377	-1497.265606	-1494.593508	EMD2
TCCACGTGTCCTGGGT-1	singlet	1	-2003.009532	-485.5167382	-1678.063358	EMD2
TCCACGTGTTGTTTGG-1	singlet	1	-441.4496672	-171.7767468	-315.6067857	EMD2
TCCACGTTCACACCGG-1	singlet	2	-703.0891668	-677.6244899	-238.7605968	EMD2
TCCACGTTCACCATCC-1	singlet	1	-4157.014445	-1259.574001	-3096.569914	EMD2
TCCACGTTCAGTCATG-1	singlet	1	-516.0888959	-216.7520885	-436.7284547	EMD2
TCCAGAAAGGGTTAAT-1	singlet	0	-420.5240651	-1163.103734	-956.0089053	EMD2
TCCAGAACAGAATTCC-1	singlet	2	-109.6709113	-73.21381183	-30.59919602	EMD2
TCCAGAACAGTTGTTG-1	singlet	1	-1597.430196	-594.3565004	-1398.342866	EMD2
TCCAGAACAGTTTCAG-1	singlet	1	-717.3840839	-250.6994048	-593.1836035	EMD2
TCCAGAACATCACGGC-1	singlet	1	-1101.300038	-372.5633649	-904.3779484	EMD2
TCCAGAACATCCTTGC-1	singlet	2	-930.3458682	-856.8377731	-389.808836	EMD2
TCCAGAAGTCGAACAG-1	singlet	0	-25.79428232	-63.54547532	-67.84065604	EMD2
TCCAGAAGTGGACCTC-1	singlet	1	-381.5827329	-152.0984598	-312.057932	EMD2
TCCAGAAGTGGATCAG-1	singlet	1	-1362.012188	-552.6385563	-1108.070075	EMD2
TCCAGAAGTGTAGGAC-1	singlet	0	-479.424286	-1636.127374	-1321.361477	EMD2
TCCAGAAGTTCAGCGC-1	singlet	1	-1752.688174	-519.4003773	-1508.738505	EMD2
TCCAGAATCGGATACT-1	singlet	1	-1052.067276	-372.2082174	-889.9545524	EMD2
TCCATCGAGAGAGGGC-1	singlet	1	-1977.128047	-638.1174783	-1625.127813	EMD2
TCCATCGAGGAGACCT-1	singlet	1	-1516.963628	-621.7615968	-1376.72137	EMD2
TCCATCGAGTGTAGTA-1	singlet	2	-1086.478563	-931.920026	-355.1178284	EMD2
TCCATCGCACATGACT-1	doublet	45293	-4422.9317	-2011.18739	-2912.955332	EMD2
TCCATCGCAGCGTTTA-1	singlet	0	-132.0147479	-555.4196183	-378.3684715	EMD2
TCCATCGCAGCTGTCG-1	singlet	1	-137.542235	-47.35706377	-138.6503417	EMD2
TCCATCGCATAACGGG-1	singlet	1	-5330.884222	-1520.365962	-4276.934802	EMD2
TCCATCGCATACAGAA-1	singlet	2	-10237.69494	-7626.301083	-1836.856616	EMD2
TCCATCGGTCGGAAAC-1	singlet	2	-1088.094136	-1012.028981	-348.0745447	EMD2
TCCATCGGTCGGTGAA-1	singlet	1	-200.5959591	-82.10526549	-165.5563758	EMD2
TCCATCGGTTCCTAGA-1	singlet	1	-230.1096743	-93.79715478	-203.2717658	EMD2
TCCATCGTCAGGAAGC-1	singlet	1	-2313.87259	-729.3879171	-1805.556135	EMD2
TCCATCGTCGCCAGAC-1	singlet	1	-3894.24036	-1223.672771	-3108.33885	EMD2
TCCATCGTCGGCGATC-1	singlet	0	-344.0625514	-962.8959283	-891.757833	EMD2
TCCATCGTCTAGCCTC-1	singlet	1	-2878.082819	-788.2832755	-2176.042599	EMD2
TCCATGCAGACTTAAG-1	singlet	1	-417.8699169	-177.8654654	-364.6596438	EMD2
TCCATGCAGGTAGCAC-1	singlet	1	-1977.485254	-600.1859048	-1530.439804	EMD2
TCCATGCCACTGGCGT-1	singlet	1	-4042.175023	-1281.497401	-3307.321116	EMD2
TCCATGCTCCACTAGA-1	singlet	1	-3843.236846	-1175.078565	-3122.853678	EMD2
TCCATGCTCGATGCTA-1	singlet	1	-1325.886233	-481.2005034	-1084.07657	EMD2
TCCCACACAATCGCAT-1	singlet	1	-1717.938781	-696.5540282	-1553.892875	EMD2
TCCCACACACGCTGTG-1	singlet	1	-156.9046158	-69.60603533	-147.7208	EMD2
TCCCACATCGCAGAGA-1	singlet	1	-81.88886056	-43.17852082	-80.26227945	EMD2
TCCCACATCTCCTGTG-1	singlet	0	-279.6125548	-760.4646627	-704.3099891	EMD2
TCCCAGTAGAAGATCT-1	singlet	1	-241.5311035	-76.95893576	-184.7743927	EMD2
TCCCAGTAGAGACAAG-1	singlet	1	-1441.094689	-419.045277	-1043.078528	EMD2
TCCCAGTAGATCGCTT-1	singlet	2	-5966.079073	-4676.089359	-1820.224845	EMD2
TCCCAGTAGGATAATC-1	singlet	1	-348.6503366	-140.7880471	-275.0715578	EMD2
TCCCAGTAGTTGGCGA-1	singlet	1	-1905.511442	-592.5154949	-1323.556496	EMD2
TCCCAGTCAATTGCGT-1	singlet	0	-111.3459643	-362.2038774	-269.8205512	EMD2
TCCCAGTCACAAAGCG-1	singlet	2	-507.0979319	-557.3307018	-208.7430784	EMD2
TCCCAGTCACAGCTGC-1	singlet	2	-177.7163953	-145.0474318	-75.26516647	EMD2
TCCCAGTCAGTCGGTC-1	singlet	0	-127.9006773	-479.4550446	-408.8172101	EMD2
TCCCAGTGTAACATGA-1	singlet	1	-2157.084854	-828.1749208	-1670.307384	EMD2
TCCCATGAGCAAGTCG-1	singlet	1	-2734.617113	-1038.875407	-2351.639368	EMD2
TCCCATGAGCACCGAA-1	singlet	1	-221.0404483	-108.2237513	-205.2689797	EMD2
TCCCATGAGGACAGCT-1	singlet	2	-537.5486653	-554.7326571	-228.2457955	EMD2
TCCCATGAGGGATCAC-1	singlet	0	-632.7993123	-2192.688579	-2021.51505	EMD2
TCCCATGCACGGCGTT-1	singlet	2	-758.8836288	-607.2641392	-263.0651214	EMD2
TCCCATGCAGCATGCC-1	doublet	1/0	-5066.148522	-3881.533872	-5775.122473	EMD2
TCCCATGCAGGACTTT-1	singlet	1	-2032.464635	-617.9391034	-1575.215142	EMD2
TCCCATGGTCGTCGGT-1	singlet	2	-743.3206752	-606.7278103	-175.1877641	EMD2
TCCCATGTCAGACATC-1	singlet	1	-247.8525735	-83.20201533	-187.7055846	EMD2
TCCGAAAAGAAACTCA-1	singlet	0	-1272.850132	-4162.735165	-4111.616578	EMD2
TCCGAAAAGCATGATA-1	singlet	0	-171.0780444	-758.0377814	-606.0090887	EMD2
TCCGAAAAGTATGATG-1	singlet	0	-406.8777776	-1080.223345	-929.2234269	EMD2
TCCGAAAAGTTTCAGC-1	singlet	1	-1121.941398	-369.0153095	-979.4938695	EMD2
TCCGAAACACACCAGC-1	singlet	0	-1467.871061	-4366.037517	-4115.082251	EMD2
TCCGAAACACCTGATA-1	singlet	1	-1005.289102	-435.8214856	-925.5965674	EMD2
TCCGAAAGTCCGGTCA-1	singlet	1	-1668.897904	-637.799938	-1359.541051	EMD2
TCCGAAATCATCTGTT-1	singlet	1	-470.1222556	-204.4697948	-381.5128671	EMD2
TCCGATCAGAATTTGG-1	singlet	2	-2467.79547	-1921.436073	-803.7878981	EMD2
TCCGATCAGCAATTAG-1	singlet	2	-955.3298891	-861.877811	-281.2110047	EMD2
TCCGATCAGCATTTGC-1	singlet	1	-5816.597834	-1782.215832	-4788.705743	EMD2
TCCGATCAGTTTGTCG-1	singlet	1	-2128.928514	-649.8608352	-1743.313383	EMD2
TCCGATCGTTCTCCTG-1	singlet	1	-765.1438396	-231.5683762	-616.0848309	EMD2
TCCGATCTCCGTGGCA-1	singlet	2	-436.0745674	-404.9127339	-173.4120047	EMD2
TCCGATCTCCTTATAC-1	singlet	1	-2542.415524	-710.1398897	-1882.719581	EMD2
TCCGATCTCGCATAGT-1	singlet	1	-119.5443464	-35.49868847	-89.83548417	EMD2
TCCGATCTCGCTAGCG-1	singlet	1	-362.5015042	-119.6558689	-298.266197	EMD2
TCCGATCTCTTGGATG-1	singlet	1	-1939.04126	-589.9548687	-1572.237818	EMD2
TCCGATCTCTTTCGAT-1	singlet	0	-968.2472721	-2924.260575	-2878.069885	EMD2
TCCGGGAAGAGCGACT-1	singlet	1	-472.3876594	-151.674308	-358.4476311	EMD2
TCCGGGAAGCGTGTTT-1	singlet	0	-316.7517031	-904.7945315	-901.217763	EMD2
TCCGGGAAGTATGGCG-1	singlet	2	-3160.994244	-2651.113324	-998.4530624	EMD2
TCCGGGACAAAGGAGA-1	singlet	0	-140.0450387	-285.7802252	-320.0818678	EMD2
TCCGGGAGTAGTGATA-1	doublet	1/0	-4106.344969	-3943.190348	-5651.377497	EMD2
TCCGGGAGTGATAGTA-1	singlet	2	-297.9611076	-298.048064	-161.0942518	EMD2

TCCGGGATCACCTCTG-1	singlet	1	-3506.87981	-1132.090117	-2873.662295	EMD2
TCCGGGATCATTCCTA-1	singlet	1	-357.6349267	-137.1127721	-297.6645716	EMD2
TCCGGGATCCGTAGGC-1	singlet	0	-217.4966727	-713.3724407	-614.4001646	EMD2
TCCGGGATCGGTGTAT-1	singlet	1	-1679.280046	-469.0179956	-1454.417947	EMD2
TCCGTGTCAAGCTCTA-1	singlet	1	-275.6843276	-113.1846168	-211.4562581	EMD2
TCCGTGTCAGAGGCAT-1	singlet	0	-613.9829807	-1870.146673	-1910.745079	EMD2
TCCGTGTGTGGCAACA-1	singlet	1	-533.5190521	-219.7919305	-486.0864665	EMD2
TCCGTGTGTGGTGATG-1	singlet	0	-473.8568509	-1654.084785	-1601.025942	EMD2
TCCGTGTGTTCCACGG-1	singlet	0	-899.2710711	-1726.024305	-1831.900078	EMD2
TCCGTGTGTTGAGTCT-1	singlet	2	-5213.744757	-4302.227854	-1631.613814	EMD2
TCCGTGTTCCCTCTAG-1	singlet	1	-964.7847908	-241.9675876	-759.0603348	EMD2
TCCTAATAGACCCGCT-1	singlet	2	-4328.73684	-3915.94293	-1610.596763	EMD2
TCCTAATAGATGGCAC-1	singlet	0	-729.2507428	-1926.444122	-1834.649136	EMD2
TCCTAATAGTAGTCCT-1	singlet	1	-2701.321874	-870.1023059	-2196.571	EMD2
TCCTAATCAAGCAGGT-1	singlet	0	-19.97866277	-75.85141761	-71.45636854	EMD2
TCCTAATCACGGTGCT-1	singlet	2	-571.5651169	-570.7478316	-274.1374314	EMD2
TCCTAATCACGTGAGA-1	singlet	1	-539.7873495	-220.5308993	-512.3081862	EMD2
TCCTAATCAGCTTCGG-1	singlet	1	-254.0315434	-111.0503211	-208.7462393	EMD2
TCCTAATGTCACAATC-1	singlet	0	-600.4028169	-1630.146102	-1466.190741	EMD2
TCCTAATGTCGGCTAC-1	singlet	1	-1234.98587	-416.2965078	-936.1793224	EMD2
TCCTAATGTTGTTTGG-1	singlet	1	-4876.696422	-1455.656941	-3727.932846	EMD2
TCCTAATTCAGGAGAC-1	singlet	1	-493.3847919	-207.4125918	-495.6203608	EMD2
TCCTAATTCGGTAGGA-1	singlet	1	-1766.727273	-567.8524008	-1454.289074	EMD2
TCCTAATTCTTGGTCC-1	singlet	2	-558.3723045	-511.9569619	-225.867232	EMD2
TCCTCCCAGAGCAAGA-1	singlet	1	-871.2705067	-244.0749705	-666.2854458	EMD2
TCCTCCCAGCGACAGT-1	singlet	1	-692.5301282	-212.4850767	-504.408143	EMD2
TCCTCCCCAACGCATT-1	singlet	1	-743.7724716	-222.6237872	-547.5692482	EMD2
TCCTCCCCACCAACAT-1	singlet	2	-3669.825932	-3050.459306	-1365.444269	EMD2
TCCTCCCCACGACAAG-1	singlet	1	-1852.620251	-582.8527739	-1323.20868	EMD2
TCCTCCCCACGCTGTG-1	singlet	1	-313.5654573	-108.2470581	-233.2234551	EMD2
TCCTCCCGTAGACGTG-1	singlet	0	-124.6553978	-376.7642461	-367.6913648	EMD2
TCCTCCCGTTAGGCTT-1	singlet	1	-2362.339026	-855.3885538	-1890.228222	EMD2
TCCTCCCTCCAGCCTT-1	singlet	1	-1630.171668	-509.4869631	-1209.579717	EMD2
TCCTCCCTCGCAAGAG-1	singlet	2	-3065.559537	-2643.458647	-1043.961747	EMD2
TCCTCCCTCTGTCAGA-1	singlet	1	-553.7104128	-210.1296332	-483.1166505	EMD2
TCCTCGAAGAAATCCA-1	singlet	1	-252.5844185	-123.9664601	-255.5384235	EMD2
TCCTCGAAGTCAATCC-1	singlet	0	-466.6575866	-960.4514569	-1000.243592	EMD2
TCCTCGACACAACATC-1	singlet	1	-1213.47265	-460.6968044	-1105.506936	EMD2
TCCTCGACAGAACCGA-1	singlet	0	-1607.97045	-3595.186549	-3780.612878	EMD2
TCCTCGAGTTCGGGTC-1	doublet	2/0	-1427.38206	-1945.56376	-1314.518142	EMD2
TCCTCGATCGCTAATG-1	singlet	1	-2243.169572	-699.1461323	-2005.009997	EMD2
TCCTCGATCGCTATTT-1	singlet	1	-1369.928291	-511.3335413	-1108.10748	EMD2
TCCTCGATCGTCGCTT-1	singlet	1	-1186.593724	-341.9022744	-919.2059951	EMD2
TCCTCGATCTATCGCC-1	singlet	1	-2269.174974	-826.2038826	-1888.095092	EMD2
TCCTCGATCTCCCATG-1	singlet	1	-864.3575046	-239.5829238	-733.9767612	EMD2
TCCTCGATCTGAACGT-1	singlet	1	-1662.735787	-532.9310239	-1444.996572	EMD2
TCCTCTTAGAAGGATG-1	singlet	1	-1308.605291	-348.4332185	-1032.819855	EMD2
TCCTCTTAGCCGCACT-1	singlet	2	-5665.249288	-4938.250051	-1677.819277	EMD2
TCCTCTTAGCCTTGAT-1	singlet	1	-6664.65173	-2085.527401	-4889.451595	EMD2
TCCTCTTCAAGAAATC-1	singlet	2	-3201.398307	-2889.2466	-766.4268503	EMD2
TCCTCTTCAGACATCT-1	singlet	1	-6019.963534	-2178.224504	-5075.643423	EMD2
TCCTCTTCAGGTTCAT-1	singlet	1	-438.7727281	-203.3953445	-434.9863648	EMD2
TCCTCTTCATTGTCGA-1	singlet	0	-2048.851537	-5913.794257	-5743.04297	EMD2
TCCTCTTGTAGGTCAG-1	singlet	1	-11.06697643	-1.644431043	-8.647118547	EMD2
TCCTCTTGTGGATCAG-1	singlet	0	-980.1076017	-3214.76066	-2887.357104	EMD2
TCCTCTTGTTGTGTTG-1	singlet	1	-2948.374832	-919.2077091	-2191.71784	EMD2
TCCTCTTTCCTTGAAG-1	singlet	1	-312.3665175	-105.551241	-292.0063456	EMD2
TCCTCTTTCGAGATAA-1	singlet	1	-3788.314428	-1201.227351	-2886.735353	EMD2
TCCTCTTTCGGACTTA-1	singlet	0	-609.3733237	-1633.111756	-1722.101164	EMD2
TCCTGCAAGTATTAGG-1	singlet	2	-1584.530178	-1650.153408	-580.1714493	EMD2
TCCTGCACAAGAGCTG-1	singlet	0	-170.2245153	-518.9322373	-535.2674314	EMD2
TCCTGCACAAGTCCCG-1	singlet	1	-2963.015874	-1007.40352	-2479.658468	EMD2
TCCTGCACACATACTG-1	singlet	1	-3942.569121	-1166.204569	-2937.364078	EMD2
TCCTGCACACATATGC-1	singlet	0	-1112.836819	-2786.807115	-2804.129839	EMD2
TCCTGCACAGCCGTCA-1	singlet	1	-427.5630403	-179.3896207	-338.8550241	EMD2
TCCTGCAGTACCCACG-1	singlet	1	-527.9733193	-165.3207434	-405.4191246	EMD2
TCCTGCAGTATCGCTA-1	singlet	1	-4032.390462	-1293.734462	-3223.82801	EMD2
TCCTGCAGTGAATTAG-1	singlet	0	-518.7109256	-1513.468658	-1445.314879	EMD2
TCCTGCAGTGGCTCTG-1	singlet	0	-1522.674988	-2774.866006	-3139.748551	EMD2
TCCTGCATCCGCAGTG-1	singlet	2	-65.87165329	-71.80216329	-31.57885409	EMD2
TCCTGCATCGCTCTCA-1	singlet	1	-296.6938396	-130.8077702	-249.87497	EMD2
TCCTGCATCTAGCCTC-1	singlet	2	-3294.423989	-2861.966379	-1321.356152	EMD2
TCCTGCATCTATGTGG-1	singlet	0	-205.4774745	-557.2458977	-458.3452812	EMD2
TCCTTCTAGCACGGAT-1	singlet	1	-1742.211567	-562.4873329	-1368.093522	EMD2
TCCTTCTAGCTGTGCC-1	singlet	0	-356.0313938	-933.6973471	-958.5475006	EMD2
TCCTTCTAGGCCCACT-1	singlet	2	-13092.55736	-10543.21403	-4164.355	EMD2
TCCTTCTAGGCTAAAT-1	singlet	1	-5047.241774	-1152.486901	-3830.232447	EMD2
TCCTTCTAGGCTGAAC-1	singlet	2	-958.5622129	-978.1177431	-424.1499996	EMD2
TCCTTCTCAACTCATG-1	unassigned	2	-3.050245362	-1.354832056	-1.274225529	EMD2
TCCTTCTCACGCTGCA-1	doublet	45323	-5059.828069	-3462.465013	-2978.699104	EMD2
TCCTTCTCACTGCATA-1	singlet	2	-355.059369	-345.7992015	-148.5648717	EMD2
TCCTTCTCAGTTTCGA-1	singlet	2	-1264.756737	-1271.259855	-471.7977702	EMD2
TCCTTCTCATAGGCGA-1	singlet	1	-279.2428583	-121.0290913	-235.1643485	EMD2
TCCTTCTCATTCGATG-1	singlet	2	-1093.553098	-1028.084617	-473.7843035	EMD2
TCCTTCTGTACCAGAG-1	singlet	1	-1153.290488	-440.6032663	-1053.380796	EMD2
TCCTTCTGTTCAATCG-1	unassigned	1	-13.02082074	-2.073537905	-4.044582311	EMD2
TCCTTCTGTTCTCCCA-1	singlet	0	-258.4489056	-663.9159485	-644.8863482	EMD2
TCCTTCTTCGGAATTC-1	singlet	2	-568.8875979	-511.6432831	-185.6853012	EMD2
TCCTTCTTCGTGGACC-1	singlet	2	-3443.792558	-3005.596306	-1198.700535	EMD2
TCCTTTCAGGAACGCT-1	singlet	1	-3226.229159	-1122.938278	-2567.514692	EMD2
TCCTTTCAGGAGAGTA-1	singlet	1	-2675.691988	-914.4065353	-2098.208725	EMD2
TCCTTTCAGGCATGCA-1	singlet	2	-2232.787509	-1741.044784	-705.750856	EMD2
TCCTTTCAGTCACGAG-1	singlet	2	-2392.763861	-1947.730358	-643.9507527	EMD2
TCCTTTCCAATTTCCT-1	singlet	0	-245.7651571	-577.4007198	-519.9681822	EMD2
TCCTTTCCACAAATGA-1	singlet	2	-234.7705884	-173.8808469	-60.37777251	EMD2
TCCTTTCCACTACCGG-1	singlet	0	-368.1553568	-1037.201117	-1052.196592	EMD2
TCCTTTCGTACGGTTT-1	singlet	1	-505.2738496	-218.9785003	-564.6112804	EMD2
TCCTTTCGTCGCTGCA-1	singlet	0	-1470.176439	-4069.97193	-3724.82856	EMD2
TCCTTTCGTGTCCATA-1	singlet	1	-264.5091477	-74.54212391	-278.6021889	EMD2
TCCTTTCGTGTCTCCT-1	singlet	1	-1008.480891	-221.9916488	-645.7066316	EMD2
TCCTTTCGTGTTGATC-1	singlet	1	-991.0551746	-371.6260002	-902.0231582	EMD2
TCCTTTCGTTATGACC-1	singlet	1	-731.248151	-276.2613345	-664.8497732	EMD2
TCCTTTCGTTGCGGCT-1	singlet	0	-408.863811	-1224.152744	-1242.322606	EMD2
TCCTTTCTCGTTAGAC-1	singlet	0	-59.48267912	-170.8769969	-145.448986	EMD2
TCCTTTCTCTGGCCAG-1	singlet	2	-1338.964596	-1034.768372	-431.2881866	EMD2
TCGAACAAGAGTTGTA-1	singlet	0	-1366.282607	-5512.374917	-5354.325812	EMD2
TCGAACAAGTAGGTTA-1	doublet	45293	-41.75227502	-23.18811318	-36.99745171	EMD2
TCGAACAAGTTGCTGT-1	singlet	2	-314.4805754	-288.7785409	-109.9613989	EMD2
TCGAACACACCACATA-1	singlet	1	-2275.100448	-653.7473315	-1996.321844	EMD2
TCGAACAGTACAAAGT-1	singlet	2	-455.7639554	-292.0006464	-108.642073	EMD2

TCGAACAGTCACGACC-1	singlet	1	-624.5973097	-230.1786317	-459.7644156	EMD2
TCGAACAGTCGCGTTG-1	singlet	1	-1375.063655	-391.88623	-1010.762389	EMD2
TCGAACAGTGACCGTC-1	singlet	1	-1704.375924	-419.3153383	-1260.782405	EMD2
TCGAACATCAACTGGT-1	singlet	2	-1727.591187	-1550.954756	-553.5099071	EMD2
TCGAACATCTGGGTCG-1	singlet	2	-1799.006303	-1695.068774	-612.0619664	EMD2
TCGAAGTAGAGCAACC-1	singlet	2	-1012.838179	-933.5843211	-367.4445502	EMD2
TCGAAGTAGTAAATGC-1	singlet	1	-2668.958327	-817.2396899	-2212.308204	EMD2
TCGAAGTCACACCAGC-1	singlet	2	-3641.471738	-2908.920798	-1153.532988	EMD2
TCGAAGTCACCGCTGA-1	singlet	2	-691.6004133	-811.0533005	-225.7914977	EMD2
TCGAAGTCATAGAAAC-1	singlet	2	-3482.957008	-3009.034495	-1132.492657	EMD2
TCGAAGTCATTCTCCG-1	singlet	0	-5.934577466	-16.34225736	-21.77255762	EMD2
TCGAAGTGTAGCGCTC-1	singlet	1	-49.31715603	-27.08224008	-76.46603823	EMD2
TCGAAGTGTATTGACC-1	singlet	0	-752.0376478	-2302.507387	-2263.561622	EMD2
TCGAAGTGTCATAACC-1	singlet	0	-202.5176506	-666.1481814	-598.9473156	EMD2
TCGAAGTTCCTACTGC-1	singlet	2	-612.8451546	-556.5234813	-259.2033179	EMD2
TCGAAGTTCGAACACT-1	singlet	2	-2442.953355	-1893.098663	-783.2237197	EMD2
TCGAAGTTCGCAGTTA-1	singlet	1	-175.0099159	-75.75223489	-157.0341837	EMD2
TCGAAGTTCTGAGGCC-1	singlet	0	-972.5652962	-3507.200616	-3116.592955	EMD2
TCGACCTAGCGACATG-1	singlet	2	-180.6533867	-131.2674775	-59.95177751	EMD2
TCGACCTCAATAGTGA-1	singlet	2	-4028.192787	-3247.333951	-1212.863609	EMD2
TCGACCTCACAGTGAG-1	singlet	0	-80.64664904	-207.6561595	-194.739425	EMD2
TCGACCTCAGAAGTTA-1	singlet	1	-435.9297267	-162.281848	-378.6726002	EMD2
TCGACCTGTCCAGCAC-1	singlet	2	-1446.342807	-1499.178378	-498.2831347	EMD2
TCGACCTTCACTTGTT-1	singlet	1	-1418.184045	-476.8938088	-1156.023822	EMD2
TCGACCTTCCGAGAAG-1	singlet	2	-1472.578539	-1410.900463	-511.9648225	EMD2
TCGACCTTCGATGCTA-1	singlet	1	-20.90145975	-10.35133638	-16.13529252	EMD2
TCGACCTTCTAGGCAT-1	singlet	0	-798.0253203	-2024.542373	-1838.454837	EMD2
TCGACGGAGGTCATCT-1	singlet	1	-379.5188565	-142.5782983	-321.0986752	EMD2
TCGACGGAGTACTGTC-1	singlet	0	-332.377905	-1054.962397	-981.400292	EMD2
TCGACGGAGTCTACCA-1	singlet	1	-259.8166065	-136.3276078	-274.1025919	EMD2
TCGACGGAGTGGTTGG-1	singlet	0	-483.9151647	-1432.912421	-1391.402747	EMD2
TCGACGGCACAATGAA-1	singlet	1	-444.5488305	-157.6504167	-391.0749043	EMD2
TCGACGGCACATGGTT-1	singlet	1	-1288.710267	-381.1713191	-1083.502699	EMD2
TCGACGGCAGCAGTTT-1	singlet	1	-2743.319695	-551.7596678	-1870.311533	EMD2
TCGACGGCATTGAAAG-1	singlet	1	-346.5338727	-156.2299965	-328.9522407	EMD2
TCGACGGGTAGTGGCA-1	singlet	1	-492.5616981	-191.2639535	-431.959926	EMD2
TCGACGGTCATACAGC-1	singlet	2	-1023.633848	-982.0962042	-330.2040752	EMD2
TCGACGGTCGTGGAAG-1	singlet	0	-205.6629958	-696.7651572	-642.6178665	EMD2
TCGATTTAGCAGTCTT-1	singlet	1	-1876.10271	-684.5587576	-1399.327904	EMD2
TCGATTTAGCTGTACT-1	singlet	1	-203.9616667	-84.52011558	-193.3071757	EMD2
TCGATTTAGGGCAAGG-1	singlet	1	-273.6607197	-102.8684504	-211.1857907	EMD2
TCGATTTCACTATGTG-1	singlet	2	-1805.08453	-1447.231602	-657.0588016	EMD2
TCGATTTCAGTGCCTG-1	singlet	1	-161.0463795	-44.96631658	-128.7033564	EMD2
TCGATTTCAGTGTACT-1	singlet	0	-134.4573931	-383.938653	-277.8349674	EMD2
TCGATTTCATGGAATA-1	singlet	1	-871.5580852	-325.1907019	-696.6478743	EMD2
TCGATTTCATGTAACC-1	singlet	1	-1438.046209	-483.1585621	-1027.630141	EMD2
TCGATTTGTGACTCGC-1	unassigned	0/2	-11.53435668	-22.65690672	-14.14794237	EMD2
TCGATTTGTGTGATGG-1	singlet	0	-221.4966804	-643.3879247	-541.0275329	EMD2
TCGATTTTCCGTGGGT-1	singlet	1	-850.9827696	-258.4557294	-683.0749534	EMD2
TCGATTTTCGAAGCAG-1	singlet	0	-105.4201067	-270.7075227	-240.6606615	EMD2
TCGATTTTCGCATTAG-1	singlet	2	-2098.664302	-1647.993526	-670.4980475	EMD2
TCGATTTTCTGAGATC-1	singlet	1	-2460.029911	-749.2129623	-1828.768687	EMD2
TCGCACTAGAATCTAG-1	singlet	0	-339.32299	-852.3565188	-759.3787239	EMD2
TCGCACTAGACCCGCT-1	singlet	1	-3566.829481	-932.2986512	-2879.806442	EMD2
TCGCACTAGGATCACG-1	singlet	1	-2084.81776	-646.0322296	-1737.716282	EMD2
TCGCACTCAAACTCGT-1	singlet	1	-168.3934979	-58.81463554	-152.3692728	EMD2
TCGCACTCACCTCTAC-1	singlet	1	-3561.396667	-1121.262743	-2844.82473	EMD2
TCGCACTCACTTCTCG-1	singlet	1	-452.9078809	-190.5671777	-373.8093493	EMD2
TCGCACTCATTAGGAA-1	singlet	1	-1262.702692	-361.7118362	-1123.902679	EMD2
TCGCACTCATTGGATC-1	singlet	0	-1823.680116	-5204.041423	-4773.569222	EMD2
TCGCACTGTAATGCTC-1	unassigned	1	-23.23453505	-4.043248626	-8.218695443	EMD2
TCGCACTGTACGATTC-1	singlet	1	-1565.277003	-536.6930885	-1158.7943	EMD2
TCGCACTGTATCGCGC-1	singlet	1	-274.4548513	-107.2342358	-221.413867	EMD2
TCGCACTGTCGGATTT-1	singlet	1	-1455.026044	-473.7156172	-1218.228137	EMD2
TCGCACTTCAACTCTT-1	singlet	1	-306.2119136	-132.9144821	-275.8225776	EMD2
TCGCACTTCTTGATTC-1	unassigned	1	-1.834527263	-1.096349548	-5.552080916	EMD2
TCGCACTTCTTTGCGC-1	singlet	1	-259.0749173	-126.9405042	-222.6484703	EMD2
TCGCAGGAGCAGCGAT-1	singlet	1	-1132.569944	-396.9759648	-1092.876415	EMD2
TCGCAGGAGGGAGGAC-1	singlet	0	-158.9248004	-473.8236944	-484.8009129	EMD2
TCGCAGGAGTAATACG-1	singlet	0	-222.7432578	-437.3517994	-426.513047	EMD2
TCGCAGGAGTACAGCG-1	singlet	1	-1197.51489	-394.2522419	-914.5556165	EMD2
TCGCAGGCACAGTACT-1	singlet	1	-1545.026949	-451.5164527	-1376.702591	EMD2
TCGCAGGCACGGGCTT-1	singlet	0	-469.1631334	-1209.382188	-1216.210818	EMD2
TCGCAGGCACTTGTCC-1	singlet	1	-2562.511182	-757.1838003	-1933.01325	EMD2
TCGCAGGCATCGAAGG-1	singlet	0	-101.2413	-199.9211428	-187.1675472	EMD2
TCGCAGGGTCCTGTTC-1	singlet	1	-212.6910496	-79.63547446	-181.291953	EMD2
TCGCAGGGTCGGATTT-1	singlet	1	-480.3870492	-156.4237037	-330.4329048	EMD2
TCGCAGGGTTCGTTCC-1	singlet	0	-351.5329887	-959.5320064	-937.2538279	EMD2
TCGCAGGTCAACGAGG-1	singlet	2	-2473.256797	-2113.674259	-877.0250962	EMD2
TCGCAGGTCATTCCTA-1	singlet	1	-597.9434615	-174.6737611	-428.9532876	EMD2
TCGCAGGTCCTAACAG-1	singlet	1	-729.6499468	-190.3353317	-564.1717683	EMD2
TCGCAGGTCTAGCCAA-1	singlet	1	-1463.879513	-406.163107	-1222.244807	EMD2
TCGCTCAAGGCACCAA-1	singlet	0	-622.858603	-1918.665904	-1852.359762	EMD2
TCGCTCAGTGTCGATT-1	singlet	0	-383.411566	-1394.777091	-1162.342221	EMD2
TCGCTCATCAGCAATC-1	singlet	2	-361.6318662	-283.0004938	-161.0542321	EMD2
TCGCTCATCGCAAGAG-1	singlet	2	-185.7693555	-105.8690842	-61.26227654	EMD2
TCGCTCATCGTGGCTG-1	singlet	0	-438.1053737	-963.9692282	-1029.661858	EMD2
TCGCTTGAGTTTGTCG-1	singlet	1	-870.7126942	-306.0164338	-773.4654369	EMD2
TCGCTTGCACCGAATT-1	singlet	1	-1058.256402	-360.757014	-919.6041914	EMD2
TCGCTTGCAGCTCATA-1	singlet	1	-138.8005016	-54.99729093	-123.9211028	EMD2
TCGCTTGCATCGAGCC-1	singlet	1	-1919.5557	-645.365895	-1592.127549	EMD2
TCGCTTGCATGTTCAG-1	singlet	1	-4696.427897	-1404.132719	-3792.666685	EMD2
TCGCTTGGTACTGTTG-1	singlet	2	-1233.196542	-1067.880147	-392.991888	EMD2
TCGCTTGGTATGGTTC-1	singlet	1	-639.1166314	-250.5986179	-632.0091864	EMD2
TCGCTTGGTCACGTGC-1	singlet	1	-2901.882324	-844.3370886	-2389.727664	EMD2
TCGCTTGGTCCTGTCT-1	singlet	2	-1370.510572	-1242.103282	-485.8753392	EMD2
TCGCTTGGTGACAGCA-1	doublet	45323	-3471.055883	-2199.097107	-2128.400828	EMD2
TCGCTTGTCAAGAAAC-1	singlet	1	-1589.873055	-418.279351	-1334.250215	EMD2
TCGCTTGTCCACAAGT-1	singlet	0	-306.4462152	-869.4995413	-826.6373076	EMD2
TCGCTTGTCTAGAGCT-1	singlet	1	-921.451759	-314.7661932	-731.4357195	EMD2
TCGGATAAGATGATTG-1	singlet	2	-307.5277397	-243.9223653	-126.5678753	EMD2
TCGGATAAGCGCTGAA-1	singlet	1	-930.7435979	-275.510668	-790.1844546	EMD2
TCGGATAAGCGTTCCG-1	singlet	1	-1618.58988	-465.6800219	-1203.735235	EMD2
TCGGATACATGAAGGC-1	singlet	1	-174.5224827	-77.79981115	-146.6562012	EMD2
TCGGATAGTCCAGCGT-1	singlet	1	-499.891902	-194.6385675	-476.4288647	EMD2
TCGGATAGTCTTGGTA-1	singlet	0	-1829.740602	-6093.819866	-5793.647497	EMD2
TCGGATAGTGTCCTAA-1	singlet	1	-393.653402	-171.9084759	-376.7751035	EMD2
TCGGATAGTTGACTAC-1	singlet	2	-364.1943164	-432.6416058	-168.5941672	EMD2
TCGGATATCCATCTCG-1	singlet	2	-3128.28407	-2835.948811	-1133.880225	EMD2
TCGGGACAGGGCCTCT-1	singlet	0	-102.2003556	-309.6857695	-227.7739856	EMD2

TCGGGACAGGTAGATT-1	singlet	2	-906.5943943	-839.812623	-367.5524626	EMD2
TCGGGACCAACCCTAA-1	singlet	1	-968.8793264	-340.0931939	-882.3564139	EMD2
TCGGGACCAGGTCAGA-1	singlet	1	-219.2695248	-88.29728574	-193.3341403	EMD2
TCGGGACCAGGTGTTT-1	singlet	0	-53.23416473	-172.3763659	-143.0065258	EMD2
TCGGGACGTGTTGATC-1	singlet	2	-620.9353863	-505.5428623	-216.9040197	EMD2
TCGGGCAAGCAAATGT-1	singlet	0	-32.08088094	-72.99040694	-70.58367769	EMD2
TCGGGCAAGGTGCAGT-1	singlet	1	-1658.590768	-523.1659388	-1349.652891	EMD2
TCGGGCACAACCTAAC-1	singlet	0	-331.9968997	-1136.99333	-1029.47108	EMD2
TCGGGCACAAGCCCAC-1	singlet	2	-2490.20115	-2161.097392	-892.7119911	EMD2
TCGGGCACAAGCGCAA-1	singlet	0	-1250.117525	-4144.830764	-3916.740227	EMD2
TCGGGCACACTTGAGT-1	singlet	1	-391.7363266	-189.2148275	-379.4934408	EMD2
TCGGGCACATCACGGC-1	singlet	2	-461.8643468	-457.207116	-187.6142914	EMD2
TCGGGCAGTCAAAGAT-1	singlet	2	-11.46846365	-8.578075234	-5.137889188	EMD2
TCGGGCAGTGTAGGAC-1	singlet	1	-900.0026269	-337.52707	-770.4068783	EMD2
TCGGGCATCCTTGAAG-1	singlet	1	-2939.085007	-962.4929483	-2265.620602	EMD2
TCGGGCATCTCTTCAA-1	singlet	2	-850.7078627	-973.0749225	-349.5672286	EMD2
TCGGGCATCTGCGGAC-1	singlet	1	-705.0211659	-278.3635271	-591.4091689	EMD2
TCGGGTGAGACAGCGT-1	singlet	0	-1779.549549	-6046.898037	-6011.296011	EMD2
TCGGGTGAGCCTCGTG-1	singlet	1	-513.6931608	-169.3468895	-409.8700494	EMD2
TCGGGTGAGCCTTTGA-1	singlet	1	-842.1394741	-253.9033435	-715.0183572	EMD2
TCGGGTGAGCGCTGAA-1	singlet	0	-124.4491822	-256.281786	-249.9198338	EMD2
TCGGGTGGTACAAAGT-1	singlet	2	-307.9703773	-272.8965294	-120.5052277	EMD2
TCGGGTGGTCTGATCA-1	singlet	1	-452.1894966	-167.0940446	-380.8452301	EMD2
TCGGGTGGTTAAAGTG-1	singlet	0	-771.0261068	-2565.571724	-2421.391035	EMD2
TCGGGTGGTTCTCCCA-1	singlet	0	-26.15274235	-90.3627716	-81.38714483	EMD2
TCGGGTGTCAATCTTC-1	singlet	1	-1881.915266	-580.9875347	-1481.802476	EMD2
TCGGGTGTCAGAGTGG-1	singlet	2	-2234.625638	-1575.311921	-623.8277443	EMD2
TCGGGTGTCGCTAAAC-1	singlet	2	-2770.544501	-2392.729015	-773.8198929	EMD2
TCGGGTGTCGTACCTC-1	singlet	2	-4055.713257	-2903.146019	-1019.233627	EMD2
TCGGTCTAGAATGTTG-1	singlet	1	-1242.146015	-420.5702715	-1067.585223	EMD2
TCGGTCTAGATAGTGT-1	singlet	1	-389.6792149	-135.4240623	-323.3436753	EMD2
TCGGTCTAGGTGCAGT-1	singlet	0	-711.2681979	-2095.951803	-1803.874142	EMD2
TCGGTCTAGTGCTAGG-1	singlet	1	-211.8122153	-84.28201303	-174.4845948	EMD2
TCGGTCTCAGCATACT-1	singlet	1	-2795.677525	-735.3017515	-1889.83373	EMD2
TCGGTCTGTCTTTATC-1	singlet	2	-1451.583785	-1713.052293	-413.1069925	EMD2
TCGGTCTGTGTTACTG-1	singlet	0	-18.08052419	-58.59725337	-51.15792125	EMD2
TCGGTCTGTTCGGTCG-1	singlet	1	-559.9590188	-139.6726139	-369.3540859	EMD2
TCGGTCTTCAGGACAG-1	singlet	2	-4036.298195	-3525.389288	-1431.439573	EMD2
TCGGTCTTCTCAGGCG-1	singlet	1	-231.810311	-93.81080237	-221.6767034	EMD2
TCGGTCTTCTCTCAAT-1	singlet	1	-1057.448779	-412.6914627	-838.4546179	EMD2
TCGTAGAAGATGCTGG-1	singlet	2	-3073.687192	-2356.088959	-852.0997292	EMD2
TCGTAGAAGCTAGAAT-1	singlet	2	-570.4955416	-546.1467178	-217.7206091	EMD2
TCGTAGAAGGAAACGA-1	singlet	1	-1831.649124	-579.7291021	-1643.358125	EMD2
TCGTAGAAGGTCGACA-1	singlet	1	-286.4789998	-167.9996325	-290.8177317	EMD2
TCGTAGACAAGCACCC-1	singlet	2	-3679.334579	-2657.643204	-1597.200591	EMD2
TCGTAGAGTGCACAAG-1	singlet	1	-70.76247718	-30.06856329	-67.08159343	EMD2
TCGTAGAGTGGGCTCT-1	singlet	2	-1142.381852	-975.8321489	-397.9712592	EMD2
TCGTAGAGTTCGGTTA-1	singlet	1	-316.2204428	-115.202065	-257.824885	EMD2
TCGTAGAGTTGCATCA-1	doublet	0/1	-1683.999312	-2120.091006	-2525.837025	EMD2
TCGTAGATCATTCATC-1	singlet	0	-18.41612871	-56.30607832	-81.20753351	EMD2
TCGTAGATCGCTCTCA-1	singlet	0	-634.7794607	-1883.678247	-1857.802266	EMD2
TCGTAGATCTCCGAAA-1	singlet	1	-2410.425989	-690.9926113	-1921.347459	EMD2
TCGTCCAAGAACGCGT-1	singlet	2	-273.8081162	-235.3208186	-112.4898592	EMD2
TCGTCCAAGAATAGTC-1	singlet	0	-652.7426946	-2030.613906	-1771.595206	EMD2
TCGTCCAAGACGTCGA-1	singlet	1	-1307.024316	-404.5420036	-986.8772962	EMD2
TCGTCCAAGTCAGCCC-1	singlet	2	-4672.943543	-3764.580996	-1610.535094	EMD2
TCGTCCACAATTTCTC-1	singlet	2	-427.7801313	-485.3533755	-171.1602114	EMD2
TCGTCCACATACTGAC-1	singlet	2	-5539.279305	-4641.522103	-1545.272664	EMD2
TCGTCCAGTCTTACAG-1	singlet	0	-207.1714858	-721.842572	-605.2951564	EMD2
TCGTCCATCTGAGGCC-1	singlet	1	-565.064059	-194.9715335	-448.0792336	EMD2
TCGTGCTAGCCTCACG-1	singlet	2	-543.8330195	-550.9121064	-182.1261798	EMD2
TCGTGCTAGCGCCCAT-1	singlet	1	-663.7394398	-321.1280342	-575.5064299	EMD2
TCGTGCTCAAGCCCAC-1	singlet	1	-349.6602216	-100.0611643	-301.2267812	EMD2
TCGTGCTCACCCTTAC-1	singlet	0	-115.6629605	-301.4626315	-299.3061735	EMD2
TCGTGCTCAGCGGTTC-1	singlet	1	-535.9131361	-186.6914569	-367.4926197	EMD2
TCGTGCTCAGTTAGAA-1	singlet	1	-674.4752675	-236.0875045	-581.4670579	EMD2
TCGTGGGAGAGAGTGA-1	singlet	1	-293.0895089	-110.341175	-222.1823924	EMD2
TCGTGGGAGCCGAATG-1	singlet	0	-535.5991418	-1210.431065	-1193.834892	EMD2
TCGTGGGCAGAATTCC-1	singlet	1	-885.3070317	-290.6784755	-689.0149756	EMD2
TCGTGGGGTCCTTTGC-1	singlet	2	-191.0239852	-193.6143167	-63.57394206	EMD2
TCGTGGGGTCGGATTT-1	singlet	1	-166.6813845	-90.2915273	-171.1905617	EMD2
TCGTGGGGTTATTCCT-1	singlet	0	-534.4383924	-1703.608677	-1619.248198	EMD2
TCGTGGGTCCGTGTAA-1	doublet	1/0	-1799.616679	-1628.332828	-2277.036093	EMD2
TCGTGGGTCGAGCTGC-1	singlet	1	-792.8433261	-268.2236816	-622.8301552	EMD2
TCGTGGGTCGCTCCTA-1	singlet	1	-294.2734622	-82.19586457	-199.4699851	EMD2
TCGTGGGTCGGACTTA-1	singlet	0	-364.4821877	-1121.270154	-1024.805822	EMD2
TCGTGGGTCTAAACGC-1	singlet	1	-1251.67915	-400.613658	-1141.119496	EMD2
TCTAACTAGCCATCCG-1	singlet	2	-102.8593071	-45.87343412	-28.63238636	EMD2
TCTAACTAGCGCTTCG-1	singlet	1	-1070.687586	-331.2437985	-816.6304653	EMD2
TCTAACTAGTTACTCG-1	singlet	1	-2297.285961	-680.3692684	-1644.426424	EMD2
TCTAACTCAAGAGGCT-1	singlet	2	-882.7941109	-806.4199305	-324.9255234	EMD2
TCTAACTCACTGGCCA-1	singlet	2	-3090.322493	-2879.163183	-1146.211637	EMD2
TCTAACTCAGACATCT-1	singlet	0	-2142.309371	-7260.151942	-7392.619513	EMD2
TCTAACTGTACAATAG-1	singlet	2	-3706.692987	-3118.448781	-1378.813637	EMD2
TCTAACTGTACACTCA-1	singlet	1	-2032.843604	-681.5966869	-1649.960952	EMD2
TCTAACTGTATCGCAT-1	singlet	1	-235.4573471	-79.68125811	-150.7251957	EMD2
TCTAACTGTCGCCACA-1	singlet	1	-311.3749979	-116.0704586	-210.3546536	EMD2
TCTAACTGTCGGTGTC-1	singlet	1	-1399.36654	-414.9787976	-1075.862271	EMD2
TCTAACTGTGCTATTG-1	singlet	2	-263.9998498	-345.6810784	-158.3101941	EMD2
TCTAACTTCAACGTGT-1	singlet	1	-371.7542862	-123.2166343	-314.7628135	EMD2
TCTAACTTCAGTGTTG-1	unassigned	45323	-75.04889476	-51.97898187	-32.40097827	EMD2
TCTAACTTCGTTGTGA-1	singlet	1	-410.1751289	-134.8792764	-274.9102928	EMD2
TCTAACTTCTTCTGTA-1	singlet	1	-852.7972771	-266.4868961	-627.2350593	EMD2
TCTACATAGCCACAAG-1	singlet	0	-461.9341161	-1621.636227	-1475.644287	EMD2
TCTACATAGCCTCAAT-1	singlet	0	-922.7938184	-1826.908234	-1876.371461	EMD2
TCTACATAGGGCGAAG-1	singlet	0	-493.5219551	-1681.301655	-1540.586171	EMD2
TCTACATCACAATTCG-1	singlet	1	-784.694394	-288.7764191	-628.9696175	EMD2
TCTACATCAGAGTGTG-1	singlet	1	-1696.470162	-525.399748	-1442.535384	EMD2
TCTACATCAGGCACTC-1	singlet	1	-2413.749902	-877.1190655	-2050.376092	EMD2
TCTACATGTAGCACGA-1	singlet	1	-1067.019466	-376.5868721	-844.6961346	EMD2
TCTACATGTCCCAAAT-1	singlet	1	-442.7151946	-199.7696287	-385.8683785	EMD2
TCTACATTCACCATCC-1	singlet	1	-5892.738482	-1976.117942	-4730.340441	EMD2
TCTACATTCCTACCAC-1	singlet	2	-1446.94529	-1284.783082	-566.0438849	EMD2
TCTACATTCGACGCTG-1	singlet	1	-91.94644909	-41.81505364	-77.11381906	EMD2
TCTACATTCGGCCCAA-1	singlet	2	-2154.103861	-1775.667167	-566.3923178	EMD2
TCTACATTCTGCCTGT-1	singlet	0	-945.9398992	-2928.062994	-2706.441606	EMD2
TCTACCGAGGACATCG-1	singlet	2	-798.188638	-727.238838	-295.8877274	EMD2
TCTACCGCAAATCCCA-1	singlet	1	-951.3161404	-354.3262055	-793.2737631	EMD2
TCTACCGCACTCACTC-1	singlet	2	-2584.760785	-2522.543481	-1078.912906	EMD2
TCTACCGCAGACCCGT-1	singlet	1	-592.618243	-255.802177	-545.9824537	EMD2

TCTACCGCATGCCATA-1	singlet	1	-5421.558573	-2482.210228	-4688.456077	EMD2
TCTACCGGTCGCGTTG-1	singlet	1	-1529.301478	-465.5916884	-1246.282567	EMD2
TCTACCGGTTTGGGAG-1	singlet	2	-3426.724151	-3214.824953	-959.0430207	EMD2
TCTACCGTCACAAGAA-1	singlet	2	-390.9196189	-312.5430593	-107.3449376	EMD2
TCTACCGTCAGCTTCC-1	singlet	1	-8542.804857	-2412.606037	-6470.645115	EMD2
TCTACCGTCGAGAAAT-1	singlet	2	-678.1962807	-613.0219035	-278.6550338	EMD2
TCTATACAGAATCGTA-1	singlet	1	-2682.552654	-766.9905966	-2044.873169	EMD2
TCTATACAGACCTCAT-1	singlet	1	-395.3756821	-149.7370701	-325.98295	EMD2
TCTATACAGAGCAACC-1	singlet	0	-1445.730997	-4682.766388	-4419.338396	EMD2
TCTATACGTACTGTTG-1	singlet	0	-576.4394549	-1655.282822	-1709.5105	EMD2
TCTATACGTAGGTTTC-1	singlet	2	-598.6971926	-498.1793812	-206.6904712	EMD2
TCTATACGTTATGGTC-1	singlet	1	-6847.497395	-1986.309677	-5216.076662	EMD2
TCTATACGTTCACGAT-1	singlet	1	-1102.338759	-348.7381449	-938.6171671	EMD2
TCTATACTCGGCACTG-1	singlet	1	-2235.159476	-842.4197876	-1777.431341	EMD2
TCTATCAAGCCGCTTG-1	singlet	1	-288.7655232	-127.8319774	-256.1313437	EMD2
TCTATCAAGCGTATGG-1	singlet	1	-82.05705159	-41.42613671	-52.46435051	EMD2
TCTATCAGTCCTCCAT-1	singlet	2	-1780.429958	-1697.943333	-718.1328916	EMD2
TCTATCAGTCGTAATC-1	doublet	0/1	-4621.510222	-4984.776822	-6849.590668	EMD2
TCTATCATCGCCATAA-1	singlet	1	-1656.015942	-537.7305336	-1357.255897	EMD2
TCTATCATCTTTCTAG-1	singlet	0	-775.3955371	-2366.562358	-2195.560084	EMD2
TCTCACGAGGTCGACA-1	singlet	0	-286.3690774	-779.1093638	-745.7625875	EMD2
TCTCACGAGTGATAAC-1	singlet	1	-652.8260799	-295.5601978	-563.9677567	EMD2
TCTCACGCAAATACAG-1	singlet	1	-44.24294028	-14.6298337	-21.12951052	EMD2
TCTCACGCAACGGCTC-1	singlet	1	-2934.695202	-865.3664318	-2355.111944	EMD2
TCTCACGCAAGCGCTC-1	singlet	0	-246.7264331	-711.3844973	-684.1216022	EMD2
TCTCACGCACCTTCGT-1	singlet	1	-8515.193798	-2499.844254	-6432.720137	EMD2
TCTCACGCAGCAGACA-1	singlet	2	-3331.809069	-2609.83361	-1071.883014	EMD2
TCTCACGCAGGTACGA-1	singlet	0	-312.7183304	-944.5838789	-986.4577609	EMD2
TCTCACGTCCATCTGC-1	singlet	1	-3194.493472	-1162.985941	-2834.400625	EMD2
TCTCACGTCGATTGAC-1	singlet	0	-141.5044253	-473.3065258	-458.46441	EMD2
TCTCAGCAGAGTCCGA-1	singlet	2	-876.7129299	-760.8260871	-382.5744343	EMD2
TCTCAGCCACCTTCGT-1	singlet	1	-1732.839346	-662.0035945	-1558.99859	EMD2
TCTCAGCCACGGCACT-1	singlet	0	-185.6968001	-472.8201115	-455.390149	EMD2
TCTCAGCCACTACTTT-1	singlet	2	-1156.732448	-1037.891542	-467.760289	EMD2
TCTCAGCTCATCGGGC-1	singlet	1	-2941.602117	-831.0118465	-2097.829147	EMD2
TCTCAGCTCCAAGGGA-1	singlet	1	-194.197818	-83.4511312	-167.8630463	EMD2
TCTCAGCTCCGAGCTG-1	singlet	2	-3198.35693	-2812.571944	-1111.078712	EMD2
TCTCCGAAGTTCTACG-1	singlet	1	-635.9430809	-215.7862314	-549.6096315	EMD2
TCTCCGACAAGTATCC-1	singlet	1	-710.6991424	-235.0806651	-655.2933436	EMD2
TCTCCGACATTGTCGA-1	singlet	1	-2571.800232	-872.9084515	-1964.709577	EMD2
TCTCCGAGTTCACGAT-1	singlet	0	-712.8502098	-2300.967849	-1982.31044	EMD2
TCTCCGATCGCGTTTC-1	singlet	0	-895.9569159	-3155.783942	-2850.899704	EMD2
TCTCTGGAGCCAGTAG-1	singlet	1	-1299.55255	-416.5920492	-956.6085729	EMD2
TCTCTGGAGGCATCGA-1	singlet	0	-63.82289688	-169.7333586	-174.7602272	EMD2
TCTCTGGCAACAGTGG-1	doublet	45293	-4944.032259	-2703.647698	-3402.894075	EMD2
TCTCTGGCACGAGGTA-1	singlet	1	-1672.493117	-594.0131998	-1346.403344	EMD2
TCTCTGGCAGGTAGTG-1	singlet	2	-1866.390243	-1676.533743	-704.9339449	EMD2
TCTCTGGGTCGGCTAC-1	singlet	1	-429.4725173	-135.8892984	-451.6339223	EMD2
TCTCTGGGTCTCCTGT-1	singlet	1	-845.3628601	-325.3073813	-584.683191	EMD2
TCTCTGGGTCTTCATT-1	singlet	2	-6448.271149	-4905.693343	-1664.813557	EMD2
TCTCTGGTCCCGAGTG-1	singlet	1	-1583.51858	-547.2154628	-1307.317965	EMD2
TCTCTGGTCTGTAAGC-1	singlet	0	-97.54340538	-317.1032474	-260.5396661	EMD2
TCTGCCAAGGACTATA-1	singlet	1	-3885.777233	-813.6919052	-2817.936945	EMD2
TCTGCCACAATGAAAC-1	singlet	0	-1036.607619	-4319.176696	-4198.898132	EMD2
TCTGCCACACCGTACG-1	singlet	2	-26.01679706	-31.50283436	-6.665084354	EMD2
TCTGCCAGTACCGTCG-1	singlet	1	-342.9630039	-140.9383754	-281.7322615	EMD2
TCTGCCAGTCCTCATC-1	singlet	1	-2055.243799	-569.2462024	-1724.230017	EMD2
TCTGCCAGTGCCGTAC-1	singlet	2	-900.1502925	-727.4988813	-344.5007537	EMD2
TCTGCCATCGGTCGGT-1	singlet	0	-818.5229868	-2960.857405	-2891.192351	EMD2
TCTGCCATCGGTGCAC-1	singlet	1	-366.0451624	-141.7602943	-312.9232032	EMD2
TCTGGCTAGAGTGTTA-1	singlet	0	-1.731474295	-6.261101494	-7.066333115	EMD2
TCTGGCTAGGTTACCT-1	singlet	1	-893.2236113	-360.5247344	-755.5756805	EMD2
TCTGGCTAGTTGTAAG-1	singlet	1	-1513.501484	-472.6484543	-1212.358929	EMD2
TCTGGCTCAGCATTGT-1	singlet	0	-1506.144484	-4179.236336	-4276.973537	EMD2
TCTGGCTCAGGTCAGA-1	singlet	1	-449.202428	-157.6217364	-409.4409446	EMD2
TCTGGCTGTCGGAAAC-1	singlet	1	-232.0331154	-122.6737078	-193.1357523	EMD2
TCTGGCTGTGGCTAGA-1	singlet	0	-417.1421596	-1401.139802	-1195.298497	EMD2
TCTGGCTGTTATGTCG-1	singlet	1	-859.5498946	-334.6265318	-671.6255404	EMD2
TCTGGCTTCACGGTCG-1	singlet	1	-466.1249514	-187.3092009	-410.1402066	EMD2
TCTGGCTTCCACGGGT-1	singlet	1	-483.2019227	-212.3124466	-418.7005235	EMD2
TCTGGCTTCTAGAACC-1	singlet	1	-472.3467502	-148.4059693	-366.9162593	EMD2
TCTGGCTTCTAGTCAG-1	singlet	1	-1953.300789	-589.9853396	-1799.322842	EMD2
TCTGGCTTCTTCGACC-1	singlet	1	-1675.351014	-648.8480679	-1360.824124	EMD2
TCTGTCGAGCATCCTA-1	singlet	2	-1932.931649	-1686.277651	-683.2566337	EMD2
TCTGTCGAGCGAGAAA-1	singlet	0	-461.5244164	-1550.382451	-1467.512036	EMD2
TCTGTCGCAAAGCAAT-1	singlet	1	-1845.884667	-557.4136041	-1719.946563	EMD2
TCTGTCGCAAAGCGTG-1	singlet	1	-830.8545269	-313.5422178	-645.4523861	EMD2
TCTGTCGCATACTTTC-1	singlet	1	-303.2915579	-95.16294104	-233.5789074	EMD2
TCTGTCGGTAGTGGCA-1	singlet	2	-289.5433198	-315.8768992	-141.6119266	EMD2
TCTGTCGGTGACGTCC-1	singlet	1	-573.0313672	-196.6914266	-471.7720725	EMD2
TCTGTCGGTGTAGTGG-1	singlet	1	-1672.524264	-563.3384156	-1302.510223	EMD2
TCTGTCGTCACCCTCA-1	singlet	0	-72.98116162	-224.5591574	-190.8331221	EMD2
TCTGTCGTCCCGAACG-1	singlet	0	-1787.084219	-6741.075676	-6337.813181	EMD2
TCTGTCGTCCTACCGT-1	singlet	1	-1325.435319	-418.9397087	-1021.807212	EMD2
TCTGTCGTCGCCGAAC-1	singlet	2	-1529.758645	-1265.00883	-487.4994835	EMD2
TCTGTCGTCTTTCCGG-1	singlet	2	-2464.261332	-2061.490549	-796.8557855	EMD2
TCTTAGTAGAAACTAC-1	singlet	1	-4133.203298	-1089.613463	-3262.773032	EMD2
TCTTAGTAGAGAGCAA-1	singlet	2	-679.0916294	-618.7153341	-267.1206074	EMD2
TCTTAGTAGGCTAACG-1	singlet	1	-1416.738622	-516.9752247	-1153.418975	EMD2
TCTTAGTCAGAAGCTG-1	singlet	1	-2460.991499	-742.6478256	-1873.245394	EMD2
TCTTAGTCAGGACTAG-1	singlet	1	-645.8738454	-245.2993808	-553.064968	EMD2
TCTTAGTGTACAGAAT-1	singlet	2	-4674.517568	-3890.658731	-1502.90833	EMD2
TCTTAGTGTCACGCTG-1	singlet	1	-1306.189217	-418.1938107	-1107.202997	EMD2
TCTTAGTGTGGCTCTG-1	singlet	1	-746.7529407	-256.9191898	-663.2054417	EMD2
TCTTAGTTCAGGGTAG-1	singlet	0	-675.2412675	-1991.45461	-1993.230921	EMD2
TCTTAGTTCTCCGTGT-1	singlet	0	-1487.410184	-5184.145971	-4698.966844	EMD2
TCTTCCTAGAAGCTGC-1	singlet	2	-214.7420535	-196.6976468	-98.13609995	EMD2
TCTTCCTAGGCCCACT-1	singlet	1	-406.3059191	-158.7422006	-297.5674407	EMD2
TCTTCCTCATGGCCAC-1	singlet	1	-1465.770432	-485.5721323	-1240.326447	EMD2
TCTTCCTGTAGCTGAG-1	singlet	2	-303.9065437	-363.3837778	-147.149243	EMD2
TCTTCCTGTATGTCTG-1	singlet	1	-2975.154228	-1001.917012	-2467.465644	EMD2
TCTTCCTGTGGTTTGT-1	singlet	2	-10320.31295	-7956.674677	-2706.644271	EMD2
TCTTCCTTCAAGGTGG-1	singlet	2	-2793.800455	-2362.593033	-838.9441669	EMD2
TCTTCCTTCCTGCCAT-1	singlet	1	-406.9463079	-158.2922204	-310.4027246	EMD2
TCTTGCGAGTAGACCG-1	singlet	0	-670.9864913	-2165.561251	-2245.52456	EMD2
TCTTGCGCACAACGAG-1	singlet	1	-2365.552395	-688.3231042	-1686.633937	EMD2
TCTTGCGGTCACCCTT-1	singlet	1	-359.1740738	-129.7867304	-278.8064459	EMD2
TCTTGCGGTGACACGA-1	singlet	1	-630.4259539	-236.4998228	-516.9483769	EMD2
TCTTGCGGTGTGTGGA-1	singlet	1	-1096.167832	-360.7001431	-845.9768727	EMD2
TCTTGCGGTTCATCGA-1	singlet	1	-396.6627835	-144.0840807	-326.905299	EMD2

TCTTGCGTCCCTGGTT-1	singlet	1	-794.0503604	-300.4501507	-725.8395162	EMD2
TCTTGCGTCCCTTTGG-1	singlet	1	-379.5263067	-143.3615959	-312.5144229	EMD2
TCTTGCGTCCTACACC-1	doublet	0/1	-3873.266051	-6516.000196	-7433.49818	EMD2
TCTTGCGTCGGCTGGT-1	singlet	1	-240.7077238	-108.2764227	-211.6902459	EMD2
TCTTGCGTCTAGACAC-1	unassigned	1	-3.669276445	-0.893383558	-2.197996563	EMD2
TCTTGCGTCTCGCTTG-1	singlet	1	-1651.959082	-618.7803572	-1428.079161	EMD2
TCTTTGAAGTATGCAA-1	singlet	2	-2800.606119	-2139.344721	-744.7484327	EMD2
TCTTTGACAAGTATAG-1	singlet	0	-300.1959742	-1478.740055	-1169.477911	EMD2
TCTTTGAGTATTGACC-1	singlet	0	-1770.359104	-5807.527284	-5314.055231	EMD2
TCTTTGAGTCGAGATG-1	singlet	1	-751.4831851	-308.2136748	-664.4722612	EMD2
TCTTTGAGTCGGCTAC-1	singlet	1	-345.0893169	-173.3148664	-360.5565942	EMD2
TCTTTGAGTGGATCAG-1	singlet	0	-241.9450627	-781.1837184	-621.8307187	EMD2
TCTTTGAGTGGATGAC-1	doublet	45323	-2820.46478	-1830.826681	-1224.108773	EMD2
TCTTTGATCACGGAGA-1	singlet	1	-1068.43631	-293.7216852	-783.749341	EMD2
TCTTTGATCCGTCACT-1	singlet	1	-1523.811189	-504.1823732	-1141.900041	EMD2
TCTTTGATCCTTGGAA-1	singlet	0	-419.8797706	-1810.669063	-1603.09466	EMD2
TGAACGTAGATAGCAT-1	singlet	1	-190.3465796	-85.20141187	-167.266479	EMD2
TGAACGTAGCGCTGCT-1	singlet	1	-797.316315	-291.4352984	-640.978788	EMD2
TGAACGTAGGCCCGTT-1	singlet	0	-1146.422781	-3530.409876	-3432.566639	EMD2
TGAACGTCACCTGTCT-1	singlet	1	-2176.746139	-631.4586319	-1843.453898	EMD2
TGAACGTCAGCTGAAG-1	singlet	1	-2442.282309	-892.0121906	-2044.67187	EMD2
TGAACGTCATATGGCT-1	singlet	2	-1502.056091	-1426.036479	-449.8931223	EMD2
TGAACGTGTACTGCCG-1	singlet	1	-2183.499314	-544.7073789	-1483.511785	EMD2
TGAACGTGTCAGGTGA-1	singlet	0	-1068.282823	-2954.730611	-2840.056473	EMD2
TGAACGTTCCTGTAGA-1	singlet	1	-359.6000738	-108.916544	-237.6174933	EMD2
TGAATCGAGAAGTGTT-1	singlet	1	-411.135399	-138.9415991	-333.8525011	EMD2
TGAATCGAGACCGCCT-1	singlet	1	-2052.146663	-678.6510906	-1823.818208	EMD2
TGAATCGAGAGCGACT-1	singlet	1	-1840.573111	-555.5710108	-1507.748868	EMD2
TGAATCGAGGGAGAAT-1	singlet	1	-2013.320398	-603.7119391	-1565.956562	EMD2
TGAATCGAGTACCCTA-1	singlet	0	-66.69910493	-243.5113845	-195.2477347	EMD2
TGAATCGAGTTGGACG-1	singlet	1	-341.1895467	-137.1145363	-298.965031	EMD2
TGAATCGCAAGTGGCA-1	singlet	0	-214.9983855	-569.7258705	-545.8643021	EMD2
TGAATCGCACTGCTTC-1	singlet	1	-992.0980802	-331.1039697	-760.3584557	EMD2
TGAATCGCACTGTGAT-1	singlet	1	-1690.728098	-636.1610899	-1375.345295	EMD2
TGAATCGCATAAGATG-1	singlet	2	-944.0560634	-912.0078201	-310.3244413	EMD2
TGAATCGGTACTCGTA-1	singlet	1	-207.4955153	-73.55839741	-160.1909109	EMD2
TGAATCGGTCCACTCT-1	singlet	0	-1141.837406	-3313.328836	-3262.288743	EMD2
TGAATCGGTTAGAGAT-1	unassigned	45293	-8.950982478	-3.252860584	-4.083628887	EMD2
TGAATCGTCAATCCGA-1	singlet	0	-1322.7343	-4182.577975	-4006.692846	EMD2
TGAATCGTCTGAGATC-1	singlet	2	-1757.851304	-1510.806202	-595.9799665	EMD2
TGAATGCAGCAGTACG-1	singlet	1	-4516.57052	-1528.305543	-3804.922081	EMD2
TGAATGCAGCGCGTTC-1	singlet	1	-6370.202237	-1660.564804	-5366.269219	EMD2
TGAATGCAGTGACACG-1	singlet	1	-2234.82835	-647.159804	-1749.457536	EMD2
TGAATGCCAGTGTGCC-1	singlet	2	-1118.568795	-891.6784494	-554.003704	EMD2
TGAATGCGTAGCGATG-1	singlet	1	-3580.858336	-1170.674705	-2659.245246	EMD2
TGAATGCGTCACAGTT-1	singlet	2	-1853.869055	-1569.775585	-572.7425515	EMD2
TGAATGCTCCGGCTTT-1	singlet	2	-1287.267831	-1299.350619	-398.0357469	EMD2
TGAATGCTCCTACTGC-1	singlet	1	-350.5800204	-133.4841767	-286.2880925	EMD2
TGAATGCTCCTGGGTG-1	doublet	45293	-31.13486626	-16.1229444	-19.01274387	EMD2
TGAATGCTCTGCTAGA-1	singlet	1	-885.1335377	-297.3570561	-739.4547986	EMD2
TGACAGTAGACCACGA-1	singlet	0	-1024.886971	-3406.5387	-3254.154391	EMD2
TGACAGTAGCGTTCAT-1	singlet	1	-3300.681936	-1186.79342	-2688.484323	EMD2
TGACAGTAGTAAATGC-1	singlet	1	-2853.125005	-942.9764452	-2024.498722	EMD2
TGACAGTAGTAATTGG-1	singlet	0	-1074.524694	-3146.660646	-3029.220899	EMD2
TGACAGTCACCTTCCA-1	singlet	1	-500.1857445	-225.1879879	-435.8065301	EMD2
TGACAGTCACGACGTC-1	singlet	0	-107.5348919	-314.8398548	-295.4580047	EMD2
TGACAGTCACGTAGAG-1	singlet	1	-77.98055785	-31.42208364	-62.90485004	EMD2
TGACAGTCAGACAAGC-1	singlet	1	-1804.628745	-617.7520883	-1513.113858	EMD2
TGACAGTCAGCATCTA-1	singlet	2	-2222.063305	-1948.1538	-758.0058207	EMD2
TGACAGTCAGGTCTCG-1	singlet	1	-2098.098636	-800.6883658	-1865.994663	EMD2
TGACAGTCATTCCTCG-1	singlet	1	-273.101344	-104.3906355	-265.1476167	EMD2
TGACAGTGTAGAGGAA-1	singlet	1	-1177.762062	-431.8223926	-918.8356798	EMD2
TGACAGTGTATTGGCT-1	singlet	2	-109.6171795	-122.8061675	-55.95001753	EMD2
TGACAGTGTTAGAAAC-1	singlet	1	-387.6463321	-153.504265	-324.0099432	EMD2
TGACAGTTCTCGTCAC-1	singlet	0	-711.6252655	-2435.168886	-2360.180291	EMD2
TGACCCTAGAAACCAT-1	singlet	1	-623.5028997	-240.360891	-585.1909321	EMD2
TGACCCTAGATGTAGT-1	singlet	1	-324.4958209	-130.6216408	-271.5058425	EMD2
TGACCCTAGTGCAGCA-1	singlet	1	-481.1577897	-173.5351356	-393.9310576	EMD2
TGACCCTCACGATAGG-1	singlet	0	-1102.253845	-3275.274322	-3396.133802	EMD2
TGACCCTCACGGGTAA-1	singlet	2	-810.4578768	-823.9177438	-304.4633374	EMD2
TGACCCTCAGTCAACT-1	singlet	1	-1952.267786	-583.4487768	-1601.553522	EMD2
TGACCCTGTAATTAGG-1	singlet	1	-291.945516	-77.89035688	-210.749352	EMD2
TGACCCTGTAGTATAG-1	singlet	1	-3632.688976	-965.357395	-2846.819228	EMD2
TGACCCTGTGAACCGA-1	singlet	1	-2083.394235	-668.6717785	-1641.097693	EMD2
TGACCCTGTTCCGGTG-1	singlet	2	-462.6635824	-505.2847518	-232.7335346	EMD2
TGACCCTTCATTGTGG-1	singlet	1	-1915.23661	-527.5784966	-1418.86342	EMD2
TGACCCTTCCTAGCCT-1	singlet	1	-45.22827833	-23.59820676	-40.78639977	EMD2
TGACCCTTCGGAGATG-1	singlet	0	-22.77534044	-39.89976412	-41.55979568	EMD2
TGACGCGAGAACTGAT-1	singlet	0	-597.3528483	-2057.682323	-2053.974896	EMD2
TGACGCGAGCCACAAG-1	singlet	2	-921.7706399	-762.5138083	-345.3599381	EMD2
TGACGCGAGCCTCTTC-1	singlet	1	-263.0561456	-107.7870257	-201.2844399	EMD2
TGACGCGAGGGTGGGA-1	singlet	2	-2241.83312	-1839.875143	-747.9057111	EMD2
TGACGCGCACAAAGCG-1	singlet	0	-6.995994081	-44.53459134	-36.09987819	EMD2
TGACGCGCACAACGCC-1	singlet	1	-1531.622307	-507.4788224	-1238.012728	EMD2
TGACGCGCACAGCTGC-1	singlet	2	-5326.586766	-4041.036286	-1456.454017	EMD2
TGACGCGCACTAAACC-1	singlet	1	-1353.100177	-490.3978432	-1079.614543	EMD2
TGACGCGCAGACCTAT-1	singlet	1	-2302.22786	-626.4498437	-1841.238669	EMD2
TGACGCGGTAGAGCTG-1	singlet	1	-1484.541489	-389.4360191	-950.8775186	EMD2
TGACGCGGTATCAAGA-1	singlet	1	-549.5163976	-194.477207	-438.3037727	EMD2
TGACGCGGTCATCACA-1	singlet	1	-390.9427957	-109.3996645	-248.5151871	EMD2
TGACGCGTCGGCATAT-1	singlet	1	-314.9774602	-83.96457247	-188.5008447	EMD2
TGACGCGTCTGTACAG-1	singlet	1	-679.5952749	-206.683696	-491.2329414	EMD2
TGACGCGTCTTCCTAA-1	singlet	1	-377.4721229	-142.8282553	-343.2568248	EMD2
TGACTCCCACACACTA-1	singlet	1	-5039.700016	-1256.546295	-3624.359391	EMD2
TGACTCCCAGGTGAGT-1	unassigned	0	-4.259171922	-5.136349419	-18.45714528	EMD2
TGACTCCCATGGAGAC-1	singlet	0	-446.397078	-1275.754707	-1192.69836	EMD2
TGACTCCGTACCCAGC-1	singlet	0	-571.7125545	-1758.448812	-1682.077457	EMD2
TGACTCCGTCCAGCGT-1	singlet	1	-2487.69228	-827.085816	-1981.380833	EMD2
TGACTCCGTCGCAACC-1	singlet	1	-3734.598006	-1285.764315	-2861.229024	EMD2
TGACTCCTCAGTCTTT-1	singlet	0	-903.7327561	-2926.487919	-2845.860063	EMD2
TGACTCCTCATCGCTC-1	singlet	1	-2020.388318	-651.4278162	-1559.306057	EMD2
TGACTCCTCTAGACAC-1	singlet	2	-1033.937013	-1004.784786	-415.2845827	EMD2
TGAGACTCAAATCAAG-1	singlet	0	-696.1933441	-1684.898395	-1602.539318	EMD2
TGAGACTCACGCTGTG-1	singlet	1	-372.032785	-151.4985344	-289.6508026	EMD2
TGAGACTCACTGTGTA-1	singlet	1	-1806.633156	-542.8194444	-1602.492182	EMD2
TGAGACTCATGTTTGG-1	singlet	2	-2101.8266	-1644.913333	-683.2861845	EMD2
TGAGACTGTTCGTGCG-1	singlet	1	-785.2070613	-286.1673381	-705.3872742	EMD2
TGAGACTGTTTACCAG-1	singlet	1	-431.7230121	-190.9239101	-336.6974568	EMD2
TGAGACTTCCGGCAAC-1	singlet	0	-270.3500883	-975.3314289	-928.9318648	EMD2
TGAGACTTCTCTGCTG-1	singlet	1	-321.6990969	-141.3960703	-254.2812432	EMD2

TGAGACTTCTTCGCTG-1	singlet	0	-300.8789331	-803.6915628	-787.0283655	EMD2
TGAGCATAGCGAGTCA-1	singlet	1	-1706.114084	-541.8999126	-1420.222196	EMD2
TGAGCATAGTGTAGAT-1	singlet	0	-43.8138787	-102.9739218	-85.29770139	EMD2
TGAGCATAGTTCCGGC-1	singlet	1	-379.1569188	-149.8896521	-304.5672834	EMD2
TGAGCATCAATGCTCA-1	doublet	45293	-285.8063419	-149.2327197	-230.6978646	EMD2
TGAGCATCACCCAAGC-1	singlet	1	-1858.760293	-368.7849288	-1210.408619	EMD2
TGAGCATCACTACCGG-1	singlet	1	-1887.962714	-603.0022236	-1466.484308	EMD2
TGAGCATCAGCTGTCG-1	singlet	2	-1430.338983	-1244.502669	-520.0034025	EMD2
TGAGCATCAGGCCCTA-1	singlet	0	-101.4690495	-325.9840455	-312.3901763	EMD2
TGAGCATCATCCGCGA-1	singlet	1	-1516.094506	-509.4987132	-1359.165732	EMD2
TGAGCATGTCACCGAC-1	singlet	1	-555.5939395	-158.8934882	-490.191154	EMD2
TGAGCATGTGCTCTCT-1	singlet	1	-800.4580887	-241.531448	-578.9813211	EMD2
TGAGCATTCCTACGAA-1	singlet	1	-2960.215916	-1062.764334	-2361.470623	EMD2
TGAGCATTCTCGTCGT-1	singlet	2	-918.0980043	-852.9979981	-322.3526641	EMD2
TGAGCGCAGAAGCGAA-1	singlet	0	-1600.108593	-3031.991731	-3438.824579	EMD2
TGAGCGCAGACGAAGA-1	singlet	1	-447.3123362	-177.1489781	-395.8507256	EMD2
TGAGCGCAGCAACTTC-1	singlet	2	-1642.314772	-1391.067741	-614.3601998	EMD2
TGAGCGCAGGTGATAT-1	singlet	1	-459.3317777	-179.0851161	-409.1031316	EMD2
TGAGCGCCACAGGATG-1	singlet	1	-1953.121496	-515.3646459	-1559.322127	EMD2
TGAGCGCCACGGCTAC-1	singlet	1	-2713.699293	-777.8804712	-2091.957574	EMD2
TGAGCGCCACTCACTC-1	doublet	2/0	-765.6014892	-1079.958678	-619.1629839	EMD2
TGAGCGCCAGAGGCTA-1	singlet	2	-472.7660587	-390.1924806	-176.0183482	EMD2
TGAGCGCCAGGCGAAT-1	singlet	0	-62.07243804	-164.7680455	-145.39594	EMD2
TGAGCGCCAGTCCCGA-1	singlet	1	-145.2234224	-54.33690463	-133.2998624	EMD2
TGAGCGCCATCGCTCT-1	singlet	1	-1847.758171	-509.8093989	-1649.981116	EMD2
TGAGGAGCAAAGGTTA-1	singlet	1	-1371.452317	-468.6173257	-1106.305636	EMD2
TGAGGAGCAGAGATTA-1	singlet	1	-2930.103659	-885.6802162	-2299.674166	EMD2
TGAGGAGCAGCTGTCG-1	singlet	2	-2170.1789	-1929.70796	-737.1645086	EMD2
TGAGGAGCAGGCGATA-1	singlet	1	-265.315325	-98.80447213	-205.8448157	EMD2
TGAGGAGCATTCTCTA-1	singlet	2	-3028.410795	-3098.033974	-947.9856865	EMD2
TGAGGAGGTCTTCAAG-1	singlet	1	-2833.59732	-800.4240369	-2047.42808	EMD2
TGAGGAGGTTGCGGAA-1	singlet	1	-1361.938865	-460.2277595	-1170.796591	EMD2
TGAGGGAAGGTGCAGT-1	singlet	1	-2776.621224	-925.6259918	-2245.679567	EMD2
TGAGGGACAAGCAGGT-1	singlet	1	-186.692648	-87.20155537	-165.4123274	EMD2
TGAGGGACAGGAGGTT-1	singlet	1	-1550.171335	-446.9549975	-1123.979986	EMD2
TGAGGGACAGTCTGGC-1	singlet	0	-410.4798874	-1376.252279	-1201.830233	EMD2
TGAGGGAGTATTTCTC-1	singlet	1	-335.0126618	-144.1814677	-307.2919568	EMD2
TGAGGGATCAGCTGTA-1	singlet	1	-979.1057455	-320.6388354	-713.2599035	EMD2
TGAGGGATCTATCGGA-1	singlet	0	-122.7871798	-293.5100705	-276.1542397	EMD2
TGAGGTTCAGAACTTC-1	singlet	1	-1189.491456	-489.7309292	-955.5350531	EMD2
TGAGGTTCATCTAGAC-1	singlet	1	-2425.214559	-801.8646616	-2169.412985	EMD2
TGAGGTTGTACGGCAA-1	singlet	1	-446.2093097	-159.719252	-373.9321849	EMD2
TGAGGTTGTCCCTAAA-1	singlet	0	-428.1198736	-1328.810152	-1251.384752	EMD2
TGAGGTTGTTCTCCCA-1	singlet	1	-4449.845682	-1135.350656	-3264.289878	EMD2
TGAGTCAAGCAGGGAG-1	singlet	1	-2225.493784	-682.4916094	-1741.48232	EMD2
TGAGTCAAGCGTCGAA-1	singlet	0	-628.8439303	-2297.561306	-2245.512793	EMD2
TGAGTCAAGGGAGAAT-1	singlet	1	-513.5498676	-204.8035292	-483.5907943	EMD2
TGAGTCACACCATATG-1	singlet	1	-2310.808451	-812.5594111	-1834.382466	EMD2
TGAGTCACAGAACATA-1	singlet	2	-383.5122058	-393.3861132	-159.5951669	EMD2
TGAGTCACAGAATCGG-1	singlet	1	-458.8527759	-160.20604	-373.1365201	EMD2
TGAGTCAGTATTGCCA-1	singlet	0	-342.8130597	-1344.578315	-1041.132851	EMD2
TGAGTCAGTTATGGTC-1	singlet	2	-656.9202336	-688.1453447	-262.9447907	EMD2
TGAGTCAGTTGGTAGG-1	singlet	1	-385.7606353	-156.5651245	-346.9543369	EMD2
TGAGTCATCCGTGGGT-1	singlet	2	-2907.405581	-2414.338636	-1244.794804	EMD2
TGAGTCATCGCCGTGA-1	singlet	0	-7.804809937	-19.50860036	-21.63181018	EMD2
TGAGTCATCGTCGCTT-1	singlet	1	-369.6892311	-146.6375242	-327.6494912	EMD2
TGAGTCATCGTGGCTG-1	singlet	2	-11.56906075	-20.3746159	-3.633472804	EMD2
TGAGTCATCTCTCTAA-1	singlet	1	-6227.309134	-1911.956659	-4806.492996	EMD2
TGAGTCATCTGTAAGC-1	singlet	1	-633.7706599	-272.7270373	-509.8509029	EMD2
TGATCAGAGGCCTTGC-1	doublet	0/1	-881.9495803	-1264.214844	-1371.784701	EMD2
TGATCAGCAACTACGT-1	singlet	1	-2424.336738	-702.9559422	-1834.322902	EMD2
TGATCAGCAGGGACTA-1	singlet	2	-1458.92694	-1236.581482	-472.2998958	EMD2
TGATCAGCAGTACTAC-1	singlet	0	-424.6338984	-1213.861091	-1101.646079	EMD2
TGATCAGGTAGGCAGT-1	singlet	1	-4615.53452	-1374.901709	-3793.346929	EMD2
TGATCAGGTATCGCAT-1	singlet	0	-1867.989365	-4161.616909	-4421.900395	EMD2
TGATCAGGTATCTTCT-1	singlet	1	-1868.954546	-469.6831788	-1431.440366	EMD2
TGATCAGGTTCGCGTG-1	singlet	1	-3390.270467	-1114.008961	-2604.826526	EMD2
TGATCAGGTTCTCACC-1	singlet	2	-1081.715264	-1130.457039	-351.5501332	EMD2
TGATCTTAGGACTAAT-1	singlet	2	-2109.978101	-1640.997794	-889.055683	EMD2
TGATCTTAGGGCAATC-1	singlet	2	-521.7760228	-407.4007972	-168.4436525	EMD2
TGATCTTCAGCTATAC-1	singlet	1	-500.9137035	-181.3632612	-455.3925752	EMD2
TGATCTTCAGGCATTT-1	singlet	1	-1722.57308	-496.0527718	-1664.060971	EMD2
TGATCTTCATGTCAGT-1	singlet	1	-1293.548652	-391.6537961	-1028.526897	EMD2
TGATCTTCATTGTGCA-1	singlet	0	-381.2152291	-856.7097515	-955.6877439	EMD2
TGATCTTGTACTGCGC-1	singlet	1	-5856.607627	-1663.75122	-4277.523672	EMD2
TGATCTTGTATCAGCT-1	singlet	1	-260.8074672	-116.9265473	-211.4432563	EMD2
TGATCTTGTCCCTCAT-1	singlet	1	-1040.861778	-408.3756027	-844.5980752	EMD2
TGATCTTGTGCTGCAC-1	singlet	0	-1149.060466	-4014.688368	-3619.581457	EMD2
TGATCTTTCCGGTAGC-1	singlet	1	-1634.743256	-551.1517864	-1293.634231	EMD2
TGATCTTTCCTTACCG-1	singlet	1	-279.7571897	-115.0891772	-210.9142441	EMD2
TGATGCACAAAGAGTT-1	singlet	0	-448.8122823	-1356.752874	-1253.541403	EMD2
TGATGCACATAACCCA-1	singlet	2	-2306.713521	-2028.929802	-824.1056321	EMD2
TGATGCAGTCAAGGCA-1	singlet	1	-472.131828	-144.0103495	-380.4735634	EMD2
TGATGCAGTCAGATTC-1	singlet	1	-397.265577	-177.7453449	-345.8978526	EMD2
TGATGGTAGCCATATC-1	singlet	1	-575.193809	-215.3016678	-545.0070101	EMD2
TGATGGTAGCGCTGCT-1	singlet	0	-873.9431993	-2118.769925	-2077.456955	EMD2
TGATGGTAGTGCGACA-1	singlet	2	-1840.488364	-1698.944943	-659.4908156	EMD2
TGATGGTAGTGGTGAC-1	singlet	2	-4070.118086	-3292.063897	-1502.339096	EMD2
TGATGGTCAAATGCTC-1	singlet	1	-46.03121775	-12.6650051	-35.63302998	EMD2
TGATGGTCAACACTAC-1	singlet	1	-2378.984391	-873.2923084	-1865.868952	EMD2
TGATGGTCAAGCGAGT-1	doublet	1/0	-1848.614289	-1566.728789	-2093.756621	EMD2
TGATGGTCACTTGAAC-1	singlet	0	-604.5040206	-1663.661097	-1629.117767	EMD2
TGATGGTGTCGCTGCA-1	singlet	0	-1762.906099	-4571.29222	-4483.253757	EMD2
TGATGGTGTCGGCTAC-1	singlet	1	-327.5340469	-130.9619535	-276.9488358	EMD2
TGATGGTTCGTCGGGT-1	singlet	0	-585.6419143	-2246.700733	-1688.748137	EMD2
TGATGGTTCTAGACCA-1	singlet	2	-165.7592511	-88.74327626	-43.991586	EMD2
TGATGGTTCTTCACGC-1	doublet	0/1	-2456.17743	-3156.634512	-3791.782453	EMD2
TGATTCTAGACGCCCT-1	doublet	0/1	-15.99196364	-16.43556993	-26.29867187	EMD2
TGATTCTAGACTTCGT-1	singlet	2	-537.936395	-579.1610817	-243.6740116	EMD2
TGATTCTAGATGGCGT-1	singlet	1	-1269.951448	-462.561137	-1075.258124	EMD2
TGATTCTAGTTAACAG-1	singlet	0	-151.8834825	-403.6973224	-374.0405104	EMD2
TGATTCTCACATTGTG-1	singlet	1	-2173.469793	-708.4404882	-1798.665403	EMD2
TGATTCTCACCCTTAC-1	singlet	1	-1886.333093	-574.7469941	-1635.484461	EMD2
TGATTCTCACCGTGCA-1	singlet	1	-1499.01107	-601.7114562	-1373.720989	EMD2
TGATTCTCATATACCG-1	singlet	2	-749.956859	-554.4145367	-228.2122401	EMD2
TGATTCTGTACGGATG-1	singlet	1	-1411.288108	-410.5787589	-1040.925565	EMD2
TGATTCTGTAGCTTAC-1	singlet	1	-291.3589199	-133.9061869	-255.8436084	EMD2
TGATTCTGTATCACCA-1	singlet	1	-2314.638749	-783.0597233	-1983.232666	EMD2
TGATTCTGTATGTCAC-1	singlet	2	-7105.929925	-5274.300102	-1589.629395	EMD2
TGATTCTGTCTGTCAA-1	singlet	0	-538.6696528	-1802.524875	-1583.954937	EMD2

TGATTCTGTGGACTGA-1	singlet	1	-5100.559717	-1404.15449	-4133.788291	EMD2
TGATTCTTCAGCGCGT-1	singlet	1	-763.33154	-212.5421078	-653.2661931	EMD2
TGATTCTTCCCAACTC-1	singlet	1	-310.8591426	-145.5439112	-301.7095573	EMD2
TGATTCTTCGCTGTTC-1	singlet	0	-145.3695016	-428.7277098	-386.8589877	EMD2
TGATTTCAGAGTGTTA-1	singlet	2	-95.31935929	-89.39374153	-28.71271617	EMD2
TGATTTCCAATACCCA-1	singlet	2	-3761.584867	-3387.618597	-1350.334296	EMD2
TGATTTCCACAGCTGC-1	doublet	0/1	-511.8323073	-757.3827948	-807.8993823	EMD2
TGATTTCCACGCGCAT-1	singlet	1	-2519.330018	-638.4702587	-1876.264664	EMD2
TGATTTCCACGCTTAA-1	singlet	1	-187.7318162	-43.11058293	-162.9655881	EMD2
TGATTTCGTATCTCGA-1	singlet	0	-161.0755654	-515.6072294	-585.2279534	EMD2
TGATTTCGTCACGCTG-1	singlet	2	-2477.280651	-2355.297216	-928.6560922	EMD2
TGATTTCGTGTCTTGA-1	singlet	1	-2035.94937	-633.434636	-1552.037579	EMD2
TGATTTCGTTTGCAGT-1	doublet	0/2	-2441.668963	-4295.452983	-3034.608251	EMD2
TGATTTCTCAGCTTGA-1	singlet	1	-1918.72362	-605.4541489	-1590.830206	EMD2
TGATTTCTCAGGAAAT-1	singlet	0	-101.7711119	-327.9579835	-338.5016101	EMD2
TGATTTCTCATACAGC-1	singlet	1	-246.1358581	-102.8720998	-194.9534267	EMD2
TGATTTCTCTGTGCAA-1	singlet	1	-300.2413112	-120.9180512	-236.5866132	EMD2
TGATTTCTCTTGAGCA-1	singlet	0	-1137.68345	-3310.965484	-3266.47208	EMD2
TGCACGGAGATGGCAC-1	doublet	0/1	-1327.215707	-2051.232608	-2514.994812	EMD2
TGCACGGAGGCTGAAC-1	singlet	0	-687.8391518	-3909.925452	-3127.839613	EMD2
TGCACGGAGTAGAATC-1	singlet	2	-2933.943132	-2193.189112	-935.0769949	EMD2
TGCACGGCAACTCATG-1	singlet	1	-395.7490939	-138.0896651	-316.7976365	EMD2
TGCACGGCAGGTTCGC-1	singlet	2	-1684.416339	-1396.550246	-548.5416316	EMD2
TGCACGGCAGTCACGC-1	singlet	1	-1612.613505	-574.7328844	-1407.710593	EMD2
TGCACGGCATAACGGG-1	singlet	0	-564.0367919	-1642.693971	-1409.529444	EMD2
TGCACGGCATGTCTAG-1	singlet	1	-604.7922235	-240.1372396	-480.0916148	EMD2
TGCACGGCATTACTCT-1	singlet	1	-2331.497891	-659.1285044	-2071.951413	EMD2
TGCACGGGTGCTGCAC-1	singlet	2	-3704.536622	-3000.024816	-1071.439614	EMD2
TGCACGGGTGCTGTCG-1	singlet	1	-404.9550122	-93.74647509	-294.95094	EMD2
TGCACGGGTTATCTTC-1	singlet	0	-59.68249658	-245.5150732	-166.7285836	EMD2
TGCACGGTCAATGCAC-1	singlet	2	-1508.621584	-1391.556376	-596.4923478	EMD2
TGCACGGTCATGTCAG-1	singlet	2	-3748.830824	-2828.427357	-1216.478191	EMD2
TGCACGGTCTTAATCC-1	singlet	2	-12.57860147	-10.60870413	-4.353154318	EMD2
TGCAGATAGAACGCGT-1	singlet	0	-912.5322923	-2803.338333	-2690.789803	EMD2
TGCAGATAGAGCCTGA-1	singlet	1	-1648.699715	-544.27186	-1331.247761	EMD2
TGCAGATAGATGGGCT-1	singlet	2	-3302.580488	-2834.908294	-1021.963166	EMD2
TGCAGATAGCGCACAA-1	singlet	1	-1357.391961	-591.247584	-1208.133814	EMD2
TGCAGATAGTATGATG-1	singlet	0	-357.3279225	-1022.626722	-966.2635738	EMD2
TGCAGATCACCCTGTT-1	doublet	45293	-90.42642609	-42.0786521	-46.81827196	EMD2
TGCAGATCAGCGGATA-1	singlet	0	-271.5514953	-697.5074487	-647.8894093	EMD2
TGCAGATCATTGTAGC-1	singlet	0	-1511.403481	-5513.655068	-5254.151842	EMD2
TGCAGATGTAACGCGA-1	singlet	0	-330.2114495	-1087.119601	-955.4910732	EMD2
TGCAGATGTTACCTTT-1	singlet	0	-360.1667033	-628.1896446	-584.632485	EMD2
TGCAGATTCTCCACTG-1	singlet	1	-746.2178106	-306.4338685	-667.1993893	EMD2
TGCAGGCAGTTAACAG-1	singlet	1	-1062.954797	-400.4538707	-920.3126911	EMD2
TGCAGGCCAAATGGCG-1	singlet	1	-454.0664448	-216.2751103	-421.0298211	EMD2
TGCAGGCCAACGCATT-1	singlet	2	-1449.083779	-1238.954756	-555.8696942	EMD2
TGCAGGCCAGTATGAA-1	doublet	45323	-3195.571687	-1993.408117	-1718.659851	EMD2
TGCAGGCCATCCTTCG-1	singlet	0	-969.5319048	-3119.412573	-2870.22592	EMD2
TGCAGGCGTATCACGT-1	doublet	45293	-710.167732	-346.3011899	-565.588221	EMD2
TGCAGGCGTGGATCGA-1	singlet	1	-354.1464971	-107.9396963	-256.4295026	EMD2
TGCAGGCTCAGAGCAG-1	singlet	1	-293.9739815	-121.7639525	-271.0304892	EMD2
TGCAGGCTCCGATCGG-1	singlet	0	-1448.633259	-3517.687487	-3442.711253	EMD2
TGCAGGCTCGCGATCG-1	singlet	0	-587.8704195	-1507.968068	-1470.90899	EMD2
TGCAGGCTCTCTAAGG-1	singlet	2	-626.3028196	-638.4369025	-312.0647147	EMD2
TGCAGTACAACGATCT-1	singlet	2	-71.89077027	-44.98346392	-22.34203229	EMD2
TGCAGTACAATCGTCA-1	singlet	1	-2504.381863	-715.4875275	-2110.696519	EMD2
TGCAGTAGTCATGGCC-1	singlet	0	-537.2045967	-2336.445932	-2007.548234	EMD2
TGCAGTAGTCTGCATA-1	singlet	1	-1429.277532	-461.0858715	-1123.966697	EMD2
TGCAGTAGTCTGTGGC-1	singlet	2	-1845.421278	-1646.763824	-445.2601669	EMD2
TGCAGTAGTTCTCTCG-1	singlet	0	-1210.541173	-4173.837676	-3990.510865	EMD2
TGCAGTATCATGGCCG-1	singlet	1	-4554.489673	-1546.533588	-3581.66624	EMD2
TGCATCCAGAGCTGCA-1	doublet	0/2	-2071.736068	-2230.573136	-2174.198712	EMD2
TGCATCCAGCAGTCTT-1	singlet	2	-1369.746014	-1346.924185	-442.0106152	EMD2
TGCATCCAGCTCGACC-1	singlet	0	-1464.197392	-2770.770386	-3182.551539	EMD2
TGCATCCCAAGTCCCG-1	singlet	1	-377.4786277	-166.7580231	-319.1153729	EMD2
TGCATCCGTGATACTC-1	singlet	1	-3552.083863	-1157.630236	-2831.907719	EMD2
TGCATCCGTGCAACGA-1	singlet	2	-432.4733132	-339.3520623	-110.356682	EMD2
TGCATCCGTGCTCGTG-1	doublet	2/0	-1091.758268	-1380.827962	-880.0478897	EMD2
TGCATCCTCAGGAAGC-1	singlet	2	-998.8237324	-919.6450366	-391.030877	EMD2
TGCATCCTCGGCGATC-1	singlet	1	-126.0298616	-50.94274408	-96.54122334	EMD2
TGCATCCTCTGGGCCA-1	singlet	0	-98.27580876	-244.9654528	-263.6950894	EMD2
TGCATGAAGCTGAGTG-1	singlet	1	-2445.916684	-583.0380626	-1632.08964	EMD2
TGCATGACAAACTCTG-1	singlet	1	-330.1643439	-168.904451	-322.5839672	EMD2
TGCATGAGTACGATGG-1	singlet	1	-1898.255733	-667.4821385	-1565.248477	EMD2
TGCATGAGTAGCGTTT-1	singlet	1	-785.2708145	-260.6221751	-678.2032557	EMD2
TGCATGAGTGTAAACA-1	singlet	0	-339.2502037	-1014.739849	-1056.727968	EMD2
TGCATGAGTGTATACC-1	singlet	2	-5.3928651	-4.891294801	-1.212086438	EMD2
TGCATGAGTTGACTGT-1	singlet	1	-1056.79503	-435.1672326	-907.7483888	EMD2
TGCATGATCCTCTAAT-1	singlet	1	-256.6637803	-127.5004406	-240.6390808	EMD2
TGCCGAGAGCGCTTCG-1	singlet	1	-525.2674661	-209.078642	-431.3127689	EMD2
TGCCGAGCAACCGCTG-1	singlet	1	-549.9273455	-180.2609259	-466.2264384	EMD2
TGCCGAGCAGGAGGTT-1	singlet	0	-73.76145598	-182.2075336	-170.2434573	EMD2
TGCCGAGGTGCATCTA-1	singlet	0	-505.7869817	-1771.875376	-1376.448297	EMD2
TGCCGAGTCCGCGAGT-1	singlet	1	-495.7067871	-198.6954517	-409.6977074	EMD2
TGCCGAGTCTCAGAAC-1	singlet	1	-677.7346051	-220.9366375	-659.5022785	EMD2
TGCGACGAGCCTGGAA-1	singlet	1	-895.4387339	-335.3076012	-763.9688814	EMD2
TGCGACGAGCGATGGT-1	singlet	1	-2598.170432	-777.8088945	-2062.086189	EMD2
TGCGACGAGCTCAGAG-1	singlet	1	-497.4652062	-143.1799876	-351.6505376	EMD2
TGCGACGAGTCGGGAT-1	doublet	45293	-2263.929214	-1088.910658	-1672.583318	EMD2
TGCGACGCAGAGGCAT-1	singlet	1	-2287.981123	-703.4241037	-2020.689675	EMD2
TGCGACGCAGTGCCTG-1	singlet	2	-425.1228957	-408.8786043	-185.4808895	EMD2
TGCGACGCATTGCAAC-1	unassigned	2	-8.398276916	-6.573417207	-5.666553785	EMD2
TGCGACGGTAGGAGGG-1	singlet	1	-1642.676907	-569.3106407	-1286.875094	EMD2
TGCGACGGTTCAATCG-1	singlet	1	-2486.960333	-820.9476841	-1937.972728	EMD2
TGCGACGTCACTCTTA-1	singlet	1	-953.8547822	-259.9444788	-811.4809385	EMD2
TGCGACGTCGCCAGAC-1	singlet	1	-746.7356117	-346.0827155	-637.3861673	EMD2
TGCGACGTCTAAGGAA-1	singlet	2	-540.564123	-515.120407	-165.6732864	EMD2
TGCGACGTCTTTACAC-1	singlet	1	-1154.00653	-377.4473264	-896.7081886	EMD2
TGCGATAAGATGCTAA-1	singlet	1	-393.137842	-171.1738198	-387.2198947	EMD2
TGCGATAAGGGCAATC-1	singlet	0	-57.77732698	-134.1454679	-182.9593636	EMD2
TGCGATAAGGTAGATT-1	singlet	1	-253.4463624	-98.76754493	-253.9595533	EMD2
TGCGATAAGTAGGAAG-1	singlet	0	-968.7735446	-2821.873825	-2771.757794	EMD2
TGCGATAGTCTTCGAA-1	singlet	2	-2126.713444	-2317.423945	-749.8507161	EMD2
TGCGATAGTGCACGCT-1	singlet	1	-355.4591068	-132.6829806	-333.5512854	EMD2
TGCGATAGTGCTTATG-1	singlet	1	-2307.483805	-748.5687559	-2028.933576	EMD2
TGCGATAGTTTGGGAG-1	singlet	1	-1569.035826	-432.3352495	-1309.360758	EMD2
TGCGATATCAAGTGGG-1	singlet	0	-4.376472959	-9.017884326	-11.84973678	EMD2
TGCGATATCTACTATC-1	singlet	2	-1547.01155	-1207.209954	-494.7124399	EMD2
TGCGGCAAGCTTACGT-1	singlet	1	-2364.117775	-711.7401228	-1913.974068	EMD2

TGCGGCAAGGACACTG-1	singlet	0	-461.3109428	-1129.457714	-1252.387945	EMD2
TGCGGCAAGTCTTCCC-1	singlet	2	-2519.690252	-2583.936979	-867.4165194	EMD2
TGCGGCAAGTGAACAT-1	singlet	0	-865.0595912	-2673.095733	-2658.91082	EMD2
TGCGGCACACGCTGAC-1	singlet	2	-4045.638166	-3317.257401	-1285.019691	EMD2
TGCGGCAGTCAGACTT-1	singlet	1	-356.5350884	-145.8695031	-309.1882387	EMD2
TGCGGCAGTGTGTTTG-1	singlet	1	-1633.23168	-521.8760779	-1489.014663	EMD2
TGCGGCATCACTCACC-1	singlet	1	-376.0138132	-143.1090978	-342.6221557	EMD2
TGCGGCATCCGGCAAC-1	singlet	0	-84.39048014	-243.1228695	-260.7133374	EMD2
TGCGGGTAGAAACCCG-1	singlet	0	-49.45256517	-163.8972159	-159.5640628	EMD2
TGCGGGTAGAAGTCAT-1	singlet	2	-2583.980397	-2130.486991	-767.0203925	EMD2
TGCGGGTAGAGCATAT-1	singlet	1	-1691.57361	-429.2159643	-1269.697932	EMD2
TGCGGGTAGGATTTCC-1	singlet	1	-690.5170958	-285.9628302	-620.0832063	EMD2
TGCGGGTAGGTAGCCA-1	singlet	1	-306.7833863	-144.3192661	-281.2433079	EMD2
TGCGGGTCACGAGGAT-1	singlet	1	-2643.626165	-940.0621258	-2112.231569	EMD2
TGCGGGTCATGATGCT-1	singlet	1	-910.381235	-296.4021714	-728.6752362	EMD2
TGCGGGTGTGATACCT-1	singlet	1	-143.4806387	-67.21300901	-124.9278409	EMD2
TGCGGGTGTGCATGTT-1	singlet	0	-585.8006292	-2239.87464	-1988.820262	EMD2
TGCGGGTGTGTTCAGT-1	singlet	0	-490.4873258	-1577.372045	-1357.800861	EMD2
TGCGGGTGTTATGGTC-1	singlet	0	-553.5911715	-1745.264985	-1571.038809	EMD2
TGCGGGTGTTGTGGAG-1	singlet	2	-2996.757565	-2154.294212	-996.1157329	EMD2
TGCGGGTTCATCACAG-1	singlet	2	-5954.785839	-4950.224985	-1882.922968	EMD2
TGCGGGTTCTGCTCTG-1	singlet	1	-1098.763355	-389.7630224	-868.3911434	EMD2
TGCTCCAAGCCTAGGA-1	singlet	1	-1578.353999	-539.784433	-1342.509173	EMD2
TGCTCCAAGGAAAGTG-1	singlet	2	-1930.22774	-1694.523286	-666.082067	EMD2
TGCTCCAAGTTCACTG-1	singlet	1	-50.78776208	-22.2879342	-27.62580915	EMD2
TGCTCCACAACCAATC-1	singlet	1	-905.9770989	-303.6395851	-794.0118744	EMD2
TGCTCCACAACCCGCA-1	singlet	1	-468.3937192	-206.5941736	-393.4515414	EMD2
TGCTCCACAATGCAAA-1	singlet	1	-2047.200594	-619.4494657	-1540.997256	EMD2
TGCTCCACATAAGATG-1	singlet	2	-2699.434507	-2211.272776	-905.2454041	EMD2
TGCTCCAGTAGCTGCC-1	singlet	1	-1113.262351	-340.3645388	-1133.944169	EMD2
TGCTCCAGTAGTGATA-1	singlet	1	-1086.409914	-353.9615518	-847.2343316	EMD2
TGCTCCAGTCAGTCTA-1	singlet	1	-4337.475052	-1400.157121	-3656.083886	EMD2
TGCTCCAGTGAAGCTG-1	singlet	2	-1127.661352	-1044.714233	-397.4542265	EMD2
TGCTCCAGTGCAATAA-1	singlet	1	-225.3182544	-83.50408249	-180.8210618	EMD2
TGCTCCAGTGGACCAA-1	singlet	1	-776.0853211	-281.1435449	-697.4632234	EMD2
TGCTCCAGTGGATCAG-1	singlet	1	-650.0361429	-244.1918094	-492.9849319	EMD2
TGCTCCATCAACCCGG-1	singlet	0	-1250.356255	-4438.318141	-4388.863857	EMD2
TGCTCGTAGACATATG-1	singlet	0	-70.25858434	-201.6299315	-196.3696048	EMD2
TGCTCGTAGAGGCGGA-1	singlet	1	-901.6038994	-323.0587701	-726.824372	EMD2
TGCTCGTAGTCGGGAT-1	singlet	1	-5197.258218	-1410.26804	-3873.447056	EMD2
TGCTCGTCAACTCGAT-1	singlet	1	-467.3164525	-192.8164035	-383.9181991	EMD2
TGCTCGTCAGTAACCT-1	singlet	1	-1656.882635	-457.9982821	-1288.670412	EMD2
TGCTCGTCAGTTGTTG-1	singlet	1	-3576.707765	-1122.408779	-2728.367104	EMD2
TGCTCGTGTCGAATTC-1	singlet	1	-956.9126931	-286.2382816	-862.3122629	EMD2
TGCTCGTGTCTGTCAA-1	singlet	1	-5793.632549	-1498.613875	-4248.548796	EMD2
TGCTCGTGTGGAGGTT-1	singlet	0	-92.08235858	-234.0932505	-217.2646775	EMD2
TGCTCGTGTTCGTAAC-1	singlet	0	-2042.741111	-7615.035195	-7380.02151	EMD2
TGCTCGTGTTCTCGCT-1	singlet	1	-1679.052137	-453.620318	-1386.355417	EMD2
TGCTCGTGTTGGTACT-1	singlet	1	-172.1005051	-75.8865386	-146.1834585	EMD2
TGCTCGTTCACTGGTA-1	singlet	2	-1848.29893	-1527.892942	-624.0311111	EMD2
TGCTCGTTCCAATGCA-1	singlet	1	-3098.845516	-818.2440816	-2648.403947	EMD2
TGCTCGTTCCTTTGAT-1	singlet	1	-4425.088318	-1138.311793	-3334.202238	EMD2
TGCTCGTTCGAGCCTG-1	singlet	2	-1366.181464	-1108.936718	-483.0454342	EMD2
TGCTCGTTCTGTGTGA-1	singlet	0	-81.58017681	-235.348709	-199.7738294	EMD2
TGCTGAAAGACCTCCG-1	singlet	1	-646.3206486	-246.6099655	-515.5679427	EMD2
TGCTGAAAGAGGTGCT-1	doublet	45293	-16.58405174	-13.744043	-16.31116619	EMD2
TGCTGAAAGCAGGTCA-1	singlet	0	-881.6809528	-2882.839829	-2663.577622	EMD2
TGCTGAAAGCTTGTGT-1	singlet	0	-59.98101393	-161.4927005	-127.2355648	EMD2
TGCTGAACAGCGCGTT-1	singlet	2	-92.79651314	-94.66598965	-40.42122949	EMD2
TGCTGAACAGGTTACT-1	singlet	1	-1361.25736	-364.7744599	-1099.997132	EMD2
TGCTGAACATGTACGT-1	singlet	0	-111.1127215	-353.2577042	-244.5451481	EMD2
TGCTGAATCGGACGTC-1	singlet	2	-1141.923053	-1069.807303	-414.8298712	EMD2
TGCTGAATCGTACACA-1	singlet	1	-1699.710721	-652.9624738	-1453.841526	EMD2
TGCTGAATCTTGGAAC-1	singlet	0	-833.5142879	-2688.906133	-2726.255289	EMD2
TGCTTCGAGAGTCAAT-1	singlet	2	-1164.15739	-1115.961964	-375.2147383	EMD2
TGCTTCGAGTCAGAGC-1	singlet	1	-1876.828624	-461.4238131	-1634.006188	EMD2
TGCTTCGCAATAGTAG-1	singlet	0	-1208.402957	-3394.333585	-3279.573843	EMD2
TGCTTCGCACTACTTT-1	singlet	0	-145.2088318	-381.0643391	-352.0820333	EMD2
TGCTTCGCACTGGCGT-1	singlet	1	-1558.059431	-523.5935019	-1314.666288	EMD2
TGCTTCGCATCGGATT-1	singlet	2	-2417.967365	-2299.32531	-730.8214877	EMD2
TGCTTCGGTAGTCACT-1	singlet	1	-360.0577516	-127.3731394	-307.5329105	EMD2
TGCTTCGGTATCACGT-1	singlet	1	-4323.983638	-1233.289773	-3712.338611	EMD2
TGCTTCGTCCACAGGC-1	singlet	2	-246.3456041	-257.5716859	-77.31839348	EMD2
TGCTTGCAGAGGGTAA-1	singlet	1	-111.2454557	-58.59300801	-119.1654457	EMD2
TGCTTGCCAAGCGAGT-1	singlet	2	-1096.196952	-978.9421623	-403.7317235	EMD2
TGCTTGCGTTGTGGCC-1	singlet	2	-380.1205445	-380.7868918	-171.9429279	EMD2
TGCTTGCTCGAGCCTG-1	doublet	0/2	-3287.03721	-6481.058169	-5089.480612	EMD2
TGCTTGCTCTTCGATT-1	singlet	1	-255.757021	-65.10009354	-206.9309976	EMD2
TGGAACTCAAGACGGT-1	singlet	1	-1498.245603	-465.0411957	-1135.148052	EMD2
TGGAACTCAGCAGAAC-1	singlet	1	-1249.456934	-404.1746595	-1069.463518	EMD2
TGGAACTCAGCTCATA-1	singlet	0	-706.8608207	-2063.445028	-1790.113392	EMD2
TGGAACTCAGTCGGTC-1	singlet	0	-981.5004019	-2049.858887	-1797.59317	EMD2
TGGAACTGTACGCGTC-1	singlet	0	-279.2714632	-707.2605008	-637.8749939	EMD2
TGGAACTGTCGCGTTG-1	singlet	0	-1255.295878	-3778.881705	-3595.522992	EMD2
TGGAACTTCAGACCCG-1	doublet	45323	-3773.452106	-2610.869716	-1881.359258	EMD2
TGGAACTTCGCCAGTG-1	doublet	2/0	-2807.939359	-3480.445906	-2205.602241	EMD2
TGGAACTTCGCGCCAA-1	singlet	2	-2964.479913	-2483.744032	-921.4623319	EMD2
TGGAACTTCTGTGCAA-1	singlet	1	-651.5411849	-220.9808998	-486.0953122	EMD2
TGGAGAGAGAGGCTGT-1	singlet	1	-359.9926021	-110.9873722	-299.0511577	EMD2
TGGAGAGAGCAGCAGT-1	unassigned	1	-8.568817978	-5.405914677	-5.499556442	EMD2
TGGAGAGAGTCACGCC-1	doublet	45323	-69.54455122	-73.05046208	-65.90909002	EMD2
TGGAGAGCACATGAAA-1	singlet	2	-721.7380525	-613.3116041	-296.0143288	EMD2
TGGAGAGCACTGGACC-1	singlet	2	-121.7684672	-73.90166312	-33.76285194	EMD2
TGGAGAGGTGTATACC-1	singlet	2	-2450.748195	-1751.720596	-732.2603226	EMD2
TGGAGAGTCGAACCAT-1	doublet	45293	-2763.162471	-1387.668685	-2047.932614	EMD2
TGGAGAGTCGGTAGGA-1	singlet	1	-114.6151608	-61.37126533	-116.999401	EMD2
TGGAGAGTCTTACCGC-1	singlet	1	-1718.207724	-567.5419717	-1571.332162	EMD2
TGGAGGAAGGTTACAA-1	singlet	1	-3603.792848	-1084.186776	-2754.922249	EMD2
TGGAGGAAGTGGTGGT-1	singlet	2	-170.1653733	-154.1476561	-61.81809473	EMD2
TGGAGGACAACACGAG-1	singlet	0	-147.170961	-303.6752532	-295.0818951	EMD2
TGGAGGACAAGACCTT-1	singlet	0	-714.563534	-2277.742237	-2146.228115	EMD2
TGGAGGACAATATCCG-1	singlet	1	-1395.064156	-447.1659281	-935.2366868	EMD2
TGGAGGACACGGTGCT-1	singlet	1	-1739.26721	-567.196997	-1614.999096	EMD2
TGGAGGACACTCCGGA-1	singlet	1	-2628.696281	-685.5046886	-2004.057171	EMD2
TGGAGGACAGTACTAC-1	singlet	0	-767.1576611	-2359.631778	-2222.467338	EMD2
TGGAGGACATGGGCAA-1	singlet	0	-508.3460259	-1528.509345	-1450.96565	EMD2
TGGAGGAGTAAGCGGT-1	singlet	1	-1192.335468	-370.8254082	-901.7978374	EMD2
TGGAGGAGTAGCACGA-1	singlet	0	-745.7507593	-2307.778851	-2154.747052	EMD2
TGGAGGAGTCCTTAAG-1	singlet	1	-914.3604271	-377.5890591	-837.2565161	EMD2
TGGAGGAGTCTACGTA-1	singlet	0	-520.1839133	-1576.476984	-1445.700678	EMD2

TGGAGGAGTGGTTTAC-1	singlet	2	-951.8575613	-877.964774	-358.6602886	EMD2
TGGAGGATCCATTGTT-1	singlet	2	-4362.325316	-4411.593092	-1241.252822	EMD2
TGGAGGATCGCAGAGA-1	singlet	1	-227.9856993	-71.85454423	-182.8583268	EMD2
TGGAGGATCTAGGCAT-1	singlet	0	-204.9976804	-518.3066034	-524.8135506	EMD2
TGGAGGATCTATGTGG-1	singlet	1	-209.9842553	-54.87453633	-158.3563422	EMD2
TGGATCAAGAAGCGCT-1	singlet	1	-4655.27125	-1535.024155	-3846.779805	EMD2
TGGATCAAGCTGCCTG-1	singlet	1	-383.6607058	-136.8991216	-342.9428444	EMD2
TGGATCAAGGACAGTC-1	singlet	2	-945.0733788	-670.5051408	-324.8892996	EMD2
TGGATCAAGTATAGAC-1	singlet	1	-943.3973828	-321.3697461	-682.0226702	EMD2
TGGATCAAGTTGTAAG-1	singlet	1	-1553.127278	-492.5970756	-1392.315546	EMD2
TGGATCACAATATCCG-1	singlet	0	-1429.318231	-4727.439438	-4199.280824	EMD2
TGGATCACAGAAGCTG-1	singlet	1	-2249.418524	-713.4417981	-1760.341148	EMD2
TGGATCACAGTCACGC-1	singlet	2	-4501.297338	-3693.324279	-1471.95147	EMD2
TGGATCACATTCGGGC-1	singlet	1	-2032.952295	-677.1355848	-1651.39152	EMD2
TGGATCAGTCACAGTT-1	singlet	0	-45.4517847	-130.8800547	-101.5143425	EMD2
TGGATCAGTCCTCATC-1	singlet	1	-1191.376208	-411.6246783	-1001.809214	EMD2
TGGATCAGTCGAGATG-1	singlet	1	-191.6919894	-71.26040835	-134.1278494	EMD2
TGGATCAGTGCATACT-1	singlet	0	-950.6286775	-4038.862527	-4278.613774	EMD2
TGGATCAGTTAAGGAT-1	singlet	1	-505.7052981	-215.2409865	-441.2696786	EMD2
TGGATCATCTCATTTG-1	singlet	1	-4673.8935	-1471.053516	-3505.301441	EMD2
TGGATGTAGAGGGTGG-1	singlet	1	-1319.870717	-434.6820844	-1176.080412	EMD2
TGGATGTAGCAGGCAT-1	singlet	1	-1176.131425	-398.2897919	-924.1770665	EMD2
TGGATGTAGTCTAACC-1	singlet	2	-2511.866684	-2269.992211	-819.7568244	EMD2
TGGATGTCAATACAGA-1	singlet	0	-408.7819906	-1195.395542	-1278.334373	EMD2
TGGATGTCACCGTCGA-1	singlet	1	-887.1945733	-332.3470191	-736.1522105	EMD2
TGGATGTCACTTTATC-1	singlet	2	-430.6935304	-305.0101464	-151.1691049	EMD2
TGGATGTCAGACTCTA-1	singlet	2	-845.5021937	-837.0811909	-243.5697252	EMD2
TGGATGTCAGCCTTCT-1	singlet	2	-3133.944511	-2883.946317	-1294.326554	EMD2
TGGATGTCAGTAACGG-1	singlet	2	-2681.983685	-2157.060105	-877.9868693	EMD2
TGGATGTGTATGACAA-1	singlet	2	-415.2868585	-481.7220118	-223.0397467	EMD2
TGGATGTGTCCGCAGT-1	singlet	0	-277.4659604	-831.1455146	-704.0137146	EMD2
TGGATGTGTGTGTCGC-1	singlet	0	-524.2703442	-1718.718489	-1497.094642	EMD2
TGGATGTTCCGATCTC-1	singlet	2	-1367.569872	-1031.353679	-395.9402441	EMD2
TGGATGTTCCGTATAG-1	singlet	1	-721.9878232	-243.1906149	-590.4491573	EMD2
TGGATGTTCCTGTACC-1	singlet	0	-838.2831883	-2441.388188	-2397.204672	EMD2
TGGATGTTCGTCAACA-1	singlet	0	-451.8841521	-1710.758701	-1628.110716	EMD2
TGGATGTTCTCACGAA-1	singlet	1	-966.3267988	-368.2438288	-813.1963251	EMD2
TGGCGTGAGACGATAT-1	doublet	45323	-630.0099295	-339.9215091	-339.1770321	EMD2
TGGCGTGAGAGTCTTC-1	singlet	1	-4302.466467	-1133.448401	-3232.043756	EMD2
TGGCGTGAGCCACTCG-1	singlet	1	-178.7242557	-78.76002922	-198.8959318	EMD2
TGGCGTGAGCCTCACG-1	singlet	2	-2096.744113	-1883.616937	-739.3445152	EMD2
TGGCGTGAGCTAATGA-1	singlet	0	-733.0703712	-3038.067013	-2692.990296	EMD2
TGGCGTGCAAAGGATT-1	singlet	2	-714.1868199	-753.6580209	-300.1352833	EMD2
TGGCGTGCACACCTGG-1	singlet	1	-180.5903601	-71.60675174	-157.7937454	EMD2
TGGCGTGGTAGGTACG-1	singlet	2	-2026.503744	-1591.715367	-622.1081653	EMD2
TGGCGTGGTGAGCAGT-1	singlet	1	-114.87137	-24.32916006	-78.23458387	EMD2
TGGCGTGGTGTCGATT-1	singlet	0	-407.6175635	-1385.916285	-1263.35123	EMD2
TGGCGTGGTTAGAAAC-1	singlet	1	-3279.217723	-1018.740906	-2703.804856	EMD2
TGGCGTGGTTCTCAGA-1	singlet	1	-380.4143686	-168.5924104	-376.130095	EMD2
TGGGAAGAGCCGATCC-1	singlet	0	-157.7457484	-395.7516061	-381.7870459	EMD2
TGGGAAGAGTGAACAT-1	singlet	1	-449.9964728	-93.28674153	-336.2632768	EMD2
TGGGAAGAGTGGTGAC-1	singlet	1	-655.085241	-209.4444927	-430.7064573	EMD2
TGGGAAGCAACCGATT-1	singlet	1	-266.0482626	-109.5124472	-219.0601073	EMD2
TGGGAAGCACAACCGC-1	singlet	0	-1278.456689	-4091.764977	-4066.118213	EMD2
TGGGAAGCACGTAACT-1	singlet	1	-835.3284392	-316.4833235	-705.4913645	EMD2
TGGGAAGCAGAGTCAG-1	singlet	1	-323.6310268	-81.1432164	-245.1890364	EMD2
TGGGAAGCAGCCTACG-1	singlet	1	-125.8512405	-67.5547415	-115.5358404	EMD2
TGGGAAGCAGGCACTC-1	singlet	1	-606.2633695	-217.6406125	-510.5107322	EMD2
TGGGAAGCAGGTCCGT-1	singlet	2	-294.6576366	-280.5012983	-101.3094337	EMD2
TGGGAAGCATAACCCA-1	singlet	0	-508.6507597	-1652.520783	-1399.47341	EMD2
TGGGAAGGTAGGAGTC-1	singlet	0	-613.0476915	-1839.100706	-1794.289435	EMD2
TGGGAAGGTTAGAAAC-1	singlet	1	-47.47273895	-10.22499486	-54.59575304	EMD2
TGGGAAGTCATTCATC-1	unassigned	45293	-68.38603621	-30.24478378	-47.18065477	EMD2
TGGGAAGTCGCACGAC-1	singlet	1	-732.5683342	-279.9857384	-630.695251	EMD2
TGGGAAGTCGCGGACT-1	singlet	1	-574.6126526	-171.2214452	-414.5036134	EMD2
TGGGAGAAGCTGGAGT-1	singlet	1	-1606.818533	-478.7675534	-1318.554484	EMD2
TGGGAGAAGTATGACA-1	singlet	0	-412.2351269	-1323.888096	-1152.04055	EMD2
TGGGAGAGTAGGCTCC-1	singlet	1	-2641.625156	-826.1271313	-2000.245994	EMD2
TGGGAGAGTGATAGTA-1	singlet	1	-2694.876143	-643.6778004	-1859.583133	EMD2
TGGGAGATCAAACTGC-1	singlet	1	-1616.97228	-585.7065906	-1310.426663	EMD2
TGGGAGATCACTGGTA-1	singlet	2	-929.0082448	-821.8699453	-317.6446955	EMD2
TGGGAGATCCAAGCAT-1	singlet	0	-119.4277027	-408.6511927	-350.42081	EMD2
TGGGAGATCCTTATGT-1	singlet	1	-1192.996311	-344.1318894	-759.6257505	EMD2
TGGGATTAGCCTCACG-1	singlet	1	-259.8243144	-100.5753776	-203.2833362	EMD2
TGGGATTAGCGATGGT-1	singlet	1	-364.2918649	-142.452491	-329.1073835	EMD2
TGGGATTAGTGCACAG-1	doublet	0/1	-2818.338042	-4391.420431	-4729.925619	EMD2
TGGGATTAGTTTCGGT-1	singlet	0	-556.6980403	-1722.783642	-1552.534602	EMD2
TGGGATTCATCCGGTG-1	singlet	0	-962.8651661	-3235.009113	-2940.157622	EMD2
TGGGATTCATGTTACG-1	singlet	1	-394.786762	-166.3191792	-373.1855724	EMD2
TGGGATTGTAACGCGA-1	singlet	0	-847.0725304	-2632.178344	-2704.036251	EMD2
TGGGATTGTCACTCAA-1	singlet	1	-1736.688447	-547.92627	-1178.167238	EMD2
TGGGATTGTCGTTATG-1	singlet	1	-2513.870199	-785.3301146	-2145.988984	EMD2
TGGGATTGTGCCTATA-1	singlet	1	-2184.227319	-830.4171291	-1837.262915	EMD2
TGGGATTGTGGATTTC-1	singlet	1	-416.7073981	-140.1175544	-283.198729	EMD2
TGGGATTGTGGCAGAT-1	singlet	1	-2434.212553	-700.2939055	-2099.587037	EMD2
TGGGATTGTGTGTGTT-1	singlet	2	-176.3851983	-135.0219873	-67.71465424	EMD2
TGGGATTTCCGATCTC-1	singlet	1	-5020.676729	-1689.32679	-4260.426906	EMD2
TGGGATTTCCGCAGTG-1	singlet	2	-382.9153249	-326.9160547	-117.4610517	EMD2
TGGGATTTCGGAATTC-1	singlet	0	-587.582467	-2001.649286	-1873.514432	EMD2
TGGGATTTCTCCGATC-1	singlet	2	-3132.252463	-2361.252907	-833.1594762	EMD2
TGGGCGTAGAGCTGCA-1	singlet	1	-1855.820119	-451.9607991	-1884.025209	EMD2
TGGGCGTAGGACTAAT-1	singlet	1	-563.4453734	-157.0563038	-438.6524091	EMD2
TGGGCGTAGTCAATCC-1	singlet	0	-449.5597491	-1492.26002	-1431.116977	EMD2
TGGGCGTAGTGCAAAT-1	singlet	1	-2066.5085	-567.215563	-1880.031699	EMD2
TGGGCGTAGTTTGGCT-1	singlet	1	-116.3670201	-42.82238368	-113.1264242	EMD2
TGGGCGTCAGCTGTTA-1	singlet	2	-287.9473057	-317.9628628	-138.982906	EMD2
TGGGCGTGTCAAGTTC-1	singlet	2	-3369.712513	-2740.411568	-950.3298889	EMD2
TGGGCGTGTCGTATTG-1	singlet	1	-250.4973599	-91.5430686	-214.3964541	EMD2
TGGGCGTGTTAGGACG-1	singlet	0	-666.8168305	-2554.435694	-2407.598505	EMD2
TGGGCGTTCACCATGA-1	singlet	0	-209.2523299	-604.5118533	-605.4375781	EMD2
TGGGCGTTCAGACCCG-1	singlet	0	-146.7851725	-397.1515133	-311.4816251	EMD2
TGGGCGTTCGGCTGTG-1	singlet	1	-513.7001487	-185.2171379	-446.1630621	EMD2
TGGGCGTTCGTCTACC-1	singlet	1	-2440.691339	-445.0039916	-1534.295545	EMD2
TGGGCGTTCGTGACTA-1	singlet	1	-1877.132985	-610.8320408	-1399.3961	EMD2
TGGGCGTTCTCAACGA-1	singlet	1	-2427.149755	-768.8487302	-1967.881637	EMD2
TGGGCTGAGCATTGAA-1	singlet	2	-324.6499846	-259.9675967	-113.0764127	EMD2
TGGGCTGAGGTAGCAC-1	singlet	1	-249.2181541	-81.8131285	-192.4513948	EMD2
TGGGCTGAGTATTGCC-1	singlet	1	-1513.879928	-414.2602627	-1258.650664	EMD2
TGGGCTGCACATGAAA-1	singlet	2	-690.2499336	-675.0379175	-287.8637025	EMD2
TGGGCTGCACGGTAGA-1	singlet	2	-404.0723844	-418.5644266	-155.4640448	EMD2

TGGGCTGCACGTATAC-1	singlet	1	-410.4685727	-150.163323	-340.3544037	EMD2
TGGGCTGTCCCTAGGG-1	singlet	1	-3349.798594	-876.3192653	-2393.187366	EMD2
TGGGCTGTCCGAGCTG-1	singlet	1	-482.4219002	-182.5888082	-403.723001	EMD2
TGGGCTGTCTAATTCC-1	singlet	1	-1739.651094	-643.3738579	-1511.185121	EMD2
TGGGCTGTCTCCGAAA-1	singlet	0	-130.6013408	-446.3100303	-425.8029429	EMD2
TGGGTTAAGCGAGTCA-1	singlet	0	-271.208742	-795.7495638	-760.664144	EMD2
TGGGTTAAGGATGTTA-1	singlet	2	-380.4754246	-375.9987004	-130.4528632	EMD2
TGGGTTACAACCGCTG-1	singlet	0	-78.20217494	-215.6959661	-184.6020392	EMD2
TGGGTTACAACGTATC-1	doublet	45293	-1993.342003	-1021.882397	-1339.795041	EMD2
TGGGTTACATCGCTAA-1	singlet	1	-239.6172975	-115.4266163	-219.6117499	EMD2
TGGGTTAGTAGCGCTC-1	singlet	0	-6.917424837	-11.02769463	-17.90681684	EMD2
TGGGTTAGTCCCGTGA-1	singlet	1	-2233.909177	-728.9348085	-1841.951788	EMD2
TGGGTTAGTGCATTAC-1	singlet	2	-2712.613834	-2500.53421	-788.9005661	EMD2
TGGGTTAGTGGGATTG-1	singlet	1	-2875.822591	-811.7673425	-2327.833231	EMD2
TGGGTTATCAAGGTGG-1	singlet	1	-754.2494097	-283.752746	-552.0856353	EMD2
TGGGTTATCATTACCT-1	singlet	1	-1263.347123	-410.9986818	-1051.406122	EMD2
TGGTACAAGAGTCACG-1	singlet	1	-1590.367374	-681.2869761	-1299.133477	EMD2
TGGTACACAAATCAAG-1	singlet	2	-1070.487945	-984.2545772	-367.7231809	EMD2
TGGTACACAGAGGGTT-1	singlet	1	-826.8318612	-245.786836	-575.4951356	EMD2
TGGTACACAGGAGGAG-1	singlet	2	-3868.086733	-2790.088089	-1449.096998	EMD2
TGGTACAGTACCTGTA-1	singlet	2	-478.5410474	-365.2554079	-165.901283	EMD2
TGGTACAGTCGTCAGC-1	singlet	0	-80.49432364	-195.6680346	-170.7411896	EMD2
TGGTACAGTGTTCGTA-1	singlet	1	-2043.034404	-550.4761927	-1615.098525	EMD2
TGGTACATCGATTGAC-1	singlet	2	-1476.089146	-1637.882327	-383.5735871	EMD2
TGGTACATCTATCACT-1	singlet	1	-3364.120466	-1067.838067	-2813.908759	EMD2
TGGTAGTAGGAACGAA-1	singlet	1	-948.8918457	-319.5755156	-807.5540795	EMD2
TGGTAGTAGGTACTGG-1	singlet	1	-480.1943936	-155.0253721	-374.2007087	EMD2
TGGTAGTAGTCCTGCG-1	singlet	1	-1195.971754	-438.4500444	-942.6347231	EMD2
TGGTAGTCAGTAACGG-1	singlet	2	-1956.488366	-1716.057021	-610.8042949	EMD2
TGGTAGTTCACGACTA-1	singlet	0	-816.9296501	-3261.079878	-2854.217999	EMD2
TGGTAGTTCCACTAGA-1	singlet	2	-146.8313949	-141.7082211	-53.07736431	EMD2
TGGTAGTTCCCATAGA-1	singlet	1	-182.4812599	-63.45951034	-134.3660666	EMD2
TGGTAGTTCGCTTAAG-1	singlet	1	-587.4272308	-232.9154085	-515.4991499	EMD2
TGGTAGTTCGTGACTA-1	singlet	1	-1380.221095	-440.9896418	-1159.953686	EMD2
TGGTGATAGACACACG-1	singlet	0	-1366.48059	-4163.175691	-3918.908656	EMD2
TGGTGATAGACGCCCT-1	singlet	2	-547.5033417	-432.9974737	-195.2694356	EMD2
TGGTGATAGTAAATGC-1	singlet	1	-849.8537675	-280.2377028	-618.2926387	EMD2
TGGTGATAGTGCTACT-1	singlet	1	-4633.722358	-1313.35712	-3668.370211	EMD2
TGGTGATCAATGTTGC-1	singlet	2	-54.42431082	-65.5026034	-17.90766377	EMD2
TGGTGATCACCGCTGA-1	singlet	2	-671.3259117	-570.8478782	-211.5169296	EMD2
TGGTGATCATCACCAA-1	singlet	1	-453.437096	-197.8004647	-422.3854538	EMD2
TGGTGATCATCCGATA-1	singlet	1	-459.7313669	-166.7037924	-354.5443843	EMD2
TGGTGATGTACTGAGG-1	singlet	1	-2216.808514	-735.9005725	-1856.633467	EMD2
TGGTGATGTCACTCAA-1	singlet	2	-1061.23461	-1020.113426	-362.3568757	EMD2
TGGTGATGTCGTGGAA-1	singlet	1	-1052.264765	-342.271782	-797.9468485	EMD2
TGGTGATTCATGAGGG-1	singlet	1	-696.4215857	-295.0967187	-663.7434066	EMD2
TGGTGATTCCGTAGGC-1	singlet	1	-1927.135224	-545.5285416	-1525.975391	EMD2
TGGTGATTCGGTCAGC-1	singlet	1	-7694.546219	-1899.411952	-5798.755122	EMD2
TGGTGATTCGTTTACT-1	singlet	0	-89.44261588	-311.8642231	-282.1998944	EMD2
TGGTTAGAGCATCCCG-1	singlet	1	-641.1800315	-204.1884217	-380.4599025	EMD2
TGGTTAGAGGATAATC-1	singlet	1	-583.4994186	-266.8389143	-474.921815	EMD2
TGGTTAGAGTTGTAGA-1	singlet	0	-67.57737517	-208.2637259	-215.6276601	EMD2
TGGTTAGCAACAGCTT-1	singlet	2	-2583.51607	-2360.172263	-737.6938649	EMD2
TGGTTAGCAGACCCGT-1	singlet	0	-632.5119635	-2540.938195	-2786.001969	EMD2
TGGTTAGCAGAGATTA-1	singlet	1	-4122.908839	-1366.689197	-3075.063102	EMD2
TGGTTAGCAGTAACGG-1	singlet	2	-3715.614476	-3079.061727	-1317.150471	EMD2
TGGTTAGGTTAATCGC-1	singlet	1	-1982.102838	-645.1857743	-1756.658855	EMD2
TGGTTAGGTTCTTAGG-1	singlet	0	-608.6886324	-2636.59249	-2157.106173	EMD2
TGGTTAGTCGGCGATC-1	singlet	0	-990.1628172	-2890.638473	-2624.386192	EMD2
TGGTTAGTCTAGTCAG-1	singlet	2	-2152.924237	-1710.195428	-684.5643575	EMD2
TGTAACGAGAAGAGCA-1	singlet	2	-524.1313128	-604.0242767	-195.6622258	EMD2
TGTAACGAGCCGGATA-1	singlet	2	-3000.600254	-2479.348765	-933.2835068	EMD2
TGTAACGAGCCTCTGG-1	singlet	0	-198.6756429	-580.9872569	-492.0972299	EMD2
TGTAACGCATGAGATA-1	singlet	1	-1896.167035	-667.8560862	-1546.241464	EMD2
TGTAACGCATGTCGTA-1	singlet	0	-131.9567499	-450.0800342	-380.0819379	EMD2
TGTAACGGTGAGATCG-1	singlet	0	-21.99885784	-42.15416007	-37.15094351	EMD2
TGTAACGGTGGCTTAT-1	singlet	2	-842.7348592	-764.8841847	-283.1076945	EMD2
TGTAACGGTTCGAACT-1	singlet	1	-860.5007676	-269.3085145	-710.815715	EMD2
TGTAACGTCGATAACC-1	singlet	2	-2871.417847	-2592.428771	-1125.798704	EMD2
TGTAACGTCTGAATCG-1	doublet	0/1	-3629.154416	-3990.857788	-5770.938177	EMD2
TGTAACGTCTTGAGCA-1	singlet	1	-3985.794431	-1330.756434	-3292.885481	EMD2
TGTAAGCAGCCTATCA-1	singlet	1	-2890.163791	-1093.196728	-2426.319779	EMD2
TGTAAGCAGTAAACTG-1	singlet	1	-1970.019088	-584.5756939	-1401.373537	EMD2
TGTAAGCCAATGTCAC-1	singlet	1	-573.362714	-180.8194377	-428.0150869	EMD2
TGTAAGCGTTCGTGCG-1	singlet	1	-277.7383607	-76.76349977	-199.973513	EMD2
TGTAAGCTCAGATTGC-1	singlet	2	-3726.79088	-3395.776358	-1416.683377	EMD2
TGTAAGCTCATTCGGA-1	singlet	1	-892.477518	-273.5973407	-643.7988519	EMD2
TGTAAGCTCCCATTCG-1	singlet	0	-646.0359917	-1770.694049	-1737.138747	EMD2
TGTAAGCTCCTACCGT-1	singlet	2	-353.4512988	-364.2849474	-140.1721361	EMD2
TGTAAGCTCGTGGCGT-1	singlet	2	-2062.08418	-1614.312798	-699.0946171	EMD2
TGTACAGAGCATCCTA-1	singlet	1	-2340.877016	-883.0832048	-2013.373627	EMD2
TGTACAGAGTGCTCAT-1	singlet	0	-175.5106829	-509.2607377	-410.7155424	EMD2
TGTACAGCACCTATCC-1	singlet	1	-1638.449165	-459.2991612	-1391.959146	EMD2
TGTACAGGTAGTAAGT-1	unassigned	1/0	-17.71526526	-15.7983869	-8.556364791	EMD2
TGTACAGGTCTAGGTT-1	singlet	1	-636.5659224	-243.4149266	-495.0542043	EMD2
TGTACAGGTTCCAAAC-1	singlet	1	-4884.557249	-1311.4343	-3894.946793	EMD2
TGTACAGTCAGCTTCC-1	singlet	2	-2593.293534	-1982.408791	-920.5994011	EMD2
TGTACAGTCGCATTGA-1	singlet	1	-631.2479039	-197.6646559	-561.846259	EMD2
TGTACAGTCGCTAAAC-1	singlet	0	-428.997885	-1350.349335	-1305.63933	EMD2
TGTACAGTCTGTTCAT-1	singlet	1	-2117.984145	-812.4879007	-2011.981745	EMD2
TGTAGACAGAAATTCG-1	singlet	0	-295.6394935	-917.1433994	-785.4227194	EMD2
TGTAGACAGAACGCGT-1	singlet	0	-272.9695862	-860.6863287	-727.2370719	EMD2
TGTAGACAGGGTCAAC-1	singlet	1	-1788.479268	-535.4521403	-1310.584094	EMD2
TGTAGACAGGTAAGGA-1	singlet	1	-3007.929807	-853.1032864	-2377.551368	EMD2
TGTAGACCAACTTCTT-1	singlet	2	-1580.38371	-1412.553166	-578.5345314	EMD2
TGTAGACCACTGGCGT-1	singlet	1	-6.918058247	-2.120432123	-11.24402869	EMD2
TGTAGACCATAGTCAC-1	singlet	2	-453.5622026	-399.1472209	-190.659224	EMD2
TGTAGACGTATGTCCA-1	singlet	1	-1823.358991	-577.6131135	-1407.666237	EMD2
TGTAGACGTCATCTAG-1	singlet	1	-229.0136984	-83.90667521	-175.1527417	EMD2
TGTAGACGTCTTCTAT-1	singlet	1	-1289.535766	-406.8907501	-1079.920349	EMD2
TGTAGACGTGGAGAAA-1	singlet	0	-249.3081809	-713.8276056	-616.4841874	EMD2
TGTAGACGTTTGCAGT-1	singlet	1	-3391.505877	-1034.517858	-3051.109163	EMD2
TGTCAGAAGAAACTGT-1	singlet	0	-938.3107298	-1911.667907	-1990.391325	EMD2
TGTCAGAAGAACGTGC-1	singlet	2	-874.6768456	-820.6416354	-289.835773	EMD2
TGTCAGAAGCCAAGGT-1	singlet	1	-263.7518239	-119.2412244	-211.4499475	EMD2
TGTCAGAAGCCATGCC-1	singlet	1	-1375.003687	-387.8572416	-1163.943929	EMD2
TGTCAGAAGGTTACCT-1	singlet	1	-445.9599388	-138.6693561	-348.7855698	EMD2
TGTCAGAAGTGATTCC-1	singlet	1	-3688.017769	-1257.946808	-3097.888334	EMD2
TGTCAGAAGTTCATCG-1	singlet	0	-83.88627255	-364.3234844	-275.015017	EMD2
TGTCAGACACCATAAC-1	singlet	2	-1966.330368	-1697.384488	-763.6416681	EMD2

TGTCAGACACTGCGTG-1	singlet	2	-3572.31446	-2800.337107	-1104.798239	EMD2
TGTCAGACATCCAATG-1	singlet	1	-430.4339822	-151.3408388	-333.2912754	EMD2
TGTCAGAGTAACCAGG-1	singlet	1	-2212.00054	-717.2868841	-1686.192797	EMD2
TGTCAGAGTACGATTC-1	singlet	0	-294.5241084	-961.3603053	-836.0817253	EMD2
TGTCAGAGTGCCTACG-1	singlet	1	-208.5123153	-55.54880273	-164.68497	EMD2
TGTCAGATCAAGTAAG-1	singlet	0	-208.8924503	-656.8269239	-593.1581379	EMD2
TGTCAGATCCTCGATC-1	singlet	1	-1667.170067	-431.4472285	-1155.652716	EMD2
TGTCCACAGGTAGATT-1	unassigned	1/0	-5.229355917	-3.305945907	-8.352944312	EMD2
TGTCCACCACTGTCGG-1	singlet	1	-2453.019054	-712.0667554	-1910.553749	EMD2
TGTCCACCAGACCTGC-1	singlet	1	-304.6081117	-123.8650675	-223.6739833	EMD2
TGTCCACCATGAATAG-1	singlet	1	-1472.017615	-523.6373631	-1192.789443	EMD2
TGTCCACGTAGTTAGA-1	singlet	1	-3192.226752	-794.9124545	-2586.758136	EMD2
TGTCCACGTCAAGGCA-1	singlet	2	-203.8694948	-192.6170427	-89.90985719	EMD2
TGTCCACGTCACCTTC-1	singlet	1	-1666.17563	-636.6805815	-1399.706876	EMD2
TGTCCACGTGTAGCAG-1	singlet	1	-966.9582012	-152.4502905	-544.5361071	EMD2
TGTCCACTCACACGAT-1	singlet	2	-3511.507386	-2956.819009	-1139.012297	EMD2
TGTCCACTCATCCTGC-1	singlet	1	-424.5121737	-170.4105691	-372.6668757	EMD2
TGTCCACTCGAACTCA-1	singlet	1	-244.7573484	-107.6217136	-215.1384746	EMD2
TGTCCCAAGCATGTTC-1	singlet	0	-693.0826562	-2139.99159	-2092.752981	EMD2
TGTCCCACACTAGGTT-1	singlet	0	-356.2878709	-1398.368809	-1336.191613	EMD2
TGTCCCACAGTAACCT-1	singlet	1	-1131.459617	-390.3575558	-1033.137162	EMD2
TGTCCCAGTAGCTGAG-1	singlet	1	-91.59089612	-26.58148544	-58.20358256	EMD2
TGTCCCAGTGCAACGA-1	singlet	0	-193.2642907	-562.7787253	-556.534271	EMD2
TGTCCCAGTGCCTGAC-1	singlet	2	-3359.371212	-2831.331293	-1101.390051	EMD2
TGTCCCAGTGGAAATT-1	singlet	1	-1555.167608	-544.4892582	-1147.64541	EMD2
TGTCCCAGTTGGTAGG-1	singlet	1	-1490.46416	-489.2164848	-1241.557617	EMD2
TGTCCCATCACTGTTT-1	singlet	1	-438.1911058	-139.8619627	-351.7308762	EMD2
TGTCCCATCGGTCGAC-1	doublet	1/0	-975.2238922	-869.814054	-1244.806776	EMD2
TGTCCCATCGTTAGTG-1	singlet	2	-1769.247801	-1529.264463	-655.096427	EMD2
TGTCCTGAGAAGATCT-1	singlet	2	-930.8681537	-853.6566313	-316.4988864	EMD2
TGTCCTGAGCACTCAT-1	singlet	1	-1897.839618	-613.7961059	-1442.530733	EMD2
TGTCCTGAGCGATCGA-1	singlet	1	-1191.938437	-395.4305312	-974.302673	EMD2
TGTCCTGAGTATGAAC-1	singlet	0	-734.9825679	-2415.377816	-2416.739018	EMD2
TGTCCTGCAAGGATGC-1	singlet	0	-887.0229455	-2717.589441	-2615.74453	EMD2
TGTCCTGCAGCGCGTT-1	singlet	1	-2332.446621	-613.5082585	-1812.221602	EMD2
TGTCCTGCAGGCTATT-1	singlet	1	-2051.877878	-648.4911161	-1685.299459	EMD2
TGTCCTGTCGAGCTGC-1	singlet	1	-809.2580421	-287.1002938	-625.7317353	EMD2
TGTCCTGTCGGTAGAG-1	singlet	2	-1682.71733	-1548.635536	-566.619592	EMD2
TGTCCTGTCGTGTGGC-1	singlet	1	-1866.32183	-644.3788431	-1553.501984	EMD2
TGTGAGTAGAACTGAT-1	singlet	1	-398.152745	-141.7802332	-305.0975823	EMD2
TGTGAGTAGACTAGAT-1	singlet	2	-654.0677813	-600.158743	-301.5808155	EMD2
TGTGAGTAGAGGCGTT-1	singlet	2	-1301.005831	-1220.3297	-434.4584395	EMD2
TGTGAGTAGCCGTTAT-1	singlet	2	-2813.538466	-2330.920399	-1060.988974	EMD2
TGTGAGTAGCTCTATG-1	singlet	2	-497.1976075	-430.227717	-196.6164614	EMD2
TGTGAGTAGCTGCGAA-1	singlet	2	-812.3778132	-743.6148846	-256.3779317	EMD2
TGTGAGTAGTGATAAC-1	singlet	1	-1718.442132	-656.4534039	-1588.92461	EMD2
TGTGAGTCAACATCGT-1	singlet	2	-630.6257577	-431.4185261	-210.8020913	EMD2
TGTGAGTCACCTGATA-1	singlet	0	-501.0532782	-1799.406295	-1633.205696	EMD2
TGTGAGTCAGCACGAA-1	singlet	1	-2971.173659	-835.2544365	-2248.831582	EMD2
TGTGAGTGTCTCTCAC-1	doublet	0/2	-11.68008476	-18.59996263	-15.16614285	EMD2
TGTGAGTTCCGCTGTT-1	singlet	1	-276.4659762	-133.3472396	-295.4684789	EMD2
TGTGAGTTCTGAGGTT-1	singlet	1	-527.7190905	-160.6236552	-370.4196691	EMD2
TGTGATGAGACTTAAG-1	singlet	1	-1411.142822	-442.8595439	-1112.080148	EMD2
TGTGATGAGAGGTTAT-1	singlet	1	-283.9289049	-95.34533012	-243.9122599	EMD2
TGTGATGAGCAACAGC-1	singlet	0	-416.1772396	-1453.06776	-1347.244408	EMD2
TGTGATGAGGAGGCAG-1	singlet	1	-986.3387308	-299.6544521	-684.2054198	EMD2
TGTGATGAGGTAAGTT-1	singlet	1	-3142.442446	-951.9566807	-2426.482334	EMD2
TGTGATGAGGTTACCT-1	singlet	1	-4980.60195	-1350.274405	-3676.004829	EMD2
TGTGATGAGTTGGACG-1	singlet	2	-1811.906589	-2002.088722	-614.1634684	EMD2
TGTGATGCAAACTCTG-1	singlet	2	-798.5976884	-948.4249451	-235.265914	EMD2
TGTGATGCAAAGCGTG-1	singlet	1	-2523.471968	-696.0120295	-2014.568209	EMD2
TGTGATGCAGCGTACC-1	singlet	0	-1255.027083	-4415.428531	-4172.111416	EMD2
TGTGATGGTACTGGGA-1	singlet	2	-961.9867004	-823.6226629	-368.958008	EMD2
TGTGATGGTCCCTAAA-1	singlet	0	-634.3673793	-1958.337671	-1864.825418	EMD2
TGTGATGGTGACAACG-1	singlet	1	-325.8021184	-138.0972723	-274.5814342	EMD2
TGTGATGTCATTTCGT-1	singlet	1	-2536.076478	-715.0619783	-1815.727179	EMD2
TGTGATGTCCCTATTA-1	singlet	0	-101.4082065	-303.5493869	-232.9670303	EMD2
TGTGATGTCGGCTATA-1	singlet	1	-396.1287362	-140.7821342	-337.1704712	EMD2
TGTGATGTCTACTCAT-1	singlet	1	-454.3274201	-205.4276594	-424.9420565	EMD2
TGTGCGGAGACGCTCC-1	doublet	0/1	-9.436399139	-10.72720426	-14.88370167	EMD2
TGTGCGGAGATACATG-1	singlet	0	-41.08236011	-98.29408701	-101.8945491	EMD2
TGTGCGGAGCACGGAT-1	singlet	1	-446.4829797	-158.4713762	-402.5787927	EMD2
TGTGCGGAGGGACTGT-1	singlet	0	-69.7313013	-291.7235847	-266.0239176	EMD2
TGTGCGGAGGTAATCA-1	singlet	2	-2891.447951	-2604.186894	-924.0311979	EMD2
TGTGCGGAGTCAACAA-1	singlet	1	-241.3384839	-80.67780804	-160.3043596	EMD2
TGTGCGGCACCAACAT-1	singlet	1	-305.8965288	-121.3104454	-234.5719078	EMD2
TGTGCGGCATGTGCTA-1	doublet	0/2	-1948.729339	-3822.457034	-3103.192877	EMD2
TGTGCGGGTACAAGTA-1	singlet	2	-3732.444726	-2942.732083	-849.2266439	EMD2
TGTGCGGGTACCGTCG-1	singlet	1	-120.8693249	-62.54779651	-133.1650903	EMD2
TGTGCGGGTATGGTAA-1	singlet	1	-296.4890528	-132.5848998	-245.5537625	EMD2
TGTGCGGGTCCTGTCT-1	singlet	1	-1851.56625	-530.9658908	-1754.085428	EMD2
TGTGCGGGTCGGTAAG-1	singlet	1	-310.2932272	-137.7756164	-237.1467609	EMD2
TGTGCGGGTGTGTGTT-1	singlet	1	-287.9996189	-128.7594477	-279.3850547	EMD2
TGTGCGGGTTCGAGCC-1	singlet	0	-333.2313095	-1009.223936	-991.0696053	EMD2
TGTGCGGTCGCAGATT-1	singlet	1	-5286.268601	-1594.035935	-4136.64366	EMD2
TGTGCGGTCTAGGCAT-1	singlet	1	-669.3898851	-230.3634382	-523.8299968	EMD2
TGTGCGGTCTCGGCTT-1	singlet	1	-2139.736676	-688.0306968	-1645.944145	EMD2
TGTGCGGTCTTCGGAA-1	singlet	2	-510.2194738	-414.0273789	-198.3592667	EMD2
TGTGGCGAGTCTACCA-1	singlet	2	-233.3079228	-181.1421227	-73.37931243	EMD2
TGTGGCGCACTGCATA-1	singlet	1	-1024.848382	-351.1871375	-858.1854637	EMD2
TGTGGCGTCACGATCA-1	singlet	1	-1423.076059	-472.3981409	-1093.611411	EMD2
TGTGGCGTCATAGCAC-1	singlet	1	-2373.295145	-738.1579164	-1913.8884	EMD2
TGTGGCGTCCGAAGGA-1	singlet	1	-264.3229779	-120.3275613	-244.0385248	EMD2
TGTGGCGTCTGGGCAC-1	doublet	1/0	-423.8391253	-409.710209	-506.7340649	EMD2
TGTGTGAAGCATGCGA-1	singlet	0	-186.3668234	-640.6435907	-587.1409314	EMD2
TGTGTGAAGTGCACTT-1	singlet	1	-907.9498006	-317.1516931	-781.7229645	EMD2
TGTGTGAAGTGTGTTC-1	singlet	2	-992.9791632	-1021.737727	-339.0461154	EMD2
TGTGTGACAACCAACT-1	singlet	1	-2396.573033	-652.1375459	-1682.034962	EMD2
TGTGTGACAGTGTACT-1	singlet	1	-2465.441995	-912.0020332	-2062.265228	EMD2
TGTGTGATCAGGACAG-1	singlet	1	-255.7538359	-98.62971235	-218.2456318	EMD2
TGTGTGATCCACAGCG-1	singlet	2	-3691.6286	-3084.285758	-1162.786689	EMD2
TGTGTGATCCCGAGAC-1	singlet	1	-380.5677057	-144.4050215	-337.8731626	EMD2
TGTGTGATCCGCACTT-1	singlet	1	-2481.82593	-711.2295738	-2276.518874	EMD2
TGTGTGATCGGCATAT-1	singlet	0	-380.7564371	-867.5312483	-931.9343118	EMD2
TGTTACTAGACCTTTG-1	singlet	0	-91.59235034	-272.459227	-237.0619772	EMD2
TGTTACTAGAGAATCT-1	singlet	0	-557.0957602	-1646.31451	-1662.846462	EMD2
TGTTACTAGCCGGATA-1	singlet	1	-807.1850784	-238.0691657	-632.1619806	EMD2
TGTTACTCAACCGTGC-1	singlet	2	-881.5762472	-1075.395324	-254.9212341	EMD2
TGTTACTCACCCAAGC-1	singlet	0	-219.4908163	-712.6921082	-628.702726	EMD2
TGTTACTCATGGCCCA-1	singlet	2	-1003.246231	-822.0573055	-389.4944225	EMD2

TGTTACTGTACTCGTA-1	singlet	1	-398.7352204	-143.9658979	-373.4080573	EMD2
TGTTACTGTCGGATTT-1	singlet	0	-66.79309571	-247.033821	-247.7960123	EMD2
TGTTACTGTTTCGACA-1	singlet	1	-3361.366142	-1063.179039	-2748.462731	EMD2
TGTTACTTCGCTCTAC-1	singlet	1	-280.9717713	-120.0132026	-300.3456292	EMD2
TGTTACTTCTCATTAC-1	singlet	1	-493.6303382	-169.6230389	-394.2838771	EMD2
TGTTCATCACGAGGTA-1	singlet	1	-1902.532779	-605.1875062	-1344.03475	EMD2
TGTTCATCAGGCGAAT-1	singlet	2	-5557.113753	-4648.805572	-1465.740299	EMD2
TGTTCATCATGGCGCT-1	singlet	2	-59.30581687	-65.93479169	-24.63620793	EMD2
TGTTCATGTGGAGAAA-1	singlet	0	-503.8414028	-1461.048296	-1468.376186	EMD2
TGTTCATGTTTCACTT-1	singlet	2	-1002.178695	-821.411811	-292.73486	EMD2
TGTTCATTCGGAATTC-1	singlet	1	-2372.152998	-738.7479746	-1842.156095	EMD2
TGTTCATTCTTCTGGC-1	singlet	1	-7865.175382	-2335.467641	-5982.272664	EMD2
TGTTCCGAGGCTAGCA-1	singlet	2	-2182.858509	-2226.056468	-750.3184887	EMD2
TGTTCCGCAGGTCCGT-1	singlet	0	-345.8418047	-1208.423021	-1064.972557	EMD2
TGTTCCGCATAAGCGG-1	singlet	1	-2093.941199	-681.9647111	-1768.108539	EMD2
TGTTCCGGTAATTAGG-1	singlet	1	-2190.346174	-639.3694854	-1701.271601	EMD2
TGTTCCGGTATGAGGC-1	singlet	2	-2804.636992	-2298.044753	-966.2185098	EMD2
TGTTCCGGTGTACGCC-1	singlet	1	-1372.031355	-448.4971801	-1054.770375	EMD2
TGTTCCGTCCCTTTGG-1	singlet	1	-2362.37945	-842.905168	-2143.926943	EMD2
TGTTCCGTCGCTCCTA-1	singlet	0	-310.8958319	-1236.653009	-1171.256809	EMD2
TGTTCCGTCTAAGGAA-1	singlet	2	-792.5267606	-723.4779711	-303.7326929	EMD2
TGTTCCGTCTGAGATC-1	singlet	1	-1597.320667	-529.0100733	-1349.349783	EMD2
TGTTCTAAGGATAATC-1	singlet	1	-10741.26354	-3030.439092	-8405.286278	EMD2
TGTTCTACAAGACGAC-1	singlet	1	-1703.099151	-634.1107607	-1390.34551	EMD2
TGTTCTACATCGATAC-1	singlet	1	-346.8860943	-149.9043758	-260.275364	EMD2
TGTTCTAGTTCAACGT-1	singlet	1	-271.4107735	-114.8103898	-224.6120987	EMD2
TGTTCTAGTTCGAGCC-1	singlet	0	-561.5416812	-1744.527835	-1710.199732	EMD2
TGTTCTATCCGTTGGG-1	singlet	0	-92.80361255	-300.6971853	-243.6197159	EMD2
TGTTCTATCGTAATGC-1	singlet	1	-971.3992388	-319.1035208	-683.583882	EMD2
TGTTCTATCGTGCTCT-1	singlet	1	-223.0515738	-82.66534619	-193.2641758	EMD2
TGTTCTATCTTACTGT-1	singlet	0	-678.3332024	-2737.611523	-2620.014561	EMD2
TGTTCTATCTTGGCTC-1	singlet	1	-11.06692745	-3.764727299	-12.03872025	EMD2
TGTTGAGAGACCATTC-1	singlet	1	-351.2685706	-144.375364	-331.5936315	EMD2
TGTTGAGAGCCGAATG-1	singlet	2	-1903.075606	-1490.991799	-586.6150712	EMD2
TGTTGAGAGTATGGAT-1	singlet	1	-248.2213955	-104.8417957	-180.4283031	EMD2
TGTTGAGAGTTGGCGA-1	singlet	1	-1207.979546	-358.0694685	-1120.944195	EMD2
TGTTGAGCAGACACCC-1	singlet	1	-2494.011333	-722.4178398	-1794.678677	EMD2
TGTTGAGCAGGTCTCG-1	singlet	2	-471.8852945	-476.542874	-213.6256355	EMD2
TGTTGAGGTACGCGTC-1	singlet	1	-408.369193	-163.1595949	-326.8147137	EMD2
TGTTGAGGTACGTACT-1	singlet	1	-2220.604769	-772.1090033	-1756.390675	EMD2
TGTTGAGGTTCCATTT-1	singlet	0	-139.0205338	-444.8250524	-406.8153041	EMD2
TGTTGAGTCGGTCAGC-1	singlet	2	-1969.37656	-1986.755072	-563.8400092	EMD2
TGTTGAGTCGTACACA-1	doublet	0/1	-3372.959069	-3883.246395	-4969.642322	EMD2
TGTTGAGTCTACTCAT-1	singlet	1	-285.5958209	-101.3977423	-269.0695234	EMD2
TGTTGAGTCTGGCCGA-1	singlet	1	-1306.291028	-428.3504412	-1232.154187	EMD2
TGTTGAGTCTTGAACG-1	singlet	1	-1291.870321	-401.3923552	-1043.282459	EMD2
TGTTGGAAGAATTGTG-1	singlet	0	-1142.192061	-3539.887729	-3245.664714	EMD2
TGTTGGAAGGTAAACT-1	singlet	1	-2119.981518	-647.6907922	-1556.018256	EMD2
TGTTGGACACCAGTAT-1	singlet	1	-1342.505322	-435.8432177	-1093.605268	EMD2
TGTTGGAGTACCGTGC-1	singlet	1	-1205.718865	-369.296588	-1014.037526	EMD2
TGTTGGAGTCTCGGAC-1	singlet	0	-146.6828345	-321.5845626	-311.5016433	EMD2
TGTTGGATCGAATGCT-1	singlet	1	-123.6555242	-35.0066035	-76.0359537	EMD2
TGTTGGATCGCCAGTG-1	singlet	1	-223.7770931	-111.97089	-214.4757325	EMD2
TGTTGGATCTGGCCGA-1	singlet	1	-247.9427831	-98.74080805	-195.7540207	EMD2
TGTTTGTAGACCATTC-1	singlet	0	-2204.035276	-7365.231405	-6759.458672	EMD2
TGTTTGTCAGAACATA-1	singlet	0	-836.2827054	-2412.376596	-2231.386829	EMD2
TGTTTGTCAGTTAGGG-1	singlet	1	-1575.731915	-585.7678255	-1239.76207	EMD2
TGTTTGTGTGAGGAAA-1	singlet	1	-228.4400058	-77.15925038	-167.7760186	EMD2
TGTTTGTGTGGCACTC-1	singlet	1	-781.5588607	-326.9485599	-672.7330168	EMD2
TGTTTGTTCAGACTGT-1	singlet	0	-176.4859209	-430.0715785	-396.3810917	EMD2
TGTTTGTTCCATATGG-1	singlet	1	-3034.862318	-821.0435296	-2440.140916	EMD2
TGTTTGTTCTACCTTA-1	singlet	1	-132.2484355	-67.0480398	-163.2111751	EMD2
TTAATCCCAATCAGCT-1	singlet	0	-1412.774798	-4564.980296	-4286.695176	EMD2
TTAATCCCACACGGAA-1	singlet	1	-3187.569581	-1005.762824	-2818.448435	EMD2
TTAATCCCAGCTTCCT-1	singlet	1	-3900.527239	-1203.289434	-3069.929732	EMD2
TTAATCCCATATGAAG-1	singlet	1	-1860.316148	-639.5731108	-1597.690325	EMD2
TTAATCCCATGCTGCG-1	singlet	1	-480.950407	-190.064766	-386.3676515	EMD2
TTAATCCCATGTGCTA-1	singlet	0	-21.22216409	-70.50417631	-79.19722068	EMD2
TTAATCCGTGAGATTA-1	singlet	1	-832.1263	-239.6187879	-485.5933533	EMD2
TTAATCCGTGGAATGC-1	singlet	2	-4039.182349	-3638.305019	-1349.605674	EMD2
TTAATCCGTTTACCAG-1	singlet	1	-234.2272743	-96.96195145	-148.5530755	EMD2
TTAATCCGTTTAGAGA-1	singlet	0	-309.6737098	-1159.641175	-1000.179587	EMD2
TTAATCCTCGGCTGTG-1	singlet	1	-491.3326953	-212.8984281	-417.0390283	EMD2
TTAATCCTCTTTGCTA-1	doublet	1/0	-120.6655591	-67.06298605	-129.9682877	EMD2
TTACAGGAGCATTTGC-1	singlet	1	-2938.827822	-889.677991	-2482.595828	EMD2
TTACAGGAGTTAGAAC-1	singlet	1	-74.9019448	-22.25667503	-87.44351047	EMD2
TTACAGGCACCTGCGA-1	singlet	2	-1700.243106	-1467.822795	-530.3085869	EMD2
TTACAGGCACTCCGGA-1	singlet	1	-2178.701553	-526.3731576	-1629.993829	EMD2
TTACAGGGTCACTACA-1	singlet	1	-1576.09437	-576.6410549	-1309.948084	EMD2
TTACAGGGTTAGGCCC-1	singlet	0	-708.4901347	-1953.00458	-1923.234672	EMD2
TTACAGGGTTGTGTAC-1	singlet	1	-705.8449665	-238.5849626	-620.5111184	EMD2
TTACAGGTCAGCTGAT-1	singlet	1	-1998.573636	-715.2662703	-1622.352878	EMD2
TTACAGGTCCAAGCCG-1	singlet	1	-114.0689536	-37.00250423	-89.55482358	EMD2
TTACAGGTCCCTCAAC-1	singlet	1	-901.5976365	-296.8417807	-662.0606602	EMD2
TTACAGGTCCGGACGT-1	singlet	0	-206.5978616	-682.0871876	-614.1300688	EMD2
TTACAGGTCTGAACGT-1	singlet	1	-565.4052606	-214.0049355	-528.548926	EMD2
TTACCATAGAAAGTCT-1	singlet	2	-3166.675266	-2708.372089	-987.6472537	EMD2
TTACCATAGAGCATTA-1	singlet	2	-32.25339686	-36.18905462	-16.55475315	EMD2
TTACCATAGATTACCC-1	singlet	1	-3935.83988	-1176.991631	-2903.906327	EMD2
TTACCATAGCTGCGAA-1	singlet	1	-1374.679475	-549.2228955	-1156.551664	EMD2
TTACCATAGGTTGTTC-1	singlet	1	-817.6364074	-224.9602355	-569.874478	EMD2
TTACCATCAGAGGCAT-1	singlet	1	-4433.164045	-1445.767684	-3353.899088	EMD2
TTACCATGTGGATTTC-1	singlet	1	-2663.498658	-927.9414706	-2311.790921	EMD2
TTACCATGTTGCCAAT-1	singlet	1	-383.2932939	-146.7392021	-328.104136	EMD2
TTACCATTCCAAGCAT-1	singlet	2	-958.7320446	-961.8097773	-387.2462231	EMD2
TTACCATTCCGCTAGG-1	singlet	1	-2081.70877	-668.3795047	-1688.502093	EMD2
TTACCGCAGGACGCTA-1	singlet	1	-627.5407905	-186.2742035	-473.5413248	EMD2
TTACCGCAGGTACATA-1	singlet	0	-138.2777113	-344.5908464	-344.1887233	EMD2
TTACCGCAGTATCCTG-1	singlet	1	-547.1279313	-233.1294559	-507.3633228	EMD2
TTACCGCCACTCACTC-1	singlet	1	-388.39393	-162.3761243	-362.2945086	EMD2
TTACCGCGTATACAGA-1	singlet	2	-2271.230129	-1930.780156	-731.4231993	EMD2
TTACCGCGTGAGGAAA-1	singlet	1	-227.2672538	-82.91959188	-205.1910902	EMD2
TTACCGCTCGGCATAT-1	singlet	1	-298.8103194	-119.0916371	-244.8727944	EMD2
TTACGCCAGAATCTAG-1	singlet	0	-394.606946	-1327.886159	-1115.575266	EMD2
TTACGCCAGAGGTATT-1	singlet	1	-542.7293164	-238.5158009	-491.9882423	EMD2
TTACGCCAGATGGGCT-1	singlet	1	-1986.947482	-673.0873964	-1567.599999	EMD2
TTACGCCAGGAGCAAA-1	singlet	0	-1327.163173	-4209.181533	-4156.247461	EMD2
TTACGCCCATTCGATG-1	singlet	1	-1473.411707	-498.0785722	-1152.651267	EMD2
TTACGCCGTAACAGTA-1	doublet	0/1	-1511.727828	-2090.349267	-2348.393088	EMD2
TTACGCCGTACGATGG-1	singlet	0	-149.8964419	-440.4965044	-424.0023493	EMD2

TTACGCCGTGGCTTGC-1	singlet	2	-5120.800943	-4051.42349	-2054.21427	EMD2
TTACGTTAGACGGTCA-1	singlet	1	-400.9667202	-168.4333453	-351.7281019	EMD2
TTACGTTAGAGCTTTC-1	singlet	1	-1621.748738	-543.8494462	-1133.306935	EMD2
TTACGTTAGTCGCTAT-1	singlet	1	-618.3569701	-261.7352786	-551.2822841	EMD2
TTACGTTCACAAACGG-1	singlet	0	-777.7814161	-2227.585974	-2142.485568	EMD2
TTACGTTCAGTTGGTT-1	singlet	1	-823.3608566	-265.3608783	-589.3883393	EMD2
TTACGTTCATACTGTG-1	singlet	0	-315.7616261	-951.8108501	-867.1651605	EMD2
TTACGTTCATCGGAAG-1	singlet	1	-3369.904194	-1037.996114	-2780.078024	EMD2
TTACGTTGTACTTGTG-1	singlet	0	-85.64030908	-246.5164574	-203.0401811	EMD2
TTACGTTGTAGTCTTG-1	singlet	1	-332.3156244	-139.840896	-312.2084592	EMD2
TTACGTTGTGTGCCTG-1	singlet	0	-500.7175598	-1417.004584	-1463.278213	EMD2
TTACGTTGTTGCACGC-1	singlet	0	-173.5732923	-529.0437515	-415.6168254	EMD2
TTACGTTTCAATCTCT-1	singlet	1	-3666.179387	-1028.111082	-3113.901524	EMD2
TTACGTTTCCGGTAGC-1	singlet	0	-786.3233713	-2189.381578	-2176.300419	EMD2
TTACGTTTCGCCTTGT-1	singlet	1	-3114.860744	-896.3404673	-2455.677402	EMD2
TTACTGTAGCATCAAA-1	singlet	2	-437.7525413	-354.0832175	-140.9151761	EMD2
TTACTGTAGTTTCTTC-1	singlet	2	-559.844992	-567.2004836	-216.7893011	EMD2
TTACTGTCACGCAGTC-1	singlet	0	-930.4373022	-4869.80832	-3825.014879	EMD2
TTACTGTCACTACTTT-1	singlet	1	-2526.522891	-886.0772895	-1922.599494	EMD2
TTACTGTCAGAATTCC-1	singlet	1	-269.4601331	-76.81245123	-238.0721723	EMD2
TTACTGTCAGAGGTAC-1	singlet	1	-3149.207171	-955.4109818	-2582.34524	EMD2
TTACTGTCAGTAACAA-1	singlet	1	-3169.650343	-1102.288848	-2683.676374	EMD2
TTACTGTGTGCCCACA-1	singlet	1	-2329.917021	-702.8265469	-1908.745527	EMD2
TTACTGTGTTAAACCC-1	singlet	2	-6436.322176	-5339.086559	-1960.640488	EMD2
TTACTGTGTTGTGTTG-1	singlet	0	-568.2904897	-1635.993374	-1660.318462	EMD2
TTACTGTTCCAGTACA-1	singlet	0	-1230.508885	-3643.31111	-3405.799093	EMD2
TTACTGTTCCGCGATG-1	singlet	1	-2459.34213	-822.6092845	-2086.216302	EMD2
TTACTGTTCGATCCCT-1	singlet	1	-275.9286482	-116.4918613	-200.6235176	EMD2
TTACTGTTCGCCTATC-1	singlet	2	-422.4424413	-282.5382766	-105.5506579	EMD2
TTAGGCAAGACTCAAA-1	singlet	1	-7950.381373	-2121.580073	-5997.432504	EMD2
TTAGGCACAATGGCAG-1	singlet	1	-4948.696835	-1449.526524	-3913.641103	EMD2
TTAGGCAGTAACCCTA-1	singlet	1	-2929.204929	-876.3317233	-2345.042843	EMD2
TTAGGCAGTGCTCTTC-1	singlet	1	-378.4809503	-172.2397067	-350.0600639	EMD2
TTAGGCATCAATCCGA-1	singlet	1	-382.8485789	-129.8095451	-321.31483	EMD2
TTAGGCATCACACGAT-1	singlet	1	-444.6836867	-167.518059	-372.6103928	EMD2
TTAGGCATCGTCAGAT-1	singlet	0	-297.8598551	-1032.534179	-949.8806301	EMD2
TTAGGCATCGTTTACT-1	singlet	1	-390.9882838	-160.9456479	-333.7538226	EMD2
TTAGGGTAGACCAGCA-1	singlet	1	-1680.087674	-501.0244072	-1491.313932	EMD2
TTAGGGTAGGGATCTG-1	singlet	1	-226.3970726	-43.24904295	-199.2259164	EMD2
TTAGGGTGTGCCTACG-1	singlet	1	-1785.818952	-494.7183685	-1462.187681	EMD2
TTAGGGTGTTCATCGA-1	singlet	1	-467.877302	-210.521715	-407.0617158	EMD2
TTAGGGTGTTTGTTCT-1	singlet	1	-388.3880854	-145.6714451	-329.7733482	EMD2
TTAGGGTTCGCTTACC-1	singlet	2	-470.6911078	-466.863548	-184.2223673	EMD2
TTAGTCTAGACATACA-1	singlet	0	-22.55652685	-71.43338487	-65.44271297	EMD2
TTAGTCTAGACGGTCA-1	singlet	1	-900.5651745	-325.6272744	-667.4418472	EMD2
TTAGTCTAGGAGAGTA-1	singlet	1	-230.7000644	-82.36974453	-179.0603032	EMD2
TTAGTCTCACTCACTC-1	singlet	1	-2181.530587	-815.2880151	-1887.309724	EMD2
TTAGTCTCAGTAGTGG-1	singlet	1	-446.9429933	-173.5456645	-415.9339114	EMD2
TTAGTCTGTAATACCC-1	singlet	1	-310.9355052	-122.0073436	-282.0596313	EMD2
TTAGTCTGTTGCTGAT-1	singlet	1	-440.2121547	-181.9074413	-371.5549256	EMD2
TTAGTCTTCAGAGTGG-1	singlet	1	-284.1215517	-98.77501948	-234.2468036	EMD2
TTAGTCTTCTAACACG-1	singlet	1	-617.1259463	-255.9637435	-562.7691697	EMD2
TTATTGCAGAGAGCAA-1	singlet	2	-914.4336789	-827.7270651	-281.575528	EMD2
TTATTGCAGCAATAAC-1	singlet	1	-2767.977521	-1017.465338	-2348.545788	EMD2
TTATTGCAGCTGAAAT-1	singlet	2	-3890.865566	-2870.985096	-1423.032301	EMD2
TTATTGCAGTCGCCCA-1	singlet	1	-2038.744706	-688.3351137	-1653.455961	EMD2
TTATTGCAGTCTTGGT-1	singlet	0	-115.029352	-372.2588395	-377.2428166	EMD2
TTATTGCCAACGGCCT-1	singlet	2	-5816.256116	-4340.174561	-2145.462283	EMD2
TTATTGCCAAGTTTGC-1	singlet	2	-408.9209112	-374.0211735	-175.270915	EMD2
TTATTGCCAGGTACGA-1	singlet	2	-7980.670739	-6169.387308	-2287.704703	EMD2
TTATTGCCATTGCAAC-1	singlet	1	-1699.515293	-582.1978884	-1481.198364	EMD2
TTATTGCGTACATTGC-1	singlet	1	-622.7061533	-236.7148552	-494.3706689	EMD2
TTATTGCGTATGATCC-1	singlet	2	-3234.410839	-3042.211204	-1052.63914	EMD2
TTATTGCGTCTTCTAT-1	singlet	1	-700.1626555	-225.7839086	-523.5369583	EMD2
TTATTGCGTGTCTTGA-1	singlet	2	-1716.05689	-1439.864084	-524.4578481	EMD2
TTATTGCTCGCGGTAC-1	singlet	2	-2083.02058	-1754.470926	-706.8862733	EMD2
TTATTGCTCTATTTCG-1	singlet	2	-2503.558929	-2408.418285	-721.0286061	EMD2
TTCAATCAGAGTCTTC-1	doublet	0/2	-2326.381646	-4165.025967	-3021.583401	EMD2
TTCAATCAGCGAGTCA-1	singlet	2	-400.4052946	-380.3125735	-167.9915561	EMD2
TTCAATCCACAACGAG-1	singlet	1	-2936.217751	-918.3828575	-2350.485751	EMD2
TTCAATCCAGGCTTGC-1	singlet	1	-748.7467943	-290.1561734	-587.6066762	EMD2
TTCAATCCATGCGGTC-1	singlet	1	-2799.329545	-605.1914019	-1873.86993	EMD2
TTCAATCCATTGTCGA-1	singlet	1	-2719.533516	-634.8528875	-1783.896284	EMD2
TTCAATCGTCCTACAA-1	singlet	1	-301.5402915	-138.3966118	-340.2447895	EMD2
TTCAATCGTCTTGCGG-1	singlet	1	-250.6959164	-102.6170562	-200.6885129	EMD2
TTCAATCGTGATGGCA-1	singlet	0	-102.4691367	-357.0047066	-327.7207212	EMD2
TTCAATCTCTCAACCC-1	singlet	1	-285.8752427	-107.4967934	-241.0495532	EMD2
TTCACCGAGCAGCAGT-1	singlet	2	-791.4631793	-847.3036993	-265.1320226	EMD2
TTCACCGAGTCCGTCG-1	singlet	0	-665.2491491	-2018.566395	-2003.485901	EMD2
TTCACCGAGTTGCGAG-1	singlet	1	-2566.526157	-875.416313	-2089.616138	EMD2
TTCACCGGTAACGCGA-1	doublet	45293	-10098.89816	-6271.585644	-7336.931994	EMD2
TTCACCGGTACGTACT-1	singlet	1	-2154.924076	-667.858048	-1636.307964	EMD2
TTCACCGGTACTCGAT-1	singlet	1	-4384.538568	-1360.58411	-3706.31827	EMD2
TTCACCGGTGTAAATG-1	singlet	0	-1300.262617	-3962.131992	-3659.53249	EMD2
TTCACCGGTTCAAGTC-1	singlet	0	-919.3978191	-2439.499277	-2322.1476	EMD2
TTCACCGGTTGTTGTG-1	singlet	1	-3214.695639	-833.3894981	-2253.502895	EMD2
TTCACCGTCAGACCTA-1	singlet	0	-1344.546971	-3640.911861	-3555.757916	EMD2
TTCACGCAGACATCAA-1	singlet	1	-1374.479899	-449.6649535	-1214.250883	EMD2
TTCACGCAGAGTATAC-1	singlet	1	-3622.562549	-1342.066915	-2987.953683	EMD2
TTCACGCAGGTTAAAC-1	singlet	0	-2288.126846	-7662.932891	-7167.09545	EMD2
TTCACGCCACGCTATA-1	singlet	1	-2116.234502	-583.7331979	-1713.343454	EMD2
TTCACGCGTCATTGCA-1	singlet	1	-1567.858703	-519.1821119	-1326.207566	EMD2
TTCACGCGTCTTCCGT-1	singlet	1	-1338.16504	-500.7876031	-1117.150898	EMD2
TTCACGCTCAAGCCAT-1	singlet	1	-1714.862514	-520.7482939	-1523.096219	EMD2
TTCACGCTCCACTTCG-1	singlet	2	-81.13908721	-47.09423754	-24.50319555	EMD2
TTCACGCTCCGCGGAT-1	singlet	2	-1409.047349	-1573.984539	-520.9845918	EMD2
TTCACGCTCCTTCAGC-1	singlet	1	-401.2484435	-163.7845729	-387.2189779	EMD2
TTCAGGAAGAAGCGGG-1	singlet	1	-1472.534393	-518.3175699	-1262.059718	EMD2
TTCAGGAAGTCGTTAC-1	singlet	1	-777.4610228	-244.1856009	-634.3931316	EMD2
TTCAGGACAATAGTCC-1	singlet	2	-5074.244642	-4197.541498	-2697.35432	EMD2
TTCAGGACATAGCACT-1	singlet	1	-2333.336354	-675.1233349	-1952.462933	EMD2
TTCAGGAGTACGGATG-1	singlet	2	-4126.383207	-3205.527951	-1088.123029	EMD2
TTCAGGAGTCTCAGAT-1	singlet	1	-1391.539106	-468.0000808	-1215.152816	EMD2
TTCAGGAGTGACTGTT-1	singlet	2	-816.9034277	-765.7307655	-335.6636644	EMD2
TTCAGGAGTGTTCATG-1	singlet	1	-2101.55389	-608.1821306	-1766.146949	EMD2
TTCAGGAGTTCATCTT-1	singlet	1	-164.9130186	-81.62475387	-130.0305181	EMD2
TTCAGGAGTTTATGCG-1	singlet	1	-2029.791254	-546.9628771	-1647.639067	EMD2
TTCAGGATCGCATTGA-1	singlet	1	-548.9979291	-195.0267528	-423.4364684	EMD2
TTCATGTAGAGTCAGC-1	doublet	0/2	-1492.674153	-2388.640871	-1676.701348	EMD2
TTCATGTAGCTACGTT-1	singlet	2	-75.87822612	-66.74745408	-36.65973584	EMD2

TTCATGTAGGGCTTCC-1	singlet	1	-240.8485271	-95.66413405	-239.9139645	EMD2
TTCATGTAGTAAACGT-1	singlet	0	-402.4571015	-1433.68222	-1303.715181	EMD2
TTCATGTCAAATCCCA-1	singlet	2	-1024.747368	-1170.578174	-379.0882873	EMD2
TTCATGTCACGACAGA-1	singlet	2	-218.145988	-200.2037896	-94.33403411	EMD2
TTCATGTCAGGTTCCG-1	singlet	0	-144.0018206	-393.0417921	-374.740034	EMD2
TTCATGTGTTCGGTCG-1	singlet	0	-181.1852964	-435.9396129	-483.3820468	EMD2
TTCATGTTCCCTCTCC-1	singlet	2	-306.1687713	-208.6668674	-90.6185002	EMD2
TTCATGTTCGGTAACT-1	singlet	1	-257.9046325	-116.8555462	-256.2161181	EMD2
TTCATGTTCGTGTCAA-1	singlet	1	-1713.68595	-598.5387446	-1456.203393	EMD2
TTCATGTTCTACTCAT-1	singlet	0	-1007.561954	-2929.570537	-2875.315375	EMD2
TTCATTGAGCCGTTGC-1	singlet	2	-1249.675179	-1112.388681	-460.5745484	EMD2
TTCATTGAGCGCAATG-1	singlet	1	-2749.51188	-863.7428191	-2206.993672	EMD2
TTCATTGAGCGGATCA-1	singlet	2	-738.2029285	-651.2641315	-266.9605702	EMD2
TTCATTGAGCGTGTCC-1	singlet	1	-367.8867751	-103.2740347	-282.7913622	EMD2
TTCATTGAGCTAGTTC-1	singlet	2	-266.0236705	-209.7571111	-85.3302055	EMD2
TTCATTGAGGCCATAG-1	singlet	1	-493.4156292	-205.3573451	-440.0495544	EMD2
TTCATTGAGTGATAAC-1	singlet	1	-1224.595508	-423.6978102	-967.1388929	EMD2
TTCATTGCAAGTATCC-1	singlet	0	-32.8806121	-114.8590097	-83.88616334	EMD2
TTCATTGGTACCCAGC-1	singlet	2	-1226.408359	-1083.889474	-431.8699276	EMD2
TTCATTGGTACGACTT-1	unassigned	1/0	-13.38074217	-8.271613791	-18.64337439	EMD2
TTCATTGGTAGCGCTC-1	singlet	1	-304.7823566	-132.9926082	-264.8710118	EMD2
TTCATTGGTGAGAGGG-1	singlet	1	-632.9625811	-205.7683372	-448.7132922	EMD2
TTCATTGTCTTAGGAC-1	singlet	1	-3224.411054	-987.9723395	-2772.374475	EMD2
TTCCAATAGGAAGAAC-1	singlet	1	-700.9610079	-153.9062453	-462.1678597	EMD2
TTCCAATAGTTCCGGC-1	singlet	2	-5198.391088	-4225.020343	-1626.407407	EMD2
TTCCAATCACCGCTAG-1	singlet	2	-784.5017394	-704.5082182	-321.9769396	EMD2
TTCCAATCATATCTGG-1	singlet	2	-1039.260038	-805.6728115	-273.0242373	EMD2
TTCCAATGTACGAGCA-1	singlet	0	-1206.323396	-3964.66233	-3440.54598	EMD2
TTCCAATGTGCTATTG-1	singlet	0	-592.3804932	-1562.079567	-1449.280089	EMD2
TTCCAATGTGCTCGTG-1	singlet	1	-379.990947	-125.4824978	-312.3688323	EMD2
TTCCAATGTGTAAATG-1	singlet	2	-1913.437032	-1551.90306	-545.8632154	EMD2
TTCCAATGTTCGAACT-1	singlet	1	-2603.575325	-760.8803777	-1816.328646	EMD2
TTCCAATGTTGCTTGA-1	singlet	1	-2014.951712	-661.7735049	-1678.392772	EMD2
TTCCAATGTTGTATGC-1	singlet	2	-1195.77807	-970.1250631	-389.8837736	EMD2
TTCCAATTCATCAGTG-1	singlet	0	-1395.231165	-4323.260589	-4156.470106	EMD2
TTCCAATTCCTATTTG-1	singlet	1	-363.183595	-142.67309	-286.0283748	EMD2
TTCCAATTCGACGCGT-1	singlet	1	-3190.341413	-824.0855057	-2257.267834	EMD2
TTCCAATTCTAGCAAC-1	singlet	2	-3521.338986	-2747.710569	-1136.504744	EMD2
TTCCAATTCTCCAATT-1	singlet	1	-651.1762824	-269.5198691	-522.0957752	EMD2
TTCCACGAGGCGTCCT-1	singlet	1	-4953.812712	-1283.382936	-3867.345547	EMD2
TTCCACGAGTATGACA-1	singlet	1	-2251.747026	-757.1787943	-1816.827782	EMD2
TTCCACGAGTGCGCTC-1	singlet	1	-7387.200239	-2219.341963	-5519.39448	EMD2
TTCCACGCAAATCGGG-1	singlet	2	-1449.576004	-1387.264747	-571.4531215	EMD2
TTCCACGCACACCGAC-1	singlet	1	-634.0747713	-264.5100158	-536.9909144	EMD2
TTCCACGCACCCTATC-1	singlet	0	-1064.216569	-3732.312355	-3729.834481	EMD2
TTCCACGCAGTTCTAG-1	singlet	0	-1060.499981	-3538.905078	-3335.058855	EMD2
TTCCACGGTCACAGTT-1	singlet	1	-589.621663	-159.6847499	-451.9608233	EMD2
TTCCACGGTTCGGGTC-1	singlet	2	-401.2130498	-383.6635459	-172.1705287	EMD2
TTCCACGTCCCGAAAT-1	singlet	0	-185.3714739	-829.6628293	-686.4407593	EMD2
TTCCGGTCAAACTGCT-1	singlet	1	-194.6104128	-90.83879971	-218.9260171	EMD2
TTCCGGTCAAAGGATT-1	singlet	1	-1112.482876	-397.4784217	-941.4706302	EMD2
TTCCGGTCAGCGGTCT-1	singlet	0	-729.4541718	-2077.489754	-2196.015302	EMD2
TTCCGGTGTACCCAGC-1	singlet	1	-2811.775601	-686.8345745	-2003.381635	EMD2
TTCCGGTGTAGAGATT-1	unassigned	1/0	-38.75150391	-25.90123526	-31.1164487	EMD2
TTCCGGTGTAGTCGTT-1	singlet	1	-472.0055897	-164.4393901	-313.1431636	EMD2
TTCCGGTGTCCAGCGT-1	singlet	2	-467.3521855	-449.7558964	-177.0639538	EMD2
TTCCGGTGTCCAGTTA-1	singlet	1	-963.0709529	-340.0837788	-850.5013988	EMD2
TTCCGGTGTCGTCAGC-1	singlet	1	-835.0172007	-350.5387451	-844.2669284	EMD2
TTCCGGTTCCCTTCCC-1	singlet	1	-385.3885812	-144.1018979	-321.0383478	EMD2
TTCCGGTTCGTTCATT-1	singlet	0	-698.256364	-1819.791595	-1874.534174	EMD2
TTCCGGTTCTGGACCG-1	singlet	1	-3545.905208	-1019.590045	-3011.29719	EMD2
TTCCGTGAGCTTACGT-1	singlet	0	-361.5673449	-1162.659394	-991.6745472	EMD2
TTCCGTGCACGTAGTT-1	singlet	1	-2233.799403	-671.3847977	-1696.26952	EMD2
TTCCGTGCACTTCCTG-1	singlet	0	-25.38817901	-108.0171615	-90.67850169	EMD2
TTCCGTGCAGGCTCTG-1	singlet	1	-1179.285827	-418.8480707	-990.3397756	EMD2
TTCCGTGGTACCGTCG-1	singlet	1	-309.7171271	-122.1900564	-291.136421	EMD2
TTCCGTGGTTGCAACT-1	singlet	1	-1608.851345	-579.8427845	-1395.922194	EMD2
TTCCGTGGTTGCTCGG-1	singlet	0	-518.1226808	-1574.121007	-1399.206321	EMD2
TTCCGTGTCGGATACT-1	singlet	0	-1145.128716	-3680.458409	-3638.507929	EMD2
TTCCTAAAGCTTGTTG-1	singlet	0	-256.4758496	-704.2247271	-729.7365039	EMD2
TTCCTAAAGTATTCCG-1	singlet	1	-519.0988421	-235.2109041	-575.1429025	EMD2
TTCCTAACATCACCAA-1	singlet	2	-214.8044116	-155.7698996	-71.2120563	EMD2
TTCCTAACATTGAAAG-1	singlet	2	-1137.470038	-900.1433795	-409.0498132	EMD2
TTCCTAAGTATGTCAC-1	singlet	1	-2255.096735	-756.3886401	-1676.133087	EMD2
TTCCTAAGTCTCAGAT-1	singlet	0	-1220.932596	-4365.700094	-4143.205666	EMD2
TTCCTAAGTTTAAGGA-1	singlet	1	-590.7047638	-227.490805	-531.6167122	EMD2
TTCCTAATCATTATCC-1	singlet	1	-4158.144382	-1214.323163	-3096.774562	EMD2
TTCCTAATCGGTGCAC-1	singlet	0	-148.9795148	-451.323832	-352.9400912	EMD2
TTCCTAATCTATCCAT-1	singlet	0	-1081.366902	-3688.135948	-3493.682329	EMD2
TTCCTAATCTTCGCTG-1	singlet	2	-109.6733752	-101.0167857	-43.51822977	EMD2
TTCCTCTAGAAGGCTC-1	singlet	1	-2025.348922	-638.0064659	-1875.088516	EMD2
TTCCTCTAGATGCGAC-1	singlet	1	-5139.451291	-1405.785418	-3896.340447	EMD2
TTCCTCTAGCATACTC-1	singlet	1	-1860.261969	-670.8191725	-1703.177152	EMD2
TTCCTCTAGTTACGAA-1	singlet	1	-9527.977788	-2535.118079	-6680.360342	EMD2
TTCCTCTCAACGTTAC-1	singlet	1	-5509.318389	-1785.806879	-4406.06135	EMD2
TTCCTCTCATATGAAG-1	singlet	0	-84.48258967	-341.6958673	-277.5480011	EMD2
TTCCTCTCATCGCTGG-1	singlet	0	-95.9847355	-232.5430359	-244.4293671	EMD2
TTCCTCTGTCGAACAG-1	singlet	0	-929.0002406	-3164.533984	-3086.882292	EMD2
TTCCTCTGTCGAGCAA-1	singlet	2	-953.6460802	-928.4004101	-412.6181907	EMD2
TTCCTCTGTCGGCCTA-1	singlet	1	-2853.081776	-955.3049637	-2278.895908	EMD2
TTCCTCTGTGCATTTG-1	singlet	1	-2562.526632	-847.1001258	-1972.202414	EMD2
TTCCTCTGTTAAGGAT-1	singlet	0	-207.509869	-582.3199308	-659.8696302	EMD2
TTCCTCTGTTAGGACG-1	singlet	0	-982.3334955	-3158.921685	-2827.801327	EMD2
TTCCTCTTCATTACTC-1	singlet	0	-362.6101146	-1004.861777	-1009.365023	EMD2
TTCCTCTTCCCGTAAA-1	doublet	0/2	-5333.269292	-10370.30743	-8163.793484	EMD2
TTCCTCTTCGTAGCTA-1	singlet	1	-2980.699575	-810.740215	-2181.531526	EMD2
TTCCTCTTCTAAGCGT-1	singlet	1	-2115.919121	-673.759868	-1958.025876	EMD2
TTCCTTCAGGCATGCA-1	singlet	1	-1888.701317	-595.3631594	-1652.400069	EMD2
TTCCTTCAGGGTTAAT-1	singlet	1	-870.9433547	-306.7862769	-713.5109573	EMD2
TTCCTTCCAAACGTGG-1	singlet	2	-1269.715165	-1257.817612	-593.5928245	EMD2
TTCCTTCCACTGGATT-1	singlet	2	-1165.125456	-986.2816126	-453.5378914	EMD2
TTCCTTCCAGTTTGGT-1	singlet	0	-480.6711464	-1284.651862	-1184.282278	EMD2
TTCCTTCGTCCATAGT-1	doublet	1/0	-2616.814583	-2475.873691	-3354.919293	EMD2
TTCCTTCGTTAGTCGT-1	singlet	1	-332.974042	-109.4811996	-255.7396879	EMD2
TTCCTTCTCAAGTCTG-1	singlet	0	-645.0939295	-1955.772376	-1934.646508	EMD2
TTCCTTCTCACACCCT-1	singlet	1	-3266.529668	-1261.132966	-2654.516383	EMD2
TTCCTTCTCACCTGGG-1	singlet	1	-330.3088824	-144.7157157	-341.9275418	EMD2
TTCCTTCTCTACTGCC-1	singlet	1	-4925.971107	-1625.370068	-4091.173019	EMD2
TTCCTTCTCTGCCTCA-1	singlet	1	-1972.218777	-582.2451133	-1740.619702	EMD2
TTCGATTAGGTTCTAC-1	singlet	2	-2808.038866	-2284.704735	-1130.274164	EMD2

TTCGATTCAGACCTAT-1	singlet	2	-1779.391301	-1599.413561	-694.6820453	EMD2
TTCGATTCAGATCCAT-1	singlet	2	-3301.610105	-3242.67956	-1018.060689	EMD2
TTCGATTCAGCTCGGT-1	singlet	1	-2745.148753	-818.0874635	-2264.19643	EMD2
TTCGATTGTCATCACA-1	singlet	1	-113.8008238	-46.84656656	-108.7898759	EMD2
TTCGATTGTCCAAATC-1	singlet	2	-2440.642043	-2454.425908	-845.0212838	EMD2
TTCGATTGTCCACACG-1	singlet	1	-447.3981745	-172.6791381	-394.0373518	EMD2
TTCGATTTCGAGTGAG-1	singlet	2	-2630.474134	-2328.557484	-902.6443922	EMD2
TTCGATTTCTAGGCAT-1	singlet	2	-4716.503997	-3839.066462	-1053.929818	EMD2
TTCGCTGAGAGAATCT-1	singlet	1	-2615.374922	-588.4150428	-2685.456887	EMD2
TTCGCTGAGCCATCCG-1	singlet	1	-447.6162661	-170.1307818	-434.7571404	EMD2
TTCGCTGCACTGCACG-1	singlet	1	-815.6759017	-331.3787027	-709.074991	EMD2
TTCGCTGCAGATTCGT-1	singlet	0	-123.6977639	-285.1263301	-281.5555376	EMD2
TTCGCTGCAGCTTTGA-1	singlet	1	-413.8427216	-144.3668902	-374.9476237	EMD2
TTCGCTGCATCAACCA-1	singlet	0	-119.5205055	-236.8140464	-222.9532064	EMD2
TTCGCTGGTAAGTAGT-1	singlet	2	-700.6956103	-732.4765263	-281.6539116	EMD2
TTCGCTGGTGCAACGA-1	singlet	2	-2483.32887	-2186.69246	-931.6863686	EMD2
TTCGCTGGTGCCCACA-1	singlet	2	-3907.576711	-3408.03013	-1363.275769	EMD2
TTCGCTGTCATTACCT-1	singlet	1	-742.6357267	-285.6677386	-585.6986266	EMD2
TTCGCTGTCCGATAGT-1	singlet	1	-2392.67837	-553.3942865	-1670.887589	EMD2
TTCGCTGTCGCTGATA-1	singlet	1	-408.1843772	-173.0766532	-354.01171	EMD2
TTCGCTGTCGGAGTAG-1	singlet	1	-617.2915946	-208.7387711	-458.9589676	EMD2
TTCGCTGTCGGTGTTA-1	singlet	2	-1702.144218	-1563.516733	-517.3260313	EMD2
TTCGCTGTCTTAGCTT-1	singlet	1	-938.2807806	-332.5653164	-766.5486412	EMD2
TTCGGTCCAAGTATAG-1	singlet	2	-311.0612767	-302.9689322	-139.5420073	EMD2
TTCGGTCCAATAGGAT-1	singlet	0	-458.6888539	-1288.330754	-1191.625342	EMD2
TTCGGTCCACCCTCTA-1	singlet	1	-2624.658998	-829.7193452	-2244.870626	EMD2
TTCGGTCCAGGACGAT-1	singlet	0	-158.6911778	-645.8886895	-445.5768404	EMD2
TTCGGTCCATCGATGT-1	singlet	0	-383.5609104	-1127.512044	-1149.827867	EMD2
TTCGGTCGTCTTCATT-1	singlet	0	-508.9144183	-1427.390985	-1278.723527	EMD2
TTCGGTCTCCAAGCTA-1	singlet	1	-518.2453758	-213.0581567	-373.0575412	EMD2
TTCGGTCTCCATCTGC-1	singlet	0	-1791.372708	-5835.81717	-5633.146407	EMD2
TTCGGTCTCGTGGAAG-1	singlet	1	-337.4506417	-139.082935	-292.4147001	EMD2
TTCGGTCTCGTTCATT-1	singlet	0	-1710.160474	-5805.262129	-5151.795159	EMD2
TTCGGTCTCTAGTACG-1	singlet	1	-495.3458193	-171.8532292	-454.0098115	EMD2
TTCTAACAGATTCGCT-1	singlet	1	-922.1794666	-337.2295194	-745.0131678	EMD2
TTCTAACAGCAGATAT-1	singlet	2	-913.7122477	-1013.136629	-510.7416785	EMD2
TTCTAACCAGAGTCAG-1	singlet	2	-1997.693472	-1726.519333	-582.7058548	EMD2
TTCTAACCATGCGTGC-1	singlet	1	-882.7710846	-245.0271075	-790.1084058	EMD2
TTCTAACGTAAGGTCG-1	singlet	1	-712.3470661	-218.7401947	-476.4467269	EMD2
TTCTAACGTGCGTCGT-1	singlet	1	-782.3713439	-296.6170065	-648.7499948	EMD2
TTCTAACTCAAGCCCG-1	singlet	0	-1365.67468	-4052.205146	-3938.652898	EMD2
TTCTAACTCACCTCAC-1	singlet	2	-4569.048177	-3834.905338	-1684.79224	EMD2
TTCTAACTCCAAATGC-1	singlet	2	-326.6347425	-278.9951959	-106.3139664	EMD2
TTCTAACTCTGTCAGA-1	singlet	2	-2601.328411	-2045.692579	-865.450756	EMD2
TTCTAACTCTTGTTAC-1	singlet	1	-783.4898833	-267.8576998	-596.4669572	EMD2
TTCTAGTAGAAGCGAA-1	singlet	1	-308.1432629	-114.291901	-300.2880583	EMD2
TTCTAGTAGATAACGT-1	singlet	0	-172.8067668	-657.6318064	-665.033906	EMD2
TTCTAGTAGTCTTCCC-1	singlet	0	-1436.154342	-4438.04651	-4054.902805	EMD2
TTCTAGTCAAACGGCA-1	singlet	0	-1871.327183	-6871.435796	-6800.877376	EMD2
TTCTAGTCAGGCTTGC-1	singlet	2	-532.4939316	-465.1432671	-209.9239256	EMD2
TTCTAGTCAGTGGGTA-1	singlet	1	-3362.743831	-1036.409428	-2628.907567	EMD2
TTCTAGTCATCCGATA-1	singlet	2	-475.17254	-432.1558977	-196.2880396	EMD2
TTCTAGTCATCGAACT-1	singlet	1	-4683.134806	-1781.385326	-3879.687314	EMD2
TTCTAGTGTAATCAGA-1	singlet	2	-674.4056851	-644.8596776	-258.3507332	EMD2
TTCTAGTGTCATTGCA-1	singlet	1	-487.2855783	-142.6038023	-379.9534011	EMD2
TTCTAGTGTCCAACGC-1	singlet	2	-2567.545695	-2162.322828	-850.1531022	EMD2
TTCTAGTGTCCACTCT-1	doublet	1/0	-7239.386769	-4237.394903	-7300.768266	EMD2
TTCTAGTGTGGCTGAA-1	singlet	1	-1826.636774	-543.6986491	-1595.12428	EMD2
TTCTAGTTCAGCATTG-1	singlet	1	-2034.210801	-529.7903173	-1663.933823	EMD2
TTCTAGTTCCGTCCTA-1	singlet	2	-678.5302602	-694.4330468	-236.4816261	EMD2
TTCTAGTTCTCTGCTG-1	singlet	1	-618.9614578	-224.9024598	-447.7920655	EMD2
TTCTCTCAGAGGTTTA-1	singlet	1	-124.5182953	-61.34311771	-97.59578019	EMD2
TTCTCTCAGGCGTTAG-1	singlet	1	-1115.448108	-341.7223556	-910.743243	EMD2
TTCTCTCCACAGCGCT-1	singlet	2	-780.1575184	-663.8523562	-291.8337002	EMD2
TTCTCTCCATGATGCT-1	singlet	0	-1012.627707	-3201.643285	-2941.423465	EMD2
TTCTCTCGTACGGCAA-1	singlet	2	-3253.156286	-2736.621185	-1112.20435	EMD2
TTCTCTCGTCGCACGT-1	singlet	2	-4100.147716	-3133.428764	-1229.651962	EMD2
TTCTCTCGTCGCGGTT-1	singlet	1	-405.345287	-152.3705743	-317.1054633	EMD2
TTCTCTCGTCTTGAAC-1	singlet	1	-2149.304378	-788.7400655	-1643.566705	EMD2
TTCTCTCTCACATTGG-1	singlet	1	-329.3409333	-128.2756987	-323.7926714	EMD2
TTCTCTCTCAGACAAA-1	singlet	0	-87.61014168	-259.2429131	-310.0071309	EMD2
TTCTCTCTCATCACAG-1	singlet	0	-427.6918601	-1400.05734	-1130.228169	EMD2
TTCTCTCTCATTTCGT-1	singlet	0	-144.2784999	-364.2998204	-356.4909341	EMD2
TTCTCTCTCTGCATGA-1	singlet	1	-360.3251429	-129.5488194	-277.3470522	EMD2
TTCTGTAAGCAGTCTT-1	singlet	1	-2015.459579	-604.9156789	-1809.501425	EMD2
TTCTGTAAGCCAAGCA-1	singlet	1	-387.2854969	-157.5650646	-308.4807498	EMD2
TTCTGTAAGGATTCAA-1	singlet	1	-2331.777523	-696.3675977	-1998.739938	EMD2
TTCTGTAAGGCGCTTC-1	singlet	1	-1687.95351	-572.3845769	-1598.875563	EMD2
TTCTGTACAAATGAGT-1	singlet	2	-65.14172967	-64.61781675	-19.60248438	EMD2
TTCTGTACAAATTAGG-1	singlet	1	-1786.292842	-556.0614891	-1343.011992	EMD2
TTCTGTACAACACTAC-1	singlet	1	-2618.560032	-960.8368944	-2141.612809	EMD2
TTCTGTAGTAGACACG-1	singlet	1	-850.1083796	-225.2593418	-656.8741455	EMD2
TTCTGTAGTAGGTACG-1	singlet	1	-255.9088488	-95.45925162	-205.3171227	EMD2
TTCTGTAGTGCTAGCC-1	singlet	2	-5069.839021	-3779.522449	-1354.657304	EMD2
TTCTGTAGTTGTATGC-1	singlet	1	-2577.850878	-848.7263543	-2163.899143	EMD2
TTCTGTATCCGTAGTA-1	doublet	45323	-72.61806052	-45.62164631	-31.68209824	EMD2
TTCTGTATCGTGCGAC-1	singlet	1	-501.293397	-217.091935	-460.7457568	EMD2
TTCTTCCCAGAAGTGC-1	singlet	1	-547.6949599	-211.5196206	-417.84072	EMD2
TTCTTCCCAGCAGTTT-1	singlet	0	-241.9821087	-565.967266	-507.7017701	EMD2
TTCTTCCCAGTTGAAA-1	singlet	1	-3867.11861	-1134.375424	-2826.040792	EMD2
TTCTTCCCAGTTGCGC-1	doublet	0/1	-412.2370074	-624.9563545	-672.0193951	EMD2
TTCTTCCGTAGGATAT-1	singlet	1	-822.8430491	-270.4589593	-659.3420623	EMD2
TTCTTCCGTCGAACGA-1	singlet	1	-3074.591919	-874.2353855	-2445.330654	EMD2
TTCTTCCGTCGTACTA-1	singlet	2	-910.7565955	-867.8124631	-348.8500754	EMD2
TTCTTCCGTTGCAACT-1	singlet	1	-1488.928302	-525.5370643	-1130.87661	EMD2
TTCTTCCTCACTTGTT-1	singlet	1	-1748.920594	-755.8195418	-1595.769341	EMD2
TTCTTCCTCGCCTAGG-1	singlet	1	-531.7428879	-188.6684949	-457.0121742	EMD2
TTCTTCCTCGGTGAAG-1	singlet	1	-1524.282107	-465.168957	-1264.832686	EMD2
TTCTTGACACAGTCCG-1	singlet	1	-2173.566159	-643.354995	-1962.034001	EMD2
TTCTTGATCGGCTTCT-1	singlet	0	-717.0265419	-2339.39277	-2063.571901	EMD2
TTCTTGATCTGCTCTG-1	singlet	1	-647.337585	-275.415201	-477.3154087	EMD2
TTGAACGAGAACTGAT-1	singlet	0	-775.0776604	-2192.222775	-2075.351304	EMD2
TTGAACGAGATGCGAC-1	singlet	1	-337.190115	-153.3618826	-311.150758	EMD2
TTGAACGAGTCGCGAA-1	singlet	2	-4433.460516	-3579.080994	-1289.55854	EMD2
TTGAACGAGTTAGTAG-1	singlet	1	-212.7406352	-95.47434677	-184.064502	EMD2
TTGAACGCAAGCACCC-1	singlet	2	-546.3302956	-526.898975	-247.0084143	EMD2
TTGAACGCATGACTAC-1	singlet	2	-2953.593248	-3014.032623	-920.4295889	EMD2
TTGAACGGTAGATTGA-1	singlet	1	-373.3923739	-140.6719556	-317.0824511	EMD2
TTGAACGGTCAAGCCC-1	singlet	1	-1827.430005	-525.6701577	-1438.940414	EMD2
TTGAACGGTTCTCTCG-1	singlet	2	-3004.625512	-2560.568386	-989.6149893	EMD2

TTGAACGTCCTTCACG-1	singlet	0	-527.1873901	-1473.085508	-1483.069792	EMD2
TTGAACGTCGACCTAA-1	singlet	0	-524.3338964	-1700.82963	-1710.736693	EMD2
TTGACCCAGCCGATTT-1	singlet	1	-3183.634841	-959.5718697	-2481.758887	EMD2
TTGACCCCAGACTGCC-1	singlet	1	-140.5024528	-60.40681296	-137.5060738	EMD2
TTGACCCGTGCTTCAA-1	singlet	2	-55.01599178	-33.6475939	-22.9942988	EMD2
TTGACCCTCAACGTGT-1	singlet	1	-328.9598658	-117.0592606	-304.8520951	EMD2
TTGACCCTCACTGTTT-1	singlet	1	-818.3633338	-291.9470067	-669.5294256	EMD2
TTGACCCTCGCGTTTC-1	singlet	2	-1224.512675	-1053.292224	-487.1124732	EMD2
TTGACCCTCTTCGCTG-1	singlet	1	-1611.224091	-515.9518683	-1454.873258	EMD2
TTGAGTGAGGTAGCAC-1	singlet	2	-812.0720866	-762.4929719	-329.0688123	EMD2
TTGAGTGCACAGCTGC-1	singlet	2	-655.4474175	-578.5178312	-254.4636044	EMD2
TTGAGTGCAGATTTCG-1	singlet	1	-467.2432307	-152.9396413	-399.7406534	EMD2
TTGAGTGCAGCAAGAC-1	singlet	2	-172.8917254	-166.596591	-90.12692254	EMD2
TTGAGTGCATCTCGTC-1	doublet	1/0	-6.978429753	-5.946826711	-8.103274656	EMD2
TTGAGTGGTATGGAAT-1	singlet	1	-1871.168821	-703.7571727	-1652.10662	EMD2
TTGAGTGGTGGTGATG-1	singlet	1	-340.4595533	-126.9109448	-231.8274485	EMD2
TTGAGTGGTTACACAC-1	singlet	1	-705.3312039	-237.4572945	-516.6047474	EMD2
TTGAGTGTCATACGGT-1	singlet	0	-1589.814568	-4160.407261	-4086.30218	EMD2
TTGAGTGTCATGTCTT-1	singlet	1	-891.5446949	-330.0398308	-847.051097	EMD2
TTGAGTGTCCAGTGCG-1	singlet	1	-1327.607623	-339.2527916	-1207.510509	EMD2
TTGAGTGTCCCTAGGG-1	doublet	45323	-1890.172841	-1353.57905	-943.0353004	EMD2
TTGAGTGTCGTTAGAC-1	singlet	0	-56.64392825	-176.4919994	-177.2592657	EMD2
TTGAGTGTCTCTAGGA-1	singlet	1	-247.8200705	-91.69005308	-216.114832	EMD2
TTGAGTGTCTTACGTT-1	singlet	1	-231.7221204	-108.2864818	-216.9565764	EMD2
TTGATGGAGGGACACT-1	singlet	0	-784.4880458	-2547.299258	-2320.681678	EMD2
TTGATGGAGGGTCTTT-1	singlet	1	-1579.9679	-449.2795278	-1490.301286	EMD2
TTGATGGAGTGGTGGT-1	singlet	0	-1131.921458	-3721.89024	-3604.847897	EMD2
TTGATGGCACAGTACT-1	doublet	1/0	-3000.9109	-2732.15202	-3502.427544	EMD2
TTGATGGCATTGACCA-1	singlet	1	-317.5070477	-132.7980316	-302.9827046	EMD2
TTGATGGGTAGGATAT-1	singlet	1	-51.47998046	-19.10653634	-41.63554778	EMD2
TTGATGGGTTCAACGT-1	singlet	2	-1909.390471	-1525.34334	-526.7878802	EMD2
TTGATGGTCTCTGCTG-1	singlet	2	-212.084194	-157.3059886	-68.95368995	EMD2
TTGCATTAGTTTAGGA-1	singlet	1	-4686.822001	-1222.945313	-3576.783511	EMD2
TTGCATTCAAACGAGC-1	singlet	1	-1498.977016	-466.3689883	-1336.944749	EMD2
TTGCATTCAACACTAC-1	singlet	1	-539.0166696	-238.125513	-466.0326048	EMD2
TTGCATTCACAATGAA-1	singlet	0	-4.091072186	-16.73865825	-17.96518463	EMD2
TTGCATTGTCAACACT-1	singlet	1	-769.7119799	-284.5866313	-589.7913691	EMD2
TTGCATTGTCTTGTCC-1	singlet	1	-463.0154917	-162.7699483	-347.7379607	EMD2
TTGCATTGTTCGTAAC-1	singlet	1	-314.2761617	-136.04583	-267.4601668	EMD2
TTGCATTTCCGCCTAT-1	singlet	0	-683.035471	-2249.011876	-2182.471345	EMD2
TTGCCTGAGAGAGGTA-1	singlet	1	-2836.244378	-795.717621	-2406.321011	EMD2
TTGCCTGAGTCGGCAA-1	singlet	1	-521.4084281	-164.7256988	-438.2182917	EMD2
TTGCCTGCAGACCATT-1	singlet	1	-389.4372792	-158.6800524	-347.8387477	EMD2
TTGCCTGCAGGCACAA-1	doublet	45293	-2515.764183	-1317.119264	-1909.855593	EMD2
TTGCCTGCAGGTTCCG-1	singlet	1	-2101.668035	-725.1734001	-1654.067051	EMD2
TTGCCTGGTCGCGTCA-1	singlet	1	-1545.778849	-423.4343848	-1140.025725	EMD2
TTGCGTCAGTCGTCTA-1	singlet	0	-126.4862346	-319.8609808	-281.757731	EMD2
TTGCGTCGTCGTCTCT-1	singlet	2	-750.6341574	-608.8723315	-269.3809643	EMD2
TTGCGTCTCGTTCCTG-1	singlet	1	-375.813936	-133.8003974	-321.1551691	EMD2
TTGCGTCTCTAGGCCG-1	doublet	0/1	-635.7211846	-669.203105	-786.8646177	EMD2
TTGCTGCAGACAGCGT-1	singlet	1	-1132.015143	-380.4321939	-1199.739996	EMD2
TTGCTGCAGAGGTGCT-1	doublet	45323	-2766.689645	-1802.424271	-1576.469135	EMD2
TTGCTGCCAAACAGGC-1	singlet	1	-442.2648946	-135.0234633	-314.6562251	EMD2
TTGCTGCCAAAGACTA-1	doublet	0/2	-2330.240854	-3655.557349	-2642.937033	EMD2
TTGCTGCCAATAGTAG-1	singlet	1	-416.4612543	-191.0555722	-381.9707515	EMD2
TTGCTGCCAGAAATCA-1	singlet	0	-126.4011666	-415.9030058	-417.8935858	EMD2
TTGCTGCCAGCTCTGG-1	singlet	0	-887.7360669	-2471.148107	-2352.970746	EMD2
TTGCTGCCATGCGGTC-1	singlet	1	-5281.286574	-1373.730768	-3767.259257	EMD2
TTGCTGCGTACCTGTA-1	singlet	0	-473.6076848	-1143.192489	-1154.733575	EMD2
TTGCTGCGTGAATATG-1	singlet	1	-1281.825299	-406.1346674	-1096.643975	EMD2
TTGCTGCGTGTCCAAT-1	singlet	1	-4776.998833	-1456.216913	-4010.351169	EMD2
TTGCTGCGTGTTTCTT-1	singlet	1	-1587.611304	-505.3319472	-1322.742907	EMD2
TTGCTGCTCATATGGC-1	singlet	1	-36.56973155	-10.44090229	-28.38350154	EMD2
TTGCTGCTCCAATCCC-1	singlet	1	-399.7959218	-173.729996	-353.3547538	EMD2
TTGCTGCTCCACATAG-1	singlet	2	-387.1376941	-372.5680552	-169.9356644	EMD2
TTGCTGCTCGACATAC-1	singlet	0	-524.7343195	-1553.38514	-1431.145184	EMD2
TTGGATGAGGAAAGGT-1	singlet	0	-332.0407685	-1024.127112	-928.379477	EMD2
TTGGATGCAAAGTGTA-1	singlet	1	-2861.31554	-794.7442439	-2133.826603	EMD2
TTGGATGCAAATGAAC-1	singlet	1	-521.9266541	-162.9271031	-482.7756299	EMD2
TTGGATGCAATGTCTG-1	singlet	0	-56.81114585	-198.0426387	-174.1442074	EMD2
TTGGATGCACGACTAT-1	singlet	1	-2216.714265	-687.8995766	-1796.740804	EMD2
TTGGATGGTACTGTTG-1	singlet	0	-1104.603418	-2201.711441	-2357.746814	EMD2
TTGGATGGTGACCGTC-1	singlet	1	-573.6092165	-166.7534448	-405.0400721	EMD2
TTGGATGGTGTATTCG-1	singlet	1	-407.9711995	-144.0542592	-318.2607176	EMD2
TTGGATGGTTAAACCC-1	singlet	1	-722.4000454	-301.9709895	-590.3960947	EMD2
TTGGATGTCAAGCTGT-1	singlet	0	-884.4833577	-2581.919243	-2435.434602	EMD2
TTGGATGTCGTTATCT-1	singlet	0	-461.9986526	-1475.902202	-1432.261872	EMD2
TTGGATGTCTTGGATG-1	singlet	1	-3498.49824	-1143.432022	-2809.389984	EMD2
TTGGGATAGCCTGAGA-1	singlet	1	-428.1044265	-160.2325918	-392.1258133	EMD2
TTGGGATCAGGTTCGC-1	singlet	0	-151.4071434	-518.2496454	-447.6690278	EMD2
TTGGGATCATTATGCG-1	singlet	0	-2801.833659	-8415.017838	-8352.719918	EMD2
TTGGGATGTGGATCGA-1	singlet	2	-1066.891379	-852.3355009	-258.8203643	EMD2
TTGGGATTCAATGCAC-1	doublet	1/0	-2390.778277	-1638.844748	-2571.257275	EMD2
TTGGGATTCATGCCCT-1	singlet	1	-230.0447784	-64.45671358	-204.361732	EMD2
TTGGGATTCGTTAGAC-1	singlet	2	-1270.179237	-934.9721146	-425.7421758	EMD2
TTGGGATTCGTTGTAG-1	singlet	0	-1922.201848	-6610.716396	-6005.519541	EMD2
TTGGGCGAGAGTTGCG-1	singlet	1	-1726.821155	-553.7471242	-1485.722972	EMD2
TTGGGCGAGCACCTGC-1	singlet	1	-191.7526268	-65.02053318	-166.502633	EMD2
TTGGGCGCAACGTATC-1	singlet	1	-2916.387535	-826.932397	-2456.307691	EMD2
TTGGGCGCAATAGGAT-1	singlet	2	-2535.377334	-2198.578875	-993.3024559	EMD2
TTGGGCGCAGGATTCT-1	singlet	1	-356.7924313	-157.8982317	-301.5998753	EMD2
TTGGGCGCAGTGGGTA-1	singlet	0	-906.9621888	-3326.710275	-3302.871075	EMD2
TTGGGCGCATGAGAAT-1	singlet	1	-1009.118311	-324.0854993	-663.8305354	EMD2
TTGGGCGGTACTGACT-1	singlet	1	-1612.360075	-578.064398	-1643.84465	EMD2
TTGGGCGGTCGACTTA-1	singlet	2	-1005.556406	-852.9912992	-356.3138273	EMD2
TTGGGCGTCAAGAGTA-1	singlet	0	-342.4699069	-1030.674058	-958.9514008	EMD2
TTGGGCGTCAGCGCAC-1	singlet	2	-2008.624218	-1554.886615	-556.8179866	EMD2
TTGGGCGTCGGAGATG-1	singlet	0	-200.3063261	-851.0877875	-739.0054782	EMD2
TTGGGCGTCTGAGGTT-1	singlet	2	-292.9028515	-311.5853679	-143.0930084	EMD2
TTGGGTACACCTATCC-1	doublet	45323	-3233.651587	-2097.268192	-1961.034372	EMD2
TTGGGTAGTCAAAGAT-1	singlet	0	-74.92880535	-186.249894	-193.7513034	EMD2
TTGGGTAGTCCAAGAG-1	singlet	0	-1846.092024	-6637.072486	-6650.261452	EMD2
TTGGGTAGTGTCTTGA-1	singlet	1	-179.1573054	-61.79165638	-110.8535264	EMD2
TTGGGTAGTGTGTTTG-1	singlet	1	-2326.77826	-618.364805	-1757.876568	EMD2
TTGGGTAGTTGTGGAG-1	singlet	1	-193.6039242	-82.01264285	-150.5070166	EMD2
TTGGGTAGTTGTTTGG-1	singlet	1	-2126.459834	-740.9306234	-1894.93485	EMD2
TTGGGTATCTCCGCAT-1	singlet	2	-4668.209987	-3385.689376	-1773.033968	EMD2
TTGGTTTAGATCCTAC-1	singlet	0	-1129.300091	-2970.021072	-3022.489791	EMD2
TTGGTTTCAAACGAGC-1	unassigned	1	-2.666866256	-1.680330579	-1.751476131	EMD2
TTGGTTTCACAGCCAC-1	singlet	1	-3330.883652	-1119.241712	-2967.240203	EMD2

TTGGTTTCACGTCTCT-1	singlet	2	-483.9166195	-454.7080664	-240.6333357	EMD2
TTGGTTTCAGCCATTA-1	doublet	2/0	-537.9101061	-647.6756761	-464.0097869	EMD2
TTGGTTTCATAGAGGC-1	singlet	1	-252.4597051	-124.1187722	-214.5053315	EMD2
TTGGTTTCATTACGGT-1	singlet	0	-1347.975962	-3262.870912	-3210.71912	EMD2
TTGGTTTGTAGCGCCT-1	singlet	1	-2902.988167	-834.0806302	-2177.700108	EMD2
TTGGTTTGTGACGCCT-1	singlet	1	-1735.106734	-569.6513828	-1476.137515	EMD2
TTGGTTTGTTGTTGAC-1	singlet	1	-457.8924061	-172.3333866	-384.1248527	EMD2
TTGGTTTTCCATCTGC-1	singlet	0	-882.6167524	-2769.282692	-2542.472784	EMD2
TTGGTTTTCCGACGGT-1	singlet	2	-2465.681972	-2138.665871	-766.2821061	EMD2
TTGGTTTTCGCTTGAA-1	singlet	1	-383.6534376	-133.1074845	-277.6766894	EMD2
TTGGTTTTCTCCTACG-1	singlet	1	-792.3060811	-294.0327497	-609.9158663	EMD2
TTGTGGAAGCGTATGG-1	singlet	1	-2672.993367	-786.2932804	-2109.485985	EMD2
TTGTGGAAGGAAAGAC-1	singlet	1	-95.53473198	-38.97991148	-72.52619357	EMD2
TTGTGGAAGGTGCATG-1	singlet	1	-194.9624026	-43.83037092	-121.9322847	EMD2
TTGTGGACAATTGCTG-1	singlet	0	-447.8962999	-1377.178782	-1338.615499	EMD2
TTGTGGACAGACAATA-1	singlet	2	-4737.341126	-3768.379778	-1065.46258	EMD2
TTGTGGAGTCAGACGA-1	singlet	2	-801.1835846	-774.3960609	-242.025372	EMD2
TTGTGGAGTCGCATTA-1	doublet	0/1	-1901.010307	-2662.838972	-3372.66783	EMD2
TTGTGGAGTCTTTCTA-1	singlet	1	-1561.483483	-458.0558372	-1180.163204	EMD2
TTGTGGAGTGAGGAAA-1	singlet	1	-3920.320864	-1191.361204	-3107.20682	EMD2
TTGTGGAGTGTCCCTT-1	singlet	1	-353.9668749	-132.9052438	-287.4027483	EMD2
TTGTGGAGTTGGGTTT-1	singlet	1	-493.3013381	-150.1974276	-425.0719339	EMD2
TTGTGGATCAGATTGC-1	singlet	1	-130.3699539	-43.35592699	-89.62197281	EMD2
TTGTGGATCGTTCAGA-1	singlet	2	-468.5700651	-423.7862346	-172.4504218	EMD2
TTGTGGATCTCCCAAC-1	singlet	1	-437.4737112	-176.0561201	-407.0389736	EMD2
TTGTGTTAGACGGAAA-1	singlet	2	-1572.071966	-1438.7369	-519.1383071	EMD2
TTGTGTTAGAGAGGTA-1	singlet	2	-348.427945	-291.2511865	-81.11111453	EMD2
TTGTGTTAGCGTCTGC-1	singlet	0	-456.6468067	-1439.417274	-1268.647226	EMD2
TTGTGTTAGGTCGTAG-1	singlet	0	-199.5727843	-604.4827028	-539.9647288	EMD2
TTGTGTTCAAAGGGTC-1	singlet	1	-139.7977625	-51.97599003	-110.353339	EMD2
TTGTGTTGTAGTGCGA-1	singlet	0	-44.73543747	-106.2361857	-146.2278065	EMD2
TTGTGTTGTCAAAGAT-1	singlet	2	-768.5955747	-692.0807324	-276.7693121	EMD2
TTGTGTTGTCTCAGAT-1	singlet	1	-333.186453	-120.3023802	-235.8990408	EMD2
TTGTGTTGTTATCTGG-1	singlet	1	-1508.560349	-428.9246889	-1105.527268	EMD2
TTGTGTTTCCTGGGTG-1	singlet	0	-626.7930175	-1817.379128	-1591.785909	EMD2
TTGTGTTTCGGACTTA-1	singlet	1	-213.7755163	-91.06755343	-193.4144741	EMD2
TTGTGTTTCTCCGAAA-1	singlet	0	-226.7040084	-672.4355509	-618.6494264	EMD2
TTGTGTTTCTGCTTTA-1	singlet	2	-3249.5898	-2650.390412	-1039.435157	EMD2
TTGTTCAAGCAGAAAG-1	singlet	1	-1222.730704	-402.2390081	-1149.102182	EMD2
TTGTTCAAGGACTGGT-1	singlet	1	-342.3580399	-133.3353685	-326.2563878	EMD2
TTGTTCAAGGCGTCCT-1	singlet	1	-2151.095845	-744.6412739	-1706.219621	EMD2
TTGTTCAAGGCTAACG-1	singlet	1	-1680.644904	-575.2884852	-1398.957056	EMD2
TTGTTCAAGGTCGACA-1	singlet	1	-1588.787452	-422.5890513	-1496.302119	EMD2
TTGTTCACAAAGCTCT-1	singlet	1	-1597.106575	-561.4386635	-1447.327931	EMD2
TTGTTCACACCTTCGT-1	singlet	2	-4516.155285	-3865.856474	-1498.689802	EMD2
TTGTTCACAGACGCTC-1	singlet	1	-358.7305582	-125.9025628	-328.8817666	EMD2
TTGTTCACAGCTACCG-1	unassigned	0/2	-80.56591749	-154.0038252	-131.8760443	EMD2
TTGTTCACATTAGGAA-1	singlet	0	-412.4141007	-1176.004991	-1079.799059	EMD2
TTGTTCAGTCAGGCAA-1	singlet	0	-436.9012118	-1683.490316	-1274.13453	EMD2
TTGTTCAGTCTACAAC-1	singlet	1	-502.3610942	-212.5580798	-431.0112444	EMD2
TTGTTCAGTGTCATTG-1	singlet	1	-437.7796473	-174.0945241	-375.026798	EMD2
TTGTTCATCAAATAGG-1	singlet	0	-645.5432876	-2105.620229	-2124.173868	EMD2
TTGTTCATCACTCGAA-1	doublet	45293	-1959.56132	-1129.58133	-1371.12554	EMD2
TTGTTCATCGTTTACT-1	singlet	1	-1461.388748	-484.3514308	-1307.51527	EMD2
TTGTTGTAGCGTTGTT-1	singlet	1	-257.4519871	-128.3866773	-248.8640659	EMD2
TTGTTGTAGGGACTGT-1	singlet	1	-307.4903565	-105.3922997	-255.017324	EMD2
TTGTTGTCATTCGATG-1	singlet	1	-126.1305557	-38.48683422	-99.54604398	EMD2
TTGTTGTGTGCCTACG-1	singlet	2	-2372.2874	-1823.187798	-858.0169242	EMD2
TTGTTGTGTGCGTTTA-1	singlet	0	-1102.292906	-3517.412904	-3222.338663	EMD2
TTGTTGTGTGCTTCAA-1	doublet	45323	-1334.676866	-929.8105781	-812.1908492	EMD2
TTGTTGTGTTCCGCTT-1	singlet	1	-371.4941323	-147.914706	-307.1767192	EMD2
TTGTTGTTCATGCCGG-1	singlet	1	-97.06561843	-36.8700871	-76.27022092	EMD2
TTGTTTGAGAAACTGT-1	singlet	0	-893.0063816	-2462.581367	-2284.41769	EMD2
TTGTTTGAGTAAGGGA-1	singlet	0	-422.7031046	-1253.571338	-1079.897986	EMD2
TTGTTTGCACAGGATG-1	singlet	1	-2420.343983	-742.9625919	-2219.047837	EMD2
TTGTTTGCACGATAGG-1	singlet	1	-605.0889599	-247.4538778	-497.7873266	EMD2
TTGTTTGGTACTGTTG-1	singlet	2	-1613.261532	-1497.148638	-478.4746628	EMD2
TTGTTTGGTAGAGACC-1	singlet	1	-397.1875937	-159.5503977	-392.7893684	EMD2
TTGTTTGGTAGATCGG-1	singlet	0	-965.8868687	-3078.02649	-2930.415323	EMD2
TTGTTTGGTCAAGCGA-1	doublet	45293	-2408.917299	-1426.779856	-1610.913272	EMD2
TTGTTTGGTCATATGC-1	unassigned	2	-2.668483459	-1.668721546	-1.455747054	EMD2
TTGTTTGTCCACCCTA-1	singlet	1	-427.1268183	-141.7592395	-340.1899286	EMD2
TTGTTTGTCCATGAGT-1	singlet	0	-107.6405418	-314.9957726	-301.9150749	EMD2
TTGTTTGTCTAGTACG-1	singlet	1	-3506.905258	-1316.762775	-2905.95739	EMD2
TTTACCAAGCCACCGT-1	singlet	1	-182.261938	-99.91209157	-192.8738625	EMD2
TTTACCAAGGCCTTGC-1	singlet	2	-2031.868288	-1988.502616	-699.8281609	EMD2
TTTACCAAGGGAGAAT-1	singlet	2	-4149.817607	-3222.804206	-1801.979828	EMD2
TTTACCAAGTCTGTAC-1	singlet	1	-458.3213178	-164.9312354	-364.8217581	EMD2
TTTACCAAGTGATTCC-1	singlet	1	-3834.352178	-1184.306402	-3285.060321	EMD2
TTTACCAAGTTTGTCG-1	singlet	0	-1669.511749	-6470.923042	-5985.130523	EMD2
TTTACCACAGCGTTTA-1	singlet	1	-4047.67949	-1106.598116	-2996.009831	EMD2
TTTACCACATCATCTT-1	singlet	1	-1521.136095	-623.9935554	-1351.046548	EMD2
TTTACCACATCTAGAC-1	singlet	1	-3896.221033	-1432.184747	-3499.366766	EMD2
TTTACCAGTGACCGAA-1	unassigned	1	-5.990637853	-4.343624443	-6.929026895	EMD2
TTTACCAGTGCAGTGA-1	singlet	1	-1134.967269	-418.7306861	-992.6862333	EMD2
TTTACCAGTGTAAACA-1	singlet	1	-1227.063438	-478.7299639	-1101.080585	EMD2
TTTACCAGTGTTATCG-1	singlet	0	-397.4734989	-1708.775754	-1532.142715	EMD2
TTTACGTAGCGTATAA-1	singlet	1	-782.6391012	-319.7986032	-600.7466303	EMD2
TTTACGTAGCTGACTT-1	doublet	0/1	-13.65791705	-17.6541581	-22.3528409	EMD2
TTTACGTCATGGAGAC-1	singlet	1	-254.8275648	-112.4276284	-200.139906	EMD2
TTTACGTGTGACTGAG-1	singlet	2	-72.42871677	-88.00302165	-26.45110492	EMD2
TTTACGTTCACTTCTA-1	singlet	0	-290.8272483	-996.1551764	-721.2657078	EMD2
TTTACGTTCCGCTAGG-1	singlet	1	-1875.695603	-633.1072527	-1508.738919	EMD2
TTTACGTTCTCATGCC-1	singlet	0	-89.1325829	-243.8842217	-215.535057	EMD2
TTTACTGAGAGTCTGG-1	singlet	1	-170.0119409	-39.38119221	-85.67218981	EMD2
TTTACTGAGATGGTCG-1	singlet	1	-197.7234544	-81.36591972	-150.2144653	EMD2
TTTACTGAGCATGTTC-1	singlet	0	-44.4744856	-129.8416759	-149.7081114	EMD2
TTTACTGCACAACGAG-1	singlet	1	-5057.611783	-1548.877778	-4185.602714	EMD2
TTTACTGCACCAATTG-1	singlet	2	-916.7765915	-878.0395744	-331.8749011	EMD2
TTTACTGCATTGGCAT-1	singlet	1	-2007.587273	-522.0736829	-1707.193074	EMD2
TTTACTGGTCTCAAGT-1	singlet	2	-1105.646449	-1128.053788	-479.0923186	EMD2
TTTACTGTCGCTGATA-1	singlet	0	-668.340505	-2178.722628	-2019.401913	EMD2
TTTAGTCCAAACGTGG-1	singlet	1	-548.7960902	-178.006983	-413.6456738	EMD2
TTTAGTCCAAATTAGG-1	singlet	1	-2283.285464	-717.2954539	-1800.544509	EMD2
TTTAGTCCACCGTACG-1	doublet	45323	-5708.968034	-3878.065228	-2469.434476	EMD2
TTTAGTCCAGTGTATC-1	singlet	1	-1724.247929	-423.7277832	-1306.131142	EMD2
TTTAGTCCATACAGCT-1	doublet	45323	-2361.405639	-1827.311948	-1379.530412	EMD2
TTTAGTCGTAGACACG-1	singlet	0	-587.6412615	-1827.645058	-1667.195903	EMD2
TTTAGTCGTAGCTGTT-1	singlet	1	-2062.799786	-705.4552028	-1576.658879	EMD2
TTTAGTCGTGGCGTAA-1	singlet	1	-5663.768226	-1383.736885	-4388.389337	EMD2

TTTAGTCGTGTCATTG-1	singlet	1	-145.6763145	-76.64877569	-114.6125122	EMD2
TTTAGTCGTTTCGACA-1	singlet	1	-2843.72141	-795.4916441	-1929.495164	EMD2
TTTAGTCTCAAGCCCG-1	singlet	0	-201.5154315	-613.8189457	-523.4011172	EMD2
TTTAGTCTCAAGCTGT-1	singlet	1	-1649.256134	-562.6379413	-1249.243618	EMD2
TTTAGTCTCACGGACC-1	singlet	0	-1234.980701	-3912.609757	-3722.192338	EMD2
TTTAGTCTCAGCCTCT-1	singlet	1	-669.3327149	-237.1581617	-615.0076489	EMD2
TTTAGTCTCAGTCCGG-1	unassigned	45293	-5.662043903	-2.952437024	-3.405769874	EMD2
TTTAGTCTCCGTAGGC-1	singlet	0	-694.893718	-2043.320637	-1984.209935	EMD2
TTTATGCCAAGGATGC-1	singlet	1	-1819.409387	-653.2768438	-1510.654063	EMD2
TTTATGCCACGCTTAA-1	singlet	1	-2344.831136	-817.07781	-1938.58045	EMD2
TTTATGCCAGGTTTAC-1	singlet	0	-796.7384309	-2555.126562	-2315.172467	EMD2
TTTATGCGTAACAAGT-1	singlet	2	-705.3615548	-716.5676955	-280.0272814	EMD2
TTTATGCGTAGGTCAG-1	singlet	1	-912.0628735	-288.6538795	-671.6952961	EMD2
TTTATGCGTTGCCATA-1	singlet	1	-239.1882899	-108.589278	-256.4237739	EMD2
TTTATGCTCACTTATC-1	singlet	2	-1663.279812	-1682.941277	-608.6366881	EMD2
TTTATGCTCCCTCTTT-1	singlet	2	-1559.236622	-1452.430185	-534.1000166	EMD2
TTTCACAAGGTATCTC-1	singlet	1	-1783.281972	-463.0772824	-1411.557413	EMD2
TTTCACACACAGCCAC-1	singlet	2	-671.3702671	-703.2616625	-254.4494135	EMD2
TTTCACAGTAGCTAAA-1	singlet	2	-2965.315959	-2411.499092	-1046.259306	EMD2
TTTCACAGTCACCCTT-1	singlet	1	-1647.524796	-579.4173513	-1383.568316	EMD2
TTTCACAGTTTACCAG-1	singlet	1	-4610.638916	-1280.151839	-3414.771718	EMD2
TTTCACATCATTCCTA-1	singlet	1	-280.3628093	-104.2756578	-259.7267326	EMD2
TTTCAGTCAATCGAAA-1	singlet	2	-8018.814452	-7296.547179	-2346.067677	EMD2
TTTCAGTCAGCCCAGT-1	singlet	1	-430.1351656	-160.7416856	-366.0707374	EMD2
TTTCAGTGTATTGGCT-1	singlet	2	-1421.668343	-1553.094566	-608.42098	EMD2
TTTCAGTGTCACTCGG-1	singlet	0	-237.1032118	-761.4879289	-735.3868096	EMD2
TTTCAGTGTCGGTAAG-1	singlet	2	-1296.193341	-1184.413561	-477.7362526	EMD2
TTTCAGTGTTACACAC-1	singlet	1	-285.5538638	-136.4677677	-240.6995155	EMD2
TTTCAGTTCTTAATCC-1	singlet	0	-516.5066288	-1545.551889	-1462.738106	EMD2
TTTCATGAGCTTTGTG-1	doublet	0/2	-3125.36939	-5204.852767	-3824.679373	EMD2
TTTCATGAGGACGGAG-1	singlet	1	-2550.558083	-780.8914875	-2145.779127	EMD2
TTTCATGAGGAGAATG-1	singlet	1	-5131.154688	-1668.091183	-4116.094056	EMD2
TTTCATGAGGTGCAGT-1	singlet	1	-1687.97404	-512.825143	-1327.058124	EMD2
TTTCATGCACACAGCC-1	singlet	2	-77.49541276	-66.27929724	-28.60126977	EMD2
TTTCATGCACGCTGTG-1	singlet	1	-2089.057257	-694.2081142	-1667.749723	EMD2
TTTCATGCACGTGTGC-1	singlet	0	-118.9461419	-377.2884765	-309.2189647	EMD2
TTTCATGCAGCTATAC-1	singlet	0	-10.28182814	-41.81516514	-33.02765791	EMD2
TTTCATGCAGCTTCGG-1	singlet	1	-1994.736432	-642.1325924	-1829.800944	EMD2
TTTCATGCATCCTATT-1	singlet	0	-608.8863124	-1911.127178	-1653.333771	EMD2
TTTCATGGTATGCTAC-1	singlet	1	-2939.85516	-911.5219117	-2357.567943	EMD2
TTTCATGGTGACCGTC-1	singlet	1	-182.0466482	-66.21129718	-142.6757858	EMD2
TTTCATGTCGATTCCC-1	singlet	0	-392.1415018	-1180.840838	-1156.448026	EMD2
TTTCATGTCGTACCTC-1	singlet	1	-661.0868917	-259.9091102	-561.6229976	EMD2
TTTCCTCAGAGGGTCT-1	singlet	1	-2944.896717	-842.1113579	-2074.792737	EMD2
TTTCCTCAGGCCCACT-1	singlet	1	-1064.366595	-427.2332719	-859.3260819	EMD2
TTTCCTCAGGGTGAAA-1	singlet	1	-178.5468128	-51.43526112	-131.2283623	EMD2
TTTCCTCAGGTACAAT-1	singlet	1	-854.7063998	-350.5144434	-698.762505	EMD2
TTTCCTCCAACCAACT-1	singlet	0	-1849.748647	-6252.905539	-6081.332208	EMD2
TTTCCTCCACCTGATA-1	singlet	1	-440.2426812	-153.7984788	-386.6691202	EMD2
TTTCCTCCATCACGGC-1	singlet	2	-1537.421431	-1430.032036	-499.4545822	EMD2
TTTCCTCGTTGGGAAC-1	singlet	2	-3016.326363	-2571.100888	-1039.632868	EMD2
TTTCCTCTCCGCCTAT-1	singlet	2	-1844.459217	-1763.044024	-661.061599	EMD2
TTTCCTCTCTGCGGGT-1	singlet	2	-1073.228746	-1049.258232	-364.4552776	EMD2
TTTCGATAGACCATGG-1	singlet	2	-349.7681834	-328.5583531	-166.2707655	EMD2
TTTCGATAGATTTGCC-1	singlet	0	-1428.84772	-5412.163256	-4588.314475	EMD2
TTTCGATAGCACGGAT-1	singlet	1	-2239.026363	-659.8375417	-1759.17903	EMD2
TTTCGATAGCCTCAAT-1	singlet	1	-383.8004044	-171.6968446	-359.4570122	EMD2
TTTCGATCACAAATGA-1	singlet	1	-932.602844	-345.0797315	-794.692449	EMD2
TTTCGATCACCCTAAA-1	singlet	1	-1876.313443	-672.506366	-1632.988419	EMD2
TTTCGATCACGCAAAG-1	singlet	1	-1646.843147	-569.6034227	-1420.618021	EMD2
TTTCGATCAGGCATTT-1	singlet	0	-28.79995372	-50.28912053	-65.38858558	EMD2
TTTCGATGTCAAGTTC-1	singlet	0	-1012.020372	-2850.646933	-2947.672179	EMD2
TTTCGATTCTCGTGGG-1	singlet	1	-1393.4605	-352.9374779	-961.3914343	EMD2
TTTCGATTCTTAAGGC-1	singlet	1	-1405.708679	-428.277738	-1074.731845	EMD2
TTTGACTAGAATGTTG-1	singlet	1	-113.6307515	-35.94153259	-115.2985418	EMD2
TTTGACTAGGGAACAA-1	singlet	2	-258.0707256	-226.8732418	-87.66809139	EMD2
TTTGACTCAGCTGTTA-1	singlet	1	-259.8077089	-84.38381844	-228.0145249	EMD2
TTTGACTCATCCTAAG-1	singlet	0	-198.2878753	-552.7181567	-471.7498076	EMD2
TTTGACTCATGGACAG-1	singlet	2	-4314.329273	-3699.769031	-1557.972452	EMD2
TTTGACTCATGTCAGT-1	singlet	2	-79.48797663	-69.04028332	-49.32394055	EMD2
TTTGACTGTCTGTGCG-1	singlet	1	-2158.464523	-551.52713	-1636.925608	EMD2
TTTGACTGTGTCCCTT-1	singlet	1	-1118.036302	-336.1024368	-992.9000487	EMD2
TTTGACTTCACGAGGA-1	unassigned	2	-14.80567927	-3.869430401	-2.640500019	EMD2
TTTGACTTCCAAAGGG-1	singlet	1	-477.708729	-170.0988952	-412.2050755	EMD2
TTTGACTTCTGCACCT-1	singlet	1	-683.3672938	-271.0729747	-542.8952487	EMD2
TTTGATCAGAAGAACG-1	doublet	0/1	-401.8381008	-702.4187435	-803.0782569	EMD2
TTTGATCAGACTTAAG-1	unassigned	1/0	-9.813463279	-6.939301024	-19.28390091	EMD2
TTTGATCAGATGATTG-1	singlet	1	-1548.641978	-530.7621669	-1242.371533	EMD2
TTTGATCAGCTAGAGC-1	singlet	0	-776.1947522	-1535.655877	-1730.361795	EMD2
TTTGATCAGGCGAACT-1	singlet	1	-613.9483983	-256.3023138	-540.6183865	EMD2
TTTGATCAGGTACAAT-1	singlet	1	-150.0062915	-33.14668351	-115.1804182	EMD2
TTTGATCAGTGATCGG-1	singlet	1	-549.3632198	-143.0414821	-355.5707777	EMD2
TTTGATCCAAGTTTGC-1	singlet	1	-1776.452279	-631.2915162	-1606.750816	EMD2
TTTGATCCACAGTGAG-1	singlet	1	-2704.61634	-898.6858133	-2268.161855	EMD2
TTTGATCCAGAAGTTA-1	singlet	0	-458.5860245	-1352.702865	-1251.8405	EMD2
TTTGATCCATGGTGGA-1	singlet	1	-318.6050438	-132.9055792	-262.0781515	EMD2
TTTGATCGTCTGTCAA-1	singlet	1	-183.8349708	-66.28604919	-135.6717478	EMD2
TTTGATCGTCTTCATT-1	singlet	1	-472.5111447	-190.5897947	-375.5018147	EMD2
TTTGATCGTTCCGCAG-1	singlet	1	-220.8292988	-87.96171731	-177.2820825	EMD2
TTTGATCGTTCGAACT-1	singlet	1	-374.3612689	-154.5703234	-319.7987732	EMD2
TTTGATCGTTCTCAGA-1	doublet	1/0	-3365.557167	-3131.265499	-4163.934041	EMD2
TTTGATCTCGGCTGAC-1	singlet	2	-904.9079461	-802.6646718	-328.602847	EMD2
TTTGATCTCTTCCAGC-1	singlet	2	-2492.156097	-1994.061734	-696.7506828	EMD2
TTTGGAGAGCAGAAAG-1	singlet	1	-1637.580209	-478.2423006	-1321.116457	EMD2
TTTGGAGAGCCTCTTC-1	singlet	1	-5760.789481	-1562.436596	-4223.299905	EMD2
TTTGGAGCAGTATGAA-1	singlet	1	-2102.057512	-650.0277987	-1920.110694	EMD2
TTTGGAGGTGTATTGC-1	doublet	0/2	-518.6267423	-811.5518696	-601.1749297	EMD2
TTTGGAGTCACTGGGC-1	singlet	1	-360.5480474	-151.86265	-306.0210665	EMD2
TTTGGAGTCGCTCATC-1	singlet	2	-2170.999845	-1769.14489	-794.2475592	EMD2
TTTGGAGTCTACGCAA-1	singlet	1	-843.57265	-308.646833	-756.0049944	EMD2
TTTGGTTAGCCAAGCA-1	singlet	1	-5646.091345	-1481.055951	-4064.896163	EMD2
TTTGGTTAGGTCCAGA-1	singlet	1	-5903.307406	-1336.196435	-4130.941606	EMD2
TTTGGTTCAATCGTCA-1	doublet	1/0	-4744.983586	-2363.330019	-4502.892901	EMD2
TTTGGTTCACACGTGC-1	singlet	1	-521.245289	-219.2375685	-416.7790263	EMD2
TTTGGTTGTAATGCTC-1	singlet	1	-454.935766	-124.8284773	-322.1904449	EMD2
TTTGGTTGTAGACGTG-1	singlet	1	-649.7096043	-217.9695741	-477.7333411	EMD2
TTTGGTTGTATAGGGC-1	singlet	1	-3757.679345	-1290.776345	-2941.642036	EMD2
TTTGGTTGTTGCTGAT-1	singlet	1	-1873.525151	-696.8446497	-1565.966661	EMD2
TTTGGTTTCAAGATAG-1	singlet	2	-3355.659382	-2308.900441	-1320.906079	EMD2
TTTGGTTTCCGACAGC-1	singlet	1	-993.3701019	-372.371622	-802.6463902	EMD2

TTTGTTGAGCATGATA-1	singlet	1	-340.9813837	-130.6669207	-312.8787932	EMD2
TTTGTTGAGCTATCCA-1	singlet	1	-397.4109953	-158.3276893	-313.0394533	EMD2
TTTGTTGAGGTATAGT-1	singlet	1	-1549.888079	-450.8567025	-1369.799549	EMD2
TTTGTTGCAAAGCTCT-1	singlet	0	-913.0292513	-2939.118043	-2775.408687	EMD2
TTTGTTGCAATCTCTT-1	singlet	2	-2111.682823	-2043.50818	-868.759274	EMD2
TTTGTTGCAATTGCGT-1	singlet	1	-1016.472048	-357.5656602	-743.6081429	EMD2
TTTGTTGCACCAGACC-1	singlet	1	-1310.337754	-401.6601784	-1001.810566	EMD2
TTTGTTGCACTTGAAC-1	singlet	1	-4464.134006	-993.0083168	-3294.487771	EMD2
TTTGTTGCAGGTGACA-1	singlet	2	-3896.526544	-3293.947394	-1121.822036	EMD2
TTTGTTGGTCCACGCA-1	singlet	1	-699.0992555	-163.4749577	-511.4266159	EMD2
TTTGTTGGTCCCGCAA-1	singlet	0	-751.1155197	-2191.173302	-2025.58833	EMD2
TTTGTTGGTCGTTTCC-1	singlet	2	-3073.968588	-2523.107864	-1040.280307	EMD2
TTTGTTGGTGACAACG-1	singlet	1	-1025.592515	-400.8140681	-794.97863	EMD2
TTTGTTGGTTGAAGTA-1	singlet	1	-1608.621028	-420.5383612	-1147.908521	EMD2
TTTGTTGTCACACCCT-1	singlet	2	-2304.796695	-1827.575582	-805.6518568	EMD2
TTTGTTGTCCGAGAAG-1	singlet	1	-2841.161119	-732.6972346	-2583.482973	EMD2
TTTGTTGTCCGTAATG-1	singlet	1	-1170.82843	-372.0404015	-889.0781033	EMD2
TTTGTTGTCGCTACGG-1	singlet	1	-304.6251828	-110.226431	-239.6722066	EMD2
AAACCCAAGATTGAGT-1	singlet	2	-222.4233734	-167.3508004	-58.38313023	Control1
AAACCCAAGTATAGGT-1	singlet	2	-800.398277	-602.1890871	-273.8575525	Control1
AAACCCACAAAGTGTA-1	singlet	1	-1585.807723	-616.3668989	-1538.865916	Control1
AAACCCACATGGCGCT-1	singlet	1	-854.695702	-390.340888	-752.1913098	Control1
AAACCCAGTACAGAAT-1	singlet	1	-1140.928052	-490.6924709	-1089.775891	Control1
AAACCCAGTCGTAATC-1	singlet	1	-3126.843588	-738.6926226	-3389.61629	Control1
AAACCCAGTGGCCTCA-1	singlet	1	-1457.793921	-574.2052829	-1342.062285	Control1
AAACCCATCAACTGGT-1	singlet	2	-959.7687574	-761.7016207	-342.0404279	Control1
AAACCCATCGCGTAGC-1	singlet	2	-1021.908464	-937.3698266	-402.0135458	Control1
AAACCCATCGTTCGCT-1	singlet	2	-572.791839	-568.9022105	-264.8444133	Control1
AAACGAAAGCCGTAAG-1	singlet	2	-103.3704925	-105.4684778	-44.33517466	Control1
AAACGAACACATATCG-1	singlet	1	-931.0144162	-361.1706063	-853.9901485	Control1
AAACGAAGTAATACCC-1	singlet	2	-6530.632951	-5748.160379	-2154.564112	Control1
AAACGAAGTAGGTAGC-1	singlet	2	-1634.630849	-1492.423187	-652.4129508	Control1
AAACGAAGTCGCGTTG-1	doublet	1/0	-71.52657821	-40.60749709	-47.50007803	Control1
AAACGAATCCATCTCG-1	singlet	1	-340.7351801	-137.5337034	-356.8014024	Control1
AAACGAATCCTAGCTC-1	singlet	2	-211.8686437	-213.3396449	-85.82446739	Control1
AAACGCTAGATAACAC-1	singlet	1	-216.2602208	-105.4298512	-204.7706862	Control1
AAACGCTGTAGCCAGA-1	doublet	1/0	-98.74706547	-70.07117404	-91.11507517	Control1
AAACGCTGTATGAAAC-1	singlet	2	-684.7100645	-532.8593373	-223.6735488	Control1
AAACGCTGTCCTGGGT-1	singlet	2	-2425.729397	-1866.355411	-781.1841267	Control1
AAACGCTTCCAAGCCG-1	singlet	1	-4822.045938	-1574.764174	-4664.108663	Control1
AAAGAACAGAAAGTCT-1	singlet	2	-1412.516161	-1442.274953	-445.4340051	Control1
AAAGAACAGGTTTGAA-1	singlet	2	-680.90499	-534.2701659	-221.7755565	Control1
AAAGAACAGTCATCCA-1	singlet	2	-632.7935293	-386.5953511	-177.7779342	Control1
AAAGAACGTCACTCAA-1	singlet	2	-155.0916443	-122.6375737	-80.01506257	Control1
AAAGAACGTCTCTCTG-1	doublet	45323	-6179.362107	-4357.017909	-3321.425432	Control1
AAAGGATAGAACAGGA-1	singlet	2	-84.45143335	-106.4793065	-33.89773689	Control1
AAAGGATAGTGAGGCT-1	singlet	2	-407.2525872	-377.6678708	-143.205082	Control1
AAAGGATCAACTGCTA-1	singlet	2	-489.8143077	-341.9035774	-207.5405354	Control1
AAAGGATCACAAGTTC-1	singlet	2	-847.9145004	-713.1036738	-287.0464448	Control1
AAAGGATGTCTAACGT-1	singlet	1	-1040.515883	-375.0408162	-911.2781557	Control1
AAAGGATGTGCAACAG-1	singlet	1	-7.560574011	-4.955551504	-12.52368394	Control1
AAAGGATTCTGCGGAC-1	singlet	2	-1713.397764	-1589.00177	-693.4715206	Control1
AAAGGGCAGCTCGACC-1	singlet	2	-5800.03893	-5107.712973	-2807.233367	Control1
AAAGGGCAGGCATGCA-1	singlet	1	-6974.057799	-2538.099779	-6556.474283	Control1
AAAGGGCAGTCTTGGT-1	singlet	2	-574.1665935	-454.9171493	-187.9617093	Control1
AAAGGGCCAAGACAAT-1	doublet	1/0	-1288.454743	-1009.848062	-1579.826146	Control1
AAAGGGCCAGAGCTAG-1	singlet	2	-515.0077941	-409.8755638	-207.1422292	Control1
AAAGGGCCAGGTTCCG-1	singlet	2	-522.8374652	-410.9333104	-179.3873118	Control1
AAAGGGCTCAGTAGGG-1	singlet	2	-1329.374109	-1112.489981	-544.7089446	Control1
AAAGGGCTCCCTCTTT-1	singlet	2	-586.2599629	-346.4491629	-176.8160562	Control1
AAAGGGCTCCGATCGG-1	singlet	2	-1504.293844	-1248.724582	-546.8371985	Control1
AAAGGGCTCTGGAGAG-1	singlet	2	-1414.527689	-1306.269827	-508.44724	Control1
AAAGGTACACACCTTC-1	singlet	2	-2317.255878	-2089.948741	-756.6290925	Control1
AAAGGTAGTCCACACG-1	singlet	1	-993.836648	-397.86185	-942.3510672	Control1
AAAGGTAGTCCGGCAT-1	singlet	1	-128.0735517	-59.89765122	-103.6490726	Control1
AAAGGTAGTCGACGCT-1	singlet	1	-2108.018295	-816.1215835	-1993.384294	Control1
AAAGGTATCTATTCGT-1	singlet	1	-2387.397962	-973.4968745	-2260.974294	Control1
AAAGTCCAGACCATTC-1	singlet	0	-302.8442985	-778.4132547	-1104.368215	Control1
AAAGTCCAGCAGGCTA-1	singlet	1	-1404.583094	-538.9579072	-1486.58808	Control1
AAAGTCCAGCATCGAG-1	singlet	1	-1718.781743	-562.3291447	-1564.607656	Control1
AAAGTCCCAACTGCTA-1	singlet	2	-2757.820752	-2573.40254	-889.5242996	Control1
AAAGTCCCATAATCGC-1	singlet	0	-212.7737959	-474.1518082	-574.4687627	Control1
AAAGTCCGTCCGTACG-1	singlet	2	-22.79363737	-9.533448515	-4.042519672	Control1
AAAGTCCGTTGTCTAG-1	singlet	1	-1142.403302	-488.1073913	-1137.158743	Control1
AAAGTCCTCCCTTCCC-1	singlet	1	-627.7089672	-253.5613594	-593.2678775	Control1
AAAGTCCTCGGATACT-1	singlet	2	-640.6117072	-476.5067338	-218.7039389	Control1
AAAGTGAAGAAGCCAC-1	singlet	1	-1179.542262	-449.2947757	-1084.505734	Control1
AAAGTGAAGCAGGGAG-1	singlet	1	-545.9538874	-218.4948943	-491.4754088	Control1
AAAGTGAAGCGTTCAT-1	singlet	2	-1512.525133	-1279.790297	-580.7513442	Control1
AAAGTGACAGCATACT-1	singlet	1	-1283.50883	-512.3951447	-1181.539346	Control1
AAAGTGAGTACAATAG-1	singlet	2	-1852.373281	-2079.08894	-666.6905478	Control1
AAAGTGAGTCACGCTG-1	doublet	45323	-3180.935432	-2398.704532	-2106.300817	Control1
AAAGTGAGTGTCGCTG-1	singlet	2	-3536.396464	-3052.217848	-1212.532617	Control1
AAAGTGAGTTTCACTT-1	singlet	1	-3970.849517	-1191.280506	-3652.71241	Control1
AAAGTGATCATGCCGG-1	singlet	2	-854.5542062	-539.147019	-238.7601246	Control1
AAAGTGATCCTTGGAA-1	singlet	2	-1091.420254	-1267.102369	-463.8138954	Control1
AAATGGAAGATCGCTT-1	singlet	2	-378.1009892	-270.2302665	-114.6417789	Control1
AAATGGAAGATGACAT-1	singlet	2	-801.2482852	-1118.883661	-306.5554721	Control1
AAATGGAAGCTTAAGA-1	singlet	2	-4538.384017	-3604.068492	-1010.18123	Control1
AAATGGAAGTATGATG-1	singlet	2	-1534.270785	-1443.25696	-544.1450542	Control1
AAATGGACACGGTAGA-1	singlet	1	-849.7894141	-362.6372297	-847.6187776	Control1
AAATGGAGTCATCGGC-1	singlet	1	-964.6208336	-381.7476406	-1004.403153	Control1
AAATGGATCAAAGGAT-1	singlet	1	-1684.979154	-610.2293738	-1499.339711	Control1
AAATGGATCAAATAGG-1	singlet	2	-3984.657733	-3130.216636	-1196.613116	Control1
AAATGGATCTTCTAAC-1	singlet	1	-1419.900614	-577.7458458	-1331.2635	Control1
AACAAAGAGGGCAATC-1	singlet	2	-2549.646966	-2443.131219	-937.1242617	Control1
AACAAAGCAGCATTGT-1	unassigned	45323	-3.861664171	-1.344537979	-1.330214196	Control1
AACAAAGTCCATCTGC-1	singlet	2	-2975.520584	-2535.726541	-904.6484186	Control1
AACAAAGTCGCCTCTA-1	singlet	2	-1112.700035	-979.0645568	-333.3162498	Control1
AACAACCAGAGAGTGA-1	singlet	1	-1339.034854	-442.4992989	-1224.148837	Control1
AACAACCAGCCATTTG-1	singlet	1	-1316.300823	-567.4555248	-1221.471995	Control1
AACAACCAGGTCATCT-1	singlet	1	-1688.85786	-666.6641015	-1521.609409	Control1
AACAACCCACACGTGC-1	singlet	1	-2009.300598	-703.3831061	-2084.193157	Control1
AACAACCCAGAACTAA-1	singlet	1	-1608.344985	-648.6883109	-1515.064767	Control1
AACAACCTCACTGATG-1	singlet	1	-1323.902979	-531.5170921	-1116.313321	Control1
AACAACCTCCCATGGG-1	singlet	1	-1469.484232	-647.6510076	-1408.578404	Control1
AACAACCTCCTGTAGA-1	singlet	1	-1594.527161	-636.8000501	-1463.077548	Control1
AACAACCTCCTTCTAA-1	singlet	2	-1024.463503	-941.4437723	-385.0481338	Control1
AACAACCTCGACCATA-1	singlet	2	-3575.578181	-3137.030896	-1298.060132	Control1
AACAACCTCGGCACTG-1	singlet	2	-1400.550147	-1506.080749	-515.4287167	Control1

AACAAGAAGCTTTGTG-1	unassigned	1/0	-9.74605743	-8.407803829	-11.31245212	Control1
AACAAGAAGTGCGACA-1	singlet	2	-1086.463886	-907.5698083	-349.9750977	Control1
AACAAGAGTAATTAGG-1	singlet	2	-7026.749556	-5927.018372	-2033.758353	Control1
AACAAGAGTTGCGGCT-1	singlet	1	-1557.787058	-616.2618392	-1465.496418	Control1
AACAAGATCAACACCA-1	singlet	1	-384.4420752	-147.832339	-369.5259189	Control1
AACAAGATCAGTGGGA-1	singlet	2	-380.2524809	-314.2123458	-138.2340967	Control1
AACAAGATCGATTGAC-1	singlet	2	-487.5823633	-458.3225373	-218.7243872	Control1
AACACACAGAGCCCAA-1	singlet	0	-1470.411101	-4250.822151	-5108.495424	Control1
AACACACAGCTAATGA-1	singlet	1	-4098.688561	-1552.736743	-3765.520631	Control1
AACACACAGCTGGCCT-1	singlet	1	-1262.518209	-522.3468282	-1160.423854	Control1
AACACACCAGCCCAGT-1	singlet	2	-562.2354534	-465.5082107	-204.3496544	Control1
AACACACCATAGATCC-1	singlet	1	-506.4776897	-192.9818283	-506.2427298	Control1
AACACACGTGAGATCG-1	singlet	2	-1229.731838	-1050.30381	-430.7922804	Control1
AACACACGTGCTCGTG-1	singlet	2	-493.677702	-432.2301773	-178.964885	Control1
AACACACGTTATAGAG-1	singlet	2	-1379.622942	-1325.616113	-570.7337143	Control1
AACAGGGAGACCAGAC-1	singlet	2	-419.5862177	-422.3797754	-215.3150969	Control1
AACAGGGAGAGGCTGT-1	singlet	2	-1078.902509	-934.5898916	-376.5998838	Control1
AACAGGGAGCATGCAG-1	singlet	2	-3371.930954	-6498.899925	-1173.135715	Control1
AACAGGGAGCGCCTAC-1	singlet	1	-986.425096	-374.6022724	-877.3444218	Control1
AACAGGGAGGACAAGA-1	singlet	0	-2059.114638	-6279.193361	-6643.974091	Control1
AACAGGGAGGATGCGT-1	singlet	2	-627.3668589	-540.5963683	-251.662127	Control1
AACAGGGCACCCTTAC-1	singlet	2	-697.109576	-473.5021285	-238.4234913	Control1
AACAGGGCAGAATGTA-1	singlet	0	-208.9133489	-509.4054742	-752.4536037	Control1
AACAGGGCAGTTACCA-1	singlet	2	-2638.592671	-2293.211044	-936.7485396	Control1
AACAGGGGTAACTTCG-1	doublet	0/1	-752.2115557	-761.5307203	-988.3720621	Control1
AACAGGGTCGGCAGTC-1	singlet	2	-1733.993486	-1563.817331	-670.2856524	Control1
AACAGGGTCTGTCCCA-1	singlet	2	-2280.321814	-2013.145247	-708.5022696	Control1
AACAGGGTCTTGCAGA-1	unassigned	45323	-33.056635	-19.61988705	-15.96826319	Control1
AACCAACAGAAGCTGC-1	singlet	0	-229.6987998	-684.0091315	-705.465719	Control1
AACCAACAGAGAGAAC-1	singlet	1	-829.9667773	-345.3616979	-816.6703837	Control1
AACCAACAGCTTCATG-1	singlet	1	-436.4952044	-140.2942049	-365.8014027	Control1
AACCAACAGGTCGACA-1	singlet	2	-2740.478323	-2332.605521	-951.8855482	Control1
AACCAACCAATAGGAT-1	singlet	2	-1603.641384	-1607.278106	-717.5800334	Control1
AACCAACCATCGAGCC-1	singlet	2	-2312.050037	-1864.643893	-706.153834	Control1
AACCAACGTTGAGTCT-1	singlet	0	-188.0628815	-440.3193092	-532.650912	Control1
AACCAACTCACCGCTT-1	singlet	1	-1339.994912	-606.5956903	-1225.737618	Control1
AACCAACTCAGTGATC-1	singlet	2	-1460.34741	-1184.256656	-484.8094291	Control1
AACCAACTCGTGGCTG-1	singlet	2	-1753.841319	-1589.405509	-700.5845736	Control1
AACCAACTCTGTGTGA-1	singlet	2	-1134.583421	-1086.620989	-464.1348931	Control1
AACCACAAGAGGTCAC-1	singlet	2	-1136.1898	-1057.900413	-436.4817393	Control1
AACCACAAGATGCGAC-1	singlet	0	-315.8126914	-824.8582995	-819.6900912	Control1
AACCACAAGCCATCCG-1	singlet	1	-725.2092591	-255.7110156	-592.8554825	Control1
AACCACACACTTGAGT-1	singlet	0	-938.999951	-2672.889543	-3121.950952	Control1
AACCACACAGGCAATG-1	singlet	2	-1475.67092	-1384.950951	-561.6248698	Control1
AACCACACAGTCGTTA-1	doublet	0/2	-2376.82036	-3012.324775	-2885.244753	Control1
AACCACACATTGGATC-1	singlet	2	-2962.903016	-2872.201705	-1121.005387	Control1
AACCACAGTACTAAGA-1	singlet	2	-1584.363305	-1358.839672	-576.6909817	Control1
AACCACAGTGAAAGTT-1	singlet	2	-2390.942453	-2002.497715	-801.8362353	Control1
AACCACAGTGGTTTAC-1	singlet	2	-1744.996451	-1509.811383	-751.6420025	Control1
AACCACATCACTGGGC-1	doublet	45293	-1709.905958	-987.0340457	-1208.081817	Control1
AACCACATCAGCCTTC-1	singlet	2	-1481.10216	-1186.268013	-538.2504925	Control1
AACCACATCCGTGGCA-1	singlet	1	-968.8099084	-342.2307976	-931.9296484	Control1
AACCATGAGCGTATGG-1	singlet	2	-1134.349235	-1014.652465	-422.2806624	Control1
AACCATGAGTGACCTT-1	singlet	1	-1616.341708	-631.7701242	-1360.458626	Control1
AACCATGAGTGGAAGA-1	singlet	2	-650.6555024	-767.1446941	-262.5638617	Control1
AACCATGCAACAAAGT-1	singlet	2	-739.3080095	-583.4240728	-270.3557882	Control1
AACCATGCAGGCTACC-1	singlet	2	-740.4990075	-547.964384	-277.4285756	Control1
AACCATGCATATGAAG-1	singlet	2	-2645.076798	-1997.802903	-805.9732058	Control1
AACCATGGTAGGACCA-1	singlet	2	-96.86797138	-57.47545212	-21.38996288	Control1
AACCATGGTTACCCAA-1	singlet	2	-2082.886718	-1688.03953	-703.0930845	Control1
AACCATGTCCGTAATG-1	singlet	0	-345.8194137	-1090.45779	-1111.67379	Control1
AACCATGTCGGAACTT-1	doublet	0/1	-1557.983462	-2499.090501	-2700.637119	Control1
AACCATGTCGTGCACG-1	singlet	2	-14942.25653	-12191.1704	-4121.173141	Control1
AACCCAAAGATGAATC-1	singlet	2	-131.1356847	-114.2269799	-41.70250442	Control1
AACCCAAAGGCTGGAT-1	singlet	1	-2455.652619	-1085.221698	-1824.245678	Control1
AACCCAAAGTAACGTA-1	singlet	2	-3421.005974	-2849.133659	-1189.765241	Control1
AACCCAACACCTAAAC-1	singlet	2	-1353.444623	-1249.349036	-518.0499858	Control1
AACCCAACATGATAGA-1	singlet	2	-1471.998338	-1267.351786	-463.8081704	Control1
AACCCAACATTCATCT-1	unassigned	0	-3.736944899	-5.941354242	-4.740210666	Control1
AACCCAAGTAAGCAAT-1	singlet	2	-2294.411602	-2056.664406	-848.7381027	Control1
AACCCAAGTAAGTTGA-1	singlet	2	-2932.121444	-2451.731247	-1025.385533	Control1
AACCCAAGTACGCTTA-1	singlet	2	-598.3330995	-475.6673224	-249.157425	Control1
AACCCAATCATTGCCC-1	singlet	2	-990.7269419	-688.5786703	-350.3233466	Control1
AACCTGAAGCCTCGTG-1	singlet	1	-3939.189809	-1153.501504	-3594.255746	Control1
AACCTGAAGGCCGCTT-1	singlet	2	-2497.037552	-2118.951311	-876.9400924	Control1
AACCTGAAGTAATACG-1	singlet	1	-9142.675366	-2332.139408	-8631.06908	Control1
AACCTGACAAACTGCT-1	doublet	45293	-2716.760621	-1427.440163	-1823.138948	Control1
AACCTGACAACACGTT-1	singlet	1	-2006.365605	-725.1604263	-1797.61725	Control1
AACCTGACAGAAGCGT-1	singlet	0	-1410.019837	-3927.332012	-4533.317783	Control1
AACCTGACATTGGCAT-1	singlet	2	-528.9318013	-429.4958251	-212.8872361	Control1
AACCTGAGTCCCGGTA-1	singlet	1	-2035.513587	-745.8503273	-1798.710944	Control1
AACCTGAGTCTTACTT-1	singlet	2	-920.0307041	-723.0990445	-335.2868445	Control1
AACCTGAGTTGCTAGT-1	singlet	1	-1968.774626	-765.6665034	-1861.98368	Control1
AACCTGAGTTGGGCCT-1	singlet	2	-815.2490094	-653.0107072	-284.6005765	Control1
AACCTGATCAGTCACA-1	singlet	0	-25.19183183	-47.05001512	-65.0095246	Control1
AACCTGATCTGGGCGT-1	singlet	2	-2720.073088	-2321.610645	-819.6085623	Control1
AACCTTTGTCACCGCA-1	singlet	1	-2135.754264	-796.4021552	-2016.857941	Control1
AACCTTTGTGACTGTT-1	singlet	0	-324.1493282	-908.864353	-1170.509952	Control1
AACCTTTTCGCGTAGC-1	singlet	1	-1119.009558	-470.6002961	-1162.519628	Control1
AACGAAAAGACCTGGA-1	singlet	1	-1075.96773	-374.8307217	-1022.417162	Control1
AACGAAAAGCGCACAA-1	singlet	2	-2070.491105	-1742.297632	-650.6133303	Control1
AACGAAACAGCCGGTT-1	singlet	2	-2771.764532	-2525.730625	-889.7337603	Control1
AACGAAAGTACATACC-1	doublet	0/2	-859.9533487	-1328.72222	-1233.832034	Control1
AACGAAAGTTGTGTAC-1	singlet	1	-959.4464776	-369.3005991	-869.1642257	Control1
AACGAAATCACTGTTT-1	singlet	2	-3602.419735	-3271.179428	-1313.057356	Control1
AACGAAATCCATAAGC-1	singlet	2	-644.609352	-521.4444773	-233.5024451	Control1
AACGAAATCGGCTCTT-1	singlet	2	-1101.408799	-904.1598585	-365.8945942	Control1
AACGAAATCGTTGCCT-1	singlet	1	-194.9315136	-71.87383587	-172.4093866	Control1
AACGGGAAGGCGCTTC-1	singlet	1	-1000.951263	-393.7010604	-946.6926942	Control1
AACGGGAAGGGTCACA-1	singlet	2	-528.6841685	-416.7463687	-214.7010288	Control1
AACGGGAAGTTCCATG-1	singlet	1	-1943.174011	-633.4831321	-1909.61303	Control1
AACGGGACATCTCCCA-1	singlet	1	-2824.458978	-904.9891817	-2455.501378	Control1
AACGGGAGTTATAGAG-1	singlet	2	-635.055481	-506.6432522	-193.7476317	Control1
AACGGGATCGGTAACT-1	singlet	2	-1127.8786	-1357.130971	-461.2949459	Control1
AACGGGATCTTCTTCC-1	unassigned	0	-2.527757738	-3.838209644	-8.925278965	Control1
AACGTCAAGACTCGAG-1	singlet	2	-398.8479475	-305.1681038	-140.7522583	Control1
AACGTCACATGTTCAG-1	singlet	2	-1659.223218	-1624.880788	-661.8127326	Control1
AACGTCAGTAACATAG-1	singlet	2	-1148.169761	-1103.891719	-417.707746	Control1
AACGTCAGTGAGGATC-1	singlet	0	-833.7261862	-2492.830112	-2906.435081	Control1
AACGTCAGTGCACAAG-1	singlet	1	-204.2133146	-39.87275457	-213.4527916	Control1

AACGTCAGTGCCAAGA-1	unassigned	0	-4.523685174	-5.186788353	-8.904380901	Control1
AACGTCAGTTCTCACC-1	singlet	1	-969.5553895	-355.6595036	-879.7384228	Control1
AACGTCAGTTGGGTAG-1	unassigned	1	-6.895810158	-1.591609545	-2.970614784	Control1
AACGTCATCACAGAGG-1	singlet	2	-2214.045027	-1829.729717	-662.568494	Control1
AACGTCATCCAAGCTA-1	singlet	2	-182.0834468	-171.9636348	-92.73345814	Control1
AACGTCATCCCGATCT-1	singlet	2	-1228.841416	-1361.253825	-564.8172624	Control1
AACGTCATCGGAGCAA-1	singlet	2	-2294.868697	-1952.07643	-784.6850193	Control1
AACGTCATCTGATGGT-1	singlet	2	-678.7338162	-446.3763598	-212.5718051	Control1
AACTTCTAGATCCAAA-1	singlet	2	-1125.73273	-972.3609651	-401.9677374	Control1
AACTTCTAGTTGAAGT-1	singlet	1	-3278.878646	-1035.797948	-3216.238888	Control1
AACTTCTCAAATTAGG-1	singlet	1	-411.1192153	-182.512737	-393.7910553	Control1
AACTTCTGTCACCGAC-1	singlet	2	-393.2915431	-338.595454	-157.5141876	Control1
AACTTCTGTGTCTAAC-1	singlet	2	-15446.86996	-13376.2213	-4814.495205	Control1
AACTTCTTCCAGCACG-1	singlet	2	-3867.004975	-3401.234812	-1380.71529	Control1
AACTTCTTCTACGCGG-1	singlet	1	-3946.5194	-1308.41959	-3597.977745	Control1
AAGAACAAGATGCAGC-1	singlet	2	-958.488804	-624.5440653	-313.8258048	Control1
AAGAACAAGCTAATGA-1	doublet	45323	-1417.518329	-1197.686182	-783.2726998	Control1
AAGAACAAGGAGGCAG-1	singlet	2	-533.7664014	-419.7794259	-169.1761081	Control1
AAGAACAAGGCTTCCG-1	singlet	1	-586.4329119	-254.0331264	-570.7902799	Control1
AAGAACAAGTAGTGCG-1	singlet	2	-531.3593945	-378.861746	-189.3312927	Control1
AAGAACAGTCTAACTG-1	singlet	0	-553.436513	-1491.970812	-1568.838775	Control1
AAGAACAGTGAGTGAC-1	singlet	2	-439.7678478	-371.4164037	-152.5459491	Control1
AAGACAAAGAATTGCA-1	singlet	1	-5158.678454	-1974.3832	-4858.539345	Control1
AAGACAAAGAGGTTAT-1	singlet	1	-1512.049399	-535.2702569	-1434.401375	Control1
AAGACAAAGGACAGTC-1	singlet	2	-1344.50175	-1146.788771	-460.7223105	Control1
AAGACAACAATAGTCC-1	singlet	2	-328.2698969	-257.6297281	-115.6643594	Control1
AAGACAACACCCTGAG-1	singlet	2	-991.5139512	-829.7199869	-337.137794	Control1
AAGACAACATACAGCT-1	singlet	2	-12541.27621	-11032.52646	-4157.780287	Control1
AAGACAACATTGAGGG-1	singlet	0	-1736.248586	-5228.312082	-5845.522731	Control1
AAGACAAGTACTAAGA-1	singlet	2	-4254.829044	-3747.297106	-1375.491804	Control1
AAGACAAGTATGTCTG-1	singlet	2	-969.0696613	-783.5855798	-333.3151406	Control1
AAGACAAGTCAGTCGC-1	singlet	2	-2041.469015	-1834.399507	-727.8078508	Control1
AAGACAAGTGGCCTCA-1	singlet	2	-2927.035394	-2349.888134	-794.111152	Control1
AAGACAAGTTCAAACC-1	singlet	1	-296.2704078	-130.0850263	-245.8521965	Control1
AAGACAATCAACACCA-1	singlet	2	-2858.088568	-2429.15506	-1012.09743	Control1
AAGACAATCACCTTGC-1	singlet	2	-560.4688548	-447.7721367	-217.9305847	Control1
AAGACAATCACTGCTC-1	singlet	2	-2265.451149	-1879.210037	-824.0672875	Control1
AAGACAATCTCAATCT-1	singlet	1	-837.2411371	-319.2644079	-823.9930738	Control1
AAGACTCCAACCACAT-1	singlet	2	-3363.89837	-2906.908533	-1083.132238	Control1
AAGACTCCAACCGGAA-1	singlet	0	-1195.278212	-3404.854347	-3635.703759	Control1
AAGACTCCAGGACTAG-1	singlet	2	-746.6758223	-640.7467323	-294.0019394	Control1
AAGACTCGTCGATTTG-1	singlet	0	-194.7605243	-544.3243121	-632.845627	Control1
AAGACTCGTCGTTGCG-1	singlet	2	-428.2693333	-301.6575342	-145.2388921	Control1
AAGACTCGTGCATACT-1	singlet	1	-1962.439603	-605.8699608	-1664.825334	Control1
AAGACTCTCCACTGAA-1	singlet	2	-157.4759909	-165.3381734	-79.51213883	Control1
AAGACTCTCGAATCCA-1	doublet	45323	-5563.518005	-3458.93912	-3343.718889	Control1
AAGATAGAGGACGGAG-1	singlet	1	-1727.502527	-612.4222065	-1650.214815	Control1
AAGATAGAGGTATCTC-1	singlet	1	-8408.792845	-2751.302848	-7193.73485	Control1
AAGATAGAGTATCTGC-1	singlet	2	-638.003765	-476.764628	-216.4622782	Control1
AAGATAGCACCTAAAC-1	singlet	1	-2341.119643	-797.7481826	-2394.464562	Control1
AAGATAGCACTAAACC-1	singlet	2	-769.8254923	-761.6030547	-321.550962	Control1
AAGATAGCACTGTCCT-1	singlet	1	-1395.477289	-569.1882424	-1362.96374	Control1
AAGATAGGTTCGGCGT-1	singlet	1	-553.5733765	-214.7264	-499.6080735	Control1
AAGATAGTCAATCGGT-1	singlet	1	-4303.373986	-1403.272921	-3967.928468	Control1
AAGATAGTCACCACAA-1	singlet	2	-757.7600356	-544.2167014	-206.1897754	Control1
AAGATAGTCCATAAGC-1	singlet	2	-641.2436143	-582.4105695	-238.0822241	Control1
AAGATAGTCCTCTCTT-1	singlet	2	-1408.798124	-1346.987272	-574.5765716	Control1
AAGCATCCAAATGAGT-1	singlet	2	-465.9867313	-317.9878773	-155.7287811	Control1
AAGCATCCACTGATTG-1	unassigned	0	-4.52137441	-5.753881664	-6.81590698	Control1
AAGCATCCAGAGCCCT-1	singlet	2	-604.2624467	-446.3824633	-252.1926715	Control1
AAGCATCCAGCCTTCT-1	doublet	45323	-3629.043788	-2728.270787	-1761.2217	Control1
AAGCATCCATGATCTG-1	singlet	2	-1513.183388	-1304.905403	-531.2952448	Control1
AAGCATCGTATGGGAC-1	singlet	2	-2747.195262	-2211.231888	-808.5861749	Control1
AAGCATCGTGCAACGA-1	singlet	2	-1214.736502	-1039.482889	-445.6638132	Control1
AAGCATCTCGCAGTTA-1	singlet	2	-2671.273706	-2417.474028	-914.320137	Control1
AAGCCATAGAGAGTGA-1	singlet	2	-455.592552	-345.8327621	-155.315617	Control1
AAGCCATAGAGCGACT-1	singlet	2	-429.8174768	-114.2485172	-54.86132155	Control1
AAGCCATAGCTGTACT-1	singlet	2	-2584.550623	-2198.459721	-900.7895697	Control1
AAGCCATAGGTTAAAC-1	singlet	2	-5258.845965	-4340.154232	-1690.788871	Control1
AAGCCATCAAGCGCAA-1	singlet	2	-450.3690045	-279.2937543	-127.6169316	Control1
AAGCCATGTACTAGCT-1	singlet	1	-2748.879152	-1087.584191	-2419.636148	Control1
AAGCCATGTCGCTCGA-1	singlet	1	-93.43889996	-38.39579249	-68.3779846	Control1
AAGCCATGTTGAAGTA-1	singlet	2	-1370.317607	-1604.872414	-511.6297855	Control1
AAGCCATGTTGGCTAT-1	singlet	1	-1200.696521	-471.2042596	-1167.642401	Control1
AAGCCATTCAGCATTG-1	singlet	2	-736.7973726	-910.6628037	-304.2716408	Control1
AAGCCATTCCAGCACG-1	singlet	1	-248.8403005	-53.76821861	-132.5392036	Control1
AAGCCATTCCCAATAG-1	singlet	0	-72.9424739	-195.7929733	-185.0471578	Control1
AAGCCATTCGCACGGT-1	singlet	2	-2359.817383	-1962.567692	-710.1530595	Control1
AAGCGAGAGACGCTCC-1	singlet	2	-886.8124233	-804.8675917	-324.3007713	Control1
AAGCGAGAGCAGGGAG-1	singlet	2	-706.2267476	-536.5007901	-240.8511266	Control1
AAGCGAGAGCGATGGT-1	singlet	2	-4664.649061	-3793.641201	-1400.07882	Control1
AAGCGAGAGTACCATC-1	singlet	2	-154.9530503	-142.0874699	-65.41452208	Control1
AAGCGAGTCCGAGCTG-1	singlet	1	-12.59467382	-2.444094753	-6.60436563	Control1
AAGCGAGTCTTGGTGA-1	singlet	2	-1380.030213	-1517.141686	-464.361651	Control1
AAGCGTTAGCCTGCCA-1	singlet	1	-1689.711871	-530.6531692	-1436.865451	Control1
AAGCGTTCACAAGTGG-1	singlet	0	-1849.116516	-5595.055592	-5893.20795	Control1
AAGCGTTGTAGGCTCC-1	singlet	2	-580.3951743	-402.2238713	-211.1104575	Control1
AAGCGTTGTCAGCGTC-1	singlet	2	-933.4844857	-1345.698889	-379.3853904	Control1
AAGCGTTTCCCTCTAG-1	singlet	2	-1114.566526	-1168.819167	-455.2088955	Control1
AAGGAATCAACGGCTC-1	singlet	2	-1906.395488	-1646.928938	-710.1998005	Control1
AAGGTAACAACAACAA-1	singlet	1	-1029.166304	-435.171925	-1022.076561	Control1
AAGGTAACATGGTACT-1	singlet	2	-625.1213174	-516.6546686	-227.284068	Control1
AAGGTAAGTGAGATAT-1	singlet	2	-888.0556894	-1048.234873	-413.7326589	Control1
AAGGTAAGTGTAGTGG-1	singlet	2	-1103.869451	-1067.902673	-406.1188317	Control1
AAGGTAAGTTGAGGAC-1	singlet	2	-945.6094905	-790.4994053	-297.6275635	Control1
AAGGTAATCATGTCAG-1	singlet	2	-3305.50209	-4570.391739	-1275.034065	Control1
AAGGTAATCTAGAACC-1	singlet	2	-3163.359859	-2625.880974	-1075.539692	Control1
AAGGTAATCTGCATAG-1	singlet	1	-796.049507	-337.1468482	-836.0325666	Control1
AAGTACCAGACCATAA-1	singlet	1	-1071.131398	-429.3313874	-996.3536403	Control1
AAGTACCAGACTCAAA-1	singlet	2	-438.2237667	-373.2014048	-166.1330323	Control1
AAGTACCCAACAGAGC-1	singlet	2	-563.8771797	-439.402164	-199.7160359	Control1
AAGTACCGTACCATAC-1	singlet	2	-276.1154284	-259.6947191	-86.74696988	Control1
AAGTACCGTCGGTGAA-1	unassigned	0/1	-7.313215229	-8.907498809	-10.30976905	Control1
AAGTACCGTGGATCGA-1	singlet	2	-689.7401869	-644.2402203	-286.2975606	Control1
AAGTACCTCCATGATG-1	singlet	2	-2578.75835	-2517.775807	-1010.06047	Control1
AAGTACCTCGCATTAG-1	singlet	1	-729.0491741	-320.1184309	-636.8480575	Control1
AAGTACCTCGTGTGGC-1	singlet	1	-655.5181298	-268.0611873	-614.4495117	Control1
AAGTCGTAGACAGCGT-1	singlet	2	-495.5776486	-373.629615	-153.2070121	Control1
AAGTCGTCAACCGTGC-1	unassigned	2/0	-9.730251847	-5.593446261	-7.285309315	Control1
AAGTCGTCAAGCGCTC-1	singlet	2	-386.6703126	-286.3431904	-137.7830497	Control1

AAGTCGTGTCACTGAT-1	singlet	2	-706.18224	-671.468443	-285.2486289	Control1
AAGTCGTGTGCTTATG-1	singlet	2	-144.8298342	-103.7913117	-54.8185551	Control1
AAGTCGTGTTGCATCA-1	singlet	2	-1404.936179	-1096.754845	-470.5196972	Control1
AAGTCGTTCATTGTGG-1	singlet	1	-1071.703565	-457.2547685	-1063.595887	Control1
AAGTGAAAGATTGAGT-1	singlet	2	-2318.448012	-2343.382381	-887.1139262	Control1
AAGTGAAAGCCTGAAG-1	singlet	2	-2598.043209	-2426.260985	-825.0931624	Control1
AAGTGAAAGCGCCTTG-1	singlet	2	-673.6749044	-472.0571959	-202.3577591	Control1
AAGTGAAAGCTGAAGC-1	singlet	2	-820.5031856	-961.8322407	-341.3083581	Control1
AAGTGAACACCGTACG-1	singlet	2	-969.9574531	-557.6642826	-324.282707	Control1
AAGTGAACACGCTTAA-1	singlet	2	-946.0519926	-631.0030274	-320.8946948	Control1
AAGTGAAGTAGTACGG-1	singlet	0	-2259.084058	-6308.457118	-6803.701466	Control1
AAGTGAAGTGCTGATT-1	singlet	1	-4199.312433	-1691.422772	-4023.303959	Control1
AAGTGAATCAACCTTT-1	singlet	2	-2695.736055	-2247.958814	-956.5083065	Control1
AAGTGAATCAGACCCG-1	singlet	2	-2028.002982	-1893.587128	-708.6304997	Control1
AAGTGAATCCACTTTA-1	singlet	2	-1854.208435	-1758.04881	-681.0549721	Control1
AAGTGAATCTCTAAGG-1	singlet	1	-336.6539871	-87.38616552	-292.1880934	Control1
AAGTTCGAGAGCATAT-1	singlet	1	-5344.702928	-1884.979216	-4966.507452	Control1
AAGTTCGAGGCATCAG-1	singlet	2	-2298.656817	-2001.917899	-769.6069076	Control1
AAGTTCGAGTAGTCTC-1	singlet	1	-3647.360692	-1063.657399	-2949.841074	Control1
AAGTTCGCAACTTGCA-1	singlet	2	-743.7306374	-723.5642524	-301.693797	Control1
AAGTTCGCACGGCTAC-1	singlet	1	-1321.074946	-425.3652526	-1178.138378	Control1
AAGTTCGGTATCAAGA-1	singlet	2	-111.1709651	-100.0093635	-37.70997086	Control1
AAGTTCGGTCCGGTGT-1	singlet	2	-880.914655	-1060.822047	-353.0358289	Control1
AAGTTCGTCAAGCCCG-1	singlet	2	-970.7705313	-832.1967574	-394.3131812	Control1
AAGTTCGTCTTTGCAT-1	unassigned	2/0	-10.66932703	-11.52383757	-8.163229912	Control1
AATAGAGAGATCGCCC-1	singlet	2	-4128.496352	-4600.369257	-1634.58412	Control1
AATAGAGAGCTATCCA-1	singlet	0	-771.4731489	-2110.574595	-2297.205647	Control1
AATAGAGAGTGATCGG-1	singlet	1	-1245.11836	-568.4487999	-1175.095636	Control1
AATAGAGCATATGCGT-1	doublet	45323	-3910.867594	-2720.02895	-1844.89776	Control1
AATAGAGCATGGGCAA-1	singlet	2	-1073.833444	-907.8007474	-372.3829896	Control1
AATAGAGTCAGGCGAA-1	singlet	1	-667.5156565	-287.6296374	-624.0694401	Control1
AATAGAGTCATTCTTG-1	singlet	1	-3421.19775	-1242.225641	-3225.172916	Control1
AATAGAGTCCTAGCGG-1	singlet	1	-1159.243378	-479.1706328	-1136.963463	Control1
AATCACGAGCACTGGA-1	singlet	1	-1503.869176	-662.4536853	-1568.688356	Control1
AATCACGAGCTCAGAG-1	singlet	2	-627.2171793	-477.1510374	-240.5059536	Control1
AATCACGAGGATCACG-1	singlet	1	-2641.474258	-1060.071482	-2565.420071	Control1
AATCACGCAAATCCCA-1	singlet	2	-580.2182868	-493.8980519	-228.0609206	Control1
AATCACGGTCACCGCA-1	singlet	2	-1361.313697	-1512.33446	-619.595422	Control1
AATCGACAGAGCAGTC-1	singlet	2	-839.2997259	-694.3719651	-184.9065391	Control1
AATCGACAGCGCCGTT-1	singlet	2	-508.5235521	-392.5481316	-200.7054376	Control1
AATCGACCACAGTATC-1	singlet	2	-2202.563123	-2038.623119	-741.1877183	Control1
AATCGACCACCAACAT-1	singlet	2	-637.1888903	-524.6039627	-230.9938725	Control1
AATCGACCATCAGCGC-1	singlet	1	-1988.644887	-715.3981247	-1948.977477	Control1
AATCGACCATGAGATA-1	singlet	1	-1715.435745	-634.5763296	-1628.687833	Control1
AATCGACTCACTCGAA-1	singlet	2	-3289.262839	-2916.614507	-1169.804954	Control1
AATCGACTCCTAGCGG-1	singlet	2	-296.7607863	-268.2233449	-111.6814464	Control1
AATCGACTCGAAGGAC-1	doublet	1/0	-1334.699004	-602.3863179	-1123.337727	Control1
AATCGACTCGAGAAAT-1	singlet	1	-1071.151543	-421.1421129	-911.2019665	Control1
AATCGACTCGCGTCGA-1	singlet	2	-921.6643544	-754.1471264	-276.6335643	Control1
AATCGACTCGGACTGC-1	singlet	2	-2357.354808	-1926.960017	-752.6983471	Control1
AATCGTGAGACTCTTG-1	singlet	2	-2760.535943	-2495.333062	-952.1100077	Control1
AATCGTGAGGATTACA-1	singlet	2	-1443.732751	-1309.029704	-564.6530532	Control1
AATCGTGCAACACGTT-1	singlet	2	-1132.199278	-910.9839531	-397.3387046	Control1
AATCGTGCACCCTTGT-1	singlet	2	-952.2132178	-878.3582025	-364.3679228	Control1
AATCGTGCACGTACAT-1	singlet	2	-1841.00063	-1584.658717	-661.8758867	Control1
AATCGTGCACTGGCCA-1	singlet	2	-541.0257306	-390.2434411	-171.9610059	Control1
AATCGTGCATAGCTGT-1	singlet	2	-2615.231743	-2284.291151	-842.7741633	Control1
AATCGTGGTAGTCGGA-1	singlet	0	-503.0295424	-1139.510826	-1317.115426	Control1
AATCGTGGTTGGTAGG-1	singlet	1	-754.0572443	-277.4200666	-682.2372363	Control1
AATCGTGTCCAAGCCG-1	singlet	1	-1311.050182	-551.3515264	-1291.817113	Control1
AATGAAGAGACAGCTG-1	singlet	2	-1396.47837	-1529.863806	-597.5169732	Control1
AATGAAGAGTGGCCTC-1	singlet	2	-575.3615228	-692.163786	-226.6014846	Control1
AATGAAGCAGCGCTTG-1	singlet	2	-1445.639663	-1278.750581	-571.3385625	Control1
AATGAAGCAGGATTCT-1	singlet	2	-681.7392004	-429.2258417	-179.4099226	Control1
AATGAAGGTTCCACGG-1	singlet	2	-1605.934738	-1454.509794	-541.3231475	Control1
AATGACCAGCGATTCT-1	singlet	2	-565.3442506	-408.0413063	-185.8520803	Control1
AATGACCAGGCCTGAA-1	singlet	2	-1700.008241	-1862.956602	-731.0521	Control1
AATGACCCATCGCTGG-1	singlet	1	-382.0220727	-165.6677101	-323.6593153	Control1
AATGACCGTCTCTCTG-1	singlet	1	-1867.20592	-757.6017836	-1985.774796	Control1
AATGACCGTTCAGGTT-1	singlet	2	-4332.726989	-3801.635731	-1370.565839	Control1
AATGCCACAATACCTG-1	singlet	2	-144.3701199	-112.5023038	-42.98042666	Control1
AATGCCACACGACGCT-1	singlet	1	-4361.992362	-1406.9321	-3861.913148	Control1
AATGCCACATAGATGA-1	singlet	2	-369.4417223	-270.6658627	-123.8484833	Control1
AATGCCAGTACGACTT-1	singlet	2	-2877.301467	-2243.401885	-767.1935073	Control1
AATGCCAGTAGGCTCC-1	singlet	2	-3796.660669	-3607.64372	-1284.87463	Control1
AATGCCAGTATCAGGG-1	singlet	2	-2529.727269	-2236.750344	-903.665475	Control1
AATGCCAGTCCAGGTC-1	singlet	2	-1072.654036	-871.1770398	-372.0012527	Control1
AATGCCAGTCGAAGCA-1	singlet	1	-1580.810276	-522.0852458	-1467.807538	Control1
AATGCCAGTGGCATCC-1	singlet	2	-1085.861096	-997.0768318	-441.9847665	Control1
AATGCCATCTAGTACG-1	singlet	2	-539.2967355	-410.3683308	-232.8032037	Control1
AATGCCATCTCAGAAC-1	singlet	1	-1203.308858	-489.6817459	-1103.745213	Control1
AATGGAAAGGTCGACA-1	singlet	1	-4145.881458	-1317.898053	-3882.48964	Control1
AATGGAACACTAGGTT-1	singlet	2	-1742.208518	-1711.815479	-713.0474767	Control1
AATGGAACAGAGCGTA-1	singlet	2	-777.5905948	-592.6689858	-194.9067698	Control1
AATGGAAGTTCTCCAC-1	singlet	2	-1956.110689	-2053.209349	-706.8223714	Control1
AATGGAATCATGCAGT-1	singlet	2	-923.58976	-740.5257121	-336.7186338	Control1
AATGGAATCTCGTGAA-1	singlet	1	-2170.326217	-811.2633642	-2035.601811	Control1
AATGGCTAGCATGCAG-1	singlet	0	-37.74356069	-81.8737051	-97.04863942	Control1
AATGGCTCAGGAGGAG-1	singlet	2	-615.6473647	-470.0349385	-246.1354393	Control1
AATGGCTCAGGCTACC-1	singlet	1	-1902.026068	-801.8280721	-1799.228765	Control1
AATGGCTTCATCTGTT-1	singlet	2	-2068.834233	-1901.180831	-778.9776475	Control1
AATGGCTTCCTCACCA-1	singlet	1	-1642.245354	-605.9019241	-1612.512254	Control1
AATTCCTAGACAAGCC-1	doublet	45323	-197.0131721	-132.0645082	-83.12592261	Control1
AATTCCTAGATGCTGG-1	singlet	2	-1665.799676	-1363.053424	-551.7726216	Control1
AATTCCTAGCATAGGC-1	singlet	1	-1983.946263	-789.626962	-1748.498782	Control1
AATTCCTAGCCTCACG-1	singlet	1	-949.8403362	-418.440996	-1006.516372	Control1
AATTCCTAGTGCGACA-1	singlet	1	-1099.777732	-397.7335586	-962.3805175	Control1
AATTCCTCAAGACAAT-1	singlet	2	-3872.587397	-3251.353565	-1215.391324	Control1
AATTCCTCAATCCTAG-1	singlet	1	-878.7728135	-394.828391	-946.7785442	Control1
AATTCCTCACGACGAA-1	singlet	2	-552.8732178	-408.761826	-149.6419091	Control1
AATTCCTCACTCATAG-1	singlet	1	-3211.223707	-850.1696115	-2007.65158	Control1
AATTCCTGTACTAGCT-1	singlet	1	-977.2361745	-358.8932189	-905.6276166	Control1
AATTCCTGTGATGAAT-1	singlet	1	-3749.854086	-1334.032302	-3481.436092	Control1
AATTTCCAGATTGCGG-1	singlet	2	-932.1083326	-1197.076644	-371.368058	Control1
AATTTCCAGGATAATC-1	singlet	2	-975.8394236	-781.0531835	-320.6298789	Control1
AATTTCCCACTCCGAG-1	singlet	1	-309.0358556	-142.9077068	-310.8741767	Control1
AATTTCCCAGCTAACT-1	singlet	0	-411.9551402	-951.9080753	-1148.30093	Control1
AATTTCCCATATACCG-1	singlet	0	-2411.578293	-7849.195132	-8903.855801	Control1
AATTTCCCATGGGATG-1	singlet	2	-402.616675	-228.1713843	-89.25713702	Control1
AATTTCCGTGACATCT-1	singlet	2	-596.1734733	-521.0933227	-268.0218784	Control1

AATTTCCGTGCTTCAA-1	singlet	1	-1602.614207	-653.8463946	-1576.801662	Control1
AATTTCCTCATCTATC-1	singlet	2	-3033.636027	-4019.808657	-1173.000304	Control1
AATTTCCTCCAACCGG-1	singlet	2	-391.2894243	-318.3975982	-174.5275281	Control1
AATTTCCTCCCAACTC-1	singlet	0	-201.8150651	-507.7223921	-571.7188046	Control1
AATTTCCTCCTACTGC-1	singlet	2	-1862.244597	-1350.356158	-829.9071172	Control1
AATTTCCTCCTCGATC-1	singlet	1	-1313.77043	-520.3612796	-1196.157536	Control1
AATTTCCTCGACCACG-1	singlet	2	-624.6519501	-439.1418804	-188.21836	Control1
AATTTCCTCGTGTCAA-1	singlet	2	-723.6852109	-509.2640623	-244.8437605	Control1
ACAAAGAAGCTTGTTG-1	singlet	1	-827.0329784	-316.65108	-816.6588277	Control1
ACAAAGACAAGAGATT-1	singlet	1	-8301.370979	-2725.441963	-7688.769049	Control1
ACAAAGACACAATGCT-1	singlet	2	-100.008829	-87.54212928	-34.33987067	Control1
ACAAAGACACATATCG-1	singlet	2	-3864.12891	-3317.097344	-1554.840141	Control1
ACAAAGACACGCGCTA-1	singlet	1	-20.02344295	-10.41458279	-24.27794646	Control1
ACAAAGACAGCAGTCC-1	singlet	2	-2283.903002	-2161.173323	-923.3960938	Control1
ACAAAGAGTATACAGA-1	singlet	2	-1718.106291	-1528.411329	-599.6724119	Control1
ACAAAGAGTCTCAGGC-1	singlet	2	-1049.366037	-1067.90526	-436.2980786	Control1
ACAAAGAGTGGACCAA-1	doublet	45323	-3789.371043	-2863.231385	-1808.991187	Control1
ACAAAGATCGAAGTGG-1	singlet	2	-1183.982752	-1049.695111	-386.1798981	Control1
ACAAAGATCGCATAGT-1	singlet	2	-761.3377469	-815.7323071	-309.8424028	Control1
ACAACCAAGCCTTGAT-1	singlet	2	-1036.37108	-740.8646911	-320.1746845	Control1
ACAACCAAGGCCTAAG-1	singlet	2	-3473.574584	-3084.507771	-985.0026925	Control1
ACAACCAAGGCTAACG-1	singlet	2	-54.3201628	-52.54561736	-23.68463651	Control1
ACAACCAAGTTCTCTT-1	singlet	2	-7190.480855	-5709.341138	-2376.353428	Control1
ACAACCACAAGATTGA-1	singlet	2	-656.20387	-596.7585804	-253.9758494	Control1
ACAACCACACTAGGTT-1	singlet	2	-3098.575955	-2717.715841	-998.707793	Control1
ACAACCAGTACTGGGA-1	singlet	2	-2021.257646	-1602.509902	-699.615477	Control1
ACAACCAGTATGCGGA-1	singlet	1	-2372.491984	-895.4748367	-2222.163137	Control1
ACAACCAGTTGAGAGC-1	singlet	2	-459.2014643	-337.2774567	-160.7102558	Control1
ACAACCATCAAGGAGC-1	singlet	2	-1097.154151	-1252.658383	-420.5002396	Control1
ACAACCATCATTACGG-1	singlet	1	-2306.141536	-868.11229	-2417.856914	Control1
ACAACCATCCTTCTGG-1	singlet	2	-1063.335313	-1105.50471	-492.7520444	Control1
ACAACCATCTCCCATG-1	singlet	1	-204.042664	-81.70011947	-243.1899816	Control1
ACAACCATCTGCTGAA-1	singlet	2	-583.5089032	-457.1541612	-238.0480236	Control1
ACAAGCTAGAAACTGT-1	singlet	2	-1254.451477	-1408.04668	-469.0861263	Control1
ACAAGCTAGCCTATTG-1	singlet	2	-793.6014612	-506.9083266	-236.254985	Control1
ACAAGCTAGCTTTCCC-1	singlet	2	-1519.795749	-1381.605108	-476.1399366	Control1
ACAAGCTAGGAGTACC-1	singlet	1	-1692.071081	-650.5950287	-1632.379209	Control1
ACAAGCTAGGTTGACG-1	singlet	2	-1677.017608	-1474.014735	-589.451888	Control1
ACAAGCTAGTTTGAGA-1	singlet	1	-962.9984547	-415.4711346	-936.9099985	Control1
ACAAGCTCAAACCACT-1	singlet	2	-8187.457316	-6828.939594	-2804.735924	Control1
ACAAGCTCAACCGTGC-1	singlet	2	-589.423098	-471.8943493	-232.9354246	Control1
ACAAGCTCACCTGTCT-1	singlet	1	-1808.57682	-644.1422001	-1481.060133	Control1
ACAAGCTCACTATCCC-1	singlet	1	-3851.968947	-1606.353536	-3559.610928	Control1
ACAAGCTGTGTCATCA-1	singlet	2	-2306.617428	-1890.477272	-748.5952371	Control1
ACAAGCTGTTAAGGGC-1	singlet	1	-2732.016382	-1241.317946	-2154.496917	Control1
ACAAGCTTCACGTAGT-1	singlet	2	-793.864404	-645.8432513	-267.2876536	Control1
ACAAGCTTCATAGGCT-1	singlet	2	-329.3574437	-297.6077017	-94.29794617	Control1
ACAAGCTTCCTCTTTC-1	singlet	2	-114.8828089	-108.489582	-38.04916769	Control1
ACAAGCTTCGTGGACC-1	singlet	2	-1007.475523	-1308.324542	-448.4166432	Control1
ACACAGTAGCATTTCG-1	singlet	1	-2532.562864	-925.9229803	-3984.493754	Control1
ACACAGTAGCCTCGTG-1	singlet	1	-4452.707995	-1561.030469	-4293.6688	Control1
ACACAGTCAAAGAACT-1	singlet	1	-1599.965714	-724.8487582	-1604.829647	Control1
ACACAGTCACAATTCG-1	singlet	1	-1341.2413	-533.9836223	-1298.182693	Control1
ACACAGTGTCGAATGG-1	singlet	2	-419.2498575	-358.7582544	-171.133338	Control1
ACACAGTGTGCCCTTT-1	singlet	2	-497.8124982	-384.3129108	-196.0353082	Control1
ACACCAAAGCAGTCTT-1	singlet	2	-1601.603436	-1538.367233	-616.323089	Control1
ACACCAAAGCATCTTG-1	singlet	2	-72.38290342	-68.05614846	-20.72342895	Control1
ACACCAAAGGCTCCCA-1	singlet	2	-733.8150297	-582.4140734	-244.289075	Control1
ACACCAAAGGTAAACT-1	singlet	1	-1112.393246	-432.5583149	-1087.741921	Control1
ACACCAAAGGTTCTTG-1	doublet	45293	-2022.644669	-1119.563073	-1288.154884	Control1
ACACCAACATCGAACT-1	singlet	2	-1779.645143	-2031.166833	-713.6689691	Control1
ACACCAAGTACAAACA-1	singlet	1	-69.09321792	-21.40840798	-59.46474885	Control1
ACACCAAGTATTTCGG-1	singlet	0	-207.210033	-539.2998797	-760.9951163	Control1
ACACCAAGTGCGCTCA-1	singlet	1	-828.7779091	-374.1532202	-804.4317928	Control1
ACACCAAGTTAGTCGT-1	singlet	1	-1417.51587	-532.0825365	-1389.498301	Control1
ACACCAATCCATTGGA-1	singlet	1	-1106.571627	-434.4558846	-1063.144689	Control1
ACACGCGAGAACTCCT-1	singlet	0	-1172.157933	-2944.637805	-3283.302122	Control1
ACACGCGAGCTCCCTT-1	singlet	1	-3038.845548	-1204.432534	-2819.581214	Control1
ACACGCGCAGGTGGAT-1	unassigned	1	-8.171382794	-4.531245468	-5.644373268	Control1
ACACGCGGTAGCGAGT-1	singlet	2	-1595.795909	-1646.536074	-552.6697629	Control1
ACACGCGGTCGCATTA-1	singlet	2	-1447.20408	-1597.331864	-598.1513628	Control1
ACACGCGGTCTCTCTG-1	singlet	1	-1342.274205	-516.5148807	-1288.042247	Control1
ACACGCGGTGATCATC-1	singlet	1	-932.0900352	-373.7847122	-887.5466865	Control1
ACACTGAAGCTCACTA-1	singlet	2	-6.538962858	-7.464310461	-2.812033158	Control1
ACACTGACAAAGCACG-1	singlet	1	-1151.587988	-421.8653774	-1013.907469	Control1
ACACTGACAATTGCTG-1	singlet	2	-2125.483456	-1730.410195	-788.4812213	Control1
ACACTGACACGACAAG-1	singlet	1	-2808.264918	-925.2753834	-2799.082463	Control1
ACACTGAGTAGAATGT-1	singlet	2	-657.4664181	-517.8244952	-247.9839317	Control1
ACACTGAGTCTACTGA-1	singlet	2	-1958.678731	-1908.51188	-698.9169123	Control1
ACACTGAGTGGAAGTC-1	singlet	2	-424.669993	-323.6505258	-168.1714011	Control1
ACACTGAGTGTAGGAC-1	singlet	1	-4068.929042	-1422.132897	-3689.535089	Control1
ACACTGATCAGGAGAC-1	singlet	2	-633.1056845	-544.8745487	-224.585872	Control1
ACACTGATCGTGACTA-1	singlet	2	-652.0147937	-474.5721761	-229.3944511	Control1
ACACTGATCTCTCGAC-1	singlet	1	-1004.575574	-372.3418915	-925.7580549	Control1
ACAGAAAAGAAGCCAC-1	singlet	1	-3677.390162	-1292.201087	-3250.350075	Control1
ACAGAAAAGAGGTTTA-1	singlet	2	-1011.546601	-1059.65305	-427.8842369	Control1
ACAGAAACAACGATCT-1	singlet	1	-2524.613723	-855.0430147	-2195.641927	Control1
ACAGAAACACATTACG-1	singlet	2	-573.9993791	-447.6952149	-204.6690041	Control1
ACAGAAACATATGAAG-1	singlet	2	-587.6352811	-430.4821065	-239.425687	Control1
ACAGAAAGTACCGGAA-1	singlet	1	-3294.996782	-1220.430764	-3121.242642	Control1
ACAGAAAGTCATCCGG-1	singlet	2	-1626.345997	-1493.060209	-616.5881556	Control1
ACAGAAAGTGTTATCG-1	singlet	2	-1191.979215	-1046.129173	-471.8672937	Control1
ACAGAAATCCTAGAGT-1	singlet	1	-3063.662181	-1118.134296	-2949.806001	Control1
ACAGAAATCTCTAAGG-1	singlet	2	-1694.01082	-1441.409424	-605.1911336	Control1
ACAGCCGAGTTGCATC-1	singlet	0	-199.6595915	-496.8933546	-694.6271384	Control1
ACAGCCGCAAACTGCT-1	singlet	2	-2812.57174	-2329.274545	-860.2915805	Control1
ACAGCCGCACGTCTCT-1	singlet	2	-653.2662056	-485.3487864	-217.8053685	Control1
ACAGCCGCATTGTCGA-1	singlet	1	-2494.21872	-813.5694204	-2530.784224	Control1
ACAGCCGGTCGAGCAA-1	singlet	2	-200.7027095	-256.7220316	-83.36108035	Control1
ACAGCCGTCAGGTAAA-1	singlet	0	-900.0092111	-2581.961366	-2852.784224	Control1
ACAGCCGTCCCATTCG-1	singlet	2	-765.5073228	-659.7369664	-344.7183648	Control1
ACAGCCGTCCTAGCTC-1	singlet	2	-1857.179129	-1909.807981	-721.6819465	Control1
ACAGCCGTCGCTCTCA-1	singlet	0	-162.277387	-374.4176641	-393.5590763	Control1
ACAGCCGTCGTTCTCG-1	singlet	2	-543.4437533	-373.0064676	-197.3074588	Control1
ACAGGGAAGACTAGAT-1	singlet	1	-1272.016552	-498.8082423	-1370.876621	Control1
ACAGGGAAGCGCACAA-1	singlet	1	-649.5847858	-246.2725331	-643.1800567	Control1
ACAGGGAAGGAACATT-1	singlet	2	-353.6453926	-268.9190061	-136.6663521	Control1
ACAGGGAAGGCTAAAT-1	singlet	2	-615.8273404	-530.2271947	-245.929676	Control1
ACAGGGAAGGTCGTCC-1	singlet	1	-793.1672703	-305.4823638	-724.8087624	Control1
ACAGGGAAGTAAACTG-1	singlet	2	-2650.005916	-2320.722823	-799.9812327	Control1

ACAGGGACAAATTGGA-1	singlet	2	-2367.207139	-2058.374446	-758.1838605	Control1
ACAGGGACATCATGAC-1	singlet	1	-137.0554376	-54.88004816	-89.90903079	Control1
ACAGGGACATGGAGAC-1	singlet	2	-1503.087265	-1272.746991	-521.3678246	Control1
ACAGGGAGTACGTACT-1	singlet	1	-923.5428951	-376.086265	-956.717564	Control1
ACAGGGAGTTCGGTAT-1	singlet	2	-1228.335205	-959.1791419	-502.7901784	Control1
ACAGGGATCGCCGAAC-1	singlet	2	-724.8421049	-620.5900798	-319.7768908	Control1
ACAGGGATCTAGACCA-1	singlet	2	-647.9370971	-627.7660445	-281.2492383	Control1
ACATCCCAGTCATGGG-1	singlet	1	-1532.436349	-581.5846136	-1392.402618	Control1
ACATCCCCAAATACAG-1	singlet	2	-508.5917196	-365.9423224	-192.2426891	Control1
ACATCCCCAATTGAAG-1	singlet	0	-283.483614	-756.8273908	-999.4702155	Control1
ACATCCCCACATACGT-1	singlet	2	-573.3459732	-408.5505427	-186.6691379	Control1
ACATCCCCACTATCCC-1	singlet	2	-6076.044756	-5536.045405	-2040.615345	Control1
ACATCCCCAGACAAAT-1	singlet	1	-3780.125735	-1346.671582	-3202.522779	Control1
ACATCCCCATACAGCT-1	singlet	1	-159.7137577	-47.63322733	-116.9541282	Control1
ACATCCCGTACAAGCG-1	singlet	1	-1039.962842	-394.8838656	-1017.081625	Control1
ACATCCCGTGACTGTT-1	singlet	2	-568.0166751	-437.2410824	-217.4436994	Control1
ACATCCCTCCCGAATA-1	singlet	2	-645.6634914	-558.0866149	-263.8161387	Control1
ACATCCCTCCTCGCAT-1	singlet	2	-469.1510503	-359.1351705	-166.4446823	Control1
ACATCCCTCGAAATCC-1	singlet	2	-1309.809975	-1929.970092	-518.9181947	Control1
ACATCGAAGCTAAATG-1	singlet	1	-2033.978278	-817.036276	-1945.726488	Control1
ACATCGAAGGTAAGTT-1	singlet	1	-147.3787276	-45.71057056	-154.5918245	Control1
ACATCGAAGTCACTCA-1	singlet	2	-1350.936985	-1253.556668	-522.1815922	Control1
ACATCGAAGTCGCCCA-1	singlet	1	-2148.170761	-779.0220187	-2166.60347	Control1
ACATCGACATTGCCGG-1	singlet	2	-281.1615491	-293.8993095	-113.7098251	Control1
ACATGCAAGACCTGGA-1	singlet	2	-289.8734461	-273.3530666	-128.1862157	Control1
ACATGCAAGAGGCTGT-1	singlet	2	-575.1202401	-530.2408426	-242.5140066	Control1
ACATGCAAGATCGGTG-1	singlet	2	-1034.713122	-923.5701571	-392.0028669	Control1
ACATGCACACGCCACA-1	singlet	2	-711.9861528	-468.9326003	-256.82529	Control1
ACATGCAGTCTCTCTG-1	singlet	2	-1029.353917	-1082.532225	-437.689037	Control1
ACATGCAGTTCGGCTG-1	singlet	2	-1729.617467	-1999.438135	-673.7562688	Control1
ACATGCATCTCTGCTG-1	singlet	2	-2053.921961	-1863.103827	-605.8135374	Control1
ACATTTCAGGCAATGC-1	singlet	2	-713.6508262	-565.2658626	-228.879673	Control1
ACATTTCAGTAATCCC-1	singlet	0	-190.7959016	-612.6593582	-545.9781799	Control1
ACATTTCCAAGAAACT-1	singlet	2	-1242.751994	-1154.870072	-481.7415416	Control1
ACATTTCCACGAGAAC-1	singlet	2	-657.0740102	-513.8771387	-258.1687532	Control1
ACATTTCCATACCACA-1	unassigned	1	-1.65806273	-0.896323555	-1.006169236	Control1
ACATTTCCATGACAGG-1	singlet	1	-2564.898163	-836.3630468	-2335.162317	Control1
ACATTTCGTATAATGG-1	singlet	1	-1585.462926	-639.8350757	-1626.136083	Control1
ACATTTCGTCGACGCT-1	singlet	2	-572.8089388	-399.685124	-209.4437743	Control1
ACATTTCGTGGATCGA-1	singlet	2	-377.3989865	-328.3890386	-167.272249	Control1
ACATTTCGTGTCGATT-1	singlet	1	-796.9235148	-292.0505364	-766.6482172	Control1
ACATTTCTCGTCAACA-1	singlet	2	-2542.498659	-2195.664885	-756.196805	Control1
ACCAAACAGCTCCGAC-1	singlet	2	-871.9817847	-695.3058187	-359.5151692	Control1
ACCAAACAGGCATCTT-1	singlet	2	-904.1819358	-906.7885906	-364.4747833	Control1
ACCAAACAGTCTTCCC-1	singlet	2	-4043.648829	-3588.092765	-1431.877456	Control1
ACCAAACCAAATGAAC-1	doublet	45323	-789.24209	-576.6433074	-292.5456171	Control1
ACCAAACGTACAAACA-1	singlet	2	-114.7184192	-104.5291737	-52.84546466	Control1
ACCAAACTCAGACTGT-1	singlet	2	-697.6560015	-567.2151003	-265.4982587	Control1
ACCAAACTCATTGAGC-1	singlet	1	-2653.721172	-1252.753866	-2084.596577	Control1
ACCAACAAGTCATAGA-1	singlet	2	-3216.484128	-2780.759596	-1032.491784	Control1
ACCAACACAGTCGGTC-1	singlet	2	-202.096761	-212.5755493	-92.13794439	Control1
ACCAACAGTATGGTAA-1	singlet	2	-5009.90527	-5066.422048	-1819.408177	Control1
ACCAACAGTCGAACGA-1	singlet	1	-741.1984323	-334.8198732	-765.5523179	Control1
ACCAACAGTCTTGTCC-1	singlet	2	-2035.008319	-1948.613597	-726.1800014	Control1
ACCAACAGTGGTTCTA-1	singlet	2	-1312.269825	-1183.359418	-513.4048571	Control1
ACCACAAAGCCTGTCG-1	singlet	2	-262.1709967	-219.6671251	-117.5097988	Control1
ACCACAACACAAAGCG-1	singlet	2	-4431.151976	-3717.224674	-1395.458978	Control1
ACCACAACAGCGTGCT-1	singlet	1	-393.5206763	-173.4583459	-353.3140311	Control1
ACCACAACATTGTCGA-1	singlet	0	-661.3038361	-1448.972843	-1617.205143	Control1
ACCACAAGTAATTAGG-1	singlet	2	-1580.319381	-1268.47149	-599.7534764	Control1
ACCACAAGTCTCGACG-1	singlet	2	-821.2463966	-678.3274757	-273.473752	Control1
ACCACAAGTGCATTAC-1	singlet	2	-2256.676173	-1679.284003	-700.9682784	Control1
ACCACAAGTTCAAAGA-1	singlet	2	-403.0495732	-412.0545741	-187.7636778	Control1
ACCACAAGTTCGAAGG-1	singlet	0	-1175.957587	-2712.054577	-2841.583933	Control1
ACCACAATCAATGTCG-1	singlet	2	-2691.966212	-2439.063834	-1046.151433	Control1
ACCACAATCTAGTCAG-1	singlet	1	-5764.327708	-1799.546231	-5264.579991	Control1
ACCATTTAGATGAAGG-1	singlet	0	-915.5974835	-2747.199346	-3006.324496	Control1
ACCATTTAGCGAACTG-1	doublet	2/0	-968.7954851	-768.230498	-392.3165353	Control1
ACCATTTAGGTTGTTC-1	singlet	2	-3140.554238	-2643.36983	-968.6313884	Control1
ACCATTTCAAGAGTAT-1	singlet	1	-2917.572801	-1415.494177	-2391.039658	Control1
ACCATTTCACGGCTAC-1	singlet	1	-1310.014791	-563.7237736	-1232.536646	Control1
ACCATTTCATTATGCG-1	singlet	2	-809.6632366	-478.6100183	-196.2692016	Control1
ACCATTTGTAGGACCA-1	singlet	2	-266.2878155	-193.8149551	-76.81723298	Control1
ACCATTTTCCACTGGG-1	singlet	1	-1286.852472	-542.7161677	-1199.132123	Control1
ACCATTTTCCGAGCTG-1	singlet	2	-195.0214969	-194.9440221	-95.78851654	Control1
ACCCAAAAGCAATAGT-1	doublet	2/0	-991.5738433	-914.8376238	-556.2017978	Control1
ACCCAAAAGGCCTTCG-1	singlet	1	-933.4844954	-407.5889795	-941.8681136	Control1
ACCCAAACAATGAACA-1	singlet	1	-4757.836423	-1814.135206	-4261.529037	Control1
ACCCAAACAGAGCTAG-1	singlet	2	-955.5787424	-840.0753701	-405.4032094	Control1
ACCCAAAGTCACTGAT-1	singlet	2	-1356.112339	-1146.363468	-411.8524631	Control1
ACCCAAAGTCCGGTGT-1	singlet	1	-217.4023712	-66.01720053	-164.9211524	Control1
ACCCAAAGTCGACGCT-1	singlet	2	-5083.100766	-4294.851527	-1282.273213	Control1
ACCCAAAGTCTATGAC-1	singlet	2	-759.0706224	-576.4375636	-276.7306678	Control1
ACCCAAAGTGCCTGAC-1	doublet	45293	-106.5147788	-50.35109493	-51.5715018	Control1
ACCCAAATCCCGATCT-1	singlet	1	-307.5378586	-105.0731821	-226.0790641	Control1
ACCCAAATCCCGTGAG-1	singlet	0	-194.8965186	-323.9659487	-389.0131894	Control1
ACCCAAATCGCGTGAC-1	singlet	1	-1319.243818	-474.4177371	-1095.986829	Control1
ACCCTCAGTCATCTAG-1	singlet	2	-769.8444948	-686.1347735	-336.7366863	Control1
ACCCTCAGTCGCTGCA-1	singlet	1	-1241.274458	-541.881465	-1105.558643	Control1
ACCCTCAGTGTCGATT-1	singlet	1	-937.3126254	-396.1088752	-949.9572557	Control1
ACCCTCATCAAATGAG-1	singlet	1	-1611.704949	-640.2574805	-1540.213437	Control1
ACCCTCATCATCACTT-1	singlet	2	-844.6537194	-697.3242311	-344.9749073	Control1
ACCCTCATCCCTCATG-1	singlet	2	-380.369279	-290.6129168	-144.3208686	Control1
ACCCTCATCCTACCAC-1	singlet	1	-4973.87144	-1988.530484	-4834.814395	Control1
ACCCTCATCGATGCTA-1	singlet	2	-516.0949285	-314.2077065	-121.9341805	Control1
ACCCTCATCTGGGAGA-1	singlet	2	-600.9008777	-494.0088015	-217.4300632	Control1
ACCCTTGAGGCTGTAG-1	singlet	2	-3698.013613	-3042.455222	-1211.521617	Control1
ACCCTTGAGGTCACTT-1	singlet	2	-2720.189809	-2267.603807	-925.361225	Control1
ACCCTTGCACTGTCGG-1	singlet	1	-2657.192518	-893.6482487	-2565.077192	Control1
ACCCTTGCAGAAATCA-1	singlet	1	-2243.976327	-732.0377331	-2057.0358	Control1
ACCCTTGCAGAGACTG-1	doublet	45293	-1391.898339	-863.8603042	-1076.468017	Control1
ACCCTTGCAGGGATAC-1	singlet	1	-1021.687767	-479.8901469	-1127.970846	Control1
ACCCTTGCATACCAGT-1	singlet	1	-654.3770725	-316.9122067	-862.2705928	Control1
ACCCTTGCATATCTGG-1	singlet	1	-1217.384575	-511.584737	-1035.333259	Control1
ACCCTTGGTGACAGGT-1	singlet	2	-1356.974698	-1099.132469	-533.7227492	Control1
ACCCTTGTCAGCCTCT-1	singlet	1	-6434.371345	-2197.51786	-6068.868314	Control1
ACCCTTGTCATTGCTT-1	singlet	2	-614.3885784	-444.0858145	-211.4331677	Control1
ACCCTTGTCGACGTCG-1	singlet	2	-462.7289513	-42.37229203	-23.78890594	Control1
ACCCTTGTCGATGCAT-1	singlet	2	-186.5641713	-183.0373374	-82.19118741	Control1
ACCGTTCAGCAGGCAT-1	singlet	2	-1179.500249	-1052.952875	-444.8531555	Control1

ACCGTTCAGCCTCATA-1	singlet	2	-6399.012407	-5362.899739	-2091.116129	Control1
ACCGTTCAGCGAGTCA-1	singlet	2	-1064.888389	-1258.227544	-449.3688162	Control1
ACCGTTCCATAGAATG-1	singlet	2	-486.7652979	-418.4232308	-167.4795379	Control1
ACCGTTCCATGTTACG-1	singlet	1	-962.338687	-428.9301376	-1023.390956	Control1
ACCGTTCGTACATTGC-1	singlet	2	-74.66269185	-53.33958044	-21.94357847	Control1
ACCGTTCGTGAATGAT-1	singlet	1	-900.8103874	-299.2528481	-771.4903619	Control1
ACCTACCAGAAGGCTC-1	singlet	2	-2658.007744	-2566.469488	-1002.535765	Control1
ACCTACCAGCGAATGC-1	singlet	2	-2683.768512	-2725.782696	-983.8516166	Control1
ACCTACCAGGTTCCAT-1	singlet	1	-147.0571171	-30.41384765	-161.5801633	Control1
ACCTACCCAGCTATTG-1	singlet	0	-1977.081928	-3846.612654	-4340.879664	Control1
ACCTACCGTAAGTTAG-1	singlet	2	-4133.202265	-3167.118597	-1165.759436	Control1
ACCTACCGTCTTACTT-1	unassigned	1/0	-9.728285797	-8.737845466	-9.073318479	Control1
ACCTGAAAGATGGGCT-1	singlet	2	-378.1927506	-234.02896	-88.72059531	Control1
ACCTGAAAGGCGAACT-1	singlet	2	-372.2817867	-257.4718766	-140.2948225	Control1
ACCTGAAAGTCTCGTA-1	singlet	2	-3070.139634	-2801.591569	-1120.189543	Control1
ACCTGAAAGTCTGGTT-1	singlet	2	-1349.80238	-1165.628273	-477.2088151	Control1
ACCTGAACAAGAGTAT-1	singlet	2	-511.4082929	-329.0159501	-160.8774343	Control1
ACCTGAACATGTGTCA-1	singlet	2	-916.8598984	-621.5479823	-296.8101987	Control1
ACCTGAACATTGAAAG-1	doublet	45293	-59.76949931	-31.67591145	-32.80611968	Control1
ACCTGAAGTAACGGTG-1	singlet	2	-951.6117729	-1111.950941	-394.1877371	Control1
ACCTGAAGTATCATGC-1	singlet	2	-1446.038341	-1447.247015	-605.1721073	Control1
ACCTGAAGTCACTCTC-1	singlet	1	-953.1219841	-381.2298408	-907.4362939	Control1
ACCTGAATCCCAGGCA-1	singlet	2	-644.3117168	-619.4013635	-209.1993765	Control1
ACCTGAATCTCATGCC-1	singlet	2	-8330.674525	-7370.754927	-2377.63765	Control1
ACCTGAATCTGCGGGT-1	unassigned	2	-3.026258367	-1.581180461	-0.911280508	Control1
ACCTGTCAGGCCCACT-1	singlet	1	-1500.519155	-573.9503523	-1400.148909	Control1
ACCTGTCCAAGCTCTA-1	singlet	2	-660.6070805	-537.7207586	-283.8907701	Control1
ACCTGTCCATTGTACG-1	singlet	2	-3960.889746	-3414.887351	-1238.621394	Control1
ACCTGTCGTCAGATTC-1	singlet	2	-605.9071189	-453.1902745	-169.0486023	Control1
ACCTGTCGTCATATGC-1	singlet	1	-2023.430403	-765.9841037	-1621.03869	Control1
ACCTGTCGTTACACTG-1	singlet	2	-422.6672455	-324.6497517	-126.4161271	Control1
ACCTGTCGTTGATCGT-1	singlet	2	-878.310225	-732.756614	-341.3476657	Control1
ACCTGTCTCCTAAGTG-1	singlet	2	-1318.010522	-1108.115287	-534.9131915	Control1
ACGATCAAGTAGGCCA-1	singlet	2	-687.8672739	-454.9065207	-220.239859	Control1
ACGATCACATCCTATT-1	singlet	2	-1891.412299	-1675.354558	-670.3247554	Control1
ACGATCAGTTAATGAG-1	singlet	2	-4559.320905	-4987.959762	-1764.133689	Control1
ACGATCATCAAGTCGT-1	singlet	2	-267.6994449	-229.79577	-94.35923416	Control1
ACGATCATCACGAACT-1	singlet	2	-727.5463898	-580.6380946	-236.4376201	Control1
ACGATCATCCAAGCAT-1	singlet	2	-1414.876236	-1262.291599	-534.5097219	Control1
ACGATCATCGTGGAAG-1	singlet	2	-1836.80037	-2237.100353	-689.1358395	Control1
ACGATGTAGTAACCGG-1	singlet	2	-676.3070045	-493.7668106	-233.3051059	Control1
ACGATGTCAATACAGA-1	singlet	0	-678.8614977	-1742.609496	-1934.427939	Control1
ACGATGTCATGACCCG-1	singlet	2	-3363.816901	-3198.095059	-1084.412639	Control1
ACGATGTGTAGTGCGA-1	singlet	2	-1945.135159	-2012.858622	-724.816527	Control1
ACGATGTGTGGATCAG-1	singlet	2	-1590.019783	-1357.427741	-494.1299145	Control1
ACGATGTGTTCTCACC-1	singlet	2	-3039.672591	-4644.259883	-1225.442723	Control1
ACGATGTTCTCTATGT-1	singlet	2	-712.1779511	-589.2025928	-264.3789262	Control1
ACGCACGAGAATACAC-1	singlet	1	-1614.024621	-671.5083039	-1484.105352	Control1
ACGCACGAGACATCCT-1	singlet	2	-3117.220748	-2573.419191	-1031.892852	Control1
ACGCACGAGGACCCAA-1	singlet	2	-1593.269945	-1516.972944	-609.1457333	Control1
ACGCACGCAATATCCG-1	unassigned	45323	-15.1859863	-12.04164343	-8.824913939	Control1
ACGCACGCAGTACTAC-1	singlet	2	-694.3556469	-465.8773009	-182.6179645	Control1
ACGCACGCATCTGCGG-1	singlet	2	-598.6592483	-369.7860868	-177.0637207	Control1
ACGCACGGTGTCTAAC-1	singlet	2	-1955.442213	-1759.103263	-661.2613833	Control1
ACGCACGGTTCGGCCA-1	singlet	1	-893.4284883	-356.2829867	-923.6963962	Control1
ACGCACGTCGCATAGT-1	singlet	2	-1295.787767	-1064.127026	-451.6574952	Control1
ACGCACGTCTACGCGG-1	singlet	2	-2050.312573	-1571.724801	-653.4155489	Control1
ACGGAAGAGAGTCAAT-1	singlet	1	-1736.660601	-544.9399545	-1502.105666	Control1
ACGGAAGAGATCGGTG-1	singlet	1	-442.3607323	-166.0730225	-414.9925166	Control1
ACGGAAGAGGATCATA-1	singlet	2	-2823.929882	-2356.860827	-833.2902927	Control1
ACGGAAGAGTACAGAT-1	singlet	2	-710.6453751	-975.3938803	-319.8747875	Control1
ACGGAAGCAGCGATTT-1	singlet	1	-659.9159305	-266.1405999	-538.7175941	Control1
ACGGAAGCATACTGAC-1	singlet	2	-587.4979669	-443.1762873	-199.9822794	Control1
ACGGAAGGTCGACTTA-1	singlet	2	-269.1358499	-256.3279829	-110.7122161	Control1
ACGGAAGGTCTCGCGA-1	singlet	1	-1140.021684	-458.7812558	-1019.853822	Control1
ACGGAAGGTGCAACAG-1	singlet	2	-1587.588951	-2017.340713	-559.3576404	Control1
ACGGAAGGTTCCTAGA-1	singlet	2	-2226.71053	-2465.168797	-868.7439645	Control1
ACGGAAGGTTGCACGC-1	singlet	0	-135.9337363	-308.239923	-347.6885787	Control1
ACGGAAGTCGACACTA-1	singlet	2	-631.3967957	-872.8384174	-219.7074076	Control1
ACGGAAGTCTGCCTGT-1	singlet	2	-1015.019587	-1186.583861	-446.5420132	Control1
ACGGGTCAGCTAAATG-1	singlet	1	-938.121395	-409.837478	-849.7104748	Control1
ACGGGTCAGGCCACTC-1	singlet	2	-1381.163591	-1299.029998	-544.7175847	Control1
ACGGGTCCAAGAGTTA-1	singlet	2	-5304.904266	-4595.583974	-1490.231182	Control1
ACGGGTCCAATATCCG-1	singlet	2	-2072.189138	-1845.05273	-762.1040208	Control1
ACGGGTCGTCGAACGA-1	singlet	2	-1123.696352	-1036.782527	-420.5705194	Control1
ACGGGTCGTCGCACAC-1	singlet	2	-2932.976635	-2367.955327	-967.4221143	Control1
ACGGGTCGTGAATTGA-1	singlet	1	-2887.423666	-907.4039245	-2365.727665	Control1
ACGGGTCTCGGTCAGC-1	singlet	2	-429.3418107	-323.1438024	-148.9353493	Control1
ACGGGTCTCTTTCTAG-1	singlet	1	-2051.069228	-809.6964585	-1871.157938	Control1
ACGGTCGAGATTGACA-1	singlet	1	-1293.352432	-504.3755493	-1285.486539	Control1
ACGGTCGAGCTAGCCC-1	singlet	2	-873.9241489	-735.2287263	-294.4165822	Control1
ACGGTCGAGGAGACCT-1	singlet	2	-1487.634224	-1285.635829	-513.7987229	Control1
ACGGTCGAGGTGATAT-1	singlet	2	-572.7734861	-556.8142396	-261.4615179	Control1
ACGGTCGCAAATCAAG-1	singlet	1	-2775.235118	-945.8664129	-2586.862794	Control1
ACGGTCGCACTTGACA-1	singlet	2	-592.4124567	-384.1224909	-187.0676605	Control1
ACGGTCGCAGGTGTGA-1	singlet	0	-52.53099508	-113.8376183	-129.775257	Control1
ACGGTCGCATGGAATA-1	singlet	2	-3062.637966	-2473.978957	-1027.597897	Control1
ACGGTCGGTGTCCGGT-1	singlet	2	-457.994043	-340.3357221	-167.8451509	Control1
ACGGTCGGTTGAAGTA-1	singlet	2	-10.1384894	-9.145928817	-1.701517572	Control1
ACGGTCGTCAAGGCTT-1	singlet	2	-567.7437919	-389.6032913	-202.9624491	Control1
ACGGTCGTCATGGCCG-1	singlet	2	-1059.891912	-1029.975945	-409.3049235	Control1
ACGGTCGTCGGTCGAC-1	singlet	2	-1082.529609	-926.1283182	-375.9758329	Control1
ACGGTCGTCTTTGATC-1	unassigned	2	-5.749792876	-2.163090463	-0.867324404	Control1
ACGGTTAAGGCTCCCA-1	singlet	0	-267.5125338	-632.8573379	-861.8734616	Control1
ACGGTTACAAAGCTAA-1	singlet	1	-1102.440809	-522.7087796	-1140.271045	Control1
ACGGTTACATCGCTGG-1	singlet	0	-1193.595993	-2629.131597	-2968.489714	Control1
ACGGTTAGTTCCCACT-1	singlet	2	-1216.610134	-1322.869842	-509.4906149	Control1
ACGGTTAGTTCCGCAG-1	singlet	2	-579.3431795	-523.2460222	-222.4497422	Control1
ACGGTTATCCGATCTC-1	doublet	45323	-3062.15645	-2243.094523	-1753.594463	Control1
ACGTAACCAACAGTGG-1	singlet	1	-1205.16942	-525.7777629	-1290.128951	Control1
ACGTAACGTGGCTGAA-1	singlet	2	-3187.049897	-2956.757847	-1161.39313	Control1
ACGTAACTCATTACCT-1	singlet	2	-587.7502625	-560.9856599	-246.2331831	Control1
ACGTACAAGCTGCGAA-1	singlet	2	-1276.509751	-1097.182306	-460.5492416	Control1
ACGTACAAGGTTAAAC-1	singlet	2	-1730.475069	-1687.227305	-611.01351	Control1
ACGTACAAGTCTTCGA-1	singlet	2	-1182.220917	-1094.160833	-428.8052878	Control1
ACGTACAGTCGAAACG-1	singlet	0	-256.1795992	-559.2986536	-670.0401661	Control1
ACGTACAGTTTACACG-1	singlet	2	-605.1870151	-456.1350487	-218.6434236	Control1
ACGTACATCGAACGCC-1	singlet	1	-11060.34046	-3778.91024	-9814.459649	Control1
ACGTAGTAGCAGGCTA-1	singlet	2	-1389.144679	-1193.703347	-535.8936582	Control1
ACGTAGTAGCCGTTAT-1	singlet	2	-1402.99334	-1558.598739	-586.2612567	Control1

ACGTAGTAGGGCTAAC-1	singlet	1	-882.0893761	-341.8905548	-821.7907128	Control1
ACGTAGTAGTAACGTA-1	singlet	2	-2023.412065	-1899.746474	-837.8972545	Control1
ACGTAGTAGTGACCTT-1	singlet	2	-2343.819928	-2012.13013	-720.8768126	Control1
ACGTAGTCATGCCGAC-1	singlet	2	-7306.253264	-6226.900866	-2279.303577	Control1
ACGTAGTGTACAGTTC-1	singlet	2	-593.2118899	-713.7199827	-231.7304788	Control1
ACGTAGTGTACGCTAT-1	unassigned	45323	-171.041273	-111.187716	-75.88825697	Control1
ACGTAGTGTTTACCAG-1	singlet	0	-105.8583281	-349.9629492	-403.126472	Control1
ACGTAGTTCCGATAAC-1	singlet	0	-175.0485579	-373.5985973	-514.8862425	Control1
ACGTCCTAGGCTAAAT-1	singlet	1	-722.649727	-247.1698587	-678.0254571	Control1
ACGTCCTCACAAGCAG-1	singlet	0	-32.65844938	-98.12692947	-88.54323728	Control1
ACGTCCTCATCCGTTC-1	singlet	2	-696.5909423	-572.1974977	-266.7681909	Control1
ACGTCCTGTATCAAGA-1	singlet	2	-1489.576828	-1532.694225	-492.6715363	Control1
ACGTCCTTCCGATCGG-1	singlet	0	-1594.643947	-4662.824099	-4879.470727	Control1
ACGTTCCAGGATACCG-1	singlet	2	-381.45933	-205.1284982	-99.06738806	Control1
ACGTTCCAGGTGCCTC-1	singlet	2	-620.7674444	-441.4327526	-233.675846	Control1
ACGTTCCAGTCATGGG-1	unassigned	0/1	-4.679429054	-7.371443272	-7.824632046	Control1
ACGTTCCAGTGTAGAT-1	singlet	2	-498.3428057	-445.1813456	-179.4880154	Control1
ACGTTCCCAAACAGGC-1	singlet	2	-519.7862512	-327.1787493	-162.6741402	Control1
ACGTTCCCAACCGCCA-1	singlet	2	-2102.832931	-1689.686855	-627.6249618	Control1
ACGTTCCCACCAGCTG-1	singlet	2	-726.0853832	-468.2616397	-215.8946852	Control1
ACGTTCCCACCCTGTT-1	singlet	2	-759.8607419	-552.4079407	-255.2996561	Control1
ACGTTCCCACTCCTGT-1	singlet	1	-2301.781919	-693.5127077	-2168.792521	Control1
ACGTTCCCAGACAAGC-1	singlet	2	-382.7109965	-305.9404608	-131.6321159	Control1
ACGTTCCGTCATAAAG-1	singlet	2	-1372.743004	-1245.761166	-573.973475	Control1
ACGTTCCTCCTCACTG-1	doublet	2/0	-13087.44443	-11722.70011	-6345.982761	Control1
ACGTTCCTCGGTGTAT-1	singlet	1	-1262.985129	-472.6765763	-1314.216614	Control1
ACTACGACACCGCTAG-1	singlet	2	-593.8434615	-414.4243143	-197.9768428	Control1
ACTACGACACGTGAGA-1	unassigned	45323	-26.09020203	-16.09758199	-14.81322905	Control1
ACTACGACAGACAATA-1	doublet	45323	-21.30029407	-30.10901774	-24.36065671	Control1
ACTACGACATATGGCT-1	singlet	2	-428.6954554	-350.5098385	-193.4634286	Control1
ACTACGAGTAGCGCCT-1	singlet	2	-643.5474488	-556.9821121	-277.785527	Control1
ACTACGAGTTAGAAGT-1	singlet	0	-402.941931	-1326.565606	-1004.885704	Control1
ACTACGAGTTAGGAGC-1	singlet	2	-114.3064193	-105.3628615	-31.18068684	Control1
ACTACGAGTTTACCAG-1	singlet	1	-1199.344038	-485.6250498	-1257.194356	Control1
ACTACGATCACTCGAA-1	singlet	0	-1743.666739	-5015.871446	-5305.756749	Control1
ACTATCTCAGATTTCG-1	singlet	2	-710.9052183	-539.9431059	-259.1616464	Control1
ACTATCTGTATGTGTC-1	singlet	2	-2805.151466	-2227.253861	-961.2099992	Control1
ACTATCTGTTCTAAGC-1	singlet	2	-678.3594238	-477.5320772	-209.9490289	Control1
ACTATGGCAAGAGGTC-1	singlet	2	-822.1440744	-736.5583039	-282.7195899	Control1
ACTATGGCACGAGGAT-1	singlet	1	-1177.674886	-529.54095	-1144.032728	Control1
ACTATGGCACGGAAGT-1	singlet	2	-104.5350899	-91.97809444	-30.52139383	Control1
ACTATGGGTTCTTGCC-1	singlet	1	-924.737428	-366.6681943	-883.7888024	Control1
ACTATGGTCCTACGAA-1	singlet	0	-828.4902484	-2038.035607	-2487.553183	Control1
ACTATGGTCGCTACAA-1	singlet	1	-3126.903508	-1125.992392	-2600.913517	Control1
ACTATTCAGCATCGAG-1	singlet	1	-2662.924241	-837.1616602	-2522.0271	Control1
ACTATTCCAAATTGGA-1	singlet	2	-524.0352662	-494.2599663	-224.6620935	Control1
ACTATTCCACCATATG-1	singlet	2	-827.3207697	-634.1894374	-306.8621939	Control1
ACTATTCGTATCGTAC-1	singlet	2	-530.8949395	-426.4324697	-206.7327238	Control1
ACTATTCGTCCATCTC-1	singlet	2	-3518.996113	-2965.595163	-1205.679291	Control1
ACTATTCGTGGCTTGC-1	singlet	2	-356.3984762	-315.6687711	-152.3043066	Control1
ACTATTCGTGTCCACG-1	singlet	2	-2175.760176	-1889.951562	-733.0273245	Control1
ACTATTCGTGTCTTAG-1	doublet	45293	-5486.260623	-3008.954781	-4524.377558	Control1
ACTATTCTCAAGAGTA-1	singlet	1	-2421.985858	-798.8884966	-2051.076488	Control1
ACTATTCTCCCATGGG-1	singlet	1	-1222.544005	-500.8993633	-1206.807195	Control1
ACTATTCTCCCATTTA-1	singlet	2	-4656.723365	-3886.762118	-1526.181333	Control1
ACTATTCTCGGAAACG-1	singlet	2	-610.5482317	-526.2068553	-263.8250809	Control1
ACTATTCTCGGTCACG-1	singlet	1	-621.2840774	-191.2438288	-503.8884294	Control1
ACTATTCTCTTTCCAA-1	doublet	2/0	-14.70897793	-16.41835124	-11.3635419	Control1
ACTCCCAAGCAATAGT-1	singlet	2	-2456.57475	-2144.818654	-850.2079678	Control1
ACTCCCAAGGAACTCG-1	unassigned	0	-5.280455536	-5.889758554	-6.572058131	Control1
ACTCCCAAGGTCTACT-1	singlet	2	-806.4251688	-560.883891	-293.1848664	Control1
ACTCCCAAGTAAATGC-1	singlet	1	-1677.897042	-648.651913	-1671.77132	Control1
ACTCCCACATTGCTTT-1	singlet	2	-5158.779225	-4380.405341	-1493.240652	Control1
ACTCCCAGTTTGTTGG-1	singlet	1	-2536.894553	-680.4802988	-2707.452017	Control1
ACTCTCGAGACTTCAC-1	singlet	2	-2109.500166	-2223.46308	-706.7980148	Control1
ACTCTCGAGGGTACGT-1	singlet	2	-499.0958046	-371.9619425	-195.3654528	Control1
ACTCTCGAGTAGATCA-1	singlet	1	-6866.994338	-1975.273417	-6285.341818	Control1
ACTCTCGCAAATACAG-1	singlet	2	-544.3939741	-390.2269721	-175.4206164	Control1
ACTCTCGCACTACACA-1	singlet	2	-9124.399662	-7544.733619	-2820.610746	Control1
ACTCTCGGTACCTTCC-1	unassigned	2	-7.900200421	-1.65719825	-1.157189177	Control1
ACTCTCGGTGTTAAAG-1	singlet	1	-1416.241332	-572.3429751	-1396.066077	Control1
ACTCTCGTCAGACCTA-1	singlet	2	-2043.417098	-1811.401448	-723.7141934	Control1
ACTCTCGTCCGTTTCG-1	singlet	2	-563.533165	-583.3338948	-242.033349	Control1
ACTGATGAGTAGCATA-1	singlet	2	-1727.887018	-1422.461949	-645.64605	Control1
ACTGATGCACGATAGG-1	singlet	1	-2207.689127	-789.9502565	-1905.31004	Control1
ACTGATGCACTAACGT-1	singlet	1	-967.0714299	-438.2800592	-920.9513782	Control1
ACTGATGCATACAGAA-1	singlet	2	-3696.588924	-3177.634385	-1249.767179	Control1
ACTGATGCATCTGGGC-1	singlet	2	-569.5635249	-468.4200738	-205.7914192	Control1
ACTGATGGTCAGGTGA-1	singlet	1	-205.7980882	-39.6978941	-207.5237969	Control1
ACTGATGGTGGGTATG-1	singlet	0	-701.7853613	-2126.170969	-2342.120201	Control1
ACTGATGGTTGTGGCC-1	singlet	2	-441.3010182	-415.5254617	-187.1663914	Control1
ACTGATGTCGGCATAT-1	singlet	2	-853.3778572	-540.0759522	-247.5161242	Control1
ACTGCAAAGACGATAT-1	singlet	2	-473.3011793	-358.0387261	-179.5301937	Control1
ACTGCAAAGAGTAACT-1	singlet	2	-1976.285401	-1959.669334	-725.4444079	Control1
ACTGCAAAGATGAAGG-1	singlet	2	-896.0093611	-827.4385128	-343.2775435	Control1
ACTGCAAAGGATTTGA-1	singlet	2	-723.2347684	-587.2104386	-282.6656484	Control1
ACTGCAAAGTAAACAC-1	singlet	2	-2769.343755	-2642.901653	-1049.495217	Control1
ACTGCAAAGTTGGAGC-1	singlet	2	-2075.815243	-1921.075715	-739.3643226	Control1
ACTGCAACAAAGAACT-1	singlet	1	-1292.003155	-491.3644474	-1174.96389	Control1
ACTGCAACAATGTGGG-1	singlet	1	-3549.784937	-1137.940121	-3289.614624	Control1
ACTGCAACAGAACGCA-1	singlet	2	-566.0242079	-425.5390264	-217.5274109	Control1
ACTGCAAGTATCAGCT-1	singlet	1	-1123.623685	-451.7684076	-1044.522818	Control1
ACTGCAAGTGATTGGG-1	singlet	0	-1696.677957	-5342.03109	-5848.820248	Control1
ACTGCAATCGGATTAC-1	singlet	2	-2903.274866	-2358.711309	-956.4012717	Control1
ACTGCAATCGTAGCCG-1	singlet	1	-562.809451	-268.6120256	-553.0484842	Control1
ACTGCAATCGTTCTCG-1	singlet	2	-2630.679297	-2750.370002	-972.5315897	Control1
ACTGCAATCTCTGGTC-1	singlet	1	-911.6218573	-370.1767448	-863.9779999	Control1
ACTGCAATCTTCGGTC-1	singlet	1	-3513.973652	-1092.818871	-3827.964954	Control1
ACTGTCCAGGTAACTA-1	singlet	1	-3816.947879	-1420.253619	-3644.754935	Control1
ACTGTCCAGTCATTGC-1	doublet	1/0	-4440.191319	-4033.229885	-5713.195878	Control1
ACTGTCCCAAGACGGT-1	singlet	2	-329.8291684	-334.080729	-150.1637074	Control1
ACTGTCCGTCGTACAT-1	singlet	2	-636.7833466	-627.6564042	-227.9564383	Control1
ACTGTCCGTTTCGGCG-1	singlet	1	-1231.536189	-441.9684868	-1072.153503	Control1
ACTGTCCTCCATTGGA-1	unassigned	0/1	-9.11782704	-10.27132678	-15.57711387	Control1
ACTGTGACACTACAGT-1	singlet	2	-682.4955381	-567.5504738	-243.6826153	Control1
ACTGTGATCATTCATC-1	singlet	2	-1531.568359	-1459.534281	-579.3013205	Control1
ACTGTGATCGTGGGAA-1	singlet	1	-3087.481973	-1000.181671	-2582.056246	Control1
ACTGTGATCGTGTTCC-1	singlet	2	-1054.902061	-1052.050149	-428.5047514	Control1
ACTGTGATCTGCATGA-1	singlet	1	-943.3078527	-425.0228272	-937.5773286	Control1
ACTTAGGAGACCATTC-1	singlet	2	-636.6889794	-431.4184807	-208.0581402	Control1

ACTTAGGAGAGGCCAT-1	singlet	2	-196.6068596	-204.554349	-89.32513694	Control1
ACTTAGGAGCGTTAGG-1	singlet	2	-5414.527136	-4146.770746	-1314.058164	Control1
ACTTAGGAGTTAACGA-1	singlet	2	-760.4168858	-574.5164976	-280.3718681	Control1
ACTTAGGAGTTTCGGT-1	singlet	1	-42.56244386	-16.01176121	-36.6247094	Control1
ACTTAGGCAGAGGCAT-1	singlet	2	-546.568318	-556.9349914	-228.0011836	Control1
ACTTAGGGTGTTCGTA-1	singlet	1	-261.5189467	-99.13936118	-240.4425608	Control1
ACTTAGGGTTATCCAG-1	unassigned	2	-3.241855931	-2.995524095	-1.66537244	Control1
ACTTAGGTCTAAGAAG-1	doublet	45293	-1153.884337	-782.0780431	-785.5925698	Control1
ACTTATCAGAATAGTC-1	singlet	2	-759.3390936	-642.2749799	-318.625852	Control1
ACTTATCAGACAGTCG-1	singlet	1	-690.9436368	-256.6309286	-648.0084234	Control1
ACTTATCAGGACTAAT-1	singlet	2	-5344.980924	-4457.306344	-1753.362868	Control1
ACTTATCGTAACAAGT-1	doublet	2/0	-266.5637033	-28.55927423	-22.05316824	Control1
ACTTATCGTTCGTGCG-1	singlet	2	-928.9624067	-855.9164891	-314.6135243	Control1
ACTTATCTCCTTCTTC-1	singlet	2	-1556.824944	-1379.629584	-624.6553732	Control1
ACTTATCTCGCGGTAC-1	singlet	0	-799.4011892	-2254.915849	-2392.099788	Control1
ACTTCCGAGCCAAGTG-1	singlet	2	-905.8873008	-831.8724284	-334.700139	Control1
ACTTCCGAGGATCACG-1	singlet	2	-3173.103056	-2859.055937	-1103.805887	Control1
ACTTCCGAGTGAATAC-1	singlet	1	-674.0112846	-275.1066008	-685.0399475	Control1
ACTTCCGCAAAGGGTC-1	singlet	1	-1039.720614	-422.6095371	-1094.440167	Control1
ACTTCCGCACCAACAT-1	singlet	1	-1009.721624	-438.5327503	-880.8394857	Control1
ACTTCCGCACGGCCAT-1	singlet	2	-5700.715031	-4871.977572	-1771.507503	Control1
ACTTCCGCAGAAGTTA-1	singlet	2	-2218.770083	-1803.396037	-779.6926965	Control1
ACTTCCGCATACAGGG-1	singlet	2	-2024.309973	-1795.397579	-724.7635251	Control1
ACTTCCGGTAGAGACC-1	singlet	1	-550.3043859	-270.9371326	-550.1928517	Control1
ACTTCCGGTGATCATC-1	singlet	2	-11671.54766	-9689.47297	-3070.786454	Control1
ACTTCCGGTTAGGAGC-1	singlet	2	-308.5584445	-271.4286013	-120.9930562	Control1
ACTTCCGGTTATTCTC-1	singlet	1	-1168.773234	-395.9106132	-958.9384673	Control1
ACTTCCGTCGACACCG-1	singlet	1	-1307.469439	-621.1731821	-1349.316058	Control1
ACTTCGCAGCAACCAG-1	singlet	2	-804.6013665	-598.2588076	-304.3817505	Control1
ACTTCGCCACGCTGAC-1	singlet	1	-1156.645416	-522.0500163	-1256.532728	Control1
ACTTCGCCAGAATTCC-1	singlet	2	-1013.09854	-1314.869414	-444.0840222	Control1
ACTTCGCGTAACGATA-1	unassigned	45293	-15.41894308	-5.743978779	-7.324933298	Control1
ACTTCGCGTGATTGGG-1	singlet	2	-875.0779068	-552.1380977	-253.7878901	Control1
ACTTCGCTCAAACTGC-1	singlet	1	-1302.352442	-545.5162228	-1233.250936	Control1
ACTTCGCTCCATTGCC-1	singlet	2	-2613.668315	-2407.607038	-1038.751951	Control1
ACTTCGCTCCGCCTAT-1	singlet	2	-2302.602929	-1907.918408	-753.3703308	Control1
ACTTCGCTCCTTGAAG-1	singlet	1	-678.8611207	-264.3498122	-656.1451661	Control1
ACTTCGCTCGAGTCCG-1	singlet	2	-402.2293886	-357.3020887	-166.8763928	Control1
ACTTTCAAGCAACTTC-1	singlet	2	-1470.71048	-1571.94669	-684.7588581	Control1
ACTTTCAAGGAAGTCC-1	singlet	2	-2287.025965	-1921.036527	-742.8841902	Control1
ACTTTCAAGGAGAGTA-1	singlet	1	-2004.312581	-792.7409863	-1928.424534	Control1
ACTTTCACAAATGAGT-1	singlet	2	-3002.893689	-2591.537451	-1105.913563	Control1
ACTTTCACACCTGTCT-1	singlet	2	-4227.12911	-3634.89838	-1501.690173	Control1
ACTTTCAGTAGCTTTG-1	singlet	2	-1100.212631	-912.5250339	-454.129429	Control1
ACTTTCAGTCAACCAT-1	singlet	2	-1298.723572	-1226.472924	-485.0835395	Control1
ACTTTCAGTGCAATGG-1	singlet	2	-974.5484519	-669.4910303	-256.6803103	Control1
ACTTTCATCAAGCTGT-1	singlet	2	-489.213375	-446.578749	-197.6998031	Control1
ACTTTCATCATTTACC-1	singlet	2	-4583.977741	-3887.815392	-1460.192243	Control1
ACTTTGTAGCAACAAT-1	singlet	2	-266.7949974	-261.8836314	-134.7629513	Control1
ACTTTGTAGCCTTGAT-1	singlet	2	-716.363927	-458.4121907	-237.7253496	Control1
ACTTTGTCAAACGTGG-1	singlet	2	-548.7416301	-454.4488588	-211.9492249	Control1
ACTTTGTCACGCAGTC-1	singlet	2	-3078.22387	-2556.930943	-1121.425753	Control1
ACTTTGTGTACCGGAA-1	singlet	2	-362.7953765	-302.1925056	-127.619406	Control1
ACTTTGTTCCATGATG-1	singlet	2	-703.2609319	-518.5307918	-232.6735423	Control1
ACTTTGTTCGATTGGT-1	singlet	2	-572.1126262	-437.7064443	-249.9327292	Control1
AGAAATGAGTGCTCAT-1	singlet	2	-1254.694926	-1284.539532	-474.3163024	Control1
AGAAATGAGTTGCATC-1	singlet	2	-5527.692133	-4619.296297	-1698.667442	Control1
AGAAATGCAGAGATTA-1	singlet	2	-956.5843157	-693.2336855	-327.5671023	Control1
AGAAATGCAGCAGACA-1	singlet	1	-2011.218616	-750.8297218	-1891.960333	Control1
AGAAATGCATTACGGT-1	singlet	1	-888.4805131	-403.3758478	-872.8561797	Control1
AGAAATGGTCGAATTC-1	singlet	1	-1305.560125	-510.6163972	-1011.351806	Control1
AGAAATGTCACATCAG-1	unassigned	1	-3.003983324	-1.261987203	-2.320762369	Control1
AGAAATGTCACGGGAA-1	singlet	2	-1774.268861	-1575.349006	-687.3975464	Control1
AGAAATGTCCATGCAA-1	singlet	2	-711.9596298	-574.0731431	-253.2827596	Control1
AGAAATGTCGCAGTTA-1	singlet	1	-6441.477886	-2340.465849	-6180.034928	Control1
AGAAATGTCGCTTAAG-1	singlet	1	-1161.525418	-437.9810828	-1159.613681	Control1
AGAACAAAGTACTGGG-1	singlet	2	-765.8962025	-521.2264091	-273.4828651	Control1
AGAACAACAAAGACTA-1	singlet	2	-6772.750529	-5723.770632	-2112.829299	Control1
AGAACAAGTCCTACAA-1	singlet	2	-683.5719633	-575.2818566	-303.8038486	Control1
AGAACAAGTCGCTTGG-1	singlet	2	-207.0589781	-205.9554077	-107.1149321	Control1
AGAACAAGTGACCGTC-1	singlet	2	-68.54612201	-45.14403339	-27.93591707	Control1
AGAACAAGTTTAAGGA-1	singlet	2	-1249.575512	-1394.714759	-521.5479813	Control1
AGAACAATCATTCCTA-1	singlet	2	-342.6358764	-249.300531	-132.4847623	Control1
AGAACAATCTGCTTAT-1	doublet	45323	-447.7191219	-306.3766924	-196.2186221	Control1
AGAACAATCTTCTCAA-1	singlet	2	-1109.741328	-743.071988	-304.1843687	Control1
AGAACCTAGAAATTCG-1	singlet	2	-1137.461166	-1005.270857	-355.4091982	Control1
AGAACCTAGAGTTGAT-1	singlet	2	-2576.553544	-2784.67422	-953.8500893	Control1
AGAACCTAGGTGCTAG-1	singlet	1	-1120.954403	-463.163592	-1166.133122	Control1
AGAACCTAGTATAACG-1	singlet	2	-560.742538	-408.4929765	-211.7301068	Control1
AGAACCTCACCTGAAT-1	singlet	0	-624.8213921	-1702.583307	-2051.880317	Control1
AGAACCTCAGCACCCA-1	singlet	2	-1065.339312	-814.3283334	-393.4135906	Control1
AGAACCTCATTCAGGT-1	singlet	1	-2124.871149	-801.5308612	-2043.889683	Control1
AGAACCTGTAAGAACT-1	singlet	2	-717.6788729	-592.2271793	-255.222713	Control1
AGAACCTGTAGCTGTT-1	singlet	2	-868.9354661	-616.7833702	-275.3188971	Control1
AGAACCTGTATAGGGC-1	singlet	2	-812.9783086	-630.2940641	-273.8751015	Control1
AGAACCTTCAGTCTTT-1	singlet	2	-746.7996587	-536.1400954	-239.583857	Control1
AGAAGCGAGCTCTTCC-1	singlet	2	-2858.320448	-2839.145783	-1048.755646	Control1
AGAAGCGAGGAGACCT-1	singlet	2	-1246.428779	-1115.814572	-452.1304621	Control1
AGAAGCGAGTCTGTAC-1	singlet	2	-2317.073798	-2238.684755	-769.4377655	Control1
AGAAGCGCATGATCTG-1	singlet	2	-724.246657	-651.0636901	-275.6955025	Control1
AGAAGCGGTAACTAAG-1	singlet	2	-520.0217508	-454.3267045	-218.0569355	Control1
AGAAGCGGTCACCGAC-1	singlet	1	-802.4391943	-355.810663	-824.5380213	Control1
AGAAGCGGTCCAGCCA-1	singlet	2	-1415.715106	-1253.651976	-619.7709569	Control1
AGAAGCGGTGGTAATA-1	singlet	2	-361.1863081	-271.2878498	-123.6365272	Control1
AGAAGTAAGATGATTG-1	singlet	2	-1774.620712	-1721.283823	-741.8320222	Control1
AGAAGTAAGCAATAGT-1	singlet	0	-323.4354766	-772.3208587	-848.1390461	Control1
AGAAGTAAGGCGTTAG-1	singlet	2	-2788.524954	-2259.8328	-931.4465249	Control1
AGAAGTAAGTCGGGAT-1	singlet	2	-7615.308663	-6600.999828	-2475.644435	Control1
AGAAGTAAGTGATGGC-1	singlet	2	-2343.390749	-2012.561065	-838.7935295	Control1
AGAAGTACAATTGTGC-1	singlet	2	-2025.38682	-1686.179662	-666.5202875	Control1
AGAAGTACAGTATTCG-1	singlet	1	-640.0947305	-246.2301131	-597.3514424	Control1
AGAAGTACATAAGCGG-1	singlet	2	-733.9980256	-596.4747958	-223.5350113	Control1
AGAAGTAGTAACTTCG-1	singlet	2	-265.7686595	-212.1587839	-87.2250723	Control1
AGAAGTAGTCGTGGTC-1	singlet	2	-954.299889	-1125.575269	-386.5969086	Control1
AGAAGTAGTCTTAGTG-1	singlet	1	-2131.713913	-788.2086353	-2032.725086	Control1
AGAAGTAGTGATTCTG-1	singlet	2	-30.29117441	-33.64002539	-10.94030331	Control1
AGAAGTATCCAGCACG-1	singlet	2	-358.4194799	-374.7412634	-169.518603	Control1
AGAAGTATCGGCCAAC-1	singlet	2	-1412.14363	-1198.298536	-466.3012947	Control1
AGACAAAAGCAACCAG-1	singlet	1	-826.4652128	-319.9593811	-665.859578	Control1
AGACAAAAGCTGTACT-1	singlet	2	-10.44909182	-7.168296982	-2.305718928	Control1

AGACAAAAGTCGCCAC-1	singlet	2	-316.937995	-316.1906809	-148.2515013	Control1
AGACAAAAGTCTCTGA-1	singlet	2	-674.0030621	-440.7341967	-200.0083926	Control1
AGACAAAAGTTTCAGC-1	singlet	2	-909.0681429	-663.2976722	-315.9584286	Control1
AGACAAACAAATAAGC-1	singlet	1	-1449.596608	-593.4155027	-1401.948423	Control1
AGACAAACAACGCATT-1	singlet	2	-1100.352997	-1279.434972	-451.3931815	Control1
AGACAAACACGAGGTA-1	singlet	1	-2295.383343	-853.6568922	-2014.041771	Control1
AGACAAACATTGGCAT-1	singlet	2	-1232.228637	-1417.997759	-517.4918331	Control1
AGACAAATCATCTCTA-1	singlet	2	-933.8355566	-920.8238749	-341.0159701	Control1
AGACAAATCGCTGTTC-1	singlet	1	-1263.238902	-505.1968121	-1205.53497	Control1
AGACAAATCGGCTGGT-1	singlet	2	-522.6592418	-398.7646557	-183.4771316	Control1
AGACACTAGAATCCCT-1	singlet	2	-9427.436216	-7943.73335	-2756.325915	Control1
AGACACTAGCAATAGT-1	unassigned	2	-5.793041911	-5.099470717	-2.093817026	Control1
AGACACTAGTCCTGTA-1	singlet	2	-643.7744099	-454.0775423	-154.8711576	Control1
AGACACTAGTTACGAA-1	singlet	2	-1619.996264	-1425.221646	-526.9433472	Control1
AGACACTCAAGAGATT-1	singlet	2	-1769.029155	-1513.021587	-618.1873071	Control1
AGACACTCAGGTTTAC-1	singlet	1	-715.5726434	-334.0789158	-714.0457999	Control1
AGACACTCATAATGCC-1	singlet	1	-1306.339942	-591.7922644	-1224.308886	Control1
AGACACTCATCCGAGC-1	singlet	0	-869.5340045	-2394.841859	-2498.447487	Control1
AGACACTCATCTGTTT-1	singlet	2	-1830.325926	-1781.819452	-647.988173	Control1
AGACACTGTCCTACAA-1	singlet	2	-1331.652117	-1371.254762	-561.3685817	Control1
AGACACTGTTTGTTGG-1	singlet	2	-1647.240088	-1372.611099	-593.9947774	Control1
AGACACTTCAACACCA-1	singlet	1	-9271.108251	-2633.871611	-8368.045867	Control1
AGACACTTCAGGTAAA-1	singlet	0	-150.8603234	-352.8640326	-554.3247128	Control1
AGACACTTCGTTCTAT-1	singlet	2	-1909.046249	-1648.375715	-692.4663693	Control1
AGACACTTCTCGGCTT-1	singlet	2	-1955.25214	-1549.505772	-674.2983089	Control1
AGACACTTCTCGTTTA-1	singlet	1	-939.5285489	-323.8836203	-841.3648569	Control1
AGACACTTCTTGCAGA-1	singlet	2	-865.4182312	-820.3022226	-375.511756	Control1
AGACAGGAGGAGGGTG-1	singlet	1	-824.3924059	-417.756993	-829.1438626	Control1
AGACAGGAGGCACTAG-1	singlet	2	-654.5325655	-579.1674731	-278.8719442	Control1
AGACAGGCACTGAGTT-1	singlet	1	-1755.356481	-678.3252891	-1658.225736	Control1
AGACAGGCACTGTGAT-1	singlet	2	-4634.691189	-4027.318203	-1482.508785	Control1
AGACAGGCATCTAGAC-1	singlet	1	-847.4551153	-344.6477404	-865.1344596	Control1
AGACAGGCATGGAATA-1	singlet	2	-1674.103159	-1623.463597	-570.0411569	Control1
AGACAGGCATGGCCCA-1	singlet	2	-544.1109818	-439.7264996	-237.6364733	Control1
AGACAGGGTTGGGAAC-1	singlet	2	-805.9341331	-803.2038355	-327.5550387	Control1
AGACAGGGTTTCGTTT-1	singlet	1	-799.4700453	-384.3614749	-888.0379918	Control1
AGACCATAGCACGTCC-1	singlet	2	-644.35509	-632.6481979	-239.4953404	Control1
AGACCATAGCTAATCC-1	singlet	0	-855.5582926	-2034.484658	-1990.002649	Control1
AGACCATAGGACAACC-1	singlet	1	-2824.69205	-905.7026651	-2772.369491	Control1
AGACCATAGGCACAAC-1	singlet	1	-1836.315535	-738.0327131	-1650.056807	Control1
AGACCATAGGTATTGA-1	singlet	2	-849.7792953	-654.30758	-286.7840769	Control1
AGACCATCATCCTCAC-1	singlet	1	-1648.088198	-649.433858	-1491.771835	Control1
AGACCATTCCCATTTA-1	singlet	1	-788.3807066	-290.1880035	-741.8320007	Control1
AGACCCGAGGCATTTC-1	singlet	1	-698.8226593	-281.3707987	-725.6656553	Control1
AGACCCGAGTACAGAT-1	singlet	1	-624.4408106	-262.4541571	-656.8302315	Control1
AGACCCGCAAAGAGTT-1	singlet	2	-537.2344088	-421.8167964	-216.8329327	Control1
AGACCCGCAAATCGGG-1	singlet	0	-1859.632581	-5488.441151	-6181.554136	Control1
AGACCCGCAGCAGTCC-1	singlet	2	-5063.508744	-4168.118547	-1710.253992	Control1
AGACCCGCAGCCGTCA-1	singlet	2	-585.1737673	-414.4551504	-159.4003118	Control1
AGACCCGCATAGCACT-1	singlet	1	-1250.039771	-412.6164407	-1056.756876	Control1
AGACCCGGTAGGGAGG-1	doublet	2/0	-67.5577675	-61.7081657	-32.57329088	Control1
AGACCCGGTCATGGCC-1	singlet	1	-1058.989262	-400.2888166	-1064.344706	Control1
AGACCCGGTCTCCTGT-1	singlet	2	-1171.084407	-980.2040943	-421.4213106	Control1
AGACCCGGTGAGTAGC-1	singlet	2	-1256.011345	-1205.339409	-514.165032	Control1
AGACCCGGTGATTAGA-1	singlet	1	-965.8519356	-407.5444965	-942.0449728	Control1
AGACCCGTCTCGCCTA-1	singlet	2	-511.1947543	-427.3122026	-206.2691775	Control1
AGACTCAAGAGGGTGG-1	singlet	2	-841.3821447	-872.3475211	-311.7480784	Control1
AGACTCAAGATAGTGT-1	singlet	1	-504.3169016	-126.4402561	-345.7588587	Control1
AGACTCAAGCCTGTCG-1	singlet	1	-3940.104672	-1395.742259	-3487.937089	Control1
AGACTCAAGGCATGCA-1	singlet	1	-1138.601053	-450.4503917	-978.778448	Control1
AGACTCACAAGATGTA-1	singlet	2	-423.5170455	-439.473934	-182.3968291	Control1
AGACTCACAATCGTCA-1	singlet	2	-2498.107539	-2252.386081	-808.2345435	Control1
AGACTCAGTACAAGTA-1	singlet	1	-8892.83002	-2705.996303	-8321.831791	Control1
AGACTCAGTCTCTCTG-1	singlet	2	-624.2855994	-408.1194401	-206.4557974	Control1
AGACTCAGTCTGTAAC-1	singlet	2	-8327.881635	-7064.805133	-2353.632596	Control1
AGACTCAGTGTCCTAA-1	singlet	2	-575.5310842	-474.302798	-215.1481959	Control1
AGACTCAGTTGAGAGC-1	singlet	2	-340.0642593	-263.2031774	-121.0909856	Control1
AGACTCATCATTACTC-1	singlet	2	-2247.14258	-2196.473453	-836.9593871	Control1
AGACTCATCGAGTGAG-1	singlet	2	-300.4559968	-300.1740991	-90.14642674	Control1
AGACTCATCTCGCCTA-1	singlet	1	-1013.679853	-372.8090427	-1062.59977	Control1
AGACTCATCTCGCTTG-1	singlet	2	-1244.891792	-1436.764945	-548.0399561	Control1
AGAGAATAGACTTCGT-1	singlet	0	-446.2141652	-1156.328301	-1177.123802	Control1
AGAGAATAGCCAACCC-1	singlet	1	-1073.527593	-448.17536	-1105.526434	Control1
AGAGAATAGGAGAGTA-1	singlet	2	-749.0748856	-538.7739829	-284.6414319	Control1
AGAGAATCATCGATAC-1	singlet	2	-592.6996053	-488.1192937	-249.9330346	Control1
AGAGAATTCATTTCCA-1	singlet	2	-3385.310263	-4965.058643	-1133.54469	Control1
AGAGAATTCATTTGTC-1	singlet	2	-825.7977687	-537.5883306	-251.0891649	Control1
AGAGAATTCCATCAGA-1	singlet	2	-541.9859395	-468.5259027	-217.3998547	Control1
AGAGAATTCCTGGTCT-1	singlet	0	-213.3484457	-558.9790533	-707.6868343	Control1
AGAGAATTCGACGCTG-1	singlet	1	-912.3336338	-374.1711091	-901.5541517	Control1
AGAGAATTCTGCCCTA-1	singlet	1	-1352.48626	-570.886157	-1253.225743	Control1
AGAGAGCAGATGCAGC-1	singlet	2	-2008.131796	-1856.671417	-761.1699894	Control1
AGAGAGCAGATTCGCT-1	singlet	2	-1281.918075	-1159.379249	-444.9124594	Control1
AGAGAGCAGCTGTACT-1	singlet	2	-645.3479023	-480.5782265	-222.8742525	Control1
AGAGAGCAGCTTCTAG-1	singlet	2	-788.9987301	-581.8112539	-269.2520948	Control1
AGAGAGCCAAGACGGT-1	singlet	1	-953.8623825	-358.4139564	-806.7353222	Control1
AGAGAGCCACACAGCC-1	singlet	2	-438.8888815	-343.2493572	-192.537725	Control1
AGAGAGCCAGACCAGA-1	singlet	1	-310.4330261	-130.1260879	-274.7242558	Control1
AGAGAGCGTAGGACTG-1	singlet	0	-598.9024039	-1562.131654	-1812.663427	Control1
AGAGAGCGTGTCGATT-1	singlet	0	-308.5018221	-718.4602227	-727.4995624	Control1
AGAGAGCTCACCGGTG-1	singlet	2	-2325.074551	-2339.109148	-838.8056513	Control1
AGAGCAGAGACAGCGT-1	singlet	1	-6873.886316	-2157.451594	-5927.288494	Control1
AGAGCAGAGTTCCATG-1	doublet	45323	-2384.693973	-1573.273865	-1491.595777	Control1
AGAGCAGCAAACGTGG-1	singlet	2	-1103.200833	-1098.926599	-408.7318024	Control1
AGAGCAGCACTCTGCT-1	doublet	45323	-2286.917213	-1641.818186	-1267.564894	Control1
AGAGCAGCATTGCCTC-1	singlet	1	-1285.74288	-546.222602	-1096.655804	Control1
AGAGCAGGTCACCGCA-1	singlet	2	-1577.038768	-1290.245588	-561.4365163	Control1
AGAGCAGGTCATGGCC-1	singlet	2	-588.9671998	-392.2176774	-187.6477635	Control1
AGAGCAGTCACTGTTT-1	singlet	2	-519.384592	-403.5960077	-208.5169678	Control1
AGAGCAGTCATAGCAC-1	singlet	2	-1343.910391	-1207.116277	-468.7669792	Control1
AGAGCAGTCGGCTTCT-1	singlet	2	-146.0408388	-147.9146648	-53.84758886	Control1
AGAGCAGTCGTAACCA-1	singlet	2	-713.5519921	-530.9944428	-296.8972466	Control1
AGAGCAGTCTGCGATA-1	singlet	2	-1169.214959	-1384.587144	-460.3500355	Control1
AGAGCAGTCTTCGTAT-1	singlet	2	-640.0475147	-538.2321601	-251.2901582	Control1
AGAGCCCAGCTTTGTG-1	singlet	2	-493.7425362	-358.9453536	-184.0704426	Control1
AGAGCCCAGTGGTGGT-1	singlet	1	-935.5386051	-387.1318925	-896.0605892	Control1
AGAGCCCCAGTCTGGC-1	singlet	2	-1674.939458	-1348.780453	-543.499837	Control1
AGAGCCCGTTCCAGGC-1	singlet	1	-184.5881516	-38.43875537	-113.4515644	Control1
AGAGCCCTCAGCGGAA-1	singlet	1	-1499.447173	-476.6076274	-1301.692707	Control1
AGAGCCCTCCCAGCGA-1	singlet	1	-1620.648257	-552.1767938	-1527.201858	Control1

AGAGCCCTCCTACACC-1	singlet	1	-3042.300151	-1167.234655	-2853.688735	Control1
AGAGCCCTCCTCTCGA-1	singlet	2	-1051.684543	-885.4708253	-413.5112921	Control1
AGATAGAAGCTAAGTA-1	singlet	2	-658.2215406	-452.380661	-183.2426567	Control1
AGATAGAAGGTCGACA-1	singlet	2	-634.1911719	-645.3197076	-247.8111493	Control1
AGATAGAAGTATTCCG-1	singlet	1	-1112.263147	-478.1791736	-1037.132454	Control1
AGATAGAAGTGTACAA-1	singlet	2	-1565.098875	-1559.013875	-532.6230818	Control1
AGATAGACAGAACTCT-1	singlet	2	-70.03124634	-62.02872322	-32.5140745	Control1
AGATAGACAGCCTATA-1	singlet	1	-2364.608647	-778.0379404	-1983.8174	Control1
AGATAGACAGTAGATA-1	singlet	2	-1438.220915	-1374.55732	-531.9018473	Control1
AGATAGAGTCACTCAA-1	singlet	2	-842.6358784	-652.6922961	-339.1931752	Control1
AGATAGAGTCCTGGTG-1	singlet	1	-151.3942504	-42.3091153	-117.7615118	Control1
AGATAGATCACTTTGT-1	singlet	1	-2571.09789	-869.3951671	-2209.569684	Control1
AGATAGATCCGTGTCT-1	singlet	2	-644.2327617	-553.7409245	-253.9839816	Control1
AGATAGATCGAATCCA-1	singlet	2	-1967.275477	-1780.505309	-727.3322974	Control1
AGATCCAAGAGTACCG-1	singlet	2	-1661.769976	-1506.808309	-616.0036146	Control1
AGATCCAAGTATAGGT-1	singlet	1	-1315.367532	-461.8133989	-1187.418052	Control1
AGATCCACAGCGTGAA-1	singlet	2	-1046.022089	-926.4746979	-379.5315083	Control1
AGATCCACAGGGTTGA-1	singlet	2	-1108.383384	-934.3757953	-422.0969273	Control1
AGATCCAGTATGTCTG-1	singlet	2	-5868.413221	-4911.58648	-1806.970009	Control1
AGATCCAGTATTGAGA-1	singlet	2	-1818.763042	-1640.371611	-676.6316548	Control1
AGATCCAGTCAGGTGA-1	singlet	1	-1917.715707	-700.4971543	-1631.403201	Control1
AGATCCAGTCGATTCA-1	singlet	2	-2470.576673	-2374.014192	-984.4933505	Control1
AGATCCATCGGTGAAG-1	singlet	1	-1308.93841	-538.7420976	-1325.660754	Control1
AGATCCATCTACGCAA-1	singlet	1	-176.3770816	-75.72874042	-152.9887195	Control1
AGATCGTAGGGTGAAA-1	unassigned	1/0	-15.88747325	-6.049797226	-9.569309781	Control1
AGATCGTAGGTACCTT-1	singlet	2	-3661.829641	-2974.075414	-1295.963439	Control1
AGATCGTAGTTAGTGA-1	singlet	1	-2171.634293	-738.423318	-1922.567499	Control1
AGATCGTAGTTGAATG-1	doublet	45293	-1501.570954	-861.1116999	-1085.357143	Control1
AGATCGTCAATAGTAG-1	singlet	2	-1242.881689	-1101.076407	-456.1450247	Control1
AGATCGTCACAATGAA-1	singlet	1	-3080.462203	-794.4413292	-3238.958877	Control1
AGATCGTGTATCCTTT-1	singlet	2	-13.80845438	-8.593393511	-4.986735337	Control1
AGATCGTGTCAGGTGA-1	singlet	2	-858.8682971	-844.9312046	-372.2223362	Control1
AGATCGTGTCCGACGT-1	singlet	2	-3330.849322	-5073.969925	-1133.63337	Control1
AGATCGTGTTGGCCTG-1	singlet	1	-271.0326745	-90.13043235	-245.4901228	Control1
AGATCGTTCGAATCCA-1	singlet	2	-1002.156639	-907.1729293	-418.54992	Control1
AGATCGTTCTCTCGAC-1	singlet	2	-289.504069	-279.9269857	-121.4093364	Control1
AGATCGTTCTTTGCGC-1	singlet	2	-229.0316002	-192.4988417	-99.5153443	Control1
AGATGAAAGTAACGTA-1	singlet	2	-1688.576108	-1507.051728	-628.6360244	Control1
AGATGAACAACACACT-1	singlet	2	-779.7931297	-613.559824	-240.9607135	Control1
AGATGAACACGCGTCA-1	unassigned	1	-5.606037522	-3.133722271	-12.42525495	Control1
AGATGAAGTAGCGTTT-1	singlet	1	-1652.115549	-714.8328082	-1608.148421	Control1
AGATGAAGTCTACATG-1	singlet	1	-599.2438018	-246.3523643	-611.1899564	Control1
AGATGAAGTCTGATCA-1	singlet	2	-2080.95965	-1497.557574	-605.8204311	Control1
AGATGCTCAACGTATC-1	singlet	1	-911.2709078	-353.2627169	-859.5282991	Control1
AGATGCTCACCTCTAC-1	singlet	2	-767.372929	-558.2810866	-260.2273362	Control1
AGATGCTCATCACCAA-1	singlet	2	-523.6058391	-404.0635149	-193.0960386	Control1
AGATGCTGTCCCTCAT-1	singlet	1	-3493.010233	-1297.992831	-3115.762591	Control1
AGATGCTGTTGCTCAA-1	unassigned	2	-5.968774506	-5.489405703	-5.248876541	Control1
AGATGCTGTTTAAGGA-1	singlet	2	-2098.452095	-2120.06948	-733.7581981	Control1
AGATGCTTCCCAGCGA-1	singlet	2	-1397.931205	-1348.329783	-529.3567845	Control1
AGCATCAAGCCGTTAT-1	singlet	2	-646.1532214	-541.7889319	-273.4238905	Control1
AGCATCAAGGGAGTGG-1	singlet	2	-658.3422738	-561.22264	-232.2590979	Control1
AGCATCAAGTAATACG-1	singlet	0	-4.477058989	-15.15746449	-10.44316181	Control1
AGCATCACAACCTAAC-1	singlet	2	-2167.023399	-1972.669554	-735.4976223	Control1
AGCATCACAGAACATA-1	singlet	2	-722.9741126	-487.2106871	-229.1770861	Control1
AGCATCATCACATTGG-1	singlet	2	-700.6434512	-487.7102007	-245.395319	Control1
AGCATCATCGACATTG-1	singlet	0	-1832.043581	-6043.209248	-6605.64133	Control1
AGCCAATAGCCTATTG-1	singlet	2	-1514.926148	-1331.312379	-650.3005398	Control1
AGCCAATCAACGACAG-1	singlet	2	-704.4970687	-557.8933216	-252.380843	Control1
AGCCAATCAAGAGTTA-1	singlet	2	-3596.526755	-2999.687553	-1154.318804	Control1
AGCCAATCATCCTAAG-1	singlet	1	-197.9087218	-50.81788642	-153.0939017	Control1
AGCCAATGTCGAGTGA-1	singlet	2	-8722.010117	-7074.240446	-2263.274645	Control1
AGCCAATGTTCTCGTC-1	singlet	2	-2821.564577	-3184.765111	-1157.871431	Control1
AGCCAATGTTGCCATA-1	singlet	0	-1884.667604	-5581.548437	-6034.731771	Control1
AGCCAATTCAGCGGAA-1	singlet	2	-830.8237465	-634.4120598	-268.7476769	Control1
AGCCAATTCGCATGAT-1	singlet	2	-687.0872071	-538.8064909	-269.4689056	Control1
AGCCAATTCGTCGATA-1	doublet	45293	-2789.688845	-1756.530642	-2082.10582	Control1
AGCCACGAGTAATTGG-1	singlet	2	-2040.801422	-1901.163236	-747.6477972	Control1
AGCCACGCACCTCGTT-1	singlet	0	-72.45080915	-435.391291	-623.7959433	Control1
AGCCACGCAGCTCGGT-1	singlet	0	-58.80025562	-195.8510141	-191.9348838	Control1
AGCCACGGTCGAAGCA-1	unassigned	1	-0.997509797	-0.947417651	-1.624579526	Control1
AGCCACGTCCCATTCG-1	singlet	1	-3220.294098	-1062.25657	-2905.738598	Control1
AGCCACGTCTAACACG-1	unassigned	1	-7.925695165	-6.067101273	-9.407142697	Control1
AGCCACGTCTCTATGT-1	singlet	2	-745.4274181	-557.1963143	-271.3038291	Control1
AGCCAGCAGCGACAGT-1	singlet	1	-3047.031943	-1015.757792	-2611.432895	Control1
AGCCAGCCATCCTATT-1	singlet	2	-1860.808232	-1555.545393	-624.1913534	Control1
AGCCAGCGTACCTTCC-1	singlet	2	-1984.0571	-1878.011049	-778.5966592	Control1
AGCCAGCGTCACTGAT-1	singlet	2	-1195.014332	-1468.692545	-450.7560524	Control1
AGCCAGCGTTAGAAGT-1	singlet	2	-1330.716694	-1163.266778	-480.0441842	Control1
AGCCAGCTCATGCCCT-1	singlet	2	-2566.190496	-2117.985483	-849.1948339	Control1
AGCCAGCTCATTACCT-1	singlet	1	-238.1373393	-90.10776258	-197.8028065	Control1
AGCCAGCTCCAGTGCG-1	singlet	1	-1301.65266	-704.5480718	-1122.664279	Control1
AGCCAGCTCGGTATGT-1	singlet	2	-928.7279921	-1126.213344	-363.8775518	Control1
AGCCAGCTCGTTAGAC-1	singlet	2	-329.158621	-279.3933873	-119.6394859	Control1
AGCGATTAGCGCACAA-1	singlet	2	-1042.960423	-1096.02056	-470.0975937	Control1
AGCGATTCAGTAGAGC-1	singlet	2	-808.6683158	-656.5364833	-275.5090881	Control1
AGCGATTGTCTTGGTA-1	singlet	2	-691.6967663	-569.1846243	-244.0401914	Control1
AGCGATTTCAGACTGT-1	singlet	2	-434.4016979	-408.5041622	-180.3886506	Control1
AGCGATTTCAGGAAGC-1	singlet	2	-3874.383409	-3168.595786	-1178.541723	Control1
AGCGATTTCCAACTGA-1	singlet	0	-40.17239796	-128.8563553	-129.7553418	Control1
AGCGATTTCGCCTCTA-1	singlet	2	-2208.574731	-1675.505563	-735.0402525	Control1
AGCGATTTCTTAAGGC-1	singlet	2	-3030.8694	-2546.697841	-921.337118	Control1
AGCGCCAAGAAAGTCT-1	singlet	1	-1409.273633	-587.4694748	-1305.352581	Control1
AGCGCCAAGAAGTCTA-1	singlet	1	-119.313438	-28.95087601	-95.70574468	Control1
AGCGCCACAATGTCTG-1	singlet	1	-147.068529	-55.17199184	-102.5904464	Control1
AGCGCCACAGTAGTGG-1	singlet	2	-496.0468269	-391.2643911	-200.5430183	Control1
AGCGCCAGTCTCCTGT-1	singlet	2	-658.7401378	-480.5336076	-244.1501893	Control1
AGCGCCAGTGCCTTTC-1	singlet	2	-384.3941345	-351.7004777	-151.9063537	Control1
AGCGCCATCGTAACCA-1	singlet	2	-6449.865507	-5447.095051	-1984.317993	Control1
AGCGCTGAGAGGGTCT-1	singlet	2	-480.8459827	-443.5405956	-175.5977718	Control1
AGCGCTGAGCTCATAC-1	singlet	2	-1464.646782	-1336.694844	-632.2941399	Control1
AGCGCTGAGTGGATAT-1	singlet	2	-583.6126065	-451.419826	-237.3688809	Control1
AGCGCTGCAAAGTGTA-1	singlet	2	-2283.441654	-1854.98926	-786.6478736	Control1
AGCGCTGCACCCTATC-1	singlet	2	-963.1012446	-867.4768452	-397.4355282	Control1
AGCGCTGGTGACCGAA-1	singlet	1	-4282.371834	-1472.405428	-4324.195167	Control1
AGCGCTGTCCGATCTC-1	singlet	1	-1062.082212	-443.3381896	-999.8162568	Control1
AGCGTATAGCATCGAG-1	singlet	2	-438.4532443	-341.0028228	-162.2386967	Control1
AGCGTATAGCCAAGTG-1	singlet	0	-137.6014086	-377.4850079	-414.6266965	Control1
AGCGTATAGCGCCGTT-1	singlet	1	-2736.865575	-775.5316833	-3046.680008	Control1
AGCGTATAGCGGGTAT-1	singlet	2	-2004.114568	-1785.285445	-758.4916865	Control1

AGCGTATCAATCTCTT-1	singlet	1	-6213.835514	-2313.386444	-5748.936249	Control1
AGCGTATCAGGCGTTC-1	singlet	1	-934.349487	-377.6859452	-959.7929416	Control1
AGCGTATCAGGGCTTC-1	singlet	2	-634.0645249	-666.0471197	-240.6687269	Control1
AGCGTATGTCGGCACT-1	singlet	2	-1542.607335	-1270.483876	-542.242726	Control1
AGCGTATTCCCGAAAT-1	singlet	2	-2746.129633	-3564.9581	-971.4450346	Control1
AGCGTCGAGACCACGA-1	singlet	1	-60.75443265	-9.433861623	-12.61031266	Control1
AGCGTCGGTCACGACC-1	singlet	2	-304.6846015	-246.3558427	-100.4779046	Control1
AGCGTCGGTTCACCGG-1	singlet	2	-504.1678538	-368.9964883	-173.8861215	Control1
AGCTACAAGATGATTG-1	singlet	0	-195.7006199	-710.4993995	-702.9744638	Control1
AGCTACAAGCAACCAG-1	singlet	2	-775.4596201	-522.0622102	-276.3532961	Control1
AGCTACAAGGGCAGGA-1	singlet	1	-557.8919433	-178.5185702	-617.1057465	Control1
AGCTACAAGTACCGGA-1	singlet	1	-1167.096344	-441.4228862	-1114.310608	Control1
AGCTACACAAATGCGG-1	singlet	2	-2601.555957	-2914.197229	-1083.091004	Control1
AGCTACACAATGACCT-1	singlet	1	-3299.457934	-1284.219973	-3056.141861	Control1
AGCTACACAGTGCCTG-1	singlet	2	-1039.135499	-921.8430954	-460.6689341	Control1
AGCTACACATCCGCGA-1	singlet	1	-821.615299	-305.2599695	-699.058854	Control1
AGCTACACATGGGCAA-1	singlet	2	-1862.098815	-1644.150705	-751.5608336	Control1
AGCTACAGTCACAGTT-1	singlet	1	-58.02095897	-8.10166112	-35.1602315	Control1
AGCTACATCATTCACT-1	singlet	0	-1231.125401	-3340.198276	-3591.938472	Control1
AGCTACATCTAGCATG-1	singlet	1	-2097.906356	-775.0061635	-1672.981306	Control1
AGCTACATCTCTGACC-1	singlet	1	-3208.310278	-1081.515671	-2844.624253	Control1
AGCTACATCTGTACAG-1	singlet	2	-2245.373977	-2009.668667	-972.4709245	Control1
AGCTCAACACAATGAA-1	singlet	2	-1371.817358	-1160.32889	-537.0285766	Control1
AGCTCAACATCACCAA-1	singlet	2	-643.5638306	-553.9953319	-247.8431591	Control1
AGCTCAACATCCCACT-1	singlet	2	-809.9773407	-627.1773587	-301.7563335	Control1
AGCTCAAGTTTCTATC-1	singlet	2	-800.5529847	-695.4435354	-315.0990471	Control1
AGCTCAATCAAGAGGC-1	singlet	1	-929.3746011	-320.1094815	-878.4364617	Control1
AGCTCAATCACCATAG-1	singlet	2	-3237.146887	-2823.572216	-1112.216914	Control1
AGCTCAATCTGCGATA-1	singlet	1	-6536.756975	-2357.989746	-6119.128172	Control1
AGCTCAATCTTCTGTA-1	singlet	2	-4348.346568	-3741.067117	-1517.048669	Control1
AGCTTCCAGCATCTTG-1	singlet	2	-495.4591207	-383.2835642	-182.8177835	Control1
AGCTTCCAGGTCTGGA-1	singlet	1	-371.6455124	-150.6758308	-315.319196	Control1
AGCTTCCAGTGAGGTC-1	singlet	1	-1647.511904	-601.5165454	-1462.840152	Control1
AGCTTCCCAGCAGTTT-1	unassigned	45323	-14.72833745	-9.650427575	-6.417321349	Control1
AGCTTCCGTAACTTCG-1	singlet	2	-3062.720599	-5667.10089	-1049.732432	Control1
AGCTTCCGTCACTTCC-1	singlet	1	-1293.221834	-504.5038166	-1258.89528	Control1
AGCTTCCTCCTCACTG-1	doublet	45323	-8926.570594	-5435.718944	-4861.47114	Control1
AGCTTCCTCGGTAGAG-1	singlet	1	-851.825762	-392.8003176	-836.1342164	Control1
AGCTTCCTCGTAGCTA-1	singlet	1	-1153.456581	-511.8246145	-1096.459199	Control1
AGCTTCCTCTGCTTAT-1	singlet	1	-1188.524938	-537.605992	-1189.904397	Control1
AGGAAATAGGTAAACT-1	singlet	2	-3642.577328	-3281.753078	-1165.150012	Control1
AGGAAATAGTGGGAAA-1	singlet	2	-77.92094166	-59.88283291	-24.12596492	Control1
AGGAAATCAAGCGAAC-1	singlet	2	-1129.230522	-1027.027099	-459.8820081	Control1
AGGAAATTCAAGTGGG-1	singlet	2	-1438.290613	-1125.400528	-461.6364009	Control1
AGGAAATTCCTTATAC-1	singlet	2	-3195.970378	-2642.99999	-974.9483876	Control1
AGGAATACAATAAGGT-1	singlet	1	-1593.242704	-567.7867323	-1541.782388	Control1
AGGAATACATCTGTTT-1	singlet	2	-1198.043189	-1141.179649	-520.3333926	Control1
AGGAATACATTCGATG-1	singlet	2	-1913.636535	-1635.522147	-656.8607554	Control1
AGGAATAGTTAGTCGT-1	singlet	1	-1829.885739	-765.4588521	-1809.436953	Control1
AGGAATATCAGTCATG-1	singlet	2	-1215.543528	-1050.950213	-400.3763374	Control1
AGGAATATCCACGAAT-1	singlet	2	-1448.65119	-1227.773687	-528.8859947	Control1
AGGAATATCCACTTCG-1	singlet	0	-3434.828301	-11366.50386	-12428.45876	Control1
AGGAATATCTGAGGTT-1	singlet	2	-870.327235	-1154.659255	-383.9940554	Control1
AGGACGAAGCATGTTC-1	singlet	1	-1105.267962	-444.6723381	-1109.591225	Control1
AGGACGACAGCCCAGT-1	singlet	2	-1818.607118	-1538.784981	-625.0771894	Control1
AGGACGACAGCGTGAA-1	singlet	1	-1605.352366	-607.195143	-1425.27817	Control1
AGGACGAGTAAGCGGT-1	unassigned	1	-2.253154109	-0.865984655	-1.751421211	Control1
AGGACGAGTATTCCTT-1	singlet	0	-251.9930932	-565.3043675	-623.3294441	Control1
AGGACGATCGACTCCT-1	singlet	0	-37.47786228	-74.29712354	-83.77771752	Control1
AGGACTTAGACCATGG-1	singlet	1	-1378.215848	-582.0672494	-1311.116505	Control1
AGGACTTAGCACTTTG-1	singlet	2	-1397.479952	-1315.21044	-602.0625899	Control1
AGGACTTAGCCTCCAG-1	singlet	0	-35.16647764	-77.74588023	-66.25868962	Control1
AGGACTTCAACGGTAG-1	singlet	2	-2180.49666	-1871.663407	-773.4971956	Control1
AGGACTTCAAGCCATT-1	singlet	2	-2201.07212	-1759.260181	-742.9902493	Control1
AGGACTTCAATTTCTC-1	singlet	0	-2051.207039	-5906.481747	-6712.462335	Control1
AGGACTTCACCGGAAA-1	singlet	2	-1495.351306	-1618.867045	-598.0584732	Control1
AGGACTTCAGAAGTGC-1	singlet	2	-330.378219	-296.023986	-142.0076543	Control1
AGGACTTCAGGACATG-1	singlet	1	-2634.471876	-903.286276	-2387.11589	Control1
AGGACTTGTGACACGA-1	singlet	1	-2663.861182	-916.8995536	-2128.136019	Control1
AGGACTTGTTCGGGTC-1	singlet	2	-433.9763426	-299.8394323	-166.3567808	Control1
AGGACTTTCAAAGGAT-1	singlet	2	-466.1826281	-394.7888159	-159.0673531	Control1
AGGACTTTCAAGAGGC-1	singlet	2	-91.29553809	-78.89431804	-37.10655555	Control1
AGGACTTTCATGTCTT-1	singlet	0	-252.5572305	-643.4361308	-739.7378212	Control1
AGGACTTTCCCGGTAG-1	singlet	2	-4072.595546	-3107.143703	-1290.727737	Control1
AGGACTTTCGTGTGAT-1	singlet	2	-1867.007942	-1605.313399	-517.3898055	Control1
AGGAGGTAGAGCCCAA-1	singlet	1	-1153.427727	-521.6457038	-1127.44201	Control1
AGGAGGTAGCACAAAT-1	unassigned	2	-11.67580253	-7.666793875	-6.421508705	Control1
AGGAGGTGTAACCCGC-1	singlet	2	-1117.711341	-1370.36888	-484.4149517	Control1
AGGAGGTGTCAGGAGT-1	singlet	1	-920.4462851	-372.4684137	-839.356943	Control1
AGGAGGTGTGCAATAA-1	singlet	1	-1459.769736	-576.0881393	-1368.255167	Control1
AGGAGGTTCTCCAAGA-1	singlet	1	-1179.738777	-500.8002994	-1102.32241	Control1
AGGATAAAGATACTGA-1	singlet	0	-978.3338118	-5017.645697	-3140.508591	Control1
AGGATAAAGGAAGTAG-1	singlet	1	-981.1813801	-354.2261036	-954.4527423	Control1
AGGATAAAGTTGGACG-1	singlet	1	-2235.842924	-835.8389991	-2002.001771	Control1
AGGATAACAGCCCAGT-1	singlet	2	-811.6520842	-691.9994872	-274.6562945	Control1
AGGATAACAGGAATAT-1	singlet	1	-2321.333003	-712.9881798	-2045.467267	Control1
AGGATAAGTATGAAGT-1	singlet	2	-1151.678023	-1004.478214	-458.9856248	Control1
AGGATAAGTGTGTGGA-1	singlet	2	-2968.432706	-2625.308131	-1050.955921	Control1
AGGATAAGTGTTGAGG-1	singlet	2	-1869.224735	-1665.930442	-695.2234728	Control1
AGGATAATCGGAGCAA-1	singlet	2	-326.5554671	-257.5780396	-118.7078827	Control1
AGGATAATCGTCGACG-1	singlet	2	-462.8188339	-380.9276315	-176.1901398	Control1
AGGATCTAGAGCCGAT-1	singlet	2	-235.9299483	-241.891692	-102.317628	Control1
AGGATCTAGGTGCCTC-1	singlet	0	-3506.131228	-10662.72564	-11456.62999	Control1
AGGATCTCAAGGTACG-1	singlet	2	-2868.398015	-2950.159122	-930.878048	Control1
AGGATCTGTAGCACAG-1	doublet	45323	-4969.693012	-3901.299787	-2320.10841	Control1
AGGATCTGTGACGCCT-1	singlet	2	-894.2258706	-724.7029162	-271.5237871	Control1
AGGATCTGTGCCTTTC-1	singlet	2	-1917.048762	-1733.136515	-786.9052124	Control1
AGGATCTTCGCTCATC-1	singlet	1	-1182.436984	-496.4674013	-1142.855628	Control1
AGGATCTTCTGCCCTA-1	singlet	1	-1104.051501	-465.7324276	-1153.92429	Control1
AGGCATTAGGAGAGGC-1	singlet	1	-1024.476675	-452.6690313	-1107.175014	Control1
AGGCATTAGGTTTGAA-1	singlet	2	-1475.957541	-1194.052027	-506.3779065	Control1
AGGCATTAGTCCCGAC-1	singlet	1	-848.7418487	-329.1909127	-829.057645	Control1
AGGCATTCAAATACGA-1	singlet	1	-3580.50721	-1131.387474	-3253.151267	Control1
AGGCATTCAACTCGAT-1	singlet	2	-552.2594068	-494.7952275	-245.3284664	Control1
AGGCATTCAGACCAGA-1	singlet	2	-1357.160305	-1230.931243	-516.7528601	Control1
AGGCATTCAGCAAGAC-1	singlet	2	-130.5264903	-108.4624443	-51.12737624	Control1
AGGCATTTCCCTCTTT-1	singlet	0	-2279.955315	-6997.736966	-7689.562138	Control1
AGGCCACAGGTCATTC-1	singlet	2	-2234.244644	-1810.886573	-668.9300865	Control1
AGGCCACAGGTTGCCC-1	singlet	0	-1163.734284	-3586.926368	-4017.319508	Control1
AGGCCACCACTTGTCC-1	singlet	2	-831.088623	-604.1430895	-252.953905	Control1

AGGCCACGTTCGGGTC-1	singlet	1	-679.3938086	-275.3181381	-667.8989688	Control1
AGGCCACTCTACGGGC-1	singlet	1	-3427.015851	-1355.149311	-3269.684623	Control1
AGGCTGCAGCTAATCC-1	singlet	1	-1101.768076	-460.7574722	-1129.403844	Control1
AGGCTGCAGCTGAAGC-1	singlet	2	-2080.274656	-1750.867992	-813.9706982	Control1
AGGCTGCAGTCACGAG-1	singlet	0	-95.91517114	-273.2044887	-288.1649365	Control1
AGGCTGCCAATTGTGC-1	singlet	2	-1075.727885	-1255.409529	-449.5516717	Control1
AGGCTGCCATCATGAC-1	singlet	1	-45.90801595	-27.72736545	-70.09914052	Control1
AGGCTGCGTAGAGTTA-1	singlet	2	-431.6655166	-416.4681889	-169.7690759	Control1
AGGCTGCGTTCGGCCA-1	singlet	2	-563.0118202	-515.5286308	-239.9525492	Control1
AGGCTGCTCAACACGT-1	singlet	1	-776.5154388	-286.1847846	-631.2737358	Control1
AGGCTGCTCGATTGGT-1	singlet	1	-265.2928091	-31.33086442	-188.8227857	Control1
AGGGAGTAGCGAAACC-1	singlet	2	-3851.431413	-3952.548918	-1125.939934	Control1
AGGGAGTAGTCATGGG-1	singlet	2	-785.3929125	-582.335428	-297.7303254	Control1
AGGGAGTAGTTGTCAC-1	singlet	2	-568.8723836	-465.3745359	-173.5961435	Control1
AGGGAGTCAAGAGCTG-1	singlet	2	-774.3391235	-471.6291302	-254.5067979	Control1
AGGGAGTCAAGCAGGT-1	singlet	0	-1375.382607	-4174.512441	-4570.360165	Control1
AGGGAGTGTAACAAGT-1	singlet	1	-2385.448889	-847.2132002	-2244.887106	Control1
AGGGAGTGTCAAAGTA-1	unassigned	2/0	-185.9794866	-197.9034745	-74.39566515	Control1
AGGGAGTGTCAACATC-1	singlet	2	-1705.321752	-2309.761716	-626.1348519	Control1
AGGGAGTGTTGTTTGG-1	unassigned	2	-4.657804204	-8.801876713	-2.96895055	Control1
AGGGAGTTCGCTTTAT-1	singlet	1	-982.4888149	-413.0773486	-960.8863497	Control1
AGGGCCTAGCGTATAA-1	singlet	2	-614.2645784	-472.5324401	-241.7063351	Control1
AGGGCCTCACAGCTTA-1	singlet	2	-911.8733006	-814.2423368	-355.1221736	Control1
AGGGCCTCAGCTCTGG-1	singlet	2	-391.6042896	-361.8731151	-189.4040022	Control1
AGGGCCTGTACAACGG-1	singlet	2	-496.1232224	-425.9390234	-205.3934987	Control1
AGGGCCTGTAGCTTTG-1	singlet	1	-1642.32265	-598.2628999	-1533.274119	Control1
AGGGCCTGTCCAGGTC-1	singlet	2	-477.5360757	-328.8029825	-137.9335984	Control1
AGGGCCTGTCTCACAA-1	singlet	2	-1289.492546	-1179.318774	-526.255991	Control1
AGGGCCTGTTGCTTGA-1	singlet	2	-1682.166396	-1504.276985	-682.3215634	Control1
AGGGCTCAGATGAAGG-1	singlet	2	-245.013984	-215.5805986	-84.42158985	Control1
AGGGCTCAGCGACATG-1	singlet	1	-8535.573569	-2672.257385	-7248.404814	Control1
AGGGCTCAGGCTCAAG-1	singlet	2	-2122.888501	-1985.990894	-692.7386607	Control1
AGGGCTCAGTATGACA-1	singlet	2	-1062.945339	-862.7585845	-428.9942605	Control1
AGGGCTCCAAATGGTA-1	singlet	0	-179.0988048	-414.1770192	-474.9489702	Control1
AGGGCTCCAACGATCT-1	singlet	2	-503.6771794	-575.9137192	-230.3763952	Control1
AGGGCTCCAGAGTAAT-1	singlet	2	-2354.339366	-2025.778437	-863.2579877	Control1
AGGGCTCGTCGATGCC-1	singlet	2	-1948.233497	-1831.020607	-625.8236623	Control1
AGGGCTCGTTCGTAAC-1	singlet	2	-437.6223021	-345.6216199	-188.3814899	Control1
AGGGCTCGTTGGTGTT-1	singlet	1	-5196.085353	-1488.35793	-4807.394635	Control1
AGGGCTCTCCTCACGT-1	singlet	2	-1767.937568	-1536.522165	-622.3135788	Control1
AGGGTCCAGAGGCTGT-1	singlet	2	-1452.591832	-1271.930994	-537.9001336	Control1
AGGGTCCAGCTGACCC-1	singlet	2	-929.0895541	-725.6942908	-316.4751053	Control1
AGGGTCCAGGGTGGGA-1	singlet	2	-1160.850422	-1019.369926	-498.0145317	Control1
AGGGTCCAGTTCAACC-1	singlet	2	-626.7813018	-577.1684594	-230.8694757	Control1
AGGGTCCCAGCTGCCA-1	singlet	1	-1935.072925	-674.9311376	-1938.314908	Control1
AGGGTCCGTGCATCTA-1	singlet	2	-1076.098784	-956.1935068	-389.1699597	Control1
AGGGTCCTCAATCCGA-1	singlet	2	-542.7024297	-401.8488195	-187.4531763	Control1
AGGGTCCTCAGCCCAG-1	singlet	2	-1756.865576	-1493.054306	-594.3641319	Control1
AGGGTCCTCAGTAGGG-1	singlet	2	-8104.828111	-6880.597118	-2170.927197	Control1
AGGGTGAAGAGTCGAC-1	singlet	2	-610.3131222	-481.2707524	-233.3230882	Control1
AGGGTGAAGCATTTGC-1	singlet	1	-1192.130668	-488.1878018	-1169.479762	Control1
AGGGTGAAGCGGGTAT-1	singlet	2	-647.66634	-489.5356052	-198.8275514	Control1
AGGGTGAAGTTGCGCC-1	singlet	2	-6040.409433	-4953.470323	-1706.85663	Control1
AGGGTGACAAGTTCCA-1	singlet	2	-4830.325008	-3559.513767	-1446.999169	Control1
AGGGTGACACCTATCC-1	singlet	2	-871.316611	-663.761344	-285.7728777	Control1
AGGGTGACATGTTACG-1	singlet	1	-2168.144024	-771.6521132	-2257.133788	Control1
AGGGTGAGTAAGGAGA-1	singlet	2	-1493.230032	-1483.19682	-559.0071634	Control1
AGGGTGAGTAGGCAAC-1	singlet	2	-106.0334737	-99.31259034	-36.87052257	Control1
AGGGTGAGTGAATTGA-1	singlet	2	-3279.208756	-2629.140117	-1103.911484	Control1
AGGGTGAGTTGTGCCG-1	singlet	1	-82.61219743	-48.42687487	-102.3902645	Control1
AGGGTGATCCGCGGAT-1	singlet	2	-2469.258031	-2226.832016	-891.0874551	Control1
AGGGTTTAGATACGAT-1	singlet	1	-2383.779669	-832.5549815	-2199.082555	Control1
AGGGTTTCACTGCATA-1	singlet	2	-704.815611	-490.2160726	-264.6817142	Control1
AGGGTTTGTGAGTCAG-1	singlet	2	-4088.891539	-3250.982488	-1362.727028	Control1
AGGGTTTGTTAGGAGC-1	singlet	0	-22.97909425	-34.17140748	-38.02814782	Control1
AGGGTTTGTTGCGGAA-1	singlet	2	-1376.395039	-1602.762178	-551.9918575	Control1
AGGGTTTTCTTGGGCG-1	singlet	1	-1215.328953	-462.7522937	-1201.291993	Control1
AGGTAGGAGACGCTCC-1	singlet	2	-1271.569703	-1096.869495	-465.7658349	Control1
AGGTAGGAGGACCCAA-1	singlet	2	-263.5060417	-201.5394482	-102.4825239	Control1
AGGTAGGAGGCTGGAT-1	singlet	2	-2789.268668	-2226.185539	-972.1488875	Control1
AGGTAGGAGTCGTTAC-1	singlet	2	-777.9457738	-471.3882716	-168.5769338	Control1
AGGTAGGCAACCGCTG-1	singlet	2	-410.5290131	-312.1132408	-161.8480777	Control1
AGGTAGGCACAATCTG-1	singlet	1	-2666.496402	-959.5004285	-2479.013278	Control1
AGGTAGGCATAACTCG-1	singlet	2	-1866.929397	-2267.515558	-674.1470822	Control1
AGGTAGGGTGTCCTAA-1	singlet	2	-15580.3038	-13332.01554	-4410.550068	Control1
AGGTAGGTCAAGCCGC-1	singlet	2	-1134.362424	-838.4873227	-354.2104827	Control1
AGGTAGGTCCACTGGG-1	singlet	2	-662.3723177	-472.7541178	-200.694211	Control1
AGGTAGGTCCATTCGC-1	singlet	1	-222.2938913	-25.4785431	-47.60280811	Control1
AGGTCATAGGTTAAAC-1	singlet	2	-2465.486799	-2921.835551	-961.9121481	Control1
AGGTCATAGTCACTCA-1	singlet	1	-5195.042384	-1823.625195	-4836.070581	Control1
AGGTCATCAACCTAAC-1	singlet	2	-615.5915331	-1183.361276	-280.7939183	Control1
AGGTCATCAAGTTGGG-1	singlet	2	-814.7376026	-793.1387385	-316.3784483	Control1
AGGTCATCAATCGCGC-1	singlet	2	-575.5735918	-448.7459392	-188.4956103	Control1
AGGTCATCAGTGTACT-1	singlet	2	-3216.902814	-2837.709016	-1056.630207	Control1
AGGTCATCATAACAGA-1	singlet	1	-1219.220469	-496.7225501	-1200.621732	Control1
AGGTCATCATAGATCC-1	singlet	1	-924.744472	-383.0130563	-951.8588274	Control1
AGGTCATGTGGATACG-1	singlet	2	-3764.58114	-3989.129073	-1393.003795	Control1
AGGTCATTCGACGAGA-1	singlet	2	-368.5304695	-271.8966353	-160.700891	Control1
AGGTCATTCTCCTGAC-1	singlet	1	-59.17915023	-26.32813198	-50.35302231	Control1
AGGTCATTCTGGACTA-1	singlet	2	-764.5710443	-618.8682183	-251.3329907	Control1
AGGTCTAAGCGTGAAC-1	singlet	1	-3641.250283	-1277.866665	-3293.435644	Control1
AGGTCTAAGGGTAGCT-1	singlet	1	-2058.104408	-714.2990944	-1837.605883	Control1
AGGTCTAAGGTACAAT-1	singlet	1	-867.0075238	-386.5688336	-853.9335859	Control1
AGGTCTAAGTTCCGGC-1	singlet	0	-414.2045237	-1187.746386	-1153.646258	Control1
AGGTCTACAGACGCTC-1	singlet	2	-332.1752808	-243.9151565	-104.5201701	Control1
AGGTCTACATCCCGTT-1	singlet	2	-875.0176775	-682.3576844	-305.5597299	Control1
AGGTCTAGTCATCCCT-1	singlet	2	-52.88611055	-53.11556615	-22.8465118	Control1
AGGTCTAGTCCTTTGC-1	singlet	0	-94.26715502	-227.3328124	-249.1392746	Control1
AGGTCTAGTGACAACG-1	singlet	2	-1801.945506	-1803.359943	-724.8812139	Control1
AGGTCTATCGTTGTTT-1	singlet	1	-97.77562583	-29.07920252	-62.28361757	Control1
AGGTCTATCTTAAGGC-1	singlet	1	-634.8124415	-266.0555665	-623.9752085	Control1
AGGTGTTAGACCGCCT-1	singlet	1	-687.7492043	-314.2814223	-701.9857428	Control1
AGGTGTTAGCAGCCCT-1	singlet	2	-331.2654875	-322.8167016	-145.0816509	Control1
AGGTGTTAGTAGGATT-1	singlet	2	-625.2957358	-526.6536301	-248.3086435	Control1
AGGTGTTAGTAGTCTC-1	singlet	1	-1432.58531	-632.9930291	-1044.887568	Control1
AGGTGTTCACTCCGGA-1	singlet	1	-1120.278746	-407.088961	-1020.415361	Control1
AGGTGTTCAGTTAAAG-1	singlet	1	-2611.132185	-1118.520408	-2128.300933	Control1
AGGTGTTGTGTCGATT-1	singlet	2	-1534.635939	-1439.031703	-567.7256435	Control1
AGGTGTTGTTGTGGAG-1	singlet	0	-270.7776366	-682.6704227	-850.4844836	Control1
AGGTGTTTCACCCTTG-1	singlet	2	-4087.992786	-3498.408128	-1393.23971	Control1

AGGTGTTTCCTACGAA-1	singlet	2	-494.2458963	-365.2503594	-189.1692893	Control1
AGGTGTTTCGACCATA-1	singlet	1	-2503.373642	-818.0797469	-2277.41871	Control1
AGGTGTTTCTATTTCG-1	singlet	2	-629.1514344	-472.3250729	-215.4346152	Control1
AGGTTACAGAATTTGG-1	singlet	1	-1468.119029	-550.5454737	-1495.122667	Control1
AGGTTACAGCTCGTGC-1	singlet	2	-5936.84832	-4996.824489	-1980.021636	Control1
AGGTTACAGGAAGTCC-1	singlet	2	-3682.442893	-2913.977976	-1347.851955	Control1
AGGTTACCACTTACAG-1	singlet	2	-2312.786936	-2320.372006	-875.535892	Control1
AGGTTACGTGCTTATG-1	singlet	2	-720.415141	-1012.29379	-324.8756	Control1
AGGTTACGTTGCATTG-1	singlet	1	-1017.814177	-373.5578883	-1005.412335	Control1
AGGTTGTAGGCATTTC-1	singlet	2	-1867.975197	-1586.55871	-605.2229225	Control1
AGGTTGTCAAGTGCTT-1	singlet	1	-1928.376632	-779.1822674	-1544.599782	Control1
AGGTTGTCACTCCTGT-1	singlet	2	-19.621019	-8.497676923	-4.322237852	Control1
AGGTTGTCAGACACAG-1	singlet	2	-731.6769454	-558.2201447	-255.7782269	Control1
AGGTTGTGTCTCAGAT-1	singlet	1	-1278.371931	-510.1525511	-976.0959193	Control1
AGGTTGTGTTGTTGCA-1	singlet	0	-155.030739	-417.0442448	-507.614384	Control1
AGGTTGTTCCCTCGTA-1	singlet	1	-2408.398816	-826.0561756	-2278.692951	Control1
AGGTTGTTCCTAACAG-1	singlet	2	-674.8293892	-679.816566	-296.7276362	Control1
AGTAACCAGCCGAATG-1	singlet	1	-704.9779123	-287.2581825	-735.5326697	Control1
AGTAACCAGTTGGAGC-1	singlet	2	-531.1379701	-476.1461623	-157.9755552	Control1
AGTAACCCACGTCATA-1	singlet	2	-1067.421322	-793.0718686	-376.2780594	Control1
AGTAACCCAGCACAGA-1	unassigned	45323	-5.1812672	-4.951962944	-2.678893444	Control1
AGTAACCGTGTCCGTG-1	singlet	2	-2094.894049	-1809.750556	-792.6987538	Control1
AGTAACCTCGCTGACG-1	singlet	2	-1052.704799	-1390.123938	-476.352216	Control1
AGTAACCTCTGTCGCT-1	singlet	2	-1403.825408	-1684.083762	-553.5618629	Control1
AGTACCAAGTTGTACC-1	doublet	0/2	-4425.353427	-7352.956493	-6008.499769	Control1
AGTACCACAAATCCCA-1	singlet	2	-2324.686732	-2120.408876	-834.3055909	Control1
AGTACCACACATACGT-1	singlet	2	-2159.293715	-1886.528148	-782.3718944	Control1
AGTACCACAGACAAAT-1	singlet	2	-5745.118004	-4874.124327	-1656.468402	Control1
AGTACCACAGCTATTG-1	singlet	1	-778.3411032	-304.4898577	-698.9068434	Control1
AGTACCACATGGCCCA-1	singlet	2	-578.4753638	-471.9426909	-210.6981974	Control1
AGTACCAGTCCAGCAC-1	singlet	0	-104.458844	-318.262053	-393.3719057	Control1
AGTACCATCACTACGA-1	singlet	2	-3996.976866	-3498.751768	-1373.929424	Control1
AGTACTGAGGTTTACC-1	singlet	2	-715.0569035	-410.1856566	-213.627628	Control1
AGTACTGCAGGTTCGC-1	singlet	1	-2305.033934	-809.0518129	-2115.44362	Control1
AGTACTGCAGTTCACA-1	singlet	2	-788.8933546	-666.9627052	-271.9798229	Control1
AGTACTGGTAACTTCG-1	singlet	1	-1178.91174	-461.142423	-1198.391668	Control1
AGTACTGGTATGCGGA-1	singlet	1	-2222.38251	-795.1037288	-1916.954974	Control1
AGTACTGGTCCAAATC-1	singlet	2	-1489.568897	-1319.726129	-621.4095233	Control1
AGTACTGGTGCCGTTG-1	singlet	2	-455.5262014	-310.6093355	-145.1622899	Control1
AGTACTGTCATGGTAC-1	singlet	2	-6419.135143	-5397.87242	-1942.435535	Control1
AGTAGCTAGGATATGT-1	singlet	2	-770.5089926	-567.6154477	-230.4200634	Control1
AGTAGCTCAAACGGCA-1	singlet	2	-241.2266212	-187.6357437	-95.41949367	Control1
AGTAGCTCAGGTAGTG-1	singlet	2	-1690.767274	-1608.769291	-689.8534511	Control1
AGTAGCTCATCCAATG-1	singlet	2	-1224.462293	-1066.792543	-510.4525225	Control1
AGTAGCTCATGCGTGC-1	singlet	1	-405.2519301	-204.9571129	-395.5105632	Control1
AGTAGCTGTCCTACGG-1	singlet	2	-677.4857244	-593.1249332	-295.5250657	Control1
AGTAGCTGTCTTACTT-1	singlet	1	-1222.009093	-514.902201	-1151.775746	Control1
AGTAGCTGTCTTCAAG-1	singlet	2	-1263.289156	-1135.615832	-477.4186151	Control1
AGTAGCTGTGTACGCC-1	singlet	2	-582.7836046	-401.7665919	-204.3578101	Control1
AGTAGCTGTTGCTCAA-1	singlet	2	-542.3151901	-435.0222185	-230.717795	Control1
AGTAGCTTCAATCTTC-1	singlet	1	-2708.786855	-1018.581155	-2508.881959	Control1
AGTAGCTTCGCTAAAC-1	singlet	2	-589.3790928	-466.4031297	-231.7831319	Control1
AGTAGCTTCTACTCAT-1	singlet	0	-234.6326468	-662.3822295	-698.5847281	Control1
AGTAGCTTCTGGGAGA-1	singlet	2	-717.5940385	-541.8218218	-248.1660716	Control1
AGTAGTCAGGTCCAGA-1	singlet	1	-4.573126973	-0.789132393	-8.487303712	Control1
AGTAGTCCAAGGTTGG-1	singlet	1	-1067.696413	-426.4072537	-1062.927837	Control1
AGTAGTCCATAACCCA-1	singlet	2	-555.4481042	-417.7043825	-198.3937739	Control1
AGTAGTCGTCACCGAC-1	singlet	0	-245.4089479	-560.0279301	-665.7222986	Control1
AGTAGTCGTGTATACC-1	singlet	1	-1618.511123	-342.9967335	-1699.830925	Control1
AGTAGTCTCACAGAGG-1	singlet	2	-547.1612254	-380.795572	-173.9772351	Control1
AGTAGTCTCACTGATG-1	singlet	2	-647.8112326	-441.6713303	-179.749677	Control1
AGTAGTCTCCGCAGTG-1	singlet	2	-1258.942606	-1096.251717	-370.7477331	Control1
AGTAGTCTCCTACGGG-1	unassigned	45323	-459.5652944	-41.26946741	-26.20380238	Control1
AGTCAACAGGATACAT-1	singlet	2	-1329.651249	-1220.049608	-480.8382682	Control1
AGTCAACAGTTGCTGT-1	singlet	2	-4633.622117	-4210.748837	-1306.631209	Control1
AGTCAACGTACCGTGC-1	singlet	2	-6222.414509	-5649.404014	-2050.02665	Control1
AGTCAACGTCGATTCA-1	singlet	1	-1251.153579	-511.1287818	-1108.316388	Control1
AGTCAACGTGCATGTT-1	singlet	2	-1437.392611	-1183.504552	-513.6869998	Control1
AGTCAACGTGCCTTTC-1	singlet	2	-643.5770026	-410.8193761	-185.9693584	Control1
AGTCAACTCTAACGCA-1	singlet	2	-1127.418051	-914.5940133	-423.5835024	Control1
AGTCACAAGCTATCTG-1	singlet	2	-1086.337218	-1169.95871	-471.4158364	Control1
AGTCACACAGCCTACG-1	singlet	2	-2117.340768	-1844.938942	-833.7455198	Control1
AGTCACAGTATCGTAC-1	singlet	2	-3075.50763	-4515.407753	-1025.422954	Control1
AGTCACAGTGGACCTC-1	singlet	0	-1489.047631	-4106.375108	-4370.714898	Control1
AGTCACAGTTGCCTAA-1	singlet	2	-1421.224768	-1147.682815	-493.730232	Control1
AGTCACATCTAAGCCA-1	singlet	1	-2156.371989	-444.7474897	-1142.106687	Control1
AGTCATGAGTTACGAA-1	singlet	2	-2464.245721	-2105.498586	-902.6445705	Control1
AGTCATGCAATAACGA-1	singlet	1	-3698.08669	-1431.976873	-3573.394533	Control1
AGTCATGCAGATGCGA-1	singlet	2	-2860.026642	-3576.416071	-1055.887838	Control1
AGTCATGGTAACTTCG-1	singlet	2	-240.5035924	-196.2667116	-97.03098503	Control1
AGTCATGGTACGATCT-1	singlet	2	-786.8111552	-541.7267062	-245.0997997	Control1
AGTCATGGTACTGAGG-1	singlet	2	-1951.355865	-1597.002988	-581.7132932	Control1
AGTCATGGTAGTCTTG-1	singlet	1	-1815.601135	-608.3033527	-1770.121062	Control1
AGTCATGGTCACCACG-1	singlet	2	-560.453438	-436.0387652	-245.1325342	Control1
AGTCATGTCTTTCGAT-1	singlet	2	-1318.601121	-1094.120534	-420.1177723	Control1
AGTCTCCCACTCCACT-1	singlet	1	-174.8716225	-75.42102691	-165.969516	Control1
AGTCTCCCACTCCGAG-1	singlet	2	-1008.143865	-871.1764095	-385.204098	Control1
AGTCTCCCAGCACACC-1	singlet	1	-4928.616402	-1403.221151	-4429.391632	Control1
AGTCTCCGTACCCACG-1	singlet	2	-540.4402427	-375.6778021	-168.2370277	Control1
AGTCTCCGTAGACAGC-1	singlet	2	-4337.386619	-3625.273298	-1508.628575	Control1
AGTCTCCGTCCCGCAA-1	singlet	1	-981.2673548	-378.8117249	-944.3935076	Control1
AGTCTCCTCCAAGAGG-1	singlet	2	-1190.170857	-1001.028298	-406.8901354	Control1
AGTCTCCTCGCTATTT-1	singlet	2	-765.6866702	-594.2102578	-273.2349666	Control1
AGTGACTCATTGACCA-1	singlet	0	-7.162485666	-15.35402051	-12.25549423	Control1
AGTGACTGTTAGGCTT-1	singlet	2	-893.5196291	-640.0482992	-262.9569746	Control1
AGTGACTTCAACACCA-1	singlet	2	-1553.183045	-1391.633602	-602.31615	Control1
AGTGACTTCCGCCTAT-1	singlet	1	-535.6632677	-202.8277042	-513.964208	Control1
AGTGACTTCGTGTGAT-1	singlet	1	-693.0973417	-289.1452491	-687.2600932	Control1
AGTGATCAGGACGCAT-1	singlet	1	-3111.097124	-986.9973383	-2576.310945	Control1
AGTGATCAGGCATTTC-1	singlet	2	-3892.863374	-3214.306436	-1235.175137	Control1
AGTGATCAGGGCAATC-1	singlet	2	-729.823007	-572.7395884	-271.4099955	Control1
AGTGATCCACAGTGAG-1	singlet	1	-543.7691447	-232.3056709	-595.280475	Control1
AGTGATCCAGTCGTTA-1	singlet	1	-512.0233852	-220.1453676	-525.1322562	Control1
AGTGATCCATTGCAAC-1	singlet	1	-1137.179912	-453.8018755	-1029.298626	Control1
AGTGATCGTATAGGGC-1	singlet	2	-2582.585637	-4335.423956	-865.9944599	Control1
AGTGATCGTCAATGGG-1	singlet	2	-945.724313	-726.3088258	-337.7977484	Control1
AGTGATCGTGGTCTTA-1	singlet	2	-1212.483383	-1114.787507	-499.1659463	Control1
AGTGATCTCCCTATTA-1	singlet	2	-574.5888626	-487.9040726	-253.1787489	Control1
AGTGATCTCCTGGCTT-1	singlet	2	-2721.999811	-2290.738958	-946.879876	Control1
AGTGATCTCCTGTACC-1	singlet	2	-875.3822955	-630.9941194	-284.8117779	Control1

AGTGATCTCGGCTCTT-1	singlet	1	-2130.807881	-739.5992699	-2118.68582	Control1
AGTGCCGAGCTGTACT-1	singlet	1	-17.3105536	-7.816776594	-15.07977134	Control1
AGTGCCGCAACTCCCT-1	singlet	2	-788.3151328	-669.2921584	-286.9029966	Control1
AGTGCCGCAACTGATC-1	doublet	45323	-1730.937112	-1549.752658	-1190.45383	Control1
AGTGCCGCAAGGTCAG-1	singlet	2	-578.80227	-546.8210886	-284.1404625	Control1
AGTGCCGCACAACGCC-1	singlet	2	-2313.738152	-1919.732033	-765.776134	Control1
AGTGCCGCAGTAGTGG-1	singlet	2	-611.2205738	-653.9062805	-234.3651952	Control1
AGTGCCGCATCATTGG-1	singlet	2	-258.1286921	-169.5228927	-88.61047644	Control1
AGTGCCGGTACGAAAT-1	singlet	1	-1542.12588	-566.171076	-1447.536299	Control1
AGTGCCGTCAGATTGC-1	singlet	0	-2215.563687	-6578.067406	-6855.286511	Control1
AGTGCCGTCGCACGAC-1	singlet	2	-1721.845766	-1514.334085	-660.2518517	Control1
AGTGCCGTCGCCAGTG-1	singlet	1	-581.2196749	-253.7579541	-532.192021	Control1
AGTGTTGAGGGCAAGG-1	singlet	2	-1405.788659	-1276.276982	-501.9761743	Control1
AGTGTTGCACACTGGC-1	singlet	1	-873.0527932	-357.9311319	-910.8277753	Control1
AGTGTTGCAGTCTCTC-1	singlet	2	-1870.172952	-2244.687055	-756.977887	Control1
AGTGTTGTCGCGATCG-1	singlet	2	-423.9153938	-293.8570973	-144.5032647	Control1
AGTTAGCAGTACGAGC-1	singlet	2	-255.2228249	-229.6489157	-91.21043277	Control1
AGTTAGCAGTCTGGAG-1	singlet	1	-1942.200353	-678.1455788	-1984.694621	Control1
AGTTAGCAGTTTGTCG-1	singlet	0	-88.9165315	-172.4424343	-225.6910209	Control1
AGTTAGCCAACCAATC-1	singlet	1	-1130.99999	-460.2227287	-1120.156354	Control1
AGTTAGCCAACTGGTT-1	singlet	2	-2075.363015	-2355.600291	-765.012578	Control1
AGTTAGCCACTTGAGT-1	singlet	2	-3338.821265	-2780.82218	-1141.874072	Control1
AGTTAGCGTACGGATG-1	singlet	1	-691.3501576	-270.050865	-605.3791526	Control1
AGTTAGCGTAGCGCTC-1	singlet	2	-2095.167968	-1803.837815	-684.270079	Control1
AGTTAGCGTGACGTCC-1	singlet	2	-710.6746044	-464.2708818	-250.5833565	Control1
AGTTAGCGTTCGTGCG-1	singlet	2	-1375.944315	-1230.978782	-447.6709107	Control1
AGTTAGCTCGTCTCAC-1	singlet	2	-2275.227978	-2068.258856	-778.8992527	Control1
AGTTCCCAGACGGAAA-1	singlet	2	-435.2346707	-360.122345	-174.5662714	Control1
AGTTCCCAGTAAGAGG-1	singlet	1	-9812.578597	-2926.653738	-9147.805884	Control1
AGTTCCCCACATTCGA-1	singlet	2	-818.4852041	-655.1729525	-350.0269515	Control1
AGTTCCCCACCCATAA-1	singlet	1	-161.754486	-51.75254008	-132.2849113	Control1
AGTTCCCCATCCCGTT-1	doublet	2/0	-1769.594453	-1965.517813	-1257.6753	Control1
AGTTCCCGTGACTCGC-1	singlet	0	-263.054439	-729.3670391	-1014.795246	Control1
AGTTCCCTCACACCCT-1	singlet	2	-661.7379049	-502.0328079	-272.2348584	Control1
AGTTCCCTCACTGAAC-1	singlet	2	-4669.71118	-3929.516716	-1483.573109	Control1
AGTTCGAAGCATTTCG-1	singlet	1	-1233.176308	-565.9298145	-1171.968971	Control1
AGTTCGACAGAGATGC-1	singlet	2	-566.9385987	-385.8008589	-179.762075	Control1
AGTTCGACATAGTCGT-1	singlet	1	-2817.3011	-933.8103319	-2467.825569	Control1
AGTTCGATCTAGCCTC-1	singlet	1	-134.915235	-44.67924264	-109.8528659	Control1
ATACCGAAGCAAATGT-1	singlet	2	-1100.279854	-1032.793366	-388.438233	Control1
ATACCGAAGCCATGCC-1	singlet	2	-5402.683569	-5653.27115	-1737.151651	Control1
ATACCGAAGGTACTGG-1	singlet	2	-433.8515213	-409.2547503	-200.5828317	Control1
ATACCGAAGGTGGCTA-1	singlet	2	-1644.471169	-1564.412094	-566.7525075	Control1
ATACCGACAACAGCTT-1	singlet	2	-1317.532605	-1549.541865	-534.5885286	Control1
ATACCGACAGATGCGA-1	singlet	1	-1859.221039	-731.8809017	-1830.918371	Control1
ATACCGAGTTTACCTT-1	singlet	2	-406.6171025	-318.2028826	-160.5980928	Control1
ATACCGATCAGGAAAT-1	singlet	2	-2503.519611	-2458.57586	-1010.930889	Control1
ATACCGATCTGAATGC-1	unassigned	2/0	-31.18917373	-46.98494938	-25.53805255	Control1
ATACCTTAGACGTCGA-1	singlet	2	-421.7074996	-354.3731208	-180.5113138	Control1
ATACCTTAGCAAGGAA-1	singlet	2	-1180.785897	-1141.37473	-485.5351509	Control1
ATACCTTAGCTGAAGC-1	singlet	2	-1771.068075	-1455.863318	-647.7203463	Control1
ATACCTTCAAGAGTAT-1	singlet	2	-680.048586	-447.1283599	-240.1828663	Control1
ATACCTTCAAGTACCT-1	singlet	1	-1197.951855	-500.3936531	-1158.858007	Control1
ATACCTTCAATGGCAG-1	singlet	2	-1584.109259	-1409.769778	-601.7681274	Control1
ATACCTTCACAGCATT-1	singlet	2	-5360.375991	-4325.799186	-1921.619886	Control1
ATACCTTCATCCGGTG-1	singlet	1	-1823.666634	-670.324221	-2020.885548	Control1
ATACCTTGTAAGTCAA-1	singlet	1	-1258.688577	-547.0445576	-1320.050182	Control1
ATACCTTGTGACCTGC-1	singlet	2	-4829.77452	-3919.623109	-1363.987489	Control1
ATACCTTTCAGGTGTT-1	singlet	1	-788.8136324	-309.5470583	-731.6406616	Control1
ATACTTCAGCTCAGAG-1	singlet	2	-1595.585457	-1804.197236	-681.4522271	Control1
ATACTTCCATGGAACG-1	singlet	1	-3639.89877	-1405.262379	-3518.789471	Control1
ATACTTCGTCAGCGTC-1	singlet	2	-659.567722	-640.9963344	-259.4075347	Control1
ATACTTCGTGACAGGT-1	singlet	2	-518.7855922	-495.1157669	-209.9913026	Control1
ATACTTCTCATCACTT-1	singlet	1	-1203.799091	-502.9205745	-1021.401561	Control1
ATACTTCTCGAACCTA-1	singlet	2	-806.9785689	-558.7999118	-240.6494095	Control1
ATACTTCTCGGTTCAA-1	singlet	1	-1604.801552	-565.1823866	-1449.424755	Control1
ATAGACCAGAAGGTAG-1	singlet	1	-769.8440785	-272.0203621	-569.9885611	Control1
ATAGACCAGAGCCGAT-1	singlet	2	-659.2776217	-609.0885281	-282.9263185	Control1
ATAGACCCACCTGCGA-1	singlet	0	-205.1338443	-600.5729784	-643.442829	Control1
ATAGACCCACTGTGTA-1	singlet	2	-3731.137886	-3001.64693	-1162.095141	Control1
ATAGACCCAGCAAGAC-1	singlet	2	-4135.984178	-3490.126511	-1157.392204	Control1
ATAGACCGTACCAATC-1	singlet	0	-1739.972992	-5514.546034	-5487.83516	Control1
ATAGACCGTCTTTATC-1	doublet	0/2	-60.77184778	-99.71356008	-91.27319334	Control1
ATAGACCTCCAATCCC-1	singlet	2	-838.0035453	-776.212398	-381.3826391	Control1
ATAGACCTCCATCGTC-1	singlet	2	-645.7353068	-456.6406952	-201.5311824	Control1
ATAGACCTCTTAAGGC-1	singlet	2	-873.2393706	-664.399475	-293.5812293	Control1
ATAGAGAAGAACTCCT-1	singlet	1	-637.7108958	-239.0288175	-537.8481223	Control1
ATAGAGAAGGCGTTGA-1	singlet	2	-2194.92034	-2741.839442	-895.7247938	Control1
ATAGAGACAATGCAGG-1	singlet	2	-2543.317304	-2411.033377	-903.13173	Control1
ATAGAGACACGTTCGG-1	singlet	2	-1921.03528	-1689.872193	-673.3101174	Control1
ATAGAGATCCGTTGAA-1	singlet	1	-2993.977653	-1193.685911	-2734.523598	Control1
ATAGAGATCGAACGCC-1	singlet	1	-597.8775399	-215.8717503	-587.0565438	Control1
ATAGAGATCTCATTTG-1	singlet	1	-211.4402563	-64.71018663	-187.5673011	Control1
ATAGAGATCTGGCCTT-1	singlet	2	-1444.546285	-1377.760494	-573.9889956	Control1
ATAGAGATCTTAGCTT-1	singlet	1	-1085.145922	-483.3475119	-1071.910647	Control1
ATAGGCTAGAACCCGA-1	singlet	1	-1207.45996	-443.2388552	-1067.674826	Control1
ATAGGCTAGGAAAGAC-1	singlet	2	-3205.29332	-3083.309089	-1207.927105	Control1
ATAGGCTAGGTACAAT-1	singlet	2	-2020.113486	-1869.373006	-712.9569705	Control1
ATAGGCTAGGTGATCG-1	singlet	2	-1664.964831	-1568.688901	-526.2133578	Control1
ATAGGCTAGTAGTGCG-1	singlet	2	-2944.297601	-3833.538933	-993.2288476	Control1
ATAGGCTCAAGACCGA-1	singlet	2	-475.0084956	-353.7645856	-133.6745676	Control1
ATAGGCTCAGCGGTCT-1	singlet	1	-2992.59311	-1007.7975	-2852.803702	Control1
ATAGGCTCATCCGTTC-1	singlet	2	-838.3983466	-586.4443174	-273.8438518	Control1
ATAGGCTGTCCAAAGG-1	singlet	1	-11779.43261	-3327.358978	-11401.57393	Control1
ATAGGCTGTCGTCTCT-1	singlet	2	-653.5977882	-493.2696921	-227.8589772	Control1
ATAGGCTGTCTCCTGT-1	singlet	2	-1241.040105	-1403.027047	-525.27704	Control1
ATAGGCTGTTAGCTAC-1	singlet	2	-706.0712434	-570.4269633	-268.9030602	Control1
ATAGGCTGTTCGGTTA-1	singlet	2	-111.7914221	-86.74341549	-41.76178008	Control1
ATAGGCTTCAGCGCGT-1	singlet	2	-807.7039949	-857.0767117	-337.9553491	Control1
ATAGGCTTCCAACCAA-1	singlet	2	-702.7291755	-538.6439974	-262.5019073	Control1
ATAGGCTTCCGCAGTG-1	singlet	1	-1154.859553	-473.703875	-1165.71516	Control1
ATATCCTAGACAGCTG-1	singlet	2	-3554.545605	-5262.425484	-1222.78454	Control1
ATATCCTAGCGTGAGT-1	singlet	2	-2471.36503	-2290.029547	-890.0263691	Control1
ATATCCTAGTCGCCAC-1	singlet	2	-390.9599123	-348.9685261	-122.3896327	Control1
ATATCCTCAATAGAGT-1	singlet	0	-341.4221694	-860.7347597	-968.5364078	Control1
ATATCCTCATATTCGG-1	doublet	45323	-3905.340821	-2926.993247	-1776.021114	Control1
ATATCCTCATGTTCGA-1	singlet	2	-3050.815204	-2789.374955	-1189.939614	Control1
ATATCCTGTCAGTTTG-1	singlet	2	-659.326829	-711.9869385	-283.6291125	Control1
ATATCCTGTCTAGTGT-1	singlet	2	-870.8648836	-636.5560385	-292.6666206	Control1
ATATCCTGTGTCCGGT-1	singlet	1	-1037.558496	-422.996803	-903.5757843	Control1

ATATCCTTCACATACG-1	singlet	2	-2278.648626	-1934.860357	-762.0454954	Control1
ATATCCTTCTTGTTAC-1	singlet	2	-781.5038334	-635.9758929	-293.5169556	Control1
ATCACAGAGAAGCGAA-1	singlet	2	-531.1636226	-400.1419608	-165.3337752	Control1
ATCACAGAGACTCTAC-1	singlet	2	-2678.807987	-2283.147844	-848.6030416	Control1
ATCACAGAGCAGATAT-1	singlet	0	-199.4283713	-459.8047317	-689.1101526	Control1
ATCACAGCATGTCAGT-1	singlet	0	-2168.408675	-6698.349433	-7681.800666	Control1
ATCACAGGTGAGCAGT-1	doublet	0/2	-790.3795096	-983.8405203	-906.0391241	Control1
ATCACAGGTTATGACC-1	singlet	2	-572.499947	-493.2531741	-218.6235563	Control1
ATCACAGTCAATCCAG-1	singlet	2	-2322.914613	-1903.994596	-866.2755715	Control1
ATCACAGTCATTACGG-1	singlet	2	-883.1110711	-667.9336134	-282.1971594	Control1
ATCACAGTCGGTAACT-1	singlet	2	-304.1624032	-298.8895121	-129.7865784	Control1
ATCACAGTCTCGCTCA-1	singlet	2	-514.9405449	-442.952629	-197.4648814	Control1
ATCACAGTCTGGCCGA-1	singlet	2	-631.9529895	-473.7006028	-211.276255	Control1
ATCACGAAGATTGTGA-1	singlet	1	-1687.957129	-703.9919406	-1721.116258	Control1
ATCACGAAGCCATCCG-1	singlet	2	-1198.388214	-1086.117656	-417.330303	Control1
ATCACGAAGTCTCCTC-1	singlet	2	-685.0595816	-626.5078995	-210.8667388	Control1
ATCACGACAAGCGAAC-1	singlet	1	-1041.656084	-435.1417621	-886.7808397	Control1
ATCACGACAAGGTCGA-1	singlet	2	-7663.987741	-6458.010536	-2250.346345	Control1
ATCACGACAGGTCAAG-1	singlet	2	-908.1633395	-824.939843	-309.4793629	Control1
ATCACGACATCATTTC-1	singlet	2	-968.2332378	-759.7016006	-347.2084745	Control1
ATCACGAGTCGAGCTC-1	singlet	2	-466.2061244	-429.8999429	-187.9921192	Control1
ATCACGAGTGGTTTGT-1	singlet	0	-1257.042059	-3891.96004	-4247.470891	Control1
ATCACGAGTTCCATTT-1	singlet	2	-577.726993	-445.0644068	-214.5495276	Control1
ATCACGATCGGACGTC-1	singlet	1	-694.2923309	-293.7142168	-605.5481538	Control1
ATCACGATCTAGTGAC-1	singlet	1	-1106.546451	-425.3189317	-1091.575125	Control1
ATCACTTAGACCTTTG-1	singlet	1	-1983.487518	-644.7411393	-1799.737268	Control1
ATCACTTAGCTGACTT-1	singlet	2	-109.225876	-98.19020604	-52.16370395	Control1
ATCACTTAGGATTCCT-1	singlet	1	-1730.763054	-709.110803	-1523.059348	Control1
ATCACTTAGTCAGGGT-1	doublet	0/1	-9.757209433	-11.57983836	-17.91477955	Control1
ATCACTTAGTTGCTGT-1	singlet	0	-164.6667272	-384.0207382	-368.1930219	Control1
ATCACTTCAGCAGACA-1	singlet	1	-4191.547196	-1398.140963	-3611.201267	Control1
ATCACTTGTAGCGTCC-1	singlet	1	-1025.894582	-380.9126233	-1024.403654	Control1
ATCACTTGTGGTAACG-1	singlet	2	-1739.01792	-1411.165386	-560.9484205	Control1
ATCACTTTCGAGTCCG-1	singlet	2	-1958.815464	-1962.280814	-749.535928	Control1
ATCAGGTAGCCGTAAG-1	singlet	2	-1318.286804	-1157.696734	-521.6117439	Control1
ATCAGGTAGCTGGTGA-1	singlet	1	-5794.012657	-2155.841351	-5573.179204	Control1
ATCAGGTAGTATAGGT-1	singlet	0	-1196.005091	-5095.856282	-6112.016007	Control1
ATCAGGTCACCAGCGT-1	singlet	0	-1814.798871	-5018.686159	-5543.184326	Control1
ATCAGGTCAGACACCC-1	singlet	1	-1261.786654	-502.7842229	-1293.160315	Control1
ATCAGGTCATTGACTG-1	singlet	1	-1129.163472	-487.4945976	-1184.511421	Control1
ATCAGGTGTTAGAAGT-1	singlet	2	-606.9152724	-517.7699263	-211.1989043	Control1
ATCAGGTTCAAGTCGT-1	singlet	2	-418.0233952	-357.6202008	-154.657358	Control1
ATCAGGTTCCTCTAAT-1	singlet	2	-1211.267305	-1189.478962	-508.3615937	Control1
ATCATTCAGCATTTGC-1	singlet	2	-2590.918435	-2584.859851	-924.5553773	Control1
ATCATTCAGGAAGTAG-1	singlet	1	-1103.590674	-461.2294712	-1104.688778	Control1
ATCATTCCACGAAGAC-1	singlet	2	-59.05286251	-68.75547328	-23.50757095	Control1
ATCATTCCACGATTCA-1	singlet	2	-379.8635275	-287.3878358	-141.2321279	Control1
ATCATTCCAGTCTACA-1	singlet	2	-3562.718576	-2906.262689	-1294.815464	Control1
ATCATTCCATGGCCCA-1	singlet	2	-560.9297964	-395.5414031	-158.1795152	Control1
ATCATTCGTCCGAAGA-1	singlet	2	-2116.503938	-2177.446203	-776.5277495	Control1
ATCATTCGTCCTGTCT-1	singlet	0	-220.0737283	-579.598579	-752.8258262	Control1
ATCATTCGTGAGCGAT-1	unassigned	1	-2.31548807	-1.937162877	-2.549464213	Control1
ATCATTCGTGCCGTAC-1	singlet	2	-558.94438	-463.1814796	-223.3912966	Control1
ATCATTCGTTCCGGTG-1	singlet	2	-808.1592554	-661.2196399	-297.5242269	Control1
ATCATTCTCAAAGCCT-1	singlet	1	-597.3025618	-250.1765108	-560.9012045	Control1
ATCATTCTCACCTTGC-1	singlet	2	-2176.598248	-1880.81221	-769.2831406	Control1
ATCATTCTCGCGAAGA-1	singlet	2	-441.7772779	-391.8798445	-192.5785226	Control1
ATCATTCTCTAGAACC-1	singlet	2	-1442.860886	-1304.52194	-600.1953832	Control1
ATCATTCTCTGGTGCG-1	singlet	2	-1690.034591	-1545.369115	-551.8607502	Control1
ATCCACCAGAAACCAT-1	singlet	1	-411.8458806	-22.7390061	-36.84465329	Control1
ATCCACCAGACCAGCA-1	singlet	2	-1612.274828	-1593.862499	-726.9700088	Control1
ATCCACCAGCATGCGA-1	singlet	2	-1610.734091	-1751.799346	-684.7640302	Control1
ATCCACCAGCCGAACA-1	singlet	1	-1667.416105	-787.1722971	-1701.95915	Control1
ATCCACCAGGTTCATC-1	singlet	2	-4344.534492	-3957.846962	-1684.746478	Control1
ATCCACCAGTCATCCA-1	singlet	1	-1335.7572	-591.3512823	-1402.322132	Control1
ATCCACCCATCCTAAG-1	singlet	2	-764.8574555	-613.3702034	-228.2953072	Control1
ATCCACCGTATGGAGC-1	singlet	1	-5617.627839	-1432.110874	-5350.815759	Control1
ATCCACCGTTCGCGTG-1	singlet	2	-730.5302553	-529.8477087	-226.99817	Control1
ATCCACCGTTCGGTAT-1	singlet	2	-565.5323153	-418.5676442	-184.7853013	Control1
ATCCACCTCGCGGTAC-1	singlet	2	-242.50736	-237.4216989	-126.640004	Control1
ATCCACCTCGGCATAT-1	singlet	1	-1539.523904	-641.5176312	-1653.221136	Control1
ATCCACCTCTATACGG-1	unassigned	0/1	-12.70208335	-15.02358823	-22.07094938	Control1
ATCCATTAGCGCAATG-1	singlet	2	-1115.296441	-1038.744356	-533.4826106	Control1
ATCCATTAGGGATCAC-1	singlet	2	-295.9537444	-235.6328627	-123.8757177	Control1
ATCCATTCACGTACTA-1	singlet	1	-1217.534923	-475.7822814	-1103.391762	Control1
ATCCATTCAGCGACAA-1	singlet	2	-1150.842391	-1031.655184	-465.5677723	Control1
ATCCATTCATGGAACG-1	singlet	2	-2132.928616	-1798.621723	-716.5113521	Control1
ATCCATTGTAGCGATG-1	singlet	2	-861.4156217	-721.0129134	-296.379607	Control1
ATCCATTGTGCAACGA-1	singlet	2	-518.320645	-457.5763022	-194.0240244	Control1
ATCCATTTCCCTCGTA-1	singlet	2	-3816.81237	-4656.506148	-1404.109399	Control1
ATCCATTTCTAACGCA-1	unassigned	2	-7.870531598	-6.792586561	-3.255733692	Control1
ATCCATTTCTCGACGG-1	singlet	1	-4197.107459	-1483.793397	-4156.02378	Control1
ATCCCTGAGACTACCT-1	singlet	2	-3512.677949	-3144.759119	-1191.120772	Control1
ATCCCTGAGCTAATGA-1	singlet	2	-485.8482065	-438.0285732	-214.5196929	Control1
ATCCCTGAGGAAAGTG-1	singlet	2	-4437.984912	-4247.020712	-1597.993565	Control1
ATCCCTGCAATAAGGT-1	singlet	2	-236.8324549	-198.5541791	-92.38870728	Control1
ATCCCTGCACTTGTGA-1	singlet	2	-2431.843302	-2075.540087	-909.839562	Control1
ATCCCTGGTAGACACG-1	singlet	2	-606.2169172	-476.4687763	-249.4221686	Control1
ATCCCTGGTCGTGTTA-1	singlet	2	-332.1336144	-243.8677134	-92.27822631	Control1
ATCCCTGTCAAAGGAT-1	singlet	1	-1206.934266	-477.5976671	-1135.421334	Control1
ATCCCTGTCTACCTTA-1	singlet	1	-6408.634342	-2244.648117	-5694.171091	Control1
ATCCGTCAGGTAAAGG-1	singlet	2	-780.027013	-614.3332067	-256.1648834	Control1
ATCCGTCCACTTGAAC-1	singlet	2	-2702.328594	-2269.404794	-861.4481956	Control1
ATCCGTCCAGCTATAC-1	unassigned	1/0	-18.31295592	-11.47431993	-19.37951361	Control1
ATCCGTCGTAGTGCGA-1	singlet	1	-921.4971463	-425.3706989	-898.1400617	Control1
ATCCGTCGTCCCTGTT-1	singlet	2	-2166.168094	-1865.302771	-777.1441131	Control1
ATCCGTCTCCTACACC-1	singlet	2	-7347.979285	-5867.739478	-2366.035674	Control1
ATCCTATCACAGCGCT-1	singlet	1	-1110.595214	-414.8190296	-1085.934144	Control1
ATCCTATCATTATGCG-1	singlet	2	-1369.784656	-1292.159796	-575.3592993	Control1
ATCCTATCATTGAAGA-1	singlet	1	-1010.564933	-396.453152	-999.6961628	Control1
ATCCTATTCTTAGTTC-1	unassigned	1	-9.742477275	-4.504161084	-14.2198855	Control1
ATCGATGAGCTTGTTG-1	singlet	2	-1557.795711	-1395.95306	-598.2263415	Control1
ATCGATGAGCTTTGTG-1	singlet	2	-228.2479253	-181.1204741	-82.54546352	Control1
ATCGATGAGGGTCACA-1	doublet	2/0	-11458.7168	-12472.7537	-8315.149736	Control1
ATCGATGAGTCAATCC-1	singlet	1	-9890.834766	-2666.445616	-8880.245787	Control1
ATCGATGCAATCTAGC-1	singlet	1	-1874.017623	-630.4689079	-1922.364357	Control1
ATCGATGCATTACTCT-1	singlet	2	-1723.107233	-1440.742168	-584.720034	Control1
ATCGATGGTACTGACT-1	singlet	1	-1789.088826	-675.4126452	-1613.023102	Control1
ATCGATGGTAGACAAT-1	singlet	2	-610.3181577	-445.7247052	-205.693124	Control1
ATCGATGGTGAATGAT-1	singlet	2	-109.793478	-98.75897993	-58.08466141	Control1

ATCGCCTAGAGAGGTA-1	singlet	2	-1902.985646	-1709.763275	-719.5515237	Control1
ATCGCCTAGATTAGTG-1	singlet	2	-804.3627568	-738.8370466	-336.9387976	Control1
ATCGCCTAGGGTAATT-1	singlet	2	-303.2945515	-195.1070728	-93.49435607	Control1
ATCGCCTAGGTAAGTT-1	singlet	2	-755.3253309	-1084.591531	-319.2253417	Control1
ATCGCCTCAATGTCTG-1	singlet	1	-1211.077854	-510.1061248	-1261.185698	Control1
ATCGCCTCACACAGAG-1	singlet	1	-1613.271639	-676.0016042	-1598.556887	Control1
ATCGCCTCACCAGCCA-1	singlet	1	-289.2826745	-62.52059259	-205.724085	Control1
ATCGCCTCAGAATTCC-1	singlet	2	-2119.444894	-2062.01708	-781.846901	Control1
ATCGCCTCAGACAAGC-1	singlet	1	-995.279102	-413.3313576	-877.6252207	Control1
ATCGCCTGTCAGTCTA-1	singlet	1	-1682.650795	-531.3196026	-1660.755715	Control1
ATCGCCTGTGACGTCC-1	singlet	2	-485.9667144	-309.260388	-163.0194541	Control1
ATCGCCTGTGAGATCG-1	singlet	0	-37.3097246	-78.88575825	-104.2233576	Control1
ATCGCCTGTGCATCTA-1	singlet	2	-452.1704095	-380.8112291	-159.3222194	Control1
ATCGCCTGTGCTGTCG-1	singlet	1	-72.42260846	-24.11559229	-57.13571635	Control1
ATCGCCTGTTATTCCT-1	singlet	2	-734.3615687	-624.8125077	-272.0724364	Control1
ATCGCCTGTTGGGTAG-1	singlet	0	-46.217943	-202.5682515	-197.9581774	Control1
ATCGCCTTCGATTGGT-1	singlet	2	-452.569172	-355.4054701	-168.9553186	Control1
ATCGGATAGAGAGTTT-1	singlet	2	-2453.394021	-2218.474541	-864.53983	Control1
ATCGGATAGAGCAAGA-1	singlet	2	-3505.639823	-4480.746049	-1298.074222	Control1
ATCGGATCACCAGTAT-1	singlet	2	-6530.202141	-5555.778252	-1856.926393	Control1
ATCGGATCACGTACAT-1	singlet	2	-2468.512613	-2121.88089	-889.127823	Control1
ATCGGATCAGCTGTCG-1	singlet	2	-1098.72633	-889.5150131	-445.4376737	Control1
ATCGGATCAGGATCTT-1	singlet	2	-680.3400698	-606.7858178	-246.8008254	Control1
ATCGGATGTCTTCATT-1	singlet	2	-2028.424217	-1687.598988	-547.961116	Control1
ATCGGATTCCTCTAAT-1	singlet	2	-7726.281035	-6163.209572	-2188.842976	Control1
ATCGGATTCTCCTACG-1	singlet	2	-1348.195776	-1201.953303	-526.8129987	Control1
ATCGGCGAGATCCTAC-1	singlet	2	-1250.437326	-1235.617735	-466.0596666	Control1
ATCGGCGAGCGATTCT-1	singlet	1	-352.5506307	-145.4734545	-301.1827889	Control1
ATCGGCGAGCGTTAGG-1	singlet	1	-164.246099	-57.54071585	-164.9712652	Control1
ATCGGCGAGGTGCTAG-1	singlet	2	-2589.685908	-2272.663848	-945.0202018	Control1
ATCGGCGCACCAGGTC-1	singlet	2	-2040.462661	-1797.347303	-659.0215186	Control1
ATCGGCGCACTATCGA-1	singlet	1	-510.0502278	-237.2627164	-493.8760417	Control1
ATCGGCGCATACACCA-1	singlet	2	-775.6525134	-537.1475485	-260.8917825	Control1
ATCGGCGGTAACACCT-1	singlet	2	-1993.241009	-1795.44288	-873.8360201	Control1
ATCGGCGTCCGTTTCG-1	singlet	2	-719.6077912	-539.807063	-229.0221793	Control1
ATCGGCGTCGATTGGT-1	singlet	2	-1022.277874	-1336.452106	-458.4876903	Control1
ATCGGCGTCGGCATCG-1	singlet	1	-357.8759582	-164.3370879	-360.2600169	Control1
ATCGGCGTCTATACTC-1	singlet	2	-1941.022519	-1623.492887	-668.0580411	Control1
ATCGGCGTCTCGACGG-1	doublet	45323	-2419.054755	-1638.61467	-1417.638686	Control1
ATCGGCGTCTGGTCAA-1	singlet	1	-975.4248139	-318.8930952	-709.9764544	Control1
ATCGTAGAGCAGTAAT-1	singlet	2	-1651.981403	-1438.538028	-601.6960994	Control1
ATCGTAGAGCGCTGCT-1	singlet	1	-709.8252003	-308.0406221	-709.7105875	Control1
ATCGTAGCAAGTGCAG-1	singlet	1	-2163.69404	-759.5729721	-2086.395069	Control1
ATCGTAGCACCCTGAG-1	singlet	2	-1964.913433	-1726.797429	-687.1714535	Control1
ATCGTAGCACCTGAAT-1	doublet	1/0	-1427.881095	-1140.806232	-1770.740954	Control1
ATCGTAGCAGTAACGG-1	singlet	2	-8948.347603	-9439.256321	-2738.60462	Control1
ATCGTAGGTCATATGC-1	singlet	1	-2598.951346	-894.3812458	-1997.592456	Control1
ATCGTAGGTCTGATCA-1	singlet	1	-1451.866229	-564.1864403	-1422.612947	Control1
ATCGTAGGTGTACGCC-1	singlet	2	-819.5083403	-702.2893826	-359.3171229	Control1
ATCGTAGGTGTCCGTG-1	singlet	1	-1211.811656	-462.3665597	-1172.398301	Control1
ATCGTAGTCGGCCCAA-1	singlet	1	-1511.447746	-605.9184363	-1511.148511	Control1
ATCGTAGTCGTTACCC-1	singlet	1	-2324.854223	-787.9871271	-2306.496619	Control1
ATCGTAGTCGTTAGAC-1	singlet	2	-6243.016286	-5181.066775	-1937.132226	Control1
ATCGTCCAGTCGCCCA-1	singlet	1	-1711.266286	-668.8907842	-1755.225954	Control1
ATCGTCCAGTGACCTT-1	singlet	1	-1376.120293	-528.5725095	-1436.571307	Control1
ATCGTCCCAACCGTGC-1	singlet	2	-617.8921903	-460.0777092	-206.5295385	Control1
ATCGTCCCAGTTGAAA-1	singlet	2	-2125.912263	-2054.697495	-786.4236291	Control1
ATCGTCCCATAGAATG-1	singlet	2	-1807.937037	-1656.111292	-636.4462677	Control1
ATCGTCCGTGAATGAT-1	singlet	2	-512.6845983	-412.8817264	-156.0025458	Control1
ATCGTCCTCAACACGT-1	singlet	2	-128.5437742	-134.5266343	-62.14705879	Control1
ATCGTCCTCCGTCACT-1	singlet	2	-280.5481214	-193.378817	-113.2368385	Control1
ATCGTCCTCGAATGCT-1	singlet	0	-131.7706317	-461.7046407	-473.8253648	Control1
ATCGTGAAGAAACTGT-1	singlet	2	-423.0330011	-307.8645078	-152.9793101	Control1
ATCGTGAAGGCCTGAA-1	singlet	2	-1275.490479	-1179.627917	-533.0854055	Control1
ATCGTGACACTACAGT-1	unassigned	2	-10.12334507	-12.73308844	-8.076860212	Control1
ATCGTGACAGTTCCAA-1	singlet	1	-700.5705799	-272.4346343	-638.5091007	Control1
ATCGTGAGTACATTGC-1	singlet	2	-618.2087599	-500.2931754	-267.3405745	Control1
ATCGTGAGTACCGGCT-1	singlet	2	-427.3144608	-282.8966985	-140.9166059	Control1
ATCGTGAGTACTCGCG-1	doublet	2/0	-137.4070495	-141.8763576	-81.05399757	Control1
ATCGTGAGTGACTGAG-1	singlet	2	-2700.127746	-2192.683712	-916.5591726	Control1
ATCGTGAGTTTCGACA-1	singlet	1	-806.5837802	-321.1420123	-911.6883893	Control1
ATCGTGATCTCGCGTT-1	singlet	2	-7145.428877	-6104.24136	-2569.565993	Control1
ATCGTGATCTCTCGCA-1	singlet	2	-803.9990641	-650.3361314	-292.1090773	Control1
ATCTCTAAGGGCAGTT-1	singlet	2	-409.625867	-389.6532958	-167.7689036	Control1
ATCTCTACAAGGCAAC-1	singlet	1	-3949.197155	-1534.276634	-3708.878284	Control1
ATCTCTACAGCTGAGA-1	singlet	2	-578.3970385	-552.3229491	-280.2938662	Control1
ATCTCTACATCAGCTA-1	singlet	1	-557.9813701	-269.309683	-685.6775722	Control1
ATCTCTAGTAAGCTCT-1	singlet	1	-1189.509013	-471.240809	-1176.229783	Control1
ATCTCTAGTCATCGGC-1	singlet	1	-822.6436425	-329.9557141	-705.8194129	Control1
ATCTCTATCTCTCGCA-1	singlet	2	-244.4559619	-206.5243668	-87.19152075	Control1
ATCTCTATCTTGGTGA-1	singlet	1	-2010.197313	-699.4003428	-1761.005652	Control1
ATCTTCAAGGCTTAGG-1	singlet	1	-751.1087416	-286.1328617	-725.8764257	Control1
ATCTTCAAGTCGCCCA-1	singlet	1	-1020.944206	-384.2281481	-1038.956399	Control1
ATCTTCACAACGATCT-1	singlet	2	-3663.613489	-3139.616113	-1245.57598	Control1
ATCTTCACAAGTACCT-1	singlet	2	-2947.935989	-2721.287364	-1123.796084	Control1
ATCTTCACACGAGAAC-1	singlet	1	-3431.984093	-1202.005306	-3409.450354	Control1
ATCTTCAGTAGCGTAG-1	singlet	2	-383.1765038	-308.1013477	-172.9329658	Control1
ATCTTCAGTAGGCTGA-1	singlet	2	-602.7055221	-524.1467557	-228.2437767	Control1
ATCTTCAGTCTTGCGG-1	singlet	2	-1306.330741	-1252.238159	-520.2961688	Control1
ATCTTCAGTTCACCGG-1	singlet	2	-463.4205932	-332.6369152	-146.7992768	Control1
ATCTTCAGTTCTTAGG-1	singlet	2	-2525.028158	-2270.583403	-767.106278	Control1
ATCTTCATCTACACAG-1	singlet	1	-3444.311603	-1188.685208	-3222.253381	Control1
ATCTTCATCTGCGTCT-1	singlet	2	-165.5571128	-231.2852196	-59.62826587	Control1
ATGAAAGAGACAGCGT-1	unassigned	1	-2.224805665	-0.905723781	-1.633272328	Control1
ATGAAAGAGAGGTTAT-1	singlet	2	-2823.562794	-2338.479059	-953.9600851	Control1
ATGAAAGCAACTGTGT-1	singlet	1	-478.4146421	-213.8654833	-479.7291611	Control1
ATGAAAGCAGCTATTG-1	singlet	2	-4462.011656	-3854.209854	-1397.848205	Control1
ATGAAAGCAGGCACAA-1	singlet	1	-1031.120542	-429.2103157	-990.6747016	Control1
ATGAAAGCATCATCCC-1	singlet	2	-1158.654274	-991.195315	-383.7306999	Control1
ATGAAAGCATCCGGTG-1	singlet	2	-950.5300945	-812.2325416	-335.835592	Control1
ATGAAAGCATTGCTGA-1	singlet	1	-2367.077413	-764.0562678	-4880.684028	Control1
ATGAAAGGTGTAGCAG-1	singlet	2	-2180.646511	-1907.439215	-736.6887704	Control1
ATGAAAGTCATGCTAG-1	singlet	2	-3860.708789	-3280.597814	-1328.850913	Control1
ATGAAAGTCGTGTCAA-1	singlet	1	-19.07336918	-11.19044855	-21.67650567	Control1
ATGAAAGTCTGGGCGT-1	singlet	2	-383.5985449	-287.5151571	-122.1251915	Control1
ATGACCAAGATAGGGA-1	singlet	2	-1021.399073	-938.7616193	-367.6771246	Control1
ATGACCAAGCGGTAAC-1	singlet	2	-6962.823903	-6186.220191	-2391.290772	Control1
ATGACCAAGTTTAGGA-1	singlet	2	-1206.402917	-1572.788076	-501.7248027	Control1
ATGACCACACAGTATC-1	singlet	1	-1606.280904	-644.9428484	-1470.296966	Control1
ATGACCAGTACAGTCT-1	singlet	2	-1918.815593	-1659.241212	-716.158552	Control1

ATGACCAGTCACGTGC-1	singlet	2	-658.8703627	-547.1243887	-211.3611093	Control1
ATGACCAGTCACTAGT-1	singlet	2	-1604.919581	-1321.515752	-558.5161273	Control1
ATGACCAGTCCAGGTC-1	singlet	2	-2602.372801	-2608.089908	-941.1173703	Control1
ATGACCAGTGGAACAC-1	singlet	2	-936.4903606	-805.4624585	-317.3844971	Control1
ATGACCAGTTGCATGT-1	singlet	1	-1528.262947	-547.534668	-1436.29297	Control1
ATGAGGGAGGCTCTAT-1	singlet	2	-1153.187605	-1267.966015	-471.3220464	Control1
ATGAGGGCAAGACGAC-1	singlet	1	-1118.961975	-485.5680322	-1179.537031	Control1
ATGAGGGCATTGCTGA-1	singlet	1	-715.9259459	-268.9162011	-707.7740872	Control1
ATGAGGGGTATTCTCT-1	singlet	2	-4771.020418	-4109.934814	-1356.196456	Control1
ATGAGGGGTGCTCGTG-1	singlet	1	-987.4915721	-396.7920471	-957.4966914	Control1
ATGAGGGTCAACCGAT-1	doublet	45323	-9113.642865	-6806.582127	-4570.440709	Control1
ATGAGGGTCCGGCAGT-1	singlet	1	-102.2545261	-39.6765654	-96.13969936	Control1
ATGAGGGTCGATACTG-1	singlet	2	-376.5603097	-255.6218387	-140.4607135	Control1
ATGAGGGTCTGGGCAC-1	singlet	2	-2339.340051	-1901.013138	-805.7212326	Control1
ATGAGTCAGGTTTGAA-1	singlet	2	-680.0961321	-712.1729229	-280.3706151	Control1
ATGAGTCCAAGTCCCG-1	singlet	2	-1659.828938	-1435.343126	-655.314002	Control1
ATGAGTCCACAAGCTT-1	singlet	2	-983.981274	-875.2369706	-381.5915343	Control1
ATGAGTCCACACACTA-1	singlet	2	-699.2649284	-547.9897413	-261.3619927	Control1
ATGAGTCCACCACATA-1	singlet	1	-3923.603247	-1503.454304	-3655.424263	Control1
ATGAGTCCACGGGTAA-1	singlet	2	-1568.290108	-1467.956381	-540.1157719	Control1
ATGAGTCCAGCATACT-1	singlet	1	-1762.172194	-652.359529	-1740.17046	Control1
ATGAGTCGTGTTCATG-1	singlet	2	-775.2221451	-625.4944501	-240.0165892	Control1
ATGATCGAGAAGCCAC-1	singlet	1	-899.9445819	-344.1629566	-791.7276697	Control1
ATGATCGAGAAGGTAG-1	singlet	1	-630.3556184	-242.5178886	-628.1180183	Control1
ATGATCGAGTCTCCTC-1	singlet	2	-663.4212072	-460.2392668	-227.5907664	Control1
ATGATCGCACAAAGTA-1	singlet	2	-646.9810194	-444.2481763	-208.6382844	Control1
ATGATCGCATGATCTG-1	singlet	2	-548.9746732	-504.9968621	-232.1390162	Control1
ATGATCGCATTGAAAG-1	singlet	1	-4249.814454	-1523.964916	-4123.30887	Control1
ATGATCGGTGAACCGA-1	unassigned	45293	-10.26217651	-8.703701822	-10.67892847	Control1
ATGATCGGTTAACAGA-1	singlet	2	-1849.496567	-1791.931531	-720.3627776	Control1
ATGATCGGTTGGGCCT-1	singlet	2	-1176.388786	-1107.359746	-465.6395187	Control1
ATGATCGTCAGGAAAT-1	singlet	2	-547.6918832	-354.6277537	-180.4262106	Control1
ATGCATGAGACGAGCT-1	singlet	2	-1189.6341	-965.3570405	-447.9158852	Control1
ATGCATGTCACGTAGT-1	singlet	2	-586.7322426	-354.3314422	-201.7784552	Control1
ATGCATGTCCGTGGGT-1	singlet	1	-1261.730737	-470.1773284	-1273.975435	Control1
ATGCATGTCGAACACT-1	singlet	2	-352.9366179	-311.0983936	-120.9636132	Control1
ATGCCTCAGACAGCTG-1	singlet	2	-4346.225933	-3719.051553	-1550.266801	Control1
ATGCCTCAGCAAATCA-1	singlet	1	-1864.296901	-738.2224593	-1643.132672	Control1
ATGCCTCAGGCCTGAA-1	singlet	2	-1692.60868	-1525.200847	-702.1300395	Control1
ATGCCTCCACACACGC-1	singlet	1	-2719.063866	-1024.014336	-2506.112879	Control1
ATGCCTCGTCATCCCT-1	singlet	2	-388.0729761	-227.5729608	-115.3724669	Control1
ATGCCTCGTTGTGGAG-1	singlet	2	-1638.682955	-1522.083378	-582.876231	Control1
ATGCGATAGAGGCGTT-1	singlet	1	-812.0769176	-291.2404228	-755.7604961	Control1
ATGCGATCAAGCACAG-1	singlet	2	-2098.223466	-1721.442156	-685.9499361	Control1
ATGCGATCACTGGCGT-1	singlet	2	-715.071334	-553.2750682	-236.333779	Control1
ATGCGATGTATGGAAT-1	singlet	2	-2015.707091	-1970.892071	-686.0417188	Control1
ATGCGATGTTTGAAAG-1	singlet	2	-913.7716384	-1123.368151	-363.1401238	Control1
ATGCGATTCACCATCC-1	singlet	2	-1603.299067	-1525.324897	-714.6552322	Control1
ATGCGATTCGCTCTAC-1	singlet	2	-3184.421524	-3015.65334	-1256.400506	Control1
ATGGAGGAGCTTGTGT-1	singlet	2	-657.9891752	-847.0557428	-321.3039081	Control1
ATGGAGGCAGGTAGTG-1	singlet	2	-829.1264085	-1108.438952	-348.5286216	Control1
ATGGAGGTCAAGATAG-1	singlet	2	-3633.402058	-2961.255199	-1179.909728	Control1
ATGGAGGTCAAGTGTC-1	singlet	1	-1746.888126	-642.0695802	-1622.440424	Control1
ATGGAGGTCGACCATA-1	singlet	1	-1726.71478	-663.7594315	-1848.387188	Control1
ATGGAGGTCGGCTTGG-1	singlet	2	-1836.666929	-1662.535847	-571.9474703	Control1
ATGGAGGTCTGAGAGG-1	singlet	1	-627.8596763	-235.1492236	-642.9684026	Control1
ATGGATCAGACTGTTC-1	singlet	2	-1846.378192	-1979.841366	-718.5955718	Control1
ATGGATCAGTCGTTAC-1	singlet	2	-484.0012627	-421.7793311	-177.9851643	Control1
ATGGATCCAAGTGGGT-1	singlet	2	-517.4580316	-401.1509976	-191.6038614	Control1
ATGGATCGTAGACACG-1	singlet	2	-784.197336	-593.3511214	-235.0757613	Control1
ATGGATCGTCCTGTCT-1	singlet	2	-656.0097284	-598.8595121	-237.9301493	Control1
ATGGATCTCATACAGC-1	singlet	2	-2059.91721	-1881.499108	-716.8664981	Control1
ATGGATCTCGCTCATC-1	singlet	1	-4918.492019	-1610.062593	-4648.016306	Control1
ATGGATCTCTCCAAGA-1	singlet	0	-705.5610354	-2143.053333	-2520.365481	Control1
ATGGGAGGTGAACGGT-1	singlet	2	-1643.901087	-1520.456589	-484.9823687	Control1
ATGGGAGGTTACGATC-1	singlet	2	-952.2033346	-710.5921422	-296.7140703	Control1
ATGGGAGTCAATCCAG-1	singlet	2	-1764.236523	-1646.752577	-636.2094363	Control1
ATGGGAGTCATCAGTG-1	singlet	2	-3232.964879	-2639.542785	-1184.786958	Control1
ATGGGAGTCGAGAGAC-1	singlet	1	-1257.523563	-419.9717936	-1150.527412	Control1
ATGGGAGTCTCTCTAA-1	singlet	2	-549.7693477	-438.9172354	-200.6127188	Control1
ATGGGTTAGCCTGGAA-1	singlet	2	-1133.085695	-965.5845412	-409.0346315	Control1
ATGGGTTCAAGTCGTT-1	singlet	2	-2424.719211	-2217.271996	-815.2006963	Control1
ATGGGTTGTCACCTTC-1	singlet	1	-479.5942582	-193.1844178	-408.223569	Control1
ATGGGTTTCCAGTTCC-1	singlet	0	-329.1535535	-968.3087584	-970.4052348	Control1
ATGGTTGAGCTACAAA-1	singlet	2	-514.0603234	-391.7370482	-175.2267014	Control1
ATGGTTGAGGCCCGTT-1	singlet	1	-2491.624698	-864.1239666	-1980.397302	Control1
ATGGTTGCAGTCTTCC-1	singlet	2	-710.9193125	-501.0361221	-210.204741	Control1
ATGGTTGGTGGATCGA-1	singlet	2	-497.5175768	-335.6264744	-156.6081407	Control1
ATGGTTGGTTAGAGTA-1	singlet	0	-211.0529729	-554.2801178	-628.405272	Control1
ATGGTTGGTTCCACAA-1	singlet	2	-656.4316631	-581.3224255	-249.4225766	Control1
ATGTCCCAGATAGTGT-1	singlet	2	-604.4857635	-470.2402363	-246.4791451	Control1
ATGTCCCAGGATATAC-1	singlet	0	-1433.089945	-5631.318297	-5482.035281	Control1
ATGTCCCGTTCTATCT-1	singlet	2	-2361.861629	-2095.699203	-954.6286368	Control1
ATGTCCCTCCATCAGA-1	singlet	0	-46.82483693	-142.8337724	-152.5376336	Control1
ATGTCTTAGAATTTGG-1	singlet	1	-696.8033584	-254.8272984	-740.2892149	Control1
ATGTCTTAGGCACTCC-1	singlet	1	-1045.365028	-429.9911078	-909.0111965	Control1
ATGTCTTAGTGCACTT-1	singlet	1	-2292.98208	-870.4857179	-2362.841209	Control1
ATGTCTTCAACACGTT-1	singlet	2	-323.9655406	-288.469213	-104.5892164	Control1
ATGTCTTCAATTGTGC-1	singlet	0	-292.3594295	-688.226999	-783.2026793	Control1
ATGTCTTGTACAAAGT-1	singlet	1	-3965.008618	-1423.347552	-3506.30885	Control1
ATGTCTTGTATCAGGG-1	singlet	2	-1797.323964	-1471.888457	-623.6334716	Control1
ATGTCTTGTGGACTAG-1	singlet	2	-3059.480655	-3013.810765	-1170.239457	Control1
ATGTCTTTCCCATACC-1	singlet	2	-1195.468085	-1062.944785	-490.539422	Control1
ATGTCTTTCGGACTTA-1	singlet	1	-1331.344122	-510.1651455	-1186.76163	Control1
ATTACCTAGTCACTGT-1	singlet	2	-999.908585	-903.7721983	-464.8467	Control1
ATTACCTGTAACAAGT-1	singlet	2	-4765.259081	-4358.677139	-1517.668695	Control1
ATTACCTGTAGGAGTC-1	singlet	1	-1698.273578	-742.6159453	-1544.310966	Control1
ATTACCTGTCTTACAG-1	singlet	2	-2118.707182	-1696.20062	-805.6126525	Control1
ATTACCTGTTTGTGGT-1	singlet	2	-1982.817637	-1674.522323	-735.8689673	Control1
ATTACCTTCAGCAATC-1	singlet	2	-368.2738922	-259.2338912	-127.3300485	Control1
ATTACCTTCGGACAAG-1	singlet	1	-605.2026016	-250.1233142	-635.4729531	Control1
ATTACCTTCGTAGCCG-1	singlet	2	-876.2864793	-602.8949124	-270.5848991	Control1
ATTACCTTCTTTCAGT-1	singlet	2	-857.9866076	-603.8848223	-261.4133236	Control1
ATTACTCCAAGCAATA-1	singlet	2	-6434.012972	-5338.515674	-2008.63269	Control1
ATTACTCCAGAACTCT-1	singlet	1	-1411.075338	-628.4383473	-1464.704502	Control1
ATTACTCCAGCACAAG-1	singlet	2	-2114.104463	-1727.163048	-756.1410043	Control1
ATTACTCCATCCAACA-1	singlet	2	-1731.182788	-2154.641919	-756.4953396	Control1
ATTACTCCATGGAAGC-1	singlet	2	-92.25111318	-77.58627774	-24.26197333	Control1
ATTACTCGTAACGATA-1	singlet	2	-683.7560973	-486.6417684	-194.8779413	Control1
ATTACTCGTGTTAAAG-1	singlet	2	-642.1764491	-521.9854752	-222.3722386	Control1

ATTACTCTCGATTTCT-1	singlet	2	-903.9770217	-993.0454435	-377.3796077	Control1
ATTATCCAGACCTCCG-1	singlet	2	-531.8115268	-437.6408725	-164.0311085	Control1
ATTATCCAGCCGAATG-1	singlet	2	-74.01493187	-56.23118727	-26.67455412	Control1
ATTATCCAGGACCCAA-1	singlet	1	-1555.419621	-591.6338555	-1551.89701	Control1
ATTATCCCAATCTAGC-1	singlet	0	-34.88882432	-84.18064764	-103.4912604	Control1
ATTATCCCAGAGTTCT-1	singlet	1	-4538.699346	-1701.782412	-4052.694329	Control1
ATTATCCCATAGAAAC-1	singlet	2	-682.2224668	-417.8232622	-227.2429993	Control1
ATTATCCGTAGGACTG-1	unassigned	45323	-109.0320562	-82.58117889	-44.56260044	Control1
ATTATCCGTCAAAGTA-1	singlet	1	-947.8515499	-374.8976754	-948.7099031	Control1
ATTATCCGTCAAGTTC-1	singlet	2	-531.158038	-405.616131	-203.186116	Control1
ATTATCCGTTCCGGTG-1	singlet	2	-617.9630277	-475.7118488	-226.2961357	Control1
ATTATCCGTTTGGGTT-1	singlet	0	-1617.407745	-4910.780515	-5466.557196	Control1
ATTATCCTCGAGAACG-1	singlet	1	-1529.047997	-644.9164784	-1524.95864	Control1
ATTATCCTCGATAACC-1	singlet	2	-1686.939908	-1542.032998	-635.408217	Control1
ATTATCCTCGCCGATG-1	singlet	2	-610.1333022	-379.29427	-187.345215	Control1
ATTCACTCACGCAGTC-1	singlet	2	-510.8221236	-393.0366654	-201.2986774	Control1
ATTCACTCAGAGCGTA-1	singlet	1	-3518.019068	-1129.266528	-2931.827926	Control1
ATTCACTCAGCCATTA-1	singlet	2	-2172.918088	-1856.577238	-756.9436414	Control1
ATTCACTCATATCGGT-1	singlet	2	-1349.698126	-1179.921193	-445.411634	Control1
ATTCACTGTCACCTTC-1	singlet	2	-2571.421891	-2316.552324	-987.1186653	Control1
ATTCACTGTCGAACAG-1	singlet	2	-928.4647999	-711.0015667	-331.6378349	Control1
ATTCACTTCCATCTGC-1	singlet	2	-1984.520165	-1699.352178	-762.8993502	Control1
ATTCACTTCGACACTA-1	singlet	1	-1589.930719	-693.7155851	-1518.073831	Control1
ATTCAGGAGCGTGCCT-1	singlet	1	-962.7353969	-352.846078	-997.6306715	Control1
ATTCAGGAGCTGACAG-1	singlet	0	-195.6422358	-432.8580888	-579.7872381	Control1
ATTCAGGAGTATCCTG-1	singlet	1	-1293.12375	-530.9854289	-1241.113043	Control1
ATTCAGGAGTGGCCTC-1	singlet	1	-621.7352562	-269.0076963	-670.4354056	Control1
ATTCAGGCACTCTGCT-1	singlet	2	-1400.134598	-1559.404858	-629.6107706	Control1
ATTCAGGCATAAGCAA-1	singlet	2	-1605.281645	-1693.380782	-690.0893821	Control1
ATTCAGGCATTAGGCT-1	singlet	2	-1158.13034	-829.5417975	-334.8563392	Control1
ATTCAGGGTCCTTGTC-1	singlet	2	-557.1011544	-488.7217086	-216.6139178	Control1
ATTCAGGGTCTAATCG-1	singlet	2	-953.282759	-848.6530592	-385.5190167	Control1
ATTCAGGTCCAACCGG-1	singlet	2	-1222.20758	-1135.743816	-477.0698983	Control1
ATTCATCAGAAGCGGG-1	singlet	1	-754.8565727	-293.8668607	-719.4398356	Control1
ATTCATCAGCGATGAC-1	singlet	2	-1008.374455	-946.2044314	-346.9888631	Control1
ATTCATCAGGCTCACC-1	singlet	2	-735.188904	-613.3794756	-280.1545836	Control1
ATTCATCAGTAACGAT-1	singlet	2	-1669.394831	-1509.753169	-729.7816155	Control1
ATTCATCAGTCCTGCG-1	singlet	2	-1949.660306	-1581.237746	-654.3523038	Control1
ATTCATCCAGACAAGC-1	singlet	0	-1124.102418	-3380.93878	-3905.738471	Control1
ATTCATCCAGTAGAAT-1	singlet	2	-5401.674504	-4558.938113	-1823.962556	Control1
ATTCATCGTATGCTAC-1	singlet	1	-448.6481259	-155.4294534	-396.4414236	Control1
ATTCATCTCCATAGAC-1	singlet	2	-4773.628566	-4116.63725	-1485.520348	Control1
ATTCCATAGATCACCT-1	singlet	2	-729.3163505	-609.9289702	-289.385791	Control1
ATTCCATAGGTTGGAC-1	singlet	2	-490.6193098	-321.0891072	-129.0182313	Control1
ATTCCATCAATCTGCA-1	singlet	2	-2438.676666	-2301.319829	-898.0456234	Control1
ATTCCATCATAGATCC-1	singlet	0	-1781.094722	-3416.596639	-3317.358747	Control1
ATTCCATCATTCTTCA-1	singlet	2	-877.4398297	-819.7417755	-340.1997662	Control1
ATTCCATGTAGACAGC-1	singlet	2	-4076.628405	-3605.052478	-1586.885214	Control1
ATTCCATGTAGCTGAG-1	singlet	1	-2962.392617	-923.9168915	-2542.586711	Control1
ATTCCATGTAGTACGG-1	singlet	1	-1589.367334	-615.8905338	-1546.420651	Control1
ATTCCATGTCGGATTT-1	singlet	0	-775.4595965	-2121.330949	-2437.763109	Control1
ATTCCATGTTCAAACC-1	unassigned	1	-4.339166879	-3.608513673	-3.946236906	Control1
ATTCCATGTTCATCGA-1	singlet	2	-1228.801575	-1147.25874	-503.415806	Control1
ATTCCCGAGAGTGAAG-1	singlet	1	-949.6739666	-427.4560418	-908.0529832	Control1
ATTCCCGAGCCAAGTG-1	singlet	2	-212.6998899	-155.6732195	-62.06044069	Control1
ATTCCCGAGCTCAGAG-1	singlet	1	-2245.480054	-712.2272146	-2104.477505	Control1
ATTCCCGAGGACAAGA-1	singlet	1	-5544.138133	-1708.048855	-4621.23532	Control1
ATTCCCGAGTAAGGGA-1	unassigned	1	-11.11744432	-4.840583379	-5.954711403	Control1
ATTCCCGAGTTCCATG-1	singlet	2	-600.0667681	-372.9570657	-196.7268737	Control1
ATTCCCGCAGAGACTG-1	singlet	2	-1800.508865	-1611.318296	-651.9493291	Control1
ATTCCCGCAGTGACCC-1	singlet	2	-745.4317414	-498.5888322	-302.3094916	Control1
ATTCCCGCATTGCAAC-1	singlet	2	-2532.670894	-2117.052063	-959.5966952	Control1
ATTCCCGGTAGAAACT-1	singlet	2	-1308.108269	-1532.675614	-574.5811325	Control1
ATTCCCGGTTATCTTC-1	singlet	0	-1509.616828	-3875.379096	-4133.962393	Control1
ATTCCCGTCATTGCTT-1	singlet	2	-798.9925354	-682.2412965	-306.1751331	Control1
ATTCCCGTCGCGAAGA-1	singlet	0	-164.4574155	-479.3110735	-637.9995407	Control1
ATTCCTAAGAAACTGT-1	singlet	2	-762.7645424	-703.0806453	-292.9842967	Control1
ATTCCTAAGCCGGAAT-1	singlet	2	-581.4880818	-359.0914145	-183.849624	Control1
ATTCCTACAAACGAGC-1	singlet	2	-321.5525669	-293.9899728	-129.1798422	Control1
ATTCCTACAATAACCC-1	singlet	1	-1315.100505	-510.3578689	-1223.997988	Control1
ATTCCTACAGCGACCT-1	singlet	2	-642.6779906	-441.0018427	-213.739303	Control1
ATTCGTTAGAATACAC-1	singlet	1	-2036.237586	-677.9897377	-1734.500859	Control1
ATTCGTTAGACTAAGT-1	singlet	2	-1122.31365	-947.7541976	-375.1024558	Control1
ATTCGTTAGGAAACGA-1	singlet	2	-843.5263366	-659.6106205	-317.3992512	Control1
ATTCGTTAGGTCGCCT-1	singlet	2	-1204.815717	-1085.371585	-435.6344968	Control1
ATTCGTTAGTGCTCGC-1	singlet	2	-565.6534373	-448.4801439	-214.3001751	Control1
ATTCGTTCACAATTCG-1	singlet	1	-1374.803421	-586.8321857	-1283.81701	Control1
ATTCGTTCACATGTTG-1	singlet	2	-618.4049482	-420.2379126	-199.9046119	Control1
ATTCGTTGTAACGTTC-1	singlet	0	-1355.27282	-3974.779656	-4241.026793	Control1
ATTCGTTGTCTAGTGT-1	singlet	2	-335.5137479	-331.5537332	-139.2210363	Control1
ATTCGTTTCCTATTTG-1	unassigned	45323	-15.92979174	-11.9774855	-5.865867149	Control1
ATTCTACAGCTGGCCT-1	singlet	1	-1479.015207	-583.0466679	-1470.016867	Control1
ATTCTACAGTATCTGC-1	singlet	2	-1649.414344	-1823.684542	-717.0911492	Control1
ATTCTACCAACTGTGT-1	singlet	2	-1821.582108	-1762.085727	-704.8074581	Control1
ATTCTACCAAGTTTGC-1	singlet	2	-60.88875787	-51.46023601	-24.9900823	Control1
ATTCTACCAATCCTAG-1	singlet	2	-1058.178485	-852.9603747	-395.7943444	Control1
ATTCTACCACTTGGGC-1	singlet	2	-29.39755128	-46.69595758	-9.143672576	Control1
ATTCTACCATTCTTCA-1	singlet	2	-2711.835013	-2387.732954	-994.270348	Control1
ATTCTACGTACAAAGT-1	singlet	2	-3281.986597	-2815.89455	-1273.466446	Control1
ATTCTACGTATCACGT-1	singlet	1	-6321.58377	-1678.116685	-5720.611228	Control1
ATTCTACTCTGGACCG-1	singlet	1	-1659.546792	-685.5710357	-1519.031647	Control1
ATTCTTGAGACATGCG-1	singlet	0	-772.0769225	-1848.695787	-2030.803722	Control1
ATTCTTGAGCCTTGAT-1	singlet	1	-911.6539802	-385.8170236	-910.2432641	Control1
ATTCTTGAGGAGTCTG-1	singlet	2	-2092.082624	-2061.184593	-776.1006267	Control1
ATTCTTGAGTCGGGAT-1	singlet	1	-7.300400782	-1.877895865	-15.38873953	Control1
ATTCTTGCACCTCAGG-1	singlet	1	-4554.304972	-1870.325834	-4537.043181	Control1
ATTCTTGGTTGTGGCC-1	singlet	2	-3097.100081	-2562.626402	-1059.309476	Control1
ATTGGGTAGACCATTC-1	singlet	2	-1744.723586	-1576.838845	-616.1083612	Control1
ATTGGGTAGATTAGTG-1	singlet	2	-1133.702489	-1226.811922	-457.8493664	Control1
ATTGGGTAGGCTGTAG-1	singlet	2	-4328.29782	-3891.591651	-1179.074013	Control1
ATTGGGTCAAGATGGC-1	singlet	2	-697.6479028	-522.7594606	-233.967314	Control1
ATTGGGTCAGCTGCCA-1	singlet	1	-1677.266556	-586.8217242	-1578.256952	Control1
ATTGGGTCATGTGGTT-1	singlet	2	-922.3267223	-864.8798286	-316.2321729	Control1
ATTGGGTGTAGTGATA-1	singlet	2	-5787.359848	-5006.464116	-1605.334823	Control1
ATTGGGTGTGCTCCGA-1	singlet	1	-413.0661626	-160.5493749	-358.0302729	Control1
ATTGGGTGTGTTATCG-1	singlet	2	-978.8151369	-1323.467227	-406.3286672	Control1
ATTGGGTGTTGACGGA-1	singlet	2	-2341.934839	-1985.360843	-688.6351561	Control1
ATTGGGTGTTTGTTGG-1	singlet	1	-963.6304618	-406.4776552	-992.9140613	Control1
ATTGGGTTCACTGATG-1	singlet	2	-560.5735364	-404.4524286	-178.4043005	Control1
ATTGGGTTCGAGAAGC-1	singlet	1	-685.1035468	-288.0534048	-713.8147695	Control1

ATTGGGTTCGGAGTAG-1	singlet	1	-413.1674022	-185.4822714	-382.4826912	Control1
ATTGTTCAGCAGCAGT-1	singlet	1	-852.5391428	-354.1791555	-783.7619552	Control1
ATTGTTCCAGGGTCTC-1	singlet	2	-361.1154519	-241.5169614	-128.1306936	Control1
ATTGTTCGTAAGCAAT-1	singlet	2	-4481.16287	-3999.833778	-1464.996502	Control1
ATTGTTCGTTGGGCCT-1	doublet	1/0	-1121.273689	-760.3785531	-1280.299053	Control1
ATTGTTCTCATCACCC-1	singlet	1	-494.3063446	-210.8614382	-519.8246014	Control1
ATTTACCAGTCGGGAT-1	singlet	2	-1192.826915	-1097.934492	-431.2939256	Control1
ATTTACCCAGAGTAAT-1	doublet	45323	-1233.955699	-961.6896153	-794.9372821	Control1
ATTTACCCATGCACTA-1	singlet	2	-3124.47411	-2613.986191	-1265.805665	Control1
ATTTACCGTCGCTCGA-1	singlet	2	-764.9407365	-896.5429899	-304.8631131	Control1
ATTTACCGTGCAGTGA-1	singlet	2	-5004.607668	-5177.463202	-1520.101911	Control1
ATTTACCGTTCAGTAC-1	singlet	2	-905.678215	-693.3048731	-332.5445477	Control1
ATTTACCTCATCCTGC-1	singlet	1	-3252.487996	-999.4816244	-2797.34166	Control1
ATTTACCTCATGCGGC-1	singlet	1	-1911.91641	-720.1644809	-1770.141565	Control1
ATTTACCTCCGGCTTT-1	singlet	1	-1444.113068	-585.7403316	-1433.281339	Control1
ATTTACCTCTGTGTGA-1	singlet	2	-1247.255644	-1476.129679	-575.3239755	Control1
ATTTCACAGAGAGAAC-1	singlet	2	-1374.473671	-1132.02283	-468.1357187	Control1
ATTTCACAGGAGCTGT-1	singlet	0	-2.879917303	-6.673564885	-6.435775159	Control1
ATTTCACCAAATCAGA-1	singlet	1	-586.2285668	-228.4650762	-602.1361339	Control1
ATTTCACCAACCGTAT-1	singlet	1	-1312.170663	-542.8112634	-1407.88111	Control1
ATTTCACTCAAGTCGT-1	singlet	0	-133.7386774	-355.2324643	-408.5882179	Control1
ATTTCACTCATTCGGA-1	unassigned	2	-3.515418533	-1.407245128	-0.998237656	Control1
ATTTCACTCTAACGGT-1	singlet	2	-1378.491313	-1212.166724	-482.8550728	Control1
ATTTCACTCTCATGCC-1	singlet	1	-825.8749225	-381.3029272	-879.2887217	Control1
ATTTCACTCTGGCTGG-1	singlet	0	-1099.466722	-2934.189371	-3265.758686	Control1
ATTTCTGAGCTAAACA-1	singlet	2	-1251.21746	-1196.523883	-512.5065649	Control1
ATTTCTGAGTGCGACA-1	singlet	1	-700.6913751	-270.877061	-673.7308528	Control1
ATTTCTGCACTTCATT-1	singlet	2	-931.5724906	-1090.099142	-370.1415218	Control1
ATTTCTGCATCGAAGG-1	singlet	2	-872.1892799	-883.9301745	-320.1360409	Control1
ATTTCTGCATGGGCAA-1	singlet	1	-991.6326034	-353.6291262	-818.8387078	Control1
ATTTCTGGTAAGATTG-1	singlet	2	-110.3913673	-94.83598674	-24.9987325	Control1
ATTTCTGGTCGAATTC-1	singlet	2	-336.2415427	-231.6467019	-118.1826084	Control1
ATTTCTGTCCTGATAG-1	singlet	0	-2642.170245	-4583.490993	-4246.88218	Control1
ATTTCTGTCGACTCCT-1	singlet	0	-92.00109345	-258.9394124	-364.8372133	Control1
ATTTCTGTCTCCGTGT-1	singlet	2	-1915.393086	-1886.480151	-680.1990013	Control1
ATTTCTGTCTGGGCGT-1	singlet	2	-368.3833718	-265.3335518	-118.9933973	Control1
CAAAGAAAGTCAGCCC-1	doublet	45323	-1724.375425	-1125.482607	-1101.948799	Control1
CAAAGAACAAACTAGA-1	singlet	1	-2328.59535	-680.2262333	-1942.884224	Control1
CAAAGAACAACTGATC-1	singlet	2	-2452.76214	-2054.024161	-912.8246995	Control1
CAAAGAACAAGTGCTT-1	singlet	2	-1089.772076	-1030.087062	-476.5113277	Control1
CAAAGAAGTCCCTCAT-1	singlet	0	-1659.994233	-5249.607122	-5969.304437	Control1
CAAAGAAGTGTCATGT-1	singlet	2	-1049.391149	-906.0184262	-369.1374732	Control1
CAAAGAAGTTATCTTC-1	singlet	2	-581.5626346	-504.3867805	-247.2246211	Control1
CAAAGAATCATCTATC-1	singlet	2	-2698.059318	-3186.423707	-1042.193987	Control1
CAACAACAGAACGTGC-1	doublet	45323	-214.2519782	-156.3146856	-128.2874101	Control1
CAACAACAGCCGTTAT-1	singlet	1	-2982.229654	-871.6388957	-3101.05879	Control1
CAACAACCACAGCTTA-1	singlet	2	-1382.028117	-1224.789091	-602.3839199	Control1
CAACAACCACCGGAAA-1	singlet	1	-2042.059958	-847.639645	-1839.16827	Control1
CAACAACCAGTTCCAA-1	singlet	2	-446.9986098	-343.4780531	-149.5563926	Control1
CAACAACCATTGAAAG-1	singlet	2	-1699.207193	-1417.520989	-656.6760902	Control1
CAACAACGTACAAGTA-1	singlet	1	-1707.958015	-758.7025959	-1686.018045	Control1
CAACAACTCACCTGGG-1	singlet	2	-190.953336	-142.3563956	-55.97841707	Control1
CAACAACTCCCTCAAC-1	singlet	2	-3157.916274	-2822.124784	-1176.002975	Control1
CAACAGTAGCCTATCA-1	singlet	1	-789.3728129	-285.540053	-664.5250136	Control1
CAACAGTAGTGTACCT-1	singlet	0	-1128.950565	-3306.305136	-3576.2237	Control1
CAACAGTCACAGCATT-1	singlet	1	-1128.832804	-443.9729206	-1084.550718	Control1
CAACAGTCACTTCCTG-1	singlet	2	-1812.9879	-1693.72123	-736.5829128	Control1
CAACAGTCATTGACCA-1	singlet	1	-2579.846479	-1031.593498	-2586.41839	Control1
CAACAGTGTCACAGTT-1	singlet	1	-6791.656587	-2366.301021	-6197.130726	Control1
CAACAGTGTCGCACAC-1	singlet	2	-659.1873406	-522.6432833	-252.0925496	Control1
CAACAGTGTTTGCAGT-1	singlet	1	-1600.260607	-569.8703419	-1361.543836	Control1
CAACAGTTCATCACCC-1	singlet	1	-1628.104536	-751.1096529	-1478.909754	Control1
CAACAGTTCCCTCGAT-1	singlet	1	-291.0175802	-93.82104618	-231.4296547	Control1
CAACAGTTCTAGCCAA-1	singlet	2	-1562.21115	-1533.145378	-722.6025886	Control1
CAACCAAAGACTAAGT-1	singlet	2	-3977.968249	-3533.006325	-1399.873076	Control1
CAACCAAAGGGAGTTC-1	singlet	2	-1408.566388	-1281.88015	-487.8060138	Control1
CAACCAAGTCTGCATA-1	singlet	2	-2977.326319	-2476.229408	-1072.043176	Control1
CAACCAAGTGCGCTCA-1	singlet	2	-1215.830464	-2460.219044	-414.8212067	Control1
CAACCAATCGTGACTA-1	singlet	1	-206.2764852	-36.68454421	-57.82564734	Control1
CAACCTCAGAGCCATG-1	singlet	0	-1046.809144	-2861.901567	-3211.16305	Control1
CAACCTCAGCAATTCC-1	singlet	2	-654.2648236	-551.3456964	-233.0950686	Control1
CAACCTCCACCTGAAT-1	singlet	2	-1333.550582	-1306.735107	-530.5981203	Control1
CAACCTCCATGACCCG-1	singlet	2	-483.6463814	-341.1311974	-192.1991438	Control1
CAACCTCGTTCTCCTG-1	singlet	2	-3499.935299	-3109.367083	-1100.610506	Control1
CAACCTCTCCATAGGT-1	singlet	2	-1823.813705	-1525.856327	-652.4829125	Control1
CAACCTCTCGAGCTGC-1	singlet	0	-1494.852612	-4273.342944	-4579.061581	Control1
CAACCTCTCGGATACT-1	singlet	2	-65.57827322	-68.16600362	-20.03295274	Control1
CAACCTCTCTGCTTAT-1	singlet	1	-5422.056525	-2203.756051	-5026.128491	Control1
CAACGATGTTGTTTGG-1	singlet	2	-368.7486653	-266.137396	-141.5981096	Control1
CAACGATTCAGCTTCC-1	singlet	2	-975.1945223	-636.9519684	-271.3016209	Control1
CAACGATTCAGGAGAC-1	singlet	1	-683.1263922	-270.0759813	-650.0186636	Control1
CAACGATTCGCCAATA-1	doublet	45323	-97.36712666	-40.60771501	-37.19402672	Control1
CAACGATTCGGAGTAG-1	doublet	45293	-3317.942925	-1929.554838	-2073.949825	Control1
CAACGATTCTCCCATG-1	singlet	2	-121.3268406	-100.1351346	-47.66822476	Control1
CAACGGCAGCCGGAAT-1	singlet	1	-931.2249572	-363.3895317	-830.1588191	Control1
CAACGGCAGCGCTGAA-1	doublet	45293	-862.2231572	-634.60821	-636.7198986	Control1
CAACGGCCACCGGCTA-1	singlet	0	-701.8238953	-1882.362776	-2018.416274	Control1
CAACGGCCACTGGCGT-1	singlet	2	-790.1091308	-677.4243107	-304.0064911	Control1
CAACGGCGTTAAGGGC-1	singlet	1	-1271.918918	-481.1356404	-1089.217081	Control1
CAACGGCTCAGACTGT-1	singlet	2	-1082.181219	-1273.278098	-450.2230121	Control1
CAACGGCTCAGGTGTT-1	singlet	1	-3200.315726	-592.0447561	-1649.666362	Control1
CAACGGCTCCTACAAG-1	singlet	2	-1206.941641	-989.1598908	-519.4998527	Control1
CAACGGCTCTGCATAG-1	doublet	45323	-244.1597073	-314.2676201	-119.2445941	Control1
CAAGACTCAAGCACAG-1	singlet	1	-2453.129057	-820.5908094	-2299.243633	Control1
CAAGACTCACTATCGA-1	singlet	2	-641.5007074	-481.3010836	-248.7467304	Control1
CAAGACTTCGGTAGAG-1	singlet	2	-1987.297167	-1790.317654	-677.0168834	Control1
CAAGACTTCGTGGCTG-1	singlet	2	-984.4949579	-893.7515204	-387.033106	Control1
CAAGAGGAGGCTAGCA-1	singlet	2	-707.178216	-931.5100563	-263.4532368	Control1
CAAGAGGAGGTCCCTG-1	singlet	2	-573.5326917	-436.7945878	-192.5591783	Control1
CAAGAGGCACAAATGA-1	singlet	2	-10973.8258	-9038.561566	-2632.716276	Control1
CAAGAGGCAGATAAAC-1	singlet	0	-1726.966984	-5273.113839	-5572.014606	Control1
CAAGAGGCATCATTGG-1	singlet	2	-344.2106558	-295.9563411	-116.017909	Control1
CAAGAGGGTCATCAGT-1	singlet	1	-1033.361229	-414.6371005	-1022.329639	Control1
CAAGAGGGTGATATAG-1	singlet	1	-2343.345876	-700.9575919	-1890.684528	Control1
CAAGAGGTCATTGAGC-1	singlet	2	-677.0525722	-503.1239441	-230.6895043	Control1
CAAGCTAAGTCCTGCG-1	singlet	1	-1096.411974	-453.9364308	-1101.657999	Control1
CAAGCTACACCGGTCA-1	singlet	0	-139.8488022	-497.9958527	-571.3508473	Control1
CAAGCTAGTACCTTCC-1	singlet	0	-1001.488107	-3233.165747	-3501.222948	Control1
CAAGCTAGTATCGCAT-1	singlet	1	-1877.216063	-553.3855378	-1432.770051	Control1
CAAGCTAGTCGGCTAC-1	singlet	2	-1669.700292	-1579.809119	-526.0736199	Control1

CAAGCTATCCACAGCG-1	singlet	2	-2696.37071	-2355.383472	-919.7781237	Control1
CAAGCTATCCTACGAA-1	singlet	2	-653.0766387	-468.2649974	-197.0345788	Control1
CAAGCTATCGCCTCTA-1	singlet	2	-4375.255338	-4615.693273	-1574.359366	Control1
CAAGCTATCGTTAGTG-1	singlet	2	-1561.746243	-1357.736695	-521.2065934	Control1
CAAGGGAAGACGACGT-1	singlet	2	-1832.352644	-1733.612709	-711.3269209	Control1
CAAGGGAAGCCGTTGC-1	singlet	1	-1183.709067	-455.5864816	-1126.053367	Control1
CAAGGGAAGGAGTCTG-1	singlet	2	-1664.802662	-1291.474591	-543.8512912	Control1
CAAGGGAAGTCTTGGT-1	singlet	2	-4168.184915	-3224.158988	-1386.443917	Control1
CAAGGGACAAACCATC-1	singlet	1	-2802.119871	-965.3018099	-2664.423755	Control1
CAAGGGACATTCTTCA-1	singlet	0	-6562.341823	-20820.31702	-22849.5341	Control1
CAATACGAGCACTCTA-1	singlet	2	-4900.773087	-4101.449196	-1439.152435	Control1
CAATACGAGCGCATCC-1	singlet	2	-997.4053226	-941.8404912	-391.2578753	Control1
CAATACGCAATAACCC-1	singlet	2	-1837.735946	-1613.253359	-690.4425041	Control1
CAATACGCACGCTGAC-1	singlet	2	-586.5861559	-624.315537	-275.4514846	Control1
CAATACGCAGAAGCGT-1	singlet	2	-773.8053238	-746.5171447	-347.0498537	Control1
CAATACGCAGGGCTTC-1	singlet	2	-450.2460057	-337.8824462	-144.0259249	Control1
CAATACGGTACAAGTA-1	singlet	2	-504.5355486	-389.2709299	-176.6019485	Control1
CAATACGGTAGGATAT-1	singlet	1	-2764.181579	-877.4078163	-2591.919065	Control1
CAATACGGTGGCCACT-1	singlet	2	-1902.390687	-1811.182502	-717.9564867	Control1
CAATACGGTTCGTGCG-1	singlet	1	-650.0838935	-229.9394691	-564.1563804	Control1
CAATACGGTTCTGACA-1	singlet	2	-1457.175558	-1356.093668	-464.1267719	Control1
CAATACGTCAACGTGT-1	singlet	2	-1633.221132	-1463.76633	-515.4873628	Control1
CAATACGTCAATCCGA-1	singlet	2	-5200.399547	-4611.386498	-1801.870262	Control1
CAATACGTCATTCGTT-1	singlet	2	-713.3072027	-533.436306	-242.464414	Control1
CAATACGTCCACTTCG-1	singlet	2	-7040.214595	-6098.393648	-2462.853669	Control1
CAATCGAAGAAGCGCT-1	singlet	1	-1363.603109	-488.4695588	-1246.78034	Control1
CAATCGAAGACTCTTG-1	singlet	0	-1833.704851	-6411.951281	-6624.765366	Control1
CAATCGAAGCGCAATG-1	singlet	2	-323.8291948	-296.349759	-139.0099601	Control1
CAATCGAAGGTACTGG-1	singlet	2	-577.7992147	-484.1029483	-217.219227	Control1
CAATCGACAGAATTCC-1	singlet	2	-765.3132581	-640.0176437	-233.3130722	Control1
CAATCGACAGCTTCCT-1	singlet	2	-615.9898823	-468.307183	-215.9348165	Control1
CAATCGAGTAAGGAGA-1	singlet	2	-1387.987599	-1032.71243	-377.1830112	Control1
CAATCGAGTAGCGATG-1	singlet	2	-1615.950488	-1454.93469	-626.1408585	Control1
CAATCGAGTATCCCTC-1	singlet	2	-1486.900427	-1325.784233	-512.164589	Control1
CAATCGAGTTCATCGA-1	doublet	45323	-2036.346481	-1495.708552	-1018.547229	Control1
CAATCGATCCACGTGG-1	doublet	45293	-1100.345259	-658.5217533	-798.8494786	Control1
CAATCGATCCGTGTCT-1	singlet	2	-672.4775882	-577.591824	-262.0404675	Control1
CAATGACAGGATATGT-1	singlet	2	-1150.695953	-1285.613306	-520.6986168	Control1
CAATGACAGGTACATA-1	singlet	2	-730.8702147	-625.0419764	-300.3616921	Control1
CAATGACCATCCTGTC-1	doublet	1/0	-991.2903393	-661.4631833	-1217.182174	Control1
CAATGACGTATTTCCT-1	singlet	2	-5771.872683	-4932.063214	-2113.754253	Control1
CAATGACTCGAGCTGC-1	singlet	2	-295.9553131	-257.4418648	-106.4003729	Control1
CAATGACTCTGCGGGT-1	singlet	2	-5193.513898	-4537.865998	-1619.221577	Control1
CAATTTCAGCAAATCA-1	singlet	1	-440.9908496	-218.7548922	-498.0179993	Control1
CAATTTCAGCACTGGA-1	singlet	2	-1885.826169	-1695.523185	-662.3706185	Control1
CAATTTCCATACTGTG-1	singlet	0	-1052.527954	-2728.225653	-3081.144552	Control1
CAATTTCGTATCAAGA-1	singlet	2	-2584.375864	-2144.709565	-926.5722521	Control1
CAATTTCGTTGCCAAT-1	singlet	1	-1114.553146	-418.0648941	-982.8560974	Control1
CAATTTCTCACCTACC-1	singlet	0	-731.2500558	-1669.607219	-1989.711411	Control1
CAATTTCTCCTAGCGG-1	singlet	0	-858.2899026	-2187.209017	-2654.650935	Control1
CACAACAAGCTCCGAC-1	singlet	2	-798.5559228	-559.8839844	-257.6461025	Control1
CACAACAAGGACAACC-1	singlet	1	-1772.764414	-804.2316263	-1758.956182	Control1
CACAACAGTACAATAG-1	singlet	1	-1731.928918	-686.7186205	-1637.693204	Control1
CACAACAGTCCAAATC-1	singlet	2	-4966.79755	-4255.347031	-1807.035123	Control1
CACAACAGTCCAATCA-1	singlet	2	-1711.418792	-1544.027578	-678.2689612	Control1
CACAACAGTGTCCATA-1	singlet	2	-681.5340036	-523.6711254	-280.1710284	Control1
CACAACAGTGTGATGG-1	singlet	1	-1043.486868	-432.4036804	-1073.969074	Control1
CACAACAGTTGACGGA-1	singlet	1	-2473.397317	-846.0585835	-2132.152785	Control1
CACAACAGTTGCTTGA-1	singlet	0	-316.0948161	-696.494285	-1014.015976	Control1
CACAACATCCCAAGTA-1	singlet	1	-504.5303913	-261.556063	-571.4290392	Control1
CACAACATCCTATGGA-1	singlet	1	-1592.980092	-582.2149494	-1551.011949	Control1
CACAACATCGACATAC-1	singlet	2	-5027.223879	-4485.52981	-1634.045895	Control1
CACAACATCGGACCAC-1	singlet	0	-30.67144505	-72.8750829	-79.76028934	Control1
CACACAAAGTTGTCAC-1	singlet	1	-1047.440097	-399.525582	-1065.862649	Control1
CACACAAAGTTTCAGC-1	singlet	2	-632.4202874	-496.3667436	-229.922697	Control1
CACACAACAAGCTGTT-1	singlet	2	-1190.324698	-1106.157326	-576.1137292	Control1
CACACAACAGACCTAT-1	singlet	1	-558.2559444	-244.3366588	-570.1767786	Control1
CACACAAGTCTTGCGG-1	doublet	45323	-3091.061794	-2549.122232	-1718.14493	Control1
CACACAATCTATACGG-1	singlet	2	-1197.257626	-1050.416364	-443.2896343	Control1
CACAGATAGCACTAGG-1	singlet	2	-2686.990974	-2106.116428	-843.4641331	Control1
CACAGATAGTAGTCAA-1	singlet	2	-414.093278	-338.7958472	-173.9459967	Control1
CACAGATCATGTCTAG-1	singlet	2	-1068.650856	-1529.833958	-383.1004413	Control1
CACAGATGTACGATTC-1	singlet	1	-907.4614795	-335.9015663	-820.2833197	Control1
CACAGATGTAGCTTGT-1	singlet	0	-1601.36609	-4311.504725	-4604.621128	Control1
CACAGATGTATTGACC-1	singlet	0	-1739.951469	-4681.866328	-5290.580989	Control1
CACAGATGTCAATGGG-1	doublet	45323	-8614.659291	-5569.593974	-3904.11842	Control1
CACAGATGTCCTACGG-1	singlet	1	-2540.643202	-955.6516312	-2404.974907	Control1
CACAGATGTGCGAACA-1	singlet	2	-668.986732	-561.3223684	-228.0009114	Control1
CACAGATGTTCCGCTT-1	singlet	2	-978.1739869	-745.5323208	-355.3739782	Control1
CACAGATGTTTACGTG-1	singlet	2	-2436.438719	-2166.085151	-724.8138653	Control1
CACAGATTCGCTAATG-1	singlet	0	-370.8016124	-786.19838	-1106.277985	Control1
CACAGGCAGATGAACT-1	singlet	2	-8294.263236	-7350.596548	-2624.278774	Control1
CACAGGCAGGGCAACT-1	singlet	2	-1408.716792	-1542.664531	-583.6808493	Control1
CACAGGCAGGTACCTT-1	singlet	1	-1044.937645	-419.5076576	-1004.428338	Control1
CACAGGCCACCGCTGA-1	singlet	2	-1619.587515	-1427.861939	-682.7780181	Control1
CACAGGCCATTCTTCA-1	singlet	1	-1776.11904	-611.7748423	-2016.023792	Control1
CACAGGCGTTTGGCTA-1	singlet	1	-613.8003344	-240.1594376	-593.9398081	Control1
CACAGGCTCAAGCGTT-1	singlet	0	-187.3660017	-543.3852519	-771.8198991	Control1
CACAGGCTCTACTCAT-1	singlet	2	-1912.616952	-1766.063874	-716.4200392	Control1
CACAGGCTCTGACGCG-1	singlet	1	-745.0596837	-260.8761607	-754.9359863	Control1
CACATGAAGACGTCGA-1	unassigned	2	-14.96858711	-4.193886418	-3.651119527	Control1
CACATGAAGCCGAACA-1	singlet	1	-1082.269968	-442.3286911	-906.0422191	Control1
CACATGACACGCTGAC-1	singlet	2	-2346.0324	-1826.219642	-706.9016967	Control1
CACATGAGTAACATCC-1	singlet	2	-4119.856743	-3569.27208	-1442.186001	Control1
CACCAAAAGTCAGAGC-1	singlet	2	-532.0523882	-382.0985159	-197.9369625	Control1
CACCAAAAGTGAGTTA-1	singlet	2	-286.5271948	-244.3578257	-115.5829093	Control1
CACCAAAAGTTGAAGT-1	singlet	0	-628.8507966	-1598.637503	-1838.684463	Control1
CACCAAACAACCCTAA-1	singlet	2	-1718.550237	-1829.898944	-691.370479	Control1
CACCAAACAAGGATGC-1	singlet	1	-1303.595167	-498.7724183	-1272.551293	Control1
CACCAAACACTTGACA-1	singlet	2	-1498.860526	-1263.481038	-553.5177134	Control1
CACCAAACAGACCTGC-1	singlet	2	-671.4184809	-603.9838482	-230.9488129	Control1
CACCAAAGTATGCGGA-1	singlet	1	-833.8040733	-263.0770412	-729.908747	Control1
CACCAAAGTATGGAGC-1	singlet	1	-1818.731094	-605.1427928	-1497.432406	Control1
CACCAAAGTGAGACCA-1	singlet	2	-433.5842439	-455.1586581	-189.1935663	Control1
CACCAAAGTGGTCTGC-1	singlet	2	-264.6043603	-247.0915225	-137.6952815	Control1
CACCAAATCATCGACA-1	singlet	1	-2482.130647	-1013.055468	-2429.353966	Control1
CACCGTTAGACTCTAC-1	singlet	2	-1805.168047	-1618.476037	-744.922335	Control1
CACCGTTAGTACAGCG-1	singlet	1	-1031.546174	-379.1428925	-925.0453244	Control1
CACCGTTAGTGCACAG-1	singlet	1	-843.342696	-323.6108627	-771.513609	Control1
CACCGTTCACTTGGGC-1	unassigned	0	-1.457062587	-5.388847557	-3.006223675	Control1

CACCGTTCAGCGTACC-1	singlet	0	-241.0727626	-646.1855186	-787.1079115	Control1
CACCGTTGTAACAGTA-1	singlet	1	-2947.148952	-1068.719382	-2884.552498	Control1
CACCGTTGTCACAGAG-1	singlet	2	-1949.455789	-1705.889311	-647.529635	Control1
CACCGTTGTTGCGGAA-1	singlet	2	-2411.913132	-2124.728275	-991.5494925	Control1
CACCGTTTCCGTTGAA-1	singlet	1	-1327.008204	-545.2992039	-1198.903653	Control1
CACCGTTTCGTGTGAT-1	singlet	1	-909.6778345	-358.0712107	-926.516721	Control1
CACCGTTTCTCATGCC-1	unassigned	1	-7.857535046	-6.373222198	-11.00405812	Control1
CACGAATAGTCGCGAA-1	singlet	2	-2127.265082	-1945.966525	-822.8811722	Control1
CACGAATCAGTTGCGC-1	singlet	2	-3827.623001	-3135.779716	-1315.404858	Control1
CACGGGTAGCAAATCA-1	singlet	2	-474.0219088	-403.8137661	-188.8925953	Control1
CACGGGTAGCCGAACA-1	singlet	2	-548.5429531	-477.3531852	-249.8145219	Control1
CACGGGTAGTCCCAAT-1	singlet	2	-1989.190931	-1767.417646	-705.1173942	Control1
CACGGGTAGTCGTTAC-1	singlet	1	-570.0952891	-209.8564982	-580.8407946	Control1
CACGGGTGTGTAGGAC-1	unassigned	2	-9.399920381	-1.724569566	-1.035756548	Control1
CACGGGTGTTAGAGTA-1	singlet	2	-1404.88907	-1189.833863	-470.9858059	Control1
CACGGGTGTTGCACGC-1	singlet	1	-1050.986871	-430.8170334	-1039.463254	Control1
CACGGGTTCCATTTGT-1	singlet	2	-1155.710337	-1062.907062	-467.3615848	Control1
CACGGGTTCCTGTTAT-1	singlet	2	-1819.429477	-1633.002011	-720.6412627	Control1
CACGGGTTCTGGACCG-1	singlet	2	-1511.005578	-1333.480852	-512.4425467	Control1
CACGTGGCAACAGAGC-1	singlet	1	-1232.801085	-468.0033331	-1141.300996	Control1
CACGTGGCACAGAAGC-1	singlet	2	-689.4167368	-530.9028329	-263.5462214	Control1
CACGTGGCAGTGTATC-1	singlet	2	-797.7218484	-674.9052681	-282.322883	Control1
CACGTGGCAGTTGGTT-1	singlet	1	-522.5687683	-223.0442715	-561.2140176	Control1
CACGTGGGTCCTGGGT-1	singlet	1	-2501.770991	-851.8939656	-2254.332242	Control1
CACGTGGGTGTCCACG-1	singlet	2	-1201.840347	-1119.333103	-457.7777161	Control1
CACGTGGTCAGAGTGG-1	singlet	2	-605.9347083	-458.6514063	-224.7223615	Control1
CACGTGGTCGATTGGT-1	singlet	0	-26.22931719	-94.66992035	-105.2528428	Control1
CACGTTCAGATTGAGT-1	singlet	1	-1396.748564	-479.4327933	-1306.669861	Control1
CACGTTCCACGTACTA-1	singlet	0	-1899.045775	-5591.598436	-6211.314805	Control1
CACGTTCCAGGCATTT-1	singlet	2	-853.712095	-757.4427494	-339.0255716	Control1
CACGTTCGTAGAAACT-1	singlet	2	-669.6838846	-493.0601202	-184.1764763	Control1
CACGTTCGTGATACTC-1	unassigned	2/0	-18.53868763	-22.69124006	-8.889235952	Control1
CACGTTCGTTCCTAAG-1	singlet	2	-783.9079769	-1049.494663	-335.7953464	Control1
CACTAAGAGGGCTGAT-1	singlet	2	-674.7234952	-583.539304	-204.8867544	Control1
CACTAAGAGTAACCGG-1	singlet	2	-645.1660667	-533.0742576	-217.2886328	Control1
CACTAAGAGTTCGCAT-1	singlet	2	-8101.684383	-7046.308564	-2179.168966	Control1
CACTAAGCAACCGACC-1	singlet	0	-1581.365456	-4332.586873	-4861.768407	Control1
CACTAAGGTACTCAAC-1	singlet	1	-2968.88375	-1133.349692	-2685.983981	Control1
CACTAAGGTGAGGAAA-1	singlet	2	-5113.875935	-4845.81008	-1474.754887	Control1
CACTAAGTCAACGCTA-1	singlet	2	-1334.145881	-1348.799884	-514.349438	Control1
CACTAAGTCCGGTTCT-1	singlet	2	-790.8477197	-589.3251916	-275.4608851	Control1
CACTGAACACGCCACA-1	singlet	2	-680.6684588	-511.1847427	-236.4036155	Control1
CACTGAACACTGCGAC-1	singlet	1	-361.6996756	-42.07083673	-117.4687689	Control1
CACTGAACATGGCGCT-1	singlet	1	-3618.399385	-1159.947206	-3492.331203	Control1
CACTGAAGTATCGAGG-1	unassigned	2	-3.590884079	-3.098108386	-2.401792531	Control1
CACTGAAGTATTGCCA-1	singlet	1	-1455.516501	-583.2169203	-1362.441773	Control1
CACTGAAGTTCAGCTA-1	singlet	2	-758.7668132	-601.3751672	-244.8470057	Control1
CACTGAATCGCGTCGA-1	singlet	2	-268.1086923	-258.5108821	-85.14680208	Control1
CACTGAATCTCATTAC-1	singlet	1	-3388.53017	-1328.766742	-3238.551002	Control1
CACTGAATCTGCTCTG-1	singlet	2	-1258.160544	-1101.816775	-475.6989492	Control1
CACTGGGAGAGGGTCT-1	singlet	2	-814.3067966	-558.3030729	-266.1775193	Control1
CACTGGGAGGTACAAT-1	singlet	2	-1427.359387	-1282.410647	-569.107144	Control1
CACTGGGAGGTGCCTC-1	singlet	1	-922.9304633	-383.9243734	-818.2536399	Control1
CACTGGGGTAGGTACG-1	singlet	2	-1343.280736	-1170.226506	-478.8856292	Control1
CACTGGGTCAAAGGTA-1	singlet	2	-961.9847927	-791.7336279	-395.8556029	Control1
CACTGGGTCAATCGGT-1	singlet	1	-2511.796306	-853.6249289	-2039.953231	Control1
CACTGGGTCCCGAGGT-1	singlet	2	-2945.81201	-2696.292815	-1051.438724	Control1
CACTGGGTCGGAACTT-1	singlet	2	-2728.021785	-2316.976757	-866.089586	Control1
CACTGTCAGAGGGTGG-1	singlet	1	-3222.437971	-1092.566346	-2912.293403	Control1
CACTGTCAGTACGAGC-1	singlet	2	-678.4207196	-506.3553635	-288.0360579	Control1
CACTGTCAGTAGGCCA-1	singlet	2	-4435.879834	-3678.367412	-1382.06271	Control1
CACTGTCCACCACATA-1	singlet	2	-2757.721951	-3020.102559	-978.2898932	Control1
CACTGTCCACTGTCGG-1	singlet	0	-319.7331464	-851.1039082	-1065.101557	Control1
CACTGTCCACTTGTGA-1	singlet	1	-143.3148913	-80.20131552	-188.6943967	Control1
CACTGTCCATCGATAC-1	singlet	2	-252.5384273	-239.1523682	-83.41302938	Control1
CACTGTCGTAGTCTTG-1	singlet	2	-538.4031138	-386.3380184	-175.0582224	Control1
CACTGTCGTCGTTGCG-1	singlet	2	-1632.211412	-1470.179145	-593.3682343	Control1
CACTGTCGTGGTCCCA-1	doublet	45323	-1339.711672	-1268.023435	-793.8109547	Control1
CACTGTCGTTAAGCAA-1	singlet	0	-380.7162541	-986.7800509	-1038.332166	Control1
CACTGTCGTTAAGGGC-1	singlet	1	-1985.369681	-768.388433	-1698.64444	Control1
CACTGTCTCGCCGATG-1	singlet	1	-1109.549579	-457.3255717	-1033.010108	Control1
CACTGTCTCGGATAAA-1	singlet	2	-489.3460704	-409.6392299	-206.2054399	Control1
CACTTCGAGAGTGGCT-1	singlet	2	-737.243726	-709.1039721	-353.5731758	Control1
CACTTCGAGCGACCCT-1	singlet	2	-1764.689527	-1642.018597	-571.5316575	Control1
CACTTCGAGGACACTG-1	singlet	1	-1276.411741	-458.1081177	-1047.165545	Control1
CACTTCGAGTAAAGCT-1	singlet	2	-863.0396746	-1053.816473	-387.3249936	Control1
CACTTCGCAGCAGACA-1	singlet	2	-1154.628141	-1111.372495	-416.9087163	Control1
CACTTCGCAGTGCCTG-1	singlet	1	-6425.194735	-2435.234689	-6250.707105	Control1
CACTTCGCATTGTAGC-1	singlet	2	-500.3200553	-457.5048604	-195.6671351	Control1
CACTTCGGTACCATAC-1	singlet	2	-806.396991	-629.1219613	-312.8119532	Control1
CACTTCGGTTTCCAAG-1	singlet	1	-904.3455069	-373.578739	-875.4901718	Control1
CACTTCGTCACTACTT-1	singlet	2	-548.5073334	-415.28554	-201.5498391	Control1
CACTTCGTCAGTCCGG-1	singlet	1	-987.6622466	-360.9723725	-1002.514423	Control1
CACTTCGTCATCGGGC-1	singlet	1	-3585.772928	-1262.808573	-3573.817186	Control1
CACTTCGTCTCGAACA-1	singlet	2	-746.1179743	-555.5254477	-229.8244947	Control1
CAGAGCCAGATAACAC-1	singlet	2	-2044.200079	-1594.639226	-677.4990033	Control1
CAGAGCCAGGTACCTT-1	singlet	2	-5505.41383	-4954.928701	-1674.926476	Control1
CAGAGCCAGTCATGAA-1	singlet	1	-879.3946522	-350.4671961	-847.2024382	Control1
CAGAGCCCAACGGCCT-1	unassigned	45323	-7.674249264	-9.127113595	-8.01425981	Control1
CAGAGCCCAGTGTATC-1	singlet	2	-872.038079	-998.0423763	-353.2860201	Control1
CAGAGCCCATCCGTGG-1	singlet	1	-895.7409247	-370.2587084	-821.6926147	Control1
CAGAGCCGTGCGAGTA-1	singlet	2	-360.6481087	-275.1443159	-119.3106496	Control1
CAGAGCCGTTGTACGT-1	singlet	2	-498.3625094	-409.4519591	-199.920226	Control1
CAGATACAGGCACTAG-1	singlet	2	-2062.377963	-1857.97365	-737.3139165	Control1
CAGATACAGGTGCTGA-1	doublet	2/0	-912.5258476	-867.230663	-487.4586401	Control1
CAGATACCAAGGACAC-1	singlet	2	-249.1403668	-193.4934613	-92.72641146	Control1
CAGATACCACCGTGGT-1	singlet	1	-376.1286097	-134.5522213	-371.1635166	Control1
CAGATACGTCGCGTTG-1	singlet	2	-669.7926163	-923.4816783	-279.3463353	Control1
CAGATACTCGACCACG-1	singlet	2	-1493.424666	-1292.334571	-522.0695145	Control1
CAGATCAAGACTAGAT-1	singlet	2	-790.583886	-672.7520701	-301.5105654	Control1
CAGATCAAGAGTGGCT-1	singlet	2	-428.5785126	-259.7227546	-127.1134696	Control1
CAGATCAAGATCACCT-1	singlet	1	-132.2166935	-70.691472	-137.6532769	Control1
CAGATCAAGGCGTTAG-1	singlet	2	-296.4373723	-176.8391281	-80.12914516	Control1
CAGATCACAAAGGGTC-1	singlet	0	-295.5272652	-882.5893731	-1107.546222	Control1
CAGATCACAACAGATA-1	singlet	2	-3725.516084	-3075.188629	-1297.326895	Control1
CAGATCAGTAACCAGG-1	singlet	1	-992.373818	-433.8463046	-1072.803317	Control1
CAGATCAGTAGCACAG-1	singlet	2	-3768.487731	-3335.834131	-1163.908875	Control1
CAGATCAGTAGCTAAA-1	singlet	2	-520.4596038	-443.0393671	-243.228239	Control1
CAGATCAGTCGCGGTT-1	singlet	1	-165.1380269	-27.0877694	-48.92168622	Control1
CAGATCATCGCGAAGA-1	singlet	1	-710.0243755	-311.7146043	-650.8946144	Control1

CAGATTGAGTAAACTG-1	unassigned	45323	-110.3743956	-76.53543218	-32.45927547	Control1
CAGATTGCACCATATG-1	singlet	2	-560.9928098	-429.7561949	-170.4567585	Control1
CAGATTGGTCGTTATG-1	singlet	2	-490.8740942	-395.7919444	-225.6182678	Control1
CAGATTGGTGCCTTTC-1	singlet	2	-712.654153	-742.5776242	-382.7128787	Control1
CAGATTGTCCACTTCG-1	singlet	2	-412.6262788	-266.2068709	-141.6877825	Control1
CAGATTGTCCATCTAT-1	singlet	1	-1440.126397	-658.7623907	-1329.208499	Control1
CAGATTGTCGAGAAGC-1	singlet	1	-639.6108175	-254.6609158	-631.3078373	Control1
CAGATTGTCGTGACTA-1	singlet	1	-192.6226331	-95.36952184	-188.8289271	Control1
CAGATTGTCTATTTCG-1	singlet	2	-879.6552523	-554.0876159	-272.011524	Control1
CAGCAATAGGTAATCA-1	singlet	2	-671.9713362	-496.2123984	-252.5426876	Control1
CAGCAATAGTATGAGT-1	singlet	2	-639.6388131	-486.27104	-224.5507154	Control1
CAGCAATCACAAGTTC-1	singlet	2	-546.1439716	-370.4883699	-189.1179632	Control1
CAGCAATGTAACAAGT-1	singlet	2	-2224.163576	-2117.297729	-1014.546413	Control1
CAGCAATGTCCACATA-1	singlet	2	-838.6731635	-552.7426467	-227.3817942	Control1
CAGCAATGTGCAACGA-1	singlet	2	-1632.258174	-1501.015242	-664.768255	Control1
CAGCAATGTTACGATC-1	singlet	2	-1008.191585	-863.9858532	-321.5477526	Control1
CAGCAATTCGATACTG-1	singlet	2	-858.2306922	-754.7676985	-312.4576662	Control1
CAGCACGAGGTCACTT-1	singlet	2	-1661.130315	-1674.733816	-665.340516	Control1
CAGCACGCATACCACA-1	singlet	1	-926.1589816	-380.8923679	-885.723487	Control1
CAGCACGGTAACAAGT-1	singlet	2	-784.0730363	-582.7123031	-264.1882127	Control1
CAGCACGGTAGGAGTC-1	singlet	0	-178.1847075	-484.4849207	-537.5083408	Control1
CAGCACGTCGCCGTGA-1	unassigned	1	-1.19302417	-0.931203322	-4.036447165	Control1
CAGCAGCAGAGTCACG-1	singlet	2	-6257.49431	-5410.134746	-1793.352188	Control1
CAGCAGCAGCGTGAGT-1	singlet	2	-609.8601302	-495.6118597	-259.7637441	Control1
CAGCAGCAGGCTTAGG-1	singlet	2	-302.8900729	-311.0559431	-112.1239147	Control1
CAGCAGCAGTGCAACG-1	singlet	1	-445.0816619	-180.1533168	-475.2408428	Control1
CAGCAGCCAACGGGTA-1	singlet	1	-1746.675626	-595.3123434	-1723.677653	Control1
CAGCAGCCACATAACC-1	singlet	2	-1582.188847	-1482.57367	-675.1745355	Control1
CAGCAGCCAGATTTCG-1	singlet	2	-522.7727666	-412.8544316	-196.9578055	Control1
CAGCAGCCAGCTGTGC-1	singlet	2	-3749.589172	-3145.552333	-1177.998514	Control1
CAGCAGCGTCAGACGA-1	singlet	2	-473.2448574	-383.3500677	-189.0080441	Control1
CAGCAGCGTTCAAGTC-1	singlet	1	-757.0604198	-264.8931369	-668.5402342	Control1
CAGCAGCTCGACCAAT-1	singlet	2	-840.5580719	-794.8794842	-331.0054613	Control1
CAGCAGCTCTGTGCAA-1	singlet	1	-961.3794002	-499.3640704	-782.6862656	Control1
CAGCCAGAGTAACGTA-1	singlet	2	-4234.230244	-3697.049899	-1382.608068	Control1
CAGCCAGCAACTGAAA-1	singlet	1	-1537.146772	-663.675952	-1546.466258	Control1
CAGCCAGGTACTCGAT-1	singlet	2	-1690.23699	-1625.996781	-652.3528969	Control1
CAGCCAGGTATTCCTT-1	singlet	1	-906.048143	-340.9134081	-873.2436469	Control1
CAGCCAGGTTAGTCGT-1	singlet	1	-1252.129727	-521.0332688	-1183.717796	Control1
CAGCCAGGTTTCGTGA-1	singlet	2	-9549.871472	-8118.258343	-3079.252623	Control1
CAGCCAGTCGACATCA-1	singlet	2	-365.7487168	-245.2840652	-136.7724869	Control1
CAGCGTGAGACATGCG-1	singlet	2	-1588.282358	-1526.462182	-586.2673875	Control1
CAGCGTGAGACGGTTG-1	singlet	2	-1173.118783	-987.1054894	-445.188466	Control1
CAGCGTGAGGTTCACT-1	singlet	2	-742.0358987	-365.3611193	-182.9720513	Control1
CAGCGTGCAAGAGTTA-1	unassigned	0/2	-9.963688841	-10.21947626	-11.33760011	Control1
CAGCGTGCACAGTCCG-1	singlet	1	-397.6016552	-148.2164424	-349.5623516	Control1
CAGCGTGCAGAAACCG-1	singlet	2	-753.1309422	-660.3723056	-275.8328478	Control1
CAGCGTGCAGGTTCCG-1	singlet	0	-260.3812596	-663.2770841	-813.5118556	Control1
CAGCGTGGTATCAAGA-1	singlet	2	-1301.645117	-1236.437342	-474.8707296	Control1
CAGCGTGTCCAGTACA-1	singlet	2	-231.7106583	-256.3557249	-95.3469513	Control1
CAGCGTGTCCGGACTG-1	singlet	0	-625.1312503	-1873.939483	-1996.636945	Control1
CAGCGTGTCTGGAAGG-1	singlet	2	-1748.428625	-1701.557007	-639.7777141	Control1
CAGGCCAAGGTTTACC-1	singlet	2	-99.65693389	-82.52773075	-45.49995992	Control1
CAGGCCAAGTACGTCT-1	singlet	2	-520.7090718	-417.4191843	-194.3393194	Control1
CAGGCCACAATGACCT-1	singlet	2	-552.6482534	-431.2010797	-224.2584031	Control1
CAGGCCACACCGTGGT-1	singlet	2	-1842.348303	-1586.5757	-714.8790735	Control1
CAGGCCAGTAGATCGG-1	singlet	2	-462.3976735	-342.0229904	-177.5139902	Control1
CAGGCCAGTAGGGTAC-1	singlet	1	-1023.844049	-355.685928	-1033.883889	Control1
CAGGCCATCCATTCAT-1	singlet	2	-802.1546248	-554.4783617	-224.8813225	Control1
CAGGCCATCTAGTTCT-1	singlet	0	-102.8619928	-285.913706	-377.2414124	Control1
CAGGCCATCTTCCCGA-1	singlet	2	-4119.228308	-3716.315699	-1065.035589	Control1
CAGGGCTAGTCAATCC-1	singlet	2	-1217.945538	-1055.939285	-477.1932726	Control1
CAGGGCTAGTGCAACG-1	singlet	2	-1674.302406	-1469.883913	-564.406995	Control1
CAGGGCTAGTTCTACG-1	singlet	2	-473.885135	-349.6315326	-190.0420558	Control1
CAGGGCTCACACCTTC-1	singlet	0	-238.6271767	-541.2155975	-654.7536779	Control1
CAGGGCTCACCGGCTA-1	singlet	1	-1614.784069	-681.7448595	-1770.80182	Control1
CAGGGCTCAGCTGTTA-1	singlet	2	-224.5087065	-170.8751181	-84.56486658	Control1
CAGGGCTCATGACAAA-1	singlet	2	-984.1890925	-1181.7762	-403.1528005	Control1
CAGGGCTGTATAATGG-1	singlet	1	-1452.750738	-597.5239844	-1394.968078	Control1
CAGGGCTGTTGGTGTT-1	singlet	1	-1108.47081	-484.8306356	-1028.324599	Control1
CAGGGCTTCAACTCTT-1	singlet	2	-551.2408757	-449.9514792	-217.8733334	Control1
CAGGGCTTCGAGATGG-1	singlet	2	-948.6144909	-938.255493	-373.0299017	Control1
CAGGGCTTCGTTCTAT-1	singlet	2	-1654.16491	-1313.614627	-494.1522283	Control1
CAGGTATAGAATTGCA-1	singlet	2	-4169.1097	-3586.124022	-1470.722993	Control1
CAGGTATCATAGTCGT-1	singlet	2	-291.3700659	-203.864186	-114.5472183	Control1
CAGGTATGTAACACGG-1	singlet	2	-2404.126058	-2244.625366	-877.7866309	Control1
CAGGTATGTTAAGACA-1	singlet	1	-1389.335605	-537.9177652	-1395.365585	Control1
CAGGTATTCGCAGTCG-1	doublet	1/0	-15.18590457	-8.647516864	-11.73780043	Control1
CAGGTATTCGCTGCGA-1	singlet	2	-828.4997223	-806.4402519	-356.0558131	Control1
CAGTGCGAGCACTCGC-1	singlet	1	-3836.756737	-1266.11256	-3228.45943	Control1
CAGTGCGAGCGGTAGT-1	singlet	2	-614.4153842	-461.6433442	-256.2623893	Control1
CAGTGCGAGTTTCAGC-1	singlet	1	-4494.084447	-1205.023497	-4022.741655	Control1
CAGTGCGGTACCGGCT-1	singlet	0	-1643.384163	-5263.781068	-5542.432949	Control1
CAGTGCGGTTGGTAGG-1	singlet	2	-1895.037239	-1778.013778	-751.7444204	Control1
CAGTTAGAGATACATG-1	singlet	1	-6198.863738	-1701.669368	-6044.922524	Control1
CAGTTAGAGATGATTG-1	singlet	2	-11694.6924	-10050.87434	-3607.854412	Control1
CAGTTAGAGCAGTAAT-1	singlet	1	-946.2515528	-429.833981	-908.349838	Control1
CAGTTAGCAACGAGGT-1	singlet	1	-6602.14535	-2349.271342	-5944.198823	Control1
CAGTTAGCAACGTATC-1	singlet	2	-614.287461	-446.4405798	-198.5067173	Control1
CAGTTAGCAGACGCTC-1	singlet	2	-796.0280327	-567.6694909	-269.7789833	Control1
CAGTTAGGTAACATGA-1	singlet	2	-885.0459075	-826.1533603	-323.1335545	Control1
CAGTTAGGTAAGCAAT-1	singlet	1	-929.9094107	-384.5127433	-878.0801995	Control1
CAGTTAGGTAGTGTGG-1	singlet	2	-568.6558781	-491.9542989	-196.315816	Control1
CAGTTAGGTCAATGGG-1	singlet	1	-2571.105821	-827.8452536	-2197.499157	Control1
CAGTTAGGTCTAGGTT-1	singlet	2	-537.4937265	-411.1573818	-184.285301	Control1
CAGTTAGTCCACCTCA-1	singlet	2	-4091.814135	-3482.838012	-1384.018387	Control1
CAGTTAGTCCTTACCG-1	singlet	0	-805.0704484	-1810.052536	-1774.683165	Control1
CAGTTAGTCTCGGCTT-1	singlet	2	-683.3683885	-503.8499898	-284.632328	Control1
CAGTTCCAGTGAACAT-1	singlet	2	-656.0517545	-560.0300421	-206.8300772	Control1
CAGTTCCCACTCAGAT-1	singlet	2	-708.7938531	-516.2761465	-275.9521013	Control1
CAGTTCCCAGCATGCC-1	singlet	2	-1965.137853	-1863.311351	-772.2660394	Control1
CAGTTCCCAGGCACAA-1	singlet	2	-2358.885736	-2300.445782	-774.8535585	Control1
CAGTTCCCATATCTGG-1	singlet	2	-1545.686066	-1510.599118	-573.266999	Control1
CAGTTCCGTCTTGCTC-1	singlet	0	-1151.999269	-4485.487067	-3993.301513	Control1
CAGTTCCGTTTCGTTT-1	singlet	2	-949.84702	-853.015864	-360.9702361	Control1
CAGTTCCTCGATTTCT-1	singlet	2	-2454.501735	-2335.909837	-1134.488817	Control1
CAGTTCCTCGTAATGC-1	singlet	2	-1048.106961	-991.2414134	-355.3745871	Control1
CAGTTCCTCTTACGGA-1	singlet	2	-889.3418141	-780.2114879	-309.2356078	Control1
CATAAGCCACACGCCA-1	singlet	2	-701.9188072	-558.339848	-242.6183647	Control1
CATAAGCCACCATTCC-1	singlet	2	-825.7021527	-610.4810355	-264.2944269	Control1

CATAAGCCAGAAACCG-1	singlet	2	-648.3359238	-545.8349045	-265.8016171	Control1
CATAAGCGTAGCTTAC-1	singlet	1	-917.1784184	-331.7198111	-989.0351613	Control1
CATAAGCGTCACAGAG-1	singlet	2	-1777.908984	-1361.501964	-551.8879877	Control1
CATAAGCGTGAGTCAG-1	singlet	1	-10899.60528	-3007.613654	-10076.48402	Control1
CATAAGCTCGAGTACT-1	singlet	2	-441.4997334	-378.9348423	-173.1265932	Control1
CATACAGAGAACAGGA-1	doublet	45293	-1842.59394	-1166.371882	-1267.169659	Control1
CATACAGCAAGCACCC-1	singlet	2	-780.4541925	-619.2317298	-270.9660516	Control1
CATACAGCAATTGAGA-1	singlet	2	-2566.351374	-2384.135609	-833.9666269	Control1
CATACAGCAGGTGGAT-1	singlet	0	-1.505684736	-5.234183012	-7.067449282	Control1
CATACAGCAGTTAGGG-1	singlet	2	-2053.454207	-1750.877196	-727.8645865	Control1
CATACAGGTAGATGTA-1	singlet	1	-1335.165459	-454.6951834	-1256.194992	Control1
CATACAGGTCAACATC-1	doublet	45323	-4186.880841	-3196.411921	-2354.574732	Control1
CATACAGGTCTCGCGA-1	singlet	2	-983.1041035	-991.2133667	-388.9163766	Control1
CATACAGTCAAACTGC-1	singlet	2	-224.5896937	-204.775162	-91.81596604	Control1
CATACAGTCGAAATCC-1	singlet	2	-685.4298414	-541.3387906	-264.3463413	Control1
CATACAGTCTCGTGGG-1	singlet	2	-1256.047882	-1443.462972	-599.8060406	Control1
CATACCCAGCCTCTGG-1	singlet	1	-4796.847293	-1832.943184	-4497.954928	Control1
CATACCCGTGACATCT-1	singlet	2	-2018.851008	-1611.186	-660.2961657	Control1
CATACCCGTGCTATTG-1	singlet	2	-2847.110053	-2542.65023	-970.9596706	Control1
CATACCCGTTTACCAG-1	unassigned	45293	-34.74024789	-17.02670658	-19.65241506	Control1
CATACCCTCGACATAC-1	singlet	0	-112.1296304	-368.9929148	-366.4378776	Control1
CATACCCTCGACTCCT-1	singlet	2	-7467.233359	-6328.381757	-2419.813972	Control1
CATACCCTCTCGGGAC-1	singlet	2	-968.4984312	-856.969273	-358.7883806	Control1
CATACCCTCTGCATAG-1	singlet	1	-3850.989574	-1053.196627	-2597.224368	Control1
CATACTTAGCAAATGT-1	singlet	1	-721.8249047	-283.3806082	-655.6956322	Control1
CATACTTAGGGCATGT-1	singlet	1	-7983.876293	-2828.908734	-7458.846433	Control1
CATACTTCACTTCAAG-1	singlet	1	-11167.42684	-3751.152413	-10124.69584	Control1
CATACTTCAGAACCGA-1	singlet	2	-878.2213857	-715.5735843	-317.0780436	Control1
CATACTTGTCCGCAGT-1	singlet	2	-1797.258153	-1421.409656	-584.631931	Control1
CATACTTGTGGCTTAT-1	singlet	2	-669.788684	-523.648331	-260.0695778	Control1
CATACTTTCGAACGGA-1	singlet	2	-1504.526733	-1395.583158	-498.4666774	Control1
CATAGACAGAAACACT-1	singlet	1	-3953.927407	-1410.981708	-3424.412338	Control1
CATAGACAGAGTGTGC-1	singlet	2	-609.712953	-581.1627893	-249.5245566	Control1
CATAGACAGATGAACT-1	doublet	45323	-9334.734066	-10276.44541	-5776.937731	Control1
CATAGACAGCGAAACC-1	singlet	2	-517.0992008	-390.6799749	-188.3986657	Control1
CATAGACAGCTACTAC-1	singlet	1	-1697.527524	-668.5582988	-1734.283758	Control1
CATAGACAGGGACAGG-1	singlet	1	-2166.028156	-921.3487431	-2090.708374	Control1
CATAGACCATCCGATA-1	singlet	2	-1216.042133	-1364.713396	-524.3819179	Control1
CATAGACTCATTGTTC-1	singlet	1	-1666.638558	-700.7668449	-1435.742852	Control1
CATCAAGAGACATCCT-1	singlet	1	-1168.04326	-471.5574745	-1167.114686	Control1
CATCAAGAGGATCACG-1	singlet	0	-166.5434737	-489.3175127	-616.9080371	Control1
CATCAAGAGTTGAAAC-1	singlet	2	-3342.94728	-3653.279724	-1192.24737	Control1
CATCAAGCATGACCCG-1	singlet	2	-1142.545109	-895.139992	-458.2041886	Control1
CATCAAGGTGCCTACG-1	singlet	2	-1468.450074	-1318.482825	-581.7334122	Control1
CATCAAGGTGTGGTCC-1	singlet	2	-6237.068205	-5387.344025	-2028.658418	Control1
CATCAAGGTTGTACGT-1	singlet	2	-554.6272531	-501.9032216	-226.6394502	Control1
CATCAAGTCCTCCACA-1	singlet	0	-5694.928373	-16357.47577	-18277.09954	Control1
CATCAAGTCGCGCCAA-1	singlet	2	-7137.721739	-6480.628663	-2146.58713	Control1
CATCCACAGCGAATGC-1	singlet	2	-831.4139781	-642.7179107	-309.0473592	Control1
CATCCACAGCGCCTCA-1	singlet	2	-646.9397856	-484.6056346	-238.1818254	Control1
CATCCACAGGACACTG-1	singlet	2	-901.1449114	-725.6538809	-351.5150664	Control1
CATCCACAGGGCAGAG-1	singlet	2	-468.0602879	-456.2205587	-172.6120546	Control1
CATCCACCAACATCGT-1	singlet	1	-1834.831173	-737.5507729	-1740.287534	Control1
CATCCACCAACTTCTT-1	singlet	2	-906.6845644	-596.371177	-290.6909966	Control1
CATCCACCATCTCCCA-1	singlet	2	-46.52494594	-31.36842133	-17.81753738	Control1
CATCCACCATGCCGAC-1	singlet	2	-747.5997248	-554.6098271	-254.5229766	Control1
CATCCACGTAGTTACC-1	singlet	2	-1191.560954	-1114.538443	-429.9209988	Control1
CATCCACTCACTCGAA-1	singlet	1	-1220.138366	-421.3774902	-905.5920503	Control1
CATCCACTCCCGTAAA-1	singlet	2	-4095.414838	-3801.457113	-1451.04077	Control1
CATCCACTCGGTTAGT-1	singlet	2	-3698.455478	-2901.629531	-1036.183704	Control1
CATCCCAAGCAGTACG-1	singlet	2	-889.8650306	-654.2571861	-296.7014677	Control1
CATCCCAAGCCGATCC-1	singlet	2	-5319.294468	-4615.54754	-1870.216964	Control1
CATCCCAAGGCATGGT-1	singlet	1	-1143.108667	-449.2905074	-1148.937461	Control1
CATCCCAAGTATGAGT-1	singlet	1	-5036.813801	-1688.799131	-4023.407228	Control1
CATCCCATCCAAGAGG-1	singlet	2	-8418.126336	-7274.357209	-2129.659201	Control1
CATCCGTAGAAACTCA-1	singlet	1	-949.0939806	-412.294734	-923.5645248	Control1
CATCCGTAGTATAGAC-1	singlet	1	-262.4758076	-64.59228662	-200.1513723	Control1
CATCCGTCAAATCGGG-1	singlet	2	-219.2588749	-173.5599681	-76.88098689	Control1
CATCCGTCAACAGCCC-1	singlet	2	-692.9074197	-547.5872858	-253.1707454	Control1
CATCCGTCAAGAATGT-1	singlet	1	-1187.82268	-472.0008752	-1182.562565	Control1
CATCCGTCAATGAAAC-1	singlet	2	-1196.908907	-1228.558207	-558.4981538	Control1
CATCCGTCACACCAGC-1	singlet	2	-129.9342477	-99.8277293	-44.19985693	Control1
CATCCGTCACACGCCA-1	singlet	2	-3180.512018	-3004.08915	-1043.843356	Control1
CATCCGTCACCCATAA-1	singlet	1	-1526.802238	-652.6535325	-1471.949876	Control1
CATCCGTCATAAGATG-1	singlet	1	-567.9188989	-244.4825455	-692.4971992	Control1
CATCCGTCATCTAGAC-1	singlet	2	-5365.764953	-4159.16735	-1503.720928	Control1
CATCCGTCATGTTTGG-1	singlet	2	-2577.043135	-3998.543682	-883.6205482	Control1
CATCCGTCATTGTACG-1	singlet	2	-1129.923975	-1037.186551	-463.4694967	Control1
CATCCGTTCCTCACCA-1	singlet	2	-4685.835266	-3862.612987	-1635.446081	Control1
CATCCGTTCGACACCG-1	singlet	2	-858.6711813	-818.6428723	-408.2875358	Control1
CATCCGTTCTATGTGG-1	singlet	2	-1846.929136	-1633.454874	-732.4310221	Control1
CATCCGTTCTTGTTAC-1	singlet	2	-639.4892406	-551.0976093	-225.0128118	Control1
CATCGCTAGCATCAGG-1	singlet	2	-1025.023962	-1088.710774	-482.198868	Control1
CATCGCTAGCTAGCCC-1	singlet	1	-158.4477691	-24.0099877	-163.6390778	Control1
CATCGCTAGGATATAC-1	singlet	2	-2880.551265	-2277.923475	-965.4765256	Control1
CATCGCTCAGTATGAA-1	singlet	2	-1421.93015	-1374.538393	-560.5664454	Control1
CATCGCTGTTGGTGTT-1	singlet	1	-887.5937584	-349.3675685	-912.8380523	Control1
CATCGCTTCAGCTAGT-1	singlet	0	-374.4981273	-1142.006125	-1233.770645	Control1
CATCGCTTCTCGTGAA-1	singlet	2	-2420.551027	-2316.502093	-846.6299907	Control1
CATCGGGAGAGCTGAC-1	singlet	2	-334.0792826	-345.9842644	-137.9671057	Control1
CATCGGGAGCCGAACA-1	singlet	1	-8137.328836	-2269.349653	-7517.16605	Control1
CATCGGGAGGAGGCAG-1	singlet	2	-1549.077342	-1328.864369	-615.336103	Control1
CATCGGGAGTCCTACA-1	singlet	1	-2867.074245	-971.8618337	-2546.757533	Control1
CATCGGGAGTCTTGGT-1	singlet	2	-480.7577943	-381.5974028	-201.9709673	Control1
CATCGGGCAAATCGGG-1	singlet	2	-1307.49531	-1325.964782	-371.2593243	Control1
CATCGGGCAAGGTCTT-1	singlet	2	-710.1690226	-675.6997137	-282.5523378	Control1
CATCGGGCAGTGGTGA-1	singlet	0	-647.7025597	-3009.240053	-2263.139229	Control1
CATCGGGTCATACAGC-1	singlet	0	-284.9659053	-600.2826197	-908.6076188	Control1
CATCGGGTCATTACGG-1	singlet	0	-26.10868286	-61.58321759	-49.26257597	Control1
CATCGGGTCCATTTAC-1	singlet	1	-862.4323277	-358.3743702	-865.1304182	Control1
CATCGGGTCGTAGCTA-1	singlet	2	-534.6336461	-385.0649215	-190.271441	Control1
CATCGGGTCTGGAGAG-1	singlet	2	-389.5791437	-308.1501111	-133.3673308	Control1
CATCGGGTCTTCTAAC-1	singlet	1	-1145.190953	-464.9225512	-1043.327142	Control1
CATCGTCAGCTTTCCC-1	singlet	2	-3357.143552	-3173.261413	-976.3706083	Control1
CATCGTCAGGTCACTT-1	singlet	2	-3827.337948	-3194.512409	-1222.323643	Control1
CATCGTCCAACCAGAG-1	singlet	2	-1230.670109	-1015.918025	-491.4589965	Control1
CATCGTCGTAGAGCTG-1	singlet	2	-657.5440682	-971.8001709	-256.6978471	Control1
CATCGTCGTATCGTTG-1	singlet	2	-385.6930032	-226.6461638	-128.8477391	Control1
CATCGTCGTTCTCGTC-1	singlet	2	-196.765444	-147.0643486	-68.15712371	Control1
CATCGTCTCGCGCCAA-1	doublet	45323	-3574.415605	-2720.409498	-1646.314487	Control1

CATGAGTAGTAAACGT-1	singlet	2	-3717.803433	-6818.453807	-1291.566013	Control1
CATGAGTCACATAACC-1	singlet	2	-799.0771044	-671.1697678	-295.885965	Control1
CATGAGTCATTACGGT-1	singlet	2	-87.77956805	-49.92322023	-24.93236423	Control1
CATGAGTGTCCCTAAA-1	singlet	2	-311.7184723	-269.0129674	-106.3527335	Control1
CATGAGTTCCGTGTCT-1	singlet	2	-598.072161	-481.4279678	-259.8234999	Control1
CATGCAAAGAGGTTTA-1	singlet	2	-1449.083241	-1260.204127	-541.9608391	Control1
CATGCAAAGGTACAGC-1	singlet	1	-163.0230066	-58.41059642	-187.0735112	Control1
CATGCAAAGGTAGCCA-1	singlet	2	-1011.758975	-1066.32931	-433.1388854	Control1
CATGCAACAAGCGCAA-1	singlet	2	-580.6366944	-402.0889244	-210.0863815	Control1
CATGCAACAAGGACAC-1	singlet	2	-1240.571237	-1130.535528	-467.8833734	Control1
CATGCAACAGTTGCGC-1	singlet	2	-1236.247433	-1104.931357	-455.5746152	Control1
CATGCAACATCGTGCG-1	singlet	1	-3023.616826	-1045.3748	-2698.984851	Control1
CATGCAAGTTAATGAG-1	singlet	2	-1688.268357	-1466.006486	-636.0006255	Control1
CATGCAATCCTTGGAA-1	singlet	2	-4592.009513	-3828.597643	-1616.364613	Control1
CATGCAATCGGCCCAA-1	singlet	2	-1863.99364	-1593.179334	-689.6156873	Control1
CATGCAATCTCTAGGA-1	singlet	0	-899.225592	-3154.189061	-3035.821597	Control1
CATGCCTAGACTTCCA-1	singlet	1	-1614.45755	-681.4037237	-1473.068959	Control1
CATGCCTAGCTCGCAC-1	singlet	1	-1355.741073	-609.4929249	-1335.784129	Control1
CATGCCTAGGTAGTAT-1	singlet	2	-52.16503255	-28.62232164	-10.90493189	Control1
CATGCCTAGTCCTGCG-1	singlet	2	-1091.4386	-897.253923	-375.5598518	Control1
CATGCCTGTCCTCAGG-1	singlet	1	-2166.137416	-761.8019514	-2137.412734	Control1
CATGCCTGTCGGCACT-1	singlet	2	-559.27471	-366.0964545	-198.2114225	Control1
CATGCCTGTGTGGTCC-1	singlet	2	-1205.183432	-1024.058081	-466.2311453	Control1
CATGCCTTCGTCCTTG-1	singlet	2	-782.1185947	-551.3380367	-250.3761728	Control1
CATGCCTTCTTCCGTG-1	singlet	1	-1945.020535	-801.0604084	-1930.352955	Control1
CATGCGGAGAAGCTCG-1	singlet	0	-273.9388533	-639.1423463	-692.4790897	Control1
CATGCGGAGGGATCAC-1	singlet	2	-1851.616481	-1771.477941	-715.7991031	Control1
CATGCGGAGGGCAGGA-1	doublet	45323	-2982.0596	-2296.278974	-1805.105797	Control1
CATGCGGCACTCCTTG-1	singlet	2	-752.7024334	-814.303676	-359.5317761	Control1
CATGCGGGTAACAGTA-1	singlet	2	-2350.749001	-1965.193876	-736.7410619	Control1
CATGCGGGTATCGCGC-1	singlet	2	-2745.024851	-2346.718948	-954.1273386	Control1
CATGCTCAGTATTGCC-1	singlet	2	-2515.54283	-1958.656947	-848.9712544	Control1
CATGCTCCAGAATTCC-1	singlet	2	-3632.645786	-3270.695255	-1107.066552	Control1
CATGCTCGTGCAGGAT-1	singlet	1	-459.6805392	-169.3197745	-420.6355596	Control1
CATGCTCTCATGCCGG-1	singlet	1	-2294.704487	-866.1921943	-1841.980813	Control1
CATGCTCTCATGTCTT-1	singlet	1	-2317.078224	-1014.45209	-2165.330082	Control1
CATGCTCTCGCCTTGT-1	singlet	2	-145.3059173	-120.7628211	-43.05603778	Control1
CATGGATAGAAGCTGC-1	singlet	2	-770.5415542	-762.9437708	-312.9100716	Control1
CATGGATAGCGTTCCG-1	doublet	45323	-2080.534848	-1348.822805	-1280.223223	Control1
CATGGATCAACAAGAT-1	doublet	45323	-3194.288855	-2320.075809	-1437.453332	Control1
CATGGATCACGCGCTA-1	singlet	2	-594.9830374	-535.0861185	-216.5493741	Control1
CATGGATTCGCGGACT-1	singlet	2	-794.4524018	-745.5707667	-273.3815611	Control1
CATGGATTCTACTCAT-1	singlet	0	-187.8954497	-362.5076438	-499.1826455	Control1
CATGGTAAGAGAGTGA-1	singlet	2	-282.1194156	-228.5093771	-120.7002046	Control1
CATGGTACAGGAATCG-1	singlet	2	-617.3807955	-508.4175517	-241.8054423	Control1
CATGGTACAGGCGATA-1	singlet	1	-1075.596984	-407.5559348	-956.8302919	Control1
CATGGTACAGGTATGG-1	singlet	1	-884.9738833	-337.7795994	-827.6136149	Control1
CATGGTAGTTGTTGCA-1	singlet	1	-341.7582126	-147.8048243	-353.9175437	Control1
CATTCATAGATGTTAG-1	singlet	1	-763.8054664	-294.7208214	-623.2633565	Control1
CATTCATAGCACTCAT-1	singlet	1	-22.26452242	-3.53654661	-9.519598255	Control1
CATTCATAGCTGACTT-1	singlet	2	-2359.882936	-2077.297013	-846.6286553	Control1
CATTCATAGCTTCGTA-1	singlet	1	-2364.75197	-705.2692208	-2188.549122	Control1
CATTCATAGTCTACCA-1	singlet	1	-1505.872483	-621.3065177	-1492.163575	Control1
CATTCATCACTCAGAT-1	singlet	2	-529.5019714	-375.2990797	-197.1056219	Control1
CATTCATGTAGTTAGA-1	singlet	2	-531.9141493	-382.9726422	-162.0008864	Control1
CATTCATGTTAAGACA-1	singlet	2	-426.5783878	-349.6238187	-166.4788074	Control1
CATTCATTCCGCTGTT-1	singlet	2	-9921.630805	-8381.356945	-2974.476742	Control1
CATTCATTCGTGGAAG-1	singlet	2	-1869.026538	-1719.216559	-703.7789756	Control1
CATTCATTCTACGCGG-1	singlet	2	-436.5740955	-310.8020118	-159.2116742	Control1
CATTCATTCTCCCTAG-1	singlet	0	-1125.75678	-3118.715142	-3471.204495	Control1
CATTCCGCAAAGTGTA-1	singlet	1	-751.3794541	-294.7163138	-706.1462128	Control1
CATTCCGGTATACCCA-1	singlet	2	-1620.728703	-1373.024964	-572.1219857	Control1
CATTCCGGTCCGGTGT-1	singlet	2	-641.130085	-529.9666925	-250.0789006	Control1
CATTCCGGTCGGTACC-1	singlet	2	-1246.948722	-1168.641789	-523.2146345	Control1
CATTCCGGTGATACAA-1	singlet	1	-398.4405471	-177.2707162	-393.7788587	Control1
CATTCCGTCACTTTGT-1	singlet	1	-1561.53824	-635.7742413	-1541.176365	Control1
CATTCCGTCATTGCGA-1	singlet	1	-86.90031142	-46.14567229	-106.6838729	Control1
CATTCCGTCTGCTTTA-1	singlet	2	-418.972903	-277.1883929	-160.3903348	Control1
CATTCTAAGCTCGACC-1	singlet	2	-697.1871701	-601.5321098	-320.9107237	Control1
CATTCTAAGGGCAGTT-1	singlet	2	-652.8670796	-628.5926392	-281.7234761	Control1
CATTCTAAGGTCGTGA-1	singlet	1	-1139.872659	-447.4322849	-993.245861	Control1
CATTCTACAATTGAAG-1	singlet	2	-829.654982	-569.6643161	-235.7646571	Control1
CATTCTACACTGTTCC-1	unassigned	45293	-23.53198019	-8.952242763	-10.64168161	Control1
CATTCTACAGCCTTCT-1	singlet	1	-4116.622904	-1534.944733	-3817.647135	Control1
CATTCTACAGCGCGTT-1	singlet	2	-1221.383661	-1053.783648	-439.4395232	Control1
CATTCTATCGCAGTGC-1	singlet	2	-991.8084138	-989.746904	-372.9354553	Control1
CATTCTATCTTCTGTA-1	singlet	2	-496.8920274	-641.3580311	-175.4254753	Control1
CATTGAGAGCAAATCA-1	singlet	1	-1069.249759	-461.5742913	-1075.725109	Control1
CATTGAGAGCACTCGC-1	singlet	2	-980.0219523	-901.7932742	-316.0107556	Control1
CATTGAGAGTTGCTCA-1	singlet	2	-7736.555413	-6491.165894	-2458.510126	Control1
CATTGAGCAAAGACTA-1	singlet	1	-1574.699195	-637.9376277	-1456.0061	Control1
CATTGAGGTATTGAGA-1	singlet	2	-1502.91562	-1486.590858	-546.2093784	Control1
CATTGAGGTCAGATTC-1	singlet	0	-417.7240607	-1083.148394	-1150.169369	Control1
CATTGAGGTTGGACTT-1	singlet	2	-290.705716	-266.6541795	-150.6581173	Control1
CATTGCCCACCGCTAG-1	singlet	2	-2085.499352	-1890.195049	-793.5865524	Control1
CATTGCCCACTTGACA-1	singlet	2	-1211.779353	-946.706546	-446.5024309	Control1
CATTGCCCAGGTTTAC-1	singlet	2	-1587.378111	-1227.842554	-486.13637	Control1
CATTGCCCATGGCCAC-1	singlet	2	-1916.100892	-1686.749308	-660.3144662	Control1
CATTGCCCATTCTTCA-1	singlet	2	-3966.74237	-4592.339639	-1476.071485	Control1
CATTGCCTCACTCGAA-1	singlet	2	-5051.842998	-4399.3985	-1685.467423	Control1
CATTGCCTCGCACTCT-1	singlet	2	-3976.312682	-3461.967303	-1451.501068	Control1
CATTGCCTCGTAACAC-1	singlet	1	-1200.319157	-515.6995744	-1148.187904	Control1
CATTGTTAGAATCTAG-1	singlet	0	-1192.442466	-3295.841341	-3706.477737	Control1
CATTGTTCAATACAGA-1	singlet	2	-735.2133883	-575.838101	-259.1049478	Control1
CATTGTTCAATACCCA-1	singlet	2	-965.6556304	-744.0492306	-343.3976925	Control1
CATTGTTCATATGGCT-1	singlet	2	-2508.391567	-2163.933347	-875.3493413	Control1
CATTGTTGTACGATGG-1	doublet	2/0	-1240.085761	-1163.952215	-527.4525933	Control1
CATTGTTGTGCGCTCA-1	singlet	1	-1064.122021	-524.1058255	-1133.495422	Control1
CATTGTTGTGCGTTTA-1	singlet	2	-1017.278459	-711.5313719	-323.40795	Control1
CATTTCACACCCAACG-1	singlet	0	-1549.190582	-4490.269491	-4949.165696	Control1
CATTTCACACTGTGTA-1	singlet	2	-2898.660326	-2253.295992	-904.4627266	Control1
CATTTCACAGTTCTAG-1	singlet	2	-6697.049608	-5393.866938	-2199.439821	Control1
CATTTCACATTGGATC-1	doublet	45293	-1835.25922	-1164.258315	-1398.952879	Control1
CATTTCAGTCAAATCC-1	singlet	2	-5975.860766	-4991.204768	-1732.534084	Control1
CATTTCAGTGCGAGTA-1	singlet	2	-811.7704784	-726.4306003	-350.0180919	Control1
CATTTCAGTGGGACAT-1	singlet	2	-2051.283643	-1805.826084	-704.0635903	Control1
CATTTCAGTTGTCATG-1	singlet	1	-82.17338563	-24.04909845	-74.71606115	Control1
CATTTCATCAGTCACA-1	singlet	2	-1331.678669	-1039.424614	-452.4482506	Control1
CATTTCATCCCGTTGT-1	singlet	2	-487.3864012	-373.245148	-186.8633085	Control1
CATTTCATCTGCCTCA-1	singlet	2	-2174.20183	-1685.609594	-706.1470951	Control1

CCAAGCGAGAAACACT-1	singlet	1	-3119.778736	-979.3904751	-2469.485605	Control1
CCAAGCGAGCACCGTC-1	singlet	2	-573.2473389	-502.7737523	-242.565912	Control1
CCAAGCGAGTACAGCG-1	singlet	2	-5599.646757	-5132.496209	-1873.421364	Control1
CCAAGCGAGTCCTGTA-1	singlet	2	-521.2309821	-367.1113576	-149.2329646	Control1
CCAAGCGCAACGATCT-1	singlet	2	-517.3000573	-379.7763675	-163.5579238	Control1
CCAAGCGCACTCACTC-1	singlet	2	-11.83545114	-11.73850913	-7.12804559	Control1
CCAAGCGCAGTCTGGC-1	singlet	2	-705.7413863	-513.1600688	-258.7178294	Control1
CCAAGCGGTGTCCATA-1	singlet	0	-375.4041565	-1047.147094	-1211.432081	Control1
CCAAGCGGTTCGGGTC-1	singlet	2	-970.7977268	-1138.845781	-419.0388362	Control1
CCAAGCGGTTTGTGGT-1	doublet	2/0	-1228.548128	-1626.405205	-1145.194202	Control1
CCAAGCGTCCTAAGTG-1	singlet	2	-479.8145523	-445.5357882	-174.6700183	Control1
[truncated: 2,372,937 more chars]
